# Transcriptome analysis reveals molecular signature and cell-type difference of *Homo sapiens* endothelial-to-mesenchymal transition

**DOI:** 10.1093/g3journal/jkad243

**Published:** 2023-10-20

**Authors:** Ronald Bronson, Junfang Lyu, Jianhua Xiong

**Affiliations:** Department of Medicine, Division of Endocrinology, Diabetes and Metabolism, Johns Hopkins University School of Medicine, St.Petersburg, FL 33701, USA; Institute for Fundamental Biomedical Research, Johns Hopkins All Children's Hospital, St.Petersburg, FL 33701, USA; Department of Medicine, Division of Endocrinology, Diabetes and Metabolism, Johns Hopkins University School of Medicine, St.Petersburg, FL 33701, USA; Institute for Fundamental Biomedical Research, Johns Hopkins All Children's Hospital, St.Petersburg, FL 33701, USA; Department of Medicine, Division of Endocrinology, Diabetes and Metabolism, Johns Hopkins University School of Medicine, St.Petersburg, FL 33701, USA; Institute for Fundamental Biomedical Research, Johns Hopkins All Children's Hospital, St.Petersburg, FL 33701, USA

**Keywords:** transcriptome, *Homo sapiens* endothelial cell, endothelial-to-mesenchymal transition, chemotaxis, acetate

## Abstract

Endothelial-to-mesenchymal transition (EndoMT), a specific form of epithelial-to-mesenchymal transition, drives a growing number of human (*Homo sapiens*) pathological conditions. This emerging knowledge opens a path to discovering novel therapeutic targets for many EndoMT-associated disorders. Here, we constructed an atlas of the endothelial-cell transcriptome and demonstrated EndoMT-induced global changes in transcriptional gene expression. Our gene ontology analyses showed that EndoMT could be a specific checkpoint for leukocyte chemotaxis, adhesion, and transendothelial migration. We also identified distinct gene expression signatures underlying EndoMT across arterial, venous, and microvascular endothelial cells. We performed protein–protein interaction network analyses, identifying a class of highly connected hub genes in endothelial cells from different vascular beds. Moreover, we found that the short-chain fatty acid acetate strongly inhibits the transcriptional program of EndoMT in endothelial cells from different vascular beds across tissues. Our results reveal the molecular signature and cell-type difference of EndoMT across distinct tissue- and vascular-bed-specific endothelial cells, providing a powerful discovery tool and resource value. These results suggest that therapeutically manipulating the endothelial transcriptome could treat an increasing number of EndoMT-associated pathological conditions.

## Introduction

The human (*Homo sapiens*) endothelium is arguably among the largest organs in the human body, weighing almost 1 kilogram of a typical person's body and covering a surface area of about 4,000–7,000 square meters (Aird 2005). It is a monolayer of endothelial cells and includes approximately 10 trillion cells, constituting the inner cellular lining of the blood vessels and the lymphatic system (Galley and Webster 2004). The vascular endothelial cells lining all blood vessels build the vasculature, which regulates nutrient and gas exchanges between the bloodstream and surrounding tissues (Galley and Webster 2004; Aird 2005). Endothelial-to-mesenchymal transition (EndoMT), a specific form of epithelial-to-mesenchymal transition (EMT), is defined as the transdifferentiation of endothelial cells into mesenchymal cells characterized by the loss of endothelial features and the acquisition of mesenchymal, fibroblast-like properties (Kovacic et al. 2012). In 1977, this cellular conversion process was initially observed during normal cardiac development, contributing to valve formation and heart septation (Markwald et al. 1977; Kovacic et al. 2012). Emerging evidence has linked abnormal EndoMT to a wide range of pathological conditions, including but not limited to pulmonary hypertension (Xiong 2015a; Xiong 2015b), atherosclerosis (Chen et al. 2015; Evrard et al. 2016), vein graft stenosis (Cooley et al. 2014), cancer progression (Potenta et al. 2008), and fibrosis in key organs such as the lung, heart, and kidney (Zeisberg et al. 2007, 2008; Piera-Velazquez et al. 2011). Despite the importance of EndoMT in human diseases, the molecular signature and cell-type difference underlying EndoMT remain poorly understood.

The vasculature includes macrovasculature and microvasculature. The macrovasculature is composed of arteries and veins, while the microvasculature consists of small vessels (arterioles, capillaries, and venules) with a diameter <150 μm (Climie et al. 2019). For instance, primary human pulmonary microvascular endothelial cells (HPMVECs) are located in microvasculature, while primary human aortic endothelial cells (HAECs) and primary human umbilical vein endothelial cells (HUVECs) are derived from the largest artery (aorta) and the umbilical vein, respectively. Tissue-specific endothelial cells from different vascular beds exhibit markedly distinct gene expression and functional features (Nolan et al. 2013; Jambusaria et al. 2020; Kalucka et al. 2020). Therefore, developing effective therapeutic strategies for the increasing number of disorders associated with EndoMT would benefit from a transcriptome atlas of vascular endothelial cells undergoing EndoMT.

Here, we report a series of global transcriptional profiles investigating the molecular signature and cell-type difference of EndoMT. We also report a novel role for acetate in suppressing the EndoMT program. We first examined gene expression regulation in different types of endothelial cells undergoing EndoMT and then analyzed the endothelial molecular signatures and cell-type difference upon EndoMT induction. Finally, we explored the role of acetate as a robust inhibitor of EndoMT.

## Methods and materials

### Cell culture

All cells were cultured in a 37°C tissue-culture incubator with a 5% CO_2_ atmosphere and regularly tested for Mycoplasma contamination using MycoAlert Mycoplasma Detection Kit (LT07-318; Lonza). Primary human endothelial cells were obtained from Lonza (HUVECs [Human Umbilical Vein Endothelial Cells]: C2519A; HAECs [Human Aortic Endothelial Cells]: CC-2535; HPMVECs [Human Pulmonary MicroVascular Endothelial Cells]: CC-2527) and used between passages 4 and 9. Endothelial cells were maintained in Endothelial Cell Growth Medium MV2 Kit (C-22121; PromoCell) and grown on fibronectin (F2006; Sigma-Aldrich)-coated 6-well cell culture plates as previously described (Baudin et al. 2007; Xiong et al. 2018). Cell morphology was examined using a Nikon Eclipse TS 100 microscope (Nikon, Tokyo, Japan) and images were acquired using Nikon's NIS (Nikon Instruments Software)-Elements Imaging Software (version 5.21.03).

### Chemicals and reagents

For induction of EndoMT, subconfluent endothelial cells on fibronectin-coated plates were incubated with 10 ng/mL recombinant human TGF-β1 (240-B-010/CF; R&D Systems) and 1 ng/mL recombinant human IL-1β (579404; BioLegend) for 4 days, with equal amounts of cytokines and fresh media changed every other day. Sodium acetate (S5636; Sigma-Aldrich) was added, where indicated, at a concentration of 40 mM as previously employed (Xiong et al. 2018; Lyu et al. 2022). Because endothelial cell function may be affected by changes in cellular pH (Faes et al. 2016; Deguchi et al. 2022), an acetate solution (pH 7.4) was used with the same pH as the culture media.

### Real-time quantitative reverse transcription PCR (real-time qPCR)

Endothelial cells were seeded in 6-well cell culture plates and treated with or without 10 ng/mL TGF-β1 and 1 ng/mL IL-1β, and with or without 40 mM acetate as indicated, for 4 days. Total RNAs were extracted from cultured endothelial cells using the Direct-zol RNA Miniprep Plus Kit (R2072; Zymo Research). During RNA purification, DNase I treatment was performed for DNase digestion (Zymo Research). Complementary DNA (cDNA) was prepared using the iScript cDNA Synthesis Kit (1708891, Bio-Rad Laboratories). Real-time qPCR was performed on a CFX Connect Real-Time System (Bio-Rad Laboratories) with HotStart 2X SYBR Green qPCR Master Mix (K1070, APExBIO) according to the manufacturer's instructions. Data were acquired using CFX Maestro software (Bio-Rad Laboratories) and then analyzed using the comparative cycling threshold (ΔΔCt) methodology. The primer oligos were synthesized and purified by Integrated DNA Technologies (Coralville, IA). The primer sequences were used to detect human gene expression: *ANPEP*, forward primer, 5′- GACCAAAGTAAAGCGTGGAATCG-3′, and reverse primer, 5′-TCTCAGCGTCACCCGGTAG-3′; *ARGLU1*, forward primer, 5′-GACCGCATCGACATCTTCGG-3′, and reverse primer, 5′-CGCTCGAACTCCGCTTTCTT-3′; *CCL3L1*, forward primer, 5′-CACCTCCCGACAGATTCCAC-3′, and reverse primer, 5′-GGTCACTGACGTATTTCTGGAC-3′; *CD44*, forward primer, 5′-GACAAGTTTTGGTGGCACG-3′, and reverse primer, 5′-CACGTGGAATACACCTGCAA-3′; *CXCL5*, forward primer, 5′-AGCTGCGTTGCGTTTGTTTAC-3′, and reverse primer, 5′-TGGCGAACACTTGCAGATTAC-3′; *CXCL11*, forward primer, 5′-GACGCTGTCTTTGCATAGGC-3′, and reverse primer, 5′-GGATTTAGGCATCGTTGTCCTTT-3′; *DDX39B*, forward primer, 5′-TCCAGGCCGTATCCTAGCC-3′, and reverse primer, 5′-GCATGTCGAGCTGTTCAAGC-3′; *ERV3-1*, forward primer, 5′-TGTTCTTGCTACTCCCCTTATCC-3′, and reverse primer, 5′-GTTCCCCGACCACGTAGTG-3′; *GAPDH*, forward primer, 5′-AAGGTGAAGGTCGGAGTCAA-3′, and reverse primer, 5′-AATGAAGGGGTCATTGATGG-3′; *ICAM2*, forward primer, 5′-TCGGTTACAGGACCCTGACT-3′, and reverse primer, 5′-CTTTGGCCTCACGTGTACCT-3′; *ICOSLG*, forward primer, 5′-GCAGCCTTCGAGCTGATACTC-3′, and reverse primer, 5′-GTTTTCGACTCACTGGTTTGC-3′; *INSIG2*, forward primer, 5′-CTTGATGATTCGAGGAGTAGTGC-3′, and reverse primer, 5′-CAGGTGGAAAGAGCGTCACAT-3′; *KIT*, forward primer, 5′-GGGATTTTCTCTGCGTTCTG-3′, and reverse primer, 5′-GATGGATGGATGGTGGAGAC-3′; *LY6E*, forward primer, 5′-CAGCTCGCTGATGTGCTTCT-3′, and reverse primer, 5′-CAGACACAGTCACGCAGTAGT-3′; *LYVE1*, forward primer, 5′-AGGCTCTTTGCGTGCAGAA-3′, and reverse primer, 5′-GGTTCGCCTTTTTGCTCACAA-3′; *MCM4*, forward primer, 5′-TTCTTTGACCGTTACCCTGAC-3′, and reverse primer, 5′-GGGATGTCCTGATCACCATG-3′; *MMP1*, forward primer, 5′-AAAATTACACGCCAGATTTGCC-3′, and reverse primer, 5′-GGTGTGACATTACTCCAGAGTTG-3′; *PECAM1*, forward primer, 5′-CCTTCTGCTCTGTTCAAGCC-3′, and reverse primer, 5′-GGGTCAGGTTCTTCCCATTT-3′; *RPL7*, forward primer, 5′-CAAGGCTTCGATTAACATGCTGA-3′, and reverse primer, 5′-GCCATAACCACGCTTGTAGATT-3′; *SAMHD1*, forward primer, 5′-CTGGAACTCCATCCCGACTAC-3′, and reverse primer, 5′-AGTAATGCGCCTGTGATTTCAT-3′;

*SERPINE1*, forward primer, 5′-ACAACAGGAGGAGAAACCCA-3′, and reverse primer, 5′-AGCTCCTTGTACAGATGCCG-3′; *STAT1*, forward primer, 5′-CGGCTGAATTTCGGCACCT-3′, and reverse primer, 5′-CAGTAACGATGAGAGGACCCT-3′; *TAGLN*, forward primer, 5′-GTCCGAACCCAGACACAAGT-3′, and reverse primer, 5′-CTCATGCCATAGGAAGGACC-3′; *TSPAN13*, forward primer, 5′-GCGCCCTCAACCTGCTTTA-3′, and reverse primer, 5′-ACTCGGAGACTGGAAATCAGC-3′; *TSPAN18*, forward primer, 5′-GCTGTTACACGGTGATCCTCA-3′, and reverse primer, 5′-CATGGCGAAAAGCTCGATGG-3′; *UBE2L6*, forward primer, 5′-TGGACGAGAACGGACAGATTT-3′, and reverse primer, 5′-GGCTCCCTGATATTCGGTCTATT-3′.

### Western blot analysis

Western blot analysis was performed according to a previously described protocol with some modifications (Xiong et al. 2018). In brief, endothelial cells were lysed using Radioimmunoprecipitation assay (RIPA) buffer (BP-115; Boston BioProducts) supplemented with Protease Inhibitor Cocktail (11836170001; Roche Life Sciences) and Phosphatase Inhibitor Cocktail (04906837001; Roche Life Sciences). Protein lysates were centrifuged (14,000 g) for 10 minutes at 4°C, and then the supernatants were heated at 95°C with addition of 4x Laemmli Sample Buffer (1610747; Bio-Rad Laboratories) for 10 minutes. For immunoblotting, cell lysates were subjected to SDS–polyacrylamide-gel electrophoresis (SDS–PAGE) separation with a Mini-PROTEAN Tetra Electrophoresis Cell (Bio-Rad Laboratories), and then transferred to a nitrocellulose membrane using Trans-Blot Turbo Transfer System (Bio-Rad Laboratories). The membranes were blocked in phosphate-buffered saline (PBS) + 0.1% Tween-20 supplemented with 5% (w/v) Blotting Grade Blocker nonfat dry milk (1706404; Bio-Rad Laboratories), or bovine serum albumin (BSA; 03116964001; Roche Life Sciences) for 1 hour at room temperature. After blocking, the membranes were incubated with the indicated primary antibodies overnight at 4°C, followed by incubation with horseradish peroxidase (HRP)-conjugated secondary antibodies for 1 hour at room temperature. The protein bands were detected with Clarity Western ECL Substrate (1705061, Bio-Rad Laboratories) or SuperSignal West Pico PLUS Chemiluminescent Substrate (34,579, Thermo Fisher Scientific) under an Odyssey XF Imager (LI-COR Biosciences) and analyzed using Empiria Studio software (LI-COR Biosciences). The following validated primary rabbit monoclonal antibodies from Cell Signaling Technology were used: anti-pSMAD2 (3,108), anti-SMAD2 (5,339), and anti-GAPDH (5,174). The goat antirabbit IgG (H + L)- HRP) conjugate (170–6,515) secondary antibodies were purchased from Bio-Rad Laboratories.

### RNA library preparation and sequencing

Endothelial cells were treated, where indicated, with or without 10 ng/mL TGF-β1 and 1 ng/mL IL-1β, and with or without 40 mM acetate for 4 days, and then total RNAs were isolated using RNeasy Plus 96 Kit (74,192, Qiagen) following the manufacturer's instruction. Isolated RNA sample quality was assessed by High Sensitivity RNA Tapestation (Agilent Technologies Inc., CA, USA) and quantified by Qubit 2.0 RNA HS assay (Thermo Fisher Scientific, MA, USA). Paramagnetic beads coupled with oligo d(T)25 were combined with total RNA to isolate poly(A)+ transcripts based on NEBNext Poly(A) mRNA Magnetic Isolation Module manual (E7490, New England BioLabs). Before first strand synthesis, samples were randomly primed (5´ d(N6) 3´ [N = A, C, G, T]) and fragmented based on the manufacturer's recommendations. The first strand was synthesized with the Protoscript II Reverse Transcriptase with a longer extension period, approximately 40 minutes at 42°C. All remaining steps for library construction were used per the NEBNext Ultra II Directional RNA Library Prep Kit for Illumina (E7760L, New England BioLabs). The final libraries quantity was assessed by Qubit 2.0 (Thermo Fisher Scientific, MA, USA) and the quality was assessed by TapeStation D1000 ScreenTape (Agilent Technologies Inc., CA, USA). The final library size was about 430 bp with an insert size of about 300 bp. Illumina 8-nt dual-indices were used. Equimolar pooling of libraries was performed based on Quality Control (QC) values and sequenced on an Illumina Novaseq platform (Illumina, CA, USA) with a read length configuration of 150 PE for 40 M PE reads per sample (20 M in each direction).

### RNA-seq data processing and bioinformatic analyses

FastQC (version v0.11.8) was applied to check the quality of raw reads. Trimmomatic (version v0.38) was applied to cut adaptors and trim low-quality bases with default setting. Spliced Transcripts Alignment to a Reference (STAR) Aligner version 2.7.1a was used to align the reads. Picard tools (version 2.20.4) were applied to mark duplicates of mapping. The StringTie version 2.0.4 was used to assemble the RNA-Seq alignments into potential transcripts. The featureCounts (version 1.6.0)/HTSeq was used to count mapped reads for genomic features such as genes, exons, promoters, gene bodies, genomic bins, and chromosomal locations. The RNA-Seq data were analyzed and visualized using an R programming environment. The DESeq2 (version 1.14.1) was used for the differential analysis. For volcano plots, adjusted *P*-value was rounded to 1E-307 while it was zero. The Gene Ontology (GO) analysis was done using the ClusterProfiler package (Yu et al. 2012). The protein–protein networks were generated using the web-based tool STRING database (version 12.0) and visualized using Cytoscape (version: 3.10.0) (Shannon et al. 2003; Szklarczyk et al. 2023). The RNA-Seq data were deposited in the NCBI's Gene Expression Omnibus (GEO) repository with the accession number GSE236730.]

### Statistical analysis

All statistics were calculated using GraphPad Prism software (version 9.5.1; GraphPad Software). Unless mentioned otherwise, the statistical significance of differences between groups was determined by unpaired 2-sided Student's *t*-test. Results are represented as the mean ± standard error of the mean (SEM) and *P*-values < 0.05 were considered significant.

## Results and discussion

### Dramatic global alterations in gene expression accompany EndoMT

**Fig. 1.**
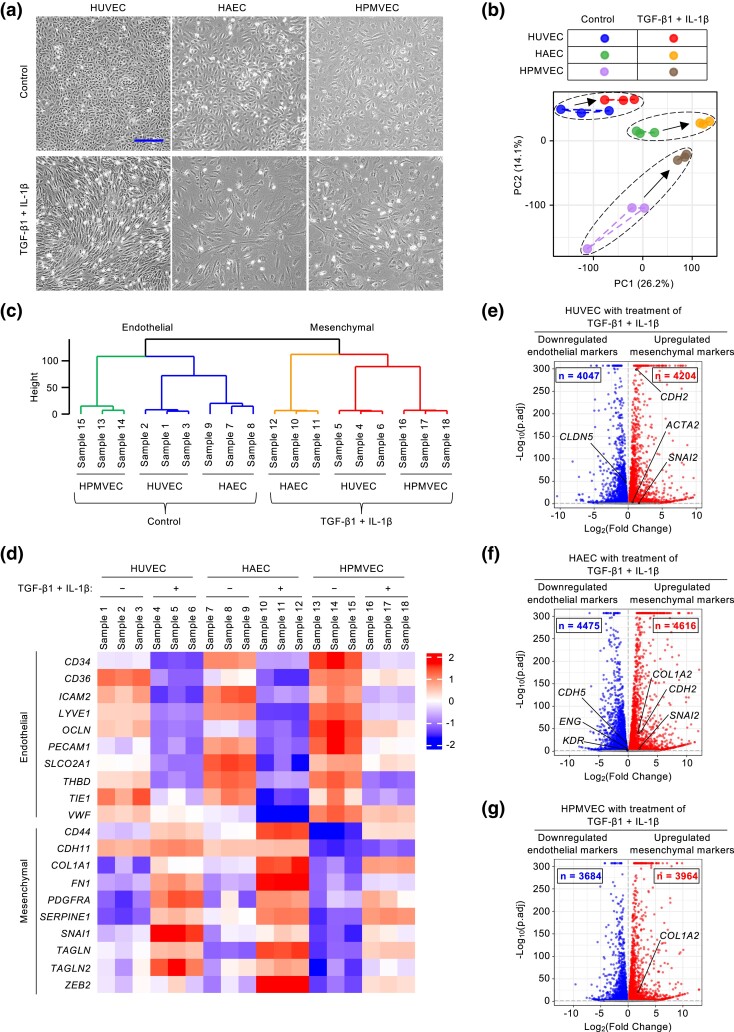
A dramatic global change in gene transcription accompanies EndoMT induction. a) TGF-β1 and IL-1β induce morphological changes consistent with EndoMT in HUVECs, HAECs, and HPMVECs. Scale bar, 300 µm. b) PCA of RNA-Seq data from endothelial cells with or without cytokine treatment. c) Hierarchically clustered dendrogram of RNA-Seq data from endothelial cells with or without cytokine treatment (n = 3 biological replicates). d) Heatmap analysis of transcription expression data for EndoMT markers in endothelial cells with or without cytokine treatment (n = 3 biological replicates). e–g) Volcano plots of significantly differentially expressed genes in cytokine-treated HUVECs (e), HAECs (f), and HPMVECs (g), compared with their corresponding control cells. The −log_10_(adjusted *P-*value) was plotted against the log_2_(Fold-Change) in gene expression. Upregulated genes in cytokine-treated endothelial cells (log_2_[Fold-Change] > 0 and adjusted *P-*value < 0.05) are depicted as red dots; genes that were downregulated in cytokine-treated endothelial cells (log_2_[Fold-Change] < 0 and adjusted *P-*value < 0.05) are depicted in blue (n = 3 biological replicates); gray dots indicate genes that were not significantly regulated by cytokine treatment.

We recently established that primary HPMVECs cultures could be stimulated to undergo EndoMT by treating cells with a cytokine combination of transforming growth factor β1 (TGF-β1) and interleukin-1β (IL-1β) (Xiong et al. 2018, Xiong 2018a). This design was based on 2 premises: first, TGF-β signaling and IL-1β-associated inflammation are key culprits of EndoMT-linked endothelial cell dysfunction (Rieder et al. 2011; Xiong 2015a; Xu and Kovacic 2023); second, as a potent inducer of EndoMT, this cytokine combination allows us to map endothelial cell changes in the transcriptome over a short time course (Xiong et al. 2010; Jing et al. 2023; Lyu et al. 2023). Under this design, cytokine-treated endothelial cells underwent a clear morphological transition 4 days later from normal polygonal cobblestone monolayers into mesenchymal-like cells, adopting an elongated spindle-shaped appearance (Fig. 1a).

To identify potential novel molecular mechanisms of EndoMT, we performed transcriptome profiles of human primary endothelial cells from different vascular beds (HUVECs from veins, HAECs from arteries, and HPMVECs from microvascular vessels) using the design described above. We first assessed these transcriptional datasets using a pan-endothelial marker list from the PanglaoDB database (Franzen et al. 2019; Jambusaria et al. 2020). We found that hierarchical clustering of the RNA-sequencing (RNA-Seq) profiles focused on 186 pan-endothelial genes separated all replicate baseline samples (Supplementary Fig. 1a). This result indicated that many classical endothelial cell markers were induced coordinately across all 3 cell types. In addition, there were distinct transcriptional patterns for many classic pan-endothelial markers suggesting there is also cell-type difference across endothelial cells derived from different vascular beds.

To further explore and visualize the RNA-seq datasets, we took advantage of 2 data-driven modeling approaches: principal component analysis (PCA, a method for data condensation) and clustering (a means for data organization) (Janes and Yaffe 2006). PCA biplot showed that the gene expression patterns for each type of endothelial cell clustered apart from each other and there was a distinct shift in gene expression patterns in endothelial cells after cytokine treatment (Fig. 1b). We built a clustering tree to group similar samples and identified cell-type difference among normal microvascular (HPMVEC) and macrovascular (HUVEC and HAEC) endothelial cells (Fig. 1c).

This responsiveness to cytokine treatment manifested as a marked decrease in numerous well-known endothelial markers and a simultaneous increase in mesenchymal markers (Fig. 1d, Supplementary Fig. 1b). We found 20 shared markers (10 endothelial markers [*CD34*, *CD36*, *ICAM2*, *LYVE1*, *OCLN*, *PECAM1*, *SLCO2A1*, *THBD*, *TIE1,* and *VWF*] and 10 mesenchymal markers [*CD44, CDH11, COL1A1, FN1, PDGFRA, SERPINE1, SNAI1, TAGLN, TAGLN2,* and *ZEB2*]). We also found several specific markers with significant changes in cytokine-treated HUVECs, HAECs, and HPMVECs (Fig. 1e–g). Further, a volcano plot revealed a remarkable number of genes with a significantly altered expression (Fig. 1e–g).

### EndoMT promotes cell chemotaxis and related biological processes

**Fig. 2.**
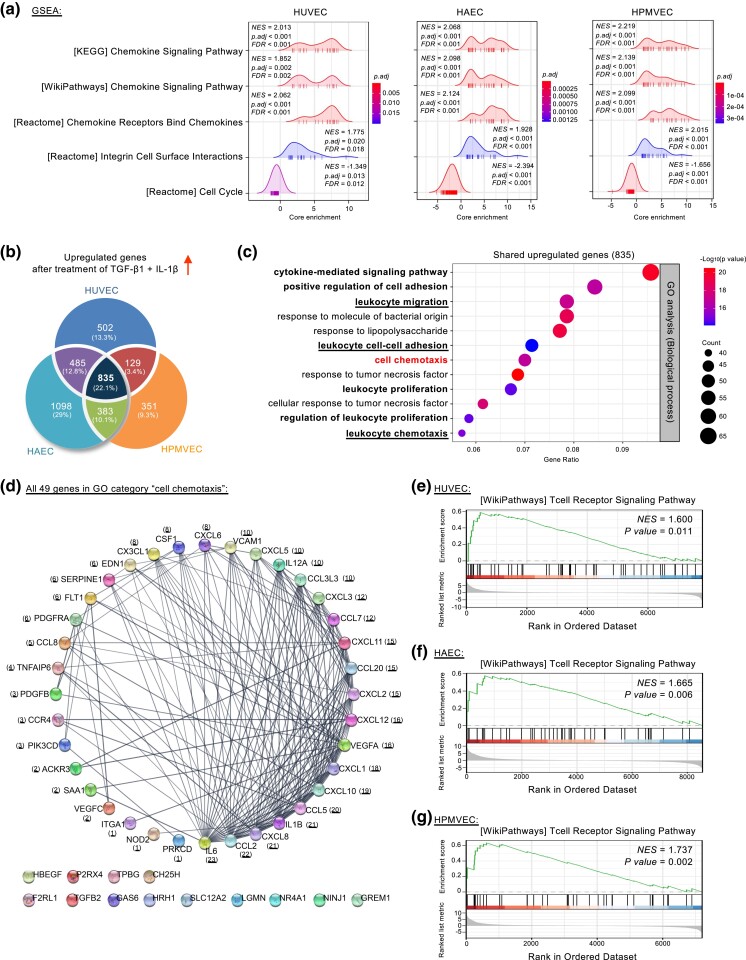
EndoMT modulates cell chemotaxis and related processes. a) Ridge plots of GSEA-defined cell cycle and chemotaxis-related pathways shared by cytokine-treated HUVECs, HAECs, and HPMVECs using differentially expressed genes from RNA-Seq data (adjusted *P-*value < 0.05). A significant positive NES (normalized enrichment score) value indicates the gene set at the top of the ranked transcriptome data and a negative NES indicates the opposite. b) Venn diagram showing overlapping and unique sets of upregulated genes in cytokine-treated endothelial cells (log_2_[Fold-Change] > 0.5 and adjusted *P-*value < 0.05). c) Bubble plot depicting top 12 enriched GO biological processes for the shared 835 upregulated genes in cytokine-treated endothelial cells. d) Cytoscape-based degree sorted circle plot showing STRING database-derived protein–protein interactions using all 49 genes of the GO category “cell chemotaxis” in Fig. 2c. The number in parenthesis indicates the number of protein–protein interactions. e–g) GSEA plots showing “T cell receptor signaling pathway” enrichment in cytokine-treated endothelial cells using differentially expressed genes from RNA-Seq data (adjusted *P-*value < 0.05).

To better understand the molecular features of EndoMT, we conducted gene set enrichment analysis (GSEA) on the above-mentioned differentially expressed genes (Fig. 1e–g). GSEA indicated significant enrichment of the “chemokine signaling pathway” and its related “chemokine receptors bind chemokines” categories. These appeared at the top of the ranked transcriptome data across all 3 types of endothelial cells (HUVECs, HAECs, and HPMVECs) (Fig. 2a).

We next asked how these endothelial cells resemble each other transcriptomically during EndoMT. To this end, we identified 835 upregulated genes shared across all 3 samples using adjusted *P*-value < 0.05 and Log_2_(fold-change) > 0.5 as a cutoff (Fig. 2b, Supplementary Tables 1–3). GO analysis of these shared upregulated genes identified the biological process “cell chemotaxis” and multiple related categories including (but not limited to) “cytokine-mediated signaling pathway,” “positive regulation of cell adhesion,” “leukocyte migration,” “leukocyte cell-cell adhesion,” “leukocyte proliferation,” and “leukocyte chemotaxis” (Fig. 2c).

Chemotaxis is the directed movement of cells in response to an extracellular chemoattractant gradient (Wu 2005; Roussos et al. 2011). The chemokines (chemotactic cytokines) are a large family of small, secreted chemoattractant proteins that can target circulating leukocytes (e.g. T-cells, monocytes, and neutrophils) to sites of inflammation or injury (Baggiolini 1998; Campbell and Butcher 2000; Charo and Ransohoff 2006; Raman et al. 2011). These immune responses depend on the trafficking of leukocytes into targeted tissues, which requires rolling, adhesion, and diapedesis through endothelial barriers (Salmi and Jalkanen 2005; Ley et al. 2007; Friedl and Weigelin 2008; Vestweber 2015; Alon et al. 2021). Since our GO analysis highlighted “leukocyte chemotaxis,” “leukocyte cell-cell adhesion,” and “leukocyte migration,” we reasoned that EndoMT might enhance the transcription of key genes for potentiating leukocyte activation, attachment, and transendothelial migration. Supporting this notion, we found robust regulation of all 49 differentially expressed genes in the GO category “cell chemotaxis”, including 2 important chemokine receptor-encoding genes (C–C motif chemokine receptor 4 [*CCR4*] and atypical chemokine receptor 3 [*ACKR3*, also known as C-X-C chemokine receptor type 7, *CCR7*]) along with 16 genes encoding critical chemokines (Supplementary Fig. 2a). Chemokines are classified into 4 families (CC, CXC, CX3C, and C) based on the position of conserved cysteine residues (Baggiolini 1998; Campbell and Butcher 2000; Charo and Ransohoff 2006; Raman et al. 2011). The GO-enriched chemokine-encoding genes included six encoding CC chemokines (*CCL2*, *CCL20*, *CCL3L1*, *CCL5*, *CCL7*, and *CCL8*), 1 encoding CXC chemokine (*CX3CL1*) and nine encoding CX3C chemokines (*CXCL1*, *CXCL2*, *CXCL3*, *CXCL5*, *CXCL6*, *CXCL8*, *CXCL10*, *CXCL11*, and *CXCL12*) (Supplementary Fig. 2a). EndoMT also stimulated genes typical of cell adhesion (*ITGA1*, *VCAM1*, and *TPBG*) and cytokines, growth factors, and their receptors (*CSF1*, *EDN1*, *HBEGF*, *IL1B*, *IL6*, *IL12A*, *PDGFB*, *PDGFRA*, *TGFB2*, *VEGFA*, and *VEGFC*) (Supplementary Fig. 2a).

To uncover the potential protein–protein association network architecture, we then searched the 49 genes in the GO category “cell chemotaxis” in the STRING database using a high-confidence interaction threshold score of 0.7 for connecting nodes (Szklarczyk et al. 2023). Subsequently, the network was viewed in Cytoscape and set to a degree-sorted circle layout (Shannon et al. 2003). Notably, this analysis identified 36 highly connected “hub” genes, most of which encode crucial regulators of leukocyte adhesion, migration, and chemotaxis (Fig. 2d). The proteins from hub nodes with the most protein–protein interactions were secreted, indicating that EndoMT could affect function through a secretome expression profile. Additionally, our GO results, combined with the GSEA analysis, showed that EndoMT could play a role in “leukocyte proliferation” and “regulation of leukocyte proliferation,” probably through the T-cell receptor signaling pathway (Fig. 2c and e–g). In contrast to the shared upregulated genes across endothelial cells (HUVECs, HAECs, and HPMVECs) undergoing EndoMT, shared downregulated genes were in the “regulation of mitotic cell cycle” and related biological processes (Supplementary Fig. 2b and c, Table 1–3). The inhibition of cell cycle progression aligns with the burgeoning role of cell cycle arrest in regulating EMT, a similar phenomenon of EndoMT (Kovacic et al. 2012; Chei et al. 2019; Lyu et al. 2022). Together, these results demonstrated that EndoMT of tissue- and vascular-bed-specific endothelial cells share a transcriptional program associated with cell chemotaxis. More importantly, our data suggest that EndoMT could act as a checkpoint for leukocyte chemotaxis, extravasation, and entry into extravascular tissues during immune responses.

### Transcriptional profiling of vascular-bed-specific EndoMT reveals vascular heterogeneity

**Fig. 3.**
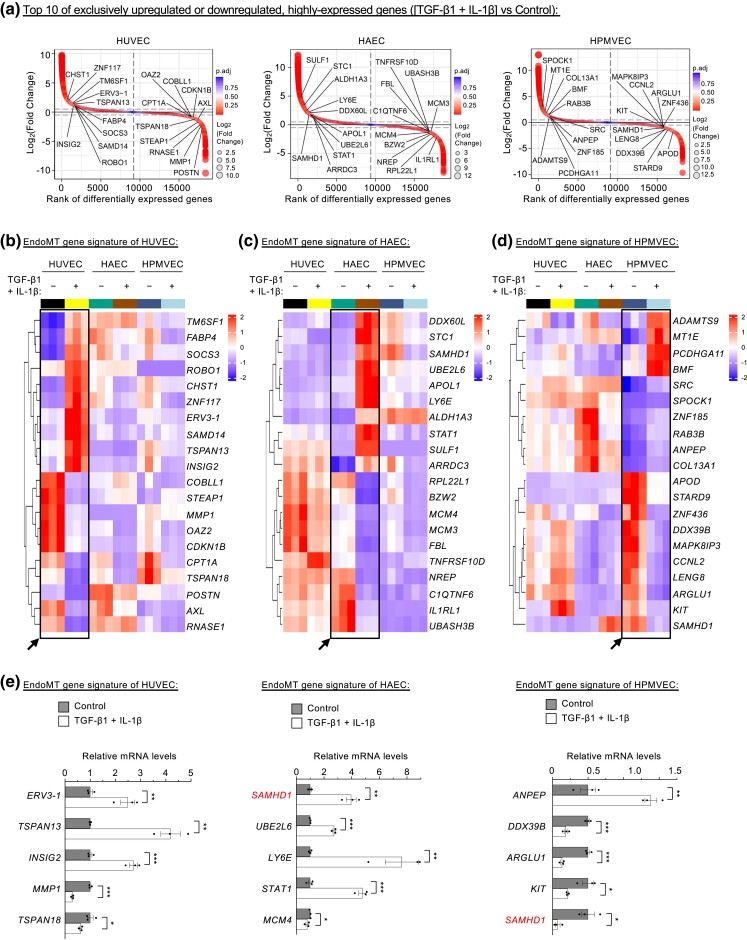
Vascular-bed-specific EndoMT shows unique signature expression profiles. a) Fold-change-based ranking of all genes differentially expressed in endothelial cells treated or not treated with cytokines (n = 3 biological replicates). b–d) Hierarchically clustered heatmaps of the expression of the 10 most strongly upregulated or downregulated, highly expressed signature genes in HUVECs (b), HAECs (c), and HPMVECs (d). e) Real-time qPCR analysis of signature gene expression in endothelial cells (n = 3 biological replicates). *P < 0.05; **P < 0.01; ***P < 0.001.

To visualize the significantly upregulated or downregulated genes following induction of EndoMT in the different endothelial subtypes, we first performed differential expression analysis and used log2-transformed fold-change values to rank the genes (Fig. 3a). To characterize the transcriptional heterogeneity of EndoMT, we focused on the unique gene expression profiles comprising 936 transcripts in HUVECs (502 upregulated and 434 downregulated), 2,323 transcripts in HAECs (1,098 upregulated and 1,225 downregulated) and 1,125 transcripts in HPMVECs (351 upregulated and 774 downregulated) (Fig. 2b, Supplementary Fig. 2b). Among these unique genes with high expression levels (more than 1,000 FPKM [fragments per kilobase of transcript per million mapped reads] on average), we identified the ten most strongly upregulated or downregulated in HUVEC-specific genes relative to HAECs and HPMVECs (Fig. 3a and b). The upregulated gene set contains *TM6SF1*, *FABP4*, *SOCS3*, *ROBO1*, *CHST1*, *ZNF117*, *ERV3-1*, *SAMD14*, *TSPAN13*, and *INSIG2*. In contrast, the downregulated set includes *COBLL1*, *STEAP1*, *MMP1*, *OAZ2*, *CDKN1B*, *CPT1A*, *TSPAN18*, *POSTN*, *AXL*, and *RNASE1* (Fig. 3b). Using these gene identifiers, we revealed 4 groups of protein–protein interactions from the STRING database (Supplementary Fig. 3a). For instance, the network containing fatty acid binding protein 4 (FABP4)-insulin-induced gene 2 (INSIG2)-carnitine palmitoyltransferase 1A (CPT1A) group relates to lipid uptake, transport, and metabolism, while the transcription of *ERV3-1/ZNF117* is linked to obesity and type 2 diabetes (Glastonbury et al. 2016). The class of suppressor of cytokine signaling 3 (SOCS3)-matrix metallopeptidase 1 (MMP1)-periostin (POSTN)-AXL receptor tyrosine kinase (AXL) proteins regulate kinase activity or cell adhesion (Szklarczyk et al. 2023). The tetraspanin family of cell-surface membrane proteins TSPAN13 and TSPAN18 have roles in cell adhesion and migration (Zhou et al. 2023).

In addition to HUVECs, we identified genes that could represent EndoMT heterogeneity and signature specifically in HAECs (ten upregulated [*DDX60L*, *STC1*, *SAMHD1*, *UBE2L6*, *APOL1*, *LY6E*, *ALDH1A3*, *STAT1*, *SULF1*, and *ARRDC3*] and ten downregulated [*RPL22L1*, *BZW2*, *MCM4*, *MCM3*, *FBL*, *TNFRSF10D*, *NREP*, *C1QTNF6*, *IL1RL1*, and *UBASH3B*]) (Fig. 3a and c). We also identified the unique gene signature for HPMVECs which contained 10 upregulated (*ADAMTS9*, *MT1E*, *PCDHGA11*, *BMF*, *SRC*, *SPOCK1*, *ZNF185*, *RAB3B*, *ANPEP*, and *COL13A1*) and 10 downregulated (*APOD*, *STARD9*, *ZNF436*, *DDX39B*, *MAPK8IP3*, *CCNL2*, *LENG8*, *ARGLU1*, *KIT*, and *SAMHD1*) (Fig. 3a and d). When these genes were analyzed using the STRING database-derived networks, there were 2 clusters of protein–protein interactions for HAECs and HPMVECs (Supplementary Fig. 3b and c). It is important to note that signal transducer and activator of transcription (STAT) proteins are among the best-known latent cytoplasmic signal-dependent transcription-factor pathways in immune responses (Shuai and Liu 2003). The “STAT1-centric” module in HACEs includes STAT1, UBE2L6, LY6E, and SAMHD1 (Supplementary Fig. 3b). In the second cluster of HAECs, minichromosome maintenance complex components 3 and 4 (MCM3 and MCM4) are part of the MCM2-7 complex needed for chromatin replication, epigenome integrity, and cell cycle (Alabert and Groth 2012).

Further, we found that 2 signature genes in HPMVECs’ EndoMT representing splicing factors: DExD-box helicase 39B (*DDX39B*) and arginine and glutamate rich 1 (*ARGLU1*) (Parra et al. 2020). Since SRC proto-oncogene, nonreceptor tyrosine kinase (SRC) modulates actin cytoskeleton, and cell adhesion (Frame et al. 2002), the “SRC-centric” module composed of SRC, KIT, MAPK8IP3, CCNL2, ANPEP, and SAMHD1 could control EndoMT-mediated leukocyte adhesion and migration (Supplementary Fig. 3c). Sterile Alpha Motif (SAM) and Histidine-Aspartic Domain (HD) domain-containing deoxynucleoside triphosphate triphosphohydrolase 1 (SAMHD1) is a triphosphohydrolase enzyme controlling the intracellular level of deoxyribonucleoside triphosphates (dNTPs) (Ballana and Este 2015). Interestingly, SAMHD1 was an upregulated signature gene of EndoMT in HAECs but acted as a downregulated EndoMT signature for HPMVECs (Fig. 3c and d, Supplementary Fig. 3b and c). We also found that chromosome 7 for HUVECs and chromosome 8 for HAECs appeared to be more active when they had more loci-based signature genes (Supplementary Fig. 3d–f). The potentially divergent regulatory role of SAMHD1 in the EndoMT of HAECs and HPMVECs warrants further investigation (Fig. 3e). Moreover, using the complete list of differentially expressed genes in HUVECs only, or HPMVECs only (Fig. 2b and Supplementary 2b), we identified very few experimentally validated protein–protein associations specific for EndoMT in different endothelial cell types (Supplementary Fig. 4a–c). In comparison with HUVECs or HPMVECs, HAECs have much more experimentally validated protein–protein interactions for EndoMT signature (Fig. 2b, Supplementary 2b, 4d and e), which merit future studies. Overall, our analysis revealed the vascular cell-type difference of EndoMT and identified a panel of unique EndoMT signature genes for distinct tissue- and vascular-bed-specific HUVECs, HAECs, and HPMVECs.

### Acetate modulates transcriptional programming of endothelial cells undergoing EndoMT

**Fig. 4.**
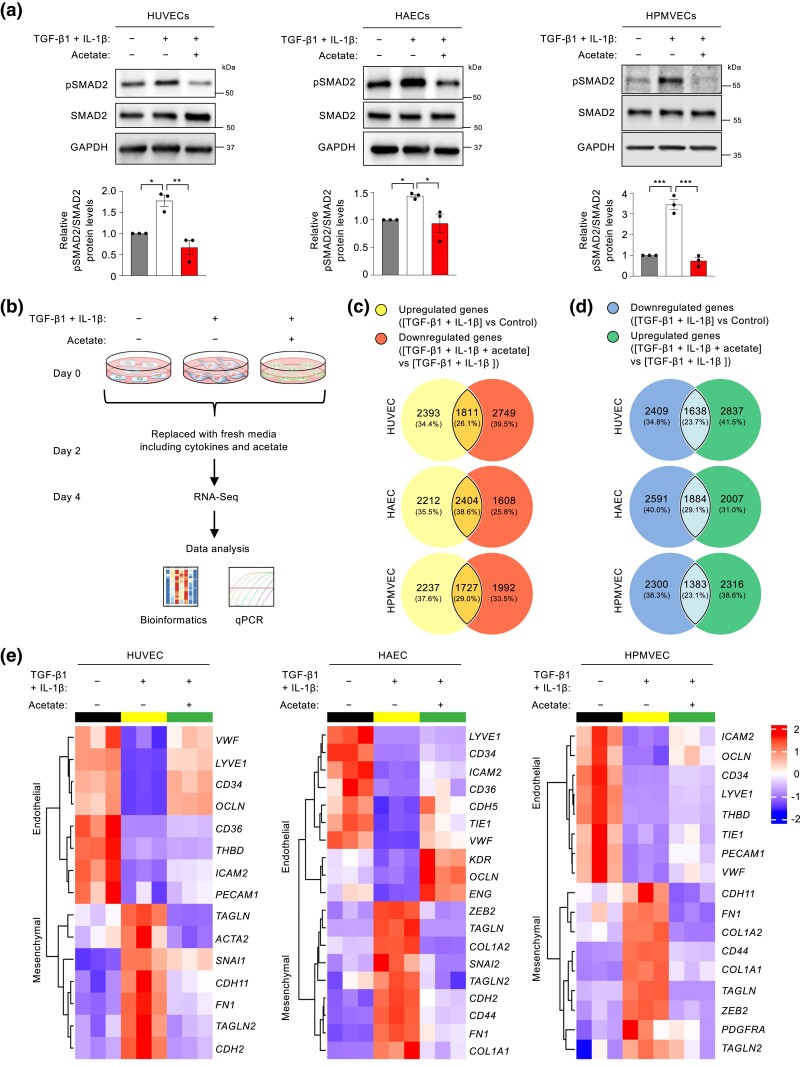
Acetate inhibits EndoMT programming. a) Representative western blots and quantifications of phosphorylated SMAD2 (pSMAD2) in the presence or absence of acetate following control or cytokine treatment (n = 3 independent experiments). b) Schematic overview of RNA-Seq analysis for endothelial cell gene expression in the presence or absence of acetate following control or cytokine treatment (n = 3 biological replicates). c and d) Venn diagrams showing common differentially expressed genes of cytokine-upregulated genes and acetate-suppressed genes (c), and of cytokine-downregulated genes and acetate-restored genes (d) (adjusted *P-*value < 0.05). e) Hierarchically clustered heatmaps of EndoMT marker expression negatively modulated by acetate.

Mounting evidence suggests that dysregulated metabolism is a hallmark of endothelial cell abnormalities (Xiong 2018b; Li et al. 2019). Our recent work demonstrates that the short-chain fatty acid acetate inhibits EndoMT phenotypes by regulating the TGF-β/SMAD2 signaling pathway (Xiong et al. 2018). Consistent with these findings, cytokine addition increased phosphorylation of the TGF-β downstream effector SMAD2, while acetate treatment substantially inhibited cytokine-stimulated SMAD2 activation in all 3 tissue- and vascular-bed-specific endothelial cells that are under evaluation in the current study (Fig. 4a). We also evaluated the effects of acetate on the transcriptome responses to these cells following cytokine treatment (Fig. 4b). Using adjusted *P*-value (< 0.05) selection, we identified differentially expressed genes between cells treated with cytokines only vs with acetate plus cytokines (Fig. 4c and d). Interestingly, our analyses showed that acetate treatment reversed the transcriptional expression of an appreciable fraction (40–50%) of the significantly altered genes in endothelial cells undergoing EndoMT in response to cytokine treatment (Fig. 4c and d). In particular, acetate blunted the induction of 2404 genes that were upregulated in cytokine-treated HAECs, accounting for ∼52% (calculated from the equation 2404/[2212 + 2404] or 38.6%/[35.5%+38.6%]) of all the upregulated genes in cytokine-treated HAECs (Fig. 4c). By contrast, acetate supplementation resulted in an increase in expression of 1884 genes that were downregulated by cytokine treatment in HAECs, accounting for ∼42% of all the downregulated genes in cytokine-treated HAECs (Fig. 4d). In this context, it is particularly intriguing that acetate inhibited the expression of many EndoMT markers, corroborating that acetate functions as a potent regulator of EndoMT across distinct vascular beds (Fig. 4e).

We applied a more stringent filtration criterion to examine how acetate negatively modulates the EndoMT-associated transcriptional expression patterns (adjusted *P*-value < 0.05 and Log2|fold-change| > 1). This analysis identified 619 genes (331 downregulated and 288 upregulated) in HUVECs, 1,063 genes (623 downregulated and 440 upregulated) in HAECs, and 712 genes (456 downregulated and 256 upregulated) in HPMVECs. Treating endothelial cells with acetate reversed these effects (Supplementary Fig. 5a and b, Table 4–6). When these genes were then subject to GO enrichment analysis, we found that the genes that were altered in HUVECs, HAECs, and HPMVECs shared the resulting biological process categories “leukocyte chemotaxis,” “leukocyte cell-cell adhesion,” and “leukocyte migration” (Supplementary Fig. 5c).

**Fig. 5.**
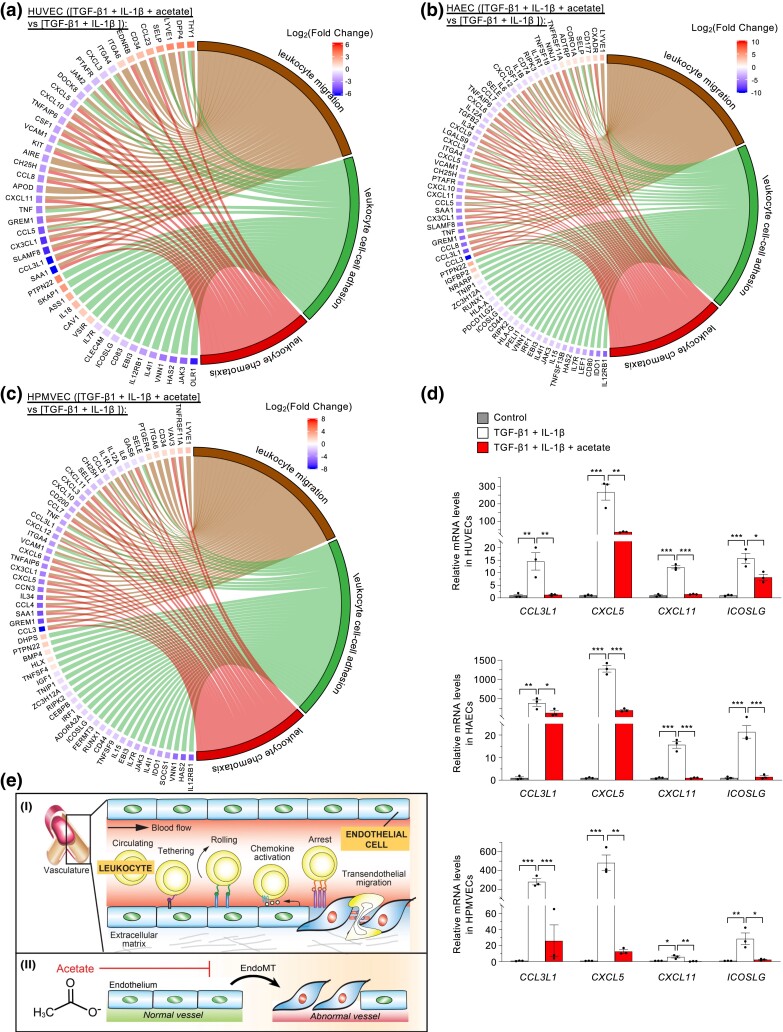
Acetate modulates EndoMT-associated leukocyte chemotaxis and related biological processes. a–c) Chord diagrams depicting the pairwise relationships between genes of the common GO biological process categories “leukocyte chemotaxis,” “leukocyte cell-cell adhesion,” and “leukocyte migration” that were shared by acetate-treated HUVECs (a), HAECs (b), and HPMVECs (c). d) Real-time qPCR analysis of the expression of common genes in GO categories “leukocyte chemotaxis,” “leukocyte cell-cell adhesion,” and “leukocyte migration” (n = 3 biological replicates). e) A model for how EndoMT promotes leukocyte chemotaxis and migration (I), which acetate can inhibit (II). Data represent mean ± SEM and significance via 1-way ANOVA with Tukey's multiple comparison test (d). *P < 0.05; **P < 0.01; ***P < 0.001.

We generated chord diagrams to visualize the pairwise relationships among genes of these 3 categories. We identified 24 genes shared by these common categories in HUVECs, HAECs, and HPMVECs (Fig. 5a–c, Supplementary Fig. 5d). This list encompassed 2 genes upregulated (*LYVE1* and *PTPN22*) and 22 genes downregulated by acetate (*CCL3L1*, *CCL5*, *CH25H*, *CX3CL1*, *CXCL10*, *CXCL11*, *CXCL3*, *CXCL5*, *EBI3*, *GREM1*, *HAS2*, *ICOSLG*, *IL12RB1*, *IL4I1*, *IL7R*, *ITGA4*, *JAK3*, *SAA1*, *TNF*, *TNFAIP6*, *VCAM1*, and *VNN1*). We then used a hierarchically clustered heatmap to display the expression patterns of these genes and validated several patterns using real-time quantitative reverse transcription PCR (qPCR) (Fig. 5d, Supplementary Fig. 5e). Based on these results, we propose a model in which EndoMT orchestrates multiple biological processes to boost leukocyte chemotaxis and transendothelial migration, which can be repressed, at least in part, by the metabolic regulator acetate (Fig. 5e).

Increasing evidence indicates cell-type differences and unique molecular signatures for endothelial cells isolated from distinct tissue types and vascular beds (Nolan et al. 2013; Jambusaria et al. 2020; Kalucka et al. 2020). The link to solute transporter gene expression is particularly intriguing since a previous report established that different endothelial cells exhibit specific SLC (solute carrier) transport gene expression patterns (Jambusaria et al. 2020). The SLC transporters function as gatekeepers of small compounds (e.g. nucleotides, inorganic ions, amino acids, and drugs) by coordinating their uptake and efflux by cells or organelles (Hediger et al. 2013). We identified 85 highly expressed genes (more than 1000 FPKM, on average) of the SLC gene series in our RNA-Seq results of EndoMT models. We performed a violin plot analysis to show the frequency distribution of gene expression data (Supplementary Fig. 6a). We also generated hierarchically clustered heatmaps to display the gene expression patterns of transporters (Supplementary Fig. 6b). These initial efforts suggested that EndoMT-linked HUVECs had a relatively higher median score (Supplementary Fig. 6a and 6b). There were more regulated genes in HAECs upon induction of EndoMT, most of whose expression was restored by acetate treatment (Supplementary Fig. 6b). The obscure role of SLC transporters in EndoMT heterogeneity may provide exciting potential for further investigation.

This study created a transcriptome atlas of EndoMT in typical types of tissue- and vascular-bed-specific human primary venous, arterial, and microvascular endothelial cells, including HUVECs, HAECs, and HPMVECs. Our results suggest that EndoMT potentially promotes leukocyte chemotaxis via a robust set of secreted factors. Fortunately, several biochemical tools, including BioID2 and TurboID, are now available to label secreted proteins and future studies will explore this in more details (Kim et al. 2021; Liu et al. 2021). Our data also suggest that genes driving EndoMT in cells from different vascular beds have a core set of common genes but there is also substantial cell-type difference. These findings are broadly consistent with recent reports showing transcriptional heterogeneity in endothelial cells across distinct tissues and vascular beds (Nolan et al. 2013; Jambusaria et al. 2020; Kalucka et al. 2020). Supporting this notion, several recent RNA-Seq datasets suggest EndoMT as a heterogeneous process driven by different internal and external factors (Monteiro et al. 2021; Stürzebecher et al. 2022; Zhu et al. 2023). Single-cell RNA-Seq clustering data in HUVECs shows that treatment of IL-1β only (Stürzebecher et al. 2022) or a combination of TGF-β2 and IL-1β (Monteiro et al. 2021) induces distinct cell populations undergoing EndoMT. High-depth bulk RNA-Seq results suggest glycose-related metabolic reprograming and loss of long noncoding RNA MIR503HG as major drivers of EndoMT in HUVECs and human pulmonary artery endothelial cells undergoing EndoMT after 7 days of cotreatment of TGF-β2 and IL-1β (Monteiro et al. 2021), and in human umbilical artery endothelial cells undergoing EndoMT after 7 days of TGF-β2 stimulation (Zhu et al. 2023), respectively.

The current work and previous studies were limited by a lack of evidence demonstrating how the EndoMT signature genes contribute to functionality. Nevertheless, our analysis suggests that the “lipid metabolism-centric,” “STAT1-centric,” and “SRC-centric” protein–protein interaction networks are likely central to unique EndoMT phenotypes of HUVECs, HAECs, and HPMVECs, respectively. Additional studies are required to solidify this conclusion. Our findings in Fig. 4, together with our recent protein expression data (Xiong et al. 2018), consolidate the idea that acetate could robustly and quickly inhibit EndoMT markers. Moreover, we cannot exclude that other mechanisms might contribute to this inhibitory role of acetate on EndoMT. For example, acetate might upregulate the expression of fibroblast growth factor receptor 1 (FGFR1), which has been reported as a key inhibitor of TGF-β signaling for preventing EndoMT (Chen et al. 2014). Indeed, our RNA-Seq results reveal that acetate significantly increased *FGFR1* gene expression in endothelial cells undergoing EndoMT (for HUVEC, 1.4-fold-change, adjusted *P-*value = 5.60 × 10^−28^; for HAECs, 2.2-fold-change, adjusted *P* value = 2.93 × 10^−64^; for HPMVECs, 1.7-fold-change, adjusted *P-*value = 1.10 × 10^−33^). Additionally, our results, together with another report (Zhu et al. 2023), suggest that the time factor is vital for the effects of acetate on EndoMT. Acetate inhibits EndoMT markers early (i.e. 4 days) after induction of EndoMT (Fig. 4e) but promotes EndoMT process after 7 days of TGF-β treatment alone (Zhu et al. 2023), potentially due to dynamic EndoMT-associated metabolism (Xiong et al. 2018). These studies suggest that a short-term dietary supplementation with acetate may be beneficial for preventing EndoMT-linked pathological conditions. On the contrary, long-term intake of a high-acetate diet can potentially exacerbate EndoMT-associated vascular inflammation. This time-dependent, opposing effects of acetate on EndoMT may merit further experimental evaluation. Given the growing number of pathological conditions associated with EndoMT, our observations suggest that endothelial transcriptome may have therapeutic implications for a wide range of EndoMT-linked diseases.

## Supplementary Material

jkad243_Supplementary_Data

## Data Availability

The RNA-Seq data were deposited in the NCBI's Gene Expression Omnibus (GEO) repository with the accession number GSE236730. Supplemental material available at G3 online.
