# Supplementary material for: Transcriptome analysis reveals molecular signature and cell-type difference of Homo sapiens endothelial-to-mesenchymal transition
Source: G3 (Bethesda). 2023 Oct 20;13(12):jkad243. doi: 10.1093/g3journal/jkad243 (PMC10700110; doi:10.1093/g3journal/jkad243)
Supplement: jkad243_Supplementary_Data [file jkad243_supplementary_data.zip › Supplemental_Material_G3-2023-404591.pdf]

Supplementary figure 1

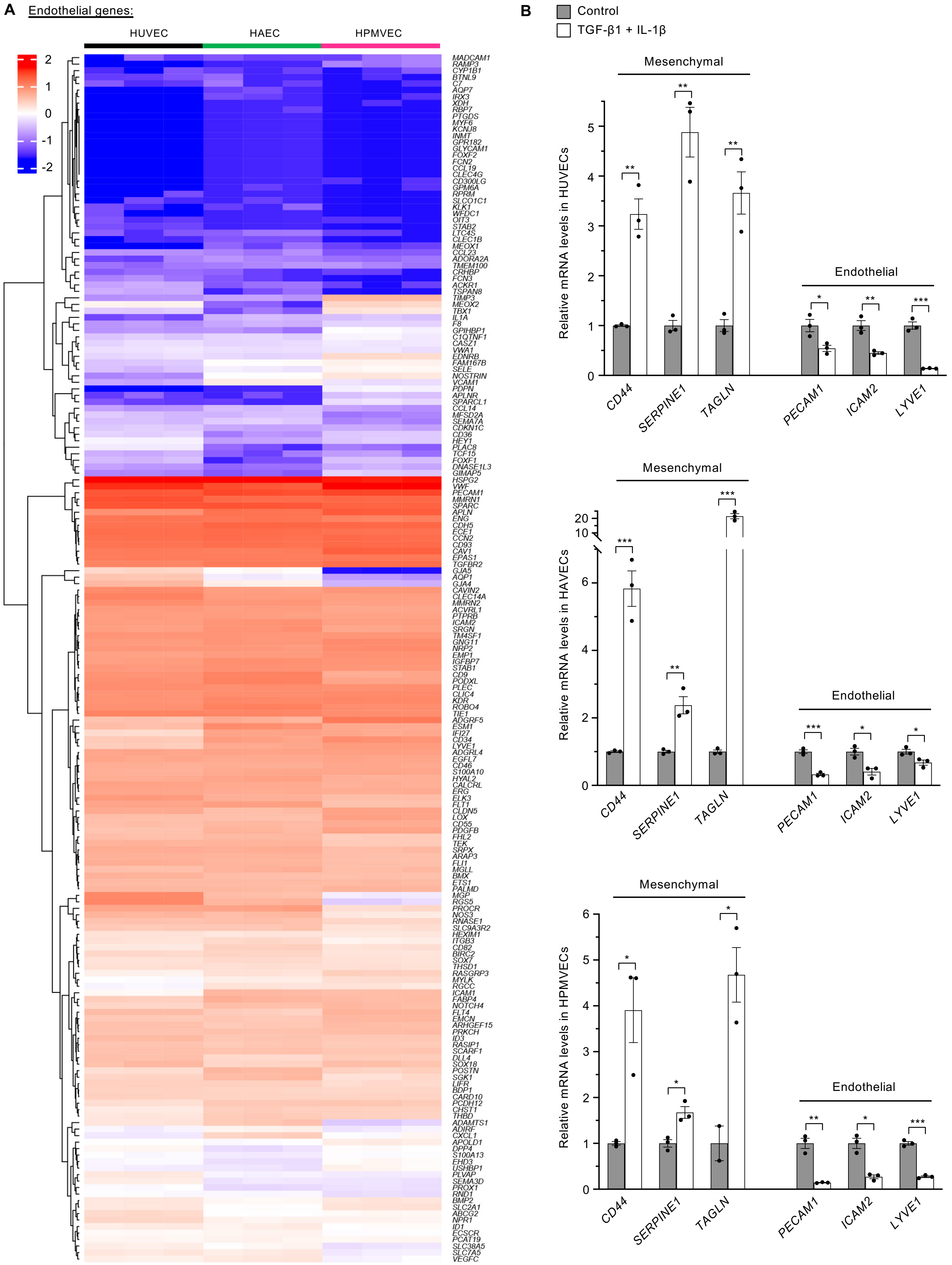

Supplementary figure 2

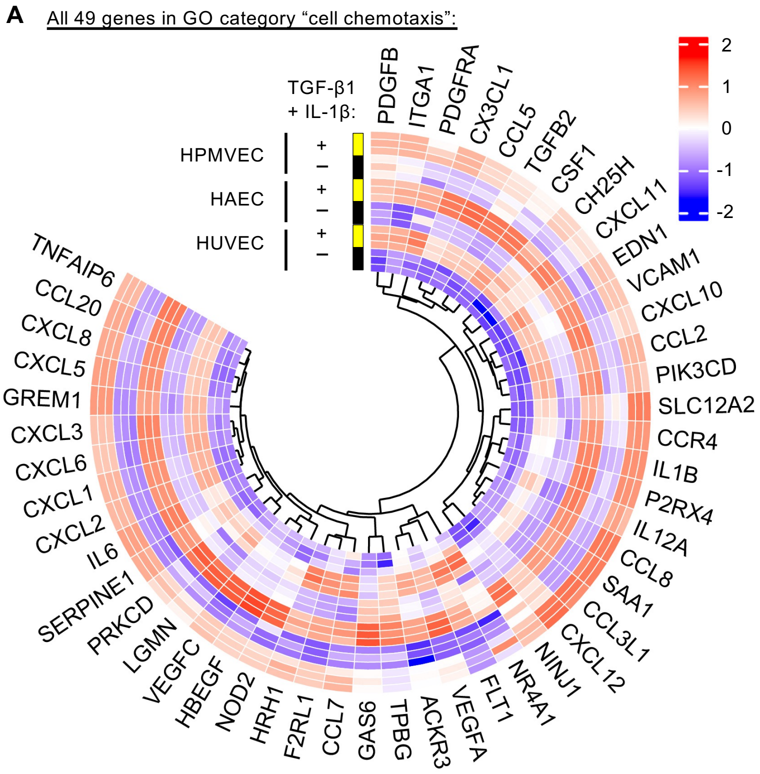

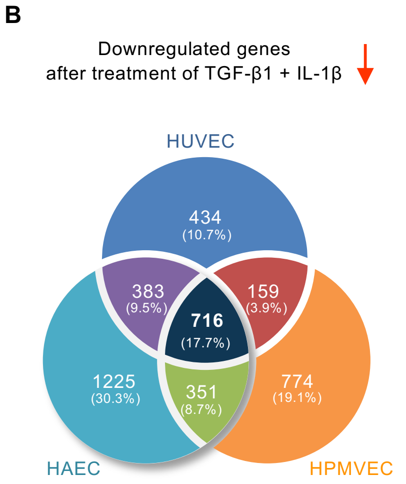

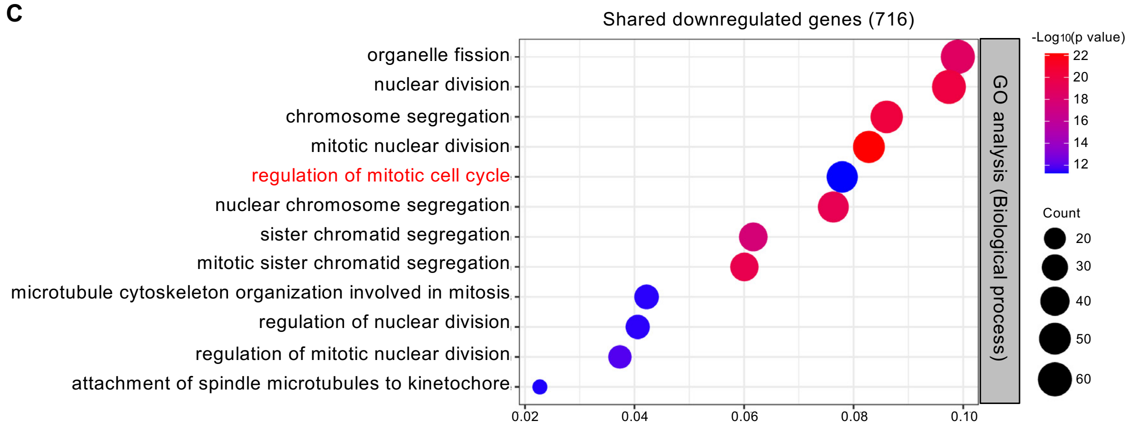

Supplementary figure 3

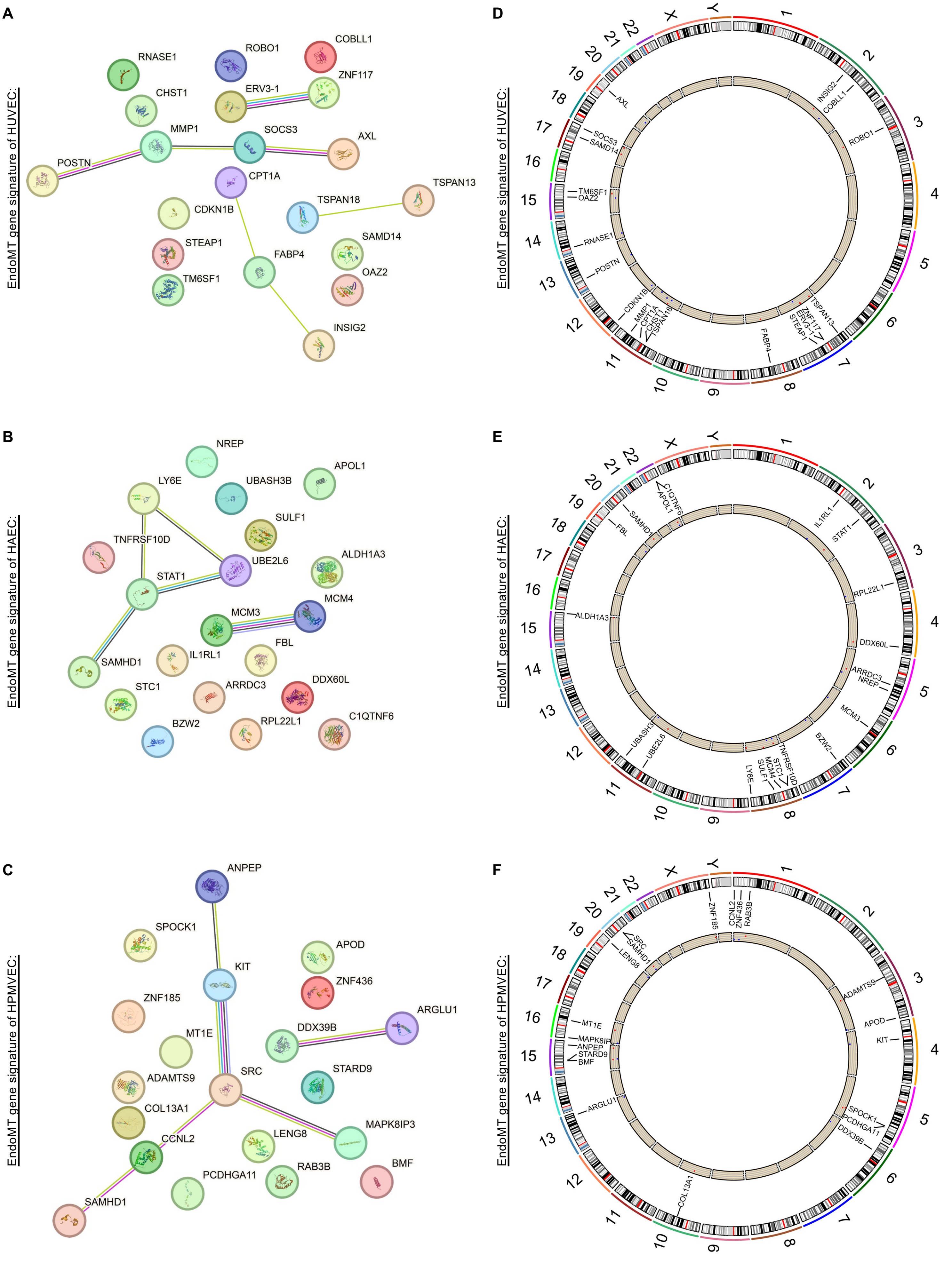

Supplementary figure 4

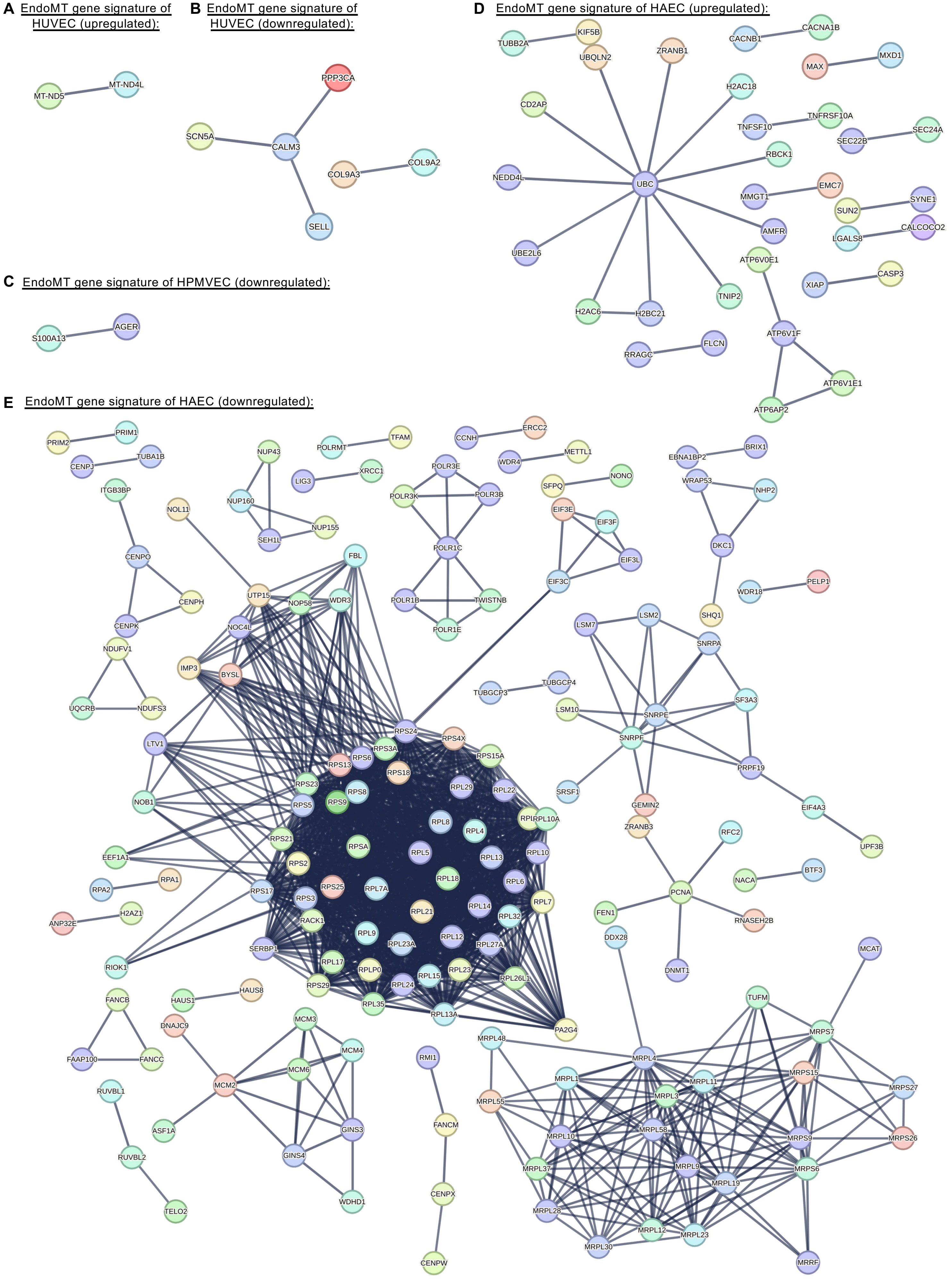

Supplementary figure 5

A

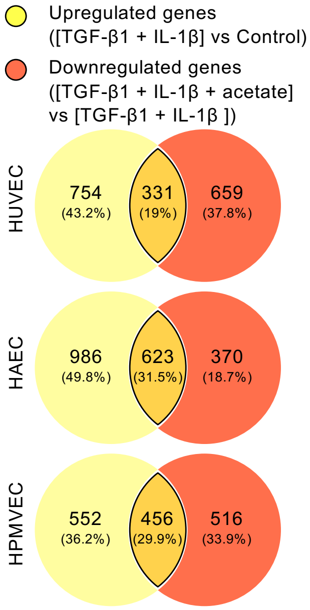

C

HUVEC

ONTOLOGY	ID	Description	p.adjust	qvalue	Count	zscore
BP	GO:0050900	leukocyte migration	9.507E-06	6.946E-06	31	-2.69407953
BP	GO:0007159	leukocyte cell-cell adhesion	0.0001857	0.00013568	27	-1.73205081
BP	GO:0030595	leukocyte chemotaxis	0.0013059	0.00095412	18	-2.82842712

HAEC

ONTOLOGY	ID	Description	p.adjust	qvalue	Count	zscore
BP	GO:0050900	leukocyte migration	4.1108E-06	3.1153E-06	42	-4.3204938
BP	GO:0007159	leukocyte cell-cell adhesion	4.0476E-06	3.0674E-06	42	-4.3204938
BP	GO:0030595	leukocyte chemotaxis	1.2303E-05	9.3237E-06	30	-4.3817805

HPMVEC

ONTOLOGY	ID	Description	p.adjust	qvalue	Count	zscore
BP	GO:0050900	leukocyte migration	2.14413E-06	1.6078E-06	34	-3.7729689
BP	GO:0007159	leukocyte cell-cell adhesion	2.98693E-07	2.2398E-07	36	-4.3333333
BP	GO:0030595	leukocyte chemotaxis	3.71303E-05	2.7843E-05	23	-3.9617739

B

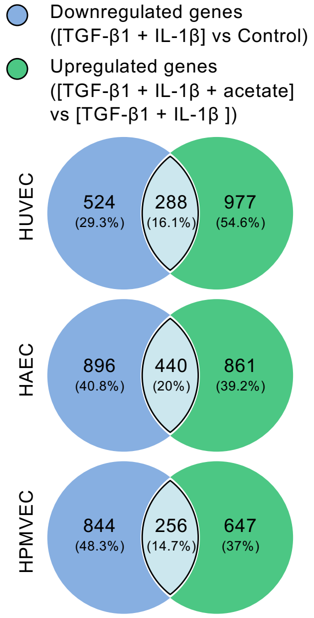

D

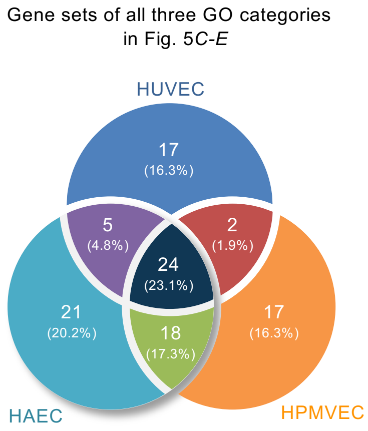

E

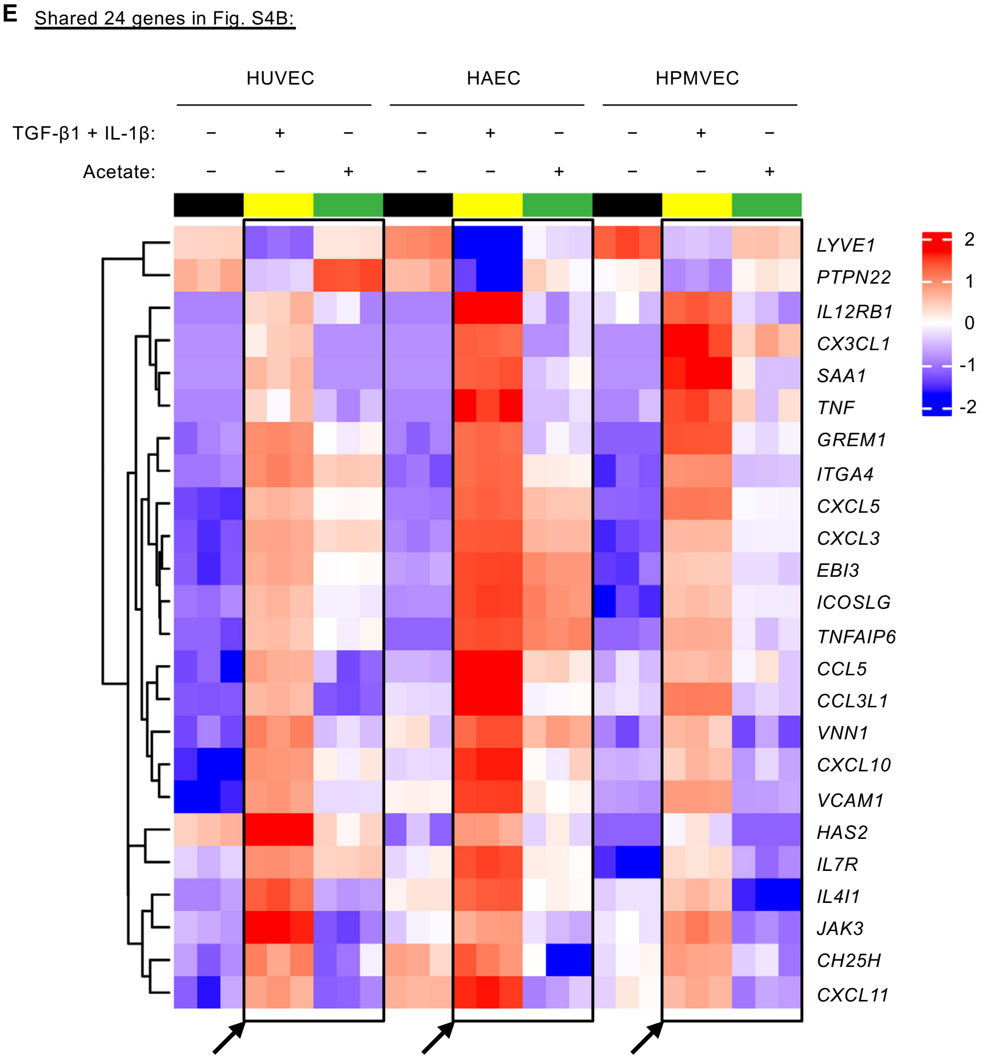

Supplementary figure 6

A 85 highly-expressed SLC transporter genes:

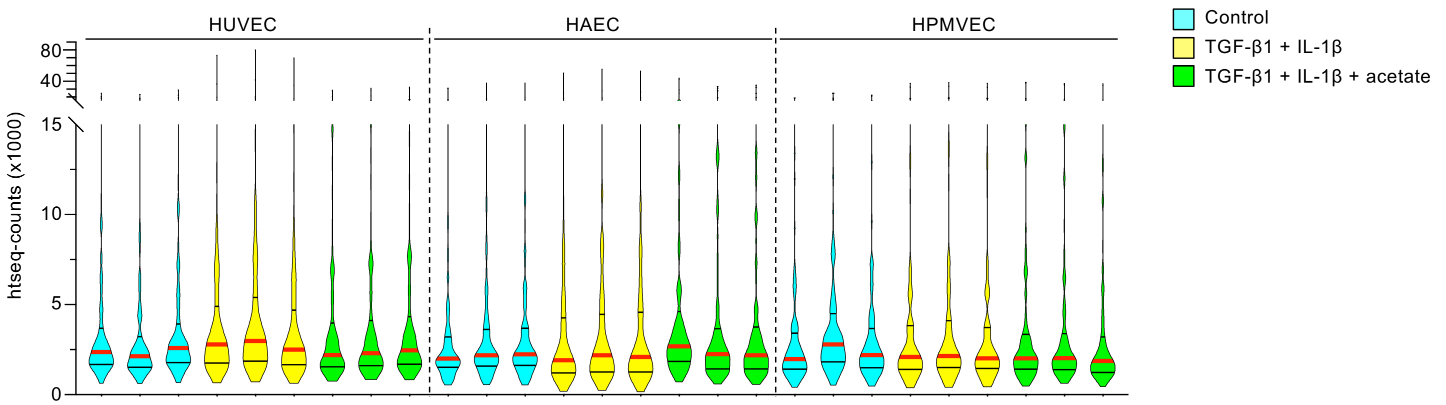

B 85 highly-expressed SLC transporter genes:

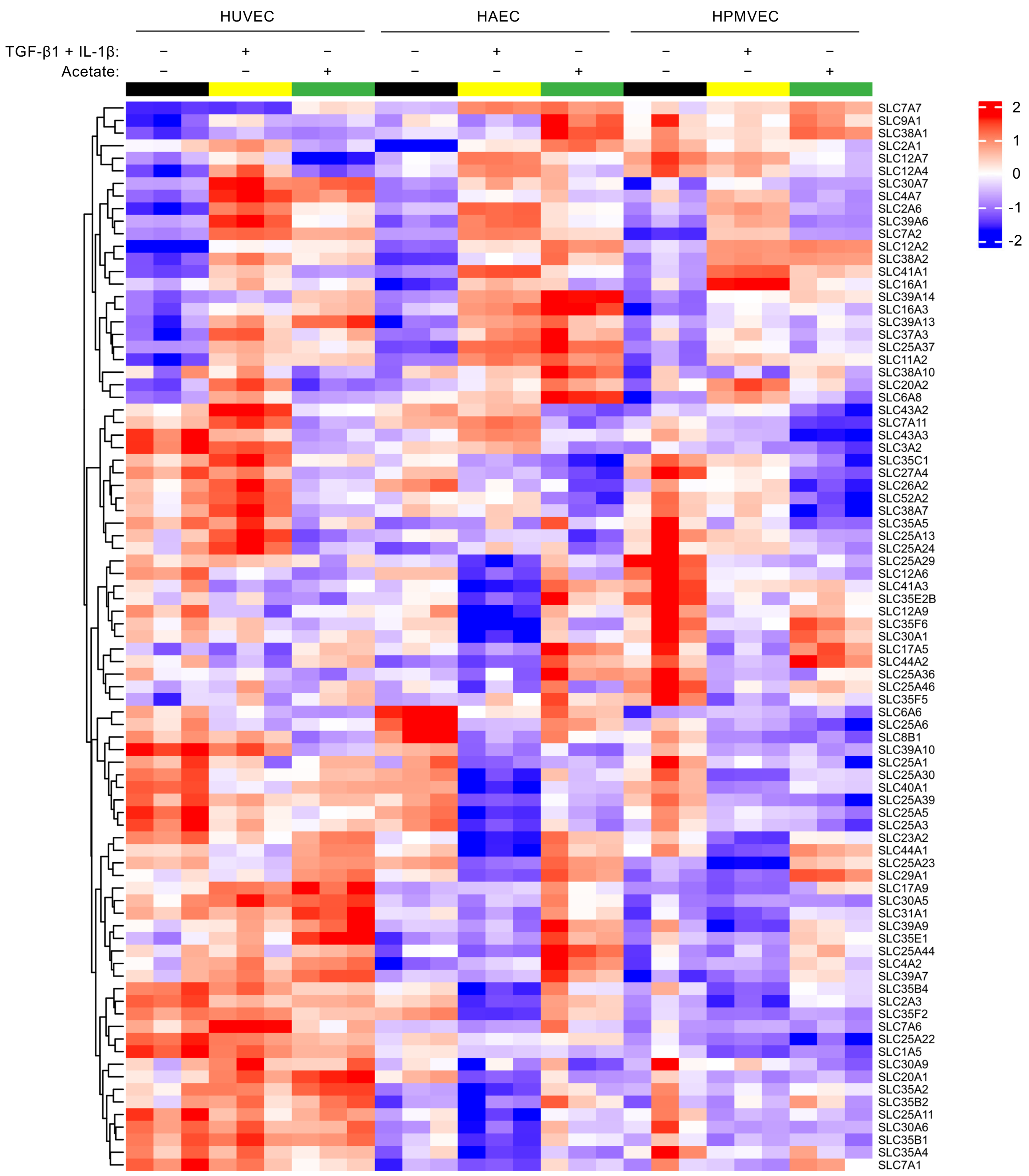

Table S1: RNA-Seq analysis of the differentially expressed genes in HUVECs with or without cytokine treatment (adjusted *p* value < 0.05 and Log2|Fold Change| > 0.5).

EnsemblID	GeneSymbol	baseMean	baseMean_Control	baseMean_TGF-ß1 + IL-1ß	log2FoldChange	lfcSE	stat	pvalue	padj
ENSG00000166920	C15orf48	272.439145	0.6089016	544.2693885	9.736389247	1.027598753	9.47489399	2.67E-21	2.53E-20
ENSG00000041982	TNC	759.4234004	1.9050442	1516.941757	9.630240985	0.593474347	16.2268867	3.26E-59	7.12E-58
ENSG00000203685	STUM	127.3101368	0.294802	254.3254716	9.530362852	1.189965143	8.00894287	1.16E-15	8.74E-15
ENSG00000163673	DCLK3	124.4847298	0.294802	248.6746577	9.498113467	1.190475333	7.97842106	1.48E-15	1.11E-14
ENSG00000285744	AC083837.1	105.2148206	0.3538437	210.0757975	9.2544359	1.192576125	7.76003787	8.49E-15	6.20E-14
ENSG00000154451	GBP5	101.9972029	0.3538437	203.640562	9.20982177	1.192968639	7.72008708	1.16E-14	8.40E-14
ENSG00000261618	LINC02605	43.89585333	0	87.79170666	8.958551114	1.213146245	7.3845599	1.53E-13	1.05E-12
ENSG00000164400	CSF2	1135.273475	5.2670979	2265.279851	8.706686584	0.355057883	24.5218794	8.63E-133	4.13E-131
ENSG00000123610	TNFAIP6	130.1008243	0.6679433	259.5337053	8.668052991	1.035182287	8.37345567	5.60E-17	4.48E-16
ENSG00000125730	C3	1268.723187	6.4224134	2531.023962	8.605574817	0.832352588	10.3388575	4.70E-25	5.06E-24
ENSG00000166923	GREM1	186.1623581	0.9434476	371.3812686	8.600544432	0.845019541	10.1779237	2.49E-24	2.62E-23
ENSG00000138944	SHISAL1	31.8992945	0	63.79858901	8.49577347	1.241606309	6.84256628	7.78E-12	4.89E-11
ENSG00000124875	CXCL6	14432.68686	80.630513	28784.74321	8.461662129	0.093477076	90.5212539	0	0
ENSG00000124102	PI3	110.0586894	0.6679433	219.4494355	8.424529388	1.035853721	8.13293346	4.19E-16	3.22E-15
ENSG00000236453	AC003092.1	29.54995754	0	59.09991508	8.385614086	1.229756508	6.81892231	9.17E-12	5.74E-11
ENSG00000173391	OLR1	57.19195816	0.3140996	114.0698167	8.37404311	1.20460057	6.95171771	3.61E-12	2.31E-11
ENSG00000169245	CXCL10	144.530419	0.982043	288.078795	8.232091081	0.848501888	9.70191251	2.96E-22	2.91E-21
ENSG00000050730	TNIP3	604.3316423	3.8903186	1204.772966	8.18349999	0.40831973	20.0418921	2.38E-89	8.10E-88
ENSG00000222000	AC092675.1	24.64063911	0	49.28127821	8.124415683	1.248929869	6.5051016	7.76E-11	4.58E-10
ENSG00000110848	CD69	394.6330937	2.7508547	786.5153326	8.098459134	0.491490822	16.4773354	5.34E-61	1.20E-59
ENSG00000007908	SELE	14315.31626	103.28107	28527.35144	8.091330901	0.083455839	96.9534428	0	0
ENSG00000126549	STATH	22.74880072	0	45.49760143	8.007115838	1.246796568	6.42215101	1.34E-10	7.81E-10
ENSG00000081041	CXCL2	3804.946029	30.354071	7579.537987	7.966021456	0.152459087	52.250224	0	0
ENSG00000175445	LPL	573.1921781	4.8534704	1141.530886	7.898245577	0.384771587	20.5271019	1.23E-93	4.40E-92
ENSG00000145649	GZMA	20.92153942	0	41.84307885	7.887310178	1.250109128	6.30929732	2.80E-10	1.59E-09
ENSG00000163734	CXCL3	1572.120881	13.182077	3131.059685	7.86884913	0.229038752	34.3559728	1.15E-258	1.16E-256
ENSG00000168398	BDKRB2	39.46177257	0.3538437	78.56970146	7.835453255	1.217403127	6.43620267	1.22E-10	7.12E-10
ENSG00000163121	NEURL3	38.68434747	0.3140996	77.0545953	7.808892957	1.217600541	6.41334551	1.42E-10	8.25E-10
ENSG00000137462	TLR2	214.0614226	1.9061929	426.2166522	7.79970938	0.604782317	12.896722	4.70E-38	7.02E-37
ENSG00000163735	CXCL5	1771.811579	16.090017	3527.533141	7.788645547	0.211218925	36.8747524	1.17E-297	1.37E-295
ENSG00000108342	CSF3	6653.992171	61.575547	13246.4088	7.748928989	0.108363558	71.5086246	0	0
ENSG00000038427	VCAN	1920.026677	19.21442	3820.838934	7.663295193	0.196125787	39.0733686	0	0
ENSG00000183813	CCR4	34.32296228	0.294802	68.3511226	7.634046697	1.224535485	6.23423885	4.54E-10	2.54E-09
ENSG00000233930	KRTAP5-AS1	17.17130274	0	34.34260548	7.606415022	1.270398086	5.98742639	2.13E-09	1.14E-08
ENSG00000198771	RCSD1	30.14652829	0.3140996	59.97895694	7.443268805	1.235055182	6.02666902	1.67E-09	8.99E-09
ENSG00000163739	CXCL1	27816.96168	337.72129	55296.20208	7.3544869	0.049264761	149.28494	0	0
ENSG00000170054	SERPINA9	13.59521265	0	27.19042529	7.266749126	1.294183264	5.61493053	1.97E-08	9.78E-08
ENSG00000115009	CCL20	143.6724979	1.8857465	285.4592493	7.220952228	0.610417552	11.8295292	2.75E-32	3.58E-31
ENSG00000112299	VNN1	24.39381543	0.3538437	48.43378718	7.134868998	1.245118652	5.73027236	1.00E-08	5.10E-08
ENSG00000178860	MSC	24.14563522	0.294802	47.99646848	7.125703244	1.240395517	5.74470252	9.21E-09	4.70E-08
ENSG00000169429	CXCL8	19264.76762	312.15146	38217.38377	6.931005831	0.051919249	133.495881	0	0
ENSG00000248371	LINC02056	37.38449884	0.5896039	74.17939376	6.86475928	1.068325409	6.42571937	1.31E-10	7.63E-10
ENSG00000213886	UBD	10.20094923	0	20.40189846	6.851122059	1.323345896	5.17712118	2.25E-07	1.02E-06
ENSG00000271503	CCL5	55.78126343	1.021787	110.5407399	6.8463819	0.873844874	7.8347795	4.70E-15	3.47E-14
ENSG00000203635	AC144450.1	18.25364585	0.3140996	36.19319206	6.719427464	1.2689139	5.29541639	1.19E-07	5.53E-07
ENSG00000261239	ANKRD26P1	50.48492159	0.9037036	100.0661396	6.713684893	0.881964649	7.61219274	2.69E-14	1.92E-13
ENSG00000185101	ANO9	17.89232493	0.3140996	35.47055022	6.693902807	1.287706956	5.19831222	2.01E-07	9.17E-07
ENSG00000042062	RIPOR3	8.670017934	0	17.34003587	6.620583661	1.351468315	4.8988079	9.64E-07	4.13E-06
ENSG00000259342	AC025580.1	8.644591272	0	17.28918254	6.617031544	1.354111885	4.88662098	1.03E-06	4.38E-06
ENSG00000164761	TNFRSF11B	535.5696065	10.90504	1060.234173	6.570508367	0.25835635	25.4319601	1.12E-142	5.71E-141
ENSG00000173432	SAA1	8.373224387	0	16.74644877	6.564112455	1.357854631	4.83417908	1.34E-06	5.66E-06
ENSG00000105246	EBI3	424.6223475	8.7812357	840.4634593	6.556794739	0.289209883	22.6714062	8.58E-114	3.51E-112
ENSG00000227507	LTB	168.5565719	3.8521298	333.2610139	6.443881462	0.44370644	14.5228486	8.68E-48	1.58E-46
ENSG00000253522	MIR3142HG	229.4651184	5.2057588	453.724478	6.388826414	0.378854726	16.8635257	8.35E-64	1.97E-62
ENSG00000226067	LINC00623	7.381716182	0	14.76343236	6.38061828	1.388553081	4.59515619	4.32E-06	1.73E-05
ENSG00000261040	WFDC21P	7.038720923	0	14.07744185	6.317492471	1.381769309	4.57203126	4.83E-06	1.93E-05
ENSG00000042980	ADAM28	63.39043272	1.5716469	125.2092186	6.294860673	0.688063834	9.14865796	5.76E-20	5.19E-19
ENSG00000148848	ADAM12	587.9911797	15.152719	1160.82964	6.274258624	0.492119008	12.7494743	3.14E-37	4.57E-36
ENSG00000224429	LINC00539	6.808953858	0	13.61790772	6.265625794	1.397913079	4.48212831	7.39E-06	2.89E-05
ENSG00000153823	PID1	6.718420734	0	13.43684147	6.249330008	1.406516277	4.44312669	8.87E-06	3.44E-05
ENSG00000184530	C6orf58	36.72000216	0.982043	72.45796136	6.241367411	0.891541878	7.00064413	2.55E-12	1.64E-11
ENSG00000166927	MS4A7	6.456241542	0	12.91248308	6.195481039	1.410097273	4.39365507	1.11E-05	4.29E-05
ENSG00000276085	CCL3L1	6.400388586	0	12.80077717	6.178386488	1.415475437	4.36488428	1.27E-05	4.86E-05
ENSG00000179362	HMGN2P46	6.169628604	0	12.33925721	6.127933737	1.422070835	4.30916209	1.64E-05	6.19E-05
ENSG00000230067	HSPD1P6	12.00128116	0.3140996	23.68846268	6.106654906	1.302400807	4.68876775	2.75E-06	1.13E-05
ENSG00000162692	VCAM1	1890.055892	54.129385	3725.9824	6.094919526	0.122353269	49.8141125	0	0
ENSG00000182326	C1S	286.1844761	8.6469652	563.721987	6.082068378	0.30576185	19.8915214	4.82E-88	1.62E-86
ENSG00000115008	IL1A	120.1729005	3.5959232	236.7498778	6.073726812	0.475017829	12.7863134	1.96E-37	2.86E-36
ENSG00000125538	IL1B	190.7435537	5.4812631	376.0058444	6.037183401	0.368588642	16.37919	2.69E-60	5.97E-59
ENSG00000187689	AMTN	520.0206657	15.91215	1024.129182	6.031026398	0.224385773	26.8779358	3.98E-159	2.32E-157
ENSG00000080224	EPHA6	11.3166998	0.294802	22.33859763	6.021795624	1.320993678	4.55853478	5.15E-06	2.04E-05
ENSG00000160224	AIRE	5.620024903	0	11.24004981	5.994815819	1.441517501	4.15868404	3.20E-05	0.000117
ENSG00000096996	IL12RB1	5.595591157	0	11.19118231	5.981963839	1.442901801	4.14578721	3.39E-05	0.000124
ENSG00000198732	SMOC1	20.2731689	0.6089016	39.9374362	5.972476948	1.111063064	5.3754617	7.64E-08	3.62E-07
ENSG00000169436	COL22A1	10.6423183	0.3538437	20.93079293	5.923964551	1.319024187	4.49117204	7.08E-06	2.78E-05
ENSG00000228613	AC141930.1	10.32484357	0.294802	20.35488518	5.890543336	1.328297054	4.43465813	9.22E-06	3.58E-05
ENSG00000179148	ALOXE3	5.130991583	0	10.26198317	5.862410339	1.451708257	4.03828408	5.38E-05	0.000192
ENSG00000186827	TNFRSF4	65.09828852	2.1793998	128.0171772	5.842076737	0.600068574	9.73568187	2.12E-22	2.09E-21
ENSG00000235531	MSC-AS1	5.050523535	0	10.10104707	5.835421246	1.475774002	3.95414287	7.68E-05	0.000267
ENSG00000144191	CNGA3	9.988650028	0.3140996	19.66320041	5.832756251	1.342558431	4.3445083	1.40E-05	5.31E-05

ENSG00000182050	MGAT4C	9.891199656	0.294802	19.48759735	5.826056277	1.323833846	4.40089691	1.08E-05	4.16E-05
ENSG00000189292	ALKAL2	4.689550631	0	9.379101263	5.737394742	1.497593746	3.83107552	0.000128	0.000432
ENSG00000158714	SLAMF8	16.98114391	0.5896039	33.37268389	5.718080761	1.123707609	5.08858418	3.61E-07	1.61E-06
ENSG00000056558	TRAF1	831.434016	31.459199	1631.408833	5.718041686	0.163642576	34.9422616	1.70E-267	1.80E-265
ENSG00000151025	GPR158	4.504644342	0	9.009288685	5.671331179	1.486295955	3.81574824	0.000136	0.000459
ENSG00000100365	NCF4	8.751848902	0.294802	17.20889584	5.64469496	1.374025332	4.10814475	3.99E-05	0.000144
ENSG00000135373	EHF	4.377511031	0	8.755022062	5.636975084	1.513381082	3.72475588	0.000196	0.000646
ENSG00000235947	EGOT	16.1065675	0.6281993	31.58493572	5.631156707	1.137674641	4.9497075	7.43E-07	3.22E-06
ENSG00000113645	WWC1	24.17874081	0.9627453	47.39473633	5.629522127	0.916552516	6.14206172	8.15E-10	4.47E-09
ENSG00000135378	PRRG4	16.00893338	0.6281993	31.38966748	5.621013399	1.126684654	4.9889855	6.07E-07	2.65E-06
ENSG00000095752	IL11	23.74373429	0.9037036	46.58376501	5.613347961	0.924480748	6.07189276	1.26E-09	6.85E-09
ENSG00000229647	MYOSLID	4.267891813	0	8.535783626	5.596962904	1.601696052	3.49439764	0.000475	0.001481
ENSG00000080709	KCNN2	212.7694509	8.7634933	416.7754086	5.594763722	0.308377997	18.1425516	1.47E-73	3.94E-72
ENSG00000213940	AC026477.1	8.373989726	0.3140996	16.43387981	5.579862612	1.355901903	4.11524064	3.87E-05	0.00014
ENSG00000003989	SLC7A2	39278.26246	1612.2714	76944.25356	5.574183973	0.030942907	180.144158	0	0
ENSG00000006210	CX3CL1	1101.018552	46.152732	2155.884373	5.551836302	0.134961172	41.136545	0	0
ENSG00000182752	PAPPA	560.3800323	23.989144	1096.770921	5.517323498	0.186767724	29.5410972	8.55E-192	6.02E-190
ENSG00000283265	AL356234.3	14.96747331	0.6679433	29.2670033	5.51555295	1.13306222	4.86782884	1.13E-06	4.80E-06
ENSG00000283399	AC004381.2	4.050754741	0	8.101509483	5.514363997	1.550312301	3.55693752	0.000375	0.001188
ENSG00000286201	AC011751.1	3.85513848	0	7.710276959	5.444603386	1.575554486	3.45567445	0.000549	0.001697
ENSG00000148346	LCN2	3.85287044	0	7.705740879	5.443290197	1.542459629	3.52896769	0.000417	0.001311
ENSG00000163362	INAVA	3.827443777	0	7.654887554	5.435299953	1.537695816	3.53470426	0.000408	0.001284
ENSG00000226835	AC097059.1	3.823719269	0	7.647438538	5.434363988	1.550336382	3.50528056	0.000456	0.001425
ENSG00000108700	CCL8	7.510610154	0.294802	14.72641835	5.426716055	1.389785132	3.90471587	9.43E-05	0.000325
ENSG00000259867	AC105411.1	3.78630751	0	7.572615019	5.422361609	1.536821523	3.52829625	0.000418	0.001314
ENSG00000281571	AC241585.2	3.741446734	0	7.482893467	5.408193032	1.552994914	3.48242804	0.000497	0.001545
ENSG00000232810	TNF	3.728998084	0	7.457996169	5.39327721	1.579093583	3.41542596	0.000637	0.00195
ENSG00000104043	ATP8B4	7.234638972	0.3140996	14.1551783	5.362148933	1.383778634	3.87500486	0.000107	0.000365
ENSG00000114646	CSPG5	127.1228509	5.9701906	248.2755112	5.35922341	0.378559446	14.1568873	1.69E-45	2.95E-44
ENSG00000162873	KLHDC8A	3.598140265	0	7.196280529	5.350074543	1.568362197	3.41124936	0.000647	0.001979
ENSG00000073756	PTGS2	9658.414297	465.37178	18851.45682	5.339714707	0.047284436	112.927532	0	0
ENSG00000103522	IL21R	3.595408672	0	7.190817344	5.339116269	1.645646484	3.24438834	0.001177	0.003437
ENSG00000259354	AC025580.2	7.07835449	0.3140996	13.84260934	5.335673316	1.386639575	3.84791651	0.000119	0.000406
ENSG00000128342	LIF	98.62943238	4.7728337	192.4860311	5.329780855	0.421898081	12.6328635	1.39E-36	1.99E-35
ENSG00000166523	CLEC4E	6.932779981	0.3140996	13.55146032	5.30390394	1.397941688	3.79408096	0.000148	0.000498
ENSG00000172986	GXYLT2	83.55205449	4.2638665	162.8402425	5.291843045	0.453674738	11.6643987	1.94E-31	2.49E-30
ENSG00000197859	ADAMTSL2	25.03932329	1.2178032	48.86084338	5.267727857	0.820470029	6.42037816	1.36E-10	7.89E-10
ENSG00000139044	B4GALNT3	68.29137433	3.3597563	133.2229923	5.252214651	0.497257491	10.5623641	4.45E-26	4.96E-25
ENSG00000164690	SHH	43.02899898	2.1998462	83.85815178	5.231011914	0.621056445	8.42276408	3.68E-17	2.96E-16
ENSG00000224396	METTL15P3	3.321244383	0	6.642488767	5.229981727	1.618257922	3.23185918	0.00123	0.003581
ENSG00000228741	AL445985.1	12.22949423	0.7076874	23.7513011	5.199658606	1.15989716	4.48286175	7.36E-06	2.89E-05
ENSG00000205057	CLLU1OS	3.207089085	0	6.41417817	5.175804595	1.673348774	3.09308177	0.001981	0.005573
ENSG00000108691	CCL2	23449.4619	1279.0395	45619.88436	5.157952063	0.034814527	148.155167	0	0
ENSG00000213512	GBP7	3.101657928	0	6.203315855	5.13468226	1.66717454	3.07987084	0.002071	0.005808
ENSG00000137571	SLCO5A1	3.093397339	0	6.186794678	5.131084141	1.608620988	3.18974089	0.001424	0.004096
ENSG00000151023	ENKUR	141.9948418	7.8786808	276.1110028	5.114827831	0.333575935	15.3333238	4.58E-53	9.06E-52
ENSG00000254143	AC022733.2	3.029102449	0	6.058204898	5.105539588	1.682978001	3.03363418	0.002416	0.006694
ENSG00000227908	AC016596.1	6.079371619	0.3538437	11.80489956	5.099107578	1.420005623	3.59090661	0.00033	0.001052
ENSG00000171631	P2RY6	67.30159552	3.9100228	130.6931682	5.08838806	0.49363477	10.3080017	6.48E-25	6.96E-24
ENSG00000115232	ITGA4	2263.509017	130.44477	4396.573267	5.074678622	0.087082231	58.2745593	0	0
ENSG00000165323	FAT3	206.5558557	12.084654	401.0270572	5.04815285	0.271465319	18.5959403	3.47E-77	9.84E-76
ENSG00000144802	NFKBIZ	2385.940273	140.64327	4631.237278	5.037888204	0.083433787	60.3818714	0	0
ENSG00000115919	KYNU	66.00386026	3.8517232	128.1559973	5.033730056	1.22257003	4.11733474	3.83E-05	0.000139
ENSG00000005102	MEOX1	2.880796348	0	5.761592696	5.018671175	1.728523066	2.90344472	0.003691	0.009821
ENSG00000248458	AL139147.1	36.91584258	2.2395902	71.59209495	4.999363165	0.642453426	7.78167407	7.16E-15	5.25E-14
ENSG00000090339	ICAM1	13145.28931	805.35292	25485.2257	4.983221095	0.040706129	122.419429	0	0
ENSG00000179826	MRGPRX3	2.797067344	0	5.594134688	4.98192109	1.668121294	2.98654607	0.002821	0.007699
ENSG00000227517	LINC01483	10.36398794	0.6486456	20.07933024	4.971442344	1.175573712	4.22894991	2.35E-05	8.73E-05
ENSG00000256448	AP000763.3	2.769372642	0	5.538745284	4.969605592	1.665867706	2.98319343	0.002853	0.007779
ENSG00000253227	AC090192.2	5.442869418	0.3140996	10.57163919	4.936576815	1.463408965	3.37334056	0.000743	0.002245
ENSG00000163082	SGPP2	5.424959989	0.294802	10.55511802	4.934123856	1.447328048	3.40912612	0.000652	0.001993
ENSG00000156219	ART3	2.657485384	0	5.314970767	4.904975164	1.703185478	2.87988315	0.003978	0.010508
ENSG00000258183	LINC02392	5.29183413	0.294802	10.2888663	4.903600921	1.444886419	3.39376221	0.000689	0.002096
ENSG00000112096	SOD2	25256.07266	1634.5977	48877.54759	4.903136494	0.032428581	151.197996	0	0
ENSG00000132185	FCRLA	24.33713429	1.4933075	47.18096108	4.901707717	0.764507889	6.41158553	1.44E-10	8.34E-10
ENSG00000125726	CD70	2.632058721	0	5.264117443	4.893134209	1.687136191	2.90026036	0.003729	0.009914
ENSG00000128285	MCHR1	9.709416631	0.6486456	18.77018762	4.881504941	1.208872305	4.038065	5.39E-05	0.000192
ENSG00000099869	IGF2-AS	2.561771283	0	5.123542566	4.860760608	1.714744958	2.8346843	0.004587	0.011972
ENSG00000233705	SLC26A4-AS1	2.530352072	0	5.060704145	4.845830771	1.713478467	2.82806634	0.004683	0.012193
ENSG00000258860	AL133163.2	2.467050099	0	4.934100197	4.798629846	1.719834418	2.79016968	0.005268	0.01357
ENSG00000151883	PARP8	13.79973672	0.982043	26.61743048	4.795847273	0.994569832	4.82203172	1.42E-06	6.00E-06
ENSG00000197320	AC060834.1	2.369067958	0	4.738135916	4.74946676	1.778567085	2.67038944	0.007576	0.018873
ENSG00000237988	OR2I1P	2.355626393	0	4.711252785	4.743213773	1.743407162	2.7206575	0.006515	0.016478
ENSG00000159261	CLDN14	770.1934157	58.102302	1482.284529	4.673390264	0.131433359	35.5571088	6.45E-277	7.15E-275
ENSG00000243225	AC007686.1	2.241471094	0	4.482942189	4.665463782	1.75042168	2.66533706	0.007691	0.019133
ENSG00000250331	LINC01340	2.234022077	0	4.468044155	4.661903782	1.823037446	2.55721779	0.010551	0.025483
ENSG00000101746	NOL4	2.216044432	0	4.432088864	4.651852475	1.756583412	2.64823887	0.008091	0.020026
ENSG00000226377	AC084809.1	2.216044432	0	4.432088864	4.651852475	1.756583412	2.64823887	0.008091	0.020026
ENSG00000143226	FCGR2A	4.439839668	0.3140996	8.565579693	4.639412354	1.488221358	3.11742089	0.001824	0.005165
ENSG00000198535	C2CD4A	820.9810912	63.200086	1578.762096	4.637316533	0.12563826	36.9100664	3.19E-298	3.73E-296
ENSG00000232554	RSU1P2	2.180900713	0	4.361801426	4.632714304	1.779590082	2.60324799	0.009235	0.022594
ENSG00000115461	IGFBP5	40.16143417	3.0456567	77.27721164	4.608584337	0.560235381	8.22615724	1.93E-16	1.51E-15
ENSG00000136244	IL6	1120.18442	88.377963	2151.990877	4.593372605	0.107279362	42.816927	0	0
ENSG00000023445	BIRC3	318.3960099	24.952225	611.8397951	4.591016876	0.204195421	22.4834468	6.03E-112	2.43E-110
ENSG00000100767	PAPLN	3420.51083	272.18046	6568.841205	4.588960374	0.062133169	73.8568538	0	0

ENSG00000180720	CHRM4	7.937081191	0.6486456	15.22551674	4.576737261	1.252405142	3.65435841	0.000258	0.000837
ENSG00000204291	COL15A1	2.113874231	0	4.227748461	4.575963613	1.795800647	2.54814677	0.01083	0.026083
ENSG00000183778	B3GALT5	2.098164625	0	4.196329251	4.566797052	1.78451609	2.55912349	0.010494	0.025367
ENSG00000285413	AP001056.2	2.094440117	0	4.188880234	4.565088332	1.811840284	2.51958651	0.011749	0.028092
ENSG00000164287	CDC20B	2.072737963	0	4.145475926	4.552211089	1.782893806	2.55327102	0.010672	0.025743
ENSG00000165379	LRFN5	2.057028358	0	4.114056715	4.543058347	1.790022982	2.53798884	0.011149	0.026772
ENSG00000255921	AC026310.2	4.166668295	0.3140996	8.019236948	4.539897512	1.529606822	2.96801599	0.002997	0.008134
ENSG00000108309	RUNDC3A	4.139853381	0.294802	7.9849048	4.534901383	1.503888666	3.0154502	0.002566	0.007065
ENSG00000145936	KCNMB1	19.30358909	1.6113909	36.99578727	4.532015914	0.775626723	5.84303735	5.13E-09	2.67E-08
ENSG00000120708	TGFBI	16083.79597	1365.7078	30801.88419	4.495650686	0.033028672	136.113577	0	0
ENSG00000077943	ITGA8	67.09922558	5.6981323	128.5003189	4.485468877	0.41166073	10.896033	1.20E-27	1.40E-26
ENSG00000178776	C5orf46	22.25592501	1.9050442	42.60680582	4.473304454	0.728610148	6.13950337	8.28E-10	4.54E-09
ENSG00000162366	PDZK1IP1	7.432270048	0.6679433	14.19659678	4.467475318	1.249050101	3.57669825	0.000348	0.001106
ENSG00000187037	GPR141	3.927715942	0.294802	7.560629922	4.458604126	1.517966626	2.93722145	0.003312	0.008901
ENSG00000171611	PTCRA	1.942873059	0	3.885746119	4.453718534	1.881157467	2.36754159	0.017907	0.040875
ENSG00000243742	RPLP0P2	1.929431494	0	3.858862988	4.445274927	1.83889405	2.41736327	0.015633	0.036312
ENSG00000075223	SEMA3C	34.65768391	3.1448491	66.17051876	4.433158705	1.133895679	3.90967069	9.24E-05	0.000319
ENSG00000188064	WNT7B	3.859232992	0.294802	7.423664021	4.426602953	1.609089747	2.75099817	0.005941	0.015143
ENSG00000169181	GSG1L	1.898012283	0	3.796024567	4.425363984	1.830419907	2.41767693	0.01562	0.036285
ENSG00000206195	DUXAP8	1.884570718	0	3.769141436	4.417165436	1.891288648	2.33553215	0.019516	0.044169
ENSG00000176907	TCIM	2720.861873	243.84499	5197.878757	4.414432633	0.066618045	66.2648182	0	0
ENSG00000153233	PTPRR	17.69575616	1.5909446	33.80056774	4.40269376	0.811254916	5.42701644	5.73E-08	2.74E-07
ENSG00000077264	PAK3	1.853151507	0	3.706303014	4.396744437	1.883746979	2.33404193	0.019594	0.044329
ENSG00000184261	KCNK12	3.676710276	0.294802	7.058618589	4.365196965	1.553118792	2.81060083	0.004945	0.012816
ENSG00000166592	RRAD	6.799998476	0.6486456	12.95135131	4.342020394	1.248916986	3.4766285	0.000508	0.001576
ENSG00000110693	SOX6	3.643302827	0.3140996	6.972506012	4.338741961	1.583923262	2.73923748	0.006158	0.015648
ENSG00000211448	DIO2	300.9088236	28.707124	573.1105231	4.30613818	0.190296276	22.6285993	2.27E-113	9.24E-112
ENSG00000145113	MUC4	6.591079425	0.6281993	12.55395957	4.304820982	1.308725768	3.2893224	0.001004	0.002972
ENSG00000278112	AC145423.3	3.421448331	0.3140996	6.528797021	4.247226756	1.565842723	2.71242232	0.006679	0.016845
ENSG00000163874	ZC3H12A	1019.392837	102.77143	1936.014245	4.235935924	0.105490602	40.1546283	0	0
ENSG00000152463	OLAH	9.32202206	0.9037036	17.74034055	4.230082635	1.043928385	4.05208125	5.08E-05	0.000181
ENSG00000049249	TNFRSF9	24.97997239	2.4560528	47.503892	4.228859962	0.640431499	6.60314174	4.03E-11	2.41E-10
ENSG00000142661	MYOM3	78.31028722	7.9558715	148.6647029	4.221816665	0.362995956	11.6304785	2.88E-31	3.69E-30
ENSG00000178573	MAF	92.935431	9.5865601	176.2843019	4.20263863	0.329753286	12.7447968	3.33E-37	4.84E-36
ENSG00000183570	PCBP3	3.333157599	0.3538437	6.312471521	4.193333883	1.587728421	2.64109014	0.008264	0.020412
ENSG00000072858	SIDT1	3.269949491	0.294802	6.24509702	4.180294269	1.607132712	2.60108841	0.009293	0.022727
ENSG00000104415	CCN4	3.244522829	0.294802	6.194243695	4.170544101	1.606827291	2.59551485	0.009445	0.023075
ENSG00000087085	ACHE	21.3066881	2.2781856	40.33519064	4.165050531	0.697322772	5.97291627	2.33E-09	1.24E-08
ENSG00000180921	FAM83H	8.868064242	0.9230012	16.81312724	4.14549841	1.060316869	3.90967882	9.24E-05	0.000319
ENSG00000149633	KIAA1755	56.66133874	6.070125	107.2525525	4.142952171	0.418624239	9.89658932	4.31E-23	4.34E-22
ENSG00000273415	AP000904.1	23.87870057	2.6922196	45.0651815	4.123905266	0.6576175	6.2709786	3.59E-10	2.02E-09
ENSG00000141668	CBLN2	78.00964974	8.3116059	147.7076936	4.113385426	0.36626139	11.2307372	2.88E-29	3.52E-28
ENSG00000236452	AC123023.1	11.61799911	1.2575472	21.97845097	4.111633549	0.947559335	4.3391832	1.43E-05	5.43E-05
ENSG00000163131	CTSS	2684.584618	296.31009	5072.859145	4.097340425	0.062446868	65.6132258	0	0
ENSG00000127533	F2RL3	401.7839554	45.088903	758.4790073	4.075197205	0.157088978	25.9419678	2.24E-148	1.20E-146
ENSG00000187957	DNER	56.99397975	6.6431352	107.3448243	4.057363196	0.424499867	9.55798461	1.20E-21	1.15E-20
ENSG00000144644	GADL1	25.82629438	3.0858073	48.56678142	4.055031173	0.631818459	6.41803214	1.38E-10	8.01E-10
ENSG00000165478	HEPACAM	5.641956963	0.6679433	10.61597061	4.042346344	1.30697602	3.09290016	0.001982	0.005575
ENSG00000156042	CFAP70	3.024842508	0.3538437	5.695841337	4.04189595	1.664057046	2.42894074	0.015143	0.03532
ENSG00000271856	LINC01215	22.0385567	2.5536898	41.52342355	4.021938064	0.666039816	6.03858503	1.55E-09	8.38E-09
ENSG00000183486	MX2	101.4498947	11.743958	191.1558312	4.017956905	0.885942189	4.535236	5.75E-06	2.28E-05
ENSG00000136883	KIF12	19.1015607	2.2009948	36.00212655	4.01657261	0.70021022	5.7362382	9.68E-09	4.93E-08
ENSG00000231535	LINC00278	2.965728595	0.3538437	5.577613511	4.016121106	1.669753692	2.40521768	0.016163	0.037414
ENSG00000107105	ELAVL2	18.77101567	2.1215068	35.42052456	4.001251962	0.709521852	5.63936396	1.71E-08	8.53E-08
ENSG00000106236	NPTX2	34.98930467	4.1264854	65.85212392	3.991644287	0.521335397	7.65657638	1.91E-14	1.37E-13
ENSG00000189120	SP6	136.6671027	16.18495	257.1492554	3.983569304	0.273198227	14.5812414	3.70E-48	6.77E-47
ENSG00000137203	TFAP2A	15.86469847	1.9447882	29.7846087	3.952418832	0.762815568	5.18135575	2.20E-07	1.00E-06
ENSG00000011201	ANOS1	20.56859276	2.4560528	38.68113275	3.935972021	0.666898238	5.90190796	3.59E-09	1.89E-08
ENSG00000223387	LINC02068	2.773467154	0.294802	5.252132346	3.927985854	1.639372432	2.3960302	0.016574	0.038245
ENSG00000237604	AP001056.1	2.769742646	0.294802	5.244683329	3.926586675	1.643990349	2.38844874	0.01692	0.038936
ENSG00000233785	AC131011.1	2.766018137	0.294802	5.237234312	3.925433845	1.683360132	2.33190377	0.019706	0.04455
ENSG00000285280	AL390957.1	2.757757549	0.294802	5.220713135	3.920896536	1.644619389	2.38407534	0.017122	0.039328
ENSG00000261357	AC099518.2	2.783553898	0.3538437	5.213264118	3.919367318	1.631897651	2.40172373	0.016318	0.037716
ENSG00000261488	TBILA	7.734499043	0.9627453	14.5062528	3.918972876	1.08625374	3.6077877	0.000309	0.000991
ENSG00000234678	ELF3-AS1	7.478519393	0.9230012	14.03403754	3.886748275	1.091421894	3.56117858	0.000369	0.00117
ENSG00000151617	EDNRA	14.96353745	1.8664488	28.06062606	3.883383661	0.767787069	5.05789146	4.24E-07	1.88E-06
ENSG00000141431	ASXL3	14.93658611	1.8857465	27.98742568	3.876802805	0.768729275	5.04313148	4.58E-07	2.02E-06
ENSG00000108688	CCL7	24.52088209	3.1807405	45.86102372	3.846837855	1.191984204	3.22725573	0.00125	0.003633
ENSG00000183775	KCTD16	43.88645147	5.8343647	81.93853821	3.828402475	0.452034394	8.46927252	2.47E-17	2.00E-16
ENSG00000134853	PDGFRA	62.59550025	8.1927804	116.9982201	3.822480134	0.379087076	10.0833829	6.54E-24	6.81E-23
ENSG00000280649	AC245100.8	7.070438574	0.8844059	13.25647126	3.817455079	1.12486125	3.39371196	0.00069	0.002096
ENSG00000259863	SH3RF3-AS1	66.49143618	8.6438546	124.3390178	3.810554017	0.370998418	10.2710789	9.51E-25	1.02E-23
ENSG00000108551	RASD1	414.4537775	55.194362	773.7131927	3.803139843	0.148578425	25.5968512	1.65E-144	8.62E-143
ENSG00000188452	CERKL	2.547819793	0.3140996	4.781540223	3.799401639	1.663413267	2.28409964	0.022366	0.049865
ENSG00000028277	POU2F2	1699.522044	229.09271	3169.951373	3.792439858	0.153339051	24.7323811	4.80E-135	2.32E-133
ENSG00000143340	FAM163A	14.35634514	2.0242763	26.68841396	3.781271335	0.789354869	4.7903313	1.67E-06	6.99E-06
ENSG00000105499	PLA2G4C	2391.292579	326.79357	4455.791585	3.772001783	0.063942755	58.9902917	0	0
ENSG00000118503	TNFAIP3	1395.675883	191.65343	2599.698337	3.759082023	0.080858321	46.4897362	0	0
ENSG00000167207	NOD2	9.174344863	1.2563986	17.09229116	3.749638596	0.988957504	3.79150629	0.00015	0.000503
ENSG00000184374	COLEC10	32.43120527	4.5586685	60.30374206	3.745430632	0.515105584	7.27119012	3.56E-13	2.40E-12
ENSG00000131203	IDO1	6.664073124	0.9037036	12.42444268	3.717355698	1.111274021	3.34512967	0.000822	0.00247
ENSG00000120162	MOB3B	6.788246949	0.982043	12.59445094	3.710473496	1.113604124	3.33195021	0.000862	0.002577
ENSG00000134321	RSAD2	52.94354748	7.5202424	98.36685255	3.704704173	1.052721356	3.51916882	0.000433	0.001357
ENSG00000242419	PCDHGC4	6.572015315	0.9230012	12.22102938	3.69060805	1.11869105	3.29904137	0.00097	0.002878
ENSG00000116132	PRRX1	72.8241621	10.551603	135.0967214	3.681868623	0.335260238	10.9821214	4.66E-28	5.48E-27

ENSG00000222345	SNORD19C	4.437915983	0.6486456	8.227186326	3.673112606	1.406947822	2.61069568	0.009036	0.022149
ENSG00000162881	OXER1	4.344469923	0.6486456	8.040294205	3.645708477	1.406939425	2.59123343	0.009563	0.023321
ENSG00000155966	AFF2	34.06013103	5.1255287	62.99473335	3.62112158	0.517469511	6.99774866	2.60E-12	1.67E-11
ENSG00000043462	LCP2	29.88179118	4.5984125	55.16516984	3.617554151	0.527010246	6.86429567	6.68E-12	4.21E-11
ENSG00000227158	AC073621.1	8.30135902	1.2382496	15.36446847	3.605096307	1.020252584	3.53353313	0.00041	0.00129
ENSG00000167244	IGF2	165.1008759	25.544197	304.6575547	3.588246114	0.232045237	15.4635629	6.11E-54	1.22E-52
ENSG00000135114	OASL	30.97760941	4.7126433	57.24257551	3.584837201	0.546221687	6.5629712	5.27E-11	3.14E-10
ENSG00000237499	AL357060.1	12.37761223	1.8857465	22.86947793	3.578750892	0.819167459	4.36876594	1.25E-05	4.78E-05
ENSG00000186340	THBS2	105.156525	16.407563	193.9054875	3.572050955	0.273970083	13.0381059	7.43E-39	1.13E-37
ENSG00000273812	BX640514.2	18.02792396	2.7304084	33.32543954	3.555957248	0.681636293	5.21679565	1.82E-07	8.34E-07
ENSG00000170961	HAS2	160.9410288	25.505195	296.3768625	3.53723063	0.224057009	15.7871903	3.81E-56	7.88E-55
ENSG00000048052	HDAC9	443.3660463	71.441871	815.2902216	3.521580409	0.134476982	26.1872357	3.71E-151	2.06E-149
ENSG00000116147	TNR	68.86282011	11.142355	126.5832849	3.504814123	0.343639023	10.1991156	2.00E-24	2.11E-23
ENSG00000139329	LUM	21.55492225	3.4755424	39.63430209	3.500459144	0.630961531	5.54781705	2.89E-08	1.42E-07
ENSG00000162654	GBP4	1480.098591	240.82833	2719.368851	3.496733532	0.077557133	45.0859052	0	0
ENSG00000086205	FOLH1	5.790376508	0.9230012	10.65775177	3.490984682	1.15696819	3.0173558	0.00255	0.007025
ENSG00000110169	HPX	3.864715755	0.6089016	7.120529906	3.490747575	1.439022193	2.42577744	0.015276	0.035593
ENSG00000105639	JAK3	547.5006915	91.024431	1003.976952	3.471275171	0.124314262	27.9233864	1.39E-171	8.62E-170
ENSG00000160223	ICOSLG	526.1386081	88.022635	964.2545813	3.44962321	0.121161527	28.4712754	2.66E-178	1.76E-176
ENSG00000272419	LINC01145	11.39475995	1.9050442	20.88447568	3.445855904	0.826457819	4.16942744	3.05E-05	0.000112
ENSG00000120658	ENOX1	7.544615107	1.2575472	13.83168297	3.438182756	1.056740577	3.25357314	0.00114	0.003338
ENSG00000259727	AC103740.2	11.50891126	2.00383	21.01399257	3.436155471	0.816109468	4.21041001	2.55E-05	9.45E-05
ENSG00000163888	CAMK2N2	5.528265534	0.9037036	10.1528275	3.435995421	1.184810131	2.90003886	0.003731	0.009919
ENSG00000153237	CCDC148	3.811964078	0.6679433	6.955984836	3.424300578	1.391900446	2.46016199	0.013887	0.032678
ENSG00000169248	CXCL11	142.6910221	24.127603	261.2544416	3.40967683	0.252524213	13.5023758	1.51E-41	2.45E-40
ENSG00000105371	ICAM4	20.62441835	3.6140722	37.6347645	3.407289677	0.62420546	5.45860281	4.80E-08	2.31E-07
ENSG00000113361	CDH6	10.81857163	1.7881095	19.84903381	3.406227859	0.851451677	4.00049463	6.32E-05	0.000223
ENSG00000268355	AC243960.3	5.404393178	0.9037036	9.90508279	3.397123876	1.171538631	2.89971136	0.003735	0.009925
ENSG00000224594	RPL29P19	3.629419694	0.6089016	6.649937784	3.395180606	1.422099796	2.38744188	0.016966	0.039023
ENSG00000112149	CD83	30.98699443	5.3238423	56.65014658	3.393206271	0.499389669	6.79470659	1.09E-11	6.74E-11
ENSG00000003987	MTMR7	79.42110578	13.553663	145.2885488	3.388399759	0.328257953	10.3223691	5.58E-25	6.00E-24
ENSG00000105825	TFPI2	26328.81718	4702.5664	47955.06791	3.350236297	0.025680206	130.45987	0	0
ENSG00000148680	HTR7	10.7443254	1.9845323	19.50411853	3.334086673	0.836086924	3.98772732	6.67E-05	0.000235
ENSG00000078098	FAP	252.423788	45.772698	459.0748776	3.329180421	0.180493916	18.4448346	5.74E-76	1.60E-74
ENSG00000233427	AL009181.1	17.83315256	3.3011212	32.36518387	3.323786279	0.651704411	5.10014391	3.39E-07	1.52E-06
ENSG00000184347	SLIT3	30.92380596	5.5414535	56.30615841	3.313632475	0.510777748	6.48742529	8.73E-11	5.14E-10
ENSG00000132965	ALOX5AP	5.080436698	0.8844059	9.276467509	3.313121602	1.211301495	2.73517503	0.006235	0.01582
ENSG00000168685	IL7R	551.0605617	100.82583	1001.295297	3.305491983	0.1178214	28.0551069	3.46E-173	2.19E-171
ENSG00000277117	FP565260.3	768.7092951	143.13143	1394.28716	3.283135007	0.100092608	32.8009736	5.70E-236	5.30E-234
ENSG00000132692	BCAN	3.44913937	0.6486456	6.2496331	3.283074714	1.425476994	2.30314114	0.021271	0.047719
ENSG00000119508	NR4A3	17.21134772	3.2204845	31.20221094	3.279334287	0.717066223	4.5732656	4.80E-06	1.91E-05
ENSG00000229656	ITGB1-DT	57.8004957	10.687429	104.9135628	3.279257298	0.351375906	9.33261854	1.03E-20	9.55E-20
ENSG00000138135	CH25H	23.82033088	4.4201387	43.22052307	3.275539444	0.55559732	5.8955278	3.73E-09	1.96E-08
ENSG00000184408	KCND2	6.744242972	1.2563986	12.23208738	3.263229245	1.076891243	3.03023102	0.002444	0.006762
ENSG00000221869	CEBPD	795.366953	152.37415	1438.359757	3.239663636	0.20170787	16.0611662	4.77E-58	1.03E-56
ENSG00000181634	TNFSF15	5263.264056	1013.1697	9513.35842	3.231954259	0.108061905	29.9083591	1.53E-196	1.11E-194
ENSG00000127585	FBXL16	6.27019921	1.1792079	11.36119057	3.193671161	1.089226807	2.9320534	0.003367	0.009034
ENSG00000285925	AC007391.2	7.940156133	1.5716469	14.30866538	3.174501796	0.95101288	3.33802188	0.000844	0.002526
ENSG00000170743	SYT9	313.560993	62.777906	564.3440803	3.172084663	0.149548334	21.2111	7.54E-100	2.83E-98
ENSG00000152503	TRIM36	20.48175507	4.0674437	36.89606645	3.159402852	0.586628747	5.38569388	7.22E-08	3.42E-07
ENSG00000267532	MIR497HG	6.197507297	1.2178032	11.17721138	3.156569863	1.061273224	2.97432347	0.002936	0.007989
ENSG00000160179	ABCG1	2580.976507	520.61574	4641.337269	3.153785116	0.058273467	54.1204307	0	0
ENSG00000084636	COL16A1	286.2454413	58.431176	514.0597065	3.152188706	0.169154578	18.6349595	1.67E-77	4.79E-76
ENSG00000167034	NKX3-1	177.3012549	35.714953	318.8875565	3.14878065	0.212220322	14.8373191	8.40E-50	1.57E-48
ENSG00000108576	SLC6A4	31.8851919	6.5806474	57.18973635	3.146307404	0.491146102	6.40605186	1.49E-10	8.64E-10
ENSG00000270164	LINC01480	16.14755594	3.399907	28.89520489	3.146138215	0.699381908	4.49845525	6.84E-06	2.69E-05
ENSG00000156920	ADGRG4	12.4572747	2.5332435	22.3813059	3.13885092	0.768978558	4.08184453	4.47E-05	0.000161
ENSG00000133106	EPSTI1	165.4508895	33.865911	297.0358676	3.133007746	0.238559194	13.1330413	2.13E-39	3.29E-38
ENSG00000110375	UPK2	4.687418949	0.9627453	8.412092615	3.127441355	1.194674099	2.61781967	0.008849	0.021741
ENSG00000166016	ABTB2	66.38521862	13.637003	119.1334338	3.122007413	0.322259478	9.68786838	3.40E-22	3.32E-21
ENSG00000157601	MX1	1867.220111	386.62289	3347.817331	3.113755182	0.252054079	12.3535203	4.66E-35	6.43E-34
ENSG00000104951	IL4I1	835.0546929	173.23848	1496.870902	3.111667342	0.094906111	32.7867965	9.08E-236	8.39E-234
ENSG00000138311	ZNF365	81.56703336	16.758296	146.3757711	3.100352984	0.299816924	10.3408205	4.61E-25	4.97E-24
ENSG00000279447	AL118508.3	4.540345402	0.9422989	8.138391878	3.098048669	1.256152187	2.46630042	0.013652	0.032201
ENSG00000105376	ICAM5	7.52229004	1.5523492	13.49223087	3.09142235	0.938288907	3.29474464	0.000985	0.002919
ENSG00000137959	IFI44L	829.7036402	174.51017	1484.897111	3.089720675	0.209020358	14.7819127	1.92E-49	3.56E-48
ENSG00000172379	ARNT2	8.775380174	1.8074071	15.74335321	3.077190386	0.917753409	3.35295991	0.0008	0.002406
ENSG00000038945	MSR1	31.42688137	6.6813239	56.17243879	3.070748016	0.49472896	6.20692999	5.40E-10	3.00E-09
ENSG00000171236	LRG1	31.43065078	6.7199193	56.14138227	3.065258229	0.456001757	6.72203162	1.79E-11	1.10E-10
ENSG00000154721	JAM2	40.15751466	8.6056658	71.70936349	3.062494403	0.44943943	6.81403144	9.49E-12	5.93E-11
ENSG00000183876	ARSI	4.435025031	0.9434476	7.926602459	3.0577928	1.250671779	2.44492028	0.014488	0.033944
ENSG00000110944	IL23A	5.730312565	1.1792079	10.28141728	3.05281831	1.115887107	2.73577702	0.006223	0.015798
ENSG00000058085	LAMC2	694.5082014	149.08999	1239.926411	3.052474943	0.226253355	13.4914019	1.76E-41	2.84E-40
ENSG00000158315	RHBDL2	16.19716409	3.4971375	28.89719072	3.043196871	0.630994779	4.82285587	1.42E-06	5.98E-06
ENSG00000261408	TEN1-CDK3	5.909502127	1.2972913	10.52171297	3.039742836	1.078314905	2.81897507	0.004818	0.01251
ENSG00000173918	C1QTNF1	640.5982693	140.09585	1141.100691	3.035295512	0.115000418	26.3937781	1.62E-153	9.14E-152
ENSG00000185745	IFIT1	287.4383121	62.536101	512.3405232	3.033080287	0.547390263	5.54098327	3.01E-08	1.47E-07
ENSG00000134326	CMPK2	72.92127545	16.110463	129.7320877	3.016222999	0.308520055	9.77642441	1.42E-22	1.41E-21
ENSG00000251136	AF117829.1	99.04047376	22.00387	176.0770779	3.011638371	0.280806187	10.7249716	7.77E-27	8.80E-26
ENSG00000205795	CYS1	5.759301653	1.2575472	10.26105606	3.007451771	1.089049159	2.76153904	0.005753	0.014701
ENSG00000159403	C1R	459.4165195	102.68265	816.1503937	2.996237945	0.123249986	24.3102498	1.53E-130	7.11E-129
ENSG00000227706	AL713998.1	4.330913907	0.982043	7.679784853	2.995987819	1.305414313	2.29504747	0.02173	0.048595
ENSG00000256262	USP30-AS1	4.322653319	0.982043	7.663263676	2.994487222	1.275108955	2.34841674	0.018853	0.042828
ENSG00000249816	LINC00964	6.937568268	1.5126052	12.36253136	2.990347068	1.016746501	2.94109403	0.003271	0.008802
ENSG00000119714	GPR68	85.55405153	19.119822	151.9882812	2.990164619	0.282172346	10.5969443	3.08E-26	3.45E-25

ENSG00000164932	CTHRC1	6446.589879	1446.7441	11446.43564	2.982721456	0.039967438	74.6287873	0	0
ENSG00000140479	PCSK6	15.71092659	3.5766255	27.84522767	2.981950888	0.658446369	4.52876807	5.93E-06	2.34E-05
ENSG00000267370	AC008752.3	5.777670653	1.3756307	10.17971063	2.959585584	1.080764135	2.7384195	0.006174	0.015685
ENSG00000159708	LRRC36	23.81290586	5.4612234	42.16458835	2.957646733	0.54109755	5.4660139	4.60E-08	2.22E-07
ENSG00000186469	GNG2	37.30537258	8.4512844	66.15946077	2.955019037	0.46934175	6.29609242	3.05E-10	1.73E-09
ENSG00000189056	RELN	5071.804859	1160.284	8983.325749	2.953868451	0.042356413	69.7383989	0	0
ENSG00000144837	PLA1A	46.23217075	10.374071	82.09027052	2.952849957	0.432501251	6.827379	8.65E-12	5.41E-11
ENSG00000179761	PIPOX	9.760704436	2.2600366	17.26137231	2.943896682	0.809043766	3.63873601	0.000274	0.000886
ENSG00000178538	CA8	200.1429338	46.373789	353.9120785	2.934232603	0.185649111	15.8052608	2.86E-56	5.95E-55
ENSG00000127863	TNFRSF19	104.5351124	24.30669	184.7635351	2.930397112	0.272911876	10.7375214	6.78E-27	7.69E-26
ENSG00000108821	COL1A1	12.80791659	3.0858073	22.53002583	2.927108508	0.762461915	3.83902258	0.000124	0.000419
ENSG00000008517	IL32	5246.859895	1232.9013	9260.818507	2.909002516	0.040041403	72.6498648	0	0
ENSG00000213626	LBH	556.7712655	130.79705	982.7454762	2.90730873	0.112062001	25.9437517	2.14E-148	1.15E-146
ENSG00000278986	AC091060.1	6.769110958	1.6306886	11.90753331	2.891816667	0.973004834	2.97204759	0.002958	0.008033
ENSG00000174939	ASPHD1	45.28587468	10.847553	79.72419598	2.879049094	0.422892021	6.8080005	9.90E-12	6.17E-11
ENSG00000118971	CCND2	138.5233335	33.143521	243.9031461	2.878348789	0.219494566	13.113531	2.75E-39	4.24E-38
ENSG00000170075	GPR37L1	7.730355269	1.7881095	13.67260108	2.878192758	0.963304193	2.98783373	0.00281	0.007672
ENSG00000165449	SLC16A9	13.35852225	3.2216332	23.49541134	2.877839939	0.695168875	4.13977098	3.48E-05	0.000127
ENSG00000267325	LINC01415	37.14402721	8.8614658	65.42658859	2.850920047	0.466891922	6.106167	1.02E-09	5.57E-09
ENSG00000162849	KIF26B	72.08091313	17.743449	126.4183771	2.844744026	0.302768085	9.39578565	5.68E-21	5.31E-20
ENSG00000240350	AC017002.3	9.253161701	2.2793342	16.22698916	2.843050519	0.847069288	3.35633762	0.00079	0.002379
ENSG00000074047	GLI2	11.65265261	2.8496405	20.45566473	2.835087466	0.743849585	3.81137198	0.000138	0.000466
ENSG00000172602	RND1	2275.035875	562.34518	3987.726575	2.828517256	0.067051303	42.1843743	0	0
ENSG00000087258	GNAO1	5.079790479	1.2371009	8.922480069	2.822822158	1.155666441	2.44259248	0.014582	0.034142
ENSG00000261707	AC092134.1	11.75516685	2.9484262	20.56190745	2.822245824	0.760642034	3.71034691	0.000207	0.000681
ENSG00000229056	HECW2-AS1	5.235494301	1.316589	9.154399641	2.819453841	1.106775792	2.54744806	0.010851	0.026132
ENSG00000104856	RELB	1011.576617	251.85661	1771.296621	2.811590662	0.088455822	31.7852528	1.03E-221	8.57E-220
ENSG00000105357	MYH14	11.43110356	2.8291941	20.03301299	2.809039703	0.73065248	3.84456329	0.000121	0.000411
ENSG00000234996	AC098934.2	7.499390636	1.8460025	13.15277878	2.807330743	0.992141324	2.8295674	0.004661	0.012144
ENSG00000182873	PRKCZ-AS1	7.624625594	1.9050442	13.34420698	2.80481993	0.916369126	3.06079706	0.002207	0.006164
ENSG00000270607	AC009549.1	161.409957	40.260067	282.5598465	2.804510213	0.201009857	13.9521029	3.05E-44	5.19E-43
ENSG00000164251	F2RL1	10831.21848	2726.1307	18936.30628	2.795912874	0.029131878	95.9743434	0	0
ENSG00000135333	EPHA7	5.237784008	1.3756307	9.099937341	2.795371474	1.128296356	2.47751529	0.01323	0.031291
ENSG00000172183	ISG20	346.2248851	87.50948	604.9402906	2.789959139	0.134108612	20.8037283	4.00E-96	1.46E-94
ENSG00000111335	OAS2	1445.717531	366.5115	2524.923558	2.785011229	0.224140528	12.4252908	1.91E-35	2.66E-34
ENSG00000267607	AC011511.5	5.001522254	1.2563986	8.746645941	2.78131932	1.204683659	2.30875492	0.020957	0.047101
ENSG00000158457	TSPAN33	31.40335166	7.9763179	54.83038545	2.780379407	0.440090434	6.31774561	2.65E-10	1.51E-09
ENSG00000064651	SLC12A2	7539.529495	1924.4921	13154.56691	2.775036962	0.037658126	73.6902558	0	0
ENSG00000154678	PDE1C	440.2291702	112.67479	767.7835482	2.771278602	0.120410913	23.0151781	3.29E-117	1.39E-115
ENSG00000227482	AL157702.2	11.02073538	2.7497061	19.29176469	2.769671041	0.850932259	3.25486666	0.001134	0.003325
ENSG00000133401	PDZD2	283.7250962	72.472546	494.977646	2.768499842	0.153092461	18.0838418	4.27E-73	1.14E-71
ENSG00000092969	TGFB2	1501.705772	385.38844	2618.023102	2.761395954	0.068767171	40.1557303	0	0
ENSG00000110042	DTX4	47.88548694	12.578583	83.19239086	2.754308087	0.421201842	6.53916439	6.19E-11	3.67E-10
ENSG00000121898	CPXM2	13.45941531	3.4392444	23.4795862	2.753081766	0.687953319	4.00184386	6.29E-05	0.000222
ENSG00000174348	PODN	254.3146707	65.365525	443.2638165	2.752653473	0.159196046	17.2909663	5.50E-67	1.36E-65
ENSG00000255893	AP000786.1	5.926694166	1.4933075	10.36008084	2.748022281	1.100978799	2.49598111	0.012561	0.029858
ENSG00000107562	CXCL12	62.85149994	16.130096	109.5729036	2.742057958	0.331982099	8.25965607	1.46E-16	1.14E-15
ENSG00000172738	TMEM217	343.4385951	89.690841	597.1863489	2.741344175	0.138053687	19.8570878	9.57E-88	3.21E-86
ENSG00000163347	CLDN1	22.03709181	5.7764717	38.29771193	2.73333905	0.525368975	5.20270358	1.96E-07	8.97E-07
ENSG00000254887	AC010247.1	19.63746845	5.1459751	34.12896185	2.732361955	0.587576368	4.65022439	3.32E-06	1.35E-05
ENSG00000167984	NLRC3	207.3259257	54.108939	360.5429127	2.730834726	0.181718814	15.0278041	4.83E-51	9.25E-50
ENSG00000243650	RN7SL834P	7.179201233	1.8471512	12.5112513	2.725756404	0.930604986	2.92901547	0.0034	0.009116
ENSG00000244588	RAD21L1	19.37241196	5.069933	33.6748909	2.723803291	0.577346552	4.71779607	2.38E-06	9.84E-06
ENSG00000184545	DUSP8	327.6578616	86.098293	569.2174302	2.720836091	0.140471477	19.3693135	1.40E-83	4.45E-82
ENSG00000189058	APOD	231.2906825	60.887158	401.6942073	2.720702368	0.16095136	16.9038793	4.21E-64	9.93E-63
ENSG00000148154	UGCG	4156.260833	1095.833	7216.688686	2.720014997	0.042293488	64.3128559	0	0
ENSG00000242173	ARHGDIG	66.43924684	17.424348	115.4541455	2.719887326	0.318032033	8.5522433	1.21E-17	9.90E-17
ENSG00000259518	LINC01583	36.11989676	9.4503277	62.78946585	2.717682452	0.426523701	6.37170326	1.87E-10	1.07E-09
ENSG00000280721	LINC01943	36.10244456	9.5116667	62.69322238	2.714305973	0.434833094	6.24217892	4.32E-10	2.42E-09
ENSG00000275993	SIK1B	198.207719	52.474062	343.941376	2.708533805	0.179876477	15.0577432	3.07E-51	5.90E-50
ENSG00000258602	LINC01629	9.344626227	2.4163087	16.27294372	2.705286591	0.859425776	3.14778387	0.001645	0.004689
ENSG00000163545	NUAK2	157.1059355	41.863418	272.3484534	2.697238681	0.205863275	13.1020877	3.20E-39	4.92E-38
ENSG00000155307	SAMSN1	472.4044697	125.93399	818.874951	2.695698282	0.116567221	23.1256974	2.55E-118	1.08E-116
ENSG00000131409	LRRC4B	11.98049895	3.2216332	20.73936473	2.693444863	0.707172658	3.80875142	0.00014	0.000471
ENSG00000144218	AFF3	27.18705692	7.2300352	47.14407868	2.691555746	0.467800259	5.75364314	8.73E-09	4.46E-08
ENSG00000260949	AP006545.1	5.846595805	1.5523492	10.1408424	2.689095562	1.020449775	2.63520619	0.008409	0.020738
ENSG00000279781	AC079336.6	4.754679	1.2961426	8.213215397	2.677455061	1.158929463	2.31028302	0.020872	0.046922
ENSG00000053524	MCF2L2	38.61700814	10.391478	66.84253835	2.673099764	0.398987398	6.69970976	2.09E-11	1.28E-10
ENSG00000188517	COL25A1	294.5852117	79.538834	509.6315893	2.671805911	0.160401445	16.656994	2.69E-62	6.20E-61
ENSG00000164093	PITX2	4.845344578	1.356333	8.33435616	2.669534348	1.139991749	2.34171374	0.019195	0.043519
ENSG00000126709	IFI6	2253.358537	613.02105	3893.696019	2.667820315	0.072474884	36.8102735	1.26E-296	1.46E-294
ENSG00000233452	STXBP5-AS1	95.01148889	25.640614	164.3823634	2.663023322	0.263399272	10.1102152	4.98E-24	5.19E-23
ENSG00000237807	AC022034.1	11.35348296	3.0649544	19.64201153	2.656294706	0.787222136	3.37426323	0.00074	0.002238
ENSG00000011465	DCN	8.368727464	2.338376	14.39908297	2.655295786	0.880516632	3.01561117	0.002565	0.007062
ENSG00000273312	AL121749.1	5.86998768	1.6499863	10.08998908	2.645671423	1.049777721	2.52022059	0.011728	0.028045
ENSG00000268670	AC016586.1	6.800530486	1.8664488	11.73461212	2.629790445	0.946283604	2.77907219	0.005451	0.013993
ENSG00000213949	ITGA1	1808.201564	504.52212	3111.881004	2.629533934	0.065895963	39.904325	0	0
ENSG00000162882	HAAO	47.8902525	13.30016	82.48034487	2.626275976	0.35366209	7.42594712	1.12E-13	7.76E-13
ENSG00000183691	NOG	27.26319681	7.6031765	46.92321711	2.618312227	0.460315112	5.68808662	1.28E-08	6.48E-08
ENSG00000070404	FSTL3	1951.099354	546.47749	3355.721221	2.616090678	0.06154632	42.5060458	0	0
ENSG00000279794	AC024580.1	6.738922214	1.8653002	11.61254425	2.613451477	1.061231552	2.46265904	0.013791	0.032484
ENSG00000123342	MMP19	140.9199086	39.729912	242.1099054	2.611332496	0.204860127	12.7469046	3.24E-37	4.72E-36
ENSG00000105963	ADAP1	12.2518918	3.4176494	21.08613422	2.610340854	0.741980204	3.51807345	0.000435	0.001362
ENSG00000226979	LTA	7.810852234	2.1793998	13.44230465	2.609900877	0.941956478	2.77072342	0.005593	0.014323
ENSG00000026559	KCNG1	55.41348926	15.878556	94.94842293	2.608900717	0.362266222	7.20161185	5.95E-13	3.97E-12

ENSG00000131831	RAI2	89.41529553	25.287177	153.5434137	2.595376213	0.259527684	10.0003829	1.52E-23	1.56E-22
ENSG00000142149	HUNK	10.31134563	3.0267656	17.59592563	2.580983395	0.801375937	3.22068991	0.001279	0.003713
ENSG00000249464	LINC01091	102.1042308	29.513262	174.6951999	2.57821141	0.246670327	10.4520533	1.43E-25	1.57E-24
ENSG00000205502	C2CD4B	1246.839739	360.52111	2133.158365	2.564472888	0.090215828	28.4259752	9.66E-178	6.34E-176
ENSG00000237989	LINC01679	16.68866238	4.8739168	28.50340795	2.560888037	0.604757526	4.23456993	2.29E-05	8.53E-05
ENSG00000231890	DARS-AS1	5.427275585	1.5512005	9.30335064	2.559511275	1.076644763	2.37730342	0.01744	0.039968
ENSG00000138448	ITGAV	48005.61626	13928.796	82082.43628	2.559174889	0.02295473	111.487909	0	0
ENSG00000107968	MAP3K8	329.7464455	95.389238	564.103653	2.556392037	0.13970259	18.2988164	8.46E-75	2.30E-73
ENSG00000137726	FXYD6	38.06947447	10.983379	65.15556973	2.552951109	0.404316495	6.31423931	2.71E-10	1.55E-09
ENSG00000101670	LIPG	23270.91751	6778.4585	39763.37651	2.552312562	0.02720201	93.8280874	0	0
ENSG00000164114	MAP9	94.31555637	27.458536	161.1725763	2.550631895	0.511323861	4.98829038	6.09E-07	2.66E-06
ENSG00000025708	TYMP	142.8270363	41.395679	244.2583939	2.547350805	0.211754354	12.0297446	2.48E-33	3.30E-32
ENSG00000099617	EFNA2	40.39574073	11.868192	68.92328975	2.544634608	0.398551952	6.3846999	1.72E-10	9.89E-10
ENSG00000164283	ESM1	19108.84691	5590.0471	32627.64674	2.544145044	0.030994038	82.084982	0	0
ENSG00000204482	LST1	39.03759249	11.396265	66.67892038	2.543939039	0.387324649	6.56797611	5.10E-11	3.04E-10
ENSG00000232517	AC112198.2	122.844028	36.030608	209.6574479	2.541931021	0.232313184	10.9418285	7.27E-28	8.49E-27
ENSG00000010030	ETV7	17.11292065	5.0494867	29.17635464	2.521925063	0.575413694	4.38280335	1.17E-05	4.50E-05
ENSG00000133424	LARGE1	91.88239578	27.133922	156.6308697	2.512808032	0.27503708	9.13625186	6.47E-20	5.81E-19
ENSG00000168314	MOBP	177.6347053	53.127709	302.1417015	2.509284694	0.181039454	13.8604301	1.10E-43	1.84E-42
ENSG00000233098	CCDC144NL-A	7.457851389	2.2600366	12.65566621	2.496620555	0.920802561	2.71135275	0.006701	0.016894
ENSG00000117594	HSD11B1	6.594219532	2.043574	11.14486507	2.496241133	0.969323795	2.57523971	0.010017	0.024324
ENSG00000108370	RGS9	12.57991714	3.7714931	21.38834123	2.49455447	0.685402414	3.63954725	0.000273	0.000884
ENSG00000111110	PPM1H	263.8593775	80.343894	447.3748608	2.485279393	0.155752164	15.9566283	2.56E-57	5.37E-56
ENSG00000160111	CPAMD8	162.9641478	49.226981	276.7013143	2.482635723	0.21331423	11.6383972	2.63E-31	3.37E-30
ENSG00000169583	CLIC3	23.18111368	7.091912	39.27031538	2.474381842	0.511115326	4.84114194	1.29E-06	5.47E-06
ENSG00000245648	AC022075.1	391.3271025	119.57521	663.0789935	2.468205413	0.121310276	20.3462188	5.01E-92	1.77E-90
ENSG00000244701	AC004918.1	118.4955413	36.171029	200.8200539	2.467938787	0.21987663	11.2241978	3.10E-29	3.78E-28
ENSG00000180914	OXTR	16.60109839	5.1085284	28.09366841	2.461338532	0.590441136	4.16864338	3.06E-05	0.000112
ENSG00000165949	IFI27	6735.839815	2092.9364	11378.74321	2.442135826	0.038963922	62.6768475	0	0
ENSG00000143387	CTSK	1161.128345	361.95619	1960.300497	2.438383971	0.075267432	32.3962689	3.10E-230	2.70E-228
ENSG00000267702	AP005131.6	9.391606984	2.9870216	15.79619237	2.43486806	0.803021319	3.03213377	0.002428	0.006724
ENSG00000186056	MATN1-AS1	7.162887978	2.2202925	12.10548343	2.433212811	0.931770745	2.61138571	0.009018	0.022107
ENSG00000102385	DRP2	45.43044772	14.065334	76.79556153	2.432085936	0.367636355	6.61546635	3.70E-11	2.22E-10
ENSG00000171533	MAP6	15.29213838	4.7932801	25.79099671	2.428803221	0.612174798	3.96749953	7.26E-05	0.000254
ENSG00000185269	NOTUM	10.78171387	3.3007146	18.2627131	2.426469581	0.786487659	3.08519728	0.002034	0.00571
ENSG00000269899	AC025857.2	15.09256038	4.7944287	25.39069203	2.407283221	0.613106627	3.9263696	8.62E-05	0.000299
ENSG00000127561	SYNGR3	35.45971747	11.415969	59.50346605	2.397119417	0.42888909	5.58913591	2.28E-08	1.13E-07
ENSG00000175175	PPM1E	7.776325739	2.4367551	13.11589638	2.390564011	0.871264686	2.74378619	0.006074	0.015452
ENSG00000205710	C17orf107	53.91167299	17.388457	90.43488919	2.384240128	0.326257779	7.30784148	2.71E-13	1.85E-12
ENSG00000130762	ARHGEF16	38.97058895	12.398347	65.54283059	2.381080103	0.401736692	5.92696697	3.09E-09	1.63E-08
ENSG00000214814	FER1L6	7.828362663	2.5139458	13.14277951	2.371331933	0.85019126	2.78917468	0.005284	0.013608
ENSG00000205517	RGL3	5.783595618	1.8664488	9.700742387	2.354915532	0.982517897	2.39681693	0.016538	0.038173
ENSG00000152784	PRDM8	144.5236312	47.689301	241.3579618	2.354675431	0.224917828	10.4690475	1.20E-25	1.32E-24
ENSG00000165685	TMEM52B	14.48903864	4.6933456	24.28473165	2.354553219	0.683622938	3.44422793	0.000573	0.001766
ENSG00000171004	HS6ST2	57.728122	19.020294	96.43594992	2.352354885	0.314539455	7.47872754	7.50E-14	5.24E-13
ENSG00000130176	CNN1	6.799884267	2.2191439	11.38062468	2.351685033	0.917953227	2.56187893	0.010411	0.02519
ENSG00000113396	SLC27A6	11.66935746	3.8316834	19.50703146	2.350566831	0.68110773	3.45109405	0.000558	0.001724
ENSG00000162004	CCDC78	15.55325044	5.1289747	25.97752614	2.346784913	0.60597911	3.87271586	0.000108	0.000368
ENSG00000178882	RFLNA	1118.381291	367.99043	1868.772157	2.345994222	0.073617868	31.8671851	7.61E-223	6.36E-221
ENSG00000104432	IL7	7.831132844	2.6331779	13.02908777	2.333589138	0.84084086	2.77530416	0.005515	0.014142
ENSG00000124145	SDC4	2747.043213	910.03903	4584.0474	2.331017943	0.048837944	47.7296492	0	0
ENSG00000168811	IL12A	57.01413222	18.741679	95.28658523	2.330642795	0.326160667	7.14568932	8.95E-13	5.91E-12
ENSG00000164284	GRPEL2	1714.373834	571.39174	2857.355933	2.323780928	0.059402301	39.1193757	0	0
ENSG00000113319	RASGRF2	218.931303	73.419511	364.4430947	2.318689625	0.168104339	13.7931575	2.80E-43	4.67E-42
ENSG00000184371	CSF1	5399.713041	1806.257	8993.169067	2.315642758	0.042224915	54.8406737	0	0
ENSG00000100311	PDGFB	14007.42565	4687.9837	23326.86757	2.314588332	0.033396558	69.3061939	0	0
ENSG00000279821	AC145098.2	31.62757592	10.728728	52.52642395	2.305317545	0.428107026	5.38490939	7.25E-08	3.44E-07
ENSG00000273204	AC104506.1	9.596733627	3.2625259	15.93094137	2.30398716	0.78308353	2.94219846	0.003259	0.008773
ENSG00000176463	SLCO3A1	15.45463868	5.3060999	25.60317749	2.299511735	0.613180145	3.75014056	0.000177	0.000588
ENSG00000162840	MT2P1	9.270143401	3.0854007	15.45488607	2.294944838	0.819956649	2.79886118	0.005128	0.013252
ENSG00000013293	SLC7A14	78.62622973	26.95883	130.2936297	2.29284711	0.326203848	7.02887817	2.08E-12	1.35E-11
ENSG00000272720	AL022322.1	6.743540534	2.338376	11.14870511	2.291172513	0.967212692	2.36884041	0.017844	0.040747
ENSG00000243649	CFB	29.96146934	10.156866	49.76607233	2.289023711	0.424258175	5.39535557	6.84E-08	3.25E-07
ENSG00000204850	AC011484.1	8.581190674	2.9472776	14.21510379	2.288824789	0.801624005	2.85523484	0.004301	0.011281
ENSG00000270157	AC004918.3	212.9200845	72.524591	353.3155785	2.288558944	0.165274986	13.8469771	1.33E-43	2.22E-42
ENSG00000108679	LGALS3BP	99.30698101	33.990145	164.6238172	2.287856497	0.244636879	9.3520507	8.60E-21	7.98E-20
ENSG00000187123	LYPD6	317.9160123	108.43556	527.3964645	2.282175032	0.132017289	17.2869406	5.90E-67	1.45E-65
ENSG00000204642	HLA-F	114.1332047	38.97444	189.2919697	2.281392628	0.23902329	9.54464575	1.37E-21	1.31E-20
ENSG00000138696	BMPR1B	222.4289685	76.250596	368.6073409	2.274973885	0.162821928	13.9721591	2.31E-44	3.93E-43
ENSG00000185338	SOCS1	131.590385	45.099242	218.0815285	2.274779594	0.242817685	9.36826159	7.37E-21	6.87E-20
ENSG00000185215	TNFAIP2	3098.831183	1062.994	5134.668368	2.274156529	0.048668865	46.7271333	0	0
ENSG00000238113	LINC01410	12.19293085	4.2048248	20.18103688	2.27054875	0.667069849	3.40376462	0.000665	0.002029
ENSG00000138623	SEMA7A	293.0929146	101.4788	484.7070263	2.262178638	0.152619878	14.8223067	1.05E-49	1.96E-48
ENSG00000140416	TPM1	32635.86305	11279.744	53991.98236	2.258654345	0.023293983	96.9629945	0	0
ENSG00000134569	LRP4	474.5698885	164.27708	784.8626933	2.258169483	0.110257257	20.4809148	3.19E-93	1.14E-91
ENSG00000197046	SIGLEC15	19.67244509	6.8754494	32.46944077	2.25710032	0.556654447	4.05476024	5.02E-05	0.000179
ENSG00000182901	RGS7	74.73090765	25.958567	123.5032486	2.247409839	0.304713771	7.3754784	1.64E-13	1.13E-12
ENSG00000164694	FNDC1	7.163241061	2.4549041	11.87157802	2.247200902	0.904867072	2.48345969	0.013011	0.030833
ENSG00000165124	SVEP1	12.50912024	4.3213529	20.69688752	2.23808283	0.682834971	3.27763358	0.001047	0.003085
ENSG00000104312	RIPK2	2605.882578	913.70318	4298.061973	2.23579986	0.054327942	41.1537742	0	0
ENSG00000183762	KREMEN1	59.62274672	21.025273	98.22022073	2.23441753	0.305442914	7.31533595	2.57E-13	1.75E-12
ENSG00000123989	CHPF	36.48655845	12.868384	60.10473322	2.233456204	0.400748827	5.57320709	2.50E-08	1.23E-07
ENSG00000138435	CHRNA1	94.88947071	33.518218	156.2607237	2.228688949	0.241769813	9.21822672	3.02E-20	2.74E-19
ENSG00000165801	ARHGEF40	772.0261217	271.76598	1272.286263	2.227449716	0.097645949	22.8114913	3.53E-115	1.45E-113
ENSG00000268883	PNMA6B	12.64631464	4.4201387	20.87249059	2.227384192	0.654578216	3.40277776	0.000667	0.002036

ENSG00000273192	AL671710.1	7.140452125	2.5139458	11.76695844	2.22132423	0.957299099	2.32040773	0.020319	0.045818
ENSG00000239704	CDRT4	6.344232747	2.2395902	10.44887529	2.220202519	0.96441738	2.30211791	0.021329	0.047825
ENSG00000077150	NFKB2	2764.373612	977.58602	4551.161204	2.220115066	0.047265323	46.9713291	0	0
ENSG00000237181	AC147651.4	49.08284196	17.34486	80.8208238	2.212713864	0.356694184	6.20339205	5.53E-10	3.07E-09
ENSG00000166670	MMP10	1849.924585	657.80217	3042.047004	2.209003182	0.065893587	33.5237962	2.17E-246	2.09E-244
ENSG00000227683	AL358394.1	38.76762705	13.691044	63.84421021	2.202569315	0.404870429	5.44018322	5.32E-08	2.55E-07
ENSG00000027869	SH2D2A	59.74209692	21.142136	98.34205753	2.198995566	0.334116837	6.58151677	4.66E-11	2.78E-10
ENSG00000128165	ADM2	21.87162166	7.7810437	35.9621996	2.197565492	0.549636976	3.99821262	6.38E-05	0.000225
ENSG00000273486	AC096992.2	15.25935618	5.4998187	25.01889362	2.196421891	0.617187474	3.55875967	0.000373	0.00118
ENSG00000117525	F3	66.06485146	23.735235	108.3944682	2.194446559	0.284094507	7.72435406	1.12E-14	8.14E-14
ENSG00000183770	FOXL2	10.68192178	3.8316834	17.53216011	2.18977716	0.745370006	2.93783912	0.003305	0.008886
ENSG00000115738	ID2	166.3101619	60.123946	272.4963778	2.188781964	0.189708367	11.5376143	8.53E-31	1.08E-29
ENSG00000184838	PRR16	82.18250545	29.750171	134.6148403	2.188498011	0.269542228	8.11931408	4.69E-16	3.60E-15
ENSG00000267577	AC010327.3	6.280881882	2.2997806	10.26198317	2.182505682	0.950832478	2.29536299	0.021712	0.048573
ENSG00000166444	ST5	1330.972632	480.582	2181.363266	2.178950066	0.072828955	29.9187329	1.12E-196	8.19E-195
ENSG00000260549	MT1L	869.6694014	315.42576	1423.913044	2.172182498	0.089582057	24.247964	6.95E-130	3.23E-128
ENSG00000125637	PSD4	12.96846695	4.6751966	21.26173728	2.167362398	0.643523924	3.36795932	0.000757	0.002286
ENSG00000278869	BX539320.1	6.094106869	2.2202925	9.967921211	2.162543189	0.932589993	2.31885738	0.020403	0.04599
ENSG00000174938	SEZ6L2	387.3897234	141.83455	632.9449013	2.159781011	0.121417433	17.7880634	8.75E-71	2.27E-69
ENSG00000185972	CCIN	31.06078084	11.298628	50.82293415	2.154610287	0.440029055	4.89651822	9.75E-07	4.18E-06
ENSG00000269155	AL009178.2	16.48201401	6.0712737	26.89275436	2.151721037	0.579058155	3.715898	0.000202	0.000667
ENSG00000087303	NID2	3370.774678	1238.6235	5502.925819	2.151688604	0.045232579	47.5694437	0	0
ENSG00000116711	PLA2G4A	3145.729208	1158.9622	5132.496249	2.148077441	0.049029023	43.8123645	0	0
ENSG00000131459	GFPT2	1933.193699	713.60957	3152.777833	2.142918007	0.061008763	35.1247573	2.82E-270	3.04E-268
ENSG00000260231	KDM7A-DT	54.9523427	20.432965	89.47172058	2.128551677	0.313763611	6.78393415	1.17E-11	7.24E-11
ENSG00000286215	AL356534.1	17.74926607	6.6392825	28.8592496	2.116591996	0.565848768	3.74056129	0.000184	0.000609
ENSG00000250067	YJEFN3	22.48876779	8.509584	36.46795153	2.112289567	0.534771003	3.94989548	7.82E-05	0.000272
ENSG00000232926	AC000078.1	8.308070915	3.1035497	13.51259209	2.106480373	0.817465506	2.57684313	0.009971	0.024227
ENSG00000081059	TCF7	160.3882444	60.870157	259.9063314	2.093284584	0.183268164	11.4219761	3.25E-30	4.05E-29
ENSG00000142178	SIK1	44.83822556	17.029612	72.64683932	2.084438464	0.381336726	5.46613616	4.60E-08	2.22E-07
ENSG00000089127	OAS1	503.9516959	192.22366	815.6797287	2.080757112	0.114774005	18.1291671	1.88E-73	5.02E-72
ENSG00000160326	SLC2A6	3116.25115	1201.2097	5031.292639	2.064475597	0.048375307	42.6762271	0	0
ENSG00000260101	AC008074.2	11.76194266	4.6574542	18.86643109	2.049569925	0.689406794	2.9729471	0.00295	0.008015
ENSG00000187583	PLEKHN1	93.71569398	36.388233	151.0431546	2.043739813	0.264846103	7.71670714	1.19E-14	8.62E-14
ENSG00000127472	PLA2G5	8.291731694	3.2806749	13.3027885	2.03389133	0.830993265	2.4475425	0.014383	0.033724
ENSG00000162551	ALPL	153.9087691	60.831633	246.9859049	2.031227944	0.210949485	9.62897797	6.03E-22	5.84E-21
ENSG00000257093	KIAA1147	7608.253018	2992.348	12224.15799	2.030607645	0.030850751	65.8203631	0	0
ENSG00000132530	XAF1	715.9388253	281.7163	1150.161354	2.027019395	0.109365265	18.5343984	1.09E-76	3.08E-75
ENSG00000282034	AC106886.5	7.345687915	2.9075335	11.7838423	2.025364808	0.865617925	2.33979074	0.019295	0.043728
ENSG00000219992	AL391422.1	8.18530762	3.2806749	13.08994036	2.020483373	0.876811791	2.30435242	0.021203	0.047584
ENSG00000228536	LYPLAL1-AS1	13.64424629	5.364735	21.9237576	2.019752056	0.630879487	3.20148634	0.001367	0.003944
ENSG00000175841	FAM172BP	8.754550404	3.4585421	14.05055872	2.013047036	0.771378143	2.60967601	0.009063	0.022206
ENSG00000175264	CHST1	3233.779093	1288.5015	5179.056655	2.007029495	0.043904023	45.7140222	0	0
ENSG00000100906	NFKBIA	3716.784529	1482.6585	5950.910525	2.005332344	0.042078195	47.6572808	0	0
ENSG00000182545	RNASE10	34.80570843	14.010551	55.6008654	2.002074003	0.410620228	4.87573156	1.08E-06	4.62E-06
ENSG00000141682	PMAIP1	1126.25841	453.47455	1799.042268	1.991120657	0.073604941	27.0514537	3.67E-161	2.14E-159
ENSG00000168140	VASN	34.86346403	14.088484	55.63844384	1.983066284	0.383908112	5.16547117	2.40E-07	1.09E-06
ENSG00000104723	TUSC3	847.5024259	342.86996	1352.134887	1.980203401	0.080810081	24.5044106	1.33E-132	6.33E-131
ENSG00000179862	CITED4	323.4211448	131.07601	515.7662845	1.975136733	0.165672304	11.9219489	9.10E-33	1.20E-31
ENSG00000096696	DSP	11.75551651	4.7546847	18.75634832	1.971810582	0.654547798	3.0124776	0.002591	0.007127
ENSG00000105088	OLFM2	135.0654491	54.832071	215.2988269	1.967440417	0.220769893	8.91172429	5.02E-19	4.35E-18
ENSG00000143869	GDF7	282.4948387	114.94273	450.0469503	1.963064981	0.163496286	12.0067864	3.27E-33	4.36E-32
ENSG00000267648	AC060766.5	18.56645782	7.5248371	29.60807853	1.963000666	0.526447081	3.72877111	0.000192	0.000636
ENSG00000274333	CU633967.1	13.21650738	5.3431399	21.08987481	1.962825076	0.718918844	2.73024569	0.006329	0.016046
ENSG00000196209	SIRPB2	785.14123	321.77176	1248.5107	1.957440732	0.082310345	23.7812238	5.22E-125	2.32E-123
ENSG00000250539	KRT8P33	10.32647023	4.2650152	16.38792525	1.956905646	0.777984408	2.5153533	0.011891	0.028388
ENSG00000129667	RHBDF2	2223.410663	915.83621	3530.985116	1.947472872	0.054650863	35.6348057	4.05E-278	4.52E-276
ENSG00000173868	PHOSPHO1	8.437637619	3.4562447	13.4190305	1.946200238	0.850047673	2.28951893	0.022049	0.049237
ENSG00000225828	FAM229A	57.07958192	23.637598	90.52156618	1.93929385	0.308333133	6.28960577	3.18E-10	1.80E-09
ENSG00000110328	GALNT18	17.18859333	7.0154633	27.36172334	1.937675882	0.616376652	3.14365555	0.001669	0.004749
ENSG00000259238	AC092755.2	12.33730078	5.1675701	19.50703146	1.927609654	0.635132129	3.03497424	0.002406	0.006667
ENSG00000077942	FBLN1	10.56825234	4.3622457	16.77425901	1.927600883	0.704332112	2.73677836	0.006204	0.015759
ENSG00000113657	DPYSL3	23428.91238	9757.6543	37100.17048	1.9266633	0.024295766	79.3003727	0	0
ENSG00000123572	NRK	307.6856388	128.37804	486.9932355	1.919996953	0.134170337	14.3101448	1.89E-46	3.35E-45
ENSG00000148053	NTRK2	13.18225481	5.5623065	20.80220315	1.918384809	0.667026118	2.87602653	0.004027	0.010629
ENSG00000164056	SPRY1	692.5121475	289.83512	1095.189174	1.918150169	0.094730777	20.248437	3.67E-91	1.28E-89
ENSG00000146267	FAXC	31.91726708	13.341053	50.49348132	1.917643225	0.399782085	4.79672125	1.61E-06	6.78E-06
ENSG00000151136	BTBD11	67.98442002	28.548961	107.4198789	1.916715893	0.272663161	7.02961078	2.07E-12	1.34E-11
ENSG00000204876	AC021218.1	15.07264237	6.3058852	23.83939951	1.908378042	0.595032267	3.20718413	0.00134	0.003877
ENSG00000120549	KIAA1217	599.8800119	252.56397	947.1960578	1.906828962	0.099959448	19.0760254	4.00E-81	1.22E-79
ENSG00000204991	SPIRE2	35.60657805	14.889549	56.32360669	1.905432908	0.430754708	4.42347553	9.71E-06	3.76E-05
ENSG00000156804	FBXO32	967.4026972	407.16404	1527.641351	1.903286509	0.087949212	21.6407455	7.43E-104	2.90E-102
ENSG00000068078	FGFR3	78.42850417	32.911542	123.9454661	1.901196695	0.271665363	6.99830362	2.59E-12	1.67E-11
ENSG00000111886	GABRR2	8.278247877	3.4959888	13.06050698	1.898119	0.799626079	2.37375825	0.017608	0.040283
ENSG00000124225	PMEPA1	4385.642089	1856.6442	6914.639952	1.896937136	0.038542387	49.2169083	0	0
ENSG00000276710	CSPG4P10	12.59608688	5.3431399	19.84903381	1.88378536	0.637852871	2.95332269	0.003144	0.008487
ENSG00000229989	MIR181A1HG	8.22378083	3.4971375	12.95042421	1.880583658	0.786407869	2.39135916	0.016786	0.038672
ENSG00000169403	PTAFR	18.422599	7.8184904	29.0267076	1.880120303	0.534390533	3.51825151	0.000434	0.001361
ENSG00000057657	PRDM1	108.2738445	46.387343	170.1603456	1.877395338	0.215000547	8.73204914	2.50E-18	2.11E-17
ENSG00000182667	NTM	141.7862882	61.0678	222.5047762	1.876599576	0.205752253	9.12067572	7.47E-20	6.69E-19
ENSG00000106003	LFNG	436.3403241	187.10354	685.5771058	1.870753721	0.112401131	16.6435489	3.37E-62	7.75E-61
ENSG00000138735	PDE5A	239.9761373	102.98512	376.9671588	1.863295587	0.161298863	11.5518209	7.23E-31	9.19E-30
ENSG00000106366	SERPINE1	336313.954	145620.12	527007.787	1.855612433	0.018326891	101.250806	0	0
ENSG00000134516	DOCK2	72.53914768	31.380046	113.6982493	1.855348168	0.279068021	6.64837254	2.96E-11	1.79E-10
ENSG00000118946	PCDH17	226.420849	98.491304	354.3503944	1.845527873	0.161857269	11.4021933	4.08E-30	5.08E-29

ENSG00000157680	DGKI	8.76542573	3.8112371	13.71961437	1.837672288	0.774287673	2.37337149	0.017627	0.04032
ENSG00000162490	DRAXIN	85.56849622	37.464132	133.6728605	1.835496601	0.258895651	7.08971586	1.34E-12	8.79E-12
ENSG00000142920	AZIN2	29.72855367	12.965614	46.49149321	1.835257375	0.412603566	4.44799204	8.67E-06	3.37E-05
ENSG00000072041	SLC6A15	78.61608939	34.678128	122.5540509	1.827531946	0.254966482	7.16773409	7.62E-13	5.05E-12
ENSG00000129910	CDH15	16.30014439	7.110803	25.48948574	1.819543065	0.576322684	3.15716024	0.001593	0.004548
ENSG00000164099	PRSS12	199.6198293	88.395441	310.8442177	1.816293253	0.168101729	10.8047268	3.27E-27	3.74E-26
ENSG00000171608	PIK3CD	1019.716266	451.92602	1587.506514	1.811481226	0.074029839	24.4696092	3.11E-132	1.47E-130
ENSG00000132821	VSTM2L	19.04601063	8.3479039	29.74411733	1.810709186	0.549296383	3.29641563	0.000979	0.002903
ENSG00000152926	ZNF117	1055.123393	469.1402	1641.10659	1.80927691	0.0750173	24.1181289	1.61E-128	7.42E-127
ENSG00000162772	ATF3	180.6308464	80.090249	281.1714437	1.80848272	0.189006519	9.56836162	1.09E-21	1.04E-20
ENSG00000245694	CRNDE	25.52569425	11.298628	39.75276098	1.807918808	0.465649448	3.88257479	0.000103	0.000355
ENSG00000111817	DSE	3351.402033	1490.0794	5212.724641	1.807286612	0.048269797	37.4413551	8.30E-307	1.03E-304
ENSG00000170458	CD14	21.78526341	9.7466849	33.8238419	1.80724333	0.484868488	3.7272856	0.000194	0.00064
ENSG00000145901	TNIP1	10330.94409	4591.6678	16070.2204	1.806881464	0.028261112	63.935257	0	0
ENSG00000117228	GBP1	1532.884576	682.88306	2382.886092	1.804218464	0.060821154	29.664325	2.22E-193	1.57E-191
ENSG00000213344	PCNPP3	13.10444648	5.9161502	20.2927428	1.797530696	0.676367345	2.6576249	0.007869	0.019545
ENSG00000146374	RSPO3	228.8274573	102.18446	355.4704574	1.795061199	0.150491039	11.9280272	8.46E-33	1.11E-31
ENSG00000104938	CLEC4M	21.84078295	9.7042369	33.97732898	1.795001758	0.485757597	3.69526235	0.00022	0.000721
ENSG00000105605	CACNG7	25.73232491	11.572241	39.89240875	1.790993304	0.436725811	4.10095593	4.11E-05	0.000149
ENSG00000180535	BHLHA15	9.682000265	4.2816089	15.08239162	1.789685316	0.770915414	2.32150672	0.02026	0.045701
ENSG00000278709	NKILA	61.5461824	27.722448	95.36991649	1.788343391	0.288099067	6.20739043	5.39E-10	2.99E-09
ENSG00000137473	TTC29	59.73274148	26.915975	92.54950776	1.787566455	0.30765387	5.81031682	6.24E-09	3.22E-08
ENSG00000121933	TMIGD3	8.579479975	3.8895765	13.26938346	1.784805554	0.769930353	2.31813897	0.020442	0.046061
ENSG00000130508	PXDN	81576.53482	36749.637	126403.4329	1.782311054	0.022702715	78.5065156	0	0
ENSG00000130589	HELZ2	2922.986362	1317.3966	4528.576113	1.780869295	0.118609819	15.0145183	5.90E-51	1.13E-49
ENSG00000234155	LINC02535	19.63588015	8.8232771	30.44848324	1.780723727	0.519464227	3.42800068	0.000608	0.001871
ENSG00000263528	IKBKE	1839.533013	831.28467	2847.781361	1.778546087	0.05726682	31.0571824	9.13E-212	7.20E-210
ENSG00000217801	AL390719.1	11.19354437	5.066487	17.32060176	1.771137784	0.735310609	2.40869336	0.01601	0.037097
ENSG00000213062	AL021068.1	16.25626225	7.3890113	25.1235132	1.770539881	0.570548482	3.10322424	0.001914	0.005401
ENSG00000100368	CSF2RB	6028.237403	2736.9148	9319.56003	1.768249296	0.035167111	50.2813357	0	0
ENSG00000105855	ITGB8	904.7555977	409.96827	1399.542928	1.768218333	0.081547858	21.6831978	2.96E-104	1.16E-102
ENSG00000150347	ARID5B	1207.786609	549.09946	1866.473755	1.768117609	0.076279214	23.1795468	7.32E-119	3.13E-117
ENSG00000157404	KIT	3562.807072	1622.861	5502.753129	1.763588286	0.046599757	37.8454394	0	0
ENSG00000163132	MSX1	205.1851894	93.369628	317.0007513	1.763364528	0.162666492	10.8403673	2.22E-27	2.55E-26
ENSG00000279355	AGPAT4-IT1	8.987744095	4.0469973	13.92849085	1.762342917	0.759694253	2.31980551	0.020351	0.04588
ENSG00000238000	AC116347.1	17.73307887	8.1545917	27.31156606	1.757763638	0.54183359	3.24410238	0.001178	0.00344
ENSG00000184441	AP001062.1	224.2567729	102.24884	346.2647101	1.756824947	0.171237021	10.2596094	1.07E-24	1.14E-23
ENSG00000260578	AC110597.1	11.11984388	5.1085284	17.13115939	1.747464436	0.672178749	2.59970199	0.00933	0.022813
ENSG00000145247	OCIAD2	2635.503977	1212.7432	4058.264803	1.743213578	0.046191529	37.7388154	0	0
ENSG00000187498	COL4A1	251524.6853	115906.9	387142.4678	1.739849445	0.020273337	85.8195876	0	0
ENSG00000119900	OGFRL1	2667.330025	1234.061	4100.599034	1.732447903	0.04750953	36.4652715	3.94E-291	4.47E-289
ENSG00000122641	INHBA	3742.722604	1735.0263	5750.418903	1.727721645	0.041764081	41.3686016	0	0
ENSG00000246898	LINC00920	141.2780721	65.602098	216.9540458	1.723962663	0.186317595	9.25281731	2.19E-20	1.99E-19
ENSG00000078081	LAMP3	676.1109756	314.20103	1038.020922	1.723627131	0.089924701	19.1674491	6.92E-82	2.15E-80
ENSG00000088899	LZTS3	304.2962202	141.32051	467.2719345	1.720839671	0.131337373	13.1024371	3.19E-39	4.90E-38
ENSG00000151014	NOCT	807.7740566	376.4676	1239.080514	1.718081198	0.079604091	21.5828255	2.60E-103	1.01E-101
ENSG00000240288	GHRLOS	11.91144625	5.6009018	18.22199067	1.717796427	0.676192358	2.5403961	0.011073	0.026606
ENSG00000139899	CBLN3	105.7508279	49.308767	162.1928891	1.712693211	0.232867494	7.35479729	1.91E-13	1.31E-12
ENSG00000213801	ZNF321P	15.23458399	7.1929951	23.2761729	1.712356906	0.59411026	2.88222073	0.003949	0.01044
ENSG00000172331	BPGM	2535.050467	1185.2098	3884.891091	1.711844645	0.049112323	34.8557048	3.49E-266	3.65E-264
ENSG00000102878	HSF4	16.37732512	7.6803672	25.07428302	1.70906045	0.569475787	3.0011117	0.00269	0.007374
ENSG00000167604	AD000864.1	98.37606532	45.835115	150.9170156	1.707725783	0.237342084	7.19520851	6.24E-13	4.15E-12
ENSG00000276855	AC015922.3	19.82384602	9.3122045	30.33548753	1.707399722	0.527696599	3.23557083	0.001214	0.003537
ENSG00000168874	ATOH8	811.7144051	381.33885	1242.089962	1.705963638	0.089342092	19.0947357	2.79E-81	8.62E-80
ENSG00000145817	YIPF5	6707.73641	3153.6451	10261.82776	1.702362218	0.034407788	49.4760721	0	0
ENSG00000005187	ACSM3	471.5760746	221.48031	721.6718375	1.701319034	0.115202494	14.7680746	2.35E-49	4.35E-48
ENSG00000138675	FGF5	660.6144575	310.56333	1010.665586	1.69916183	0.091401766	18.5900328	3.87E-77	1.10E-75
ENSG00000106799	TGFBR1	1413.90965	666.72096	2161.098339	1.696758021	0.06951337	24.4090889	1.37E-131	6.42E-130
ENSG00000213088	ACKR1	117.3115297	55.606912	179.0161472	1.691778035	0.209353127	8.0809781	6.42E-16	4.90E-15
ENSG00000135919	SERPINE2	1625.551334	770.92625	2480.176417	1.688946105	0.064696248	26.1057815	3.13E-150	1.72E-148
ENSG00000198832	SELENOM	1542.306588	730.20049	2354.412684	1.687914984	0.059781955	28.2345232	2.20E-175	1.41E-173
ENSG00000164823	OSGIN2	2605.798794	1233.7854	3977.812166	1.687844234	0.052284536	32.2819013	1.26E-228	1.08E-226
ENSG00000232082	RPS6KA2-IT1	21.29462955	10.218612	32.37064706	1.684601949	0.501995846	3.35580854	0.000791	0.002383
ENSG00000267658	AC099811.3	21.5957458	10.37644	32.81505209	1.683083049	0.566742351	2.96974992	0.00298	0.008091
ENSG00000133657	ATP13A3	7302.505028	3478.0436	11126.96649	1.678075325	0.032412794	51.7720052	0	0
ENSG00000282057	AC092807.3	57.03705227	27.187627	86.88647771	1.671909162	0.31689334	5.27593657	1.32E-07	6.12E-07
ENSG00000253816	AC138866.1	10.52538338	5.0097426	16.04102414	1.668478059	0.680659166	2.45126804	0.014235	0.033415
ENSG00000134775	FHOD3	62.01753989	29.589233	94.44584718	1.666541913	0.301897628	5.52022196	3.39E-08	1.66E-07
ENSG00000018236	CNTN1	17.19871859	8.1950778	26.20235939	1.66584172	0.580520481	2.8695658	0.00411	0.010832
ENSG00000165072	MAMDC2	392.6144893	188.34679	596.8821854	1.66212451	0.11508788	14.4422202	2.81E-47	5.06E-46
ENSG00000164733	CTSB	75862.44818	36430.649	115294.2472	1.662040258	0.021215122	78.342245	0	0
ENSG00000278531	AL512324.3	19.03594282	9.0953353	28.97655032	1.659584922	0.528036291	3.14293723	0.001673	0.004758
ENSG00000240184	PCDHGC3	1434.449326	689.85782	2179.040826	1.657922788	0.062370697	26.5817581	1.10E-155	6.32E-154
ENSG00000179542	SLITRK4	54.58663091	26.505794	82.66746806	1.65163549	0.312355268	5.28768252	1.24E-07	5.76E-07
ENSG00000121039	RDH10	552.62086	267.72646	837.5152619	1.646855881	0.104473146	15.7634372	5.55E-56	1.14E-54
ENSG00000114270	COL7A1	23.09749335	11.1991	34.99588699	1.646671479	0.485156324	3.39410495	0.000689	0.002094
ENSG00000234745	HLA-B	14086.9552	6823.4298	21350.48061	1.645875287	0.033632347	48.9372719	0	0
ENSG00000130477	UNC13A	19.59122996	9.4503277	29.73213223	1.643070609	0.499377033	3.29024064	0.001001	0.002963
ENSG00000235385	LINC02154	53.09904672	25.815849	80.38224464	1.640646407	0.328032298	5.00147826	5.69E-07	2.49E-06
ENSG00000140323	DISP2	45.87280845	22.241521	69.50409626	1.63943722	0.32689836	5.01512831	5.30E-07	2.33E-06
ENSG00000145365	TIFA	996.2130448	485.00083	1507.425257	1.638642472	0.077965083	21.0176453	4.52E-98	1.68E-96
ENSG00000183196	CHST6	87.84307752	42.650186	133.0359686	1.635753311	0.246324559	6.64064241	3.12E-11	1.89E-10
ENSG00000206337	HCP5	15.6341716	7.6054739	23.66286934	1.634820633	0.618786349	2.64197915	0.008242	0.020369
ENSG00000115604	IL18R1	696.1915526	339.58551	1052.797597	1.633545021	0.0856229	19.0783661	3.82E-81	1.17E-79
ENSG00000198108	CHSY3	638.5857889	311.57819	965.5933883	1.632687776	0.089794375	18.1825174	7.10E-74	1.91E-72

ENSG00000131016	AKAP12	41309.01374	20176.049	62441.97818	1.629872234	0.022709291	71.7711642	0	0
ENSG00000065320	NTN1	24.21654987	11.789446	36.64365403	1.623343694	0.486388408	3.33754602	0.000845	0.00253
ENSG00000060558	GNA15	11.03060312	5.404479	16.65672722	1.619606263	0.688574371	2.35211523	0.018667	0.042431
ENSG00000144668	ITGA9	10.9553417	5.364735	16.54594841	1.617395038	0.685623831	2.35901228	0.018324	0.041718
ENSG00000172403	SYNPO2	15.49103415	7.6236229	23.35844544	1.613116749	0.555048663	2.90626184	0.003658	0.009746
ENSG00000019549	SNAI2	17.58145591	8.7407496	26.42216224	1.607026046	0.605701243	2.65316617	0.007974	0.019781
ENSG00000189077	TMEM120A	1875.057027	927.05123	2823.062823	1.604689381	0.058043276	27.6464305	3.08E-168	1.87E-166
ENSG00000039139	DNAH5	63.85230693	31.667214	96.0374	1.600710505	0.292143886	5.47918537	4.27E-08	2.07E-07
ENSG00000223403	MEG9	61.852622	30.664318	93.04092607	1.596084275	0.302377755	5.27844475	1.30E-07	6.04E-07
ENSG00000276116	FUT8-AS1	71.31192982	35.59998	107.0238792	1.590242078	0.273835532	5.80728902	6.35E-09	3.28E-08
ENSG00000265222	AC079336.2	10.67600275	5.2840982	16.06790727	1.588711187	0.68424051	2.32186076	0.02024	0.045663
ENSG00000175471	MCTP1	5315.783099	2652.9327	7978.633461	1.588147569	0.034888274	45.5209551	0	0
ENSG00000133805	AMPD3	183.3338619	91.650597	275.017127	1.579993891	0.173060309	9.12972997	6.87E-20	6.16E-19
ENSG00000114529	C3orf52	176.4529773	88.774326	264.131629	1.577287315	0.174967808	9.01472868	1.97E-19	1.74E-18
ENSG00000167608	TMC4	15.10177054	7.5634325	22.6401086	1.574258452	0.564886875	2.78685613	0.005322	0.013698
ENSG00000166770	ZNF667-AS1	20.81314771	10.433519	31.19277609	1.571507215	0.482254969	3.25866464	0.001119	0.003283
ENSG00000081377	CDC14B	66.97934933	33.768274	100.1904244	1.571025163	0.308830066	5.08702143	3.64E-07	1.62E-06
ENSG00000196739	COL27A1	3555.760852	1795.6288	5315.892918	1.564935483	0.0467016	33.5092476	3.53E-246	3.39E-244
ENSG00000183307	TMEM121B	28.39248288	14.344691	42.44027492	1.563724293	0.416929492	3.75057252	0.000176	0.000587
ENSG00000137872	SEMA6D	777.5046758	394.15141	1160.857945	1.561636974	0.083802979	18.6346237	1.68E-77	4.81E-76
ENSG00000253540	FAM86HP	15.73628084	7.9570202	23.51554149	1.560140863	0.560189374	2.78502402	0.005352	0.013766
ENSG00000020577	SAMD4A	3946.292741	1999.9183	5892.667144	1.559757184	0.041825233	37.2922529	2.19E-304	2.69E-302
ENSG00000171223	JUNB	2059.88569	1043.4572	3076.314154	1.559716024	0.053718594	29.0349378	2.38E-185	1.64E-183
ENSG00000139200	PIANP	29.78663397	15.109123	44.46414539	1.559006026	0.408581338	3.81565647	0.000136	0.000459
ENSG00000128487	SPECC1	894.7507867	453.43281	1336.068764	1.55682035	0.07740492	20.1126794	5.72E-90	1.96E-88
ENSG00000205464	ATP6AP1L	20.26695035	10.273801	30.26009959	1.555497932	0.539242628	2.88459749	0.003919	0.010368
ENSG00000231185	SPRY4-AS1	14.52854036	7.426458	21.63062275	1.551154656	0.570488397	2.71899423	0.006548	0.016552
ENSG00000261008	LINC01572	30.03995929	15.265395	44.81452385	1.550713693	0.399736989	3.879335	0.000105	0.000359
ENSG00000171017	LRRC8E	91.65026044	46.386124	136.9143973	1.549501194	0.24744654	6.26196347	3.80E-10	2.14E-09
ENSG00000152689	RASGRP3	1963.943391	1000.8525	2927.034237	1.549289459	0.054393329	28.4830786	1.90E-178	1.26E-176
ENSG00000100592	DAAM1	2361.44605	1203.5494	3519.342735	1.548176092	0.047774347	32.4060124	2.26E-230	1.98E-228
ENSG00000187608	ISG15	1593.190172	811.29148	2375.088866	1.54735128	0.070488219	21.9519133	8.30E-107	3.28E-105
ENSG00000186684	CYP27C1	99.34436567	50.650061	148.0386701	1.546787161	0.249556638	6.19814072	5.71E-10	3.17E-09
ENSG00000137965	IFI44	746.7628397	381.81625	1111.709427	1.541876271	0.09059255	17.0199015	5.85E-65	1.40E-63
ENSG00000086991	NOX4	2190.438706	1119.8849	3260.992466	1.541833387	0.050815787	30.3416219	3.24E-202	2.45E-200
ENSG00000135454	B4GALNT1	17.66756008	9.0764443	26.2586759	1.541516643	0.526907396	2.9255931	0.003438	0.009205
ENSG00000140092	FBLN5	2063.254505	1055.4349	3071.074133	1.540889057	0.055377624	27.8251204	2.15E-170	1.33E-168
ENSG00000285816	AP000944.2	47.22215262	24.266204	70.17810168	1.537976016	0.333010601	4.61839957	3.87E-06	1.56E-05
ENSG00000115828	QPCT	1358.941172	697.3098	2020.572543	1.534854295	0.066289465	23.1538193	1.33E-118	5.65E-117
ENSG00000120262	CCDC170	47.27681095	24.101819	70.45180242	1.534135503	0.375054883	4.09042935	4.31E-05	0.000155
ENSG00000141424	SLC39A6	1920.906938	990.31091	2851.502966	1.527900082	0.057329042	26.6514149	1.72E-156	9.90E-155
ENSG00000276136	AC016957.2	17.65411986	9.0181446	26.29009511	1.525169178	0.531256672	2.87087064	0.004093	0.01079
ENSG00000273174	AC108673.2	39.29212037	20.237691	58.34655006	1.522678989	0.357599507	4.25805673	2.06E-05	7.72E-05
ENSG00000145431	PDGFC	663.0161078	342.50553	983.5266808	1.519827203	0.09078895	16.7402222	6.67E-63	1.55E-61
ENSG00000111339	ART4	2569.302461	1329.8931	3808.711829	1.518390393	0.0463148	32.7841294	9.91E-236	9.11E-234
ENSG00000183044	ABAT	208.7140606	107.90189	309.526234	1.517848185	0.157271354	9.65114207	4.86E-22	4.73E-21
ENSG00000176857	GJA1P1	11.11634928	5.7764717	16.45622686	1.515554304	0.649955816	2.33178051	0.019712	0.044554
ENSG00000236830	CBR3-AS1	22.29727325	11.572241	33.02230543	1.514284204	0.467440818	3.2395207	0.001197	0.003493
ENSG00000179256	SMCO3	26.51209515	13.731195	39.29299578	1.514233427	0.470284704	3.21982283	0.001283	0.003723
ENSG00000099822	HCN2	119.36818	62.010434	176.7259256	1.513155259	0.216477901	6.98988327	2.75E-12	1.77E-11
ENSG00000105877	DNAH11	70.67866496	36.640658	104.7166714	1.512460394	0.261393139	5.78615184	7.20E-09	3.70E-08
ENSG00000134871	COL4A2	188745.2133	98026.654	279463.7722	1.511366648	0.021056357	71.7772137	0	0
ENSG00000278341	AC138028.6	36.30834387	18.862467	53.75422114	1.509747926	0.369201243	4.08922763	4.33E-05	0.000156
ENSG00000179630	LACC1	357.8797542	186.26051	529.4990014	1.508277448	0.120244605	12.5434106	4.32E-36	6.11E-35
ENSG00000166741	NNMT	8003.852947	4165.548	11842.15787	1.507297629	0.030025147	50.2011743	0	0
ENSG00000111859	NEDD9	4488.593568	2340.4543	6636.732857	1.50537903	0.043164098	34.8757212	1.74E-266	1.82E-264
ENSG00000169604	ANTXR1	1231.547785	641.94551	1821.150063	1.505229584	0.070675624	21.2977189	1.19E-100	4.52E-99
ENSG00000221843	C2orf16	33.82348493	17.559025	50.08794452	1.505189732	0.447466738	3.3638025	0.000769	0.002319
ENSG00000170801	HTRA3	1082.327203	564.11508	1600.539324	1.503229257	0.080450135	18.6852297	6.53E-78	1.89E-76
ENSG00000145358	DDIT4L	153.0680253	79.74692	226.3891303	1.503034965	0.180331985	8.33482183	7.76E-17	6.15E-16
ENSG00000126562	WNK4	108.6391962	56.858716	160.4196763	1.502402377	0.220003496	6.82899318	8.55E-12	5.36E-11
ENSG00000135932	CAB39	5795.079737	3043.318	8546.841446	1.490754885	0.036224128	41.1536441	0	0
ENSG00000116741	RGS2	515.6897682	271.36838	760.0111554	1.487832886	0.111035545	13.399609	6.08E-41	9.74E-40
ENSG00000075643	MOCOS	111.8433237	58.744798	164.9418494	1.487417726	0.20960118	7.09641866	1.28E-12	8.38E-12
ENSG00000091129	NRCAM	9403.780555	4946.4807	13861.08043	1.48699803	0.029496545	50.412618	0	0
ENSG00000006016	CRLF1	89.08880033	46.904351	131.2732493	1.484658731	0.245407518	6.04976875	1.45E-09	7.83E-09
ENSG00000249685	AC079921.2	15.99447966	8.35135	23.63760936	1.484398883	0.585921614	2.53344278	0.011295	0.027087
ENSG00000130309	COLGALT1	22007.91119	11601.1	32414.72221	1.482630607	0.025701301	57.6869863	0	0
ENSG00000152661	GJA1	37426.2885	19754.409	55098.168	1.480097807	0.022540965	65.6625752	0	0
ENSG00000206530	CFAP44	61.25619752	32.278006	90.23438883	1.476100759	0.284201093	5.19386024	2.06E-07	9.38E-07
ENSG00000147454	SLC25A37	1756.734638	933.42993	2580.039348	1.469450761	0.061774646	23.7872793	4.52E-125	2.01E-123
ENSG00000144810	COL8A1	24342.71597	12932.147	35753.28542	1.467202735	0.024905498	58.9107972	0	0
ENSG00000196074	SYCP2	12.4517086	6.6018359	18.30158133	1.466656654	0.64170363	2.28556702	0.02228	0.049697
ENSG00000115267	IFIH1	1021.171289	542.7887	1499.55388	1.465292985	0.074128963	19.7668081	5.75E-87	1.91E-85
ENSG00000154928	EPHB1	93.23590061	49.846699	136.6251025	1.463454203	0.235226542	6.22146715	4.93E-10	2.75E-09
ENSG00000253159	PCDHGA12	672.4659213	358.33516	986.5966856	1.460546053	0.0869027	16.806682	2.18E-63	5.09E-62
ENSG00000166922	SCG5	124.8973453	66.504247	183.290444	1.458599645	0.199308023	7.31831877	2.51E-13	1.71E-12
ENSG00000136842	TMOD1	275.4830518	147.37837	403.5877361	1.45551177	0.146900553	9.9081436	3.84E-23	3.88E-22
ENSG00000152932	RAB3C	36.3430231	19.413475	53.27257102	1.455363069	0.364818368	3.98928124	6.63E-05	0.000233
ENSG00000110446	SLC15A3	271.9315953	145.9854	397.8777894	1.453342638	0.147091353	9.88054436	5.06E-23	5.09E-22
ENSG00000078401	EDN1	18345.16072	9825.4533	26864.86818	1.451258903	0.023872685	60.7916086	0	0
ENSG00000184922	FMNL1	146.6164474	78.533712	214.6991829	1.449923404	0.184025719	7.87891723	3.30E-15	2.45E-14
ENSG00000113739	STC2	4044.911243	2169.0621	5920.760344	1.447356118	0.046022704	31.4487413	4.37E-217	3.57E-215
ENSG00000107201	DDX58	2183.983826	1174.3999	3193.567756	1.443695922	0.055009775	26.2443526	8.29E-152	4.63E-150
ENSG00000110911	SLC11A2	2658.30904	1433.4766	3883.141486	1.436161314	0.050140694	28.6426292	1.98E-180	1.33E-178

ENSG00000156453	PCDH1	17041.71252	9204.8855	24878.53955	1.434575261	0.030007402	47.8073789	0	0
ENSG00000125148	MT2A	33035.56059	17845.68	48225.44142	1.4338782	0.03246056	44.1729344	0	0
ENSG00000111912	NCOA7	9426.712759	5096.4709	13756.95464	1.432935935	0.032113973	44.6203259	0	0
ENSG00000171791	BCL2	261.0253604	141.30094	380.7497768	1.432662131	0.143576437	9.9783931	1.90E-23	1.94E-22
ENSG00000238197	PAXBP1-AS1	14.47833421	7.7810437	21.1756247	1.427262107	0.605074311	2.35882119	0.018333	0.041734
ENSG00000168675	LDLRAD4	97.52045464	53.067925	141.972984	1.426348565	0.231696311	6.15611252	7.46E-10	4.10E-09
ENSG00000165617	DACT1	27.31497692	14.874511	39.75544285	1.425564714	0.41993465	3.39472991	0.000687	0.00209
ENSG00000185022	MAFF	3328.692747	1807.2719	4850.113549	1.423203157	0.04565232	31.1748264	2.34E-213	1.86E-211
ENSG00000110218	PANX1	2180.919061	1185.1921	3176.646037	1.423010299	0.04999808	28.4612992	3.53E-178	2.33E-176
ENSG00000108932	SLC16A6	55.49109007	30.279584	80.70259597	1.422443573	0.301090822	4.72430068	2.31E-06	9.55E-06
ENSG00000131773	KHDRBS3	355.500162	193.88994	517.1103795	1.420583133	0.126549176	11.2255423	3.06E-29	3.73E-28
ENSG00000136560	TANK	3848.033518	2094.2801	5601.786901	1.420345092	0.041592974	34.1486783	1.40E-255	1.39E-253
ENSG00000116032	GRIN3B	14.35479529	7.7390023	20.97058826	1.420036451	0.611724913	2.32136442	0.020267	0.045712
ENSG00000197927	C2orf27A	452.9862042	246.49172	659.480688	1.419278674	0.106888262	13.2781528	3.10E-40	4.88E-39
ENSG00000233117	LINC00702	57.75883896	31.400492	84.11718555	1.417855154	0.308102043	4.60190118	4.19E-06	1.68E-05
ENSG00000172164	SNTB1	162.5678728	88.759623	236.3761229	1.416400084	0.194024011	7.30012784	2.87E-13	1.95E-12
ENSG00000253837	AC090197.1	20.49771664	11.157058	29.83837496	1.415552435	0.503906957	2.80915438	0.004967	0.012866
ENSG00000152669	CCNO	20.75984431	11.257735	30.2619538	1.414431108	0.498321499	2.8383907	0.004534	0.011852
ENSG00000213928	IRF9	33.01144474	18.133591	47.88929865	1.409710437	0.387149288	3.64125799	0.000271	0.000878
ENSG00000154654	NCAM2	89.64508693	49.000817	130.2893568	1.4084453	0.227401988	6.19363672	5.88E-10	3.25E-09
ENSG00000136514	RTP4	33.05298768	18.058291	48.0476845	1.406666041	0.411805238	3.41585272	0.000636	0.001948
ENSG00000143469	SYT14	311.9105541	171.07798	452.7431329	1.402815664	0.130382765	10.7592109	5.36E-27	6.09E-26
ENSG00000101384	JAG1	6128.943202	3367.2848	8890.601633	1.400648621	0.037434118	37.4163648	2.12E-306	2.61E-304
ENSG00000124243	BCAS4	49.5256586	27.212668	71.83864931	1.400335813	0.334953908	4.18068212	2.91E-05	0.000107
ENSG00000133065	SLC41A1	2445.680729	1346.9794	3544.38206	1.397721579	0.052288458	26.7309774	2.05E-157	1.19E-155
ENSG00000134070	IRAK2	1210.05101	666.18086	1753.921165	1.396629389	0.064961911	21.4992043	1.58E-102	6.13E-101
ENSG00000180071	ANKRD18A	37.14717234	20.611239	53.68310604	1.391803963	0.388711829	3.58055469	0.000343	0.001091
ENSG00000035664	DAPK2	125.5280318	69.495528	181.560536	1.3861912	0.197023552	7.03566242	1.98E-12	1.29E-11
ENSG00000124216	SNAI1	932.8079493	516.73357	1348.882325	1.386175609	0.081581018	16.9913988	9.51E-65	2.27E-63
ENSG00000144476	ACKR3	2363.859997	1311.224	3416.495982	1.383463607	0.05567063	24.8508704	2.53E-136	1.24E-134
ENSG00000256802	AC022613.1	143.4836005	79.770477	207.1967237	1.381850797	0.195077719	7.08359112	1.40E-12	9.18E-12
ENSG00000064932	SBNO2	4588.284053	2547.0444	6629.52367	1.37965005	0.037151949	37.1353342	7.56E-302	9.03E-300
ENSG00000205885	C1RL-AS1	55.1449516	30.671617	79.6182866	1.37957157	0.296282511	4.65627068	3.22E-06	1.31E-05
ENSG00000229152	ANKRD10-IT1	40.34835491	22.276263	58.42044647	1.379527785	0.365747122	3.77180763	0.000162	0.000542
ENSG00000223804	AC244669.1	19.12025379	10.670835	27.56967272	1.377558753	0.509182347	2.70543306	0.006822	0.017156
ENSG00000198517	MAFK	1804.187495	1003.0643	2605.310692	1.374716328	0.069839761	19.6838636	2.97E-86	9.75E-85
ENSG00000136404	TM6SF1	1109.194746	618.75193	1599.637559	1.371616423	0.069982077	19.5995388	1.56E-85	5.09E-84
ENSG00000025039	RRAGD	19.39040808	10.82481	27.95600647	1.37055222	0.495765471	2.76451729	0.005701	0.01458
ENSG00000146232	NFKBIE	1018.220287	567.68745	1468.753126	1.368931533	0.077028704	17.7717066	1.17E-70	3.03E-69
ENSG00000090530	P3H2	150.3898781	83.636832	217.1429238	1.368930838	0.18587108	7.36494798	1.77E-13	1.22E-12
ENSG00000188002	AC026412.1	47.46919516	26.500792	68.43759786	1.367429045	0.338160459	4.04372838	5.26E-05	0.000188
ENSG00000107099	DOCK8	112.9298793	63.323577	162.5361812	1.365683729	0.208493096	6.55025875	5.74E-11	3.41E-10
ENSG00000120337	TNFSF18	395.2845255	220.77803	569.7910188	1.365233378	0.111381741	12.2572458	1.54E-34	2.09E-33
ENSG00000120051	CFAP58	44.2330485	24.910254	63.55584251	1.361669523	0.365728224	3.7231732	0.000197	0.00065
ENSG00000213462	ERV3-1	1333.087771	747.60082	1918.574716	1.361559136	0.066775173	20.3902001	2.04E-92	7.23E-91
ENSG00000166750	SLFN5	5568.231775	3120.3297	8016.133874	1.361316134	0.03622996	37.5743209	0	0
ENSG00000268027	AC243960.1	33.08204459	18.504028	47.66006095	1.361243073	0.408776303	3.33004399	0.000868	0.002593
ENSG00000026508	CD44	16966.15462	9524.0003	24408.30896	1.357688783	0.024479201	55.4629524	0	0
ENSG00000179044	EXOC3L1	508.8085255	285.50025	732.1168027	1.354834331	0.105042469	12.8979672	4.62E-38	6.91E-37
ENSG00000072682	P4HA2	3197.032289	1798.1899	4595.874696	1.354624107	0.046514787	29.1224404	1.87E-186	1.29E-184
ENSG00000197647	ZNF433	18.00562361	10.079676	25.93157159	1.353902293	0.528725486	2.56069043	0.010446	0.025263
ENSG00000129116	PALLD	3971.559632	2235.3415	5707.777727	1.352298465	0.041105735	32.8980485	2.34E-237	2.21E-235
ENSG00000130635	COL5A1	47781.42703	26921.453	68641.40138	1.350380137	0.023338483	57.8606643	0	0
ENSG00000129465	RIPK3	139.5720882	78.696646	200.4475301	1.346655649	0.216867628	6.20957431	5.31E-10	2.96E-09
ENSG00000259345	AC013652.1	56.32540104	31.887122	80.76367962	1.345390035	0.32206355	4.17740546	2.95E-05	0.000108
ENSG00000139926	FRMD6	8000.012937	4520.2849	11479.74097	1.344661681	0.02973444	45.2223649	0	0
ENSG00000279419	AC004925.1	19.76879597	11.297885	28.23970648	1.332209357	0.513851758	2.59259472	0.009525	0.023244
ENSG00000119917	IFIT3	377.6569493	214.70642	540.6074755	1.331518306	0.119842409	11.1105769	1.11E-28	1.34E-27
ENSG00000101187	SLCO4A1	354.3893866	201.20747	507.5713015	1.331020341	0.119030976	11.1821342	4.99E-29	6.05E-28
ENSG00000106537	TSPAN13	2428.785158	1383.3029	3474.267452	1.329170581	0.047445133	28.0148986	1.07E-172	6.74E-171
ENSG00000095015	MAP3K1	1081.905647	615.90471	1547.906582	1.328217919	0.072278413	18.3764123	2.03E-75	5.60E-74
ENSG00000148677	ANKRD1	23746.45738	13530.44	33962.47466	1.327339972	0.027182442	48.8307839	0	0
ENSG00000128641	MYO1B	6854.902099	3908.7706	9801.033586	1.326773801	0.034077118	38.9344492	0	0
ENSG00000172985	SH3RF3	1994.277449	1137.5634	2850.991482	1.326589097	0.053826672	24.6455717	4.11E-134	1.98E-132
ENSG00000136867	SLC31A2	259.2098767	147.63065	370.7891029	1.326018499	0.135622303	9.77728932	1.41E-22	1.40E-21
ENSG00000158321	AUTS2	19.57396736	11.178653	27.96928136	1.325678144	0.54859554	2.41649457	0.015671	0.03639
ENSG00000163297	ANTXR2	7842.943741	4474.3567	11211.53073	1.324849072	0.030725461	43.1189323	0	0
ENSG00000198691	ABCA4	174.9750035	99.534614	250.4153926	1.324712744	0.18054748	7.33719875	2.18E-13	1.49E-12
ENSG00000196932	TMEM26	26.48128754	15.168164	37.7944108	1.324565656	0.437512209	3.02749415	0.002466	0.006818
ENSG00000167771	RCOR2	30.90245134	17.702556	44.10324624	1.321969438	0.39595505	3.33868564	0.000842	0.002521
ENSG00000075426	FOSL2	5878.11368	3360.2298	8395.997601	1.321525156	0.032968855	40.0840471	0	0
ENSG00000258405	ZNF578	69.03200867	39.630384	98.4336333	1.318675422	0.263000987	5.01395616	5.33E-07	2.34E-06
ENSG00000156140	ADAMTS3	43.84512583	25.228949	62.46130281	1.315521087	0.329845622	3.98829331	6.66E-05	0.000234
ENSG00000131323	TRAF3	2696.855457	1548.3658	3845.345103	1.311256003	0.054536384	24.0436916	9.72E-128	4.43E-126
ENSG00000105696	TMEM59L	38.16949737	21.927421	54.41157376	1.310755992	0.35133021	3.7308377	0.000191	0.000631
ENSG00000131015	ULBP2	362.1725561	208.5797	515.7654161	1.310254797	0.125909498	10.4063222	2.32E-25	2.53E-24
ENSG00000135750	KCNK1	29.51454797	16.989126	42.03997023	1.308268052	0.475156817	2.75333954	0.005899	0.015058
ENSG00000182791	CCDC87	20.03740593	11.57339	28.50142211	1.305997942	0.484607874	2.69495815	0.00704	0.017635
ENSG00000242622	AC092910.3	16.8376611	9.6270462	24.04827599	1.30492919	0.543253562	2.40206283	0.016303	0.037691
ENSG00000010319	SEMA3G	3895.786596	2245.734	5545.839239	1.30489405	0.043744193	29.8301092	1.59E-195	1.15E-193
ENSG00000272269	AL138724.1	166.6339654	96.332421	236.9355095	1.302615171	0.16902545	7.70662153	1.29E-14	9.31E-14
ENSG00000135124	P2RX4	2412.89605	1393.3998	3432.392331	1.301133215	0.050355515	25.8389416	3.24E-147	1.72E-145
ENSG00000175746	C15orf54	891.4201893	515.53592	1267.304463	1.299831774	0.081270481	15.9938979	1.41E-57	2.98E-56
ENSG00000064547	LPAR2	48.97968681	28.214751	69.74462302	1.29766045	0.326793392	3.9708895	7.16E-05	0.000251
ENSG00000158023	WDR66	103.4476062	59.728732	147.1664806	1.297173591	0.225264293	5.75845187	8.49E-09	4.35E-08

ENSG00000065534	MYLK	1023.737937	593.00949	1454.466381	1.29658962	0.07632562	16.9876069	1.01E-64	2.42E-63
ENSG00000204764	RANBP17	270.1252105	156.80406	383.4463601	1.295175372	0.145334161	8.91170638	5.03E-19	4.35E-18
ENSG00000116017	ARID3A	674.1183259	391.13335	957.1032988	1.292307136	0.091035775	14.1955966	9.76E-46	1.71E-44
ENSG00000067798	NAV3	1575.09727	912.83759	2237.356946	1.291648535	0.060129008	21.4812881	2.33E-102	9.00E-101
ENSG00000167549	CORO6	101.2970065	58.742501	143.8515124	1.289168479	0.222596114	5.79151385	6.98E-09	3.59E-08
ENSG00000006451	RALA	23030.20045	13421.818	32638.58327	1.282223012	0.023382627	54.8365683	0	0
ENSG00000261888	AC144831.1	27.8473836	16.24514	39.44962686	1.282095859	0.443524186	2.89070111	0.003844	0.010192
ENSG00000065621	GSTO2	35.65860935	20.766362	50.55085656	1.282076241	0.363775782	3.52435843	0.000425	0.001332
ENSG00000146592	CREB5	399.0195338	232.66444	565.3746234	1.281994424	0.108419013	11.8244429	2.92E-32	3.80E-31
ENSG00000100092	SH3BP1	23.66027698	13.811831	33.50872269	1.281331327	0.442236104	2.89739195	0.003763	0.009995
ENSG00000178053	MLF1	197.4669765	115.58773	279.3462194	1.279174355	0.161297977	7.93050465	2.18E-15	1.63E-14
ENSG00000130222	GADD45G	24.50953657	14.325393	34.69367998	1.278820029	0.471573495	2.7118149	0.006692	0.016873
ENSG00000170323	FABP4	5490.417506	3207.504	7773.331003	1.277589965	0.037757823	33.8364306	5.75E-251	5.62E-249
ENSG00000090376	IRAK3	1141.774879	668.18649	1615.36327	1.27556137	0.072270899	17.6497234	1.02E-69	2.62E-68
ENSG00000280594	BTG3-AS1	36.64574316	21.453603	51.83788319	1.273326927	0.357046805	3.56627453	0.000362	0.001149
ENSG00000160606	TLCD1	73.63416183	43.29035	103.9779734	1.272454765	0.286268169	4.44497468	8.79E-06	3.42E-05
ENSG00000241127	YAE1	387.943013	226.70307	549.182955	1.272106402	0.125068928	10.1712426	2.66E-24	2.80E-23
ENSG00000273038	AL365203.2	771.29461	452.6253	1089.963925	1.270811528	0.083698578	15.1831914	4.57E-52	8.89E-51
ENSG00000242600	MBL1P	16.86661057	9.8802134	23.85300775	1.269082143	0.555063112	2.2863745	0.022232	0.049609
ENSG00000231925	TAPBP	9105.978772	5353.6803	12858.27727	1.26497131	0.034026269	37.1763156	1.65E-302	1.99E-300
ENSG00000267470	ZNF571-AS1	28.85820441	16.855933	40.86047613	1.262895074	0.427386206	2.95492708	0.003127	0.00845
ENSG00000159216	RUNX1	1042.927276	614.71124	1471.143308	1.260426951	0.076595088	16.4557151	7.63E-61	1.71E-59
ENSG00000185507	IRF7	552.663528	325.61767	779.7093868	1.257737547	0.094109723	13.3645866	9.74E-41	1.55E-39
ENSG00000249348	UGDH-AS1	77.65105495	45.818928	109.483182	1.257113873	0.252045559	4.98764539	6.11E-07	2.67E-06
ENSG00000010818	HIVEP2	1251.548864	738.80434	1764.293392	1.255501487	0.06303336	19.9180478	2.84E-88	9.59E-87
ENSG00000188611	ASAH2	27.14907182	16.070719	38.22742449	1.25417259	0.417593334	3.00333479	0.00267	0.007323
ENSG00000132359	RAP1GAP2	123.8123841	72.908511	174.7162571	1.253520083	0.203950041	6.14621149	7.94E-10	4.36E-09
ENSG00000253210	AC040970.1	25.06636226	14.854065	35.27865988	1.252778875	0.492917413	2.54155938	0.011036	0.026523
ENSG00000251429	AC098679.2	17.39950546	10.255245	24.54376541	1.246461986	0.534543396	2.33182562	0.01971	0.044554
ENSG00000258976	AC013451.2	25.95003915	15.32403	36.57604847	1.242204454	0.434289814	2.8603122	0.004232	0.011123
ENSG00000228742	LINC02577	98.59561337	58.681568	138.5096585	1.239014207	0.234075199	5.29323146	1.20E-07	5.59E-07
ENSG00000158286	RNF207	113.6351795	67.75727	159.5130874	1.236725498	0.226215333	5.46702773	4.58E-08	2.20E-07
ENSG00000125657	TNFSF9	74.70790867	44.347144	105.068673	1.23405301	0.288330047	4.28000142	1.87E-05	7.03E-05
ENSG00000143851	PTPN7	33.06403601	19.588638	46.5394336	1.233566138	0.398880558	3.09257023	0.001984	0.005579
ENSG00000173705	SUSD5	2876.040314	1717.5315	4034.549162	1.232578291	0.053790646	22.9143611	3.34E-116	1.40E-114
ENSG00000111331	OAS3	3103.615854	1853.5497	4353.682004	1.23121857	0.044787881	27.4899938	2.31E-166	1.39E-164
ENSG00000073146	MOV10L1	519.0135071	310.06069	727.9663257	1.229757314	0.101461628	12.1204177	8.23E-34	1.11E-32
ENSG00000158966	CACHD1	1207.834934	722.28645	1693.383418	1.229257039	0.078870482	15.585768	9.10E-55	1.85E-53
ENSG00000147650	LRP12	1207.129403	722.32451	1691.934291	1.227721232	0.066488948	18.4650421	3.95E-76	1.10E-74
ENSG00000019582	CD74	72.81544578	43.463145	102.1677465	1.227227652	0.269155446	4.55954976	5.13E-06	2.04E-05
ENSG00000179277	MEIS3P1	40.80351223	24.325581	57.28144374	1.227178489	0.352258245	3.48374667	0.000494	0.001538
ENSG00000234993	CUBNP2	26.98548905	16.1328	37.8381778	1.222295128	0.432505998	2.82607671	0.004712	0.012258
ENSG00000269834	ZNF528-AS1	82.87947351	49.784546	115.9744006	1.222102318	0.240318074	5.08535334	3.67E-07	1.64E-06
ENSG00000182986	ZNF320	23.79653573	14.145564	33.44750741	1.221954172	0.490320382	2.49215455	0.012697	0.030162
ENSG00000082126	MPP4	177.7534476	106.64171	248.8651881	1.221905831	0.178064893	6.86213779	6.78E-12	4.27E-11
ENSG00000147251	DOCK11	30.28774181	18.174077	42.40140669	1.221175069	0.384950674	3.17228973	0.001512	0.00433
ENSG00000111266	DUSP16	1342.660267	808.88084	1876.43969	1.214392169	0.065481239	18.5456505	8.84E-77	2.50E-75
ENSG00000152894	PTPRK	9609.777982	5790.5822	13428.97373	1.214104442	0.029790663	40.7545294	0	0
ENSG00000139278	GLIPR1	3842.805606	2315.5897	5370.021493	1.213219091	0.040538096	29.9278752	8.54E-197	6.26E-195
ENSG00000105835	NAMPT	10774.73292	6495.688	15053.7778	1.212805348	0.031927195	37.9865927	0	0
ENSG00000145476	CYP4V2	46.06825911	27.779935	64.3565835	1.21041891	0.317850595	3.80813794	0.00014	0.000472
ENSG00000105810	CDK6	2995.721224	1811.9374	4179.505055	1.20767901	0.05879954	20.5389195	9.67E-94	3.46E-92
ENSG00000137177	KIF13A	5090.335496	3076.1217	7104.549278	1.20710426	0.035077928	34.4120739	1.66E-259	1.71E-257
ENSG00000198429	ZNF69	66.13746227	40.078012	92.1969124	1.205962556	0.27347877	4.40971179	1.04E-05	3.99E-05
ENSG00000077238	IL4R	6771.091898	4099.1933	9442.990514	1.203536639	0.035063373	34.3246113	3.37E-258	3.39E-256
ENSG00000164136	IL15	89.39572856	54.203536	124.5879207	1.201981202	0.234332314	5.12938734	2.91E-07	1.31E-06
ENSG00000134294	SLC38A2	28034.03627	16995.448	39072.6248	1.201311724	0.024587555	48.8585279	0	0
ENSG00000254027	AC009902.2	21.45716605	13.007656	29.90667657	1.198296803	0.466076897	2.57102811	0.01014	0.024586
ENSG00000153094	BCL2L11	973.6906664	591.59265	1355.788682	1.197147705	0.075996439	15.7526816	6.58E-56	1.36E-54
ENSG00000062716	VMP1	4896.438983	2974.813	6818.064946	1.195887787	0.036625921	32.6514048	7.65E-234	6.83E-232
ENSG00000124508	BTN2A2	1084.03647	659.52685	1508.546086	1.195810958	0.073190341	16.3383711	5.26E-60	1.17E-58
ENSG00000149591	TAGLN	4300.328854	2611.9468	5988.710861	1.195654026	0.042728427	27.9826359	2.64E-172	1.65E-170
ENSG00000110002	VWA5A	915.0571091	556.74609	1273.368125	1.19383595	0.075003455	15.9170795	4.82E-57	1.01E-55
ENSG00000185885	IFITM1	184.3824603	112.3831	256.38182	1.193517717	0.170246249	7.01053752	2.37E-12	1.53E-11
ENSG00000168394	TAP1	2296.290943	1398.5488	3194.033118	1.19279235	0.050647639	23.5507986	1.23E-122	5.37E-121
ENSG00000251442	LINC01094	123.7875101	75.152696	172.4223242	1.191413059	0.212122742	5.61662106	1.95E-08	9.69E-08
ENSG00000170558	CDH2	6755.965491	4117.8445	9394.08648	1.190222415	0.032093398	37.0862076	4.69E-301	5.53E-299
ENSG00000279069	AC015813.5	21.00195211	12.832086	29.17181856	1.189332541	0.477372283	2.49141515	0.012724	0.030216
ENSG00000100979	PLTP	3841.203157	2341.914	5340.49231	1.189327504	0.041622456	28.5741792	1.41E-179	9.41E-178
ENSG00000155324	GRAMD2B	185.1064474	112.92485	257.2880461	1.189073349	0.174769376	6.80367107	1.02E-11	6.36E-11
ENSG00000123240	OPTN	6580.31973	4010.6209	9150.018573	1.18894319	0.03846964	30.9060126	9.92E-210	7.73E-208
ENSG00000162511	LAPTM5	5441.716653	3323.3457	7560.087565	1.186435507	0.03449118	34.3982288	2.68E-259	2.72E-257
ENSG00000112715	VEGFA	496.1129039	303.00193	689.2238782	1.184909009	0.100946312	11.7380119	8.14E-32	1.05E-30
ENSG00000100228	RAB36	108.170268	66.262302	150.0782341	1.184480476	0.242654977	4.881336	1.05E-06	4.50E-06
ENSG00000259429	UBE2Q2P2	39.30772465	23.946696	54.66875332	1.18361852	0.355159888	3.33263569	0.00086	0.002571
ENSG00000271533	Z83843.1	27.73046139	16.935827	38.52509542	1.183448228	0.409337665	2.89112957	0.003839	0.01018
ENSG00000115414	FN1	649235.3948	396967.68	901503.1139	1.183325637	0.023830701	49.6555113	0	0
ENSG00000214530	STARD10	612.6715374	375.13053	850.2125455	1.182576021	0.098618328	11.9914426	3.94E-33	5.23E-32
ENSG00000138642	HERC6	624.593857	381.72321	867.4645037	1.182495612	0.101120482	11.6939277	1.37E-31	1.77E-30
ENSG00000155893	PXYLP1	47.3373354	28.881545	65.79312554	1.181882635	0.331598471	3.56419808	0.000365	0.001157
ENSG00000178078	STAP2	365.8093325	223.46599	508.1526724	1.181408518	0.117030494	10.0948776	5.82E-24	6.07E-23
ENSG00000121753	ADGRB2	79.31089801	48.547446	110.0743505	1.180539468	0.241439002	4.8895972	1.01E-06	4.32E-06
ENSG00000185155	MIXL1	31.21615104	19.197013	43.23528948	1.176909758	0.391755812	3.00419221	0.002663	0.007309
ENSG00000102760	RGCC	796.3845636	489.12002	1103.649108	1.176394673	0.081240008	14.4804845	1.61E-47	2.92E-46
ENSG00000187720	THSD4	3251.481723	1997.037	4505.926467	1.174873564	0.044352179	26.4896471	1.28E-154	7.26E-153

ENSG00000169902	TPST1	1422.343772	873.35555	1971.331996	1.174010207	0.060659668	19.3540492	1.88E-83	5.96E-82
ENSG00000102359	SRPX2	6340.574667	3900.3563	8780.792988	1.170209438	0.034599978	33.8211037	9.66E-251	9.39E-249
ENSG00000246695	RASSF8-AS1	104.7387003	64.3627	145.1147005	1.169832356	0.215499122	5.42847853	5.68E-08	2.72E-07
ENSG00000196843	ARID5A	1131.208083	696.20277	1566.213401	1.168480878	0.08147748	14.3411515	1.21E-46	2.15E-45
ENSG00000131669	NINJ1	1361.460219	839.31995	1883.600483	1.167780436	0.063794311	18.3054008	7.49E-75	2.04E-73
ENSG00000084112	SSH1	5951.637866	3672.6085	8230.667228	1.165360179	0.040811258	28.5548701	2.44E-179	1.63E-177
ENSG00000163040	CCDC74A	144.213747	88.718764	199.7087297	1.164953751	0.206623687	5.63804552	1.72E-08	8.59E-08
ENSG00000062282	DGAT2	39.02940954	24.124228	53.93459135	1.163480107	0.359213536	3.2389651	0.0012	0.003498
ENSG00000214944	ARHGEF28	6701.389121	4139.7781	9263.000152	1.162019645	0.030712058	37.8359425	0	0
ENSG00000197646	PDCD1LG2	580.1787109	358.21018	802.1472403	1.1618265	0.09123657	12.7342193	3.82E-37	5.54E-36
ENSG00000102755	FLT1	20476.87264	12656.144	28297.60144	1.161179859	0.025329628	45.8427516	0	0
ENSG00000125629	INSIG2	1253.04564	773.97259	1732.118688	1.160615668	0.077690198	14.9390232	1.84E-50	3.48E-49
ENSG00000205683	DPF3	257.4816786	158.93398	356.029379	1.160233707	0.151072654	7.67997169	1.59E-14	1.14E-13
ENSG00000272168	CASC15	383.0445371	237.36671	528.7223594	1.160039764	0.12005756	9.66236333	4.36E-22	4.25E-21
ENSG00000247081	BAALC-AS1	62.15449679	38.551853	85.75714089	1.159789645	0.285037769	4.06889813	4.72E-05	0.000169
ENSG00000167100	SAMD14	1103.22776	682.92418	1523.531336	1.158468518	0.067447412	17.1758781	4.03E-66	9.83E-65
ENSG00000178038	ALS2CL	291.5145836	180.7322	402.296966	1.157084061	0.133258971	8.6829731	3.86E-18	3.23E-17
ENSG00000109320	NFKB1	4062.844635	2515.8488	5609.840511	1.157061952	0.039503999	29.2897427	1.40E-188	9.69E-187
ENSG00000153714	LURAP1L	195.2573209	120.9427	269.5719461	1.156414817	0.178711941	6.47083128	9.75E-11	5.70E-10
ENSG00000136205	TNS3	4659.15089	2886.6585	6431.643265	1.154715099	0.039149323	29.4951484	3.32E-191	2.33E-189
ENSG00000144366	GULP1	1037.324705	642.90018	1431.749234	1.153499391	0.072189605	15.9787464	1.80E-57	3.78E-56
ENSG00000156265	MAP3K7CL	148.5465102	92.002407	205.0906129	1.152814024	0.398044683	2.89619249	0.003777	0.010032
ENSG00000223361	FTH1P10	43.03467096	26.622657	59.44668456	1.151216415	0.350556503	3.28396822	0.001024	0.003022
ENSG00000060656	PTPRU	192.5022405	119.8296	265.1748826	1.150307827	0.162340137	7.08578819	1.38E-12	9.04E-12
ENSG00000239213	NCK1-DT	53.69359144	33.264237	74.12294562	1.149412682	0.312291798	3.68057275	0.000233	0.000761
ENSG00000166532	RIMKLB	1661.204944	1032.6171	2289.792759	1.148876508	0.070558716	16.2825596	1.31E-59	2.89E-58
ENSG00000169855	ROBO1	1883.782071	1174.2643	2593.299823	1.143941526	0.060488777	18.9116325	9.15E-80	2.72E-78
ENSG00000108960	MMD	578.2574047	361.10315	795.4116549	1.14069749	0.091774512	12.4293495	1.81E-35	2.53E-34
ENSG00000184557	SOCS3	1134.844647	708.96971	1560.719581	1.139442215	0.067779345	16.8110537	2.03E-63	4.73E-62
ENSG00000170006	TMEM154	1161.693687	725.26598	1598.121396	1.138197188	0.065738912	17.3139037	3.69E-67	9.16E-66
ENSG00000159200	RCAN1	1129.424727	706.5181	1552.331356	1.137473745	0.070170766	16.2100802	4.28E-59	9.34E-58
ENSG00000206538	VGLL3	604.4586901	377.74026	831.1771241	1.13696108	0.092675398	12.2682082	1.34E-34	1.83E-33
ENSG00000213066	FGFR1OP	226.6371659	141.41222	311.862109	1.136642147	0.161645121	7.03171329	2.04E-12	1.32E-11
ENSG00000033867	SLC4A7	3144.132277	1966.7692	4321.495336	1.136602441	0.043645737	26.0415455	1.68E-149	9.10E-148
ENSG00000163491	NEK10	79.11840227	49.231169	109.0056352	1.136227292	0.268207068	4.23638086	2.27E-05	8.46E-05
ENSG00000227076	AL158166.1	44.27729262	27.762934	60.79165085	1.135300455	0.335412548	3.38478826	0.000712	0.002159
ENSG00000268205	AC005261.1	267.0825643	167.19628	366.9688477	1.135105117	0.148154764	7.66161741	1.84E-14	1.32E-13
ENSG00000147883	CDKN2B	430.3184888	269.18587	591.4511071	1.134280724	0.116959676	9.6980495	3.07E-22	3.01E-21
ENSG00000130303	BST2	2472.521756	1547.9171	3397.126458	1.133781754	0.06035046	18.78663	9.72E-79	2.83E-77
ENSG00000176749	CDK5R1	257.2061518	161.15235	353.259958	1.133525436	0.139370604	8.13317448	4.18E-16	3.22E-15
ENSG00000184384	MAML2	1525.435818	956.29007	2094.581568	1.131969324	0.059810918	18.9257977	6.99E-80	2.09E-78
ENSG00000170921	TANC2	2386.918744	1496.7936	3277.043884	1.131790415	0.05446713	20.7793288	6.66E-96	2.43E-94
ENSG00000233251	AC007743.1	1316.647139	825.55486	1807.739415	1.131328161	0.061873148	18.2846389	1.10E-74	2.98E-73
ENSG00000141441	GAREM1	61.54189645	38.441916	84.64187728	1.13132452	0.312693716	3.6179957	0.000297	0.000955
ENSG00000143494	VASH2	51.55450012	32.177665	70.93133508	1.129964184	0.341002312	3.31365549	0.000921	0.00274
ENSG00000182489	XKRX	76.2757662	47.937395	104.6141371	1.129813156	0.258651472	4.36809095	1.25E-05	4.79E-05
ENSG00000255031	AP002807.1	82.48107069	51.772525	113.1896166	1.128530254	0.236290343	4.77603206	1.79E-06	7.47E-06
ENSG00000213190	MLLT11	4414.70388	2774.6928	6054.714951	1.125209518	0.038269873	29.4019661	5.18E-190	3.62E-188
ENSG00000169621	APLF	110.3690572	69.412187	151.3259275	1.124056277	0.211504464	5.31457471	1.07E-07	4.99E-07
ENSG00000119630	PGF	9027.365442	5676.8126	12377.91824	1.123914669	0.031635745	35.5267333	1.90E-276	2.08E-274
ENSG00000135547	HEY2	115.7077531	72.813242	158.6022637	1.123793001	0.196781415	5.71086962	1.12E-08	5.70E-08
ENSG00000084710	EFR3B	85.04130568	53.582229	116.5003821	1.121953144	0.236245469	4.7490991	2.04E-06	8.50E-06
ENSG00000152229	PSTPIP2	663.2409363	417.41031	909.0715597	1.121549763	0.091122966	12.3080911	8.19E-35	1.12E-33
ENSG00000221963	APOL6	751.7289683	475.16517	1028.292767	1.115692517	0.087639722	12.7304434	4.01E-37	5.81E-36
ENSG00000143878	RHOB	40107.8923	25333.818	54881.96696	1.115251325	0.076837608	14.5143941	9.82E-48	1.79E-46
ENSG00000124212	PTGIS	311.5333951	196.87493	426.1918572	1.115021127	0.129007902	8.6430452	5.47E-18	4.56E-17
ENSG00000171246	NPTX1	84.47554187	53.248761	115.702323	1.113783692	0.278483285	3.99946334	6.35E-05	0.000224
ENSG00000164430	CGAS	289.0587876	183.00143	395.116148	1.113748856	0.135630778	8.21162329	2.18E-16	1.70E-15
ENSG00000137628	DDX60	1256.770364	796.02451	1717.516213	1.110947992	0.0692516	16.0421997	6.48E-58	1.39E-56
ENSG00000175600	SUGCT	46.84827943	29.744021	63.95253822	1.110521187	0.342817902	3.23939089	0.001198	0.003494
ENSG00000049192	ADAMTS6	950.6398762	601.85348	1299.426271	1.110238098	0.074417038	14.9191386	2.47E-50	4.68E-49
ENSG00000102287	GABRE	125.2190844	79.349551	171.0886177	1.109026107	0.20239242	5.4795832	4.26E-08	2.06E-07
ENSG00000156869	FRRS1	89.36410666	56.682333	122.0458804	1.107481749	0.236282472	4.68710921	2.77E-06	1.14E-05
ENSG00000158186	MRAS	335.0975656	212.45287	457.7422591	1.106377845	0.117699617	9.40001225	5.46E-21	5.11E-20
ENSG00000196511	TPK1	514.4759546	326.40336	702.5485488	1.105142986	0.096885591	11.4066805	3.87E-30	4.82E-29
ENSG00000136052	SLC41A2	456.1442893	290.03024	622.2583421	1.102768777	0.107131007	10.2936471	7.53E-25	8.07E-24
ENSG00000214548	MEG3	3042.59858	1934.577	4150.620207	1.101344959	0.175298748	6.28267441	3.33E-10	1.88E-09
ENSG00000267534	S1PR2	74.06337673	47.143964	100.9827893	1.101204551	0.29267648	3.76253176	0.000168	0.000561
ENSG00000005238	FAM214B	2166.194548	1378.7879	2953.601182	1.098390784	0.051422337	21.3601879	3.14E-101	1.19E-99
ENSG00000196639	HRH1	1838.378536	1170.6774	2506.079719	1.097176042	0.052886135	20.7460053	1.33E-95	4.84E-94
ENSG00000109846	CRYAB	53.975597	34.544599	73.40659462	1.095314789	0.302637231	3.61923344	0.000295	0.000951
ENSG00000154642	C21orf91	359.5608148	230.04175	489.0798829	1.093807383	0.127873921	8.5537956	1.19E-17	9.78E-17
ENSG00000260604	AL590004.3	558.1957851	356.1129	760.2786676	1.09319688	0.099734776	10.9610401	5.88E-28	6.90E-27
ENSG00000167552	TUBA1A	29964.76604	19127.673	40801.85926	1.093037263	0.022235245	49.1578681	0	0
ENSG00000163291	PAQR3	1100.808972	703.58172	1498.036221	1.092412577	0.077475613	14.1000831	3.79E-45	6.56E-44
ENSG00000175426	PCSK1	296.5183379	189.25027	403.7864088	1.091181382	0.14594731	7.47654332	7.63E-14	5.32E-13
ENSG00000168528	SERINC2	673.91898	430.84955	916.9884082	1.091106663	0.099123643	11.0075319	3.52E-28	4.15E-27
ENSG00000063127	SLC6A16	38.91730144	24.8504	52.98420332	1.090724283	0.391548544	2.78566809	0.005342	0.013742
ENSG00000110031	LPXN	520.0387698	332.05742	708.0201213	1.089305806	0.098028666	11.1121149	1.10E-28	1.31E-27
ENSG00000164930	FZD6	4394.481722	2809.8865	5979.076897	1.088756223	0.038321397	28.4111829	1.47E-177	9.63E-176
ENSG00000232187	FTH1P7	25.48417542	16.328074	34.64027641	1.088530342	0.41955855	2.59446588	0.009474	0.023136
ENSG00000154016	GRAP	3017.846172	1930.9116	4104.780729	1.088405324	0.042314358	25.7218917	6.65E-146	3.49E-144
ENSG00000187398	LUZP2	36.71900371	23.597112	49.84089585	1.086873766	0.354273886	3.06789128	0.002156	0.006029
ENSG00000158292	GPR153	292.5916368	187.65225	397.5310199	1.081415748	0.129908104	8.32446718	8.47E-17	6.70E-16
ENSG00000265982	AC103810.3	32.03681863	20.467708	43.60592972	1.08096004	0.433522004	2.49343754	0.012651	0.030061

ENSG00000148700	ADD3	4206.523504	2700.1177	5712.929273	1.080682254	0.039720143	27.2074107	5.31E-163	3.13E-161
ENSG00000156273	BACH1	1546.212119	993.32857	2099.095667	1.078647194	0.058846445	18.3298617	4.78E-75	1.31E-73
ENSG00000235770	LINC00607	4217.292087	2710.5615	5724.022633	1.078636753	0.038607463	27.9385553	9.08E-172	5.66E-170
ENSG00000259583	AC015712.2	84.74620417	54.244358	115.2480503	1.078511796	0.257546413	4.18764053	2.82E-05	0.000104
ENSG00000108582	CPD	14924.77438	9597.2044	20252.34438	1.077835559	0.032914868	32.7461605	3.44E-235	3.13E-233
ENSG00000187193	MT1X	35.11631753	22.593067	47.63956811	1.076109643	0.362557602	2.96810669	0.002996	0.008133
ENSG00000234814	SVIL2P	55.02115262	35.385002	74.65730327	1.074909014	0.299705492	3.58655095	0.000335	0.001068
ENSG00000177989	ODF3B	29.7841166	19.252608	40.3156249	1.073918273	0.424090347	2.53228653	0.011332	0.027169
ENSG00000203326	ZNF525	78.34609215	50.399191	106.2929929	1.073498001	0.254574668	4.21682962	2.48E-05	9.20E-05
ENSG00000166452	AKIP1	1061.432874	684.49634	1438.369405	1.071775634	0.067701871	15.8308126	1.91E-56	3.97E-55
ENSG00000161960	EIF4A1	83.08385654	53.794168	112.3735448	1.068048561	0.259433478	4.11684941	3.84E-05	0.000139
ENSG00000134668	SPOCD1	1959.814128	1265.9532	2653.675011	1.067363801	0.050679338	21.0611237	1.81E-98	6.72E-97
ENSG00000147416	ATP6V1B2	5977.815317	3862.554	8093.076676	1.06653883	0.034163455	31.218705	5.94E-214	4.75E-212
ENSG00000104863	LIN7B	29.30032509	18.896061	39.70458953	1.063093851	0.429032728	2.47788521	0.013216	0.031263
ENSG00000075420	FNDC3B	23656.96574	15320.14	31993.79173	1.062591774	0.026671067	39.8406168	0	0
ENSG00000104728	ARHGEF10	36.1855156	23.302238	49.06879275	1.062061851	0.391417617	2.71337263	0.00666	0.016809
ENSG00000162618	ADGRL4	19244.91051	12469.775	26020.04604	1.061662548	0.031273175	33.9480257	1.30E-252	1.29E-250
ENSG00000279191	AC068491.4	31.26810169	20.195649	42.3405541	1.058569245	0.393541874	2.68985161	0.007148	0.017887
ENSG00000154874	CCDC144B	134.2006413	86.936063	181.4652197	1.058120491	0.186569576	5.67145252	1.42E-08	7.12E-08
ENSG00000110330	BIRC2	3572.936329	2319.9262	4825.946473	1.057957599	0.044498087	23.7753502	6.01E-125	2.66E-123
ENSG00000196263	ZNF471	72.46976203	46.964135	97.97538896	1.057682886	0.278093097	3.80334102	0.000143	0.000481
ENSG00000270055	AC127502.2	181.8862117	118.1838	245.5886232	1.056459138	0.169130739	6.24640525	4.20E-10	2.35E-09
ENSG00000091656	ZFHX4	188.7140126	122.74125	254.6867764	1.052639471	0.1604988	6.5585504	5.43E-11	3.23E-10
ENSG00000023902	PLEKHO1	4151.675418	2700.9844	5602.366417	1.052497556	0.041699411	25.2401063	1.45E-140	7.33E-139
ENSG00000128567	PODXL	36829.12517	23989.635	49668.6151	1.050206174	0.028952913	36.2729024	4.33E-288	4.88E-286
ENSG00000125845	BMP2	2211.729923	1440.5507	2982.909116	1.050133741	0.05848079	17.9569006	4.24E-72	1.12E-70
ENSG00000234465	PINLYP	76.45716244	49.930375	102.98395	1.04945302	0.260626508	4.02665496	5.66E-05	0.000201
ENSG00000170745	KCNS3	23.54858938	15.405073	31.69210556	1.047529621	0.451826141	2.31843518	0.020426	0.04603
ENSG00000158683	PKD1L1	436.0068277	284.75229	587.261367	1.047350098	0.106181488	9.86377307	5.98E-23	6.00E-22
ENSG00000134107	BHLHE40	2481.000976	1619.9766	3342.025307	1.044507458	0.046361991	22.5293917	2.14E-112	8.66E-111
ENSG00000113742	CPEB4	1015.623541	663.44062	1367.806462	1.043799003	0.071635795	14.5709139	4.30E-48	7.85E-47
ENSG00000156675	RAB11FIP1	94.62904181	61.954839	127.3032448	1.043487878	0.22855897	4.56550831	4.98E-06	1.98E-05
ENSG00000215252	GOLGA8B	188.7651735	123.47497	254.0553798	1.043189401	0.171927566	6.06760989	1.30E-09	7.03E-09
ENSG00000114554	PLXNA1	3562.282971	2328.8567	4795.709287	1.041395853	0.040453061	25.7433142	3.83E-146	2.02E-144
ENSG00000118985	ELL2	2015.628428	1319.2392	2712.017648	1.041341471	0.0562831	18.50185	2.00E-76	5.61E-75
ENSG00000161011	SQSTM1	24031.87218	15716.481	32347.26299	1.04118941	0.025281287	41.1841926	0	0
ENSG00000186567	CEACAM19	148.5462478	97.478848	199.6136473	1.040873797	0.203289384	5.12015815	3.05E-07	1.37E-06
ENSG00000068079	IFI35	440.6621902	288.21309	593.1112892	1.039444816	0.111714895	9.30444252	1.35E-20	1.24E-19
ENSG00000099282	TSPAN15	2124.524204	1390.9359	2858.112468	1.039331548	0.052841492	19.6688532	3.99E-86	1.31E-84
ENSG00000167772	ANGPTL4	1333.282495	872.63606	1793.928935	1.038671537	0.081601983	12.7285085	4.11E-37	5.95E-36
ENSG00000198742	SMURF1	2244.523772	1469.3545	3019.693008	1.037492644	0.055082958	18.8350931	3.89E-79	1.14E-77
ENSG00000278864	AC055811.4	67.90312243	44.343363	91.46288205	1.037218632	0.275847566	3.76011523	0.00017	0.000566
ENSG00000101194	SLC17A9	1411.455513	925.61407	1897.296958	1.035103786	0.06710661	15.4247664	1.12E-53	2.22E-52
ENSG00000248866	USP46-AS1	56.51191017	36.970203	76.05361719	1.034089332	0.312997053	3.30383089	0.000954	0.002832
ENSG00000171016	PYGO1	505.489324	331.93299	679.0456563	1.033646891	0.101160353	10.2179052	1.65E-24	1.75E-23
ENSG00000257354	AC048341.1	37.00373648	24.2805	49.72697304	1.031607483	0.391271121	2.63655411	0.008375	0.020667
ENSG00000136274	NACAD	238.8943799	157.15743	320.6313331	1.028220765	0.140938351	7.29553564	2.97E-13	2.02E-12
ENSG00000140563	MCTP2	31.71374668	20.806848	42.62064513	1.027934694	0.381670738	2.69324995	0.007076	0.017718
ENSG00000143178	TBX19	37.31437091	24.579897	50.04884523	1.025136663	0.347211525	2.95248455	0.003152	0.008508
ENSG00000113916	BCL6	677.0510456	446.33242	907.7696758	1.023923948	0.083893644	12.2050242	2.92E-34	3.95E-33
ENSG00000196562	SULF2	5180.478861	3416.3546	6944.603124	1.023755527	0.042509179	24.083164	3.75E-128	1.72E-126
ENSG00000176170	SPHK1	3232.13424	2130.5146	4333.753859	1.023443271	0.05003892	20.4529447	5.66E-93	2.01E-91
ENSG00000137285	TUBB2B	66.53760077	43.810503	89.26469831	1.023198048	0.265870794	3.84847855	0.000119	0.000405
ENSG00000188158	NHS	65.65929287	43.125153	88.19343277	1.022789173	0.292307207	3.4990214	0.000467	0.001458
ENSG00000197208	SLC22A4	419.9790108	277.11693	562.8410909	1.022280342	0.106657659	9.58468757	9.27E-22	8.94E-21
ENSG00000213694	S1PR3	2375.918706	1568.5977	3183.239667	1.021006912	0.053886203	18.9474643	4.63E-80	1.39E-78
ENSG00000170775	GPR37	842.8532281	556.80422	1128.90224	1.020721064	0.078702708	12.9693259	1.83E-38	2.76E-37
ENSG00000102879	CORO1A	166.895218	110.08859	223.7018502	1.020418429	0.171730727	5.94196767	2.82E-09	1.49E-08
ENSG00000163013	FBXO41	95.83623984	63.365212	128.3072675	1.0198492	0.215733638	4.72735364	2.27E-06	9.41E-06
ENSG00000196923	PDLIM7	6144.221449	4066.5028	8221.940116	1.016268814	0.033741695	30.11908	2.73E-199	2.03E-197
ENSG00000272221	AL645933.2	61.75321366	40.806481	82.699946	1.013810074	0.283116154	3.58089801	0.000342	0.001089
ENSG00000130066	SAT1	14745.42923	9785.6603	19705.19816	1.009651127	0.032328042	31.2314346	3.99E-214	3.20E-212
ENSG00000198805	PNP	10366.75447	6880.981	13852.52797	1.008899275	0.029408277	34.3066435	6.25E-258	6.24E-256
ENSG00000180530	NRIP1	2861.789302	1901.484	3822.094612	1.007687779	0.043087684	23.3869099	5.81E-121	2.51E-119
ENSG00000196503	ARL9	39.20531549	26.030014	52.38061696	1.007553179	0.347669096	2.89802341	0.003755	0.009976
ENSG00000132793	LPIN3	52.06846355	34.515299	69.62162805	1.007389698	0.309864961	3.25106039	0.00115	0.003365
ENSG00000205562	AL049775.1	141.1975943	93.862743	188.5324455	1.0066923	0.194639494	5.1720865	2.31E-07	1.05E-06
ENSG00000198286	CARD11	3133.400215	2082.9347	4183.865754	1.005276329	0.0445935	22.5431133	1.57E-112	6.37E-111
ENSG00000176853	FAM91A1	3579.103289	2381.5283	4776.678235	1.004294704	0.041016882	24.484911	2.14E-132	1.02E-130
ENSG00000008300	CELSR3	43.28430591	28.763868	57.80474339	1.001543813	0.327024494	3.06259571	0.002194	0.006129
ENSG00000111711	GOLT1B	1942.263372	1295.345	2589.181783	0.99961957	0.055933287	17.8716399	1.96E-71	5.15E-70
ENSG00000169826	CSGALNACT2	1966.993247	1312.6154	2621.371071	0.998021232	0.050280684	19.8489989	1.12E-87	3.76E-86
ENSG00000182957	SPATA13	666.6983163	445.62494	887.771691	0.997066782	0.091006116	10.9560415	6.22E-28	7.28E-27
ENSG00000253958	CLDN23	25.52172322	17.055059	33.98838697	0.99702882	0.425209781	2.34479277	0.019038	0.043194
ENSG00000006118	TMEM132A	9396.053929	6272.2432	12519.86467	0.996799718	0.031631777	31.5126061	5.84E-218	4.79E-216
ENSG00000113248	PCDHB15	42.92800816	28.684787	57.17122934	0.996337155	0.324146283	3.07372691	0.002114	0.005922
ENSG00000177337	DLGAP1-AS1	148.7374101	99.499607	197.9752128	0.995818004	0.176911022	5.62892008	1.81E-08	9.03E-08
ENSG00000143507	DUSP10	60.73540032	40.473084	80.99771665	0.99504971	0.274498275	3.62497619	0.000289	0.000931
ENSG00000130363	RSPH3	249.486466	166.89022	332.0827102	0.99378343	0.137780063	7.21282463	5.48E-13	3.66E-12
ENSG00000144749	LRIG1	5509.471292	3683.9799	7334.962719	0.993589936	0.038720843	25.6603384	3.24E-145	1.70E-143
ENSG00000127666	TICAM1	1031.796376	689.42317	1374.169585	0.993378145	0.072291698	13.7412478	5.75E-43	9.53E-42
ENSG00000166503	HDGFL3	7521.279552	5035.2316	10007.32747	0.991309321	0.035021114	28.3060475	2.91E-176	1.89E-174
ENSG00000116771	AGMAT	68.67816378	45.911157	91.44517052	0.989583076	0.289562862	3.4175069	0.000632	0.001938
ENSG00000172349	IL16	45.38584126	30.317366	60.45431621	0.988588711	0.321730654	3.07272154	0.002121	0.005939
ENSG00000130755	GMFG	428.8512757	287.14794	570.5546141	0.98721679	0.108037558	9.13771851	6.38E-20	5.73E-19

ENSG00000164292	RHOBTB3	4225.461769	2837.1015	5613.82207	0.985899299	0.04171207	23.6358276	1.65E-123	7.23E-122
ENSG00000146555	SDK1	60.07841716	40.174429	79.98240493	0.984245159	0.295134239	3.33490672	0.000853	0.002552
ENSG00000254838	GVINP1	522.4644837	351.30932	693.6196463	0.983635483	0.097989584	10.0381637	1.04E-23	1.07E-22
ENSG00000138411	HECW2	5416.778727	3641.7629	7191.794604	0.982180905	0.038925222	25.2325063	1.76E-140	8.86E-139
ENSG00000153246	PLA2R1	2905.736027	1953.3242	3858.14782	0.981845571	0.045938499	21.3730442	2.38E-101	9.08E-100
ENSG00000166224	SGPL1	1729.522865	1163.3982	2295.647486	0.980603081	0.057298267	17.1140094	1.17E-65	2.82E-64
ENSG00000188549	CCDC9B	1627.486049	1094.6608	2160.311332	0.979601718	0.064429233	15.2043052	3.31E-52	6.46E-51
ENSG00000196878	LAMB3	1453.661897	977.99968	1929.324111	0.979542949	0.063925218	15.3232634	5.35E-53	1.06E-51
ENSG00000139263	LRIG3	3343.306635	2251.1893	4435.423963	0.97883257	0.044115494	22.1879544	4.49E-109	1.79E-107
ENSG00000160323	ADAMTS13	46.59247697	31.511348	61.67360564	0.978184032	0.351180785	2.78541445	0.005346	0.013751
ENSG00000272899	ATP6V1FNB	31.02693218	20.946933	41.10693104	0.976991627	0.402801977	2.42548866	0.015288	0.035604
ENSG00000170525	PFKFB3	3965.798633	2672.8416	5258.755696	0.975212907	0.042443236	22.9768747	7.94E-117	3.34E-115
ENSG00000176658	MYO1D	7847.58548	5294.9282	10400.24274	0.973853562	0.0295849	32.9172504	1.25E-237	1.18E-235
ENSG00000286001	AC021087.5	27.03140058	18.17367	35.88913085	0.97078353	0.420501665	2.30863183	0.020964	0.047111
ENSG00000164687	FABP5	11395.9624	7704.1897	15087.73508	0.96889735	0.036488244	26.5536852	2.33E-155	1.33E-153
ENSG00000087903	RFX2	483.3461636	326.31413	640.3781933	0.968698665	0.116235581	8.33392543	7.82E-17	6.20E-16
ENSG00000108556	CHRNE	72.5213773	49.182944	95.8598111	0.968691769	0.262718809	3.68718088	0.000227	0.000742
ENSG00000131435	PDLIM4	3031.050453	2051.3438	4010.757066	0.967809083	0.046240131	20.9300681	2.85E-97	1.05E-95
ENSG00000167703	SLC43A2	5400.385057	3657.4069	7143.363249	0.965783234	0.035463355	27.2332732	2.62E-163	1.55E-161
ENSG00000229644	NAMPTP1	60.63518201	41.059649	80.21071552	0.964467283	0.279210584	3.45426476	0.000552	0.001706
ENSG00000108947	EFNB3	164.9400408	112.07123	217.8088543	0.961103211	0.180197379	5.33361372	9.63E-08	4.52E-07
ENSG00000234975	FTH1P2	63.35958599	42.928395	83.79077728	0.960648501	0.273305055	3.51493133	0.00044	0.001377
ENSG00000067066	SP100	6680.735218	4536.3102	8825.160276	0.960340694	0.034968851	27.4627469	4.89E-166	2.92E-164
ENSG00000280287	AC131212.3	88.53839043	60.086835	116.9899462	0.959810914	0.257833376	3.72260151	0.000197	0.000651
ENSG00000140937	CDH11	2937.014446	1995.2742	3878.754685	0.958629925	0.045508355	21.0649216	1.67E-98	6.21E-97
ENSG00000054967	RELT	732.026814	497.20224	966.851385	0.958546077	0.091887457	10.4317402	1.78E-25	1.94E-24
ENSG00000135931	ARMC9	1447.800344	986.60198	1908.998712	0.954337443	0.067851875	14.0650121	6.23E-45	1.07E-43
ENSG00000154640	BTG3	1119.544472	762.43156	1476.657381	0.953968396	0.067718533	14.0872573	4.55E-45	7.84E-44
ENSG00000219507	FTH1P8	45.71896571	31.061352	60.37657975	0.953929909	0.312566458	3.05192667	0.002274	0.006331
ENSG00000091428	RAPGEF4	475.6773666	324.15425	627.2004879	0.951820205	0.107875479	8.82332307	1.11E-18	9.46E-18
ENSG00000233621	LINC01137	45.10808985	30.607167	59.60901274	0.950910273	0.350457566	2.71333926	0.006661	0.016809
ENSG00000137877	SPTBN5	1022.594843	697.50698	1347.6827	0.950285176	0.083354208	11.4005664	4.15E-30	5.17E-29
ENSG00000215105	TTC3P1	94.31829624	64.351443	124.2851492	0.949677962	0.239729398	3.96145808	7.45E-05	0.00026
ENSG00000135828	RNASEL	128.8823962	88.048489	169.7163033	0.949008033	0.207737181	4.56831092	4.92E-06	1.96E-05
ENSG00000119866	BCL11A	29.21185066	19.961444	38.462257	0.948596104	0.399719751	2.37315294	0.017637	0.040334
ENSG00000232807	AL137186.2	133.4470166	91.385908	175.5081248	0.946702194	0.207073841	4.57180969	4.84E-06	1.93E-05
ENSG00000154319	FAM167A	44.7969294	30.495234	59.09862528	0.946401345	0.350352911	2.70128009	0.006907	0.017345
ENSG00000134363	FST	1020.985185	697.71779	1344.252578	0.944440932	0.075068266	12.5810942	2.68E-36	3.81E-35
ENSG00000099860	GADD45B	1194.723054	817.17736	1572.268749	0.944094682	0.068222695	13.8384255	1.49E-43	2.50E-42
ENSG00000234883	MIR155HG	48.5059017	33.207157	63.80464595	0.943944936	0.30755242	3.06921642	0.002146	0.006003
ENSG00000180801	ARSJ	3796.285675	2597.8162	4994.75511	0.943395884	0.038918605	24.2402286	8.38E-130	3.87E-128
ENSG00000105426	PTPRS	452.8000012	310.06944	595.5305656	0.943116054	0.104462073	9.02831077	1.74E-19	1.54E-18
ENSG00000197978	GOLGA6L9	119.571617	81.890835	157.2523986	0.942854108	0.193431529	4.87435586	1.09E-06	4.65E-06
ENSG00000239521	CASTOR3	421.8911247	288.98019	554.8020571	0.942679989	0.104443346	9.02575438	1.78E-19	1.57E-18
ENSG00000160058	BSDC1	3642.326107	2493.1894	4791.462771	0.942528874	0.040408725	23.3248854	2.48E-120	1.07E-118
ENSG00000152076	CCDC74B	27.82579146	19.035739	36.6158438	0.942404001	0.410999793	2.29295493	0.021851	0.048835
ENSG00000197951	ZNF71	737.0294893	504.43398	969.6250029	0.941947243	0.082280706	11.4479722	2.41E-30	3.02E-29
ENSG00000140511	HAPLN3	3535.329735	2422.081	4648.578469	0.939541792	0.042420371	22.1483636	1.08E-108	4.31E-107
ENSG00000196345	ZKSCAN7	36.98190854	25.466264	48.49755271	0.939050407	0.37332678	2.51535774	0.011891	0.028388
ENSG00000152778	IFIT5	547.6893074	375.74602	719.6325926	0.937571628	0.103257532	9.07993451	1.09E-19	9.68E-19
ENSG00000219200	RNASEK	84.99611226	58.257426	111.7347987	0.9375623	0.23319594	4.02049151	5.81E-05	0.000206
ENSG00000243753	HLA-L	29.32711771	20.02197	38.63226525	0.936981795	0.407785994	2.29772922	0.021577	0.048312
ENSG00000104447	TRPS1	291.4461612	200.39029	382.5020318	0.936569863	0.132086115	7.09060041	1.34E-12	8.74E-12
ENSG00000197714	ZNF460	127.6595206	87.825948	167.4930935	0.936285728	0.201410597	4.64864183	3.34E-06	1.36E-05
ENSG00000103021	CCDC113	85.69601726	59.046563	112.3454713	0.936047149	0.251821149	3.71711094	0.000202	0.000664
ENSG00000162695	SLC30A7	2652.112158	1823.385	3480.83934	0.934556886	0.058804311	15.8926593	7.12E-57	1.49E-55
ENSG00000117479	SLC19A2	365.0089841	251.10243	478.9155353	0.933920332	0.116201526	8.03707459	9.20E-16	6.98E-15
ENSG00000279227	AC009303.4	30.17308164	20.725469	39.62069385	0.933707567	0.400569662	2.33094929	0.019756	0.044642
ENSG00000100344	PNPLA3	274.6016171	188.80179	360.4014429	0.933612047	0.132893073	7.02528752	2.14E-12	1.38E-11
ENSG00000131871	SELENOS	2137.949464	1469.9037	2805.995254	0.932377303	0.050036962	18.6337712	1.71E-77	4.88E-76
ENSG00000078053	AMPH	568.538636	390.87174	746.2055336	0.932367017	0.090870933	10.2603438	1.06E-24	1.13E-23
ENSG00000113108	APBB3	125.5745841	86.178117	164.9710517	0.932066497	0.205393717	4.53795039	5.68E-06	2.25E-05
ENSG00000136383	ALPK3	4764.880232	3282.3418	6247.418653	0.92862869	0.035520105	26.1437483	1.16E-150	6.39E-149
ENSG00000109906	ZBTB16	431.3000019	297.34172	565.258282	0.928255396	0.111897394	8.29559443	1.08E-16	8.51E-16
ENSG00000140859	KIFC3	2672.28895	1843.1759	3501.40202	0.924771227	0.048702842	18.988034	2.14E-80	6.46E-79
ENSG00000175538	KCNE3	106.8910129	73.888734	139.8932913	0.922903195	0.221587755	4.16495576	3.11E-05	0.000114
ENSG00000159128	IFNGR2	2744.450232	1894.9802	3593.920249	0.922667592	0.045800099	20.145537	2.94E-90	1.01E-88
ENSG00000188313	PLSCR1	2531.6852	1749.8336	3313.536754	0.920942136	0.050028683	18.4082826	1.13E-75	3.13E-74
ENSG00000168575	SLC20A2	1173.084479	810.75952	1535.409436	0.920662274	0.068200828	13.499283	1.58E-41	2.55E-40
ENSG00000146457	WTAP	3401.520261	2351.7134	4451.327153	0.920623572	0.041026174	22.439908	1.61E-111	6.46E-110
ENSG00000145779	TNFAIP8	408.1488281	281.75586	534.5417923	0.920412041	0.111106705	8.28403689	1.19E-16	9.36E-16
ENSG00000269378	AC022149.1	255.2518398	176.74506	333.7586208	0.920377501	0.142735323	6.44814109	1.13E-10	6.59E-10
ENSG00000215788	TNFRSF25	518.2769779	358.97	677.5839537	0.918782345	0.130579413	7.03619603	1.98E-12	1.28E-11
ENSG00000121281	ADCY7	922.0067876	638.26446	1205.749117	0.917969161	0.073151594	12.5488607	4.03E-36	5.71E-35
ENSG00000176697	BDNF	237.6589929	164.65063	310.6673542	0.917931368	0.138973733	6.60507096	3.97E-11	2.38E-10
ENSG00000153790	C7orf31	91.4807008	63.336318	119.6250831	0.916314099	0.235418008	3.89228551	9.93E-05	0.000342
ENSG00000234771	SLC25A25-AS1	54.03801506	37.544769	70.53126146	0.916051732	0.300933003	3.04403878	0.002334	0.006487
ENSG00000152495	CAMK4	61.47163317	42.479211	80.46405504	0.915549651	0.281699751	3.25009039	0.001154	0.003374
ENSG00000151229	SLC2A13	311.9373164	216.75514	407.1194919	0.914497721	0.136565136	6.69642155	2.14E-11	1.30E-10
ENSG00000273132	AL355312.4	149.6529749	103.69591	195.610036	0.911928449	0.176745038	5.1595703	2.48E-07	1.12E-06
ENSG00000189152	GRAPL	58.09206064	40.280514	75.90360746	0.911708466	0.284594351	3.20353676	0.001358	0.003921
ENSG00000248429	FAM198B-AS1	363.1018957	252.12645	474.0773452	0.911289578	0.114025696	7.99196681	1.33E-15	1.00E-14
ENSG00000006638	TBXA2R	113.0912547	78.57616	147.6063496	0.911223041	0.204993302	4.44513569	8.78E-06	3.41E-05
ENSG00000197461	PDGFA	1531.411995	1063.4695	1999.354444	0.910580921	0.062084672	14.6667589	1.05E-48	1.93E-47
ENSG00000038382	TRIO	12567.60101	8729.9285	16405.27356	0.910114891	0.0278359	32.695723	1.80E-234	1.63E-232

ENSG00000171056	SOX7	2414.279937	1677.9716	3150.588265	0.90905062	0.049356987	18.4178708	9.44E-76	2.63E-74
ENSG00000100968	NFATC4	1922.650059	1336.5442	2508.7559	0.908660078	0.055703682	16.3123881	8.06E-60	1.78E-58
ENSG00000167996	FTH1	106101.264	73798.598	138403.9296	0.907224555	0.051872939	17.4893608	1.73E-68	4.34E-67
ENSG00000196118	CCDC189	78.41352745	54.558529	102.2685261	0.906157617	0.239478331	3.78388146	0.000154	0.000518
ENSG00000083223	TUT7	1875.512557	1306.2832	2444.741889	0.904846259	0.059091185	15.3127114	6.29E-53	1.24E-51
ENSG00000206503	HLA-A	14835.37596	10330.112	19340.64033	0.904587842	0.029690285	30.4674688	7.03E-204	5.34E-202
ENSG00000111664	GNB3	129.5268782	90.217551	168.8362055	0.903701752	0.200494472	4.50736494	6.56E-06	2.59E-05
ENSG00000225697	SLC26A6	654.8980058	456.55276	853.2432521	0.902453252	0.091646741	9.84708502	7.06E-23	7.06E-22
ENSG00000125347	IRF1	1411.428888	984.07315	1838.784629	0.901990277	0.058161264	15.5084366	3.04E-54	6.13E-53
ENSG00000234964	FABP5P7	114.5403456	79.743474	149.3372168	0.901170249	0.208162656	4.32916387	1.50E-05	5.67E-05
ENSG00000161091	MFSD12	2358.1129	1644.707	3071.518801	0.900637604	0.047371737	19.0121298	1.35E-80	4.10E-79
ENSG00000126458	RRAS	2427.461289	1693.3855	3161.537043	0.900587479	0.056317359	15.9912947	1.47E-57	3.10E-56
ENSG00000165259	HDX	375.5731649	262.24341	488.9029199	0.900308554	0.119550994	7.53074921	5.05E-14	3.54E-13
ENSG00000135740	SLC9A5	138.8003588	97.064001	180.5367166	0.898792616	0.189973085	4.73115767	2.23E-06	9.25E-06
ENSG00000261971	MMP25-AS1	168.1199533	117.58575	218.6541577	0.897540097	0.173116829	5.18459182	2.16E-07	9.85E-07
ENSG00000225630	MTND2P28	50.75303841	35.322514	66.18356259	0.896132287	0.324349864	2.76285698	0.00573	0.014646
ENSG00000006459	KDM7A	622.139971	435.17566	809.1042839	0.89517427	0.094979761	9.42489492	4.31E-21	4.04E-20
ENSG00000010327	STAB1	26136.63925	18283.132	33990.14683	0.894494088	0.025586718	34.9593135	9.35E-268	9.99E-266
ENSG00000184524	CEND1	48.44172118	33.875101	63.00834159	0.894425701	0.325619298	2.74684488	0.006017	0.015319
ENSG00000178343	SHISA3	349.448681	244.31725	454.5801113	0.894336618	0.117494149	7.61175448	2.70E-14	1.92E-13
ENSG00000137522	RNF121	2261.234588	1581.2337	2941.235469	0.894295787	0.053745102	16.6395773	3.60E-62	8.27E-61
ENSG00000113555	PCDH12	2678.965354	1876.8453	3481.085371	0.892534692	0.055655098	16.0368901	7.06E-58	1.51E-56
ENSG00000226496	LINC00323	39.15966165	27.269006	51.05031765	0.891204357	0.374951864	2.37685005	0.017461	0.040002
ENSG00000270804	AC010326.4	100.5467289	70.356783	130.7366748	0.89085656	0.215606776	4.13185791	3.60E-05	0.000131
ENSG00000167994	RAB3IL1	90.76464048	63.720947	117.8083344	0.88998459	0.255748291	3.47992389	0.000502	0.001559
ENSG00000146205	ANO7	113.4400604	79.395374	147.4847467	0.88925311	0.214498739	4.14572653	3.39E-05	0.000124
ENSG00000146072	TNFRSF21	5898.799784	4137.6459	7659.95371	0.889102734	0.038063524	23.3583925	1.13E-120	4.89E-119
ENSG00000228315	GUSBP11	106.0032714	74.394998	137.6115453	0.88820971	0.229994719	3.86187002	0.000113	0.000384
ENSG00000101460	MAP1LC3A	765.6961239	536.89511	994.4971331	0.887215763	0.081747207	10.853163	1.93E-27	2.22E-26
ENSG00000122786	CALD1	18408.14171	12919.347	23896.93611	0.887102885	0.024953552	35.5501648	8.26E-277	9.10E-275
ENSG00000163293	NIPAL1	89.62947596	62.826538	116.4324139	0.885820919	0.25479496	3.47660299	0.000508	0.001576
ENSG00000117595	IRF6	1025.478416	720.80391	1330.152927	0.885766497	0.074017124	11.9670482	5.29E-33	7.01E-32
ENSG00000153214	TMEM87B	2896.309203	2034.6297	3757.988733	0.885579023	0.051080207	17.3370287	2.47E-67	6.15E-66
ENSG00000058091	CDK14	1420.783251	999.59404	1841.972458	0.882975343	0.066839345	13.210413	7.64E-40	1.19E-38
ENSG00000276248	AL442125.1	61.85803472	43.596003	80.12006687	0.882772882	0.288160317	3.06347831	0.002188	0.006112
ENSG00000130734	ATG4D	417.4615343	293.59513	541.3279412	0.882527815	0.117628091	7.50269605	6.25E-14	4.38E-13
ENSG00000158092	NCK1	1312.619487	922.23285	1703.00612	0.882270644	0.073907606	11.937481	7.55E-33	9.96E-32
ENSG00000134250	NOTCH2	5805.262824	4084.4981	7526.027525	0.88145476	0.036790093	23.9590252	7.44E-127	3.38E-125
ENSG00000187801	ZFP69B	156.3206965	109.94202	202.6993778	0.880843545	0.172412728	5.10892413	3.24E-07	1.45E-06
ENSG00000174718	RESF1	7173.41563	5052.7849	9294.046329	0.879812177	0.031923561	27.5599639	3.36E-167	2.03E-165
ENSG00000112763	BTN2A1	1512.940212	1065.2973	1960.58313	0.879493727	0.057962571	15.1734767	5.30E-52	1.03E-50
ENSG00000108669	CYTH1	2402.612571	1691.7621	3113.463052	0.87900763	0.0491652	17.8786545	1.73E-71	4.55E-70
ENSG00000207561	MIR635	31.92274221	22.474984	41.37050089	0.878280233	0.382417217	2.29665453	0.021638	0.048431
ENSG00000110697	PITPNM1	1452.346567	1023.0354	1881.657724	0.87801038	0.063328446	13.8643917	1.04E-43	1.75E-42
ENSG00000082512	TRAF5	706.6237519	498.34273	914.9047704	0.87789951	0.081318063	10.7958734	3.60E-27	4.11E-26
ENSG00000152413	HOMER1	285.5285078	200.9979	370.0591143	0.877436558	0.132382748	6.62802798	3.40E-11	2.05E-10
ENSG00000168268	NT5DC2	10371.29393	7310.4519	13432.13597	0.877410187	0.03057106	28.7006791	3.74E-181	2.55E-179
ENSG00000007129	CEACAM21	49.71463632	35.051269	64.37800345	0.877213205	0.315551793	2.77993415	0.005437	0.013962
ENSG00000220323	HIST2H2BD	63.29677981	44.757061	81.83649821	0.875348236	0.283942535	3.08283589	0.00205	0.005754
ENSG00000206341	HLA-H	149.339008	105.37135	193.3066683	0.874336086	0.175960424	4.96893602	6.73E-07	2.93E-06
ENSG00000276256	AC011043.1	278.3338469	196.88593	359.7817682	0.873924038	0.137127772	6.3730638	1.85E-10	1.06E-09
ENSG00000240053	LY6G5B	32.8527456	23.144076	42.56141568	0.873065662	0.367789072	2.37382165	0.017605	0.040281
ENSG00000163430	FSTL1	43666.33152	30864.93	56467.73288	0.871598003	0.022366894	38.9682182	0	0
ENSG00000123983	ACSL3	6266.698692	4428.9501	8104.44726	0.871536197	0.033094844	26.3345011	7.72E-153	4.33E-151
ENSG00000268516	AC020915.3	103.0176163	72.567408	133.4678241	0.870008672	0.256221818	3.39552923	0.000685	0.002085
ENSG00000204520	MICA	1980.28643	1401.652	2558.920827	0.868408129	0.050564764	17.1741756	4.15E-66	1.01E-64
ENSG00000215769	ARHGAP27P1-]	53.57375113	37.89476	69.25274258	0.868146793	0.302001551	2.87464349	0.004045	0.010672
ENSG00000123094	RASSF8	3068.195907	2172.0142	3964.377568	0.867052847	0.046546762	18.6275654	1.92E-77	5.47E-76
ENSG00000142173	COL6A2	107.1739835	76.083474	138.2644934	0.866976641	0.22488702	3.85516532	0.000116	0.000394
ENSG00000108797	CNTNAP1	2419.846045	1715.0768	3124.615334	0.866902595	0.051053907	16.980142	1.15E-64	2.74E-63
ENSG00000259877	AC009113.1	54.53479847	38.767573	70.30202376	0.866851568	0.293373061	2.95477562	0.003129	0.008453
ENSG00000175265	GOLGA8A	557.9678842	396.13737	719.7983982	0.862689144	0.12698243	6.79376779	1.09E-11	6.78E-11
ENSG00000164736	SOX17	1655.584918	1174.6535	2136.516331	0.862677953	0.067509872	12.7785452	2.16E-37	3.16E-36
ENSG00000066583	ISOC1	877.3519075	622.58699	1132.116823	0.862486996	0.073541985	11.7278178	9.18E-32	1.19E-30
ENSG00000160602	AC010761.1	78.44856764	55.647398	101.249737	0.862398567	0.255579655	3.37428489	0.00074	0.002238
ENSG00000221890	NPTXR	107.4826943	76.135481	138.8299076	0.861225012	0.222658737	3.86791474	0.00011	0.000375
ENSG00000280132	AC026471.6	43.02977992	30.472896	55.58666341	0.86093173	0.329506568	2.6127908	0.008981	0.022028
ENSG00000168386	FILIP1L	1466.289133	1042.1476	1890.430648	0.859933054	0.06286045	13.6800335	1.34E-42	2.21E-41
ENSG00000189050	RNFT1	225.0762391	160.28001	289.8724684	0.859738407	0.156957898	5.47750969	4.31E-08	2.08E-07
ENSG00000072310	SREBF1	3631.873251	2579.757	4683.989495	0.85961301	0.045296953	18.977281	2.63E-80	7.91E-79
ENSG00000133874	RNF122	607.7483254	431.93598	783.5606692	0.858988522	0.086630531	9.91554034	3.56E-23	3.61E-22
ENSG00000130827	PLXNA3	2957.799213	2103.7046	3811.893817	0.858032272	0.044718209	19.1875367	4.70E-82	1.47E-80
ENSG00000100647	SUSD6	2301.142833	1635.6135	2966.672183	0.857892691	0.050019514	17.1511602	6.16E-66	1.50E-64
ENSG00000170689	HOXB9	45.58243969	32.596772	58.56810768	0.855711481	0.335251392	2.55244721	0.010697	0.0258
ENSG00000150093	ITGB1	80313.1704	57181.01	103445.3307	0.855298379	0.020232532	42.2734236	0	0
ENSG00000269028	MTRNR2L12	51.68911322	36.740593	66.63763353	0.855077641	0.321211893	2.66203605	0.007767	0.019306
ENSG00000157224	CLDN12	431.1445829	307.07644	555.2127238	0.854993133	0.109416911	7.8140858	5.54E-15	4.08E-14
ENSG00000137331	IER3	13087.11181	9318.9684	16855.25521	0.85484655	0.036135377	23.656777	1.01E-123	4.42E-122
ENSG00000164307	ERAP1	6026.064887	4292.6248	7759.504935	0.854703165	0.035084141	24.3615243	4.38E-131	2.05E-129
ENSG00000006327	TNFRSF12A	3152.617034	2243.9594	4061.274657	0.854695565	0.046453987	18.3987558	1.34E-75	3.73E-74
ENSG00000075275	CELSR1	488.5230552	348.58402	628.4620907	0.852698907	0.099784466	8.54540735	1.28E-17	1.05E-16
ENSG00000175155	YPEL2	4311.317434	3073.8012	5548.833628	0.852292482	0.037528454	22.7105671	3.52E-114	1.45E-112
ENSG00000132003	ZSWIM4	1678.730647	1196.6735	2160.787785	0.852261535	0.056275283	15.144509	8.24E-52	1.60E-50
ENSG00000240065	PSMB9	211.3907382	150.75546	272.0260168	0.851889004	0.144267828	5.9049132	3.53E-09	1.86E-08
ENSG00000185947	ZNF267	922.7832641	659.16834	1186.398184	0.848938076	0.074158851	11.4475624	2.42E-30	3.03E-29

ENSG00000118523	CCN2	85769.92546	61243.428	110296.4232	0.848756864	0.071065337	11.9433313	7.03E-33	9.29E-32
ENSG00000279672	AP006621.5	58.77543336	41.983798	75.56706831	0.84846063	0.281093552	3.01842794	0.002541	0.007004
ENSG00000148143	ZNF462	1155.24065	825.63384	1484.84746	0.848397351	0.070895999	11.9667875	5.30E-33	7.02E-32
ENSG00000165097	KDM1B	714.9643967	510.48443	919.4443624	0.84718015	0.086116681	9.83758481	7.76E-23	7.75E-22
ENSG00000099308	MAST3	227.5251079	162.47931	292.5709087	0.84688872	0.148017712	5.72153634	1.06E-08	5.36E-08
ENSG00000171606	ZNF274	785.2606976	561.41703	1009.104364	0.846495842	0.078688695	10.757528	5.46E-27	6.20E-26
ENSG00000180758	GPR157	372.99401	266.60173	479.3862883	0.844863304	0.120062466	7.03686449	1.97E-12	1.28E-11
ENSG00000065802	ASB1	1195.36903	855.53088	1535.207181	0.843239419	0.065473735	12.8790487	5.91E-38	8.79E-37
ENSG00000161912	ADCY10P1	79.39617466	56.991502	101.8008469	0.841156143	0.261247456	3.21976778	0.001283	0.003723
ENSG00000260401	AP002761.4	148.2870444	106.13193	190.4421621	0.840236944	0.175916413	4.77634196	1.79E-06	7.46E-06
ENSG00000157181	ODR4	1030.932922	739.14288	1322.722966	0.840227123	0.071212718	11.7988351	3.96E-32	5.14E-31
ENSG00000156466	GDF6	140.9111448	100.82812	180.9941654	0.839161359	0.203920818	4.11513335	3.87E-05	0.00014
ENSG00000206573	THUMPD3-AS1	193.4585689	138.41527	248.5018681	0.839083856	0.16089808	5.21500228	1.84E-07	8.41E-07
ENSG00000198814	GK	355.0048046	254.46378	455.5458301	0.838867979	0.11663554	7.19221585	6.37E-13	4.24E-12
ENSG00000105290	APLP1	259.870062	186.14976	333.5903673	0.837963152	0.142916208	5.86331785	4.54E-09	2.37E-08
ENSG00000159231	CBR3	549.9805338	394.4511	705.5099652	0.836557997	0.095381104	8.77068894	1.78E-18	1.50E-17
ENSG00000224531	SMIM13	1554.225138	1116.7452	1991.705052	0.836364374	0.064009208	13.0663135	5.13E-39	7.83E-38
ENSG00000163110	PDLIM5	11244.3016	8076.8045	14411.79874	0.835792681	0.032076112	26.0565461	1.13E-149	6.17E-148
ENSG00000204525	HLA-C	9478.66921	6807.463	12149.87543	0.835684274	0.030096377	27.7669389	1.09E-169	6.67E-168
ENSG00000171928	TVP23B	1183.581237	849.8433	1517.319176	0.835662059	0.069315865	12.0558556	1.81E-33	2.41E-32
ENSG00000184588	PDE4B	4761.013658	3421.1577	6100.869575	0.835174615	0.041125632	20.3078854	1.10E-91	3.85E-90
ENSG00000060642	PIGV	324.2561568	233.26453	415.2477851	0.835159261	0.134233678	6.22168202	4.92E-10	2.74E-09
ENSG00000130513	GDF15	5011.174546	3599.7681	6422.580944	0.835144321	0.0344474	24.2440453	7.64E-130	3.54E-128
ENSG00000197312	DDI2	2550.943424	1833.7113	3268.175536	0.834921357	0.047367873	17.6263216	1.55E-69	3.94E-68
ENSG00000073849	ST6GAL1	16563.38568	11902.262	21224.50907	0.834900674	0.027260661	30.6265753	5.42E-206	4.19E-204
ENSG00000013375	PGM3	4301.854574	3091.241	5512.468167	0.834610776	0.036987669	22.5645681	9.66E-113	3.93E-111
ENSG00000175040	CHST2	998.7367569	717.74225	1279.731259	0.834094853	0.078589076	10.6133688	2.58E-26	2.90E-25
ENSG00000213398	LCAT	155.3403905	111.69727	198.9835083	0.833147935	0.185411766	4.49350088	7.01E-06	2.75E-05
ENSG00000173926	3-Mar	2156.163631	1549.87	2762.457237	0.832814465	0.050584497	16.4638281	6.67E-61	1.49E-59
ENSG00000143162	CREG1	505.959221	363.43787	648.4805712	0.832519505	0.101641303	8.19075985	2.60E-16	2.01E-15
ENSG00000280143	AP000892.3	67.48237161	48.426658	86.53808508	0.832447129	0.264928678	3.1421556	0.001677	0.00477
ENSG00000226328	NUP50-DT	138.9780948	99.884341	178.0718483	0.832418804	0.18809226	4.42558777	9.62E-06	3.72E-05
ENSG00000197780	TAF13	1274.747097	917.73224	1631.761952	0.831160313	0.062487021	13.3013272	2.27E-40	3.60E-39
ENSG00000245067	IGFBP7-AS1	66.40695012	47.646375	85.16752546	0.830762999	0.285232655	2.91258026	0.003585	0.009564
ENSG00000149289	ZC3H12C	1394.71188	1003.4112	1786.012546	0.8302862	0.062438065	13.2977567	2.39E-40	3.77E-39
ENSG00000117758	STX12	4011.038956	2889.2353	5132.842568	0.829596011	0.040349612	20.5601981	6.24E-94	2.24E-92
ENSG00000270362	HMGN3-AS1	64.33477218	46.373047	82.29649731	0.829304173	0.282748288	2.93301218	0.003357	0.009009
ENSG00000154262	ABCA6	1992.224391	1434.9676	2549.481142	0.82887927	0.052444611	15.8048513	2.88E-56	5.98E-55
ENSG00000091039	OSBPL8	4218.966285	3043.4514	5394.481191	0.826706379	0.042627859	19.3935705	8.75E-84	2.79E-82
ENSG00000223745	CCDC18-AS1	48.34722466	34.957078	61.73737117	0.826497426	0.320423775	2.57938858	0.009898	0.024075
ENSG00000198843	SELENOT	4177.751809	3013.5922	5341.911433	0.825899703	0.043910086	18.8088837	6.39E-79	1.86E-77
ENSG00000100558	PLEK2	266.5556892	192.14843	340.9629441	0.824976382	0.133645467	6.17287216	6.71E-10	3.70E-09
ENSG00000119669	IRF2BPL	1425.839328	1029.5252	1822.153466	0.82356113	0.059186749	13.91462	5.16E-44	8.73E-43
ENSG00000153982	GDPD1	245.9570344	177.4059	314.5081723	0.823310584	0.153571869	5.36107681	8.27E-08	3.90E-07
ENSG00000135299	ANKRD6	120.2135848	86.825685	153.601485	0.823072443	0.194387879	4.23417575	2.29E-05	8.54E-05
ENSG00000119636	BBOF1	111.3294868	80.55995	142.0990235	0.823016213	0.204546944	4.02360551	5.73E-05	0.000203
ENSG00000108771	DHX58	163.1104159	117.78406	208.4367692	0.822571208	0.166287667	4.94667598	7.55E-07	3.27E-06
ENSG00000088543	C3orf18	95.35448496	68.925963	121.7830066	0.821469463	0.215057487	3.81976686	0.000134	0.000452
ENSG00000130479	MAP1S	3944.025031	2850.9859	5037.06421	0.820630968	0.040523117	20.2509339	3.49E-91	1.22E-89
ENSG00000260996	BX255925.1	44.44605244	32.049138	56.84296686	0.820593066	0.357523108	2.29521686	0.021721	0.04858
ENSG00000163697	APBB2	9448.574942	6833.2656	12063.88425	0.820463836	0.030684576	26.7386403	1.67E-157	9.71E-156
ENSG00000129467	ADCY4	1815.609466	1313.4872	2317.731686	0.820377497	0.062436025	13.1394896	1.96E-39	3.03E-38
ENSG00000260852	FBXL19-AS1	162.0772057	117.14001	207.0143998	0.820317796	0.176308476	4.65274169	3.28E-06	1.33E-05
ENSG00000140564	FURIN	16743.41631	12108.851	21377.98118	0.819959141	0.028672832	28.5970756	7.31E-180	4.90E-178
ENSG00000163376	KBTBD8	118.817991	85.862868	151.7731138	0.81902482	0.21405999	3.82614621	0.00013	0.000441
ENSG00000119801	YPEL5	2772.098468	2006.7438	3537.453087	0.81893383	0.044938456	18.2234526	3.36E-74	9.07E-73
ENSG00000235823	OLMALINC	148.5761511	107.71628	189.4360219	0.818830485	0.192356674	4.25683428	2.07E-05	7.76E-05
ENSG00000124201	ZNFX1	4974.494221	3600.8625	6348.125928	0.818018934	0.034295311	23.8522095	9.61E-126	4.32E-124
ENSG00000146242	TPBG	556.5844626	402.38925	710.7796775	0.816763304	0.113307634	7.20836959	5.66E-13	3.78E-12
ENSG00000169359	SLC33A1	737.637488	534.69575	940.5792297	0.815998539	0.089690784	9.09790847	9.21E-20	8.22E-19
ENSG00000121966	CXCR4	3055.890645	2215.6329	3896.148434	0.815608247	0.048633762	16.7704126	4.02E-63	9.34E-62
ENSG00000267745	AC060766.7	51.16952425	37.095179	65.24386986	0.814124814	0.307879701	2.6442952	0.008186	0.020241
ENSG00000160233	LRRC3	638.4204589	462.75069	814.0902318	0.814087401	0.100910703	8.06740396	7.18E-16	5.47E-15
ENSG00000224982	TMEM233	58.77016284	42.47421	75.0661157	0.813965164	0.288134298	2.82495062	0.004729	0.012298
ENSG00000121653	MAPK8IP1	338.066257	245.45364	430.6788734	0.813171364	0.14023272	5.79872773	6.68E-09	3.44E-08
ENSG00000135596	MICAL1	1659.575643	1203.8108	2115.340502	0.812680343	0.059052396	13.7620216	4.31E-43	7.18E-42
ENSG00000147202	DIAPH2	2238.491923	1626.3915	2850.59233	0.811201046	0.053023361	15.2989368	7.77E-53	1.53E-51
ENSG00000055813	CCDC85A	545.0166392	395.51786	694.5154139	0.81104091	0.107493923	7.54499313	4.52E-14	3.18E-13
ENSG00000197256	KANK2	3023.142469	2194.5734	3851.71152	0.810740297	0.044201259	18.3420182	3.82E-75	1.05E-73
ENSG00000124067	SLC12A4	3474.006784	2523.6014	4424.412181	0.810243091	0.041148848	19.6905411	2.60E-86	8.57E-85
ENSG00000214176	PLEKHM1P1	342.0917743	248.09797	436.0855809	0.808935003	0.128249205	6.30752452	2.84E-10	1.61E-09
ENSG00000070961	ATP2B1	3526.759367	2566.0828	4487.435979	0.807098522	0.044385081	18.1840047	6.91E-74	1.86E-72
ENSG00000174125	TLR1	221.7861115	161.09627	282.4759508	0.807018237	0.150677753	5.35592162	8.51E-08	4.01E-07
ENSG00000105464	GRIN2D	176.7900273	128.75281	224.8272447	0.806865043	0.161944055	4.98236902	6.28E-07	2.74E-06
ENSG00000204271	SPIN3	158.66659	115.22249	202.1106895	0.806767178	0.191768584	4.20698303	2.59E-05	9.59E-05
ENSG00000172578	KLHL6	1174.783066	854.14846	1495.417669	0.806733089	0.064395428	12.5278006	5.26E-36	7.42E-35
ENSG00000095397	WHRN	44.09844922	32.024097	56.17280148	0.806585669	0.316453726	2.54882658	0.010809	0.026039
ENSG00000099139	PCSK5	387.4535903	282.49478	492.4124017	0.804864998	0.129076539	6.23556382	4.50E-10	2.52E-09
ENSG00000130775	THEMIS2	594.4067183	432.90276	755.9106804	0.804705302	0.091963796	8.75024019	2.13E-18	1.80E-17
ENSG00000137496	IL18BP	510.5339128	371.50493	649.5628919	0.804668973	0.098554129	8.16474133	3.22E-16	2.49E-15
ENSG00000123358	NR4A1	129.9647851	94.591619	165.3379513	0.804515984	0.207179215	3.88318869	0.000103	0.000354
ENSG00000169330	MINAR1	71.02537625	51.970909	90.07984302	0.802653766	0.267989272	2.99509663	0.002744	0.007508
ENSG00000228589	SPCS2P4	36.65741401	26.720701	46.59412696	0.802232505	0.346552601	2.31489391	0.020619	0.046414
ENSG00000161940	BCL6B	7100.898543	5179.2459	9022.551216	0.800355424	0.032861075	24.3557288	5.04E-131	2.35E-129
ENSG00000161040	FBXL13	167.664402	122.45703	212.871771	0.800222796	0.177169954	4.51669585	6.28E-06	2.48E-05

ENSG00000078804	TP53INP2	3304.245831	2414.5366	4193.955056	0.797238686	0.046389408	17.1857915	3.39E-66	8.30E-65
ENSG00000267519	AC020916.1	283.6118593	207.06131	360.1624077	0.796790553	0.15138868	5.26321091	1.42E-07	6.54E-07
ENSG00000166394	CYB5R2	291.3033263	212.69719	369.9094673	0.795816372	0.12752846	6.24030409	4.37E-10	2.45E-09
ENSG00000118473	SGIP1	834.4772334	610.70102	1058.253444	0.795481667	0.0790208	10.0667377	7.75E-24	8.04E-23
ENSG00000145246	ATP10D	2537.365073	1855.5677	3219.16242	0.794718371	0.049778896	15.9649658	2.24E-57	4.71E-56
ENSG00000237440	AC008554.1	174.8286755	128.08757	221.5697805	0.794414876	0.170571496	4.65737181	3.20E-06	1.30E-05
ENSG00000116774	OLFML3	74.78101876	54.827954	94.73408326	0.793541688	0.293330291	2.70528382	0.006825	0.017161
ENSG00000281649	EBLN3P	1549.536972	1133.9868	1965.087188	0.792734352	0.059858116	13.2435568	4.92E-40	7.70E-39
ENSG00000186594	MIR22HG	1011.819888	741.17311	1282.466667	0.791591697	0.080501006	9.8333144	8.09E-23	8.08E-22
ENSG00000112893	MAN2A1	6708.552355	4914.6257	8502.478967	0.791507716	0.035167334	22.5069015	3.55E-112	1.44E-110
ENSG00000240771	ARHGEF25	215.567239	157.94082	273.1936574	0.791238568	0.15168906	5.21618743	1.83E-07	8.37E-07
ENSG00000151151	IPMK	619.3349758	453.79809	784.8718649	0.790277311	0.113747222	6.94766252	3.71E-12	2.37E-11
ENSG00000086544	ITPKC	1776.292408	1301.9329	2250.651893	0.789808942	0.052209443	15.1277028	1.06E-51	2.06E-50
ENSG00000151726	ACSL1	1493.810553	1095.2476	1892.373545	0.788267959	0.064678715	12.1874401	3.63E-34	4.90E-33
ENSG00000135905	DOCK10	2153.290702	1579.8541	2726.727285	0.787968251	0.060162597	13.0973111	3.41E-39	5.23E-38
ENSG00000205413	SAMD9	1457.921816	1069.8546	1845.989014	0.785968384	0.071968219	10.9210482	9.14E-28	1.06E-26
ENSG00000104936	DMPK	796.7389689	585.60511	1007.872829	0.785679201	0.088813717	8.84637224	9.04E-19	7.72E-18
ENSG00000117597	UTP25	1531.76371	1124.835	1938.692444	0.785479612	0.057782685	13.593685	4.37E-42	7.14E-41
ENSG00000270069	MIR222HG	62.16224887	45.594425	78.73007314	0.785031629	0.303321453	2.58811772	0.00965	0.023511
ENSG00000051523	CYBA	864.8730341	634.94194	1094.804133	0.783842864	0.078695844	9.96040996	2.27E-23	2.31E-22
ENSG00000112425	EPM2A	202.8452364	149.18996	256.5005099	0.783752254	0.159259866	4.92121634	8.60E-07	3.70E-06
ENSG00000132334	PTPRE	6273.793866	4611.4159	7936.171866	0.783629003	0.031988742	24.4970246	1.59E-132	7.57E-131
ENSG00000146350	TBC1D32	96.96591847	71.205298	122.7265393	0.783336275	0.234922265	3.33444884	0.000855	0.002555
ENSG00000173846	PLK3	1039.008726	764.18392	1313.833528	0.783145915	0.07426816	10.5448406	5.37E-26	5.97E-25
ENSG00000177409	SAMD9L	2154.382814	1584.5812	2724.184446	0.782625324	0.050926783	15.3676567	2.70E-53	5.36E-52
ENSG00000174021	GNG5	542.1707811	398.76979	685.5717743	0.782556422	0.093516893	8.36807555	5.86E-17	4.67E-16
ENSG00000163840	DTX3L	2254.970649	1658.5893	2851.351956	0.782185386	0.047152445	16.5884373	8.45E-62	1.92E-60
ENSG00000184988	TMEM106A	459.3090045	338.31967	580.2983366	0.779061863	0.122910073	6.33847045	2.32E-10	1.33E-09
ENSG00000227671	AL390728.4	535.738874	394.85305	676.6246981	0.77858945	0.114485637	6.80076099	1.04E-11	6.48E-11
ENSG00000158246	TENT5B	88.38525858	64.896302	111.8742154	0.778209247	0.249829802	3.11495763	0.00184	0.005205
ENSG00000108433	GOSR2	2092.295639	1540.981	2643.610252	0.777954895	0.057584241	13.5098575	1.37E-41	2.22E-40
ENSG00000158201	ABHD3	238.5367754	175.96322	301.1103307	0.777756504	0.14098552	5.51657011	3.46E-08	1.69E-07
ENSG00000115520	COQ10B	891.7619542	656.82168	1126.702231	0.776288044	0.085144883	9.1172601	7.70E-20	6.90E-19
ENSG00000143630	HCN3	68.87681832	50.849117	86.90451975	0.775685138	0.282441981	2.74635214	0.006026	0.01534
ENSG00000105137	SYDE1	1514.978869	1116.6361	1913.32166	0.775657545	0.071424647	10.8598022	1.79E-27	2.07E-26
ENSG00000132481	AC087289.1	2181.75962	1608.8101	2754.709162	0.775179083	0.059876128	12.9463795	2.46E-38	3.71E-37
ENSG00000070778	AL162171.1	1347.114001	993.38947	1700.838533	0.774795778	0.063263157	12.2471879	1.74E-34	2.37E-33
ENSG00000285517	AC010198.2	119.2516291	88.157383	150.345875	0.774135964	0.202457579	3.82369466	0.000131	0.000445
ENSG00000138495	COX17	383.3525993	282.5083	484.1969021	0.774120509	0.127022759	6.09434494	1.10E-09	5.97E-09
ENSG00000104219	ZDHHC2	2637.601285	1948.7387	3326.463827	0.772735563	0.051675461	14.953627	1.47E-50	2.81E-49
ENSG00000148288	GBGT1	1008.941199	745.2722	1272.610194	0.772336569	0.072856679	10.6007654	2.96E-26	3.31E-25
ENSG00000108395	TRIM37	3366.082351	2486.6941	4245.470553	0.7721942	0.04263477	18.1118415	2.57E-73	6.86E-72
ENSG00000242265	PEG10	3542.919688	2617.3647	4468.474702	0.772068338	0.040713699	18.9633552	3.43E-80	1.03E-78
ENSG00000121578	B4GALT4	341.1016484	252.1983	430.0049967	0.771820038	0.116771587	6.60965614	3.85E-11	2.31E-10
ENSG00000073605	GSDMB	54.802648	40.587386	69.01791007	0.771026553	0.306195714	2.51808408	0.0118	0.028194
ENSG00000168026	TTC21A	99.76390983	73.930776	125.5970438	0.770076315	0.243601624	3.16121174	0.001571	0.004488
ENSG00000151338	MIPOL1	1001.227099	740.52722	1261.926978	0.769574064	0.069120479	11.1338068	8.59E-29	1.03E-27
ENSG00000106624	AEBP1	8652.945828	6401.4997	10904.39197	0.768380043	0.030014428	25.600356	1.51E-144	7.90E-143
ENSG00000124762	CDKN1A	12420.92367	9197.5238	15644.32352	0.766464755	0.026721037	28.6839449	6.05E-181	4.09E-179
ENSG00000137563	GGH	1898.198376	1405.4625	2390.934227	0.765626228	0.056553914	13.5379883	9.33E-42	1.52E-40
ENSG00000074964	ARHGEF10L	218.0154394	161.31003	274.7208481	0.765120287	0.150188036	5.0944157	3.50E-07	1.56E-06
ENSG00000160190	SLC37A1	566.1458178	419.41644	712.8751953	0.764417282	0.090436958	8.45248775	2.85E-17	2.30E-16
ENSG00000100911	PSME2	2426.559813	1799.1526	3053.967032	0.764364056	0.051482052	14.8471949	7.25E-50	1.36E-48
ENSG00000163945	UVSSA	494.4286157	366.01768	622.839552	0.764361632	0.107048284	7.14034455	9.31E-13	6.14E-12
ENSG00000115255	REEP6	91.14727439	67.546867	114.7476621	0.764328209	0.219575432	3.48093684	0.0005	0.001553
ENSG00000110651	CD81	16496.74705	12227.392	20766.10256	0.764194033	0.023922427	31.9446695	6.41E-224	5.40E-222
ENSG00000127080	IPPK	807.5383653	598.74023	1016.336501	0.763934242	0.075421009	10.1289317	4.11E-24	4.31E-23
ENSG00000118515	SGK1	3596.200932	2665.8988	4526.50305	0.763523371	0.047688717	16.0105665	1.08E-57	2.29E-56
ENSG00000180155	LYNX1	194.0462301	143.84486	244.2475992	0.761813738	0.158431104	4.80848594	1.52E-06	6.41E-06
ENSG00000196839	ADA	1152.330501	854.78963	1449.87137	0.76170005	0.067771546	11.2392308	2.62E-29	3.20E-28
ENSG00000150687	PRSS23	29356.16327	21793.712	36918.61486	0.76036738	0.021591348	35.2163003	1.13E-271	1.22E-269
ENSG00000196586	MYO6	8667.792082	6442.3327	10893.25149	0.757925746	0.02999026	25.2723969	6.43E-141	3.26E-139
ENSG00000109576	AADAT	60.20991619	44.738841	75.68099112	0.757132702	0.307649414	2.46102436	0.013854	0.032608
ENSG00000163453	IGFBP7	21639.37497	16094.175	27184.57493	0.756223693	0.079999368	9.45287083	3.30E-21	3.11E-20
ENSG00000179387	ELMOD2	935.5816428	695.89994	1175.263345	0.755909764	0.079571396	9.49976883	2.10E-21	2.00E-20
ENSG00000173193	PARP14	5165.396525	3845.5271	6485.265943	0.754498406	0.040518796	18.6209481	2.17E-77	6.18E-76
ENSG00000178852	EFCAB13	44.09418029	32.869572	55.31878854	0.754253254	0.328499701	2.29605461	0.021673	0.048496
ENSG00000238058	AL355574.1	54.56091154	40.592316	68.52950697	0.754126032	0.29705834	2.53864622	0.011128	0.026725
ENSG00000103005	USB1	3387.586844	2523.9025	4251.271151	0.753029157	0.044165696	17.0500913	3.49E-65	8.42E-64
ENSG00000196458	ZNF605	580.656756	432.93665	728.3768607	0.752304669	0.08935686	8.41910372	3.79E-17	3.05E-16
ENSG00000267102	AC060766.1	112.495798	83.91626	141.0753357	0.751636102	0.20096269	3.74017734	0.000184	0.00061
ENSG00000137752	CASP1	325.0884634	242.0298	408.1471221	0.751206513	0.122347586	6.13993733	8.26E-10	4.52E-09
ENSG00000279059	AC007485.2	40.35878013	30.119459	50.59810091	0.750648179	0.327608529	2.29129621	0.021946	0.049025
ENSG00000130305	NSUN5	156.4627512	116.84487	196.0806282	0.7495626	0.171534881	4.36973867	1.24E-05	4.76E-05
ENSG00000171940	ZNF217	2363.289684	1762.8769	2963.702458	0.749360001	0.047224457	15.8680492	1.05E-56	2.20E-55
ENSG00000259642	ST20-AS1	103.8076384	77.711458	129.9038185	0.746910178	0.218134368	3.42408299	0.000617	0.001895
ENSG00000221944	TIGD1	83.59455906	62.426129	104.7629887	0.746579376	0.292153688	2.55543368	0.010606	0.025601
ENSG00000089639	GMIP	1263.401729	943.33905	1583.464409	0.746332964	0.063389828	11.7737023	5.33E-32	6.92E-31
ENSG00000176261	ZBTB8OS	488.9702833	365.3631	612.5774694	0.744996929	0.099056801	7.5209064	5.44E-14	3.82E-13
ENSG00000245532	NEAT1	2486.088513	1858.1771	3113.999907	0.744934873	0.184065747	4.04711297	5.19E-05	0.000185
ENSG00000168389	MFSD2A	152.1721191	113.88496	190.459277	0.743624023	0.200765435	3.70394447	0.000212	0.000698
ENSG00000166173	LARP6	556.4431948	416.44845	696.4379376	0.743387231	0.090972588	8.17155198	3.04E-16	2.36E-15
ENSG00000259207	ITGB3	2287.889154	1711.3105	2864.467852	0.743086392	0.048641505	15.2767969	1.09E-52	2.14E-51
ENSG00000136603	SKIL	5205.775877	3895.8105	6515.741292	0.742937553	0.039698249	18.7146177	3.76E-78	1.09E-76
ENSG00000135899	SP110	857.7276764	641.9269	1073.528454	0.7422224	0.076552153	9.69564368	3.15E-22	3.08E-21

ENSG00000260920	AL031985.3	96.59222272	72.637744	120.5467016	0.739869347	0.246811399	2.99771141	0.00272	0.007451
ENSG00000198053	SIRPA	7669.661242	5746.9093	9592.413212	0.739470895	0.031276496	23.6430221	1.39E-123	6.11E-122
ENSG00000106070	GRB10	16788.20309	12582.615	20993.79148	0.738872024	0.028574094	25.8581086	1.97E-147	1.05E-145
ENSG00000174130	TLR6	92.45513816	69.097274	115.8130024	0.73836738	0.255000355	2.89555433	0.003785	0.010049
ENSG00000162645	GBP2	2228.145603	1670.067	2786.224185	0.738101752	0.051613017	14.3006898	2.17E-46	3.83E-45
ENSG00000223705	NSUN5P1	128.9246714	96.695967	161.1533761	0.738075372	0.215825182	3.41978339	0.000627	0.001922
ENSG00000100403	ZC3H7B	10975.41308	8227.0814	13723.74476	0.737962129	0.027279719	27.0516767	3.65E-161	2.14E-159
ENSG00000019144	PHLDB1	7507.252106	5627.4208	9387.083439	0.737672313	0.032200063	22.9090329	3.78E-116	1.57E-114
ENSG00000164951	PDP1	2688.817639	2016.8367	3360.798537	0.736937847	0.045945088	16.0395351	6.77E-58	1.45E-56
ENSG00000069482	GAL	254.5030355	190.60642	318.3996477	0.736287287	0.159184262	4.62537741	3.74E-06	1.51E-05
ENSG00000269352	PTOV1-AS2	103.6022624	77.638968	129.5655568	0.735897994	0.219930707	3.34604477	0.00082	0.002463
ENSG00000271122	AC018647.2	301.0902247	226.44738	375.7330707	0.735691704	0.136731209	5.38056899	7.43E-08	3.52E-07
ENSG00000253731	PCDHGA6	131.4627115	98.884962	164.0404605	0.735519751	0.196709499	3.7391166	0.000185	0.000612
ENSG00000171621	SPSB1	3966.230243	2976.2135	4956.246969	0.735427373	0.043900491	16.7521445	5.46E-63	1.27E-61
ENSG00000189067	LITAF	345.0100103	258.99878	431.0212356	0.735377533	0.118270684	6.21774987	5.04E-10	2.81E-09
ENSG00000125744	RTN2	408.7891078	307.41336	510.1648589	0.733474893	0.117572717	6.2384787	4.42E-10	2.47E-09
ENSG00000146433	TMEM181	4793.162734	3601.2202	5985.105311	0.733310135	0.036615054	20.0275585	3.17E-89	1.08E-87
ENSG00000136147	PHF11	501.954062	377.39574	626.5123799	0.733228471	0.098789418	7.42213578	1.15E-13	7.98E-13
ENSG00000059377	TBXAS1	63.18209804	47.59085	78.77334582	0.732899876	0.274539048	2.66956515	0.007595	0.018917
ENSG00000261659	Z92544.2	55.11591607	41.334004	68.89782803	0.732895748	0.294611053	2.48767228	0.012858	0.030494
ENSG00000151640	DPYSL4	1175.180201	884.27141	1466.088997	0.730417642	0.072174353	10.1201827	4.50E-24	4.70E-23
ENSG00000160209	PDXK	2292.829854	1724.2121	2861.447575	0.730070283	0.053827026	13.5632663	6.61E-42	1.08E-40
ENSG00000142871	CCN1	24069.45763	18111.06	30027.85555	0.729165894	0.024852882	29.3392894	3.27E-189	2.27E-187
ENSG00000188157	AGRN	14451.10389	10875.733	18026.47436	0.728887596	0.030325467	24.0354948	1.18E-127	5.39E-126
ENSG00000066629	EML1	3173.966806	2390.9925	3956.941141	0.726540527	0.04238527	17.141345	7.29E-66	1.77E-64
ENSG00000129925	TMEM8A	6605.409372	4977.5319	8233.28682	0.725887468	0.036638388	19.8122109	2.34E-87	7.77E-86
ENSG00000027697	IFNGR1	2140.793398	1614.5902	2666.996567	0.725292198	0.052944461	13.6991137	1.03E-42	1.70E-41
ENSG00000100612	DHRS7	1172.321374	884.27347	1460.369276	0.724169187	0.063606944	11.3850649	4.96E-30	6.17E-29
ENSG00000103253	HAGHL	146.9679639	110.93176	183.0041644	0.724125676	0.180364328	4.01479429	5.95E-05	0.000211
ENSG00000128284	APOL3	2571.972887	1940.6508	3203.295018	0.723921591	0.04842426	14.9495643	1.57E-50	2.98E-49
ENSG00000182010	RTKN2	62.10915944	46.901312	77.31700697	0.72374025	0.273014062	2.65092663	0.008027	0.019893
ENSG00000241889	AC079944.2	47.3660863	35.760847	58.9713253	0.721317796	0.30551271	2.36100749	0.018225	0.041535
ENSG00000014914	MTMR11	266.7791556	201.83892	331.7193873	0.721294649	0.138329564	5.21432026	1.84E-07	8.44E-07
ENSG00000197930	ERO1A	4447.505189	3359.3142	5535.696215	0.721146411	0.038150976	18.9024366	1.09E-79	3.23E-78
ENSG00000100600	LGMN	4961.192056	3747.4076	6174.976529	0.720674799	0.035695913	20.1892804	1.22E-90	4.20E-89
ENSG00000108465	CDK5RAP3	97.220036	73.534484	120.9055879	0.719893208	0.226011732	3.18520283	0.001447	0.004156
ENSG00000177663	IL17RA	2090.371057	1580.4394	2600.302719	0.71965048	0.052334613	13.7509468	5.03E-43	8.34E-42
ENSG00000271643	AC112220.2	252.9307888	191.14291	314.7186719	0.719475085	0.140914996	5.10573823	3.30E-07	1.48E-06
ENSG00000141380	SS18	2729.713967	2064.6047	3394.823215	0.718483996	0.045545208	15.7751832	4.61E-56	9.53E-55
ENSG00000173530	TNFRSF10D	8279.608885	6264.3121	10294.90569	0.71689534	0.036097751	19.8598337	9.06E-88	3.04E-86
ENSG00000213516	RBMXL1	593.4656924	449.336	737.5953886	0.716342821	0.09136139	7.84076097	4.48E-15	3.31E-14
ENSG00000167291	TBC1D16	8700.786613	6589.4227	10812.15052	0.714737514	0.029556276	24.1822586	3.42E-129	1.58E-127
ENSG00000237187	NR2F1-AS1	399.2373755	302.31691	496.1578361	0.713436125	0.106869832	6.67574857	2.46E-11	1.50E-10
ENSG00000182197	EXT1	5603.047802	4247.8053	6958.290278	0.712634246	0.035216137	20.2360142	4.72E-91	1.64E-89
ENSG00000057019	DCBLD2	5157.532247	3909.7879	6405.276566	0.712514368	0.033972976	20.9729745	1.16E-97	4.27E-96
ENSG00000151012	SLC7A11	6981.600428	5293.3769	8669.823975	0.712094753	0.030685708	23.2060718	3.95E-119	1.69E-117
ENSG00000087245	MMP2	45703.41119	34665.383	56741.43978	0.711008098	0.029989513	23.7085579	2.94E-124	1.30E-122
ENSG00000198668	CALM1	25694.56672	19493.985	31895.1487	0.710321804	0.021979419	32.3175885	3.96E-229	3.44E-227
ENSG00000143367	TUFT1	181.5404063	137.49812	225.582695	0.709993184	0.182973936	3.88029684	0.000104	0.000358
ENSG00000183723	CMTM4	563.2794289	427.67523	698.8836321	0.709835875	0.091975711	7.7176449	1.18E-14	8.56E-14
ENSG00000102096	PIM2	276.2446172	210.15398	342.3352585	0.706169064	0.131042725	5.38884602	7.09E-08	3.37E-07
ENSG00000166033	HTRA1	13726.96573	10434.352	17019.57952	0.705955391	0.03062387	23.0524555	1.39E-117	5.88E-116
ENSG00000171451	DSEL	1602.058454	1218.6839	1985.433045	0.70367321	0.057032166	12.3381814	5.64E-35	7.77E-34
ENSG00000140332	TLE3	3541.05904	2694.8923	4387.225751	0.703561597	0.054254219	12.9678689	1.86E-38	2.81E-37
ENSG00000214021	TTLL3	210.4683445	160.00051	260.9361784	0.70347747	0.154303963	4.55903696	5.14E-06	2.04E-05
ENSG00000138496	PARP9	1452.998413	1105.799	1800.197783	0.702723626	0.061004772	11.5191584	1.06E-30	1.34E-29
ENSG00000175505	CLCF1	139.8181932	106.54522	173.0911676	0.702702175	0.188693225	3.72404561	0.000196	0.000648
ENSG00000104361	NIPAL2	406.8734835	309.8089	503.9380672	0.700251701	0.108435627	6.45776414	1.06E-10	6.20E-10
ENSG00000197971	MBP	445.9238423	340.09948	551.748208	0.700112945	0.10234982	6.84039253	7.90E-12	4.96E-11
ENSG00000278948	AL031587.5	616.7420615	469.64625	763.8378766	0.700001007	0.087871578	7.96618223	1.64E-15	1.23E-14
ENSG00000188596	CFAP54	613.6683061	468.12053	759.2160822	0.699869688	0.093067441	7.52002718	5.48E-14	3.84E-13
ENSG00000124549	BTN2A3P	190.3443742	145.21597	235.4727803	0.699037271	0.162437706	4.30341754	1.68E-05	6.35E-05
ENSG00000162512	SDC3	3248.735105	2477.1906	4020.279599	0.69875146	0.041004614	17.0408009	4.09E-65	9.86E-64
ENSG00000184916	JAG2	5225.76017	3984.4745	6467.045832	0.698400757	0.037421272	18.6632021	9.86E-78	2.84E-76
ENSG00000130821	SLC6A8	2241.036584	1709.8401	2772.233042	0.69813739	0.054079629	12.9094338	3.98E-38	5.96E-37
ENSG00000198792	TMEM184B	7332.420303	5591.9165	9072.924085	0.698115855	0.033098583	21.0920164	9.42E-99	3.52E-97
ENSG00000005302	MSL3	2738.627201	2091.7451	3385.509312	0.695094296	0.046218877	15.0391862	4.07E-51	7.80E-50
ENSG00000109079	TNFAIP1	6043.980555	4615.9356	7472.025492	0.694370953	0.036364875	19.0945508	2.80E-81	8.63E-80
ENSG00000112183	RBM24	184.9416252	141.58186	228.3013943	0.694353397	0.185408108	3.74500017	0.00018	0.000599
ENSG00000103335	PIEZO1	25841.35036	19740.534	31942.16674	0.694266006	0.029112982	23.8472998	1.08E-125	4.84E-124
ENSG00000197496	SLC2A10	908.2393587	693.60475	1122.873963	0.694194637	0.073545015	9.43904405	3.76E-21	3.54E-20
ENSG00000119681	LTBP2	42710.19144	32670.988	52749.39513	0.691206053	0.02467749	28.0095774	1.24E-172	7.80E-171
ENSG00000095951	HIVEP1	2541.45012	1946.3228	3136.577399	0.689945795	0.050045038	13.7864976	3.07E-43	5.12E-42
ENSG00000139112	GABARAPL1	2893.392394	2214.5754	3572.209366	0.689786082	0.043986849	15.6816435	2.02E-55	4.13E-54
ENSG00000140941	MAP1LC3B	6212.401943	4758.0579	7666.745952	0.688110147	0.032821448	20.9652584	1.36E-97	5.01E-96
ENSG00000126705	AHDC1	1773.55413	1359.1033	2188.004959	0.687980293	0.0581395	11.8332682	2.63E-32	3.43E-31
ENSG00000178026	LRRC75B	131.7364238	100.90687	162.5659773	0.687973744	0.189100635	3.63813555	0.000275	0.000888
ENSG00000157657	ZNF618	6562.209928	5027.6932	8096.726691	0.687717036	0.032033838	21.468456	3.07E-102	1.18E-100
ENSG00000011028	AC080038.1	8890.839301	6810.2648	10971.41382	0.687127414	0.036497628	18.8266322	4.57E-79	1.34E-77
ENSG00000135063	FAM189A2	194.5717407	149.44739	239.6960922	0.686867289	0.161173645	4.26166008	2.03E-05	7.60E-05
ENSG00000269858	EGLN2	175.4178229	134.3993	216.436341	0.686611761	0.1785514	3.84545717	0.00012	0.000409
ENSG00000125089	SH3TC1	5418.749903	4153.0803	6684.419512	0.686069342	0.037097706	18.4935784	2.33E-76	6.53E-75
ENSG00000155304	HSPA13	3592.960886	2755.9943	4429.927447	0.685871308	0.043804305	15.6576234	2.95E-55	6.01E-54
ENSG00000151835	SACS	8162.453492	6261.5785	10063.3285	0.684668776	0.035387841	19.3475715	2.14E-83	6.74E-82
ENSG00000262001	DLGAP1-AS2	273.3068865	209.92562	336.688153	0.684384219	0.139688433	4.89936214	9.61E-07	4.12E-06

ENSG00000104611	SH2D4A	135.8817718	104.22844	167.5351057	0.684236663	0.180889792	3.78261624	0.000155	0.00052
ENSG00000144935	TRPC1	188.6876253	144.5731	232.8021503	0.684044698	0.168044211	4.0706234	4.69E-05	0.000168
ENSG00000115594	IL1R1	778.471557	597.76086	959.1822575	0.683645621	0.086256072	7.92576803	2.27E-15	1.69E-14
ENSG00000104774	MAN2B1	2933.484909	2250.2234	3616.74646	0.683258803	0.052070407	13.1218255	2.47E-39	3.82E-38
ENSG00000170581	STAT2	2298.388524	1764.3721	2832.404975	0.683165408	0.054347452	12.57033	3.07E-36	4.36E-35
ENSG00000185477	GPRIN3	386.8617996	296.73612	476.9874755	0.683116469	0.109417189	6.24322807	4.29E-10	2.40E-09
ENSG00000162614	NEXN	1180.276132	906.41333	1454.138937	0.681071703	0.072204946	9.4324799	4.01E-21	3.77E-20
ENSG00000120889	TNFRSF10B	17339.76362	13322.307	21357.22011	0.680921495	0.026921356	25.2929863	3.82E-141	1.94E-139
ENSG00000204516	MICB	1884.385875	1448.6736	2320.098149	0.679674894	0.051976362	13.0766154	4.48E-39	6.85E-38
ENSG00000204267	TAP2	1190.273347	915.22448	1465.322214	0.679137207	0.064382362	10.5484979	5.16E-26	5.75E-25
ENSG00000177679	SRRM3	179.8351988	138.13402	221.5363755	0.678065541	0.166093512	4.08243244	4.46E-05	0.00016
ENSG00000204262	COL5A2	29510.11437	22698.676	36321.55315	0.677951502	0.02435556	27.8355951	1.61E-170	9.97E-169
ENSG00000092964	DPYSL2	8178.767607	6294.4381	10063.09708	0.677362909	0.034790224	19.4699209	1.98E-84	6.35E-83
ENSG00000158710	TAGLN2	27168.50406	20905.109	33431.89924	0.677252565	0.021280096	31.8256355	2.86E-222	2.38E-220
ENSG00000205423	CNEP1R1	670.5193272	516.40998	824.6286705	0.677169858	0.091456317	7.4042984	1.32E-13	9.09E-13
ENSG00000166833	NAV2	4435.93151	3415.0174	5456.845633	0.67695692	0.039795548	17.0108705	6.82E-65	1.63E-63
ENSG00000228495	LINC01013	1885.065966	1450.6079	2319.524058	0.676774984	0.052343427	12.9295123	3.07E-38	4.61E-37
ENSG00000167378	IRGQ	1737.916464	1338.4253	2137.407595	0.676551795	0.062517583	10.8217843	2.71E-27	3.11E-26
ENSG00000148803	FUOM	317.3347389	244.22628	390.4432018	0.675190489	0.129793417	5.20203955	1.97E-07	9.00E-07
ENSG00000008513	ST3GAL1	4725.938793	3640.0321	5811.845519	0.674694251	0.034834186	19.368739	1.42E-83	4.49E-82
ENSG00000107130	NCS1	3293.057764	2537.8766	4048.238954	0.67371448	0.043166612	15.6073051	6.49E-55	1.32E-53
ENSG00000126822	PLEKHG3	243.6028122	188.05321	299.1524133	0.672781652	0.14526259	4.63148601	3.63E-06	1.47E-05
ENSG00000119729	RHOQ	1756.321155	1354.7112	2157.931085	0.672330306	0.053292487	12.6158554	1.73E-36	2.46E-35
ENSG00000081087	OSTM1	2120.470846	1636.2575	2604.684153	0.672214849	0.053760081	12.5039777	7.10E-36	1.00E-34
ENSG00000231365	WARS2-AS1	157.4136863	121.40503	193.4223459	0.671873779	0.173169784	3.87985574	0.000105	0.000359
ENSG00000164741	DLC1	6309.503462	4868.9989	7750.008033	0.670844332	0.035205612	19.0550395	5.97E-81	1.82E-79
ENSG00000103241	FOXF1	124.8384462	96.194562	153.4823301	0.669047688	0.19419295	3.44527281	0.00057	0.00176
ENSG00000271605	MILR1	56.71773306	43.756463	69.67900329	0.668933495	0.282251173	2.36999368	0.017788	0.040645
ENSG00000145242	EPHA5	80.79775301	62.224529	99.3709775	0.668599579	0.251048981	2.66322363	0.00774	0.019241
ENSG00000259865	AL390728.6	87.5647821	67.797421	107.3321432	0.667660535	0.254643387	2.62194335	0.008743	0.021507
ENSG00000109436	TBC1D9	4684.993221	3620.6673	5749.319111	0.667367559	0.038269504	17.4386259	4.20E-68	1.05E-66
ENSG00000167615	LENG8	3152.768972	2436.4666	3869.071373	0.667236079	0.141430276	4.717774	2.38E-06	9.84E-06
ENSG00000162434	JAK1	25722.70648	19888.968	31556.44537	0.666292298	0.025244478	26.3935856	1.62E-153	9.16E-152
ENSG00000100100	PIK3IP1	861.8202306	666.75337	1056.88709	0.665531392	0.075724906	8.78880449	1.51E-18	1.28E-17
ENSG00000272476	AL024507.2	223.723257	172.88998	274.5565369	0.665295859	0.167374397	3.97489623	7.04E-05	0.000247
ENSG00000101850	GPR143	384.3837772	297.52819	471.2393594	0.664561774	0.128053834	5.18970617	2.11E-07	9.59E-07
ENSG00000162298	SYVN1	2113.166067	1635.9121	2590.420048	0.664152192	0.053186985	12.4871186	8.78E-36	1.23E-34
ENSG00000164124	TMEM144	161.3470719	124.84438	197.849767	0.663317495	0.195698076	3.3894942	0.0007	0.002126
ENSG00000176871	WSB2	3927.25254	3040.0208	4814.484289	0.663216279	0.041252811	16.0768748	3.71E-58	7.99E-57
ENSG00000186866	POFUT2	2785.567196	2156.6668	3414.467572	0.662976474	0.046933679	14.125815	2.63E-45	4.56E-44
ENSG00000135503	ACVR1B	2043.443708	1581.9052	2504.982196	0.662315261	0.053873886	12.2938089	9.78E-35	1.34E-33
ENSG00000107796	ACTA2	234.7984705	182.12736	287.4695816	0.661884726	0.159215583	4.15716047	3.22E-05	0.000118
ENSG00000088881	EBF4	162.8161237	125.94014	199.6921091	0.66181156	0.167807815	3.94386613	8.02E-05	0.000278
ENSG00000157800	SLC37A3	1765.541879	1367.6923	2163.391473	0.661686577	0.056936017	11.6215817	3.20E-31	4.09E-30
ENSG00000134954	ETS1	8129.326658	6300.7104	9957.942944	0.661052078	0.032618441	20.2662073	2.56E-91	8.95E-90
ENSG00000151240	DIP2C	722.6309113	560.17019	885.0916289	0.660975975	0.093397344	7.07703182	1.47E-12	9.62E-12
ENSG00000009830	POMT2	1543.534242	1195.8506	1891.217883	0.660734054	0.059232492	11.1549257	6.77E-29	8.20E-28
ENSG00000113070	HBEGF	1159.095741	898.62097	1419.57051	0.659518869	0.066915239	9.856034	6.45E-23	6.47E-22
ENSG00000166225	FRS2	691.0459552	535.96181	846.1300983	0.65941941	0.087751999	7.51457992	5.71E-14	4.00E-13
ENSG00000158411	MITD1	588.3773468	456.50642	720.2482692	0.658089735	0.096023039	6.85345662	7.21E-12	4.54E-11
ENSG00000049130	KITLG	1542.412354	1197.3809	1887.443811	0.658032924	0.059305849	11.0955823	1.32E-28	1.57E-27
ENSG00000137831	UACA	9681.727288	7513.8715	11849.5831	0.657528904	0.032328148	20.3392075	5.79E-92	2.04E-90
ENSG00000069020	MAST4	7010.237116	5441.1848	8579.28945	0.657476192	0.033629385	19.5506457	4.07E-85	1.33E-83
ENSG00000162924	REL	683.5276018	531.08763	835.9675727	0.657213195	0.090229149	7.28382351	3.24E-13	2.19E-12
ENSG00000144655	CSRNP1	952.028058	738.3956	1165.660513	0.657151288	0.077457956	8.48397399	2.18E-17	1.77E-16
ENSG00000142303	ADAMTS10	156.2998633	121.38384	191.2158883	0.656479222	0.170429197	3.8519176	0.000117	0.000399
ENSG00000083799	CYLD	1795.069841	1394.4655	2195.674209	0.656290407	0.055389471	11.8486492	2.19E-32	2.86E-31
ENSG00000130881	LRP3	793.1136494	616.15981	970.0674923	0.655016052	0.077771866	8.42227515	3.69E-17	2.97E-16
ENSG00000276547	PCDHGB5	572.9046303	444.83822	700.971037	0.654411352	0.092240494	7.0946211	1.30E-12	8.49E-12
ENSG00000172432	GTPBP2	2329.661535	1809.4226	2849.900446	0.653954791	0.05546036	11.791391	4.32E-32	5.61E-31
ENSG00000013392	RWDD2A	377.3968638	293.37274	461.4209829	0.652983498	0.117724174	5.54672396	2.91E-08	1.43E-07
ENSG00000112276	BVES	692.6410034	538.92498	846.3570311	0.651083373	0.097955659	6.64671526	3.00E-11	1.81E-10
ENSG00000017260	ATP2C1	5767.375053	4488.6535	7046.09657	0.650995834	0.03695394	17.616412	1.84E-69	4.68E-68
ENSG00000152760	TCTEX1D1	130.9906653	102.14289	159.838437	0.649576049	0.192816805	3.36887673	0.000755	0.00228
ENSG00000232759	AC002480.1	348.3364068	271.06614	425.6066756	0.648654435	0.119227396	5.44048144	5.31E-08	2.55E-07
ENSG00000138600	SPPL2A	3057.615997	2382.0368	3733.195179	0.648532748	0.045860388	14.1414578	2.11E-45	3.66E-44
ENSG00000039560	RAI14	20321.37538	15828.55	24814.20034	0.648468391	0.022988375	28.2085359	4.60E-175	2.93E-173
ENSG00000187566	NHLRC1	82.88982785	64.482674	101.2969814	0.647404227	0.231881358	2.79196324	0.005239	0.01351
ENSG00000163328	GPR155	134.987672	105.16218	164.8131602	0.647113391	0.195387559	3.31194777	0.000926	0.002756
ENSG00000109814	UGDH	2284.446425	1781.3148	2787.578092	0.646520093	0.052403316	12.3373889	5.70E-35	7.84E-34
ENSG00000260398	AC068700.1	63.8988638	49.926929	77.87079874	0.645774862	0.268853139	2.4019614	0.016307	0.037697
ENSG00000119048	UBE2B	1863.052307	1453.3875	2272.71715	0.645632427	0.054535464	11.8387629	2.46E-32	3.22E-31
ENSG00000171992	SYNPO	17214.95973	13435.152	20994.76739	0.644137625	0.030565326	21.0741292	1.37E-98	5.13E-97
ENSG00000119771	KLHL29	767.2929355	598.7116	935.8742706	0.644023126	0.081275004	7.92399996	2.30E-15	1.71E-14
ENSG00000132274	TRIM22	6331.790495	4943.244	7720.336996	0.64368155	0.031846036	20.2122972	7.63E-91	2.64E-89
ENSG00000115295	CLIP4	1066.531332	832.70776	1300.354903	0.643386355	0.078543466	8.19146887	2.58E-16	2.00E-15
ENSG00000149115	TNKS1BP1	10536.23394	8223.2492	12849.21869	0.643343099	0.031472917	20.4411653	7.20E-93	2.56E-91
ENSG00000181104	F2R	38084.02466	29740.434	46427.61534	0.6427303	0.025552154	25.1536644	1.29E-139	6.40E-138
ENSG00000158669	GPAT4	4918.205324	3841.6034	5994.807276	0.642517527	0.035273631	18.2152363	3.91E-74	1.05E-72
ENSG00000122694	GLIPR2	1160.401654	905.64919	1415.154114	0.642189272	0.066561136	9.64811172	5.01E-22	4.87E-21
ENSG00000138835	RGS3	12488.40166	9753.5246	15223.27872	0.642135387	0.025328629	25.3521571	8.51E-142	4.33E-140
ENSG00000007384	RHBDF1	1791.193828	1399.409	2182.978622	0.641768465	0.059329718	10.8169816	2.86E-27	3.28E-26
ENSG00000162604	TM2D1	661.3697385	516.43259	806.3068917	0.641367432	0.088664997	7.23360348	4.70E-13	3.15E-12
ENSG00000231389	HLA-DPA1	158.1626839	123.78011	192.5452605	0.640390689	0.169765868	3.77219929	0.000162	0.000542
ENSG00000130962	PRRG1	1864.911892	1458.8553	2270.968466	0.63966787	0.061065317	10.4751421	1.12E-25	1.24E-24

ENSG00000185737	NRG3	545.2767389	426.92804	663.6254357	0.638576759	0.099738297	6.40252319	1.53E-10	8.83E-10
ENSG00000144026	ZNF514	224.8661445	175.96667	273.7656228	0.638179566	0.151954099	4.19981804	2.67E-05	9.87E-05
ENSG00000131634	TMEM204	1394.524853	1090.4144	1698.635346	0.638074323	0.063615378	10.0301899	1.12E-23	1.16E-22
ENSG00000165684	SNAPC4	1469.525683	1150.3369	1788.714463	0.637844787	0.058187648	10.9618589	5.83E-28	6.84E-27
ENSG00000273311	DGCR11	141.8701158	110.91436	172.8258752	0.637695009	0.18031038	3.53665168	0.000405	0.001276
ENSG00000156313	RPGR	1204.547057	942.69836	1466.395758	0.637044985	0.065715421	9.69399527	3.20E-22	3.13E-21
ENSG00000150048	CLEC1A	566.791181	444.04863	689.5337331	0.636937036	0.092766087	6.86605475	6.60E-12	4.16E-11
ENSG00000115652	UXS1	2348.257868	1839.1796	2857.336165	0.636896092	0.049874706	12.7699216	2.41E-37	3.52E-36
ENSG00000185621	LMLN	581.4589315	455.8081	707.1097597	0.635822925	0.099541906	6.38748998	1.69E-10	9.72E-10
ENSG00000166579	NDEL1	2207.203703	1728.6651	2685.742263	0.63535585	0.048853822	13.0052436	1.14E-38	1.74E-37
ENSG00000241764	AC002467.1	61.75222074	48.313576	75.19086544	0.633438874	0.270022713	2.34587256	0.018983	0.043079
ENSG00000198786	MT-ND5	93917.00665	73631.004	114203.0089	0.633219805	0.089901048	7.04351973	1.87E-12	1.22E-11
ENSG00000187678	SPRY4	1027.425727	805.78173	1249.069723	0.632930794	0.067830872	9.33101363	1.05E-20	9.68E-20
ENSG00000139636	LMBR1L	1845.765121	1446.9186	2244.611599	0.632876975	0.057199531	11.0643735	1.87E-28	2.22E-27
ENSG00000175544	CABP4	71.16944186	55.815334	86.52354973	0.632777125	0.260189294	2.43198755	0.015016	0.035066
ENSG00000196628	TCF4	16974.98276	13316.358	20633.60798	0.632085492	0.024729291	25.560195	4.23E-144	2.19E-142
ENSG00000172057	ORMDL3	1242.907618	974.16687	1511.64837	0.631961289	0.066521331	9.50012997	2.10E-21	2.00E-20
ENSG00000260267	AC026471.1	178.739607	140.25101	217.2282087	0.631954459	0.177018125	3.5699986	0.000357	0.001134
ENSG00000242802	AP5Z1	2324.834117	1823.8681	2825.800097	0.631497842	0.046531546	13.5713917	5.92E-42	9.67E-41
ENSG00000183891	TTC32	89.46252405	70.139578	108.7854696	0.631396144	0.236278642	2.6722523	0.007534	0.018781
ENSG00000166710	B2M	32176.34145	25256.471	39096.21226	0.630565675	0.024180014	26.0779701	6.48E-150	3.55E-148
ENSG00000166446	CDYL2	3301.433823	2592.991	4009.876635	0.630260005	0.045408915	13.8796535	8.41E-44	1.41E-42
ENSG00000188092	GPR89B	80.15010155	62.952733	97.34746972	0.629736054	0.240625671	2.6170776	0.008869	0.021785
ENSG00000119682	AREL1	3257.418445	2558.1977	3956.639159	0.628856059	0.046996845	13.3808144	7.83E-41	1.25E-39
ENSG00000165915	SLC39A13	4353.201113	3419.0818	5287.320386	0.628537349	0.036709663	17.1218503	1.02E-65	2.47E-64
ENSG00000127191	TRAF2	722.7831171	567.18663	878.3796074	0.628091404	0.087469823	7.18066393	6.94E-13	4.61E-12
ENSG00000165806	CASP7	2603.313133	2046.9988	3159.627511	0.627290511	0.046400594	13.519019	1.21E-41	1.96E-40
ENSG00000183060	LYSMD4	278.336266	218.7838	337.8887337	0.626993738	0.131633194	4.76318866	1.91E-06	7.94E-06
ENSG00000083857	FAT1	800.3350385	628.95955	971.7105243	0.626468339	0.080378455	7.79398332	6.49E-15	4.77E-14
ENSG00000164877	MICALL2	1200.172005	943.27944	1457.064568	0.626271668	0.064261808	9.74562793	1.93E-22	1.90E-21
ENSG00000181638	ZFP41	258.9531666	203.57211	314.3342246	0.625768597	0.132081447	4.73774789	2.16E-06	8.97E-06
ENSG00000184867	ARMCX2	2835.575637	2229.8911	3441.260147	0.625279762	0.043694756	14.3101785	1.89E-46	3.35E-45
ENSG00000100441	KHNYN	4040.270975	3179.8797	4900.662224	0.623724049	0.039273769	15.881441	8.52E-57	1.78E-55
ENSG00000010404	IDS	396.124538	311.7692	480.4798716	0.623535609	0.107690771	5.79005615	7.04E-09	3.62E-08
ENSG00000119699	TGFB3	156.2992643	123.13606	189.4624723	0.623148473	0.172238137	3.61794713	0.000297	0.000955
ENSG00000103404	USP31	2073.284251	1632.6779	2513.890577	0.622258071	0.052982336	11.7446327	7.52E-32	9.75E-31
ENSG00000143702	CEP170	10578.60691	8332.4636	12824.75624	0.622200899	0.027212963	22.8641367	1.06E-115	4.37E-114
ENSG00000033178	UBA6	3813.631382	3004.6129	4622.649861	0.621758419	0.042046914	14.7872545	1.77E-49	3.29E-48
ENSG00000271425	NBPF10	128.6864191	101.52348	155.8493611	0.621243329	0.189469751	3.2788523	0.001042	0.003074
ENSG00000132475	H3F3B	11270.65742	8880.6706	13660.64422	0.621012259	0.028963861	21.440935	5.55E-102	2.13E-100
ENSG00000176915	ANKLE2	4600.824997	3625.316	5576.334008	0.620901794	0.03852824	16.1154985	1.99E-58	4.29E-57
ENSG00000090674	MCOLN1	1177.204408	927.20331	1427.205501	0.620401156	0.068959316	8.99662573	2.33E-19	2.04E-18
ENSG00000164161	HHIP	71140.07508	56093.084	86187.06626	0.619755099	0.020644585	30.0202259	5.34E-198	3.95E-196
ENSG00000068971	PPP2R5B	1104.051769	870.38005	1337.723489	0.619034546	0.068503986	9.03647482	1.62E-19	1.43E-18
ENSG00000168917	SLC35G2	561.959043	443.17682	680.7412643	0.617885592	0.092512282	6.67895738	2.41E-11	1.47E-10
ENSG00000116852	KIF21B	513.6681927	405.35989	621.9764963	0.617247908	0.100388828	6.14857171	7.82E-10	4.30E-09
ENSG00000167657	DAPK3	5513.421514	4351.8108	6675.032268	0.617169373	0.034769013	17.7505578	1.71E-70	4.40E-69
ENSG00000099968	BCL2L13	2775.150479	2190.1102	3360.19071	0.616449015	0.047005699	13.1143462	2.73E-39	4.20E-38
ENSG00000196449	YRDC	752.462293	594.97547	909.9491158	0.615294654	0.091720725	6.70834926	1.97E-11	1.20E-10
ENSG00000158985	CDC42SE2	1571.613827	1242.2443	1900.983362	0.615239233	0.064892867	9.48084528	2.52E-21	2.39E-20
ENSG00000150540	HNMT	303.216914	239.58119	366.8526351	0.614785747	0.128344946	4.79010482	1.67E-06	6.99E-06
ENSG00000197608	ZNF841	370.6777643	293.23561	448.119917	0.614596272	0.118250429	5.19741261	2.02E-07	9.21E-07
ENSG00000090975	PITPNM2	1121.966683	886.51721	1357.416152	0.613391179	0.067244155	9.12185129	7.39E-20	6.62E-19
ENSG00000113583	C5orf15	1730.322966	1369.2157	2091.430224	0.613324192	0.066508845	9.2216936	2.92E-20	2.66E-19
ENSG00000134352	IL6ST	49762.74768	39350.696	60174.79948	0.612951673	0.02970672	20.6334351	1.38E-94	4.98E-93
ENSG00000109089	CDR2L	2349.738856	1857.8524	2841.625263	0.612283347	0.051035805	11.997133	3.68E-33	4.89E-32
ENSG00000040487	PQLC2	676.378831	534.87084	817.8868236	0.61215386	0.088499649	6.91702017	4.61E-12	2.93E-11
ENSG00000182240	BACE2	12044.81782	9529.8004	14559.83523	0.611911478	0.028391697	21.5524802	5.02E-103	1.95E-101
ENSG00000070540	WIPI1	1946.593911	1541.0021	2352.185728	0.611807383	0.059298978	10.3173344	5.88E-25	6.32E-24
ENSG00000179454	KLHL28	386.756602	306.60026	466.912946	0.611671743	0.137225619	4.45741652	8.30E-06	3.23E-05
ENSG00000113580	NR3C1	1891.079814	1496.4117	2285.747899	0.611062319	0.054952035	11.1199216	1.00E-28	1.20E-27
ENSG00000120686	UFM1	2606.192453	2062.9538	3149.431077	0.610762898	0.048851703	12.5023869	7.24E-36	1.02E-34
ENSG00000217128	FNIP1	1409.824303	1116.3992	1703.249398	0.610661565	0.058999248	10.350328	4.17E-25	4.51E-24
ENSG00000157600	TMEM164	1991.785584	1576.0312	2407.539934	0.610504244	0.053207532	11.4740192	1.78E-30	2.25E-29
ENSG00000117226	GBP3	1658.696113	1312.9884	2004.403859	0.610101471	0.058942675	10.3507598	4.15E-25	4.49E-24
ENSG00000114480	GBE1	5145.315761	4072.592	6218.039517	0.610098946	0.037098287	16.4454747	9.04E-61	2.02E-59
ENSG00000170681	CAVIN4	112.6600521	89.162912	136.1571922	0.609987015	0.199119287	3.06342507	0.002188	0.006113
ENSG00000205730	ITPRIPL2	3401.192113	2693.7793	4108.604924	0.609925893	0.045347214	13.4501295	3.07E-41	4.94E-40
ENSG00000142102	PGGHG	1164.224355	921.71311	1406.735599	0.609866057	0.077373763	7.88207825	3.22E-15	2.39E-14
ENSG00000171729	TMEM51	1034.642507	819.5046	1249.780412	0.608664809	0.074168468	8.20651724	2.28E-16	1.77E-15
ENSG00000100376	FAM118A	459.4778693	364.00917	554.9465715	0.608533089	0.110368304	5.51365807	3.51E-08	1.71E-07
ENSG00000213420	GPC2	143.1108258	113.26662	172.9550294	0.607382688	0.178966076	3.39384258	0.000689	0.002096
ENSG00000230257	NFE4	131.3624958	104.01238	158.7126097	0.607265311	0.204914652	2.96350362	0.003042	0.00824
ENSG00000167904	TMEM68	403.273413	319.54025	487.0065805	0.606923882	0.108397664	5.59904945	2.16E-08	1.07E-07
ENSG00000107249	GLIS3	365.9690938	290.16041	441.777781	0.605854719	0.11717982	5.17029911	2.34E-07	1.06E-06
ENSG00000183087	GAS6	4977.546492	3947.0798	6008.013198	0.605321907	0.045625042	13.2673173	3.58E-40	5.63E-39
ENSG00000136888	ATP6V1G1	3408.752777	2705.0625	4112.443019	0.604603542	0.046910915	12.888334	5.24E-38	7.80E-37
ENSG00000176788	BASP1	5617.162181	4457.6856	6776.63873	0.604235062	0.090328445	6.68931101	2.24E-11	1.37E-10
ENSG00000113971	NPHP3	155.8707446	123.89434	187.847151	0.604157737	0.175615375	3.4402326	0.000581	0.001792
ENSG00000112210	RAB23	820.1057603	650.81489	989.3966308	0.604080135	0.077771947	7.76732688	8.02E-15	5.86E-14
ENSG00000161638	ITGA5	135274.8551	107395.49	163154.2237	0.603313152	0.019436916	31.039551	1.58E-211	1.24E-209
ENSG00000132109	TRIM21	1117.62659	887.06403	1348.189146	0.602917517	0.067077709	8.98834397	2.51E-19	2.19E-18
ENSG00000203930	LINC00632	109.5881252	87.32898	131.8472707	0.601225461	0.231198698	2.60047078	0.00931	0.022765
ENSG00000163932	PRKCD	885.0454127	702.95013	1067.140697	0.600502057	0.081262561	7.38965217	1.47E-13	1.01E-12
ENSG00000187231	SESTD1	2454.872219	1951.0606	2958.683827	0.600460204	0.047237141	12.7116118	5.10E-37	7.37E-36

ENSG00000187961	KLHL17	363.9723622	289.3021	438.6426215	0.600175849	0.13558488	4.42656918	9.57E-06	3.71E-05
ENSG00000175416	CLTB	3312.109259	2632.2657	3991.952791	0.600032373	0.04677704	12.8274977	1.15E-37	1.70E-36
ENSG00000112242	E2F3	1591.785386	1265.4458	1918.124939	0.600016265	0.056735006	10.5757682	3.86E-26	4.30E-25
ENSG00000165246	NLGN4Y	1598.678479	1272.1292	1925.227731	0.599084147	0.058980553	10.1573165	3.07E-24	3.23E-23
ENSG00000114374	USP9Y	1270.108443	1010.9507	1529.266145	0.59887812	0.063783262	9.38926768	6.04E-21	5.64E-20
ENSG00000204681	GABBR1	508.384867	404.35701	612.4127226	0.598280107	0.113053895	5.2919902	1.21E-07	5.63E-07
ENSG00000178031	ADAMTSL1	3252.713706	2587.9202	3917.507239	0.597617049	0.041058604	14.555221	5.41E-48	9.86E-47
ENSG00000143322	ABL2	12561.42631	9998.3902	15124.4624	0.597589245	0.028169119	21.2143394	7.04E-100	2.65E-98
ENSG00000136048	DRAM1	2634.042437	2096.9226	3171.162319	0.59748207	0.046244924	12.9199491	3.47E-38	5.21E-37
ENSG00000174695	TMEM167A	4029.21862	3207.9702	4850.46706	0.597409338	0.04409884	13.5470533	8.25E-42	1.34E-40
ENSG00000213859	KCTD11	399.5713097	317.67373	481.468894	0.597131211	0.112327208	5.31599801	1.06E-07	4.96E-07
ENSG00000173083	HPSE	1374.969909	1095.0928	1654.84706	0.597082954	0.062516255	9.55084333	1.29E-21	1.23E-20
ENSG00000104825	NFKBIB	1218.770176	970.09663	1467.44372	0.596260439	0.063701245	9.36026353	7.95E-21	7.40E-20
ENSG00000267107	PCAT19	1108.937204	883.88847	1333.985933	0.596228713	0.077063388	7.73686085	1.02E-14	7.42E-14
ENSG00000198324	PHETA1	957.7624063	761.7876	1153.737216	0.595919837	0.080535937	7.39942763	1.37E-13	9.42E-13
ENSG00000151208	DLG5	2085.441094	1660.0934	2510.788814	0.595599889	0.053075683	11.2217094	3.19E-29	3.89E-28
ENSG00000072201	LNX1	128.7379914	102.52556	154.950423	0.595361724	0.188004863	3.16673578	0.001542	0.004409
ENSG00000128039	SRD5A3	1170.410604	931.82862	1408.992583	0.59535371	0.065860176	9.03966167	1.57E-19	1.39E-18
ENSG00000131943	C19orf12	853.9761685	680.15612	1027.796222	0.595098529	0.08014461	7.42530942	1.13E-13	7.79E-13
ENSG00000277462	ZNF670	183.7109394	146.39625	221.0256254	0.594659191	0.15455529	3.8475499	0.000119	0.000406
ENSG00000184208	C22orf46	331.9313164	264.47775	399.3848821	0.594197116	0.117698004	5.04848931	4.45E-07	1.97E-06
ENSG00000152952	PLOD2	15233.45254	12143.338	18323.56706	0.593672913	0.024250241	24.4811137	2.35E-132	1.11E-130
ENSG00000131979	GCH1	204.9728124	163.36149	246.5841381	0.593596318	0.155294487	3.82239143	0.000132	0.000447
ENSG00000255769	GOLGA2P10	226.4966131	180.2015	272.7917296	0.593445128	0.148781923	3.98869107	6.64E-05	0.000234
ENSG00000099992	TBC1D10A	204.2736489	162.8613	245.6859955	0.593237243	0.146930064	4.03754837	5.40E-05	0.000192
ENSG00000170113	NIPA1	1535.996168	1225.4189	1846.573406	0.593233693	0.059664282	9.94286159	2.71E-23	2.75E-22
ENSG00000112137	PHACTR1	565.8339126	451.40546	680.2623661	0.592784973	0.09145097	6.48199767	9.05E-11	5.31E-10
ENSG00000117151	CTBS	565.6668529	451.16044	680.1732677	0.592344213	0.096987937	6.1074009	1.01E-09	5.53E-09
ENSG00000145860	RNF145	5330.368335	4251.2238	6409.512833	0.592275559	0.034986088	16.9288876	2.76E-64	6.54E-63
ENSG00000157483	MYO1E	4705.026698	3753.5198	5656.533557	0.592001103	0.035108202	16.8621878	8.54E-64	2.00E-62
ENSG00000250317	SMIM20	385.3507444	306.95648	463.7450045	0.591403935	0.127472011	4.63948071	3.49E-06	1.42E-05
ENSG00000177058	SLC38A9	745.092244	594.56562	895.6188637	0.591368786	0.082171922	7.19672574	6.17E-13	4.11E-12
ENSG00000077585	GPR137B	689.3543616	550.30657	828.4021491	0.591245711	0.092365704	6.40113898	1.54E-10	8.90E-10
ENSG00000164050	PLXNB1	1857.801725	1481.9091	2233.694383	0.590396672	0.055542884	10.6295646	2.17E-26	2.44E-25
ENSG00000100554	ATP6V1D	2343.059553	1869.914	2816.205135	0.59026005	0.047392033	12.4548372	1.32E-35	1.84E-34
ENSG00000196418	ZNF124	283.0102885	226.04153	339.9790516	0.588929018	0.148927414	3.95447017	7.67E-05	0.000267
ENSG00000184402	SS18L1	522.0331153	417.22373	626.8424966	0.588657389	0.097384184	6.04469193	1.50E-09	8.07E-09
ENSG00000120279	MYCT1	6407.861163	5119.8254	7695.896915	0.588541468	0.03556595	16.5478913	1.66E-61	3.75E-60
ENSG00000205808	PLPP6	357.5255687	285.5358	429.5153332	0.58767841	0.115536994	5.08649557	3.65E-07	1.63E-06
ENSG00000125968	ID1	1585.918367	1267.2754	1904.561368	0.587469121	0.064850687	9.05879566	1.32E-19	1.17E-18
ENSG00000137802	MAPKBP1	2059.516652	1645.9793	2473.053966	0.587085944	0.051956422	11.2995837	1.32E-29	1.62E-28
ENSG00000064225	ST3GAL6	599.9019652	480.05317	719.7507589	0.587081919	0.100950586	5.81553752	6.04E-09	3.13E-08
ENSG00000131584	ACAP3	1061.812348	849.42076	1274.203932	0.586167426	0.073375786	7.98856762	1.37E-15	1.03E-14
ENSG00000058799	YIPF1	685.7345678	548.25049	823.2186489	0.585903772	0.091519701	6.40194149	1.53E-10	8.86E-10
ENSG00000154813	DPH3	1913.758639	1529.9646	2297.552661	0.585814561	0.053481962	10.953498	6.39E-28	7.48E-27
ENSG00000178607	ERN1	1147.46501	917.85755	1377.072468	0.58533705	0.065296111	8.9643478	3.12E-19	2.71E-18
ENSG00000177674	AGTRAP	963.8778323	771.8194	1155.936262	0.584273423	0.071787923	8.1388818	3.99E-16	3.07E-15
ENSG00000180747	SMG1P3	97.43323237	78.122046	116.7444184	0.580827975	0.222161302	2.61444261	0.008937	0.021942
ENSG00000258484	SPESP1	133.2051101	106.75553	159.6546889	0.580798651	0.201684154	2.87974359	0.00398	0.010511
ENSG00000164574	GALNT10	9116.805912	7306.1632	10927.44863	0.580466903	0.028230351	20.5618027	6.03E-94	2.18E-92
ENSG00000106080	FKBP14	3078.97182	2469.4036	3688.540008	0.579504821	0.041884209	13.8358784	1.55E-43	2.59E-42
ENSG00000232533	AC093673.1	196.7756506	157.58535	235.9659506	0.579324411	0.155043949	3.73651739	0.000187	0.000618
ENSG00000158079	PTPDC1	349.4175157	279.90412	418.9309134	0.579130684	0.121189792	4.77870847	1.76E-06	7.38E-06
ENSG00000173905	GOLIM4	5668.56132	4545.1086	6792.014087	0.579053474	0.033513018	17.2784639	6.83E-67	1.68E-65
ENSG00000134851	TMEM165	4552.449227	3651.9006	5452.997874	0.578698589	0.050920459	11.364756	6.26E-30	7.76E-29
ENSG00000140545	MFGE8	2828.793538	2268.9456	3388.641492	0.57844155	0.047171959	12.2624026	1.44E-34	1.97E-33
ENSG00000242732	RTL5	188.1762582	150.83488	225.5176397	0.5783453	0.156806182	3.68828125	0.000226	0.000739
ENSG00000072401	UBE2D1	948.8168119	761.63198	1136.001642	0.576973962	0.072119784	8.00021762	1.24E-15	9.36E-15
ENSG00000158195	WASF2	15513.50728	12453.185	18573.82979	0.576820634	0.024163005	23.8720569	5.98E-126	2.70E-124
ENSG00000196083	IL1RAP	298.8461543	239.99712	357.6951909	0.576687725	0.122099246	4.72310636	2.32E-06	9.60E-06
ENSG00000160345	C9orf116	71.5754819	57.526253	85.62471107	0.57654091	0.249263585	2.31297688	0.020724	0.046622
ENSG00000155975	VPS37A	1455.577887	1168.9728	1742.182941	0.576264285	0.060528698	9.5205134	1.72E-21	1.64E-20
ENSG00000117475	BLZF1	1080.48697	867.53744	1293.436497	0.576235045	0.065055878	8.85754006	8.18E-19	7.02E-18
ENSG00000165175	MID1IP1	963.6142156	773.6583	1153.570132	0.575647712	0.08497178	6.77457519	1.25E-11	7.71E-11
ENSG00000186575	NF2	4422.723096	3552.0143	5293.431863	0.575450882	0.036925444	15.5841292	9.33E-55	1.89E-53
ENSG00000137193	PIM1	252.6342127	202.68533	302.5830913	0.575023529	0.137630815	4.17801442	2.94E-05	0.000108
ENSG00000034677	RNF19A	2960.46317	2378.6906	3542.235745	0.574768185	0.04352256	13.2062126	8.08E-40	1.26E-38
ENSG00000163482	STK36	598.0192359	480.59276	715.4457097	0.57386154	0.101999317	5.62613122	1.84E-08	9.17E-08
ENSG00000163605	PPP4R2	2838.477813	2281.9673	3394.988281	0.573243787	0.046912851	12.2193339	2.45E-34	3.32E-33
ENSG00000169084	DHRSX	674.6535619	542.88151	806.425611	0.572965606	0.089241621	6.42038543	1.36E-10	7.89E-10
ENSG00000070882	OSBPL3	2084.555414	1675.9392	2493.171608	0.572896763	0.051445131	11.1360735	8.37E-29	1.01E-27
ENSG00000142166	IFNAR1	4801.326914	3862.2906	5740.363208	0.572738232	0.04029223	14.2146076	7.44E-46	1.30E-44
ENSG00000105497	ZNF175	777.8546562	625.76337	929.9459452	0.572268613	0.077621172	7.37258399	1.67E-13	1.15E-12
ENSG00000150630	VEGFC	1278.21168	1028.7292	1527.694129	0.570725567	0.061918117	9.21742453	3.04E-20	2.76E-19
ENSG00000198853	RUSC2	2391.37427	1925.2482	2857.500315	0.5703007	0.047282759	12.0614937	1.69E-33	2.25E-32
ENSG00000143801	PSEN2	890.7574114	717.37666	1064.138163	0.570199363	0.072441955	7.87112061	3.51E-15	2.60E-14
ENSG00000154237	LRRK1	2274.777054	1831.9412	2717.612952	0.56979649	0.049763217	11.4501538	2.35E-30	2.95E-29
ENSG00000228594	FNDC10	82.56141305	66.507764	98.61506223	0.569631803	0.235412167	2.41972117	0.015532	0.036114
ENSG00000160953	PWWP3A	1946.677001	1566.7017	2326.652344	0.569306817	0.054464015	10.4528985	1.42E-25	1.56E-24
ENSG00000263711	AC079062.1	222.5365074	179.34457	265.7284433	0.569056078	0.141427457	4.02366055	5.73E-05	0.000203
ENSG00000072364	AFF4	8222.113748	6625.2082	9819.019285	0.568296932	0.031557536	18.0082798	1.68E-72	4.46E-71
ENSG00000161791	FMNL3	6922.678632	5578.41	8266.947252	0.567873812	0.031690363	17.9194478	8.31E-72	2.19E-70
ENSG00000135916	ITM2C	222.0865849	178.73134	265.4418304	0.567849059	0.143015225	3.97054971	7.17E-05	0.000251
ENSG00000196387	ZNF140	644.3855318	519.56736	769.2037007	0.567770146	0.092869716	6.11361993	9.74E-10	5.32E-09
ENSG00000134769	DTNA	710.1064331	572.29899	847.9138748	0.567674536	0.083697017	6.78249428	1.18E-11	7.31E-11

ENSG00000151327	FAM177A1	1288.986513	1039.1604	1538.812608	0.56643018	0.063981877	8.85297842	8.52E-19	7.29E-18
ENSG00000116675	DNAJC6	360.4307692	290.29434	430.5671968	0.566095561	0.114541354	4.94228104	7.72E-07	3.34E-06
ENSG00000129003	VPS13C	6107.171191	4925.6059	7288.736475	0.565950674	0.033931653	16.6791364	1.86E-62	4.29E-61
ENSG00000197217	ENTPD4	2784.772936	2247.2203	3322.325558	0.564423043	0.043936202	12.8464231	9.01E-38	1.33E-36
ENSG00000145979	TBC1D7	755.3426294	609.54523	901.1400291	0.564407374	0.081382965	6.93520291	4.06E-12	2.58E-11
ENSG00000129680	MAP7D3	1926.002977	1553.7687	2298.237251	0.564315345	0.058962236	9.57079274	1.06E-21	1.02E-20
ENSG00000163191	S100A11	6968.267238	5626.4916	8310.0429	0.563087424	0.035209517	15.992478	1.44E-57	3.04E-56
ENSG00000128590	DNAJB9	929.6461637	751.19624	1108.096092	0.563054532	0.077027309	7.30980401	2.68E-13	1.82E-12
ENSG00000164506	STXBP5	1889.781026	1526.7151	2252.846933	0.562308213	0.053682726	10.4746583	1.13E-25	1.24E-24
ENSG00000083520	DIS3	3268.27209	2640.6258	3895.918351	0.562054475	0.045953411	12.230963	2.12E-34	2.88E-33
ENSG00000131002	TXLNGY	508.8816897	411.16321	606.6001743	0.561744805	0.099560029	5.64227241	1.68E-08	8.39E-08
ENSG00000128335	APOL2	2473.929151	1997.5005	2950.357759	0.56164014	0.050830912	11.0491847	2.21E-28	2.62E-27
ENSG00000197321	SVIL	9127.665967	7373.9279	10881.40408	0.561169332	0.030702034	18.2779201	1.24E-74	3.36E-73
ENSG00000130813	C19orf66	1208.529476	976.04745	1441.0115	0.560913716	0.069270504	8.09743952	5.61E-16	4.29E-15
ENSG00000068724	TTC7A	692.0895682	558.77016	825.4089802	0.560872915	0.083020446	6.75584079	1.42E-11	8.74E-11
ENSG00000285410	GABPB1-IT1	122.3380023	98.730103	145.9459014	0.56067457	0.195902771	2.86200429	0.00421	0.011072
ENSG00000173559	NABP1	709.1669094	573.69399	844.6398279	0.560346177	0.092399593	6.06437928	1.32E-09	7.17E-09
ENSG00000159363	ATP13A2	3973.433086	3211.561	4735.305199	0.560196264	0.039405618	14.2161521	7.27E-46	1.28E-44
ENSG00000198355	PIM3	6700.056452	5416.2358	7983.877082	0.559657636	0.040092282	13.9592361	2.76E-44	4.70E-43
ENSG00000179627	AL590327.1	252.8036529	204.40133	301.2059804	0.559364459	0.134897388	4.14659223	3.37E-05	0.000123
ENSG00000174943	KCTD13	308.0064039	249.4343	366.5785101	0.558359295	0.144554487	3.86262166	0.000112	0.000383
ENSG00000050130	JKAMP	1343.201906	1087.6277	1598.77607	0.557397605	0.067361448	8.27472717	1.29E-16	1.01E-15
ENSG00000138018	SELENOI	1600.262812	1294.3371	1906.18851	0.557312422	0.057554462	9.68321841	3.55E-22	3.47E-21
ENSG00000154553	PDLIM3	485.1884426	392.82778	577.5491017	0.557292781	0.101439361	5.49385143	3.93E-08	1.91E-07
ENSG00000189420	ZFP92	206.066414	166.68305	245.4497737	0.557072545	0.158444875	3.51587606	0.000438	0.001372
ENSG00000140403	DNAJA4	819.2091837	663.70164	974.7167314	0.556350963	0.081905416	6.79260288	1.10E-11	6.83E-11
ENSG00000181751	C5orf30	435.8142828	352.60509	519.023474	0.556338162	0.104164758	5.34094424	9.25E-08	4.35E-07
ENSG00000107863	ARHGAP21	8050.700152	6519.7194	9581.680888	0.555973304	0.031528643	17.6339118	1.35E-69	3.45E-68
ENSG00000101842	VSIG1	130.1828537	105.42505	154.9406548	0.555802379	0.188001741	2.95636826	0.003113	0.008412
ENSG00000100345	MYH9	130083.2261	105346.79	154819.6628	0.555427843	0.019230371	28.8828462	1.96E-183	1.34E-181
ENSG00000143479	DYRK3	664.5756619	538.5615	790.5898222	0.555405143	0.086119536	6.44923522	1.12E-10	6.55E-10
ENSG00000170537	TMC7	544.548118	441.10351	647.9927297	0.555344354	0.093386003	5.94676219	2.73E-09	1.45E-08
ENSG00000083444	PLOD1	20390.14164	16518.48	24261.80301	0.554558264	0.022017289	25.1873994	5.51E-140	2.75E-138
ENSG00000167554	ZNF610	97.11031679	78.926629	115.2940049	0.554127933	0.226313451	2.44849756	0.014345	0.033643
ENSG00000185189	NRBP2	978.0460283	792.46095	1163.631109	0.554040225	0.070541776	7.85407255	4.03E-15	2.98E-14
ENSG00000018510	AGPS	5603.682723	4543.4304	6663.935049	0.552584335	0.037513459	14.7302954	4.12E-49	7.60E-48
ENSG00000138386	NAB1	1339.114978	1086.2546	1591.975391	0.552451208	0.063283843	8.72973545	2.55E-18	2.15E-17
ENSG00000155158	TTC39B	232.8797905	189.1067	276.6528796	0.552107589	0.144643921	3.81701204	0.000135	0.000456
ENSG00000069812	HES2	669.4113217	542.47994	796.3427083	0.552080814	0.083732905	6.59335555	4.30E-11	2.57E-10
ENSG00000188295	ZNF669	269.8141358	218.80121	320.8270663	0.551084047	0.130496704	4.22297291	2.41E-05	8.96E-05
ENSG00000105173	CCNE1	409.2561454	331.80908	486.7032153	0.550863292	0.126142472	4.36699301	1.26E-05	4.82E-05
ENSG00000172936	MYD88	2523.012574	2047.9965	2998.028674	0.550709707	0.046069429	11.9539078	6.19E-33	8.19E-32
ENSG00000196116	TDRD7	1177.096591	955.76642	1398.426766	0.550122032	0.067725724	8.12279297	4.56E-16	3.50E-15
ENSG00000137185	ZSCAN9	402.581279	326.89729	478.265269	0.549784899	0.111741271	4.92015973	8.65E-07	3.72E-06
ENSG00000134058	CDK7	1266.018266	1027.7867	1504.249863	0.549774397	0.064514237	8.521753	1.57E-17	1.29E-16
ENSG00000274602	PI4KAP1	279.0805529	226.6831	331.4780036	0.549616293	0.151393363	3.63038565	0.000283	0.000913
ENSG00000047410	TPR	12427.84359	10089.313	14766.37452	0.54960799	0.02911376	18.8779462	1.73E-79	5.11E-78
ENSG00000150938	CRIM1	48779.93986	39604.655	57955.22438	0.549288854	0.023090108	23.7889257	4.35E-125	1.94E-123
ENSG00000131067	GGT7	173.3344013	140.62201	206.0467946	0.549233144	0.171550854	3.20157627	0.001367	0.003944
ENSG00000137834	SMAD6	913.1672645	741.39774	1084.936792	0.54902328	0.080785159	6.79609082	1.07E-11	6.68E-11
ENSG00000163577	EIF5A2	351.5950603	285.88953	417.3005946	0.548833142	0.123220233	4.45408298	8.43E-06	3.28E-05
ENSG00000169991	IFFO2	1680.771189	1365.483	1996.059375	0.54877914	0.057992881	9.46287084	3.00E-21	2.83E-20
ENSG00000068366	ACSL4	5324.609823	4327.8556	6321.364066	0.546627154	0.037679673	14.5072159	1.09E-47	1.98E-46
ENSG00000127418	FGFRL1	1725.964137	1403.0446	2048.883645	0.54653954	0.063258442	8.63978818	5.63E-18	4.68E-17
ENSG00000107020	PLGRKT	330.7510331	268.86767	392.6343958	0.545943387	0.135033902	4.04300978	5.28E-05	0.000188
ENSG00000182963	GJC1	1737.700366	1412.6274	2062.773326	0.545238612	0.055193277	9.87871416	5.15E-23	5.18E-22
ENSG00000234072	AC074117.1	167.954114	136.68323	199.2249943	0.545173934	0.16475135	3.30907112	0.000936	0.002784
ENSG00000196123	KIAA0895L	641.9148864	522.51089	761.3188794	0.544114294	0.084135114	6.46714873	9.99E-11	5.84E-10
ENSG00000205593	DENND6B	122.6995583	99.881231	145.5178859	0.544033603	0.217623362	2.49988603	0.012423	0.029558
ENSG00000186105	LRRC70	141.0535494	114.95379	167.153308	0.543525334	0.191962683	2.83141143	0.004634	0.012083
ENSG00000116514	RNF19B	1350.701329	1098.656	1602.746671	0.543214933	0.065445863	8.30021811	1.04E-16	8.19E-16
ENSG00000182636	NDN	143.6554854	117.04508	170.2658923	0.542971176	0.176509209	3.07616344	0.002097	0.005876
ENSG00000160218	TRAPPC10	1612.134659	1313.6817	1910.587627	0.54223326	0.066759587	8.12217815	4.58E-16	3.51E-15
ENSG00000104998	IL27RA	572.9762455	466.667	679.2854898	0.541305918	0.088037567	6.14857881	7.82E-10	4.30E-09
ENSG00000138138	ATAD1	1402.014741	1141.8482	1662.181295	0.541135253	0.063989075	8.45668196	2.75E-17	2.22E-16
ENSG00000139372	TDG	1847.691843	1505.557	2189.826735	0.540652236	0.05202428	10.3923061	2.69E-25	2.92E-24
ENSG00000108511	HOXB6	343.8976296	280.22788	407.5673743	0.540566925	0.114036075	4.74031508	2.13E-06	8.86E-06
ENSG00000196715	VKORC1L1	3551.590682	2894.5165	4208.664872	0.540523887	0.042317561	12.7730398	2.32E-37	3.39E-36
ENSG00000198513	ATL1	139.6741036	113.85852	165.4896837	0.540469432	0.200052715	2.70163508	0.0069	0.017329
ENSG00000243317	STMP1	1549.41531	1262.7151	1836.115549	0.539937969	0.06744789	8.00526102	1.19E-15	8.99E-15
ENSG00000137509	PRCP	20995.22108	17114.435	24876.00693	0.539905937	0.028913515	18.6731342	8.19E-78	2.36E-76
ENSG00000176597	B3GNT5	2878.247808	2347.1888	3409.30682	0.53963162	0.050838958	10.6145294	2.55E-26	2.87E-25
ENSG00000226419	SLC16A1-AS1	233.2184993	190.13427	276.3027322	0.539002144	0.156966497	3.43386745	0.000595	0.001832
ENSG00000215717	TMEM167B	1514.282198	1235.2334	1793.330993	0.53856173	0.058080978	9.27260096	1.82E-20	1.66E-19
ENSG00000222041	CYTOR	1177.896682	960.49166	1395.301706	0.538178693	0.065519295	8.21404894	2.14E-16	1.66E-15
ENSG00000113448	PDE4D	1028.565907	839.37392	1217.757892	0.538103215	0.069684119	7.72203508	1.14E-14	8.29E-14
ENSG00000050165	DKK3	14306.073	11668.421	16943.72471	0.538090105	0.025348884	21.2273684	5.34E-100	2.01E-98
ENSG00000147905	ZCCHC7	831.7348501	678.63339	984.8363149	0.538087618	0.07878847	6.82952239	8.52E-12	5.34E-11
ENSG00000101199	ARFGAP1	3956.926943	3228.9509	4684.902992	0.536103662	0.039756952	13.4845262	1.93E-41	3.11E-40
ENSG00000061656	SPAG4	90.71625405	73.934893	107.4976153	0.536033269	0.227297344	2.35829095	0.018359	0.041788
ENSG00000111647	UHRF1BP1L	1202.922838	981.76944	1424.076232	0.535991607	0.062127925	8.62722532	6.29E-18	5.21E-17
ENSG00000068912	ERLEC1	2928.694762	2390.8029	3466.586603	0.535759728	0.046803745	11.4469414	2.44E-30	3.05E-29
ENSG00000172071	EIF2AK3	1331.459963	1087.2857	1575.634208	0.53563766	0.063004581	8.5015669	1.87E-17	1.53E-16
ENSG00000065600	TMEM206	776.5966707	634.37617	918.8171686	0.535414154	0.078314027	6.83675933	8.10E-12	5.08E-11
ENSG00000214193	SH3D21	117.7498792	96.325529	139.1742291	0.533786697	0.223278374	2.390678	0.016817	0.038729

ENSG00000197622	CDC42SE1	4638.415591	3793.7145	5483.116712	0.532405647	0.039465336	13.4904629	1.78E-41	2.87E-40
ENSG00000132432	SEC61G	2542.98876	2078.7533	3007.224252	0.532314153	0.050767935	10.4852434	1.01E-25	1.12E-24
ENSG00000162636	FAM102B	630.8230201	516.02771	745.6183339	0.532296951	0.093817582	5.67374411	1.40E-08	7.03E-08
ENSG00000086065	CHMP5	2328.778108	1904.5347	2753.021512	0.532230981	0.05299319	10.0433846	9.82E-24	1.02E-22
ENSG00000109790	KLHL5	3761.580035	3076.1574	4447.002658	0.53177275	0.037544315	14.1638686	1.53E-45	2.67E-44
ENSG00000160336	ZNF761	406.4347395	332.38981	480.4796699	0.531011929	0.107186451	4.95409563	7.27E-07	3.15E-06
ENSG00000117298	ECE1	80866.63896	66153.443	95579.8347	0.530915413	0.019270947	27.5500426	4.42E-167	2.66E-165
ENSG00000008710	PKD1	2945.557398	2410.5763	3480.538511	0.528990241	0.045902075	11.524321	9.95E-31	1.26E-29
ENSG00000185291	IL3RA	899.4212254	737.2645	1061.577949	0.528435728	0.076945543	6.86765873	6.53E-12	4.12E-11
ENSG00000116663	FBXO6	104.6775622	85.768413	123.5867115	0.52812019	0.217337752	2.42995148	0.015101	0.035244
ENSG00000198563	DDX39B	1229.261631	1007.1381	1451.385189	0.528007864	0.087104074	6.06180444	1.35E-09	7.28E-09
ENSG00000103064	SLC7A6	3196.872491	2618.4625	3775.282443	0.527977969	0.044346994	11.9056089	1.11E-32	1.46E-31
ENSG00000156671	SAMD8	1739.499718	1425.596	2053.403426	0.527915304	0.060432637	8.73559933	2.42E-18	2.04E-17
ENSG00000086619	ERO1B	249.0178639	204.23586	293.7998641	0.527637278	0.140944451	3.74358319	0.000181	0.000602
ENSG00000133678	TMEM254	281.0000245	230.26169	331.7383593	0.527451468	0.131824832	4.0011541	6.30E-05	0.000223
ENSG00000119661	DNAL1	605.4719205	496.4381	714.5057451	0.52702418	0.087624067	6.0146053	1.80E-09	9.65E-09
ENSG00000141526	SLC16A3	5624.707454	4607.0275	6642.38745	0.526972012	0.041564465	12.6784266	7.79E-37	1.12E-35
ENSG00000089505	CMTM1	96.72019094	79.417447	114.022935	0.525748128	0.225267994	2.3338785	0.019602	0.044343
ENSG00000117394	SLC2A1	1780.6995	1459.9192	2101.479786	0.525675749	0.05946594	8.83994684	9.58E-19	8.17E-18
ENSG00000169504	CLIC4	26104.92313	21409.833	30800.01291	0.524939315	0.032290322	16.2568621	2.00E-59	4.40E-58
ENSG00000100321	SYNGR1	135.273584	110.93251	159.6146625	0.524647849	0.179124095	2.92896301	0.003401	0.009117
ENSG00000182405	PGBD4	94.65545921	77.750796	111.5601227	0.52429024	0.229063045	2.2888469	0.022088	0.049318
ENSG00000174233	ADCY6	2831.332945	2323.4384	3339.227442	0.523661865	0.04603509	11.3752763	5.55E-30	6.89E-29
ENSG00000179820	MYADM	4736.898855	3888.5704	5585.22734	0.52251907	0.045201908	11.5596686	6.60E-31	8.41E-30
ENSG00000134470	IL15RA	1070.626725	878.45998	1262.793473	0.521969365	0.073720102	7.08042114	1.44E-12	9.39E-12
ENSG00000067646	ZFY	444.4190134	364.84294	523.9950826	0.521709021	0.110843962	4.70669772	2.52E-06	1.04E-05
ENSG00000174796	THAP6	161.1352778	132.10905	190.1615066	0.521492117	0.178079475	2.92842349	0.003407	0.00913
ENSG00000092531	SNAP23	7476.396197	6140.8023	8811.990133	0.521291872	0.033604886	15.5123834	2.86E-54	5.77E-53
ENSG00000100299	ARSA	1606.245715	1319.0221	1893.469377	0.521227313	0.060657741	8.59292323	8.48E-18	7.00E-17
ENSG00000197302	ZNF720	587.7110303	482.30184	693.1202174	0.521117618	0.095181349	5.47499721	4.38E-08	2.11E-07
ENSG00000213047	DENND1B	120.1616766	98.373149	141.9502041	0.520911213	0.215872633	2.41304888	0.01582	0.036712
ENSG00000222009	BTBD19	639.7347175	525.87738	753.592052	0.520856451	0.090674358	5.74425295	9.23E-09	4.71E-08
ENSG00000069399	BCL3	857.0964344	704.43416	1009.758707	0.520726866	0.086738393	6.00341839	1.93E-09	1.03E-08
ENSG00000175048	ZDHHC14	1226.909078	1007.5936	1446.224559	0.520473059	0.06456592	8.06111112	7.56E-16	5.75E-15
ENSG00000186480	INSIG1	2663.890269	2189.6406	3138.139952	0.519829879	0.048314069	10.7593894	5.35E-27	6.08E-26
ENSG00000212907	MT-ND4L	10811.79493	8888.2105	12735.37937	0.519013308	0.08803087	5.89581031	3.73E-09	1.96E-08
ENSG00000166347	CYB5A	621.0930641	510.56111	731.6250188	0.518665692	0.090635251	5.72256034	1.05E-08	5.33E-08
ENSG00000131724	IL13RA1	7952.338187	6539.8279	9364.848521	0.51836563	0.031289358	16.566835	1.21E-61	2.74E-60
ENSG00000137713	PPP2R1B	2291.688906	1884.4455	2698.932287	0.518294914	0.047872708	10.8265217	2.58E-27	2.96E-26
ENSG00000188234	AGAP4	120.8582816	99.223625	142.4929379	0.518098653	0.197481565	2.62352921	0.008702	0.021423
ENSG00000109270	LAMTOR3	1096.296441	901.9504	1290.642477	0.517951143	0.066631943	7.77331586	7.65E-15	5.59E-14
ENSG00000249915	PDCD6	91.43273699	75.253108	107.6123658	0.517771577	0.226033821	2.29068187	0.021982	0.049098
ENSG00000137478	FCHSD2	3573.824678	2939.3696	4208.279708	0.517562785	0.039513607	13.0983432	3.37E-39	5.16E-38
ENSG00000183386	FHL3	1666.514167	1371.0848	1961.943529	0.517478806	0.06588935	7.85375496	4.04E-15	2.99E-14
ENSG00000154930	ACSS1	993.345996	817.48181	1169.210182	0.517066842	0.075203577	6.87556181	6.17E-12	3.90E-11
ENSG00000063587	ZNF275	789.4312643	649.11136	929.7511713	0.517014758	0.081205827	6.36671994	1.93E-10	1.11E-09
ENSG00000132963	POMP	3911.453753	3219.3917	4603.51576	0.516592408	0.040361916	12.7990061	1.66E-37	2.44E-36
ENSG00000182541	LIMK2	3490.131704	2871.74	4108.523455	0.516573857	0.043953868	11.7526369	6.84E-32	8.88E-31
ENSG00000102780	DGKH	1937.888268	1594.5398	2281.236749	0.516338993	0.050433961	10.2379226	1.34E-24	1.43E-23
ENSG00000178719	GRINA	7075.797931	5825.2369	8326.358929	0.515655881	0.03578266	14.4107756	4.43E-47	7.95E-46
ENSG00000270959	LPP-AS2	123.2860328	101.59993	144.9721398	0.51546905	0.196226333	2.62691068	0.008616	0.021228
ENSG00000047644	WWC3	4646.78794	3825.8534	5467.722451	0.51542273	0.035863575	14.3717609	7.78E-47	1.39E-45
ENSG00000137494	ANKRD42	464.0180429	382.19922	545.836866	0.515348931	0.109338535	4.71333306	2.44E-06	1.00E-05
ENSG00000131591	C1orf159	481.7189846	396.23766	567.2003127	0.515306589	0.101728223	5.06552235	4.07E-07	1.81E-06
ENSG00000196352	CD55	6715.686215	5530.2563	7901.116115	0.5149411	0.036692245	14.0340583	9.65E-45	1.65E-43
ENSG00000113504	SLC12A7	2589.673176	2132.8937	3046.452672	0.514876569	0.04881924	10.5465913	5.27E-26	5.86E-25
ENSG00000147065	MSN	68027.65503	56032.796	80022.51417	0.514179241	0.021379856	24.0497053	8.41E-128	3.85E-126
ENSG00000100883	SRP54	3374.887507	2780.2706	3969.504436	0.513521862	0.039636842	12.9556706	2.18E-38	3.29E-37
ENSG00000164164	OTUD4	3153.286321	2599.6202	3706.952401	0.513246721	0.050411805	10.181082	2.41E-24	2.54E-23
ENSG00000004864	SLC25A13	1800.161721	1483.16	2117.163436	0.512918623	0.05712542	8.97881582	2.74E-19	2.39E-18
ENSG00000187676	B3GLCT	1058.561318	872.68163	1244.441004	0.512201659	0.070097645	7.30697381	2.73E-13	1.86E-12
ENSG00000139116	KIF21A	711.5044986	586.28865	836.7203439	0.512137745	0.091549508	5.59410701	2.22E-08	1.10E-07
ENSG00000104880	ARHGEF18	160.0744881	131.85169	188.2972824	0.510979138	0.171811285	2.97407203	0.002939	0.007993
ENSG00000196498	NCOR2	12331.46428	10171.662	14491.26657	0.510753578	0.030619734	16.680536	1.82E-62	4.19E-61
ENSG00000114850	SSR3	13061.78049	10779.244	15344.31746	0.509880925	0.028592705	17.8325531	3.95E-71	1.03E-69
ENSG00000082397	EPB41L3	6684.609665	5516.3246	7852.894698	0.509560702	0.036311376	14.0330872	9.78E-45	1.67E-43
ENSG00000132031	MATN3	288.2217168	237.66682	338.7766167	0.508896575	0.130553826	3.89798284	9.70E-05	0.000334
ENSG00000198142	SOWAHC	198.6217146	164.27756	232.9658677	0.50887169	0.158519071	3.2101607	0.001327	0.003839
ENSG00000184903	IMMP2L	142.5174352	117.74973	167.2851441	0.508497767	0.178082278	2.85540916	0.004298	0.011277
ENSG00000109787	KLF3	2884.659042	2382.7716	3386.546468	0.508387136	0.051410606	9.88875992	4.66E-23	4.69E-22
ENSG00000204389	HSPA1A	889.0932079	733.88205	1044.304364	0.507948742	0.0782158	6.49419609	8.35E-11	4.92E-10
ENSG00000103811	CTSH	178.4578677	147.34463	209.5711043	0.507408284	0.167991464	3.02044087	0.002524	0.006962
ENSG00000114841	DNAH1	209.5279395	173.08221	245.9736671	0.507110544	0.147408407	3.44017384	0.000581	0.001792
ENSG00000157693	TMEM268	442.1701335	365.83195	518.5083166	0.50627065	0.108463963	4.66763925	3.05E-06	1.24E-05
ENSG00000244879	GABPB1-AS1	225.8389802	186.51205	265.1659127	0.506121329	0.140829096	3.59386906	0.000326	0.001041
ENSG00000120008	WDR11	4408.805624	3644.1565	5173.454796	0.505635968	0.038036992	13.2932689	2.53E-40	3.99E-39
ENSG00000171680	PLEKHG5	2924.901593	2417.534	3432.269207	0.505045773	0.048607111	10.3903681	2.74E-25	2.98E-24
ENSG00000060982	BCAT1	17536.34749	14503.585	20569.10974	0.504451943	0.026692766	18.8984511	1.17E-79	3.48E-78
ENSG00000135164	DMTF1	1053.577842	871.41378	1235.7419	0.503685889	0.080183238	6.2816856	3.35E-10	1.89E-09
ENSG00000135720	DYNC1LI2	7852.464483	6496.0939	9208.835031	0.503432793	0.028668172	17.5606871	4.93E-69	1.25E-67
ENSG00000006576	PHTF2	1117.726705	924.89823	1310.555177	0.503362863	0.065094364	7.7328179	1.05E-14	7.64E-14
ENSG00000137094	DNAJB5	781.0694812	646.52469	915.614269	0.502641067	0.082350489	6.10368044	1.04E-09	5.65E-09
ENSG00000160285	LSS	5498.07999	4549.7245	6446.435473	0.502028939	0.041244254	12.172094	4.38E-34	5.90E-33
ENSG00000197712	FAM114A1	4851.990188	4017.8138	5686.166592	0.501449931	0.036880465	13.5966271	4.19E-42	6.87E-41
ENSG00000203879	GDI1	4635.242444	3838.7475	5431.737415	0.50072229	0.036750249	13.6250039	2.84E-42	4.67E-41

ENSG00000144136	SLC20A1	5664.224172	4693.0022	6635.446164	0.500037894	0.03348264	14.9342433	1.97E-50	3.73E-49
ENSG00000133131	MORC4	714.4230814	837.37354	591.4726236	-0.501187589	0.086274825	-5.8091985	6.28E-09	3.24E-08
ENSG00000160014	CALM3	14614.24689	17127.58	12100.91426	-0.501408345	0.026237205	-19.110585	2.06E-81	6.37E-80
ENSG00000166436	TRIM66	222.4560041	260.98586	183.9261456	-0.501600004	0.154912811	-3.2379504	0.001204	0.00351
ENSG00000116191	RALGPS2	1211.464428	1420.8519	1002.077001	-0.501899823	0.071556398	-7.0140454	2.32E-12	1.49E-11
ENSG00000125863	MKKS	449.9074095	527.4969	372.3179167	-0.501998845	0.104285524	-4.8136963	1.48E-06	6.25E-06
ENSG00000169760	NLGN1	763.9215489	896.09911	631.7439872	-0.502001519	0.083169981	-6.0358498	1.58E-09	8.52E-09
ENSG00000008405	CRY1	1000.757453	1172.843	828.6719369	-0.502261056	0.069579041	-7.2185683	5.25E-13	3.52E-12
ENSG00000003096	KLHL13	1125.619425	1319.4626	931.7762729	-0.502327814	0.072349329	-6.9430888	3.84E-12	2.45E-11
ENSG00000219481	NBPF1	825.6305541	967.66292	683.5981927	-0.502782873	0.077291594	-6.5050137	7.77E-11	4.58E-10
ENSG00000117543	DPH5	605.6713963	710.40322	500.9395753	-0.502858945	0.107777305	-4.665722	3.08E-06	1.25E-05
ENSG00000205544	TMEM256	116.4105779	136.54545	96.27570986	-0.503711639	0.201468613	-2.5001991	0.012412	0.029536
ENSG00000166037	CEP57	1635.219196	1917.2254	1353.212999	-0.504163776	0.058145268	-8.670762	4.29E-18	3.59E-17
ENSG00000133597	ADCK2	666.5617006	782.03453	551.0888695	-0.504894267	0.085475222	-5.9069079	3.49E-09	1.83E-08
ENSG00000196468	FGF16	119.7138358	140.20075	99.22692527	-0.504949493	0.211485935	-2.3876268	0.016958	0.039008
ENSG00000163050	COQ8A	373.396914	437.93713	308.8566946	-0.50504987	0.111930801	-4.5121617	6.42E-06	2.53E-05
ENSG00000065717	TLE2	1210.069677	1419.7924	1000.346927	-0.505749564	0.066582858	-7.5957923	3.06E-14	2.17E-13
ENSG00000160062	ZBTB8A	499.1286368	585.68316	412.5741105	-0.506158388	0.096823418	-5.2276443	1.72E-07	7.88E-07
ENSG00000065308	TRAM2	9252.58904	10859.403	7645.775542	-0.506402544	0.028864779	-17.54396	6.62E-69	1.67E-67
ENSG00000231298	MANCR	966.0495845	1134.0509	798.0482834	-0.506655904	0.070890621	-7.147009	8.87E-13	5.86E-12
ENSG00000095383	TBC1D2	455.0731072	533.82164	376.3245726	-0.506808875	0.106253197	-4.7698224	1.84E-06	7.70E-06
ENSG00000106617	PRKAG2	1158.689751	1360.6248	956.7546723	-0.506854478	0.066592113	-7.6113289	2.71E-14	1.93E-13
ENSG00000124126	PREX1	8118.987304	9532.7441	6705.230539	-0.507157447	0.030574575	-16.587555	8.57E-62	1.95E-60
ENSG00000005022	SLC25A5	8704.145664	10218.337	7189.954282	-0.507491856	0.029573054	-17.160617	5.24E-66	1.28E-64
ENSG00000110799	VWF	233669.6817	274355.48	192983.8874	-0.507577946	0.019474848	-26.063256	9.52E-150	5.19E-148
ENSG00000104915	STX10	1116.338993	1310.8865	921.7915265	-0.508283383	0.065352992	-7.7775075	7.40E-15	5.41E-14
ENSG00000175573	C11orf68	1242.190465	1458.9631	1025.417799	-0.508284888	0.065533425	-7.7561166	8.76E-15	6.40E-14
ENSG00000052126	PLEKHA5	598.2284404	702.86214	493.5947386	-0.508814152	0.088415537	-5.7548047	8.67E-09	4.44E-08
ENSG00000213390	ARHGAP19	658.150611	773.11042	543.1908027	-0.509031769	0.095697283	-5.3191873	1.04E-07	4.88E-07
ENSG00000101445	PPP1R16B	9948.314779	11686.298	8210.331707	-0.509053219	0.027277372	-18.662107	1.01E-77	2.89E-76
ENSG00000162542	TMCO4	335.8696014	394.50085	277.2383538	-0.510441251	0.115106663	-4.4345065	9.23E-06	3.58E-05
ENSG00000197872	FAM49A	2404.599829	2826.239	1982.960695	-0.510599156	0.050604708	-10.089954	6.12E-24	6.37E-23
ENSG00000112130	RNF8	1037.644639	1219.7153	855.5739945	-0.510704918	0.071269488	-7.1658283	7.73E-13	5.12E-12
ENSG00000145284	SCD5	2123.580548	2495.9331	1751.227956	-0.51090566	0.049116788	-10.401854	2.43E-25	2.64E-24
ENSG00000166128	RAB8B	2551.907625	3000.0129	2103.802383	-0.510938712	0.049793376	-10.261178	1.05E-24	1.12E-23
ENSG00000198331	HYLS1	396.0413039	465.67296	326.4096487	-0.511121321	0.108648344	-4.7043636	2.55E-06	1.05E-05
ENSG00000162878	PKDCC	120.2235974	141.46722	98.97997604	-0.51163617	0.198718246	-2.5746814	0.010033	0.024354
ENSG00000081923	ATP8B1	5231.684075	6151.7937	4311.574463	-0.511877084	0.038526092	-13.286504	2.77E-40	4.37E-39
ENSG00000135597	REPS1	1016.358428	1195.311	837.4058988	-0.512055313	0.080704303	-6.3448329	2.23E-10	1.27E-09
ENSG00000159399	HK2	951.2723878	1118.3041	784.2407022	-0.512442464	0.071005922	-7.2168975	5.32E-13	3.56E-12
ENSG00000119950	MXI1	242.2821144	284.96942	199.5948069	-0.512717456	0.134400964	-3.8148347	0.000136	0.00046
ENSG00000136824	SMC2	2859.853932	3363.3088	2356.399064	-0.512825965	0.049954147	-10.265934	1.00E-24	1.07E-23
ENSG00000052723	SIKE1	1171.379245	1378.007	964.7514599	-0.512894913	0.069038904	-7.4290709	1.09E-13	7.58E-13
ENSG00000161542	PRPSAP1	1841.070424	2164.7757	1517.365137	-0.513484959	0.056578665	-9.0755933	1.13E-19	1.01E-18
ENSG00000278535	DHRS11	168.7397224	198.63848	138.8409656	-0.513759762	0.165019807	-3.1133218	0.00185	0.00523
ENSG00000137492	THAP12	2118.350384	2491.4345	1745.266229	-0.513902903	0.051207118	-10.035771	1.06E-23	1.10E-22
ENSG00000189057	FAM111B	1412.245961	1661.3643	1163.127603	-0.514010174	0.061477629	-8.3609303	6.22E-17	4.96E-16
ENSG00000152492	CCDC50	6633.086822	7802.3991	5463.774554	-0.514121793	0.032390452	-15.872634	9.80E-57	2.05E-55
ENSG00000141447	OSBPL1A	913.4970336	1074.4775	752.516613	-0.51439406	0.070795701	-7.265894	3.71E-13	2.50E-12
ENSG00000168661	ZNF30	151.6435397	178.49376	124.7933198	-0.514850491	0.172224969	-2.9894068	0.002795	0.007639
ENSG00000074071	MRPS34	1764.765833	2075.8588	1453.672893	-0.515018845	0.058152854	-8.8562953	8.27E-19	7.09E-18
ENSG00000182095	TNRC18	5080.912757	5978.8557	4182.96977	-0.515579325	0.043405872	-11.878101	1.54E-32	2.02E-31
ENSG00000115271	GCA	205.6070388	242.3452	168.8688824	-0.516200922	0.167989688	-3.0728131	0.002121	0.005938
ENSG00000143554	SLC27A3	1050.222797	1236.0267	864.4189398	-0.516454623	0.071570042	-7.2160727	5.35E-13	3.58E-12
ENSG00000136710	CCDC115	729.5941825	859.02964	600.158721	-0.517095953	0.082189502	-6.2915086	3.14E-10	1.78E-09
ENSG00000150593	PDCD4	2309.363771	2718.6013	1900.126261	-0.517189706	0.048110926	-10.749943	5.93E-27	6.73E-26
ENSG00000102384	CENPI	619.2569643	729.0831	509.4308286	-0.517251773	0.085834441	-6.0261565	1.68E-09	9.01E-09
ENSG00000278845	MRPL45	1526.053722	1796.855	1255.252476	-0.51731178	0.055873045	-9.2587003	2.07E-20	1.89E-19
ENSG00000035687	ADSS	4422.211698	5207.0032	3637.420222	-0.517584326	0.038822169	-13.332185	1.50E-40	2.39E-39
ENSG00000120334	CENPL	393.0979974	462.58411	323.6118824	-0.518510504	0.120967184	-4.2863733	1.82E-05	6.84E-05
ENSG00000106268	NUDT1	237.8740088	280.0272	195.7208148	-0.518742037	0.143258647	-3.6210173	0.000293	0.000945
ENSG00000180628	PCGF5	1477.430057	1740.9955	1213.864656	-0.519025178	0.069418564	-7.476749	7.62E-14	5.31E-13
ENSG00000138613	APH1B	886.9616068	1045.388	728.5352466	-0.519214626	0.0828905	-6.2638617	3.76E-10	2.11E-09
ENSG00000175602	CCDC85B	3482.013405	4101.5345	2862.492314	-0.519647562	0.049718848	-10.451722	1.44E-25	1.57E-24
ENSG00000105011	ASF1B	1131.530945	1332.9473	930.1146229	-0.520300257	0.068316918	-7.6159796	2.62E-14	1.86E-13
ENSG00000116133	DHCR24	10121.44713	11927.755	8315.139454	-0.520556999	0.027355047	-19.029651	9.69E-81	2.94E-79
ENSG00000106089	STX1A	260.462943	306.63327	214.2926196	-0.520574907	0.135754563	-3.834677	0.000126	0.000426
ENSG00000178996	SNX18	1460.752468	1721.9762	1199.528715	-0.520772222	0.057510184	-9.0553043	1.36E-19	1.21E-18
ENSG00000176422	SPRYD4	373.1446824	439.87303	306.4163316	-0.521317752	0.111759245	-4.6646499	3.09E-06	1.26E-05
ENSG00000185238	PRMT3	646.9372862	762.61332	531.2612557	-0.521406793	0.083739971	-6.2264983	4.77E-10	2.66E-09
ENSG00000232677	LINC00665	190.1898359	224.29839	156.0812806	-0.521441654	0.152261115	-3.4246541	0.000616	0.001892
ENSG00000101255	TRIB3	1249.807792	1473.3966	1026.219032	-0.521566461	0.06661295	-7.8298058	4.89E-15	3.60E-14
ENSG00000162496	DHRS3	1001.613381	1180.9381	822.2886836	-0.521935446	0.067718489	-7.707429	1.28E-14	9.25E-14
ENSG00000006634	DBF4	838.1962864	988.80124	687.5913369	-0.522865456	0.080846088	-6.4674181	9.97E-11	5.83E-10
ENSG00000182224	CYB5D1	112.6413349	132.80007	92.48259823	-0.523920533	0.204425468	-2.5628927	0.01038	0.02512
ENSG00000187824	TMEM220	105.5019851	124.55177	86.45220357	-0.524697377	0.207063262	-2.5339955	0.011277	0.027051
ENSG00000153048	CARHSP1	1156.769611	1364.9512	948.5880374	-0.52477166	0.06386011	-8.2175189	2.08E-16	1.62E-15
ENSG00000139910	NOVA1	216.7693885	255.87412	177.664659	-0.525036469	0.143447026	-3.6601419	0.000252	0.00082
ENSG00000226950	DANCR	663.0867333	783.14247	543.0309954	-0.525639656	0.089482419	-5.8742227	4.25E-09	2.22E-08
ENSG00000042286	AIFM2	366.0496949	432.33637	299.7630159	-0.525778919	0.117510119	-4.4743289	7.67E-06	3.00E-05
ENSG00000157111	TMEM171	322.8757985	380.88544	264.8661565	-0.526508884	0.127411044	-4.1323646	3.59E-05	0.000131
ENSG00000100629	CEP128	447.5711407	528.47935	366.6629295	-0.526785519	0.104022989	-5.0641259	4.10E-07	1.82E-06
ENSG00000008441	NFIX	1689.294151	1995.0401	1383.548224	-0.527384377	0.060525824	-8.713378	2.95E-18	2.48E-17
ENSG00000114346	ECT2	1592.567677	1880.1121	1305.023216	-0.527536152	0.055765962	-9.4598233	3.08E-21	2.91E-20
ENSG00000005189	REXO5	150.7606915	178.17195	123.3494282	-0.527937827	0.190890505	-2.7656579	0.005681	0.014531

ENSG00000076248	UNG	1554.468753	1835.8993	1273.038207	-0.528181882	0.055662129	-9.4890707	2.33E-21	2.22E-20
ENSG00000257167	TMPO-AS1	108.6998398	128.26996	89.12971824	-0.528518544	0.221071463	-2.3907136	0.016816	0.038729
ENSG00000125510	OPRL1	97.11043719	114.62573	79.59514407	-0.528599422	0.226161652	-2.3372637	0.019425	0.043997
ENSG00000135046	ANXA1	12086.80683	14279.071	9894.542285	-0.529404965	0.025611747	-20.670396	6.40E-95	2.32E-93
ENSG00000115159	GPD2	1454.312862	1718.7738	1189.851943	-0.529641697	0.058878356	-8.9955246	2.35E-19	2.06E-18
ENSG00000203880	PCMTD2	1117.44391	1320.3475	914.5403215	-0.530039123	0.066129807	-8.0151319	1.10E-15	8.32E-15
ENSG00000168264	IRF2BP2	4658.546816	5506.36	3810.733597	-0.530607412	0.039382028	-13.473339	2.24E-41	3.61E-40
ENSG00000066136	NFYC	1097.339914	1297.0085	897.6713106	-0.531072666	0.066170955	-8.025767	1.01E-15	7.64E-15
ENSG00000103495	MAZ	6077.581853	7185.3282	4969.835525	-0.531325157	0.045807243	-11.599152	4.16E-31	5.31E-30
ENSG00000137936	BCAR3	1813.459918	2143.8011	1483.118713	-0.531498353	0.05660454	-9.3896771	6.02E-21	5.62E-20
ENSG00000198830	HMGN2	7884.446478	9322.2929	6446.600091	-0.532051567	0.030475634	-17.458261	2.98E-68	7.46E-67
ENSG00000205356	TECPR1	3871.000099	4576.9884	3165.011754	-0.532375758	0.042035887	-12.664792	9.27E-37	1.33E-35
ENSG00000166912	MTMR10	2150.612606	2543.4966	1757.728578	-0.532391392	0.05071426	-10.497864	8.84E-26	9.76E-25
ENSG00000166582	CENPV	433.2402775	511.9586	354.5219557	-0.532530018	0.111001864	-4.7974872	1.61E-06	6.75E-06
ENSG00000080839	RBL1	857.7332269	1014.557	700.9094884	-0.532713404	0.075303827	-7.0741877	1.50E-12	9.82E-12
ENSG00000165644	COMTD1	150.7587395	178.21622	123.3012567	-0.532830542	0.1781066	-2.9916384	0.002775	0.007589
ENSG00000119333	WDR34	1673.139233	1978.6053	1367.673134	-0.533179032	0.054190921	-9.8388996	7.65E-23	7.65E-22
ENSG00000184640	9-Sep	6046.111098	7150.806	4941.416197	-0.533371755	0.035928567	-14.845339	7.46E-50	1.40E-48
ENSG00000097046	CDC7	328.2242933	388.31937	268.1292127	-0.533501323	0.120733139	-4.4188474	9.92E-06	3.83E-05
ENSG00000175879	HOXD8	414.0652573	489.5061	338.6244194	-0.533514	0.106923305	-4.9896886	6.05E-07	2.64E-06
ENSG00000122644	ARL4A	2266.244796	2679.9547	1852.534867	-0.533609308	0.047890961	-11.142172	7.82E-29	9.43E-28
ENSG00000104691	UBXN8	303.678382	359.62481	247.7319497	-0.534337042	0.13497847	-3.9586835	7.54E-05	0.000263
ENSG00000106829	TLE4	1187.270065	1405.3101	969.2300734	-0.535288516	0.081076498	-6.6022649	4.05E-11	2.42E-10
ENSG00000161682	FAM171A2	514.308965	608.58413	420.0338027	-0.53549885	0.133249505	-4.018768	5.85E-05	0.000207
ENSG00000148655	LRMDA	111.6901637	132.10864	91.27168491	-0.535822939	0.198929882	-2.6935266	0.00707	0.017705
ENSG00000047579	DTNBP1	742.4237089	878.19198	606.655435	-0.536204869	0.085950553	-6.2385273	4.42E-10	2.47E-09
ENSG00000119929	CUTC	396.2691068	469.0922	323.4460154	-0.536753018	0.108478821	-4.9479982	7.50E-07	3.25E-06
ENSG00000137404	NRM	567.9890343	672.83509	463.1429797	-0.537737517	0.092634326	-5.8049488	6.44E-09	3.32E-08
ENSG00000113810	SMC4	6401.100753	7581.2513	5220.950216	-0.537804618	0.033084784	-16.255346	2.05E-59	4.50E-58
ENSG00000222011	FAM185A	111.2116011	131.77268	90.65051865	-0.537879108	0.208797704	-2.5760777	0.009993	0.024271
ENSG00000133027	PEMT	495.0478765	586.26922	403.8265374	-0.538357338	0.097539859	-5.5193574	3.40E-08	1.66E-07
ENSG00000176225	RTTN	548.0682669	649.55927	446.5772639	-0.538980285	0.093467539	-5.766497	8.09E-09	4.15E-08
ENSG00000005448	WDR54	382.6611844	453.464	311.8583684	-0.539554831	0.111081267	-4.8572981	1.19E-06	5.05E-06
ENSG00000214756	CSKMT	149.8246956	177.76971	121.8796828	-0.539764416	0.218369121	-2.4717983	0.013444	0.031755
ENSG00000134531	EMP1	11891.22776	14096.31	9686.145291	-0.54092448	0.031763682	-17.029653	4.95E-65	1.19E-63
ENSG00000088727	KIF9	295.1522051	349.90743	240.3969807	-0.54094757	0.122219263	-4.4260418	9.60E-06	3.72E-05
ENSG00000260196	AC124798.1	118.831105	140.74716	96.91504978	-0.5410795	0.201806054	-2.6811857	0.007336	0.018327
ENSG00000172922	RNASEH2C	1008.43215	1194.6893	822.175024	-0.541111935	0.072488447	-7.4648024	8.34E-14	5.80E-13
ENSG00000151690	MFSD6	327.4059426	388.53432	266.2775674	-0.54112573	0.127041947	-4.2594257	2.05E-05	7.68E-05
ENSG00000130021	PUDP	915.5388374	1084.8885	746.189144	-0.541147786	0.073799371	-7.3326883	2.26E-13	1.54E-12
ENSG00000164087	POC1A	434.3666148	514.83675	353.8964844	-0.5416948	0.110432228	-4.9052239	9.33E-07	4.01E-06
ENSG00000224078	SNHG14	2775.509316	3291.241	2259.777626	-0.542032802	0.04418569	-12.267157	1.36E-34	1.86E-33
ENSG00000129810	SGO1	309.0610034	366.43737	251.6846376	-0.542087979	0.126356025	-4.2901633	1.79E-05	6.72E-05
ENSG00000189227	C15orf61	118.9773308	141.09245	96.86221063	-0.542321046	0.193672223	-2.8002004	0.005107	0.013201
ENSG00000240891	PLCXD2	166.7805098	197.75434	135.8066822	-0.542361709	0.163394127	-3.3193464	0.000902	0.002688
ENSG00000266208	AC080112.1	311.4755576	369.08541	253.8657064	-0.542761909	0.127732841	-4.2491962	2.15E-05	8.01E-05
ENSG00000105514	RAB3D	1672.13954	1983.0847	1361.194368	-0.542786001	0.068099039	-7.9705383	1.58E-15	1.19E-14
ENSG00000112576	CCND3	2940.310691	3486.745	2393.876341	-0.542879108	0.044911413	-12.087776	1.23E-33	1.64E-32
ENSG00000183873	SCN5A	640.6771355	759.61875	521.7355226	-0.543204525	0.091317968	-5.9484955	2.71E-09	1.44E-08
ENSG00000172915	NBEA	441.4800884	523.41834	359.5418352	-0.543599162	0.106541623	-5.1022234	3.36E-07	1.50E-06
ENSG00000184508	HDDC3	320.9655843	380.79751	261.1336636	-0.543644731	0.12184873	-4.4616364	8.13E-06	3.17E-05
ENSG00000226137	BAIAP2-DT	1396.290082	1656.5051	1136.075042	-0.543937932	0.064317225	-8.4571113	2.74E-17	2.22E-16
ENSG00000183615	FAM167B	114.1127073	135.37847	92.84694762	-0.544199177	0.200794125	-2.7102346	0.006724	0.016942
ENSG00000146416	AIG1	382.6791976	454.21346	311.1449304	-0.544365688	0.111075296	-4.9008709	9.54E-07	4.09E-06
ENSG00000204682	CASC10	131.8103268	156.44014	107.1805103	-0.544572083	0.196885651	-2.7659308	0.005676	0.014521
ENSG00000131652	THOC6	575.1981396	682.4275	467.9687813	-0.545232181	0.101890073	-5.3511806	8.74E-08	4.12E-07
ENSG00000169814	BTD	876.9361442	1041.1272	712.745114	-0.546402672	0.077648755	-7.0368504	1.97E-12	1.28E-11
ENSG00000112208	BAG2	678.7743186	805.86648	551.6821526	-0.54652702	0.091531665	-5.9709065	2.36E-09	1.25E-08
ENSG00000164611	PTTG1	1024.591626	1216.8267	832.3565245	-0.546686103	0.080645633	-6.778868	1.21E-11	7.48E-11
ENSG00000118564	FBXL5	2362.554277	2806.1716	1918.936908	-0.547077203	0.04984436	-10.975709	5.00E-28	5.88E-27
ENSG00000152284	TCF7L1	1508.476854	1791.4705	1225.483231	-0.5472136	0.060905716	-8.9846018	2.60E-19	2.27E-18
ENSG00000130520	LSM4	1863.882886	2213.985	1513.780735	-0.547963966	0.058697336	-9.3354145	1.01E-20	9.31E-20
ENSG00000171241	SHCBP1	1771.416212	2103.2177	1439.61468	-0.548305509	0.063283492	-8.6642739	4.54E-18	3.80E-17
ENSG00000173599	PC	496.6939619	589.85697	403.5309518	-0.54851392	0.122988586	-4.4598766	8.20E-06	3.20E-05
ENSG00000149743	TRPT1	366.3258953	435.45479	297.1970054	-0.549074485	0.116324685	-4.7201889	2.36E-06	9.73E-06
ENSG00000145335	SNCA	4101.602129	4871.9302	3331.274056	-0.549218276	0.039173438	-14.02017	1.17E-44	2.01E-43
ENSG00000146243	IRAK1BP1	220.6451361	262.09362	179.1966491	-0.550036698	0.147959362	-3.7174849	0.000201	0.000663
ENSG00000137269	LRRC1	658.9058332	782.92171	534.8899538	-0.550238529	0.084918698	-6.4795922	9.20E-11	5.39E-10
ENSG00000164659	KIAA1324L	2274.211916	2703.8039	1844.619884	-0.551148343	0.054634311	-10.087953	6.25E-24	6.50E-23
ENSG00000130203	APOE	228.4649039	271.54729	185.3825166	-0.552015689	0.157404523	-3.5069875	0.000453	0.001416
ENSG00000136098	NEK3	312.9051957	371.77418	254.0362089	-0.55384881	0.135425011	-4.0897084	4.32E-05	0.000156
ENSG00000166439	RNF169	1489.849056	1772.4803	1207.217836	-0.554119798	0.057134304	-9.6985481	3.06E-22	3.00E-21
ENSG00000166845	C18orf54	860.896851	1024.2021	697.5915779	-0.554395177	0.081517456	-6.8009382	1.04E-11	6.47E-11
ENSG00000154920	EME1	130.4660983	155.17637	105.7558217	-0.554411121	0.219530472	-2.5254404	0.011555	0.027657
ENSG00000171877	FRMD5	806.4256727	959.84791	653.0034375	-0.555176182	0.07645307	-7.2616598	3.82E-13	2.57E-12
ENSG00000168517	HEXIM2	82.9144524	98.738622	67.09028306	-0.555504028	0.242794102	-2.2879634	0.02214	0.049414
ENSG00000075618	FSCN1	20478.38905	24375.107	16581.67096	-0.55582837	0.027382582	-20.298611	1.32E-91	4.64E-90
ENSG00000001460	STPG1	237.3400035	282.2988	192.3812097	-0.556024052	0.142497679	-3.9019867	9.54E-05	0.000329
ENSG00000117155	SSX2IP	429.4331295	511.21474	347.6515191	-0.55624156	0.109835459	-5.0643168	4.10E-07	1.82E-06
ENSG00000147471	PLPBP	1888.129936	2248.0525	1528.207388	-0.556731216	0.055652232	-10.003754	1.47E-23	1.51E-22
ENSG00000197905	TEAD4	1291.18518	1537.4158	1044.954528	-0.557864131	0.062608775	-8.9103186	5.09E-19	4.40E-18
ENSG00000164236	ANKRD33B	580.9902157	691.55864	470.421796	-0.558568565	0.094167917	-5.9316228	3.00E-09	1.59E-08
ENSG00000118922	KLF12	1833.458802	2184.0644	1482.853216	-0.559084961	0.053208811	-10.507376	7.99E-26	8.84E-25
ENSG00000107186	MPDZ	4159.450749	4955.7906	3363.110942	-0.559543944	0.040227828	-13.909375	5.56E-44	9.38E-43
ENSG00000188312	CENPP	203.944233	243.2906	164.5978612	-0.559562996	0.157914966	-3.543445	0.000395	0.001245

ENSG00000168090	COPS6	4465.580265	5321.6785	3609.482079	-0.560042015	0.037471361	-14.945868	1.66E-50	3.14E-49
ENSG00000283498	MIR1244-2	157.1660866	187.2827	127.0494725	-0.560892369	0.185053391	-3.0309759	0.002438	0.006747
ENSG00000139880	CDH24	757.9237823	903.41567	612.4318963	-0.561146198	0.093110785	-6.0266509	1.67E-09	8.99E-09
ENSG00000118640	VAMP8	950.3993694	1132.3777	768.4210633	-0.561452216	0.086612974	-6.4823108	9.03E-11	5.30E-10
ENSG00000177602	HASPIN	348.3820142	415.10905	281.6549802	-0.56167849	0.116967028	-4.8020241	1.57E-06	6.61E-06
ENSG00000112312	GMNN	846.5900695	1008.7842	684.3959185	-0.56169491	0.083726326	-6.7087012	1.96E-11	1.20E-10
ENSG00000132199	ENOSF1	714.0868069	851.26859	576.9050268	-0.561986412	0.08403762	-6.6873195	2.27E-11	1.39E-10
ENSG00000146670	CDCA5	1368.701537	1632.417	1104.986093	-0.564142546	0.061550533	-9.1655185	4.93E-20	4.44E-19
ENSG00000176809	LRRC37A3	138.5207213	165.55359	111.4878494	-0.56419699	0.192723875	-2.9274888	0.003417	0.009153
ENSG00000168286	THAP11	887.3195313	1058.9661	715.6729937	-0.564236257	0.080354712	-7.0218191	2.19E-12	1.42E-11
ENSG00000071539	TRIP13	1242.876503	1482.9322	1002.820791	-0.56443348	0.068111805	-8.2868671	1.16E-16	9.14E-16
ENSG00000145632	PLK2	9940.625615	11860.567	8020.683799	-0.56447576	0.028723041	-19.652368	5.52E-86	1.81E-84
ENSG00000170100	ZNF778	355.3303548	423.64586	287.0148468	-0.564718818	0.12411438	-4.549987	5.36E-06	2.13E-05
ENSG00000177469	CAVIN1	52846.33287	63061.477	42631.18844	-0.564958742	0.022080155	-25.58672	2.14E-144	1.11E-142
ENSG00000029639	TFB1M	370.2049351	441.79923	298.6106388	-0.565111571	0.127288698	-4.4396052	9.01E-06	3.50E-05
ENSG00000177464	GPR4	1326.952716	1583.9294	1069.976074	-0.565174685	0.069423615	-8.1409573	3.92E-16	3.02E-15
ENSG00000173581	CCDC106	472.3843264	563.26197	381.5066807	-0.56575816	0.112218459	-5.0415784	4.62E-07	2.04E-06
ENSG00000111252	SH2B3	10616.93666	12674.046	8559.827047	-0.565970862	0.034372132	-16.465981	6.44E-61	1.44E-59
ENSG00000115380	EFEMP1	120632.0062	144024.29	97239.72316	-0.566695587	0.021212815	-26.714776	3.17E-157	1.83E-155
ENSG00000091490	SEL1L3	4999.65269	5970.1839	4029.12145	-0.56765771	0.036628603	-15.497662	3.60E-54	7.24E-53
ENSG00000148090	AUH	162.4294039	193.95388	130.9049283	-0.568069611	0.177790795	-3.1951576	0.001398	0.004026
ENSG00000183779	ZNF703	504.0822541	602.19941	405.9650997	-0.569102671	0.114648057	-4.9639103	6.91E-07	3.00E-06
ENSG00000059804	SLC2A3	2559.063175	3057.5868	2060.539517	-0.569166732	0.04530176	-12.563899	3.33E-36	4.72E-35
ENSG00000267583	AC007998.3	432.7715863	516.87426	348.6689161	-0.569401962	0.119119197	-4.7801024	1.75E-06	7.33E-06
ENSG00000124831	LRRFIP1	5864.561448	7008.3258	4720.79708	-0.570080445	0.034534708	-16.507464	3.24E-61	7.30E-60
ENSG00000204371	EHMT2	2568.943719	3070.1978	2067.689612	-0.570131745	0.046626537	-12.227624	2.21E-34	3.00E-33
ENSG00000136169	SETDB2	287.6339552	343.9852	231.2827098	-0.570543274	0.130395191	-4.3754932	1.21E-05	4.64E-05
ENSG00000082515	MRPL22	1617.1327	1932.9854	1301.280028	-0.57083852	0.059463351	-9.5998378	8.01E-22	7.73E-21
ENSG00000068137	PLEKHH3	328.4470565	392.59655	264.2975662	-0.570913494	0.121957852	-4.681236	2.85E-06	1.17E-05
ENSG00000128923	MINDY2	1058.410528	1266.071	850.7500443	-0.571511722	0.08354319	-6.8409133	7.87E-12	4.94E-11
ENSG00000228716	DHFR	1582.210103	1891.5814	1272.838765	-0.571750829	0.055590885	-10.284974	8.24E-25	8.82E-24
ENSG00000130584	ZBTB46	273.1278202	326.69883	219.5568071	-0.572529667	0.151597722	-3.7766377	0.000159	0.000532
ENSG00000165626	BEND7	744.7699767	890.39036	599.1495978	-0.572747424	0.080302624	-7.1323625	9.87E-13	6.50E-12
ENSG00000164105	SAP30	389.8645818	466.19545	313.5337175	-0.572966987	0.124161095	-4.6147063	3.94E-06	1.58E-05
ENSG00000173402	DAG1	6483.355693	7754.3938	5212.317548	-0.573106053	0.038747053	-14.790959	1.68E-49	3.12E-48
ENSG00000109501	WFS1	3585.505099	4289.2445	2881.765654	-0.573174133	0.045986541	-12.463954	1.17E-35	1.65E-34
ENSG00000166508	MCM7	5054.299243	6045.1044	4063.494056	-0.573888291	0.038559112	-14.883338	4.23E-50	7.96E-49
ENSG00000168061	SAC3D1	483.4843119	578.10674	388.8618822	-0.57392045	0.119491127	-4.8030382	1.56E-06	6.58E-06
ENSG00000105655	ISYNA1	1148.250424	1373.5486	922.9522532	-0.57424183	0.070039853	-8.1987869	2.43E-16	1.88E-15
ENSG00000160712	IL6R	504.7538404	604.3915	405.1161844	-0.574902499	0.11041018	-5.20697	1.92E-07	8.78E-07
ENSG00000103034	NDRG4	2945.647477	3525.8531	2365.441872	-0.575588753	0.044282844	-12.998008	1.26E-38	1.91E-37
ENSG00000143314	MRPL24	1234.723651	1478.2695	991.1778249	-0.575593991	0.068488985	-8.4041834	4.31E-17	3.46E-16
ENSG00000130433	CACNG6	178.0031871	213.22149	142.7848824	-0.575820432	0.169063747	-3.4059368	0.000659	0.002014
ENSG00000148175	STOM	7913.871785	9474.9899	6352.753683	-0.576189633	0.031316969	-18.39864	1.35E-75	3.73E-74
ENSG00000160298	C21orf58	206.8437332	247.55785	166.1296202	-0.576905126	0.147381255	-3.9143725	9.06E-05	0.000313
ENSG00000138640	FAM13A	345.1377042	413.14949	277.1259225	-0.577038556	0.120459416	-4.7903151	1.67E-06	6.99E-06
ENSG00000132326	PER2	132.0054134	157.97187	106.0389573	-0.577319238	0.193688647	-2.9806561	0.002876	0.007836
ENSG00000110880	CORO1C	9250.359169	11079.078	7421.640546	-0.577667118	0.028784806	-20.068474	1.39E-89	4.76E-88
ENSG00000154310	TNIK	3195.963072	3826.9276	2564.998568	-0.577691742	0.044208606	-13.067405	5.06E-39	7.73E-38
ENSG00000102010	BMX	6737.433705	8068.693	5406.174414	-0.577969188	0.031610884	-18.283867	1.11E-74	3.02E-73
ENSG00000109046	WSB1	7209.230093	8636.5712	5781.888939	-0.578849707	0.076801531	-7.5369553	4.81E-14	3.38E-13
ENSG00000146410	MTFR2	195.1505014	233.48311	156.8178934	-0.579719194	0.170671621	-3.3966935	0.000682	0.002078
ENSG00000178718	RPP25	1733.755575	2076.6913	1390.819822	-0.580053685	0.059851957	-9.6914741	3.28E-22	3.21E-21
ENSG00000133433	GSTT2B	209.0058856	250.46375	167.5480179	-0.580320192	0.149014977	-3.8943749	9.85E-05	0.000339
ENSG00000124588	NQO2	522.2120451	626.16973	418.2543577	-0.581024169	0.100548304	-5.7785576	7.53E-09	3.87E-08
ENSG00000125871	MGME1	638.4113401	765.23787	511.5848097	-0.581044351	0.08564232	-6.7845471	1.16E-11	7.21E-11
ENSG00000197417	SHPK	134.3872225	161.2269	107.5475416	-0.581857636	0.190108894	-3.0606545	0.002209	0.006166
ENSG00000139637	C12orf10	1044.655718	1252.6842	836.6272473	-0.58188846	0.068600507	-8.4822764	2.21E-17	1.79E-16
ENSG00000060140	STYK1	488.4095767	585.63524	391.183915	-0.582624617	0.104389528	-5.5812554	2.39E-08	1.18E-07
ENSG00000277476	AC005332.5	82.47198979	98.864109	66.07987011	-0.582999058	0.233653505	-2.4951436	0.012591	0.029921
ENSG00000228203	RNF144A-AS1	81.93101679	98.165947	65.6960866	-0.583057086	0.245326199	-2.3766605	0.01747	0.040017
ENSG00000133243	BTBD2	3617.622266	4339.5934	2895.651091	-0.583605253	0.04113259	-14.188391	1.08E-45	1.89E-44
ENSG00000101665	SMAD7	403.2101511	483.5396	322.8807035	-0.583656798	0.109412348	-5.3344692	9.58E-08	4.50E-07
ENSG00000127423	AUNIP	98.61288117	118.32767	78.89809556	-0.584307722	0.221793473	-2.6344676	0.008427	0.020778
ENSG00000146674	IGFBP3	173.2490782	207.98559	138.5125715	-0.585784429	0.178030906	-3.2903525	0.001001	0.002963
ENSG00000101945	SUV39H1	640.2592736	768.46523	512.0533165	-0.585888145	0.090006619	-6.5093895	7.55E-11	4.46E-10
ENSG00000165879	FRAT1	176.7671858	212.10051	141.4338592	-0.586046701	0.172782088	-3.3918255	0.000694	0.00211
ENSG00000121060	TRIM25	5269.284741	6325.6737	4212.895741	-0.58609486	0.034657771	-16.910922	3.74E-64	8.85E-63
ENSG00000183621	ZNF438	481.9189361	578.67415	385.1637214	-0.586107274	0.099365765	-5.898483	3.67E-09	1.93E-08
ENSG00000110844	PRPF40B	151.4072101	181.89556	120.9188627	-0.586110997	0.177248704	-3.3067153	0.000944	0.002806
ENSG00000112367	FIG4	571.7624127	686.98792	456.5369084	-0.586527965	0.097286891	-6.0288489	1.65E-09	8.88E-09
ENSG00000025772	TOMM34	1947.199344	2338.3731	1556.02554	-0.586590026	0.053641812	-10.935313	7.81E-28	9.11E-27
ENSG00000139405	RITA1	794.4189985	954.12862	634.7093753	-0.586724556	0.080514915	-7.2871537	3.17E-13	2.14E-12
ENSG00000101868	POLA1	1104.747109	1325.6167	883.8773992	-0.586733016	0.073590326	-7.972964	1.55E-15	1.16E-14
ENSG00000010292	NCAPD2	4353.020855	5226.4107	3479.631018	-0.587238563	0.038464518	-15.26702	1.27E-52	2.49E-51
ENSG00000138738	PRDM5	170.4521327	204.465	136.4392691	-0.587834608	0.168201074	-3.4948327	0.000474	0.001479
ENSG00000182919	C11orf54	218.9705892	262.70894	175.2322394	-0.587946564	0.149114697	-3.9429149	8.05E-05	0.000279
ENSG00000104154	SLC30A4	397.3100365	477.51563	317.104441	-0.588154381	0.127519241	-4.6122795	3.98E-06	1.60E-05
ENSG00000163638	ADAMTS9	3823.525237	4595.5463	3051.50419	-0.590715126	0.044595909	-13.245949	4.76E-40	7.47E-39
ENSG00000189343	RPS2P46	348.3820278	418.43196	278.332098	-0.59173631	0.121391134	-4.8746254	1.09E-06	4.65E-06
ENSG00000133195	SLC39A11	244.1600647	293.71551	194.6046213	-0.591859729	0.140695395	-4.2066745	2.59E-05	9.59E-05
ENSG00000172819	RARG	632.9249408	760.81367	505.0362159	-0.592270048	0.091341661	-6.4841173	8.93E-11	5.24E-10
ENSG00000102882	MAPK3	4006.990282	4818.9088	3195.071748	-0.592515466	0.041362624	-14.3249	1.53E-46	2.71E-45
ENSG00000172197	MBOAT1	68.99454042	82.894067	55.09501403	-0.593263836	0.257143709	-2.3071295	0.021048	0.047287
ENSG00000198157	HMGN5	130.6470458	157.04894	104.2451522	-0.59416239	0.190766367	-3.1146077	0.001842	0.005211

ENSG00000227051	C14orf132	124.9846361	150.54244	99.42682904	-0.594231046	0.195608183	-3.0378639	0.002383	0.006611
ENSG00000142731	PLK4	722.5061882	869.03446	575.9779157	-0.595208841	0.08980925	-6.6274781	3.41E-11	2.05E-10
ENSG00000087086	FTL	43258.17241	52058.355	34457.98976	-0.595214223	0.03243553	-18.350686	3.26E-75	8.96E-74
ENSG00000177981	ASB8	912.0538229	1098.1704	725.937226	-0.59738168	0.080441969	-7.4262438	1.12E-13	7.74E-13
ENSG00000168393	DTYMK	1366.513814	1645.4096	1087.61798	-0.597562807	0.063015685	-9.4827629	2.48E-21	2.35E-20
ENSG00000136936	XPA	399.3495017	481.12721	317.5717897	-0.598003919	0.107879991	-5.5432329	2.97E-08	1.46E-07
ENSG00000092929	UNC13D	144.2070855	173.94091	114.4732654	-0.598082065	0.194298304	-3.0781641	0.002083	0.005839
ENSG00000170915	PAQR8	675.2086606	813.17034	537.2469856	-0.598271122	0.081946738	-7.3007314	2.86E-13	1.94E-12
ENSG00000146281	PM20D2	406.6798129	490.02694	323.3326863	-0.598322768	0.110592437	-5.4101599	6.30E-08	3.00E-07
ENSG00000172458	IL17D	249.6990813	300.93888	198.4592815	-0.598971537	0.147070861	-4.0726731	4.65E-05	0.000167
ENSG00000234741	GAS5	2015.002908	2428.5868	1601.419057	-0.599554019	0.057990021	-10.338917	4.70E-25	5.06E-24
ENSG00000267508	ZNF285	111.1840691	133.9705	88.39764154	-0.599631522	0.214247848	-2.798775	0.00513	0.013254
ENSG00000053438	NNAT	132.9740533	160.21579	105.7323165	-0.599806114	0.204124459	-2.9384353	0.003299	0.008871
ENSG00000185252	ZNF74	504.7281272	608.46247	400.9937809	-0.600784337	0.099887592	-6.0146043	1.80E-09	9.65E-09
ENSG00000158467	AHCYL2	823.3499199	992.75107	653.9487658	-0.601391509	0.078762129	-7.6355416	2.25E-14	1.61E-13
ENSG00000137393	RNF144B	3863.61851	4658.5591	3068.677891	-0.601962263	0.043169481	-13.944162	3.41E-44	5.79E-43
ENSG00000138080	EMILIN1	163.3499568	197.00461	129.6953048	-0.602621028	0.173895031	-3.4654298	0.000529	0.001639
ENSG00000187098	MITF	109.9521947	132.67591	87.22848005	-0.60305052	0.21414323	-2.8161083	0.004861	0.012613
ENSG00000171848	RRM2	5454.948791	6579.0106	4330.887016	-0.6034257	0.033973718	-17.761544	1.40E-70	3.62E-69
ENSG00000144647	POMGNT2	319.7401192	385.39655	254.0836843	-0.603446479	0.127126696	-4.7468116	2.07E-06	8.59E-06
ENSG00000115109	EPB41L5	1633.133897	1970.5907	1295.677055	-0.604592531	0.056838432	-10.637037	2.00E-26	2.26E-25
ENSG00000145916	RMND5B	858.3888479	1035.3429	681.4348061	-0.604702779	0.074996019	-8.0631317	7.44E-16	5.66E-15
ENSG00000278619	MRM1	123.1275131	148.66958	97.58544623	-0.605226081	0.218526029	-2.7695835	0.005613	0.014369
ENSG00000070610	GBA2	2564.132347	3093.8129	2034.451768	-0.60560557	0.048772554	-12.416934	2.12E-35	2.95E-34
ENSG00000167733	HSD11B1L	71.95944703	87.021365	56.89752861	-0.605711924	0.26153119	-2.3160217	0.020557	0.046292
ENSG00000058804	NDC1	1691.827664	2041.9703	1341.685015	-0.606554999	0.055865849	-10.857349	1.84E-27	2.13E-26
ENSG00000159055	MIS18A	464.3697699	560.60815	368.131385	-0.606697384	0.100221343	-6.0535747	1.42E-09	7.66E-09
ENSG00000107282	APBA1	350.3359409	423.20893	277.4629531	-0.607151093	0.132028289	-4.5986439	4.25E-06	1.70E-05
ENSG00000159388	BTG2	3200.20814	3864.2661	2536.150142	-0.607197113	0.049137964	-12.356986	4.47E-35	6.17E-34
ENSG00000234664	HMGN2P5	285.024566	344.21416	225.8349758	-0.608022672	0.131669807	-4.6177836	3.88E-06	1.56E-05
ENSG00000092096	SLC22A17	78.83664488	95.268999	62.40429026	-0.608536803	0.258834937	-2.3510613	0.01872	0.042541
ENSG00000010361	FUZ	293.4792685	354.26846	232.6900817	-0.60889524	0.126583038	-4.8102435	1.51E-06	6.35E-06
ENSG00000100162	CENPM	285.7508458	345.14004	226.3616534	-0.609121774	0.128561267	-4.7379883	2.16E-06	8.96E-06
ENSG00000170390	DCLK2	115.5746131	139.43476	91.7144668	-0.60973798	0.202981462	-3.0039097	0.002665	0.007312
ENSG00000065060	UHRF1BP1	1059.843944	1280.6071	839.0807829	-0.609858507	0.070500805	-8.6503766	5.13E-18	4.28E-17
ENSG00000160447	PKN3	1414.913795	1709.5743	1120.253256	-0.610623754	0.067892871	-8.9939304	2.39E-19	2.09E-18
ENSG00000167900	TK1	1824.521553	2204.8674	1444.175722	-0.611242694	0.05482641	-11.148691	7.27E-29	8.79E-28
ENSG00000095713	CRTAC1	263.0980639	318.02038	208.1757496	-0.611462135	0.136648347	-4.474713	7.65E-06	2.99E-05
ENSG00000154783	FGD5	12062.46736	14582.2	9542.734642	-0.611918798	0.029148802	-20.992931	7.61E-98	2.82E-96
ENSG00000112514	CUTA	1378.746149	1667.3973	1090.094965	-0.61253217	0.061076173	-10.028987	1.14E-23	1.17E-22
ENSG00000144182	LIPT1	104.5138339	126.47611	82.55155942	-0.61257594	0.21113594	-2.9013343	0.003716	0.009883
ENSG00000137411	VARS2	1090.900197	1318.6383	863.1621041	-0.61316828	0.06858103	-8.9407855	3.86E-19	3.35E-18
ENSG00000100297	MCM5	3469.050817	4196.134	2741.967629	-0.614234471	0.040483289	-15.172544	5.38E-52	1.04E-50
ENSG00000037280	FLT4	4752.627786	5750.9023	3754.353308	-0.614770965	0.037161745	-16.543114	1.79E-61	4.05E-60
ENSG00000118193	KIF14	847.4611158	1025.2827	669.6395431	-0.615041293	0.079590256	-7.7275954	1.10E-14	7.95E-14
ENSG00000178467	P4HTM	442.712329	535.35409	350.0705644	-0.615307635	0.105436043	-5.8358377	5.35E-09	2.78E-08
ENSG00000228672	PROB1	149.2433132	180.45866	118.0279677	-0.615558226	0.199518586	-3.0852175	0.002034	0.00571
ENSG00000100034	PPM1F	7979.767219	9657.4146	6302.119817	-0.615604902	0.030939776	-19.896877	4.33E-88	1.46E-86
ENSG00000013583	HEBP1	1587.040127	1920.7102	1253.370006	-0.615902134	0.058217298	-10.579366	3.71E-26	4.15E-25
ENSG00000182511	FES	1328.704883	1608.2813	1049.128448	-0.616288666	0.070697699	-8.7172379	2.85E-18	2.40E-17
ENSG00000170584	NUDCD2	716.6014757	867.52334	565.6796118	-0.616994829	0.08229712	-7.4971619	6.52E-14	4.56E-13
ENSG00000109805	NCAPG	2583.055448	3128.3246	2037.786308	-0.617278401	0.049885264	-12.373963	3.62E-35	5.01E-34
ENSG00000104147	OIP5	146.7250197	177.4619	115.9881405	-0.617292996	0.1888159	-3.269285	0.001078	0.003171
ENSG00000188404	SELL	220.7559839	267.66849	173.8434739	-0.618531633	0.16135655	-3.8333221	0.000126	0.000429
ENSG00000177873	ZNF619	177.8711045	215.41756	140.3246525	-0.619172066	0.160123166	-3.8668488	0.00011	0.000377
ENSG00000187240	DYNC2H1	469.3534309	568.90225	369.8046138	-0.619633958	0.109306681	-5.6687657	1.44E-08	7.22E-08
ENSG00000129355	CDKN2D	130.7624059	158.32964	103.195175	-0.619644002	0.196582688	-3.1520782	0.001621	0.004623
ENSG00000138650	PCDH10	7824.353877	9480.347	6168.360751	-0.619919837	0.030343986	-20.429743	9.10E-93	3.22E-91
ENSG00000166780	C16orf45	813.6501796	985.3317	641.968658	-0.619954091	0.079643326	-7.7841311	7.02E-15	5.15E-14
ENSG00000080561	MID2	867.9341483	1051.7763	684.0920268	-0.620118577	0.076885472	-8.0654844	7.29E-16	5.55E-15
ENSG00000021645	NRXN3	86.83729592	105.17452	68.5000736	-0.621321512	0.231266711	-2.6866016	0.007218	0.018045
ENSG00000106853	PTGR1	1562.207551	1893.266	1231.149074	-0.621991672	0.057205346	-10.872964	1.55E-27	1.79E-26
ENSG00000124787	RPP40	352.3546056	427.34694	277.3622759	-0.622860203	0.113042915	-5.5099446	3.59E-08	1.75E-07
ENSG00000151468	CCDC3	114.0964365	138.19914	89.99373045	-0.623273658	0.21577779	-2.8884977	0.003871	0.010256
ENSG00000110047	EHD1	6962.972456	8444.459	5481.485876	-0.623490597	0.034036264	-18.318421	5.90E-75	1.61E-73
ENSG00000280832	GSEC	109.8120024	133.18832	86.43568239	-0.623513481	0.203332019	-3.0664796	0.002166	0.006055
ENSG00000256223	ZNF10	70.85716493	85.992686	55.72164347	-0.623680778	0.257476298	-2.4222842	0.015423	0.035892
ENSG00000120913	PDLIM2	310.6804692	376.57027	244.7906663	-0.62418144	0.130290867	-4.7906769	1.66E-06	6.98E-06
ENSG00000144354	CDCA7	609.5437881	739.52383	479.5637456	-0.624790698	0.092319698	-6.7676857	1.31E-11	8.07E-11
ENSG00000224877	NDUFAF8	689.6222594	836.54238	542.702142	-0.624854039	0.19855459	-3.1470138	0.001649	0.0047
ENSG00000008283	CYB561	2330.832129	2829.1887	1832.4756	-0.625461508	0.05239081	-11.938382	7.47E-33	9.86E-32
ENSG00000122592	HOXA7	904.986008	1098.8575	711.114532	-0.626465914	0.079397743	-7.8902232	3.02E-15	2.24E-14
ENSG00000139266	9-Mar	196.659618	238.66264	154.6565921	-0.626681622	0.153238449	-4.0895847	4.32E-05	0.000156
ENSG00000101871	MID1	1673.882583	2032.7992	1314.965977	-0.627202208	0.059198942	-10.594821	3.15E-26	3.52E-25
ENSG00000176105	YES1	6466.846065	7852.2365	5081.45565	-0.627224388	0.033122895	-18.936279	5.73E-80	1.72E-78
ENSG00000101096	NFATC2	1159.234996	1407.8119	910.6580793	-0.62803059	0.073891261	-8.4993893	1.91E-17	1.55E-16
ENSG00000239697	TNFSF12	408.3438915	495.98699	320.7007957	-0.628295243	0.120236639	-5.2254891	1.74E-07	7.97E-07
ENSG00000169184	MN1	421.5087171	511.76384	331.2535945	-0.628306977	0.11159437	-5.6302748	1.80E-08	8.97E-08
ENSG00000149260	CAPN5	1064.856638	1293.0156	836.6976957	-0.628451145	0.075377116	-8.3374262	7.59E-17	6.03E-16
ENSG00000112742	TTK	787.8034136	956.55986	619.0469641	-0.628670898	0.082422135	-7.6274522	2.39E-14	1.71E-13
ENSG00000157978	LDLRAP1	1270.567925	1543.8423	997.2935366	-0.629440963	0.065007576	-9.6825786	3.58E-22	3.49E-21
ENSG00000163590	PPM1L	76.62932037	93.048229	60.21041154	-0.629686181	0.252363702	-2.4951535	0.01259	0.029921
ENSG00000251322	SHANK3	8483.89944	10307.511	6660.288179	-0.629987356	0.039064336	-16.126918	1.65E-58	3.57E-57
ENSG00000119522	DENND1A	811.1412222	985.88011	636.4023308	-0.630283962	0.081538392	-7.7299042	1.08E-14	7.81E-14
ENSG00000152127	MGAT5	6470.044442	7863.468	5076.620931	-0.631191443	0.034069784	-18.526429	1.26E-76	3.56E-75

ENSG00000120278	PLEKHG1	1701.374251	2067.8077	1334.940849	-0.631236229	0.055200059	-11.435427	2.78E-30	3.48E-29
ENSG00000147813	NAPRT	1026.307553	1248.1667	804.448359	-0.632145557	0.07030937	-8.9909148	2.45E-19	2.14E-18
ENSG00000005249	PRKAR2B	2365.161979	2875.7117	1854.612238	-0.632311037	0.049343887	-12.814374	1.36E-37	2.01E-36
ENSG00000173848	NET1	1125.07121	1368.5393	881.6031401	-0.632756323	0.070409781	-8.9867674	2.55E-19	2.22E-18
ENSG00000135842	FAM129A	306.0941232	372.18028	240.0079651	-0.633500547	0.134585339	-4.7070547	2.51E-06	1.03E-05
ENSG00000163344	PMVK	726.2674372	882.75839	569.7764834	-0.634076276	0.08690086	-7.2965477	2.95E-13	2.00E-12
ENSG00000165501	LRR1	545.4278948	663.52804	427.3277453	-0.634356456	0.091464256	-6.9355668	4.05E-12	2.58E-11
ENSG00000196535	MYO18A	1471.522154	1789.3671	1153.677191	-0.634956939	0.065247873	-9.731458	2.21E-22	2.18E-21
ENSG00000229124	VIM-AS1	279.3375866	339.95513	218.7200407	-0.636095257	0.126235717	-5.0389484	4.68E-07	2.06E-06
ENSG00000221866	PLXNA4	3819.709853	4647.9551	2991.46462	-0.636271232	0.042748698	-14.883991	4.19E-50	7.89E-49
ENSG00000173715	C11orf80	345.2426168	420.43005	270.0551872	-0.636301097	0.140119539	-4.5411304	5.60E-06	2.22E-05
ENSG00000250508	AP000808.1	118.4296812	144.07873	92.78063185	-0.63758699	0.199270715	-3.1996021	0.001376	0.003969
ENSG00000168887	C2orf68	641.287788	780.87959	501.6959877	-0.638012728	0.089651035	-7.1166242	1.11E-12	7.27E-12
ENSG00000143013	LMO4	717.3013816	873.45248	561.1502822	-0.638208415	0.086056509	-7.4161551	1.21E-13	8.34E-13
ENSG00000212916	MAP10	124.3781738	151.372	97.38435211	-0.638420145	0.195132686	-3.2717232	0.001069	0.003147
ENSG00000274487	AC244154.1	92.73738648	112.94874	72.5260319	-0.638434199	0.238157215	-2.6807258	0.007346	0.018347
ENSG00000163629	PTPN13	184.5037824	224.45189	144.5556766	-0.638595134	0.16147404	-3.9547851	7.66E-05	0.000267
ENSG00000103024	NME3	742.460358	904.0917	580.8290152	-0.639156059	0.091205012	-7.007905	2.42E-12	1.56E-11
ENSG00000173207	CKS1B	1027.600042	1251.8294	803.3707031	-0.639584513	0.067994964	-9.4063512	5.14E-21	4.81E-20
ENSG00000076382	SPAG5	1319.100815	1606.5204	1031.681205	-0.639640904	0.064040365	-9.9880895	1.72E-23	1.76E-22
ENSG00000240694	PNMA2	891.1887807	1085.5805	696.7970548	-0.639726608	0.075459021	-8.4778016	2.29E-17	1.86E-16
ENSG00000126249	PDCD2L	104.7767129	127.60976	81.94366804	-0.639925785	0.221205805	-2.8928978	0.003817	0.010126
ENSG00000109685	NSD2	5585.744954	6805.4347	4366.055233	-0.640380793	0.032993246	-19.409451	6.42E-84	2.05E-82
ENSG00000171132	PRKCE	1064.867623	1297.6453	832.0899745	-0.64049697	0.069820401	-9.1734931	4.58E-20	4.14E-19
ENSG00000103018	CYB5B	4246.054647	5173.9431	3318.166181	-0.640598222	0.037113931	-17.260317	9.36E-67	2.30E-65
ENSG00000188191	PRKAR1B	1308.204207	1593.9158	1022.492569	-0.640737544	0.06649807	-9.6354307	5.67E-22	5.49E-21
ENSG00000179431	FJX1	1098.672317	1338.6376	858.7070451	-0.640798279	0.160847464	-3.983888	6.78E-05	0.000238
ENSG00000184216	IRAK1	6148.844814	7491.5093	4806.180334	-0.640981015	0.041015142	-15.627912	4.70E-55	9.57E-54
ENSG00000176393	RNPEP	2449.610988	2984.8064	1914.415536	-0.641051419	0.049011968	-13.079487	4.31E-39	6.60E-38
ENSG00000198478	SH3BGRL2	1175.154608	1432.2126	918.0966148	-0.641466901	0.065636794	-9.7729773	1.47E-22	1.46E-21
ENSG00000213672	NCKIPSD	487.7666347	594.69864	380.834629	-0.641802937	0.097016354	-6.6154098	3.71E-11	2.22E-10
ENSG00000007944	MYLIP	545.6232953	665.4611	425.7854928	-0.641936518	0.10547991	-6.0858652	1.16E-09	6.30E-09
ENSG00000185697	MYBL1	127.1079707	155.02196	99.19398236	-0.642043123	0.19895945	-3.2270049	0.001251	0.003635
ENSG00000141401	IMPA2	102.3912602	124.6757	80.10682132	-0.64264384	0.236455411	-2.7178225	0.006571	0.016601
ENSG00000134758	RNF138	880.6399237	1074.4353	686.8445932	-0.643679997	0.09330184	-6.8988993	5.24E-12	3.32E-11
ENSG00000087253	LPCAT2	4043.670167	4933.5524	3153.78798	-0.645524165	0.039301013	-16.425128	1.26E-60	2.82E-59
ENSG00000186088	GSAP	73.23977347	89.408038	57.07150853	-0.645803875	0.260453165	-2.4795394	0.013155	0.03113
ENSG00000148180	GSN	7744.92673	9450.7339	6039.119528	-0.64584082	0.032304321	-19.992397	6.41E-89	2.18E-87
ENSG00000263465	SRSF8	1828.812435	2231.3178	1426.307054	-0.64585198	0.057736819	-11.186137	4.77E-29	5.78E-28
ENSG00000169169	CPT1C	1128.605946	1377.6641	879.5477481	-0.645969466	0.068225884	-9.4680997	2.85E-21	2.70E-20
ENSG00000145349	CAMK2D	1484.719036	1812.2686	1157.169423	-0.646032673	0.063416984	-10.187061	2.27E-24	2.39E-23
ENSG00000147852	VLDLR	124.8232115	152.312	97.33442589	-0.646101279	0.18967571	-3.406347	0.000658	0.002011
ENSG00000235863	B3GALT4	114.2743593	139.43787	89.11084854	-0.646577857	0.200537294	-3.2242275	0.001263	0.003669
ENSG00000104870	FCGRT	4162.409617	5078.8432	3245.976057	-0.646596764	0.038536867	-16.778654	3.50E-63	8.15E-62
ENSG00000114315	HES1	1643.405954	2005.5008	1281.311117	-0.646667258	0.055159086	-11.723676	9.64E-32	1.25E-30
ENSG00000136108	CKAP2	2754.842061	3362.761	2146.923083	-0.646759735	0.046124486	-14.022048	1.14E-44	1.95E-43
ENSG00000181035	SLC25A42	217.6684526	265.89523	169.4416754	-0.647185263	0.159439289	-4.0591329	4.93E-05	0.000176
ENSG00000186350	RXRA	2623.17277	3202.055	2044.290544	-0.647295487	0.043979519	-14.718112	4.93E-49	9.09E-48
ENSG00000138190	EXOC6	5593.067011	6829.0253	4357.108684	-0.6474283	0.036810352	-17.588213	3.03E-69	7.69E-68
ENSG00000158828	PINK1	1520.426902	1855.7953	1185.05852	-0.647810898	0.06214063	-10.424917	1.91E-25	2.08E-24
ENSG00000124019	FAM124B	4812.325869	5876.5108	3748.140917	-0.648558615	0.037608697	-17.24491	1.22E-66	3.00E-65
ENSG00000115896	PLCL1	477.7312416	583.41069	372.0517967	-0.648778108	0.09942216	-6.525488	6.78E-11	4.01E-10
ENSG00000269190	FBXO17	532.0699219	649.95441	414.1854308	-0.649828849	0.100202725	-6.4851415	8.86E-11	5.21E-10
ENSG00000054793	ATP9A	3663.691904	4476.1575	2851.226276	-0.6501931	0.040566507	-16.02783	8.17E-58	1.74E-56
ENSG00000254703	SENCR	284.4179861	347.71225	221.123723	-0.650512232	0.128209871	-5.0738077	3.90E-07	1.73E-06
ENSG00000107872	FBXL15	338.1557458	413.61574	262.6957508	-0.650825213	0.135407345	-4.8064248	1.54E-06	6.47E-06
ENSG00000123472	ATPAF1	885.8291789	1082.5942	689.0641998	-0.6512342	0.072595159	-8.9707662	2.94E-19	2.56E-18
ENSG00000143553	SNAPIN	749.9481425	915.91658	583.9797044	-0.651534742	0.088320115	-7.3769689	1.62E-13	1.11E-12
ENSG00000126602	TRAP1	2725.244291	3330.0357	2120.452913	-0.651604778	0.046159674	-14.116321	3.01E-45	5.21E-44
ENSG00000182185	RAD51B	111.7598101	136.64768	86.87194237	-0.652005213	0.197683887	-3.2982213	0.000973	0.002885
ENSG00000122386	ZNF205	634.2421936	775.31343	493.170958	-0.653469212	0.090838195	-7.1937715	6.30E-13	4.19E-12
ENSG00000168758	SEMA4C	1171.066768	1431.2904	910.8430907	-0.653505394	0.0680368	-9.6051753	7.60E-22	7.35E-21
ENSG00000103319	EEF2K	2695.510284	3296.4742	2094.546411	-0.65430718	0.044000286	-14.870521	5.12E-50	9.62E-49
ENSG00000141971	MVB12A	1059.533475	1296.1345	822.932428	-0.654356681	0.06707022	-9.7562924	1.73E-22	1.72E-21
ENSG00000109062	SLC9A3R1	831.2597455	1016.4363	646.0831769	-0.654362272	0.074957633	-8.7297617	2.55E-18	2.15E-17
ENSG00000153832	FBXO36	101.0279	123.55986	78.49593666	-0.655249429	0.235979558	-2.7767211	0.005491	0.014085
ENSG00000259479	SORD2P	156.9899123	192.23185	121.7479784	-0.656140087	0.169327282	-3.8749815	0.000107	0.000365
ENSG00000180011	ZADH2	1034.903668	1266.3295	803.4778029	-0.656215484	0.071656006	-9.1578574	5.29E-20	4.77E-19
ENSG00000167680	SEMA6B	13261.99417	16227.52	10296.46812	-0.656292875	0.065114464	-10.079064	6.84E-24	7.10E-23
ENSG00000185920	PTCH1	371.8901936	455.40197	288.3784195	-0.656982832	0.115705486	-5.6780612	1.36E-08	6.87E-08
ENSG00000064687	ABCA7	237.0240179	290.06054	183.9874925	-0.657761639	0.137387759	-4.7876291	1.69E-06	7.07E-06
ENSG00000173113	TRMT112	3248.885242	3977.3102	2520.460236	-0.658347225	0.04581965	-14.368229	8.19E-47	1.46E-45
ENSG00000172731	LRRC20	599.6052676	734.18403	465.0265064	-0.658493122	0.092531759	-7.1164012	1.11E-12	7.28E-12
ENSG00000140451	PIF1	192.7019008	236.06509	149.3387084	-0.65878451	0.15185309	-4.3383016	1.44E-05	5.45E-05
ENSG00000129028	THAP10	139.5227114	170.94641	108.0990141	-0.65913473	0.208517345	-3.1610547	0.001572	0.00449
ENSG00000167513	CDT1	712.1338473	872.15805	552.1096444	-0.659470549	0.082746662	-7.9697541	1.59E-15	1.19E-14
ENSG00000263731	AC145207.5	58.5150998	71.653739	45.37646067	-0.659875733	0.281283378	-2.3459464	0.018979	0.043076
ENSG00000229807	XIST	1281.552485	1570.0261	993.0788263	-0.661171771	0.157340268	-4.2021777	2.64E-05	9.77E-05
ENSG00000160813	PPP1R35	425.6143904	521.41282	329.8159644	-0.661721614	0.122153625	-5.4171263	6.06E-08	2.89E-07
ENSG00000149823	VPS51	3532.124522	4330.3664	2733.882682	-0.663415789	0.039242572	-16.905512	4.10E-64	9.67E-63
ENSG00000079616	KIF22	1479.612614	1813.9184	1145.306877	-0.663807836	0.061188448	-10.848581	2.03E-27	2.33E-26
ENSG00000169851	PCDH7	1232.260914	1511.9508	952.5710535	-0.664737559	0.067461459	-9.85359	6.61E-23	6.63E-22
ENSG00000148835	TAF5	300.7783171	368.76753	232.7891064	-0.664751614	0.122090516	-5.4447441	5.19E-08	2.49E-07
ENSG00000138182	KIF20B	1471.077589	1804.2732	1137.881952	-0.665341138	0.066385085	-10.022449	1.21E-23	1.25E-22
ENSG00000174428	GTF2IRD2B	199.4652339	244.70073	154.2297376	-0.665353343	0.170594495	-3.9002041	9.61E-05	0.000331

ENSG00000125648	SLC25A23	1538.815045	1887.5019	1190.128197	-0.665445773	0.061320296	-10.851966	1.95E-27	2.25E-26
ENSG00000160211	G6PD	4304.209753	5280.9975	3327.421957	-0.66619565	0.041229349	-16.158287	9.93E-59	2.16E-57
ENSG00000213015	ZNF580	509.8415713	625.49209	394.1910549	-0.666338136	0.114252581	-5.8321495	5.47E-09	2.84E-08
ENSG00000177054	ZDHHC13	2476.727541	3038.9725	1914.482565	-0.666558852	0.052417759	-12.716279	4.80E-37	6.95E-36
ENSG00000114023	FAM162A	530.2107455	651.13037	409.2911231	-0.667817889	0.100746424	-6.6287006	3.39E-11	2.04E-10
ENSG00000128203	ASPHD2	943.7100108	1158.1327	729.2873218	-0.66817177	0.071939895	-9.2879169	1.57E-20	1.44E-19
ENSG00000120256	LRP11	130.4589131	160.04386	100.8739669	-0.668225018	0.238344199	-2.8036135	0.005053	0.013083
ENSG00000113083	LOX	4456.658891	5471.8842	3441.433568	-0.668257047	0.037294648	-17.91831	8.49E-72	2.24E-70
ENSG00000101773	RBBP8	728.9712684	895.55614	562.386394	-0.668981087	0.085943799	-7.7839366	7.03E-15	5.16E-14
ENSG00000188486	H2AFX	2679.084272	3288.9269	2069.241601	-0.669070254	0.050619206	-13.217715	6.93E-40	1.08E-38
ENSG00000100479	POLE2	263.1113693	323.0239	203.1988389	-0.669427075	0.131659546	-5.0845313	3.69E-07	1.64E-06
ENSG00000165948	IFI27L1	234.2343204	287.43862	181.0300184	-0.669585821	0.140404069	-4.7689916	1.85E-06	7.73E-06
ENSG00000149503	INCENP	2150.331353	2640.2539	1660.408822	-0.669617728	0.050097221	-13.366365	9.51E-41	1.52E-39
ENSG00000168916	ZNF608	1472.566807	1808.9092	1136.224458	-0.670002283	0.060642636	-11.04837	2.23E-28	2.65E-27
ENSG00000102908	NFAT5	3227.09799	3964.4632	2489.732745	-0.670093093	0.044199995	-15.160479	6.46E-52	1.25E-50
ENSG00000180425	C11orf71	91.40947091	112.20435	70.61459272	-0.670148398	0.22013619	-3.0442446	0.002333	0.006485
ENSG00000168672	FAM84B	2374.614572	2917.0466	1832.182532	-0.670821007	0.052190557	-12.853302	8.24E-38	1.22E-36
ENSG00000048740	CELF2	1269.822657	1559.731	979.914302	-0.671027829	0.061166821	-10.970455	5.30E-28	6.23E-27
ENSG00000260027	HOXB7	1131.184841	1389.8815	872.4881323	-0.6712743	0.065789919	-10.2033	1.92E-24	2.03E-23
ENSG00000090447	TFAP4	279.4793995	343.28271	215.6760914	-0.672525767	0.136687514	-4.9201697	8.65E-07	3.72E-06
ENSG00000164649	CDCA7L	2954.245838	3631.5492	2276.942451	-0.672572118	0.045522482	-14.774505	2.14E-49	3.96E-48
ENSG00000122952	ZWINT	1736.421875	2134.5969	1338.246876	-0.674298393	0.061469688	-10.969608	5.35E-28	6.29E-27
ENSG00000169570	DTWD2	100.7028951	123.90749	77.49830429	-0.675011753	0.22092682	-3.0553635	0.002248	0.006263
ENSG00000099256	PRTFDC1	511.0382379	628.5194	393.5570759	-0.67509207	0.09760186	-6.9167951	4.62E-12	2.93E-11
ENSG00000145945	FAM50B	331.6635545	407.66329	255.6638165	-0.675144999	0.121452616	-5.5589169	2.71E-08	1.34E-07
ENSG00000109193	SULT1E1	63.51146393	78.179533	48.84339509	-0.67663715	0.262525626	-2.5774137	0.009954	0.02419
ENSG00000115457	IGFBP2	3600.757413	4432.7231	2768.79169	-0.679495668	0.043293521	-15.695089	1.63E-55	3.35E-54
ENSG00000151414	NEK7	2052.774753	2528.2626	1577.286917	-0.680161383	0.050030956	-13.594811	4.30E-42	7.04E-41
ENSG00000101003	GINS1	737.4095616	907.63772	567.1814023	-0.680177127	0.088876016	-7.653101	1.96E-14	1.40E-13
ENSG00000172244	C5orf34	97.07573355	119.57339	74.5780753	-0.68023771	0.225004675	-3.0232159	0.002501	0.006908
ENSG00000117586	TNFSF4	1718.678219	2116.1327	1321.223758	-0.680287084	0.058513378	-11.62618	3.03E-31	3.88E-30
ENSG00000156970	BUB1B	1254.214663	1544.5363	963.8930747	-0.681050527	0.063599835	-10.708369	9.30E-27	1.05E-25
ENSG00000213465	ARL2	1102.32145	1358.2126	846.4302608	-0.6813465	0.07788427	-8.7481914	2.17E-18	1.83E-17
ENSG00000124006	OBSL1	913.9218437	1126.4587	701.3850201	-0.681362101	0.082145764	-8.2945495	1.09E-16	8.58E-16
ENSG00000136114	THSD1	1491.115149	1837.0482	1145.182065	-0.681566616	0.060546445	-11.256922	2.14E-29	2.62E-28
ENSG00000169679	BUB1	1572.126155	1936.5845	1207.667763	-0.681646427	0.062799322	-10.85436	1.90E-27	2.19E-26
ENSG00000126803	HSPA2	328.0711478	403.82113	252.3211668	-0.68165483	0.124304403	-5.4837545	4.16E-08	2.02E-07
ENSG00000110237	ARHGEF17	7147.791103	8807.021	5488.561214	-0.681766672	0.03468287	-19.657159	5.02E-86	1.65E-84
ENSG00000159314	ARHGAP27	1140.915272	1406.0677	875.762843	-0.683129127	0.072497379	-9.4228114	4.39E-21	4.12E-20
ENSG00000205485	AC004980.1	102.3071352	126.23961	78.37466428	-0.683161798	0.214442069	-3.1857639	0.001444	0.004148
ENSG00000165655	ZNF503	100.7418698	124.15054	77.3331948	-0.683678473	0.221657892	-3.0843859	0.00204	0.005725
ENSG00000147872	PLIN2	1832.206968	2259.1886	1405.225336	-0.684434122	0.060678429	-11.279694	1.65E-29	2.03E-28
ENSG00000111863	ADTRP	383.011166	472.40562	293.6167126	-0.685279051	0.109000554	-6.2869318	3.24E-10	1.83E-09
ENSG00000137804	NUSAP1	105.5443832	130.31297	80.77579634	-0.685659205	0.224394216	-3.0556011	0.002246	0.00626
ENSG00000135451	TROAP	516.2516725	636.9082	395.5951484	-0.686893639	0.093923687	-7.3133164	2.61E-13	1.77E-12
ENSG00000121152	NCAPH	1003.849543	1238.9361	768.7630041	-0.687440001	0.071604612	-9.6004989	7.96E-22	7.69E-21
ENSG00000093072	ADA2	107.4772626	132.49219	82.46233218	-0.688048025	0.228612576	-3.0096683	0.002615	0.007189
ENSG00000007968	E2F2	138.1508637	170.78365	105.5180763	-0.688795728	0.193087031	-3.5672812	0.000361	0.001145
ENSG00000023909	GCLM	1661.134966	2049.4998	1272.770133	-0.688974493	0.058812264	-11.71481	1.07E-31	1.38E-30
ENSG00000067992	PDK3	319.1548852	394.02251	244.2872629	-0.689042381	0.120409785	-5.7224783	1.05E-08	5.33E-08
ENSG00000122359	ANXA11	4974.804183	6141.8485	3807.759894	-0.689802827	0.036232655	-19.038153	8.24E-81	2.51E-79
ENSG00000100027	YPEL1	108.2999744	133.82356	82.77639266	-0.690395047	0.212387859	-3.2506333	0.001151	0.003369
ENSG00000077348	EXOSC5	375.0281579	462.876	287.180319	-0.690998541	0.118020667	-5.8548944	4.77E-09	2.49E-08
ENSG00000204428	LY6G5C	118.0890165	145.86988	90.30815362	-0.691102905	0.199944515	-3.4564734	0.000547	0.001693
ENSG00000075218	GTSE1	1408.094207	1738.9156	1077.272596	-0.691184537	0.065191441	-10.602382	2.90E-26	3.26E-25
ENSG00000092036	HAUS4	303.5621695	374.78987	232.3344711	-0.692313213	0.124056577	-5.5806248	2.40E-08	1.18E-07
ENSG00000138814	PPP3CA	3399.802776	4200.654	2598.951529	-0.692316763	0.043957064	-15.749841	6.89E-56	1.42E-54
ENSG00000170899	GSTA4	502.630049	621.38642	383.8736795	-0.693399959	0.098196231	-7.0613704	1.65E-12	1.07E-11
ENSG00000198130	HIBCH	612.4687949	757.54524	467.3923499	-0.6950455	0.093025114	-7.4715899	7.92E-14	5.52E-13
ENSG00000253368	TRNP1	458.5693256	567.02283	350.1158229	-0.695858114	0.10336873	-6.7318048	1.68E-11	1.03E-10
ENSG00000261308	FIGNL2	156.347836	193.27678	119.4188885	-0.695860986	0.177830586	-3.9130557	9.11E-05	0.000315
ENSG00000188610	FAM72B	108.8294013	134.70019	82.95861708	-0.695947701	0.210403205	-3.3076858	0.000941	0.002797
ENSG00000189046	ALKBH2	168.5193257	208.27079	128.7678604	-0.695990839	0.167901199	-4.1452404	3.39E-05	0.000124
ENSG00000116717	GADD45A	2138.662669	2645.4425	1631.882801	-0.69609358	0.051449467	-13.529656	1.05E-41	1.70E-40
ENSG00000146376	ARHGAP18	4084.039546	5051.6462	3116.432917	-0.696457022	0.038692206	-17.99993	1.95E-72	5.18E-71
ENSG00000124523	SIRT5	344.5997251	426.15329	263.0461615	-0.6968006	0.115878858	-6.0131815	1.82E-09	9.73E-09
ENSG00000183172	SMDT1	484.5680143	599.20772	369.9283076	-0.696868651	0.109430928	-6.3681142	1.91E-10	1.10E-09
ENSG00000135002	RFK	1945.52466	2406.8833	1484.166064	-0.696912102	0.056318252	-12.374534	3.59E-35	4.98E-34
ENSG00000079435	LIPE	183.7225532	227.24904	140.1960628	-0.697561396	0.161831535	-4.310417	1.63E-05	6.16E-05
ENSG00000170264	FAM161A	179.8981217	222.76223	137.0340144	-0.697685209	0.17194067	-4.057709	4.96E-05	0.000177
ENSG00000165406	8-Mar	1915.063288	2369.6085	1460.518099	-0.698993967	0.055718006	-12.545208	4.22E-36	5.97E-35
ENSG00000149564	ESAM	12451.09517	15409.695	9492.495108	-0.699026678	0.026464941	-26.413309	9.64E-154	5.47E-152
ENSG00000138119	MYOF	27107.95786	33577.654	20638.26202	-0.702060836	0.021824866	-32.167933	4.96E-227	4.22E-225
ENSG00000003249	DBNDD1	1408.112027	1743.8064	1072.417627	-0.70259118	0.063250936	-11.107997	1.15E-28	1.37E-27
ENSG00000113368	LMNB1	3249.422718	4024.6819	2474.163538	-0.70275672	0.043572481	-16.128453	1.61E-58	3.49E-57
ENSG00000184481	FOXO4	464.6312583	575.7475	353.5150201	-0.702875514	0.102763996	-6.839706	7.94E-12	4.98E-11
ENSG00000178585	CTNNBIP1	1801.266187	2232.0415	1370.490861	-0.703254597	0.054987449	-12.789366	1.88E-37	2.76E-36
ENSG00000185480	PARPBP	467.5944184	579.66134	355.5274993	-0.703389817	0.107324627	-6.5538529	5.61E-11	3.33E-10
ENSG00000138759	FRAS1	128.2961272	159.20674	97.38551028	-0.705454113	0.192988757	-3.6554156	0.000257	0.000834
ENSG00000186871	ERCC6L	458.0367296	567.94042	348.1330375	-0.705959917	0.099395149	-7.1025591	1.22E-12	8.03E-12
ENSG00000067177	PHKA1	606.6311502	751.90111	461.3611891	-0.705965827	0.089664308	-7.873432	3.45E-15	2.56E-14
ENSG00000170425	ADORA2B	393.2271244	487.47936	298.9748859	-0.706645152	0.114387394	-6.1776489	6.51E-10	3.60E-09
ENSG00000261455	LINC01003	84.04514138	104.24422	63.84606442	-0.706753943	0.249815426	-2.8291045	0.004668	0.012158
ENSG00000121005	CRISPLD1	330.2522987	409.90985	250.5947334	-0.707547987	0.127962	-5.5293602	3.21E-08	1.57E-07
ENSG00000119772	DNMT3A	2154.05353	2672.6508	1635.45627	-0.707779802	0.049562989	-14.28041	2.90E-46	5.11E-45

ENSG00000179456	ZBTB18	979.1062272	1214.9415	743.2709416	-0.708785666	0.079593974	-8.9050167	5.34E-19	4.61E-18
ENSG00000153904	DDAH1	3013.565127	3740.1028	2287.027471	-0.708893064	0.047582602	-14.898157	3.39E-50	6.39E-49
ENSG00000001461	NIPAL3	945.2643149	1173.2439	717.2847032	-0.70905197	0.071184028	-9.9608296	2.26E-23	2.31E-22
ENSG00000123485	HJURP	964.7258167	1196.5354	732.9162131	-0.709738356	0.088648866	-8.0061753	1.18E-15	8.93E-15
ENSG00000137812	KNL1	1895.849745	2353.3431	1438.35642	-0.710176958	0.054970151	-12.91932	3.50E-38	5.25E-37
ENSG00000110077	MS4A6A	128.2808933	159.25971	97.30207958	-0.710635549	0.190565936	-3.7290796	0.000192	0.000635
ENSG00000240342	RPS2P5	2712.250749	3366.1001	2058.40135	-0.710742472	0.054710301	-12.991017	1.38E-38	2.09E-37
ENSG00000137700	SLC37A4	679.8854339	843.66249	516.1083749	-0.710873412	0.087630767	-8.1121441	4.97E-16	3.81E-15
ENSG00000087586	AURKA	1325.081243	1645.0814	1005.081091	-0.712210656	0.063453329	-11.224165	3.10E-29	3.78E-28
ENSG00000178999	AURKB	905.3898147	1124.7231	686.0565684	-0.712726477	0.076852853	-9.2739105	1.79E-20	1.64E-19
ENSG00000166803	PCLAF	239.5902829	297.7662	181.4143664	-0.713888532	0.144613515	-4.9365271	7.95E-07	3.43E-06
ENSG00000092068	SLC7A8	127.9968944	158.80782	97.18596921	-0.714564833	0.219717638	-3.252196	0.001145	0.003353
ENSG00000186364	NUDT17	88.95813384	110.6327	67.28356547	-0.714786931	0.228777245	-3.1243795	0.001782	0.00505
ENSG00000181218	HIST3H2A	85.70818096	106.46096	64.95540271	-0.715453421	0.258247891	-2.7704134	0.005599	0.014335
ENSG00000233016	SNHG7	1380.688488	1716.6885	1044.688478	-0.71656115	0.061487805	-11.653712	2.20E-31	2.82E-30
ENSG00000186153	WWOX	106.6857202	132.70318	80.66826382	-0.717890031	0.228405678	-3.1430481	0.001672	0.004757
ENSG00000163947	ARHGEF3	1145.192511	1424.9691	865.4159055	-0.719036001	0.066523119	-10.808814	3.13E-27	3.58E-26
ENSG00000149476	TKFC	714.1548052	888.32985	539.979758	-0.719167769	0.081280907	-8.8479299	8.92E-19	7.62E-18
ENSG00000166938	DIS3L	1162.857588	1447.507	878.2081456	-0.719381035	0.068418897	-10.514362	7.42E-26	8.22E-25
ENSG00000132970	WASF3	1880.526921	2339.5441	1421.509759	-0.719740091	0.052730443	-13.649422	2.03E-42	3.34E-41
ENSG00000185551	NR2F2	11742.50343	14614.534	8870.472941	-0.720164513	0.065102479	-11.062014	1.92E-28	2.28E-27
ENSG00000088298	EDEM2	2057.338576	2560.6213	1554.055833	-0.721201967	0.050096278	-14.396318	5.46E-47	9.79E-46
ENSG00000143643	TTC13	892.7862595	1111.2255	674.3470145	-0.721895263	0.079793173	-9.0470806	1.47E-19	1.30E-18
ENSG00000090889	KIF4A	1745.299694	2173.4623	1317.137073	-0.72253184	0.056311653	-12.830947	1.10E-37	1.62E-36
ENSG00000126787	DLGAP5	2749.612778	3423.3775	2075.848094	-0.722601583	0.047921476	-15.078867	2.23E-51	4.30E-50
ENSG00000107614	TRDMT1	314.9548135	392.53623	237.3733926	-0.723365547	0.131481761	-5.5016418	3.76E-08	1.83E-07
ENSG00000145147	SLIT2	4214.356332	5249.3098	3179.402853	-0.723735108	0.039364864	-18.385307	1.72E-75	4.76E-74
ENSG00000162733	DDR2	4929.00705	6140.5282	3717.485854	-0.723761452	0.043372762	-16.687004	1.63E-62	3.77E-61
ENSG00000013810	TACC3	3030.275356	3775.7998	2284.75096	-0.725745628	0.046599626	-15.574065	1.09E-54	2.21E-53
ENSG00000134508	CABLES1	3813.638671	4754.3049	2872.972483	-0.726693097	0.043889749	-16.55724	1.42E-61	3.21E-60
ENSG00000151491	EPS8	946.9242252	1179.9962	713.8522647	-0.726937931	0.075257709	-9.6593151	4.49E-22	4.38E-21
ENSG00000198589	LRBA	3314.549673	4133.4247	2495.674605	-0.726951656	0.044774263	-16.235927	2.81E-59	6.16E-58
ENSG00000185187	SIGIRR	886.0855597	1104.7933	667.3778542	-0.727380672	0.075120936	-9.6827957	3.57E-22	3.48E-21
ENSG00000072952	MRVI1	90.61486503	113.20002	68.02971254	-0.728200968	0.252434005	-2.8847182	0.003918	0.010366
ENSG00000120068	HOXB8	52.61790742	65.545761	39.69005418	-0.728968509	0.306479577	-2.3785223	0.017382	0.039851
ENSG00000278771	RN7SL3	163.2123107	203.72244	122.7021771	-0.729303909	0.218123068	-3.3435432	0.000827	0.002484
ENSG00000136367	ZFHX2	117.589197	146.83614	88.34225214	-0.72941425	0.220599939	-3.3065025	0.000945	0.002807
ENSG00000184232	OAF	2306.435953	2877.0773	1735.794625	-0.729448025	0.05483734	-13.302031	2.25E-40	3.57E-39
ENSG00000164985	PSIP1	2979.796708	3719.0186	2240.574808	-0.731332804	0.048762991	-14.997702	7.60E-51	1.45E-49
ENSG00000169750	RAC3	787.4577067	982.69515	592.2202651	-0.731698354	0.07761676	-9.4270665	4.22E-21	3.96E-20
ENSG00000121621	KIF18A	564.6646995	704.84438	424.4850217	-0.732202314	0.092514338	-7.9144739	2.48E-15	1.85E-14
ENSG00000116962	NID1	15229.7636	19017.047	11442.48067	-0.732607091	0.031474098	-23.276508	7.67E-120	3.30E-118
ENSG00000197121	PGAP1	902.2868782	1127.8021	676.7716926	-0.735030999	0.074807755	-9.8255989	8.74E-23	8.72E-22
ENSG00000235109	ZSCAN31	295.0009174	368.33511	221.66672	-0.735748478	0.133949666	-5.4927235	3.96E-08	1.92E-07
ENSG00000105971	CAV2	3523.08394	4404.8873	2641.280534	-0.736998491	0.044327515	-16.626208	4.50E-62	1.03E-60
ENSG00000079691	CARMIL1	181.4084275	226.59262	136.2242334	-0.738695529	0.177402013	-4.1639636	3.13E-05	0.000115
ENSG00000276170	AC244153.1	55.25346925	69.040194	41.46674436	-0.738901021	0.282635282	-2.6143269	0.00894	0.021944
ENSG00000163535	SGO2	1108.442004	1386.8685	830.0154789	-0.740354594	0.076939677	-9.6225331	6.42E-22	6.22E-21
ENSG00000261175	LINC02188	79.00197453	98.808849	59.19509981	-0.740815037	0.237515795	-3.1190138	0.001815	0.005138
ENSG00000144645	OSBPL10	1486.974154	1861.3367	1112.611585	-0.741261762	0.06176443	-12.001434	3.49E-33	4.65E-32
ENSG00000171703	TCEA2	999.6245986	1250.8886	748.3606142	-0.741540388	0.079483114	-9.3295336	1.06E-20	9.81E-20
ENSG00000170917	NUDT6	81.4368379	101.88047	60.99320993	-0.742738771	0.249835358	-2.972913	0.00295	0.008015
ENSG00000204922	UQCC3	429.03187	537.05342	321.0103201	-0.742949139	0.10725471	-6.9269605	4.30E-12	2.74E-11
ENSG00000155849	ELMO1	1327.07323	1660.9981	993.1483561	-0.74332028	0.06550601	-11.34736	7.64E-30	9.45E-29
ENSG00000182378	PLCXD1	258.0560211	323.05371	193.0583298	-0.743472693	0.137162403	-5.4203825	5.95E-08	2.84E-07
ENSG00000143891	GALM	288.7654554	361.8767	215.6542065	-0.743637295	0.131057115	-5.6741467	1.39E-08	7.02E-08
ENSG00000116791	CRYZ	636.5253889	797.07488	475.9759015	-0.743801627	0.088912247	-8.36557	5.98E-17	4.77E-16
ENSG00000166963	MAP1A	379.3236473	474.90587	283.7414259	-0.743827194	0.116265155	-6.3976795	1.58E-10	9.10E-10
ENSG00000084693	AGBL5	793.8400863	993.84869	593.8314831	-0.7442656	0.078648162	-9.4632294	2.99E-21	2.82E-20
ENSG00000104823	ECH1	731.4282468	916.06911	546.7873856	-0.744286239	0.082479325	-9.0239129	1.81E-19	1.60E-18
ENSG00000077063	CTTNBP2	102.5570947	128.71685	76.39734212	-0.744788851	0.24439407	-3.0474915	0.002308	0.00642
ENSG00000136928	GABBR2	2306.416737	2889.2152	1723.618257	-0.744858427	0.050702418	-14.690787	7.39E-49	1.36E-47
ENSG00000158711	ELK4	6311.832558	7909.9716	4713.693492	-0.74592536	0.046612664	-16.002633	1.22E-57	2.59E-56
ENSG00000237649	KIFC1	1456.738777	1825.3113	1088.16621	-0.746805237	0.058753121	-12.710903	5.14E-37	7.43E-36
ENSG00000121691	CAT	1783.754072	2236.419	1331.089171	-0.74819205	0.053815938	-13.9028	6.09E-44	1.03E-42
ENSG00000215068	AC025171.2	102.0376078	128.20025	75.87496957	-0.748280854	0.236349411	-3.1659941	0.001546	0.004419
ENSG00000142892	PIGK	1294.642317	1623.2914	965.9932602	-0.749237332	0.065989349	-11.353913	7.09E-30	8.78E-29
ENSG00000177666	PNPLA2	3498.822235	4389.0573	2608.587152	-0.750689575	0.045117723	-16.638463	3.67E-62	8.41E-61
ENSG00000137135	ARHGEF39	87.28375832	109.47968	65.08783254	-0.751487872	0.226638556	-3.3157989	0.000914	0.002721
ENSG00000163364	LINC01116	335.0098209	420.22562	249.7940246	-0.751758208	0.121071033	-6.2092326	5.32E-10	2.96E-09
ENSG00000108387	4-Sep	98.1746384	123.30703	73.04224523	-0.752024656	0.214152744	-3.5116275	0.000445	0.001393
ENSG00000105767	CADM4	316.8135685	397.43417	236.1929714	-0.752435795	0.121244782	-6.2059231	5.44E-10	3.02E-09
ENSG00000138028	CGREF1	89.57371816	112.55079	66.59664785	-0.753406949	0.240483908	-3.1328789	0.001731	0.004915
ENSG00000170522	ELOVL6	1082.014596	1358.671	805.3581562	-0.754037574	0.068986305	-10.93025	8.26E-28	9.62E-27
ENSG00000110318	CEP126	1115.94021	1401.5046	830.3758595	-0.754140949	0.067518568	-11.169386	5.76E-29	6.97E-28
ENSG00000162520	SYNC	359.4219018	451.98953	266.8542706	-0.755487796	0.132047603	-5.7213291	1.06E-08	5.36E-08
ENSG00000167632	TRAPPC9	972.9658881	1222.7605	723.1712767	-0.756080175	0.080969593	-9.3378285	9.83E-21	9.10E-20
ENSG00000156398	SFXN2	253.2138847	318.22488	188.2028932	-0.757857961	0.134345358	-5.6411176	1.69E-08	8.45E-08
ENSG00000168765	GSTM4	111.2935247	140.17222	82.41482458	-0.758682745	0.218282957	-3.4756847	0.00051	0.001581
ENSG00000187764	SEMA4D	392.9567073	493.94815	291.9652665	-0.760421794	0.118420665	-6.4213606	1.35E-10	7.84E-10
ENSG00000005882	PDK2	686.3223845	863.14816	509.4966093	-0.76052873	0.086029792	-8.8402948	9.55E-19	8.15E-18
ENSG00000187239	FNBP1	2440.108066	3068.7973	1811.418834	-0.760530871	0.048466788	-15.691794	1.72E-55	3.52E-54
ENSG00000089820	ARHGAP4	657.9897836	828.15003	487.8295369	-0.761879217	0.088854944	-8.5744156	9.96E-18	8.20E-17
ENSG00000115525	ST3GAL5	577.0444199	725.52618	428.562661	-0.761990567	0.099627972	-7.6483597	2.04E-14	1.46E-13
ENSG00000170962	PDGFD	683.638137	860.06757	507.2087068	-0.762131287	0.09241637	-8.246713	1.63E-16	1.27E-15

ENSG00000109674	NEIL3	172.4463254	216.80519	128.0874647	-0.7626879	0.167913845	-4.5421383	5.57E-06	2.21E-05
ENSG00000124570	SERPINB6	4742.718452	5967.8804	3517.556481	-0.762897482	0.036117563	-21.122618	4.93E-99	1.85E-97
ENSG00000286169	AHRR	268.1673625	337.50898	198.8257484	-0.763629683	0.142747231	-5.3495236	8.82E-08	4.15E-07
ENSG00000186106	ANKRD46	407.3980297	513.18001	301.6160504	-0.764779168	0.127677188	-5.9899437	2.10E-09	1.12E-08
ENSG00000120802	TMPO	4833.556473	6085.7688	3581.344127	-0.765154629	0.038846414	-19.696918	2.29E-86	7.57E-85
ENSG00000277013	AC008556.1	91.3496301	114.89157	67.80769279	-0.765474983	0.229031914	-3.3422197	0.000831	0.002494
ENSG00000120693	SMAD9	426.7006883	537.20713	316.194246	-0.76603299	0.106948275	-7.1626493	7.91E-13	5.24E-12
ENSG00000244509	APOBEC3C	689.3864561	868.97515	509.7977576	-0.769467479	0.082647921	-9.3101856	1.28E-20	1.18E-19
ENSG00000133687	TMTC1	406.3285748	512.048	300.609147	-0.770229701	0.107679048	-7.1530136	8.49E-13	5.61E-12
ENSG00000107438	PDLIM1	3149.563862	3971.1776	2327.950121	-0.770816865	0.043565082	-17.693456	4.71E-70	1.21E-68
ENSG00000099219	ERMP1	2250.39337	2838.4369	1662.349849	-0.77102982	0.051810356	-14.88177	4.33E-50	8.14E-49
ENSG00000138376	BARD1	296.0277152	373.49086	218.5645678	-0.772568122	0.139957385	-5.520024	3.39E-08	1.66E-07
ENSG00000027075	PRKCH	3800.387674	4795.4231	2805.352241	-0.772630701	0.040476856	-19.088209	3.16E-81	9.73E-80
ENSG00000125910	S1PR4	41.18533415	51.927648	30.44302005	-0.774024104	0.327051157	-2.3666759	0.017949	0.040965
ENSG00000237854	LINC00674	182.6366042	230.62695	134.6462595	-0.775834588	0.162860425	-4.7638006	1.90E-06	7.92E-06
ENSG00000076770	MBNL3	187.0967685	236.13269	138.0608491	-0.775890082	0.160161931	-4.8444101	1.27E-06	5.38E-06
ENSG00000125454	SLC25A19	340.4337652	429.71049	251.1570357	-0.776751004	0.133881316	-5.8017879	6.56E-09	3.38E-08
ENSG00000011426	ANLN	4522.714843	5713.5006	3331.929048	-0.778304924	0.041762388	-18.636504	1.63E-77	4.66E-76
ENSG00000128709	HOXD9	143.8687391	181.93848	105.798995	-0.779376314	0.197117995	-3.9538567	7.69E-05	0.000268
ENSG00000232859	LYRM9	47.78290835	60.439123	35.12669367	-0.779471156	0.328347609	-2.3739206	0.0176	0.040275
ENSG00000100918	REC8	358.1125879	452.64615	263.5790276	-0.779815875	0.117443959	-6.6398977	3.14E-11	1.89E-10
ENSG00000119139	TJP2	109.0214926	137.84263	80.20035357	-0.779845723	0.235865389	-3.3063169	0.000945	0.002809
ENSG00000176890	TYMS	4156.507032	5253.9908	3059.023311	-0.781224874	0.041382627	-18.878088	1.73E-79	5.11E-78
ENSG00000188153	COL4A5	3676.532273	4648.6133	2704.451236	-0.781668735	0.040882818	-19.119737	1.73E-81	5.36E-80
ENSG00000147459	DOCK5	719.202306	909.44546	528.9591484	-0.781699734	0.081642075	-9.5747166	1.02E-21	9.84E-21
ENSG00000214063	TSPAN4	1772.110358	2241.4436	1302.777089	-0.782909838	0.060152031	-13.015518	9.99E-39	1.52E-37
ENSG00000104177	MYEF2	1133.773185	1434.1409	833.4054344	-0.783224454	0.070713451	-11.076032	1.64E-28	1.95E-27
ENSG00000176974	SHMT1	785.6275747	994.3542	576.9009528	-0.783702311	0.085026349	-9.21717	3.05E-20	2.77E-19
ENSG00000077782	FGFR1	5097.012661	6449.6042	3744.421169	-0.783951227	0.035431936	-22.125555	1.79E-108	7.13E-107
ENSG00000134690	CDCA8	1242.099243	1571.2234	912.9750581	-0.784038194	0.062748479	-12.494935	7.96E-36	1.12E-34
ENSG00000273149	AL138963.3	648.6556322	820.59643	476.7148306	-0.784058302	0.251532158	-3.1171295	0.001826	0.005169
ENSG00000144677	CTDSPL	1874.01151	2371.1367	1376.88633	-0.784092636	0.055938333	-14.01709	1.23E-44	2.09E-43
ENSG00000089685	BIRC5	1941.223009	2455.3314	1427.114619	-0.784366418	0.058607858	-13.383298	7.57E-41	1.21E-39
ENSG00000162591	MEGF6	3505.661706	4437.6629	2573.660528	-0.785099412	0.042730798	-18.373151	2.16E-75	5.93E-74
ENSG00000143416	SELENBP1	337.9679134	427.45502	248.4808108	-0.785129142	0.122640053	-6.4018983	1.53E-10	8.86E-10
ENSG00000099204	ABLIM1	11126.03795	14083.719	8168.356599	-0.785434552	0.028721159	-27.346896	1.18E-164	6.97E-163
ENSG00000050438	SLC4A8	336.483141	425.94602	247.0202664	-0.785480267	0.121961506	-6.4403949	1.19E-10	6.93E-10
ENSG00000109654	TRIM2	851.0619685	1077.1123	625.0116074	-0.785544993	0.078739326	-9.9765268	1.93E-23	1.97E-22
ENSG00000088325	TPX2	4490.159562	5686.2141	3294.105043	-0.787998815	0.040444125	-19.483641	1.51E-84	4.87E-83
ENSG00000168209	DDIT4	3078.974196	3898.9347	2259.013729	-0.788165363	0.043046538	-18.309611	6.94E-75	1.89E-73
ENSG00000161800	RACGAP1	1245.526753	1577.5306	913.5229217	-0.789234862	0.064011625	-12.329555	6.28E-35	8.63E-34
ENSG00000138160	KIF11	2127.03546	2695.3277	1558.743245	-0.789738485	0.051168584	-15.43405	9.66E-54	1.93E-52
ENSG00000114779	ABHD14B	517.9705432	656.81753	379.1235585	-0.790187897	0.102194444	-7.7322002	1.06E-14	7.67E-14
ENSG00000189060	H1F0	4601.621021	5832.521	3370.721041	-0.791315915	0.037247634	-21.24473	3.69E-100	1.39E-98
ENSG00000205090	TMEM240	71.78523826	90.973765	52.59671139	-0.792949728	0.261867085	-3.0280618	0.002461	0.006806
ENSG00000071282	LMCD1	358.0497848	454.10491	261.9946634	-0.794414227	0.119329293	-6.6573279	2.79E-11	1.69E-10
ENSG00000162522	KIAA1522	1395.611089	1771.4009	1019.821316	-0.795617745	0.061729223	-12.888835	5.20E-38	7.76E-37
ENSG00000146147	MLIP	279.3176531	354.35961	204.2756992	-0.795747064	0.126688499	-6.281131	3.36E-10	1.90E-09
ENSG00000072210	ALDH3A2	1146.937077	1456.4029	837.4712875	-0.796998043	0.072005796	-11.068526	1.78E-28	2.12E-27
ENSG00000173535	TNFRSF10C	2059.914899	2614.652	1505.177773	-0.797072321	0.051220369	-15.561628	1.33E-54	2.68E-53
ENSG00000072571	HMMR	1078.873565	1369.3954	788.3517759	-0.798152803	0.069397805	-11.501125	1.30E-30	1.65E-29
ENSG00000175063	UBE2C	931.2364309	1182.4027	680.0701424	-0.798981221	0.077584696	-10.298181	7.18E-25	7.70E-24
ENSG00000112379	ARFGEF3	1185.85621	1506.1947	865.5177437	-0.799987821	0.066267316	-12.072133	1.48E-33	1.98E-32
ENSG00000130307	USHBP1	321.3419819	408.45179	234.2321704	-0.801001364	0.127782578	-6.2684708	3.65E-10	2.05E-09
ENSG00000123179	EBPL	805.4061148	1023.698	587.1142437	-0.801892832	0.213817849	-3.750355	0.000177	0.000587
ENSG00000171115	GIMAP8	8438.173214	10726.316	6150.030789	-0.802144988	0.032172685	-24.932485	3.31E-137	1.62E-135
ENSG00000155085	AK9	103.2168436	131.33536	75.09833039	-0.802247907	0.252229783	-3.1806232	0.00147	0.004217
ENSG00000124785	NRN1	919.8003839	1169.5154	670.0853374	-0.802308761	0.074563385	-10.76009	5.31E-27	6.04E-26
ENSG00000081320	STK17B	707.402832	899.12584	515.6798243	-0.802698247	0.103977249	-7.7199412	1.16E-14	8.41E-14
ENSG00000114738	MAPKAPK3	953.9458203	1212.6607	695.2309666	-0.803437878	0.073096097	-10.991529	4.20E-28	4.94E-27
ENSG00000100490	CDKL1	186.3300067	236.55354	136.1064705	-0.803655014	0.175090952	-4.5899289	4.43E-06	1.77E-05
ENSG00000075702	WDR62	425.7509518	540.90818	310.593721	-0.803690868	0.113760409	-7.0647678	1.61E-12	1.05E-11
ENSG00000175806	MSRA	471.1136634	599.16002	343.067303	-0.803902076	0.114872144	-6.9982334	2.59E-12	1.67E-11
ENSG00000186642	PDE2A	1352.441632	1720.0856	984.7976834	-0.805358435	0.070349913	-11.447895	2.41E-30	3.02E-29
ENSG00000079308	TNS1	3150.613304	4007.985	2293.241646	-0.80584434	0.043741835	-18.422737	8.63E-76	2.41E-74
ENSG00000136161	RCBTB2	526.6831309	670.32077	383.045491	-0.806185658	0.107563152	-7.4949985	6.63E-14	4.63E-13
ENSG00000182165	TP53TG1	380.5673327	484.65203	276.4826403	-0.807942728	0.112841436	-7.1599827	8.07E-13	5.34E-12
ENSG00000101224	CDC25B	2552.299352	3250.2995	1854.299207	-0.808801691	0.0493656	-16.383913	2.49E-60	5.54E-59
ENSG00000145604	SKP2	1097.189498	1397.5772	796.8017774	-0.81048152	0.067257211	-12.050478	1.93E-33	2.57E-32
ENSG00000154839	SKA1	723.9818338	922.43944	525.5242269	-0.811885146	0.085963995	-9.444479	3.57E-21	3.37E-20
ENSG00000160117	ANKLE1	54.44603172	69.333512	39.55855146	-0.811893252	0.296821756	-2.7352889	0.006233	0.015818
ENSG00000111247	RAD51AP1	515.1005343	656.45287	373.7482001	-0.811988114	0.095995714	-8.4585872	2.71E-17	2.19E-16
ENSG00000198933	TBKBP1	795.1185888	1013.7343	576.5028357	-0.812191381	0.08148186	-9.967757	2.11E-23	2.15E-22
ENSG00000113719	ERGIC1	6642.113808	8465.696	4818.531591	-0.812782103	0.032446542	-25.049883	1.75E-138	8.63E-137
ENSG00000177570	SAMD12	331.5951446	422.65366	240.5366285	-0.812989611	0.118992705	-6.8322643	8.36E-12	5.24E-11
ENSG00000169291	SHE	6027.421376	7687.9277	4366.915078	-0.815282953	0.035512074	-22.957908	1.23E-116	5.15E-115
ENSG00000179750	APOBEC3B	182.6846204	233.01436	132.3548768	-0.815408571	0.161012035	-5.0642709	4.10E-07	1.82E-06
ENSG00000167536	DHRS13	202.8567928	258.61401	147.0995711	-0.817447158	0.160898603	-5.0805112	3.76E-07	1.67E-06
ENSG00000204947	ZNF425	54.3492615	69.338849	39.35967425	-0.817978405	0.294236443	-2.7800037	0.005436	0.013961
ENSG00000161653	NAGS	192.9869038	246.4835	139.4903048	-0.820660821	0.156194237	-5.2541044	1.49E-07	6.86E-07
ENSG00000177732	SOX12	1990.649239	2542.9262	1438.372292	-0.822598287	0.064090782	-12.834892	1.05E-37	1.55E-36
ENSG00000142945	KIF2C	1229.647932	1571.563	887.7328521	-0.82350562	0.06315407	-13.039629	7.28E-39	1.11E-37
ENSG00000132932	ATP8A2	50.55492379	64.595014	36.51483319	-0.824040759	0.336365183	-2.4498396	0.014292	0.033523
ENSG00000182287	AP1S2	2221.21349	2838.5022	1603.924773	-0.824142893	0.053754112	-15.331718	4.69E-53	9.28E-52
ENSG00000152253	SPC25	312.4172634	399.42624	225.4082823	-0.826263149	0.127376985	-6.4867539	8.77E-11	5.16E-10

ENSG00000115163	CENPA	328.2649157	419.7591	236.7707333	-0.826352569	0.119770095	-6.89949	5.22E-12	3.31E-11
ENSG00000275832	ARHGAP23	5084.54337	6502.1665	3666.920192	-0.826828987	0.046817958	-17.660509	8.45E-70	2.17E-68
ENSG00000179965	ZNF771	223.8473137	286.36343	161.3311961	-0.828279574	0.157599511	-5.2555974	1.48E-07	6.81E-07
ENSG00000143815	LBR	3357.46645	4295.0385	2419.89439	-0.828295125	0.041354262	-20.029257	3.06E-89	1.04E-87
ENSG00000165475	CRYL1	684.7394328	876.18004	493.2988224	-0.829002597	0.083740729	-9.8996343	4.18E-23	4.22E-22
ENSG00000035499	DEPDC1B	325.3071921	416.37801	234.2363731	-0.829684024	0.124503114	-6.663962	2.67E-11	1.62E-10
ENSG00000100478	AP4S1	251.8767025	322.66627	181.0871304	-0.83009612	0.13886624	-5.9776669	2.26E-09	1.20E-08
ENSG00000131196	NFATC1	1440.528718	1844.8598	1036.197619	-0.830873948	0.06112536	-13.59295	4.41E-42	7.21E-41
ENSG00000102057	KCND1	60.19665331	76.937024	43.45628268	-0.830926572	0.280748434	-2.9596837	0.00308	0.008332
ENSG00000164070	HSPA4L	919.0272634	1177.5681	660.4864011	-0.832032208	0.077019207	-10.802918	3.33E-27	3.81E-26
ENSG00000134574	DDB2	2122.423084	2718.8707	1525.97548	-0.833861298	0.054797038	-15.21727	2.72E-52	5.31E-51
ENSG00000125378	BMP4	3648.982701	4675.9752	2621.99022	-0.834374441	0.04285732	-19.468656	2.03E-84	6.50E-83
ENSG00000198794	SCAMP5	649.0012297	831.63688	466.3655767	-0.834613306	0.088399286	-9.4414032	3.68E-21	3.47E-20
ENSG00000178922	HYI	2127.851255	2726.2324	1529.470128	-0.834619334	0.062170408	-13.424704	4.33E-41	6.96E-40
ENSG00000130005	GAMT	398.496931	510.6899	286.303959	-0.835025903	0.118774195	-7.0303646	2.06E-12	1.33E-11
ENSG00000145386	CCNA2	2120.385895	2717.0701	1523.701666	-0.835143093	0.055255494	-15.114209	1.31E-51	2.52E-50
ENSG00000204131	NHSL2	3531.441862	4529.3994	2533.484282	-0.83802727	0.043613661	-19.214788	2.78E-82	8.71E-81
ENSG00000231607	DLEU2	114.2219539	146.56622	81.87768562	-0.838376462	0.222134808	-3.7741787	0.000161	0.000537
ENSG00000151702	FLI1	7387.818571	9480.9888	5294.648384	-0.840245454	0.034373254	-24.444746	5.72E-132	2.69E-130
ENSG00000114790	ARHGEF26	123.5167694	158.74926	88.28428315	-0.841117486	0.214187781	-3.9270097	8.60E-05	0.000298
ENSG00000137714	FDX1	648.9796631	833.33607	464.6232594	-0.841393333	0.097058152	-8.668961	4.36E-18	3.65E-17
ENSG00000138166	DUSP5	507.5391459	651.671	363.4072902	-0.842157525	0.105709959	-7.966681	1.63E-15	1.22E-14
ENSG00000006062	MAP3K14	271.6032609	349.00373	194.2027957	-0.844645674	0.131828046	-6.4071774	1.48E-10	8.58E-10
ENSG00000092820	EZR	5571.666877	7159.4725	3983.861243	-0.845511686	0.033586539	-25.174123	7.69E-140	3.83E-138
ENSG00000172159	FRMD3	191.8476579	246.62521	137.0701013	-0.845792792	0.166726132	-5.0729468	3.92E-07	1.74E-06
ENSG00000204366	ZBTB12	166.9848237	214.94225	119.0273928	-0.847403695	0.211979319	-3.9975772	6.40E-05	0.000226
ENSG00000175087	PDIK1L	264.9774601	340.66805	189.2868692	-0.847554345	0.140563678	-6.0296825	1.64E-09	8.83E-09
ENSG00000186185	KIF18B	836.3193906	1075.8115	596.8273311	-0.849241042	0.080199405	-10.589119	3.35E-26	3.74E-25
ENSG00000069011	PITX1	93.31095713	119.94031	66.68160226	-0.850153745	0.22680465	-3.7483964	0.000178	0.000591
ENSG00000105889	STEAP1B	1046.794475	1346.5786	747.0103163	-0.850336718	0.070860475	-12.000155	3.55E-33	4.72E-32
ENSG00000167912	AC090152.1	168.3930193	216.96257	119.823466	-0.851012688	0.208456669	-4.0824441	4.46E-05	0.00016
ENSG00000162599	NFIA	1493.932073	1921.9896	1065.874596	-0.851070831	0.064338085	-13.228103	6.04E-40	9.44E-39
ENSG00000184897	H1FX	2068.215571	2661.5233	1474.907793	-0.851704815	0.192632342	-4.4214009	9.81E-06	3.79E-05
ENSG00000173597	SULT1B1	3710.600576	4778.0601	2643.14107	-0.854282633	0.045302715	-18.857206	2.56E-79	7.54E-78
ENSG00000166510	CCDC68	217.1185295	279.76921	154.4678457	-0.854710162	0.158844991	-5.3807813	7.42E-08	3.51E-07
ENSG00000064999	ANKS1A	2153.494445	2774.1707	1532.818195	-0.856250511	0.051481076	-16.632335	4.06E-62	9.31E-61
ENSG00000186603	HPDL	97.9105165	126.01996	69.80107115	-0.857238298	0.221880281	-3.8635173	0.000112	0.000382
ENSG00000177030	DEAF1	2209.376098	2848.2447	1570.50753	-0.85743429	0.056395609	-15.203919	3.33E-52	6.50E-51
ENSG00000176896	TCEANC	32.5627539	41.949056	23.17645208	-0.857604673	0.371828292	-2.3064535	0.021085	0.047366
ENSG00000004660	CAMKK1	171.828924	221.67318	121.9846651	-0.858644997	0.174019269	-4.934195	8.05E-07	3.47E-06
ENSG00000163412	EIF4E3	259.6653054	335.121	184.2096145	-0.86058427	0.149748358	-5.7468695	9.09E-09	4.64E-08
ENSG00000024422	EHD2	16924.00817	21832.404	12015.61282	-0.861419346	0.031788491	-27.098466	1.03E-161	6.03E-160
ENSG00000124942	AHNAK	76897.55837	99197.735	54597.38143	-0.861568666	0.027158983	-31.723156	7.45E-221	6.14E-219
ENSG00000168490	PHYHIP	58.81526132	75.962351	41.66817182	-0.862164015	0.293160928	-2.940924	0.003272	0.008805
ENSG00000148488	ST8SIA6	38.93592665	50.142172	27.72968171	-0.862371126	0.357544168	-2.4119289	0.015868	0.036811
ENSG00000013297	CLDN11	5606.498035	7234.6039	3978.392121	-0.862870354	0.033365864	-25.860873	1.84E-147	9.81E-146
ENSG00000013619	MAMLD1	260.0734256	335.93118	184.2156715	-0.863281502	0.142107543	-6.0748464	1.24E-09	6.73E-09
ENSG00000182492	BGN	54775.45314	70725.338	38825.56811	-0.865189038	0.029122768	-29.708338	5.99E-194	4.30E-192
ENSG00000188732	FAM221A	53.72231429	69.292955	38.15167387	-0.866600637	0.303485639	-2.8554914	0.004297	0.011275
ENSG00000234961	AL133415.1	35.5825015	45.93816	25.226843	-0.866907187	0.36031146	-2.4059939	0.016129	0.037339
ENSG00000132846	ZBED3	956.1517218	1235.8859	676.4175063	-0.869782133	0.077120178	-11.278269	1.68E-29	2.06E-28
ENSG00000050820	BCAR1	8916.955624	11528.561	6305.350588	-0.870350041	0.034064411	-25.550128	5.47E-144	2.83E-142
ENSG00000196549	MME	634.5270755	820.62047	448.4336763	-0.870889562	0.095967273	-9.0748599	1.14E-19	1.01E-18
ENSG00000135476	ESPL1	936.2451254	1210.777	661.7132011	-0.871701551	0.079135203	-11.015345	3.22E-28	3.81E-27
ENSG00000143537	ADAM15	10865.1143	14054.128	7676.100503	-0.872466045	0.031808169	-27.428993	1.24E-165	7.36E-164
ENSG00000117650	NEK2	388.6940545	502.80562	274.5824901	-0.872511146	0.113926986	-7.6585116	1.88E-14	1.35E-13
ENSG00000143819	EPHX1	788.7528154	1020.6068	556.8988647	-0.873260303	0.08108589	-10.769572	4.79E-27	5.46E-26
ENSG00000064115	TM7SF3	2275.568423	2944.1349	1607.001996	-0.873688477	0.049259442	-17.736467	2.19E-70	5.65E-69
ENSG00000161513	FDXR	894.8204925	1157.5164	632.1245566	-0.874097726	0.078437906	-11.143818	7.68E-29	9.27E-28
ENSG00000165244	ZNF367	290.9424793	376.32515	205.5598129	-0.874375245	0.128058889	-6.8279153	8.62E-12	5.39E-11
ENSG00000164692	COL1A2	4058.848819	5251.9843	2865.713318	-0.874551536	0.046941983	-18.630477	1.82E-77	5.19E-76
ENSG00000163808	KIF15	733.6907054	950.11999	517.2614186	-0.874917858	0.086213434	-10.148278	3.37E-24	3.54E-23
ENSG00000156298	TSPAN7	122.790584	158.98024	86.6009235	-0.875394372	0.203938165	-4.29245	1.77E-05	6.66E-05
ENSG00000119943	PYROXD2	116.0384879	150.2129	81.86407738	-0.876382238	0.204533813	-4.2847792	1.83E-05	6.88E-05
ENSG00000134057	CCNB1	2820.209272	3651.6378	1988.780763	-0.876525987	0.045968542	-19.067953	4.66E-81	1.43E-79
ENSG00000197061	HIST1H4C	108.7573783	140.77876	76.73599873	-0.877625747	0.217977532	-4.0262211	5.67E-05	0.000201
ENSG00000156103	MMP16	3143.264972	4072.912	2213.61796	-0.878594462	0.052119788	-16.857215	9.29E-64	2.18E-62
ENSG00000206560	ANKRD28	2531.890706	3280.4336	1783.347844	-0.878904533	0.045331631	-19.388328	9.68E-84	3.08E-82
ENSG00000117724	CENPF	5468.345326	7084.2942	3852.396452	-0.879351954	0.037938045	-23.178631	7.48E-119	3.19E-117
ENSG00000066735	KIF26A	355.2461573	460.29288	250.1994297	-0.879615677	0.113781375	-7.7307527	1.07E-14	7.76E-14
ENSG00000011523	CEP68	1919.068958	2488.3704	1349.767482	-0.882525405	0.055155565	-16.000659	1.26E-57	2.67E-56
ENSG00000071575	TRIB2	811.88815	1053.2182	570.5580915	-0.883292105	0.08753188	-10.09109	6.05E-24	6.30E-23
ENSG00000176428	VPS37D	51.14545722	66.512694	35.77822041	-0.883858351	0.37186161	-2.3768475	0.017461	0.040002
ENSG00000181744	DIPK2A	1370.492533	1778.0911	962.8939215	-0.885258726	0.059212898	-14.950438	1.55E-50	2.94E-49
ENSG00000126351	THRA	2161.044692	2804.3402	1517.74918	-0.885735983	0.049720768	-17.814206	5.48E-71	1.43E-69
ENSG00000106772	PRUNE2	452.4433921	587.57287	317.3139141	-0.885991341	0.108504018	-8.1655165	3.20E-16	2.48E-15
ENSG00000161888	SPC24	577.4484396	749.40853	405.4883484	-0.887372352	0.10347546	-8.5756792	9.85E-18	8.12E-17
ENSG00000241717	VWFP1	392.1803357	509.46528	274.8953924	-0.888298726	0.116386124	-7.6323422	2.31E-14	1.64E-13
ENSG00000003400	CASP10	1484.049453	1927.4883	1040.610578	-0.888491186	0.067749926	-13.114275	2.73E-39	4.20E-38
ENSG00000162415	ZSWIM5	123.4886418	160.50018	86.47710087	-0.889025929	0.195054086	-4.5578432	5.17E-06	2.05E-05
ENSG00000080986	NDC80	1070.521346	1391.258	749.7846654	-0.891481226	0.070514592	-12.642507	1.23E-36	1.76E-35
ENSG00000189223	PAX8-AS1	107.4769399	139.84542	75.10846128	-0.89226211	0.213629017	-4.1766897	2.96E-05	0.000109
ENSG00000156384	SFR1	200.66017	261.07809	140.2422484	-0.89345439	0.174030658	-5.1338908	2.84E-07	1.28E-06
ENSG00000142634	EFHD2	3144.208413	4088.4158	2200.000997	-0.894279094	0.052497439	-17.034718	4.54E-65	1.09E-63
ENSG00000003436	TFPI	21192.3279	27569.341	14815.31473	-0.895659976	0.027419432	-32.665155	4.88E-234	4.38E-232

ENSG00000143409	MINDY1	464.073895	603.5041	324.643686	-0.897047539	0.114775128	-7.8156963	5.47E-15	4.03E-14
ENSG00000117791	2-Mar	338.9937507	441.17336	236.8141376	-0.897730586	0.118818108	-7.5555031	4.17E-14	2.94E-13
ENSG00000143228	NUF2	675.7271108	879.06024	472.3939855	-0.897802275	0.090242451	-9.9487798	2.55E-23	2.60E-22
ENSG00000266094	RASSF5	44.28779086	57.65197	30.92361144	-0.898082087	0.337490128	-2.6610618	0.007789	0.019359
ENSG00000170836	PPM1D	1125.287023	1465.2972	785.2768079	-0.898257522	0.073413296	-12.235624	2.01E-34	2.73E-33
ENSG00000136982	DSCC1	183.0627645	238.21042	127.9151079	-0.89914904	0.160786869	-5.5921795	2.24E-08	1.11E-07
ENSG00000204138	PHACTR4	3361.097718	4376.4671	2345.728312	-0.899226359	0.041086319	-21.886272	3.51E-106	1.38E-104
ENSG00000198915	RASGEF1A	50.11171493	65.226253	34.99717678	-0.899423379	0.306240558	-2.9369832	0.003314	0.008906
ENSG00000171604	CXXC5	233.1545206	303.30889	163.000152	-0.89979861	0.150104415	-5.9944847	2.04E-09	1.09E-08
ENSG00000172465	TCEAL1	330.6810865	430.70514	230.6570367	-0.900772541	0.131860159	-6.8312715	8.42E-12	5.28E-11
ENSG00000142875	PRKACB	4437.964847	5780.7543	3095.175371	-0.900859118	0.047961427	-18.782992	1.04E-78	3.02E-77
ENSG00000170396	ZNF804A	152.4295329	198.38024	106.4788263	-0.901047321	0.173487845	-5.1937202	2.06E-07	9.39E-07
ENSG00000079156	OSBPL6	395.4359645	515.3256	275.5463254	-0.901187229	0.133219268	-6.7646913	1.34E-11	8.23E-11
ENSG00000141480	ARRB2	519.3883521	676.72124	362.0554686	-0.901215474	0.100415441	-8.9748694	2.84E-19	2.47E-18
ENSG00000066279	ASPM	3091.053838	4027.5575	2154.550193	-0.902309256	0.046754651	-19.298813	5.50E-83	1.73E-81
ENSG00000143942	CHAC2	199.2189496	259.63345	138.8044459	-0.902797172	0.175330133	-5.1491273	2.62E-07	1.18E-06
ENSG00000137310	TCF19	1367.369015	1782.2111	952.526956	-0.904109089	0.05979603	-15.119885	1.20E-51	2.31E-50
ENSG00000083807	SLC27A5	65.1528923	84.88208	45.42370502	-0.906845783	0.316713305	-2.8633018	0.004193	0.011034
ENSG00000111445	RFC5	646.7743772	843.42728	450.1214726	-0.906846179	0.087791039	-10.329598	5.18E-25	5.57E-24
ENSG00000197008	ZNF138	109.9960289	143.68629	76.30576636	-0.907295398	0.212745309	-4.2647022	2.00E-05	7.50E-05
ENSG00000164850	GPER1	73.7484057	96.173852	51.32295965	-0.90747275	0.257258419	-3.5274754	0.00042	0.001318
ENSG00000143919	CAMKMT	110.2719989	143.74155	76.80244613	-0.907553109	0.217874478	-4.1654861	3.11E-05	0.000114
ENSG00000005884	ITGA3	5511.933689	7194.481	3829.386349	-0.909300724	0.036207117	-25.113867	3.51E-139	1.74E-137
ENSG00000152527	PLEKHH2	52.21323458	68.1176	36.30886964	-0.90953536	0.296292493	-3.0697212	0.002143	0.005996
ENSG00000101134	DOK5	298.8517003	390.35888	207.3445165	-0.909649249	0.138985054	-6.544943	5.95E-11	3.53E-10
ENSG00000184292	TACSTD2	1989.805876	2597.9815	1381.630263	-0.911141384	0.054811452	-16.623194	4.73E-62	1.08E-60
ENSG00000111665	CDCA3	405.1655218	529.47809	280.8529494	-0.91513195	0.106608786	-8.58402	9.16E-18	7.55E-17
ENSG00000136720	HS6ST1	1292.1374	1689.1095	895.1652549	-0.916343563	0.061869572	-14.810892	1.25E-49	2.32E-48
ENSG00000163751	CPA3	93.49142707	122.36399	64.61886356	-0.918237787	0.233409143	-3.9340266	8.35E-05	0.00029
ENSG00000147119	CHST7	660.0479461	862.7744	457.3214937	-0.918542948	0.091616778	-10.025925	1.17E-23	1.21E-22
ENSG00000171843	MLLT3	508.9062058	666.41109	351.4013258	-0.92049762	0.100948782	-9.1184619	7.62E-20	6.83E-19
ENSG00000189184	PCDH18	141.6409298	185.44089	97.84097331	-0.921580189	0.184042595	-5.0074288	5.52E-07	2.42E-06
ENSG00000125740	FOSB	45.38261615	59.394999	31.37023337	-0.921587596	0.311926515	-2.9545023	0.003132	0.008457
ENSG00000181885	CLDN7	298.246074	390.2965	206.1956462	-0.921897193	0.126572002	-7.2835791	3.25E-13	2.20E-12
ENSG00000132589	FLOT2	5513.573839	7217.7722	3809.375473	-0.922153775	0.033224368	-27.755344	1.50E-169	9.18E-168
ENSG00000127328	RAB3IP	528.8263748	692.02059	365.6321553	-0.922839226	0.104063436	-8.8680449	7.44E-19	6.39E-18
ENSG00000277149	TYW1B	118.3480617	155.04907	81.64705584	-0.926585736	0.222002245	-4.1737674	3.00E-05	0.00011
ENSG00000134138	MEIS2	1477.544667	1937.0397	1018.049627	-0.926848053	0.064495331	-14.370778	7.89E-47	1.41E-45
ENSG00000102893	PHKB	2213.08375	2899.9991	1526.168389	-0.926974715	0.050600558	-18.319456	5.79E-75	1.58E-73
ENSG00000176124	DLEU1	41.66281391	54.602054	28.72357349	-0.92698868	0.356869684	-2.5975551	0.009389	0.022941
ENSG00000105472	CLEC11A	333.9329533	437.50863	230.3572805	-0.927129284	0.128571599	-7.210996	5.55E-13	3.71E-12
ENSG00000065054	SLC9A3R2	2881.610184	3777.6928	1985.527604	-0.928615059	0.048156085	-19.283442	7.40E-83	2.32E-81
ENSG00000131747	TOP2A	6226.137283	8168.0505	4284.224061	-0.930485609	0.035465149	-26.236619	1.02E-151	5.66E-150
ENSG00000119711	ALDH6A1	577.553734	758.21937	396.8881003	-0.931779294	0.095929469	-9.7131705	2.65E-22	2.60E-21
ENSG00000176714	CCDC121	116.8492739	153.65377	80.04477838	-0.933615304	0.236256392	-3.9517039	7.76E-05	0.00027
ENSG00000258429	PDF	72.82406777	95.474541	50.17359497	-0.933716032	0.27732115	-3.3669124	0.00076	0.002294
ENSG00000166228	PCBD1	1137.948457	1494.8499	781.0469714	-0.9343637	0.074027278	-12.621884	1.60E-36	2.29E-35
ENSG00000276386	CNTNAP3P2	202.6467575	266.54495	138.7485622	-0.936807635	0.158251982	-5.9197213	3.22E-09	1.70E-08
ENSG00000024526	DEPDC1	867.2948566	1139.6438	594.9459484	-0.936939246	0.0841634	-11.132384	8.73E-29	1.05E-27
ENSG00000213853	EMP2	827.1991602	1086.7751	567.6231955	-0.937036346	0.075243012	-12.453467	1.34E-35	1.88E-34
ENSG00000135312	HTR1B	1292.735072	1698.6759	886.7942181	-0.937078482	0.065409807	-14.326269	1.50E-46	2.66E-45
ENSG00000139973	SYT16	29.99284459	39.415406	20.57028358	-0.937384055	0.395088303	-2.3725938	0.017664	0.040385
ENSG00000121957	GPSM2	596.9822652	784.64315	409.3213841	-0.937527381	0.092427455	-10.143386	3.55E-24	3.72E-23
ENSG00000137507	LRRC32	8656.373803	11373.505	5939.243027	-0.937532974	0.033004712	-28.406034	1.70E-177	1.11E-175
ENSG00000112297	CRYBG1	2571.704037	3382.5875	1760.820599	-0.940789376	0.051609674	-18.228935	3.04E-74	8.21E-73
ENSG00000128573	FOXP2	42.56856984	55.918308	29.21883184	-0.94160859	0.338802714	-2.7792239	0.005449	0.013989
ENSG00000079482	OPHN1	568.7526543	748.19737	389.3079368	-0.943770844	0.093793943	-10.062173	8.12E-24	8.42E-23
ENSG00000203995	ZYG11A	60.51238969	79.57257	41.45220901	-0.944080875	0.30312243	-3.11452	0.001842	0.005212
ENSG00000181031	RPH3AL	289.5390622	381.14191	197.936213	-0.944418313	0.134401466	-7.0268453	2.11E-12	1.37E-11
ENSG00000100060	MFNG	6814.288689	8969.6824	4658.89502	-0.94485539	0.037634176	-25.106313	4.24E-139	2.10E-137
ENSG00000241990	PRR34-AS1	31.92406919	42.064029	21.78410983	-0.946234428	0.383690893	-2.4661373	0.013658	0.032212
ENSG00000091127	PUS7	1915.856158	2522.1598	1309.552476	-0.94657494	0.052219383	-18.126889	1.96E-73	5.23E-72
ENSG00000164104	HMGB2	3459.664692	4555.542	2363.787405	-0.947984998	0.050318617	-18.839648	3.57E-79	1.05E-77
ENSG00000073737	DHRS9	162.5945629	214.14416	111.0449681	-0.948528317	0.167505443	-5.6626716	1.49E-08	7.48E-08
ENSG00000183208	GDPGP1	45.51051512	59.944859	31.07617141	-0.949882956	0.317094179	-2.9955862	0.002739	0.0075
ENSG00000148773	MKI67	9720.714357	12812.886	6628.542805	-0.950743427	0.028904387	-32.892703	2.79E-237	2.62E-235
ENSG00000184661	CDCA2	955.4460826	1259.3032	651.5889821	-0.951346638	0.072501513	-13.121749	2.47E-39	3.82E-38
ENSG00000158402	CDC25C	196.1779711	258.66958	133.6863665	-0.953779034	0.163423665	-5.8362357	5.34E-09	2.77E-08
ENSG00000135314	KHDC1	31.17009817	41.160732	21.17946474	-0.954200143	0.379165102	-2.5165822	0.01185	0.0283
ENSG00000160325	CACFD1	425.7385574	561.93397	289.5431472	-0.95603677	0.106721969	-8.9582002	3.30E-19	2.86E-18
ENSG00000082438	COBLL1	1495.412658	1973.5034	1017.321917	-0.956169951	0.06320269	-15.128627	1.05E-51	2.03E-50
ENSG00000173269	MMRN2	19973.48297	26359.901	13587.06454	-0.956184553	0.02755259	-34.703981	6.86E-264	7.13E-262
ENSG00000285043	AC093512.2	39.92233726	52.635742	27.2089323	-0.956928546	0.362582061	-2.6392054	0.00831	0.02052
ENSG00000134184	GSTM1	293.4450787	387.5275	199.3626563	-0.9584086	0.125180135	-7.6562356	1.91E-14	1.37E-13
ENSG00000122378	PRXL2A	18603.72404	24568.69	12638.75821	-0.958960242	0.024306	-39.453643	0	0
ENSG00000138180	CEP55	2078.374154	2745.5386	1411.2097	-0.959047247	0.05482846	-17.491778	1.66E-68	4.17E-67
ENSG00000174749	FAM241A	1921.760158	2538.8406	1304.679688	-0.959387336	0.057895562	-16.571	1.13E-61	2.56E-60
ENSG00000188511	C22orf34	386.6817421	511.35055	262.0129365	-0.962773357	0.119165328	-8.0793077	6.51E-16	4.97E-15
ENSG00000164542	KIAA0895	34.75308368	46.010756	23.49541134	-0.963466716	0.375512693	-2.5657367	0.010296	0.024934
ENSG00000106123	EPHB6	132.8130252	175.63557	89.99048416	-0.964264192	0.190743801	-5.0552846	4.30E-07	1.90E-06
ENSG00000164237	CMBL	947.1305316	1253.1625	641.0985539	-0.966520711	0.080817625	-11.959281	5.81E-33	7.68E-32
ENSG00000111846	GCNT2	863.9753907	1143.5962	584.3546146	-0.966646852	0.078084413	-12.379511	3.37E-35	4.68E-34
ENSG00000138606	SHF	85.79217223	113.37115	58.21319313	-0.966883021	0.239558634	-4.0361017	5.43E-05	0.000193
ENSG00000185909	KLHDC8B	2126.107117	2814.2796	1437.94165	-0.96815495	0.050191355	-19.289277	6.61E-83	2.07E-81
ENSG00000238045	AC009133.1	30.1846937	40.017415	20.35197224	-0.969205876	0.408235938	-2.3741317	0.01759	0.040257

ENSG00000211584	SLC48A1	525.3248587	695.42595	355.2237714	-0.970203466	0.094380527	-10.2797	8.70E-25	9.31E-24
ENSG00000196872	KIAA1211L	316.8798382	419.81955	213.9401236	-0.972989888	0.125065765	-7.779826	7.26E-15	5.32E-14
ENSG00000185614	INKA1	397.9149976	527.52601	268.3039881	-0.973576365	0.112068255	-8.6873519	3.71E-18	3.11E-17
ENSG00000129195	PIMREG	551.6111032	730.91869	372.303513	-0.975195939	0.094056486	-10.368194	3.46E-25	3.75E-24
ENSG00000198774	RASSF9	315.1418403	418.142	212.1416802	-0.976538918	0.137453136	-7.1045227	1.21E-12	7.92E-12
ENSG00000110090	CPT1A	8253.965462	10950.963	5556.967595	-0.978172432	0.032424583	-30.167618	6.30E-200	4.73E-198
ENSG00000100292	HMOX1	1657.855217	2199.2433	1116.467102	-0.978384102	0.057608165	-16.983428	1.09E-64	2.59E-63
ENSG00000213366	GSTM2	50.3863853	66.959987	33.81278391	-0.979687384	0.30157814	-3.2485358	0.00116	0.003392
ENSG00000154529	CNTNAP3B	333.6518725	442.8681	224.4356495	-0.979695787	0.128307548	-7.6355273	2.25E-14	1.61E-13
ENSG00000157551	KCNJ15	37.45853461	49.745616	25.17145359	-0.979733613	0.360850075	-2.7150711	0.006626	0.016731
ENSG00000241399	CD302	679.6760417	903.01289	456.3391922	-0.982701546	0.088708779	-11.077839	1.61E-28	1.92E-27
ENSG00000196139	AKR1C3	170.6513638	226.65025	114.6524774	-0.983863861	0.163552768	-6.0155745	1.79E-09	9.59E-09
ENSG00000162804	SNED1	1740.633737	2312.2198	1169.047655	-0.983917591	0.057929663	-16.984694	1.07E-64	2.54E-63
ENSG00000100036	SLC35E4	956.8301618	1270.8894	642.7708877	-0.983954836	0.07427645	-13.247198	4.68E-40	7.35E-39
ENSG00000140350	ANP32A	5800.494754	7708.277	3892.712512	-0.985598137	0.032589204	-30.243088	6.43E-201	4.85E-199
ENSG00000134243	SORT1	1550.267718	2061.261	1039.274479	-0.985851273	0.063628749	-15.493803	3.82E-54	7.67E-53
ENSG00000133026	MYH10	27113.1329	36037.841	18188.42453	-0.986535804	0.02503399	-39.407854	0	0
ENSG00000198121	LPAR1	70.70008695	94.234807	47.16536701	-0.988655599	0.274710709	-3.5988972	0.00032	0.001022
ENSG00000138162	TACC2	1988.090343	2644.7386	1331.442071	-0.989242775	0.052332523	-18.903021	1.08E-79	3.20E-78
ENSG00000111261	MANSC1	2987.425566	3974.1495	2000.70167	-0.989488039	0.045239401	-21.872262	4.77E-106	1.88E-104
ENSG00000143127	ITGA10	2064.887757	2747.7804	1381.995104	-0.990791896	0.060781515	-16.300875	9.73E-60	2.15E-58
ENSG00000239445	ST3GAL6-AS1	31.2057909	41.437385	20.97419724	-0.991436674	0.392614514	-2.5252166	0.011563	0.027671
ENSG00000171522	PTGER4	1022.46336	1360.1454	684.7812958	-0.9919396	0.075157721	-13.198106	9.00E-40	1.40E-38
ENSG00000198624	CCDC69	723.1617915	962.65558	483.6680048	-0.992448939	0.081114731	-12.235126	2.02E-34	2.74E-33
ENSG00000180304	OAZ2	4985.108964	6635.2752	3334.942751	-0.992590768	0.035207838	-28.192324	7.26E-175	4.62E-173
ENSG00000072954	TMEM38A	46.72997017	62.207935	31.25200554	-0.993765387	0.32037017	-3.1019286	0.001923	0.005423
ENSG00000172803	SNX32	32.57154449	43.299981	21.84310821	-0.994043089	0.398038791	-2.4973523	0.012512	0.029755
ENSG00000130940	CASZ1	179.5006728	239.23406	119.7672812	-0.997389779	0.190623134	-5.2322599	1.67E-07	7.70E-07
ENSG00000175899	A2M	779.1560446	1039.0812	519.2309263	-0.997781063	0.101298362	-9.849923	6.86E-23	6.87E-22
ENSG00000008277	ADAM22	30.32420609	40.473897	20.17451497	-0.99811089	0.386906786	-2.5797193	0.009888	0.024055
ENSG00000230438	SERPINB9P1	71.48107873	95.239258	47.72289937	-0.998359957	0.282930994	-3.5286341	0.000418	0.001313
ENSG00000185875	THNSL1	235.725494	314.39478	157.0562033	-1.000230908	0.157608331	-6.3463073	2.21E-10	1.26E-09
ENSG00000224152	AC009506.1	83.81552969	111.5553	56.07576254	-1.002451931	0.269628719	-3.7178975	0.000201	0.000662
ENSG00000165480	SKA3	666.0762795	889.10749	443.0450724	-1.00557369	0.094337772	-10.65929	1.58E-26	1.78E-25
ENSG00000224383	PRR29	33.69538921	44.831884	22.55889479	-1.005806351	0.409714696	-2.4548945	0.014093	0.033121
ENSG00000162616	DNAJB4	4712.208157	6293.6794	3130.736906	-1.006319931	0.043946959	-22.898511	4.81E-116	2.00E-114
ENSG00000138678	GPAT3	333.7607888	445.54987	221.9717084	-1.007608248	0.124427778	-8.0979365	5.59E-16	4.28E-15
ENSG00000100784	RPS6KA5	559.0409432	746.92436	371.1575234	-1.008524538	0.098865178	-10.201009	1.96E-24	2.07E-23
ENSG00000179144	GIMAP7	2919.021935	3904.5344	1933.509468	-1.014267699	0.047574596	-21.319523	7.48E-101	2.84E-99
ENSG00000254535	PABPC4L	109.9694678	147.30259	72.63634574	-1.014601659	0.228579037	-4.4387345	9.05E-06	3.51E-05
ENSG00000081189	MEF2C	1563.607763	2092.5334	1034.68215	-1.015046008	0.058150728	-17.455431	3.13E-68	7.83E-67
ENSG00000182568	SATB1	443.2322496	593.33176	293.1327433	-1.01547586	0.110117271	-9.2217674	2.92E-20	2.66E-19
ENSG00000175874	CREG2	96.70323068	129.22081	64.18564814	-1.017416154	0.234296644	-4.3424273	1.41E-05	5.36E-05
ENSG00000174684	B4GAT1	1136.332237	1522.0524	750.6120964	-1.017750242	0.071981364	-14.13908	2.18E-45	3.78E-44
ENSG00000135363	LMO2	1536.148987	2057.1053	1015.192699	-1.01788863	0.060676608	-16.775635	3.68E-63	8.56E-62
ENSG00000143443	C1orf56	104.113786	139.31286	68.9147119	-1.018397377	0.247331655	-4.1175376	3.83E-05	0.000139
ENSG00000143450	OAZ3	58.13894951	77.920958	38.35694137	-1.022403284	0.299732918	-3.4110477	0.000647	0.00198
ENSG00000183323	CCDC125	312.806889	419.39032	206.2234564	-1.024915399	0.122416424	-8.3723684	5.65E-17	4.52E-16
ENSG00000167693	NXN	3216.089134	4313.4828	2118.695438	-1.025335763	0.043614049	-23.5093	3.28E-122	1.42E-120
ENSG00000168785	TSPAN5	2982.441962	3999.8858	1964.998101	-1.02538062	0.042969418	-23.863032	7.42E-126	3.34E-124
ENSG00000203722	RAET1G	23.82062642	32.024504	15.61674927	-1.02686456	0.443494971	-2.3153917	0.020592	0.046359
ENSG00000185100	ADSSL1	48.4262201	64.938414	31.91402586	-1.026896808	0.321622372	-3.192865	0.001409	0.004054
ENSG00000250510	GPR162	464.4859586	623.04489	305.9270307	-1.028408842	0.1037319	-9.914104	3.61E-23	3.66E-22
ENSG00000029534	ANK1	117.6666192	157.8546	77.47863911	-1.029816849	0.216910517	-4.7476575	2.06E-06	8.56E-06
ENSG00000135636	DYSF	11321.19368	15202.024	7440.363191	-1.030685671	0.026846839	-38.391323	0	0
ENSG00000131477	RAMP2	988.3692989	1327.8261	648.9124561	-1.034111547	0.080344113	-12.871031	6.55E-38	9.74E-37
ENSG00000198435	NRARP	2530.02087	3400.1395	1659.902274	-1.034361469	0.05756536	-17.96847	3.44E-72	9.12E-71
ENSG00000109881	CCDC34	430.1574515	578.663	281.6519035	-1.034382037	0.12087165	-8.5576894	1.15E-17	9.46E-17
ENSG00000134222	PSRC1	348.9847637	469.33695	228.6325725	-1.036080131	0.116444871	-8.8976021	5.71E-19	4.92E-18
ENSG00000189221	MAOA	671.0124493	902.46672	439.5581774	-1.036185027	0.100244069	-10.336622	4.81E-25	5.18E-24
ENSG00000162909	CAPN2	20244.49899	27230.932	13258.06615	-1.038544228	0.023744404	-43.738484	0	0
ENSG00000198417	MT1F	29.25383013	39.311212	19.19644833	-1.039102998	0.427630757	-2.4299071	0.015103	0.035244
ENSG00000188641	DPYD	990.5149433	1332.6276	648.4022675	-1.039159878	0.071602234	-14.512953	1.00E-47	1.82E-46
ENSG00000130193	THEM6	892.4504573	1200.7504	584.1505081	-1.040495991	0.080124312	-12.986021	1.47E-38	2.22E-37
ENSG00000157456	CCNB2	1023.03767	1377.274	668.8013553	-1.041991601	0.072257566	-14.420519	3.84E-47	6.92E-46
ENSG00000107902	LHPP	105.550356	141.98656	69.11415353	-1.042759623	0.21274629	-4.9014233	9.51E-07	4.08E-06
ENSG00000072071	ADGRL1	524.3979152	705.75409	343.0417418	-1.0432896	0.100745539	-10.35569	3.94E-25	4.27E-24
ENSG00000157514	TSC22D3	1117.373331	1505.926	728.8206691	-1.04572004	0.068687538	-15.224305	2.44E-52	4.77E-51
ENSG00000149218	ENDOD1	2520.815541	3398.623	1643.008091	-1.048195661	0.049026622	-21.380132	2.05E-101	7.82E-100
ENSG00000065057	NTHL1	511.179245	688.81782	333.5406722	-1.048309383	0.09954649	-10.530852	6.23E-26	6.92E-25
ENSG00000162390	ACOT11	202.7128253	273.40685	132.0188026	-1.049594932	0.161430942	-6.50182	7.94E-11	4.68E-10
ENSG00000227630	LINC01132	26.50322288	35.721103	17.2853425	-1.049673313	0.426063979	-2.4636519	0.013753	0.032403
ENSG00000122986	HVCN1	153.0647095	206.69421	99.43520516	-1.049895026	0.189258875	-5.5474018	2.90E-08	1.42E-07
ENSG00000133863	TEX15	218.5889369	294.8226	142.355276	-1.050120362	0.149296534	-7.0337893	2.01E-12	1.30E-11
ENSG00000168078	PBK	782.1426335	1054.8787	509.406525	-1.050793863	0.079415488	-13.231599	5.76E-40	9.01E-39
ENSG00000254531	AP001816.1	71.74179483	96.804277	46.67931244	-1.051551993	0.261051536	-4.0281395	5.62E-05	0.0002
ENSG00000180592	SKIDA1	33.88278314	45.679249	22.08631684	-1.052025058	0.370115832	-2.8424211	0.004477	0.011711
ENSG00000254810	AP001189.3	69.04800777	93.245536	44.85047914	-1.052876283	0.279896283	-3.7616658	0.000169	0.000563
ENSG00000184500	PROS1	749.1959985	1010.8834	487.5091884	-1.053461361	0.08361651	-12.598724	2.15E-36	3.05E-35
ENSG00000137309	HMGA1	30718.85803	41462.002	19975.7138	-1.05367715	0.033661747	-31.301915	4.39E-215	3.54E-213
ENSG00000237352	LINC01358	21.74172962	29.33573	14.14772928	-1.05393056	0.459285443	-2.294718	0.021749	0.048632
ENSG00000055163	CYFIP2	523.7267187	707.25842	340.1950144	-1.055070322	0.096337639	-10.951798	6.51E-28	7.62E-27
ENSG00000111276	CDKN1B	1800.716993	2431.6451	1169.788903	-1.055874044	0.054150416	-19.498909	1.12E-84	3.62E-83
ENSG00000105711	SCN1B	466.7728224	630.57635	302.9692934	-1.055980446	0.100869156	-10.468814	1.20E-25	1.32E-24
ENSG00000235194	PPP1R3E	122.7330157	165.84414	79.62189558	-1.056331852	0.192791057	-5.4791538	4.27E-08	2.07E-07

ENSG00000167363	FN3K	314.9203755	425.63024	204.2105123	-1.056576591	0.128604204	-8.2157236	2.11E-16	1.64E-15
ENSG00000181804	SLC9A9	74.62895399	100.85276	48.40514929	-1.056662166	0.262540348	-4.024761	5.70E-05	0.000202
ENSG00000175356	SCUBE2	56.0236056	75.729965	36.31724576	-1.057056372	0.301283027	-3.5085162	0.000451	0.001409
ENSG00000265972	TXNIP	1056.324323	1427.1826	685.466023	-1.057569746	0.073008721	-14.485526	1.50E-47	2.71E-46
ENSG00000170379	TCAF2	347.7909083	469.77393	225.807891	-1.058592778	0.125133855	-8.4596833	2.68E-17	2.17E-16
ENSG00000181409	AATK	89.27417433	120.8132	57.73515199	-1.064603163	0.238992738	-4.4545419	8.41E-06	3.27E-05
ENSG00000283632	EXOC3L2	93.34438961	126.3705	60.31827741	-1.065273602	0.229243938	-4.6468998	3.37E-06	1.37E-05
ENSG00000111206	FOXM1	3356.108351	4542.25	2169.966746	-1.065420817	0.040437463	-26.34737	5.50E-153	3.09E-151
ENSG00000099864	PALM	374.8711723	507.5696	242.1727438	-1.066032724	0.114555947	-9.3057825	1.33E-20	1.22E-19
ENSG00000143603	KCNN3	236.7780546	320.36294	153.1931669	-1.066045666	0.144698289	-7.3673689	1.74E-13	1.19E-12
ENSG00000119326	CTNNAL1	3781.794365	5118.6669	2444.92185	-1.066308744	0.046008834	-23.176174	7.92E-119	3.37E-117
ENSG00000117461	PIK3R3	812.1334156	1099.2548	525.0120146	-1.066544054	0.085068671	-12.537448	4.66E-36	6.58E-35
ENSG00000110400	NECTIN1	1505.102301	2037.4689	972.7357009	-1.066701145	0.061698906	-17.288818	5.71E-67	1.41E-65
ENSG00000211751	TRBC1	25.36648563	34.381364	16.35160728	-1.070083975	0.442478985	-2.4183837	0.01559	0.036224
ENSG00000022567	SLC45A4	588.7370012	798.22606	379.2479456	-1.072431312	0.10247285	-10.465517	1.24E-25	1.37E-24
ENSG00000128591	FLNC	11342.41017	15384.453	7300.367239	-1.075582364	0.0303819	-35.402077	1.59E-274	1.73E-272
ENSG00000142910	TINAGL1	9792.842623	13282.056	6303.629662	-1.07579212	0.033372314	-32.236067	5.52E-228	4.72E-226
ENSG00000204248	COL11A2	24.36332144	33.028548	15.6980947	-1.075978983	0.447724255	-2.403218	0.016251	0.0376
ENSG00000259065	AC005520.2	22.81602609	30.964863	14.6671889	-1.07598329	0.456670205	-2.3561495	0.018465	0.04202
ENSG00000170345	FOS	24.52466146	33.321795	15.72752807	-1.077833454	0.451777808	-2.3857601	0.017044	0.039173
ENSG00000184489	PTP4A3	154.9873267	210.28925	99.68540067	-1.080104382	0.178992724	-6.034348	1.60E-09	8.59E-09
ENSG00000260260	SNHG19	194.3632304	263.73065	124.995806	-1.080864089	0.15963853	-6.7706968	1.28E-11	7.91E-11
ENSG00000242193	CRYZL2P	221.3295318	300.75693	141.9021321	-1.083288519	0.145136073	-7.4639509	8.40E-14	5.84E-13
ENSG00000163382	NAXE	1907.965177	2592.8346	1223.095707	-1.08430943	0.053632195	-20.21751	6.87E-91	2.38E-89
ENSG00000063180	CA11	133.1959464	181.13024	85.26165147	-1.086743986	0.199774141	-5.4398632	5.33E-08	2.56E-07
ENSG00000106789	CORO2A	116.9739756	159.2349	74.71305537	-1.088213391	0.21328155	-5.1022388	3.36E-07	1.50E-06
ENSG00000171219	CDC42BPG	21.08265281	28.646192	13.519114	-1.088684817	0.476070329	-2.2868151	0.022207	0.049558
ENSG00000107738	VSIR	196.7286258	267.55714	125.9001078	-1.090885679	0.164421655	-6.6346837	3.25E-11	1.96E-10
ENSG00000168899	VAMP5	2497.908376	3399.5029	1596.313852	-1.091152396	0.047672625	-22.888448	6.06E-116	2.51E-114
ENSG00000106546	AHR	1214.811651	1654.0893	775.5339539	-1.091607126	0.076269556	-14.312488	1.83E-46	3.24E-45
ENSG00000133710	SPINK5	108.8299684	148.45805	69.20188925	-1.098949234	0.209094721	-5.2557484	1.47E-07	6.80E-07
ENSG00000143590	EFNA3	32.15305617	43.795058	20.51105413	-1.099863461	0.37845535	-2.9061908	0.003659	0.009746
ENSG00000133101	CCNA1	85.79281735	117.11645	54.46918006	-1.100384108	0.251374874	-4.3774626	1.20E-05	4.60E-05
ENSG00000065923	SLC9A7	726.6302773	991.46999	461.7905644	-1.100729758	0.091784229	-11.992581	3.89E-33	5.16E-32
ENSG00000001617	SEMA3F	5730.696907	7816.6561	3644.737757	-1.10078836	0.037085564	-29.682395	1.30E-193	9.24E-192
ENSG00000112964	GHR	22.62928402	30.925932	14.33263557	-1.101177517	0.456672419	-2.4113073	0.015895	0.036865
ENSG00000102271	KLHL4	1508.298302	2058.8332	957.763392	-1.103279984	0.05873656	-18.783531	1.03E-78	2.99E-77
ENSG00000150510	FAM124A	683.3040075	932.51975	434.0882602	-1.103853113	0.089743009	-12.300157	9.04E-35	1.24E-33
ENSG00000119686	FLVCR2	169.8709084	232.07221	107.6696094	-1.106178686	0.168882075	-6.5500065	5.75E-11	3.42E-10
ENSG00000147408	CSGALNACT1	2692.771913	3680.0476	1705.496248	-1.109195711	0.045123394	-24.581389	2.00E-133	9.59E-132
ENSG00000162746	FCRLB	39.61934983	54.131013	25.10768806	-1.110646406	0.363159864	-3.0582851	0.002226	0.006209
ENSG00000125864	BFSP1	170.8778027	233.58819	108.167418	-1.111036884	0.178127315	-6.237319	4.45E-10	2.49E-09
ENSG00000163637	PRICKLE2	756.3545899	1034.5463	478.1628663	-1.1126099	0.085446976	-13.021056	9.29E-39	1.41E-37
ENSG00000138356	AOX1	27.49723769	37.669338	17.32513784	-1.112839274	0.435095927	-2.5576872	0.010537	0.025455
ENSG00000205476	CCDC85C	1268.202318	1734.2438	802.1608779	-1.113238512	0.070573497	-15.774172	4.68E-56	9.67E-55
ENSG00000135069	PSAT1	927.5751523	1270.2097	584.9406504	-1.11694758	0.080377115	-13.896338	6.67E-44	1.12E-42
ENSG00000137198	GMPR	1214.195723	1661.9019	766.4895669	-1.117514595	0.064589896	-17.301694	4.57E-67	1.13E-65
ENSG00000038295	TLL1	754.7135055	1032.8019	476.6251412	-1.118305519	0.090385895	-12.372567	3.68E-35	5.09E-34
ENSG00000167861	HID1	1107.529811	1516.7831	698.2765684	-1.118993553	0.068162782	-16.416489	1.46E-60	3.25E-59
ENSG00000085563	ABCB1	280.0415157	383.57367	176.5093662	-1.120596466	0.14006736	-8.0004111	1.24E-15	9.35E-15
ENSG00000154065	ANKRD29	44.29850177	60.867453	27.72955009	-1.122016963	0.378641344	-2.9632711	0.003044	0.008244
ENSG00000139182	CLSTN3	2004.368599	2748.773	1259.964162	-1.124403597	0.05208195	-21.589122	2.27E-103	8.86E-102
ENSG00000147862	NFIB	9471.941339	12996.009	5947.873358	-1.127030831	0.034696151	-32.482877	1.86E-231	1.65E-229
ENSG00000257337	AC068888.1	31.93338887	43.89114	19.97563775	-1.127439495	0.404183918	-2.789422	0.00528	0.013599
ENSG00000250144	AC104619.3	22.85581799	31.278221	14.43341512	-1.128752158	0.468949867	-2.4069783	0.016085	0.037248
ENSG00000173221	GLRX	414.8064348	569.50707	260.1058023	-1.130313078	0.107806123	-10.484683	1.02E-25	1.12E-24
ENSG00000145949	MYLK4	26.96150976	36.952796	16.97022329	-1.132347301	0.43736205	-2.5890388	0.009624	0.023451
ENSG00000213347	MXD3	290.3486329	398.7006	181.9966644	-1.132602669	0.131003889	-8.6455652	5.35E-18	4.46E-17
ENSG00000234618	RPSAP9	47.93820602	65.879087	29.99732523	-1.133368672	0.326179629	-3.4746764	0.000511	0.001587
ENSG00000111341	MGP	21648.15036	29744.167	13552.13416	-1.134138875	0.027766013	-40.846299	0	0
ENSG00000161714	PLCD3	881.9365308	1212.093	551.780022	-1.136407816	0.087724848	-12.95423	2.22E-38	3.35E-37
ENSG00000054654	SYNE2	2896.099577	3981.1148	1811.08431	-1.13678352	0.044495709	-25.548161	5.76E-144	2.96E-142
ENSG00000112984	KIF20A	1965.33879	2703.0026	1227.675021	-1.139344664	0.055364258	-20.579065	4.23E-94	1.53E-92
ENSG00000119403	PHF19	2347.301915	3230.7163	1463.887535	-1.14071752	0.058602065	-19.465483	2.15E-84	6.90E-83
ENSG00000165113	GKAP1	68.91764009	94.838301	42.99697962	-1.140789097	0.26302207	-4.3372372	1.44E-05	5.48E-05
ENSG00000175556	LONRF3	330.0892793	454.43364	205.7449209	-1.141221235	0.136073968	-8.3867712	5.00E-17	4.00E-16
ENSG00000159733	ZFYVE28	249.4386504	343.54217	155.3351336	-1.144676261	0.136482233	-8.3869983	4.99E-17	3.99E-16
ENSG00000213203	GIMAP1	1106.05464	1523.5634	688.545869	-1.145033991	0.073160305	-15.651028	3.27E-55	6.66E-54
ENSG00000116833	NR5A2	834.9201838	1149.9483	519.8920517	-1.145375588	0.076688182	-14.93549	1.94E-50	3.67E-49
ENSG00000166689	PLEKHA7	69.08567269	95.151323	43.02002271	-1.146446684	0.276203872	-4.1507263	3.31E-05	0.000121
ENSG00000146054	TRIM7	78.29415963	108.16013	48.42819238	-1.155057953	0.250524843	-4.6105525	4.02E-06	1.61E-05
ENSG00000272913	AC009237.14	29.05510827	40.28214	17.82807627	-1.15802136	0.460846612	-2.512813	0.011977	0.028574
ENSG00000158528	PPP1R9A	90.49180006	124.98372	55.99988029	-1.160132133	0.250329666	-4.6344173	3.58E-06	1.45E-05
ENSG00000225968	ELFN1	78.29755438	108.16202	48.43309115	-1.160347964	0.25287082	-4.5886985	4.46E-06	1.78E-05
ENSG00000174640	SLCO2A1	228.8461209	316.26931	141.4229328	-1.162699925	0.1503582	-7.7328667	1.05E-14	7.64E-14
ENSG00000187994	RINL	173.8142779	240.5728	107.0557606	-1.164785398	0.180110733	-6.4670515	9.99E-11	5.84E-10
ENSG00000167972	ABCA3	4298.630922	5947.9882	2649.273645	-1.166713777	0.038770916	-30.092499	6.07E-199	4.50E-197
ENSG00000158106	RHPN1	273.2287888	378.24429	168.2132845	-1.16834606	0.13396912	-8.7210102	2.76E-18	2.32E-17
ENSG00000124593	AL365205.1	33.27864549	46.011905	20.54538628	-1.169755311	0.393237702	-2.9746774	0.002933	0.007981
ENSG00000100504	PYGL	1520.739471	2107.406	934.0729435	-1.172765081	0.060128781	-19.504222	1.01E-84	3.27E-83
ENSG00000284946	AC068831.7	20.62787741	28.603002	12.65275328	-1.174380735	0.481316518	-2.4399344	0.01469	0.034377
ENSG00000074527	NTN4	10646.59789	14759.676	6533.519538	-1.175893732	0.032792383	-35.858746	1.34E-281	1.51E-279
ENSG00000151689	INPP1	989.3454663	1371.6313	607.0596791	-1.176029923	0.07340574	-16.020953	9.12E-58	1.94E-56
ENSG00000261253	AC137932.2	21.57315452	29.978632	13.16767681	-1.176090544	0.485239561	-2.4237318	0.015362	0.035763
ENSG00000196730	DAPK1	5598.887788	7762.7167	3435.058828	-1.176234946	0.036880939	-31.89276	3.36E-223	2.82E-221

ENSG00000249867	AC090833.1	24.33284488	33.693381	14.97230885	-1.178640464	0.442076135	-2.6661481	0.007673	0.01909
ENSG00000154217	PITPNC1	441.485912	612.32279	270.6490375	-1.179216837	0.108116514	-10.906908	1.07E-27	1.24E-26
ENSG00000105996	HOXA2	49.53295557	68.805247	30.26066401	-1.182051283	0.325520177	-3.6312689	0.000282	0.00091
ENSG00000179546	HTR1D	106.4869498	147.64832	65.32557798	-1.182279026	0.22498838	-5.2548448	1.48E-07	6.83E-07
ENSG00000165887	ANKRD2	41.02536361	56.945025	25.10570223	-1.183309355	0.367932641	-3.2161032	0.001299	0.003768
ENSG00000232445	AC006329.1	23.32996095	32.397574	14.26234814	-1.183609725	0.480847412	-2.4615079	0.013835	0.03258
ENSG00000112294	ALDH5A1	28.89296429	40.101905	17.68402404	-1.184067824	0.412901645	-2.8676752	0.004135	0.010891
ENSG00000185201	IFITM2	4022.62711	5587.4337	2457.820476	-1.184425361	0.045199561	-26.204355	2.37E-151	1.32E-149
ENSG00000216775	AL109918.1	72.80927205	101.07666	44.54188184	-1.184602309	0.286798291	-4.1304371	3.62E-05	0.000132
ENSG00000101447	FAM83D	1132.288231	1572.8284	691.7480754	-1.18474725	0.069910331	-16.946669	2.04E-64	4.84E-63
ENSG00000176485	PLA2G16	1048.71217	1456.6524	640.7719145	-1.184919392	0.074131769	-15.983962	1.65E-57	3.48E-56
ENSG00000180096	1-Sep	30.32888924	42.128814	18.52896482	-1.185566223	0.409452572	-2.895491	0.003786	0.01005
ENSG00000101000	PROCR	9072.156843	12602.907	5541.406823	-1.185602354	0.029370594	-40.366986	0	0
ENSG00000266714	MYO15B	134.7961641	187.34844	82.24388922	-1.186776727	0.228858802	-5.1856285	2.15E-07	9.80E-07
ENSG00000162687	KCNT2	20.12438404	28.02029	12.2284784	-1.189275319	0.501270485	-2.3725221	0.017667	0.040388
ENSG00000101353	MROH8	28.72693281	40.004674	17.44919153	-1.190201823	0.420381172	-2.8312444	0.004637	0.012087
ENSG00000134072	CAMK1	578.677026	805.16701	352.1870372	-1.191617361	0.092081092	-12.940956	2.64E-38	3.98E-37
ENSG00000144712	CAND2	259.7763064	361.41512	158.1374974	-1.191632684	0.142118763	-8.3847668	5.08E-17	4.07E-16
ENSG00000235944	ZNF815P	53.34945924	74.322808	32.37611024	-1.191777169	0.304295758	-3.9165093	8.98E-05	0.000311
ENSG00000160796	NBEAL2	3706.333187	5156.9237	2255.742695	-1.192199207	0.040155501	-29.689561	1.05E-193	7.49E-192
ENSG00000279673	AC092919.2	57.04892637	79.492605	34.60524822	-1.19442118	0.311296745	-3.8369215	0.000125	0.000423
ENSG00000170421	KRT8	766.7063603	1067.4035	466.0092407	-1.195456334	0.082745504	-14.447387	2.60E-47	4.71E-46
ENSG00000165821	SALL2	237.2971279	330.58611	144.0081435	-1.196559775	0.146780444	-8.1520381	3.58E-16	2.76E-15
ENSG00000111144	LTA4H	4283.109528	5968.0949	2598.124198	-1.198889879	0.041775148	-28.698638	3.97E-181	2.69E-179
ENSG00000138172	CALHM2	907.4507753	1265.1067	549.794856	-1.199764581	0.081981201	-14.63463	1.69E-48	3.09E-47
ENSG00000175592	FOSL1	1944.386935	2709.495	1179.278915	-1.200321085	0.052400332	-22.906746	3.98E-116	1.66E-114
ENSG00000176046	NUPR1	643.6136822	897.88685	389.3405142	-1.203296844	0.097384983	-12.356082	4.52E-35	6.24E-34
ENSG00000099875	MKNK2	4872.559402	6796.9388	2948.179972	-1.205756123	0.042665252	-28.260846	1.05E-175	6.76E-174
ENSG00000104332	SFRP1	436.3474095	608.86106	263.8337564	-1.206024773	0.115082216	-10.47968	1.07E-25	1.18E-24
ENSG00000183018	AC118754.1	2600.5329	3628.6095	1572.456308	-1.206282476	0.051579444	-23.386884	5.81E-121	2.51E-119
ENSG00000185950	IRS2	355.245645	496.08266	214.4086276	-1.206540522	0.133724655	-9.0225735	1.84E-19	1.62E-18
ENSG00000016082	ISL1	27.05698961	37.861166	16.25281356	-1.208341697	0.424561495	-2.8460935	0.004426	0.011583
ENSG00000114115	RBP1	35.6757844	49.828479	21.52309023	-1.208489601	0.432967501	-2.7911785	0.005252	0.013535
ENSG00000128815	WDFY4	232.5646031	324.92546	140.2037428	-1.208868702	0.148513505	-8.1397897	3.96E-16	3.05E-15
ENSG00000162545	CAMK2N1	755.5365204	1055.267	455.8059927	-1.208920655	0.095252473	-12.691751	6.57E-37	9.46E-36
ENSG00000160789	LMNA	24147.38995	33727.536	14567.24362	-1.211210009	0.024463964	-49.509964	0	0
ENSG00000108798	ABI3	1620.434931	2264.4089	976.4609465	-1.214307646	0.061390719	-19.779987	4.43E-87	1.47E-85
ENSG00000272767	JMJD1C-AS1	22.88548728	32.008652	13.76232263	-1.216993884	0.503160112	-2.418701	0.015576	0.036197
ENSG00000135914	HTR2B	159.1481504	222.8895	95.40679889	-1.224273789	0.174265735	-7.0253271	2.14E-12	1.38E-11
ENSG00000235257	ITGA9-AS1	49.21880904	68.944854	29.49276365	-1.224521711	0.323544838	-3.7847048	0.000154	0.000516
ENSG00000285533	AP001362.2	37.08934829	51.943429	22.23526784	-1.226677814	0.382515483	-3.206871	0.001342	0.00388
ENSG00000147174	GCNA	29.45064288	41.260666	17.64061974	-1.228271082	0.425231187	-2.8884784	0.003871	0.010256
ENSG00000137693	YAP1	2307.531232	3235.59	1379.472512	-1.229364305	0.056035674	-21.938958	1.10E-106	4.36E-105
ENSG00000015133	CCDC88C	1349.725682	1892.7899	806.6614701	-1.230035583	0.063758079	-19.292231	6.24E-83	1.96E-81
ENSG00000215386	MIR99AHG	96.82399524	135.76097	57.88701593	-1.230233595	0.221907489	-5.543903	2.96E-08	1.45E-07
ENSG00000176472	ZNF575	38.6872348	54.314622	23.0598474	-1.23185171	0.376273017	-3.2738242	0.001061	0.003124
ENSG00000163884	KLF15	91.6497291	128.69099	54.60846514	-1.232995859	0.233694896	-5.2760924	1.32E-07	6.11E-07
ENSG00000149582	TMEM25	609.6119527	855.71196	363.5119419	-1.237757065	0.092953324	-13.315899	1.87E-40	2.97E-39
ENSG00000157570	TSPAN18	4940.06012	6940.7031	2939.4171	-1.238635274	0.041120912	-30.121785	2.51E-199	1.88E-197
ENSG00000244041	LINC01011	25.85183699	36.409493	15.29418104	-1.244064203	0.454029404	-2.7400521	0.006143	0.015611
ENSG00000243244	STON1	596.5829958	839.45617	353.7098234	-1.245192934	0.108540996	-11.472098	1.82E-30	2.29E-29
ENSG00000136153	LMO7	1146.89	1613.6576	680.1223879	-1.24681872	0.070733755	-17.626927	1.53E-69	3.90E-68
ENSG00000141664	ZCCHC2	1026.706411	1445.0785	608.3343286	-1.246993825	0.080617872	-15.467958	5.71E-54	1.14E-52
ENSG00000182022	CHST15	2474.056333	3482.8554	1465.257229	-1.247631006	0.052687851	-23.679672	5.84E-124	2.58E-122
ENSG00000144118	RALB	7801.630577	10980.204	4623.057029	-1.248015856	0.030159598	-41.380388	0	0
ENSG00000172927	MYEOV	363.0082159	510.95744	215.0589962	-1.248208358	0.122437666	-10.194644	2.10E-24	2.21E-23
ENSG00000166851	PLK1	2069.441099	2913.1466	1225.735611	-1.248822785	0.05008228	-24.935422	3.07E-137	1.51E-135
ENSG00000171435	KSR2	349.9911503	493.2614	206.7208995	-1.249951633	0.13629501	-9.1709273	4.69E-20	4.23E-19
ENSG00000146021	KLHL3	871.69775	1227.8753	515.5202188	-1.250197303	0.077887157	-16.051392	5.59E-58	1.20E-56
ENSG00000247809	NR2F2-AS1	42.01203251	59.319364	24.70470151	-1.252912748	0.359863212	-3.4816361	0.000498	0.00155
ENSG00000058668	ATP2B4	8783.670941	12376.579	5190.762465	-1.253308381	0.032822908	-38.183953	0	0
ENSG00000197822	OCLN	303.3229175	427.28279	179.3630483	-1.253411594	0.125320229	-10.00167	1.50E-23	1.54E-22
ENSG00000267279	AC090409.1	27.54660351	38.783018	16.3101888	-1.253501336	0.421334762	-2.9750722	0.002929	0.007974
ENSG00000185860	CCDC190	40.87225114	57.519696	24.22480616	-1.253929982	0.359035527	-3.4924956	0.000479	0.001491
ENSG00000166262	FAM227B	34.99378822	49.299065	20.6885114	-1.254838293	0.369156728	-3.399202	0.000676	0.00206
ENSG00000236824	BCYRN1	332.7634506	469.07553	196.4513678	-1.255697839	0.123026979	-10.206687	1.85E-24	1.96E-23
ENSG00000092621	PHGDH	574.248179	809.22154	339.2748173	-1.25634387	0.097675955	-12.862366	7.33E-38	1.09E-36
ENSG00000134323	MYCN	48.75468693	68.885884	28.62348998	-1.258014815	0.320004739	-3.9312381	8.45E-05	0.000293
ENSG00000197582	GPX1P1	148.466795	209.4087	87.52489053	-1.26061118	0.187706225	-6.7158731	1.87E-11	1.15E-10
ENSG00000126460	PRRG2	42.17617526	59.455525	24.89682575	-1.261520209	0.341984743	-3.6888201	0.000225	0.000738
ENSG00000088305	DNMT3B	235.0672994	331.69672	138.4378796	-1.263133282	0.147022582	-8.5914236	8.59E-18	7.09E-17
ENSG00000166265	CYYR1	1410.548818	1992.0959	829.0016909	-1.263261896	0.064001413	-19.738031	1.02E-86	3.36E-85
ENSG00000133315	MACROD1	303.3059468	428.37692	178.234972	-1.265413819	0.134389248	-9.4160347	4.68E-21	4.40E-20
ENSG00000188906	LRRK2	46.48655774	65.603247	27.3698684	-1.265965348	0.319289782	-3.9649416	7.34E-05	0.000257
ENSG00000115756	HPCAL1	2153.700724	3042.8765	1264.52494	-1.266027888	0.056856301	-22.267152	7.69E-110	3.08E-108
ENSG00000145687	SSBP2	322.838345	455.93343	189.7432593	-1.267199469	0.127717699	-9.9218783	3.34E-23	3.39E-22
ENSG00000103145	HCFC1R1	473.4238689	669.83481	277.012924	-1.269250113	0.113735062	-11.159708	6.42E-29	7.77E-28
ENSG00000183091	NEB	64.34879475	90.966944	37.73064527	-1.269531195	0.297598629	-4.2659175	1.99E-05	7.46E-05
ENSG00000176927	EFCAB5	20.40719988	28.903954	11.91044625	-1.270060073	0.49255171	-2.5785314	0.009922	0.024128
ENSG00000129757	CDKN1C	109.4275683	155.02629	63.82884721	-1.271294536	0.236219854	-5.3818276	7.37E-08	3.50E-07
ENSG00000162817	C1orf115	1314.532216	1859.897	769.1674529	-1.272301844	0.071233388	-17.861032	2.37E-71	6.20E-70
ENSG00000063660	GPC1	2359.391674	3340.8267	1377.956607	-1.277443667	0.050049216	-25.52375	1.07E-143	5.52E-142
ENSG00000130702	LAMA5	5939.963295	8410.819	3469.107603	-1.277621906	0.036963733	-34.564202	8.72E-262	9.01E-260
ENSG00000111057	KRT18	5868.305953	8310.7148	3425.897114	-1.278791647	0.039367456	-32.483472	1.83E-231	1.62E-229
ENSG00000173200	PARP15	50.64483575	71.691592	29.59807927	-1.279534186	0.329035461	-3.8887425	0.000101	0.000346

ENSG00000276644	DACH1	937.9364568	1330.5211	545.3518114	-1.284388693	0.091268509	-14.072638	5.59E-45	9.63E-44
ENSG00000160886	LY6K	63.13271087	89.415673	36.8497492	-1.284800797	0.278334081	-4.6160384	3.91E-06	1.57E-05
ENSG00000134291	TMEM106C	1224.031294	1735.8448	712.2177404	-1.286072839	0.064638551	-19.896375	4.37E-88	1.47E-86
ENSG00000260992	DOCK9-DT	17.04586719	24.222607	9.869127499	-1.289143764	0.538339837	-2.3946654	0.016636	0.038373
ENSG00000025434	NR1H3	130.9212959	185.73487	76.1077168	-1.289977032	0.199261936	-6.4737755	9.56E-11	5.60E-10
ENSG00000180537	RNF182	95.3036109	135.2249	55.382323	-1.293654594	0.245949475	-5.2598388	1.44E-07	6.66E-07
ENSG00000156463	SH3RF2	25.0727903	35.616981	14.52859985	-1.29521375	0.445689687	-2.9060887	0.00366	0.009748
ENSG00000173267	SNCG	728.9643812	1036.2347	421.6940463	-1.297407229	0.086628391	-14.976698	1.04E-50	1.99E-49
ENSG00000154102	C16orf74	265.3568778	377.46894	153.2448157	-1.298344846	0.13455918	-9.648876	4.97E-22	4.84E-21
ENSG00000253882	AC099548.2	50.02691828	71.344234	28.70960256	-1.300609456	0.332301078	-3.913949	9.08E-05	0.000314
ENSG00000151276	MAGI1	2249.697069	3202.3608	1297.033381	-1.302829862	0.049824494	-26.148381	1.03E-150	5.68E-149
ENSG00000140876	NUDT7	41.28844112	58.7838	23.79308227	-1.305830514	0.336121695	-3.8849932	0.000102	0.000352
ENSG00000152518	ZFP36L2	4154.06696	5916.863	2391.27088	-1.30680402	0.08836979	-14.787905	1.75E-49	3.26E-48
ENSG00000196502	SULT1A1	15.68150086	22.30286	9.060142009	-1.307677742	0.559395989	-2.3376602	0.019405	0.043967
ENSG00000066027	PPP2R5A	1757.377167	2505.2599	1009.494479	-1.309756962	0.06600764	-19.842506	1.28E-87	4.27E-86
ENSG00000186665	C17orf58	904.2377534	1288.7499	519.7256524	-1.312370545	0.078828342	-16.648461	3.10E-62	7.15E-61
ENSG00000211772	TRBC2	1166.452847	1664.3367	668.5689708	-1.315962976	0.070339247	-18.708801	4.20E-78	1.21E-76
ENSG00000107485	GATA3	1178.859313	1682.6323	675.0863471	-1.316312524	0.067044118	-19.633527	8.00E-86	2.61E-84
ENSG00000108984	MAP2K6	561.9527255	802.18203	321.7234248	-1.317573633	0.093633656	-14.071582	5.68E-45	9.76E-44
ENSG00000179314	WSCD1	2691.824587	3846.3232	1537.326005	-1.323156288	0.044602579	-29.665466	2.14E-193	1.52E-191
ENSG00000124406	ATP8A1	138.6011711	198.38268	78.81966306	-1.326926149	0.195931418	-6.7724011	1.27E-11	7.82E-11
ENSG00000130052	STARD8	947.6597787	1355.2312	540.0883199	-1.327567221	0.07492824	-17.717849	3.05E-70	7.85E-69
ENSG00000179981	TSHZ1	1711.618075	2448.5818	974.6543902	-1.330461292	0.058432541	-22.769184	9.27E-115	3.81E-113
ENSG00000127824	TUBA4A	521.1122189	745.82258	296.4018621	-1.333020646	0.104053105	-12.810965	1.42E-37	2.10E-36
ENSG00000149922	TBX6	18.52746446	26.620025	10.43490436	-1.336263857	0.52780413	-2.531742	0.01135	0.027208
ENSG00000204588	LINC01123	27.15157165	38.941588	15.36155554	-1.3364524	0.413677526	-3.2306623	0.001235	0.003595
ENSG00000167600	CYP2S1	28.32997058	40.713175	15.94676651	-1.336796348	0.455202028	-2.93671	0.003317	0.008913
ENSG00000183023	SLC8A1	26.98087297	38.725867	15.2358787	-1.337440266	0.431417457	-3.100107	0.001935	0.005454
ENSG00000204634	TBC1D8	1317.661818	1888.7006	746.623082	-1.338989659	0.068601511	-19.51837	7.66E-85	2.49E-83
ENSG00000197308	GATA3-AS1	33.51873351	48.134154	18.90331348	-1.339109268	0.393661334	-3.4016784	0.00067	0.002042
ENSG00000117707	PROX1	392.1018062	561.85941	222.3442028	-1.340331166	0.113323323	-11.827496	2.81E-32	3.67E-31
ENSG00000106688	SLC1A1	517.8149657	742.64207	292.9878662	-1.341640742	0.101701068	-13.192002	9.76E-40	1.51E-38
ENSG00000138587	MNS1	81.2431847	116.40647	46.07989947	-1.34274985	0.253409903	-5.2987268	1.17E-07	5.43E-07
ENSG00000235505	CASP17P	890.9589968	1278.7121	503.2058589	-1.342903763	0.079387997	-16.915703	3.45E-64	8.17E-63
ENSG00000163554	SPTA1	26.97560709	38.663786	15.28742806	-1.343206959	0.460076913	-2.919527	0.003506	0.009373
ENSG00000123080	CDKN2C	468.5233447	672.36173	264.6849586	-1.344342233	0.109277806	-12.302061	8.83E-35	1.21E-33
ENSG00000186205	1-Mar	34.24891695	49.139753	19.35808047	-1.344355269	0.380526059	-3.5328862	0.000411	0.001293
ENSG00000114812	VIPR1	35.02439475	50.215581	19.83320867	-1.345401259	0.404589857	-3.325346	0.000883	0.002635
ENSG00000010438	PRSS3	430.1883447	617.6427	242.7339846	-1.347139303	0.1054718	-12.772507	2.34E-37	3.41E-36
ENSG00000131149	GSE1	638.3538298	916.85547	359.8521872	-1.3491929	0.099888744	-13.506956	1.42E-41	2.31E-40
ENSG00000167601	AXL	6105.183379	8770.8501	3439.516628	-1.350806864	0.034061136	-39.658303	0	0
ENSG00000204385	SLC44A4	17.72761879	25.50675	9.948487097	-1.352456157	0.555620224	-2.4341377	0.014927	0.034879
ENSG00000130707	ASS1	175.3501858	252.43595	98.26442052	-1.354421616	0.182646036	-7.4155544	1.21E-13	8.38E-13
ENSG00000049089	COL9A2	19.37118729	27.856312	10.88606237	-1.354739263	0.522429986	-2.5931499	0.00951	0.023209
ENSG00000132639	SNAP25	22.12111139	31.774376	12.46784699	-1.357306814	0.493095936	-2.7526222	0.005912	0.015087
ENSG00000151623	NR3C2	501.1687157	721.67184	280.6655923	-1.360710779	0.106225804	-12.809607	1.45E-37	2.13E-36
ENSG00000167767	KRT80	769.2466172	1107.7909	430.7023085	-1.364966299	0.084673379	-16.120371	1.84E-58	3.97E-57
ENSG00000146856	AGBL3	39.33059853	56.734889	21.92630785	-1.367765385	0.384126705	-3.5607141	0.00037	0.001171
ENSG00000187210	GCNT1	856.1613591	1234.5251	477.7976191	-1.368470028	0.083485458	-16.391717	2.19E-60	4.88E-59
ENSG00000275322	AC103746.1	59.12876309	85.433867	32.82365927	-1.368653788	0.309674715	-4.4196498	9.89E-06	3.82E-05
ENSG00000088756	ARHGAP28	1166.817045	1683.4593	650.1753194	-1.370479284	0.072016406	-19.030098	9.61E-81	2.92E-79
ENSG00000157617	C2CD2	1892.83811	2730.722	1054.954231	-1.372500093	0.053142125	-25.82697	4.42E-147	2.34E-145
ENSG00000169247	SH3TC2	89.9442088	129.8895	49.99891901	-1.375625564	0.233265319	-5.8972571	3.70E-09	1.94E-08
ENSG00000186767	SPIN4	334.3790089	483.0043	185.7537184	-1.377452189	0.136229847	-10.111236	4.93E-24	5.14E-23
ENSG00000102935	ZNF423	155.0568816	224.20698	85.90678793	-1.3782568	0.186073354	-7.4070616	1.29E-13	8.91E-13
ENSG00000117399	CDC20	2502.77088	3615.0299	1390.511859	-1.378828058	0.049567041	-27.817437	2.67E-170	1.64E-168
ENSG00000106351	AGFG2	1191.308964	1720.7478	661.8701683	-1.379870532	0.069639743	-19.814412	2.24E-87	7.45E-86
ENSG00000198865	CCDC152	113.2382243	163.70711	62.76933569	-1.388356448	0.212150364	-6.5442096	5.98E-11	3.55E-10
ENSG00000100285	NEFH	380.6958895	550.60496	210.7868169	-1.388782671	0.120752591	-11.501059	1.30E-30	1.65E-29
ENSG00000149809	TM7SF2	196.2837906	284.57739	107.9901918	-1.395023239	0.158295735	-8.8127658	1.22E-18	1.04E-17
ENSG00000243232	PCDHAC2	88.63398406	128.81104	48.45692972	-1.400789774	0.248581839	-5.6351252	1.75E-08	8.73E-08
ENSG00000125457	MIF4GD	300.3341277	436.20921	164.4590411	-1.406840169	0.126299252	-11.138943	8.11E-29	9.77E-28
ENSG00000186193	SAPCD2	880.0670164	1278.7642	481.3698398	-1.410282196	0.085003394	-16.590893	8.11E-62	1.85E-60
ENSG00000178445	GLDC	226.0854421	328.71707	123.4538167	-1.410603018	0.154161968	-9.1501363	5.69E-20	5.12E-19
ENSG00000213025	COX20P1	27.64448647	40.257435	15.03153829	-1.41341509	0.419664919	-3.367961	0.000757	0.002286
ENSG00000285922	AC104453.1	22.9917972	33.541703	12.44189096	-1.416635017	0.495875217	-2.8568377	0.004279	0.011231
ENSG00000171357	LURAP1	62.40329868	90.954945	33.85165213	-1.4177389	0.293995994	-4.8223069	1.42E-06	5.99E-06
ENSG00000198721	ECI2	2633.206792	3836.0441	1430.3695	-1.423246781	0.047651375	-29.867906	5.14E-196	3.72E-194
ENSG00000106852	LHX6	318.3216389	463.74969	172.8935831	-1.426096394	0.144227075	-9.8878549	4.70E-23	4.73E-22
ENSG00000141753	IGFBP4	37042.53283	54006.537	20078.5286	-1.427346465	0.028236496	-50.549702	0	0
ENSG00000005469	CROT	558.9876414	815.25595	302.7193318	-1.428807037	0.093416465	-15.295024	8.25E-53	1.62E-51
ENSG00000132622	HSPA12B	556.6139085	811.64842	301.579402	-1.430513061	0.104310733	-13.713958	8.38E-43	1.39E-41
ENSG00000261485	PAN3-AS1	17.08275918	24.951889	9.213629087	-1.430786393	0.525616895	-2.7221088	0.006487	0.016419
ENSG00000275719	AC008622.2	18.01982224	26.249181	9.790463939	-1.432012887	0.521252614	-2.7472532	0.00601	0.015302
ENSG00000196196	HRCT1	101.1892256	147.56005	54.81840035	-1.432492103	0.234038011	-6.1207669	9.31E-10	5.09E-09
ENSG00000131781	FMO5	20.95350093	30.592129	11.31487332	-1.432594232	0.505130451	-2.8360876	0.004567	0.011923
ENSG00000111145	ELK3	12326.23618	17993.368	6659.104258	-1.433430374	0.0332563	-43.102521	0	0
ENSG00000169252	ADRB2	58.80977418	85.739855	31.87969372	-1.433531212	0.286803706	-4.9983009	5.78E-07	2.53E-06
ENSG00000271452	AC005034.5	14.87537646	21.788891	7.961861711	-1.433750153	0.610854316	-2.3471229	0.018919	0.042961
ENSG00000198959	TGM2	59012.04561	86139.834	31884.2574	-1.433785116	0.020537039	-69.814599	0	0
ENSG00000198846	TOX	651.6295623	951.82837	351.4307592	-1.434451948	0.092638304	-15.484437	4.42E-54	8.86E-53
ENSG00000100399	CHADL	19.89230629	29.001184	10.78342861	-1.43484435	0.489564098	-2.9308611	0.00338	0.009068
ENSG00000239887	C1orf226	192.4456043	281.2463	103.6449116	-1.437632637	0.167952309	-8.559767	1.13E-17	9.30E-17
ENSG00000169083	AR	1398.960837	2046.0914	751.8302276	-1.442176849	0.067136985	-21.481108	2.34E-102	9.02E-101
ENSG00000128408	RIBC2	51.00293066	74.661949	27.34391237	-1.443224589	0.316253859	-4.5635003	5.03E-06	2.00E-05

ENSG00000152642	GPD1L	579.3479334	846.75648	311.9393833	-1.444114466	0.101279334	-14.258728	3.96E-46	6.95E-45
ENSG00000177076	ACER2	432.1151981	632.36897	231.8614282	-1.444724202	0.118791494	-12.161849	4.96E-34	6.69E-33
ENSG00000225792	AC004540.2	58.33930825	85.342715	31.33590122	-1.444953725	0.330575324	-4.3710272	1.24E-05	4.74E-05
ENSG00000184005	ST6GALNAC3	1496.626375	2189.8574	803.3953987	-1.445789256	0.062969122	-22.960289	1.16E-116	4.88E-115
ENSG00000174370	C11orf45	37.56771639	55.067161	20.06827224	-1.447007998	0.386025244	-3.7484802	0.000178	0.000591
ENSG00000135119	RNFT2	57.81096939	84.507826	31.11411253	-1.447443145	0.314568861	-4.6013555	4.20E-06	1.68E-05
ENSG00000174004	NRROS	477.8905983	700.66445	255.1167455	-1.453442237	0.112956457	-12.867279	6.88E-38	1.02E-36
ENSG00000013588	GPRC5A	724.5182177	1061.7292	387.3072117	-1.455057886	0.092595122	-15.714196	1.21E-55	2.48E-54
ENSG00000115425	PECR	106.6168187	156.47215	56.76148981	-1.455167027	0.238758036	-6.0947353	1.10E-09	5.96E-09
ENSG00000113594	LIFR	2127.065829	3117.4988	1136.632834	-1.455172322	0.056515772	-25.748075	3.39E-146	1.79E-144
ENSG00000128917	DLL4	3600.6323	5281.3857	1919.878928	-1.458659499	0.047647776	-30.613381	8.12E-206	6.25E-204
ENSG00000130590	SAMD10	140.1429462	205.64279	74.64310128	-1.458948622	0.210011594	-6.9469909	3.73E-12	2.38E-11
ENSG00000154856	APCDD1	73.97173485	108.45067	39.49280011	-1.46058788	0.266804883	-5.4743671	4.39E-08	2.12E-07
ENSG00000075213	SEMA3A	240.6010828	353.09017	128.1119993	-1.463716805	0.147667761	-9.91223	3.68E-23	3.73E-22
ENSG00000005108	THSD7A	2158.476544	3169.6044	1147.348698	-1.464963798	0.051582195	-28.400571	1.99E-177	1.29E-175
ENSG00000226278	PSPHP1	25.44040393	37.306304	13.57450341	-1.466234876	0.446006183	-3.2874766	0.001011	0.002989
ENSG00000168309	FAM107A	930.9040173	1367.9211	493.886917	-1.469230377	0.076494393	-19.207034	3.23E-82	1.01E-80
ENSG00000116396	KCNC4	746.0269746	1096.5418	395.5121505	-1.469444692	0.091720993	-16.020811	9.14E-58	1.94E-56
ENSG00000100312	ACR	14.0912071	20.769808	7.412606029	-1.472467457	0.618568282	-2.3804445	0.017292	0.039663
ENSG00000103647	CORO2B	192.5276968	283.03845	102.0169413	-1.476684927	0.168205421	-8.7790567	1.65E-18	1.40E-17
ENSG00000100065	CARD10	2022.350034	2976.0584	1068.641657	-1.477721585	0.062631254	-23.593996	4.44E-123	1.94E-121
ENSG00000085831	TTC39A	30.74650024	45.244027	16.24897352	-1.480577653	0.412811208	-3.5865733	0.000335	0.001068
ENSG00000108622	ICAM2	11020.84039	16236.402	5805.279199	-1.483947089	0.029901012	-49.628658	0	0
ENSG00000245105	A2M-AS1	18.98707134	27.974802	9.999340422	-1.48636679	0.526407088	-2.8236071	0.004749	0.012342
ENSG00000154146	NRGN	996.7352781	1469.4023	524.0682508	-1.487439918	0.072382689	-20.549664	7.75E-94	2.78E-92
ENSG00000249992	TMEM158	64.21477703	94.719404	33.71015015	-1.492510213	0.275768239	-5.4121904	6.23E-08	2.97E-07
ENSG00000171388	APLN	15960.30807	23551.609	8369.007584	-1.492987316	0.030925278	-48.277248	0	0
ENSG00000184226	PCDH9	316.4625571	467.22828	165.6968375	-1.495575491	0.127442576	-11.735289	8.40E-32	1.09E-30
ENSG00000237523	LINC00857	13.80490696	20.374665	7.235148757	-1.500153601	0.597313628	-2.5115007	0.012022	0.028666
ENSG00000143126	CELSR2	77.03131229	113.82659	40.23603424	-1.501455279	0.27663538	-5.4275606	5.71E-08	2.73E-07
ENSG00000066248	NGEF	12.45841246	18.431432	6.485392713	-1.502484736	0.624819953	-2.4046683	0.016187	0.037461
ENSG00000094755	GABRP	35.84512762	53.074482	18.61577344	-1.506512553	0.392750559	-3.8357999	0.000125	0.000425
ENSG00000253161	LINC01605	26.19838561	38.860209	13.53656229	-1.508626571	0.445318179	-3.3877498	0.000705	0.002139
ENSG00000146122	DAAM2	18.67973957	27.624405	9.735074534	-1.513415415	0.510311422	-2.9656703	0.00302	0.008188
ENSG00000163814	CDCP1	126.3108613	187.26962	65.35209842	-1.515108467	0.213498584	-7.0965738	1.28E-12	8.38E-12
ENSG00000178878	APOLD1	437.1728963	648.14845	226.1973394	-1.516435064	0.108574182	-13.966811	2.49E-44	4.23E-43
ENSG00000231999	LRRC8C-DT	33.33303522	49.412147	17.25392329	-1.521908741	0.388643301	-3.9159526	9.00E-05	0.000311
ENSG00000165424	ZCCHC24	611.0678166	906.56758	315.5680493	-1.522511691	0.094905018	-16.042478	6.45E-58	1.38E-56
ENSG00000187556	NANOS3	20.34424195	30.261029	10.42745534	-1.523024175	0.517010025	-2.945831	0.003221	0.008676
ENSG00000127528	KLF2	496.8005455	737.56372	256.0373697	-1.529280298	0.122559389	-12.477871	9.86E-36	1.38E-34
ENSG00000057704	TMCC3	3036.294751	4511.4094	1561.180069	-1.529991752	0.046678946	-32.776913	1.26E-235	1.15E-233
ENSG00000244968	LIFR-AS1	21.40910416	31.852786	10.96542196	-1.530355629	0.519741809	-2.9444536	0.003235	0.008713
ENSG00000274355	FAM74A3	46.27371006	68.742353	23.80506736	-1.53104243	0.326621644	-4.6875106	2.77E-06	1.13E-05
ENSG00000164318	EGFLAM	67.88084997	101.0068	34.75489526	-1.53292933	0.27094808	-5.6576497	1.53E-08	7.69E-08
ENSG00000147113	DIPK2B	7465.094363	11097.88	3832.308883	-1.53325763	0.033292201	-46.054559	0	0
ENSG00000270885	RASL10B	23.04028393	34.229351	11.8512168	-1.534680342	0.4698278	-3.2664741	0.001089	0.0032
ENSG00000106511	MEOX2	636.8149131	949.03269	324.597132	-1.545045676	0.115939025	-13.326364	1.63E-40	2.59E-39
ENSG00000167642	SPINT2	117.9890329	175.60241	60.37565265	-1.54602451	0.209990704	-7.3623474	1.81E-13	1.24E-12
ENSG00000136235	GPNMB	25.3896125	37.746528	13.03269674	-1.547403448	0.469405166	-3.2965199	0.000979	0.002902
ENSG00000109771	LRP2BP	19.73991896	29.43418	10.04565767	-1.547611717	0.518573749	-2.9843619	0.002842	0.007751
ENSG00000184344	GDF3	280.6026049	418.4878	142.7174085	-1.5552208	0.143443481	-10.842046	2.18E-27	2.51E-26
ENSG00000205336	ADGRG1	4527.62139	6758.7038	2296.53897	-1.556391215	0.041883457	-37.160047	3.02E-302	3.63E-300
ENSG00000168497	CAVIN2	19008.54472	28381.609	9635.480666	-1.558295087	0.027262629	-57.15865	0	0
ENSG00000053747	LAMA3	738.854799	1103.3282	374.3813808	-1.559560047	0.091563778	-17.0325	4.71E-65	1.13E-63
ENSG00000137841	PLCB2	39.75885546	59.413483	20.10422753	-1.559636296	0.357308338	-4.3649591	1.27E-05	4.86E-05
ENSG00000141338	ABCA8	38.42965745	57.572218	19.28709699	-1.56295522	0.413837804	-3.7767338	0.000159	0.000532
ENSG00000128652	HOXD3	45.09359845	67.416875	22.77032153	-1.563067493	0.373510488	-4.1848021	2.85E-05	0.000105
ENSG00000228401	AL590226.1	12.28294607	18.371648	6.194243695	-1.565636285	0.651703478	-2.4023752	0.016289	0.037668
ENSG00000198879	SFMBT2	27.85050788	41.713295	13.98772029	-1.568282937	0.418246222	-3.7496643	0.000177	0.000589
ENSG00000116667	C1orf21	826.7629329	1236.0913	417.4345482	-1.568779988	0.082873751	-18.929757	6.49E-80	1.94E-78
ENSG00000251314	AC104123.1	22.12679372	33.045884	11.20770349	-1.572573975	0.485858443	-3.2366917	0.001209	0.003525
ENSG00000186918	ZNF395	320.667352	479.9459	161.3888024	-1.572879699	0.123648282	-12.720595	4.54E-37	6.58E-36
ENSG00000251257	AC010457.1	11.51143831	17.248372	5.774504897	-1.57592935	0.683868011	-2.304435	0.021198	0.047579
ENSG00000158077	NLRP14	28.49156909	42.755122	14.22801599	-1.576923635	0.435057667	-3.6246313	0.000289	0.000932
ENSG00000154734	ADAMTS1	1042.475596	1562.4472	522.5039845	-1.578214552	0.079205813	-19.925489	2.45E-88	8.29E-87
ENSG00000186907	RTN4RL2	17.19373196	25.75451	8.632954195	-1.580150156	0.548543963	-2.8806263	0.003969	0.010489
ENSG00000092421	SEMA6A	85.18690504	127.76806	42.60574709	-1.582044579	0.23843792	-6.6350377	3.24E-11	1.96E-10
ENSG00000145990	GFOD1	1624.839133	2439.1753	810.5029871	-1.588823023	0.061177728	-25.970612	1.06E-148	5.74E-147
ENSG00000175567	UCP2	275.1134268	413.06832	137.158533	-1.589904153	0.138749529	-11.458808	2.12E-30	2.67E-29
ENSG00000115884	SDC1	619.8392423	932.01178	307.6667069	-1.597939102	0.092752802	-17.227934	1.64E-66	4.02E-65
ENSG00000198929	NOS1AP	69.41121479	104.19599	34.6264371	-1.598954034	0.283678798	-5.6364947	1.74E-08	8.67E-08
ENSG00000225511	LINC00475	16.14604079	24.224904	8.067177335	-1.599223144	0.563668382	-2.8371702	0.004552	0.011889
ENSG00000162595	DIRAS3	44.63188153	67.251343	22.01242043	-1.599513841	0.343409337	-4.6577471	3.20E-06	1.30E-05
ENSG00000186377	CYP4X1	28.89969772	43.482585	14.31681044	-1.603099167	0.480019367	-3.3396552	0.000839	0.002514
ENSG00000197852	INKA2	999.4263776	1504.874	493.9787853	-1.609243101	0.077504426	-20.763241	9.31E-96	3.39E-94
ENSG00000174791	RIN1	460.6165674	694.263	226.9701385	-1.611562948	0.104695896	-15.3928	1.83E-53	3.64E-52
ENSG00000105784	RUNDC3B	30.05449653	45.26981	14.83918299	-1.613448024	0.407333305	-3.961002	7.46E-05	0.000261
ENSG00000176435	CLEC14A	24118.04627	36396.228	11839.86431	-1.620236306	0.023464922	-69.049294	0	0
ENSG00000148057	IDNK	54.62048047	82.542185	26.69877591	-1.622439547	0.302268337	-5.3675471	7.98E-08	3.77E-07
ENSG00000099937	SERPIND1	243.4214098	367.433	119.4098164	-1.623520069	0.15072684	-10.771274	4.70E-27	5.36E-26
ENSG00000099998	GGT5	2275.535988	3438.9358	1112.136155	-1.629123683	0.049619474	-32.832345	2.04E-236	1.90E-234
ENSG00000121236	TRIM6	99.76395791	150.65856	48.86935112	-1.629752291	0.224712432	-7.2526129	4.09E-13	2.74E-12
ENSG00000178695	KCTD12	35196.86028	53205.515	17188.20573	-1.6300631	0.025023285	-65.14185	0	0
ENSG00000270547	LINC01235	348.0046997	525.94014	170.0692642	-1.634457232	0.137203854	-11.912619	1.02E-32	1.34E-31
ENSG00000164651	SP8	82.27345044	124.54825	39.99865148	-1.642098807	0.249107433	-6.5919302	4.34E-11	2.59E-10

ENSG00000162337	LRP5	7116.760352	10781.16	3452.360911	-1.64302571	0.03115182	-52.742526	0	0
ENSG00000143816	WNT9A	343.207973	520.24342	166.1725302	-1.644318801	0.134040568	-12.267322	1.36E-34	1.85E-33
ENSG00000077616	NAALAD2	30.42165164	46.019945	14.82335785	-1.64658297	0.482903816	-3.4097535	0.00065	0.001989
ENSG00000158555	GDPD5	2083.421712	3159.0446	1007.798801	-1.647335222	0.059402833	-27.731594	2.91E-169	1.77E-167
ENSG00000196812	ZSCAN16	193.5108629	293.43608	93.58564568	-1.647462634	0.16384554	-10.054974	8.73E-24	9.05E-23
ENSG00000266903	AC243964.2	23.88997668	36.328114	11.45183923	-1.648676604	0.468425792	-3.5196111	0.000432	0.001355
ENSG00000115290	GRB14	247.9247072	375.80969	120.0397214	-1.650901644	0.150102529	-10.998493	3.89E-28	4.58E-27
ENSG00000184363	PKP3	15.23186714	23.049142	7.414591861	-1.651964892	0.601957248	-2.7443226	0.006064	0.015431
ENSG00000164512	ANKRD55	51.8178659	78.486263	25.14946923	-1.653713557	0.349646772	-4.7296692	2.25E-06	9.31E-06
ENSG00000106714	CNTNAP3	823.6643911	1250.3759	396.9529274	-1.656755521	0.082499204	-20.082079	1.06E-89	3.63E-88
ENSG00000177842	ZNF620	123.8210687	188.25015	59.3919912	-1.662301358	0.209486103	-7.9351391	2.10E-15	1.57E-14
ENSG00000181019	NQO1	3633.583051	5522.2884	1744.877734	-1.662678894	0.043238591	-38.453586	0	0
ENSG00000267751	AC009005.1	21.77732576	33.221454	10.33319771	-1.667060186	0.512523343	-3.2526522	0.001143	0.003348
ENSG00000129173	E2F8	720.8374975	1097.1103	344.5647298	-1.67168211	0.088946661	-18.794209	8.42E-79	2.45E-77
ENSG00000184792	OSBP2	622.6660119	947.60228	297.7297427	-1.672599826	0.097780887	-17.105591	1.35E-65	3.26E-64
ENSG00000128849	CGNL1	5569.026213	8488.0533	2649.999098	-1.679413868	0.034185187	-49.126947	0	0
ENSG00000128833	MYO5C	770.9537301	1175.3475	366.5599624	-1.680553045	0.087800023	-19.14069	1.16E-81	3.59E-80
ENSG00000164647	STEAP1	1226.744408	1871.3067	582.1821559	-1.682803684	0.070629682	-23.825729	1.81E-125	8.09E-124
ENSG00000006534	ALDH3B1	10.25195207	15.640091	4.863812759	-1.684958185	0.701889711	-2.4006025	0.016368	0.037818
ENSG00000133056	PIK3C2B	3413.884257	5210.0985	1617.670045	-1.686104245	0.043472271	-38.785741	0	0
ENSG00000251095	AC097478.1	26.92152337	41.24022	12.60282706	-1.687625682	0.479247314	-3.5214087	0.000429	0.001346
ENSG00000197747	S100A10	5768.028069	8805.5497	2730.506422	-1.688837161	0.035481137	-47.59817	0	0
ENSG00000246889	AP000487.1	11.78982221	17.978061	5.601583705	-1.692645856	0.647736836	-2.6131691	0.008971	0.02201
ENSG00000255224	AC109322.1	11.42601592	17.485281	5.366751196	-1.693258704	0.684208374	-2.4747705	0.013332	0.031508
ENSG00000187244	BCAM	1620.572797	2477.1106	764.0349991	-1.696043671	0.06927765	-24.48183	2.31E-132	1.09E-130
ENSG00000185008	ROBO2	22.49470555	34.382513	10.60689845	-1.696446285	0.482294915	-3.5174459	0.000436	0.001365
ENSG00000183395	PMCH	26.04853022	39.88348	12.21358037	-1.699722579	0.444369142	-3.8250239	0.000131	0.000443
ENSG00000204161	TMEM273	93.76386537	143.50835	44.01937767	-1.70063358	0.238148366	-7.1410676	9.26E-13	6.11E-12
ENSG00000144339	TMEFF2	15.56156855	23.871467	7.251669934	-1.717571555	0.637300801	-2.695072	0.007037	0.017635
ENSG00000211445	GPX3	327.6866464	502.72461	152.6486784	-1.718242666	0.149461202	-11.496245	1.38E-30	1.74E-29
ENSG00000124496	TRERF1	239.9919993	368.33637	111.6476274	-1.718797826	0.153031555	-11.231656	2.85E-29	3.49E-28
ENSG00000128510	CPA4	10.37200141	15.872741	4.871261776	-1.718905696	0.736633161	-2.3334623	0.019624	0.044382
ENSG00000212864	RNF208	95.72877022	147.06183	44.39571216	-1.7247912	0.239320014	-7.2070496	5.72E-13	3.82E-12
ENSG00000143036	SLC44A3	14.12008103	21.711365	6.528797021	-1.727323791	0.605232648	-2.8539832	0.004317	0.011318
ENSG00000156113	KCNMA1	90.72071083	139.42635	42.01507293	-1.733803154	0.248032036	-6.9902388	2.74E-12	1.76E-11
ENSG00000170545	SMAGP	537.9250841	827.46643	248.3837397	-1.734162357	0.099034609	-17.51067	1.19E-68	3.00E-67
ENSG00000178726	THBD	975.5008007	1500.6708	450.3308112	-1.734887777	0.080805267	-21.469984	2.97E-102	1.14E-100
ENSG00000125848	FLRT3	13.20099695	20.355774	6.046219802	-1.735062242	0.630497489	-2.751894	0.005925	0.015108
ENSG00000105737	GRIK5	45.5487092	70.090875	21.00654355	-1.737686348	0.345224426	-5.0334977	4.82E-07	2.12E-06
ENSG00000150636	CCDC102B	16.95300507	26.211327	7.694682887	-1.752395328	0.574846053	-3.0484602	0.0023	0.0064
ENSG00000266865	AC138207.8	18.15074854	27.959693	8.341805176	-1.756600535	0.537096229	-3.2705509	0.001073	0.003158
ENSG00000203943	SAMD13	45.62325321	70.474795	20.77171104	-1.759328144	0.3493618	-5.0358343	4.76E-07	2.10E-06
ENSG00000091409	ITGA6	11184.34016	17294.057	5074.623154	-1.768548449	0.029419802	-60.11422	0	0
ENSG00000224420	ADM5	22.10143892	34.320838	9.8820397	-1.769824683	0.551102278	-3.2114269	0.001321	0.003822
ENSG00000121764	HCRTR1	15.74966057	24.416732	7.082588783	-1.770967745	0.615848208	-2.8756562	0.004032	0.01064
ENSG00000139174	PRICKLE1	1994.785487	3086.948	902.6230172	-1.773182138	0.05814679	-30.494927	3.04E-204	2.32E-202
ENSG00000163053	SLC16A14	24.33749134	37.726082	10.94890079	-1.779733741	0.45484768	-3.9128126	9.12E-05	0.000315
ENSG00000256616	AP002414.2	23.38814071	36.274003	10.50227886	-1.780871568	0.511931791	-3.4787282	0.000504	0.001565
ENSG00000264235	AP005329.1	9.200973628	14.225052	4.176895137	-1.784725963	0.770258935	-2.3170468	0.020501	0.046172
ENSG00000198542	ITGBL1	123.4966411	191.56802	55.42526234	-1.786973519	0.231699602	-7.7124583	1.23E-14	8.90E-14
ENSG00000224063	AC007319.1	9.780352713	15.206353	4.354352409	-1.787777383	0.768045346	-2.3276977	0.019928	0.045008
ENSG00000139737	SLAIN1	78.03459876	121.21097	34.85822505	-1.791042483	0.273062999	-6.5590816	5.41E-11	3.22E-10
ENSG00000087842	PIR	2062.125649	3199.3943	924.8570302	-1.793006782	0.058653587	-30.569431	3.12E-205	2.39E-203
ENSG00000257553	AC034102.4	10.74172108	16.639064	4.844378645	-1.793045453	0.740507865	-2.4213726	0.015462	0.035973
ENSG00000260912	AL158206.1	224.9408912	349.15581	100.7259724	-1.794787746	0.158300139	-11.337878	8.52E-30	1.05E-28
ENSG00000138722	MMRN1	91561.75663	142175.94	40947.56864	-1.795844234	0.0245396	-73.181478	0	0
ENSG00000105974	CAV1	28124.69226	43679.171	12570.2131	-1.796603343	0.025087826	-71.612556	0	0
ENSG00000283378	CNTNAP3C	61.75042255	95.863798	27.63704722	-1.800576805	0.311694541	-5.7767351	7.62E-09	3.91E-08
ENSG00000028137	TNFRSF1B	4178.475619	6492.8774	1864.073848	-1.80115626	0.039390129	-45.726082	0	0
ENSG00000179399	GPC5	25.44801593	39.648533	11.24749882	-1.807364531	0.450430577	-4.0125263	6.01E-05	0.000212
ENSG00000174059	CD34	2452.252874	3816.4198	1088.085923	-1.810115726	0.052603398	-34.410623	1.75E-259	1.79E-257
ENSG00000184785	SMIM10	64.64721777	100.49243	28.80200598	-1.810793891	0.285891418	-6.3338519	2.39E-10	1.36E-09
ENSG00000186868	MAPT	10.19046595	15.878556	4.502376302	-1.812915321	0.757082701	-2.3946067	0.016638	0.038374
ENSG00000238121	LINC00426	16.19951355	25.207283	7.191744449	-1.813414636	0.585267043	-3.0984397	0.001945	0.005482
ENSG00000073910	FRY	1971.540437	3071.6943	871.3865764	-1.816568025	0.053971596	-33.657853	2.39E-248	2.32E-246
ENSG00000149573	MPZL2	1202.704712	1872.8337	532.575762	-1.816751067	0.071930157	-25.257154	9.45E-141	4.77E-139
ENSG00000165092	ALDH1A1	10170.98801	15852.627	4489.349071	-1.820479016	0.028755777	-63.308287	0	0
ENSG00000163072	NOSTRIN	14.80636904	23.146779	6.465958599	-1.828912774	0.587380956	-3.1136739	0.001848	0.005226
ENSG00000165804	ZNF219	385.9943437	602.5781	169.4105896	-1.829766767	0.116638453	-15.687509	1.84E-55	3.77E-54
ENSG00000166924	NYAP1	114.8532238	179.33957	50.36687738	-1.833259538	0.212121884	-8.6424819	5.50E-18	4.58E-17
ENSG00000126878	AIF1L	874.3873297	1365.8099	382.9647128	-1.83458914	0.086351187	-21.245674	3.61E-100	1.37E-98
ENSG00000168077	SCARA3	6656.299297	10399.168	2913.430335	-1.835843462	0.041431899	-44.309904	0	0
ENSG00000072163	LIMS2	1050.036041	1641.0952	458.9769115	-1.839276101	0.074017686	-24.849144	2.64E-136	1.29E-134
ENSG00000115556	PLCD4	65.20305656	101.96299	28.44311977	-1.840990045	0.299654731	-6.1437043	8.06E-10	4.42E-09
ENSG00000130300	PLVAP	875.2485849	1370.4127	380.084478	-1.84534374	0.097073206	-19.009816	1.41E-80	4.28E-79
ENSG00000114378	HYAL1	36.3819951	56.916945	15.84704569	-1.846674708	0.390158908	-4.7331348	2.21E-06	9.17E-06
ENSG00000138449	SLC40A1	1717.516275	2689.3763	745.6562457	-1.849032895	0.05900637	-31.336157	1.50E-215	1.22E-213
ENSG00000178947	SMIM10L2A	14.99280463	23.500217	6.485392713	-1.852640805	0.582698141	-3.1794177	0.001476	0.004232
ENSG00000123892	RAB38	160.3157384	251.40284	69.22864076	-1.852992453	0.204634114	-9.0551493	1.36E-19	1.21E-18
ENSG00000111907	TPD52L1	11.00323742	17.251818	4.754657093	-1.854440282	0.724142334	-2.5608782	0.010441	0.025253
ENSG00000118407	FILIP1	473.5450937	741.8597	205.2304917	-1.855765699	0.106096491	-17.491301	1.67E-68	4.20E-67
ENSG00000260641	AC114811.2	22.36567684	35.015713	9.715640421	-1.856190111	0.487757781	-3.8055572	0.000141	0.000477
ENSG00000069702	TGFBR3	1075.610602	1686.6714	464.549755	-1.858362649	0.07371166	-25.211244	3.02E-140	1.51E-138
ENSG00000177721	ANXA2R	117.6519068	184.81394	50.48987235	-1.865875042	0.21923312	-8.5109177	1.73E-17	1.41E-16
ENSG00000137878	GCOM1	196.6938207	308.39363	84.99401051	-1.867209407	0.191590308	-9.7458448	1.92E-22	1.90E-21

ENSG00000138185	ENTPD1	2836.521854	4456.8641	1216.17962	-1.87389313	0.053658252	-34.922739	3.36E-267	3.55E-265
ENSG00000170271	FAXDC2	284.3744469	447.10703	121.6418673	-1.880456506	0.142475759	-13.198431	8.96E-40	1.39E-38
ENSG00000105929	ATP6V0A4	53.80792347	84.50316	23.11268656	-1.882783745	0.322591058	-5.8364412	5.33E-09	2.77E-08
ENSG00000105851	PIK3CG	427.0694292	672.5517	181.5871559	-1.884473572	0.116024286	-16.242061	2.54E-59	5.59E-58
ENSG00000136305	CIDEB	114.8859001	180.87755	48.89424842	-1.888272677	0.210558469	-8.9679256	3.02E-19	2.63E-18
ENSG00000114166	KAT2B	474.4678933	747.61024	201.3255425	-1.89074541	0.108147252	-17.483065	1.93E-68	4.84E-67
ENSG00000197565	COL4A6	425.5452677	671.43157	179.6589645	-1.901243292	0.115311465	-16.487895	4.48E-61	1.01E-59
ENSG00000180730	SHISA2	220.5539691	348.33234	92.77560146	-1.902261141	0.162564963	-11.701544	1.25E-31	1.62E-30
ENSG00000185585	OLFML2A	44.95141191	70.986467	18.91635731	-1.923116313	0.362151299	-5.3102566	1.09E-07	5.11E-07
ENSG00000215183	MSMP	295.7534591	468.34206	123.1648552	-1.923927714	0.144846656	-13.282514	2.92E-40	4.60E-39
ENSG00000112414	ADGRG6	11248.548	17823.082	4674.014277	-1.931024439	0.028701567	-67.279408	0	0
ENSG00000236609	ZNF853	25.14281775	39.749952	10.5356839	-1.93116892	0.469713325	-4.1113778	3.93E-05	0.000142
ENSG00000064042	LIMCH1	1908.169472	3024.3783	791.9606451	-1.932160808	0.057091716	-33.843103	4.58E-251	4.51E-249
ENSG00000113721	PDGFRB	37.8219716	59.98379	15.66015357	-1.932627518	0.378246248	-5.1094427	3.23E-07	1.45E-06
ENSG00000171346	KRT15	24.07890718	38.155561	10.00225336	-1.934506243	0.462687275	-4.1810232	2.90E-05	0.000107
ENSG00000250033	SLC7A11-AS1	25.76286388	40.820036	10.70569216	-1.93551712	0.478341209	-4.0463106	5.20E-05	0.000186
ENSG00000164035	EMCN	2420.028449	3836.898	1003.158854	-1.935869372	0.05276624	-36.68765	1.15E-294	1.32E-292
ENSG00000198719	DLL1	276.7946552	438.97663	114.6126821	-1.938591905	0.140480813	-13.799692	2.56E-43	4.27E-42
ENSG00000116574	RHOU	79.91041323	126.69135	33.12947526	-1.938948535	0.266890814	-7.2649504	3.73E-13	2.51E-12
ENSG00000167874	TMEM88	370.962226	589.80758	152.116871	-1.955275631	0.119349101	-16.382827	2.54E-60	5.63E-59
ENSG00000165140	FBP1	10.94043624	17.42935	4.451522978	-1.962018339	0.700357747	-2.8014516	0.005087	0.013158
ENSG00000164849	GPR146	321.3983386	511.63586	131.160818	-1.962472125	0.134658298	-14.573719	4.13E-48	7.55E-47
ENSG00000137875	BCL2L10	24.79721305	39.61068	9.983746349	-1.964782696	0.49053459	-4.0053907	6.19E-05	0.000219
ENSG00000185339	TCN2	2976.886515	4741.252	1212.521052	-1.967432644	0.046447931	-42.35781	0	0
ENSG00000126882	FAM78A	3250.859651	5179.8615	1321.857772	-1.968775891	0.053018388	-37.133832	8.00E-302	9.49E-300
ENSG00000110900	TSPAN11	349.2277922	556.93886	141.5167255	-1.974124423	0.13298524	-14.844688	7.53E-50	1.41E-48
ENSG00000134242	PTPN22	103.8307828	165.41073	42.25083255	-1.975253421	0.228379482	-8.6489969	5.20E-18	4.33E-17
ENSG00000168405	CMAHP	56.94541538	90.866939	23.02389211	-1.979829123	0.302388936	-6.5472935	5.86E-11	3.48E-10
ENSG00000132170	PPARG	26.60544844	42.427468	10.78342861	-1.983090356	0.486643379	-4.0750382	4.60E-05	0.000165
ENSG00000187243	MAGED4B	14.38721354	23.026805	5.747621767	-1.984075961	0.616403589	-3.2187937	0.001287	0.003736
ENSG00000117152	RGS4	1731.418879	2763.4681	699.3696194	-1.984912131	0.061127303	-32.471777	2.67E-231	2.35E-229
ENSG00000176438	SYNE3	912.0251918	1456.8338	367.2165896	-1.985808572	0.079995909	-24.823877	4.95E-136	2.40E-134
ENSG00000152128	TMEM163	114.6111961	183.18659	46.03579912	-1.99022172	0.224745065	-8.8554635	8.33E-19	7.14E-18
ENSG00000138646	HERC5	109.5782761	175.31102	43.84552937	-1.999355075	0.233791951	-8.5518559	1.21E-17	9.93E-17
ENSG00000143248	RGS5	20230.01557	32372.78	8087.250914	-2.000435253	0.033318868	-60.039112	0	0
ENSG00000272734	ADIRF-AS1	14.65523886	23.480583	5.829894302	-2.000568081	0.612204434	-3.2678105	0.001084	0.003186
ENSG00000203883	SOX18	3821.939901	6123.5875	1520.292297	-2.010099167	0.199696575	-10.065767	7.83E-24	8.12E-23
ENSG00000260400	AL513534.2	13.13034078	21.004013	5.256668426	-2.01542966	0.671513463	-3.0013243	0.002688	0.00737
ENSG00000184260	HIST2H2AC	11.12679466	17.923207	4.330382215	-2.031266847	0.77524201	-2.6201713	0.008789	0.021606
ENSG00000100336	APOL4	55.25351617	88.984638	21.52239419	-2.03160792	0.332309773	-6.1135967	9.74E-10	5.32E-09
ENSG00000256235	SMIM3	74.28826761	119.51387	29.06266289	-2.036024085	0.267864478	-7.6009484	2.94E-14	2.08E-13
ENSG00000159915	ZNF233	16.82459127	26.999245	6.649937784	-2.037404915	0.595609859	-3.4207038	0.000625	0.001916
ENSG00000185862	EVI2B	71.7252889	115.46769	27.9828896	-2.037574026	0.272882826	-7.466846	8.21E-14	5.72E-13
ENSG00000266469	AC005288.1	6.958619519	11.25929	2.657948936	-2.0527987	0.882374294	-2.3264489	0.019995	0.045153
ENSG00000004776	HSPB6	177.1979561	285.93017	68.46574145	-2.060915114	0.188692868	-10.922062	9.04E-28	1.05E-26
ENSG00000001561	ENPP4	169.3314805	273.31702	65.3459392	-2.062767376	0.181738443	-11.350198	7.40E-30	9.15E-29
ENSG00000129538	RNASE1	2979.468349	4809.7324	1149.204312	-2.063708686	0.055410637	-37.243908	1.33E-303	1.62E-301
ENSG00000169418	NPR1	1531.512541	2472.3465	590.6786091	-2.065755599	0.064050852	-32.251805	3.32E-228	2.85E-226
ENSG00000237928	NFIA-AS2	12.40503911	20.098825	4.711252785	-2.071220534	0.672296318	-3.0808149	0.002064	0.005791
ENSG00000263155	MYZAP	146.7097731	237.20496	56.21458265	-2.078240972	0.191919439	-10.828715	2.52E-27	2.89E-26
ENSG00000127241	MASP1	27.32292699	44.129604	10.51624979	-2.08267959	0.447257267	-4.6565584	3.22E-06	1.31E-05
ENSG00000186479	RGS7BP	425.2101834	687.83088	162.5894854	-2.08438893	0.118200238	-17.634389	1.34E-69	3.43E-68
ENSG00000105516	DBP	52.20970121	84.708031	19.71137187	-2.092512041	0.333724226	-6.2701832	3.61E-10	2.03E-09
ENSG00000157214	STEAP2	248.6806479	403.7433	93.61799199	-2.102038191	0.153689927	-13.677137	1.39E-42	2.30E-41
ENSG00000197635	DPP4	527.3721322	855.43501	199.3092527	-2.102352568	0.104467218	-20.12452	4.50E-90	1.55E-88
ENSG00000176244	ACBD7	34.95237246	56.643738	13.26100734	-2.105774518	0.403412468	-5.2199044	1.79E-07	8.21E-07
ENSG00000120875	DUSP4	2741.39388	4450.8846	1031.90316	-2.106340767	0.052645091	-40.010202	0	0
ENSG00000141293	SKAP1	7.432621664	12.023315	2.84192812	-2.108304469	0.856532624	-2.4614409	0.013838	0.032582
ENSG00000196611	MMP1	19228.43783	31235.83	7221.045946	-2.112427612	0.027616662	-76.491054	0	0
ENSG00000114654	EFCC1	18.83110855	30.475936	7.186281264	-2.113659955	0.632058068	-3.3440914	0.000826	0.002479
ENSG00000137727	ARHGAP20	290.3185431	472.18223	108.4548558	-2.116136145	0.157919889	-13.400061	6.04E-41	9.69E-40
ENSG00000160801	PTH1R	9.193008471	14.990297	3.395719882	-2.126144246	0.785078711	-2.7081925	0.006765	0.01703
ENSG00000171130	ATP6V0E2	590.9878227	962.75267	219.2229792	-2.133571153	0.096261846	-22.164245	7.60E-109	3.03E-107
ENSG00000141854	MISP3	9.427215433	15.325585	3.528845742	-2.138539376	0.756439811	-2.8271111	0.004697	0.012226
ENSG00000159640	ACE	4513.493062	7359.1357	1667.850425	-2.141155918	0.041822178	-51.196662	0	0
ENSG00000177685	CRACR2B	319.1525005	520.71379	117.5912134	-2.146682583	0.131764603	-16.2918	1.13E-59	2.49E-58
ENSG00000143375	CGN	11.92609723	19.529261	4.322933198	-2.148936846	0.712041331	-3.0179945	0.002545	0.007011
ENSG00000111644	ACRBP	38.39091213	62.696933	14.08489086	-2.149470383	0.389053191	-5.5248753	3.30E-08	1.61E-07
ENSG00000061918	GUCY1B1	465.7631817	760.99108	170.535288	-2.154219121	0.116275701	-18.526821	1.25E-76	3.54E-75
ENSG00000138685	FGF2	1346.215795	2202.4252	490.0064002	-2.16474243	0.072245681	-29.963624	2.92E-197	2.15E-195
ENSG00000169126	ARMC4	132.8338663	217.245	48.42272919	-2.170049988	0.202779451	-10.701528	1.00E-26	1.13E-25
ENSG00000084734	GCKR	38.41442309	62.749419	14.07942768	-2.170598484	0.424982316	-5.1075031	3.26E-07	1.46E-06
ENSG00000180881	CAPS2	19.16213135	31.278556	7.045706388	-2.172712123	0.568404282	-3.8224767	0.000132	0.000447
ENSG00000223749	MIR503HG	113.7717428	186.42818	41.11530716	-2.175293335	0.251994532	-8.6323037	6.01E-18	4.99E-17
ENSG00000079102	RUNX1T1	1220.447738	1998.9426	441.952922	-2.177171619	0.077089161	-28.242253	1.77E-175	1.14E-173
ENSG00000235831	BHLHE40-AS1	21.62437768	35.542087	7.706667984	-2.183405823	0.513434077	-4.2525534	2.11E-05	7.90E-05
ENSG00000023171	GRAMD1B	289.1091833	474.52783	103.6905328	-2.189557894	0.145036944	-15.096553	1.71E-51	3.29E-50
ENSG00000120833	SOCS2	106.1756173	174.05736	38.29387189	-2.193045888	0.232183319	-9.4453206	3.54E-21	3.34E-20
ENSG00000136160	EDNRB	142.2339571	233.70143	50.76648602	-2.201557084	0.212196538	-10.375085	3.22E-25	3.49E-24
ENSG00000172247	C1QTNF4	8.904639969	14.559598	3.249681821	-2.203810825	0.867627683	-2.5400421	0.011084	0.026629
ENSG00000249631	AC005699.1	20.20138059	33.187047	7.215714643	-2.205029799	0.544701419	-4.048144	5.16E-05	0.000184
ENSG00000116678	LEPR	2270.280653	3734.6115	805.9497575	-2.211841482	0.052313003	-42.280912	0	0
ENSG00000277304	AC142086.6	9.581822508	15.794808	3.368836752	-2.21528606	0.798026018	-2.7759572	0.005504	0.014116
ENSG00000132563	REEP2	107.0909226	176.17657	38.00527312	-2.215600709	0.232072422	-9.5470228	1.33E-21	1.28E-20
ENSG00000137463	MGARP	301.136552	497.03929	105.2338119	-2.238840988	0.141778149	-15.791157	3.58E-56	7.41E-55

ENSG00000120756	PLS1	41.70803179	68.742353	14.67371081	-2.239194682	0.374235917	-5.9833773	2.19E-09	1.16E-08
ENSG00000151572	ANO4	86.98248272	143.70592	30.25904087	-2.248030013	0.253323374	-8.8741516	7.05E-19	6.06E-18
ENSG00000166292	TMEM100	6.80847464	11.277032	2.339916787	-2.250649676	0.906494167	-2.4828066	0.013035	0.03088
ENSG00000178977	LINC00324	6.022280481	9.9404038	2.104157174	-2.253890789	0.960647172	-2.3462212	0.018965	0.04305
ENSG00000102452	NALCN	422.3486005	699.05884	145.6383628	-2.267624461	0.122639261	-18.4902	2.48E-76	6.94E-75
ENSG00000112246	SIM1	14.95159274	24.811062	5.092123355	-2.274607238	0.629126089	-3.615503	0.0003	0.000963
ENSG00000169744	LDB2	3645.281006	6044.9918	1245.570209	-2.277560752	0.047605667	-47.84222	0	0
ENSG00000152217	SETBP1	580.3565834	962.02531	198.6878554	-2.279284328	0.105225813	-21.660886	4.80E-104	1.88E-102
ENSG00000128346	C22orf23	8.180284328	13.581479	2.779089699	-2.2826141	0.953735921	-2.3933398	0.016696	0.038488
ENSG00000283696	AL592295.4	9.27412369	15.344883	3.203364577	-2.284670829	0.815519052	-2.8014929	0.005087	0.013158
ENSG00000214357	NEURL1B	12.14655614	20.179056	4.114056715	-2.289837065	0.670721493	-3.4139909	0.00064	0.00196
ENSG00000147697	GSDMC	123.2197277	204.83773	41.60172443	-2.294763048	0.22392665	-10.247834	1.21E-24	1.29E-23
ENSG00000171476	HOPX	150.3663949	250.06801	50.66477937	-2.298248797	0.199924283	-11.495596	1.39E-30	1.75E-29
ENSG00000128594	LRRC4	131.2071935	217.94117	44.47321755	-2.298562893	0.217269243	-10.579329	3.72E-26	4.15E-25
ENSG00000166823	MESP1	15.36718345	25.583941	5.150425697	-2.306153206	0.609103572	-3.786143	0.000153	0.000514
ENSG00000146678	IGFBP1	89.27383091	148.31445	30.23321646	-2.310485596	0.274193852	-8.4264675	3.56E-17	2.87E-16
ENSG00000101188	NTSR1	417.680289	695.28238	140.0781976	-2.313249511	0.118529986	-19.516154	8.00E-85	2.59E-83
ENSG00000150782	IL18	31.71416143	52.696675	10.73164818	-2.322173457	0.473918672	-4.8999408	9.59E-07	4.11E-06
ENSG00000074370	ATP2A3	38.32319083	63.891922	12.75445993	-2.327224797	0.380033172	-6.1237412	9.14E-10	5.00E-09
ENSG00000233370	AC140479.3	6.226824824	10.412331	2.041318752	-2.335962453	0.9643466	-2.4223266	0.015421	0.035892
ENSG00000125878	TCF15	41.30822991	68.962597	13.65386301	-2.350421608	0.407725388	-5.7647173	8.18E-09	4.19E-08
ENSG00000229109	AL137847.1	6.21162134	10.432777	1.990465428	-2.353724794	0.972011126	-2.4214998	0.015457	0.035965
ENSG00000092758	COL9A3	27.44190614	45.874524	9.009288685	-2.354526745	0.452198463	-5.2068438	1.92E-07	8.78E-07
ENSG00000166960	CCDC178	15.17259108	25.387112	4.958070391	-2.379148243	0.649808185	-3.6613085	0.000251	0.000817
ENSG00000137880	GCHFR	50.73543533	85.073873	16.39699741	-2.385941914	0.338304323	-7.0526498	1.76E-12	1.14E-11
ENSG00000187017	ESPN	11.96912715	20.098825	3.839428874	-2.394945221	0.709652826	-3.3748125	0.000739	0.002234
ENSG00000205221	VIT	18.46430608	31.18099	5.747621767	-2.422165651	0.560178101	-4.3239206	1.53E-05	5.81E-05
ENSG00000110675	ELMOD1	158.1351534	266.4948	49.77550718	-2.424228337	0.198220478	-12.229959	2.15E-34	2.92E-33
ENSG00000101331	CCM2L	326.8372702	551.55182	102.1227191	-2.429175824	0.136676038	-17.773239	1.14E-70	2.95E-69
ENSG00000197558	SSPO	14.2123464	24.004589	4.420103767	-2.43487632	0.675942561	-3.6021941	0.000316	0.001011
ENSG00000065609	SNAP91	15.83194302	26.640064	5.023821749	-2.440576828	0.649895873	-3.7553352	0.000173	0.000576
ENSG00000133110	POSTN	1141.191953	1930.7198	351.6641002	-2.456475546	0.078304212	-31.370925	5.04E-216	4.10E-214
ENSG00000255690	TRIL	79.42163385	134.39724	24.44603043	-2.462389722	0.570619574	-4.3152914	1.59E-05	6.03E-05
ENSG00000244471	AL137058.1	5.726086604	9.6660482	1.786125025	-2.464117919	1.028402648	-2.3960634	0.016572	0.038245
ENSG00000002587	HS3ST1	62.47295329	105.76075	19.18515927	-2.472612943	0.326508075	-7.5728998	3.65E-14	2.58E-13
ENSG00000255389	Z97989.1	10.23855869	17.36801	3.109106944	-2.482778495	0.747267131	-3.3224779	0.000892	0.00266
ENSG00000115194	SLC30A3	73.96181056	125.44898	22.47463643	-2.4838015	0.28149788	-8.8235176	1.11E-18	9.45E-18
ENSG00000185527	AC139530.1	13.12155072	22.375862	3.867239109	-2.48620808	0.794847981	-3.1279039	0.001761	0.004992
ENSG00000197632	SERPINB2	209.4546878	355.48944	63.41993534	-2.487444438	0.168278352	-14.781726	1.92E-49	3.56E-48
ENSG00000273669	AC015819.1	10.36517358	17.660108	3.070238717	-2.519660695	0.775007016	-3.2511457	0.001149	0.003364
ENSG00000133083	DCLK1	52.95378024	90.314781	15.59277907	-2.522083146	0.352052327	-7.163944	7.84E-13	5.19E-12
ENSG00000196421	C20orf204	95.6044577	162.73693	28.47198874	-2.525035633	0.274843971	-9.1871604	4.03E-20	3.65E-19
ENSG00000021300	PLEKHB1	22.57561262	38.450434	6.700791108	-2.534737683	0.547474898	-4.6298701	3.66E-06	1.48E-05
ENSG00000135324	MRAP2	292.8399281	499.73711	85.94274322	-2.535803793	0.144187785	-17.586814	3.11E-69	7.87E-68
ENSG00000117472	TSPAN1	9.228169833	15.778957	2.67738305	-2.538572068	0.822314821	-3.0871048	0.002021	0.005679
ENSG00000107742	SPOCK2	46.62884084	79.674802	13.58287953	-2.538629906	0.372677462	-6.8118686	9.63E-12	6.01E-11
ENSG00000271380	AL451085.2	5.877952813	10.020634	1.7352717	-2.541941717	1.00979197	-2.5172925	0.011826	0.028252
ENSG00000144681	STAC	19.99800016	34.228944	5.76705588	-2.555827964	0.54761696	-4.6671819	3.05E-06	1.25E-05
ENSG00000204103	MAFB	164.8131234	281.88206	47.74418769	-2.560513584	0.189440146	-13.516214	1.25E-41	2.04E-40
ENSG00000184611	KCNH7	5.962177634	10.220503	1.70385249	-2.576161818	1.098753874	-2.3446214	0.019046	0.043203
ENSG00000160180	TFF3	16.94716015	28.96022	4.934100197	-2.578923067	0.657990967	-3.9193898	8.88E-05	0.000307
ENSG00000102981	PARD6A	37.85812468	64.697988	11.01826112	-2.580905991	0.42593507	-6.0593883	1.37E-09	7.39E-09
ENSG00000123689	G0S2	294.6150148	505.09377	84.13625697	-2.583457596	0.144812673	-17.839997	3.46E-71	9.03E-70
ENSG00000204060	FOXO6	14.44004336	24.792913	4.087173585	-2.592552124	0.663219149	-3.9090429	9.27E-05	0.00032
ENSG00000188649	CC2D2B	12.03114067	20.70543	3.356851655	-2.600709177	0.743897774	-3.4960572	0.000472	0.001473
ENSG00000276772	AC025271.4	4.857787636	8.3297549	1.385820341	-2.602578046	1.130258176	-2.3026403	0.021299	0.04777
ENSG00000143850	PLEKHA6	333.4419485	573.3106	93.57329789	-2.609094024	0.146016149	-17.868531	2.07E-71	5.43E-70
ENSG00000178175	ZNF366	208.3785822	358.19104	58.56612184	-2.609744206	0.19067058	-13.687189	1.21E-42	2.00E-41
ENSG00000124749	COL21A1	44.31816581	76.086212	12.55011952	-2.612415339	0.384805951	-6.7889162	1.13E-11	7.00E-11
ENSG00000275896	PRSS2	12.61422131	21.609875	3.618567294	-2.619254362	0.748638315	-3.4986913	0.000468	0.001459
ENSG00000106484	MEST	806.437401	1387.1333	225.7415458	-2.621869182	0.089353484	-29.342663	2.96E-189	2.06E-187
ENSG00000251191	LINC00589	7.421099725	12.788896	2.053303849	-2.63308419	0.904542107	-2.9109581	0.003603	0.009611
ENSG00000138030	KHK	87.7976048	151.1116	24.48360886	-2.639085215	0.271964847	-9.7037733	2.91E-22	2.85E-21
ENSG00000267690	LDLRAD4-AS1	17.12613472	29.686128	4.566141828	-2.657109286	0.641816586	-4.1399823	3.47E-05	0.000127
ENSG00000139549	DHH	110.4824557	190.86426	30.10065502	-2.659146532	0.260218425	-10.218902	1.63E-24	1.73E-23
ENSG00000162409	PRKAA2	96.47738081	166.84481	26.10995596	-2.662403145	0.277409517	-9.5973749	8.20E-22	7.92E-21
ENSG00000265778	AC018413.1	7.635401374	13.178631	2.092172077	-2.670780514	0.950007034	-2.8113271	0.004934	0.012789
ENSG00000103485	QPRT	5.075027849	8.7642354	1.385820341	-2.671698132	1.109911007	-2.4071282	0.016079	0.037238
ENSG00000091622	PITPNM3	106.6629253	184.35384	28.97201424	-2.672640955	0.242139915	-11.037589	2.52E-28	2.98E-27
ENSG00000197301	HMGA2-AS1	7.746611471	13.357647	2.135576384	-2.678288759	0.953295625	-2.8095049	0.004962	0.012856
ENSG00000058404	CAMK2B	21.97772882	38.118114	5.837343319	-2.706390814	0.530472223	-5.1018521	3.36E-07	1.50E-06
ENSG00000144063	MALL	551.6038742	958.35616	144.8515928	-2.725431146	0.110115565	-24.750644	3.05E-135	1.48E-133
ENSG00000167779	IGFBP6	185.4852369	322.03537	48.93510248	-2.725566219	0.190812358	-14.284013	2.75E-46	4.86E-45
ENSG00000253177	AC104211.2	30.26948272	52.945989	7.592976238	-2.767875326	0.494150551	-5.6012795	2.13E-08	1.06E-07
ENSG00000123977	DAW1	71.73954897	124.99446	18.48463341	-2.768136384	0.307332004	-9.0069903	2.12E-19	1.86E-18
ENSG00000161055	SCGB3A1	9.399031223	16.387858	2.410204226	-2.769395573	0.829097399	-3.3402536	0.000837	0.00251
ENSG00000141655	TNFRSF11A	706.5087065	1232.1792	180.8382569	-2.770962893	0.106659732	-25.979466	8.45E-149	4.57E-147
ENSG00000011347	SYT7	15.0696645	26.210179	3.929150426	-2.77331825	0.665097352	-4.169793	3.05E-05	0.000112
ENSG00000283537	AC073264.3	27.15673196	47.345494	6.967969932	-2.77470142	0.492679398	-5.6318601	1.78E-08	8.89E-08
ENSG00000159904	ZNF890P	8.103700158	14.166082	2.041318752	-2.776764853	0.913303324	-3.0403534	0.002363	0.006561
ENSG00000268297	CLEC4GP1	30.55954794	53.4947	7.624395448	-2.779997324	0.487504286	-5.7025085	1.18E-08	5.97E-08
ENSG00000108001	EBF3	8.246025994	14.39988	2.092172077	-2.800670196	0.887430733	-3.155931	0.0016	0.004565
ENSG00000164867	NOS3	4203.722015	7361.1935	1046.250492	-2.812948876	0.047352725	-59.40416	0	0
ENSG00000128918	ALDH1A2	967.9007096	1695.5907	240.2106823	-2.816955288	0.087938765	-32.033146	3.77E-225	3.19E-223
ENSG00000179046	TRIML2	12.41331594	21.862636	2.963995988	-2.84852149	0.784404915	-3.6314427	0.000282	0.00091

ENSG00000166387	PPFIBP2	91.87127461	161.38452	22.35803174	-2.8528417	0.282079539	-10.113607	4.81E-24	5.02E-23
ENSG00000265107	GJA5	1440.856802	2533.0583	348.6553107	-2.864300832	0.07817716	-36.638589	6.95E-294	7.94E-292
ENSG00000274719	AC012653.2	5.658706097	9.9824452	1.334967016	-2.870740441	1.078850377	-2.6609255	0.007793	0.019365
ENSG00000140465	CYP1A1	1692.022366	2976.8908	407.1539264	-2.8712336	0.065936529	-43.545416	0	0
ENSG00000271447	MMP28	63.50565019	111.7255	15.28580492	-2.875785837	0.33278018	-8.6416981	5.54E-18	4.61E-17
ENSG00000139187	KLRG1	290.592833	511.85625	69.3294203	-2.876920022	0.152089999	-18.915905	8.44E-80	2.51E-78
ENSG00000125965	GDF5	10.11585995	17.860384	2.371335998	-2.892939858	0.807210314	-3.5838738	0.000339	0.001078
ENSG00000147642	SYBU	116.4732708	205.53234	27.41419981	-2.902567981	0.238991009	-12.145093	6.09E-34	8.19E-33
ENSG00000254827	SLC22A18AS	39.13648726	69.101127	9.171847923	-2.903618002	0.456102745	-6.3661489	1.94E-10	1.11E-09
ENSG00000137960	GIPC2	941.2173907	1660.6253	221.8094825	-2.906577417	0.086766251	-33.49894	4.99E-246	4.76E-244
ENSG00000149212	SESN3	596.8280695	1053.9323	139.7238475	-2.914735851	0.105958362	-27.508314	1.40E-166	8.39E-165
ENSG00000189129	PLAC9	41.48775074	73.182125	9.793376876	-2.917851433	0.420981761	-6.9310638	4.18E-12	2.66E-11
ENSG00000155792	DEPTOR	5.917966686	10.450113	1.385820341	-2.929609882	1.109915676	-2.6394887	0.008303	0.020506
ENSG00000259070	LINC00639	131.8996253	233.26105	30.53820479	-2.93090688	0.229068452	-12.794895	1.75E-37	2.57E-36
ENSG00000178715	AL450998.1	6.067059391	10.70943	1.424688569	-2.937720381	1.069061992	-2.747942	0.005997	0.015272
ENSG00000166268	MYRFL	4.52916096	8.0728063	0.985515657	-2.988615235	1.264138995	-2.3641508	0.018071	0.041207
ENSG00000107821	KAZALD1	31.15260915	55.208659	7.096559712	-2.989805544	0.489664448	-6.1058252	1.02E-09	5.58E-09
ENSG00000169306	IL1RAPL1	44.83541449	79.78518	9.885648676	-3.00248008	0.396303905	-7.5762061	3.56E-14	2.51E-13
ENSG00000181350	LRRC75A	195.8966273	348.62173	43.17152395	-3.009563394	0.185698763	-16.206696	4.52E-59	9.85E-58
ENSG00000185924	RTN4RL1	4.624526362	8.2321179	1.016934868	-3.013711015	1.268778996	-2.3752844	0.017535	0.040147
ENSG00000255282	WTAPP1	6.362164168	11.319074	1.405254455	-3.033134528	1.051732055	-2.8839423	0.003927	0.010388
ENSG00000041353	RAB27B	29.87198976	53.379728	6.36425195	-3.041039873	0.49486898	-6.1451414	7.99E-10	4.38E-09
ENSG00000229108	LINC02587	17.66536245	31.495832	3.834892794	-3.051377211	0.631246616	-4.8338908	1.34E-06	5.67E-06
ENSG00000120885	CLU	11924.20227	21282.366	2566.038113	-3.052783932	0.033215943	-91.907188	0	0
ENSG00000169432	SCN9A	108.4554318	193.78434	23.12652586	-3.053996957	0.272370747	-11.212647	3.53E-29	4.30E-28
ENSG00000158458	NRG2	9.48281511	16.975165	1.990465428	-3.060934474	0.857964281	-3.5676712	0.00036	0.001143
ENSG00000173947	PIFO	15.71185542	28.161117	3.262594023	-3.063608372	0.731892879	-4.1858699	2.84E-05	0.000105
ENSG00000258789	AL162171.2	9.749963277	17.407754	2.092172077	-3.066887484	0.838240296	-3.6587211	0.000253	0.000824
ENSG00000274736	CCL23	16.29010043	29.097672	3.482528498	-3.072182178	0.68416541	-4.4904085	7.11E-06	2.79E-05
ENSG00000169282	KCNAB1	66.6940988	119.38197	14.0062273	-3.105542472	0.332798891	-9.331589	1.04E-20	9.64E-20
ENSG00000174175	SELP	272.5456693	488.38289	56.70844893	-3.105668092	0.169929791	-18.276184	1.28E-74	3.46E-73
ENSG00000185739	SRL	18.30794758	32.870721	3.745171242	-3.12025762	0.616702518	-5.0595831	4.20E-07	1.86E-06
ENSG00000115252	PDE1A	35.62512213	64.061413	7.188831512	-3.149878381	0.491860645	-6.4040057	1.51E-10	8.75E-10
ENSG00000154118	JPH3	23.96658552	43.049924	4.883246873	-3.153628224	0.542067868	-5.8177738	5.96E-09	3.09E-08
ENSG00000255045	AP000866.5	5.041203135	9.0968906	0.985515657	-3.154928012	1.187701326	-2.6563311	0.0079	0.019604
ENSG00000139874	SSTR1	162.520969	292.12227	32.91967167	-3.165632812	0.220417	-14.362018	8.96E-47	1.60E-45
ENSG00000237371	AL355803.1	13.71381762	24.718833	2.70880226	-3.168701416	0.727521603	-4.355474	1.33E-05	5.06E-05
ENSG00000165071	TMEM71	78.57173414	141.72268	15.42078498	-3.174728062	0.325728061	-9.7465599	1.91E-22	1.89E-21
ENSG00000169129	AFAP1L2	27.48658012	49.586975	5.38618531	-3.182162962	0.516104482	-6.165734	7.02E-10	3.87E-09
ENSG00000211764	TRBJ2-1	15.45849847	27.897612	3.019385392	-3.184347361	0.695199576	-4.5804794	4.64E-06	1.85E-05
ENSG00000172794	RAB37	5.415230603	9.7432389	1.087222306	-3.208786593	1.197456845	-2.6796678	0.00737	0.018403
ENSG00000205038	PKHD1L1	315.8567625	570.20049	61.51303224	-3.216992329	0.156604406	-20.542157	9.05E-94	3.24E-92
ENSG00000120949	TNFRSF8	17.34583261	31.417086	3.27457912	-3.21869582	0.677314984	-4.7521403	2.01E-06	8.38E-06
ENSG00000127399	LRRC61	12.74501189	23.0484	2.441623436	-3.247539348	0.760046306	-4.2728178	1.93E-05	7.25E-05
ENSG00000185532	PRKG1	87.52382297	158.4029	16.64474213	-3.259193193	0.293352793	-11.110149	1.12E-28	1.34E-27
ENSG00000144035	NAT8	3.748074584	6.7778123	0.718336833	-3.278256185	1.398453615	-2.3442009	0.019068	0.043241
ENSG00000165272	AQP3	20.59723522	37.277305	3.917165329	-3.281069009	0.630374656	-5.2049507	1.94E-07	8.87E-07
ENSG00000140009	ESR2	5.607786218	10.179203	1.036368981	-3.307900095	1.183959208	-2.7939308	0.005207	0.013441
ENSG00000185818	NAT8L	5.770019651	10.452817	1.087222306	-3.319700781	1.139667823	-2.912867	0.003581	0.009558
ENSG00000189431	RASSF10	3.77133137	6.8557451	0.686917622	-3.324172968	1.412996445	-2.3525699	0.018644	0.042384
ENSG00000130768	SMPDL3B	17.06009985	31.011093	3.109106944	-3.324969942	0.727772188	-4.5686961	4.91E-06	1.95E-05
ENSG00000124440	HIF3A	136.9672185	249.1762	24.75823671	-3.342958693	0.240089591	-13.923797	4.54E-44	7.68E-43
ENSG00000163016	ALMS1P1	3.851528868	7.0355742	0.667483508	-3.354252981	1.435657735	-2.3363876	0.019471	0.044084
ENSG00000185432	METTL7A	690.8365551	1259.4909	122.1822525	-3.360301191	0.1079505	-31.128167	1.00E-212	7.94E-211
ENSG00000258881	AC007040.2	4.042098902	7.365861	0.718336833	-3.408783977	1.441463854	-2.3648071	0.018039	0.041142
ENSG00000127324	TSPAN8	32.47919494	59.489932	5.468457845	-3.437134683	0.487741979	-7.0470348	1.83E-12	1.19E-11
ENSG00000274213	AC015912.3	10.30008405	18.978588	1.621579954	-3.495087761	0.911134721	-3.8359725	0.000125	0.000424
ENSG00000188107	EYS	6.408880017	11.769406	1.048354078	-3.504249012	1.137417437	-3.0808821	0.002064	0.005791
ENSG00000159167	STC1	264.8962025	488.15857	41.63383968	-3.549586412	0.192083465	-18.479396	3.03E-76	8.47E-75
ENSG00000145708	CRHBP	4.524891743	8.3120125	0.737770947	-3.566053362	1.361484382	-2.6192393	0.008813	0.021659
ENSG00000134873	CLDN10	35.57615349	65.677327	5.474979758	-3.600091149	1.14347297	-3.1483833	0.001642	0.00468
ENSG00000181444	ZNF467	352.6646256	651.55467	53.77458235	-3.600859084	0.159055239	-22.639047	1.79E-113	7.31E-112
ENSG00000154188	ANGPT1	126.1559201	233.02951	19.28232984	-3.61153199	0.271568138	-13.298806	2.35E-40	3.72E-39
ENSG00000168542	COL3A1	159.3230462	294.84463	23.80145839	-3.618627771	0.233973555	-15.465969	5.89E-54	1.18E-52
ENSG00000213561	AC103591.2	48.05986165	88.939964	7.179759352	-3.62276799	0.427487197	-8.4745649	2.36E-17	1.91E-16
ENSG00000076554	TPD52	263.5378979	488.64694	38.42885196	-3.66434574	0.181213884	-20.221109	6.38E-91	2.21E-89
ENSG00000124429	POF1B	4.760583774	8.8028307	0.718336833	-3.665033643	1.331872677	-2.7517898	0.005927	0.015111
ENSG00000163995	ABLIM2	4.629939563	8.6238148	0.636064297	-3.67617575	1.368637549	-2.6860112	0.007231	0.018074
ENSG00000158825	CDA	35.78698289	66.348717	5.225249215	-3.681574566	0.495394528	-7.4316012	1.07E-13	7.44E-13
ENSG00000154027	AK5	495.0211565	918.74229	71.30001882	-3.692109779	0.305319128	-12.092625	1.16E-33	1.55E-32
ENSG00000133800	LYVE1	1870.71702	3475.0268	266.4072159	-3.703605874	0.07864778	-47.091042	0	0
ENSG00000284730	AC068987.5	4.996615545	9.2554601	0.737770947	-3.72313095	1.337609255	-2.7834219	0.005379	0.013826
ENSG00000082684	SEMA5B	4.806861402	8.9776585	0.636064297	-3.732325476	1.356641408	-2.7511511	0.005939	0.015138
ENSG00000277494	GPIHBP1	17.05941796	31.810338	2.308497576	-3.736339189	0.719535235	-5.1927119	2.07E-07	9.44E-07
ENSG00000145287	PLAC8	191.278025	355.8148	26.74125312	-3.743309188	0.214774715	-17.429003	4.97E-68	1.24E-66
ENSG00000278791	AC114498.2	2.683185066	5.048338	0.318032149	-3.771079129	1.650992006	-2.2841292	0.022364	0.049865
ENSG00000164619	BMPER	124.8576758	232.84683	16.86851664	-3.78810374	0.266889057	-14.193552	1.00E-45	1.76E-44
ENSG00000108602	ALDH3A1	2.742801119	5.1675701	0.318032149	-3.798217813	1.631252485	-2.3284058	0.019891	0.044929
ENSG00000229771	AL035665.1	2.801842834	5.2856535	0.318032149	-3.825241897	1.644592596	-2.3259511	0.020021	0.045196
ENSG00000136960	ENPP2	66.12880036	123.47209	8.785514168	-3.835131865	0.378823801	-10.123788	4.33E-24	4.54E-23
ENSG00000229666	MAST4-AS1	2.847073297	5.3446952	0.349451359	-3.839000174	1.660489889	-2.3119684	0.020779	0.046736
ENSG00000124743	KLHL31	2.821511546	5.3249909	0.318032149	-3.849546885	1.630845995	-2.3604601	0.018252	0.041586
ENSG00000065618	COL17A1	51.02195773	95.335675	6.708240125	-3.855329106	0.430934928	-8.9464299	3.67E-19	3.18E-18
ENSG00000166828	SCNN1G	2.895756745	5.422628	0.368885473	-3.87114601	1.608797077	-2.4062364	0.016118	0.037319
ENSG00000092850	TEKT2	2.945081403	5.5407114	0.349451359	-3.896534396	1.61241174	-2.4165877	0.015667	0.036385

ENSG00000214708	AC116407.1	2.975244816	5.5816042	0.368885473	-3.905397868	1.640691749	-2.3803361	0.017297	0.03967
ENSG00000196329	GIMAP5	11.12971475	20.885594	1.373835244	-3.922471454	0.917007061	-4.2774714	1.89E-05	7.10E-05
ENSG00000196169	KIF19	19.66536032	36.850229	2.480491664	-3.924858478	0.73422228	-5.3455998	9.01E-08	4.24E-07
ENSG00000101695	RNF125	66.92436213	125.86836	7.98036872	-3.988250189	0.381873516	-10.443904	1.56E-25	1.71E-24
ENSG00000223392	CLDN10-AS1	6.028354197	11.338372	0.718336833	-4.031421967	1.284336917	-3.1389131	0.001696	0.00482
ENSG00000226963	AC078883.2	5.903567446	11.139651	0.667483508	-4.033603064	1.300802977	-3.1008563	0.00193	0.005441
ENSG00000250722	SELENOP	8.808358443	16.66262	0.954096446	-4.035985999	1.068118738	-3.778593	0.000158	0.000528
ENSG00000148671	ADIRF	233.4951599	440.13991	26.85040878	-4.050357643	0.220227517	-18.391696	1.53E-75	4.23E-74
ENSG00000163520	FBLN2	223.9749408	422.5621	25.38777909	-4.053046921	0.209465647	-19.349459	2.06E-83	6.51E-82
ENSG00000131386	GALNT15	269.9507783	509.76167	30.13988594	-4.087549846	0.194724419	-20.99146	7.85E-98	2.90E-96
ENSG00000269425	AC104521.1	3.388130209	6.4073749	0.368885473	-4.09928797	1.628805934	-2.5167442	0.011844	0.02829
ENSG00000176194	CIDEA	53.1148823	100.45072	5.779040977	-4.10875502	0.449260578	-9.1455944	5.93E-20	5.33E-19
ENSG00000004799	PDK4	232.0866373	439.46589	24.70738338	-4.164821597	0.21336224	-19.519956	7.43E-85	2.41E-83
ENSG00000272146	ARF4-AS1	3.525679044	6.6824726	0.368885473	-4.172620911	1.619445154	-2.5765744	0.009978	0.024243
ENSG00000088053	GP6	9.85899712	18.662191	1.055803095	-4.17527478	1.054037811	-3.9612192	7.46E-05	0.00026
ENSG00000132840	BHMT2	3.528421257	6.7388104	0.318032149	-4.194295651	1.586032273	-2.644521	0.008181	0.020231
ENSG00000185313	SCN10A	6.659499	12.651514	0.667483508	-4.210546111	1.255325164	-3.3541478	0.000796	0.002396
ENSG00000231768	LINC01354	1.876097689	3.7521954	0	-4.306550632	1.842943331	-2.3367787	0.019451	0.044049
ENSG00000132561	MATN2	596.7975679	1135.9837	57.61146098	-4.311233568	0.139451147	-30.915727	7.34E-210	5.75E-208
ENSG00000175287	PHYHD1	1.896544046	3.7930881	0	-4.318950565	1.879664457	-2.2977242	0.021577	0.048312
ENSG00000140873	ADAMTS18	1321.471845	2524.0568	118.8868794	-4.412972012	0.199947493	-22.070654	6.05E-108	2.40E-106
ENSG00000254307	AP003692.1	2.024073009	4.048146	0	-4.416474248	1.824222085	-2.4210179	0.015477	0.036004
ENSG00000126353	CCR7	4.167621363	8.0172106	0.318032149	-4.433682784	1.498119334	-2.9594991	0.003081	0.008335
ENSG00000237614	AC073257.2	2.053019528	4.1060391	0	-4.43413117	1.785955023	-2.4827787	0.013036	0.03088
ENSG00000118308	LRMP	7.883634498	15.048932	0.718336833	-4.439143979	1.216740138	-3.6483912	0.000264	0.000855
ENSG00000012223	LTF	2.092189225	4.1843785	0	-4.456943445	1.784070771	-2.4981876	0.012483	0.029688
ENSG00000283528	TCAF2C	2.102412403	4.2048248	0	-4.462545075	1.774453148	-2.5148847	0.011907	0.028422
ENSG00000246985	SOCS2-AS1	2.101838065	4.2036761	0	-4.462700688	1.801655595	-2.4769999	0.013249	0.031328
ENSG00000272277	AL031963.3	4.402807273	8.4875824	0.318032149	-4.521277909	1.482524851	-3.0497148	0.002291	0.006375
ENSG00000187955	COL14A1	4.412827145	8.5076221	0.318032149	-4.531317083	1.544024201	-2.9347449	0.003338	0.008963
ENSG00000148123	PLPPR1	16.53158463	31.791041	1.272128595	-4.546890402	0.873180617	-5.2072736	1.92E-07	8.77E-07
ENSG00000137033	IL33	1944.080357	3734.4549	153.7057712	-4.603849456	0.086976833	-52.931905	0	0
ENSG00000082482	KCNK2	8.698160905	16.760258	0.636064297	-4.620514747	1.21417154	-3.8054876	0.000142	0.000477
ENSG00000165215	CLDN3	8.892009243	17.097101	0.686917622	-4.632494377	1.218780732	-3.8009252	0.000144	0.000485
ENSG00000282304	AC140479.5	2.357470331	4.7149407	0	-4.634797396	1.723553239	-2.6890944	0.007165	0.01792
ENSG00000151715	TMEM45B	17.85698553	34.340136	1.373835244	-4.640214184	0.859007209	-5.4018338	6.60E-08	3.14E-07
ENSG00000187513	GJA4	3445.62031	6630.6637	260.5769589	-4.666914927	0.067467165	-69.173129	0	0
ENSG00000167641	PPP1R14A	13.72040473	26.385006	1.055803095	-4.672082287	0.97650725	-4.7844829	1.71E-06	7.18E-06
ENSG00000251689	AC079140.5	2.45683042	4.9136608	0	-4.684726157	1.768730228	-2.648638	0.008082	0.020008
ENSG00000196154	S100A4	9.471018958	18.25512	0.686917622	-4.724415291	1.199825229	-3.9375862	8.23E-05	0.000286
ENSG00000279302	AC013643.3	2.51509449	5.030189	0	-4.727883517	1.697002972	-2.7860196	0.005336	0.013729
ENSG00000169933	FRMPD4	5.19526582	10.04108	0.349451359	-4.763876692	1.451606106	-3.2817971	0.001031	0.003044
ENSG00000225194	LINC00092	5.273673432	10.178461	0.368885473	-4.780165147	1.438114359	-3.3239117	0.000888	0.002648
ENSG00000137474	MYO7A	89.56900041	172.97809	6.159911548	-4.803810359	0.395370166	-12.150159	5.73E-34	7.71E-33
ENSG00000118777	ABCG2	1155.57293	2234.3326	76.81324087	-4.853597264	0.173037929	-28.049326	4.07E-173	2.57E-171
ENSG00000171345	KRT19	684.2775719	1322.9021	45.65307434	-4.872231352	0.149022695	-32.694559	1.87E-234	1.68E-232
ENSG00000230309	AL121718.1	5.867574858	11.417118	0.318032149	-4.939609537	1.423932301	-3.4689918	0.000522	0.001619
ENSG00000165521	AL121768.1	2.967520612	5.9350412	0	-4.967425211	1.671135854	-2.9724844	0.002954	0.008024
ENSG00000164116	GUCY1A1	603.3431478	1169.549	37.13728925	-4.975673329	0.161017979	-30.901353	1.15E-209	8.89E-208
ENSG00000170748	RBMXL2	6.229715393	12.141399	0.318032149	-5.033646053	1.399056946	-3.597885	0.000321	0.001026
ENSG00000129048	ACKR4	187.8305261	364.77045	10.89059845	-5.051348554	0.291368447	-17.336635	2.49E-67	6.18E-66
ENSG00000148357	HMCN2	3.202909827	6.4058197	0	-5.072170772	1.59564455	-3.1787598	0.001479	0.00424
ENSG00000155026	RSPH10B	3.241505186	6.4830104	0	-5.086963629	1.602750001	-3.1738971	0.001504	0.004307
ENSG00000254620	AL050327.1	3.360162955	6.7203259	0	-5.131674906	1.638671512	-3.1316068	0.001739	0.004934
ENSG00000163687	DNASE1L3	19.45093519	37.916355	0.985515657	-5.20635107	0.941548209	-5.529564	3.21E-08	1.57E-07
ENSG00000004468	CD38	3.742546539	7.4850931	0	-5.303327136	1.552115442	-3.4168381	0.000634	0.001942
ENSG00000260244	AC104083.1	163.5175237	319.22214	7.812910713	-5.333909877	0.347622351	-15.343978	3.89E-53	7.70E-52
ENSG00000276953	TRBV12-4	3.948414897	7.8968298	0	-5.377718991	1.533158397	-3.5076082	0.000452	0.001413
ENSG00000170011	MYRIP	1309.685737	2561.9532	57.41827801	-5.462930044	0.125946475	-43.375013	0	0
ENSG00000124479	NDP	16.00637239	31.294408	0.718336833	-5.504714056	1.136032324	-4.8455611	1.26E-06	5.35E-06
ENSG00000132470	ITGB4	219.9933602	431.01339	8.973333394	-5.589600017	0.311798246	-17.926977	7.26E-72	1.92E-70
ENSG00000166106	ADAMTS15	4.656676591	9.3133532	0	-5.611511293	1.474257493	-3.8063305	0.000141	0.000475
ENSG00000161798	AQP5	5.068413306	10.136827	0	-5.730326198	1.490068749	-3.8456791	0.00012	0.000409
ENSG00000113389	NPR3	47.54747826	93.289397	1.805559139	-5.754637439	0.700978447	-8.2094356	2.22E-16	1.73E-15
ENSG00000163815	CLEC3B	10.73790359	21.126356	0.349451359	-5.828862	1.338616928	-4.3543914	1.33E-05	5.09E-05
ENSG00000168447	SCNN1B	5.541860123	11.08372	0	-5.859183567	1.453507884	-4.0310642	5.55E-05	0.000197
ENSG00000167434	CA4	18.14313756	35.936824	0.349451359	-6.597991269	1.259154544	-5.240017	1.61E-07	7.39E-07
ENSG00000135218	CD36	90.43974829	179.14422	1.7352717	-6.702413286	0.675967954	-9.9152826	3.57E-23	3.62E-22
ENSG00000167037	SGSM1	36.42216916	72.126001	0.718336833	-6.708614314	1.065878075	-6.2939791	3.09E-10	1.75E-09
ENSG00000154096	THY1	37.39317866	74.087455	0.698902719	-6.746694271	1.071223485	-6.2981202	3.01E-10	1.71E-09
ENSG00000132514	CLEC10A	22.8168827	45.26488	0.368885473	-6.931001707	1.246292362	-5.5612968	2.68E-08	1.32E-07
ENSG00000152207	CYSLTR2	43.68568428	86.684451	0.686917622	-6.977944924	1.059960075	-6.5832149	4.60E-11	2.75E-10
ENSG00000049540	ELN	98.84753812	196.30926	1.385820341	-7.15724294	0.748894916	-9.5570724	1.21E-21	1.16E-20
ENSG00000240583	AQP1	2169.336444	4309.8333	28.83958442	-7.226292684	0.435552984	-16.591076	8.09E-62	1.84E-60
ENSG00000102575	ACP5	105.5122637	209.72098	1.303547806	-7.252936277	0.745364618	-9.7307225	2.23E-22	2.20E-21
ENSG00000276409	CCL14	18.60433369	37.208667	0	-7.612317202	1.261009258	-6.0366862	1.57E-09	8.48E-09
ENSG00000163083	INHBB	695.4974987	1386.2015	4.79352532	-8.168493491	0.392612697	-20.805475	3.86E-96	1.41E-94
ENSG00000003137	CYP26B1	132.6249611	265.24992	0	-10.4463661	1.190660131	-8.7735919	1.73E-18	1.47E-17

Table S2: RNA-Seq analysis of the differentially expressed genes in HAECs with or without cytokine treatment (adjusted *p* value < 0.05 and Log2|Fold Change| > 0.5).

EnsemblID	GeneSymbol	baseMean	baseMean_Control	baseMean_TGF-ß1 + IL-1ß	log2FoldChange	lfcSE	stat	pvalue	padj
ENSG00000041982	TNC	8000.664023	3.5555555	15997.77249	12.12578349	0.419601686	28.8983192	1.25E-183	7.60E-182
ENSG00000123610	TNFAIP6	1782.577724	0.8965022	3564.258945	11.95956406	0.835062213	14.3217641	1.60E-46	2.52E-45
ENSG00000170054	SERPINA9	217.1443375	0	434.288675	11.3642871	1.187770531	9.56774629	1.09E-21	9.05E-21
ENSG00000178860	MSC	215.8606654	0	431.7213307	11.35576415	1.186498001	9.57082452	1.06E-21	8.79E-21
ENSG00000125730	C3	32582.34524	35.25873	65129.43176	10.85344944	0.138466834	78.3830259	0	0
ENSG00000163673	DCLK3	1292.980809	1.5587648	2584.402854	10.75807777	0.649954466	16.5520484	1.55E-61	3.11E-60
ENSG00000172137	CALB2	134.8029428	0	269.6058857	10.67659425	1.19188016	8.95777495	3.31E-19	2.51E-18
ENSG00000178776	C5orf46	115.6842385	0	231.368477	10.45633175	1.194154512	8.75626366	2.02E-18	1.48E-17
ENSG00000166923	GREM1	406.7019422	0.6056649	812.7982195	10.41187235	1.026068256	10.1473487	3.40E-24	3.07E-23
ENSG00000164400	CSF2	6627.709359	9.7984633	13245.62025	10.39405651	0.256350475	40.5462736	0	0
ENSG00000124102	PI3	2128.339756	3.2668725	4253.412639	10.33995058	0.440525929	23.471832	7.91E-122	3.18E-120
ENSG00000125538	IL1B	18137.02414	30.108879	36243.93939	10.22370337	0.211377829	48.3669617	0	0
ENSG00000170209	ANKK1	188.6421311	0.2908372	376.9934249	10.19740136	1.189934372	8.56971746	1.04E-17	7.37E-17
ENSG00000277632	CCL3	92.61042516	0	185.2208503	10.13405552	1.202891074	8.42474912	3.62E-17	2.52E-16
ENSG00000142149	HUNK	816.7641981	1.4738682	1632.054528	10.09573629	0.649985184	15.532256	2.10E-54	3.76E-53
ENSG00000166920	C15orf48	1705.783191	3.5598639	3408.006518	9.895048371	0.42088803	23.5099306	3.23E-122	1.30E-120
ENSG00000182752	PAPPA	1556.065583	3.2342652	3108.896901	9.887883924	0.440847816	22.4292456	2.04E-111	7.47E-110
ENSG00000235531	MSC-AS1	74.92974084	0	149.8594817	9.828800421	1.20355109	8.16650037	3.17E-16	2.12E-15
ENSG00000135373	EHF	135.7305276	0.3169819	271.1440733	9.722616239	1.191664607	8.15885291	3.38E-16	2.25E-15
ENSG00000096996	IL12RB1	61.12833992	0	122.2566798	9.535775879	1.206693174	7.9024031	2.74E-15	1.74E-14
ENSG00000182050	MGAT4C	58.45925689	0	116.9185138	9.471753734	1.209102451	7.8337065	4.74E-15	2.98E-14
ENSG00000143333	RGS16	57.19673338	0	114.3934668	9.44018081	1.209350675	7.80599127	5.90E-15	3.70E-14
ENSG00000187037	GPR141	53.94888521	0	107.8977704	9.354025727	1.213329109	7.7093887	1.26E-14	7.82E-14
ENSG00000276085	CCL3L1	49.62761341	0	99.25522681	9.233869554	1.214501464	7.60301229	2.89E-14	1.75E-13
ENSG00000166523	CLEC4E	47.75904708	0	95.51809416	9.178629584	1.216907377	7.54258685	4.61E-14	2.76E-13
ENSG00000173432	SAA1	46.86111523	0	93.72223046	9.152070837	1.215500844	7.52946481	5.09E-14	3.03E-13
ENSG00000108700	CCL8	254.0051555	0.924801	507.08551	9.145937334	0.843252238	10.8460279	2.08E-27	2.09E-26
ENSG00000230067	HSPD1P6	169.0963385	0.5773661	337.615311	9.144407087	1.033256247	8.85008643	8.75E-19	6.51E-18
ENSG00000156427	FGF18	43.5411974	0	87.0823948	9.0461288	1.219116724	7.4202319	1.17E-13	6.86E-13
ENSG00000232810	TNF	40.22590325	0	80.4518065	8.930919237	1.22791875	7.27321676	3.51E-13	2.02E-12
ENSG00000259354	AC025580.2	38.63332927	0	77.26665855	8.873261331	1.223302929	7.25352741	4.06E-13	2.32E-12
ENSG00000163735	CXCL5	10911.78543	50.610308	21772.96056	8.754065257	0.121380797	72.1206771	0	0
ENSG00000213886	UBD	69.28130159	0.3169819	138.2456213	8.751581967	1.203958635	7.26900552	3.62E-13	2.08E-12
ENSG00000124875	CXCL6	47229.73991	220.88392	94238.59591	8.735046591	0.157028941	55.6269853	0	0
ENSG00000115009	CCL20	1308.640698	6.4409999	2610.840396	8.702190712	0.322049067	27.0213194	8.30E-161	4.32E-159
ENSG00000163734	CXCL3	7944.055981	39.192467	15848.91949	8.663559322	0.133921814	64.6911738	0	0
ENSG00000182326	C1S	1747.271034	8.5609888	3485.981079	8.65517374	0.276112806	31.3465133	1.09E-215	7.87E-214
ENSG00000269526	ERVV-1	32.39124315	0	64.7824863	8.620279186	1.233320777	6.98948672	2.76E-12	1.52E-11
ENSG00000203685	STUM	230.1455623	1.1547321	459.1363925	8.588437418	0.735009397	11.6847995	1.52E-31	1.69E-30
ENSG00000179761	PIPOX	31.5550926	0	63.1101852	8.581949734	1.233043435	6.95997358	3.40E-12	1.87E-11
ENSG00000108342	CSF3	28012.28529	150.55992	55874.01066	8.533559469	0.146060401	58.4248668	0	0
ENSG00000187689	AMTN	28.40353406	0	56.80706812	8.430438741	1.238914773	6.80469628	1.01E-11	5.39E-11
ENSG00000116132	PRRX1	1381.911046	8.343983	2755.478109	8.365848718	0.28068659	29.8049462	3.37E-195	2.15E-193
ENSG00000132185	FCRLA	23.50414417	0	47.00828833	8.156787556	1.250098398	6.52491641	6.80E-11	3.44E-10
ENSG00000154451	GBP5	423.1246991	2.954199	843.2951992	8.143635654	0.468003583	17.4007977	8.14E-68	1.82E-66
ENSG00000095752	IL11	22.72056513	0	45.44113027	8.108378889	1.254215057	6.46490316	1.01E-10	5.06E-10
ENSG00000158714	SLAMF8	22.21439462	0	44.42878924	8.077074527	1.268889364	6.36546791	1.95E-10	9.53E-10
ENSG00000163121	NEURL3	306.4177034	2.376833	610.4585739	7.998803756	0.525028008	15.2350039	2.07E-52	3.62E-51
ENSG00000175093	SPSB4	21.03166867	0	42.06333734	7.997369249	1.264857854	6.3227415	2.57E-10	1.25E-09
ENSG00000146966	DENND2A	20.66637832	0	41.33275664	7.969945821	1.261228939	6.31919042	2.63E-10	1.27E-09
ENSG00000236453	AC003092.1	72.90790671	0.5773661	145.2384474	7.928712345	1.051522126	7.54022398	4.69E-14	2.80E-13
ENSG00000170075	GPR37L1	36.3306473	0.2908372	72.37045738	7.816144889	1.226257703	6.3739823	1.84E-10	9.03E-10
ENSG00000261040	WFDC21P	18.21508901	0	36.43017802	7.788540979	1.276308595	6.1023964	1.04E-09	4.86E-09
ENSG00000134548	SPX	18.20203875	0	36.40407749	7.78561127	1.295623919	6.00915988	1.86E-09	8.53E-09
ENSG00000182674	KCNB2	18.11166688	0	36.22333377	7.779339071	1.288097885	6.03940055	1.55E-09	7.11E-09
ENSG00000146938	NLGN4X	18.11032115	0	36.22064231	7.776299713	1.32608474	5.86410467	4.52E-09	2.01E-08
ENSG00000100767	PAPLN	8368.642074	80.394831	16656.88932	7.697198179	0.213134816	36.1142226	1.36E-285	1.47E-283
ENSG00000175445	LPL	16.84382101	0	33.68764201	7.676750741	1.28312699	5.98284566	2.19E-09	9.97E-09
ENSG00000232618	AL355304.1	16.30949147	0	32.61898293	7.627591076	1.29935673	5.8702825	4.35E-09	1.94E-08
ENSG00000049249	TNFRSF9	206.0891523	2.0859957	410.0923088	7.617211906	0.56484361	13.4855237	1.90E-41	2.71E-40
ENSG00000155511	GRIA1	31.48598464	0.3169819	62.65498739	7.607316154	1.237824425	6.14571502	7.96E-10	3.73E-09
ENSG00000177614	PGBD5	31.37963767	0.2908372	62.46843811	7.606214027	1.244819054	6.11029692	9.94E-10	4.63E-09
ENSG00000042062	RIPOR3	85.25478444	0.924801	169.5847679	7.565008141	0.862451283	8.77151938	1.76E-18	1.30E-17
ENSG00000050730	TNIP3	1080.294197	11.543487	2149.044908	7.529997082	0.242459208	31.0567585	9.25E-212	6.58E-210
ENSG00000143340	FAM163A	28.97921452	0.288683	57.66974601	7.489843031	1.242800589	6.02658471	1.67E-09	7.68E-09
ENSG00000137462	TLR2	1169.082298	13.488756	2324.675839	7.435623185	0.227470984	32.6882271	2.30E-234	1.83E-232
ENSG00000276980	AC008760.2	14.04772885	0	28.09545769	7.412710869	1.308923683	5.6632109	1.49E-08	6.36E-08
ENSG00000134326	CMPK2	1316.206957	15.896337	2616.517577	7.370252422	0.210983402	34.9328542	2.36E-267	2.33E-265
ENSG00000114270	COL7A1	172.4144826	2.1382851	342.6906801	7.357386612	0.567257119	12.9701089	1.81E-38	2.40E-37
ENSG00000169436	COL22A1	118.3311425	1.4541861	235.2080988	7.302660898	0.676056458	10.8018507	3.37E-27	3.36E-26
ENSG00000128342	LIF	1490.714649	19.05509	2962.374207	7.299428641	0.193055272	37.8100457	0	0
ENSG00000170419	VSTM2A	25.02749565	0.288683	49.76630827	7.276310272	1.24984411	5.82177426	5.82E-09	2.57E-08
ENSG00000080573	COL5A3	90.40024994	1.2134841	179.5870158	7.232085524	0.754525516	9.58494494	9.25E-22	7.68E-21
ENSG00000115919	KYNU	470.4399851	6.3081224	934.5718478	7.22019159	0.331486489	21.7812546	3.49E-105	1.20E-103
ENSG00000186340	THBS2	246.2334734	3.3453065	489.1216403	7.218259869	0.456045152	15.82795	2.00E-56	3.68E-55
ENSG00000259342	AC025580.1	24.00114503	0.3169819	47.68530816	7.214009092	1.250190006	5.77033015	7.91E-09	3.45E-08
ENSG00000139985	ADAM21	12.10965333	0	24.21930665	7.19716198	1.353128052	5.31890679	1.04E-07	4.18E-07
ENSG00000268964	ERVV-2	12.02100237	0	24.04200474	7.191203558	1.330988222	5.40290548	6.56E-08	2.67E-07
ENSG00000250771	AC106865.1	11.80996597	0	23.61993194	7.162652058	1.318687229	5.43165347	5.58E-08	2.29E-07
ENSG00000178882	RFLNA	1337.763787	18.772102	2656.755472	7.143271548	0.193358327	36.9431804	9.37E-299	1.08E-296

ENSG00000145824	CXCL14	11.65008491	0	23.30016983	7.142568316	1.329533958	5.37223459	7.78E-08	3.15E-07
ENSG00000243742	RPLP0P2	83.65173222	1.1873394	166.1161251	7.120027065	0.766356734	9.2907477	1.53E-20	1.22E-19
ENSG00000233785	AC131011.1	10.89436018	0	21.78872035	7.049896896	1.348344839	5.2285563	1.71E-07	6.72E-07
ENSG00000105246	EBI3	2239.60677	33.708876	4445.504663	7.043070635	0.146499782	48.0756391	0	0
ENSG00000271503	CCL5	453.8305932	6.8833342	900.7778522	7.036558612	0.317133447	22.1880053	4.48E-109	1.60E-107
ENSG00000141052	MYOCD	10.66496993	0	21.32993986	7.016433231	1.339374801	5.23858835	1.62E-07	6.38E-07
ENSG00000084636	COL16A1	1438.931082	22.032512	2855.829652	7.015573031	0.179608666	39.060326	0	0
ENSG00000144191	CNGA3	154.5467604	2.3506883	306.7428325	7.006525114	0.538847025	13.0028093	1.18E-38	1.57E-37
ENSG00000108688	CCL7	19.65938573	0.3169819	39.00178956	6.925127336	1.272711002	5.44124104	5.29E-08	2.18E-07
ENSG00000255921	AC026310.2	9.54678718	0	19.09357436	6.856942277	1.36124717	5.03724998	4.72E-07	1.80E-06
ENSG00000169429	CXCL8	91996.68695	1586.0197	182407.3542	6.844818663	0.046143911	148.336336	0	0
ENSG00000179148	ALOXE3	33.43178081	0.5795203	66.28404137	6.79750823	1.080565955	6.29069258	3.16E-10	1.53E-09
ENSG00000187123	LYPD6	233.8831298	4.2199724	463.5462871	6.793432147	0.411250981	16.5189446	2.68E-61	5.35E-60
ENSG00000224173	AC007422.1	9.052625931	0	18.10525186	6.783199416	1.389996136	4.88001315	1.06E-06	3.91E-06
ENSG00000131459	GFPT2	957.1245512	17.554309	1896.694793	6.751739329	0.204477029	33.0195493	4.26E-239	3.54E-237
ENSG00000189292	ALKAL2	8.734279992	0	17.46855998	6.730523342	1.400768323	4.80487974	1.55E-06	5.63E-06
ENSG00000225492	GBP1P1	32.07181911	0.6339638	63.50967443	6.730473472	1.087253618	6.1903436	6.00E-10	2.84E-09
ENSG00000007908	SELE	13771.35925	258.41562	27284.30287	6.723468158	0.19866052	33.8440076	4.45E-251	4.05E-249
ENSG00000148346	LCN2	8.62239842	0	17.24479684	6.710269117	1.368529735	4.90326877	9.43E-07	3.49E-06
ENSG00000113645	WWC1	8.570849118	0	17.14169824	6.702828155	1.392158276	4.81470266	1.47E-06	5.37E-06
ENSG00000164761	TNFRSF11B	61.30971668	1.1590405	121.4603929	6.672314089	0.768237197	8.68522654	3.78E-18	2.73E-17
ENSG00000138670	RASGEF1B	8.347428283	0	16.69485657	6.665915305	1.393435926	4.7837975	1.72E-06	6.23E-06
ENSG00000165478	HEPACAM	30.35715791	0.6056649	60.10865089	6.654261465	1.087165537	6.12074357	9.31E-10	4.34E-09
ENSG00000106236	NPTX2	2665.366495	52.837029	5277.895961	6.643000022	0.12281774	54.0882779	0	0
ENSG00000136244	IL6	17894.2589	355.31312	35433.20467	6.637090663	0.065689134	101.037878	0	0
ENSG00000138316	ADAMTS14	8.111674663	0	16.22334933	6.620963295	1.378589612	4.80270795	1.57E-06	5.69E-06
ENSG00000134321	RSAD2	1297.71841	26.516853	2568.919967	6.604883343	0.443094856	14.9062514	3.00E-50	5.05E-49
ENSG00000183813	CCR4	298.1894589	6.2002978	590.1786199	6.586322406	0.85582856	7.69584321	1.41E-14	8.66E-14
ENSG00000164690	SHH	86.12051676	1.760397	170.4806365	6.574800228	0.630179151	10.433224	1.75E-25	1.65E-24
ENSG00000185811	IKZF1	7.746009144	0	15.49201829	6.554303867	1.386954028	4.72568213	2.29E-06	8.20E-06
ENSG00000149295	DRD2	7.73683222	0	15.47366444	6.552898348	1.386874888	4.72493835	2.30E-06	8.23E-06
ENSG00000253593	AC110741.1	7.73541605	0	15.4708321	6.552659415	1.392838236	4.70453729	2.54E-06	9.06E-06
ENSG00000231574	LINC02015	7.69343763	0	15.38687526	6.540988876	1.427378765	4.582518	4.59E-06	1.59E-05
ENSG00000179826	MRGPRX3	7.419883663	0	14.83976733	6.490880367	1.401455359	4.63152845	3.63E-06	1.27E-05
ENSG00000119946	CNNM1	14.16131329	0.288683	28.03394355	6.451160798	1.309584745	4.92611175	8.39E-07	3.12E-06
ENSG00000081041	CXCL2	8048.249352	182.0581	15914.4406	6.450302157	0.075819343	85.0746246	0	0
ENSG00000205436	EXOC3L4	7.202108721	0	14.40421744	6.449081941	1.421717723	4.53611982	5.73E-06	1.97E-05
ENSG00000112303	VNN2	7.107882307	0	14.21576461	6.427562848	1.451790581	4.42733472	9.54E-06	3.20E-05
ENSG00000131203	IDO1	167.4301941	3.7962576	331.0641305	6.418296807	0.433735527	14.7977198	1.52E-49	2.51E-48
ENSG00000116183	PAPPA2	38.34777007	0.8703575	75.82518266	6.407112067	0.896209179	7.14912569	8.73E-13	4.92E-12
ENSG00000235885	LINC01828	6.818089352	0	13.6361787	6.37054334	1.441982447	4.4179063	9.97E-06	3.34E-05
ENSG00000248371	LINC02056	134.7058393	3.301634	266.1100445	6.341667853	0.473043985	13.406085	5.57E-41	7.82E-40
ENSG00000179362	HMGN2P46	36.71431405	0.9509457	72.47768241	6.333662622	0.906560967	6.98647179	2.82E-12	1.55E-11
ENSG00000115232	ITGA4	3676.683783	91.79222	7261.575346	6.306789967	0.196021421	32.1739836	4.08E-227	3.09E-225
ENSG00000138944	SHISAL1	70.78177608	1.7930043	139.7705478	6.286246176	0.637458346	9.86142265	6.12E-23	5.29E-22
ENSG00000251533	LINC00605	6.273471451	0	12.5469429	6.250015577	1.432611339	4.36267354	1.28E-05	4.26E-05
ENSG00000233005	AC018742.1	6.264294526	0	12.52858905	6.248284744	1.435913738	4.35143462	1.35E-05	4.48E-05
ENSG00000113578	FGF1	6.148183232	0	12.29636646	6.21885415	1.463957285	4.24797514	2.16E-05	6.99E-05
ENSG00000038427	VCAN	2436.406952	64.778086	4808.035817	6.214605212	0.113064064	54.9653444	0	0
ENSG00000198910	L1CAM	98.45230308	2.6895065	194.2150997	6.175306322	0.532358506	11.5999017	4.13E-31	4.53E-30
ENSG00000073756	PTGS2	16667.80541	458.48993	32877.12088	6.164573149	0.057860811	106.541423	0	0
ENSG00000237927	AL078604.2	5.889452082	0	11.77890416	6.159525579	1.449377252	4.24977387	2.14E-05	6.94E-05
ENSG00000006210	CX3CL1	10921.93977	303.1589	21540.72065	6.154667689	0.067452006	91.2451391	0	0
ENSG00000183486	MX2	3306.078018	92.979968	6519.176067	6.131334504	0.230614268	26.5869695	9.60E-156	4.82E-154
ENSG00000226320	LINC01811	5.701322775	0	11.40264555	6.112840907	1.454369789	4.20308573	2.63E-05	8.46E-05
ENSG00000127863	TNFRSF19	1076.831247	31.103203	2122.55929	6.083635661	0.157961732	38.5133513	0	0
ENSG00000123572	NRK	5.536494519	0	11.07298904	6.069662804	1.463380438	4.14769984	3.36E-05	0.000107
ENSG00000166922	SCG5	394.1895089	11.680673	776.6983451	6.060596785	0.257503839	23.5359473	1.75E-122	7.08E-121
ENSG00000122133	PAEP	10.81531696	0.2908372	21.33979668	6.055131477	1.351704479	4.4796267	7.48E-06	2.54E-05
ENSG00000148344	PTGES	5.384374218	0	10.76874844	6.028046693	1.479799334	4.07355684	4.63E-05	0.000145
ENSG00000283408	MIR548A1HG	5.291887495	0	10.58377499	6.007235025	1.48404141	4.04788909	5.17E-05	0.000161
ENSG00000115461	IGFBP5	996.0303313	30.664854	1961.395808	6.00532537	0.387393333	15.5018811	3.37E-54	6.02E-53
ENSG00000261618	LINC02605	115.3023867	3.5620182	227.0427553	5.987463719	0.458659082	13.0542792	6.01E-39	8.06E-38
ENSG00000056558	TRAF1	2862.043474	89.56287	5634.524077	5.976181605	0.097881656	61.0551744	0	0
ENSG00000237988	OR2I1P	19.0039027	0.5773661	37.43043935	5.975938505	1.126825255	5.30334054	1.14E-07	4.54E-07
ENSG00000250981	AC016650.1	5.154590012	0	10.30918002	5.967143333	1.49123813	4.00146912	6.30E-05	0.000194
ENSG00000163739	CXCL1	76016.26369	2412.3304	149620.1969	5.954520924	0.132582458	44.911831	0	0
ENSG00000222000	AC092675.1	103.0048507	3.2059664	202.803735	5.953079504	0.484235203	12.2937768	9.78E-35	1.18E-33
ENSG00000134339	SAA2	5.013062806	0	10.02612561	5.924950134	1.497942197	3.95539304	7.64E-05	0.000233
ENSG00000153823	PID1	62.34322811	2.0859957	122.6004605	5.87621208	0.603429726	9.73802222	2.08E-22	1.77E-21
ENSG00000155897	ADCY8	4.768455782	0	9.536911565	5.855773195	1.506953972	3.88583414	0.000102	0.000306
ENSG00000228741	AL445985.1	43.90971385	1.5043213	86.31510641	5.852888605	0.714244775	8.19451371	2.52E-16	1.69E-15
ENSG00000120708	TGFBI	13403.75706	456.38575	26351.12836	5.8508182	0.054279848	107.789878	0	0
ENSG00000258602	LINC01629	9.336773747	0.288683	18.38486447	5.840330687	1.363817493	4.28234036	1.85E-05	6.03E-05
ENSG00000123496	IL13RA2	4.694322908	0	9.388645816	5.836410319	1.562867801	3.73442355	0.000188	0.000545
ENSG00000079215	SLC1A3	9.250656919	0.3169819	18.18433194	5.827080634	1.364620015	4.27011224	1.95E-05	6.35E-05
ENSG00000087258	GNAO1	4.656574211	0	9.313148422	5.818146839	1.570931742	3.70362803	0.000213	0.000613
ENSG00000227542	AC092614.1	17.09410074	0.6056649	33.58253656	5.811801404	1.143656742	5.08177077	3.74E-07	1.44E-06
ENSG00000197520	FAM177B	4.610689589	0	9.221379178	5.805914003	1.529204976	3.79668788	0.000147	0.000431
ENSG00000172061	LRRC15	25.36282012	0.8703575	49.85528275	5.802514168	0.927089148	6.25885243	3.88E-10	1.86E-09
ENSG00000184374	COLEC10	4.569034689	0	9.138069379	5.794939514	1.541042857	3.76040127	0.00017	0.000494
ENSG00000170961	HAS2	25.17435174	0.8725117	49.4761918	5.791595156	0.935960348	6.1878638	6.10E-10	2.89E-09
ENSG00000173093	CCDC63	4.553494393	0	9.106988787	5.790722104	1.541260577	3.75713373	0.000172	0.0005
ENSG00000140479	PCSK6	65.63461427	2.3789872	128.8902414	5.755257962	0.571610127	10.0685024	7.61E-24	6.82E-23
ENSG00000197859	ADAMTSL2	24.5803074	0.8986564	48.26195843	5.75381651	0.931565735	6.1765008	6.55E-10	3.09E-09

ENSG00000185101	ANO9	16.32460174	0.5773661	32.07183742	5.752647465	1.147249639	5.01429442	5.32E-07	2.01E-06
ENSG00000117594	HSD11B1	49.19734931	1.8474479	96.54725078	5.749135516	0.658298483	8.7333264	2.47E-18	1.80E-17
ENSG00000112096	SOD2	69664.70174	2574.9434	136754.46	5.731288368	0.047291504	121.190656	0	0
ENSG00000173918	C1QTNF1	3647.601136	137.37771	7157.824564	5.706686081	0.089643112	63.6600625	0	0
ENSG00000160716	CHRNB2	4.240076868	0	8.480153736	5.685056415	1.57201381	3.61641633	0.000299	0.000847
ENSG00000278071	AL161669.3	4.190661214	0	8.381322428	5.670951457	1.564675404	3.62436288	0.00029	0.000823
ENSG00000243225	AC007686.1	4.174422227	0	8.348844453	5.666175283	1.576124643	3.59500456	0.000324	0.000914
ENSG00000140968	IRF8	4.166661472	0	8.333322944	5.663205961	1.68364912	3.36364976	0.000769	0.002059
ENSG00000170153	RNF150	212.881814	8.5226872	417.2409407	5.639310295	0.31121896	18.1200731	2.21E-73	5.37E-72
ENSG00000259867	AC105411.1	4.091487599	0	8.182975198	5.631737726	1.603817906	3.51145707	0.000446	0.001234
ENSG00000168334	XIRP1	4.090788907	0	8.181577815	5.631624707	1.603850318	3.51131564	0.000446	0.001234
ENSG00000154655	L3MBTL4	4.054779901	0	8.109559802	5.620690156	1.564307391	3.59308547	0.000327	0.00092
ENSG00000215571	GRK6P1	4.042770636	0	8.085541273	5.617016302	1.607721183	3.49377514	0.000476	0.001313
ENSG00000148848	ADAM12	567.2374812	22.659719	1111.815244	5.616136361	0.193755378	28.9857057	9.96E-185	6.06E-183
ENSG00000283265	AL356234.3	96.89949743	3.8746916	189.9243032	5.614412083	0.449950673	12.4778391	9.86E-36	1.22E-34
ENSG00000138378	STAT4	102.8289845	4.0741696	201.5837994	5.611948542	0.708354291	7.92251648	2.33E-15	1.49E-14
ENSG00000164181	ELOVL7	4.020885756	0	8.041771512	5.610522107	1.57008409	3.57338957	0.000352	0.000987
ENSG00000128268	MGAT3	3.851827777	0	7.703655553	5.547768357	1.599984053	3.46738978	0.000526	0.001441
ENSG00000225877	PSG8-AS1	3.846880575	0	7.69376115	5.546192594	1.57558546	3.52008363	0.000431	0.001196
ENSG00000240764	PCDHGC5	3.845464405	0	7.69092881	5.545512436	1.660411443	3.33984234	0.000838	0.002234
ENSG00000144802	NFKBIZ	5348.531972	225.93227	10471.13168	5.534924443	0.069899902	79.1835796	0	0
ENSG00000250519	AP002784.1	3.803110814	0	7.606221629	5.532155579	1.62568516	3.40296862	0.000667	0.001801
ENSG00000136688	IL36G	3.754070331	0	7.508140663	5.505636321	1.633832113	3.3697687	0.000752	0.002018
ENSG00000257509	AC073487.1	3.727238249	0	7.454476499	5.496539706	1.603276682	3.42831638	0.000607	0.001651
ENSG00000023445	BIRC3	1832.929537	79.618604	3586.24047	5.492747393	0.110798295	49.5742955	0	0
ENSG00000183742	MACC1	7.374679698	0.288683	14.46067637	5.492053778	1.434116025	3.82957424	0.000128	0.000381
ENSG00000241280	AC106712.1	3.710999262	0	7.421998524	5.491227781	1.599750926	3.43255171	0.000598	0.001628
ENSG00000108679	LGALS3BP	2794.622692	121.52409	5467.721289	5.490066051	0.092603047	59.2860196	0	0
ENSG00000169245	CXCL10	598.9958156	26.234956	1171.756675	5.480565409	0.178069555	30.7776666	5.22E-208	3.62E-206
ENSG00000025708	TYMP	1217.477856	53.647837	2381.307876	5.473837222	0.286150321	19.1292367	1.44E-81	3.88E-80
ENSG00000214212	C19orf38	3.629804325	0	7.25960865	5.464436375	1.624711129	3.36332797	0.00077	0.002061
ENSG00000250742	LINC02381	3.546171006	0	7.092342011	5.424417326	1.631700603	3.324395	0.000886	0.002354
ENSG00000093134	VNN3	3.531348188	0	7.062696376	5.419353786	1.626938799	3.33101269	0.000865	0.002302
ENSG00000138795	LEF1	3.495339182	0	6.990678363	5.406918889	1.607174939	3.36423793	0.000768	0.002055
ENSG00000251230	MIR3945HG	3.495339182	0	6.990678363	5.406918889	1.607174939	3.36423793	0.000768	0.002055
ENSG00000277117	FP565260.3	1095.045999	50.76933	2139.322668	5.40516434	0.134662118	40.1385812	0	0
ENSG00000248362	AC011352.1	3.482631226	0	6.965262451	5.402567733	1.611698604	3.35209556	0.000802	0.002142
ENSG00000283538	AC005972.3	3.479100194	0	6.958200388	5.401342978	1.610100799	3.35466139	0.000795	0.002125
ENSG00000106034	CPED1	109.7408405	5.0924841	214.3891968	5.400459599	0.418657288	12.8994759	4.53E-38	5.92E-37
ENSG00000286084	AL096794.1	3.473454301	0	6.946908603	5.399384918	1.627038614	3.3185352	0.000905	0.002399
ENSG00000198829	SUCNR1	3.439560156	0	6.879120313	5.387388043	1.643331367	3.27833336	0.001044	0.002745
ENSG00000126549	STATH	3.414144244	0	6.828288489	5.377928274	1.69153444	3.17931941	0.001476	0.003793
ENSG00000180525	PRR26	3.329094755	0	6.65818951	5.335452634	1.700543316	3.13749881	0.001704	0.004329
ENSG00000259721	AC090877.2	3.321334001	0	6.642668001	5.332551122	1.636385715	3.25873727	0.001119	0.002931
ENSG00000271811	Z97200.1	3.315688108	0	6.631376216	5.330581106	1.678271006	3.17623381	0.001492	0.003828
ENSG00000162654	GBP4	2142.828129	104.73925	4180.917002	5.32264029	0.096723133	55.0296512	0	0
ENSG00000172986	GXYLT2	36.48091465	1.7647054	71.19712386	5.317246938	0.68963411	7.71024354	1.26E-14	7.77E-14
ENSG00000126259	KIRREL2	3.272617038	0	6.545234077	5.314747613	1.648880153	3.22324676	0.001267	0.003298
ENSG00000042980	ADAM28	30.20392044	1.4455693	58.96227154	5.310292017	0.748852156	7.09124221	1.33E-12	7.43E-12
ENSG00000145681	HAPLN1	3.252847019	0	6.505694039	5.307262582	1.670591943	3.17687548	0.001489	0.003821
ENSG00000279484	KLHL30-AS1	12.07398666	0.5795203	23.56845307	5.306077241	1.199796381	4.42248145	9.76E-06	3.27E-05
ENSG00000145040	UCN2	3.176275763	0	6.352551526	5.265627545	1.717816539	3.06530262	0.002174	0.005443
ENSG00000170743	SYT9	426.7004101	21.665395	831.7354256	5.256122364	0.198651518	26.4590093	2.87E-154	1.43E-152
ENSG00000116701	NCF2	3.150859851	0	6.301719703	5.255747795	1.724786175	3.04718804	0.00231	0.00575
ENSG00000160223	ICOSLG	1994.382228	102.69802	3886.066433	5.24283147	0.095493473	54.9025113	0	0
ENSG00000279903	AP006248.3	3.090133624	0	6.180267249	5.231744776	1.707908401	3.0632467	0.002189	0.005475
ENSG00000060558	GNA15	91.9451653	4.8342542	179.0560764	5.226821734	0.42277117	12.3632407	4.13E-35	5.04E-34
ENSG00000110848	CD69	174.7300606	9.2254057	340.2347154	5.202322432	0.302918376	17.1740074	4.16E-66	9.08E-65
ENSG00000164093	PITX2	33.73037604	1.7930043	65.66774777	5.197561617	0.690608775	7.52605788	5.23E-14	3.11E-13
ENSG00000130294	KIF1A	3.022739293	0	6.045478585	5.191930533	1.756403069	2.95600174	0.003117	0.007603
ENSG00000144596	GRIP2	3.022739293	0	6.045478585	5.191930533	1.756403069	2.95600174	0.003117	0.007603
ENSG00000127561	SYNGR3	295.7208065	15.785295	575.6563177	5.186390395	0.238179046	21.7751749	3.99E-105	1.37E-103
ENSG00000182585	EPGN	2.978969532	0	5.957939064	5.173598652	1.705695676	3.03313113	0.00242	0.006011
ENSG00000163888	CAMK2N2	10.94908416	0.5795203	21.31864807	5.165340041	1.201980651	4.29735706	1.73E-05	5.66E-05
ENSG00000242258	LINC00996	2.933783601	0	5.867567202	5.155071753	1.689891058	3.05053496	0.002284	0.005697
ENSG00000267095	AC025048.1	5.858372244	0.288683	11.42806146	5.153668568	1.440099029	3.5786904	0.000345	0.000969
ENSG00000279266	AC068860.1	5.839345014	0.3169819	11.36170813	5.146777327	1.468728498	3.50424012	0.000458	0.001265
ENSG00000121594	CD80	2.904836658	0	5.809673315	5.143016037	1.717816927	2.99392558	0.002754	0.006785
ENSG00000226067	LINC00623	10.76413279	0.6056649	20.92260065	5.129574525	1.192902419	4.3000789	1.71E-05	5.59E-05
ENSG00000203812	HIST2H2AA3	2.774601237	0	5.549202475	5.072412238	1.705770014	2.97367886	0.002943	0.007221
ENSG00000167207	NOD2	138.7372872	7.9836228	269.4909516	5.06570465	0.32967672	15.3656729	2.78E-53	4.89E-52
ENSG00000105825	TFPI2	30236.11346	1755.287	58716.93989	5.06428351	0.111314232	45.4953821	0	0
ENSG00000127928	GNGT1	2.73647737	0	5.472954739	5.055844801	1.719268432	2.94069542	0.003275	0.007959
ENSG00000267943	AC010328.1	2.73647737	0	5.472954739	5.055844801	1.719268432	2.94069542	0.003275	0.007959
ENSG00000007372	PAX6	5.477210526	0.3169819	10.63743915	5.051565805	1.477740041	3.41844009	0.00063	0.001708
ENSG00000267607	AC011511.5	15.43732088	0.9509457	29.92369607	5.050814885	0.991525952	5.09398153	3.51E-07	1.35E-06
ENSG00000197580	BCO2	20.4108003	1.2113298	39.61027076	5.050233902	0.857296419	5.89088417	3.84E-09	1.72E-08
ENSG00000106258	CYP3A5	10.0109309	0.5816745	19.44018734	5.032074155	1.207230468	4.16827962	3.07E-05	9.80E-05
ENSG00000100365	NCF4	5.329319949	0.3169819	10.341658	5.009068017	1.471305294	3.40450621	0.000663	0.001791
ENSG00000234076	TPRG1-AS1	5.263300929	0.2908372	10.23576463	4.99689696	1.47838287	3.37997488	0.000725	0.001949
ENSG00000269720	CCDC194	5.269311201	0.3169819	10.2216405	4.99527537	1.472127562	3.39323541	0.000691	0.001861
ENSG00000171631	P2RY6	24.5034159	1.4803308	47.52650097	4.993620617	0.785025678	6.36109207	2.00E-10	9.79E-10
ENSG00000003989	SLC7A2	30343.72125	1858.9851	58828.45737	4.984626328	0.041332384	120.598567	0	0
ENSG00000130176	CNN1	14.57644659	0.9226468	28.23024636	4.973191261	0.991212232	5.01728197	5.24E-07	1.98E-06
ENSG00000102794	ACOD1	2.58647193	0	5.172943861	4.971484676	1.795487656	2.76887711	0.005625	0.013163

ENSG00000159403	C1R	1644.311667	102.2263	3186.397036	4.967240959	0.108801923	45.6539812	0	0
ENSG00000167613	LAIR1	2.568118082	0	5.136236163	4.962899108	1.737290675	2.85668897	0.004281	0.010229
ENSG00000113361	CDH6	5.144022982	0.3169819	9.971064065	4.956550407	1.488539094	3.32980869	0.000869	0.002311
ENSG00000062582	MRPS24	18.9799724	1.2134841	36.74646075	4.938451451	0.863973004	5.71597889	1.09E-08	4.71E-08
ENSG00000114646	CSPG5	150.3477027	9.3812112	291.3141943	4.935723562	0.308232012	16.0130141	1.04E-57	1.96E-56
ENSG00000105963	ADAP1	84.45408063	5.2636632	163.644498	4.934545173	0.412376275	11.9661229	5.35E-33	6.19E-32
ENSG00000256262	USP30-AS1	46.19318191	2.9237459	89.46261787	4.913179647	0.55751041	8.81271373	1.22E-18	9.04E-18
ENSG00000077943	ITGA8	155.4302231	10.21973	300.6407165	4.887726227	0.30232011	16.1673871	8.57E-59	1.65E-57
ENSG00000159261	CLDN14	965.5228359	63.245466	1867.800206	4.884475575	0.122726071	39.7998204	0	0
ENSG00000196099	OR6M1	9.120797423	0.6056649	17.63592992	4.877109795	1.248593652	3.90608248	9.38E-05	0.000283
ENSG00000173391	OLR1	2.423059844	0	4.846119688	4.875271565	1.790522979	2.72281988	0.006473	0.014978
ENSG00000168779	SHOX2	9.04736648	0.6078191	17.48691383	4.870819081	1.240789971	3.92557902	8.65E-05	0.000262
ENSG00000236530	KPNA2P1	2.408935718	0	4.817871436	4.868222383	1.825832487	2.66630286	0.007669	0.017529
ENSG00000133048	CHI3L1	2.399758794	0	4.799517587	4.863509856	1.776521228	2.7376593	0.006188	0.014369
ENSG00000180066	C10orf91	2.394112901	0	4.788225801	4.860744137	1.780443646	2.73007469	0.006332	0.01468
ENSG00000184530	C6orf58	66.68614474	4.3431706	129.0291189	4.85978215	0.468966573	10.3627474	3.66E-25	3.43E-24
ENSG00000233327	USP32P2	2.377873913	0	4.755747826	4.852570874	1.782287964	2.72266377	0.006476	0.014984
ENSG00000145358	DDIT4L	1727.405971	116.25412	3338.557822	4.851135686	0.098606824	49.1967543	0	0
ENSG00000188452	CERKL	2.361634926	0	4.723269851	4.84428643	1.79759471	2.69487132	0.007042	0.016186
ENSG00000227908	AC016596.1	8.886444426	0.6078191	17.16506972	4.838296421	1.235966959	3.91458395	9.06E-05	0.000274
ENSG00000180921	FAM83H	26.00374398	1.7364066	50.2710814	4.818153848	0.718348576	6.70726443	1.98E-11	1.04E-10
ENSG00000026751	SLAMF7	4.65937297	0.2908372	9.027908711	4.818100703	1.565913321	3.07686297	0.002092	0.005244
ENSG00000253522	MIR3142HG	360.3991187	24.732789	696.0654484	4.81378227	0.203036705	23.7089263	2.92E-124	1.20E-122
ENSG00000198771	RCSD1	17.23563122	1.1873394	33.28392306	4.801594519	0.869434053	5.52266673	3.34E-08	1.39E-07
ENSG00000087085	ACHE	65.03214886	4.6458414	125.4184563	4.798557157	0.462973662	10.3646439	3.59E-25	3.36E-24
ENSG00000108813	DLX4	4.601494631	0.288683	8.914306236	4.793377506	1.514556376	3.16487229	0.001552	0.00397
ENSG00000206190	ATP10A	4.601494631	0.288683	8.914306236	4.793377506	1.514556376	3.16487229	0.001552	0.00397
ENSG00000242020	RN7SL68P	2.283647499	0	4.567294999	4.785816283	1.891843433	2.52971055	0.011416	0.025268
ENSG00000075223	SEMA3C	1894.723509	132.85241	3656.594611	4.785799957	0.092625879	51.6680652	0	0
ENSG00000277369	AC010654.1	4.55772487	0.288683	8.826766715	4.781702833	1.511420101	3.16371526	0.001558	0.003984
ENSG00000226450	CYP2D8P	2.270939543	0	4.541879087	4.778709834	1.87325012	2.55102604	0.010741	0.023881
ENSG00000206262	FOXL2NB	2.263877481	0	4.527754961	4.774830886	1.874240821	2.54760799	0.010846	0.024108
ENSG00000125735	TNFSF14	2.245523632	0	4.491047263	4.764781401	1.859690424	2.56213687	0.010403	0.023202
ENSG00000224821	COL4A2-AS2	2.245523632	0	4.491047263	4.764781401	1.859690424	2.56213687	0.010403	0.023202
ENSG00000110693	SOX6	2.238461569	0	4.476923137	4.760664371	1.821020432	2.61428389	0.008941	0.020199
ENSG00000128165	ADM2	179.8062547	12.789578	346.8229313	4.758465366	0.271750189	17.5104399	1.19E-68	2.70E-67
ENSG00000253981	ALG1L13P	2.231399506	0	4.462799011	4.756984268	1.861659346	2.55523884	0.010611	0.023627
ENSG00000181072	CHRM2	2.225753613	0	4.451507225	4.753787108	1.814455031	2.61995311	0.008794	0.019883
ENSG00000185885	IFITM1	1037.693664	75.096844	2000.290484	4.733839652	0.270605581	17.4935034	1.61E-68	3.63E-67
ENSG00000206579	XKR4	2.180567682	0	4.361135364	4.729152827	1.834257843	2.57823776	0.009931	0.022219
ENSG00000163435	ELF3	4.349152163	0.3169819	8.381322428	4.709330066	1.537291137	3.06339505	0.002188	0.005474
ENSG00000139200	PIANP	16.13335315	1.2134841	31.05322225	4.696128627	0.87979937	5.33772674	9.41E-08	3.79E-07
ENSG00000174808	BTC	11.96524292	0.8682033	23.06228256	4.693037003	1.032304952	4.5461731	5.46E-06	1.88E-05
ENSG00000026559	KCNG1	15.90705912	1.1873394	30.62677885	4.68531812	0.88818338	5.27516977	1.33E-07	5.27E-07
ENSG00000285744	AC083837.1	409.378895	30.70885	788.0489398	4.680968195	0.176280953	26.5540214	2.31E-155	1.15E-153
ENSG00000203772	SPRN	15.96325741	1.2156383	30.71087657	4.679197215	0.891148118	5.25075138	1.51E-07	5.99E-07
ENSG00000166592	RRAD	7.83987921	0.5795203	15.10023817	4.668536295	1.247123942	3.74344212	0.000182	0.000527
ENSG00000182901	RGS7	420.7087589	31.928797	809.488721	4.664840855	0.176638064	26.4090353	1.08E-153	5.33E-152
ENSG00000102053	ZC3H12B	2.0898723	0	4.179744599	4.658061235	1.928451917	2.41544069	0.015716	0.033858
ENSG00000148408	CACNA1B	4.172314642	0.3169819	8.027647386	4.646610088	1.566358305	2.96650522	0.003012	0.007382
ENSG00000265096	C1QTNF1-AS1	2.067987419	0	4.135974838	4.645679932	2.017233708	2.30299539	0.021279	0.044545
ENSG00000255521	AL356215.1	4.152519313	0.288683	8.0163556	4.645107464	1.542559249	3.01129922	0.002601	0.006434
ENSG00000183833	MAATS1	2.064456388	0	4.128912775	4.642794263	1.902890464	2.43986417	0.014693	0.031847
ENSG00000203364	AL390760.1	4.134165464	0.288683	7.979647902	4.639602643	1.556426063	2.98093353	0.002874	0.007065
ENSG00000226674	TEX41	2.057394325	0	4.114788649	4.63839339	1.877098061	2.47104479	0.013472	0.029455
ENSG00000011465	DCN	30.71785971	2.3789872	59.05673225	4.631859763	0.671227029	6.90058588	5.18E-12	2.81E-11
ENSG00000163017	ACTG2	2.037624306	0	4.075248611	4.626750531	1.904274225	2.4296661	0.015113	0.032665
ENSG00000104415	CCN4	2.028447381	0	4.056894762	4.621252436	1.860043866	2.48448573	0.012974	0.028469
ENSG00000259939	AC022167.1	2.028447381	0	4.056894762	4.621252436	1.860043866	2.48448573	0.012974	0.028469
ENSG00000107518	ATRNL1	2.02633252	0	4.05266504	4.620132396	1.919458721	2.40699753	0.016084	0.034579
ENSG00000183778	B3GALT5	2.02491635	0	4.049832699	4.619146514	1.874359586	2.46438653	0.013725	0.029943
ENSG00000251348	HSPD1P11	7.649660353	0.6078191	14.69150158	4.61758938	1.244776027	3.70957448	0.000208	0.000599
ENSG00000280604	AJ239328.1	2.015739425	0	4.031478851	4.613747719	1.862144524	2.47765287	0.013225	0.028959
ENSG00000183979	NPB	2.008677362	0	4.017354725	4.609441836	1.885304708	2.44493201	0.014488	0.031443
ENSG00000147481	SNTG1	1.995969406	0	3.991938813	4.601826964	1.887491351	2.43806519	0.014766	0.031998
ENSG00000250551	MIR583HG	1.990323513	0	3.980647027	4.598330876	1.915713144	2.40032329	0.016381	0.035172
ENSG00000225506	CYP4A22-AS1	4.006423316	0.2908372	7.722009402	4.588846132	1.55222428	2.95630354	0.003114	0.007599
ENSG00000076716	GPC4	3.981706096	0.2908372	7.672574961	4.581058153	1.576549748	2.90574919	0.003664	0.008854
ENSG00000102524	TNFSF13B	55.83504183	4.5108096	107.159274	4.580250604	0.459573542	9.96630611	2.14E-23	1.89E-22
ENSG00000230521	AL645929.1	7.416684183	0.6056649	14.22770344	4.573807986	1.261177578	3.62661695	0.000287	0.000816
ENSG00000130477	UNC13A	29.49628417	2.3703704	56.62219797	4.568858715	0.639347777	7.14612435	8.93E-13	5.03E-12
ENSG00000163131	CTSS	6815.357258	554.09204	13076.62247	4.561538746	0.114931791	39.6890947	0	0
ENSG00000151651	ADAM8	76.60520241	6.2819777	146.9284271	4.551576281	0.411716667	11.0551179	2.07E-28	2.14E-27
ENSG00000124145	SDC4	12238.57027	1003.7376	23473.40291	4.548503379	0.04695615	96.8670419	0	0
ENSG00000145936	KCNMB1	61.57789775	4.972826	118.1829695	4.54777205	0.447717082	10.1576916	3.06E-24	2.76E-23
ENSG00000164099	PRSS12	18.14661409	1.4760224	34.81720577	4.546732475	0.82774494	5.49291485	3.95E-08	1.64E-07
ENSG00000122254	HS3ST2	14.40871734	1.1547321	27.66270258	4.544166473	0.915341772	4.96444783	6.89E-07	2.58E-06
ENSG00000167034	NKX3-1	575.4101737	47.116276	1103.704072	4.542481336	0.14920971	30.4436041	1.46E-203	9.91E-202
ENSG00000135378	PRRG4	53.9347366	4.4585203	103.4109529	4.530584672	0.473298364	9.57236496	1.04E-21	8.66E-21
ENSG00000163235	TGFA	382.6193977	31.843606	733.3951894	4.523647846	0.187702699	24.1000682	2.50E-128	1.06E-126
ENSG00000144837	PLA1A	174.055153	14.421406	333.6889	4.517000292	0.26804889	16.8514046	1.02E-63	2.14E-62
ENSG00000146216	TTBK1	10.54848425	0.8682033	20.22876522	4.504261738	1.076846467	4.18282631	2.88E-05	9.21E-05
ENSG00000137573	SULF1	4524.88553	382.92578	8666.845277	4.501932353	0.065991645	68.2197323	0	0
ENSG00000168398	BDKRB2	6.985698335	0.5795203	13.39187641	4.501040234	1.296124075	3.47269241	0.000515	0.001415
ENSG00000229922	LINC02528	1.867150156	0	3.734300312	4.498784967	1.923872985	2.3384002	0.019366	0.040952

ENSG00000185761	ADAMTSL5	10.56968021	0.8986564	20.24070405	4.497979431	1.035530153	4.34364892	1.40E-05	4.63E-05
ENSG00000237604	AP001056.1	1.860088093	0	3.720176186	4.494273102	1.952945187	2.30127969	0.021376	0.044708
ENSG00000073282	TP63	1.8544422	0	3.708884401	4.490555991	1.921424361	2.33709746	0.019434	0.041067
ENSG00000101187	SLCO4A1	1199.684238	102.01991	2297.348562	4.489900513	0.11150298	40.2670897	0	0
ENSG00000144868	TMEM108	17.41843567	1.4738682	33.36300314	4.484902511	0.812723289	5.51836347	3.42E-08	1.43E-07
ENSG00000274460	AC092119.2	1.841734244	0	3.683468489	4.482314083	1.928997526	2.32364947	0.020144	0.042387
ENSG00000119917	IFIT3	3667.388106	314.61047	7020.165747	4.480720059	0.234575428	19.1014042	2.46E-81	6.59E-80
ENSG00000178115	GOLGA8Q	1.834672181	0	3.669344363	4.477651214	1.916610895	2.33623383	0.019479	0.041134
ENSG00000108821	COL1A1	121.422629	10.412745	232.432513	4.477542988	0.323852847	13.8258565	1.78E-43	2.65E-42
ENSG00000280453	AP006248.5	1.821964225	0	3.643928451	4.469332443	1.928066898	2.31803806	0.020447	0.042966
ENSG00000254987	AP002989.1	1.818433194	0	3.636866388	4.466961143	1.9338161	2.30992034	0.020893	0.043809
ENSG00000125726	CD70	1.812787301	0	3.625574602	4.463130284	1.970934327	2.26447438	0.023545	0.048777
ENSG00000161896	IP6K3	1.802194206	0	3.604388413	4.455959053	1.973246516	2.25818671	0.023934	0.049507
ENSG00000285799	AL645929.3	1.802194206	0	3.604388413	4.455959053	1.973246516	2.25818671	0.023934	0.049507
ENSG00000203814	HIST2H2BF	3.670742499	0.288683	7.052801973	4.455693928	1.611154778	2.76552817	0.005683	0.013284
ENSG00000236452	AC123023.1	77.77334189	6.724312	148.8223718	4.446643637	0.393898013	11.2888197	1.49E-29	1.58E-28
ENSG00000145649	GZMA	44.02967548	3.8288649	84.23048604	4.44489795	0.512290844	8.67651258	4.08E-18	2.94E-17
ENSG00000138792	ENPEP	3.592405213	0.3169819	6.867828527	4.423945511	1.613045495	2.7426043	0.006095	0.014161
ENSG00000172183	ISG20	1061.561795	95.402363	2027.721227	4.402745903	0.111032947	39.6526077	0	0
ENSG00000135919	SERPINE2	11023.85643	1002.1637	21045.54911	4.392611261	0.117862579	37.2689219	5.23E-304	6.22E-302
ENSG00000177989	ODF3B	120.8551469	11.11868	230.5916136	4.381123735	0.319418288	13.7159452	8.15E-43	1.20E-41
ENSG00000137959	IFI44L	5216.269068	483.23601	9949.302122	4.363904655	0.264443842	16.5021981	3.54E-61	7.04E-60
ENSG00000163431	LMOD1	12.70360244	1.1590405	24.24816437	4.354474478	0.931949234	4.67243742	2.98E-06	1.05E-05
ENSG00000233098	CCDC144NL-A	22.07928595	2.1404393	42.01813262	4.323896766	0.706237375	6.12244115	9.22E-10	4.30E-09
ENSG00000185215	TNFAIP2	7453.204669	708.54125	14197.86808	4.32381091	0.051734427	83.5770528	0	0
ENSG00000164746	C7orf57	9.367828035	0.8965022	17.83915392	4.319263404	1.065003571	4.05563279	5.00E-05	0.000156
ENSG00000227496	AC099066.2	9.344203465	0.8965022	17.79190478	4.310663336	1.056714574	4.07930717	4.52E-05	0.000142
ENSG00000136514	RTP4	142.0010035	13.727599	270.2744085	4.299788481	0.274006621	15.6922795	1.71E-55	3.11E-54
ENSG00000180914	OXTR	34.16390057	3.3757596	64.95204154	4.294289433	0.579739077	7.40727959	1.29E-13	7.55E-13
ENSG00000143226	FCGR2A	6.075753982	0.5773661	11.57414191	4.293561719	1.35791595	3.1618759	0.001568	0.004008
ENSG00000010030	ETV7	106.5764477	10.380138	202.7727577	4.280913517	0.316643103	13.5196803	1.20E-41	1.71E-40
ENSG00000269902	AC234772.2	3.232360276	0.288683	6.176037526	4.269679927	1.629290492	2.62057622	0.008778	0.019849
ENSG00000221869	CEBPD	876.5364992	86.465317	1666.607681	4.26793182	0.126248512	33.8057989	1.62E-250	1.46E-248
ENSG00000215196	BASP1-AS1	3.223183351	0.288683	6.157683677	4.266058954	1.646246192	2.59138577	0.009559	0.021478
ENSG00000240990	HOXA11-AS	3.21861456	0.2908372	6.146391891	4.263534113	1.690060719	2.52271061	0.011645	0.025725
ENSG00000227706	AL713998.1	3.211552498	0.2908372	6.132267765	4.2608236	1.679982656	2.53623071	0.011205	0.024844
ENSG00000138755	CXCL9	5.903164971	0.5773661	11.22896389	4.249544691	1.352139546	3.14282997	0.001673	0.004257
ENSG00000184937	WT1	8.971840261	0.9226468	17.0210337	4.237551752	1.078456822	3.92927345	8.52E-05	0.000258
ENSG00000259863	SH3RF3-AS1	61.10696287	6.1623196	116.0516062	4.218757974	0.42557863	9.91299299	3.66E-23	3.20E-22
ENSG00000251136	AF117829.1	222.6610007	22.827358	422.4946438	4.21660297	0.229449052	18.3770773	2.00E-75	4.97E-74
ENSG00000168811	IL12A	133.093491	13.712225	252.474757	4.201622145	0.27722387	15.1560619	6.91E-52	1.19E-50
ENSG00000149591	TAGLN	8540.688627	883.8626	16197.51465	4.196431043	0.066722541	62.8937535	0	0
ENSG00000205057	CLLU1OS	20.14928494	2.1099862	38.18858368	4.189961213	0.700170174	5.98420408	2.17E-09	9.89E-09
ENSG00000277007	AC096642.1	3.030485254	0.2908372	5.770133277	4.173140793	1.697941316	2.45776503	0.01398	0.030429
ENSG00000184838	PRR16	150.3421296	15.817903	284.8663566	4.171002683	0.26317096	15.8490233	1.43E-56	2.65E-55
ENSG00000135114	OASL	325.08572	34.096767	616.0746734	4.169100261	0.181897519	22.9200501	2.93E-116	1.12E-114
ENSG00000162772	ATF3	457.6460284	48.319463	866.972594	4.165063152	0.155176736	26.8407705	1.08E-158	5.49E-157
ENSG00000204291	COL15A1	8.391541142	0.8682033	15.914879	4.161293895	1.09683664	3.79390489	0.000148	0.000436
ENSG00000165617	DACT1	19.56903645	2.1121404	37.02593248	4.145265808	0.708124189	5.85386839	4.80E-09	2.13E-08
ENSG00000253831	ETV3L	2.978278118	0.3169819	5.639574337	4.129824776	1.68280602	2.45413002	0.014123	0.030699
ENSG00000100290	BIK	5.47048353	0.5795203	10.36144681	4.129365604	1.425862174	2.89604822	0.003779	0.00912
ENSG00000272549	LINC02538	2.950382966	0.2908372	5.609928702	4.124444442	1.796728652	2.29552996	0.021703	0.045311
ENSG00000273151	AC073957.3	2.959924269	0.3169819	5.602866639	4.122298238	1.710549873	2.40992578	0.015956	0.034339
ENSG00000088386	SLC15A1	2.936597908	0.288683	5.58451279	4.118825976	1.800704908	2.28734089	0.022176	0.046206
ENSG00000138061	CYP1B1	5.509670468	0.6056649	10.41367601	4.117430927	1.330922557	3.0936668	0.001977	0.00498
ENSG00000198133	TMEM229B	35.46558891	3.9072989	67.0244789	4.109669267	0.528627675	7.77422269	7.59E-15	4.73E-14
ENSG00000128641	MYO1B	8985.98338	985.27824	16986.68852	4.108824751	0.062338182	65.9118472	0	0
ENSG00000177508	IRX3	8.240887633	0.924801	15.55697424	4.106247498	1.08899043	3.770692	0.000163	0.000475
ENSG00000118503	TNFAIP3	4128.002357	458.3561	7797.648611	4.08763464	0.058917136	69.3793841	0	0
ENSG00000007306	CEACAM7	2.861048863	0.288683	5.433414701	4.085604552	1.68636085	2.42273446	0.015404	0.033247
ENSG00000133106	EPSTI1	1213.933423	138.04301	2289.823839	4.055723529	0.097821884	41.4602885	0	0
ENSG00000270210	AC104695.3	5.278848502	0.6078191	9.949877876	4.052855683	1.331134045	3.04466383	0.002329	0.005794
ENSG00000243806	RPL7P18	5.178611817	0.5816745	9.775549175	4.039613091	1.410825337	2.8632978	0.004193	0.010037
ENSG00000234883	MIR155HG	152.969035	17.666413	288.2716568	4.035220712	0.535450146	7.53612776	4.84E-14	2.89E-13
ENSG00000166016	ABTB2	51.33028421	5.8388751	96.82277454	4.030319451	0.485823418	8.29585257	1.08E-16	7.36E-16
ENSG00000272425	AC009902.3	2.762618037	0.3169819	5.208254176	4.017645598	1.702759124	2.35949145	0.0183	0.038908
ENSG00000254732	AP001931.1	2.744937569	0.288683	5.201192113	4.015942343	1.689624799	2.37682493	0.017462	0.037295
ENSG00000261239	ANKRD26P1	61.31742438	7.1634004	115.4714484	4.012104936	0.410610314	9.77107685	1.50E-22	1.28E-21
ENSG00000228702	AL645998.1	2.742848018	0.3169819	5.168714138	4.008547146	1.681324054	2.38416095	0.017118	0.036622
ENSG00000257831	AL136418.1	2.740733157	0.3169819	5.164484415	4.007768961	1.747803501	2.29303177	0.021846	0.045585
ENSG00000186684	CYP27C1	2.719521657	0.288683	5.150360289	4.004428907	1.696584824	2.36028806	0.018261	0.038854
ENSG00000188227	ZNF793	71.70300452	8.5466777	134.8593313	4.004005554	0.378911331	10.5671307	4.23E-26	4.08E-25
ENSG00000180616	SSTR2	5.03491486	0.5795203	9.490309464	4.003768935	1.351063913	2.96341934	0.003042	0.007446
ENSG00000171385	KCND3	2.699751638	0.288683	5.110820251	3.995368252	1.692794004	2.36022117	0.018264	0.038857
ENSG00000162366	PDZK1IP1	30.24732006	3.6078449	56.88679524	3.988919604	0.576335434	6.92117709	4.48E-12	2.44E-11
ENSG00000174939	ASPHD1	97.86688769	11.652374	184.0814016	3.984108504	0.310892677	12.8150606	1.35E-37	1.75E-36
ENSG00000176928	GCNT4	44.9557611	5.3027331	84.60878908	3.983857675	0.464716674	8.57265921	1.01E-17	7.19E-17
ENSG00000168685	IL7R	1315.87336	160.35685	2471.389872	3.944577587	0.252518123	15.6209683	5.24E-55	9.44E-54
ENSG00000229656	ITGB1-DT	135.2082619	16.635971	253.7805531	3.927580343	0.264022226	14.8759459	4.72E-50	7.94E-49
ENSG00000163347	CLDN1	939.4118619	116.02053	1762.803197	3.922775172	0.103447351	37.9204989	0	0
ENSG00000131797	CLUHP3	14.41056906	1.7973127	27.0238254	3.916127931	0.825035079	4.74661991	2.07E-06	7.42E-06
ENSG00000273812	BX640514.2	76.61719622	9.5989853	143.6354071	3.908931749	0.343139711	11.3916624	4.60E-30	4.95E-29
ENSG00000134198	TSPAN2	11.97836263	1.5043213	22.45240398	3.904326157	0.903834092	4.31973765	1.56E-05	5.14E-05
ENSG00000110203	FOLR3	4.707373208	0.5795203	8.835226161	3.899491684	1.398712786	2.78791452	0.005305	0.012462
ENSG00000198535	C2CD4A	346.1538011	43.839106	648.4684958	3.886450429	0.168790601	23.0252775	2.60E-117	1.00E-115

ENSG00000183876	ARSI	7.144230142	0.9226468	13.36581346	3.886152645	1.127712302	3.4460497	0.000569	0.001552
ENSG00000286062	AC092353.1	198.6495936	25.262174	372.0370131	3.876644052	0.214808458	18.0469805	8.33E-73	2.01E-71
ENSG00000143847	PPFIA4	4.617001346	0.5795203	8.654482437	3.875433223	1.423809901	2.72187546	0.006491	0.015016
ENSG00000169403	PTAFR	41.94367131	5.3267236	78.56061903	3.873224637	0.460704246	8.40718242	4.20E-17	2.91E-16
ENSG00000011347	SYT7	16.32711683	2.0859957	30.56823792	3.87171424	0.741812295	5.21926404	1.80E-07	7.05E-07
ENSG00000105855	ITGB8	6989.731215	895.66127	13083.80116	3.867540033	0.061010895	63.3909733	0	0
ENSG00000276600	RAB7B	28.21190533	3.6927415	52.73106917	3.866881453	0.617007202	6.26715773	3.68E-10	1.77E-09
ENSG00000110042	DTX4	473.7789049	61.175612	886.3821975	3.862141568	0.142953232	27.0168188	9.38E-161	4.87E-159
ENSG00000163874	ZC3H12A	1638.644297	210.72587	3066.562728	3.858969484	0.087377299	44.1644399	0	0
ENSG00000164114	MAP9	30.39463125	4.0118776	56.77738491	3.855797473	0.584359372	6.59833256	4.16E-11	2.14E-10
ENSG00000101670	LIPG	16563.88328	2142.4107	30985.35588	3.853731662	0.04825097	79.8684807	0	0
ENSG00000013293	SLC7A14	935.9752671	123.04937	1748.901165	3.827857442	0.103513323	36.9793697	2.46E-299	2.87E-297
ENSG00000204642	HLA-F	1429.313773	188.34418	2670.283366	3.827568607	0.086056317	44.4774856	0	0
ENSG00000119714	GPR68	706.3230727	93.463707	1319.182439	3.819967873	0.116159129	32.885645	3.53E-237	2.89E-235
ENSG00000134716	CYP2J2	6.683573349	0.8682033	12.49894342	3.816873385	1.169013394	3.2650382	0.001094	0.00287
ENSG00000183570	PCBP3	11.29785262	1.530466	21.06523929	3.807996891	0.891220907	4.27278676	1.93E-05	6.28E-05
ENSG00000151023	ENKUR	96.16518734	12.800643	179.5297314	3.807777419	0.307002907	12.4030663	2.52E-35	3.09E-34
ENSG00000066248	NGEF	8.935827253	1.1873394	16.68431512	3.805434553	0.998052676	3.81285943	0.000137	0.000406
ENSG00000174938	SEZ6L2	694.7181534	92.717639	1296.718668	3.802201646	0.12005285	31.6710653	3.89E-220	2.87E-218
ENSG00000185745	IFIT1	2221.948963	297.72969	4146.168241	3.800099644	0.180085617	21.1016277	7.68E-99	2.48E-97
ENSG00000138696	BMPR1B	64.4323414	8.6806469	120.1840359	3.79625684	0.382206068	9.93248709	3.01E-23	2.64E-22
ENSG00000229647	MYOSLID	9.085888068	1.2679276	16.90384854	3.796222171	1.022788483	3.71163954	0.000206	0.000594
ENSG00000185338	SOCS1	181.3842818	24.450095	338.3184689	3.787029786	0.5043604	7.50857877	5.98E-14	3.55E-13
ENSG00000258875	AL135818.1	13.14123911	1.7625512	24.519927	3.783842338	0.826078768	4.58048613	4.64E-06	1.61E-05
ENSG00000142661	MYOM3	107.9583725	14.667479	201.2492661	3.782230591	0.284186344	13.3089808	2.05E-40	2.84E-39
ENSG00000171791	BCL2	341.5998933	46.401695	636.7980919	3.781492133	0.187981107	20.1163414	5.31E-90	1.57E-88
ENSG00000235947	EGOT	26.02024563	3.4989578	48.54153346	3.771245986	0.588788564	6.40509381	1.50E-10	7.42E-10
ENSG00000176907	TCIM	5140.780606	704.89489	9576.66632	3.76450172	0.213417511	17.6391417	1.23E-69	2.82E-68
ENSG00000172164	SNTB1	56.78634367	7.8930319	105.6796555	3.76067311	0.404237517	9.30312736	1.36E-20	1.09E-19
ENSG00000189056	RELN	15444.50323	2125.8425	28763.16398	3.758764324	0.059668556	62.9940555	0	0
ENSG00000104312	RIPK2	6903.769419	953.54452	12853.99431	3.753017355	0.054472529	68.8974321	0	0
ENSG00000154721	JAM2	37.01511016	5.2253617	68.80485866	3.747823291	0.503191372	7.44810722	9.47E-14	5.57E-13
ENSG00000158315	RHBDL2	49.68835703	6.9116331	92.46508098	3.74781074	0.421172925	8.89850824	5.66E-19	4.25E-18
ENSG00000115008	IL1A	422.6399159	58.970756	786.3090759	3.735607487	0.157374215	23.7371	1.49E-124	6.18E-123
ENSG00000237989	LINC01679	32.10466004	4.5086554	59.70066464	3.734312028	0.511182824	7.30523768	2.77E-13	1.60E-12
ENSG00000103044	HAS3	1034.611709	145.05172	1924.171697	3.727926125	0.101086496	36.8785769	1.02E-297	1.15E-295
ENSG00000232702	AL158050.1	6.314037731	0.8703575	11.75771798	3.727038519	1.150912461	3.23833362	0.001202	0.003139
ENSG00000164251	F2RL1	8498.305008	1198.385	15798.22498	3.720681165	0.049612147	74.9953661	0	0
ENSG00000224594	RPL29P19	18.59446569	2.6153809	34.57355052	3.702999739	0.742652944	4.98617796	6.16E-07	2.32E-06
ENSG00000148154	UGCG	6667.18363	953.71877	12380.64849	3.699951078	0.063937815	57.8679627	0	0
ENSG00000177335	C8orf31	8.302529727	1.1851852	15.42000028	3.695063229	0.992531335	3.72286808	0.000197	0.00057
ENSG00000102096	PIM2	881.932157	126.43082	1637.433491	3.692400747	0.103585789	35.6458234	2.73E-278	2.87E-276
ENSG00000165449	SLC16A9	8.135285231	1.1590405	15.11152995	3.674071478	1.006872553	3.64899358	0.000263	0.000752
ENSG00000156966	B3GNT7	180.5145383	26.21126	334.8178168	3.672989678	0.232214773	15.8172093	2.37E-56	4.36E-55
ENSG00000110446	SLC15A3	1193.185718	174.46063	2211.91081	3.662670195	0.097029056	37.7481792	0	0
ENSG00000126709	IFI6	16427.1474	2410.5135	30443.78127	3.658705835	0.175569937	20.8390223	1.92E-96	6.09E-95
ENSG00000233930	KRTAP5-AS1	48.2722835	7.1043542	89.44021275	3.647532692	0.475528385	7.67048363	1.71E-14	1.05E-13
ENSG00000168280	KIF5C	6.110398193	0.9226468	11.29814956	3.64723172	1.168802648	3.1204855	0.001806	0.004571
ENSG00000255750	AC022509.1	5.960042408	0.8725117	11.04757313	3.636786948	1.195863988	3.04113761	0.002357	0.005858
ENSG00000028277	POU2F2	3416.54718	510.45943	6322.634928	3.628811055	0.067035697	54.1325175	0	0
ENSG00000169989	TIGD4	5.899994317	0.8682033	10.93178535	3.626179318	1.17969135	3.07383734	0.002113	0.005296
ENSG00000128271	ADORA2A	97.82055453	14.8548	180.786309	3.619550288	0.320913899	11.2788829	1.67E-29	1.77E-28
ENSG00000039139	DNAH5	106.1297939	16.061851	196.1977371	3.605527692	0.668412201	5.39416798	6.88E-08	2.80E-07
ENSG00000232977	LINC00327	9.649045277	1.4434151	17.85467543	3.600467954	0.977686198	3.68264169	0.000231	0.000663
ENSG00000090339	ICAM1	36628.47113	5604.3292	67652.61308	3.593690906	0.046607026	77.1062053	0	0
ENSG00000221963	APOL6	1610.595853	248.99259	2972.199116	3.578194947	0.079256915	45.1467856	0	0
ENSG00000157601	MX1	18313.66721	2833.1777	33794.15674	3.576161058	0.056828009	62.9295504	0	0
ENSG00000135454	B4GALNT1	7.701512656	1.183031	14.21999434	3.575108666	1.062129358	3.36598234	0.000763	0.002043
ENSG00000174348	PODN	585.0974147	91.202223	1078.992606	3.566523237	0.125240942	28.4772948	2.24E-178	1.31E-176
ENSG00000162692	VCAM1	6219.555889	969.7633	11469.34848	3.564615843	0.052329715	68.1183884	0	0
ENSG00000162877	PM20D1	11.33273491	1.7428692	20.92260065	3.560539745	0.87320083	4.07757256	4.55E-05	0.000143
ENSG00000176406	RIMS2	3.845472436	0.6056649	7.085279948	3.559369162	1.468337742	2.42408069	0.015347	0.033131
ENSG00000133805	AMPD3	1009.755862	158.48329	1861.028432	3.552603004	0.106638611	33.3144157	2.39E-243	2.07E-241
ENSG00000213949	ITGA1	1617.221656	254.96157	2979.48174	3.548725487	0.092506037	38.3620961	0	0
ENSG00000144355	DLX1	3.788994719	0.6056649	6.972324514	3.540908592	1.437625932	2.46302499	0.013777	0.030042
ENSG00000153714	LURAP1L	477.6116964	75.595956	879.6274371	3.538243268	0.13311802	26.5797467	1.16E-155	5.83E-154
ENSG00000133657	ATP13A3	29516.71456	4698.6752	54334.75389	3.531271876	0.047607125	74.1752814	0	0
ENSG00000243130	PSG11	3.785488998	0.6339638	6.9370142	3.51166844	1.47393727	2.38250875	0.017195	0.036774
ENSG00000140839	CLEC18B	9.381687293	1.5587648	17.20460976	3.500510219	0.970712399	3.60612497	0.000311	0.000878
ENSG00000171951	SCG2	3.629787451	0.5773661	6.682208039	3.499470307	1.520526701	2.3014856	0.021364	0.044688
ENSG00000151883	PARP8	52.32089856	8.4714896	96.17030755	3.498662565	0.824155407	4.24514908	2.18E-05	7.07E-05
ENSG00000074964	ARHGEF10L	78.54283724	12.798195	144.2874796	3.493795368	0.338630512	10.3174263	5.88E-25	5.44E-24
ENSG00000108576	SLC6A4	119.0984252	19.569396	218.6274542	3.491275128	0.262394572	13.3054396	2.15E-40	2.98E-39
ENSG00000229563	LINC01204	14.55664453	2.3703704	26.74291871	3.486983493	0.750008501	4.64925863	3.33E-06	1.17E-05
ENSG00000198400	NTRK1	3.59274028	0.5795203	6.605960304	3.483550237	1.507802621	2.31034897	0.020869	0.043769
ENSG00000064309	CDON	543.200642	89.454278	996.9470065	3.477663995	0.125007573	27.8196266	2.51E-170	1.43E-168
ENSG00000107242	PIP5K1B	7.231390492	1.1873394	13.2754416	3.477170077	1.024331799	3.39457398	0.000687	0.001853
ENSG00000117228	GBP1	5752.899611	951.87658	10553.92265	3.472232059	0.054835335	63.3210696	0	0
ENSG00000259471	LINC01169	3.563454268	0.5816745	6.545234077	3.470571149	1.50151099	2.31138578	0.020812	0.043659
ENSG00000112299	VNN1	48.77173779	8.133734	89.40974162	3.468304531	0.418392656	8.28959227	1.14E-16	7.75E-16
ENSG00000076864	RAP1GAP	3.544722009	0.5795203	6.509923762	3.46597974	1.502605291	2.30664684	0.021075	0.044136
ENSG00000260231	KDM7A-DT	87.8195053	14.519522	161.1194886	3.465504149	0.299401051	11.5747895	5.53E-31	6.06E-30
ENSG00000258183	LINC02392	9.070343771	1.5326202	16.60806739	3.462913267	0.93872343	3.68896009	0.000225	0.000648
ENSG00000259518	LINC01583	5.438377211	0.9226468	9.954107599	3.460471727	1.199237984	2.88555881	0.003907	0.009404
ENSG00000261488	TBILA	7.212363262	1.2156383	13.20908827	3.460038171	1.059222087	3.26658423	0.001089	0.002856

ENSG00000187595	ZNF385C	7.359941598	1.2679276	13.4519556	3.456947497	1.10785011	3.12041084	0.001806	0.004571
ENSG00000108309	RUNDC3A	3.590272318	0.6056649	6.574879712	3.456922921	1.472826011	2.34713598	0.018918	0.040099
ENSG00000183691	NOG	67.56241787	11.289565	123.8352706	3.450281989	0.349792588	9.86379387	5.97E-23	5.17E-22
ENSG00000166394	CYB5R2	278.5465135	46.527844	510.5651826	3.449628225	0.173333881	19.9016384	3.94E-88	1.13E-86
ENSG00000119900	OGFRL1	10223.1521	1730.9605	18715.34372	3.435147886	0.055847498	61.5094321	0	0
ENSG00000224429	LINC00539	5.272107474	0.8943479	9.649866998	3.428647967	1.229017103	2.78974797	0.005275	0.012394
ENSG00000129451	KLK10	24.57572488	4.1981362	44.9533136	3.426964046	0.577414817	5.93501231	2.94E-09	1.33E-08
ENSG00000254143	AC022733.2	15.44180625	2.606764	28.27684846	3.41250432	0.732980029	4.65565798	3.23E-06	1.14E-05
ENSG00000156920	ADGRG4	274.7274805	47.410804	502.0441572	3.40917532	0.179593256	18.982758	2.37E-80	6.23E-79
ENSG00000231133	HAR1B	5.158812973	0.8965022	9.421123791	3.399270786	1.223330345	2.77870225	0.005458	0.0128
ENSG00000183914	DNAH2	8.589215028	1.4760224	15.70240765	3.397425246	0.974055192	3.48791863	0.000487	0.001341
ENSG00000162078	ZG16B	6.86748021	1.1851852	12.54977524	3.397075017	1.060187737	3.20422025	0.001354	0.003503
ENSG00000183775	KCTD16	17.33590177	3.0609319	31.61087163	3.393146503	0.667177904	5.08581966	3.66E-07	1.41E-06
ENSG00000125347	IRF1	3469.918053	606.67219	6333.163914	3.384406736	0.058894658	57.4654279	0	0
ENSG00000068079	IFI35	2249.694652	393.91604	4105.473265	3.381312218	0.070519891	47.9483472	0	0
ENSG00000232082	RPS6KA2-IT1	115.164166	20.378553	209.9497787	3.368787797	0.283605145	11.8784439	1.53E-32	1.76E-31
ENSG00000164683	HEY1	183.339601	32.451719	334.2274828	3.362648418	0.202649529	16.5934184	7.78E-62	1.57E-60
ENSG00000154654	NCAM2	36.67009835	6.4574652	66.88273147	3.356049095	0.483115583	6.94667945	3.74E-12	2.05E-11
ENSG00000103241	FOXF1	6.6232075	1.1590405	12.08737449	3.355449603	1.061111675	3.16220213	0.001566	0.004004
ENSG00000150594	ADRA2A	5.024008762	0.8986564	9.149361165	3.353151128	1.221811227	2.74441015	0.006062	0.014092
ENSG00000125637	PSD4	28.20440551	5.0097417	51.39906933	3.347665351	0.515056517	6.49960779	8.05E-11	4.05E-10
ENSG00000058085	LAMC2	16972.91522	3039.8887	30905.94172	3.346091925	0.040261876	83.1081961	0	0
ENSG00000152784	PRDM8	77.60133242	13.833269	141.369396	3.340533892	0.318154125	10.4997347	8.66E-26	8.25E-25
ENSG00000276116	FUT8-AS1	21.22636993	3.7636504	38.68908951	3.334476773	0.623530658	5.34773508	8.91E-08	3.59E-07
ENSG00000247095	MIR210HG	78.67081121	14.331109	143.0105132	3.3209555	0.326926117	10.1581224	3.05E-24	2.75E-23
ENSG00000146374	RSPO3	689.9039892	127.20705	1252.600929	3.300092722	0.118672447	27.8084157	3.43E-170	1.94E-168
ENSG00000280143	AP000892.3	155.2226293	28.818792	281.6264664	3.293042337	0.233365955	14.1110657	3.25E-45	4.99E-44
ENSG00000092969	TGFB2	3233.232738	599.80692	5866.658558	3.289481143	0.058055121	56.6613435	0	0
ENSG00000261644	AC007728.2	12.54648959	2.3399173	22.75306189	3.264820765	0.803225612	4.06463728	4.81E-05	0.00015
ENSG00000279805	Z95114.3	12.8000545	2.455267	23.14484202	3.263096048	0.768995787	4.2433211	2.20E-05	7.13E-05
ENSG00000138642	HERC6	2244.72598	423.85882	4065.593144	3.261809124	0.066772934	48.8492709	0	0
ENSG00000248458	AL139147.1	10.97485621	2.0598511	19.88986135	3.258370767	0.83004564	3.92553205	8.65E-05	0.000262
ENSG00000167244	IGF2	34.6455501	6.5641981	62.7269021	3.257330073	0.4636561	7.02531483	2.14E-12	1.18E-11
ENSG00000213088	ACKR1	28.17828842	5.2962705	51.06030633	3.25493827	0.518022716	6.28338906	3.31E-10	1.60E-09
ENSG00000184347	SLIT3	4.638547913	0.8703575	8.40673834	3.247380348	1.254078963	2.58945445	0.009613	0.021583
ENSG00000096696	DSP	9.518252504	1.8496021	17.18690295	3.247048529	0.898078324	3.61555161	0.0003	0.000849
ENSG00000157368	IL34	34.47013528	6.592497	62.34777357	3.246229003	0.471447959	6.88565713	5.75E-12	3.11E-11
ENSG00000226835	AC097059.1	13.54674691	2.6046098	24.48888398	3.208032948	0.764096347	4.19846654	2.69E-05	8.63E-05
ENSG00000159167	STC1	1088.056563	213.03852	1963.074608	3.202977016	0.107871883	29.6924177	9.62E-194	6.09E-192
ENSG00000157214	STEAP2	559.4333845	109.75918	1009.10759	3.202541625	0.135814806	23.5802098	6.15E-123	2.51E-121
ENSG00000133169	BEX1	4.47832779	0.8725117	8.08414389	3.189553855	1.311305303	2.43235031	0.015001	0.032448
ENSG00000121577	POPDC2	10.52271424	2.0859957	18.95943274	3.185439609	0.834844995	3.81560604	0.000136	0.000401
ENSG00000259436	AC010247.2	16.39451119	3.2210458	29.5679766	3.180966375	0.690120091	4.60929397	4.04E-06	1.41E-05
ENSG00000206337	HCP5	63.1366323	12.581483	113.6917814	3.180305614	0.340421019	9.3422716	9.43E-21	7.58E-20
ENSG00000267769	AC011498.6	4.427456623	0.8682033	7.986709965	3.177453903	1.287147951	2.46860037	0.013564	0.029637
ENSG00000234745	HLA-B	56627.35532	11302.076	101952.6348	3.173375146	0.043654154	72.6935438	0	0
ENSG00000144218	AFF3	25.39317251	5.0946383	45.6917067	3.170559635	0.541661005	5.85340205	4.82E-09	2.14E-08
ENSG00000272574	AL596325.2	4.53092613	0.9226468	8.139205437	3.167376108	1.256743905	2.52030354	0.011725	0.025884
ENSG00000111817	DSE	6676.075278	1337.4677	12014.68284	3.166628533	0.047624803	66.4911632	0	0
ENSG00000128487	SPECC1	217.3214345	43.766337	390.8765316	3.162996676	0.185056658	17.0920448	1.70E-65	3.67E-64
ENSG00000107859	PITX3	7.444244297	1.5043213	13.38416731	3.158104377	0.982250873	3.21517085	0.001304	0.003381
ENSG00000104723	TUSC3	117.7988537	23.958099	211.6396082	3.147088878	0.25448116	12.3666871	3.96E-35	4.83E-34
ENSG00000083857	FAT1	69.07193195	14.022744	124.1211198	3.144884662	0.326222837	9.64029586	5.40E-22	4.52E-21
ENSG00000225978	HAR1A	4.351584058	0.8682033	7.834964836	3.142768984	1.299526765	2.41839496	0.015589	0.0336
ENSG00000080709	KCNN2	151.1521811	30.911251	271.3931115	3.140021873	0.226423127	13.867938	9.91E-44	1.48E-42
ENSG00000179542	SLITRK4	362.445207	74.876772	650.0136422	3.121445648	0.144382391	21.619296	1.18E-103	4.03E-102
ENSG00000121898	CPXM2	635.0275318	130.9841	1139.070964	3.120901212	0.246093662	12.6817618	7.46E-37	9.54E-36
ENSG00000132530	XAF1	3177.655886	662.24208	5693.069695	3.10500749	0.060298957	51.4935522	0	0
ENSG00000267577	AC010327.3	29.9253945	6.2493704	53.60141859	3.101719092	0.545226757	5.68886074	1.28E-08	5.50E-08
ENSG00000269981	AL627309.7	5.694973951	1.1894936	10.20045431	3.099883633	1.125121115	2.75515551	0.005866	0.013675
ENSG00000108691	CCL2	28912.52332	6050.9964	51774.05024	3.09704554	0.120170566	25.7720808	1.82E-146	8.74E-145
ENSG00000131409	LRRC4B	211.4901423	44.77559	378.2046945	3.06934305	0.214445062	14.3129575	1.82E-46	2.85E-45
ENSG00000186469	GNG2	48.49162028	10.260185	86.72305508	3.065230809	0.404760074	7.57295742	3.65E-14	2.19E-13
ENSG00000271897	AL357518.1	14.08053616	3.0304788	25.1305935	3.064501691	0.709432005	4.31965526	1.56E-05	5.14E-05
ENSG00000010818	HIVEP2	2181.518464	466.42507	3896.61186	3.063086885	0.067203319	45.5793988	0	0
ENSG00000080031	PTPRH	163.9110152	35.200746	292.6212844	3.062689506	0.234658126	13.0517087	6.21E-39	8.32E-38
ENSG00000164823	OSGIN2	5273.994773	1128.8294	9419.16017	3.0608507	0.054974235	55.6779138	0	0
ENSG00000173110	HSPA6	6.844913193	1.4541861	12.23564024	3.050066024	1.075119961	2.83695414	0.004555	0.010817
ENSG00000253102	AC004707.1	4.090738047	0.8682033	7.313272813	3.048435432	1.288144689	2.36653185	0.017956	0.038274
ENSG00000120549	KIAA1217	1328.699727	286.51918	2370.880273	3.048239827	0.09489669	32.1216664	2.20E-226	1.65E-224
ENSG00000177875	CCDC184	37.64514623	8.2601489	67.03014358	3.041327503	0.466218501	6.52339514	6.87E-11	3.48E-10
ENSG00000251194	AL133330.1	6.853716462	1.4760224	12.23141051	3.039067499	0.994745291	3.05512127	0.00225	0.005616
ENSG00000243649	CFB	35.77040951	7.8278173	63.71300173	3.033835871	0.454863458	6.66977269	2.56E-11	1.33E-10
ENSG00000180458	AC022148.1	9.752292494	2.166584	17.33800104	3.029639347	0.893796417	3.38963022	0.0007	0.001885
ENSG00000279296	PRAL	13.66864695	3.032633	24.30466088	3.017056615	0.702668597	4.29371204	1.76E-05	5.74E-05
ENSG00000152503	TRIM36	136.5922249	29.977064	243.2073852	3.016709998	0.25236617	11.953702	6.21E-33	7.18E-32
ENSG00000091128	LAMB4	37.97541351	8.4138002	67.53702686	3.010173581	0.435278542	6.91551108	4.66E-12	2.54E-11
ENSG00000127533	F2RL3	753.1681974	166.24483	1340.091562	3.008603772	0.106711653	28.1937698	6.97E-175	4.01E-173
ENSG00000187608	ISG15	5157.882795	1141.0492	9174.716406	3.007172482	0.162543979	18.5006698	2.04E-76	5.10E-75
ENSG00000072682	P4HA2	8741.183267	1936.2556	15546.11095	3.005469627	0.042842044	70.1523402	0	0
ENSG00000229950	TFAP2A-AS1	18.49845676	4.0892491	32.90766445	2.989620416	0.635016493	4.70794137	2.50E-06	8.92E-06
ENSG00000169583	CLIC3	37.27356848	8.3138241	66.23331285	2.988785409	0.50531316	5.91471911	3.32E-09	1.50E-08
ENSG00000115738	ID2	284.988293	63.709637	506.2669494	2.988071046	0.170454487	17.5300229	8.45E-69	1.92E-67
ENSG00000234678	ELF3-AS1	9.278883651	2.0838415	16.47392576	2.982348912	0.840166917	3.54971	0.000386	0.001076
ENSG00000113739	STC2	10432.30932	2348.1107	18516.50793	2.978565615	0.050862296	58.5613677	0	0

ENSG00000187957	DNER	1717.73733	387.40847	3048.066193	2.974743738	0.073774258	40.3222455	0	0
ENSG00000011201	ANOS1	121.7975539	27.56379	216.031318	2.973395807	0.246751732	12.0501517	1.94E-33	2.26E-32
ENSG00000225210	DUXAP9	5.451136542	1.2679276	9.634345489	2.972002379	1.192351837	2.49255487	0.012683	0.027873
ENSG00000213199	ASIC3	6.458739621	1.4498777	11.4676015	2.957016491	1.026463471	2.88078103	0.003967	0.009533
ENSG00000108370	RGS9	58.03933826	13.286062	102.7926149	2.949023298	0.737102849	4.00083015	6.31E-05	0.000194
ENSG00000181634	TNFSF15	1880.817261	435.43591	3326.198609	2.935375268	0.0772989	37.9743472	0	0
ENSG00000160606	TLCD1	110.4493732	25.550857	195.3478892	2.930825264	0.261842518	11.1930838	4.41E-29	4.62E-28
ENSG00000101605	MYOM1	11.55580064	2.6895065	20.42209482	2.928747544	0.755643963	3.87583001	0.000106	0.000318
ENSG00000126562	WNK4	747.3106112	173.22336	1321.397862	2.928154159	0.107296953	27.2901893	5.55E-164	2.98E-162
ENSG00000259321	AL136295.2	7.777079495	1.819149	13.73501001	2.928100703	0.924860101	3.1659931	0.001546	0.003957
ENSG00000227507	LTB	124.6158019	29.189449	220.0421544	2.913769307	0.257028214	11.3363792	8.67E-30	9.26E-29
ENSG00000182873	PRKCZ-AS1	5.005975947	1.1851852	8.826766715	2.890812453	1.131779085	2.55421971	0.010643	0.023691
ENSG00000178573	MAF	93.82593287	22.403643	165.2482229	2.884703416	0.287064044	10.048989	9.28E-24	8.28E-23
ENSG00000139364	TMEM132B	24.66630829	5.8256556	43.50696095	2.881488175	0.555383016	5.18829005	2.12E-07	8.29E-07
ENSG00000237181	AC147651.4	59.99939891	14.445396	105.5534014	2.860015603	0.35212028	8.12226892	4.58E-16	3.03E-15
ENSG00000103154	NECAB2	4.948107371	1.2134841	8.682730689	2.857957721	1.159026867	2.46582526	0.01367	0.029843
ENSG00000137193	PIM1	309.3110237	74.835103	543.7869445	2.854944447	0.163853311	17.4237825	5.45E-68	1.22E-66
ENSG00000186301	MST1P2	6.071553599	1.4760224	10.66708479	2.84404951	1.01507362	2.801816	0.005082	0.011976
ENSG00000147454	SLC25A37	2433.28813	596.76225	4269.81401	2.840122214	0.067473348	42.0925044	0	0
ENSG00000278884	CU634019.2	4.793104109	1.1568863	8.429321912	2.840086751	1.18537965	2.39593007	0.016578	0.035556
ENSG00000236393	AC091806.1	10.92405773	2.6938149	19.15430059	2.832294477	0.776245517	3.64870961	0.000264	0.000752
ENSG00000183762	KREMEN1	222.7612249	54.719268	390.803182	2.829853307	0.195177141	14.4988972	1.23E-47	1.98E-46
ENSG00000078053	AMPH	8.605501875	2.1644297	15.046574	2.82766812	0.885849121	3.19204259	0.001413	0.003638
ENSG00000145362	ANK2	12.28332423	3.0892308	21.47741769	2.826554913	0.781725862	3.6157879	0.000299	0.000849
ENSG00000145901	TNIP1	17993.7922	4445.8752	31541.70924	2.82653852	0.041305157	68.4306453	0	0
ENSG00000164530	PI16	4.866927981	1.2113298	8.522526114	2.825353921	1.165492543	2.4241716	0.015343	0.033127
ENSG00000188112	C6orf132	67.17136107	16.544612	117.7981106	2.824535924	0.319786335	8.8325723	1.02E-18	7.60E-18
ENSG00000226979	LTA	9.831664029	2.4792574	17.18407061	2.824321669	0.860409973	3.28253014	0.001029	0.002707
ENSG00000214694	ARHGEF33	7.063272791	1.7320982	12.39444743	2.817446034	1.073806597	2.62379281	0.008696	0.019677
ENSG00000141668	CBLN2	36.45007322	9.2089404	63.69120607	2.810645326	0.465317118	6.04027924	1.54E-09	7.07E-09
ENSG00000089127	OAS1	2594.480507	649.20179	4539.75922	2.807206048	0.066214024	42.3959442	0	0
ENSG00000261884	AC040162.1	7.183085282	1.8234574	12.54271318	2.79628127	0.979807201	2.8539097	0.004318	0.010305
ENSG00000140092	FBLN5	1881.874716	473.55161	3290.19782	2.795391341	0.069432574	40.260517	0	0
ENSG00000224743	TEX26-AS1	8.150057188	2.0272438	14.27287058	2.794193863	0.957517139	2.9181659	0.003521	0.008525
ENSG00000163898	LIPH	11.86042693	3.0086426	20.71221129	2.793602247	0.752408343	3.71288048	0.000205	0.000592
ENSG00000248323	LUCAT1	21.22931558	5.3528683	37.1057629	2.788243505	0.56254772	4.95645686	7.18E-07	2.69E-06
ENSG00000138735	PDE5A	622.7863822	157.64044	1087.932328	2.787345311	0.109601686	25.4315915	1.13E-142	5.26E-141
ENSG00000234948	LINC01524	4.635377259	1.1611947	8.109559802	2.780696371	1.195142746	2.32666464	0.019983	0.042066
ENSG00000162004	CCDC78	12.98918625	3.3191619	22.65921065	2.77778787	0.732177949	3.79386988	0.000148	0.000436
ENSG00000223776	LGALS8-AS1	5.788444297	1.4738682	10.10302039	2.772619458	1.088093842	2.54814369	0.01083	0.024074
ENSG00000134539	KLRD1	9.286684502	2.376833	16.19653603	2.765313089	0.893302579	3.0956063	0.001964	0.004949
ENSG00000262074	SNORD3B-2	5.748919806	1.471714	10.02612561	2.754955944	1.089223842	2.52928355	0.01143	0.025293
ENSG00000198732	SMOC1	24.22274655	6.2536788	42.19181428	2.753405648	0.514494434	5.35167238	8.71E-08	3.51E-07
ENSG00000275496	CU633906.1	5.732680819	1.471714	9.993647637	2.751057003	1.085749772	2.53378548	0.011284	0.025003
ENSG00000101335	MYL9	3417.827937	885.93373	5949.722146	2.747921224	0.070740395	38.8451494	0	0
ENSG00000197208	SLC22A4	1300.736624	337.42888	2264.044371	2.745237487	0.078645636	34.9064183	5.94E-267	5.81E-265
ENSG00000166444	ST5	988.7472463	257.39562	1720.098873	2.741325641	0.08672208	31.6104691	2.65E-219	1.94E-217
ENSG00000197046	SIGLEC15	14.90749503	3.9008363	25.91415375	2.734775379	0.679616173	4.02399985	5.72E-05	0.000177
ENSG00000260862	AC099506.1	5.615191183	1.4541861	9.776196216	2.734318415	1.18593961	2.30561353	0.021132	0.044252
ENSG00000156273	BACH1	2445.113007	639.71057	4250.515439	2.73382469	0.075095518	36.4046318	3.60E-290	4.00E-288
ENSG00000167578	RAB4B	5.722869857	1.5043213	9.94141843	2.730626082	1.118762096	2.4407567	0.014657	0.031772
ENSG00000130589	HELZ2	7640.229217	2005.6673	13274.79115	2.72632544	0.046220445	58.9852707	0	0
ENSG00000137825	ITPKA	14.70130032	3.8420844	25.56051628	2.724772723	0.682393335	3.99296503	6.53E-05	0.0002
ENSG00000130762	ARHGEF16	35.80495151	9.3658375	62.2440655	2.7184726	0.466057332	5.83291457	5.45E-09	2.41E-08
ENSG00000240065	PSMB9	747.542181	197.56392	1297.520441	2.715753855	0.098046551	27.698617	7.26E-169	4.02E-167
ENSG00000197054	ZNF763	8.968654088	2.3506883	15.58661987	2.715153772	0.887379272	3.05974442	0.002215	0.005535
ENSG00000164199	ADGRV1	7.969838245	2.1142946	13.82538187	2.714554353	0.941158152	2.88427014	0.003923	0.009439
ENSG00000204482	LST1	41.94730039	11.212194	72.68240708	2.709581051	0.398761995	6.79498319	1.08E-11	5.76E-11
ENSG00000240184	PCDHGC3	2682.243281	715.27023	4649.216328	2.700013149	0.061390209	43.9811689	0	0
ENSG00000271581	AL671883.2	14.36172258	3.7919492	24.93149593	2.699651175	0.675163676	3.99851365	6.37E-05	0.000196
ENSG00000197536	C5orf56	187.7687839	50.10925	325.4283173	2.694416974	0.196357457	13.7219997	7.50E-43	1.10E-41
ENSG00000279094	LINC01670	10.1835658	2.7700947	17.59703693	2.692952805	0.808074128	3.33255664	0.000861	0.002291
ENSG00000254887	AC010247.1	47.95774811	12.874475	83.04102155	2.692608359	0.363044545	7.41674375	1.20E-13	7.03E-13
ENSG00000230183	CNOT6LP1	22.40049287	6.0412756	38.75971014	2.690810242	0.534324417	5.03591106	4.76E-07	1.81E-06
ENSG00000137726	FXYD6	5.543214028	1.4803308	9.606097237	2.690660607	1.094868942	2.45751843	0.01399	0.030443
ENSG00000113946	CLDN16	25.28102959	6.7310688	43.83099036	2.689526725	0.560667278	4.79701033	1.61E-06	5.84E-06
ENSG00000019582	CD74	36.73047275	9.913813	63.54713247	2.68593338	0.438369183	6.12710356	8.95E-10	4.18E-09
ENSG00000235513	AL035681.1	36.56735387	9.8790515	63.25565619	2.681570692	0.430398201	6.23044122	4.65E-10	2.22E-09
ENSG00000228613	AC141930.1	5.479595562	1.471714	9.487477124	2.680396764	1.135247596	2.36106799	0.018222	0.038781
ENSG00000186827	TNFRSF4	107.9206847	29.104259	186.7371108	2.678166621	0.241048079	11.110508	1.12E-28	1.16E-27
ENSG00000138347	MYPN	33.11201431	9.047764	57.17626467	2.669708512	0.437491537	6.10230892	1.05E-09	4.86E-09
ENSG00000119508	NR4A3	18.36706855	5.0054333	31.72870382	2.656316732	0.607355252	4.37357992	1.22E-05	4.06E-05
ENSG00000137673	MMP7	16.3955655	4.5086554	28.28247556	2.655844157	0.610576901	4.34972917	1.36E-05	4.51E-05
ENSG00000153233	PTPRR	127.0834891	35.05816	219.1088183	2.653560391	0.248613371	10.673442	1.36E-26	1.33E-25
ENSG00000111331	OAS3	8156.694044	2242.7645	14070.6236	2.648984231	0.058331531	45.4125614	0	0
ENSG00000272419	LINC01145	63.90492899	17.610907	110.198951	2.646426408	0.311187483	8.50428296	1.83E-17	1.28E-16
ENSG00000146267	FAXC	45.55024335	12.583637	78.51684927	2.644329038	0.370173546	7.14348463	9.10E-13	5.12E-12
ENSG00000171533	MAP6	104.3545915	28.724216	179.9849668	2.642785583	0.255228764	10.3545758	3.99E-25	3.73E-24
ENSG00000159216	RUNX1	1652.374118	456.20554	2848.542696	2.640397242	0.081987295	32.204956	1.50E-227	1.15E-225
ENSG00000233817	AL162727.1	7.549445492	2.0881499	13.01074104	2.636541233	0.952167796	2.76898803	0.005623	0.01316
ENSG00000146457	WTAP	6173.565064	1717.9761	10629.15407	2.629316743	0.048218221	54.5295261	0	0
ENSG00000134532	SOX5	13.84217224	3.8420844	23.84226012	2.627074722	0.692368682	3.79432922	0.000148	0.000435
ENSG00000111335	OAS2	8296.89909	2312.358	14281.44017	2.626612573	0.0532447	49.3309678	0	0
ENSG00000107968	MAP3K8	331.5836049	92.707901	570.4593083	2.624584807	0.156902176	16.7275234	8.26E-63	1.70E-61
ENSG00000163545	NUAK2	275.9282437	77.271162	474.5853257	2.615799672	0.158868647	16.4651724	6.53E-61	1.30E-59

ENSG00000115267	IFIH1	2912.22367	819.0066	5005.440735	2.611958106	0.059382553	43.9852782	0	0
ENSG00000114251	WNT5A	23.26753833	6.5315908	40.00348584	2.611408705	0.517222525	5.04890753	4.44E-07	1.69E-06
ENSG00000136842	TMOD1	102.0386713	28.874327	175.2030151	2.600984441	0.257592403	10.0972871	5.68E-24	5.10E-23
ENSG00000197646	PDCD1LG2	2738.265392	776.2165	4700.314279	2.598182729	0.05909683	43.9648409	0	0
ENSG00000184441	AP001062.1	210.6452892	59.846484	361.444094	2.596617048	0.175283554	14.8138087	1.19E-49	1.98E-48
ENSG00000155307	SAMSN1	409.6204814	116.59834	702.6426251	2.59499809	0.146171723	17.7530786	1.63E-70	3.77E-69
ENSG00000196972	SMIM10L2B	7.330957826	2.1121404	12.54977524	2.578804768	0.933033202	2.76389389	0.005712	0.013345
ENSG00000229292	RFPL4AL1	14.78051747	4.3027148	25.2583201	2.576764305	0.676844133	3.80702761	0.000141	0.000415
ENSG00000137203	TFAP2A	1254.591167	360.90434	2148.277996	2.574696489	0.083055983	30.9995305	5.47E-211	3.85E-209
ENSG00000185634	SHC4	7.21158516	2.0533885	12.36978186	2.573115941	0.965368725	2.66542293	0.007689	0.017568
ENSG00000253958	CLDN23	31.10826812	8.9714842	53.24505208	2.571648068	0.446593369	5.75836599	8.49E-09	3.69E-08
ENSG00000089199	CHGB	11.49798095	3.3496149	19.64634698	2.566088945	0.740230204	3.46660935	0.000527	0.001445
ENSG00000166670	MMP10	570.1550876	165.21353	975.0966406	2.563775391	0.110766916	23.1456781	1.61E-118	6.32E-117
ENSG00000112139	MDGA1	244.0395006	70.642517	417.4364838	2.562351658	0.171138944	14.972347	1.11E-50	1.89E-49
ENSG00000122870	BICC1	63.54675733	18.355144	108.7383709	2.562293936	0.333818308	7.67571424	1.64E-14	1.01E-13
ENSG00000179630	LACC1	1170.678009	339.59255	2001.763472	2.561073681	0.086995485	29.4391562	1.73E-190	1.09E-188
ENSG00000284882	AL359762.1	16.43714095	4.7668854	28.10739652	2.560974213	0.629297736	4.06957481	4.71E-05	0.000147
ENSG00000136010	ALDH1L2	127.9136242	37.199691	218.6275575	2.559138214	0.227697844	11.2391851	2.62E-29	2.76E-28
ENSG00000171236	LRG1	18.60904422	5.4377649	31.78032356	2.555541171	0.581272532	4.39645955	1.10E-05	3.67E-05
ENSG00000162551	ALPL	40.04760889	11.660991	68.43422715	2.554736278	0.390272199	6.54603706	5.91E-11	3.00E-10
ENSG00000143387	CTSK	2945.772934	858.53913	5033.006735	2.552353125	0.0581756	43.8732581	0	0
ENSG00000026508	CD44	42260.04423	12317.841	72202.24792	2.551136853	0.04896525	52.1009664	0	0
ENSG00000112276	BVES	190.7578263	55.803062	325.7125906	2.549608513	0.192745732	13.2278339	6.06E-40	8.30E-39
ENSG00000137752	CASP1	695.9931005	204.05845	1187.927748	2.544050794	0.11499942	22.1222924	1.93E-108	6.84E-107
ENSG00000237499	AL357060.1	15.27916282	4.4563661	26.10195953	2.543751819	0.672832755	3.7806599	0.000156	0.000458
ENSG00000105371	ICAM4	29.7942562	8.8092161	50.77929634	2.540812827	0.465531936	5.45787009	4.82E-08	1.99E-07
ENSG00000143507	DUSP10	136.4410059	40.052851	232.8291604	2.534895189	0.224512792	11.2906493	1.46E-29	1.55E-28
ENSG00000142920	AZIN2	115.2747045	34.141207	196.4082016	2.527251102	0.23664416	10.6795414	1.27E-26	1.24E-25
ENSG00000102003	SYP	9.996378771	2.9498906	17.04286693	2.525847565	0.801621058	3.15092467	0.001628	0.00415
ENSG00000224715	Z82186.1	30.13721913	8.9671757	51.30726251	2.518857951	0.464650236	5.42097637	5.93E-08	2.42E-07
ENSG00000232124	AP001057.1	6.037710829	1.8213032	10.25411848	2.508431321	0.991195918	2.53071192	0.011383	0.025208
ENSG00000137628	DDX60	2294.086174	690.58185	3897.590493	2.498253098	0.080108821	31.1857431	1.66E-213	1.20E-211
ENSG00000108771	DHX58	687.7249399	207.21168	1168.238195	2.494379187	0.102990295	24.2195556	1.38E-129	5.97E-128
ENSG00000058866	DGKG	87.70709688	26.647606	148.7665881	2.483888562	0.278372249	8.92290295	4.54E-19	3.42E-18
ENSG00000150347	ARID5B	1377.159015	422.01633	2332.301701	2.466716263	0.083521251	29.5339956	1.05E-191	6.65E-190
ENSG00000203797	DDO	18.75485728	5.8070361	31.70267845	2.464742497	0.614576194	4.0105076	6.06E-05	0.000187
ENSG00000100906	NFKBIA	5084.983286	1560.9161	8609.050457	2.463041148	0.05073631	48.5459255	0	0
ENSG00000241288	LINC02614	16.48134978	5.0663394	27.89636012	2.460894587	0.620184652	3.96800304	7.25E-05	0.000221
ENSG00000144810	COL8A1	44866.58607	13803.406	75929.76634	2.459702025	0.038446263	63.9776627	0	0
ENSG00000278709	NKILA	37.88072427	11.726499	64.03494914	2.454502579	0.423814549	5.79145428	6.98E-09	3.06E-08
ENSG00000150630	VEGFC	3704.998539	1142.9191	6267.077975	2.454329907	0.056012699	43.8173831	0	0
ENSG00000152778	IFIT5	1270.078237	392.7068	2147.44967	2.45269049	0.085246641	28.7716964	4.85E-182	2.91E-180
ENSG00000149596	JPH2	34.50950296	10.616531	58.40247444	2.451487784	0.427285269	5.7373562	9.62E-09	4.17E-08
ENSG00000100092	SH3BP1	17.14438556	5.2876537	29.00111743	2.445400461	0.640529765	3.81777802	0.000135	0.000398
ENSG00000144115	THNSL2	8.569344767	2.654745	14.48394456	2.444329394	0.988621668	2.47246189	0.013419	0.029351
ENSG00000163666	HESX1	7.652092762	2.3725246	12.93166096	2.440363735	0.878763122	2.77704386	0.005486	0.012862
ENSG00000128284	APOL3	5232.917808	1633.445	8832.39063	2.435311217	0.051570364	47.2230756	0	0
ENSG00000146555	SDK1	725.53229	226.54129	1224.523289	2.432930064	0.098808257	24.6227405	7.21E-134	3.20E-132
ENSG00000164932	CTHRC1	6256.322882	1956.4516	10556.19416	2.43254966	0.053208399	45.7174003	0	0
ENSG00000245648	AC022075.1	72.37169744	22.657564	122.0858305	2.430264797	0.290415792	8.36822535	5.85E-17	4.04E-16
ENSG00000230943	LINC02541	14.612913	4.6719861	24.55383993	2.421140053	0.731676214	3.30903206	0.000936	0.002477
ENSG00000134853	PDGFRA	51.60450773	16.240261	86.96875475	2.414916123	0.419626545	5.75491744	8.67E-09	3.77E-08
ENSG00000107562	CXCL12	234.5459721	74.395073	394.6968708	2.4113399	0.174860099	13.7901094	2.92E-43	4.33E-42
ENSG00000272463	AL357054.4	17.91759805	5.7307563	30.10443979	2.40164316	0.595018988	4.03624625	5.43E-05	0.000168
ENSG00000064651	SLC12A2	10888.16801	3467.7633	18308.57277	2.400424784	0.045707919	52.5166057	0	0
ENSG00000140416	TPM1	39121.8021	12495.117	65748.48763	2.39571008	0.051740125	46.3027505	0	0
ENSG00000124243	BCAS4	93.2326693	29.80804	156.657299	2.393977911	0.255917179	9.35450257	8.40E-21	6.76E-20
ENSG00000124549	BTN2A3P	264.9721642	85.419917	444.5244115	2.386344312	0.186305222	12.8087892	1.46E-37	1.90E-36
ENSG00000242265	PEG10	4264.786629	1369.7819	7159.791387	2.385900216	0.060412578	39.4934347	0	0
ENSG00000105376	ICAM5	9.335744295	3.0347872	15.63670135	2.381094878	0.814959646	2.92173348	0.003481	0.008436
ENSG00000204876	AC021218.1	51.76045377	16.692569	86.82833898	2.377052058	0.34295534	6.93108337	4.18E-12	2.28E-11
ENSG00000169682	SPNS1	11.7617999	3.7636504	19.75994945	2.37334623	0.765999025	3.09836717	0.001946	0.004905
ENSG00000223638	RFPL4A	51.79977002	16.783928	86.81561224	2.372983051	0.352129102	6.7389575	1.60E-11	8.41E-11
ENSG00000105810	CDK6	4170.065658	1353.1131	6987.018225	2.369159316	0.053902333	43.9528155	0	0
ENSG00000250130	AC090519.1	6.633896829	2.1905744	11.07721876	2.368280092	1.036449438	2.28499337	0.022313	0.046466
ENSG00000213062	AL021068.1	12.8123357	4.1698373	21.45483412	2.36527732	0.6945287	3.40558615	0.00066	0.001785
ENSG00000145777	TSLP	27.46730961	8.9237974	46.01082177	2.364213339	0.510859922	4.62790922	3.69E-06	1.29E-05
ENSG00000166173	LARP6	998.4293769	324.57353	1672.285228	2.363148868	0.08972842	26.336682	7.29E-153	3.59E-151
ENSG00000259727	AC103740.2	22.34273032	7.3418105	37.34365017	2.361736385	0.56427325	4.18544807	2.85E-05	9.11E-05
ENSG00000188626	GOLGA8M	9.184297375	3.0064884	15.36210639	2.361025646	0.787935668	2.99647007	0.002731	0.006732
ENSG00000267325	LINC01415	32.85128713	10.690657	55.01191719	2.360999	0.448032236	5.26970787	1.37E-07	5.42E-07
ENSG00000198046	ZNF667	21.915788	7.1480268	36.68354922	2.359030459	0.526971821	4.476578	7.58E-06	2.57E-05
ENSG00000107249	GLIS3	460.7916937	150.9752	770.6081877	2.349965962	0.125788742	18.6818465	6.96E-78	1.77E-76
ENSG00000248161	AC098487.1	6.404435418	2.1121404	10.69673042	2.348079527	0.957331892	2.452733	0.014178	0.030801
ENSG00000157168	NRG1	8444.388077	2778.4972	14110.27894	2.344010792	0.055704837	42.079125	0	0
ENSG00000184254	ALDH1A3	1142.33643	375.9711	1908.701765	2.343660212	0.082641832	28.359248	6.44E-177	3.76E-175
ENSG00000213626	LBH	80.3638579	26.576991	134.1507247	2.343293855	0.292721003	8.00521258	1.19E-15	7.74E-15
ENSG00000135124	P2RX4	7040.051551	2324.8591	11755.24401	2.338441106	0.066967937	34.9188164	3.85E-267	3.79E-265
ENSG00000157680	DGKI	60.19882533	19.881007	100.5166436	2.334860754	0.320933528	7.27521605	3.46E-13	1.99E-12
ENSG00000048052	HDAC9	1273.12362	422.24597	2124.001272	2.331339275	0.084533589	27.5788513	2.00E-167	1.09E-165
ENSG00000089558	KCNH4	7.15370179	2.3725246	11.93487902	2.331185513	0.939284633	2.48187337	0.013069	0.028652
ENSG00000137965	IFI44	2154.214441	714.81365	3593.615237	2.330727881	0.065350708	35.6649214	1.38E-278	1.46E-276
ENSG00000223547	ZNF844	31.32742416	10.375535	52.27931316	2.330347644	0.472879406	4.92799563	8.31E-07	3.09E-06
ENSG00000121753	ADGRB2	155.6925293	51.640011	259.745048	2.326303802	0.198735252	11.7055418	1.19E-31	1.33E-30
ENSG00000151320	AKAP6	51.6473758	17.193655	86.10109679	2.320660307	0.344685889	6.7326815	1.67E-11	8.77E-11

ENSG00000233452	STXBP5-AS1	137.368619	45.964466	228.7727718	2.316457627	0.221659967	10.4505006	1.46E-25	1.38E-24
ENSG00000278949	AC127070.4	7.22470887	2.4248139	12.02460384	2.315333944	0.967101597	2.39409588	0.016661	0.035718
ENSG00000254109	RBPMS-AS1	14.40709922	4.9083798	23.90581869	2.303620026	0.661602772	3.48187783	0.000498	0.00137
ENSG00000285888	Z85996.2	6.981447093	2.3463799	11.61651429	2.299069955	0.926025516	2.48272852	0.013038	0.028597
ENSG00000179253	AL162457.1	6.972595203	2.3682162	11.57697425	2.28859069	0.952450566	2.40284459	0.016268	0.034939
ENSG00000159217	IGF2BP1	133.1292531	45.380637	220.8778687	2.284328351	0.216203594	10.5656354	4.30E-26	4.14E-25
ENSG00000081377	CDC14B	48.86408908	16.646742	81.08143632	2.280956438	0.351048181	6.49755949	8.16E-11	4.10E-10
ENSG00000170558	CDH2	3307.408894	1129.3382	5485.479601	2.280150429	0.159584423	14.2880513	2.60E-46	4.06E-45
ENSG00000077150	NFKB2	3224.407785	1101.9149	5346.900695	2.279067588	0.056078851	40.6404114	0	0
ENSG00000184371	CSF1	7765.100974	2661.406	12868.79596	2.273136005	0.048597984	46.7742858	0	0
ENSG00000142178	SIK1	135.5757847	46.539972	224.6115971	2.271091383	0.21564094	10.5318192	6.16E-26	5.90E-25
ENSG00000105357	MYH14	69.15501483	23.709578	114.6004519	2.269565509	0.37392861	6.06951554	1.28E-09	5.93E-09
ENSG00000168675	LDLRAD4	79.48883346	27.198059	131.7796082	2.269343347	0.2828006	8.02453513	1.02E-15	6.64E-15
ENSG00000109320	NFKB1	6045.945145	2087.5926	10004.29773	2.260627207	0.044329629	50.9958522	0	0
ENSG00000173193	PARP14	13263.12223	4580.8447	21945.39972	2.26052752	0.050638143	44.6408064	0	0
ENSG00000255150	EID3	27.2425558	9.4159727	45.06913895	2.25079962	0.491613592	4.57839176	4.69E-06	1.62E-05
ENSG00000131979	GCH1	584.370283	202.93299	965.8075764	2.248316666	0.112630973	19.9617974	1.18E-88	3.43E-87
ENSG00000168394	TAP1	6148.7276	2138.5512	10158.90396	2.247891319	0.047380978	47.4429071	0	0
ENSG00000266947	AC022916.1	8.368159681	2.8976013	13.83871808	2.240924198	0.9626857	2.32778382	0.019924	0.041969
ENSG00000003987	MTMR7	44.23798048	15.405253	73.07070783	2.240370042	0.383498366	5.84192852	5.16E-09	2.28E-08
ENSG00000113070	HBEGF	3178.418728	1115.7388	5241.098705	2.231324347	0.054971471	40.590588	0	0
ENSG00000136167	LCP1	11.17434123	3.9595883	18.38909419	2.230280293	0.728644544	3.06086186	0.002207	0.005515
ENSG00000112715	VEGFA	747.2605267	262.47444	1232.046613	2.229854228	0.10040061	22.2095686	2.78E-109	9.97E-108
ENSG00000233427	AL009181.1	15.87586834	5.53051	26.22122669	2.227785781	0.64022123	3.47971245	0.000502	0.00138
ENSG00000179869	ABCA13	22.70671578	7.9922396	37.42119198	2.225247822	0.539306923	4.12612509	3.69E-05	0.000117
ENSG00000154175	ABI3BP	38798.75676	13676.793	63920.72043	2.224460147	0.04705662	47.2719915	0	0
ENSG00000244701	AC004918.1	100.3597563	35.497278	165.2222351	2.219083646	0.245509129	9.03870115	1.59E-19	1.22E-18
ENSG00000109321	AREG	49.65613982	17.694418	81.61786193	2.216368494	0.435384634	5.09059879	3.57E-07	1.37E-06
ENSG00000205413	SAMD9	4549.811651	1613.7681	7485.85525	2.213815674	0.056816564	38.9642656	0	0
ENSG00000145779	TNFAIP8	494.2283748	175.55891	812.8978387	2.212707077	0.117835668	18.7779058	1.15E-78	2.95E-77
ENSG00000205502	C2CD4B	285.9068712	101.41903	470.3947119	2.212531089	0.16568978	13.3534554	1.13E-40	1.57E-39
ENSG00000147416	ATP6V1B2	10952.17876	3888.5691	18015.78839	2.212045299	0.041532429	53.2606771	0	0
ENSG00000137331	IER3	17050.71296	6058.7361	28042.68984	2.210369833	0.050229475	44.0054341	0	0
ENSG00000280303	ERICD	20.59022971	7.2604539	33.92000548	2.207017968	0.635956678	3.47039043	0.00052	0.001426
ENSG00000177409	SAMD9L	7275.579592	2599.1396	11952.01956	2.201484007	0.048774808	45.1356776	0	0
ENSG00000130558	OLFM1	12.40638811	4.4302214	20.38255478	2.197843059	0.6879955	3.19456023	0.0014	0.003609
ENSG00000205464	ATP6AP1L	34.96825716	12.618399	57.3181154	2.189874152	0.435295211	5.03077933	4.88E-07	1.86E-06
ENSG00000272669	AL021707.6	9.135292234	3.2754893	14.99509514	2.188670521	0.855048004	2.55970485	0.010476	0.023356
ENSG00000205517	RGL3	31.66290904	11.483349	51.84246919	2.181092101	0.45767841	4.76555602	1.88E-06	6.79E-06
ENSG00000253837	AC090197.1	52.12289954	18.87668	85.36911871	2.180070545	0.335001571	6.50764276	7.63E-11	3.85E-10
ENSG00000149289	ZC3H12C	2567.894737	928.65067	4207.138802	2.179521827	0.069920366	31.1714875	2.59E-213	1.86E-211
ENSG00000236830	CBR3-AS1	31.14704988	11.21544	51.07866018	2.178831074	0.464668038	4.68900569	2.75E-06	9.75E-06
ENSG00000197329	PELI1	2896.632694	1048.1417	4745.123652	2.177684547	0.060915915	35.7490247	6.85E-280	7.31E-278
ENSG00000165801	ARHGEF40	1151.200707	416.69651	1885.704907	2.17725519	0.078665489	27.6773873	1.31E-168	7.20E-167
ENSG00000170921	TANC2	2692.133125	975.26306	4409.003189	2.176889818	0.062917607	34.5990564	2.61E-262	2.53E-260
ENSG00000228318	AP001610.1	12.37646957	4.4868192	20.26611996	2.176657958	0.672728393	3.23556725	0.001214	0.003167
ENSG00000275993	SIK1B	152.3671686	55.235699	249.4986386	2.175813063	0.192286663	11.3154653	1.10E-29	1.17E-28
ENSG00000151014	NOCT	1151.477953	418.00775	1884.948152	2.173507065	0.092951956	23.3831235	6.35E-121	2.54E-119
ENSG00000184524	CEND1	63.60911234	22.989152	104.2290731	2.172960125	0.329917113	6.58638198	4.51E-11	2.31E-10
ENSG00000070404	FSTL3	3218.677772	1171.4483	5265.907249	2.167236454	0.073851114	29.3460226	2.69E-189	1.66E-187
ENSG00000132429	POPDC3	15.51024892	5.6590791	25.36141871	2.16321226	0.638961275	3.38551387	0.00071	0.001912
ENSG00000156804	FBXO32	1870.354466	684.12549	3056.583441	2.160259757	0.095262781	22.6768496	7.58E-114	2.84E-112
ENSG00000260101	AC008074.2	53.86474845	19.727356	88.00214109	2.159674274	0.324521394	6.65495193	2.83E-11	1.47E-10
ENSG00000204991	SPIRE2	41.19338636	15.161011	67.22576177	2.157763945	0.404697193	5.33179865	9.72E-08	3.91E-07
ENSG00000237807	AC022034.1	43.91771113	16.117357	71.71806554	2.155503815	0.37130752	5.80517146	6.43E-09	2.83E-08
ENSG00000254838	GVINP1	743.0737437	273.42794	1212.719551	2.151333759	0.102397279	21.009677	5.35E-98	1.72E-96
ENSG00000172738	TMEM217	916.8983091	336.60517	1497.191445	2.151011197	0.092976755	23.1349352	2.06E-118	8.05E-117
ENSG00000237892	KLF7-IT1	8.085499712	2.9760353	13.19496414	2.147946608	0.836571822	2.56755792	0.010242	0.022864
ENSG00000185507	IRF7	1107.536476	407.98042	1807.092537	2.14664589	0.094059876	22.8221212	2.77E-115	1.05E-113
ENSG00000215267	AKR1C7P	11.2995059	4.1895193	18.40949246	2.142423013	0.762047261	2.81140439	0.004933	0.011643
ENSG00000141294	LRRC46	8.303419994	3.1175296	13.48931034	2.14178635	0.915510272	2.33944546	0.019312	0.040851
ENSG00000060642	PIGV	529.9560002	195.78526	864.1267434	2.139608777	0.113645884	18.8269798	4.54E-79	1.18E-77
ENSG00000267454	ZNF582-AS1	9.907238898	3.7210404	16.09343743	2.13847358	0.813751059	2.6279211	0.008591	0.019463
ENSG00000274225	AP001065.1	14.11455537	5.3059499	22.92316087	2.134614395	0.799405684	2.67025171	0.007579	0.017341
ENSG00000272688	AP005329.3	20.38341447	7.6283393	33.13848965	2.133601234	0.553335394	3.85589149	0.000115	0.000344
ENSG00000145365	TIFA	1352.85359	502.6518	2203.055382	2.132483707	0.073134604	29.1583406	6.55E-187	4.02E-185
ENSG00000257093	KIAA1147	7790.070564	2897.5817	12682.55947	2.130139732	0.113080898	18.8373083	3.73E-79	9.73E-78
ENSG00000184678	HIST2H2BE	58.15366309	21.729517	94.57780872	2.129504577	0.322836867	6.59622488	4.22E-11	2.17E-10
ENSG00000106799	TGFBR1	1240.978629	462.06171	2019.895546	2.129290701	0.083747932	25.4249945	1.33E-142	6.21E-141
ENSG00000204632	HLA-G	27.22996339	10.18066	44.27926698	2.122449294	0.458831712	4.62576853	3.73E-06	1.31E-05
ENSG00000237276	ANO7L1	65.40424023	24.437643	106.370837	2.120980894	0.290895956	7.29120102	3.07E-13	1.77E-12
ENSG00000205710	C17orf107	40.48985551	15.096594	65.88311719	2.119479046	0.414870704	5.10877009	3.24E-07	1.25E-06
ENSG00000274370	AC130371.2	6.293187123	2.3463799	10.23999435	2.11517485	0.935961446	2.25989528	0.023828	0.049325
ENSG00000078098	FAP	77.79319594	29.37509	126.2113015	2.111385266	0.312219566	6.76250145	1.36E-11	7.18E-11
ENSG00000205220	PSMB10	624.5274985	235.09057	1013.964429	2.110403709	0.101298898	20.8334321	2.15E-96	6.84E-95
ENSG00000116711	PLA2G4A	1816.098189	682.55214	2949.644242	2.110213257	0.070231475	30.0465463	2.42E-198	1.59E-196
ENSG00000148798	INA	17.43959137	6.5620439	28.31713884	2.105681414	0.587428364	3.58457566	0.000338	0.000948
ENSG00000027869	SH2D2A	84.66273018	31.895127	137.4303334	2.10095928	0.271499396	7.73835711	1.01E-14	6.25E-14
ENSG00000279041	AC102945.2	18.83663515	7.1022	30.57107026	2.100130313	0.575490932	3.6492848	0.000263	0.000751
ENSG00000260578	AC110597.1	8.547098784	3.2102748	13.8839228	2.099623955	0.830379186	2.52851226	0.011455	0.025337
ENSG00000158246	TENT5B	63.67084449	24.096671	103.2450179	2.09751117	0.295452665	7.09931375	1.25E-12	7.02E-12
ENSG00000176697	BDNF	296.784244	112.87467	480.6938191	2.090241609	0.149517598	13.9799036	2.07E-44	3.12E-43
ENSG00000155980	KIF5A	22.04425441	8.4051833	35.68332547	2.089870252	0.538864097	3.87828817	0.000105	0.000315
ENSG00000115414	FN1	949229.8598	361263.92	1537195.799	2.089172233	0.037308125	55.9977809	0	0
ENSG00000163864	NMNAT3	8.695106152	3.3213161	14.06889625	2.089051189	0.8100122	2.5790367	0.009908	0.022181

ENSG00000261888	AC144831.1	23.56453772	8.999783	38.12929241	2.087600664	0.503965059	4.14235198	3.44E-05	0.000109
ENSG00000132821	VSTM2L	83.61294368	31.804536	135.4213513	2.087439824	0.260600044	8.01012843	1.15E-15	7.45E-15
ENSG00000081059	TCF7	147.1179124	56.369363	237.866462	2.08104198	0.203622667	10.22009	1.61E-24	1.47E-23
ENSG00000171451	DSEL	1096.991139	419.33744	1774.644834	2.080933252	0.079695522	26.1110437	2.73E-150	1.33E-148
ENSG00000160932	LY6E	4468.117996	1708.4474	7227.788609	2.080157527	0.062344715	33.3654186	4.35E-244	3.79E-242
ENSG00000189067	LITAF	205.4125879	78.763297	332.0618788	2.079921097	0.183237798	11.3509391	7.34E-30	7.85E-29
ENSG00000264672	SEPT4-AS1	6.972655856	2.665516	11.27979571	2.079468004	0.898758202	2.31371241	0.020684	0.043419
ENSG00000078081	LAMP3	1105.922654	423.55308	1788.29223	2.078431134	0.079794036	26.0474496	1.44E-149	7.00E-148
ENSG00000253159	PCDHGA12	1696.406323	650.29955	2742.513098	2.076885037	0.074802272	27.7649995	1.15E-169	6.42E-168
ENSG00000247934	AC022364.1	16.10133662	6.1297123	26.07296094	2.075462136	0.634518059	3.27092682	0.001072	0.002816
ENSG00000272567	AC109347.2	7.8286639	3.012951	12.64437683	2.072492106	0.909251032	2.27933984	0.022647	0.047078
ENSG00000258808	LINC02310	18.00191153	6.9617682	29.04205485	2.070860247	0.56151727	3.6879725	0.000226	0.00065
ENSG00000144476	ACKR3	2555.361548	983.81118	4126.911913	2.068393594	0.059434744	34.8010852	2.34E-265	2.28E-263
ENSG00000188313	PLSCR1	3127.136137	1207.4887	5046.783525	2.064000595	0.056029679	36.8376303	4.61E-297	5.19E-295
ENSG00000197261	C6orf141	140.1459128	54.256928	226.0348976	2.060459134	0.254221646	8.10497126	5.28E-16	3.48E-15
ENSG00000089041	P2RX7	770.0087556	298.25624	1241.761268	2.057924049	0.09349196	22.0117757	2.22E-107	7.81E-106
ENSG00000204525	HLA-C	39766.60435	15432.562	64100.64646	2.054397598	0.03498406	58.7238187	0	0
ENSG00000144366	GULP1	2781.336909	1081.5908	4481.08306	2.050525114	0.05950324	34.4607303	3.11E-260	2.94E-258
ENSG00000198832	SELENOM	2825.930149	1099.3049	4552.555447	2.04928811	0.066410798	30.8577546	4.41E-209	3.08E-207
ENSG00000164136	IL15	154.8031633	60.269431	249.3368958	2.049203611	0.189518276	10.8126966	3.00E-27	2.99E-26
ENSG00000214548	MEG3	184.160482	71.928418	296.392546	2.044511439	0.457872983	4.46523712	8.00E-06	2.71E-05
ENSG00000214530	STARD10	1037.808713	405.89935	1669.718073	2.04079901	0.096820932	21.0780765	1.26E-98	4.07E-97
ENSG00000231890	DARS-AS1	9.096386988	3.5315651	14.6612089	2.040227714	0.863617383	2.36242085	0.018156	0.038657
ENSG00000134070	IRAK2	2576.108399	1007.4027	4144.81408	2.039974551	0.05837562	34.9456597	1.51E-267	1.50E-265
ENSG00000067798	NAV3	2059.426774	806.66827	3312.185281	2.036219449	0.075193371	27.0797734	1.70E-161	8.89E-160
ENSG00000137496	IL18BP	946.0496406	370.81401	1521.285267	2.035376368	0.086443288	23.5457999	1.39E-122	5.62E-121
ENSG00000271425	NBPF10	302.6292263	118.76554	486.4929134	2.033581348	0.148573992	13.6873306	1.21E-42	1.77E-41
ENSG00000170373	CST1	36.98575441	14.478298	59.49321092	2.031172829	0.446524104	4.54885372	5.39E-06	1.86E-05
ENSG00000119922	IFIT2	547.1331075	215.33231	878.9339013	2.028792399	0.113338581	17.9002806	1.17E-71	2.78E-70
ENSG00000070961	ATP2B1	4117.422644	1621.7222	6613.123085	2.028452418	0.06128562	33.0983419	3.14E-240	2.67E-238
ENSG00000163536	SERPINI1	24.41126284	9.6752651	39.14726054	2.024145109	0.494642441	4.09213796	4.27E-05	0.000134
ENSG00000172403	SYNPO2	24.24815824	9.5989853	38.89733115	2.023269715	0.479344736	4.22090734	2.43E-05	7.84E-05
ENSG00000025039	RRAGD	27.89852202	11.083625	44.71341948	2.013609845	0.46850113	4.2979829	1.72E-05	5.64E-05
ENSG00000164342	TLR3	577.0402237	229.10916	924.9712827	2.013291238	0.112861223	17.8386445	3.54E-71	8.32E-70
ENSG00000182667	NTM	79.32314592	31.498325	127.1479667	2.010923753	0.281598847	7.1410937	9.26E-13	5.21E-12
ENSG00000133065	SLC41A1	3916.091855	1557.8971	6274.286649	2.009560413	0.049364587	40.7085432	0	0
ENSG00000065534	MYLK	2258.465713	899.31937	3617.612056	2.007951381	0.061174648	32.8232603	2.74E-236	2.22E-234
ENSG00000187583	PLEKHN1	54.33759371	21.60204	87.07314744	2.00748679	0.342556502	5.86030852	4.62E-09	2.05E-08
ENSG00000282034	AC106886.5	9.821441104	3.9355978	15.70728441	2.007336078	0.812628037	2.47017822	0.013505	0.029517
ENSG00000090376	IRAK3	1616.988926	645.30205	2588.675802	2.004795629	0.073403086	27.3121437	3.04E-164	1.64E-162
ENSG00000285761	AL645939.5	13.21252791	5.2504438	21.17461204	2.000483733	0.715009725	2.79784129	0.005145	0.012106
ENSG00000169860	P2RY1	14.03267406	5.598173	22.46717514	1.998387694	0.757609699	2.63775358	0.008346	0.018953
ENSG00000248932	AC097103.2	17.24807085	6.9463946	27.54974714	1.995683831	0.600728713	3.32210495	0.000893	0.002372
ENSG00000204622	HLA-J	11.17490309	4.5021928	17.84761336	1.993405349	0.752731883	2.6482276	0.008092	0.018411
ENSG00000267390	AC036176.1	10.33975772	4.1547578	16.52475759	1.989229289	0.809279883	2.4580239	0.01397	0.030411
ENSG00000206341	HLA-H	380.1249067	153.15577	607.094042	1.987311685	0.123420779	16.1019215	2.47E-58	4.73E-57
ENSG00000273802	HIST1H2BG	20.69919324	8.3113757	33.08701079	1.987014237	0.546103757	3.63852878	0.000274	0.000781
ENSG00000242173	ARHGDIG	60.2650622	24.347053	96.18307187	1.986486061	0.307121112	6.46808696	9.93E-11	4.96E-10
ENSG00000159128	IFNGR2	4412.780463	1783.1267	7042.434239	1.981200861	0.0536537	36.925708	1.79E-298	2.05E-296
ENSG00000169248	CXCL11	477.4634625	193.30456	761.6223696	1.979739997	0.119493151	16.5678115	1.19E-61	2.40E-60
ENSG00000253210	AC040970.1	34.69313195	14.046735	55.33952928	1.978371381	0.399649104	4.95027102	7.41E-07	2.77E-06
ENSG00000217801	AL390719.1	38.33364391	15.537836	61.12945136	1.976798245	0.384543226	5.14063988	2.74E-07	1.06E-06
ENSG00000181381	DDX60L	3366.186972	1366.4488	5365.925166	1.973605237	0.054421489	36.2651823	5.73E-288	6.33E-286
ENSG00000171714	ANO5	79.5721636	32.301314	126.8430133	1.970131914	0.277644911	7.095869	1.29E-12	7.19E-12
ENSG00000272273	IER3-AS1	20.30198199	8.2504695	32.35349445	1.963065093	0.521377929	3.76514805	0.000166	0.000485
ENSG00000019549	SNAI2	42.11015627	17.191501	67.02881192	1.957103041	0.379880702	5.15188856	2.58E-07	1.00E-06
ENSG00000065320	NTN1	12.44387345	5.1143204	19.77342653	1.952198249	0.689326621	2.83203664	0.004625	0.010976
ENSG00000141682	PMAIP1	1348.460656	556.13138	2140.789934	1.945519249	0.073084318	26.6202013	3.96E-156	2.00E-154
ENSG00000107201	DDX58	5018.118854	2070.6762	7965.561508	1.944151645	0.047410278	41.0069657	0	0
ENSG00000163291	PAQR3	1316.354987	544.19666	2088.513311	1.940045019	0.081641704	23.7629168	8.08E-125	3.36E-123
ENSG00000175538	KCNE3	33.99412751	14.100884	53.88737107	1.93755038	0.484464667	3.99936365	6.35E-05	0.000195
ENSG00000204267	TAP2	2209.139697	916.47566	3501.803731	1.933097474	0.064131584	30.1426748	1.34E-199	8.82E-198
ENSG00000266302	AC098850.3	9.334606569	3.8703832	14.7988299	1.932140033	0.806897813	2.39452878	0.016642	0.035684
ENSG00000138675	FGF5	3072.859463	1277.4961	4868.222778	1.929756591	0.054777915	35.2287341	7.26E-272	7.38E-270
ENSG00000163975	MELTF	207.8508146	86.547471	329.1541582	1.927151939	0.174275372	11.0580854	2.00E-28	2.07E-27
ENSG00000008516	MMP25	17.00674152	7.0326771	26.98080598	1.926911168	0.586675823	3.28445641	0.001022	0.00269
ENSG00000280649	AC245100.8	35.90955107	15.008451	56.8106508	1.923555355	0.392278033	4.90355104	9.41E-07	3.49E-06
ENSG00000124143	ARHGAP40	31.86750466	13.273431	50.46157866	1.921281915	0.435464704	4.41202673	1.02E-05	3.42E-05
ENSG00000100342	APOL1	6292.166069	2630.5049	9953.827236	1.920525561	0.05397195	35.583772	2.50E-277	2.61E-275
ENSG00000266538	AC005838.2	9.429312636	3.9857329	14.87289234	1.9200582	0.810255581	2.36969451	0.017803	0.03797
ENSG00000170581	STAT2	2908.510086	1216.9987	4600.021522	1.919057852	0.06206328	30.9209866	6.24E-210	4.37E-208
ENSG00000164061	BSN	10.70904023	4.4803566	16.9377239	1.919030511	0.703052529	2.72956917	0.006342	0.014701
ENSG00000285106	AC016831.7	17.1120061	7.1677088	27.05630337	1.91890056	0.584135114	3.28502861	0.00102	0.002685
ENSG00000225131	PSME2P2	15.01170635	6.3102766	23.71313614	1.912303448	0.63209001	3.02536572	0.002483	0.006157
ENSG00000198843	SELENOT	7379.024828	3099.6513	11658.39834	1.910994041	0.052043374	36.7192575	3.60E-295	4.03E-293
ENSG00000273312	AL121749.1	7.696164763	3.2145832	12.17774635	1.906636627	0.843872939	2.25938828	0.023859	0.049369
ENSG00000182107	TMEM30B	7.879791934	3.3496149	12.40996894	1.905421412	0.836973188	2.27656207	0.022812	0.047375
ENSG00000158457	TSPAN33	23.35554977	9.8701405	36.84095903	1.904158819	0.50456462	3.77386512	0.000161	0.00047
ENSG00000170458	CD14	38.93509239	16.436493	61.43369196	1.9032086	0.383009764	4.96908638	6.73E-07	2.52E-06
ENSG00000238000	AC116347.1	30.8203435	13.083632	48.55705497	1.903078548	0.462737249	4.11265475	3.91E-05	0.000124
ENSG00000156587	UBE2L6	3887.011829	1640.6961	6133.327563	1.902190321	0.061869635	30.7451356	1.42E-207	9.81E-206
ENSG00000184922	FMNL1	136.8036689	57.692351	215.9149865	1.900764345	0.228869762	8.30500424	9.98E-17	6.82E-16
ENSG00000267532	MIR497HG	13.19546972	5.5240474	20.86689206	1.90015219	0.724111339	2.62411606	0.008687	0.019663
ENSG00000131435	PDLIM4	5120.767209	2163.8394	8077.694995	1.899640266	0.066988911	28.3575333	6.76E-177	3.93E-175
ENSG00000233325	MIPEPP3	14.10558072	5.9606874	22.25047406	1.896968957	0.618441109	3.06733969	0.00216	0.005407

ENSG00000130303	BST2	17847.96432	7565.9519	28129.97674	1.894478288	0.054764798	34.5929931	3.22E-262	3.10E-260
ENSG00000173821	AC124319.1	28046.07607	11911.526	44180.62591	1.891111717	0.042273977	44.7346538	0	0
ENSG00000270157	AC004918.3	232.3351821	98.917908	365.7524565	1.88842062	0.167036164	11.3054597	1.23E-29	1.31E-28
ENSG00000131669	NINJ1	2967.913737	1266.5109	4669.316596	1.881763056	0.057594538	32.6725957	3.83E-234	3.03E-232
ENSG00000103811	CTSH	62.98591136	26.815568	99.15625463	1.881154675	0.330689623	5.68858091	1.28E-08	5.50E-08
ENSG00000185518	SV2B	10.91885494	4.625391	17.21231887	1.880486043	0.818965287	2.29617308	0.021666	0.045244
ENSG00000104951	IL4I1	1022.496222	436.92926	1608.063186	1.879423903	0.078828406	23.8419626	1.23E-125	5.12E-124
ENSG00000115318	LOXL3	13.74886707	5.8671739	21.6305602	1.878296414	0.745219693	2.52045998	0.01172	0.025875
ENSG00000130518	IQCN	12.91805767	5.5706424	20.26547292	1.876928951	0.767730332	2.44477634	0.014494	0.031453
ENSG00000170017	ALCAM	26441.39464	11331.128	41551.66126	1.874520319	0.038365082	48.8600631	0	0
ENSG00000179256	SMCO3	23.59045888	10.115445	37.06547252	1.872460668	0.481624129	3.88780494	0.000101	0.000304
ENSG00000231925	TAPBP	15729.49494	6751.2303	24707.75961	1.871634028	0.040575113	46.1276352	0	0
ENSG00000159231	CBR3	529.027773	227.02597	831.0295762	1.868758338	0.127583248	14.6473645	1.40E-48	2.29E-47
ENSG00000165949	IFI27	27103.14949	11661.642	42544.65649	1.867251163	0.0373341	50.0146293	0	0
ENSG00000129465	RIPK3	153.5309249	66.39342	240.6684301	1.863103894	0.225451273	8.26388721	1.41E-16	9.57E-16
ENSG00000053524	MCF2L2	78.73598373	34.023704	123.4482639	1.860692712	0.281101268	6.61929675	3.61E-11	1.86E-10
ENSG00000006459	KDM7A	842.1565062	364.11866	1320.194357	1.858749427	0.0906114	20.513417	1.63E-93	5.04E-92
ENSG00000278212	AC134878.2	11.85481241	5.1426192	18.56700557	1.85821803	0.683636141	2.71813896	0.006565	0.015171
ENSG00000134363	FST	485.6068807	209.86932	761.3444455	1.856744699	0.137423416	13.5111232	1.34E-41	1.92E-40
ENSG00000108556	CHRNE	35.91088055	15.52922	56.29254146	1.856536844	0.398244235	4.66180469	3.13E-06	1.11E-05
ENSG00000130508	PXDN	25784.28819	11184.575	40384.00097	1.852291331	0.118534282	15.6266296	4.80E-55	8.65E-54
ENSG00000136826	KLF4	77.2236048	33.423579	121.0256309	1.851663556	0.292974585	6.32021906	2.61E-10	1.27E-09
ENSG00000131831	RAI2	68.59562632	29.768675	107.4225772	1.851046575	0.280929776	6.589001	4.43E-11	2.27E-10
ENSG00000184988	TMEM106A	580.7467042	252.02555	909.467861	1.850662364	0.103114212	17.9476945	5.00E-72	1.19E-70
ENSG00000123096	SSPN	100.4916925	43.512739	157.4706458	1.850117079	0.245145734	7.54700909	4.45E-14	2.67E-13
ENSG00000101347	SAMHD1	4717.698464	2056.1363	7379.260593	1.843696898	0.057080033	32.3002073	6.95E-229	5.35E-227
ENSG00000244306	AL589743.1	17.10362786	7.4693171	26.73793864	1.842043833	0.577638153	3.18892342	0.001428	0.003677
ENSG00000154642	C21orf91	333.116654	145.77209	520.4612179	1.840038482	0.147663754	12.461003	1.22E-35	1.51E-34
ENSG00000136052	SLC41A2	868.9385652	380.51127	1357.365864	1.835608478	0.084024521	21.8461047	8.47E-106	2.93E-104
ENSG00000234518	PTGES3P1	43.81822829	19.252414	68.38404237	1.835571233	0.36104808	5.08400774	3.70E-07	1.42E-06
ENSG00000152413	HOMER1	19.24365988	8.4442532	30.04306652	1.835062068	0.561166472	3.27008501	0.001075	0.002824
ENSG00000123095	BHLHE41	96.144059	42.075787	150.2123312	1.832103325	0.243046523	7.53807667	4.77E-14	2.85E-13
ENSG00000239713	APOBEC3G	293.6560161	129.01035	458.3016811	1.828701125	0.148686857	12.2990099	9.17E-35	1.11E-33
ENSG00000026950	BTN3A1	856.653694	376.76981	1336.537583	1.826211811	0.087735829	20.814892	3.17E-96	1.01E-94
ENSG00000087237	CETP	94.01373269	41.388442	146.6390234	1.823971691	0.237362784	7.68432042	1.54E-14	9.46E-14
ENSG00000140450	ARRDC4	210.3117487	92.863707	327.7597905	1.82258746	0.167171529	10.9024992	1.12E-27	1.13E-26
ENSG00000179403	VWA1	405.8820063	179.08372	632.6802935	1.821278266	0.130505136	13.955606	2.91E-44	4.37E-43
ENSG00000154511	DIPK1A	49.46883986	21.828431	77.10924874	1.821161237	0.35875496	5.07633745	3.85E-07	1.48E-06
ENSG00000198429	ZNF69	92.03117968	40.65713	143.4052289	1.81985697	0.252119353	7.21823594	5.27E-13	3.00E-12
ENSG00000135899	SP110	1691.519989	747.08005	2635.959931	1.819015168	0.067118967	27.1013583	9.49E-162	4.98E-160
ENSG00000280287	AC131212.3	123.5159867	54.689848	192.3421252	1.818094512	0.221974156	8.1926611	2.56E-16	1.71E-15
ENSG00000186352	ANKRD37	90.75325147	40.04394	141.4625626	1.817203348	0.270918634	6.70756131	1.98E-11	1.04E-10
ENSG00000124201	ZNFX1	10072.36959	4452.7012	15692.03802	1.817180461	0.043525639	41.7496557	0	0
ENSG00000206503	HLA-A	36474.49192	16164.466	56784.51826	1.812750283	0.03903084	46.4440502	0	0
ENSG00000124508	BTN2A2	946.1116726	419.74437	1472.478975	1.811662868	0.091584619	19.7813005	4.31E-87	1.23E-85
ENSG00000269899	AC025857.2	25.60590061	11.357228	39.85457305	1.811289603	0.459173522	3.94467346	7.99E-05	0.000243
ENSG00000138496	PARP9	3009.080274	1335.137	4683.023544	1.81084062	0.060328261	30.0164567	5.99E-198	3.90E-196
ENSG00000115604	IL18R1	644.8236047	286.26856	1003.378648	1.810284875	0.098400706	18.397072	1.39E-75	3.45E-74
ENSG00000130635	COL5A1	67163.83303	29826.934	104500.7321	1.808837319	0.043600251	41.4868561	0	0
ENSG00000279118	AC093535.2	24.99395825	11.195728	38.79218812	1.808602243	0.61140867	2.95809061	0.003096	0.007557
ENSG00000185022	MAFF	5774.008645	2570.5793	8977.438027	1.803854404	0.053400896	33.7794782	3.95E-250	3.55E-248
ENSG00000167785	ZNF558	33.64403638	15.023531	52.26454199	1.802436188	0.434041813	4.1526787	3.29E-05	0.000105
ENSG00000136048	DRAM1	6400.677165	2856.0503	9945.303983	1.799777767	0.045353651	39.683195	0	0
ENSG00000162645	GBP2	3570.789934	1595.0846	5546.495273	1.797787666	0.050358893	35.6995073	4.02E-279	4.27E-277
ENSG00000136560	TANK	6113.713673	2743.0174	9484.409951	1.790311078	0.057460998	31.1569784	4.08E-213	2.91E-211
ENSG00000268205	AC005261.1	303.0245549	136.2909	469.7582069	1.789492752	0.157122581	11.3891507	4.74E-30	5.09E-29
ENSG00000279232	AC008522.1	10.62114361	4.7711938	16.47109342	1.788778156	0.710720158	2.51685299	0.011841	0.02612
ENSG00000164692	COL1A2	5305.340714	2384.3831	8226.298361	1.786836879	0.130250418	13.7184733	7.87E-43	1.16E-41
ENSG00000099822	HCN2	167.2825447	75.225299	259.3397908	1.785888706	0.184410704	9.68430068	3.52E-22	2.96E-21
ENSG00000078487	ZCWPW1	31.17490796	14.031361	48.31845494	1.785864571	0.42353775	4.21654167	2.48E-05	7.99E-05
ENSG00000188060	RAB42	78.60341496	35.466824	121.7400055	1.778643496	0.258471818	6.88138269	5.93E-12	3.20E-11
ENSG00000113742	CPEB4	1718.380108	777.89257	2658.867646	1.774032926	0.08400763	21.1175215	5.49E-99	1.78E-97
ENSG00000141424	SLC39A6	1660.806191	751.41874	2570.193643	1.773713905	0.070560865	25.1373607	1.94E-139	8.80E-138
ENSG00000138448	ITGAV	49158.24185	22284.719	76031.76498	1.770597685	0.037651027	47.0265446	0	0
ENSG00000226380	AC016831.1	13.66599785	6.1884643	21.14353145	1.767711226	0.642242388	2.7524051	0.005916	0.013776
ENSG00000106785	TRIM14	1491.866949	677.2481	2306.485796	1.767229234	0.073753484	23.9612984	7.05E-127	2.97E-125
ENSG00000124225	PMEPA1	3301.696386	1499.4433	5103.949446	1.76677792	0.054866357	32.2014804	1.68E-227	1.28E-225
ENSG00000154263	ABCA10	9.915326718	4.5129638	15.31768959	1.763494112	0.755870847	2.33306274	0.019645	0.041443
ENSG00000203688	LINC02487	13.82777949	6.3278044	21.32775456	1.763071336	0.680156357	2.59215593	0.009538	0.021435
ENSG00000111912	NCOA7	4905.326529	2233.0277	7577.625344	1.762837783	0.045853593	38.4449211	0	0
ENSG00000117394	SLC2A1	1118.678974	509.61454	1727.743406	1.761769541	0.07806185	22.5688929	8.76E-113	3.24E-111
ENSG00000214193	SH3D21	106.2274202	48.408668	164.0461726	1.761721529	0.234294105	7.5192738	5.51E-14	3.27E-13
ENSG00000020577	SAMD4A	6983.134469	3180.9961	10785.27288	1.761588872	0.048936174	35.9976832	9.09E-284	9.76E-282
ENSG00000164674	SYTL3	172.9037264	78.796522	267.0109308	1.761018861	0.17869641	9.85480828	6.53E-23	5.65E-22
ENSG00000130305	NSUN5	126.2305697	57.649741	194.8113979	1.757682678	0.212208141	8.28282397	1.20E-16	8.19E-16
ENSG00000139192	TAPBPL	556.5387672	254.58413	858.4934056	1.756603899	0.123556127	14.2170521	7.18E-46	1.12E-44
ENSG00000283959	AP002851.1	12.55364392	5.7744288	19.33285901	1.756562704	0.687014766	2.55680488	0.010564	0.023533
ENSG00000152661	GJA1	30223.90454	13805.621	46642.1876	1.756367183	0.039459481	44.5106507	0	0
ENSG00000135604	STX11	456.5600455	208.93868	704.1814138	1.753111736	0.111644791	15.7025843	1.45E-55	2.65E-54
ENSG00000204261	PSMB8-AS1	68.30396562	31.37433	105.2336017	1.752119374	0.302194263	5.7979902	6.71E-09	2.95E-08
ENSG00000100311	PDGFB	14099.56813	6472.2347	21726.90158	1.747204595	0.108848219	16.0517518	5.56E-58	1.05E-56
ENSG00000121281	ADCY7	657.1561555	302.13575	1012.176565	1.744625471	0.108659818	16.0558475	5.20E-58	9.84E-57
ENSG00000164307	ERAP1	8518.065283	3927.7177	13108.41292	1.739021749	0.051618252	33.6900551	8.08E-249	7.20E-247
ENSG00000243811	APOBEC3D	14.37248487	6.6752394	22.06973034	1.735861035	0.611853627	2.83705278	0.004553	0.010815
ENSG00000259426	AC027237.3	15.52380621	7.1523352	23.89527725	1.735428545	0.626612555	2.76954001	0.005614	0.013139

ENSG00000128298	BAIAP2L2	21.36035926	9.9005936	32.82012493	1.731558336	0.519509877	3.33306143	0.000859	0.002287
ENSG00000206532	AC117402.1	12.81403007	5.9302343	19.69782584	1.730682082	0.64054339	2.70189671	0.006895	0.015863
ENSG00000206538	VGLL3	295.3554015	137.02195	453.6888537	1.728609431	0.140516559	12.3018201	8.86E-35	1.07E-33
ENSG00000247081	BAALC-AS1	95.49352772	44.40647	146.5805858	1.724227433	0.267231711	6.45218123	1.10E-10	5.49E-10
ENSG00000136205	TNS3	4803.715131	2232.2656	7375.164666	1.723695897	0.053176958	32.4143382	1.72E-230	1.33E-228
ENSG00000176463	SLCO3A1	31.38309474	14.608727	48.15746245	1.723080229	0.419513339	4.1073312	4.00E-05	0.000126
ENSG00000285925	AC007391.2	9.611344983	4.4803566	14.7423334	1.718385583	0.72596608	2.36703288	0.017931	0.038227
ENSG00000166507	NDST2	20.48880591	9.546696	31.43091582	1.717597403	0.533157091	3.22155971	0.001275	0.003316
ENSG00000099617	EFNA2	14.27720666	6.7013841	21.85302926	1.715353184	0.64138675	2.67444437	0.007485	0.01714
ENSG00000135318	NT5E	24852.02843	11648.667	38055.39027	1.708034767	0.048035809	35.5575308	6.36E-277	6.60E-275
ENSG00000132000	PODNL1	31.81361021	14.936774	48.69044625	1.706980038	0.426407667	4.0031645	6.25E-05	0.000192
ENSG00000189077	TMEM120A	1667.9512	782.32148	2553.580916	1.706818957	0.079653574	21.4280272	7.32E-102	2.44E-100
ENSG00000117226	GBP3	2099.036421	984.86785	3213.204987	1.706356759	0.059068423	28.8877996	1.70E-183	1.03E-181
ENSG00000149201	CCDC81	126.8728902	59.621156	194.1246245	1.705112774	0.217705604	7.83219513	4.79E-15	3.02E-14
ENSG00000272482	AC254633.1	9.522389292	4.4803566	14.56442202	1.704834006	0.751840772	2.26754663	0.023357	0.048398
ENSG00000229953	AL590666.2	13.9374743	6.5577355	21.31721311	1.70047923	0.627087069	2.71171152	0.006694	0.015438
ENSG00000236345	SCAT8	13.46820444	6.3844022	20.55200672	1.69903795	0.635077655	2.67532314	0.007466	0.017103
ENSG00000188599	NPIPP1	13.32483203	6.3256502	20.32401385	1.688437728	0.63290609	2.66775396	0.007636	0.01746
ENSG00000269834	ZNF528-AS1	70.36968384	33.419101	107.3202665	1.688161006	0.306227836	5.51276144	3.53E-08	1.47E-07
ENSG00000233058	LINC00884	21.39896379	10.14159	32.65633767	1.687783556	0.513025398	3.28986355	0.001002	0.002643
ENSG00000167604	AD000864.1	97.28741511	46.18224	148.3925899	1.686705406	0.245225148	6.87819101	6.06E-12	3.27E-11
ENSG00000147650	LRP12	1721.373637	816.09077	2626.656501	1.686576196	0.073339433	22.9968536	5.01E-117	1.93E-115
ENSG00000261716	AC239868.1	38.89426602	18.476956	59.31157598	1.685013192	0.395701625	4.25829233	2.06E-05	6.69E-05
ENSG00000171608	PIK3CD	1377.915173	653.79106	2102.039284	1.683207114	0.088147131	19.0954272	2.76E-81	7.37E-80
ENSG00000134668	SPOCD1	3992.296374	1897.5556	6087.03715	1.680950675	0.064735583	25.9664097	1.19E-148	5.76E-147
ENSG00000102554	KLF5	28.03556169	13.284202	42.7869217	1.680689288	0.463634353	3.62503183	0.000289	0.000821
ENSG00000168062	BATF2	198.7097746	94.503678	302.9158717	1.680587011	0.178110004	9.43566882	3.89E-21	3.17E-20
ENSG00000258807	AL359237.1	11.96977443	5.7156769	18.22387198	1.680345975	0.698101258	2.40702327	0.016083	0.034579
ENSG00000186088	GSAP	169.5597163	80.912647	258.2067852	1.678555093	0.19192098	8.74607401	2.21E-18	1.61E-17
ENSG00000105639	JAK3	274.4727024	130.61527	418.3301389	1.67696228	0.148704291	11.2771613	1.70E-29	1.80E-28
ENSG00000090530	P3H2	404.5074746	192.81073	616.20422	1.676600795	0.134086672	12.5038587	7.11E-36	8.84E-35
ENSG00000135205	CCDC146	20.24016513	9.7447881	30.73554214	1.667924592	0.604960493	2.75708019	0.005832	0.013601
ENSG00000107984	DKK1	3805.630314	1823.2933	5787.967363	1.66586467	0.060059578	27.7368696	2.51E-169	1.40E-167
ENSG00000161960	EIF4A1	34.18109029	16.386358	51.9758229	1.663884423	0.412399022	4.03464687	5.47E-05	0.00017
ENSG00000198517	MAFK	1992.599598	956.6138	3028.585398	1.662540549	0.067481281	24.6370628	5.06E-134	2.25E-132
ENSG00000110911	SLC11A2	3593.579928	1727.9081	5459.251772	1.659825413	0.050696658	32.7403319	4.17E-235	3.35E-233
ENSG00000136732	GYPC	82.51777616	39.904277	125.1312755	1.65078258	0.285739246	5.77723432	7.59E-09	3.32E-08
ENSG00000134775	FHOD3	243.7954359	117.7351	369.8557749	1.649068242	0.157032042	10.5014761	8.50E-26	8.11E-25
ENSG00000135407	AVIL	11.68782043	5.6458597	17.72978117	1.647027378	0.670004724	2.45823248	0.013962	0.030407
ENSG00000008517	IL32	7642.58526	3705.0007	11580.16985	1.644205138	0.049798001	33.0174926	4.56E-239	3.77E-237
ENSG00000115828	QPCT	2679.744411	1299.6149	4059.873938	1.643162477	0.055219375	29.7569915	1.41E-194	8.94E-193
ENSG00000285437	POLR2J3	30.26223401	14.719768	45.80469971	1.643068358	0.426947154	3.84841155	0.000119	0.000354
ENSG00000130066	SAT1	10921.83472	5299.1222	16544.54724	1.642676811	0.046583184	35.2633006	2.15E-272	2.19E-270
ENSG00000179397	CATSPERE	19.12682283	9.3189191	28.93472653	1.640262432	0.543179294	3.01974403	0.00253	0.006267
ENSG00000215374	FAM66B	20.09942535	9.7351088	30.46374194	1.640002395	0.539254347	3.04124094	0.002356	0.005857
ENSG00000106003	LFNG	485.7890893	235.99926	735.5789228	1.639732983	0.107515752	15.2510953	1.62E-52	2.83E-51
ENSG00000196209	SIRPB2	1480.675755	719.56324	2241.788269	1.639655382	0.067937759	24.1346698	1.08E-128	4.63E-127
ENSG00000134871	COL4A2	195352.5536	95053.709	295651.398	1.637088095	0.098104867	16.6871241	1.63E-62	3.32E-61
ENSG00000156453	PCDH1	20639.9738	10054.958	31224.98968	1.634768998	0.054718971	29.875726	4.07E-196	2.62E-194
ENSG00000123342	MMP19	251.432025	122.52627	380.3377829	1.632709725	0.154034118	10.5996629	2.99E-26	2.90E-25
ENSG00000164220	F2RL2	607.5146502	297.09488	917.9344249	1.629848267	0.121089931	13.4598166	2.70E-41	3.82E-40
ENSG00000227376	FTH1P16	14.89489813	7.3353479	22.4544484	1.627447111	0.62575624	2.60076849	0.009302	0.020947
ENSG00000256043	CTSO	1130.044507	553.47236	1706.616652	1.626065115	0.090214844	18.0243632	1.25E-72	3.01E-71
ENSG00000168140	VASN	23.80973146	11.648065	35.97139752	1.625544301	0.562494843	2.88988303	0.003854	0.00929
ENSG00000183196	CHST6	29.88496095	14.649951	45.11997077	1.624811407	0.449436964	3.61521534	0.0003	0.00085
ENSG00000104635	SLC39A14	6217.144778	3047.5439	9386.745659	1.622779563	0.047777898	33.9650679	7.31E-253	6.73E-251
ENSG00000205363	INSYN1	19.26743735	9.40951	29.12536465	1.62064634	0.531582038	3.04872291	0.002298	0.005726
ENSG00000204764	RANBP17	15.12558327	7.4105651	22.84060142	1.619410663	0.585867189	2.76412589	0.005708	0.013337
ENSG00000008513	ST3GAL1	10811.28645	5317.8284	16304.74449	1.616206975	0.041051743	39.3699965	0	0
ENSG00000123989	CHPF	47.83485411	23.530076	72.13963217	1.613631711	0.348123362	4.6352296	3.57E-06	1.25E-05
ENSG00000105146	AURKC	15.19267988	7.517298	22.86806175	1.613576676	0.611120015	2.64035973	0.008282	0.018819
ENSG00000100911	PSME2	3199.943572	1576.7877	4823.099422	1.612550879	0.054646201	29.508929	2.21E-191	1.39E-189
ENSG00000104863	LIN7B	47.38721851	23.397967	71.3764702	1.611312982	0.408110358	3.94822859	7.87E-05	0.000239
ENSG00000104856	RELB	1017.405932	502.06095	1532.750918	1.609873878	0.090035455	17.8804437	1.67E-71	3.97E-70
ENSG00000285816	AP000944.2	39.7837903	19.739189	59.82839124	1.609623029	0.39766627	4.04767301	5.17E-05	0.000161
ENSG00000188921	HACD4	197.5629319	97.677216	297.4486474	1.607027187	0.164222463	9.78567218	1.30E-22	1.11E-21
ENSG00000260549	MT1L	1584.165461	783.45264	2384.878285	1.605221216	0.069378228	23.1372473	1.95E-118	7.65E-117
ENSG00000242732	RTL5	315.4289803	156.24851	474.6094475	1.604212415	0.137662095	11.6532616	2.21E-31	2.44E-30
ENSG00000264343	NOTCH2NLA	20.40681657	10.097917	30.71571576	1.604107496	0.572967641	2.79964763	0.005116	0.012046
ENSG00000138646	HERC5	109.0565438	54.041602	164.0714852	1.603951386	0.227788564	7.04140436	1.90E-12	1.06E-11
ENSG00000127585	FBXL16	24.24283517	12.045635	36.44003485	1.602227627	0.480420086	3.33505545	0.000853	0.002271
ENSG00000243753	HLA-L	65.05278491	32.252241	97.85332854	1.600611586	0.292850608	5.46562494	4.61E-08	1.91E-07
ENSG00000163840	DTX3L	3536.655266	1755.0215	5318.289078	1.599712633	0.056617505	28.2547356	1.24E-175	7.17E-174
ENSG00000261490	AC005674.2	14.48785989	7.2307692	21.74495059	1.598134194	0.639288691	2.49986308	0.012424	0.027339
ENSG00000162407	PLPP3	2023.462869	1007.7701	3039.155676	1.591882685	0.078866459	20.1845335	1.34E-90	4.00E-89
ENSG00000115594	IL1R1	1690.34347	842.17685	2538.510088	1.591562363	0.065209742	24.4068188	1.45E-131	6.33E-130
ENSG00000166741	NNMT	7449.743602	3714.6873	11184.79989	1.590389287	0.041254747	38.5504558	0	0
ENSG00000263528	IKBKE	1187.485402	592.50291	1782.467898	1.588580411	0.072756644	21.8341628	1.10E-105	3.80E-104
ENSG00000137563	GGH	3531.511237	1763.5681	5299.45437	1.587603647	0.049556382	32.0363107	3.41E-225	2.54E-223
ENSG00000114861	FOXP1	1623.728296	810.91988	2436.536709	1.587524348	0.070337915	22.569966	8.55E-113	3.17E-111
ENSG00000111266	DUSP16	1564.42412	781.11966	2347.728585	1.586763598	0.071166848	22.2963872	4.01E-110	1.45E-108
ENSG00000236017	ASMTL-AS1	22.13135888	11.163121	33.09959666	1.584453714	0.638890395	2.48000866	0.013138	0.028779
ENSG00000183793	NPIPA5	23.80988508	11.912464	35.70730643	1.582648981	0.53553963	2.95524158	0.003124	0.007621
ENSG00000049130	KITLG	3364.832402	1686.6514	5043.013426	1.580729863	0.070021939	22.57478	7.67E-113	2.85E-111
ENSG00000115415	STAT1	12123.98426	6088.3218	18159.64677	1.576836167	0.052118547	30.254799	4.51E-201	3.02E-199

ENSG00000108551	RASD1	335.3864762	168.35697	502.4159795	1.576061235	0.138848886	11.3509102	7.34E-30	7.85E-29
ENSG00000081148	IMPG2	20.73552604	10.449661	31.02139132	1.572661907	0.515826756	3.04881801	0.002297	0.005725
ENSG00000129116	PALLD	3312.429482	1669.8109	4955.048061	1.569288065	0.052560303	29.8569069	7.14E-196	4.58E-194
ENSG00000227076	AL158166.1	37.75691097	19.039243	56.47457927	1.567983544	0.403353839	3.88736487	0.000101	0.000304
ENSG00000197714	ZNF460	181.6698845	91.935366	271.4044033	1.565934343	0.191246651	8.18803537	2.66E-16	1.78E-15
ENSG00000160326	SLC2A6	3821.34439	1931.1601	5711.528663	1.56387341	0.056250285	27.8020532	4.10E-170	2.30E-168
ENSG00000123685	BATF3	18.34981844	9.2733866	27.42625026	1.563620939	0.549022037	2.84801126	0.004399	0.010472
ENSG00000188404	SELL	203.8894239	103.05009	304.7287576	1.561235565	0.172486382	9.05135551	1.41E-19	1.09E-18
ENSG00000017260	ATP2C1	8290.624118	4201.0042	12380.24407	1.559468266	0.042109868	37.0333214	3.33E-300	3.94E-298
ENSG00000163297	ANTXR2	5031.732077	2551.0235	7512.440703	1.558417061	0.047742033	32.6424528	1.03E-233	8.09E-232
ENSG00000135932	CAB39	5744.667555	2912.2462	8577.088939	1.558003797	0.0468584	33.2491887	2.10E-242	1.81E-240
ENSG00000014914	MTMR11	444.1332685	225.37425	662.892286	1.554577061	0.128263579	12.1201753	8.26E-34	9.70E-33
ENSG00000232759	AC002480.1	499.147771	253.76703	744.5285112	1.553103926	0.113741452	13.654687	1.89E-42	2.75E-41
ENSG00000164733	CTSB	132313.0255	67262.287	197363.7641	1.55299356	0.038856618	39.9672856	0	0
ENSG00000134470	IL15RA	1650.348471	838.61628	2462.080663	1.552863231	0.067127369	23.1330866	2.15E-118	8.39E-117
ENSG00000133107	TRPC4	61.94157987	31.424494	92.45866596	1.552650517	0.292102076	5.31543814	1.06E-07	4.26E-07
ENSG00000148143	ZNF462	2030.250038	1032.5187	3027.981398	1.552508271	0.06412471	24.2107649	1.71E-129	7.37E-128
ENSG00000010319	SEMA3G	4124.208911	2100.2238	6148.194015	1.549768646	0.177195452	8.74609721	2.21E-18	1.61E-17
ENSG00000172985	SH3RF3	1909.153323	972.40124	2845.905405	1.548789417	0.069734963	22.2096541	2.77E-109	9.97E-108
ENSG00000204592	HLA-E	40818.80286	20809.129	60828.47696	1.54748141	0.033974931	45.5477423	0	0
ENSG00000120337	TNFSF18	1858.797915	948.43439	2769.161443	1.546365216	0.061400776	25.1847831	5.88E-140	2.69E-138
ENSG00000156675	RAB11FIP1	56.64865582	28.876187	84.42112417	1.546081399	0.343815936	4.49682879	6.90E-06	2.35E-05
ENSG00000129667	RHBDF2	2589.762136	1321.6283	3857.895938	1.544690459	0.060849819	25.3852926	3.67E-142	1.70E-140
ENSG00000267534	S1PR2	81.00015254	41.432883	120.5674223	1.544625412	0.268904852	5.74413366	9.24E-09	4.01E-08
ENSG00000116741	RGS2	358.8485913	183.44306	534.2541218	1.542197236	0.122717949	12.5670063	3.21E-36	4.02E-35
ENSG00000170745	KCNS3	19.78107741	10.100072	29.46208323	1.541415985	0.505785897	3.04756616	0.002307	0.005745
ENSG00000120658	ENOX1	177.8400367	91.041047	264.6390263	1.539951271	0.176981209	8.70121341	3.28E-18	2.38E-17
ENSG00000172602	RND1	733.9730277	376.50045	1091.445604	1.536532897	0.09023677	17.0277915	5.11E-65	1.09E-63
ENSG00000100344	PNPLA3	55.67877637	28.671604	82.68594912	1.534703492	0.320464768	4.78899288	1.68E-06	6.07E-06
ENSG00000182636	NDN	87.82826493	45.09334	130.5631895	1.531528865	0.253664227	6.03762259	1.56E-09	7.19E-09
ENSG00000182600	SNORC	14.67180761	7.5738958	21.76971946	1.531080248	0.607758961	2.51922283	0.011761	0.025957
ENSG00000116514	RNF19B	1949.453079	1002.9473	2895.958867	1.528775001	0.071427035	21.4033103	1.24E-101	4.13E-100
ENSG00000169554	ZEB2	2945.246894	1516.1287	4374.365115	1.528097127	0.070552132	21.6591205	4.99E-104	1.70E-102
ENSG00000177679	SRRM3	114.8299255	59.286646	170.3732049	1.526199186	0.219997702	6.9373415	4.00E-12	2.18E-11
ENSG00000091129	NRCAM	20186.66448	10406.795	29966.534	1.525723852	0.044894	33.9850279	3.71E-253	3.43E-251
ENSG00000223653	AL078459.1	19.12175435	9.8593695	28.38413921	1.525635851	0.519320258	2.93775532	0.003306	0.008033
ENSG00000152894	PTPRK	9489.677186	4892.5826	14086.77182	1.525572664	0.042188599	36.1607801	2.52E-286	2.77E-284
ENSG00000134569	LRP4	171.277182	88.25481	254.2995536	1.524382281	0.181307447	8.40772018	4.18E-17	2.90E-16
ENSG00000179978	AC140134.1	12.7332722	6.5946512	18.87189322	1.523826827	0.656686712	2.32047763	0.020315	0.042722
ENSG00000108582	CPD	24510.9169	12654.233	36367.60124	1.523125665	0.046745479	32.5833794	7.05E-233	5.52E-231
ENSG00000105499	PLA2G4C	5163.0891	2666.6866	7659.491607	1.522380282	0.067236202	22.6422708	1.66E-113	6.21E-112
ENSG00000125657	TNFSF9	121.6238455	62.853884	180.3938067	1.517812282	0.23186419	6.54612635	5.90E-11	3.00E-10
ENSG00000101384	JAG1	5808.31304	3010.6446	8605.981435	1.515166631	0.046040595	32.9093622	1.61E-237	1.33E-235
ENSG00000196358	NTNG2	15.42808594	7.9704033	22.88576856	1.515008307	0.62143413	2.43792259	0.014772	0.032004
ENSG00000152463	OLAH	69.66762358	36.058502	103.2767455	1.5145589	0.300145809	5.04607713	4.51E-07	1.72E-06
ENSG00000225138	SLC9A3-AS1	68.638507	35.712898	101.5641165	1.513020585	0.292719985	5.16883254	2.36E-07	9.17E-07
ENSG00000166770	ZNF667-AS1	66.89774874	34.761952	99.03354567	1.512780329	0.277995323	5.44174741	5.28E-08	2.17E-07
ENSG00000274020	LINC01138	30.8547943	15.976157	45.73343204	1.512553303	0.458730857	3.29725651	0.000976	0.002579
ENSG00000090539	CHRD	28.22463179	14.577211	41.87205217	1.512396857	0.50999236	2.96552846	0.003022	0.007403
ENSG00000133687	TMTC1	82.90018343	43.088995	122.7113715	1.510980731	0.262865728	5.74810852	9.02E-09	3.92E-08
ENSG00000170801	HTRA3	2134.503192	1110.4767	3158.529723	1.508315185	0.06541519	23.057568	1.24E-117	4.78E-116
ENSG00000163453	IGFBP7	45300.31095	23605.32	66995.30227	1.504937959	0.045502318	33.0738745	7.06E-240	5.97E-238
ENSG00000005073	HOXA11	19.59940344	10.261248	28.93755887	1.503981423	0.515732173	2.91620632	0.003543	0.008575
ENSG00000140945	CDH13	16089.02916	8390.7536	23787.3047	1.503422786	0.052336126	28.7262913	1.79E-181	1.07E-179
ENSG00000205085	FAM71F2	12.69309964	6.6490947	18.73710455	1.502854459	0.636605838	2.36072994	0.018239	0.038812
ENSG00000205885	C1RL-AS1	46.91457826	24.608499	69.22065731	1.500180133	0.384167642	3.9050143	9.42E-05	0.000284
ENSG00000271605	MILR1	72.36288791	37.791662	106.9341135	1.499389302	0.268518515	5.58393265	2.35E-08	9.93E-08
ENSG00000227766	AL671277.1	12.56844394	6.5946512	18.5422367	1.498025807	0.650160684	2.3040855	0.021218	0.044427
ENSG00000155465	SLC7A7	1892.44053	989.91112	2794.969938	1.497991109	0.064552894	23.2056384	3.99E-119	1.57E-117
ENSG00000230795	HLA-K	34.77705854	18.210109	51.34400778	1.496847795	0.38658785	3.87194733	0.000108	0.000323
ENSG00000113369	ARRDC3	2168.26072	1134.7926	3201.728859	1.496345602	0.070851158	21.1195644	5.26E-99	1.71E-97
ENSG00000075426	FOSL2	9931.239562	5207.2203	14655.25882	1.492922853	0.045480594	32.8254913	2.55E-236	2.07E-234
ENSG00000133401	PDZD2	797.6779338	419.09447	1176.261397	1.490438963	0.10631109	14.0196	1.18E-44	1.80E-43
ENSG00000128335	APOL2	4266.489338	2240.6345	6292.344135	1.489711878	0.047708281	31.225436	4.81E-214	3.48E-212
ENSG00000087494	PTHLH	162.3026763	85.120047	239.4853055	1.488570762	0.197840909	7.52407967	5.31E-14	3.16E-13
ENSG00000144644	GADL1	25.45301524	13.362636	37.54339478	1.487262969	0.443054848	3.35683714	0.000788	0.002108
ENSG00000020181	ADGRA2	3928.685851	2068.9865	5788.385175	1.484825541	0.067488972	22.00101	2.82E-107	9.87E-106
ENSG00000184545	DUSP8	528.6009895	278.35186	778.8501161	1.484228773	0.10383945	14.2934961	2.40E-46	3.76E-45
ENSG00000234975	FTH1P2	142.6899501	75.138542	210.2413583	1.483191555	0.193608984	7.66075791	1.85E-14	1.13E-13
ENSG00000146350	TBC1D32	57.20880099	30.192096	84.22550598	1.482249177	0.333182075	4.44876627	8.64E-06	2.91E-05
ENSG00000172426	RSPH9	18.74375001	9.9095046	27.57799539	1.480505595	0.537051449	2.75672954	0.005838	0.013614
ENSG00000259660	DNM1P47	20.33477675	10.747255	29.92229868	1.480069633	0.530748151	2.78864775	0.005293	0.012435
ENSG00000105835	NAMPT	15700.42976	8295.1382	23105.72129	1.478008541	0.04630316	31.9202518	1.40E-223	1.03E-221
ENSG00000203896	LIME1	15.32827101	8.1512618	22.50528022	1.470966454	0.589497728	2.49528774	0.012586	0.027665
ENSG00000225828	FAM229A	36.50279326	19.480959	53.52462711	1.466548924	0.429643263	3.41341073	0.000642	0.001737
ENSG00000092010	PSME1	5040.82264	2680.2356	7401.409714	1.46568511	0.053519533	27.3859847	4.03E-165	2.19E-163
ENSG00000110057	UNC93B1	923.1080916	491.00036	1355.215819	1.463762456	0.083720461	17.4839273	1.90E-68	4.28E-67
ENSG00000276256	AC011043.1	470.1322973	250.39986	689.8647359	1.462864997	0.108706431	13.4570236	2.80E-41	3.96E-40
ENSG00000105464	GRIN2D	135.4311336	72.120988	198.7412788	1.460990346	0.198909316	7.34500715	2.06E-13	1.19E-12
ENSG00000158023	WDR66	130.7437867	69.876235	191.6113379	1.458863353	0.222984955	6.54242954	6.05E-11	3.07E-10
ENSG00000178752	ERFE	13.90393729	7.4019483	20.40592627	1.456198096	0.633415372	2.29896236	0.021507	0.044957
ENSG00000100979	PLTP	4200.631108	2245.0214	6156.240859	1.45504154	0.047808825	30.4345804	1.92E-203	1.30E-201
ENSG00000038945	MSR1	117.4536455	62.990159	171.9171323	1.451834664	0.219706959	6.60805042	3.89E-11	2.00E-10
ENSG00000257800	FNBP1P1	48.84726257	26.176204	71.51832093	1.449293214	0.328399985	4.41319513	1.02E-05	3.41E-05
ENSG00000148677	ANKRD1	55253.13077	29697.8	80808.46139	1.444187705	0.037798866	38.2071697	0	0

ENSG00000235385	LINC02154	348.7279084	187.26414	510.1916812	1.443604223	0.147544379	9.78420349	1.32E-22	1.13E-21
ENSG00000133424	LARGE1	99.71356505	53.568047	145.8590834	1.443485954	0.244645764	5.90031043	3.63E-09	1.63E-08
ENSG00000135842	FAM129A	1093.0438	588.08446	1598.003138	1.441391894	0.082849782	17.3976547	8.60E-68	1.92E-66
ENSG00000104320	NBN	3267.424145	1759.362	4775.486263	1.440413541	0.050343771	28.6115541	4.83E-180	2.87E-178
ENSG00000267519	AC020916.1	270.8348434	145.93666	395.7330237	1.440370037	0.389350975	3.69941294	0.000216	0.000623
ENSG00000175544	CABP4	19.94447069	10.824303	29.06463843	1.439944954	0.610760287	2.35762702	0.018392	0.039067
ENSG00000187229	AC100800.1	43.86621732	23.686944	64.04549058	1.439422496	0.350733672	4.1040328	4.06E-05	0.000128
ENSG00000233117	LINC00702	192.5804423	103.7936	281.3672896	1.438878258	0.408214699	3.52480756	0.000424	0.001176
ENSG00000220323	HIST2H2BD	49.73741309	26.896127	72.57869901	1.438119327	0.352773282	4.07661067	4.57E-05	0.000143
ENSG00000257354	AC048341.1	66.86993131	36.050359	97.68950371	1.436931123	0.310571806	4.62672753	3.71E-06	1.30E-05
ENSG00000083799	CYLD	3352.104609	1809.4926	4894.716602	1.436231051	0.053281186	26.9556886	4.89E-160	2.53E-158
ENSG00000133321	RARRES3	126.457437	68.188931	184.7259434	1.434859341	0.210415226	6.81918019	9.16E-12	4.89E-11
ENSG00000105559	PLEKHA4	319.8900784	172.99169	466.7884659	1.434497698	0.139133373	10.3102345	6.33E-25	5.86E-24
ENSG00000100968	NFATC4	1857.468413	1003.838	2711.098829	1.43437694	0.073972029	19.3908017	9.23E-84	2.55E-82
ENSG00000277734	TRAC	97.38480149	52.713537	142.056066	1.433834207	0.262715962	5.45773541	4.82E-08	1.99E-07
ENSG00000140379	BCL2A1	38.44838622	20.828707	56.06806555	1.433561163	0.407349595	3.51924043	0.000433	0.001199
ENSG00000160469	BRSK1	21.62033141	11.730808	31.50985502	1.433528006	0.546572505	2.62275909	0.008722	0.019727
ENSG00000166710	B2M	55666.99691	30112.929	81221.06519	1.431528089	0.045265306	31.6252825	1.66E-219	1.22E-217
ENSG00000232187	FTH1P7	72.30454194	39.181402	105.4276817	1.429973894	0.263851765	5.41961087	5.97E-08	2.44E-07
ENSG00000140044	JDP2	106.2634734	57.647767	154.8791797	1.428561993	0.285105567	5.01064222	5.42E-07	2.05E-06
ENSG00000234709	UPF3AP3	16.97250358	9.1666537	24.77835342	1.427790567	0.558579376	2.55611043	0.010585	0.023574
ENSG00000122420	PTGFR	16.5724685	9.0280819	24.11685509	1.424404556	0.574264885	2.4803964	0.013124	0.028758
ENSG00000241764	AC002467.1	68.05409746	37.08325	99.02494535	1.423977428	0.302953078	4.70032335	2.60E-06	9.24E-06
ENSG00000166452	AKIP1	873.9640849	475.30189	1272.626281	1.42269631	0.098282022	14.4756516	1.73E-47	2.77E-46
ENSG00000162706	CADM3	1558.56604	846.90315	2270.228926	1.421400976	0.094070332	15.1099815	1.39E-51	2.39E-50
ENSG00000150093	ITGB1	123856.6569	67361.658	180351.6559	1.420842046	0.039462375	36.0049806	6.99E-284	7.55E-282
ENSG00000155893	PXYLP1	40.21852977	21.904711	58.53234876	1.420670682	0.383754832	3.70202682	0.000214	0.000617
ENSG00000135063	FAM189A2	383.0051472	208.65445	557.3558411	1.420039598	0.131129845	10.8292632	2.50E-27	2.51E-26
ENSG00000168461	RAB31	3643.217408	1983.6844	5302.750402	1.418626403	0.052315212	27.1169005	6.22E-162	3.28E-160
ENSG00000104419	NDRG1	4502.349866	2451.818	6552.881766	1.417729443	0.053033068	26.732933	1.95E-157	9.88E-156
ENSG00000057019	DCBLD2	20602.26236	11228.852	29975.67274	1.416417184	0.0464926	30.4654329	7.48E-204	5.13E-202
ENSG00000095015	MAP3K1	1026.692177	560.60191	1492.782442	1.414068053	0.082427615	17.1552707	5.74E-66	1.25E-64
ENSG00000229644	NAMPTP1	80.76009431	44.071486	117.4487028	1.413774265	0.251689785	5.61713009	1.94E-08	8.25E-08
ENSG00000163661	PTX3	61450.07968	33544.479	89355.68055	1.41345102	0.042679196	33.1180333	1.63E-240	1.40E-238
ENSG00000134250	NOTCH2	7117.313769	3886.7841	10347.84344	1.41230689	0.052923957	26.6855875	6.92E-157	3.49E-155
ENSG00000187498	COL4A1	249322.219	136265.16	362379.279	1.411087927	0.110773318	12.7385182	3.61E-37	4.64E-36
ENSG00000167996	FTH1	248681.6119	135937.59	361425.6302	1.410740193	0.041771456	33.7728282	4.94E-250	4.42E-248
ENSG00000196263	ZNF471	108.8901378	59.471074	158.3092019	1.408635103	0.232627341	6.05532908	1.40E-09	6.46E-09
ENSG00000111674	ENO2	617.4486096	338.07456	896.8226587	1.407781683	0.098014512	14.3629923	8.83E-47	1.39E-45
ENSG00000075275	CELSR1	310.9754938	170.41768	451.5333028	1.406948413	0.340851267	4.12774881	3.66E-05	0.000116
ENSG00000139926	FRMD6	10646.88146	5841.0354	15452.72749	1.403787081	0.05198172	27.005399	1.28E-160	6.61E-159
ENSG00000113916	BCL6	1019.587617	559.51263	1479.66261	1.401832037	0.082344819	17.0239253	5.46E-65	1.17E-63
ENSG00000105696	TMEM59L	26.77099828	14.698226	38.84377028	1.400793795	0.518052593	2.70396059	0.006852	0.015768
ENSG00000127666	TICAM1	1239.771994	681.14788	1798.396113	1.399912717	0.076987934	18.1835341	6.97E-74	1.70E-72
ENSG00000145817	YIPF5	5231.466775	2884.9219	7578.011617	1.393262407	0.047411338	29.3866927	8.12E-190	5.04E-188
ENSG00000088899	LZTS3	699.172944	385.50109	1012.844798	1.392739293	0.098194409	14.1834887	1.16E-45	1.79E-44
ENSG00000211448	DIO2	250.0178195	137.79236	362.2432791	1.392387677	0.154653078	9.00329754	2.19E-19	1.67E-18
ENSG00000245067	IGFBP7-AS1	161.2728705	89.086866	233.4588748	1.389833982	0.22775002	6.10245382	1.04E-09	4.86E-09
ENSG00000074590	NUAK1	4038.661528	2233.1592	5844.163855	1.388375465	0.062086585	22.3619235	9.24E-111	3.37E-109
ENSG00000196839	ADA	1205.13787	667.32541	1742.950325	1.384938303	0.072181483	19.1868919	4.76E-82	1.29E-80
ENSG00000106686	SPATA6L	30.31261804	16.816829	43.80840678	1.384626123	0.425612991	3.25325155	0.001141	0.002985
ENSG00000180801	ARSJ	4505.324629	2495.9912	6514.658051	1.383854934	0.049146698	28.157638	1.93E-174	1.11E-172
ENSG00000156500	FAM122C	66.63696465	37.026652	96.24727748	1.383428012	0.287894475	4.80533019	1.54E-06	5.62E-06
ENSG00000180530	NRIP1	1998.487758	1108.4029	2888.572637	1.382591305	0.06398309	21.6086984	1.49E-103	5.03E-102
ENSG00000117758	STX12	7766.794818	4307.613	11225.97664	1.382021848	0.045525023	30.3574112	2.01E-202	1.35E-200
ENSG00000068078	FGFR3	20.83954289	11.547795	30.13129066	1.379835834	0.507751193	2.71754326	0.006577	0.015189
ENSG00000196517	SLC6A9	36.9666889	20.547872	53.38550543	1.378294056	0.405645166	3.39778252	0.000679	0.001833
ENSG00000270607	AC009549.1	416.2204527	231.13245	601.3084529	1.376526971	0.141488492	9.72889707	2.27E-22	1.93E-21
ENSG00000118985	ELL2	2934.571075	1633.3264	4235.815768	1.374828544	0.056185874	24.4692917	3.14E-132	1.37E-130
ENSG00000112297	CRYBG1	1259.974003	703.81284	1816.13517	1.368688979	0.103742405	13.1931487	9.61E-40	1.31E-38
ENSG00000165072	MAMDC2	1123.507901	627.67061	1619.345195	1.367707081	0.076750192	17.8202431	4.92E-71	1.15E-69
ENSG00000111801	BTN3A3	1079.594222	602.96254	1556.225904	1.367564482	0.075491713	18.1154251	2.41E-73	5.83E-72
ENSG00000100600	LGMN	6091.418064	3402.9299	8779.906272	1.36713298	0.045560178	30.0071911	7.91E-198	5.14E-196
ENSG00000164292	RHOBTB3	6264.734156	3500.9346	9028.533669	1.366639264	0.042365457	32.2583386	2.69E-228	2.06E-226
ENSG00000075643	MOCOS	60.54857514	33.759808	87.33734184	1.366384831	0.331897436	4.11688879	3.84E-05	0.000121
ENSG00000130813	C19orf66	1865.463807	1042.5444	2688.383197	1.365994055	0.061887378	22.0722559	5.84E-108	2.06E-106
ENSG00000275131	AC241952.1	114.326972	64.07475	164.5791939	1.365833832	0.260186045	5.24945076	1.53E-07	6.03E-07
ENSG00000136147	PHF11	730.0160058	408.24615	1051.785861	1.365575422	0.098833795	13.8168875	2.02E-43	2.99E-42
ENSG00000095397	WHRN	16.20791166	9.1043617	23.31146162	1.363675436	0.582496058	2.34108955	0.019228	0.040686
ENSG00000121933	TMIGD3	14.82939163	8.2766142	21.38216906	1.363133332	0.584804244	2.33092243	0.019757	0.041657
ENSG00000111110	PPM1H	455.345642	254.91147	655.7798181	1.362718077	0.118813718	11.4693665	1.88E-30	2.04E-29
ENSG00000178685	PARP10	1381.783704	773.66297	1989.904437	1.36263014	0.07265883	18.7538133	1.80E-78	4.61E-77
ENSG00000118804	STBD1	32.8898186	18.404985	47.37465254	1.361641311	0.389636693	3.49464343	0.000475	0.001309
ENSG00000170340	B3GNT2	1350.996601	757.91282	1944.08038	1.359979541	0.07332318	18.5477435	8.50E-77	2.14E-75
ENSG00000118946	PCDH17	198.7652151	111.50863	286.0218048	1.359154922	0.176296904	7.70946564	1.26E-14	7.81E-14
ENSG00000082126	MPP4	468.9573907	263.61333	674.3014499	1.356170285	0.116525426	11.6384066	2.63E-31	2.91E-30
ENSG00000139946	PELI2	137.5912757	77.38002	197.8025317	1.35513801	0.213136198	6.35808475	2.04E-10	9.97E-10
ENSG00000101104	PABPC1L	20.52746908	11.616226	29.43871174	1.355088832	0.579163224	2.33973563	0.019297	0.040824
ENSG00000185972	CCIN	62.19096852	34.848558	89.53337938	1.354145833	0.365566835	3.704236	0.000212	0.000611
ENSG00000197142	ACSL5	688.4501454	387.30678	989.5935091	1.353092229	0.096524035	14.0181896	1.21E-44	1.83E-43
ENSG00000106366	SERPINE1	567268.5294	319583.09	814953.9649	1.350524767	0.041991469	32.1618842	6.02E-227	4.55E-225
ENSG00000113971	NPHP3	176.6220491	99.675838	253.5682601	1.350064	0.18266079	7.39109908	1.46E-13	8.50E-13
ENSG00000223685	LINC00571	18.46169426	10.441044	26.48234457	1.347018766	0.552212072	2.43931423	0.014715	0.031892
ENSG00000075826	SEC31B	45.70879436	25.876133	65.54145612	1.346899466	0.464075127	2.90233065	0.003704	0.008948
ENSG00000188517	COL25A1	366.4061205	206.87499	525.9372491	1.34657225	0.125920986	10.6937873	1.09E-26	1.07E-25

ENSG00000139899	CBLN3	262.2210287	148.3821	376.0599574	1.344165057	0.150823557	8.91216919	5.00E-19	3.76E-18
ENSG00000171017	LRRC8E	116.2621945	65.671372	166.8530175	1.341965874	0.216811153	6.18956108	6.03E-10	2.86E-09
ENSG00000204262	COL5A2	35691.44202	20194.419	51188.46518	1.341914832	0.036352672	36.9137877	2.78E-298	3.16E-296
ENSG00000268895	A1BG-AS1	264.4615298	149.91348	379.0095818	1.337678378	0.154714007	8.64613619	5.33E-18	3.82E-17
ENSG00000272645	GTF2IP20	34.10018811	19.336248	48.86412791	1.336217783	0.392026117	3.40849175	0.000653	0.001767
ENSG00000269378	AC022149.1	391.1940342	222.57217	559.8158946	1.331113857	0.119415295	11.1469294	7.41E-29	7.72E-28
ENSG00000176293	ZNF135	20.90811584	11.873394	29.94283783	1.330318206	0.519508656	2.56072385	0.010445	0.023291
ENSG00000112837	TBX18	2008.041042	1142.3386	2873.743468	1.330266611	0.062218013	21.3807312	2.02E-101	6.68E-100
ENSG00000140853	NLRC5	1119.328723	637.65751	1600.999937	1.329105636	0.084999518	15.6366256	4.10E-55	7.40E-54
ENSG00000196172	ZNF681	20.27471191	11.513034	29.03639017	1.329042277	0.512149712	2.59502689	0.009458	0.021269
ENSG00000125845	BMP2	1085.283219	618.42374	1552.142693	1.327736832	0.085981391	15.4421418	8.52E-54	1.52E-52
ENSG00000113657	DPYSL3	12758.09163	7274.8909	18241.29235	1.326070938	0.040335344	32.8761527	4.82E-237	3.94E-235
ENSG00000161040	FBXL13	449.3909289	256.43821	642.343646	1.325463079	0.117203968	11.3090291	1.18E-29	1.26E-28
ENSG00000123240	OPTN	13777.96053	7863.1865	19692.73455	1.324588326	0.038341124	34.547457	1.56E-261	1.48E-259
ENSG00000060656	PTPRU	320.0430903	182.76035	457.3258328	1.321863984	0.137642512	9.60360257	7.72E-22	6.43E-21
ENSG00000232926	AC000078.1	17.69272052	10.112997	25.27244423	1.321050322	0.57572483	2.29458633	0.021757	0.045419
ENSG00000226564	FTH1P20	79.10074178	45.160415	113.0410687	1.3204699	0.277602494	4.75669321	1.97E-06	7.07E-06
ENSG00000116774	OLFML3	157.5467538	90.144545	224.9489627	1.320290166	0.187913629	7.02604796	2.12E-12	1.18E-11
ENSG00000171940	ZNF217	2062.58527	1180.2005	2944.970022	1.319634346	0.065824498	20.0477692	2.11E-89	6.23E-88
ENSG00000164463	CREBRF	1035.573969	593.15024	1477.997698	1.317368262	0.076945433	17.1208116	1.04E-65	2.25E-64
ENSG00000206195	DUXAP8	43.64235995	25.07453	62.21019014	1.316713522	0.373391662	3.52636027	0.000421	0.001169
ENSG00000187688	TRPV2	1145.475536	656.31529	1634.635781	1.315455839	0.080553573	16.3301985	6.02E-60	1.18E-58
ENSG00000286215	AL356534.1	36.16049949	20.780726	51.54027301	1.315366996	0.379718259	3.46406043	0.000532	0.001457
ENSG00000122779	TRIM24	1407.69125	807.51966	2007.862839	1.314239216	0.069223754	18.9853793	2.25E-80	5.94E-79
ENSG00000155324	GRAMD2B	394.0131992	226.20138	561.8250175	1.312090095	0.120504374	10.8883193	1.31E-27	1.32E-26
ENSG00000055332	EIF2AK2	4961.904032	2851.0422	7072.765881	1.311297242	0.056264448	23.3059649	3.86E-120	1.54E-118
ENSG00000154640	BTG3	1039.89402	597.89963	1481.888414	1.309538924	0.07636967	17.1473692	6.58E-66	1.43E-64
ENSG00000169871	TRIM56	3847.425073	2213.3189	5481.531274	1.308622687	0.053256314	24.5721604	2.51E-133	1.11E-131
ENSG00000131016	AKAP12	51159.49567	29428.3	72890.69091	1.308547335	0.044462429	29.4304061	2.24E-190	1.40E-188
ENSG00000178015	GPR150	38.34479121	22.015781	54.67380123	1.307054182	0.473243736	2.76190487	0.005747	0.013422
ENSG00000267309	AC092295.2	42.76566045	24.593155	60.93816619	1.30469259	0.380476892	3.42909811	0.000606	0.001647
ENSG00000267796	LIN37	33.13638286	19.106317	47.16644848	1.303479653	0.410594208	3.17461773	0.0015	0.003849
ENSG00000240476	LINC00973	19.83131583	11.380156	28.28247556	1.303338867	0.561054035	2.32301844	0.020178	0.042448
ENSG00000277782	AC068870.2	59.2427663	34.105383	84.38014917	1.303162907	0.303133933	4.29896744	1.72E-05	5.62E-05
ENSG00000160323	ADAMTS13	64.25648718	37.200753	91.31222088	1.302187878	0.324246466	4.01604339	5.92E-05	0.000183
ENSG00000179820	MYADM	6967.889716	4020.5333	9915.246145	1.301886627	0.050240417	25.9131334	4.74E-148	2.29E-146
ENSG00000171606	ZNF274	866.2398231	500.3318	1232.147846	1.300046305	0.092304059	14.0843893	4.74E-45	7.23E-44
ENSG00000268516	AC020915.3	148.2706533	85.755842	210.7854649	1.299280345	0.197083159	6.59254882	4.32E-11	2.22E-10
ENSG00000106665	CLIP2	3249.537536	1878.1847	4620.890375	1.29837784	0.060081463	21.61029	1.44E-103	4.87E-102
ENSG00000180155	LYNX1	204.4531537	118.07412	290.8321832	1.297971975	0.199197498	6.5160054	7.22E-11	3.64E-10
ENSG00000132274	TRIM22	9600.977283	5553.4169	13648.53767	1.2976225	0.046831342	27.7084201	5.53E-169	3.07E-167
ENSG00000174136	RGMB	2316.207844	1340.0951	3292.320575	1.296227538	0.067482998	19.208209	3.16E-82	8.55E-81
ENSG00000144749	LRIG1	5179.322474	3000.302	7358.3429	1.294397277	0.04543595	28.4883948	1.63E-178	9.58E-177
ENSG00000071242	RPS6KA2	14992.60673	8687.6147	21297.59873	1.293635296	0.043962149	29.4261162	2.55E-190	1.58E-188
ENSG00000258405	ZNF578	116.673688	67.761647	165.5857294	1.292263835	0.215509277	5.99632578	2.02E-09	9.20E-09
ENSG00000126218	F10	27.82641863	16.169646	39.4831912	1.291984359	0.433475073	2.98052746	0.002878	0.007072
ENSG00000178053	MLF1	210.4461401	122.3283	298.5639837	1.290654938	0.177446322	7.27349502	3.50E-13	2.01E-12
ENSG00000183458	AC138932.1	238.9308281	138.89309	338.9685628	1.287966582	0.159702005	8.06481158	7.33E-16	4.81E-15
ENSG00000151789	ZNF385D	36.30158463	21.107416	51.49575291	1.287068226	0.403681262	3.18832789	0.001431	0.003683
ENSG00000114923	SLC4A3	20.70280326	12.058561	29.3470458	1.285355091	0.522735968	2.45889927	0.013936	0.030358
ENSG00000134627	PIWIL4	25.31063271	14.800357	35.8209089	1.283612595	0.464842118	2.76139477	0.005756	0.01344
ENSG00000186907	RTN4RL2	25.01842293	14.63378	35.40306583	1.283492865	0.511042803	2.51151735	0.012021	0.026493
ENSG00000180758	GPR157	276.6018181	160.90825	392.2953864	1.282580007	0.154178348	8.31880757	8.89E-17	6.08E-16
ENSG00000042832	TG	17.30792378	10.10438	24.51146755	1.278170855	0.542655282	2.35540112	0.018503	0.039289
ENSG00000160285	LSS	5816.876342	3396.6699	8237.082759	1.277710995	0.047031975	27.1668584	1.60E-162	8.52E-161
ENSG00000269001	AC092070.2	100.4273698	58.804948	142.0497919	1.276320705	0.248348488	5.13923285	2.76E-07	1.07E-06
ENSG00000159200	RCAN1	1092.843626	640.28175	1545.405507	1.271955214	0.077268492	16.4614991	6.94E-61	1.37E-59
ENSG00000013375	PGM3	4633.002276	2713.883	6552.121511	1.271814265	0.046962477	27.0814988	1.63E-161	8.51E-160
ENSG00000176853	FAM91A1	3408.922769	1999.3097	4818.535841	1.269561354	0.053503481	23.7285751	1.83E-124	7.54E-123
ENSG00000143110	C1orf162	19.18728872	11.226505	27.14807262	1.269349386	0.511141719	2.48336095	0.013015	0.028553
ENSG00000276077	CU633904.1	21.32209836	12.503049	30.14114749	1.268438511	0.490114076	2.5880475	0.009652	0.021656
ENSG00000168874	ATOH8	549.5518925	322.35005	776.7537353	1.267164898	0.114264353	11.0897657	1.41E-28	1.46E-27
ENSG00000130775	THEMIS2	483.391994	283.65646	683.127532	1.266995934	0.108608861	11.6656774	1.91E-31	2.12E-30
ENSG00000158373	HIST1H2BD	32.89251212	19.312552	46.47247218	1.266382542	0.39997178	3.16617973	0.001545	0.003955
ENSG00000280594	BTG3-AS1	28.60392125	16.758875	40.4489677	1.265993437	0.535424494	2.36446679	0.018056	0.038471
ENSG00000013374	NUB1	2967.932405	1743.5839	4192.280933	1.265953145	0.053426044	23.6954311	4.02E-124	1.65E-122
ENSG00000064932	SBNO2	4013.879553	2357.4508	5670.308336	1.265836607	0.056247507	22.5047592	3.73E-112	1.37E-110
ENSG00000156265	MAP3K7CL	317.9203263	186.94798	448.8926746	1.264613497	0.135786912	9.31322081	1.24E-20	9.91E-20
ENSG00000124134	KCNS1	18.12998882	10.653447	25.60633046	1.262600144	0.536925705	2.35153603	0.018696	0.039659
ENSG00000180543	TSPYL5	152.1897602	89.513827	214.8656936	1.26210338	0.194804579	6.47881783	9.24E-11	4.63E-10
ENSG00000171174	RBKS	43.70659143	25.729561	61.68362136	1.26159605	0.346299929	3.64307337	0.000269	0.000768
ENSG00000114529	C3orf52	180.5740911	106.57564	254.5725446	1.259151859	0.192130162	6.55363971	5.62E-11	2.86E-10
ENSG00000171812	COL8A2	83.71292001	49.344534	118.0813058	1.259029234	0.248953393	5.05728892	4.25E-07	1.63E-06
ENSG00000254087	LYN	2805.948858	1653.5612	3958.336522	1.25880427	0.057520293	21.8845246	3.65E-106	1.27E-104
ENSG00000179627	AL590327.1	307.7213262	181.36627	434.0763821	1.258675347	0.135209549	9.30907139	1.29E-20	1.03E-19
ENSG00000184384	MAML2	2003.357331	1181.0044	2825.710216	1.258548579	0.061225041	20.5561085	6.79E-94	2.10E-92
ENSG00000130827	PLXNA3	3691.598827	2177.3135	5205.884194	1.257617374	0.053247578	23.6183018	2.50E-123	1.02E-121
ENSG00000186470	BTN3A2	1566.465654	923.96499	2208.966315	1.257555526	0.072661847	17.3069578	4.17E-67	9.25E-66
ENSG00000123870	ZNF137P	35.1941927	20.867777	49.5206086	1.254205541	0.406140406	3.08810826	0.002014	0.005067
ENSG00000231205	ZNF826P	31.97383994	18.829791	45.11788877	1.252622968	0.438589922	2.85602314	0.00429	0.010246
ENSG00000086991	NOX4	678.5217814	401.60134	955.4422276	1.251462313	0.106159218	11.7885412	4.47E-32	5.06E-31
ENSG00000260604	AL590004.3	1186.09565	702.06046	1670.130841	1.251066902	0.08510273	14.7006671	6.38E-49	1.05E-47
ENSG00000005238	FAM214B	3596.444607	2127.4607	5065.428498	1.250931733	0.062592456	19.9853434	7.39E-89	2.15E-87
ENSG00000157224	CLDN12	405.3529188	240.07387	570.6319673	1.250659275	0.118372679	10.5654386	4.31E-26	4.15E-25
ENSG00000112763	BTN2A1	1559.883439	923.94871	2195.81817	1.248880525	0.070156253	17.8014143	6.89E-71	1.61E-69

ENSG00000229619	MBNL1-AS1	83.11519502	49.283628	116.946762	1.248472692	0.246740751	5.05985609	4.20E-07	1.60E-06
ENSG00000196345	ZKSCAN7	32.85827139	19.457292	46.25925048	1.246654193	0.431980232	2.88590565	0.003903	0.009395
ENSG00000259345	AC013652.1	55.82501029	33.120445	78.52957601	1.244819589	0.306568433	4.06049499	4.90E-05	0.000153
ENSG00000131981	LGALS3	2855.595589	1694.1663	4017.024911	1.244766815	0.065361681	19.0442901	7.33E-81	1.95E-79
ENSG00000270069	MIR222HG	90.30973876	53.697384	126.9220934	1.244645036	0.249448298	4.98959122	6.05E-07	2.28E-06
ENSG00000160602	AC010761.1	55.158147	32.81745	77.49884356	1.244592007	0.323629253	3.84573395	0.00012	0.000357
ENSG00000145476	CYP4V2	92.676546	54.963481	130.3896111	1.243513044	0.234472606	5.30344702	1.14E-07	4.54E-07
ENSG00000246695	RASSF8-AS1	147.9713587	87.86506	208.0776579	1.242456843	0.188931918	6.57621464	4.83E-11	2.46E-10
ENSG00000106070	GRB10	11882.22408	7066.9907	16697.45742	1.240310263	0.04339924	28.5790781	1.22E-179	7.25E-178
ENSG00000008710	PKD1	3700.435209	2200.9156	5199.954824	1.240130473	0.058831681	21.0792969	1.23E-98	3.97E-97
ENSG00000106701	FSD1L	122.566583	73.014245	172.1189214	1.240119277	0.211322233	5.86838054	4.40E-09	1.96E-08
ENSG00000183087	GAS6	15595.36207	9279.8184	21910.90571	1.23948688	0.039934928	31.0376644	1.67E-211	1.18E-209
ENSG00000086065	CHMP5	3068.446313	1826.6141	4310.278563	1.239088254	0.053371273	23.2163892	3.11E-119	1.23E-117
ENSG00000177380	PPFIA3	136.3250446	81.030798	191.6192912	1.238429085	0.19897792	6.22395232	4.85E-10	2.31E-09
ENSG00000197256	KANK2	2351.187786	1399.9184	3302.457169	1.238388509	0.055472546	22.3243497	2.14E-110	7.81E-109
ENSG00000203326	ZNF525	107.5982439	64.260535	150.9359532	1.235000806	0.224936366	5.49044528	4.01E-08	1.66E-07
ENSG00000133121	STARD13	2307.483142	1375.9969	3238.969366	1.234720766	0.056805404	21.7359738	9.38E-105	3.21E-103
ENSG00000131067	GGT7	118.7763513	70.916386	166.6363164	1.234469824	0.234162472	5.27185169	1.35E-07	5.36E-07
ENSG00000177191	B3GNT8	126.0487792	75.299718	176.7978401	1.231726127	0.209942559	5.86696729	4.44E-09	1.97E-08
ENSG00000114268	PFKFB4	144.2634953	86.156658	202.3703328	1.23143398	0.188299889	6.53974882	6.16E-11	3.13E-10
ENSG00000223745	CCDC18-AS1	21.26756408	12.768804	29.76632383	1.230803394	0.512866324	2.39985223	0.016402	0.035214
ENSG00000185880	TRIM69	1126.520959	673.86618	1579.175737	1.229043436	0.072933782	16.8514974	1.02E-63	2.14E-62
ENSG00000278662	GOLGA6L10	25.41314943	15.207929	35.61836952	1.228150069	0.463506183	2.64969511	0.008056	0.018341
ENSG00000188549	CCDC9B	3389.500402	2030.1322	4748.868652	1.226446429	0.055260594	22.1938699	3.94E-109	1.41E-107
ENSG00000204681	GABBR1	313.6610065	188.00905	439.3129596	1.226411381	0.141832092	8.64692443	5.29E-18	3.79E-17
ENSG00000166147	FBN1	26421.54292	15824.284	37018.80209	1.22620926	0.056293158	21.7825631	3.40E-105	1.17E-103
ENSG00000106624	AEBP1	5651.176906	3384.948	7917.405809	1.225914478	0.044276709	27.6875698	9.86E-169	5.45E-167
ENSG00000182957	SPATA13	420.7068156	252.14243	589.2711975	1.224970564	0.114665906	10.6829537	1.22E-26	1.20E-25
ENSG00000050165	DKK3	31213.98143	18726.544	43701.41842	1.222625277	0.040294739	30.3420569	3.20E-202	2.15E-200
ENSG00000118971	CCND2	448.2509043	269.10389	627.3979196	1.222565691	0.138735745	8.81218961	1.23E-18	9.08E-18
ENSG00000145780	FEM1C	1947.521237	1168.5133	2726.529135	1.222131804	0.066671848	18.3305525	4.72E-75	1.16E-73
ENSG00000235652	AL356599.1	19.93795497	12.038879	27.83703128	1.214951631	0.530403877	2.29061604	0.021986	0.04585
ENSG00000267470	ZNF571-AS1	26.1335641	15.745931	36.52119692	1.213927631	0.461663148	2.62946617	0.008552	0.019384
ENSG00000084112	SSH1	6982.169249	4206.8352	9757.50334	1.213726428	0.044415409	27.3266973	2.04E-164	1.11E-162
ENSG00000258976	AC013451.2	44.94811899	27.06409	62.83214843	1.211884682	0.342979616	3.53340148	0.00041	0.00114
ENSG00000270164	LINC01480	54.52511623	32.91235	76.13788271	1.211613186	0.299364006	4.04729079	5.18E-05	0.000161
ENSG00000260852	FBXL19-AS1	133.3886283	80.379012	186.3982445	1.211526905	0.215628032	5.61859651	1.93E-08	8.19E-08
ENSG00000198947	DMD	862.7610551	520.48697	1205.03514	1.211014812	0.080848721	14.9787751	1.01E-50	1.72E-49
ENSG00000105643	ARRDC2	912.6301579	550.81312	1274.447195	1.210317848	0.086072317	14.061639	6.54E-45	9.95E-44
ENSG00000113583	C5orf15	2261.887756	1366.2971	3157.478376	1.209109796	0.059478839	20.3284026	7.21E-92	2.19E-90
ENSG00000140332	TLE3	2721.088097	1643.2899	3798.886306	1.208373235	0.060453659	19.9884218	6.95E-89	2.03E-87
ENSG00000237264	FTH1P11	73.58748185	44.622384	102.5525799	1.20464727	0.283594167	4.24778578	2.16E-05	6.99E-05
ENSG00000203930	LINC00632	148.2708208	89.793864	206.7477776	1.204242845	0.18834092	6.39395222	1.62E-10	7.97E-10
ENSG00000136867	SLC31A2	339.0999438	205.4394	472.7604915	1.204150202	0.130676363	9.21475144	3.12E-20	2.46E-19
ENSG00000196739	COL27A1	3043.331816	1843.258	4243.405615	1.203661617	0.079619904	15.1175969	1.24E-51	2.13E-50
ENSG00000213928	IRF9	23.08011834	14.043195	32.11704214	1.20273566	0.520408586	2.31113724	0.020825	0.043683
ENSG00000176788	BASP1	10482.37774	6356.9519	14607.80358	1.200057212	0.062926247	19.0708531	4.41E-81	1.18E-79
ENSG00000110218	PANX1	2069.744492	1256.3648	2883.124181	1.198650967	0.061250081	19.569786	2.80E-85	7.86E-84
ENSG00000174021	GNG5	476.1425763	288.78833	663.4968193	1.198412544	0.115169642	10.4056288	2.34E-25	2.20E-24
ENSG00000067066	SP100	9865.050274	5990.579	13739.52159	1.19760545	0.038584126	31.0388128	1.62E-211	1.15E-209
ENSG00000119938	PPP1R3C	162.012058	98.31472	225.7093956	1.197342302	0.179615679	6.66613467	2.63E-11	1.37E-10
ENSG00000214562	NUTM2D	78.39822989	47.600602	109.1958573	1.196506799	0.258517168	4.6283456	3.69E-06	1.29E-05
ENSG00000175197	DDIT3	292.639844	177.98576	407.2939233	1.195987446	0.154065713	7.76283978	8.30E-15	5.17E-14
ENSG00000121578	B4GALT4	301.5491949	183.32388	419.7745129	1.195524835	0.136636855	8.74965129	2.14E-18	1.57E-17
ENSG00000240032	LNCSRLR	36.21397197	21.990993	50.43695066	1.19517683	0.376776949	3.17210708	0.001513	0.003881
ENSG00000146072	TNFRSF21	7514.001422	4570.5755	10457.42731	1.194255835	0.053566521	22.2948179	4.15E-110	1.50E-108
ENSG00000102921	AC026470.1	1989.104985	1210.9164	2767.293617	1.192541185	0.059869891	19.9188803	2.79E-88	8.08E-87
ENSG00000077684	JADE1	1642.85599	999.73962	2285.972364	1.192292665	0.074461079	16.0122936	1.05E-57	1.98E-56
ENSG00000160058	BSDC1	4580.469575	2789.6477	6371.291442	1.19172175	0.046365577	25.7027265	1.09E-145	5.21E-144
ENSG00000219507	FTH1P8	103.1042971	62.745471	143.4631228	1.191266151	0.219523787	5.42659258	5.74E-08	2.35E-07
ENSG00000162729	IGSF8	727.8185912	443.42683	1012.210357	1.190958266	0.088554812	13.448826	3.13E-41	4.42E-40
ENSG00000122786	CALD1	26695.58998	16266.754	37124.42644	1.190549861	0.043595006	27.3093178	3.29E-164	1.77E-162
ENSG00000109107	ALDOC	180.0588174	109.89265	250.2249896	1.189366101	0.172315359	6.902264	5.12E-12	2.78E-11
ENSG00000196878	LAMB3	1848.083919	1126.3262	2569.8416	1.189045072	0.077022926	15.4375474	9.15E-54	1.63E-52
ENSG00000197182	MIRLET7BHG	156.6378459	95.530609	217.7451659	1.188967845	0.181476509	6.551635	5.69E-11	2.89E-10
ENSG00000125148	MT2A	45992.12409	28044.653	63939.59549	1.188960338	0.105418012	11.2785312	1.68E-29	1.77E-28
ENSG00000196876	SCN8A	569.5691889	347.2709	791.8674788	1.188383008	0.100863734	11.7820644	4.83E-32	5.46E-31
ENSG00000137285	TUBB2B	95.04907928	58.036246	132.0619122	1.187626987	0.231997765	5.11913115	3.07E-07	1.19E-06
ENSG00000131871	SELENOS	2037.447947	1243.79	2831.105938	1.186853278	0.058971705	20.1258092	4.39E-90	1.30E-88
ENSG00000205562	AL049775.1	132.0114503	80.538623	183.4842779	1.185604542	0.212867775	5.56967602	2.55E-08	1.07E-07
ENSG00000279329	AC020910.5	21.82446819	13.323566	30.32537059	1.18491918	0.505021956	2.3462726	0.018962	0.040174
ENSG00000119771	KLHL29	948.6067101	580.6654	1316.548019	1.181088968	0.08592746	13.7451865	5.44E-43	8.01E-42
ENSG00000164308	ERAP2	424.8638144	260.2497	589.477929	1.180833487	0.127039347	9.29502169	1.47E-20	1.17E-19
ENSG00000242588	AC108010.1	48.85558947	29.98645	67.72472934	1.179712702	0.32453122	3.63512855	0.000278	0.000791
ENSG00000163132	MSX1	315.6609168	193.35844	437.9633939	1.178814964	0.147503339	7.99178492	1.33E-15	8.62E-15
ENSG00000179862	CITED4	367.4776839	225.44853	509.5068406	1.175161622	0.163132202	7.20373788	5.86E-13	3.33E-12
ENSG00000118058	KMT2A	6336.045761	3894.2085	8777.882989	1.172545904	0.047907163	24.4753775	2.70E-132	1.19E-130
ENSG00000184208	C22orf46	329.0032703	202.56225	455.4442956	1.171829658	0.14536235	8.0614386	7.54E-16	4.94E-15
ENSG00000122641	INHBA	5649.876473	3476.7527	7823.000229	1.170114452	0.116896593	10.0098251	1.38E-23	1.22E-22
ENSG00000272221	AL645933.2	82.03524845	50.432989	113.6375077	1.169064352	0.250255659	4.67148019	2.99E-06	1.06E-05
ENSG00000163738	MTHFD2L	374.8933799	230.86413	518.9226274	1.168989451	0.126588835	9.23453832	2.59E-20	2.05E-19
ENSG00000132109	TRIM21	1824.977528	1124.0484	2525.906667	1.168278706	0.060954712	19.1663395	7.07E-82	1.91E-80
ENSG00000155287	SLC25A28	678.0834281	418.95657	937.2102812	1.161887445	0.091791453	12.6579045	1.01E-36	1.29E-35
ENSG00000205746	AC126755.1	123.9972885	76.590574	171.404003	1.160808224	0.201130011	5.7714322	7.86E-09	3.43E-08
ENSG00000166257	SCN3B	140.8137663	87.04348	194.5840521	1.159110389	0.200439067	5.78285665	7.34E-09	3.21E-08

ENSG00000145247	OCIAD2	2716.918794	1680.4173	3753.420245	1.159049661	0.060572948	19.1347739	1.30E-81	3.49E-80
ENSG00000115548	KDM3A	1683.775287	1042.2134	2325.337177	1.158444762	0.071756507	16.1441076	1.25E-58	2.40E-57
ENSG00000054967	RELT	572.3712967	354.48396	790.2586329	1.155638802	0.098711817	11.7071981	1.17E-31	1.31E-30
ENSG00000183426	NPIPA1	177.9845741	110.5169	245.4522478	1.154481548	0.183858281	6.27919255	3.40E-10	1.64E-09
ENSG00000223804	AC244669.1	24.54280501	15.214392	33.87121808	1.154218915	0.441667089	2.61332334	0.008967	0.020246
ENSG00000171016	PYGO1	86.55058216	53.747225	119.3539393	1.15419695	0.269820186	4.27765235	1.89E-05	6.16E-05
ENSG00000283050	GTF2IP12	74.83290487	46.393875	103.2719345	1.15092805	0.274993179	4.18529672	2.85E-05	9.12E-05
ENSG00000087245	MMP2	48838.4155	30354.16	67322.67117	1.149224282	0.045114965	25.4732391	3.90E-143	1.83E-141
ENSG00000175505	CLCF1	145.0163867	90.083639	199.9491348	1.149207686	0.190944183	6.018553	1.76E-09	8.06E-09
ENSG00000179104	TMTC2	318.885181	198.44765	439.3227132	1.148758204	0.140241213	8.19130256	2.58E-16	1.73E-15
ENSG00000117475	BLZF1	1263.54385	786.35502	1740.732679	1.145959417	0.071931098	15.9313488	3.84E-57	7.17E-56
ENSG00000210195	MT-TT	96.98738967	60.353559	133.6212203	1.145528669	0.231813758	4.94159052	7.75E-07	2.89E-06
ENSG00000136717	BIN1	3441.784402	2143.6084	4739.960359	1.144183547	0.069219705	16.5297373	2.24E-61	4.49E-60
ENSG00000175746	C15orf54	849.1555361	528.94811	1169.362966	1.144073777	0.100661199	11.3655886	6.20E-30	6.65E-29
ENSG00000167549	CORO6	62.18335966	38.713721	85.65299862	1.14401334	0.335822033	3.40660596	0.000658	0.001778
ENSG00000139278	GLIPR1	5357.600162	3339.2177	7375.982647	1.143042001	0.045433612	25.1585105	1.14E-139	5.19E-138
ENSG00000124212	PTGIS	616.5203353	384.67308	848.367595	1.142243821	0.103865309	10.9973564	3.93E-28	4.02E-27
ENSG00000204390	HSPA1L	45.38880493	28.296961	62.47912043	1.142122983	0.327533652	3.48704012	0.000488	0.001346
ENSG00000163932	PRKCD	1166.72127	727.32666	1606.115878	1.141445561	0.086316066	13.2240221	6.38E-40	8.73E-39
ENSG00000198742	SMURF1	2237.645607	1395.6233	3079.667884	1.141323381	0.060304377	18.9260455	6.96E-80	1.83E-78
ENSG00000109743	BST1	518.6365005	323.79264	713.4803629	1.138926613	0.104240511	10.92595	8.66E-28	8.78E-27
ENSG00000224982	TMEM233	188.8809083	117.9146	259.8472178	1.13847605	0.174983156	6.50620364	7.71E-11	3.88E-10
ENSG00000105792	CFAP69	49.52068476	30.965694	68.07567535	1.138301871	0.337643941	3.37130845	0.000748	0.002008
ENSG00000117602	RCAN3	227.1913252	141.94682	312.4358268	1.137557013	0.156544824	7.26665363	3.69E-13	2.11E-12
ENSG00000137094	DNAJB5	1031.190246	644.77467	1417.605824	1.1369814	0.077356152	14.6980088	6.64E-49	1.09E-47
ENSG00000137802	MAPKBP1	2941.305481	1838.7088	4043.902197	1.1367596	0.054687475	20.7864706	5.74E-96	1.81E-94
ENSG00000205181	LINC00654	97.03331739	60.721444	133.3451904	1.136704905	0.229517276	4.95258974	7.32E-07	2.74E-06
ENSG00000172432	GTPBP2	2485.953777	1554.848	3417.059525	1.136005394	0.055963527	20.2990315	1.31E-91	3.96E-90
ENSG00000163013	FBXO41	99.95774497	62.434184	137.4813061	1.133767116	0.246975143	4.59061224	4.42E-06	1.53E-05
ENSG00000155363	MOV10	3941.390773	2467.5822	5415.199354	1.133686236	0.050071656	22.6412771	1.70E-113	6.34E-112
ENSG00000111981	ULBP1	246.0409854	154.28681	337.7951635	1.130934207	0.143003889	7.90841573	2.61E-15	1.66E-14
ENSG00000062716	VMP1	3838.344628	2407.4218	5269.267408	1.130733447	0.056430333	20.0376885	2.58E-89	7.61E-88
ENSG00000250251	PKD1P6	238.7815367	150.13925	327.4238223	1.127988107	0.160401335	7.03228629	2.03E-12	1.13E-11
ENSG00000175106	TVP23C	137.3825638	86.182034	188.5830936	1.12790809	0.19363156	5.82502195	5.71E-09	2.52E-08
ENSG00000197951	ZNF71	824.852293	518.17642	1131.52817	1.125781065	0.105382693	10.6827889	1.23E-26	1.20E-25
ENSG00000125744	RTN2	495.2019786	311.43262	678.9713373	1.124042037	0.111728752	10.0604546	8.26E-24	7.38E-23
ENSG00000165568	AKR1E2	27.78941877	17.465104	38.11373333	1.123099786	0.437389158	2.56773577	0.010237	0.022855
ENSG00000136883	KIF12	49.774695	31.349571	68.19981927	1.122628197	0.343929186	3.26412599	0.001098	0.002878
ENSG00000175471	MCTP1	3941.393159	2483.163	5399.623314	1.121092134	0.072174551	15.5330671	2.07E-54	3.71E-53
ENSG00000137941	TTLL7	511.9166329	322.75287	701.0803917	1.120438459	0.120009205	9.33627095	9.98E-21	7.99E-20
ENSG00000092148	HECTD1	14359.77697	9050.0217	19669.5322	1.120029592	0.041558743	26.9505163	5.63E-160	2.90E-158
ENSG00000090661	CERS4	143.9151474	90.820027	197.0102678	1.118701894	0.195709501	5.71613484	1.09E-08	4.70E-08
ENSG00000132669	RIN2	4577.725818	2889.9891	6265.462583	1.116605284	0.051686229	21.6035356	1.66E-103	5.61E-102
ENSG00000270629	NBPF14	417.697136	263.90476	571.4895148	1.116542884	0.122956585	9.08078964	1.08E-19	8.33E-19
ENSG00000168961	LGALS9	1860.128346	1174.5924	2545.664337	1.115744134	0.072168203	15.4603287	6.43E-54	1.15E-52
ENSG00000225407	AC025188.1	32.98431996	20.887459	45.08118108	1.115347905	0.414775995	2.68903678	0.007166	0.016457
ENSG00000011028	AC080038.1	10228.43006	6465.1683	13991.69185	1.113904894	0.04894518	22.7582143	1.19E-114	4.49E-113
ENSG00000137767	SQOR	653.1674059	412.83905	893.4957633	1.113421004	0.091297533	12.1955213	3.28E-34	3.89E-33
ENSG00000173210	ABLIM3	6471.860824	4092.2626	8851.459021	1.11276809	0.045921822	24.2317933	1.03E-129	4.46E-128
ENSG00000230487	PSMG3-AS1	207.5684951	131.41407	283.7229223	1.112357855	0.171745323	6.47678687	9.37E-11	4.69E-10
ENSG00000166046	TCP11L2	387.9886788	245.68635	530.2910031	1.110501747	0.123581915	8.98595679	2.56E-19	1.95E-18
ENSG00000169902	TPST1	1425.998582	902.61749	1949.379672	1.110296671	0.067349263	16.4856544	4.65E-61	9.24E-60
ENSG00000158286	RNF207	63.94674986	40.568988	87.32451179	1.110225147	0.298173817	3.72341595	0.000197	0.000568
ENSG00000170525	PFKFB3	7949.273726	5035.4025	10863.14497	1.10894874	0.045294006	24.4833441	2.22E-132	9.80E-131
ENSG00000234465	PINLYP	40.25278984	25.571308	54.93427207	1.108544667	0.372499311	2.97596434	0.002921	0.00717
ENSG00000196116	TDRD7	1409.86688	893.65953	1926.074231	1.108311516	0.069451326	15.9581045	2.50E-57	4.68E-56
ENSG00000108797	CNTNAP1	7951.788103	5040.3583	10863.21788	1.107611374	0.047164579	23.4839662	5.95E-122	2.40E-120
ENSG00000120008	WDR11	4503.809797	2856.8254	6150.794211	1.106716854	0.048817573	22.6704602	8.77E-114	3.28E-112
ENSG00000138769	CDKL2	30.77329717	19.591232	41.95536197	1.103721763	0.409363455	2.69619027	0.007014	0.016125
ENSG00000155962	CLIC2	141.5928989	89.976906	193.2088919	1.102292647	0.191420505	5.75848781	8.49E-09	3.69E-08
ENSG00000214176	PLEKHM1P1	245.3564932	156.28726	334.4257267	1.101076826	0.170104876	6.47292922	9.61E-11	4.81E-10
ENSG00000110330	BIRC2	3377.412367	2148.7588	4606.065939	1.100572974	0.05293902	20.7894476	5.39E-96	1.71E-94
ENSG00000215252	GOLGA8B	51.90051366	33.047087	70.75394002	1.100303288	0.390473628	2.81786838	0.004834	0.011434
ENSG00000188015	S100A3	172.9383443	109.93726	235.9394299	1.099250926	0.197452294	5.56717223	2.59E-08	1.09E-07
ENSG00000269352	PTOV1-AS2	43.59791536	27.816326	59.37950515	1.098957783	0.381391448	2.88144317	0.003959	0.009517
ENSG00000111711	GOLT1B	1410.741368	898.53264	1922.950091	1.098142088	0.068208818	16.0997085	2.56E-58	4.89E-57
ENSG00000167772	ANGPTL4	3770.912276	2402.5538	5139.270797	1.096483047	0.053852127	20.3609978	3.71E-92	1.13E-90
ENSG00000160111	CPAMD8	70.84020893	45.134564	96.5458534	1.095185752	0.265769002	4.12081824	3.78E-05	0.000119
ENSG00000162433	AK4	1406.106069	897.12337	1915.088772	1.094051621	0.073698775	14.8449092	7.51E-50	1.26E-48
ENSG00000224080	UBE2FP1	52.22184509	33.293778	71.14991229	1.092827611	0.306274131	3.56813554	0.00036	0.001006
ENSG00000180596	HIST1H2BC	46.73955146	29.851418	63.62768508	1.092194921	0.341207183	3.20097282	0.00137	0.003537
ENSG00000141574	SECTM1	155.4985629	99.243061	211.7540643	1.090963722	0.187296009	5.82481029	5.72E-09	2.52E-08
ENSG00000140859	KIFC3	2843.795795	1817.9893	3869.6023	1.08932526	0.061737646	17.6444249	1.12E-69	2.57E-68
ENSG00000086289	EPDR1	51.81025857	33.250076	70.37044089	1.089063906	0.362352899	3.00553906	0.002651	0.00655
ENSG00000186480	INSIG1	3575.300934	2287.6995	4862.902319	1.087455169	0.059884852	18.1591026	1.09E-73	2.66E-72
ENSG00000189420	ZFP92	140.5815899	90.052891	191.1102883	1.084948006	0.20050851	5.41098234	6.27E-08	2.56E-07
ENSG00000273174	AC108673.2	51.63433809	33.019406	70.24927019	1.081827625	0.364904242	2.96468909	0.00303	0.007418
ENSG00000091656	ZFHX4	883.1762116	567.71459	1198.637835	1.078496319	0.097179599	11.0979705	1.28E-28	1.33E-27
ENSG00000138135	CH25H	40.18044582	25.857836	54.50305521	1.076811174	0.383401938	2.80856999	0.004976	0.011739
ENSG00000137872	SEMA6D	351.3566036	226.58468	476.1285309	1.072673811	0.162353066	6.60704376	3.92E-11	2.02E-10
ENSG00000228315	GUSBP11	46.45077496	30.021505	62.88004461	1.071330463	0.355186813	3.01624504	0.002559	0.006335
ENSG00000254681	PKD1P5	365.5250319	236.0164	495.0336619	1.070756079	0.145739622	7.34704858	2.03E-13	1.18E-12
ENSG00000114127	XRN1	2885.324967	1862.0232	3908.626756	1.070307173	0.062947171	17.0032609	7.77E-65	1.66E-63
ENSG00000155380	SLC16A1	6745.419174	4357.5387	9133.299695	1.067454272	0.044061929	24.2262266	1.18E-129	5.09E-128
ENSG00000166224	SGPL1	1236.030949	798.65644	1673.40546	1.066549853	0.072520347	14.7069049	5.82E-49	9.56E-48

ENSG00000175356	SCUBE2	28.95669112	18.803617	39.10976493	1.065763834	0.451678447	2.35956318	0.018296	0.038908
ENSG00000197461	PDGFA	2317.890109	1499.6259	3136.154317	1.064581702	0.059750986	17.8169729	5.22E-71	1.22E-69
ENSG00000164430	CGAS	168.5722604	109.177	227.9675192	1.064188732	0.185636067	5.73266148	9.89E-09	4.28E-08
ENSG00000145860	RNF145	5954.53055	3853.5086	8055.552523	1.063846008	0.042248187	25.1808679	6.49E-140	2.96E-138
ENSG00000153560	UBP1	4892.439277	3167.3518	6617.526799	1.063224662	0.048324623	22.0017167	2.77E-107	9.74E-106
ENSG00000130707	ASS1	179.5880152	116.43793	242.7381009	1.061889002	0.187757986	5.65562628	1.55E-08	6.64E-08
ENSG00000169891	REPS2	373.2283473	241.75908	504.6976154	1.06178026	0.120362239	8.82153961	1.13E-18	8.37E-18
ENSG00000120217	CD274	2054.578276	1331.3881	2777.768469	1.061079859	0.058072935	18.271504	1.40E-74	3.43E-73
ENSG00000118515	SGK1	8607.438861	5583.2721	11631.60558	1.058590527	0.05557538	19.0478323	6.85E-81	1.82E-79
ENSG00000197965	MPZL1	7721.957999	5010.09	10433.82596	1.058347321	0.04124631	25.6592001	3.34E-145	1.59E-143
ENSG00000112210	RAB23	1050.547698	681.75325	1419.342143	1.058292193	0.081633	12.9640243	1.96E-38	2.59E-37
ENSG00000268912	AC012313.5	26.66504077	17.341906	35.98817554	1.057958574	0.462733155	2.28632542	0.022235	0.046309
ENSG00000100647	SUSD6	2530.444051	1642.7125	3418.175639	1.056823488	0.0539845	19.5764244	2.46E-85	6.91E-84
ENSG00000153214	TMEM87B	2989.359414	1942.7915	4035.927311	1.054961757	0.055499212	19.0085899	1.45E-80	3.83E-79
ENSG00000166348	USP54	778.5466863	505.95181	1051.141563	1.054145948	0.087684871	12.0219821	2.72E-33	3.18E-32
ENSG00000176845	METRNL	1027.192841	667.78772	1386.597957	1.054131519	0.080780576	13.0493192	6.41E-39	8.58E-38
ENSG00000131015	ULBP2	608.572364	395.36488	821.7798455	1.054031742	0.099964061	10.5441069	5.41E-26	5.19E-25
ENSG00000168679	SLC16A4	195.826372	127.48741	264.1653338	1.051830772	0.163587732	6.4297656	1.28E-10	6.34E-10
ENSG00000113580	NR3C1	1706.856586	1110.942	2302.771134	1.051617311	0.061017674	17.2346347	1.46E-66	3.21E-65
ENSG00000122884	P4HA1	2695.878103	1754.4431	3637.313071	1.051555946	0.055777617	18.852651	2.79E-79	7.30E-78
ENSG00000099992	TBC1D10A	266.9162384	173.82498	360.0074949	1.05016236	0.138903168	7.5603917	4.02E-14	2.41E-13
ENSG00000184014	DENND5A	8607.363871	5606.0114	11608.71638	1.050030098	0.041806554	25.1163989	3.29E-139	1.49E-137
ENSG00000187479	C11orf96	231.1016176	150.47792	311.7253167	1.050010768	0.176082672	5.9631692	2.47E-09	1.12E-08
ENSG00000100902	PSMA6	111.9279394	72.955198	150.9006804	1.04939072	0.216976604	4.83642338	1.32E-06	4.84E-06
ENSG00000143995	MEIS1	537.6057293	350.2019	725.0095537	1.04876578	0.11002766	9.53183755	1.55E-21	1.27E-20
ENSG00000075420	FNDC3B	26980.36829	17585.587	36375.14946	1.048583899	0.039940732	26.2534975	6.52E-152	3.20E-150
ENSG00000153790	C7orf31	138.592053	90.333252	186.8508542	1.047486616	0.190532235	5.49768714	3.85E-08	1.60E-07
ENSG00000204271	SPIN3	131.2142088	85.658788	176.7696295	1.046715472	0.199103964	5.25713025	1.46E-07	5.79E-07
ENSG00000083223	TUT7	2071.794099	1351.6539	2791.934312	1.046610881	0.060264624	17.3669195	1.47E-67	3.27E-66
ENSG00000086062	B4GALT1	6795.952809	4434.204	9157.701601	1.046150328	0.046005268	22.7397942	1.81E-114	6.80E-113
ENSG00000273117	AC144652.1	40.90346105	26.612076	55.19484621	1.046119903	0.373998564	2.79712278	0.005156	0.012132
ENSG00000008735	MAPK8IP2	37.29166493	24.315831	50.26749872	1.045609863	0.378060047	2.76572431	0.00568	0.013277
ENSG00000157833	GAREM2	363.5004135	237.18604	489.8147911	1.044416996	0.136951005	7.62620908	2.42E-14	1.47E-13
ENSG00000131323	TRAF3	2433.104179	1590.211	3275.997388	1.042468316	0.058572547	17.7978997	7.34E-71	1.71E-69
ENSG00000123094	RASSF8	3621.37829	2368.8958	4873.860758	1.041291978	0.050986728	20.4228046	1.05E-92	3.21E-91
ENSG00000213859	KCTD11	547.7865711	358.4028	737.1703436	1.039798486	0.100609301	10.3350135	4.89E-25	4.55E-24
ENSG00000100503	NIN	7520.194001	4925.6559	10114.73211	1.038183178	0.040992018	25.3264718	1.63E-141	7.52E-140
ENSG00000168026	TTC21A	85.05940347	55.742156	114.3766512	1.038138371	0.246530305	4.21099698	2.54E-05	8.18E-05
ENSG00000171729	TMEM51	963.3261627	630.92491	1295.727419	1.038125955	0.083008182	12.5063088	6.90E-36	8.59E-35
ENSG00000282057	AC092807.3	103.4529802	67.906387	138.9995735	1.035824096	0.223982578	4.62457441	3.75E-06	1.31E-05
ENSG00000168234	TTC39C	197.6016212	129.43684	265.7664048	1.035798204	0.167965602	6.16672814	6.97E-10	3.28E-09
ENSG00000169594	BNC1	1029.852995	675.33406	1384.371927	1.034621981	0.095745589	10.8059493	3.23E-27	3.21E-26
ENSG00000223361	FTH1P10	106.6901331	69.975178	143.4050881	1.034079838	0.237910247	4.34651239	1.38E-05	4.58E-05
ENSG00000196611	MMP1	508.0111571	333.37138	682.650932	1.032637106	0.12232772	8.44156261	3.13E-17	2.18E-16
ENSG00000121653	MAPK8IP1	286.9524215	188.72101	385.1838369	1.030589205	0.138601221	7.43564304	1.04E-13	6.12E-13
ENSG00000221890	NPTXR	97.36602131	64.02417	130.7078726	1.03027902	0.239528219	4.30128451	1.70E-05	5.57E-05
ENSG00000134954	ETS1	8372.762969	5507.2144	11238.31157	1.029270094	0.044344064	23.2110008	3.53E-119	1.39E-117
ENSG00000198108	CHSY3	71.72381311	47.320507	96.1271191	1.028824424	0.314063589	3.2758475	0.001053	0.002769
ENSG00000115520	COQ10B	1057.677948	696.4236	1418.9323	1.027900087	0.080461818	12.7750045	2.26E-37	2.92E-36
ENSG00000116017	ARID3A	448.3207926	295.05089	601.5906913	1.027387351	0.107385075	9.56731977	1.10E-21	9.08E-21
ENSG00000154556	SORBS2	7441.338726	4901.572	9981.105449	1.025999513	0.055506556	18.4842943	2.76E-76	6.89E-75
ENSG00000180573	HIST1H2AC	264.8790146	174.57751	355.180517	1.025427257	0.142337412	7.2042005	5.84E-13	3.32E-12
ENSG00000018869	ZNF582	50.71751005	33.468174	67.96684634	1.024923545	0.331911835	3.08793913	0.002015	0.005069
ENSG00000160179	ABCG1	2744.366092	1810.1285	3678.603719	1.022595328	0.05519794	18.5259692	1.27E-76	3.20E-75
ENSG00000132359	RAP1GAP2	615.9345739	406.55495	825.3141946	1.022285287	0.112453928	9.09070324	9.84E-20	7.61E-19
ENSG00000182541	LIMK2	3447.228514	2274.6701	4619.78691	1.021947651	0.051618401	19.7981268	3.09E-87	8.88E-86
ENSG00000173846	PLK3	988.6951295	652.62022	1324.770037	1.021259332	0.084260071	12.1203237	8.24E-34	9.69E-33
ENSG00000171649	ZIK1	53.79927148	35.578928	72.01961468	1.020935086	0.323286987	3.15798386	0.001589	0.004059
ENSG00000151327	FAM177A1	1633.439014	1078.9617	2187.916345	1.019749962	0.065578224	15.5501309	1.59E-54	2.85E-53
ENSG00000139597	N4BP2L1	46.0204778	30.424475	61.61648011	1.019058668	0.323686126	3.1482927	0.001642	0.004182
ENSG00000050344	NFE2L3	4761.032709	3146.5307	6375.534727	1.018705222	0.046007261	22.1422707	1.24E-108	4.41E-107
ENSG00000130309	COLGALT1	24181.15924	15990.2	32372.11846	1.017473468	0.039821969	25.5505563	5.41E-144	2.56E-142
ENSG00000273270	AC090114.2	154.4126662	102.01041	206.8149189	1.017081003	0.198585909	5.12161718	3.03E-07	1.17E-06
ENSG00000126561	STAT5A	802.5446334	531.20758	1073.881682	1.016831942	0.095781461	10.6161665	2.51E-26	2.43E-25
ENSG00000168386	FILIP1L	4620.684158	3057.3715	6183.996794	1.016617933	0.06005203	16.9289518	2.75E-64	5.82E-63
ENSG00000178764	ZHX2	470.113361	311.26917	628.957547	1.015578592	0.121824521	8.33640537	7.66E-17	5.25E-16
ENSG00000116663	FBXO6	203.2242727	134.52395	271.9245946	1.014043904	0.186153519	5.44735285	5.11E-08	2.11E-07
ENSG00000119729	RHOQ	3608.968278	2391.2312	4826.705404	1.013149431	0.056777684	17.8441485	3.21E-71	7.56E-70
ENSG00000141526	SLC16A3	7270.179787	4817.0982	9723.261353	1.01296958	0.056666242	17.8760677	1.81E-71	4.29E-70
ENSG00000196776	CD47	2496.123714	1654.324	3337.923399	1.012779362	0.053852653	18.8067609	6.65E-79	1.72E-77
ENSG00000073150	PANX2	245.2208667	162.42467	328.0170637	1.012766355	0.164875539	6.14261134	8.12E-10	3.80E-09
ENSG00000143801	PSEN2	802.7616619	531.86178	1073.66154	1.012309731	0.088181851	11.4797968	1.67E-30	1.81E-29
ENSG00000254612	AP001000.1	72.08816501	47.883885	96.29244462	1.009809037	0.259965521	3.88439603	0.000103	0.000307
ENSG00000137267	TUBB2A	1220.795246	809.88601	1631.704483	1.009766585	0.078604422	12.8461803	9.04E-38	1.18E-36
ENSG00000100417	PMM1	258.9038842	171.9228	345.8849721	1.008132017	0.141061663	7.146747	8.89E-13	5.01E-12
ENSG00000073849	ST6GAL1	12692.79563	8434.2421	16951.34914	1.007097974	0.036225376	27.8008974	4.23E-170	2.37E-168
ENSG00000158683	PKD1L1	1061.186158	705.52303	1416.849284	1.007011747	0.087737927	11.4774964	1.71E-30	1.86E-29
ENSG00000196998	WDR45	893.5163015	593.5934	1193.439203	1.006913715	0.082199635	12.249613	1.69E-34	2.02E-33
ENSG00000204923	FBXO48	66.46133492	44.225905	88.69676443	1.006113515	0.273108224	3.68393708	0.00023	0.00066
ENSG00000169330	MINAR1	91.19576221	60.610403	121.7811213	1.005362776	0.276390351	3.63747421	0.000275	0.000784
ENSG00000176597	B3GNT5	3865.293518	2573.1081	5157.478894	1.002864194	0.057338573	17.4902189	1.70E-68	3.84E-67
ENSG00000148655	LRMDA	52.75412017	35.24011	70.26813021	1.001674999	0.324594981	3.08592264	0.002029	0.005098
ENSG00000214021	TTLL3	118.9192388	79.419865	158.4186122	1.000774073	0.26083172	3.83685724	0.000125	0.00037
ENSG00000164736	SOX17	2128.091175	1417.8256	2838.356779	1.000508555	0.072210547	13.8554352	1.18E-43	1.76E-42
ENSG00000006118	TMEM132A	13555.34341	9037.8601	18072.82676	0.999771958	0.043605376	22.9277226	2.46E-116	9.43E-115

ENSG00000281649	EBLN3P	1644.17479	1096.7701	2191.579508	0.99963322	0.072970035	13.699229	1.03E-42	1.50E-41
ENSG00000160213	CSTB	7079.64769	4727.7948	9431.500578	0.995964915	0.050436664	19.7468436	8.54E-87	2.43E-85
ENSG00000174796	THAP6	162.8693565	108.86031	216.8783992	0.99583761	0.190434624	5.22928862	1.70E-07	6.69E-07
ENSG00000166532	RIMKLB	922.107453	616.85602	1227.358889	0.994180735	0.105766072	9.39980766	5.47E-21	4.42E-20
ENSG00000110031	LPXN	551.6994306	368.58874	734.8101243	0.99371682	0.11907491	8.34530817	7.10E-17	4.88E-16
ENSG00000259877	AC009113.1	29.72436551	19.912523	39.53620832	0.993349117	0.421933309	2.35427992	0.018559	0.039403
ENSG00000214944	ARHGEF28	6821.702335	4567.9651	9075.439591	0.990679791	0.05107892	19.3950811	8.49E-84	2.35E-82
ENSG00000107863	ARHGAP21	8035.783718	5380.8825	10690.6849	0.990364123	0.045439235	21.7953522	2.57E-105	8.87E-104
ENSG00000243147	MRPL33	1541.289636	1032.0153	2050.563948	0.990246873	0.068628987	14.4289886	3.40E-47	5.42E-46
ENSG00000171223	JUNB	1302.151703	871.91439	1732.389012	0.989846056	0.084075451	11.7733065	5.36E-32	6.03E-31
ENSG00000160209	PDXK	2401.337444	1608.3986	3194.276269	0.989270455	0.063069347	15.6854399	1.90E-55	3.46E-54
ENSG00000159202	UBE2Z	5948.133018	3987.5478	7908.718248	0.987629936	0.046377494	21.2954573	1.25E-100	4.10E-99
ENSG00000278948	AL031587.5	774.5718856	519.37405	1029.769721	0.987061424	0.106721778	9.24892226	2.27E-20	1.80E-19
ENSG00000120686	UFM1	2928.534417	1964.8378	3892.231	0.986623485	0.055490664	17.7799907	1.01E-70	2.34E-69
ENSG00000163644	PPM1K	343.3453612	230.47686	456.2138631	0.985055583	0.133605303	7.37287788	1.67E-13	9.72E-13
ENSG00000272269	AL138724.1	108.866635	73.075768	144.6575017	0.983758674	0.22340616	4.40345367	1.07E-05	3.56E-05
ENSG00000227827	AC138969.2	74.87656288	50.304391	99.44873486	0.983344804	0.289324702	3.39875855	0.000677	0.001827
ENSG00000161091	MFSD12	2509.04837	1685.4165	3332.680221	0.983122421	0.05962045	16.4896848	4.35E-61	8.65E-60
ENSG00000178078	STAP2	178.02262	119.7431	236.3021362	0.983030502	0.176622535	5.56571391	2.61E-08	1.10E-07
ENSG00000196268	ZNF493	78.46012997	52.878993	104.0412673	0.982573528	0.31713543	3.09827738	0.001946	0.004906
ENSG00000157111	TMEM171	494.1848771	331.75537	656.6143886	0.98250658	0.133281602	7.37165946	1.69E-13	9.81E-13
ENSG00000126458	RRAS	3201.008955	2152.3565	4249.66137	0.980987823	0.052854865	18.560029	6.77E-77	1.70E-75
ENSG00000204264	PSMB8	1657.80663	1114.8969	2200.716397	0.980858571	0.077391892	12.6739191	8.25E-37	1.05E-35
ENSG00000065526	SPEN	5462.550913	3673.9166	7251.185193	0.980786655	0.050732019	19.332695	2.85E-83	7.81E-82
ENSG00000272695	GAS6-DT	123.1577801	82.942075	163.3734857	0.977154024	0.215339177	4.53774385	5.69E-06	1.95E-05
ENSG00000203879	GDI1	4272.529305	2883.8104	5661.248172	0.972958158	0.049314981	19.7294644	1.20E-86	3.41E-85
ENSG00000198792	TMEM184B	8492.418697	5731.8298	11253.00759	0.972934856	0.053664914	18.1298131	1.85E-73	4.51E-72
ENSG00000169926	KLF13	3002.123153	2027.0753	3977.171042	0.972231436	0.055175605	17.6206756	1.71E-69	3.90E-68
ENSG00000116117	PARD3B	1365.055471	922.0644	1808.046543	0.971824451	0.076663222	12.6765407	7.98E-37	1.02E-35
ENSG00000182179	UBA7	1369.841863	926.48155	1813.202174	0.969252902	0.072204161	13.4237817	4.39E-41	6.18E-40
ENSG00000188002	AC026412.1	88.20012413	59.66592	116.7343282	0.968973925	0.235401544	4.11625985	3.85E-05	0.000122
ENSG00000130779	CLIP1	7276.259925	4921.3698	9631.150039	0.968872258	0.044973513	21.5431773	6.14E-103	2.07E-101
ENSG00000090776	EFNB1	4757.48845	3217.5773	6297.399557	0.968342449	0.049997358	19.3678725	1.44E-83	3.96E-82
ENSG00000090006	LTBP4	1124.645173	760.73919	1488.551152	0.968276753	0.080552537	12.0204378	2.77E-33	3.24E-32
ENSG00000197063	MAFG	2250.522356	1522.5109	2978.533821	0.968160649	0.057999597	16.6925409	1.49E-62	3.05E-61
ENSG00000263823	AC009831.1	40.47546866	27.431975	53.51896243	0.967705301	0.374679044	2.582758	0.009801	0.021967
ENSG00000196639	HRH1	1685.730727	1141.9906	2229.470886	0.965877339	0.068461303	14.1083693	3.37E-45	5.17E-44
ENSG00000102385	DRP2	60.5861543	40.976884	80.19542451	0.964962536	0.316082215	3.05288463	0.002267	0.005656
ENSG00000102802	MEDAG	1151.459148	780.2565	1522.661796	0.964340064	0.072312277	13.3357724	1.43E-40	1.99E-39
ENSG00000276248	AL442125.1	83.83262862	56.811223	110.8540343	0.963678733	0.253196754	3.80604695	0.000141	0.000416
ENSG00000168016	TRANK1	2226.673011	1510.2536	2943.092434	0.963242971	0.060505131	15.9200213	4.60E-57	8.59E-56
ENSG00000143162	CREG1	871.9081419	591.57362	1152.24266	0.962150079	0.085795114	11.2145091	3.46E-29	3.64E-28
ENSG00000170175	CHRNB1	442.5614723	300.20016	584.9227889	0.961983953	0.11634218	8.26857422	1.36E-16	9.21E-16
ENSG00000149633	KIAA1755	40.81360294	27.650072	53.97713346	0.9614428	0.349694186	2.74938171	0.005971	0.013892
ENSG00000110651	CD81	22048.86405	14962.854	29134.87387	0.961283098	0.036472398	26.3564541	4.33E-153	2.13E-151
ENSG00000175984	DENND2C	27.85862549	18.941895	36.77535604	0.961146877	0.422692241	2.27386922	0.022974	0.047689
ENSG00000134294	SLC38A2	22103.33941	15011.29	29195.38835	0.959796307	0.039588124	24.2445516	7.55E-130	3.28E-128
ENSG00000196867	ZFP28	265.0179852	180.01363	350.0223443	0.959778126	0.140904259	6.81156223	9.65E-12	5.15E-11
ENSG00000151388	ADAMTS12	58.41291229	39.650973	77.17485173	0.957122241	0.301154058	3.17818145	0.001482	0.003806
ENSG00000189134	NKAPL	39.42951018	26.777267	52.08175384	0.95704345	0.369402026	2.59079102	0.009576	0.021508
ENSG00000060491	OGFR	2494.696934	1695.8034	3293.59044	0.957038188	0.068342173	14.0036255	1.48E-44	2.24E-43
ENSG00000242802	AP5Z1	2143.953111	1457.4624	2830.443812	0.957035262	0.05937415	16.1187195	1.88E-58	3.61E-57
ENSG00000196208	GREB1	39.66824225	26.948446	52.38803887	0.956794145	0.361001986	2.65038471	0.00804	0.018308
ENSG00000138764	CCNG2	1431.543549	974.06643	1889.02067	0.956660157	0.074703268	12.8061354	1.51E-37	1.96E-36
ENSG00000158528	PPP1R9A	64.30334227	43.800805	84.8058798	0.954789608	0.310721546	3.07281429	0.002121	0.005313
ENSG00000171928	TVP23B	1429.805735	974.133	1885.47847	0.95383604	0.075962453	12.5566778	3.65E-36	4.58E-35
ENSG00000282851	BISPR	162.9817809	111.11905	214.8445074	0.953526292	0.182167393	5.23434122	1.66E-07	6.52E-07
ENSG00000243302	AC018638.4	114.2026078	77.934458	150.4707577	0.952841413	0.224892657	4.23687205	2.27E-05	7.32E-05
ENSG00000188157	AGRN	25278.21957	17236.804	33319.63535	0.950812362	0.038292431	24.8302951	4.22E-136	1.88E-134
ENSG00000226711	FAM66C	30.35603224	20.748119	39.9639458	0.950761271	0.409045752	2.32433967	0.020107	0.042318
ENSG00000234814	SVIL2P	36.85187583	25.106369	48.59738293	0.950744938	0.377657383	2.51748007	0.01182	0.026077
ENSG00000113504	SLC12A7	3480.321824	2372.8533	4587.790365	0.950600727	0.059205345	16.0559951	5.19E-58	9.83E-57
ENSG00000139263	LRIG3	1434.042156	978.03769	1890.046619	0.950572389	0.069184797	13.7396138	5.88E-43	8.65E-42
ENSG00000169826	CSGALNACT2	2058.275861	1403.9985	2712.55319	0.950530975	0.058001585	16.3880174	2.33E-60	4.59E-59
ENSG00000185010	F8	64.44224064	43.984141	84.90034051	0.948686769	0.285475801	3.32317754	0.00089	0.002363
ENSG00000159433	STARD9	831.0560047	567.98079	1094.131221	0.947625322	0.105932167	8.94558611	3.70E-19	2.80E-18
ENSG00000043143	JADE2	2501.004125	1709.0308	3292.977419	0.945859975	0.062492295	15.1356254	9.43E-52	1.62E-50
ENSG00000262001	DLGAP1-AS2	200.3665133	136.82232	263.9107061	0.945713368	0.167418148	5.64881036	1.62E-08	6.90E-08
ENSG00000049246	PER3	124.1838932	84.8532	163.5145861	0.944116785	0.209235301	4.51222514	6.42E-06	2.19E-05
ENSG00000083844	ZNF264	704.4097916	481.79438	927.0252076	0.943028609	0.096439578	9.77843978	1.39E-22	1.19E-21
ENSG00000226419	SLC16A1-AS1	195.7387053	133.92195	257.5554631	0.942951759	0.17592329	5.36001663	8.32E-08	3.36E-07
ENSG00000135341	MAP3K7	2383.796443	1631.9187	3135.674209	0.942640397	0.056023193	16.8258955	1.58E-63	3.27E-62
ENSG00000133794	ARNTL	488.0763152	333.93718	642.2154508	0.942198275	0.107043621	8.80200301	1.34E-18	9.92E-18
ENSG00000008300	CELSR3	93.11970142	63.670567	122.5688362	0.942004478	0.266831412	3.53033577	0.000415	0.001153
ENSG00000130164	LDLR	3884.446346	2660.899	5107.993659	0.940574451	0.06648924	14.1462657	1.97E-45	3.04E-44
ENSG00000166579	NDEL1	1979.91268	1356.6234	2603.201987	0.940252736	0.061942442	15.1794587	4.84E-52	8.38E-51
ENSG00000137821	LRRC49	186.3634424	127.77102	244.9558683	0.939652863	0.174447271	5.3864578	7.19E-08	2.92E-07
ENSG00000130940	CASZ1	218.6360065	149.86659	287.4054244	0.939477127	0.156282223	6.01141389	1.84E-09	8.41E-09
ENSG00000122591	FAM126A	3422.038139	2347.1604	4496.915839	0.938470571	0.054268598	17.2930682	5.31E-67	1.17E-65
ENSG00000164056	SPRY1	954.300463	655.11655	1253.484378	0.937569819	0.114836835	8.16436485	3.23E-16	2.15E-15
ENSG00000160710	ADAR	15530.38806	10657.6	20403.17588	0.936895593	0.038912708	24.0768539	4.37E-128	1.86E-126
ENSG00000100226	GTPBP1	1958.079931	1343.9774	2572.182424	0.935675313	0.064603266	14.4834057	1.54E-47	2.48E-46
ENSG00000186866	POFUT2	3686.607433	2531.6504	4841.564428	0.935445399	0.053221886	17.5763294	3.74E-69	8.50E-68
ENSG00000105137	SYDE1	1193.761135	820.06146	1567.460814	0.934518247	0.075968251	12.3014316	8.90E-35	1.08E-33
ENSG00000101460	MAP1LC3A	910.2701983	624.98679	1195.553609	0.934507474	0.086442401	10.8107533	3.06E-27	3.05E-26

ENSG00000248187	AC078850.1	1464.451907	1005.7915	1923.112303	0.934315147	0.07449279	12.5423568	4.38E-36	5.48E-35
ENSG00000137817	PARP6	579.9950121	398.7927	761.1973236	0.934024179	0.113575918	8.22378715	1.97E-16	1.33E-15
ENSG00000140443	IGF1R	1491.729086	1025.5846	1957.873565	0.933510911	0.090383479	10.3283357	5.25E-25	4.86E-24
ENSG00000168528	SERINC2	1758.457854	1208.2499	2308.665759	0.933217685	0.069523226	13.4231067	4.43E-41	6.23E-40
ENSG00000197746	PSAP	102858.1373	70707.081	135009.1937	0.933152337	0.036899189	25.2892368	4.20E-141	1.93E-139
ENSG00000156671	SAMD8	2357.274519	1621.9867	3092.562385	0.931380838	0.06071394	15.3404776	4.10E-53	7.21E-52
ENSG00000102359	SRPX2	7759.508434	5341.0308	10177.98606	0.930261527	0.04352514	21.3729705	2.38E-101	7.86E-100
ENSG00000150540	HNMT	234.1030488	161.14429	307.0618067	0.929949433	0.149274194	6.2298071	4.67E-10	2.23E-09
ENSG00000100345	MYH9	200600.3961	138210.94	262989.8489	0.928136107	0.039829043	23.3029981	4.13E-120	1.65E-118
ENSG00000163814	CDCP1	1171.39144	806.98646	1535.796418	0.927541884	0.084835265	10.9334471	7.98E-28	8.10E-27
ENSG00000160013	PTGIR	181.2592294	124.82543	237.6930245	0.927344932	0.172360001	5.38027922	7.44E-08	3.01E-07
ENSG00000132793	LPIN3	231.3909528	159.53477	303.2471322	0.927105981	0.15653753	5.92257961	3.17E-09	1.43E-08
ENSG00000154262	ABCA6	1747.062118	1205.3567	2288.767559	0.925981123	0.084729533	10.9286702	8.41E-28	8.52E-27
ENSG00000081665	ZNF506	160.0525734	110.42666	209.6784847	0.9256879	0.189354595	4.88864766	1.02E-06	3.75E-06
ENSG00000167995	BEST1	322.1925673	222.07554	422.3095952	0.925264071	0.138605611	6.6755167	2.46E-11	1.28E-10
ENSG00000113248	PCDHB15	66.20217325	45.7456	86.65874617	0.92480959	0.280887773	3.29245228	0.000993	0.002621
ENSG00000275183	LENG9	142.2655385	98.105269	186.4258082	0.924653076	0.195352601	4.73325194	2.21E-06	7.91E-06
ENSG00000105656	ELL	832.553204	574.59172	1090.514688	0.923131967	0.102378015	9.01689654	1.93E-19	1.48E-18
ENSG00000221968	FADS3	7019.162345	4851.1601	9187.164624	0.921291989	0.046009038	20.0241525	3.39E-89	9.97E-88
ENSG00000166073	GPR176	2250.175148	1555.1581	2945.192173	0.920993249	0.058643355	15.704989	1.40E-55	2.55E-54
ENSG00000134851	TMEM165	4680.542251	3236.3006	6124.783947	0.920231936	0.047301306	19.4546836	2.66E-84	7.40E-83
ENSG00000188868	ZNF563	37.72187214	26.153276	49.29046801	0.920204673	0.382562843	2.40536866	0.016156	0.034714
ENSG00000114841	DNAH1	190.6982813	131.99253	249.4040371	0.919540584	0.164419796	5.59263913	2.24E-08	9.45E-08
ENSG00000185404	SP140L	1070.571627	740.87713	1400.266126	0.918547811	0.078230939	11.7414903	7.81E-32	8.75E-31
ENSG00000228544	CCDC183-AS1	45.35320182	31.315607	59.39079693	0.917967625	0.349386166	2.62737256	0.008605	0.019489
ENSG00000131725	WDR44	1539.34591	1065.8195	2012.872306	0.917889412	0.072498199	12.6608581	9.74E-37	1.24E-35
ENSG00000165915	SLC39A13	4460.524452	3087.087	5833.961912	0.917782327	0.051144664	17.9448306	5.27E-72	1.25E-70
ENSG00000256628	ZBTB11-AS1	136.271862	94.245182	178.2985416	0.917689352	0.204153672	4.4950911	6.95E-06	2.37E-05
ENSG00000119048	UBE2B	2026.669486	1403.2511	2650.087865	0.917524216	0.061785863	14.8500672	6.95E-50	1.16E-48
ENSG00000162511	LAPTM5	4304.245657	2979.8056	5628.685732	0.917245969	0.056416385	16.2585031	1.94E-59	3.77E-58
ENSG00000170113	NIPA1	1404.18702	972.42515	1835.948887	0.916663393	0.069783936	13.1357366	2.05E-39	2.78E-38
ENSG00000225969	ABHD11-AS1	36.03528276	24.967029	47.10353696	0.916594787	0.376067195	2.43731652	0.014797	0.032046
ENSG00000141458	NPC1	4221.893586	2924.3867	5519.400498	0.916284033	0.048717522	18.8081003	6.48E-79	1.68E-77
ENSG00000150787	PTS	487.5067375	337.80152	637.2119589	0.91622648	0.106515065	8.60184879	7.84E-18	5.59E-17
ENSG00000183060	LYSMD4	361.6925838	250.60553	472.7796333	0.914816077	0.121511689	7.52862616	5.13E-14	3.05E-13
ENSG00000242247	ARFGAP3	2192.839383	1520.7281	2864.950699	0.914226498	0.060390417	15.1386022	9.01E-52	1.55E-50
ENSG00000149243	KLHL35	30.12803186	20.890705	39.365359	0.914133871	0.399392271	2.28881212	0.02209	0.046043
ENSG00000141380	SS18	1944.888017	1349.3576	2540.4184	0.912804792	0.061065075	14.9480664	1.60E-50	2.71E-49
ENSG00000105877	DNAH11	245.9692273	170.51122	321.4272344	0.912695954	0.147866569	6.1724294	6.72E-10	3.17E-09
ENSG00000153443	UBALD1	633.9949525	439.85888	828.1310278	0.911885361	0.104993986	8.6851199	3.78E-18	2.73E-17
ENSG00000106346	USP42	667.8936708	463.35248	872.4348664	0.911720625	0.098430724	9.26256137	2.00E-20	1.58E-19
ENSG00000123609	NMI	1132.460072	786.20043	1478.719716	0.911549829	0.071405638	12.7657964	2.55E-37	3.28E-36
ENSG00000196850	PPTC7	1226.530405	851.53218	1601.528625	0.911265207	0.070144192	12.9913137	1.37E-38	1.82E-37
ENSG00000116032	GRIN3B	49.6121839	34.395926	64.82844136	0.911084272	0.331464291	2.74866493	0.005984	0.013921
ENSG00000146205	ANO7	99.13464076	68.756294	129.5129876	0.910744438	0.245682313	3.70700043	0.00021	0.000605
ENSG00000111339	ART4	1512.481264	1050.4612	1974.501346	0.910690592	0.068165824	13.3599293	1.04E-40	1.45E-39
ENSG00000233461	AL445524.1	94.59787695	65.807466	123.388288	0.908789979	0.229056662	3.9675335	7.26E-05	0.000222
ENSG00000137200	CMTR1	2481.951148	1725.7872	3238.115118	0.908039765	0.055529192	16.3524757	4.18E-60	8.19E-59
ENSG00000128965	CHAC1	125.7652471	87.352937	164.177557	0.907175751	0.226388643	4.00716104	6.15E-05	0.000189
ENSG00000148700	ADD3	2638.147512	1835.3224	3440.972603	0.906920952	0.055882484	16.2290737	3.14E-59	6.09E-58
ENSG00000269825	AC022150.4	211.1602166	147.0193	275.301131	0.906361783	0.163084247	5.55762927	2.73E-08	1.15E-07
ENSG00000171105	INSR	1518.491543	1057.0149	1979.968233	0.906247064	0.078385382	11.5614294	6.46E-31	7.08E-30
ENSG00000104936	DMPK	508.7928652	354.14149	663.4442426	0.90624207	0.119803273	7.56441829	3.90E-14	2.34E-13
ENSG00000166889	PATL1	2930.091756	2038.4738	3821.709688	0.906227637	0.054377436	16.6655089	2.33E-62	4.74E-61
ENSG00000120063	GNA13	3121.414566	2172.3349	4070.494233	0.906002193	0.055320806	16.3772413	2.78E-60	5.47E-59
ENSG00000175265	GOLGA8A	105.8139914	73.777546	137.8504369	0.905355038	0.257912353	3.51032057	0.000448	0.001238
ENSG00000038382	TRIO	14045.34127	9785.0597	18305.62284	0.90355925	0.038037144	23.7546555	9.84E-125	4.08E-123
ENSG00000068971	PPP2R5B	1557.125137	1084.9125	2029.337782	0.903011876	0.067093714	13.4589639	2.73E-41	3.86E-40
ENSG00000172057	ORMDL3	1127.340733	785.89611	1468.78536	0.902054276	0.072523872	12.4380324	1.62E-35	2.00E-34
ENSG00000126368	NR1D1	435.5440614	303.46874	567.6193854	0.90201228	0.118645454	7.60258609	2.90E-14	1.76E-13
ENSG00000051523	CYBA	2455.956652	1711.8142	3200.099101	0.901864384	0.066496065	13.562673	6.67E-42	9.60E-41
ENSG00000120910	PPP3CC	777.8162461	542.09854	1013.533953	0.901767242	0.095844921	9.40860748	5.03E-21	4.07E-20
ENSG00000153179	RASSF3	4962.309502	3460.1782	6464.440849	0.90139653	0.049822557	18.092137	3.68E-73	8.88E-72
ENSG00000100583	SAMD15	46.99449027	32.768407	61.22057356	0.89839492	0.355488985	2.52720888	0.011497	0.025419
ENSG00000086061	DNAJA1	12254.81151	8558.7929	15950.83009	0.898347619	0.043393854	20.7021858	3.31E-95	1.04E-93
ENSG00000257704	INAFM1	115.415196	80.634735	150.1956565	0.898252208	0.253741676	3.5400263	0.0004	0.001114
ENSG00000027697	IFNGR1	2450.303813	1712.0831	3188.524517	0.89759612	0.068664183	13.072261	4.74E-39	6.37E-38
ENSG00000156642	NPTN	4609.59043	3220.2878	5998.893091	0.897466318	0.048329162	18.5698712	5.63E-77	1.42E-75
ENSG00000100321	SYNGR1	119.2507608	83.257994	155.2435277	0.896033872	0.226785148	3.95102536	7.78E-05	0.000237
ENSG00000125746	EML2	545.3424497	381.27149	709.4134047	0.896001212	0.102210589	8.76622687	1.85E-18	1.36E-17
ENSG00000114480	GBE1	7100.150283	4963.8715	9236.429054	0.895926343	0.043233268	20.723077	2.15E-95	6.73E-94
ENSG00000141736	ERBB2	1350.513354	944.08001	1756.946697	0.89584253	0.082614203	10.8436867	2.14E-27	2.15E-26
ENSG00000010404	IDS	383.2615126	268.17814	498.3448855	0.894171295	0.117762994	7.59297354	3.13E-14	1.89E-13
ENSG00000166347	CYB5A	1075.745447	752.72874	1398.762151	0.89403691	0.075340179	11.8666368	1.76E-32	2.02E-31
ENSG00000267745	AC060766.7	48.82779489	34.21073	63.44485936	0.892872621	0.321689771	2.77557045	0.00551	0.012915
ENSG00000116815	CD58	259.255333	181.66139	336.8492791	0.891887159	0.151551357	5.88504899	3.98E-09	1.78E-08
ENSG00000059728	MXD1	410.4764223	287.48777	533.4650753	0.890900889	0.113731359	7.83337944	4.75E-15	2.99E-14
ENSG00000140105	WARS	21331.86127	14949.261	27714.46112	0.890616517	0.043960544	20.2594516	2.93E-91	8.81E-90
ENSG00000271643	AC112220.2	186.3357989	130.6726	241.998995	0.89019773	0.164274684	5.41895871	5.99E-08	2.45E-07
ENSG00000083444	PLOD1	33550.12624	23524.48	43575.77206	0.889355182	0.038168303	23.3008833	4.34E-120	1.72E-118
ENSG00000055813	CCDC85A	304.0135618	213.08455	394.9425705	0.889159397	0.133086268	6.68107542	2.37E-11	1.24E-10
ENSG00000165806	CASP7	2967.916916	2081.4935	3854.340328	0.88870666	0.052076887	17.0652801	2.69E-65	5.79E-64
ENSG00000189136	UBE2Q2P1	60.66257893	42.617774	78.70738409	0.888644421	0.328122966	2.70826645	0.006764	0.015584
ENSG00000081087	OSTM1	2835.860864	1989.9176	3681.804078	0.888113582	0.056563967	15.7010485	1.49E-55	2.71E-54
ENSG00000105605	CACNG7	174.4116733	122.31404	226.5093029	0.887629891	0.189326273	4.68836087	2.75E-06	9.78E-06

ENSG00000204852	TCTN1	762.6351204	535.6214	989.648842	0.88690183	0.094862796	9.34931149	8.82E-21	7.10E-20
ENSG00000103260	METRN	592.3124194	415.43926	769.1855812	0.886777555	0.117183164	7.56744845	3.81E-14	2.29E-13
ENSG00000145685	LHFPL2	5000.129398	3510.615	6489.643824	0.886640577	0.046618456	19.0190895	1.19E-80	3.14E-79
ENSG00000119636	BBOF1	129.26718	90.87261	167.6617495	0.886091553	0.206201602	4.29720983	1.73E-05	5.66E-05
ENSG00000163900	TMEM41A	712.4502015	500.43957	924.4608365	0.885599259	0.089347882	9.91181029	3.70E-23	3.23E-22
ENSG00000159921	GNE	678.2092299	476.38597	880.0324877	0.884675835	0.096381479	9.17889874	4.36E-20	3.42E-19
ENSG00000132205	EMILIN2	185.011866	130.16933	239.8543988	0.884588978	0.189264888	4.67381451	2.96E-06	1.05E-05
ENSG00000100889	PCK2	577.9367858	406.24991	749.6236597	0.883403271	0.105110622	8.40450996	4.30E-17	2.98E-16
ENSG00000167987	VPS37C	1585.928639	1114.835	2057.022309	0.883275102	0.074087122	11.9221138	9.08E-33	1.05E-31
ENSG00000196923	PDLIM7	5733.142008	4030.346	7435.937997	0.88315738	0.055207796	15.9969687	1.34E-57	2.52E-56
ENSG00000013392	RWDD2A	499.0756046	351.21134	646.9398738	0.882889781	0.112031138	7.88075351	3.25E-15	2.06E-14
ENSG00000070778	AL162171.1	1535.913451	1080.0196	1991.80732	0.882562227	0.080734942	10.9316017	8.14E-28	8.26E-27
ENSG00000232931	LINC00342	80.23195785	56.585751	103.8781647	0.881883686	0.321591558	2.74224762	0.006102	0.014175
ENSG00000137842	TMEM62	584.8803629	411.40684	758.3538837	0.88132052	0.103537504	8.51208968	1.71E-17	1.20E-16
ENSG00000227825	SLC9A7P1	116.0967896	81.595742	150.5978372	0.880511004	0.243078034	3.62233885	0.000292	0.000829
ENSG00000138600	SPPL2A	2898.109688	2040.673	3755.546385	0.88026315	0.057135926	15.4064738	1.48E-53	2.61E-52
ENSG00000100418	DESI1	3846.643618	2709.2607	4984.026585	0.878895996	0.05785328	15.1918092	4.01E-52	6.95E-51
ENSG00000160218	TRAPPC10	1534.75357	1081.3703	1988.13684	0.878008024	0.067866621	12.9372586	2.77E-38	3.66E-37
ENSG00000171658	NMRAL2P	51.34481983	36.120499	66.56914021	0.877910294	0.37456604	2.34380644	0.019088	0.040413
ENSG00000010318	PHF7	102.0152551	71.981325	132.0491855	0.877856046	0.225921804	3.88566323	0.000102	0.000306
ENSG00000188177	ZC3H6	298.2116611	210.38097	386.0423508	0.877657258	0.145374494	6.03721626	1.57E-09	7.20E-09
ENSG00000085449	WDFY1	3608.718735	2544.7733	4672.664189	0.87714792	0.059183885	14.8207223	1.08E-49	1.79E-48
ENSG00000158092	NCK1	1345.819205	949.43033	1742.208077	0.876705956	0.075544344	11.6051833	3.88E-31	4.26E-30
ENSG00000196083	IL1RAP	491.5703431	346.70537	636.4353198	0.876471358	0.104438843	8.39222926	4.77E-17	3.30E-16
ENSG00000068028	RASSF1	982.3316961	693.29084	1271.372556	0.874874561	0.08459315	10.3421443	4.54E-25	4.23E-24
ENSG00000063601	MTMR1	1125.773558	794.50192	1457.045199	0.874535697	0.080308929	10.8896447	1.29E-27	1.30E-26
ENSG00000123965	PMS2P5	53.25750023	37.693223	68.82177755	0.873181189	0.319211474	2.73543171	0.00623	0.014456
ENSG00000177674	AGTRAP	961.3851636	679.07254	1243.697791	0.872908705	0.083469709	10.4577901	1.35E-25	1.28E-24
ENSG00000082996	RNF13	1595.17232	1127.3649	2062.979768	0.872885373	0.073134085	11.9354111	7.74E-33	8.92E-32
ENSG00000151151	IPMK	651.8492127	460.49654	843.2018823	0.872617663	0.09731019	8.96738216	3.04E-19	2.30E-18
ENSG00000154265	ABCA5	599.6375678	423.99772	775.2774177	0.871947673	0.103516291	8.42328938	3.66E-17	2.55E-16
ENSG00000091039	OSBPL8	3496.132965	2471.545	4520.72096	0.871478002	0.058258136	14.9589064	1.36E-50	2.30E-49
ENSG00000103021	CCDC113	92.87328378	65.610465	120.1361021	0.870814934	0.241929551	3.59945666	0.000319	0.000899
ENSG00000049192	ADAMTS6	1244.117535	879.85314	1608.381926	0.870638123	0.078433976	11.1002677	1.25E-28	1.30E-27
ENSG00000217128	FNIP1	1312.772221	928.87033	1696.674109	0.869479576	0.076223633	11.4069553	3.86E-30	4.16E-29
ENSG00000172331	BPGM	1772.507496	1254.8255	2290.1895	0.868629913	0.068931098	12.6014229	2.07E-36	2.61E-35
ENSG00000241127	YAE1	464.3851585	328.76898	600.0013347	0.868586251	0.13042512	6.65965462	2.74E-11	1.43E-10
ENSG00000206573	THUMPD3-AS1	154.743396	109.74327	199.7435189	0.868503977	0.216882696	4.00448718	6.22E-05	0.000191
ENSG00000256806	C17orf100	36.03701548	25.439816	46.63421502	0.868214359	0.384241899	2.2595515	0.023849	0.049353
ENSG00000011422	PLAUR	2929.124416	2073.1316	3785.117241	0.867961398	0.066530793	13.0460101	6.70E-39	8.95E-38
ENSG00000111859	NEDD9	5294.133926	3749.0158	6839.252077	0.867300661	0.059316885	14.6214803	2.05E-48	3.34E-47
ENSG00000186174	BCL9L	5136.358573	3637.2535	6635.46365	0.86712869	0.047643323	18.2004243	5.12E-74	1.25E-72
ENSG00000109113	RAB34	178.0923926	126.22025	229.9645343	0.867040332	0.174724659	4.96232379	6.97E-07	2.61E-06
ENSG00000108395	TRIM37	3404.631546	2412.549	4396.714044	0.866285939	0.054698233	15.8375489	1.71E-56	3.17E-55
ENSG00000197780	TAF13	1373.954998	973.88002	1774.029976	0.866119368	0.070791865	12.2347302	2.03E-34	2.42E-33
ENSG00000073331	ALPK1	321.1667673	227.6013	414.7322313	0.865123105	0.130027416	6.65338995	2.86E-11	1.48E-10
ENSG00000135929	CYP27A1	1121.391223	795.37355	1447.408893	0.864656972	0.080187841	10.7828938	4.15E-27	4.11E-26
ENSG00000100299	ARSA	2831.077614	2006.9949	3655.160311	0.86438221	0.055349694	15.6167477	5.60E-55	1.01E-53
ENSG00000173559	NABP1	999.9495923	709.37302	1290.526161	0.864287498	0.08734225	9.89541138	4.36E-23	3.80E-22
ENSG00000118263	KLF7	2275.869386	1613.9428	2937.796021	0.864176268	0.058315608	14.8189534	1.10E-49	1.84E-48
ENSG00000241316	SUCLG2-AS1	40.09349175	28.466755	51.72022882	0.86411865	0.380087854	2.27347083	0.022998	0.047728
ENSG00000131773	KHDRBS3	362.4897715	257.28337	467.6961701	0.863704735	0.127704874	6.76328718	1.35E-11	7.14E-11
ENSG00000223485	LINC01615	43.40151662	30.837419	55.96561399	0.863374828	0.352058259	2.45236351	0.014192	0.030829
ENSG00000182247	UBE2E2	992.4110309	704.11384	1280.708221	0.8631481	0.08150786	10.5897529	3.32E-26	3.21E-25
ENSG00000169967	MAP3K2	933.4181161	662.65422	1204.182009	0.862975098	0.088490501	9.75217775	1.81E-22	1.54E-21
ENSG00000151726	ACSL1	2169.123147	1539.8326	2798.413689	0.861508727	0.063569485	13.5522371	7.69E-42	1.10E-40
ENSG00000182197	EXT1	6772.774971	4810.7551	8734.794827	0.860439739	0.046490958	18.5076792	1.79E-76	4.49E-75
ENSG00000150687	PRSS23	47539.90317	33772.521	61307.28577	0.860174314	0.038909676	22.1069516	2.71E-108	9.59E-107
ENSG00000000971	CFH	6440.748947	4577.2314	8304.266454	0.859809921	0.058598838	14.6728151	9.63E-49	1.58E-47
ENSG00000155158	TTC39B	219.4845405	156.14931	282.8197756	0.859685554	0.164235357	5.23447307	1.65E-07	6.51E-07
ENSG00000167984	NLRC3	87.76045006	62.491227	113.0296736	0.858879807	0.249157274	3.44713921	0.000567	0.001546
ENSG00000198053	SIRPA	7044.294179	5009.1183	9079.470019	0.858098449	0.045901291	18.6944297	5.50E-78	1.40E-76
ENSG00000171621	SPSB1	2388.560951	1699.1656	3077.956286	0.85767084	0.068258644	12.5650144	3.29E-36	4.12E-35
ENSG00000158201	ABHD3	124.5960718	88.867648	160.3244957	0.856561493	0.250080872	3.42513798	0.000614	0.00167
ENSG00000225697	SLC26A6	400.5879224	285.28168	515.8941671	0.854643257	0.128729562	6.63905978	3.16E-11	1.63E-10
ENSG00000103222	ABCC1	14060.96012	10016.798	18105.12243	0.853897189	0.039733637	21.4905366	1.91E-102	6.40E-101
ENSG00000164951	PDP1	3465.964807	2470.5079	4461.421708	0.852964361	0.052234608	16.3294872	6.09E-60	1.19E-58
ENSG00000197930	ERO1A	5025.505483	3582.0255	6468.985437	0.852863283	0.043481398	19.61444	1.16E-85	3.28E-84
ENSG00000117151	CTBS	695.5392698	495.7862	895.2923397	0.85233936	0.096295377	8.85130093	8.65E-19	6.44E-18
ENSG00000163945	UVSSA	359.9605428	256.85039	463.070691	0.851702188	0.160931273	5.29233488	1.21E-07	4.81E-07
ENSG00000087074	PPP1R15A	3615.088302	2577.6304	4652.546197	0.851514325	0.055238687	15.4151804	1.29E-53	2.29E-52
ENSG00000100403	ZC3H7B	13797.39053	9842.7081	17752.07299	0.850810509	0.037742566	22.5424658	1.59E-112	5.86E-111
ENSG00000077585	GPR137B	489.0813846	349.08173	629.0810438	0.849854673	0.105696572	8.04051311	8.95E-16	5.84E-15
ENSG00000261971	MMP25-AS1	134.6565902	96.280102	173.0330781	0.848826311	0.235607909	3.6027072	0.000315	0.000889
ENSG00000230715	AC018638.2	65.67570373	46.893576	84.45783187	0.848375692	0.274217166	3.09380957	0.001976	0.004978
ENSG00000116990	MYCL	96.22372992	68.758448	123.6890117	0.845725328	0.234031638	3.61372222	0.000302	0.000855
ENSG00000269940	AL049840.3	458.7980374	328.3139	589.2821794	0.844850158	0.111140629	7.60163198	2.92E-14	1.77E-13
ENSG00000086619	ERO1B	224.9033322	160.8058	289.0008683	0.844669179	0.157218496	5.37258146	7.76E-08	3.14E-07
ENSG00000164930	FZD6	4984.127496	3565.6948	6402.560193	0.844540928	0.044993436	18.7703141	1.32E-78	3.39E-77
ENSG00000136425	CIB2	102.322891	73.229096	131.4166858	0.844243037	0.222765673	3.78982553	0.000151	0.000443
ENSG00000104381	GDAP1	56.02004613	40.1857	71.85439246	0.842810834	0.334018985	2.5232423	0.011628	0.02569
ENSG00000273136	NBPF26	182.1963422	130.54831	233.8443714	0.842522523	0.181827254	4.63364267	3.59E-06	1.26E-05
ENSG00000219200	RNASEK	84.38309195	60.399092	108.3670924	0.842472868	0.246022626	3.4243715	0.000616	0.001674
ENSG00000155256	ZFYVE27	690.8705121	494.99239	886.7486369	0.84170941	0.089090564	9.44779524	3.46E-21	2.83E-20
ENSG00000163659	TIPARP	2247.27833	1610.1951	2884.361512	0.84120699	0.057891213	14.5308234	7.73E-48	1.25E-46

ENSG00000140464	PML	6280.184394	4500.0137	8060.355081	0.840608008	0.045207743	18.5943371	3.57E-77	9.01E-76
ENSG00000161011	SQSTM1	27965.20739	20051.597	35878.81746	0.839353259	0.037131078	22.6051411	3.86E-113	1.44E-111
ENSG00000162413	KLHL21	1982.546376	1421.0692	2544.023596	0.839246378	0.069022085	12.1590992	5.13E-34	6.06E-33
ENSG00000142089	IFITM3	16423.12575	11777.91	21068.34183	0.838854608	0.058188384	14.4161867	4.09E-47	6.50E-46
ENSG00000149557	FEZ1	2007.18776	1440.2676	2574.107899	0.83842713	0.070490499	11.8941864	1.27E-32	1.46E-31
ENSG00000118197	DDX59	442.0142647	317.06989	566.9586374	0.837608591	0.110817881	7.55842455	4.08E-14	2.45E-13
ENSG00000279198	AC008894.3	42.91462494	30.832817	54.99643325	0.837409018	0.361587683	2.31592241	0.020563	0.043192
ENSG00000138380	CARF	97.48200263	70.024222	124.9397837	0.836990348	0.2414887	3.46596072	0.000528	0.001448
ENSG00000134802	SLC43A3	1253.77354	899.74907	1607.798008	0.836875893	0.07140309	11.7204437	1.00E-31	1.12E-30
ENSG00000158186	MRAS	781.7377561	561.26668	1002.208831	0.836837235	0.088167357	9.49146334	2.28E-21	1.87E-20
ENSG00000158985	CDC42SE2	1485.152933	1065.9684	1904.337488	0.836277864	0.069357403	12.057514	1.77E-33	2.07E-32
ENSG00000229320	KRT8P12	63.77058855	45.889543	81.65163399	0.835718068	0.323870403	2.5804089	0.009868	0.022101
ENSG00000034713	GABARAPL2	1839.637422	1321.7459	2357.528939	0.835709826	0.071116702	11.7512455	6.96E-32	7.81E-31
ENSG00000178773	CPNE7	502.8009851	361.13075	644.47122	0.834995336	0.117710743	7.09362047	1.31E-12	7.31E-12
ENSG00000204516	MICB	1379.968265	991.50258	1768.433955	0.834423998	0.073278048	11.3870937	4.85E-30	5.21E-29
ENSG00000170145	SIK2	2830.665363	2034.2309	3627.099872	0.834406417	0.062532645	13.343533	1.29E-40	1.79E-39
ENSG00000177426	TGIF1	1382.805848	993.69826	1771.913441	0.833487995	0.071618853	11.6378295	2.65E-31	2.92E-30
ENSG00000158966	CACHD1	675.650313	485.64169	865.6589386	0.833041062	0.095975535	8.67972304	3.97E-18	2.86E-17
ENSG00000127329	PTPRB	19860.73122	14282.494	25438.96869	0.832900561	0.058048164	14.3484393	1.09E-46	1.72E-45
ENSG00000228327	AL669831.1	38.80725719	27.948435	49.66607958	0.832889224	0.361787346	2.30215134	0.021327	0.044625
ENSG00000124151	NCOA3	2798.378095	2012.8988	3583.857415	0.832125036	0.05956434	13.9701881	2.37E-44	3.57E-43
ENSG00000102934	PLLP	683.4932228	491.88885	875.0976002	0.831661896	0.091011833	9.13795346	6.36E-20	4.97E-19
ENSG00000196187	TMEM63A	1233.52787	888.5761	1578.479641	0.828912144	0.070324995	11.7868782	4.56E-32	5.16E-31
ENSG00000174943	KCTD13	303.4321553	218.48543	388.3788792	0.828630003	0.147764066	5.60779104	2.05E-08	8.69E-08
ENSG00000272405	AL365181.3	62.62256947	45.146398	80.09874093	0.82858962	0.280510793	2.95386003	0.003138	0.007652
ENSG00000148120	C9orf3	440.71518	317.7472	563.6831552	0.827325199	0.111291488	7.4338587	1.05E-13	6.20E-13
ENSG00000111450	STX2	1302.813306	939.59245	1666.034162	0.826979318	0.071056749	11.6382937	2.63E-31	2.91E-30
ENSG00000002549	LAP3	6900.943912	4976.3522	8825.5356	0.826885779	0.049066942	16.8521972	1.01E-63	2.11E-62
ENSG00000172748	ZNF596	61.58984093	44.450436	78.72924548	0.826864435	0.343438713	2.40760405	0.016058	0.034538
ENSG00000120370	GORAB	260.5486044	187.77947	333.3177342	0.826645164	0.146557172	5.64042793	1.70E-08	7.24E-08
ENSG00000267102	AC060766.1	122.1677017	88.102222	156.2331818	0.826279281	0.201043166	4.10995955	3.96E-05	0.000125
ENSG00000082641	NFE2L1	31861.90835	22986.805	40737.01142	0.825528603	0.03537239	23.3382192	1.82E-120	7.26E-119
ENSG00000185869	ZNF829	87.6708669	63.282676	112.0590579	0.825048397	0.239896886	3.43917926	0.000583	0.00159
ENSG00000197496	SLC2A10	473.3783813	341.86309	604.8936714	0.824925566	0.128849561	6.40223807	1.53E-10	7.56E-10
ENSG00000088543	C3orf18	171.7610564	123.82869	219.6934218	0.82464002	0.187515048	4.39772715	1.09E-05	3.65E-05
ENSG00000163191	S100A11	7472.530765	5392.5673	9552.494237	0.824569531	0.053769297	15.3353229	4.44E-53	7.80E-52
ENSG00000106829	TLE4	664.3120822	479.80709	848.8170728	0.822939524	0.096571339	8.52157101	1.57E-17	1.11E-16
ENSG00000116704	SLC35D1	401.1928819	290.02575	512.3600142	0.82221087	0.116817888	7.03839868	1.94E-12	1.08E-11
ENSG00000065665	SEC61A2	169.8276952	122.80111	216.8542774	0.821397189	0.170299885	4.82323983	1.41E-06	5.16E-06
ENSG00000198680	TUSC1	263.7151868	190.57385	336.8565196	0.82059937	0.144901722	5.66314436	1.49E-08	6.36E-08
ENSG00000178038	ALS2CL	350.4575254	253.68901	447.2260382	0.820156168	0.134417547	6.10155586	1.05E-09	4.88E-09
ENSG00000160233	LRRC3	575.9414704	416.31466	735.5682781	0.820140108	0.106623754	7.69190803	1.45E-14	8.92E-14
ENSG00000132294	EFR3A	3368.005006	2435.9942	4300.015844	0.819738549	0.057484795	14.2600933	3.88E-46	6.06E-45
ENSG00000131584	ACAP3	698.0577895	505.40156	890.714016	0.818331198	0.104796803	7.80874199	5.78E-15	3.62E-14
ENSG00000135241	PNPLA8	1747.635615	1265.0342	2230.236993	0.817653506	0.064230146	12.7300584	4.03E-37	5.17E-36
ENSG00000205364	MT1M	55.24045523	40.027769	70.45314123	0.817153882	0.301358561	2.71156684	0.006697	0.015439
ENSG00000100376	FAM118A	477.0343286	345.80303	608.2656306	0.816332395	0.132896636	6.14261144	8.12E-10	3.80E-09
ENSG00000119537	KDSR	3416.252071	2475.0891	4357.415057	0.816111834	0.048449164	16.844704	1.15E-63	2.38E-62
ENSG00000142606	MMEL1	43.72206014	31.716394	55.72772672	0.815847452	0.332242557	2.45557782	0.014066	0.030594
ENSG00000078401	EDN1	21502.93425	15586.259	27419.60955	0.815004877	0.055608628	14.6560867	1.23E-48	2.01E-47
ENSG00000065911	MTHFD2	2103.270951	1524.4765	2682.065364	0.814818182	0.060958373	13.3667968	9.45E-41	1.32E-39
ENSG00000162695	SLC30A7	2218.369749	1608.4708	2828.268744	0.814501293	0.062079169	13.1203641	2.52E-39	3.40E-38
ENSG00000142552	RCN3	6837.257635	4956.8348	8717.680468	0.814265145	0.05031124	16.1845574	6.48E-59	1.25E-57
ENSG00000154553	PDLIM3	613.5596043	445.14001	781.9791986	0.814124163	0.099359296	8.19373921	2.53E-16	1.70E-15
ENSG00000187720	THSD4	7790.096508	5650.3471	9929.845877	0.813442626	0.047075385	17.2795745	6.70E-67	1.48E-65
ENSG00000105321	CCDC9	779.6214023	565.78764	993.4551604	0.813342923	0.09324986	8.72218921	2.73E-18	1.99E-17
ENSG00000067191	CACNB1	139.4473976	101.12282	177.7719728	0.81296368	0.191190956	4.25210321	2.12E-05	6.87E-05
ENSG00000186594	MIR22HG	1231.455273	893.93986	1568.970688	0.812316178	0.089751857	9.05068938	1.42E-19	1.09E-18
ENSG00000119682	AREL1	2792.075061	2026.1073	3558.042778	0.811922887	0.055791821	14.5527225	5.61E-48	9.08E-47
ENSG00000164327	RICTOR	1565.703297	1137.018	1994.38861	0.81161638	0.088453767	9.17559991	4.49E-20	3.52E-19
ENSG00000198668	CALM1	19287.82599	14003.066	24572.58606	0.81138609	0.037443467	21.6696305	3.97E-104	1.36E-102
ENSG00000107864	CPEB3	162.9849459	118.4873	207.482588	0.810581744	0.183711519	4.41225324	1.02E-05	3.42E-05
ENSG00000006451	RALA	20949.84763	15218.068	26681.62724	0.810183244	0.053009086	15.283856	9.80E-53	1.72E-51
ENSG00000138495	COX17	360.7745956	262.13117	459.4180214	0.810049271	0.128176827	6.31977941	2.62E-10	1.27E-09
ENSG00000135452	TSPAN31	865.7508747	628.9424	1102.559352	0.810032283	0.082560468	9.81138186	1.01E-22	8.66E-22
ENSG00000030582	GRN	35944.13315	26121.898	45766.36784	0.809033424	0.03915646	20.6615569	7.68E-95	2.39E-93
ENSG00000142303	ADAMTS10	62.46732484	45.510743	79.42390639	0.808623713	0.35482997	2.27890478	0.022673	0.047121
ENSG00000172936	MYD88	2362.964314	1717.992	3007.936669	0.807602256	0.058528408	13.798466	2.60E-43	3.86E-42
ENSG00000141452	RMC1	703.5923029	511.75743	895.4271754	0.807511156	0.090486236	8.9241325	4.49E-19	3.39E-18
ENSG00000213066	FGFR1OP	170.5075614	124.09607	216.9190549	0.807419123	0.206972022	3.90110275	9.58E-05	0.000288
ENSG00000205423	CNEP1R1	581.5442525	423.2595	739.8290063	0.806613957	0.105524327	7.64386737	2.11E-14	1.29E-13
ENSG00000125826	RBCK1	3203.632553	2331.093	4076.17208	0.805820207	0.054216978	14.8628758	5.74E-50	9.63E-49
ENSG00000189223	PAX8-AS1	418.5039762	304.63283	532.3751267	0.805318811	0.117908212	6.83004855	8.49E-12	4.54E-11
ENSG00000067082	KLF6	11748.12241	8552.6963	14943.5521	0.805195733	0.041504115	19.4003832	7.66E-84	2.12E-82
ENSG00000125733	TRIP10	3679.079042	2678.3234	4679.834732	0.80485693	0.048277912	16.6713285	2.12E-62	4.31E-61
ENSG00000143061	IGSF3	38.94130909	28.425531	49.45708761	0.801982276	0.351753216	2.2799572	0.02261	0.047007
ENSG00000102755	FLT1	18989.60502	13844.399	24134.81124	0.801878704	0.039754116	20.1709607	1.76E-90	5.25E-89
ENSG00000111670	GNPTAB	3954.204337	2883.0828	5025.325916	0.801528252	0.048269939	16.6051226	6.40E-62	1.29E-60
ENSG00000111897	SERINC1	8764.883112	6391.2714	11138.49485	0.80151643	0.040071107	20.0023531	5.25E-89	1.54E-87
ENSG00000147905	ZCCHC7	801.2928474	584.4127	1018.172994	0.801206342	0.083129521	9.63804839	5.52E-22	4.62E-21
ENSG00000103005	USB1	3241.926198	2364.712	4119.140431	0.80025924	0.05315328	15.0556889	3.17E-51	5.40E-50
ENSG00000185189	NRBP2	1092.251565	797.28866	1387.214466	0.799626945	0.09227635	8.66556752	4.49E-18	3.23E-17
ENSG00000100139	MICALL1	4366.436792	3187.0678	5545.805761	0.798974032	0.049548231	16.1251778	1.70E-58	3.25E-57
ENSG00000277203	F8A1	109.8453953	80.059402	139.6313886	0.79891729	0.237042283	3.37035773	0.000751	0.002015
ENSG00000144320	LNPK	502.0133305	366.45113	637.5755283	0.798797642	0.103607548	7.70984026	1.26E-14	7.79E-14

ENSG00000156463	SH3RF2	69.15305023	50.536182	87.76991851	0.798082052	0.267852346	2.97955969	0.002887	0.007091
ENSG00000136754	ABI1	2890.704868	2111.7139	3669.695808	0.797160594	0.050870274	15.6704601	2.41E-55	4.37E-54
ENSG00000235162	C12orf75	898.0960836	655.85421	1140.337953	0.796461057	0.104462236	7.62439217	2.45E-14	1.49E-13
ENSG00000109436	TBC1D9	5731.688624	4188.8159	7274.561339	0.796170048	0.045254096	17.5933257	2.77E-69	6.31E-68
ENSG00000003402	CFLAR	11043.17761	8073.169	14013.1862	0.795682403	0.038901244	20.4539063	5.55E-93	1.71E-91
ENSG00000280433	FP565260.6	93.23773513	68.173234	118.3022366	0.79551858	0.230465491	3.45179045	0.000557	0.001521
ENSG00000241839	PLEKHO2	1655.133448	1209.6634	2100.60351	0.795383744	0.071881748	11.0651697	1.85E-28	1.91E-27
ENSG00000136274	NACAD	125.044347	91.52673	158.5619636	0.795242792	0.20992625	3.78820081	0.000152	0.000445
ENSG00000188042	ARL4C	828.6966311	605.75639	1051.636873	0.795017736	0.085967474	9.24788996	2.29E-20	1.81E-19
ENSG00000159346	ADIPOR1	3824.010573	2796.7028	4851.318377	0.794226388	0.050785419	15.6388664	3.96E-55	7.16E-54
ENSG00000112655	PTK7	844.0473342	617.62687	1070.467801	0.793802994	0.089539261	8.86541821	7.62E-19	5.69E-18
ENSG00000123106	CCDC91	452.6280224	331.4828	573.7732422	0.793245784	0.122204414	6.49113858	8.52E-11	4.28E-10
ENSG00000140323	DISP2	391.6034532	286.40427	496.8026371	0.792569495	0.124361477	6.37311097	1.85E-10	9.08E-10
ENSG00000147251	DOCK11	71.93892022	52.746438	91.13140201	0.792146174	0.29033756	2.72836271	0.006365	0.014745
ENSG00000278311	GGNBP2	2022.145515	1480.858	2563.433068	0.792123087	0.059398616	13.3357163	1.43E-40	1.99E-39
ENSG00000033627	ATP6V0A1	1512.654332	1107.7738	1917.534824	0.79195291	0.064466079	12.2848003	1.09E-34	1.31E-33
ENSG00000100592	DAAM1	1509.809811	1105.7719	1913.847763	0.791727576	0.067465637	11.7352717	8.41E-32	9.41E-31
ENSG00000167615	LENG8	1609.297286	1178.955	2039.639545	0.791002464	0.190470496	4.15288708	3.28E-05	0.000105
ENSG00000109906	ZBTB16	282.9220755	207.22443	358.6197208	0.790350207	0.136997259	5.76909505	7.97E-09	3.47E-08
ENSG00000128590	DNAJB9	987.6478442	724.24797	1251.047716	0.788126472	0.078570244	10.0308518	1.12E-23	9.92E-23
ENSG00000090674	MCOLN1	1124.57976	824.87974	1424.279779	0.787216886	0.074284546	10.5973171	3.07E-26	2.97E-25
ENSG00000167657	DAPK3	5848.313219	4291.4108	7405.215679	0.786970949	0.046443763	16.9445993	2.11E-64	4.47E-63
ENSG00000092621	PHGDH	101.352662	74.496435	128.2088885	0.786201598	0.256297167	3.06753917	0.002158	0.005405
ENSG00000112137	PHACTR1	543.8230902	399.27106	688.3751196	0.785863211	0.098770315	7.95647161	1.77E-15	1.14E-14
ENSG00000144026	ZNF514	200.7155688	147.54544	253.8856961	0.78545967	0.173684509	4.52233578	6.12E-06	2.09E-05
ENSG00000006327	TNFRSF12A	3026.618715	2222.376	3830.861421	0.785302581	0.058435329	13.4388322	3.58E-41	5.06E-40
ENSG00000132622	HSPA12B	104.7050864	77.003223	132.4069494	0.784079256	0.226928301	3.45518497	0.00055	0.001503
ENSG00000146278	PNRC1	2207.916779	1622.2336	2793.599983	0.783572824	0.060332298	12.9876177	1.44E-38	1.91E-37
ENSG00000143321	HDGF	11048.85013	8119.7724	13977.92783	0.783451862	0.043146802	18.1578199	1.11E-73	2.72E-72
ENSG00000152229	PSTPIP2	394.0475617	289.58931	498.5058122	0.78205756	0.141149223	5.54064376	3.01E-08	1.26E-07
ENSG00000122861	PLAU	4658.283019	3425.1482	5891.417835	0.781981835	0.052725718	14.8311272	9.22E-50	1.54E-48
ENSG00000257702	LBX2-AS1	95.75399369	70.481606	121.0263812	0.781263659	0.234672808	3.32916143	0.000871	0.002316
ENSG00000259642	ST20-AS1	124.8048642	91.948585	157.6611431	0.781116733	0.215945009	3.61720207	0.000298	0.000844
ENSG00000197375	SLC22A5	185.162748	136.24894	234.0765564	0.780627905	0.169297388	4.61098611	4.01E-06	1.40E-05
ENSG00000176915	ANKLE2	3988.920156	2935.5823	5042.258021	0.780506977	0.050724455	15.3871929	2.00E-53	3.52E-52
ENSG00000197927	C2orf27A	397.0676911	292.45797	501.6774079	0.780336882	0.12990136	6.00714945	1.89E-09	8.63E-09
ENSG00000113163	COL4A3BP	1960.593636	1442.8183	2478.368997	0.78028049	0.060128964	12.9767826	1.66E-38	2.20E-37
ENSG00000168310	IRF2	1007.874423	741.80367	1273.945179	0.779964676	0.074959049	10.4052104	2.35E-25	2.21E-24
ENSG00000121964	GTDC1	780.1720034	574.26833	986.0756751	0.779679464	0.087642048	8.89618029	5.78E-19	4.33E-18
ENSG00000149115	TNKS1BP1	10880.60189	8009.8016	13751.40221	0.779656251	0.041134104	18.9540107	4.09E-80	1.08E-78
ENSG00000173281	PPP1R3B	3679.571454	2710.2216	4648.921305	0.778870822	0.061548776	12.6545298	1.06E-36	1.34E-35
ENSG00000259865	AL390728.6	54.63802741	40.253098	69.02295712	0.77836986	0.306317378	2.54105681	0.011052	0.024532
ENSG00000169504	CLIC4	32262.60126	23768.963	40756.23904	0.777966205	0.037839839	20.5594482	6.33E-94	1.96E-92
ENSG00000154767	XPC	3344.337538	2464.1618	4224.513277	0.777424443	0.052601218	14.7795903	1.98E-49	3.28E-48
ENSG00000113269	RNF130	1900.373495	1401.0455	2399.701537	0.776601618	0.059246421	13.1079921	2.96E-39	4.00E-38
ENSG00000243679	AC018638.5	108.3927485	79.995401	136.7900964	0.776464926	0.241421822	3.21621683	0.001299	0.00337
ENSG00000172943	PHF8	1248.55287	920.35774	1576.748003	0.775926953	0.075821264	10.2336325	1.40E-24	1.28E-23
ENSG00000100207	TCF20	1702.431487	1255.3802	2149.482784	0.775871882	0.066686391	11.634636	2.75E-31	3.03E-30
ENSG00000082458	DLG3	130.9086996	96.580468	165.2369311	0.775682901	0.203051062	3.82013714	0.000133	0.000394
ENSG00000154608	CEP170P1	110.916096	81.743964	140.088228	0.775469644	0.223362868	3.47179302	0.000517	0.001419
ENSG00000163660	CCNL1	973.6535091	718.55007	1228.756952	0.775017467	0.09183486	8.43925139	3.19E-17	2.22E-16
ENSG00000008294	SPAG9	5056.32798	3731.684	6380.97193	0.773986057	0.045596579	16.9746521	1.27E-64	2.69E-63
ENSG00000150403	TMCO3	5143.862296	3796.5591	6491.165451	0.773876061	0.044559099	17.367408	1.46E-67	3.25E-66
ENSG00000125122	LRRC29	63.51171185	46.877434	80.14599007	0.773001997	0.288639146	2.67809134	0.007404	0.016971
ENSG00000131943	C19orf12	937.3774326	692.53828	1182.216588	0.771963997	0.081720991	9.44633666	3.51E-21	2.87E-20
ENSG00000166188	ZNF319	617.035907	455.71461	778.3572066	0.771940717	0.099397483	7.76619981	8.09E-15	5.04E-14
ENSG00000068912	ERLEC1	2732.292313	2019.8583	3444.726324	0.770670251	0.056551358	13.6277938	2.74E-42	3.97E-41
ENSG00000076344	RGS11	153.0062972	113.19534	192.8172527	0.770306119	0.193494754	3.98101811	6.86E-05	0.00021
ENSG00000105829	BET1	948.6658863	701.65513	1195.676646	0.7701501	0.086486005	8.90491015	5.34E-19	4.01E-18
ENSG00000163517	HDAC11	458.251342	338.78032	577.7223682	0.76967898	0.106101245	7.25419367	4.04E-13	2.31E-12
ENSG00000214274	ANG	81.01972435	59.980453	102.0589952	0.768799472	0.259388455	2.96389241	0.003038	0.007435
ENSG00000155975	VPS37A	1358.493982	1004.8327	1712.155238	0.768639077	0.069222092	11.1039562	1.20E-28	1.25E-27
ENSG00000224531	SMIM13	1106.850183	818.92378	1394.776589	0.768525362	0.072806288	10.5557553	4.78E-26	4.59E-25
ENSG00000169155	ZBTB43	485.8645009	359.51728	612.2117177	0.768270997	0.116434295	6.59832224	4.16E-11	2.14E-10
ENSG00000139112	GABARAPL1	3536.307551	2616.5133	4456.101756	0.768240648	0.053192265	14.4427101	2.79E-47	4.46E-46
ENSG00000119801	YPEL5	3699.449709	2737.6171	4661.282327	0.768233185	0.051480188	14.9228902	2.34E-50	3.94E-49
ENSG00000170919	TPT1-AS1	127.3092501	94.209803	160.4086967	0.766601972	0.205781424	3.72532154	0.000195	0.000564
ENSG00000147050	KDM6A	1056.439484	782.70688	1330.17209	0.765765912	0.085176001	8.99039524	2.46E-19	1.88E-18
ENSG00000101850	GPR143	110.324458	81.786251	138.8626652	0.764964323	0.227262244	3.36599828	0.000763	0.002043
ENSG00000118640	VAMP8	153.636019	113.88083	193.3912115	0.76476605	0.192595338	3.97084404	7.16E-05	0.000219
ENSG00000170876	TMEM43	5221.55086	3870.3215	6572.780227	0.764210203	0.045273534	16.8798442	6.33E-64	1.33E-62
ENSG00000148110	MFSD14B	3766.884561	2792.8404	4740.928769	0.763465422	0.049353203	15.46942	5.58E-54	9.97E-53
ENSG00000169660	HEXD	296.1763246	219.56111	372.7915372	0.763047228	0.139846266	5.45632896	4.86E-08	2.00E-07
ENSG00000181458	TMEM45A	323.9497409	240.27751	407.6219759	0.762790435	0.134747158	5.66090183	1.51E-08	6.44E-08
ENSG00000279672	AP006621.5	81.90240087	60.69884	103.1059619	0.762688452	0.24756464	3.08076489	0.002065	0.00518
ENSG00000143570	SLC39A1	52.72421551	39.060682	66.38774944	0.762463726	0.313145434	2.434855	0.014898	0.03225
ENSG00000113845	TIMMDC1	1966.444844	1458.7175	2474.172164	0.762344035	0.065559392	11.6282962	2.96E-31	3.26E-30
ENSG00000109756	RAPGEF2	4865.662643	3609.9693	6121.356006	0.762045831	0.047664292	15.9877721	1.55E-57	2.92E-56
ENSG00000204389	HSPA1A	758.7947345	563.00323	954.5862559	0.761653677	0.086620526	8.79299298	1.46E-18	1.07E-17
ENSG00000143669	LYST	1274.485659	945.89762	1603.073696	0.761514378	0.076122705	10.0037745	1.47E-23	1.30E-22
ENSG00000144445	KANSL1L	333.2828772	247.41521	419.1505478	0.76106547	0.125013808	6.08785125	1.14E-09	5.31E-09
ENSG00000158669	GPAT4	4777.576383	3546.8608	6008.291979	0.760410108	0.044995308	16.8997643	4.52E-64	9.51E-63
ENSG00000112308	C6orf62	6119.205689	4543.7679	7694.643502	0.760298585	0.052243104	14.5530898	5.58E-48	9.04E-47
ENSG00000113108	APBB3	59.85687046	44.52887	75.18487052	0.760257382	0.305265695	2.49047762	0.012757	0.028023
ENSG00000123992	DNPEP	1593.182567	1183.4287	2002.936411	0.75843405	0.073930664	10.258721	1.08E-24	9.92E-24

ENSG00000205084	TMEM231	388.5577282	288.58269	488.5327695	0.758172049	0.117466067	6.45439207	1.09E-10	5.41E-10
ENSG00000105402	NAPA	2705.127318	2010.4393	3399.81529	0.757543536	0.057563143	13.1602185	1.49E-39	2.02E-38
ENSG00000197016	ZNF470	346.8020877	257.91471	435.689467	0.756991035	0.126811195	5.96943383	2.38E-09	1.08E-08
ENSG00000156030	ELMSAN1	3640.084848	2707.4728	4572.696904	0.756373962	0.053183344	14.2220085	6.69E-46	1.04E-44
ENSG00000077238	IL4R	5247.622609	3903.9094	6591.3358	0.755814816	0.047676603	15.8529503	1.34E-56	2.49E-55
ENSG00000130522	JUND	2052.409759	1526.3646	2578.454932	0.755621893	0.086773452	8.70798475	3.09E-18	2.24E-17
ENSG00000124357	NAGK	3476.828008	2586.3721	4367.283962	0.755489227	0.054669299	13.8192595	1.95E-43	2.90E-42
ENSG00000143702	CEP170	9452.526387	7032.6995	11872.35324	0.755444714	0.041624435	18.1490682	1.31E-73	3.18E-72
ENSG00000129566	TEP1	1995.550343	1484.9832	2506.117448	0.754747976	0.059039743	12.7837272	2.02E-37	2.61E-36
ENSG00000132031	MATN3	375.9916145	279.81623	472.1669975	0.753948868	0.126927755	5.93998428	2.85E-09	1.29E-08
ENSG00000164031	DNAJB14	2300.582573	1713.6395	2887.525643	0.752989403	0.060749256	12.3950391	2.78E-35	3.41E-34
ENSG00000168803	ADAL	102.1545052	76.047789	128.261221	0.752771064	0.251398464	2.99433438	0.00275	0.006777
ENSG00000125124	BBS2	1951.279736	1453.7722	2448.787299	0.752176447	0.061818007	12.1675945	4.63E-34	5.46E-33
ENSG00000164284	GRPEL2	1259.10532	938.60079	1579.609852	0.752126002	0.090195119	8.33887698	7.50E-17	5.15E-16
ENSG00000118508	RAB32	3209.211643	2390.9518	4027.471478	0.7519158	0.054991146	13.6733975	1.46E-42	2.13E-41
ENSG00000274602	PI4KAP1	79.91172871	59.570726	100.2527309	0.751613155	0.288214788	2.607823	0.009112	0.020554
ENSG00000176700	SCAND2P	119.3260733	88.982553	149.6695939	0.751273565	0.210651843	3.56642295	0.000362	0.001012
ENSG00000101745	ANKRD12	2314.506878	1725.8779	2903.135888	0.750682976	0.061908635	12.1256587	7.72E-34	9.08E-33
ENSG00000112893	MAN2A1	7832.81407	5840.9875	9824.640651	0.750381312	0.043296612	17.3311786	2.74E-67	6.08E-66
ENSG00000167617	CDC42EP5	1252.079142	934.52039	1569.637898	0.748527002	0.083275439	8.98856864	2.50E-19	1.91E-18
ENSG00000173040	EVC2	599.412611	447.79505	751.0301727	0.746257265	0.097912778	7.62165351	2.50E-14	1.52E-13
ENSG00000169692	AGPAT2	1526.387725	1140.6232	1912.152288	0.745038239	0.072975152	10.2094784	1.80E-24	1.63E-23
ENSG00000137177	KIF13A	6507.97041	4863.7326	8152.208267	0.744885792	0.043095141	17.2846818	6.14E-67	1.35E-65
ENSG00000129473	BCL2L2	1162.84098	869.74348	1455.938481	0.744166089	0.075694411	9.83118935	8.26E-23	7.13E-22
ENSG00000122417	ODF2L	1565.895714	1171.0143	1960.777131	0.744125146	0.067585006	11.0102106	3.41E-28	3.49E-27
ENSG00000138685	FGF2	1156.579118	864.85366	1448.304574	0.744048064	0.08134548	9.14676587	5.87E-20	4.59E-19
ENSG00000205189	ZBTB10	763.6550972	571.0562	956.2539901	0.743931256	0.090030773	8.26307753	1.42E-16	9.64E-16
ENSG00000083290	ULK2	911.8209292	681.96117	1141.68069	0.743365295	0.077752643	9.56064346	1.17E-21	9.67E-21
ENSG00000107020	PLGRKT	441.5126812	330.24872	552.7766448	0.742980633	0.108264419	6.86264833	6.76E-12	3.64E-11
ENSG00000196843	ARID5A	897.0125368	670.94925	1123.075824	0.742453478	0.080972342	9.1692232	4.76E-20	3.74E-19
ENSG00000160685	ZBTB7B	1342.847057	1004.6935	1681.000634	0.742170234	0.076590482	9.69011044	3.32E-22	2.80E-21
ENSG00000072840	EVC	2839.336297	2125.1965	3553.476101	0.741813616	0.055002723	13.4868526	1.87E-41	2.66E-40
ENSG00000108515	ENO3	96.48475718	72.110541	120.8589737	0.741659244	0.248335914	2.98651625	0.002822	0.006944
ENSG00000143878	RHOB	30717.28747	22994.17	38440.40465	0.741338616	0.044471346	16.6700288	2.16E-62	4.40E-61
ENSG00000205730	ITPRIPL2	2955.975447	2213.1666	3698.784287	0.741265777	0.052547957	14.1064623	3.47E-45	5.31E-44
ENSG00000147912	FBXO10	331.085027	247.9823	414.1877491	0.741025008	0.128905786	5.74857831	9.00E-09	3.91E-08
ENSG00000286156	AC026273.1	85.34826654	63.930333	106.7661998	0.740965408	0.251673201	2.94415697	0.003238	0.007877
ENSG00000165322	ARHGAP12	1240.205906	928.74628	1551.66553	0.740963305	0.071447683	10.3707115	3.37E-25	3.16E-24
ENSG00000167191	GPRC5B	2876.825616	2154.3919	3599.259371	0.740782687	0.064747887	11.4410326	2.61E-30	2.82E-29
ENSG00000132003	ZSWIM4	1033.460177	773.79791	1293.122439	0.740612378	0.085779459	8.63391288	5.93E-18	4.24E-17
ENSG00000105993	DNAJB6	5352.448366	4010.6292	6694.267491	0.73892184	0.044379386	16.650114	3.02E-62	6.13E-61
ENSG00000141068	KSR1	2326.325141	1743.1206	2909.529696	0.738328212	0.069028998	10.6959138	1.06E-26	1.04E-25
ENSG00000213614	HEXA	3312.658193	2483.4389	4141.877464	0.738236287	0.053671618	13.7546866	4.77E-43	7.05E-42
ENSG00000024048	UBR2	2301.496507	1726.1343	2876.858746	0.737382903	0.058770685	12.5467808	4.14E-36	5.19E-35
ENSG00000269958	AL049840.4	336.7156782	252.47942	420.9519353	0.737250282	0.1218533	6.05031036	1.45E-09	6.66E-09
ENSG00000162604	TM2D1	756.3390461	567.27258	945.4055146	0.737052588	0.093586115	7.87566173	3.39E-15	2.15E-14
ENSG00000136436	CALCOCO2	2933.7493	2200.9526	3666.546008	0.736447009	0.050781372	14.5023063	1.17E-47	1.89E-46
ENSG00000178951	ZBTB7A	2334.910644	1751.6473	2918.173975	0.735734662	0.066678097	11.0341281	2.62E-28	2.69E-27
ENSG00000189050	RNFT1	147.2962897	110.66701	183.9255676	0.735220774	0.208675479	3.52327345	0.000426	0.001182
ENSG00000104219	ZDHHC2	2247.056848	1687.2498	2806.863925	0.734211248	0.058996832	12.4449266	1.49E-35	1.84E-34
ENSG00000177337	DLGAP1-AS1	134.9504974	101.45131	168.4496803	0.734051014	0.204245683	3.59396098	0.000326	0.000917
ENSG00000178381	ZFAND2A	300.7955358	225.8841	375.7069669	0.734046867	0.129600323	5.66392775	1.48E-08	6.33E-08
ENSG00000135622	SEMA4F	748.2153902	562.26059	934.1701911	0.732587658	0.096439833	7.59631822	3.05E-14	1.84E-13
ENSG00000144824	PHLDB2	4223.267987	3176.0468	5270.48921	0.73073011	0.045363793	16.1082233	2.23E-58	4.27E-57
ENSG00000115762	PLEKHB2	5795.60946	4358.2614	7232.957541	0.730575288	0.044209103	16.5254494	2.41E-61	4.81E-60
ENSG00000249464	LINC01091	57.07185583	42.923216	71.22049535	0.728899483	0.299567025	2.43317663	0.014967	0.032381
ENSG00000042286	AIFM2	596.5298468	448.62766	744.4320343	0.728858984	0.114936673	6.34139622	2.28E-10	1.11E-09
ENSG00000164620	RELL2	216.3709085	162.77721	269.9646065	0.727862596	0.15970145	4.55764552	5.17E-06	1.78E-05
ENSG00000196562	SULF2	17702.76167	13330.392	22075.13163	0.72772466	0.143752095	5.06235865	4.14E-07	1.58E-06
ENSG00000111885	MAN1A1	1816.317277	1368.7785	2263.856021	0.725554392	0.070325533	10.3170835	5.90E-25	5.46E-24
ENSG00000189195	BTBD8	134.2507339	101.12819	167.3732745	0.725273201	0.201672961	3.59628379	0.000323	0.00091
ENSG00000033170	FUT8	1269.828156	957.58855	1582.067758	0.72440766	0.072553571	9.98445219	1.78E-23	1.58E-22
ENSG00000143772	ITPKB	3633.829987	2742.4236	4525.236337	0.722694199	0.051698197	13.9790987	2.09E-44	3.15E-43
ENSG00000136770	DNAJC1	1508.469733	1138.4906	1878.448891	0.722590122	0.070235678	10.2880778	7.98E-25	7.35E-24
ENSG00000120279	MYCT1	6556.959975	4949.3352	8164.584733	0.722327889	0.049247942	14.6671689	1.05E-48	1.71E-47
ENSG00000135905	DOCK10	1772.883438	1337.7492	2208.017628	0.722201804	0.071670713	10.0766656	7.01E-24	6.28E-23
ENSG00000153993	SEMA3D	308.8602792	233.05477	384.6657933	0.72194524	0.130760559	5.52112384	3.37E-08	1.40E-07
ENSG00000144655	CSRNP1	867.3595649	654.52247	1080.196664	0.721801164	0.091113214	7.92202502	2.34E-15	1.49E-14
ENSG00000148803	FUOM	315.1293794	238.10812	392.1506376	0.721173436	0.132548701	5.44081859	5.30E-08	2.18E-07
ENSG00000168884	TNIP2	2276.318157	1718.807	2833.829352	0.72092821	0.058407936	12.3429839	5.31E-35	6.47E-34
ENSG00000103043	VAC14	2839.008886	2143.8372	3534.180531	0.720748218	0.055718186	12.9356009	2.83E-38	3.73E-37
ENSG00000023287	RB1CC1	3639.448569	2749.8828	4529.014359	0.720177872	0.05376621	13.3946185	6.50E-41	9.11E-40
ENSG00000101986	ABCD1	1042.060292	786.99864	1297.121946	0.719715004	0.089703965	8.02322396	1.03E-15	6.71E-15
ENSG00000198399	ITSN2	3508.992362	2651.5644	4366.420335	0.719695351	0.051003567	14.1106865	3.26E-45	5.01E-44
ENSG00000102081	FMR1	1197.608514	905.3623	1489.854726	0.719412122	0.072850123	9.87523554	5.33E-23	4.62E-22
ENSG00000174738	NR1D2	1249.007517	944.56256	1553.452474	0.718790835	0.0806626	8.91107945	5.05E-19	3.80E-18
ENSG00000105290	APLP1	527.8467354	398.98902	656.7044506	0.718728558	0.10194927	7.04986467	1.79E-12	9.97E-12
ENSG00000197928	ZNF677	374.8110473	283.5	466.1220908	0.718683864	0.123219748	5.83253799	5.46E-09	2.41E-08
ENSG00000055208	TAB2	3464.900165	2619.7085	4310.091854	0.718527392	0.05302473	13.5507976	7.84E-42	1.12E-40
ENSG00000158079	PTPDC1	376.9242593	284.90287	468.945648	0.718171877	0.123475816	5.81629584	6.02E-09	2.65E-08
ENSG00000134058	CDK7	1236.59596	935.01594	1538.175975	0.717975556	0.072626327	9.8858856	4.79E-23	4.17E-22
ENSG00000069667	RORA	1124.000461	850.19672	1397.804205	0.71770005	0.083693173	8.57537152	9.88E-18	7.02E-17
ENSG00000246898	LINC00920	84.97522822	64.253807	105.6966495	0.717592237	0.257028262	2.79188067	0.00524	0.012321
ENSG00000164180	TMEM161B	195.6368066	147.90459	243.3690248	0.717338612	0.177053544	4.05153489	5.09E-05	0.000158
ENSG00000108960	MMD	423.782323	320.54716	527.0174818	0.716686906	0.130706071	5.48319525	4.18E-08	1.73E-07

ENSG00000198142	SOWAHC	159.8592229	120.896	198.8224409	0.716431124	0.176578066	4.05730531	4.96E-05	0.000155
ENSG00000109270	LAMTOR3	1022.43733	773.57781	1271.29685	0.716268477	0.086055757	8.32330692	8.55E-17	5.85E-16
ENSG00000164970	FAM219A	2372.903799	1796.1533	2949.654334	0.715496584	0.058477189	12.2354818	2.01E-34	2.40E-33
ENSG00000271122	AC018647.2	224.3600792	169.89358	278.8265803	0.715021797	0.159600049	4.48008508	7.46E-06	2.53E-05
ENSG00000179431	FJX1	1246.781882	944.49846	1549.065305	0.713366402	0.075082526	9.50109753	2.08E-21	1.71E-20
ENSG00000131018	SYNE1	4755.5018	3603.9916	5907.011956	0.71304438	0.056942456	12.5221923	5.65E-36	7.04E-35
ENSG00000136026	CKAP4	20012.73569	15165.559	24859.91195	0.712923162	0.037977106	18.7724459	1.27E-78	3.26E-77
ENSG00000204520	MICA	3273.235901	2480.3783	4066.093473	0.712640974	0.053906849	13.2198596	6.74E-40	9.21E-39
ENSG00000124216	SNAI1	632.0021694	478.78762	785.2167166	0.712470809	0.105392678	6.76015474	1.38E-11	7.29E-11
ENSG00000154237	LRRK1	1427.535902	1081.9318	1773.140016	0.712118852	0.067582155	10.5370841	5.83E-26	5.59E-25
ENSG00000143164	DCAF6	3408.320617	2584.2884	4232.352837	0.711709434	0.051464446	13.8291478	1.70E-43	2.54E-42
ENSG00000104774	MAN2B1	3266.616474	2477.8803	4055.352673	0.710751207	0.052559415	13.5228142	1.15E-41	1.64E-40
ENSG00000183726	TMEM50A	5518.437769	4186.8624	6850.013181	0.710246202	0.046021666	15.4328658	9.84E-54	1.75E-52
ENSG00000118507	AKAP7	80.04887361	60.781288	99.31645921	0.710233034	0.267736488	2.65273157	0.007984	0.018198
ENSG00000141298	SSH2	4321.375205	3278.7194	5364.030977	0.710108808	0.049390755	14.3773629	7.18E-47	1.13E-45
ENSG00000118369	USP35	481.501468	365.55664	597.4462946	0.70990667	0.11081522	6.40621992	1.49E-10	7.37E-10
ENSG00000174718	RESF1	8140.649614	6177.9846	10103.31461	0.70984949	0.049951418	14.2107977	7.85E-46	1.22E-44
ENSG00000088881	EBF4	92.06233842	69.863813	114.2608634	0.709245785	0.231847835	3.05910032	0.00222	0.005546
ENSG00000143514	TP53BP2	1495.525024	1135.3038	1855.7462	0.709184183	0.064776024	10.9482512	6.77E-28	6.89E-27
ENSG00000148358	GPR107	6232.59559	4732.321	7732.870165	0.708679227	0.045555574	15.5563669	1.44E-54	2.58E-53
ENSG00000167552	TUBA1A	31042.30934	23575.668	38508.95041	0.707810125	0.045928461	15.411144	1.38E-53	2.43E-52
ENSG00000137449	CPEB2	714.9731643	543.21096	886.7353664	0.707400847	0.09369477	7.55005694	4.35E-14	2.61E-13
ENSG00000116668	SWT1	220.7277655	167.79766	273.6578661	0.707355754	0.163716987	4.32060086	1.56E-05	5.12E-05
ENSG00000131591	C1orf159	339.1593462	257.48459	420.8341031	0.707325159	0.130194458	5.43283616	5.55E-08	2.27E-07
ENSG00000163069	SGCB	5401.966528	4104.1945	6699.738529	0.707034738	0.057398385	12.3180249	7.24E-35	8.78E-34
ENSG00000185947	ZNF267	886.5327713	673.7753	1099.290247	0.70699704	0.081295245	8.69665922	3.42E-18	2.48E-17
ENSG00000113732	ATP6V0E1	5368.896373	4080.4128	6657.379918	0.705963754	0.052619715	13.4163356	4.85E-41	6.82E-40
ENSG00000163430	FSTL1	48093.09887	36555.578	59630.61964	0.705938571	0.032869738	21.4768542	2.56E-102	8.58E-101
ENSG00000164197	RNF180	246.116286	187.40584	304.8267353	0.704297183	0.154237719	4.56630964	4.96E-06	1.71E-05
ENSG00000214106	PAXIP1-AS2	370.4462067	281.90435	458.988061	0.703973857	0.120113412	5.86090964	4.60E-09	2.04E-08
ENSG00000237187	NR2F1-AS1	398.2061526	303.0151	493.3972054	0.703514164	0.117548922	5.98486273	2.17E-09	9.85E-09
ENSG00000162298	SYVN1	1648.547066	1254.4649	2042.629242	0.702862077	0.067006067	10.4895289	9.65E-26	9.19E-25
ENSG00000104324	CPQ	739.9399599	563.28363	916.5962918	0.702454674	0.091797155	7.65224882	1.97E-14	1.21E-13
ENSG00000164647	STEAP1	961.8513399	732.01346	1191.689219	0.70242454	0.084167443	8.34556107	7.09E-17	4.87E-16
ENSG00000134049	IER3IP1	880.1030195	670.15901	1090.047025	0.702267307	0.094560211	7.42666817	1.11E-13	6.53E-13
ENSG00000147894	C9orf72	213.9640306	163.1274	264.8006658	0.701892632	0.190758657	3.67947983	0.000234	0.000671
ENSG00000152484	USP12	2553.453991	1945.2938	3161.614173	0.700444249	0.056065003	12.4934311	8.11E-36	1.01E-34
ENSG00000161955	TNFSF13	71.14167999	54.156184	88.12717623	0.700219297	0.26083879	2.68449067	0.007264	0.01667
ENSG00000153879	CEBPG	1226.326618	934.86558	1517.787653	0.699947672	0.081338178	8.60540141	7.61E-18	5.43E-17
ENSG00000109171	SLAIN2	1888.351227	1438.873	2337.829453	0.699797273	0.068644693	10.1944847	2.10E-24	1.90E-23
ENSG00000127314	RAP1B	6135.951679	4678.0069	7593.896476	0.698877662	0.045311567	15.4238247	1.13E-53	2.00E-52
ENSG00000168917	SLC35G2	452.7881597	345.14058	560.4357427	0.698786328	0.115093156	6.07148461	1.27E-09	5.86E-09
ENSG00000080822	CLDND1	1144.527016	873.00952	1416.044509	0.698732834	0.083396247	8.3784686	5.36E-17	3.71E-16
ENSG00000110697	PITPNM1	1248.091376	951.43048	1544.752272	0.698044244	0.081546194	8.56010814	1.13E-17	8.00E-17
ENSG00000182489	XKRX	81.62975486	62.274838	100.9846715	0.697451689	0.244039917	2.85794101	0.004264	0.010192
ENSG00000232807	AL137186.2	63.63011144	48.57123	78.68899267	0.697370458	0.279459338	2.49542728	0.012581	0.027658
ENSG00000107829	FBXW4	631.0726121	481.3231	780.8221285	0.697239249	0.096657964	7.2134692	5.45E-13	3.10E-12
ENSG00000104447	TRPS1	115.8250962	88.429975	143.2202179	0.696762165	0.213316752	3.26632652	0.00109	0.002858
ENSG00000276107	AC037198.1	232.331068	177.51729	287.1448503	0.696473602	0.178620052	3.89919045	9.65E-05	0.00029
ENSG00000164078	MST1R	73.97056737	56.525168	91.41596653	0.696060779	0.301728288	2.30691257	0.02106	0.04411
ENSG00000112425	EPM2A	152.1100054	116.2046	188.0154089	0.695990194	0.18751037	3.71174242	0.000206	0.000594
ENSG00000162512	SDC3	3941.38872	3008.5996	4874.177867	0.695695977	0.052118029	13.3484705	1.21E-40	1.68E-39
ENSG00000163605	PPP4R2	2345.795036	1791.4576	2900.132496	0.695464747	0.064582102	10.7686918	4.84E-27	4.78E-26
ENSG00000157800	SLC37A3	1791.994708	1368.5663	2215.423157	0.695443222	0.064761197	10.7385788	6.71E-27	6.61E-26
ENSG00000134955	SLC37A2	125.8421095	96.006642	155.6775769	0.695388092	0.198712749	3.49946391	0.000466	0.001287
ENSG00000070444	MNT	779.334143	595.25613	963.4121596	0.695098842	0.089026317	7.8077906	5.82E-15	3.64E-14
ENSG00000117691	NENF	443.8782756	338.84104	548.9155091	0.695079809	0.117961077	5.89245051	3.81E-09	1.70E-08
ENSG00000140265	ZSCAN29	513.4485436	392.27946	634.6176229	0.694998481	0.105523642	6.5861874	4.51E-11	2.31E-10
ENSG00000135916	ITM2C	230.3424221	176.06138	284.6234611	0.693988416	0.158588698	4.37602694	1.21E-05	4.02E-05
ENSG00000115216	NRBP1	7599.11822	5806.0397	9392.196706	0.693666864	0.042449752	16.340893	5.05E-60	9.88E-59
ENSG00000197622	CDC42SE1	3837.604998	2932.8128	4742.39717	0.693647859	0.049258011	14.0819299	4.91E-45	7.48E-44
ENSG00000165118	C9orf64	349.7807314	267.38247	432.1789956	0.693166819	0.129007536	5.3730723	7.74E-08	3.13E-07
ENSG00000165490	DDIAS	479.8421592	366.61609	593.0682309	0.692659851	0.112658618	6.14830772	7.83E-10	3.68E-09
ENSG00000140941	MAP1LC3B	7111.544131	5436.344	8786.744239	0.692476177	0.045860595	15.09959	1.63E-51	2.79E-50
ENSG00000173705	SUSD5	806.6130041	617.00101	996.224998	0.689952767	0.089034575	7.74926804	9.24E-15	5.75E-14
ENSG00000174996	KLC2	1608.259267	1230.4268	1986.091752	0.6898937	0.071332581	9.67150902	3.98E-22	3.36E-21
ENSG00000196712	NF1	2414.406126	1847.9074	2980.904843	0.689806804	0.058579564	11.7755538	5.22E-32	5.88E-31
ENSG00000243156	MICAL3	1779.655814	1362.2224	2197.089219	0.689612945	0.06829193	10.0980152	5.64E-24	5.06E-23
ENSG00000185386	MAPK11	1439.572977	1101.8244	1777.321552	0.689363651	0.075601901	9.11833753	7.63E-20	5.93E-19
ENSG00000119401	TRIM32	923.0238418	706.69537	1139.352311	0.688955383	0.080210446	8.58934735	8.75E-18	6.23E-17
ENSG00000127957	PMS2P3	67.84154966	51.9842	83.69889962	0.688524035	0.26900526	2.55951886	0.010482	0.023366
ENSG00000174233	ADCY6	2295.410954	1758.7176	2832.104342	0.68769517	0.062258445	11.0458134	2.30E-28	2.36E-27
ENSG00000007384	RHBDF1	1866.69927	1430.1111	2303.287453	0.687692618	0.061478801	11.1858495	4.78E-29	5.00E-28
ENSG00000001629	ANKIB1	3430.540172	2628.6369	4232.44346	0.687574759	0.052386107	13.1251356	2.36E-39	3.19E-38
ENSG00000240771	ARHGEF25	273.1894846	209.2643	337.1146643	0.687068908	0.135999002	5.05201434	4.37E-07	1.67E-06
ENSG00000149679	CABLES2	493.2262519	378.08433	608.3681761	0.685799336	0.109369082	6.27050462	3.60E-10	1.73E-09
ENSG00000083312	TNPO1	21266.59638	16306.629	26226.56362	0.685597075	0.043845753	15.6365673	4.10E-55	7.40E-54
ENSG00000224914	LINC00863	254.7659889	195.28691	314.2450643	0.68540919	0.148085734	4.62846197	3.68E-06	1.29E-05
ENSG00000105185	PDCD5	1711.252632	1312.3433	2110.161973	0.685091054	0.063306036	10.8218915	2.71E-27	2.71E-26
ENSG00000279253	AL121753.2	80.44377352	61.755132	99.13241455	0.684224591	0.255816205	2.67467259	0.00748	0.01713
ENSG00000196141	SPATS2L	2626.267201	2014.9506	3237.583841	0.684062583	0.056166676	12.1791537	4.01E-34	4.76E-33
ENSG00000135926	TMBIM1	8547.323652	6562.1226	10532.5247	0.682335098	0.046150633	14.7849563	1.83E-49	3.03E-48
ENSG00000128881	TTBK2	1011.320143	776.93914	1245.701146	0.682068798	0.082936735	8.2239649	1.97E-16	1.33E-15
ENSG00000176170	SPHK1	4396.322321	3375.7735	5416.871169	0.681802708	0.060700146	11.2323075	2.83E-29	2.98E-28
ENSG00000256802	AC022613.1	63.75822775	48.998959	78.51749631	0.681668422	0.296799477	2.29673053	0.021634	0.045188

ENSG00000204524	ZNF805	288.0477869	221.42202	354.6735586	0.681208946	0.136418222	4.99353339	5.93E-07	2.23E-06
ENSG00000144935	TRPC1	297.4358028	228.65574	366.2158695	0.681109811	0.134989711	5.04564242	4.52E-07	1.72E-06
ENSG00000120688	WBP4	661.3012918	508.42361	814.1789786	0.680947451	0.102265505	6.65862306	2.76E-11	1.44E-10
ENSG00000125266	EFNB2	3150.229849	2420.6318	3879.82787	0.680793902	0.080971528	8.40781843	4.18E-17	2.90E-16
ENSG00000170222	ADPRM	250.4581261	192.51051	308.4057453	0.680758631	0.141336833	4.81656915	1.46E-06	5.32E-06
ENSG00000152952	PLOD2	24670.8375	18955.306	30386.36893	0.680723757	0.048149786	14.137628	2.23E-45	3.43E-44
ENSG00000139624	CERS5	1464.309994	1125.4674	1803.152583	0.680347791	0.065723898	10.3516044	4.12E-25	3.84E-24
ENSG00000073712	FERMT2	4601.200573	3536.575	5665.826187	0.679890757	0.045736076	14.8655244	5.52E-50	9.26E-49
ENSG00000161647	MPP3	99.71689968	76.745762	122.6880376	0.679183677	0.241080334	2.81725045	0.004844	0.011454
ENSG00000236104	ZBTB22	707.1164817	543.50858	870.7243851	0.678970802	0.091991786	7.38077638	1.57E-13	9.17E-13
ENSG00000112242	E2F3	1158.013449	890.76854	1425.258357	0.678841862	0.085824623	7.90963993	2.58E-15	1.64E-14
ENSG00000116044	NFE2L2	3545.385645	2726.3732	4364.398064	0.678679717	0.047791648	14.2008017	9.06E-46	1.40E-44
ENSG00000128274	A4GALT	1499.317516	1153.1872	1845.447881	0.678577579	0.069310265	9.79043409	1.24E-22	1.06E-21
ENSG00000169762	TAPT1	526.6589676	404.98305	648.3348885	0.678377	0.105992117	6.40025901	1.55E-10	7.66E-10
ENSG00000149781	FERMT3	7812.573848	6009.2215	9615.92622	0.677929964	0.054761782	12.3796184	3.37E-35	4.12E-34
ENSG00000120690	ELF1	3087.351538	2375.2791	3799.423967	0.677812292	0.055950056	12.1145954	8.84E-34	1.04E-32
ENSG00000167554	ZNF610	131.5811055	101.29583	161.8663787	0.676824276	0.20544274	3.29446676	0.000986	0.002603
ENSG00000161940	BCL6B	6556.034666	5046.1828	8065.886521	0.676554082	0.043203495	15.6597072	2.85E-55	5.17E-54
ENSG00000188786	MTF1	1037.587348	799.00461	1276.170084	0.675794188	0.079886919	8.45938479	2.69E-17	1.88E-16
ENSG00000130363	RSPH3	317.5245761	244.3605	390.6886507	0.67562223	0.141323596	4.78067534	1.75E-06	6.32E-06
ENSG00000197712	FAM114A1	4798.251207	3695.8492	5900.653244	0.67489741	0.046900933	14.3898505	5.99E-47	9.47E-46
ENSG00000118689	FOXO3	1842.937269	1419.5251	2266.349472	0.674787965	0.065526314	10.2979692	7.20E-25	6.64E-24
ENSG00000114554	PLXNA1	4661.86686	3591.1302	5732.603484	0.674354472	0.060531091	11.1406298	7.96E-29	8.29E-28
ENSG00000110455	ACCS	98.22931512	75.757132	120.7014982	0.673841786	0.233551677	2.88519352	0.003912	0.009414
ENSG00000164877	MICALL2	775.1671433	597.24548	953.0888094	0.673675834	0.091248236	7.38289156	1.55E-13	9.03E-13
ENSG00000105520	PLPPR2	1890.152295	1456.494	2323.81063	0.673060868	0.080156926	8.3967899	4.59E-17	3.18E-16
ENSG00000080815	PSEN1	3046.327267	2348.5	3744.154524	0.672971223	0.054106285	12.4379493	1.63E-35	2.00E-34
ENSG00000204152	TIMM23B	107.5454527	82.829971	132.2609347	0.672958855	0.249469487	2.69755978	0.006985	0.016063
ENSG00000005893	LAMP2	11906.16845	9182.1831	14630.15384	0.67221324	0.04236375	15.8676521	1.06E-56	1.97E-55
ENSG00000110925	CSRNP2	1718.438436	1325.4639	2111.412998	0.671997573	0.06428212	10.45388	1.41E-25	1.33E-24
ENSG00000131626	PPFIA1	3408.139717	2628.6959	4187.583511	0.671764069	0.047933454	14.014514	1.27E-44	1.93E-43
ENSG00000136141	LRCH1	661.8746588	510.78491	812.9583959	0.671711679	0.094057424	7.1415062	9.23E-13	5.19E-12
ENSG00000130340	SNX9	4180.791428	3226.35	5135.232856	0.670273295	0.046485314	14.4190335	3.93E-47	6.25E-46
ENSG00000221944	TIGD1	59.54897707	45.988457	73.10949752	0.669713419	0.280881781	2.38432488	0.01711	0.03661
ENSG00000115446	UNC50	790.6952442	610.93477	970.4557142	0.668325973	0.086716049	7.70706205	1.29E-14	7.96E-14
ENSG00000133313	CNDP2	5473.809991	4227.8859	6719.73406	0.668315823	0.048144798	13.8813715	8.22E-44	1.23E-42
ENSG00000258738	AL121603.2	71.74306304	55.457781	88.02834492	0.668255885	0.26257745	2.54498581	0.010928	0.024278
ENSG00000124067	SLC12A4	3958.941683	3058.1862	4859.697159	0.668180114	0.050024953	13.3569363	1.08E-40	1.50E-39
ENSG00000187961	KLHL17	198.1931112	153.11933	243.2668925	0.668139885	0.170281845	3.92372941	8.72E-05	0.000264
ENSG00000166225	FRS2	617.1615208	476.90066	757.422377	0.667657447	0.100961696	6.61297772	3.77E-11	1.94E-10
ENSG00000108061	SHOC2	2265.415492	1751.1021	2779.728861	0.666992434	0.05910086	11.2856638	1.54E-29	1.64E-28
ENSG00000174125	TLR1	236.9093457	183.06624	290.7524561	0.666727966	0.150227041	4.43813552	9.07E-06	3.05E-05
ENSG00000100330	MTMR3	1900.782722	1469.7208	2331.844636	0.665706902	0.068444117	9.72628376	2.33E-22	1.98E-21
ENSG00000120889	TNFRSF10B	17033.70695	13175.542	20891.87187	0.665123629	0.036709321	18.118658	2.27E-73	5.50E-72
ENSG00000130529	TRPM4	1182.798865	915.11231	1450.485419	0.664880205	0.071403054	9.31164942	1.26E-20	1.00E-19
ENSG00000100614	PPM1A	1360.196801	1052.8387	1667.554942	0.664312878	0.074910149	8.86812916	7.44E-19	5.56E-18
ENSG00000124226	RNF114	1489.132544	1152.4067	1825.858387	0.664309467	0.0659933	10.0663169	7.78E-24	6.96E-23
ENSG00000167977	KCTD5	1385.973109	1072.3582	1699.588067	0.663440052	0.079636726	8.3308303	8.03E-17	5.50E-16
ENSG00000114395	CYB561D2	219.8191603	170.12274	269.5155796	0.662825287	0.155846909	4.2530538	2.11E-05	6.84E-05
ENSG00000145431	PDGFC	453.7484691	351.17964	556.3172962	0.662724179	0.129017624	5.13669497	2.80E-07	1.08E-06
ENSG00000102547	CAB39L	380.4474876	294.40648	466.4884926	0.662621166	0.120844234	5.48326672	4.18E-08	1.73E-07
ENSG00000148450	MSRB2	357.5201701	276.69929	438.3410499	0.662080153	0.12868222	5.14507873	2.67E-07	1.04E-06
ENSG00000173039	RELA	5506.470373	4266.2867	6746.654047	0.660825127	0.050266139	13.1465264	1.78E-39	2.42E-38
ENSG00000146433	TMEM181	4110.872945	3185.9874	5035.75847	0.660799587	0.053719543	12.3009161	8.96E-35	1.08E-33
ENSG00000139636	LMBR1L	1786.46212	1384.5256	2188.398609	0.660664958	0.059944443	11.0212877	3.02E-28	3.10E-27
ENSG00000180488	MIGA1	738.0634148	572.39336	903.733469	0.65992926	0.092026368	7.17108886	7.44E-13	4.21E-12
ENSG00000129003	VPS13C	6746.896727	5231.8495	8261.943952	0.659399357	0.048505018	13.5944566	4.32E-42	6.24E-41
ENSG00000233621	LINC01137	76.12341168	58.919852	93.32697096	0.659346154	0.268240345	2.45804245	0.01397	0.030411
ENSG00000168487	BMP1	4473.336155	3468.43	5478.242305	0.659339742	0.051586963	12.7811312	2.09E-37	2.70E-36
ENSG00000072310	SREBF1	3086.950488	2393.3853	3780.51564	0.65899475	0.056797854	11.6024586	4.00E-31	4.40E-30
ENSG00000141428	C18orf21	428.0859581	332.05677	524.115144	0.658592544	0.110732741	5.94758637	2.72E-09	1.23E-08
ENSG00000092964	DPYSL2	8776.261697	6807.9932	10744.53019	0.658268024	0.050551245	13.0217966	9.20E-39	1.23E-37
ENSG00000158158	CNNM4	393.2478875	305.07227	481.4235085	0.656862674	0.122234248	5.37380225	7.71E-08	3.12E-07
ENSG00000147883	CDKN2B	468.2775148	363.40071	573.1543229	0.656838249	0.106347635	6.17633154	6.56E-10	3.10E-09
ENSG00000115295	CLIP4	1456.611181	1130.9155	1782.30684	0.656831154	0.06796355	9.66446205	4.27E-22	3.59E-21
ENSG00000114626	ABTB1	382.7271296	297.21453	468.2397329	0.656380703	0.11844049	5.54186076	2.99E-08	1.25E-07
ENSG00000166471	TMEM41B	882.8173141	685.65291	1079.981715	0.65633203	0.106188615	6.18081356	6.38E-10	3.01E-09
ENSG00000145979	TBC1D7	693.9043762	538.83229	848.9764613	0.656245943	0.089357298	7.34406654	2.07E-13	1.20E-12
ENSG00000139146	SINHCAF	1960.700131	1522.5389	2398.861405	0.656242655	0.061630721	10.6479795	1.78E-26	1.74E-25
ENSG00000135185	TMEM243	441.8883543	343.36924	540.4074724	0.65562824	0.111257201	5.89290611	3.79E-09	1.70E-08
ENSG00000270194	AC097359.2	66.73734042	51.861799	81.61288187	0.655424851	0.286618571	2.28674942	0.02221	0.046263
ENSG00000268858	AL118506.1	199.721368	155.17623	244.2665068	0.655127991	0.16879768	3.88114334	0.000104	0.000311
ENSG00000184635	ZNF93	118.7620656	92.14871	145.3754213	0.655015481	0.229543266	2.85355999	0.004323	0.010314
ENSG00000253352	TUG1	4935.387239	3834.9351	6035.839418	0.65473806	0.056107534	11.6693431	1.83E-31	2.03E-30
ENSG00000213190	MLLT11	3878.659807	3012.8939	4744.425689	0.654558779	0.058402489	11.2077206	3.74E-29	3.92E-28
ENSG00000198087	CD2AP	3276.702882	2546.3861	4007.019689	0.654191428	0.050606632	12.9269901	3.17E-38	4.16E-37
ENSG00000166949	SMAD3	5161.606381	4010.7223	6312.490444	0.654025808	0.053039425	12.3309369	6.17E-35	7.50E-34
ENSG00000112234	FBXL4	578.0436824	449.44768	706.6396848	0.653371234	0.102711787	6.36120988	2.00E-10	9.78E-10
ENSG00000162852	CNST	925.0221468	719.15873	1130.885559	0.653171515	0.079783262	8.18682391	2.68E-16	1.80E-15
ENSG00000130734	ATG4D	405.6749301	315.39097	495.9588944	0.653151009	0.114170813	5.72082295	1.06E-08	4.58E-08
ENSG00000132694	ARHGEF11	1937.416622	1506.2739	2368.559368	0.653124708	0.05990352	10.9029437	1.12E-27	1.13E-26
ENSG00000129474	AJUBA	2220.115434	1726.2945	2713.936341	0.652693958	0.058535886	11.1503216	7.13E-29	7.44E-28
ENSG00000141699	RETREG3	1650.766308	1283.9905	2017.54209	0.652006003	0.067616991	9.64263565	5.28E-22	4.42E-21
ENSG00000103404	USP31	1253.757347	975.58878	1531.925913	0.651919982	0.077929385	8.36552197	5.98E-17	4.13E-16
ENSG00000142961	MOB3C	383.7517718	298.72083	468.7827144	0.651873392	0.129625467	5.02889908	4.93E-07	1.87E-06

ENSG00000085998	POMGNT1	3036.248206	2362.0755	3710.420863	0.651612857	0.055056509	11.8353465	2.56E-32	2.92E-31
ENSG00000197774	EME2	294.1401865	228.74066	359.5397112	0.651134545	0.142164317	4.58015458	4.65E-06	1.61E-05
ENSG00000116273	PHF13	891.9599466	693.92194	1089.997957	0.650954213	0.079962241	8.14077004	3.93E-16	2.61E-15
ENSG00000146425	DYNLT1	2651.053401	2062.9707	3239.136116	0.650662745	0.059268804	10.9781655	4.87E-28	4.96E-27
ENSG00000050130	JKAMP	1260.253377	981.07697	1539.429786	0.650430687	0.069057299	9.41871022	4.57E-21	3.71E-20
ENSG00000228742	LINC02577	121.4318621	94.631658	148.232066	0.649915284	0.226297674	2.87194859	0.004079	0.009787
ENSG00000168038	ULK4	86.50670509	67.431151	105.5822591	0.649848507	0.253396654	2.56455047	0.010331	0.023052
ENSG00000066455	GOLGA5	2296.940063	1788.4784	2805.401733	0.649764727	0.057413539	11.3172736	1.08E-29	1.15E-28
ENSG00000185722	ANKFY1	3174.217576	2472.2991	3876.136053	0.648963547	0.050973949	12.7312786	3.96E-37	5.09E-36
ENSG00000033867	SLC4A7	2378.438029	1852.5661	2904.309934	0.648936597	0.061532474	10.5462458	5.29E-26	5.08E-25
ENSG00000121858	TNFSF10	1730.231954	1348.5556	2111.908308	0.648060813	0.069188982	9.36653205	7.50E-21	6.04E-20
ENSG00000146826	C7orf43	430.5205454	335.31904	525.7220488	0.647974228	0.123759558	5.23575097	1.64E-07	6.47E-07
ENSG00000198818	SFT2D1	316.1269311	246.30615	385.9477116	0.647482811	0.134564908	4.81167668	1.50E-06	5.45E-06
ENSG00000181104	F2R	34507.1322	26896.475	42117.78938	0.647063635	0.037818334	17.1097867	1.25E-65	2.72E-64
ENSG00000127191	TRAF2	553.3018205	431.15223	675.4514119	0.6461369	0.104286286	6.19579932	5.80E-10	2.75E-09
ENSG00000100097	LGALS1	43841.0138	34189.446	53492.58146	0.64572129	0.048984011	13.1822871	1.11E-39	1.51E-38
ENSG00000101782	RIOK3	3364.239863	2623.998	4104.481734	0.645560143	0.053998105	11.955237	6.10E-33	7.05E-32
ENSG00000179476	C14orf28	94.24807313	73.565466	114.9306803	0.645314218	0.232836818	2.77152996	0.005579	0.013067
ENSG00000142949	PTPRF	31565.32017	24621.05	38509.59075	0.645293676	0.039874945	16.1829359	6.65E-59	1.28E-57
ENSG00000095637	SORBS1	121.2880833	94.495564	148.0806026	0.644953106	0.210531291	3.06345486	0.002188	0.005474
ENSG00000162976	PQLC3	547.1879096	427.18108	667.1947396	0.644313028	0.102598706	6.27993327	3.39E-10	1.63E-09
ENSG00000119231	SENP5	1669.678475	1303.079	2036.277984	0.644158711	0.065710579	9.80296814	1.09E-22	9.39E-22
ENSG00000130311	DDA1	2422.55955	1890.8286	2954.290458	0.643623839	0.054739832	11.7578702	6.43E-32	7.23E-31
ENSG00000008086	CDKL5	241.9175619	188.99293	294.8421941	0.643564825	0.15163188	4.24425804	2.19E-05	7.10E-05
ENSG00000103642	LACTB	2112.406161	1649.4398	2575.372505	0.643201813	0.064914995	9.90837035	3.83E-23	3.35E-22
ENSG00000173786	CNP	5741.848778	4484.4171	6999.280436	0.641993326	0.04656262	13.7877405	3.02E-43	4.48E-42
ENSG00000068400	GRIPAP1	1577.361892	1232.7444	1921.979382	0.641435791	0.068839219	9.31788301	1.19E-20	9.48E-20
ENSG00000284968	AC093827.4	94.73008982	73.961205	115.4989744	0.641104482	0.23626998	2.71344028	0.006659	0.015367
ENSG00000128039	SRD5A3	897.9623432	701.71526	1094.209429	0.64084049	0.081500456	7.86302946	3.75E-15	2.37E-14
ENSG00000122557	HERPUD2	1261.68593	986.18161	1537.190251	0.640227984	0.072286555	8.8568058	8.23E-19	6.14E-18
ENSG00000099968	BCL2L13	4159.398301	3252.0353	5066.761294	0.639460802	0.048841987	13.0924404	3.64E-39	4.89E-38
ENSG00000116954	RRAGC	1302.856114	1018.8626	1586.849672	0.639451754	0.07114855	8.98755847	2.53E-19	1.92E-18
ENSG00000150938	CRIM1	60947.74202	47663.581	74231.90296	0.639183777	0.047745695	13.3872547	7.18E-41	1.00E-39
ENSG00000103126	AXIN1	937.8741044	733.41881	1142.329396	0.638543072	0.081570208	7.82814074	4.95E-15	3.11E-14
ENSG00000270231	NBPF8	468.2116474	366.55155	569.871741	0.638150196	0.110281791	5.78654184	7.19E-09	3.15E-08
ENSG00000277072	STAG3L2	144.2565927	112.86653	175.6466592	0.6376677	0.200268271	3.18406753	0.001452	0.003733
ENSG00000141959	PFKL	9095.465414	7116.9595	11073.97131	0.637652337	0.042960674	14.8426988	7.76E-50	1.30E-48
ENSG00000135148	TRAFD1	1452.094316	1136.4382	1767.750446	0.63718285	0.066987073	9.5120271	1.87E-21	1.54E-20
ENSG00000010278	CD9	43385.29352	33968.891	52801.6959	0.636322646	0.043577039	14.6022461	2.72E-48	4.42E-47
ENSG00000138035	PNPT1	1515.909979	1186.9376	1844.882363	0.636126585	0.077107981	8.24981514	1.59E-16	1.07E-15
ENSG00000182220	ATP6AP2	6074.836347	4757.9222	7391.750534	0.635602013	0.044608211	14.248543	4.58E-46	7.14E-45
ENSG00000159363	ATP13A2	4232.907796	3315.5617	5150.253874	0.635060379	0.049195645	12.908874	4.01E-38	5.26E-37
ENSG00000197013	ZNF429	66.41403224	52.079867	80.74819712	0.635037096	0.270622842	2.34657611	0.018947	0.040146
ENSG00000176182	MYPOP	394.0926838	308.49955	479.6858205	0.635021613	0.137006283	4.63498168	3.57E-06	1.25E-05
ENSG00000100368	CSF2RB	2328.628909	1824.6873	2832.570523	0.634029308	0.060340367	10.5075481	7.97E-26	7.61E-25
ENSG00000168734	PKIG	4732.578216	3709.8435	5755.312917	0.633338242	0.045818419	13.8238729	1.83E-43	2.72E-42
ENSG00000166750	SLFN5	4624.305955	3626.225	5622.386902	0.632966898	0.051563329	12.2755244	1.23E-34	1.47E-33
ENSG00000143862	ARL8A	1760.239	1380.2527	2140.225335	0.632777148	0.066989221	9.44595467	3.52E-21	2.87E-20
ENSG00000102901	CENPT	893.5851623	700.59794	1086.572381	0.632756813	0.090022766	7.0288533	2.08E-12	1.15E-11
ENSG00000108669	CYTH1	2231.678569	1750.408	2712.949154	0.632142446	0.05696323	11.0973772	1.29E-28	1.34E-27
ENSG00000270959	LPP-AS2	134.6369451	105.68158	163.5923064	0.632077305	0.197263515	3.20422813	0.001354	0.003503
ENSG00000151012	SLC7A11	6647.172924	5214.2862	8080.059621	0.631731328	0.042808928	14.7569993	2.77E-49	4.57E-48
ENSG00000157613	CREB3L1	127.7716021	100.10696	155.4362478	0.631645606	0.206320917	3.06147149	0.002203	0.005505
ENSG00000133678	TMEM254	277.0556627	217.385	336.726326	0.631601965	0.156089905	4.04639855	5.20E-05	0.000162
ENSG00000114933	INO80D	733.6884124	575.64072	891.7361011	0.630781678	0.087712762	7.19144696	6.41E-13	3.63E-12
ENSG00000110719	TCIRG1	2398.748697	1882.1953	2915.302118	0.630565481	0.065241132	9.66515234	4.24E-22	3.57E-21
ENSG00000011132	APBA3	506.2244605	397.15756	615.2913573	0.630513171	0.108905485	5.78954467	7.06E-09	3.09E-08
ENSG00000197608	ZNF841	314.2726743	246.78789	381.7574631	0.630461097	0.134477678	4.68822119	2.76E-06	9.78E-06
ENSG00000114209	PDCD10	1233.652677	968.41251	1498.892848	0.629948822	0.069659475	9.04326108	1.52E-19	1.17E-18
ENSG00000173264	GPR137	1048.530838	822.95418	1274.107494	0.629750637	0.078512233	8.02105114	1.05E-15	6.82E-15
ENSG00000107099	DOCK8	198.2287721	155.66501	240.7925365	0.629329583	0.158424944	3.97241474	7.11E-05	0.000217
ENSG00000172638	EFEMP2	3697.905687	2903.9519	4491.859454	0.629172355	0.051134865	12.3041756	8.60E-35	1.04E-33
ENSG00000131100	ATP6V1E1	4581.979891	3598.3778	5565.581963	0.629026607	0.04666411	13.4798802	2.05E-41	2.92E-40
ENSG00000101400	SNTA1	329.1946344	258.58025	399.8090227	0.628991164	0.140905099	4.46393472	8.05E-06	2.72E-05
ENSG00000155252	PI4K2A	1500.342674	1178.2135	1822.471835	0.628803814	0.067425969	9.32584025	1.10E-20	8.81E-20
ENSG00000052841	TTC17	2443.702449	1921.0858	2966.319113	0.627389106	0.060312355	10.4023315	2.42E-25	2.28E-24
ENSG00000010327	STAB1	26902.31705	21149.842	32654.79198	0.626702049	0.037174378	16.8584407	9.10E-64	1.91E-62
ENSG00000169760	NLGN1	2308.530779	1814.7395	2802.322084	0.626298815	0.069344306	9.03172667	1.69E-19	1.30E-18
ENSG00000213753	CENPBD1P1	862.1082058	677.91981	1046.296597	0.626242151	0.082176179	7.62072612	2.52E-14	1.53E-13
ENSG00000038219	BOD1L1	3938.535589	3098.3519	4778.71926	0.625472217	0.060554863	10.3290171	5.21E-25	4.83E-24
ENSG00000127481	UBR4	12401.53742	9757.815	15045.25983	0.624704186	0.038887725	16.0643027	4.54E-58	8.61E-57
ENSG00000142632	ARHGEF19	328.6475217	258.71858	398.5764636	0.624660738	0.136393521	4.57984171	4.65E-06	1.61E-05
ENSG00000270055	AC127502.2	198.5729267	156.27147	240.8743832	0.624396379	0.170941469	3.65269107	0.00026	0.000741
ENSG00000019995	ZRANB1	1515.172458	1192.4449	1837.899975	0.623865134	0.069313484	9.00063156	2.24E-19	1.71E-18
ENSG00000187676	B3GLCT	1052.581009	828.41225	1276.749764	0.623729289	0.077444216	8.05391699	8.02E-16	5.24E-15
ENSG00000186591	UBE2H	9547.5451	7515.5513	11579.53891	0.623722459	0.040947576	15.2322192	2.16E-52	3.77E-51
ENSG00000132963	POMP	3286.763254	2588.0317	3985.494806	0.623117512	0.057173328	10.8987448	1.17E-27	1.18E-26
ENSG00000035664	DAPK2	185.9925343	146.43792	225.5471466	0.622134636	0.180964149	3.43788889	0.000586	0.001598
ENSG00000146830	GIGYF1	1516.653982	1194.993	1838.315005	0.621852395	0.065684017	9.46733199	2.87E-21	2.35E-20
ENSG00000084764	MAPRE3	273.0286278	215.05783	330.9994281	0.621582588	0.155360161	4.00091364	6.31E-05	0.000194
ENSG00000011566	MAP4K3	1133.470791	893.4555	1373.486077	0.621163876	0.07625605	8.14576518	3.77E-16	2.50E-15
ENSG00000166033	HTRA1	14122.6627	11131.83	17113.49591	0.620602357	0.05408498	11.4745786	1.77E-30	1.92E-29
ENSG00000119844	AFTPH	1062.278007	837.68372	1286.872291	0.620003585	0.075485858	8.21350645	2.15E-16	1.45E-15
ENSG00000153982	GDPD1	183.2227826	144.3189	222.126662	0.619777402	0.197602699	3.13648248	0.00171	0.004343
ENSG00000136231	IGF2BP3	4860.343923	3832.3364	5888.35141	0.619311296	0.050520723	12.2585596	1.51E-34	1.81E-33

ENSG00000221978	CCNL2	1230.766912	970.80013	1490.733694	0.619124527	0.212154674	2.91826956	0.00352	0.008523
ENSG00000179454	KLHL28	330.8677872	260.72051	401.0150689	0.619057819	0.136840775	4.52392803	6.07E-06	2.08E-05
ENSG00000235823	OLMALINC	92.51850014	72.985652	112.0513488	0.61881286	0.235256258	2.63037789	0.008529	0.019337
ENSG00000036054	TBC1D23	1549.607558	1222.6514	1876.563732	0.618453533	0.07366098	8.39594497	4.62E-17	3.20E-16
ENSG00000113615	SEC24A	2518.427197	1986.5546	3050.299764	0.61842213	0.055762823	11.0902228	1.40E-28	1.45E-27
ENSG00000215154	AC141586.1	119.7282508	94.541362	144.9151402	0.618382999	0.206791486	2.99036973	0.002786	0.00686
ENSG00000100852	ARHGAP5	2522.620672	1990.4541	3054.787256	0.617984572	0.058044684	10.6467041	1.81E-26	1.76E-25
ENSG00000182218	HHIPL1	375.7335626	296.42705	455.0400704	0.617354117	0.141277164	4.36980824	1.24E-05	4.13E-05
ENSG00000157827	FMNL2	3868.449857	3053.7141	4683.185664	0.616980588	0.049611017	12.4363625	1.66E-35	2.04E-34
ENSG00000100485	SOS2	1829.883922	1444.8292	2214.938669	0.616931505	0.06211841	9.93154044	3.04E-23	2.66E-22
ENSG00000260077	AC104794.2	79.40584243	62.766984	96.04470052	0.616422578	0.2537096	2.42963836	0.015114	0.032665
ENSG00000049449	RCN1	12229.93055	9657.3607	14802.50042	0.616209529	0.037454026	16.4524246	8.06E-61	1.60E-59
ENSG00000132432	SEC61G	2008.728567	1587.29	2430.167182	0.615170911	0.061871185	9.94276921	2.71E-23	2.38E-22
ENSG00000237172	B3GNT9	475.2193153	375.42737	575.0112603	0.614825201	0.122317029	5.02648902	5.00E-07	1.89E-06
ENSG00000065615	CYB5R4	524.8667364	414.64025	635.093219	0.614150306	0.113800266	5.39673874	6.79E-08	2.76E-07
ENSG00000166401	SERPINB8	1488.058931	1176.3293	1799.78854	0.612784322	0.069481465	8.81939262	1.15E-18	8.53E-18
ENSG00000159842	ABR	5909.614116	4672.8508	7146.377481	0.612629206	0.045683256	13.4103665	5.26E-41	7.39E-40
ENSG00000182318	ZSCAN22	98.06204779	77.502744	118.6213517	0.61214394	0.241210141	2.5378035	0.011155	0.024747
ENSG00000205593	DENND6B	146.5956116	115.82645	177.3647745	0.611960764	0.192145517	3.18488182	0.001448	0.003724
ENSG00000100241	SBF1	3618.740392	2862.387	4375.093736	0.611633184	0.060583721	10.0956688	5.77E-24	5.18E-23
ENSG00000134108	ARL8B	3274.008846	2590.8434	3957.174273	0.611135876	0.060190901	10.1532933	3.20E-24	2.89E-23
ENSG00000196967	ZNF585A	132.8964781	105.07518	160.7177765	0.610961297	0.200206639	3.05165353	0.002276	0.005678
ENSG00000047644	WWC3	3483.813769	2757.1127	4210.514887	0.610909548	0.04765524	12.8193574	1.28E-37	1.66E-36
ENSG00000274070	CASTOR2	124.2834525	98.339803	150.2271024	0.610891779	0.206380069	2.96003283	0.003076	0.007517
ENSG00000155090	KLF10	2337.12969	1849.5267	2824.732651	0.610787224	0.055595009	10.9863678	4.44E-28	4.54E-27
ENSG00000111540	RAB5B	5107.72196	4042.9019	6172.542012	0.610406885	0.044623503	13.6790444	1.35E-42	1.98E-41
ENSG00000186908	ZDHHC17	595.2997959	471.45936	719.1402322	0.610194403	0.100775933	6.05496159	1.40E-09	6.47E-09
ENSG00000182648	LINC01006	101.0610116	80.026945	122.0950779	0.609881831	0.224585126	2.71559315	0.006616	0.015274
ENSG00000078142	PIK3C3	1512.840313	1198.0428	1827.6378	0.609748113	0.068250424	8.93398283	4.11E-19	3.10E-18
ENSG00000167378	IRGQ	1353.571205	1072.1889	1634.953545	0.60936517	0.069810383	8.72886152	2.57E-18	1.87E-17
ENSG00000183723	CMTM4	449.5456921	356.08162	543.0097617	0.609357803	0.116807641	5.21676321	1.82E-07	7.14E-07
ENSG00000117000	RLF	830.0485205	657.41883	1002.678209	0.608931892	0.0875267	6.95709871	3.47E-12	1.91E-11
ENSG00000165914	TTC7B	2092.542364	1657.4634	2527.621369	0.608634894	0.057531303	10.5791953	3.72E-26	3.59E-25
ENSG00000108799	EZH1	586.0881707	464.3116	707.8647414	0.608194185	0.098517948	6.17343534	6.68E-10	3.15E-09
ENSG00000153066	TXNDC11	2371.569811	1879.4595	2863.68012	0.607369546	0.05818767	10.4381142	1.66E-25	1.57E-24
ENSG00000155304	HSPA13	2531.097891	2006.0853	3056.110469	0.607326414	0.056141352	10.8178089	2.83E-27	2.83E-26
ENSG00000040487	PQLC2	562.064173	445.38812	678.7402304	0.606933504	0.097959236	6.19577619	5.80E-10	2.75E-09
ENSG00000143603	KCNN3	78.10780413	61.884793	94.33081497	0.606915194	0.257456309	2.35735219	0.018406	0.039092
ENSG00000129493	HEATR5A	1056.213889	837.34892	1275.07886	0.606833807	0.075818132	8.00380848	1.21E-15	7.83E-15
ENSG00000188171	ZNF626	145.070066	115.16307	174.9770667	0.606522503	0.207449656	2.92370937	0.003459	0.008387
ENSG00000165195	PIGA	259.5007872	205.76104	313.2405357	0.606046766	0.140485948	4.31393171	1.60E-05	5.27E-05
ENSG00000099954	CECR2	353.1250914	280.13298	426.1172076	0.605826873	0.125755801	4.81748652	1.45E-06	5.30E-06
ENSG00000104343	UBE2W	867.3270154	688.1026	1046.551431	0.605223488	0.084858934	7.13211277	9.88E-13	5.55E-12
ENSG00000185561	TLCD2	430.4686589	341.77633	519.1609833	0.604825716	0.126439909	4.78350324	1.72E-06	6.24E-06
ENSG00000183696	UPP1	1530.244963	1214.3117	1846.178246	0.604716515	0.063987078	9.45060362	3.37E-21	2.76E-20
ENSG00000183337	BCOR	1222.798296	970.60831	1474.988286	0.604706352	0.079037001	7.65092735	2.00E-14	1.22E-13
ENSG00000151348	EXT2	6615.522027	5249.9824	7981.061638	0.604322349	0.045612348	13.2490954	4.57E-40	6.28E-39
ENSG00000169621	APLF	151.4666215	120.15481	182.7784379	0.604317608	0.192810804	3.13425179	0.001723	0.004373
ENSG00000082397	EPB41L3	7202.10684	5715.9318	8688.281904	0.603921795	0.043452371	13.8984775	6.47E-44	9.70E-43
ENSG00000111666	CHPT1	864.6491327	686.14033	1043.157938	0.603582218	0.094879832	6.36154394	2.00E-10	9.77E-10
ENSG00000179044	EXOC3L1	131.4890346	104.49454	158.4835306	0.603245063	0.205877366	2.93011842	0.003388	0.008224
ENSG00000267317	AC027307.2	118.4436813	94.099825	142.7875379	0.602918565	0.209260144	2.88119158	0.003962	0.009522
ENSG00000136603	SKIL	4076.358896	3237.7227	4914.995064	0.60257601	0.070576725	8.53788568	1.37E-17	9.67E-17
ENSG00000170949	ZNF160	816.9222002	649.13416	984.7102403	0.602336	0.090562187	6.65107612	2.91E-11	1.51E-10
ENSG00000156860	FBRS	4115.154283	3268.1175	4962.191086	0.602042307	0.05471954	11.0023276	3.72E-28	3.81E-27
ENSG00000166340	TPP1	14130.82257	11226.676	17034.96885	0.601571843	0.05429519	11.0796526	1.57E-28	1.63E-27
ENSG00000119965	C10orf88	480.5884276	382.09019	579.0866676	0.601226293	0.109421179	5.49460625	3.92E-08	1.63E-07
ENSG00000085491	SLC25A24	2059.593371	1636.7973	2482.389436	0.601043839	0.065765816	9.1391527	6.29E-20	4.92E-19
ENSG00000143479	DYRK3	539.5883568	428.63496	650.5417492	0.600613651	0.103975546	5.77648953	7.63E-09	3.33E-08
ENSG00000138433	CIR1	1130.957658	899.23598	1362.679336	0.599999609	0.072281882	8.30082988	1.03E-16	7.06E-16
ENSG00000074621	SLC24A1	371.7418955	295.69737	447.7864166	0.599457586	0.141970723	4.22240286	2.42E-05	7.79E-05
ENSG00000068697	LAPTM4A	9780.196255	7778.8906	11781.50191	0.598835767	0.042480597	14.096689	3.98E-45	6.09E-44
ENSG00000213516	RBMXL1	426.9522033	339.52671	514.3777	0.598823035	0.113261175	5.28709891	1.24E-07	4.95E-07
ENSG00000263753	LINC00667	792.6251197	630.58757	954.6626727	0.598722781	0.088805684	6.74194208	1.56E-11	8.25E-11
ENSG00000140545	MFGE8	3440.092832	2737.1655	4143.020177	0.598265746	0.051076873	11.7130457	1.09E-31	1.22E-30
ENSG00000137207	YIPF3	4462.786562	3549.9291	5375.644014	0.598260815	0.052118729	11.4788066	1.69E-30	1.83E-29
ENSG00000100003	SEC14L2	860.7318181	684.82619	1036.637445	0.598080115	0.081917661	7.3009911	2.86E-13	1.65E-12
ENSG00000076108	BAZ2A	5552.497244	4417.7853	6687.209182	0.598063043	0.044118648	13.5557882	7.32E-42	1.05E-40
ENSG00000105186	ANKRD27	1425.023599	1133.8363	1716.210886	0.597295496	0.070626829	8.45706232	2.74E-17	1.91E-16
ENSG00000177105	RHOG	1871.700697	1489.664	2253.737358	0.596440468	0.070065904	8.51256372	1.70E-17	1.20E-16
ENSG00000184402	SS18L1	380.7732835	303.4742	458.072366	0.596250733	0.134365401	4.43753175	9.10E-06	3.06E-05
ENSG00000143469	SYT14	334.7434999	266.73134	402.7556645	0.595650896	0.148557414	4.00956695	6.08E-05	0.000188
ENSG00000144426	NBEAL1	718.3224581	572.21046	864.4344539	0.5956154	0.098633188	6.03869158	1.55E-09	7.14E-09
ENSG00000182963	GJC1	1199.187781	955.36258	1443.012982	0.595341445	0.078838725	7.55138352	4.31E-14	2.58E-13
ENSG00000109680	TBC1D19	155.4717399	123.86263	187.0808539	0.595337118	0.185112037	3.2160908	0.001299	0.003371
ENSG00000183255	PTTG1IP	23933.6589	19065.125	28802.19251	0.59524021	0.035844089	16.6063701	6.27E-62	1.27E-60
ENSG00000123358	NR4A1	101.0994328	80.434842	121.764024	0.594879689	0.245595734	2.42219064	0.015427	0.033285
ENSG00000054267	ARID4B	2466.642249	1965.6623	2967.622189	0.594665563	0.054987637	10.814532	2.94E-27	2.93E-26
ENSG00000163625	WDFY3	7005.872906	5582.6955	8429.050294	0.594545652	0.045053571	13.1964158	9.20E-40	1.26E-38
ENSG00000173681	BCLAF3	262.1040422	209.04402	315.1640602	0.594485029	0.146724986	4.05169594	5.08E-05	0.000158
ENSG00000176142	TMEM39A	753.3590264	600.24518	906.4728731	0.594483831	0.089740813	6.62445334	3.49E-11	1.80E-10
ENSG00000133069	TMCC2	157.1070086	125.04138	189.1726396	0.594242438	0.198024666	3.0008506	0.002692	0.006642
ENSG00000132823	OSER1	928.2526581	739.88861	1116.616704	0.593725584	0.089181596	6.65749003	2.79E-11	1.45E-10
ENSG00000167565	SERTAD3	725.8826332	578.37431	873.3909551	0.593702929	0.097932886	6.06234489	1.34E-09	6.19E-09
ENSG00000092330	TINF2	1524.077215	1214.6613	1833.493138	0.593473922	0.065556283	9.05289163	1.39E-19	1.07E-18

ENSG00000099326	MZF1	229.2374724	182.81096	275.6639877	0.593428271	0.167183374	3.5495651	0.000386	0.001077
ENSG00000135766	EGLN1	2476.898769	1974.6588	2979.1387	0.593212291	0.056381028	10.5214877	6.88E-26	6.57E-25
ENSG00000186162	CIDECP1	158.4556916	126.15459	190.7567917	0.593168681	0.198666989	2.98574355	0.002829	0.00696
ENSG00000144843	ADPRH	431.8176022	344.05832	519.5768852	0.593007884	0.114975398	5.15769371	2.50E-07	9.72E-07
ENSG00000134970	TMED7	3416.702497	2724.7365	4108.668494	0.592694839	0.0555909	10.6617241	1.54E-26	1.50E-25
ENSG00000197948	FCHSD1	901.2222176	718.73526	1083.70918	0.5924188	0.089449745	6.62292326	3.52E-11	1.81E-10
ENSG00000188419	CHM	1241.316062	990.65775	1491.974375	0.591924318	0.078564235	7.53427201	4.91E-14	2.93E-13
ENSG00000198961	PJA2	5460.488249	4357.0972	6563.879341	0.591393096	0.043960999	13.4526764	2.97E-41	4.20E-40
ENSG00000185619	PCGF3	3074.00732	2452.7821	3695.232523	0.591085047	0.057153486	10.3420646	4.55E-25	4.23E-24
ENSG00000059769	DNAJC25	214.9941366	171.52862	258.4596503	0.590886846	0.171649627	3.44240098	0.000577	0.001572
ENSG00000205133	TRIQK	612.2993189	488.85034	735.7482996	0.590662479	0.098632268	5.98853187	2.12E-09	9.65E-09
ENSG00000121413	ZSCAN18	166.7372267	133.03625	200.4382079	0.590306282	0.176619333	3.34225179	0.000831	0.002216
ENSG00000085433	WDR47	1284.275193	1025.0662	1543.484234	0.589491166	0.074111845	7.95407484	1.80E-15	1.16E-14
ENSG00000196151	WDSUB1	184.1322538	146.98622	221.2782867	0.589461014	0.161950683	3.63975628	0.000273	0.000778
ENSG00000096968	JAK2	780.4513558	623.49053	937.412183	0.589340019	0.090586981	6.50579159	7.73E-11	3.89E-10
ENSG00000198855	FICD	476.7322737	380.69	572.7745473	0.589281562	0.103510396	5.69296983	1.25E-08	5.37E-08
ENSG00000196371	FUT4	424.1589387	338.70342	509.614459	0.588974144	0.117156452	5.02724464	4.98E-07	1.89E-06
ENSG00000113319	RASGRF2	1682.011869	1343.4799	2020.54384	0.588141732	0.064404075	9.13205774	6.72E-20	5.24E-19
ENSG00000184903	IMMP2L	126.0406331	100.86161	151.2196546	0.587250401	0.217544761	2.6994463	0.006945	0.015974
ENSG00000146242	TPBG	821.202926	656.56266	985.8431905	0.586240964	0.093086232	6.29782677	3.02E-10	1.46E-09
ENSG00000117036	ETV3	1271.260673	1016.8249	1525.696418	0.586135254	0.077086886	7.60356641	2.88E-14	1.74E-13
ENSG00000134153	EMC7	2034.231869	1626.693	2441.770698	0.586065822	0.063133979	9.28289066	1.65E-20	1.31E-19
ENSG00000125304	TM9SF2	7746.01705	6194.0748	9297.959299	0.586059296	0.043500242	13.4725524	2.27E-41	3.22E-40
ENSG00000258890	CEP95	328.9803977	263.3497	394.6110939	0.586033011	0.154696983	3.78826401	0.000152	0.000445
ENSG00000189058	APOD	299.092228	239.2952	358.8892605	0.585273544	0.129366472	4.52415169	6.06E-06	2.08E-05
ENSG00000100228	RAB36	175.9910665	140.60214	211.379991	0.585112031	0.184876098	3.16488738	0.001551	0.00397
ENSG00000223959	AFG3L1P	162.8941452	130.41222	195.3760718	0.584916405	0.174483514	3.35227319	0.000802	0.002141
ENSG00000135924	DNAJB2	1517.607855	1214.3926	1820.823148	0.584831237	0.06743346	8.67271586	4.22E-18	3.04E-17
ENSG00000125952	MAX	2486.745347	1989.5774	2983.913318	0.584326776	0.058158268	10.0471833	9.45E-24	8.43E-23
ENSG00000121931	LRIF1	824.0862853	659.61365	988.5589216	0.584018132	0.086766612	6.73090853	1.69E-11	8.87E-11
ENSG00000168575	SLC20A2	1037.911247	830.66335	1245.15915	0.584004117	0.075097646	7.77659684	7.45E-15	4.65E-14
ENSG00000158710	TAGLN2	25250.59193	20207.825	30293.35727	0.583974835	0.048605334	12.0146245	2.98E-33	3.47E-32
ENSG00000076685	NT5C2	2089.315663	1672.9361	2505.695214	0.583069487	0.061936128	9.4140448	4.77E-21	3.87E-20
ENSG00000106397	PLOD3	8381.463353	6711.1796	10051.74706	0.582533645	0.047869709	12.1691494	4.54E-34	5.36E-33
ENSG00000152939	MARVELD2	297.0463193	238.0926	356.0000346	0.582403404	0.153806098	3.78660803	0.000153	0.000448
ENSG00000109586	GALNT7	5992.483961	4799.8851	7185.082793	0.58228335	0.050090175	11.6247019	3.09E-31	3.40E-30
ENSG00000197321	SVIL	8445.739071	6763.8126	10127.66556	0.582225712	0.040821866	14.2625942	3.74E-46	5.85E-45
ENSG00000181649	PHLDA2	1018.369057	815.14037	1221.597741	0.582131608	0.10055744	5.78904565	7.08E-09	3.10E-08
ENSG00000116717	GADD45A	3205.375123	2567.0907	3843.659578	0.582114005	0.056089267	10.3783493	3.11E-25	2.92E-24
ENSG00000269858	EGLN2	126.8585712	101.58667	152.1304729	0.581846181	0.209363213	2.77912329	0.005451	0.012785
ENSG00000171302	CANT1	4620.213805	3700.4435	5539.984082	0.581769842	0.052778442	11.0228688	2.96E-28	3.04E-27
ENSG00000078804	TP53INP2	2556.86753	2048.3586	3065.376425	0.581314533	0.056896032	10.2171366	1.66E-24	1.51E-23
ENSG00000138834	MAPK8IP3	751.0903916	602.10999	900.0707933	0.581276545	0.124602022	4.66506512	3.09E-06	1.09E-05
ENSG00000254901	BORCS8	204.9027181	164.13003	245.6754014	0.58124362	0.15524302	3.74408859	0.000181	0.000525
ENSG00000105136	ZNF419	184.6099211	148.04193	221.1779171	0.580959301	0.181381309	3.20297226	0.00136	0.003515
ENSG00000182871	COL18A1	57195.62193	45834.651	68556.59317	0.580874715	0.045718444	12.7054788	5.51E-37	7.07E-36
ENSG00000099999	RNF215	721.646885	578.44401	864.8497558	0.579963585	0.085827711	6.75729994	1.41E-11	7.43E-11
ENSG00000163683	SMIM14	1488.27021	1193.4871	1783.053289	0.579053115	0.063560081	9.11032687	8.21E-20	6.38E-19
ENSG00000137824	RMDN3	1796.607114	1440.5557	2152.658563	0.578706268	0.066451986	8.70863768	3.08E-18	2.23E-17
ENSG00000136051	WASHC4	5124.339355	4111.1238	6137.554922	0.578385966	0.051690524	11.1894002	4.60E-29	4.81E-28
ENSG00000176171	BNIP3	2292.173948	1838.8253	2745.522546	0.578305992	0.061367698	9.42362213	4.36E-21	3.54E-20
ENSG00000138032	PPM1B	273.5979031	219.45358	327.7422246	0.578203059	0.13804899	4.18839035	2.81E-05	9.00E-05
ENSG00000112304	ACOT13	540.7545302	433.93723	647.5718298	0.577750221	0.100943914	5.72347752	1.04E-08	4.51E-08
ENSG00000260404	AC110079.1	105.6602203	84.852289	126.4681521	0.577309657	0.24113318	2.39415271	0.016659	0.035717
ENSG00000118680	MYL12B	17951.57005	14407.377	21495.76286	0.577219799	0.036489344	15.8188592	2.31E-56	4.25E-55
ENSG00000140937	CDH11	1599.426002	1283.9834	1914.868648	0.57609521	0.067530909	8.53083753	1.45E-17	1.03E-16
ENSG00000197860	SGTB	845.0756371	678.59051	1011.56076	0.575667992	0.080567641	7.14515138	8.99E-13	5.06E-12
ENSG00000047410	TPR	9120.110487	7324.7634	10915.4576	0.575590847	0.043265435	13.3037111	2.20E-40	3.04E-39
ENSG00000085117	CD82	3119.037921	2504.9449	3733.130952	0.575465644	0.051326576	11.2118456	3.57E-29	3.75E-28
ENSG00000253710	ALG11	323.1465537	259.78506	386.5080525	0.575350819	0.135045732	4.26041469	2.04E-05	6.63E-05
ENSG00000038002	AGA	524.2210891	421.32161	627.1205674	0.575328144	0.124431974	4.62363592	3.77E-06	1.32E-05
ENSG00000171867	PRNP	6081.079868	4885.1787	7276.981062	0.575104224	0.049194654	11.6903805	1.43E-31	1.59E-30
ENSG00000111199	TRPV4	387.8327689	311.27483	464.3907051	0.575004491	0.133666108	4.30179721	1.69E-05	5.55E-05
ENSG00000198408	OGA	3551.054551	2853.9678	4248.141338	0.574172159	0.051025305	11.2526943	2.25E-29	2.37E-28
ENSG00000214413	BBIP1	476.9152182	383.92772	569.9027183	0.571710191	0.121589583	4.70196687	2.58E-06	9.17E-06
ENSG00000116641	DOCK7	1311.859798	1055.2968	1568.422781	0.571672422	0.0773585	7.38991089	1.47E-13	8.58E-13
ENSG00000092931	MFSD11	873.1938949	702.6508	1043.73699	0.57136706	0.082525693	6.92350518	4.41E-12	2.40E-11
ENSG00000226752	CUTALP	228.7831417	184.29536	273.2709252	0.570267539	0.167190394	3.410887	0.000648	0.001752
ENSG00000267107	PCAT19	927.0611652	746.51801	1107.604325	0.570016706	0.089649173	6.3583041	2.04E-10	9.96E-10
ENSG00000148824	MTG1	129.041945	104.00715	154.0751461	0.569977609	0.209855534	2.71604755	0.006607	0.015256
ENSG00000111652	COPS7A	1589.04927	1279.0566	1899.041947	0.569908202	0.064180797	8.87973087	6.70E-19	5.01E-18
ENSG00000130396	AFDN	5866.398469	4723.3719	7009.425059	0.569772243	0.048234478	11.8125512	3.36E-32	3.82E-31
ENSG00000171206	TRIM8	4768.359255	3838.8147	5697.903785	0.56942854	0.06329693	8.99614785	2.34E-19	1.78E-18
ENSG00000166450	PRTG	184.6271518	148.75689	220.4974179	0.569102694	0.177970486	3.19773635	0.001385	0.003573
ENSG00000082438	COBLL1	1344.955532	1083.0318	1606.879246	0.568930088	0.080271353	7.08758564	1.36E-12	7.62E-12
ENSG00000151835	SACS	7748.128147	6240.0875	9256.168785	0.56876897	0.044893222	12.6693729	8.74E-37	1.11E-35
ENSG00000007168	PAFAH1B1	6607.214634	5321.3162	7893.113087	0.568751929	0.04149089	13.7078748	9.11E-43	1.33E-41
ENSG00000203805	PLPP4	485.6256258	390.90651	580.3447458	0.56871789	0.123171448	4.61728671	3.89E-06	1.36E-05
ENSG00000164889	SLC4A2	5467.973694	4403.4439	6532.503441	0.56864923	0.049508328	11.4859307	1.55E-30	1.69E-29
ENSG00000030110	BAK1	1818.711764	1464.9735	2172.449996	0.567890721	0.066643268	8.52135158	1.58E-17	1.11E-16
ENSG00000119820	YIPF4	1659.205036	1337.5716	1980.838509	0.566477661	0.069721699	8.12484021	4.48E-16	2.97E-15
ENSG00000136783	NIPSNAP3A	1105.320612	891.2857	1319.355529	0.566292634	0.081565506	6.94279559	3.84E-12	2.10E-11
ENSG00000109220	CHIC2	880.8318557	710.05204	1051.611673	0.565994234	0.081613076	6.93509257	4.06E-12	2.22E-11
ENSG00000167110	GOLGA2	4100.623621	3306.5986	4894.648618	0.565889283	0.047128031	12.0074885	3.25E-33	3.78E-32
ENSG00000132718	SYT11	2549.2495	2055.7469	3042.752102	0.565798833	0.058006086	9.75412872	1.77E-22	1.51E-21

ENSG00000188167	TMPPE	96.81096467	78.088433	115.5334968	0.565687453	0.23826983	2.37414637	0.01759	0.037545
ENSG00000165029	ABCA1	1815.516675	1464.2722	2166.761101	0.565468324	0.073741372	7.66826425	1.74E-14	1.07E-13
ENSG00000127838	PNKD	1285.430461	1036.634	1534.226935	0.565351087	0.069607991	8.12192796	4.59E-16	3.04E-15
ENSG00000013441	CLK1	561.2483091	453.00472	669.491897	0.565192921	0.132016	4.28124562	1.86E-05	6.06E-05
ENSG00000152689	RASGRP3	1687.967494	1361.6647	2014.270256	0.565152703	0.074534488	7.58243222	3.39E-14	2.04E-13
ENSG00000149292	TTC12	169.9795977	137.14485	202.8143421	0.564790923	0.176206517	3.20527829	0.001349	0.003492
ENSG00000172939	OXSR1	2199.480122	1774.7416	2624.218664	0.56428806	0.05553445	10.1610453	2.96E-24	2.68E-23
ENSG00000265808	SEC22B	3615.878906	2917.7838	4313.974018	0.564106801	0.047349097	11.9137817	1.00E-32	1.15E-31
ENSG00000104689	TNFRSF10A	1112.13058	897.93808	1326.32308	0.563791922	0.084769943	6.65084701	2.91E-11	1.51E-10
ENSG00000131791	PRKAB2	684.2062277	552.32424	816.0882204	0.56349961	0.097493215	5.77988542	7.48E-09	3.27E-08
ENSG00000180628	PCGF5	2232.910568	1802.466	2663.355101	0.563484954	0.062636783	8.99607106	2.34E-19	1.78E-18
ENSG00000115091	ACTR3	13502.89737	10903.701	16102.09336	0.562440024	0.037016637	15.1942496	3.86E-52	6.70E-51
ENSG00000228649	SNHG26	102.1318227	82.448039	121.8156062	0.562372518	0.228294177	2.46336777	0.013764	0.03002
ENSG00000177058	SLC38A9	568.4899985	459.00948	677.9705125	0.562319107	0.101812258	5.5230983	3.33E-08	1.39E-07
ENSG00000279369	AC046185.3	168.9868653	136.48504	201.4886916	0.561837084	0.170167646	3.30166808	0.000961	0.00254
ENSG00000100883	SRP54	2808.673294	2268.8674	3348.479178	0.561583135	0.056399181	9.95729233	2.34E-23	2.07E-22
ENSG00000138821	SLC39A8	190.681325	154.08057	227.2820775	0.5612296	0.160644214	3.49361852	0.000477	0.001314
ENSG00000135835	KIAA1614	116.3224042	93.948622	138.6961865	0.561191182	0.218954764	2.56304623	0.010376	0.023149
ENSG00000186577	SMIM29	813.3495345	657.01128	969.6877882	0.561093213	0.092343161	6.07617508	1.23E-09	5.70E-09
ENSG00000074695	LMAN1	8984.548508	7260.3988	10708.69822	0.560659051	0.041159064	13.621764	2.97E-42	4.30E-41
ENSG00000239521	CASTOR3	383.4989048	310.07891	456.9188965	0.560253085	0.133203342	4.20599873	2.60E-05	8.35E-05
ENSG00000173083	HPSE	1773.329792	1433.3868	2113.272748	0.559848106	0.065093706	8.60064883	7.93E-18	5.65E-17
ENSG00000104756	KCTD9	1619.175708	1309.0608	1929.290638	0.559651247	0.063880168	8.76095452	1.94E-18	1.42E-17
ENSG00000157110	RBPMS	793.7803021	641.65924	945.9013681	0.559632904	0.08541062	6.55226371	5.67E-11	2.88E-10
ENSG00000244754	N4BP2L2	1248.906667	1010.3061	1487.50728	0.559383337	0.083013735	6.73844319	1.60E-11	8.44E-11
ENSG00000116977	LGALS8	2389.047488	1932.3666	2845.728421	0.559025808	0.067926492	8.22986419	1.87E-16	1.26E-15
ENSG00000153246	PLA2R1	1548.50815	1253.0438	1843.972548	0.558283152	0.070832166	7.88177441	3.23E-15	2.05E-14
ENSG00000104957	CCDC130	494.0893529	399.56668	588.6120253	0.558062822	0.108676307	5.1350919	2.82E-07	1.09E-06
ENSG00000184840	TMED9	10119.03172	8186.0206	12052.04282	0.557985404	0.038737237	14.4043678	4.86E-47	7.70E-46
ENSG00000285410	GABPB1-IT1	107.1005076	86.747982	127.4530328	0.557927623	0.244944685	2.27776987	0.02274	0.047235
ENSG00000213923	CSNK1E	2389.19435	1933.0405	2845.348169	0.557685415	0.055961047	9.96560008	2.16E-23	1.90E-22
ENSG00000064607	SUGP2	1526.2727	1235.7517	1816.793676	0.556899116	0.075111309	7.41431779	1.22E-13	7.16E-13
ENSG00000153130	SCOC	2012.151604	1628.5543	2395.748932	0.556801975	0.060508292	9.20207728	3.51E-20	2.76E-19
ENSG00000005700	IBTK	2468.567666	1998.2213	2938.914068	0.556686939	0.057987801	9.60006985	7.99E-22	6.65E-21
ENSG00000072364	AFF4	7195.323067	5824.4188	8566.227333	0.556564271	0.047130857	11.8089148	3.51E-32	3.98E-31
ENSG00000103253	HAGHL	158.9281329	128.45839	189.3978753	0.556557569	0.209786603	2.65297002	0.007979	0.018189
ENSG00000198380	GFPT1	4101.793067	3320.651	4882.935138	0.556421464	0.048020107	11.5872601	4.78E-31	5.24E-30
ENSG00000184602	SNN	2821.506372	2284.3115	3358.70126	0.556234895	0.058968093	9.43281124	3.99E-21	3.25E-20
ENSG00000100612	DHRS7	1475.627991	1194.6654	1756.590602	0.556164515	0.06702416	8.29797072	1.06E-16	7.23E-16
ENSG00000150991	UBC	39064.80946	31632.516	46497.10328	0.555698126	0.034792697	15.9716887	2.01E-57	3.77E-56
ENSG00000144747	TMF1	2895.601477	2345.3629	3445.840039	0.55560988	0.057271757	9.70128929	2.98E-22	2.52E-21
ENSG00000105173	CCNE1	263.8060578	213.48628	314.1258347	0.555433034	0.157445203	3.52778633	0.000419	0.001163
ENSG00000237440	AC008554.1	146.1305466	118.50103	173.7600667	0.55443644	0.227470881	2.43739523	0.014794	0.032043
ENSG00000124762	CDKN1A	14936.63597	12101.521	17771.75055	0.554273294	0.041812445	13.25618	4.16E-40	5.72E-39
ENSG00000175155	YPEL2	5035.979431	4081.0783	5990.880552	0.554247554	0.063944603	8.66762049	4.41E-18	3.18E-17
ENSG00000124788	ATXN1	1343.690294	1089.21	1598.170546	0.553133781	0.067914989	8.1445023	3.81E-16	2.53E-15
ENSG00000151292	CSNK1G3	1244.429869	1009.1761	1479.683635	0.552925916	0.074910474	7.38115631	1.57E-13	9.15E-13
ENSG00000102595	UGGT2	1490.009503	1208.7383	1771.280705	0.552430839	0.084718808	6.5207579	7.00E-11	3.53E-10
ENSG00000102780	DGKH	1903.831236	1544.3892	2263.273312	0.551845658	0.0682476	8.08593505	6.17E-16	4.06E-15
ENSG00000132950	ZMYM5	443.2715424	359.46939	527.0736965	0.551575108	0.109667468	5.0295235	4.92E-07	1.87E-06
ENSG00000204619	PPP1R11	1555.158455	1261.882	1848.434934	0.550277837	0.06964784	7.9008601	2.77E-15	1.76E-14
ENSG00000135535	CD164	9153.337325	7428.9152	10877.75941	0.550177753	0.048536624	11.3353115	8.77E-30	9.37E-29
ENSG00000075945	KIFAP3	1323.965647	1074.6703	1573.260958	0.550048267	0.067052727	8.20321992	2.34E-16	1.57E-15
ENSG00000143367	TUFT1	293.6494428	238.56846	348.7304282	0.549706301	0.13763891	3.99382923	6.50E-05	0.000199
ENSG00000166888	STAT6	7486.781517	6077.6227	8895.940293	0.549483346	0.043583345	12.6076452	1.92E-36	2.42E-35
ENSG00000101194	SLC17A9	665.0671049	539.99416	790.1400504	0.549371585	0.098660492	5.56830371	2.57E-08	1.08E-07
ENSG00000128595	CALU	28984.11873	23532.162	34436.07549	0.549289816	0.033872186	16.2165447	3.85E-59	7.46E-58
ENSG00000137103	TMEM8B	136.8709506	110.92807	162.8138295	0.549246836	0.226862835	2.42105251	0.015476	0.03337
ENSG00000162086	ZNF75A	312.6029971	253.84163	371.3643639	0.549070816	0.132211887	4.15296103	3.28E-05	0.000105
ENSG00000185624	P4HB	68897.4452	55949.759	81845.13142	0.548757691	0.033362993	16.4480955	8.65E-61	1.71E-59
ENSG00000101160	CTSZ	16208.87819	13164.308	19253.44822	0.548450429	0.042515804	12.8999191	4.51E-38	5.90E-37
ENSG00000013297	CLDN11	333.7256542	271.13724	396.3140728	0.548057976	0.123581936	4.43477416	9.22E-06	3.10E-05
ENSG00000170871	KIAA0232	1933.752113	1571.3185	2296.185741	0.547221331	0.061118986	8.95337705	3.45E-19	2.61E-18
ENSG00000092439	TRPM7	1679.15205	1364.897	1993.407143	0.547089972	0.067652527	8.08676335	6.13E-16	4.03E-15
ENSG00000140992	PDPK1	1914.402609	1555.4054	2273.399836	0.547004416	0.062388965	8.76764692	1.82E-18	1.34E-17
ENSG00000057252	SOAT1	2178.759207	1770.6303	2586.888105	0.546832281	0.059904192	9.12844766	6.95E-20	5.41E-19
ENSG00000128394	APOBEC3F	311.4565755	252.93022	369.9829296	0.546381504	0.141961363	3.8488043	0.000119	0.000353
ENSG00000130150	MOSPD2	741.9280032	603.46375	880.3922585	0.546206484	0.092281382	5.91892395	3.24E-09	1.46E-08
ENSG00000184867	ARMCX2	2370.445613	1927.0854	2813.80578	0.545768502	0.054899266	9.94127129	2.75E-23	2.42E-22
ENSG00000161638	ITGA5	150751.3426	122575.39	178927.2914	0.545692643	0.0387424	14.0851533	4.69E-45	7.16E-44
ENSG00000103335	PIEZO1	27883.11744	22671.327	33094.90832	0.545639212	0.044566572	12.2432395	1.83E-34	2.19E-33
ENSG00000124224	PPP4R1L	254.3559688	206.91359	301.7983501	0.545568319	0.141150848	3.86514375	0.000111	0.000332
ENSG00000131966	ACTR10	1847.614552	1502.8905	2192.338637	0.545001202	0.064881937	8.399891	4.47E-17	3.10E-16
ENSG00000101966	XIAP	2419.610745	1968.2549	2870.966607	0.544800795	0.062108912	8.77170089	1.76E-18	1.30E-17
ENSG00000155970	MICU3	129.2157405	105.03472	153.396757	0.544716646	0.225119475	2.41967803	0.015534	0.033493
ENSG00000154222	CC2D1B	1679.374063	1366.0069	1992.741266	0.544600239	0.062475414	8.71703292	2.86E-18	2.08E-17
ENSG00000177706	FAM20C	1046.182189	850.68817	1241.676205	0.544467955	0.088031623	6.18491325	6.21E-10	2.94E-09
ENSG00000091844	RGS17	124.5572378	101.28323	147.8312451	0.544161058	0.237424963	2.29192858	0.02191	0.045697
ENSG00000068366	ACSL4	5682.236885	4623.6657	6740.808085	0.543837652	0.05520059	9.85202609	6.72E-23	5.81E-22
ENSG00000181690	PLAG1	226.9066929	184.63258	269.1808021	0.543562298	0.159185616	3.41464456	0.000639	0.001729
ENSG00000100258	LMF2	3249.926772	2644.6095	3855.244028	0.543307877	0.054175672	10.028632	1.14E-23	1.01E-22
ENSG00000182827	ACBD3	2476.476714	2016.902	2936.051458	0.541835138	0.054698126	9.90591768	3.92E-23	3.43E-22
ENSG00000169231	THBS3	507.815309	413.5153	602.1153189	0.541644685	0.101974518	5.31156898	1.09E-07	4.35E-07
ENSG00000141580	WDR45B	3045.90058	2481.4977	3610.303473	0.541029	0.050849094	10.6398946	1.94E-26	1.90E-25
ENSG00000160336	ZNF761	336.8191886	274.35152	399.2868527	0.540646159	0.123318791	4.38413443	1.16E-05	3.88E-05

ENSG00000174437	ATP2A2	15319.63646	12482.116	18157.15672	0.540616956	0.038966562	13.8738686	9.12E-44	1.36E-42
ENSG00000122741	DCAF10	1452.903388	1183.7655	1722.041239	0.540527501	0.066052946	8.18324591	2.76E-16	1.85E-15
ENSG00000178343	SHISA3	714.4440699	582.00842	846.8797153	0.539792991	0.104107871	5.18493927	2.16E-07	8.44E-07
ENSG00000099246	RAB18	2830.398892	2308.1056	3352.692161	0.539091622	0.055586021	9.69833088	3.06E-22	2.59E-21
ENSG00000186020	ZNF529	212.5633551	173.20182	251.9248919	0.538916316	0.162302208	3.32044969	0.000899	0.002384
ENSG00000157404	KIT	1076.517616	878.18396	1274.85127	0.538778193	0.090988046	5.9214173	3.19E-09	1.44E-08
ENSG00000144233	AMMECR1L	1587.820486	1295.118	1880.522987	0.538569358	0.070761943	7.61100297	2.72E-14	1.65E-13
ENSG00000118873	RAB3GAP2	2292.503614	1869.7912	2715.216027	0.538460605	0.055817247	9.64684991	5.07E-22	4.25E-21
ENSG00000229180	AC006001.3	325.2595724	265.19243	385.3267197	0.53803247	0.132627872	4.05670741	4.98E-05	0.000155
ENSG00000149948	HMGA2	2869.118404	2340.2086	3398.028186	0.537911482	0.057136133	9.41455878	4.75E-21	3.85E-20
ENSG00000129038	LOXL1	133.1257581	108.5423	157.7092177	0.537866796	0.200207053	2.68655269	0.007219	0.016573
ENSG00000156239	N6AMT1	219.9668908	179.45331	260.4804679	0.537626698	0.151265127	3.5542012	0.000379	0.001059
ENSG00000112343	TRIM38	2314.000783	1888.3817	2739.619885	0.537554169	0.064915348	8.28084865	1.22E-16	8.33E-16
ENSG00000134698	AGO4	867.511405	707.9697	1027.053106	0.537379649	0.086464848	6.21500716	5.13E-10	2.44E-09
ENSG00000151748	SAV1	1484.538885	1211.2312	1757.846589	0.537347269	0.065081425	8.25653819	1.50E-16	1.02E-15
ENSG00000214455	RCN1P2	201.7380494	164.56346	238.9126408	0.537042777	0.16618257	3.23164322	0.001231	0.003209
ENSG00000152804	HHEX	2908.596955	2373.7077	3443.486226	0.536692438	0.052159997	10.2893494	7.87E-25	7.26E-24
ENSG00000132510	KDM6B	1426.618532	1163.8984	1689.338638	0.5363669	0.086394706	6.20833061	5.36E-10	2.54E-09
ENSG00000167081	PBX3	838.415434	684.29589	992.5349736	0.536360885	0.081005796	6.62126556	3.56E-11	1.83E-10
ENSG00000077713	SLC25A43	839.3301173	685.11149	993.5487496	0.536319309	0.082367051	6.51133313	7.45E-11	3.76E-10
ENSG00000174516	PELI3	240.7416749	196.48933	284.9940175	0.536002975	0.151662969	3.53417172	0.000409	0.001137
ENSG00000162819	BROX	1806.862997	1475.1201	2138.605929	0.535238491	0.073049811	7.32703458	2.35E-13	1.36E-12
ENSG00000104133	SPG11	1659.043799	1355.3465	1962.741114	0.534763097	0.074209375	7.20613936	5.76E-13	3.27E-12
ENSG00000013561	RNF14	2009.505791	1641.3958	2377.615797	0.534572829	0.061323218	8.717299	2.85E-18	2.07E-17
ENSG00000162889	MAPKAPK2	4363.518654	3563.5591	5163.478186	0.53453054	0.057444844	9.30510902	1.34E-20	1.07E-19
ENSG00000129472	RAB2B	908.9049244	742.64399	1075.165861	0.534289257	0.079446118	6.72517766	1.75E-11	9.22E-11
ENSG00000138138	ATAD1	1467.293462	1198.4692	1736.117732	0.534266614	0.065356103	8.17470119	2.97E-16	1.98E-15
ENSG00000138356	AOX1	518.7519087	423.56099	613.942832	0.534239329	0.110082053	4.85310107	1.22E-06	4.47E-06
ENSG00000026297	RNASET2	321.4278744	262.55521	380.3005409	0.53415218	0.139035409	3.84184275	0.000122	0.000363
ENSG00000144840	RABL3	877.8912169	717.11027	1038.672162	0.533928016	0.080025803	6.67194822	2.52E-11	1.32E-10
ENSG00000231074	HCG18	582.4183601	475.93131	688.905412	0.533924196	0.096501728	5.53279416	3.15E-08	1.32E-07
ENSG00000179241	LDLRAD3	1757.158316	1435.6059	2078.710687	0.533800896	0.067524211	7.90532592	2.67E-15	1.70E-14
ENSG00000100949	RABGGTA	836.7962939	683.58335	990.009242	0.533497031	0.083509362	6.38846973	1.68E-10	8.25E-10
ENSG00000132475	H3F3B	6639.704235	5426.5906	7852.817899	0.533259799	0.044468019	11.9919847	3.91E-33	4.55E-32
ENSG00000106266	SNX8	1892.826171	1547.184	2238.468333	0.532777538	0.060636158	8.78646597	1.54E-18	1.14E-17
ENSG00000223960	PRKRA-AS1	215.922189	176.35597	255.4884086	0.532683788	0.160635685	3.3160987	0.000913	0.002419
ENSG00000264247	LINC00909	117.0040467	95.619046	138.3890478	0.532500318	0.214551506	2.481923	0.013068	0.028651
ENSG00000151229	SLC2A13	306.8997845	251.00957	362.790002	0.531947407	0.139886728	3.80270105	0.000143	0.000421
ENSG00000198814	GK	310.4185471	253.76411	367.0729861	0.531854312	0.128762729	4.13049893	3.62E-05	0.000115
ENSG00000169032	MAP2K1	3214.284392	2628.4549	3800.113903	0.531653746	0.057659209	9.22062154	2.95E-20	2.33E-19
ENSG00000143751	SDE2	744.7036529	608.98352	880.4237898	0.531630135	0.086669259	6.13401036	8.57E-10	4.01E-09
ENSG00000111843	TMEM14C	2433.629876	1990.7027	2876.557028	0.531269059	0.053577271	9.91594094	3.55E-23	3.11E-22
ENSG00000187231	SESTD1	1781.860281	1457.6927	2106.027856	0.531221306	0.066585479	7.97803532	1.49E-15	9.61E-15
ENSG00000204209	DAXX	2346.854827	1919.2211	2774.488589	0.531085896	0.058604965	9.06213152	1.28E-19	9.85E-19
ENSG00000106105	GARS	9400.657172	7689.6267	11111.68767	0.530995477	0.041050575	12.9351532	2.85E-38	3.75E-37
ENSG00000179833	SERTAD2	998.2162399	816.73627	1179.696211	0.530642456	0.078762405	6.73725565	1.61E-11	8.51E-11
ENSG00000164305	CASP3	3288.309193	2690.136	3886.48241	0.530443022	0.054040715	9.81561819	9.64E-23	8.31E-22
ENSG00000152409	JMY	596.990062	488.51798	705.4621414	0.530027658	0.101379899	5.22813359	1.71E-07	6.73E-07
ENSG00000169446	MMGT1	934.9503553	765.1837	1104.717013	0.529380812	0.090692523	5.83709435	5.31E-09	2.35E-08
ENSG00000167994	RAB3IL1	229.0773191	187.43343	270.7212126	0.529264124	0.154126255	3.43396473	0.000595	0.00162
ENSG00000170759	KIF5B	11774.16258	9639.9691	13908.3561	0.529001782	0.042229651	12.5267856	5.33E-36	6.65E-35
ENSG00000160055	TMEM234	325.1062186	266.28356	383.9288727	0.528616929	0.130954599	4.03664273	5.42E-05	0.000168
ENSG00000211455	STK38L	1838.630112	1506.1575	2171.102756	0.528003486	0.066234528	7.97172572	1.56E-15	1.01E-14
ENSG00000107175	CREB3	2252.851683	1844.8772	2660.826181	0.527627635	0.072562401	7.27136402	3.56E-13	2.04E-12
ENSG00000174574	AKIRIN1	2471.026339	2024.4358	2917.616878	0.527334893	0.054586914	9.66046351	4.44E-22	3.73E-21
ENSG00000135245	HILPDA	183.197713	150.17569	216.2197332	0.527173825	0.175658846	3.00112313	0.00269	0.006637
ENSG00000179832	MROH1	2571.074466	2106.7603	3035.388672	0.526964286	0.054237074	9.71594236	2.58E-22	2.18E-21
ENSG00000273311	DGCR11	124.5452406	102.01103	147.0794501	0.525569067	0.224006044	2.34622718	0.018965	0.040174
ENSG00000168209	DDIT4	4340.714077	3558.5319	5122.896274	0.525557898	0.05011811	10.4863869	9.98E-26	9.49E-25
ENSG00000178719	GRINA	7476.5363	6129.2525	8823.820123	0.525423852	0.05659461	9.28399104	1.63E-20	1.30E-19
ENSG00000176148	TCP11L1	671.8748071	550.84821	792.9014088	0.525342011	0.092327675	5.68997333	1.27E-08	5.46E-08
ENSG00000109089	CDR2L	1861.694641	1526.4209	2196.968414	0.524744004	0.06934953	7.56665553	3.83E-14	2.30E-13
ENSG00000006607	FARP2	1471.5328	1206.9234	1736.142181	0.5244585	0.067196208	7.80488239	5.96E-15	3.73E-14
ENSG00000196204	RNF216P1	448.5466548	367.78938	529.3039287	0.524047489	0.117776175	4.44952035	8.61E-06	2.90E-05
ENSG00000095951	HIVEP1	2128.984074	1746.7875	2511.180662	0.524024457	0.062463849	8.38924384	4.89E-17	3.38E-16
ENSG00000054282	SDCCAG8	1227.889029	1007.6	1448.17801	0.523777448	0.071992528	7.27544177	3.45E-13	1.99E-12
ENSG00000122482	ZNF644	1730.952351	1420.8293	2041.07542	0.522826629	0.070759941	7.38873752	1.48E-13	8.65E-13
ENSG00000122971	ACADS	560.205985	459.58284	660.8291336	0.522764907	0.101594814	5.14558652	2.67E-07	1.04E-06
ENSG00000003056	M6PR	3763.727011	3089.6911	4437.76292	0.522581148	0.05069686	10.3079588	6.49E-25	5.99E-24
ENSG00000135931	ARMC9	1324.802833	1087.5705	1562.035126	0.522504073	0.069172865	7.55359883	4.23E-14	2.54E-13
ENSG00000136986	DERL1	2354.537649	1933.1037	2775.971556	0.522299456	0.055909504	9.34187249	9.46E-21	7.60E-20
ENSG00000038210	PI4K2B	578.1352586	474.56018	681.7103366	0.522204268	0.112924038	4.62438536	3.76E-06	1.31E-05
ENSG00000204070	SYS1	951.1998275	780.75523	1121.644429	0.522118246	0.084652938	6.16775103	6.93E-10	3.26E-09
ENSG00000079308	TNS1	3592.496152	2949.3763	4235.615966	0.522057439	0.055537738	9.40004862	5.45E-21	4.42E-20
ENSG00000081014	AP4E1	691.2300772	567.6018	814.8583528	0.521977493	0.093694452	5.57106087	2.53E-08	1.07E-07
ENSG00000100242	SUN2	5981.118823	4912.2976	7049.939997	0.521094347	0.041556522	12.539412	4.54E-36	5.68E-35
ENSG00000119720	NRDE2	521.1804516	428.072	614.2889013	0.520733264	0.109781011	4.74338193	2.10E-06	7.53E-06
ENSG00000174010	KLHL15	202.1859061	165.90583	238.4659776	0.520729877	0.168720885	3.08633917	0.002026	0.005092
ENSG00000023318	ERP44	3333.837023	2739.4379	3928.236161	0.520129184	0.049666673	10.4723983	1.16E-25	1.10E-24
ENSG00000127990	SGCE	2272.680215	1868.0409	2677.319501	0.51979872	0.058716952	8.85261753	8.55E-19	6.37E-18
ENSG00000146373	RNF217	881.3271715	724.66443	1037.989909	0.519603232	0.091921669	5.65267403	1.58E-08	6.75E-08
ENSG00000188021	UBQLN2	1218.39947	1001.4626	1435.336308	0.519060025	0.077668317	6.68303426	2.34E-11	1.22E-10
ENSG00000268043	NBPF12	262.8366562	216.20016	309.4731479	0.519046005	0.150262271	3.45426701	0.000552	0.001508
ENSG00000124615	MOCS1	576.4288017	473.75383	679.1037717	0.518919895	0.102878006	5.04403144	4.56E-07	1.73E-06
ENSG00000170085	SIMC1	412.0908298	338.77406	485.4075974	0.518680924	0.115396544	4.49476998	6.96E-06	2.37E-05

ENSG00000112658	SRF	1880.607242	1545.6543	2215.560151	0.518672269	0.069169426	7.49857698	6.45E-14	3.82E-13
ENSG00000154803	FLCN	1040.824483	855.83859	1225.810376	0.518580288	0.077165762	6.72034167	1.81E-11	9.52E-11
ENSG00000223509	AC135983.2	119.6927807	98.506027	140.8795349	0.517837708	0.215141281	2.40696581	0.016086	0.034579
ENSG00000009830	POMT2	1022.266352	840.42915	1204.103558	0.517829454	0.080532805	6.4300437	1.28E-10	6.33E-10
ENSG00000182087	TMEM259	4788.623027	3938.3035	5638.942591	0.517695328	0.047758905	10.8397654	2.23E-27	2.24E-26
ENSG00000099290	WASHC2A	3038.314877	2499.3171	3577.312678	0.517646741	0.051698501	10.0127997	1.34E-23	1.19E-22
ENSG00000099917	MED15	4350.005488	3577.1707	5122.840249	0.517624869	0.054396182	9.51583093	1.80E-21	1.48E-20
ENSG00000173960	UBXN2A	1155.37045	950.24979	1360.491111	0.517534537	0.072693777	7.11937882	1.08E-12	6.08E-12
ENSG00000148572	NRBF2	950.5297424	782.12056	1118.93892	0.516870277	0.076550787	6.75199171	1.46E-11	7.70E-11
ENSG00000134107	BHLHE40	2158.085032	1775.4456	2540.724497	0.51680275	0.071184408	7.26005547	3.87E-13	2.21E-12
ENSG00000166946	CCNDBP1	1533.823953	1262.0971	1805.550757	0.516390461	0.063246331	8.16474969	3.22E-16	2.15E-15
ENSG00000174227	PIGG	2000.831826	1647.2035	2354.460171	0.515616908	0.065115603	7.91848479	2.40E-15	1.53E-14
ENSG00000204310	AGPAT1	1856.372078	1527.668	2185.076133	0.515508911	0.071007275	7.25994504	3.87E-13	2.21E-12
ENSG00000187824	TMEM220	165.243151	136.15853	194.3277734	0.515359917	0.196414362	2.62384029	0.008694	0.019676
ENSG00000126773	PCNX4	2119.001608	1744.8125	2493.190681	0.515289261	0.062527034	8.24106349	1.71E-16	1.15E-15
ENSG00000165097	KDM1B	553.9489036	455.9196	651.9782075	0.514823321	0.108314744	4.75303085	2.00E-06	7.20E-06
ENSG00000108510	MED13	3245.550474	2672.44	3818.660969	0.514748988	0.056157698	9.16613411	4.90E-20	3.84E-19
ENSG00000130821	SLC6A8	2429.466678	2000.2022	2858.731183	0.514639766	0.05945526	8.65591649	4.89E-18	3.51E-17
ENSG00000182952	HMGN4	1410.194203	1161.6795	1658.708885	0.513831404	0.069193823	7.42597215	1.12E-13	6.57E-13
ENSG00000122008	POLK	1086.386747	894.89181	1277.881682	0.513743075	0.084437593	6.08429318	1.17E-09	5.42E-09
ENSG00000185475	TMEM179B	910.0041539	749.47589	1070.532419	0.513698933	0.081451089	6.30683937	2.85E-10	1.38E-09
ENSG00000136925	TSTD2	599.8338605	494.22006	705.4476603	0.513672881	0.106063088	4.84308812	1.28E-06	4.69E-06
ENSG00000111224	PARP11	308.7954143	254.47099	363.1198369	0.513656984	0.131639062	3.90201035	9.54E-05	0.000287
ENSG00000105085	MED26	179.8543408	148.07037	211.6383141	0.513576279	0.167652433	3.06333926	0.002189	0.005474
ENSG00000138758	11-Sep	11910.52716	9813.243	14007.81128	0.513464811	0.043567334	11.7855458	4.63E-32	5.24E-31
ENSG00000115993	TRAK2	3941.526918	3247.7517	4635.30212	0.513296271	0.050237159	10.2174623	1.66E-24	1.51E-23
ENSG00000033327	GAB2	3270.66118	2695.035	3846.287368	0.513065528	0.051467472	9.96873388	2.09E-23	1.85E-22
ENSG00000136383	ALPK3	3435.833507	2832.2202	4039.446788	0.512676389	0.065275273	7.8540673	4.03E-15	2.54E-14
ENSG00000167555	ZNF528	419.8968746	346.13181	493.6619436	0.51257929	0.110085712	4.65618371	3.22E-06	1.13E-05
ENSG00000165115	KIF27	148.8230887	122.65563	174.9905438	0.512502252	0.196619559	2.606568	0.009145	0.020622
ENSG00000180611	MB21D2	337.134363	277.79155	396.4771754	0.5124498	0.128309742	3.99384952	6.50E-05	0.000199
ENSG00000090581	GNPTG	2230.140875	1838.3394	2621.942365	0.512433329	0.058583079	8.74712182	2.19E-18	1.60E-17
ENSG00000059378	PARP12	2721.191642	2242.8717	3199.511543	0.512339174	0.05301391	9.6642404	4.28E-22	3.60E-21
ENSG00000162971	TYW5	321.0097956	264.60054	377.4190522	0.512022154	0.13150968	3.89341798	9.88E-05	0.000297
ENSG00000152061	RABGAP1L	765.9214153	631.41592	900.4269062	0.511914088	0.089118825	5.74417455	9.24E-09	4.01E-08
ENSG00000146067	FAM193B	515.8360355	425.36084	606.3112321	0.511798653	0.104925501	4.87773373	1.07E-06	3.95E-06
ENSG00000135503	ACVR1B	1228.616155	1013.0613	1444.17101	0.511657133	0.069305126	7.38267377	1.55E-13	9.05E-13
ENSG00000176261	ZBTB8OS	468.823572	386.61188	551.0352614	0.51155471	0.107449131	4.76090134	1.93E-06	6.93E-06
ENSG00000185808	PIGP	296.7407311	244.63948	348.8419863	0.511523003	0.135772848	3.76749115	0.000165	0.000481
ENSG00000113384	GOLPH3	2697.246435	2224.4544	3170.038422	0.510779298	0.054814016	9.31840678	1.18E-20	9.44E-20
ENSG00000198467	TPM2	6344.345523	5232.8547	7455.836309	0.51051622	0.04993984	10.2226243	1.57E-24	1.43E-23
ENSG00000138767	CNOT6L	2234.966215	1843.753	2626.179449	0.510478169	0.066426041	7.68491036	1.53E-14	9.42E-14
ENSG00000152348	ATG10	161.6665675	133.44349	189.8896399	0.510428705	0.179722638	2.84009132	0.00451	0.010722
ENSG00000158747	NBL1	2369.990508	1955.6083	2784.372714	0.509852445	0.060768088	8.39013476	4.86E-17	3.36E-16
ENSG00000177283	FZD8	1059.544842	874.17689	1244.912795	0.50974965	0.084971827	5.9990431	1.98E-09	9.06E-09
ENSG00000159256	MORC3	1107.887918	914.56482	1301.211018	0.509673073	0.088987093	5.72749438	1.02E-08	4.41E-08
ENSG00000169905	TOR1AIP2	3812.787419	3146.4721	4479.102721	0.509596355	0.048368352	10.5357394	5.91E-26	5.66E-25
ENSG00000153071	DAB2	16243.8738	13404.765	19082.98269	0.509547758	0.041963084	12.1427623	6.27E-34	7.38E-33
ENSG00000168591	TMUB2	1384.684192	1142.4965	1626.871868	0.509364435	0.067450313	7.55169864	4.30E-14	2.57E-13
ENSG00000163877	SNIP1	778.4090423	642.48586	914.3322218	0.509333207	0.084314464	6.04087584	1.53E-09	7.05E-09
ENSG00000244045	TMEM199	407.7003331	336.59172	478.8089433	0.509071334	0.124464328	4.09009829	4.31E-05	0.000135
ENSG00000099331	MYO9B	8659.799904	7148.0352	10171.56459	0.508627032	0.050538335	10.0641826	7.95E-24	7.11E-23
ENSG00000163527	STT3B	8376.384993	6915.5767	9837.193297	0.508491931	0.040056503	12.6943664	6.35E-37	8.13E-36
ENSG00000164117	FBXO8	407.371203	336.17848	478.5639283	0.508413128	0.116291608	4.37188147	1.23E-05	4.09E-05
ENSG00000109787	KLF3	2430.573794	2006.846	2854.301621	0.508376343	0.062380496	8.14960405	3.65E-16	2.43E-15
ENSG00000185485	SDHAP1	163.5933527	135.19678	191.9899227	0.508332693	0.206446058	2.46230273	0.013805	0.030096
ENSG00000116871	MAP7D1	10140.14376	8372.4435	11907.84403	0.508031209	0.051177136	9.92691752	3.18E-23	2.78E-22
ENSG00000110002	VWA5A	569.1290703	469.84329	668.4148537	0.508009626	0.101955147	4.9826776	6.27E-07	2.36E-06
ENSG00000154813	DPH3	1580.092786	1304.8983	1855.287307	0.507888904	0.066021304	7.69280334	1.44E-14	8.87E-14
ENSG00000169026	SLC49A3	137.5560612	113.46313	161.648993	0.507674914	0.199980024	2.53862813	0.011129	0.024692
ENSG00000159461	AMFR	4203.44962	3471.1896	4935.709593	0.507613004	0.053013386	9.57518552	1.02E-21	8.43E-21
ENSG00000115275	MOGS	3868.146075	3195.6942	4540.597993	0.50665258	0.047646578	10.6335565	2.08E-26	2.03E-25
ENSG00000072609	CHFR	1831.235664	1512.9548	2149.516519	0.506581631	0.066225551	7.64933813	2.02E-14	1.23E-13
ENSG00000184497	TMEM255B	2077.490706	1716.5448	2438.436601	0.506517902	0.062020761	8.16690883	3.16E-16	2.11E-15
ENSG00000128524	ATP6V1F	3402.102055	2811.2141	3992.990052	0.505877771	0.060207333	8.40226179	4.38E-17	3.04E-16
ENSG00000129467	ADCY4	1864.958888	1541.9944	2187.923341	0.505453123	0.086073244	5.87236053	4.30E-09	1.91E-08
ENSG00000136888	ATP6V1G1	2641.992786	2184.1579	3099.827715	0.505374887	0.055704825	9.07237185	1.16E-19	9.00E-19
ENSG00000166446	CDYL2	2851.067942	2356.8231	3345.312817	0.505089395	0.05908206	8.54894697	1.24E-17	8.80E-17
ENSG00000143641	GALNT2	11998.25945	9920.8656	14075.65326	0.504479138	0.051843094	9.73088413	2.23E-22	1.89E-21
ENSG00000159899	NPR2	958.1058953	792.31513	1123.896664	0.504307724	0.083851719	6.01428012	1.81E-09	8.27E-09
ENSG00000135164	DMTF1	593.7706787	491.38561	696.1557451	0.504106774	0.125084234	4.03013838	5.57E-05	0.000173
ENSG00000175426	PCSK1	271.0894197	224.22905	317.9497847	0.503937206	0.137863207	3.65534225	0.000257	0.000734
ENSG00000197808	ZNF461	151.0960782	125.04985	177.1423054	0.503645523	0.198882008	2.53238354	0.011329	0.025097
ENSG00000184831	APOO	250.9529106	207.62189	294.2839353	0.503576366	0.149417337	3.3702673	0.000751	0.002015
ENSG00000137947	GTF2B	412.3175283	341.03935	483.595706	0.503339093	0.115338006	4.36403497	1.28E-05	4.23E-05
ENSG00000168005	SPINDOC	1866.170226	1543.663	2188.677416	0.503060002	0.062536708	8.04423538	8.68E-16	5.67E-15
ENSG00000132481	AC087289.1	1638.645601	1355.3754	1921.915843	0.502853198	0.07766019	6.47504468	9.48E-11	4.74E-10
ENSG00000196449	YRDC	472.6081112	390.83421	554.3820113	0.502759795	0.115993193	4.33439052	1.46E-05	4.82E-05
ENSG00000134056	MRPS36	459.1001166	379.99023	538.2099999	0.502506915	0.108729636	4.62161865	3.81E-06	1.33E-05
ENSG00000049759	NEDD4L	1522.280579	1260.4759	1784.085231	0.501671514	0.075017146	6.68742471	2.27E-11	1.19E-10
ENSG00000144566	RAB5A	2568.563674	2126.4735	3010.653892	0.501614441	0.057263436	8.75976846	1.96E-18	1.44E-17
ENSG00000161791	FMNL3	6779.766356	5613.0997	7946.43301	0.501508151	0.048261902	10.3913881	2.71E-25	2.55E-24
ENSG00000107897	ACBD5	1459.071468	1208.5677	1709.575233	0.500851964	0.07746866	6.46522047	1.01E-10	5.05E-10
ENSG00000169604	ANTXR1	4240.278704	3511.6909	4968.866501	0.50083563	0.144227515	3.47253871	0.000516	0.001415
ENSG00000227671	AL390728.4	301.0898667	249.64438	352.5353492	0.500319172	0.168902235	2.96218207	0.003055	0.007473

ENSG00000119523	ALG2	1360.293159	1126.5728	1594.013526	0.500006776	0.069729134	7.17070104	7.46E-13	4.22E-12
ENSG00000149428	HYOU1	16199.09311	18978.847	13419.33971	-0.5000858	0.039697199	-12.597509	2.18E-36	2.74E-35
ENSG00000175592	FOSL1	2089.197088	2447.2457	1731.148463	-0.500314885	0.073995314	-6.7614402	1.37E-11	7.23E-11
ENSG00000141456	PELP1	1523.214266	1784.346	1262.082487	-0.500338217	0.069530038	-7.1960009	6.20E-13	3.52E-12
ENSG00000125445	MRPS7	1231.061381	1442.4255	1019.697283	-0.50043119	0.078934673	-6.3398145	2.30E-10	1.12E-09
ENSG00000051825	MPHOSPH9	506.5821653	593.44205	419.7222837	-0.500516937	0.104712642	-4.7799094	1.75E-06	6.34E-06
ENSG00000007376	RPUSD1	1051.575733	1231.9407	871.2108044	-0.50067346	0.085631293	-5.8468515	5.01E-09	2.22E-08
ENSG00000126216	TUBGCP3	680.1258259	797.13235	563.1192974	-0.50167136	0.102735876	-4.8831176	1.04E-06	3.85E-06
ENSG00000134480	CCNH	672.0568675	787.76163	556.3521004	-0.502232539	0.098741188	-5.086353	3.65E-07	1.40E-06
ENSG00000125871	MGME1	569.8263431	667.91978	471.7329107	-0.50254738	0.104335392	-4.816653	1.46E-06	5.32E-06
ENSG00000136104	RNASEH2B	193.9669551	227.53883	160.3950787	-0.502606838	0.166819363	-3.0128807	0.002588	0.006402
ENSG00000133243	BTBD2	2631.658062	3085.5178	2177.798301	-0.503187145	0.067116241	-7.4972486	6.52E-14	3.86E-13
ENSG00000128578	STRIP2	430.2972352	504.55572	356.0387491	-0.503225389	0.117184407	-4.2943033	1.75E-05	5.73E-05
ENSG00000100982	PCIF1	577.9593429	677.88871	478.0299781	-0.503568816	0.099802699	-5.0456433	4.52E-07	1.72E-06
ENSG00000151502	VPS26B	1937.567957	2272.0245	1603.11138	-0.503615163	0.062530384	-8.0539273	8.02E-16	5.24E-15
ENSG00000139998	RAB15	1063.192773	1246.7551	879.6304103	-0.503737863	0.077223016	-6.523157	6.88E-11	3.48E-10
ENSG00000124126	PREX1	10235.15058	12003.466	8466.835133	-0.503744125	0.042725001	-11.790383	4.38E-32	4.96E-31
ENSG00000106344	RBM28	1102.657903	1293.6727	911.6430776	-0.503833672	0.081545926	-6.1785266	6.47E-10	3.06E-09
ENSG00000275023	MLLT6	2604.795905	3054.9093	2154.682523	-0.503886497	0.057031227	-8.8352736	9.99E-19	7.42E-18
ENSG00000103876	FAH	532.1070285	623.7638	440.4502607	-0.503964719	0.12704047	-3.966962	7.28E-05	0.000222
ENSG00000168116	KIAA1586	395.5649461	464.07727	327.0526189	-0.504131196	0.121349874	-4.1543611	3.26E-05	0.000104
ENSG00000144642	RBMS3	570.9457049	669.73226	472.1591475	-0.504243363	0.108736571	-4.6372932	3.53E-06	1.24E-05
ENSG00000105926	MPP6	601.9857151	705.97291	497.9985166	-0.504279515	0.100663504	-5.0095565	5.46E-07	2.06E-06
ENSG00000116649	SRM	2193.572609	2572.5577	1814.58751	-0.504305449	0.07907226	-6.3777796	1.80E-10	8.82E-10
ENSG00000057935	MTA3	1383.009214	1622.5803	1143.438101	-0.504676131	0.075799169	-6.6580695	2.77E-11	1.44E-10
ENSG00000135390	ATP5MC2	3466.258738	4066.2824	2866.235074	-0.504772524	0.060874684	-8.2919942	1.11E-16	7.59E-16
ENSG00000107815	TWNK	418.4503783	491.13404	345.7667173	-0.504777727	0.13778135	-3.6636143	0.000249	0.000712
ENSG00000065548	ZC3H15	2174.442741	2551.02	1797.865531	-0.504985566	0.058866041	-8.5785549	9.61E-18	6.83E-17
ENSG00000166439	RNF169	1499.839142	1759.901	1239.777242	-0.505098654	0.073950354	-6.8302398	8.48E-12	4.54E-11
ENSG00000173611	SCAI	194.6800867	228.50273	160.8574419	-0.505613796	0.171795351	-2.9431169	0.003249	0.007902
ENSG00000113569	NUP155	1106.514768	1298.6127	914.4168257	-0.505781131	0.072877786	-6.9401275	3.92E-12	2.14E-11
ENSG00000112578	BYSL	784.4017582	920.38163	648.4218843	-0.50591205	0.09329392	-5.4227762	5.87E-08	2.40E-07
ENSG00000125898	FAM110A	216.9117991	254.50501	179.3185917	-0.506090496	0.195906382	-2.5833283	0.009785	0.021933
ENSG00000122026	RPL21	11197.95253	13142.366	9253.539167	-0.506153587	0.04313093	-11.735281	8.40E-32	9.41E-31
ENSG00000171453	POLR1C	478.1669857	561.41965	394.9143222	-0.507318354	0.108638848	-4.6697693	3.02E-06	1.07E-05
ENSG00000164338	UTP15	451.1925163	529.93885	372.4461807	-0.507432081	0.124295586	-4.0824626	4.46E-05	0.00014
ENSG00000108389	MTMR4	1126.533073	1322.7053	930.3608066	-0.507817941	0.074903615	-6.7796186	1.20E-11	6.39E-11
ENSG00000099821	POLRMT	1288.857531	1513.332	1064.383054	-0.508349562	0.072957347	-6.9677638	3.22E-12	1.77E-11
ENSG00000174748	RPL15	23332.1795	27400.823	19263.53555	-0.508408123	0.041114743	-12.365592	4.01E-35	4.90E-34
ENSG00000271254	AC240274.1	295.5490568	347.15352	243.944597	-0.508984246	0.145194071	-3.5055443	0.000456	0.001259
ENSG00000142892	PIGK	1175.166676	1380.5142	969.8191256	-0.509092978	0.072283942	-7.0429609	1.88E-12	1.05E-11
ENSG00000198618	PPIAP22	2197.300166	2581.3453	1813.255041	-0.509113042	0.067525139	-7.5396075	4.71E-14	2.82E-13
ENSG00000005448	WDR54	276.1125413	324.33875	227.886329	-0.50914682	0.139984606	-3.6371629	0.000276	0.000785
ENSG00000146731	CCT6A	7230.64764	8493.7642	5967.531041	-0.50922874	0.043925364	-11.593045	4.47E-31	4.90E-30
ENSG00000183527	PSMG1	811.282868	953.31555	669.2501829	-0.509631993	0.093844246	-5.4306152	5.62E-08	2.30E-07
ENSG00000213015	ZNF580	391.8122452	460.10345	323.5210364	-0.509772602	0.135870911	-3.7518892	0.000176	0.00051
ENSG00000162714	ZNF496	1414.475354	1661.9037	1167.047023	-0.509824095	0.069288161	-7.3580261	1.87E-13	1.09E-12
ENSG00000162244	RPL29	18115.5753	21283.685	14947.46606	-0.509914204	0.043307815	-11.774184	5.30E-32	5.97E-31
ENSG00000100294	MCAT	312.3688038	366.95225	257.7853595	-0.509924978	0.127327528	-4.004829	6.21E-05	0.000191
ENSG00000170088	TMEM192	1027.048719	1206.8201	847.2773845	-0.510112758	0.074467988	-6.8500946	7.38E-12	3.97E-11
ENSG00000169981	ZNF35	317.1579477	372.67055	261.645342	-0.510817504	0.126237009	-4.0464956	5.20E-05	0.000162
ENSG00000005801	ZNF195	656.6170671	771.87003	541.3641049	-0.510885426	0.09442242	-5.4106368	6.28E-08	2.56E-07
ENSG00000172733	PURG	155.5708816	182.84029	128.3014738	-0.510886112	0.212303955	-2.40639	0.016111	0.034629
ENSG00000053254	FOXN3	2193.047772	2577.87	1808.225513	-0.511039627	0.062503843	-8.1761314	2.93E-16	1.96E-15
ENSG00000178996	SNX18	1373.061827	1614.0017	1132.120951	-0.511126826	0.067312799	-7.5933082	3.12E-14	1.88E-13
ENSG00000188643	S100A16	16112.97815	18937.609	13288.34775	-0.511191317	0.050197818	-10.183537	2.35E-24	2.13E-23
ENSG00000160284	SPATC1L	268.4786019	315.40855	221.5486519	-0.511212272	0.146539006	-3.4885747	0.000486	0.001338
ENSG00000083845	RPS5	9511.999514	11179.621	7844.377577	-0.511301792	0.055997695	-9.130765	6.80E-20	5.30E-19
ENSG00000135540	NHSL1	2026.785584	2381.9352	1671.635979	-0.511333208	0.062293636	-8.2084341	2.24E-16	1.51E-15
ENSG00000230202	AL450405.1	121.0036823	142.12874	99.87862006	-0.511353737	0.213831534	-2.391386	0.016785	0.035971
ENSG00000132640	BTBD3	673.8430887	792.02856	555.6576179	-0.511385656	0.103905825	-4.9216264	8.58E-07	3.19E-06
ENSG00000150756	ATPSCKMT	275.1258137	323.43632	226.8153062	-0.51140213	0.147590424	-3.4650089	0.00053	0.001453
ENSG00000065717	TLE2	779.5946469	916.45516	642.734132	-0.511543245	0.091884173	-5.5672618	2.59E-08	1.09E-07
ENSG00000124588	NQO2	607.4786792	713.83732	501.1200403	-0.511823023	0.105993267	-4.8288258	1.37E-06	5.02E-06
ENSG00000179409	GEMIN4	881.8132456	1036.7663	726.8601596	-0.511924287	0.085928768	-5.9575425	2.56E-09	1.16E-08
ENSG00000132122	SPATA6	150.4416679	176.90073	123.9826075	-0.512678244	0.193321554	-2.6519456	0.008003	0.018239
ENSG00000128915	ICE2	977.4651432	1149.448	805.4823304	-0.512782383	0.082806489	-6.1925386	5.92E-10	2.80E-09
ENSG00000218336	TENM3	2110.85131	2481.8165	1739.886095	-0.512865062	0.064036531	-8.0089451	1.16E-15	7.52E-15
ENSG00000082153	BZW1	8583.459282	10093.384	7073.534596	-0.512957057	0.043016027	-11.924789	8.79E-33	1.01E-31
ENSG00000082781	ITGB5	10566.52469	12426.05	8706.999633	-0.513181264	0.040212932	-12.761598	2.69E-37	3.46E-36
ENSG00000075239	ACAT1	2004.307689	2357.2318	1651.383598	-0.513238443	0.061596884	-8.3322144	7.93E-17	5.44E-16
ENSG00000084693	AGBL5	754.1054245	886.92541	621.2854406	-0.513277152	0.099091359	-5.1798377	2.22E-07	8.67E-07
ENSG00000110074	FOXRED1	447.4171427	526.18202	368.6522614	-0.513299685	0.11421536	-4.4941389	6.99E-06	2.38E-05
ENSG00000145414	NAF1	331.7391825	390.41862	273.0597479	-0.514034267	0.13491003	-3.8102005	0.000139	0.00041
ENSG00000130332	LSM7	857.5092256	1008.766	706.2524913	-0.514045727	0.0877615	-5.8573033	4.70E-09	2.09E-08
ENSG00000019485	PRDM11	312.1131959	367.08823	257.1381637	-0.514159398	0.142213704	-3.6153998	0.0003	0.00085
ENSG00000154122	ANKH	947.1654579	1114.55	779.7809476	-0.514168553	0.0887444	-5.793814	6.88E-09	3.02E-08
ENSG00000110063	DCPS	670.2461593	788.15743	552.3348865	-0.514257273	0.107277195	-4.7937241	1.64E-06	5.94E-06
ENSG00000170456	DENND5B	999.0942849	1175.4947	822.6938613	-0.51441702	0.078578173	-6.5465637	5.89E-11	2.99E-10
ENSG00000127528	KLF2	463.1321325	544.65847	381.6057932	-0.514469344	0.127335083	-4.0402797	5.34E-05	0.000166
ENSG00000198612	COPS8	1830.327717	2153.3917	1507.263713	-0.514519784	0.069896713	-7.3611442	1.82E-13	1.06E-12
ENSG00000141577	CEP131	437.5196345	514.76342	360.2758533	-0.514667634	0.116492847	-4.4180192	9.96E-06	3.34E-05
ENSG00000166352	C11orf74	353.5232269	416.17562	290.870832	-0.515121189	0.141079738	-3.6512769	0.000261	0.000745
ENSG00000138036	DYNC2LI1	204.9749004	241.29331	168.656487	-0.515224949	0.163301933	-3.155045	0.001605	0.004096
ENSG00000083123	BCKDHB	247.5401746	291.25097	203.8293746	-0.515676671	0.155744279	-3.3110473	0.000929	0.002461

ENSG00000064102	INTS13	1040.845579	1225.0351	856.6560983	-0.515758355	0.074998756	-6.8768921	6.12E-12	3.30E-11
ENSG00000115073	ACTR1B	1521.462674	1791.2335	1251.691864	-0.517238117	0.067950747	-7.6119563	2.70E-14	1.64E-13
ENSG00000047230	CTPS2	679.7017396	800.22683	559.1766521	-0.517946623	0.09521684	-5.4396536	5.34E-08	2.19E-07
ENSG00000144713	RPL32	10807.58562	12727.294	8887.87713	-0.518164837	0.045066558	-11.497768	1.35E-30	1.47E-29
ENSG00000105220	GPI	5346.877571	6296.3479	4397.407196	-0.518221632	0.057697062	-8.9817681	2.66E-19	2.02E-18
ENSG00000076067	RBMS2	3608.102758	4249.528	2966.677469	-0.518428124	0.054367838	-9.5355663	1.49E-21	1.23E-20
ENSG00000137809	ITGA11	346.916463	408.75671	285.0762116	-0.519639076	0.165555284	-3.1387647	0.001697	0.004313
ENSG00000162733	DDR2	6046.24292	7124.0371	4968.448713	-0.519712309	0.053689385	-9.6799826	3.67E-22	3.09E-21
ENSG00000177728	TMEM94	1690.261703	1991.1177	1389.405683	-0.519883205	0.069571067	-7.4726927	7.86E-14	4.64E-13
ENSG00000125691	RPL23	13063.71425	15394.003	10733.42584	-0.520185986	0.038433139	-13.53483	9.74E-42	1.39E-40
ENSG00000086504	MRPL28	1287.293948	1516.671	1057.916872	-0.520248755	0.078161852	-6.6560444	2.81E-11	1.46E-10
ENSG00000107960	STN1	530.2640832	625.06841	435.4597575	-0.520649958	0.105799537	-4.9210986	8.61E-07	3.20E-06
ENSG00000196950	SLC39A10	1585.143825	1868.3277	1301.959906	-0.520768774	0.064897542	-8.0244761	1.02E-15	6.64E-15
ENSG00000100216	TOMM22	1524.107752	1796.0236	1252.191863	-0.520846922	0.079868701	-6.5212895	6.97E-11	3.52E-10
ENSG00000148334	PTGES2	1112.346093	1310.6449	914.0473014	-0.520939313	0.080360382	-6.482539	9.02E-11	4.52E-10
ENSG00000149273	RPS3	36572.9908	43107.802	30038.17987	-0.521142428	0.038785482	-13.436533	3.69E-41	5.21E-40
ENSG00000101365	IDH3B	1416.232559	1669.1629	1163.302181	-0.521290107	0.074316002	-7.0145069	2.31E-12	1.28E-11
ENSG00000118181	RPS25	11764.09975	13868.767	9659.432551	-0.521830624	0.041048408	-12.712567	5.04E-37	6.46E-36
ENSG00000153933	DGKE	268.3601145	316.58082	220.1394098	-0.521835662	0.162904601	-3.2033206	0.001359	0.003512
ENSG00000162980	ARL5A	877.9943978	1034.9981	720.9906513	-0.521992104	0.085750371	-6.0873452	1.15E-09	5.32E-09
ENSG00000119599	DCAF4	389.2231723	459.10881	319.3375307	-0.522682172	0.119194168	-4.3851321	1.16E-05	3.86E-05
ENSG00000121851	POLR3GL	281.2597999	331.70361	230.8159862	-0.523278093	0.144510154	-3.6210472	0.000293	0.000833
ENSG00000130021	PUDP	1049.811984	1238.1394	861.4845394	-0.523449127	0.08549422	-6.1226259	9.20E-10	4.30E-09
ENSG00000160050	CCDC28B	262.0911845	309.22003	214.9623396	-0.523488341	0.142683442	-3.6688794	0.000244	0.000698
ENSG00000147140	NONO	10321.84454	12174.727	8468.962031	-0.523612734	0.038188667	-13.711207	8.70E-43	1.27E-41
ENSG00000182742	HOXB4	489.918497	577.81908	402.0179184	-0.523723555	0.110543809	-4.7377014	2.16E-06	7.74E-06
ENSG00000168002	POLR2G	1106.825067	1305.3005	908.3496453	-0.523761771	0.08580664	-6.1039771	1.03E-09	4.81E-09
ENSG00000108604	SMARCD2	1501.956648	1771.559	1232.354316	-0.524115162	0.068014068	-7.7059817	1.30E-14	8.02E-14
ENSG00000244509	APOBEC3C	2596.895149	3063.4231	2130.367152	-0.524476254	0.056155521	-9.3397094	9.66E-21	7.75E-20
ENSG00000070087	PFN2	2036.071957	2402.3979	1669.745982	-0.524555729	0.057664363	-9.0967055	9.31E-20	7.21E-19
ENSG00000132004	FBXW9	259.7678944	306.34645	213.1893412	-0.524603023	0.162541848	-3.2274951	0.001249	0.003252
ENSG00000182173	TSEN54	231.1168875	272.53668	189.6970983	-0.52479268	0.1820404	-2.8828363	0.003941	0.009478
ENSG00000143924	EML4	1099.551082	1297.4546	901.647517	-0.524831242	0.074460942	-7.0484099	1.81E-12	1.01E-11
ENSG00000138794	CASP6	686.2954731	809.81292	562.7780297	-0.525440689	0.09633645	-5.4542252	4.92E-08	2.03E-07
ENSG00000138829	FBN2	108.9660533	128.57309	89.3590131	-0.52612918	0.221128567	-2.3792909	0.017346	0.037063
ENSG00000162694	EXTL2	1137.34476	1343.1861	931.5034624	-0.527344063	0.075758003	-6.9609023	3.38E-12	1.86E-11
ENSG00000070814	TCOF1	1705.385367	2013.2632	1397.507534	-0.527504985	0.067681735	-7.7939045	6.50E-15	4.06E-14
ENSG00000177732	SOX12	1753.225208	2069.7342	1436.716234	-0.527609816	0.070771375	-7.4551302	8.98E-14	5.29E-13
ENSG00000166012	TAF1D	1445.674484	1707.2399	1184.10906	-0.527831304	0.066818494	-7.8994792	2.80E-15	1.78E-14
ENSG00000205268	PDE7A	244.7421126	289.21199	200.2722354	-0.528435925	0.146945642	-3.596132	0.000323	0.00091
ENSG00000108819	PPP1R9B	2626.678749	3102.4173	2150.940241	-0.528774603	0.056631627	-9.33709	9.90E-21	7.94E-20
ENSG00000110172	CHORDC1	1029.034092	1215.8623	842.2058601	-0.529249795	0.077580404	-6.821952	8.98E-12	4.80E-11
ENSG00000136942	RPL35	6963.989222	8227.4772	5700.501289	-0.529457605	0.045271483	-11.695168	1.35E-31	1.50E-30
ENSG00000093217	XYLB	154.4384559	182.48347	126.3934426	-0.52990732	0.18380384	-2.8830046	0.003939	0.009475
ENSG00000134901	KDELC1	1081.883576	1278.6891	885.0780609	-0.529909284	0.090505721	-5.8549811	4.77E-09	2.12E-08
ENSG00000029993	HMGB3	1161.04247	1371.9231	950.1618062	-0.529997431	0.073128968	-7.2474349	4.25E-13	2.43E-12
ENSG00000196531	NACA	8214.15984	9707.1294	6721.190278	-0.530108513	0.043234038	-12.261369	1.46E-34	1.75E-33
ENSG00000141543	EIF4A3	1671.080179	1974.4089	1367.751432	-0.530182295	0.066716047	-7.9468482	1.91E-15	1.23E-14
ENSG00000233276	GPX1	10615.50047	12544.149	8686.851936	-0.53026726	0.0635342	-8.3461704	7.05E-17	4.85E-16
ENSG00000164465	DCBLD1	2103.149677	2485.4365	1720.862888	-0.530527512	0.056281262	-9.4263614	4.25E-21	3.45E-20
ENSG00000105287	PRKD2	3002.834018	3548.4799	2457.188092	-0.530537218	0.057224734	-9.2711173	1.84E-20	1.46E-19
ENSG00000105552	BCAT2	842.8033039	996.06744	689.5391682	-0.530857582	0.088552789	-5.9948149	2.04E-09	9.29E-09
ENSG00000168505	GBX2	264.0410298	312.01199	216.0700701	-0.531151125	0.142711889	-3.7218422	0.000198	0.000572
ENSG00000138363	ATIC	1293.379509	1529.1537	1057.605354	-0.53158	0.070713593	-7.5173666	5.59E-14	3.32E-13
ENSG00000145741	BTF3	11166.60225	13201.371	9131.833824	-0.531619292	0.037524539	-14.167244	1.46E-45	2.26E-44
ENSG00000147955	SIGMAR1	1699.067013	2008.3418	1389.79223	-0.531850491	0.07605252	-6.9932001	2.69E-12	1.48E-11
ENSG00000174444	RPL4	35324.97178	41774.874	28875.06992	-0.532842116	0.037492269	-14.212053	7.71E-46	1.20E-44
ENSG00000091490	SEL1L3	11366.52974	13443.423	9289.636695	-0.533091844	0.040601846	-13.129744	2.22E-39	3.01E-38
ENSG00000173848	NET1	604.8782875	715.34075	494.4158206	-0.533296882	0.098737299	-5.4011694	6.62E-08	2.69E-07
ENSG00000189298	ZKSCAN3	171.3811777	202.86716	139.8951979	-0.533391637	0.215503983	-2.4750895	0.01332	0.029151
ENSG00000130204	AC011481.1	1495.831106	1769.068	1222.594213	-0.533872573	0.077472163	-6.8911536	5.53E-12	3.00E-11
ENSG00000088808	PPP1R13B	841.5035044	995.39843	687.6085816	-0.533903566	0.081147542	-6.5794176	4.72E-11	2.41E-10
ENSG00000165644	COMTD1	140.5201727	166.09823	114.942113	-0.534146718	0.196814588	-2.713959	0.006648	0.015345
ENSG00000184967	NOC4L	321.2272441	379.75153	262.7029534	-0.534178516	0.13990952	-3.8180284	0.000135	0.000398
ENSG00000181467	RAP2B	1772.540427	2097.2527	1447.828171	-0.534502216	0.060949184	-8.7696369	1.79E-18	1.32E-17
ENSG00000142494	SLC47A1	206.0879112	243.89276	168.2830607	-0.534714532	0.158943612	-3.3641776	0.000768	0.002056
ENSG00000182551	ADI1	1738.56583	2057.2181	1419.913532	-0.534730458	0.063351598	-8.4406784	3.16E-17	2.20E-16
ENSG00000198730	CTR9	2519.080525	2981.1595	2057.001562	-0.534993928	0.055942266	-9.5633225	1.14E-21	9.43E-21
ENSG00000168763	CNNM3	474.046563	560.81095	387.2821785	-0.535220276	0.114080677	-4.6915945	2.71E-06	9.63E-06
ENSG00000198900	TOP1	5215.845685	6173.0763	4258.615084	-0.535625597	0.047137295	-11.363096	6.38E-30	6.83E-29
ENSG00000170485	NPAS2	1287.347885	1523.581	1051.11475	-0.535900957	0.069961248	-7.6599685	1.86E-14	1.14E-13
ENSG00000167792	NDUFV1	2746.97206	3250.7089	2243.235234	-0.535910343	0.063393381	-8.4537271	2.82E-17	1.97E-16
ENSG00000073050	XRCC1	544.6021352	644.6875	444.5167681	-0.535982883	0.101029727	-5.3051997	1.13E-07	4.50E-07
ENSG00000135521	LTV1	352.9699582	417.87579	288.064128	-0.536106186	0.125379093	-4.2758818	1.90E-05	6.20E-05
ENSG00000169718	DUS1L	1296.102928	1534.0696	1058.136293	-0.536260591	0.070817127	-7.5724703	3.66E-14	2.20E-13
ENSG00000215030	RPL13P12	1276.576429	1510.9092	1042.243632	-0.536781094	0.08016911	-6.69561	2.15E-11	1.12E-10
ENSG00000077152	UBE2T	193.0042294	228.46576	157.5427014	-0.536964814	0.173042264	-3.1030848	0.001915	0.004832
ENSG00000171490	RSL1D1	3531.509953	4181.7212	2881.298736	-0.537225378	0.052478189	-10.237117	1.35E-24	1.24E-23
ENSG00000100814	CCNB1IP1	417.2729311	493.93881	340.6070542	-0.537480452	0.131719983	-4.0804777	4.49E-05	0.000141
ENSG00000196535	MYO18A	1215.925098	1439.8977	991.9524802	-0.537588506	0.072234998	-7.4422167	9.90E-14	5.82E-13
ENSG00000167526	RPL13	19441.24639	23022.733	15859.76023	-0.537823318	0.044032061	-12.214357	2.61E-34	3.10E-33
ENSG00000173581	CCDC106	410.8532867	486.46592	335.2406493	-0.537977849	0.129355798	-4.1589001	3.20E-05	0.000102
ENSG00000101019	UQCC1	633.535717	750.39234	516.6790965	-0.538637733	0.09330871	-5.7726415	7.80E-09	3.41E-08
ENSG00000146243	IRAK1BP1	158.1811559	187.50186	128.8604549	-0.539154543	0.183607002	-2.9364596	0.00332	0.008065
ENSG00000205581	HMGN1	4496.814894	5327.6838	3665.946024	-0.539471866	0.046085579	-11.705872	1.19E-31	1.33E-30

ENSG00000271976	AC012467.2	89.09716854	105.51964	72.67469798	-0.539860018	0.235862028	-2.2888806	0.022086	0.04604
ENSG00000170144	HNRNPA3	8098.97267	9597.7924	6600.152933	-0.540115997	0.043182709	-12.507691	6.78E-36	8.44E-35
ENSG00000137936	BCAR3	1866.069954	2211.0605	1521.079455	-0.540479777	0.083888922	-6.4428027	1.17E-10	5.83E-10
ENSG00000213339	QTRT1	461.2133135	546.95646	375.4701671	-0.540736785	0.138654214	-3.8998943	9.62E-05	0.00029
ENSG00000143149	ALDH9A1	1686.719678	1999.4264	1374.012945	-0.540750221	0.073992055	-7.30822	2.71E-13	1.56E-12
ENSG00000171365	CLCN5	831.4583913	985.40829	677.5084968	-0.540952918	0.09658941	-5.6005407	2.14E-08	9.04E-08
ENSG00000063177	RPL18	11818.83142	14009.973	9627.690145	-0.541312185	0.046290387	-11.693836	1.37E-31	1.52E-30
ENSG00000008324	SS18L2	348.3975335	413.13588	283.6591853	-0.541387028	0.135039925	-4.0090886	6.10E-05	0.000188
ENSG00000065268	WDR18	655.9195139	777.34272	534.4963045	-0.541605992	0.112853696	-4.799187	1.59E-06	5.78E-06
ENSG00000196465	MYL6B	1888.371031	2239.4785	1537.263573	-0.542804476	0.059325281	-9.1496318	5.71E-20	4.47E-19
ENSG00000167283	ATP5MG	336.4807297	399.15751	273.8039466	-0.542832391	0.127914494	-4.2437129	2.20E-05	7.12E-05
ENSG00000011258	MBTD1	429.6274941	509.79333	349.461656	-0.542860953	0.126091465	-4.305295	1.67E-05	5.47E-05
ENSG00000203668	CHML	641.9933465	761.60837	522.3783274	-0.542867421	0.095826835	-5.6650877	1.47E-08	6.30E-08
ENSG00000213465	ARL2	773.8529301	917.8298	629.8760649	-0.543675202	0.093089747	-5.8403339	5.21E-09	2.31E-08
ENSG00000136144	RCBTB1	1102.384284	1307.5427	897.2258339	-0.543709669	0.072846441	-7.4637781	8.41E-14	4.96E-13
ENSG00000134809	TIMM10	645.7631756	765.99382	525.5325357	-0.543805517	0.10004717	-5.4354912	5.46E-08	2.24E-07
ENSG00000168237	GLYCTK	213.275453	252.84562	173.7052869	-0.544091907	0.174318181	-3.1212574	0.001801	0.00456
ENSG00000074201	CLNS1A	1797.817624	2133.3233	1462.311965	-0.544638462	0.062949447	-8.6519975	5.06E-18	3.63E-17
ENSG00000185414	MRPL30	965.129617	1145.18	785.0792365	-0.545265723	0.079394706	-6.8677844	6.52E-12	3.52E-11
ENSG00000131196	NFATC1	892.4134918	1059.0833	725.743642	-0.545650138	0.082315576	-6.6287593	3.39E-11	1.75E-10
ENSG00000084774	CAD	2842.312853	3373.9236	2310.702117	-0.546431859	0.060548716	-9.0246647	1.80E-19	1.38E-18
ENSG00000127824	TUBA4A	288.8423703	342.69044	234.9942958	-0.546836522	0.152436713	-3.587302	0.000334	0.000939
ENSG00000178075	GRAMD1C	272.8609423	324.02794	221.6939445	-0.546853917	0.13852329	-3.9477399	7.89E-05	0.00024
ENSG00000276345	AC004556.1	143.1001134	169.93601	116.2642184	-0.548643898	0.185105649	-2.9639501	0.003037	0.007435
ENSG00000111640	GAPDH	85623.33347	101713.95	69532.71599	-0.548779201	0.056245888	-9.7567879	1.73E-22	1.47E-21
ENSG00000088387	DOCK9	7492.846575	8902.0458	6083.647322	-0.549100415	0.04584724	-11.976739	4.70E-33	5.46E-32
ENSG00000111731	C2CD5	789.6133531	938.49517	640.7315368	-0.549917414	0.086611873	-6.3492151	2.16E-10	1.05E-09
ENSG00000107186	MPDZ	2812.375357	3342.134	2282.616751	-0.549939524	0.05635334	-9.7587742	1.69E-22	1.45E-21
ENSG00000154217	PITPNC1	302.8127293	359.75379	245.8716667	-0.550097521	0.129909286	-4.2344742	2.29E-05	7.40E-05
ENSG00000124831	LRRFIP1	6142.586533	7300.9874	4984.185635	-0.550738927	0.042563754	-12.939153	2.71E-38	3.57E-37
ENSG00000196440	ARMCX4	1300.672397	1546.4339	1054.910855	-0.551128605	0.076976458	-7.1597034	8.09E-13	4.56E-12
ENSG00000167136	ENDOG	283.5870166	337.00965	230.1643822	-0.551159782	0.136320645	-4.0431131	5.27E-05	0.000164
ENSG00000136527	TRA2B	2056.213528	2444.24	1668.187096	-0.551288313	0.070439619	-7.8263955	5.02E-15	3.16E-14
ENSG00000165526	RPUSD4	994.1138387	1181.5219	806.7057735	-0.551296032	0.080220846	-6.8722291	6.32E-12	3.41E-11
ENSG00000167515	TRAPPC2L	967.7791356	1150.2269	785.3313512	-0.551325792	0.080221576	-6.8725375	6.31E-12	3.40E-11
ENSG00000167862	MRPL58	396.2379211	471.04523	321.4306105	-0.551416156	0.117883022	-4.6776554	2.90E-06	1.03E-05
ENSG00000213741	RPS29	4324.048834	5140.7206	3507.377055	-0.551436478	0.050741031	-10.867664	1.64E-27	1.66E-26
ENSG00000160917	CPSF4	551.0350958	655.15412	446.9160672	-0.551600726	0.106286238	-5.1897662	2.11E-07	8.22E-07
ENSG00000103034	NDRG4	3453.596897	4105.5969	2801.596915	-0.551664095	0.052540676	-10.499753	8.66E-26	8.25E-25
ENSG00000099783	HNRNPM	6316.375026	7512.361	5120.389009	-0.552950427	0.050188092	-11.017562	3.14E-28	3.22E-27
ENSG00000172687	ZNF738	96.72773138	115.07634	78.37912496	-0.55309823	0.237482514	-2.3290061	0.019859	0.041847
ENSG00000187790	FANCM	303.4278985	360.86347	245.9923312	-0.55314019	0.129137954	-4.2833278	1.84E-05	6.01E-05
ENSG00000137040	RANBP6	1164.166624	1385.1592	943.1740437	-0.553471518	0.081138513	-6.821317	9.02E-12	4.82E-11
ENSG00000181817	LSM10	567.9736114	675.62914	460.3180822	-0.553739251	0.10350457	-5.3499015	8.80E-08	3.55E-07
ENSG00000158715	SLC45A3	588.8650431	700.54135	477.18874	-0.553839004	0.099666157	-5.5569415	2.75E-08	1.15E-07
ENSG00000175591	P2RY2	164.5902154	195.7505	133.4299351	-0.554061134	0.179411987	-3.0882058	0.002014	0.005066
ENSG00000147996	CBWD5	248.9518432	296.39613	201.5075516	-0.5543038	0.15318799	-3.6184547	0.000296	0.00084
ENSG00000212802	RPL15P3	103.6333652	123.16885	84.09788268	-0.554543555	0.236652168	-2.3432853	0.019115	0.040456
ENSG00000171960	PPIH	276.8742431	329.4079	224.3405848	-0.554646338	0.139545039	-3.9746761	7.05E-05	0.000216
ENSG00000132600	PRMT7	455.5778348	542.38224	368.7734321	-0.555092686	0.113000175	-4.912317	9.00E-07	3.34E-06
ENSG00000163933	RFT1	364.314696	433.86904	294.7603568	-0.555738609	0.128677825	-4.3188375	1.57E-05	5.16E-05
ENSG00000172009	THOP1	1267.108034	1508.035	1026.181041	-0.555776233	0.080280451	-6.9229336	4.42E-12	2.41E-11
ENSG00000170854	RIOX2	789.1684798	939.38996	638.9470025	-0.555984673	0.090823155	-6.1216181	9.26E-10	4.32E-09
ENSG00000149150	SLC43A1	347.6472889	413.76998	281.5245962	-0.556179586	0.14015591	-3.9682921	7.24E-05	0.000221
ENSG00000164032	H2AFZ	5332.629739	6348.0057	4317.253741	-0.556375201	0.052235293	-10.651327	1.72E-26	1.68E-25
ENSG00000138430	OLA1	2962.553014	3526.918	2398.188061	-0.556559476	0.058753891	-9.4727254	2.73E-21	2.23E-20
ENSG00000159685	CHCHD6	183.5923867	218.65395	148.5308204	-0.557844996	0.176939246	-3.1527488	0.001617	0.004127
ENSG00000171792	RHNO1	599.737857	714.08623	485.3894877	-0.558100037	0.097535074	-5.7220445	1.05E-08	4.55E-08
ENSG00000205208	C4orf46	317.7669385	378.34073	257.1931501	-0.558408892	0.131900113	-4.233574	2.30E-05	7.43E-05
ENSG00000088826	SMOX	702.0573281	836.02872	568.0859407	-0.558691496	0.108263002	-5.1605025	2.46E-07	9.58E-07
ENSG00000116898	MRPS15	1283.815442	1529.6755	1037.955424	-0.559431957	0.071531456	-7.8207825	5.25E-15	3.29E-14
ENSG00000137404	NRM	271.9734608	323.86437	220.082548	-0.559537573	0.155546788	-3.59723	0.000322	0.000906
ENSG00000185291	IL3RA	902.5116124	1075.363	729.6602242	-0.55965483	0.096790035	-5.7821534	7.38E-09	3.22E-08
ENSG00000110700	RPS13	8813.611529	10502.25	7124.973178	-0.559874911	0.050976545	-10.98299	4.61E-28	4.71E-27
ENSG00000185504	FAAP100	1288.884856	1535.7671	1042.002603	-0.559960536	0.070960639	-7.8911428	2.99E-15	1.90E-14
ENSG00000161547	SRSF2	3036.590128	3618.1655	2455.014732	-0.560015954	0.054214565	-10.329622	5.18E-25	4.80E-24
ENSG00000181938	GINS3	338.2779538	403.07735	273.4785573	-0.560250466	0.128243323	-4.3686521	1.25E-05	4.15E-05
ENSG00000081320	STK17B	295.1844072	351.83243	238.5363822	-0.560598236	0.141180276	-3.9707971	7.16E-05	0.000219
ENSG00000172889	EGFL7	8082.682089	9633.6491	6531.715042	-0.560772314	0.041981832	-13.3575	1.07E-40	1.49E-39
ENSG00000162139	NEU3	371.3244219	442.68719	299.9616523	-0.560872955	0.117945171	-4.7553702	1.98E-06	7.12E-06
ENSG00000173041	ZNF680	184.5023418	219.9274	149.0772812	-0.560876201	0.16349849	-3.4304672	0.000603	0.00164
ENSG00000161016	RPL8	23812.31268	28384.284	19240.34111	-0.56104933	0.048698748	-11.520816	1.04E-30	1.13E-29
ENSG00000144791	LIMD1	1742.700784	2077.8277	1407.573827	-0.561258359	0.063479375	-8.8415861	9.44E-19	7.02E-18
ENSG00000169592	INO80E	999.276351	1190.9024	807.6503263	-0.561301322	0.082002401	-6.8449377	7.65E-12	4.11E-11
ENSG00000070785	EIF2B3	587.6169134	700.66564	474.5681907	-0.561866443	0.099416184	-5.6516597	1.59E-08	6.79E-08
ENSG00000113761	ZNF346	696.3687795	830.19386	562.543697	-0.56201154	0.091608175	-6.1349496	8.52E-10	3.99E-09
ENSG00000116191	RALGPS2	765.2416584	912.55571	617.9276055	-0.562088685	0.085574772	-6.5683924	5.09E-11	2.59E-10
ENSG00000164574	GALNT10	5565.416215	6637.1777	4493.654697	-0.562753991	0.043142655	-13.044028	6.87E-39	9.18E-38
ENSG00000117174	ZNHIT6	849.0468251	1012.7554	685.3382374	-0.563056608	0.080807704	-6.9678581	3.22E-12	1.77E-11
ENSG00000127220	ABHD8	281.1701427	335.23677	227.1035191	-0.563232557	0.146541975	-3.8434896	0.000121	0.00036
ENSG00000069122	ADGRF5	6509.18639	7765.2563	5253.116471	-0.56382015	0.144178117	-3.9105806	9.21E-05	0.000278
ENSG00000105967	TFEC	1078.963617	1287.7753	870.1519448	-0.56409637	0.09592941	-5.8803277	4.09E-09	1.83E-08
ENSG00000138326	RPS24	15990.44969	19077.431	12903.46825	-0.564116809	0.035574698	-15.857248	1.25E-56	2.33E-55
ENSG00000230479	AP000695.1	494.102278	589.57081	398.6337481	-0.564123754	0.108973218	-5.1767193	2.26E-07	8.81E-07
ENSG00000242612	DECR2	260.8464312	311.13163	210.5612331	-0.564378816	0.173515281	-3.2526174	0.001143	0.002991

ENSG00000158711	ELK4	3979.628893	4749.0251	3210.232666	-0.564535632	0.057876898	-9.7540756	1.77E-22	1.51E-21
ENSG00000221914	PPP2R2A	3434.375902	4098.2525	2770.499276	-0.564649383	0.053626323	-10.529332	6.33E-26	6.05E-25
ENSG00000000460	C1orf112	160.5506842	191.60831	129.4930579	-0.564786823	0.176215972	-3.205083	0.00135	0.003494
ENSG00000129197	RPAIN	666.1024902	794.84759	537.3573949	-0.564807963	0.090610958	-6.2333296	4.57E-10	2.18E-09
ENSG00000107819	SFXN3	2557.03561	3051.3397	2062.731508	-0.564996775	0.057644164	-9.8014567	1.11E-22	9.53E-22
ENSG00000136840	ST6GALNAC4	1451.715587	1732.0217	1171.409425	-0.565290263	0.088772725	-6.3678372	1.92E-10	9.39E-10
ENSG00000147403	RPL10	32126.48988	38341.703	25911.27688	-0.565395641	0.037487835	-15.08211	2.12E-51	3.63E-50
ENSG00000213619	NDUFS3	903.1287365	1077.9781	728.2793713	-0.566566263	0.083644083	-6.7735367	1.26E-11	6.66E-11
ENSG00000037241	RPL26L1	625.975557	747.33695	504.6141648	-0.566746081	0.09448936	-5.9979883	2.00E-09	9.11E-09
ENSG00000151849	CENPJ	239.40667	285.89944	192.9138987	-0.566770941	0.157996295	-3.587242	0.000334	0.000939
ENSG00000146757	ZNF92	327.4196529	391.17519	263.6641152	-0.56753246	0.131847062	-4.3044756	1.67E-05	5.49E-05
ENSG00000172819	RARG	431.7831488	515.71253	347.8537671	-0.567884532	0.108743236	-5.2222516	1.77E-07	6.94E-07
ENSG00000133961	NUMB	4916.154362	5871.0228	3961.28594	-0.568006853	0.047937664	-11.848864	2.18E-32	2.49E-31
ENSG00000136450	SRSF1	1021.08526	1219.6292	822.5413001	-0.56814269	0.07730566	-7.3493027	1.99E-13	1.16E-12
ENSG00000172922	RNASEH2C	748.0255015	893.34634	602.7046584	-0.568240084	0.0880811	-6.4513282	1.11E-10	5.52E-10
ENSG00000163820	FYCO1	2940.850332	3512.4485	2369.252182	-0.568267435	0.053782837	-10.565962	4.29E-26	4.13E-25
ENSG00000197632	SERPINB2	231.2753922	276.04863	186.5021592	-0.568493324	0.174421312	-3.2593111	0.001117	0.002926
ENSG00000051596	THOC3	616.4417043	736.25925	496.6241539	-0.568847213	0.104229427	-5.457645	4.82E-08	1.99E-07
ENSG00000162129	CLPB	1140.886088	1362.5758	919.19633	-0.569062508	0.086131341	-6.6069157	3.92E-11	2.02E-10
ENSG00000131238	PPT1	4109.660027	4909.7545	3309.565558	-0.569204551	0.050336877	-11.307904	1.20E-29	1.28E-28
ENSG00000161921	CXCL16	1049.444213	1254.0397	844.8487017	-0.569698413	0.087682474	-6.4972895	8.18E-11	4.11E-10
ENSG00000203880	PCMTD2	850.4316303	1016.4116	684.4516913	-0.569786511	0.089656706	-6.3552024	2.08E-10	1.02E-09
ENSG00000144559	TAMM41	227.8246366	272.39947	183.2498043	-0.569868398	0.152753929	-3.7306301	0.000191	0.000553
ENSG00000170889	RPS9	17182.34082	20533.535	13831.14616	-0.57012717	0.043206019	-13.19555	9.31E-40	1.27E-38
ENSG00000148153	INIP	721.7311579	862.75149	580.7108283	-0.570147126	0.095717176	-5.9565811	2.58E-09	1.17E-08
ENSG00000230453	ANKRD18B	223.0499088	266.68379	179.4160256	-0.570316151	0.152274298	-3.7453212	0.00018	0.000523
ENSG00000126453	BCL2L12	302.3044317	361.39592	243.2129466	-0.570506072	0.146553644	-3.892814	9.91E-05	0.000298
ENSG00000125166	GOT2	4825.55377	5767.8483	3883.25927	-0.570972297	0.052421561	-10.891936	1.26E-27	1.27E-26
ENSG00000111275	ALDH2	2008.63922	2401.148	1616.130411	-0.571117246	0.058898954	-9.69656	3.12E-22	2.64E-21
ENSG00000170962	PDGFD	671.5286882	802.9867	540.0706787	-0.571172155	0.112194941	-5.0908905	3.56E-07	1.37E-06
ENSG00000178966	RMI1	206.702709	247.12363	166.2817877	-0.571294066	0.158374276	-3.6072403	0.000309	0.000874
ENSG00000105364	MRPL4	1084.094117	1295.7101	872.4780924	-0.571654742	0.088196927	-6.4815721	9.08E-11	4.55E-10
ENSG00000151414	NEK7	1972.724724	2358.3729	1587.076549	-0.572006681	0.06950254	-8.2300112	1.87E-16	1.26E-15
ENSG00000117748	RPA2	710.8016577	849.90316	571.7001578	-0.572211984	0.090408126	-6.3292097	2.46E-10	1.20E-09
ENSG00000048342	CC2D2A	631.7994049	755.60276	507.9960465	-0.572216218	0.09753081	-5.8670303	4.44E-09	1.97E-08
ENSG00000185219	ZNF445	750.6481676	897.9543	603.3420349	-0.573184897	0.088661303	-6.4648824	1.01E-10	5.06E-10
ENSG00000152147	GEMIN6	270.1301159	323.00561	217.2546202	-0.573825052	0.147403157	-3.8928953	9.91E-05	0.000298
ENSG00000159111	MRPL10	849.2065502	1015.7381	682.6749881	-0.574243504	0.090193288	-6.3668097	1.93E-10	9.45E-10
ENSG00000133612	AGAP3	2541.402202	3040.6956	2042.108779	-0.574413281	0.057256068	-10.032356	1.10E-23	9.78E-23
ENSG00000148229	POLE3	1476.378485	1766.8397	1185.917275	-0.57448557	0.068732119	-8.3583276	6.36E-17	4.38E-16
ENSG00000123374	CDK2	863.3004376	1032.7603	693.8405813	-0.574625172	0.090610166	-6.3417296	2.27E-10	1.11E-09
ENSG00000114391	RPL24	11838.36211	14165.673	9511.050895	-0.57462843	0.045093749	-12.742973	3.41E-37	4.39E-36
ENSG00000133816	MICAL2	14393.43686	17222.576	11564.29722	-0.574694814	0.046154548	-12.451532	1.37E-35	1.69E-34
ENSG00000198598	MMP17	300.3690018	359.42173	241.3162729	-0.574733366	0.13458305	-4.2704736	1.95E-05	6.34E-05
ENSG00000154822	PLCL2	521.9858077	624.73514	419.2364739	-0.575076897	0.105838869	-5.4335132	5.53E-08	2.26E-07
ENSG00000184110	EIF3C	692.7757612	829.18741	556.3641144	-0.575399196	0.096717164	-5.9492976	2.69E-09	1.22E-08
ENSG00000104884	ERCC2	2688.807117	3217.9467	2159.66752	-0.57585684	0.067386502	-8.5455815	1.28E-17	9.05E-17
ENSG00000184207	PGP	584.5151207	699.42783	469.6024105	-0.575926879	0.109979464	-5.2366765	1.63E-07	6.44E-07
ENSG00000150779	TIMM8B	942.3948352	1127.7176	757.0720686	-0.575952761	0.097140561	-5.9290656	3.05E-09	1.37E-08
ENSG00000178464	RPL10P16	132.8383171	159.05526	106.6213759	-0.576848786	0.204844533	-2.8160321	0.004862	0.011489
ENSG00000239306	RBM14	1027.925293	1230.7482	825.1023984	-0.577490521	0.089115263	-6.480265	9.16E-11	4.59E-10
ENSG00000160767	FAM189B	1670.805121	2000.3705	1341.239762	-0.577641414	0.07730078	-7.4726467	7.86E-14	4.64E-13
ENSG00000188917	TRMT2B	781.0501173	935.30908	626.7911551	-0.577824816	0.093690739	-6.1673632	6.94E-10	3.27E-09
ENSG00000138663	COPS4	1217.067917	1457.9726	976.1631895	-0.578305403	0.073174634	-7.9030857	2.72E-15	1.73E-14
ENSG00000176396	EID2	448.4415226	537.04984	359.8332038	-0.578421661	0.121308178	-4.7682	1.86E-06	6.71E-06
ENSG00000008311	AASS	648.3228041	776.74412	519.9014865	-0.578757569	0.099642541	-5.8083381	6.31E-09	2.78E-08
ENSG00000121897	LIAS	135.7195255	162.5085	108.9305473	-0.57958341	0.206231429	-2.8103544	0.004949	0.011679
ENSG00000214078	CPNE1	2178.755738	2611.4306	1746.080843	-0.58104595	0.062803003	-9.2518817	2.21E-20	1.75E-19
ENSG00000137968	SLC44A5	167.5312096	200.91811	134.1443097	-0.581399122	0.181307555	-3.206701	0.001343	0.003476
ENSG00000056277	ZNF280C	136.5012087	163.62653	109.3758883	-0.581495043	0.209178225	-2.7799024	0.005438	0.012757
ENSG00000156381	ANKRD9	468.3105748	561.16301	375.4581353	-0.581735198	0.121731348	-4.7788446	1.76E-06	6.37E-06
ENSG00000090621	PABPC4	6962.637676	8348.3265	5576.948867	-0.582247032	0.049188781	-11.836988	2.51E-32	2.87E-31
ENSG00000166441	RPL27A	17165.94286	20583.42	13748.46589	-0.582256202	0.040352845	-14.429124	3.39E-47	5.42E-46
ENSG00000166123	GPT2	723.3746588	867.41978	579.3295349	-0.58229317	0.090922539	-6.4042775	1.51E-10	7.46E-10
ENSG00000012963	UBR7	672.387775	806.15588	538.6196653	-0.582491633	0.090798132	-6.415238	1.41E-10	6.96E-10
ENSG00000116962	NID1	13476.11297	16161.535	10790.69142	-0.582745673	0.108709618	-5.3605715	8.30E-08	3.35E-07
ENSG00000196636	SDHAF3	101.4316596	121.63437	81.22895173	-0.583089624	0.239996709	-2.4295734	0.015117	0.032667
ENSG00000137411	VARS2	633.8369207	760.05019	507.6236523	-0.583121916	0.095593574	-6.1000117	1.06E-09	4.93E-09
ENSG00000018408	WWTR1	9764.139773	11711.862	7816.417816	-0.583282411	0.039341695	-14.826062	9.94E-50	1.66E-48
ENSG00000155660	PDIA4	15532.20582	18630.459	12433.95306	-0.583338782	0.0353132	-16.519001	2.68E-61	5.35E-60
ENSG00000224032	EPB41L4A-AS1	204.229547	245.01792	163.4411707	-0.583340119	0.157703245	-3.6989735	0.000216	0.000624
ENSG00000197050	ZNF420	126.0285786	151.15927	100.8978823	-0.583381122	0.197136169	-2.95928	0.003084	0.007531
ENSG00000132199	ENOSF1	308.9914376	370.84839	247.1344808	-0.583998787	0.140948412	-4.1433513	3.42E-05	0.000109
ENSG00000156467	UQCRB	3874.43193	4648.349	3100.513626	-0.58400463	0.054551641	-10.705537	9.59E-27	9.44E-26
ENSG00000109016	DHRS7B	291.0191518	349.08417	232.9541298	-0.584118881	0.131899952	-4.4284996	9.49E-06	3.18E-05
ENSG00000099385	BCL7C	806.2406152	967.23115	645.250082	-0.584319327	0.092977175	-6.2845459	3.29E-10	1.58E-09
ENSG00000143621	ILF2	5215.947965	6258.01	4173.885959	-0.584563427	0.048837404	-11.969584	5.13E-33	5.94E-32
ENSG00000198740	ZNF652	401.9363228	482.49999	321.3726509	-0.584568951	0.137319637	-4.2569946	2.07E-05	6.73E-05
ENSG00000168918	INPP5D	2323.366044	2787.9352	1858.796925	-0.584681629	0.055388819	-10.55595	4.77E-26	4.59E-25
ENSG00000170891	CYTL1	4996.854256	5995.9623	3997.746175	-0.584740345	0.046606326	-12.546373	4.16E-36	5.21E-35
ENSG00000129351	ILF3	8487.9476	10185.303	6790.592646	-0.584943934	0.04133746	-14.150457	1.86E-45	2.86E-44
ENSG00000070501	POLB	306.4079286	367.7532	245.0626529	-0.585167419	0.131454194	-4.4514929	8.53E-06	2.88E-05
ENSG00000100147	CCDC134	77.19359018	92.629497	61.75768379	-0.585194624	0.249126812	-2.3489829	0.018825	0.039914
ENSG00000198816	ZNF358	951.4586215	1141.5239	761.3933362	-0.585749072	0.090886011	-6.444876	1.16E-10	5.76E-10
ENSG00000129657	SEC14L1	23156.82835	27795.652	18518.00439	-0.585906615	0.034365468	-17.049284	3.54E-65	7.57E-64

ENSG00000232573	RPL3P4	112.5236254	135.12711	89.92014186	-0.585911187	0.214741416	-2.7284499	0.006363	0.014743
ENSG00000132781	MUTYH	134.2055123	161.11399	107.2970359	-0.586008715	0.2079581	-2.8179172	0.004834	0.011433
ENSG00000124098	FAM210B	4165.569529	5000.9739	3330.165151	-0.586609916	0.049477968	-11.855982	2.00E-32	2.29E-31
ENSG00000149547	EI24	4935.210168	5925.8125	3944.607871	-0.587415709	0.045307491	-12.96509	1.93E-38	2.55E-37
ENSG00000106348	IMPDH1	2821.534537	3388.2341	2254.835015	-0.587690979	0.070302964	-8.3594055	6.30E-17	4.34E-16
ENSG00000148459	PDSS1	97.68364588	117.22247	78.14482038	-0.587726451	0.236339735	-2.4867864	0.01289	0.028302
ENSG00000151746	BICD1	805.6017524	967.55213	643.6513747	-0.587929742	0.092099634	-6.3836273	1.73E-10	8.50E-10
ENSG00000138771	SHROOM3	432.6591723	519.57247	345.7458785	-0.587939549	0.111186814	-5.2878532	1.24E-07	4.93E-07
ENSG00000146540	C7orf50	1457.24102	1750.4182	1164.063815	-0.588287988	0.073962877	-7.9538278	1.81E-15	1.16E-14
ENSG00000196756	SNHG17	433.9084356	520.99366	346.8232131	-0.588664026	0.126108505	-4.6679169	3.04E-06	1.07E-05
ENSG00000117395	EBNA1BP2	2335.907078	2805.9318	1865.882393	-0.588705729	0.064238979	-9.164307	4.99E-20	3.91E-19
ENSG00000118564	FBXL5	2366.399968	2842.696	1890.103904	-0.5889183	0.058797628	-10.016021	1.30E-23	1.15E-22
ENSG00000119335	SET	8993.406558	10804.066	7182.747054	-0.588993193	0.039736892	-14.822327	1.05E-49	1.75E-48
ENSG00000183688	RFLNB	1866.870549	2243.2614	1490.479723	-0.590026518	0.059262682	-9.9561225	2.37E-23	2.09E-22
ENSG00000116750	UCHL5	877.6086371	1054.7317	700.4855838	-0.590031559	0.088842602	-6.6413134	3.11E-11	1.61E-10
ENSG00000184428	TOP1MT	556.8525048	669.06321	444.6418032	-0.590088606	0.099880622	-5.9079389	3.46E-09	1.56E-08
ENSG00000163743	RCHY1	551.1892551	662.4072	439.9713063	-0.590288158	0.103253526	-5.7168813	1.08E-08	4.68E-08
ENSG00000171858	RPS21	3760.573236	4519.3261	3001.820345	-0.590546954	0.050094638	-11.788626	4.47E-32	5.06E-31
ENSG00000115364	MRPL19	1269.927545	1526.5715	1013.283611	-0.590580091	0.075156756	-7.8579774	3.90E-15	2.47E-14
ENSG00000126351	THRA	1653.847479	1987.7116	1319.983312	-0.590995424	0.071596596	-8.2545185	1.53E-16	1.03E-15
ENSG00000175581	MRPL48	354.3908727	426.31069	282.4710524	-0.591527792	0.134659184	-4.3927772	1.12E-05	3.73E-05
ENSG00000162885	B3GALNT2	1107.825515	1332.196	883.4550533	-0.591614971	0.086235744	-6.8604379	6.86E-12	3.69E-11
ENSG00000145912	NHP2	2023.803988	2432.5671	1615.040838	-0.59161556	0.067677562	-8.7416795	2.30E-18	1.68E-17
ENSG00000145335	SNCA	2240.884645	2694.4654	1787.303917	-0.591948173	0.058134352	-10.182416	2.38E-24	2.15E-23
ENSG00000155906	RMND1	469.9154397	565.08876	374.7421192	-0.592535068	0.10901128	-5.435539	5.46E-08	2.24E-07
ENSG00000122863	CHST3	857.5948899	1031.267	683.9227493	-0.592840045	0.101557961	-5.8374552	5.30E-09	2.34E-08
ENSG00000165996	HACD1	327.0214971	393.15365	260.8893454	-0.593288016	0.13915724	-4.2634362	2.01E-05	6.54E-05
ENSG00000133216	EPHB2	5131.568195	6171.8888	4091.247626	-0.59342012	0.04814836	-12.324825	6.66E-35	8.08E-34
ENSG00000188559	RALGAPA2	3079.713963	3704.458	2454.969902	-0.593438499	0.050109155	-11.842916	2.34E-32	2.67E-31
ENSG00000130475	FCHO1	103.0378553	123.89314	82.18257338	-0.593462237	0.254873046	-2.3284621	0.019888	0.041898
ENSG00000092820	EZR	4557.915931	5483.0695	3632.762385	-0.593576544	0.047797745	-12.418505	2.07E-35	2.55E-34
ENSG00000113758	DBN1	11833.19184	14234.545	9431.838507	-0.593905727	0.051833876	-11.457868	2.15E-30	2.32E-29
ENSG00000156521	TYSND1	227.6161228	273.73429	181.4979545	-0.593991919	0.161538662	-3.6770883	0.000236	0.000677
ENSG00000189159	JPT1	4982.067617	5993.0391	3971.09617	-0.594022676	0.053915459	-11.017669	3.14E-28	3.22E-27
ENSG00000162836	ACP6	195.3044759	234.84022	155.7687367	-0.594385479	0.165209879	-3.5977599	0.000321	0.000905
ENSG00000074800	ENO1	39077.53704	47019.792	31135.2822	-0.594760575	0.046827663	-12.701052	5.83E-37	7.47E-36
ENSG00000170035	UBE2E3	1637.687802	1970.7482	1304.627367	-0.595802269	0.065265875	-9.1288482	6.92E-20	5.39E-19
ENSG00000174749	FAM241A	1540.993239	1855.1916	1226.794834	-0.596284136	0.066986989	-8.9014918	5.51E-19	4.13E-18
ENSG00000147394	ZNF185	3152.159445	3794.277	2510.04186	-0.596409833	0.058152574	-10.255949	1.11E-24	1.02E-23
ENSG00000104983	CCDC61	150.3866191	180.98995	119.7832901	-0.596496638	0.2047717	-2.9129838	0.00358	0.008661
ENSG00000143537	ADAM15	15171.42038	18266.179	12076.66216	-0.597036105	0.048863686	-12.218401	2.48E-34	2.96E-33
ENSG00000173894	CBX2	436.7397772	525.65393	347.8256221	-0.597284983	0.114168775	-5.2315966	1.68E-07	6.61E-07
ENSG00000116221	MRPL37	1987.335285	2392.7765	1581.894038	-0.597400367	0.069564989	-8.5876585	8.88E-18	6.31E-17
ENSG00000131389	SLC6A6	2341.39708	2819.2045	1863.589662	-0.597450568	0.055316075	-10.800668	3.42E-27	3.40E-26
ENSG00000133943	DGLUCY	1175.843424	1416.1384	935.5484559	-0.597790323	0.072488408	-8.2467024	1.63E-16	1.10E-15
ENSG00000152234	ATP5F1A	10184.00976	12263.896	8104.123488	-0.597817788	0.044120026	-13.549806	7.94E-42	1.14E-40
ENSG00000135048	CEMIP2	4350.905558	5240.3849	3461.426256	-0.598150964	0.050772107	-11.781094	4.89E-32	5.52E-31
ENSG00000162910	MRPL55	421.5053765	507.80215	335.2086024	-0.598216395	0.113933147	-5.2505913	1.52E-07	5.99E-07
ENSG00000174780	SRP72	4289.705932	5166.5296	3412.882275	-0.598450413	0.046153933	-12.966401	1.90E-38	2.51E-37
ENSG00000116560	SFPQ	5571.572208	6711.6618	4431.482622	-0.598931275	0.042710611	-14.023009	1.13E-44	1.71E-43
ENSG00000139132	FGD4	1074.394408	1294.3846	854.4042489	-0.598949172	0.07352712	-8.1459627	3.76E-16	2.50E-15
ENSG00000089157	RPLP0	42498.04677	51196.282	33799.81192	-0.599068826	0.043895145	-13.647724	2.08E-42	3.03E-41
ENSG00000181409	AATK	125.7278863	151.57948	99.87629388	-0.599321501	0.2082341	-2.8781141	0.004001	0.009607
ENSG00000108479	GALK1	736.8680735	887.51033	586.2258183	-0.599534925	0.099543105	-6.0228674	1.71E-09	7.86E-09
ENSG00000120253	NUP43	908.8302933	1095.2103	722.450312	-0.599729928	0.080732128	-7.4286401	1.10E-13	6.44E-13
ENSG00000065413	ANKRD44	102.8599624	124.04348	81.67644044	-0.600334379	0.231719612	-2.5907793	0.009576	0.021508
ENSG00000166341	DCHS1	8913.155572	10743.14	7083.171504	-0.601029083	0.050306536	-11.947336	6.70E-33	7.75E-32
ENSG00000079156	OSBPL6	119.2810984	143.94574	94.61645754	-0.601111428	0.242314341	-2.4807093	0.013112	0.028736
ENSG00000004487	KDM1A	3233.756685	3898.0204	2569.49297	-0.60111421	0.056559121	-10.628068	2.21E-26	2.15E-25
ENSG00000104231	ZFAND1	453.0458997	546.27355	359.8182542	-0.601299712	0.113592372	-5.2934867	1.20E-07	4.79E-07
ENSG00000168899	VAMP5	3060.125123	3688.6893	2431.560968	-0.601331971	0.061123207	-9.8380305	7.72E-23	6.67E-22
ENSG00000137513	NARS2	309.3112335	372.81078	245.8116908	-0.601976363	0.13296389	-4.5273673	5.97E-06	2.05E-05
ENSG00000123064	DDX54	2286.061869	2755.7955	1816.328237	-0.602104972	0.076086177	-7.9134607	2.50E-15	1.60E-14
ENSG00000092208	GEMIN2	135.9026269	163.82489	107.9803675	-0.602265302	0.20209142	-2.9801627	0.002881	0.007079
ENSG00000104823	ECH1	598.7014564	721.95509	475.4478251	-0.602441455	0.097073107	-6.2060593	5.43E-10	2.58E-09
ENSG00000170537	TMC7	394.1324211	475.13422	313.1306192	-0.602635848	0.129717797	-4.6457453	3.39E-06	1.19E-05
ENSG00000119333	WDR34	834.1713898	1005.7913	662.5514692	-0.602903558	0.090715688	-6.6460782	3.01E-11	1.56E-10
ENSG00000042317	SPATA7	271.9837557	327.96383	216.0036792	-0.602970526	0.13755029	-4.3836369	1.17E-05	3.89E-05
ENSG00000130119	GNL3L	1658.915009	2000.6673	1317.162671	-0.603180608	0.061842896	-9.7534341	1.78E-22	1.52E-21
ENSG00000266208	AC080112.1	203.0119073	244.80481	161.2190045	-0.603363593	0.179097662	-3.3689083	0.000755	0.002023
ENSG00000167112	TRUB2	1271.295432	1533.272	1009.318833	-0.603804622	0.075917683	-7.9540408	1.81E-15	1.16E-14
ENSG00000100347	SAMM50	1093.79449	1319.2407	868.3482509	-0.603935084	0.078518517	-7.6916262	1.45E-14	8.94E-14
ENSG00000181924	COA4	1296.605045	1564.1859	1029.024236	-0.604037171	0.070643716	-8.5504728	1.23E-17	8.69E-17
ENSG00000185347	TEDC1	105.4172938	127.10386	83.73073055	-0.604369021	0.22370448	-2.7016402	0.0069	0.015873
ENSG00000136938	ANP32B	3322.994205	4009.1712	2636.817189	-0.604539629	0.048410084	-12.487886	8.69E-36	1.08E-34
ENSG00000113648	H2AFY	2608.01769	3146.5026	2069.532731	-0.604615851	0.05284525	-11.441253	2.60E-30	2.81E-29
ENSG00000223773	CD99P1	468.6730843	565.4201	371.9260645	-0.604657934	0.117921976	-5.1276102	2.93E-07	1.14E-06
ENSG00000080298	RFX3	325.1039167	392.16473	258.0431013	-0.604885803	0.127902972	-4.7292553	2.25E-06	8.06E-06
ENSG00000198455	ZXDB	537.1535411	647.96178	426.3453038	-0.605096646	0.101820159	-5.9427981	2.80E-09	1.27E-08
ENSG00000132196	HSD17B7	217.2159448	262.22185	172.2100436	-0.605571696	0.161554897	-3.7483958	0.000178	0.000517
ENSG00000177084	POLE	820.8848819	990.91975	650.850013	-0.605776726	0.087258171	-6.9423496	3.86E-12	2.11E-11
ENSG00000130726	TRIM28	5887.786734	7105.4915	4670.082001	-0.605848645	0.051783555	-11.699634	1.28E-31	1.43E-30
ENSG00000114107	CEP70	224.6691298	271.19003	178.1482314	-0.605961129	0.153013429	-3.9601827	7.49E-05	0.000228
ENSG00000148835	TAF5	154.2820538	186.39859	122.1655201	-0.606272131	0.206956437	-2.9294674	0.003395	0.008238
ENSG00000145439	CBR4	527.4449768	636.77089	418.119065	-0.606368212	0.10435371	-5.810701	6.22E-09	2.74E-08

ENSG00000088035	ALG6	291.1901061	351.60247	230.7777402	-0.606624807	0.13386413	-4.5316457	5.85E-06	2.01E-05
ENSG00000169239	CA5B	171.7058629	207.08666	136.3250699	-0.606871649	0.193001935	-3.1443812	0.001664	0.004236
ENSG00000074582	BCS1L	376.7586021	454.86281	298.6543931	-0.607025795	0.130427874	-4.6541109	3.25E-06	1.15E-05
ENSG00000167264	DUS2	273.6908757	330.46112	216.9206307	-0.607564708	0.134741968	-4.5090978	6.51E-06	2.22E-05
ENSG00000078124	ACER3	1069.59679	1291.5943	847.5992943	-0.607628426	0.075396987	-8.0590545	7.69E-16	5.03E-15
ENSG00000156508	EEF1A1	287631.9285	347335.66	227928.197	-0.607752105	0.033086889	-18.368367	2.35E-75	5.83E-74
ENSG00000078687	TNRC6C	614.1008609	741.6476	486.5541176	-0.607831949	0.102962319	-5.9034407	3.56E-09	1.60E-08
ENSG00000102743	SLC25A15	351.0462548	423.89703	278.1954779	-0.608328304	0.12796912	-4.7537117	2.00E-06	7.17E-06
ENSG00000005156	LIG3	611.089139	738.0165	484.1617773	-0.608372006	0.093493086	-6.5071336	7.66E-11	3.86E-10
ENSG00000144959	NCEH1	1986.895126	2399.4734	1574.316853	-0.608430988	0.068498241	-8.8824324	6.54E-19	4.90E-18
ENSG00000188846	RPL14	17050.73626	20598.295	13503.17742	-0.609189133	0.036898489	-16.509867	3.12E-61	6.21E-60
ENSG00000164659	KIAA1324L	2422.491406	2926.9673	1918.015541	-0.609313364	0.071898258	-8.474661	2.36E-17	1.65E-16
ENSG00000108984	MAP2K6	184.9650285	223.65399	146.2760634	-0.610342237	0.197840972	-3.0850144	0.002035	0.005112
ENSG00000113048	MRPS27	1589.745673	1921.514	1257.977311	-0.611513851	0.065671089	-9.3117666	1.26E-20	1.00E-19
ENSG00000165804	ZNF219	636.1802748	768.7288	503.6317511	-0.611577493	0.103014422	-5.9368143	2.91E-09	1.31E-08
ENSG00000169100	SLC25A6	26461.00543	31987.025	20934.98561	-0.611676505	0.046001261	-13.296951	2.41E-40	3.33E-39
ENSG00000182004	SNRPE	900.7606747	1088.9574	712.5639927	-0.611738696	0.079135572	-7.7302619	1.07E-14	6.65E-14
ENSG00000103018	CYB5B	3395.662769	4105.1466	2686.178962	-0.611887741	0.049531547	-12.353495	4.66E-35	5.68E-34
ENSG00000117592	PRDX6	5406.998169	6537.1485	4276.847791	-0.612241146	0.053081627	-11.533956	8.90E-31	9.71E-30
ENSG00000198042	MAK16	664.2787704	803.32025	525.2372888	-0.612576633	0.1036909	-5.9077183	3.47E-09	1.56E-08
ENSG00000177733	HNRNPA0	4617.995099	5584.0446	3651.945601	-0.612785327	0.046768021	-13.102657	3.18E-39	4.29E-38
ENSG00000262814	MRPL12	172.0441567	208.0754	136.0129136	-0.613777765	0.181051933	-3.3900647	0.000699	0.001882
ENSG00000100304	TTLL12	1347.862922	1630.4531	1065.272762	-0.615075757	0.085412596	-7.2012301	5.97E-13	3.39E-12
ENSG00000198589	LRBA	2531.94728	3063.476	2000.418595	-0.615235998	0.064719442	-9.5062006	1.98E-21	1.63E-20
ENSG00000104915	STX10	859.2360288	1039.8681	678.6039786	-0.615378762	0.084951577	-7.2438768	4.36E-13	2.49E-12
ENSG00000165506	DNAAF2	244.9685202	296.60745	193.3295941	-0.6154214	0.153406741	-4.0116972	6.03E-05	0.000186
ENSG00000176225	RTTN	266.8694178	323.06221	210.6766264	-0.615621702	0.162289554	-3.7933538	0.000149	0.000437
ENSG00000234546	LNCTAM34A	119.363807	144.53388	94.1937377	-0.615655348	0.207986907	-2.9600678	0.003076	0.007517
ENSG00000087157	PGS1	776.3240869	939.59404	613.0541382	-0.615821327	0.094912525	-6.4883041	8.68E-11	4.36E-10
ENSG00000142541	RPL13A	36890.80626	44651.727	29129.8854	-0.616251246	0.037666826	-16.360583	3.66E-60	7.18E-59
ENSG00000186468	RPS23	13696.62456	16578.684	10814.56522	-0.616334369	0.043913022	-14.035344	9.47E-45	1.44E-43
ENSG00000129158	SERGEF	159.3614155	192.90622	125.8166139	-0.616493942	0.194467371	-3.1701665	0.001524	0.003904
ENSG00000119471	HSDL2	985.2401346	1192.4193	778.0609193	-0.616567944	0.088531093	-6.9644226	3.30E-12	1.81E-11
ENSG00000112367	FIG4	555.9655655	673.22557	438.7055565	-0.617025984	0.105915357	-5.8256517	5.69E-09	2.51E-08
ENSG00000128656	CHN1	536.4141194	649.72977	423.0984726	-0.617558205	0.113145986	-5.4580655	4.81E-08	1.99E-07
ENSG00000085511	MAP3K4	710.0244645	859.58695	560.4619747	-0.617666933	0.092170422	-6.7013573	2.06E-11	1.08E-10
ENSG00000064115	TM7SF3	2042.859732	2473.7267	1611.992776	-0.617883643	0.064319191	-9.6065208	7.50E-22	6.25E-21
ENSG00000138092	CENPO	620.9373974	751.62933	490.2454642	-0.617938465	0.108323393	-5.7045708	1.17E-08	5.02E-08
ENSG00000085415	SEH1L	562.6492109	681.26721	444.0312119	-0.618005541	0.100502592	-6.1491503	7.79E-10	3.66E-09
ENSG00000123416	TUBA1B	24485.93664	29651.625	19320.24876	-0.618094931	0.055914151	-11.054356	2.09E-28	2.15E-27
ENSG00000204628	RACK1	27627.12699	33457.988	21796.30646	-0.618346686	0.044213176	-13.985575	1.91E-44	2.88E-43
ENSG00000114686	MRPL3	1353.048807	1638.6584	1067.439228	-0.618411131	0.080360914	-7.6954218	1.41E-14	8.69E-14
ENSG00000111252	SH2B3	9692.075168	11739.236	7644.914786	-0.618844263	0.040643981	-15.225976	2.38E-52	4.14E-51
ENSG00000170089	AC106795.1	304.5291264	368.75475	240.3035009	-0.6190942	0.144584443	-4.2818867	1.85E-05	6.04E-05
ENSG00000114948	ADAM23	1714.712538	2077.1008	1352.324229	-0.619426835	0.06457078	-9.5929898	8.56E-22	7.11E-21
ENSG00000158850	B4GALT3	1172.312621	1419.8319	924.7933535	-0.619453758	0.079362104	-7.80541	5.93E-15	3.71E-14
ENSG00000235194	PPP1R3E	76.29912346	92.398474	60.19977309	-0.619543127	0.255763536	-2.4223278	0.015421	0.033276
ENSG00000160813	PPP1R35	359.3418393	435.14702	283.5366548	-0.619818546	0.132949182	-4.6620711	3.13E-06	1.10E-05
ENSG00000160124	CCDC58	252.2048272	305.61263	198.797025	-0.620173326	0.145036588	-4.2759785	1.90E-05	6.20E-05
ENSG00000130935	NOL11	1006.166054	1219.2117	793.1204361	-0.620520214	0.081704051	-7.5947301	3.08E-14	1.86E-13
ENSG00000163913	IFT122	627.8847768	761.20847	494.561085	-0.620645412	0.099279103	-6.2515212	4.06E-10	1.95E-09
ENSG00000167083	GNGT2	101.6483436	123.32294	79.97374337	-0.620706415	0.240069975	-2.5855229	0.009723	0.021805
ENSG00000260314	MRC1	747.9432962	906.60722	589.2793752	-0.620757069	0.096200249	-6.4527595	1.10E-10	5.47E-10
ENSG00000115875	SRSF7	1778.162987	2154.951	1401.374944	-0.620929478	0.060453102	-10.271259	9.50E-25	8.72E-24
ENSG00000130304	SLC27A1	519.138474	629.26552	409.0114291	-0.621416863	0.102670833	-6.052516	1.43E-09	6.57E-09
ENSG00000113460	BRIX1	507.8963337	616.00634	399.7863264	-0.621981265	0.111378966	-5.5843692	2.35E-08	9.91E-08
ENSG00000147155	EBP	232.9115886	282.42231	183.4008648	-0.622076486	0.158915497	-3.9145112	9.06E-05	0.000274
ENSG00000106853	PTGR1	2565.292775	3110.2751	2020.310482	-0.622551646	0.053395855	-11.659176	2.06E-31	2.28E-30
ENSG00000143457	GOLPH3L	1156.62067	1402.4762	910.7651881	-0.622796544	0.071968601	-8.6537258	4.98E-18	3.58E-17
ENSG00000186665	C17orf58	396.0391637	480.27568	311.8026425	-0.623167026	0.11713232	-5.3201971	1.04E-07	4.16E-07
ENSG00000231500	RPS18	27805.85697	33719.754	21891.96012	-0.623189174	0.035766513	-17.423817	5.44E-68	1.22E-66
ENSG00000222011	FAM185A	66.80167308	80.999253	52.60409291	-0.623475212	0.27081769	-2.3021953	0.021324	0.044624
ENSG00000165912	PACSIN3	347.2953564	421.13965	273.4510594	-0.623712023	0.120833561	-5.161745	2.45E-07	9.51E-07
ENSG00000198242	RPL23A	13470.35778	16337.76	10602.95536	-0.623788784	0.039225522	-15.902626	6.08E-57	1.13E-55
ENSG00000278845	MRPL45	1006.299953	1220.6729	791.9269944	-0.623887614	0.081327073	-7.6713399	1.70E-14	1.04E-13
ENSG00000112130	RNF8	779.8487184	946.04608	613.6513558	-0.624563283	0.089280204	-6.9955405	2.64E-12	1.46E-11
ENSG00000103024	NME3	699.2205089	848.24663	550.1943886	-0.624665487	0.09945004	-6.2811989	3.36E-10	1.62E-09
ENSG00000109265	KIAA1211	416.2322432	505.08078	327.3837104	-0.624995544	0.122682491	-5.0944152	3.50E-07	1.35E-06
ENSG00000134748	PRPF38A	739.1260332	896.65494	581.5971218	-0.624996276	0.087142353	-7.1721299	7.38E-13	4.18E-12
ENSG00000134243	SORT1	1645.538556	1996.6798	1294.397267	-0.625068866	0.093887578	-6.6576312	2.78E-11	1.44E-10
ENSG00000066056	TIE1	26096.28152	31665.099	20527.46431	-0.625365912	0.035644001	-17.544773	6.52E-69	1.48E-67
ENSG00000130511	SSBP4	1728.297676	2096.9841	1359.611252	-0.625981424	0.090296724	-6.9324932	4.13E-12	2.26E-11
ENSG00000142937	RPS8	21674.29491	26305.907	17042.68306	-0.626258425	0.035441589	-17.670156	7.12E-70	1.63E-68
ENSG00000040341	STAU2	1398.798029	1697.9405	1099.655533	-0.626321952	0.076442331	-8.1933916	2.54E-16	1.70E-15
ENSG00000107872	FBXL15	227.9032589	276.56181	179.2447077	-0.62702847	0.187634898	-3.3417476	0.000833	0.002219
ENSG00000132773	TOE1	250.5884225	303.98502	197.1918276	-0.627097351	0.160792612	-3.9000383	9.62E-05	0.00029
ENSG00000102265	TIMP1	2143.253337	2601.8777	1684.628975	-0.627657654	0.075757754	-8.2850616	1.18E-16	8.04E-16
ENSG00000187325	TAF9B	499.9759079	607.10021	392.8516008	-0.627757051	0.10384985	-6.0448528	1.50E-09	6.88E-09
ENSG00000144736	SHQ1	542.3141862	658.44847	426.1799031	-0.62789879	0.108729701	-5.7748599	7.70E-09	3.36E-08
ENSG00000186283	TOR3A	655.2680967	795.68132	514.8548719	-0.628009769	0.091832241	-6.8386632	7.99E-12	4.28E-11
ENSG00000183431	SF3A3	2086.014719	2533.0796	1638.949789	-0.628023106	0.05704249	-11.009742	3.43E-28	3.51E-27
ENSG00000125351	UPF3B	448.0187863	544.2646	351.7729751	-0.628203551	0.123000001	-5.1073459	3.27E-07	1.26E-06
ENSG00000173113	TRMT112	2112.853826	2565.6846	1660.023019	-0.628500146	0.060041255	-10.467805	1.21E-25	1.15E-24
ENSG00000146263	MMS22L	321.5748573	390.52378	252.6259303	-0.628558226	0.138125485	-4.5506318	5.35E-06	1.84E-05
ENSG00000050405	LIMA1	4008.00837	4868.7194	3147.297382	-0.629494017	0.048777769	-12.905347	4.20E-38	5.50E-37

ENSG00000144867	SRPRB	1582.158513	1921.7823	1242.534765	-0.629707163	0.070580143	-8.9218743	4.58E-19	3.45E-18
ENSG00000244313	AC024293.1	86.14661613	104.75631	67.53692356	-0.629976806	0.266518158	-2.3637294	0.018092	0.038535
ENSG00000135617	PRADC1	397.5357228	482.74113	312.3303185	-0.630358411	0.149411345	-4.2189461	2.45E-05	7.90E-05
ENSG00000071073	MGAT4A	3639.69928	4422.7157	2856.682866	-0.630377437	0.054817944	-11.499472	1.33E-30	1.44E-29
ENSG00000172766	NAA16	329.1436236	399.8381	258.4491464	-0.631290436	0.141637018	-4.4571006	8.31E-06	2.81E-05
ENSG00000253731	PCDHGA6	122.621041	149.00889	96.23319092	-0.631461299	0.236851565	-2.6660634	0.007675	0.017539
ENSG00000237854	LINC00674	173.4282929	210.66139	136.1951956	-0.631936055	0.200662609	-3.1492467	0.001637	0.004172
ENSG00000143434	SEMA6C	2111.97777	2567.1856	1656.769933	-0.632047277	0.072653701	-8.6994505	3.33E-18	2.42E-17
ENSG00000204922	UQCC3	312.8351797	380.04346	245.6268958	-0.632092678	0.149570563	-4.22605	2.38E-05	7.67E-05
ENSG00000160447	PKN3	997.6941396	1212.6914	782.6968657	-0.632149942	0.077695692	-8.136229	4.08E-16	2.70E-15
ENSG00000166289	PLEKHF1	232.6158441	282.70748	182.5242037	-0.632238573	0.157390543	-4.0170048	5.89E-05	0.000182
ENSG00000158169	FANCC	301.4867955	366.41507	236.5585182	-0.632553386	0.138509282	-4.5668664	4.95E-06	1.71E-05
ENSG00000230438	SERPINB9P1	332.7247782	404.54408	260.9054763	-0.632650223	0.133217036	-4.7490189	2.04E-06	7.33E-06
ENSG00000112320	SOBP	359.1588787	436.55804	281.7597168	-0.632898956	0.127742606	-4.954486	7.25E-07	2.71E-06
ENSG00000175061	SNHG29	6097.692053	7414.5572	4780.826952	-0.632948756	0.048048227	-13.173197	1.25E-39	1.70E-38
ENSG00000151692	RNF144A	148.0072556	179.97907	116.0354376	-0.632989466	0.220450078	-2.8713506	0.004087	0.009805
ENSG00000131019	ULBP3	369.7335049	449.53998	289.9270296	-0.633360114	0.124902998	-5.0708159	3.96E-07	1.52E-06
ENSG00000162408	NOL9	776.9675383	945.03322	608.9018539	-0.633370947	0.091651441	-6.910649	4.82E-12	2.62E-11
ENSG00000130349	C6orf203	273.9786919	333.40313	214.55425	-0.6335099	0.145760697	-4.3462326	1.38E-05	4.58E-05
ENSG00000138439	FAM117B	177.6655876	216.05838	139.2727992	-0.633531818	0.192546149	-3.2902856	0.001001	0.00264
ENSG00000119772	DNMT3A	1093.099498	1329.6433	856.5557288	-0.633609006	0.075937914	-8.3437768	7.20E-17	4.94E-16
ENSG00000174177	CTU2	446.79068	543.17917	350.4021856	-0.633615352	0.113808135	-5.5673995	2.59E-08	1.09E-07
ENSG00000124444	ZNF576	226.5476312	275.40227	177.692996	-0.633656913	0.152796996	-4.1470509	3.37E-05	0.000107
ENSG00000104731	KLHDC4	618.0975226	751.72562	484.4694221	-0.633832398	0.095988185	-6.6032335	4.02E-11	2.07E-10
ENSG00000149084	HSD17B12	2766.299418	3364.2651	2168.333754	-0.63388539	0.061217999	-10.354559	3.99E-25	3.73E-24
ENSG00000115942	ORC2	644.6136673	784.25466	504.972679	-0.634397546	0.094879102	-6.6863781	2.29E-11	1.20E-10
ENSG00000101158	NELFCD	1626.577466	1978.4892	1274.665762	-0.634404627	0.071649125	-8.8543248	8.42E-19	6.28E-18
ENSG00000135093	USP30	320.4015649	389.90484	250.8982859	-0.634528231	0.141001116	-4.5001646	6.79E-06	2.32E-05
ENSG00000075336	TIMM21	347.7991193	423.20408	272.3941607	-0.634670586	0.124406683	-5.1015795	3.37E-07	1.30E-06
ENSG00000153208	MERTK	1419.542335	1727.3859	1111.698818	-0.634970712	0.075868091	-8.3694041	5.79E-17	4.00E-16
ENSG00000187522	HSPA14	241.123336	293.32263	188.9240419	-0.634973478	0.14622142	-4.3425476	1.41E-05	4.65E-05
ENSG00000235552	RPL6P27	142.9316513	173.98642	111.8768792	-0.635083299	0.214597466	-2.9594166	0.003082	0.007528
ENSG00000115042	FAHD2A	409.9801966	498.68501	321.275386	-0.635114583	0.113284762	-5.6063549	2.07E-08	8.76E-08
ENSG00000261468	AC096921.2	394.9013587	480.49046	309.3122587	-0.63528972	0.135195049	-4.6990605	2.61E-06	9.29E-06
ENSG00000135723	FHOD1	2425.792247	2951.7763	1899.808166	-0.635945349	0.060733487	-10.471082	1.17E-25	1.11E-24
ENSG00000184216	IRAK1	5290.938488	6438.536	4143.34099	-0.636179194	0.048397174	-13.144966	1.82E-39	2.47E-38
ENSG00000099256	PRTFDC1	210.8503965	256.5908	165.1099924	-0.63635164	0.153431399	-4.1474669	3.36E-05	0.000107
ENSG00000105607	GCDH	433.0109012	526.96229	339.0595159	-0.636564163	0.113541078	-5.6064657	2.06E-08	8.76E-08
ENSG00000278771	RN7SL3	107.2258381	130.50281	83.94886659	-0.636749157	0.218593369	-2.912939	0.00358	0.008661
ENSG00000143493	INTS7	490.0995486	596.66631	383.5327877	-0.638113244	0.107090727	-5.9586228	2.54E-09	1.15E-08
ENSG00000176531	PHLDB3	185.1296522	225.23111	145.0281989	-0.63812593	0.188442881	-3.3863096	0.000708	0.001907
ENSG00000101849	TBL1X	1258.983346	1532.7563	985.210409	-0.638140937	0.070500994	-9.0515168	1.41E-19	1.08E-18
ENSG00000184232	OAF	1763.491677	2147.0098	1379.973558	-0.638176967	0.069137898	-9.2304941	2.69E-20	2.13E-19
ENSG00000138050	THUMPD2	158.9646974	193.64957	124.2798236	-0.638249828	0.183506228	-3.4780827	0.000505	0.001388
ENSG00000138777	PPA2	903.288678	1100.0334	706.5439582	-0.63873014	0.083468674	-7.6523337	1.97E-14	1.21E-13
ENSG00000091436	MAP3K20	1316.225292	1602.9937	1029.456879	-0.638892996	0.072547714	-8.8065214	1.29E-18	9.54E-18
ENSG00000041802	LSG1	1917.843679	2335.9088	1499.778578	-0.639052497	0.059365779	-10.764661	5.05E-27	4.99E-26
ENSG00000108963	DPH1	319.8788166	389.64564	250.1119966	-0.639129921	0.12600315	-5.0723329	3.93E-07	1.51E-06
ENSG00000137822	TUBGCP4	496.4309034	604.91307	387.9487404	-0.639132858	0.1167174	-5.4759004	4.35E-08	1.80E-07
ENSG00000106355	LSM5	341.7683777	416.38469	267.1520688	-0.639150831	0.132911072	-4.8088607	1.52E-06	5.52E-06
ENSG00000131080	EDA2R	344.7763814	419.95108	269.6016841	-0.63955075	0.134946652	-4.7392858	2.14E-06	7.69E-06
ENSG00000100350	FOXRED2	626.0431434	762.52845	489.5578372	-0.63961011	0.095797014	-6.6767228	2.44E-11	1.27E-10
ENSG00000065183	WDR3	1213.590717	1478.5101	948.6713363	-0.639924383	0.074384518	-8.6029244	7.77E-18	5.55E-17
ENSG00000166025	AMOTL1	4975.912072	6063.0631	3888.761073	-0.640760159	0.043908494	-14.59308	3.11E-48	5.05E-47
ENSG00000181274	FRAT2	258.8010495	315.18721	202.4148905	-0.640821271	0.145485524	-4.4047081	1.06E-05	3.54E-05
ENSG00000030066	NUP160	1064.587897	1297.1256	832.0501701	-0.640877751	0.073694543	-8.6964072	3.43E-18	2.48E-17
ENSG00000105889	STEAP1B	1178.122143	1435.7984	920.4458915	-0.641395638	0.080901796	-7.9280766	2.23E-15	1.42E-14
ENSG00000092036	HAUS4	188.5944122	230.01051	147.1783189	-0.642206991	0.179979614	-3.5682207	0.000359	0.001005
ENSG00000026103	FAS	446.583514	544.60896	348.5580688	-0.642534598	0.12103834	-5.3085212	1.11E-07	4.42E-07
ENSG00000169689	CENPX	487.2597484	594.07994	380.4395593	-0.642585327	0.114635998	-5.6054411	2.08E-08	8.80E-08
ENSG00000106268	NUDT1	124.7864564	152.04147	97.53144686	-0.643069307	0.233780628	-2.7507382	0.005946	0.013842
ENSG00000144401	METTL21A	219.8682115	268.15601	171.5804137	-0.64376939	0.158098641	-4.0719477	4.66E-05	0.000146
ENSG00000106004	HOXA5	172.8713313	211.00898	134.7336867	-0.644176095	0.18187371	-3.5418868	0.000397	0.001106
ENSG00000144677	CTDSPL	1933.806515	2358.5722	1509.040848	-0.644415644	0.06519175	-9.8849263	4.84E-23	4.21E-22
ENSG00000138780	GSTCD	289.8926933	353.62219	226.1631961	-0.644720079	0.132196961	-4.876966	1.08E-06	3.97E-06
ENSG00000105373	NOP53	4699.798934	5732.839	3666.758874	-0.644940786	0.046108807	-13.987366	1.86E-44	2.81E-43
ENSG00000124207	CSE1L	3493.020367	4261.2949	2724.745876	-0.645013292	0.060262309	-10.703428	9.81E-27	9.65E-26
ENSG00000116205	TCEANC2	447.6530097	546.27906	349.0269597	-0.64536482	0.114488364	-5.6369468	1.73E-08	7.38E-08
ENSG00000148303	RPL7A	31907.9638	38933.514	24882.41317	-0.64591904	0.038043524	-16.978423	1.19E-64	2.52E-63
ENSG00000172795	DCP2	983.938351	1201.2123	766.6644165	-0.646712663	0.085045008	-7.6043577	2.86E-14	1.73E-13
ENSG00000026025	VIM	190629.6824	232658.79	148600.5716	-0.646785694	0.036329669	-17.803237	6.67E-71	1.56E-69
ENSG00000134333	LDHA	10986.66546	13408.866	8564.465006	-0.646879492	0.061998686	-10.433761	1.74E-25	1.64E-24
ENSG00000176619	LMNB2	3929.069739	4795.4931	3062.646371	-0.647148474	0.059770361	-10.827247	2.56E-27	2.56E-26
ENSG00000183207	RUVBL2	1907.599433	2328.3492	1486.849712	-0.647321675	0.067689659	-9.5630807	1.14E-21	9.45E-21
ENSG00000101665	SMAD7	121.6174463	148.61908	94.6158105	-0.647387891	0.224692443	-2.8812179	0.003961	0.009522
ENSG00000196177	ACADSB	509.6766997	622.42813	396.9252736	-0.648333831	0.110606734	-5.8616127	4.58E-09	2.04E-08
ENSG00000134072	CAMK1	523.697065	639.10027	408.2938569	-0.648446445	0.11831987	-5.4804527	4.24E-08	1.76E-07
ENSG00000138621	PPCDC	149.5362267	182.5421	116.5303539	-0.648741461	0.197181311	-3.2900758	0.001002	0.002641
ENSG00000107949	BCCIP	1392.74698	1700.8937	1084.600255	-0.648848903	0.07448535	-8.7110942	3.01E-18	2.19E-17
ENSG00000003249	DBNDD1	1658.988699	2025.916	1292.061395	-0.649723798	0.074535705	-8.7169471	2.86E-18	2.08E-17
ENSG00000071994	PDCD2	1211.307893	1479.8833	942.7325014	-0.650058769	0.072596456	-8.9544147	3.42E-19	2.58E-18
ENSG00000169288	MRPL1	500.2084027	611.04165	389.375155	-0.650984157	0.115317583	-5.6451422	1.65E-08	7.05E-08
ENSG00000011523	CEP68	1600.106556	1955.4979	1244.715178	-0.651352842	0.06359047	-10.242932	1.27E-24	1.17E-23
ENSG00000122034	GTF3A	1518.10707	1855.0568	1181.157354	-0.651523479	0.072228404	-9.0203222	1.88E-19	1.44E-18
ENSG00000165283	STOML2	2388.366477	2918.9435	1857.789423	-0.651700919	0.062643174	-10.403383	2.39E-25	2.25E-24

ENSG00000198624	CCDC69	683.5241281	835.25279	531.7954657	-0.652101256	0.096544618	-6.754403	1.43E-11	7.58E-11
ENSG00000132436	FIGNL1	457.1461925	558.40295	355.8894335	-0.652127885	0.128526121	-5.0738938	3.90E-07	1.49E-06
ENSG00000095539	SEMA4G	222.9163671	272.27417	173.5585594	-0.652835381	0.171534014	-3.8058655	0.000141	0.000416
ENSG00000243244	STON1	287.5599634	351.71278	223.4071454	-0.652957688	0.162175635	-4.0262379	5.67E-05	0.000175
ENSG00000078668	VDAC3	2156.451601	2636.6512	1676.251966	-0.652996422	0.065649198	-9.9467541	2.61E-23	2.29E-22
ENSG00000176422	SPRYD4	292.0581228	357.12224	226.9940055	-0.653367003	0.131779824	-4.95802	7.12E-07	2.67E-06
ENSG00000168890	TMEM150A	563.6187838	689.20978	438.0277863	-0.65347416	0.119504045	-5.4682179	4.55E-08	1.88E-07
ENSG00000007255	TRAPPC6A	205.1887232	250.81587	159.5615779	-0.653519182	0.167821822	-3.8941252	9.86E-05	0.000296
ENSG00000163682	RPL9	9916.520264	12124.836	7708.204459	-0.653533231	0.044871302	-14.564615	4.72E-48	7.65E-47
ENSG00000170846	AC093323.1	179.0859615	218.9231	139.2488183	-0.653706837	0.170698889	-3.8295905	0.000128	0.000381
ENSG00000141295	SCRN2	615.3617198	752.69844	478.024998	-0.653716148	0.100058217	-6.5333579	6.43E-11	3.26E-10
ENSG00000100726	TELO2	988.6330949	1208.723	768.5432247	-0.654106966	0.087997528	-7.4332426	1.06E-13	6.22E-13
ENSG00000234608	MAPKAPK5-AS	215.3483325	263.34604	167.3506252	-0.654204147	0.162758378	-4.0194806	5.83E-05	0.00018
ENSG00000160211	G6PD	3310.853771	4048.4957	2573.211882	-0.654211477	0.0658104	-9.9408524	2.76E-23	2.43E-22
ENSG00000165671	NSD1	4116.913481	5034.7285	3199.098436	-0.654223971	0.047828207	-13.678622	1.36E-42	1.99E-41
ENSG00000169213	RAB3B	3610.794407	4415.6076	2805.981178	-0.654248844	0.055304532	-11.829932	2.73E-32	3.11E-31
ENSG00000008405	CRY1	616.0500563	753.55472	478.545396	-0.654687864	0.096949534	-6.7528727	1.45E-11	7.66E-11
ENSG00000010292	NCAPD2	2056.302177	2514.9194	1597.684952	-0.654970636	0.059471356	-11.013212	3.30E-28	3.38E-27
ENSG00000106009	BRAT1	933.1303907	1141.3508	724.9099721	-0.65537269	0.083484174	-7.8502626	4.15E-15	2.62E-14
ENSG00000181873	IBA57	347.9553677	425.57908	270.3316553	-0.655753625	0.122715701	-5.3436815	9.11E-08	3.67E-07
ENSG00000174307	PHLDA3	1682.282652	2057.7481	1306.817243	-0.655815934	0.078651689	-8.3382308	7.54E-17	5.17E-16
ENSG00000183963	SMTN	4785.271615	5854.3792	3716.164071	-0.656119259	0.050734084	-12.932514	2.95E-38	3.88E-37
ENSG00000183741	CBX6	3238.773069	3962.3719	2515.174252	-0.656170056	0.053221038	-12.329148	6.31E-35	7.66E-34
ENSG00000134419	RPS15A	1279.200713	1565.6323	992.7691373	-0.656331503	0.077528977	-8.4656283	2.55E-17	1.78E-16
ENSG00000265681	RPL17	161.4212713	197.60544	125.2371031	-0.65710447	0.183740022	-3.576273	0.000349	0.000977
ENSG00000123505	AMD1	1759.122816	2152.9429	1365.302736	-0.657757159	0.067190276	-9.7894696	1.25E-22	1.07E-21
ENSG00000130702	LAMA5	10952.11506	13405.819	8498.410939	-0.657834316	0.055684421	-11.813615	3.32E-32	3.77E-31
ENSG00000186918	ZNF395	130.1738294	159.47171	100.8759457	-0.658531152	0.202485938	-3.2522315	0.001145	0.002995
ENSG00000044446	PHKA2	449.5939891	550.46451	348.7234695	-0.658665846	0.106789154	-6.1679096	6.92E-10	3.26E-09
ENSG00000110315	RNF141	2003.588098	2453.626	1553.550161	-0.659122725	0.059557104	-11.067071	1.81E-28	1.87E-27
ENSG00000175764	TTLL11	207.6779937	254.19416	161.1618234	-0.659321876	0.179183587	-3.6795886	0.000234	0.000671
ENSG00000053372	MRTO4	1247.375566	1527.2837	967.4674325	-0.659435669	0.072235342	-9.1289894	6.91E-20	5.39E-19
ENSG00000171953	ATPAF2	276.7575067	338.91969	214.5953283	-0.659451879	0.135435796	-4.8691107	1.12E-06	4.13E-06
ENSG00000141499	WRAP53	367.3457683	449.75094	284.9405975	-0.659678751	0.12218726	-5.398916	6.70E-08	2.73E-07
ENSG00000099810	MTAP	908.1937247	1112.0274	704.3600004	-0.659768903	0.086328067	-7.642577	2.13E-14	1.30E-13
ENSG00000100105	PATZ1	519.5952318	636.24416	402.9463026	-0.659781353	0.102395671	-6.4434497	1.17E-10	5.80E-10
ENSG00000139343	SNRPF	611.0949775	748.6037	473.5862551	-0.659877327	0.101854403	-6.4786333	9.26E-11	4.63E-10
ENSG00000163050	COQ8A	259.5089524	318.05528	200.962629	-0.660641276	0.144403632	-4.574963	4.76E-06	1.65E-05
ENSG00000109861	CTSC	5392.051621	6605.0875	4179.015735	-0.660726909	0.047156507	-14.011363	1.33E-44	2.01E-43
ENSG00000006062	MAP3K14	171.28161	209.78693	132.7762868	-0.660773704	0.17545906	-3.7659709	0.000166	0.000484
ENSG00000213281	NRAS	3557.266233	4358.637	2755.895472	-0.660976915	0.061262873	-10.789192	3.87E-27	3.84E-26
ENSG00000106617	PRKAG2	1061.167648	1300.5742	821.7611441	-0.661696264	0.075606818	-8.7518068	2.10E-18	1.54E-17
ENSG00000165511	C10orf25	95.70314432	117.2909	74.11538595	-0.661773135	0.23706398	-2.7915381	0.005246	0.012332
ENSG00000168286	THAP11	510.6221226	625.68818	395.5560693	-0.661936038	0.117741763	-5.6219307	1.89E-08	8.03E-08
ENSG00000224861	YBX1P1	66.6536156	81.725374	51.58185748	-0.662590887	0.291853862	-2.2702831	0.02319	0.048085
ENSG00000185838	GNB1L	77.68406158	95.157058	60.21106488	-0.664352982	0.265377794	-2.5034234	0.0123	0.027079
ENSG00000119686	FLVCR2	401.2469723	491.9563	310.537643	-0.66443388	0.126818888	-5.2392344	1.61E-07	6.36E-07
ENSG00000162946	DISC1	378.8166657	464.63623	292.9971026	-0.664897846	0.132563915	-5.0156775	5.28E-07	2.00E-06
ENSG00000118894	EEF2KMT	316.734694	388.46319	245.006194	-0.665325044	0.128624027	-5.1726342	2.31E-07	8.99E-07
ENSG00000136098	NEK3	262.6180737	322.23833	202.9978149	-0.665388463	0.140541017	-4.7344788	2.20E-06	7.86E-06
ENSG00000161010	MRNIP	151.2464377	185.50722	116.985655	-0.665422421	0.180486447	-3.6868276	0.000227	0.000653
ENSG00000249353	NPM1P27	753.9673324	924.8594	583.0752679	-0.665588769	0.086374943	-7.7058085	1.30E-14	8.03E-14
ENSG00000023909	GCLM	1879.481679	2305.6582	1453.305196	-0.666021887	0.074347799	-8.9581924	3.30E-19	2.50E-18
ENSG00000162063	CCNF	749.2485449	918.85518	579.6419072	-0.666098873	0.097290042	-6.8465267	7.57E-12	4.06E-11
ENSG00000220842	RPL21P16	86.53808733	106.12719	66.9489815	-0.666680711	0.252559507	-2.6396975	0.008298	0.018849
ENSG00000096746	HNRNPH3	2460.091446	3018.8698	1901.313097	-0.666805396	0.05942897	-11.220208	3.25E-29	3.42E-28
ENSG00000159259	CHAF1B	229.7162824	281.9225	177.510067	-0.667404608	0.148731427	-4.487314	7.21E-06	2.45E-05
ENSG00000088448	ANKRD10	530.4577235	651.23911	409.6763411	-0.66744635	0.118793985	-5.6185197	1.93E-08	8.19E-08
ENSG00000276045	ORAI1	1044.637379	1282.0568	807.2179083	-0.66759725	0.085349607	-7.8219135	5.20E-15	3.26E-14
ENSG00000147872	PLIN2	1911.874754	2346.3496	1477.399871	-0.667640257	0.06498958	-10.273035	9.32E-25	8.57E-24
ENSG00000205544	TMEM256	102.1625847	125.454	78.87117135	-0.66793452	0.228910212	-2.9178887	0.003524	0.00853
ENSG00000100578	KIAA0586	679.9524608	834.94774	524.9571795	-0.668452453	0.094471371	-7.0757145	1.49E-12	8.29E-12
ENSG00000167815	PRDX2	3475.82126	4267.8555	2683.787044	-0.669682087	0.053779671	-12.452328	1.36E-35	1.68E-34
ENSG00000124006	OBSL1	504.9443406	619.98983	389.8988539	-0.669966206	0.10326578	-6.4877853	8.71E-11	4.37E-10
ENSG00000112576	CCND3	2557.245443	3140.5736	1973.917278	-0.670332925	0.064262671	-10.43114	1.79E-25	1.69E-24
ENSG00000262879	AC068152.1	324.1114029	398.01603	250.2067766	-0.670358588	0.13451564	-4.9835029	6.24E-07	2.35E-06
ENSG00000162607	USP1	852.9716099	1047.7035	658.2397401	-0.670534886	0.082416217	-8.1359581	4.09E-16	2.71E-15
ENSG00000029639	TFB1M	285.7701382	351.00253	220.5377459	-0.670562098	0.133993039	-5.0044547	5.60E-07	2.12E-06
ENSG00000115687	PASK	174.8449303	214.73654	134.9533235	-0.670565486	0.174211793	-3.8491394	0.000119	0.000353
ENSG00000119711	ALDH6A1	339.313994	416.83234	261.7956521	-0.67133788	0.122997081	-5.4581611	4.81E-08	1.99E-07
ENSG00000158301	GPRASP2	392.3235203	481.85313	302.7939131	-0.671772551	0.124357235	-5.4019579	6.59E-08	2.68E-07
ENSG00000185420	SMYD3	364.860421	448.31774	281.4031061	-0.671810068	0.120312751	-5.5838643	2.35E-08	9.93E-08
ENSG00000099800	TIMM13	1580.875119	1942.5327	1219.217498	-0.67207413	0.069763772	-9.6335692	5.77E-22	4.82E-21
ENSG00000138443	ABI2	1862.175737	2288.4619	1435.889559	-0.672188969	0.065727495	-10.226907	1.50E-24	1.37E-23
ENSG00000123815	COQ8B	246.356197	302.66091	190.0514861	-0.672842125	0.149357018	-4.5049247	6.64E-06	2.27E-05
ENSG00000182512	GLRX5	746.6824895	917.764	575.6009744	-0.673204	0.10226122	-6.5831798	4.60E-11	2.36E-10
ENSG00000166974	MAPRE2	6109.393212	7513.5784	4705.208024	-0.675471939	0.045235728	-14.932266	2.03E-50	3.43E-49
ENSG00000147130	ZMYM3	1180.246939	1452.0274	908.4664548	-0.675583843	0.077244182	-8.7460806	2.21E-18	1.61E-17
ENSG00000108312	UBTF	2747.761862	3380.5208	2115.002934	-0.676090733	0.069910743	-9.6707702	4.01E-22	3.38E-21
ENSG00000116161	CACYBP	1504.40945	1850.7217	1158.097228	-0.676200934	0.066768938	-10.127478	4.17E-24	3.76E-23
ENSG00000100316	RPL3	50708.35437	62380.796	39035.91249	-0.676324346	0.036050948	-18.760238	1.60E-78	4.09E-77
ENSG00000135083	CCNJL	568.6220111	699.51238	437.7316399	-0.676344673	0.099407065	-6.8037887	1.02E-11	5.42E-11
ENSG00000101096	NFATC2	387.1166219	476.40773	297.8255155	-0.676505332	0.133546742	-5.065682	4.07E-07	1.56E-06
ENSG00000065308	TRAM2	9440.019256	11615.981	7264.057046	-0.677240672	0.045615197	-14.846821	7.29E-50	1.22E-48
ENSG00000204392	LSM2	603.3502607	742.30859	464.3919334	-0.677292239	0.095751651	-7.0734262	1.51E-12	8.42E-12

ENSG00000080503	SMARCA2	2442.549054	3005.9459	1879.152198	-0.677416163	0.060487505	-11.199274	4.11E-29	4.31E-28
ENSG00000198830	HMGN2	3040.032553	3741.2123	2338.852813	-0.677480936	0.054643379	-12.398226	2.67E-35	3.28E-34
ENSG00000115053	NCL	19700.21599	24243.729	15156.70258	-0.677687621	0.037660099	-17.994844	2.14E-72	5.12E-71
ENSG00000166197	NOLC1	2504.39322	3082.0559	1926.730496	-0.677703668	0.060300055	-11.238857	2.63E-29	2.77E-28
ENSG00000196205	EEF1A1P5	2902.653196	3572.3263	2232.980119	-0.678074888	0.060729543	-11.165487	6.02E-29	6.28E-28
ENSG00000134138	MEIS2	1070.561471	1317.7911	823.3318849	-0.678359772	0.074501039	-9.1053733	8.60E-20	6.67E-19
ENSG00000110987	BCL7A	443.4879869	546.1003	340.8756746	-0.678915643	0.112721568	-6.0229436	1.71E-09	7.85E-09
ENSG00000147419	CCDC25	1543.107803	1900.0069	1186.208714	-0.679069904	0.07011014	-9.6857587	3.47E-22	2.92E-21
ENSG00000102804	TSC22D1	2541.659446	3130.1803	1953.138572	-0.68004695	0.060725533	-11.198699	4.14E-29	4.34E-28
ENSG00000179348	GATA2	1020.072818	1255.7601	784.3855043	-0.680076479	0.084025591	-8.0936828	5.79E-16	3.81E-15
ENSG00000148180	GSN	12912.16238	15901.672	9922.652273	-0.680437179	0.0402122	-16.921163	3.14E-64	6.63E-63
ENSG00000105953	OGDH	4264.553699	5251.8557	3277.251697	-0.680572668	0.053855232	-12.637076	1.32E-36	1.67E-35
ENSG00000175792	RUVBL1	1359.777323	1675.1573	1044.397315	-0.680931573	0.076149708	-8.9420116	3.82E-19	2.89E-18
ENSG00000163918	RFC4	191.9619059	236.40727	147.5165382	-0.68125557	0.166010643	-4.1036861	4.07E-05	0.000128
ENSG00000240445	FOXO3B	97.68521472	120.18685	75.18357644	-0.681288765	0.250631768	-2.7182858	0.006562	0.015167
ENSG00000128203	ASPHD2	514.0386288	633.49044	394.5868134	-0.682811775	0.109648172	-6.2272974	4.75E-10	2.26E-09
ENSG00000129317	PUS7L	638.616937	787.19765	490.0362281	-0.682919445	0.095028074	-7.186502	6.65E-13	3.76E-12
ENSG00000174547	MRPL11	798.5919054	984.12627	613.0575424	-0.682978128	0.086114639	-7.931034	2.17E-15	1.39E-14
ENSG00000184640	9-Sep	4947.096558	6097.0521	3797.140973	-0.683621937	0.055259037	-12.371224	3.74E-35	4.57E-34
ENSG00000117155	SSX2IP	258.7184804	319.10248	198.3344834	-0.68408616	0.145070907	-4.7155296	2.41E-06	8.61E-06
ENSG00000247077	PGAM5	1006.344023	1240.5499	772.1381497	-0.684214227	0.089120471	-7.6774081	1.62E-14	9.97E-14
ENSG00000072736	NFATC3	715.7270136	882.32687	549.127155	-0.684864127	0.08987867	-7.6198738	2.54E-14	1.54E-13
ENSG00000157240	FZD1	207.0155763	255.23984	158.7913163	-0.685003243	0.159503313	-4.294602	1.75E-05	5.72E-05
ENSG00000145375	SPATA5	246.3308879	303.94687	188.714909	-0.68521258	0.157780111	-4.3428324	1.41E-05	4.65E-05
ENSG00000034533	ASTE1	115.3196267	142.26085	88.37839971	-0.685349914	0.207199513	-3.3076811	0.000941	0.002489
ENSG00000237550	RPL9P9	2060.754545	2541.2214	1580.28766	-0.685558254	0.060272076	-11.374393	5.61E-30	6.01E-29
ENSG00000242299	AC073861.1	79.63471078	98.148467	61.12095434	-0.685768104	0.25925259	-2.6451736	0.008165	0.018565
ENSG00000162944	RFTN2	114.2468306	141.05135	87.44230642	-0.686376941	0.225619352	-3.04219	0.002349	0.00584
ENSG00000173950	XXYLT1	2859.623624	3527.4095	2191.83772	-0.686744935	0.059406532	-11.560091	6.56E-31	7.18E-30
ENSG00000104129	DNAJC17	248.1573813	306.31998	189.994783	-0.686980413	0.152051178	-4.5180868	6.24E-06	2.13E-05
ENSG00000130826	DKC1	1596.815435	1970.1616	1223.469261	-0.68741262	0.066019294	-10.412299	2.18E-25	2.05E-24
ENSG00000204576	PRR3	161.4285752	199.26772	123.58943	-0.687696752	0.176189273	-3.9031704	9.49E-05	0.000286
ENSG00000096654	ZNF184	238.6984497	294.86588	182.5310215	-0.688949941	0.177869932	-3.8733356	0.000107	0.000321
ENSG00000138111	MFSD13A	154.2866913	190.56707	118.0063146	-0.688969811	0.190718093	-3.6125037	0.000303	0.000858
ENSG00000232517	AC112198.2	59.62791447	73.615601	45.64022784	-0.689152658	0.289724957	-2.3786444	0.017376	0.037124
ENSG00000278535	DHRS11	119.703018	147.79951	91.60652951	-0.68930668	0.216465048	-3.1843787	0.001451	0.00373
ENSG00000164494	PDSS2	267.8693588	330.62445	205.1142662	-0.689676431	0.137532396	-5.0146471	5.31E-07	2.01E-06
ENSG00000183172	SMDT1	403.0359911	497.56085	308.5111325	-0.690131856	0.118452047	-5.8262552	5.67E-09	2.50E-08
ENSG00000079435	LIPE	124.1287819	153.16796	95.08960634	-0.690264003	0.213209051	-3.2374986	0.001206	0.003147
ENSG00000144381	HSPD1	7642.955903	9437.5763	5848.335496	-0.690379847	0.046744213	-14.769311	2.31E-49	3.81E-48
ENSG00000138801	PAPSS1	3233.640277	3993.8459	2473.434624	-0.690750784	0.059007713	-11.70611	1.19E-31	1.32E-30
ENSG00000219481	NBPF1	466.1419741	575.77423	356.5097221	-0.690934073	0.120313641	-5.7427742	9.31E-09	4.04E-08
ENSG00000009413	REV3L	2576.218121	3182.1557	1970.280589	-0.691173586	0.055490149	-12.455789	1.30E-35	1.61E-34
ENSG00000021826	CPS1	165.2452482	204.11336	126.3771332	-0.691935597	0.175548209	-3.9415702	8.09E-05	0.000246
ENSG00000248441	LINC01197	145.4871959	179.63175	111.3426389	-0.692165904	0.194636897	-3.5561906	0.000376	0.001052
ENSG00000174442	ZWILCH	713.0528577	881.52864	544.5770785	-0.693321068	0.101772546	-6.8124567	9.59E-12	5.12E-11
ENSG00000105655	ISYNA1	773.273904	955.50265	591.0451622	-0.69407692	0.089598877	-7.7464913	9.45E-15	5.87E-14
ENSG00000165716	DIPK1B	4202.281836	5195.5531	3209.010546	-0.69526928	0.050924981	-13.652814	1.94E-42	2.82E-41
ENSG00000108439	PNPO	503.2061252	622.33246	384.0797923	-0.695511445	0.104309264	-6.6677821	2.60E-11	1.35E-10
ENSG00000111144	LTA4H	2856.003634	3532.4871	2179.520148	-0.696271243	0.056611842	-12.299039	9.17E-35	1.11E-33
ENSG00000105849	TWISTNB	939.1055058	1161.3486	716.8624232	-0.696417326	0.079637125	-8.7448828	2.23E-18	1.63E-17
ENSG00000145349	CAMK2D	1182.76757	1462.9298	902.6053684	-0.696449642	0.076833561	-9.0643936	1.25E-19	9.66E-19
ENSG00000229124	VIM-AS1	307.4284742	380.12807	234.7288825	-0.697075502	0.144239738	-4.8327563	1.35E-06	4.93E-06
ENSG00000143891	GALM	202.9864992	251.06271	154.9102885	-0.697447453	0.170923067	-4.0804759	4.49E-05	0.000141
ENSG00000104205	SGK3	338.2540649	418.77876	257.7293691	-0.697646829	0.14894613	-4.6838869	2.81E-06	9.98E-06
ENSG00000187741	FANCA	236.4606011	292.40444	180.5167598	-0.698100325	0.161695083	-4.3173875	1.58E-05	5.19E-05
ENSG00000163071	SPATA18	756.4500662	936.31457	576.5855639	-0.698446314	0.095949879	-7.2792829	3.36E-13	1.93E-12
ENSG00000146143	PRIM2	276.6901682	342.59269	210.7876502	-0.699487335	0.148691628	-4.7042819	2.55E-06	9.07E-06
ENSG00000243943	ZNF512	381.1230473	471.62151	290.6245886	-0.699630894	0.121243945	-5.7704398	7.91E-09	3.45E-08
ENSG00000128694	OSGEPL1	73.88611037	91.48043	56.29179112	-0.700059448	0.257602358	-2.7175972	0.006576	0.015189
ENSG00000138413	IDH1	2926.836435	3623.3336	2230.339238	-0.700088822	0.053995309	-12.965734	1.91E-38	2.53E-37
ENSG00000099901	RANBP1	2040.574378	2526.2564	1554.892375	-0.700550624	0.064980118	-10.781	4.23E-27	4.19E-26
ENSG00000179965	ZNF771	147.9849032	183.08468	112.8851314	-0.700789841	0.211379049	-3.3153231	0.000915	0.002425
ENSG00000172927	MYEOV	328.703686	407.18023	250.2271373	-0.702541748	0.124905481	-5.624587	1.86E-08	7.92E-08
ENSG00000234664	HMGN2P5	111.8441004	138.43125	85.2569512	-0.702729503	0.22814274	-3.0801811	0.002069	0.005189
ENSG00000215021	PHB2	4338.884976	5375.7436	3302.026328	-0.703615132	0.054112557	-13.002807	1.18E-38	1.57E-37
ENSG00000141971	MVB12A	710.9396905	880.842	541.037384	-0.704164095	0.093082467	-7.5649488	3.88E-14	2.33E-13
ENSG00000116251	RPL22	1567.424468	1942.829	1192.019953	-0.704484219	0.073001669	-9.6502481	4.90E-22	4.11E-21
ENSG00000112992	NNT	2550.878385	3161.5463	1940.210513	-0.704490588	0.056592765	-12.448421	1.43E-35	1.76E-34
ENSG00000198722	UNC13B	3785.28148	4691.3849	2879.178102	-0.704519935	0.051624802	-13.646927	2.11E-42	3.06E-41
ENSG00000102471	NDFIP2	467.3868676	579.50019	355.2735427	-0.705225138	0.112106695	-6.2906603	3.16E-10	1.53E-09
ENSG00000140691	ARMC5	576.5978027	714.63223	438.5633781	-0.705681203	0.103041014	-6.8485468	7.46E-12	4.01E-11
ENSG00000214706	IFRD2	1630.310926	2021.2352	1239.386672	-0.706284376	0.082091082	-8.6036676	7.72E-18	5.51E-17
ENSG00000148090	AUH	147.7501429	183.37	112.130288	-0.707431741	0.189111813	-3.740812	0.000183	0.000532
ENSG00000116830	TTF2	580.1393528	719.92121	440.3574969	-0.708143924	0.098781799	-7.1687693	7.57E-13	4.28E-12
ENSG00000176454	LPCAT4	682.9617078	847.20347	518.7199471	-0.708465849	0.097263593	-7.2839778	3.24E-13	1.87E-12
ENSG00000154928	EPHB1	145.3879275	180.43348	110.3423776	-0.7093817	0.216054349	-3.2833484	0.001026	0.002699
ENSG00000113810	SMC4	2648.193484	3286.6843	2009.702689	-0.709492139	0.054693626	-12.972117	1.76E-38	2.34E-37
ENSG00000054793	ATP9A	2701.940956	3353.3963	2050.485608	-0.709648717	0.055648135	-12.752426	3.02E-37	3.89E-36
ENSG00000141447	OSBPL1A	782.0805185	970.91629	593.2447449	-0.710650852	0.094284264	-7.5373219	4.80E-14	2.86E-13
ENSG00000115325	DOK1	362.3649228	449.93829	274.7915563	-0.711206593	0.126145188	-5.6380002	1.72E-08	7.34E-08
ENSG00000167363	FN3K	261.2722593	324.25575	198.2887725	-0.711445159	0.145535098	-4.8884782	1.02E-06	3.75E-06
ENSG00000175414	ARL10	1293.168909	1605.8053	980.5325223	-0.711846648	0.074310972	-9.579294	9.77E-22	8.11E-21
ENSG00000141101	NOB1	1429.144305	1774.6087	1083.679862	-0.711948107	0.078051607	-9.1215048	7.41E-20	5.77E-19
ENSG00000117318	ID3	2993.049326	3716.3719	2269.726769	-0.712037649	0.065902859	-10.804351	3.28E-27	3.27E-26

ENSG00000156253	RWDD2B	657.6218897	817.11301	498.1307725	-0.712366995	0.102852248	-6.9261198	4.33E-12	2.36E-11
ENSG00000234912	SNHG20	126.4831986	157.20052	95.76587583	-0.714145711	0.210357821	-3.3949092	0.000687	0.001851
ENSG00000180198	RCC1	1290.282608	1603.9134	976.6518223	-0.716172565	0.076319043	-9.3839301	6.36E-21	5.13E-20
ENSG00000165046	LETM2	121.0180517	150.38925	91.64685746	-0.71657423	0.216292131	-3.3129926	0.000923	0.002444
ENSG00000121895	TMEM156	131.0101728	162.83366	99.18668818	-0.716585149	0.22428963	-3.1949099	0.001399	0.003606
ENSG00000047579	DTNBP1	513.8972786	639.13041	388.6641471	-0.717319213	0.112989865	-6.348527	2.17E-10	1.06E-09
ENSG00000159314	ARHGAP27	670.4138439	833.8367	506.9909836	-0.717515305	0.093413367	-7.6810774	1.58E-14	9.70E-14
ENSG00000070214	SLC44A1	3100.621186	3856.0848	2345.1576	-0.717603101	0.059469478	-12.066746	1.58E-33	1.85E-32
ENSG00000260027	HOXB7	189.5309736	235.67507	143.3868751	-0.718211417	0.172751844	-4.1574747	3.22E-05	0.000103
ENSG00000244716	BX679664.3	71.42022255	88.792044	54.04840114	-0.718234162	0.287026908	-2.5023234	0.012338	0.02716
ENSG00000106144	CASP2	838.3921167	1043.2152	633.5690803	-0.718490093	0.086975458	-8.2608371	1.45E-16	9.81E-16
ENSG00000122644	ARL4A	533.907341	664.43103	403.3836528	-0.718536314	0.106178088	-6.7672749	1.31E-11	6.95E-11
ENSG00000271601	LIX1L	1517.931106	1888.1522	1147.71	-0.718549688	0.064174802	-11.196757	4.23E-29	4.43E-28
ENSG00000108468	CBX1	2585.232008	3216.3579	1954.106112	-0.718711069	0.057524704	-12.493955	8.05E-36	1.00E-34
ENSG00000177971	IMP3	458.2749049	570.34238	346.2074257	-0.718958039	0.114542388	-6.2767858	3.46E-10	1.66E-09
ENSG00000198168	SVIP	960.3219802	1194.8346	725.8093201	-0.719351429	0.082765605	-8.6914296	3.58E-18	2.59E-17
ENSG00000186642	PDE2A	1038.857201	1292.6062	785.1081975	-0.719361511	0.079733001	-9.0221301	1.84E-19	1.41E-18
ENSG00000125703	ATG4C	225.3353566	280.49227	170.1784403	-0.720223903	0.151601681	-4.7507646	2.03E-06	7.27E-06
ENSG00000132589	FLOT2	4802.480093	5976.8196	3628.140574	-0.720408251	0.045861602	-15.70831	1.33E-55	2.43E-54
ENSG00000175455	CCDC14	348.3875041	433.72852	263.0464899	-0.72043965	0.12839397	-5.6111642	2.01E-08	8.53E-08
ENSG00000156531	PHF6	1259.776845	1568.4007	951.1529694	-0.720889753	0.080948928	-8.9054886	5.32E-19	3.99E-18
ENSG00000071246	VASH1	5280.420472	6573.2707	3987.57021	-0.720988184	0.070825495	-10.179783	2.44E-24	2.21E-23
ENSG00000180917	CMTR2	725.3765691	903.44748	547.3056594	-0.722718622	0.093269927	-7.7486779	9.29E-15	5.77E-14
ENSG00000139117	CPNE8	547.8997959	682.4038	413.3957951	-0.722772396	0.099021296	-7.2991612	2.90E-13	1.67E-12
ENSG00000168061	SAC3D1	239.9399204	298.82529	181.0545547	-0.722986693	0.148227718	-4.8775405	1.07E-06	3.96E-06
ENSG00000167632	TRAPPC9	800.9321688	997.59886	604.2654766	-0.723139541	0.086527364	-8.3573509	6.41E-17	4.41E-16
ENSG00000140455	AC007950.1	918.0340026	1143.4157	692.6523263	-0.7234265	0.085457431	-8.4653434	2.55E-17	1.79E-16
ENSG00000066027	PPP2R5A	911.8190044	1135.8578	687.7802564	-0.723842232	0.083394624	-8.6797229	3.97E-18	2.86E-17
ENSG00000169738	DCXR	506.0312011	630.38628	381.6761226	-0.724104146	0.11753175	-6.1609237	7.23E-10	3.40E-09
ENSG00000198933	TBKBP1	664.8826359	828.16239	501.6028769	-0.724112023	0.097749502	-7.4078334	1.28E-13	7.52E-13
ENSG00000181163	NPM1	21116.705	26309.964	15923.44638	-0.724468863	0.037578434	-19.278847	8.09E-83	2.20E-81
ENSG00000022567	SLC45A4	177.8371663	221.45019	134.2241401	-0.724602511	0.181503511	-3.9922211	6.55E-05	0.000201
ENSG00000140961	OSGIN1	304.2832492	379.0919	229.4745981	-0.724734149	0.135789444	-5.3371906	9.44E-08	3.80E-07
ENSG00000152133	GPATCH11	385.5824842	480.39921	290.7657547	-0.724774442	0.12893117	-5.6214059	1.89E-08	8.06E-08
ENSG00000168090	COPS6	3640.982569	4536.8684	2745.096713	-0.725020869	0.056308756	-12.87581	6.16E-38	8.04E-37
ENSG00000171877	FRMD5	991.233717	1235.3392	747.1282207	-0.72509828	0.083389403	-8.6953288	3.46E-18	2.50E-17
ENSG00000261236	BOP1	1527.880199	1903.5416	1152.218792	-0.725112899	0.079966321	-9.0677287	1.22E-19	9.38E-19
ENSG00000167733	HSD11B1L	74.20445858	92.491987	55.91692989	-0.7252047	0.266846286	-2.7176871	0.006574	0.015189
ENSG00000134262	AP4B1	578.0085994	720.52288	435.4943174	-0.725609215	0.11407588	-6.3607593	2.01E-10	9.81E-10
ENSG00000114738	MAPKAPK3	739.6457597	921.71389	557.5776255	-0.726097047	0.096190445	-7.5485361	4.40E-14	2.64E-13
ENSG00000198554	WDHD1	422.02826	526.20359	317.8529321	-0.72648148	0.121107295	-5.99866	1.99E-09	9.08E-09
ENSG00000272142	AL359643.3	64.66109879	80.556151	48.76604695	-0.727356886	0.296228004	-2.4553954	0.014073	0.030602
ENSG00000144724	PTPRG	3921.908841	4893.0205	2950.797224	-0.729408731	0.05228597	-13.950372	3.13E-44	4.70E-43
ENSG00000159199	ATP5MC1	876.6608462	1093.5693	659.7523709	-0.729613353	0.091031261	-8.0149758	1.10E-15	7.16E-15
ENSG00000078070	MCCC1	561.8827621	700.86376	422.901767	-0.729674918	0.099468395	-7.3357464	2.20E-13	1.28E-12
ENSG00000173898	SPTBN2	1929.647332	2407.0172	1452.277513	-0.729763513	0.067658355	-10.786007	4.01E-27	3.98E-26
ENSG00000273149	AL138963.3	788.7461365	983.93013	593.5621443	-0.730078383	0.116663084	-6.2580069	3.90E-10	1.87E-09
ENSG00000254531	AP001816.1	80.89935138	101.05053	60.74817511	-0.731059613	0.258841742	-2.8243498	0.004738	0.01122
ENSG00000159423	ALDH4A1	327.3628684	408.4411	246.284633	-0.731158368	0.138751138	-5.2695666	1.37E-07	5.42E-07
ENSG00000106089	STX1A	321.6091646	401.36119	241.8571443	-0.731539669	0.134383309	-5.4436795	5.22E-08	2.15E-07
ENSG00000055044	NOP58	1085.632942	1355.1907	816.0752119	-0.731758703	0.072812174	-10.04995	9.19E-24	8.21E-23
ENSG00000068784	SRBD1	484.2648413	604.68493	363.844753	-0.73206082	0.107361637	-6.8186443	9.19E-12	4.91E-11
ENSG00000123700	KCNJ2	559.9795321	699.21258	420.7464884	-0.732077563	0.112434992	-6.5111186	7.46E-11	3.76E-10
ENSG00000139318	DUSP6	3581.412127	4471.4801	2691.344177	-0.732186535	0.057014796	-12.842044	9.53E-38	1.24E-36
ENSG00000010361	FUZ	197.2810424	246.32769	148.2343922	-0.733288789	0.163095187	-4.4960787	6.92E-06	2.36E-05
ENSG00000148950	IMMP1L	63.27310756	79.020464	47.52575063	-0.733390542	0.282498517	-2.5960863	0.009429	0.021211
ENSG00000181035	SLC25A42	202.9842586	253.48803	152.4804901	-0.733829694	0.160536987	-4.5710942	4.85E-06	1.68E-05
ENSG00000259330	INAFM2	1009.649163	1260.7953	758.5030407	-0.734002168	0.07849017	-9.3515171	8.64E-21	6.95E-20
ENSG00000074410	CA12	89.28435691	111.57248	66.99623065	-0.734343006	0.243659864	-3.0138037	0.00258	0.006385
ENSG00000078237	TIGAR	667.9263386	834.63681	501.2158702	-0.734969204	0.097477056	-7.53992	4.70E-14	2.81E-13
ENSG00000171033	PKIA	1254.087802	1566.9122	941.2634149	-0.735048612	0.073605534	-9.9863227	1.75E-23	1.55E-22
ENSG00000139637	C12orf10	656.845334	820.3871	493.3035702	-0.735473997	0.118089089	-6.2281283	4.72E-10	2.25E-09
ENSG00000153815	CMIP	4915.650892	6142.8758	3688.425982	-0.735681426	0.048199974	-15.263108	1.35E-52	2.36E-51
ENSG00000185834	RPL12P4	53.95674474	67.384082	40.52940759	-0.735920603	0.315613501	-2.3317146	0.019716	0.041583
ENSG00000111875	ASF1A	802.6685045	1003.5687	601.7683209	-0.736966005	0.093213469	-7.906218	2.65E-15	1.69E-14
ENSG00000101220	C20orf27	819.7239353	1024.4552	614.9926405	-0.737137232	0.094062318	-7.8366901	4.63E-15	2.92E-14
ENSG00000100348	TXN2	1570.908547	1963.5415	1178.275584	-0.737610401	0.071118095	-10.371628	3.34E-25	3.13E-24
ENSG00000197535	MYO5A	10754.27291	13445.629	8062.916908	-0.737632335	0.041088034	-17.952485	4.59E-72	1.09E-70
ENSG00000175087	PDIK1L	171.4455123	214.5347	128.3563288	-0.737687922	0.203444315	-3.6259943	0.000288	0.000818
ENSG00000175806	MSRA	451.3243926	564.19499	338.4537919	-0.737792856	0.11545531	-6.3902895	1.66E-10	8.15E-10
ENSG00000110427	KIAA1549L	4121.10063	5152.5603	3089.640973	-0.738262686	0.056555514	-13.05377	6.05E-39	8.10E-38
ENSG00000246067	RAB30-AS1	103.7747925	129.75353	77.79605967	-0.738329387	0.226922424	-3.2536643	0.001139	0.002982
ENSG00000079150	FKBP7	345.3712735	431.91241	258.8301409	-0.73873934	0.125088527	-5.9057322	3.51E-09	1.58E-08
ENSG00000135966	TGFBRAP1	2398.240613	2999.3599	1797.121332	-0.739013707	0.057367828	-12.882023	5.68E-38	7.42E-37
ENSG00000283498	MIR1244-2	75.45594972	94.403407	56.5084922	-0.739737496	0.265441184	-2.7868226	0.005323	0.012501
ENSG00000261371	PECAM1	127865.5217	159947.55	95783.48948	-0.739741386	0.043088191	-17.168077	4.60E-66	1.00E-64
ENSG00000083635	NUFIP1	242.2930359	303.15465	181.4314227	-0.739883289	0.146406851	-5.0536111	4.34E-07	1.65E-06
ENSG00000269893	SNHG8	433.1173554	541.82009	324.4146165	-0.740002212	0.119541662	-6.1903289	6.00E-10	2.84E-09
ENSG00000161981	SNRNP25	609.2783636	762.51944	456.0372834	-0.741142101	0.096255379	-7.6997473	1.36E-14	8.41E-14
ENSG00000166228	PCBD1	1132.164071	1416.6376	847.6905668	-0.741508709	0.083973995	-8.8302183	1.04E-18	7.76E-18
ENSG00000142544	CTU1	137.0533299	171.44815	102.6585108	-0.741815501	0.214826054	-3.4530984	0.000554	0.001514
ENSG00000178904	DPY19L3	706.3119613	884.3381	528.2858197	-0.743500296	0.095385763	-7.7946674	6.46E-15	4.03E-14
ENSG00000148187	MRRF	671.8705076	841.22181	502.5192095	-0.743936401	0.101765321	-7.3103135	2.67E-13	1.54E-12
ENSG00000181885	CLDN7	267.2864068	334.53818	200.0346298	-0.744015156	0.151897059	-4.8981538	9.67E-07	3.58E-06
ENSG00000013016	EHD3	148.518106	186.14762	110.8885943	-0.744102161	0.196761703	-3.7817428	0.000156	0.000456

ENSG00000177192	PUS1	566.3619923	709.15512	423.5688642	-0.74413128	0.113369608	-6.5637634	5.25E-11	2.68E-10
ENSG00000140398	NEIL1	107.96746	135.24662	80.68829639	-0.744162135	0.305822405	-2.4333146	0.014961	0.032373
ENSG00000170515	PA2G4	2747.042646	3440.0566	2054.028671	-0.744312215	0.057785955	-12.880504	5.80E-38	7.57E-37
ENSG00000176641	RNF152	797.680403	999.35814	596.0026615	-0.745168858	0.098003924	-7.6034594	2.88E-14	1.75E-13
ENSG00000151491	EPS8	581.0286036	727.81273	434.2444742	-0.745845854	0.097799955	-7.6262393	2.42E-14	1.47E-13
ENSG00000138668	HNRNPD	5878.895867	7367.4624	4390.329361	-0.746661836	0.050713223	-14.723218	4.57E-49	7.52E-48
ENSG00000089009	RPL6	20170.40025	25280.239	15060.56168	-0.747276313	0.040960791	-18.243699	2.32E-74	5.70E-73
ENSG00000106628	POLD2	1379.95613	1729.2337	1030.678597	-0.747585769	0.086343469	-8.6582781	4.79E-18	3.44E-17
ENSG00000163125	RPRD2	1429.674175	1792.4665	1066.881841	-0.74802989	0.071921998	-10.400572	2.46E-25	2.32E-24
ENSG00000131351	HAUS8	149.9429219	188.12611	111.7597316	-0.74924931	0.195032594	-3.841662	0.000122	0.000363
ENSG00000156384	SFR1	193.5259938	242.82301	144.2289763	-0.749830628	0.169913784	-4.4130065	1.02E-05	3.41E-05
ENSG00000170364	SETMAR	203.3370924	255.1751	151.4990888	-0.750243049	0.177509314	-4.2264996	2.37E-05	7.65E-05
ENSG00000138101	DTNB	231.8931657	291.11037	172.6759613	-0.751863721	0.163280271	-4.6047432	4.13E-06	1.44E-05
ENSG00000177464	GPR4	2111.802976	2649.7023	1573.903634	-0.752209947	0.073227651	-10.272212	9.40E-25	8.64E-24
ENSG00000138190	EXOC6	2252.275361	2827.4544	1677.096356	-0.752894572	0.074467763	-10.110342	4.97E-24	4.47E-23
ENSG00000136824	SMC2	1178.555029	1480.062	877.0480695	-0.755452641	0.079729625	-9.4751811	2.66E-21	2.18E-20
ENSG00000092847	AGO1	1556.248959	1955.1446	1157.353349	-0.756361793	0.063162591	-11.974838	4.81E-33	5.58E-32
ENSG00000234741	GAS5	1134.844884	1425.8972	843.792545	-0.75688151	0.088411303	-8.5609135	1.12E-17	7.94E-17
ENSG00000114491	UMPS	696.2727554	874.66552	517.8799937	-0.757384025	0.095093558	-7.9646197	1.66E-15	1.07E-14
ENSG00000244300	GATA2-AS1	307.3655886	386.23142	228.4997527	-0.758264929	0.136693612	-5.5471863	2.90E-08	1.22E-07
ENSG00000066735	KIF26A	219.7691107	276.17531	163.3629162	-0.758392322	0.161954147	-4.6827595	2.83E-06	1.00E-05
ENSG00000130347	RTN4IP1	99.17972453	124.72761	73.63183658	-0.759004544	0.229218527	-3.3112705	0.000929	0.002459
ENSG00000155846	PPARGC1B	92.18690989	115.92486	68.44896075	-0.759052864	0.271175246	-2.799123	0.005124	0.012063
ENSG00000178952	TUFM	4309.303423	5417.2193	3201.3875	-0.759252338	0.054560761	-13.915721	5.08E-44	7.63E-43
ENSG00000079462	PAFAH1B3	375.2455702	471.71396	278.7771834	-0.759332337	0.121789383	-6.2347991	4.52E-10	2.16E-09
ENSG00000170271	FAXDC2	205.4508657	258.40364	152.4980936	-0.759643322	0.159441378	-4.7644052	1.89E-06	6.82E-06
ENSG00000104408	EIF3E	7633.030645	9600.0861	5665.975193	-0.76065024	0.044272959	-17.180922	3.69E-66	8.07E-65
ENSG00000111602	TIMELESS	768.4983811	966.76153	570.2352353	-0.760958418	0.087693793	-8.677449	4.05E-18	2.92E-17
ENSG00000100605	ITPK1	1288.664771	1620.6416	956.687936	-0.760979585	0.079156989	-9.6135489	7.01E-22	5.85E-21
ENSG00000089006	SNX5	1605.053507	2019.3682	1190.73886	-0.762392414	0.066774836	-11.417361	3.42E-30	3.70E-29
ENSG00000143578	CREB3L4	115.0548373	144.68048	85.4291979	-0.762624156	0.231942223	-3.2879919	0.001009	0.002659
ENSG00000178665	ZNF713	64.99063775	81.719176	48.26209931	-0.762763316	0.327722093	-2.3274699	0.01994	0.041995
ENSG00000124802	EEF1E1	122.5838994	154.27851	90.88928502	-0.762832546	0.241967719	-3.1526211	0.001618	0.004128
ENSG00000166845	C18orf54	584.2771691	735.31375	433.2405926	-0.763433809	0.096681543	-7.8963759	2.87E-15	1.82E-14
ENSG00000224578	HNRNPA1P48	101.1377473	127.219	75.05649688	-0.763440782	0.243840372	-3.1309039	0.001743	0.004421
ENSG00000182518	FAM104B	126.436365	159.21764	93.65508921	-0.763730733	0.213879019	-3.5708539	0.000356	0.000996
ENSG00000235173	HGH1	574.5425525	722.99819	426.0869149	-0.763818463	0.098803531	-7.7306798	1.07E-14	6.63E-14
ENSG00000079337	RAPGEF3	630.9309341	794.1585	467.703373	-0.764127573	0.093545301	-8.1685297	3.12E-16	2.08E-15
ENSG00000166068	SPRED1	909.2791853	1144.658	673.9003473	-0.764693741	0.084041223	-9.0990316	9.11E-20	7.06E-19
ENSG00000135709	KIAA0513	358.0170526	450.91163	265.1224724	-0.766017343	0.140937113	-5.4351713	5.47E-08	2.25E-07
ENSG00000215068	AC025171.2	54.24000212	68.321191	40.15881365	-0.766192778	0.308387979	-2.4845092	0.012973	0.028469
ENSG00000100129	EIF3L	4375.315829	5510.6347	3239.996984	-0.766430089	0.057070393	-13.429557	4.06E-41	5.72E-40
ENSG00000163344	PMVK	523.0564238	658.85489	387.2579534	-0.766857272	0.112564191	-6.8126219	9.58E-12	5.11E-11
ENSG00000196372	ASB13	280.7735948	353.8289	207.71829	-0.767104478	0.139871706	-5.4843435	4.15E-08	1.72E-07
ENSG00000165138	ANKS6	709.1624746	893.33537	524.9895823	-0.767159625	0.098887192	-7.757927	8.63E-15	5.37E-14
ENSG00000101844	ATG4A	1485.007045	1870.5195	1099.494606	-0.767351448	0.07676702	-9.9958478	1.59E-23	1.41E-22
ENSG00000172840	PDP2	447.3087261	563.99529	330.6221656	-0.769111968	0.118879277	-6.4696891	9.82E-11	4.91E-10
ENSG00000168807	SNTB2	4358.426109	5494.2408	3222.611443	-0.769764333	0.050712472	-15.178994	4.87E-52	8.43E-51
ENSG00000164077	MON1A	488.874868	616.18064	361.5690916	-0.770370821	0.108223582	-7.1183268	1.09E-12	6.13E-12
ENSG00000213366	GSTM2	68.01960315	85.77877	50.26043666	-0.770385252	0.272189059	-2.8303314	0.00465	0.011032
ENSG00000230629	RPS23P8	53.05019658	66.797805	39.30258835	-0.770602684	0.324531915	-2.3745051	0.017572	0.037513
ENSG00000159399	HK2	503.23988	634.54653	371.9332299	-0.770867723	0.102674418	-7.507885	6.01E-14	3.56E-13
ENSG00000266835	GAPLINC	51.28565879	64.698266	37.87305129	-0.771144501	0.311533206	-2.4753204	0.013312	0.029136
ENSG00000106462	EZH2	374.7301946	472.58777	276.8726221	-0.771533832	0.13008899	-5.9308157	3.01E-09	1.36E-08
ENSG00000175556	LONRF3	181.8491787	229.23558	134.4627778	-0.77205159	0.176146056	-4.3830195	1.17E-05	3.90E-05
ENSG00000132906	CASP9	252.6783315	318.84625	186.5104121	-0.7721288	0.148435828	-5.2017684	1.97E-07	7.73E-07
ENSG00000113552	GNPDA1	2229.825755	2812.9079	1646.743638	-0.772548991	0.063475141	-12.17089	4.44E-34	5.25E-33
ENSG00000273015	AC008124.1	164.6247196	207.61601	121.6334275	-0.772619114	0.177097078	-4.362687	1.28E-05	4.26E-05
ENSG00000164442	CITED2	1416.540929	1786.8176	1046.26426	-0.772674926	0.07348661	-10.514499	7.41E-26	7.07E-25
ENSG00000154258	ABCA9	328.4387568	414.50505	242.3724588	-0.772742859	0.164846313	-4.6876563	2.76E-06	9.81E-06
ENSG00000035141	FAM136A	947.4206641	1195.6445	699.1968476	-0.773957992	0.078176815	-9.9000962	4.16E-23	3.63E-22
ENSG00000256525	POLG2	100.3232034	126.69198	73.95443103	-0.774521652	0.229826751	-3.3700239	0.000752	0.002016
ENSG00000143443	C1orf56	79.82091674	100.66544	58.97639566	-0.774565279	0.270047974	-2.8682507	0.004127	0.009895
ENSG00000167912	AC090152.1	91.06705358	114.93455	67.19955794	-0.774923644	0.238788331	-3.2452325	0.001174	0.003067
ENSG00000203875	SNHG5	3769.435424	4758.5235	2780.338326	-0.775080496	0.062693114	-12.363088	4.14E-35	5.04E-34
ENSG00000068024	HDAC4	344.1283902	434.57843	253.6783551	-0.775683713	0.147492984	-5.2591228	1.45E-07	5.73E-07
ENSG00000213553	RPLP0P6	209.103555	263.84881	154.3583038	-0.776009413	0.162872743	-4.7645137	1.89E-06	6.82E-06
ENSG00000162409	PRKAA2	93.51660521	118.12748	68.90573439	-0.776814135	0.256039523	-3.0339618	0.002414	0.005995
ENSG00000215492	HNRNPA1P7	72.48117529	91.505512	53.45683882	-0.777131325	0.264073375	-2.9428613	0.003252	0.007907
ENSG00000049541	RFC2	479.5878463	605.69221	353.4834846	-0.777275653	0.107006252	-7.263834	3.76E-13	2.16E-12
ENSG00000197958	RPL12	16901.48639	21350.319	12452.65339	-0.777908614	0.048019567	-16.199826	5.06E-59	9.77E-58
ENSG00000169683	LRRC45	371.1239123	469.04609	273.2017395	-0.778315779	0.125573976	-6.1980659	5.72E-10	2.71E-09
ENSG00000198355	PIM3	3206.788138	4051.824	2361.752304	-0.778761747	0.059682299	-13.048454	6.48E-39	8.67E-38
ENSG00000013503	POLR3B	460.8742631	582.17797	339.5705538	-0.778906467	0.133851333	-5.8191909	5.91E-09	2.61E-08
ENSG00000233476	EEF1A1P6	425.0498636	537.14573	312.9540019	-0.779120078	0.115175111	-6.7646566	1.34E-11	7.08E-11
ENSG00000155849	ELMO1	974.8733822	1231.6234	718.1233713	-0.779257781	0.083404259	-9.3431413	9.35E-21	7.52E-20
ENSG00000103047	TANGO6	436.8927916	552.03808	321.7475027	-0.779273405	0.124825002	-6.2429273	4.29E-10	2.05E-09
ENSG00000147471	PLPBP	1199.057739	1515.1866	882.9289072	-0.77954229	0.072344137	-10.775473	4.49E-27	4.45E-26
ENSG00000100823	APEX1	4196.406417	5303.4907	3089.322139	-0.779577404	0.045701738	-17.057938	3.05E-65	6.54E-64
ENSG00000228782	MRPL45P2	64.92579234	82.080922	47.77066239	-0.780379873	0.285175629	-2.7364887	0.00621	0.014415
ENSG00000037897	METTL1	150.268742	189.84853	110.688953	-0.780412596	0.184167062	-4.2375254	2.26E-05	7.30E-05
ENSG00000196700	ZNF512B	1894.073131	2394.6317	1393.514535	-0.781073151	0.063483316	-12.303597	8.66E-35	1.05E-33
ENSG00000134824	FADS2	11643.55032	14720.654	8566.446546	-0.781180969	0.054850361	-14.242039	5.02E-46	7.82E-45
ENSG00000117519	CNN3	8104.000378	10247.597	5960.404055	-0.781806588	0.040148598	-19.472824	1.87E-84	5.20E-83
ENSG00000037757	MRI1	529.2455571	669.8137	388.6774176	-0.784299421	0.103544682	-7.5745022	3.61E-14	2.17E-13

ENSG00000148339	SLC25A25	580.4401211	734.43338	426.4468641	-0.784864702	0.099533408	-7.8854398	3.13E-15	1.99E-14
ENSG00000151917	BEND6	110.3622735	139.67351	81.05104034	-0.784874894	0.231866476	-3.3850296	0.000712	0.001915
ENSG00000119927	GPAM	411.6942378	521.14106	302.2474146	-0.78535693	0.113787418	-6.9019664	5.13E-12	2.78E-11
ENSG00000147274	RBMX	3658.514242	4630.2823	2686.746169	-0.785360214	0.050308303	-15.610946	6.13E-55	1.10E-53
ENSG00000130159	ECSIT	544.7849264	689.39634	400.1735115	-0.785910336	0.102970388	-7.6323917	2.30E-14	1.40E-13
ENSG00000162972	MAIP1	272.2804298	344.61674	199.9441171	-0.786311912	0.136885163	-5.744318	9.23E-09	4.00E-08
ENSG00000170915	PAQR8	346.6836646	438.85168	254.5156452	-0.786502118	0.131582867	-5.9772381	2.27E-09	1.03E-08
ENSG00000099991	CABIN1	2313.969098	2930.2999	1697.63831	-0.788015712	0.060362178	-13.054793	5.97E-39	8.01E-38
ENSG00000069974	RAB27A	1140.436135	1444.1326	836.7396303	-0.788326914	0.079873837	-9.8696513	5.64E-23	4.88E-22
ENSG00000160193	WDR4	282.060667	357.20206	206.919274	-0.788707524	0.136228802	-5.7895798	7.06E-09	3.09E-08
ENSG00000124784	RIOK1	500.4650485	633.83094	367.0991523	-0.788941312	0.108965757	-7.2402682	4.48E-13	2.55E-12
ENSG00000111846	GCNT2	600.3478736	760.3993	440.296443	-0.789374951	0.112572735	-7.0121326	2.35E-12	1.30E-11
ENSG00000136720	HS6ST1	831.0975803	1053.0378	609.1574104	-0.789814863	0.084601117	-9.3357498	1.00E-20	8.03E-20
ENSG00000042088	TDP1	625.3532307	792.34011	458.3663469	-0.790427812	0.096832632	-8.1628248	3.27E-16	2.18E-15
ENSG00000167680	SEMA6B	16210.82994	20543.443	11878.21674	-0.790436994	0.047394362	-16.67787	1.90E-62	3.87E-61
ENSG00000255310	AF131215.5	62.93698919	79.761455	46.11252299	-0.790570093	0.290100809	-2.7251565	0.006427	0.014884
ENSG00000251322	SHANK3	6325.444859	8016.2236	4634.666069	-0.790824007	0.048635599	-16.260188	1.89E-59	3.68E-58
ENSG00000253846	PCDHGA10	255.2859641	323.58697	186.9849582	-0.791036134	0.169551508	-4.6654621	3.08E-06	1.09E-05
ENSG00000122483	CCDC18	160.8328935	203.83079	117.8349967	-0.791663053	0.204164098	-3.8775821	0.000105	0.000316
ENSG00000197077	KIAA1671	5073.828978	6434.3339	3713.324035	-0.793153898	0.049580273	-15.997368	1.33E-57	2.51E-56
ENSG00000110844	PRPF40B	96.76976333	122.59284	70.9466883	-0.793178205	0.267982435	-2.9598142	0.003078	0.00752
ENSG00000150967	ABCB9	381.6632487	483.98106	279.3454399	-0.793257012	0.116475735	-6.8104916	9.73E-12	5.18E-11
ENSG00000170584	NUDCD2	559.1507869	709.40253	408.8990456	-0.794198121	0.104663423	-7.5881153	3.25E-14	1.96E-13
ENSG00000065054	SLC9A3R2	2893.529834	3671.014	2116.0457	-0.795261318	0.07267295	-10.943017	7.18E-28	7.30E-27
ENSG00000198034	RPS4X	26298.79032	33369.204	19228.377	-0.795313537	0.041336766	-19.239859	1.72E-82	4.66E-81
ENSG00000100558	PLEK2	592.4051964	751.57833	433.232058	-0.795921584	0.131332198	-6.060369	1.36E-09	6.27E-09
ENSG00000069275	NUCKS1	7245.066762	9195.3364	5294.797114	-0.796011545	0.046548999	-17.100508	1.47E-65	3.18E-64
ENSG00000154760	SLFN13	43.35721247	54.987766	31.7266594	-0.796162408	0.350798274	-2.2695733	0.023233	0.048164
ENSG00000120458	MSANTD2	207.268079	263.32984	151.2063184	-0.799618562	0.160094339	-4.9946711	5.89E-07	2.22E-06
ENSG00000133561	GIMAP6	5726.631897	7274.6426	4178.621186	-0.799662014	0.043680303	-18.307154	7.26E-75	1.78E-73
ENSG00000211584	SLC48A1	449.0498387	570.43793	327.6617471	-0.799971876	0.119389568	-6.7005174	2.08E-11	1.09E-10
ENSG00000119929	CUTC	296.3456844	376.40245	216.2889189	-0.800089947	0.139107962	-5.7515755	8.84E-09	3.84E-08
ENSG00000163382	NAXE	1255.099099	1594.4981	915.7000663	-0.800168977	0.080074371	-9.9928226	1.64E-23	1.45E-22
ENSG00000123213	NLN	1759.729817	2235.2829	1284.17678	-0.800205214	0.066261363	-12.076498	1.41E-33	1.65E-32
ENSG00000243927	MRPS6	228.6833344	290.71722	166.649446	-0.800287692	0.172531048	-4.6385141	3.51E-06	1.23E-05
ENSG00000145982	FARS2	360.4081794	457.99636	262.8199977	-0.80293164	0.132420211	-6.0635128	1.33E-09	6.15E-09
ENSG00000204371	EHMT2	1474.147899	1874.0908	1074.204961	-0.802961671	0.067255774	-11.938925	7.42E-33	8.57E-32
ENSG00000103168	TAF1C	855.2441101	1087.1649	623.323272	-0.802996846	0.092363779	-8.69385	3.50E-18	2.54E-17
ENSG00000106780	MEGF9	1559.038597	1982.5705	1135.50666	-0.803708661	0.067433675	-11.918506	9.48E-33	1.09E-31
ENSG00000135643	KCNMB4	153.89385	195.73291	112.0547906	-0.803715957	0.189547407	-4.2401844	2.23E-05	7.22E-05
ENSG00000141401	IMPA2	57.75919153	73.421817	42.09656565	-0.804650164	0.292147651	-2.7542585	0.005883	0.013709
ENSG00000267365	KCNJ2-AS1	53.80252112	68.347659	39.25738364	-0.804667014	0.319740995	-2.5166214	0.011849	0.026134
ENSG00000130787	HIP1R	3518.608909	4475.408	2561.80977	-0.804683821	0.056966264	-14.12562	2.64E-45	4.06E-44
ENSG00000213390	ARHGAP19	382.3839438	486.77222	277.9956676	-0.807507237	0.120048452	-6.726511	1.74E-11	9.14E-11
ENSG00000242071	RPL7AP6	125.1569076	159.41252	90.901299	-0.807903894	0.227987368	-3.5436345	0.000395	0.001099
ENSG00000157978	LDLRAP1	978.6215701	1245.8592	711.3839822	-0.80968487	0.084643033	-9.5658773	1.11E-21	9.21E-21
ENSG00000255717	SNHG1	377.4734208	480.95082	273.9960196	-0.810462652	0.142247193	-5.6975652	1.22E-08	5.23E-08
ENSG00000186153	WWOX	113.4933003	144.62264	82.36396414	-0.811270951	0.22886331	-3.5447838	0.000393	0.001095
ENSG00000139239	RPL14P1	64.33068872	81.924852	46.7365257	-0.811688546	0.283594262	-2.8621473	0.004208	0.010066
ENSG00000123472	ATPAF1	691.9873309	881.59682	502.3778368	-0.811747515	0.09891167	-8.2067921	2.27E-16	1.53E-15
ENSG00000159055	MIS18A	197.7037523	252.05246	143.3550442	-0.812525435	0.161615507	-5.0275215	4.97E-07	1.88E-06
ENSG00000135486	HNRNPA1	19326.37947	24630.579	14022.17949	-0.812735673	0.036710379	-22.139125	1.33E-108	4.73E-107
ENSG00000176485	PLA2G16	1401.336292	1786.1641	1016.508505	-0.813088074	0.075798536	-10.726963	7.61E-27	7.49E-26
ENSG00000088727	KIF9	201.4302536	256.78275	146.0777538	-0.813584283	0.1660256	-4.9003544	9.57E-07	3.55E-06
ENSG00000239887	C1orf226	141.971378	181.04575	102.8970075	-0.814289721	0.190535534	-4.2736896	1.92E-05	6.26E-05
ENSG00000099889	ARVCF	1504.210566	1918.1125	1090.30865	-0.814748887	0.084444135	-9.6483775	4.99E-22	4.19E-21
ENSG00000197417	SHPK	70.01295456	89.226824	50.79908515	-0.814927197	0.283927497	-2.8701947	0.004102	0.009838
ENSG00000179958	DCTPP1	1005.012236	1281.3421	728.6823775	-0.814990325	0.080560352	-10.116519	4.67E-24	4.20E-23
ENSG00000168765	GSTM4	84.00698165	107.17115	60.84281427	-0.815677707	0.266181723	-3.0643641	0.002181	0.005459
ENSG00000261455	LINC01003	52.70790929	67.249818	38.16600011	-0.816871168	0.308037502	-2.6518562	0.008005	0.018241
ENSG00000120327	PCDHB14	93.54353581	119.30649	67.78057881	-0.817424758	0.238240201	-3.431095	0.000601	0.001636
ENSG00000213186	TRIM59	282.4889836	360.56614	204.4118305	-0.818449063	0.134433985	-6.0881113	1.14E-09	5.30E-09
ENSG00000152240	HAUS1	682.398891	871.2285	493.5692831	-0.818719352	0.092393653	-8.8612077	7.92E-19	5.91E-18
ENSG00000085662	AKR1B1	3625.487314	4626.8487	2624.125902	-0.818725539	0.075902668	-10.786519	3.99E-27	3.96E-26
ENSG00000130748	TMEM160	217.7552249	277.75732	157.7531283	-0.818825726	0.191440431	-4.2771828	1.89E-05	6.17E-05
ENSG00000075213	SEMA3A	63.16307323	80.611068	45.71507819	-0.819459546	0.277434044	-2.9537094	0.00314	0.007655
ENSG00000065978	YBX1	24137.60705	30813.54	17461.67391	-0.81948313	0.05511914	-14.867488	5.36E-50	9.01E-49
ENSG00000167700	MFSD3	298.5322277	380.91875	216.1457084	-0.819554602	0.160168155	-5.1168386	3.11E-07	1.20E-06
ENSG00000285867	BX470102.2	106.6358953	136.03686	77.23493092	-0.819854061	0.224489761	-3.6520777	0.00026	0.000743
ENSG00000103037	SETD6	123.4551262	157.6438	89.26645594	-0.820771741	0.216545823	-3.7902913	0.00015	0.000442
ENSG00000184508	HDDC3	276.9196024	353.49323	200.3459701	-0.821989631	0.175750196	-4.6770339	2.91E-06	1.03E-05
ENSG00000170425	ADORA2B	312.11443	398.68758	225.5412754	-0.822189667	0.141677424	-5.8032511	6.50E-09	2.86E-08
ENSG00000143401	ANP32E	1326.116634	1693.8519	958.3814139	-0.822355812	0.072878283	-11.283963	1.57E-29	1.67E-28
ENSG00000101224	CDC25B	1373.124687	1753.8291	992.4202555	-0.822445676	0.075776707	-10.853542	1.92E-27	1.93E-26
ENSG00000100027	YPEL1	112.3100632	143.54292	81.0772066	-0.822959872	0.243046394	-3.3860197	0.000709	0.001909
ENSG00000182810	DDX28	232.7029565	297.28732	168.1185888	-0.823271054	0.155204585	-5.3044248	1.13E-07	4.52E-07
ENSG00000112144	ICK	725.5966585	927.28731	523.9060112	-0.8234943	0.093907675	-8.7691906	1.80E-18	1.32E-17
ENSG00000076356	PLXNA2	19739.53278	25237.447	14241.61844	-0.825457027	0.036632771	-22.533295	1.96E-112	7.20E-111
ENSG00000125257	ABCC4	1904.13793	2434.1979	1374.077921	-0.825559216	0.06347033	-13.00701	1.12E-38	1.49E-37
ENSG00000198755	RPL10A	12724.91016	16270.17	9179.650085	-0.825777293	0.043358804	-19.045204	7.20E-81	1.92E-79
ENSG00000241749	RPSAP52	54.27329787	69.335814	39.21078153	-0.826161513	0.315787127	-2.6161976	0.008892	0.020091
ENSG00000143126	CELSR2	70.03389963	89.630239	50.43756013	-0.826169462	0.290736417	-2.8416442	0.004488	0.010671
ENSG00000149136	SSRP1	4621.934862	5910.6412	3333.228528	-0.826462816	0.048454657	-17.056417	3.13E-65	6.71E-64
ENSG00000259953	AL138756.1	147.0510531	188.23683	105.865276	-0.827351998	0.205887436	-4.0184676	5.86E-05	0.000181
ENSG00000073536	NLE1	468.4714703	599.38358	337.5593582	-0.827850718	0.110754338	-7.4746573	7.74E-14	4.58E-13

ENSG00000240891	PLCXD2	130.9632064	167.54132	94.3850886	-0.828881861	0.207668572	-3.9913688	6.57E-05	0.000201
ENSG00000146909	NOM1	1156.379186	1480.1708	832.587608	-0.829394008	0.081122376	-10.223986	1.55E-24	1.41E-23
ENSG00000137154	RPS6	25476.77338	32607.453	18346.0939	-0.829748332	0.038397192	-21.60961	1.46E-103	4.94E-102
ENSG00000168071	CCDC88B	49.9617189	63.957095	35.96634231	-0.830280562	0.337558874	-2.4596615	0.013907	0.030297
ENSG00000168569	TMEM223	198.0241171	253.64197	142.4062617	-0.830397329	0.168042899	-4.9415794	7.75E-07	2.89E-06
ENSG00000131089	ARHGEF9	319.6403424	409.36996	229.9107293	-0.83044092	0.141059817	-5.8871544	3.93E-09	1.76E-08
ENSG00000165424	ZCCHC24	559.6981087	716.64976	402.7464547	-0.831306696	0.098042693	-8.4790276	2.27E-17	1.59E-16
ENSG00000063046	EIF4B	15183.77606	19444.614	10922.938	-0.8319167	0.038183543	-21.78731	3.06E-105	1.06E-103
ENSG00000164938	TP53INP1	2662.92528	3410.409	1915.441519	-0.831919245	0.066493668	-12.511255	6.48E-36	8.08E-35
ENSG00000128602	SMO	288.297004	369.4624	207.1316044	-0.832918015	0.137650174	-6.0509769	1.44E-09	6.63E-09
ENSG00000124787	RPP40	208.3537473	266.75266	149.9548336	-0.833128413	0.174800455	-4.7661684	1.88E-06	6.77E-06
ENSG00000083457	ITGAE	295.4996635	378.45525	212.5440771	-0.833931125	0.137619978	-6.0596662	1.36E-09	6.29E-09
ENSG00000160298	C21orf58	54.5780963	70.028824	39.12736844	-0.833935734	0.362445774	-2.3008565	0.0214	0.044748
ENSG00000214026	MRPL23	168.5207045	215.87034	121.1710643	-0.835204126	0.182029774	-4.588283	4.47E-06	1.55E-05
ENSG00000281398	SNHG4	143.4877306	183.91183	103.0636271	-0.836231403	0.187229438	-4.4663457	7.96E-06	2.69E-05
ENSG00000185730	ZNF696	172.1376854	220.57001	123.7053587	-0.836680793	0.178675576	-4.6826814	2.83E-06	1.00E-05
ENSG00000153944	MSI2	852.344744	1092.7223	611.9671534	-0.83725272	0.085351335	-9.8094859	1.02E-22	8.82E-22
ENSG00000180318	ALX1	47.1159857	60.462417	33.76955443	-0.837279712	0.360492745	-2.322598	0.020201	0.042491
ENSG00000138738	PRDM5	83.83284865	107.62423	60.04147206	-0.83729567	0.293386426	-2.8539005	0.004319	0.010305
ENSG00000160789	LMNA	20074.37786	25744.809	14403.94631	-0.83790357	0.044517671	-18.821819	5.00E-79	1.30E-77
ENSG00000184117	NIPSNAP1	1165.25606	1494.3713	836.1408369	-0.837909402	0.077764018	-10.775027	4.52E-27	4.47E-26
ENSG00000149476	TKFC	547.3566599	701.92521	392.788108	-0.838347966	0.117028431	-7.1636265	7.86E-13	4.44E-12
ENSG00000143919	CAMKMT	87.39650325	112.05123	62.74177657	-0.838881482	0.244646362	-3.4289555	0.000606	0.001648
ENSG00000185950	IRS2	70.76967143	90.829232	50.71011067	-0.839964256	0.270519443	-3.1050051	0.001903	0.004804
ENSG00000122386	ZNF205	485.9788635	623.74448	348.2132477	-0.841393693	0.115953005	-7.2563337	3.98E-13	2.27E-12
ENSG00000108064	TFAM	1093.382578	1403.4647	783.3004325	-0.841532463	0.075602056	-11.131079	8.86E-29	9.21E-28
ENSG00000160172	FAM86C2P	61.99436639	79.609987	44.37874533	-0.841721003	0.281417079	-2.991009	0.002781	0.006847
ENSG00000234851	RPL23AP42	150.3514466	192.94912	107.753772	-0.842318237	0.191960368	-4.3879799	1.14E-05	3.81E-05
ENSG00000156802	ATAD2	587.434799	754.37867	420.4909319	-0.842359264	0.105717275	-7.968038	1.61E-15	1.04E-14
ENSG00000099953	MMP11	317.0983652	407.39128	226.8054493	-0.843219379	0.134809568	-6.2548927	3.98E-10	1.91E-09
ENSG00000152455	SUV39H2	285.4277553	366.74165	204.113864	-0.843909218	0.137625861	-6.1319087	8.68E-10	4.06E-09
ENSG00000214756	CSKMT	72.91038685	93.738373	52.08240088	-0.84517296	0.266760559	-3.1682831	0.001533	0.003929
ENSG00000108578	BLMH	1893.850603	2433.2633	1354.437886	-0.845271942	0.059557186	-14.19261	1.02E-45	1.58E-44
ENSG00000086200	IPO11	1948.206667	2502.9588	1393.45455	-0.845312486	0.059072749	-14.309686	1.90E-46	2.99E-45
ENSG00000165304	MELK	536.1571603	689.04569	383.2686309	-0.845344882	0.106370885	-7.9471454	1.91E-15	1.23E-14
ENSG00000149260	CAPN5	913.9771629	1174.4069	653.5474381	-0.846451782	0.099091033	-8.5421632	1.32E-17	9.32E-17
ENSG00000155085	AK9	118.0911728	151.79728	84.38506351	-0.846534103	0.214915442	-3.9389171	8.19E-05	0.000248
ENSG00000139405	RITA1	576.8022455	741.39045	412.2140397	-0.84693395	0.1117373	-7.5796887	3.46E-14	2.09E-13
ENSG00000107957	SH3PXD2A	2410.443471	3098.6428	1722.244182	-0.847019221	0.056147995	-15.085476	2.02E-51	3.45E-50
ENSG00000013573	DDX11	277.0574657	356.28927	197.825659	-0.848659533	0.138370019	-6.1332617	8.61E-10	4.03E-09
ENSG00000105750	ZNF85	103.1058534	132.70416	73.50755179	-0.848948834	0.234510057	-3.6200956	0.000294	0.000835
ENSG00000272325	NUDT3	1420.258005	1826.6185	1013.897495	-0.849674752	0.067278715	-12.629176	1.46E-36	1.84E-35
ENSG00000104998	IL27RA	265.5117177	341.47041	189.5530247	-0.849688451	0.144516967	-5.8795065	4.11E-09	1.84E-08
ENSG00000180900	SCRIB	2999.985466	3858.6089	2141.362008	-0.849772233	0.066113715	-12.853191	8.25E-38	1.08E-36
ENSG00000072201	LNX1	217.9630134	280.44834	155.4776914	-0.849847014	0.159511286	-5.3278175	9.94E-08	3.99E-07
ENSG00000112541	PDE10A	927.4439655	1192.76	662.1278957	-0.849912581	0.083085813	-10.229335	1.47E-24	1.34E-23
ENSG00000183091	NEB	50.71256112	65.124753	36.30036942	-0.851185743	0.370493766	-2.2974361	0.021594	0.045114
ENSG00000156049	GNA14	298.8571678	384.53288	213.1814536	-0.851898804	0.132855533	-6.4122192	1.43E-10	7.10E-10
ENSG00000005187	ACSM3	630.2905653	811.36852	449.2126152	-0.852414668	0.124582213	-6.8421859	7.80E-12	4.18E-11
ENSG00000058600	POLR3E	689.2656365	887.35344	491.177834	-0.853741748	0.090447637	-9.4390719	3.76E-21	3.07E-20
ENSG00000132646	PCNA	1603.589057	2065.0775	1142.100653	-0.854297885	0.064501766	-13.244566	4.85E-40	6.66E-39
ENSG00000148019	CEP78	729.2464607	939.39072	519.1021982	-0.854336687	0.095037214	-8.9894964	2.48E-19	1.89E-18
ENSG00000267041	ZNF850	74.13720962	95.551059	52.72336006	-0.854337132	0.272193619	-3.1387111	0.001697	0.004314
ENSG00000107554	DNMBP	3852.171312	4963.0223	2741.320351	-0.856589354	0.047425897	-18.061637	6.39E-73	1.54E-71
ENSG00000099875	MKNK2	3496.51963	4505.3008	2487.738482	-0.856990668	0.060818298	-14.091	4.31E-45	6.60E-44
ENSG00000160208	RRP1B	2058.729365	2652.8995	1464.559267	-0.85731934	0.062308776	-13.759207	4.48E-43	6.63E-42
ENSG00000244398	AC116533.1	159.3800755	205.35695	113.4032031	-0.85790686	0.19292015	-4.4469531	8.71E-06	2.93E-05
ENSG00000164985	PSIP1	1696.679461	2187.5793	1205.779573	-0.858757444	0.072525105	-11.84083	2.40E-32	2.74E-31
ENSG00000175390	EIF3F	7577.605845	9770.3881	5384.823546	-0.859681867	0.040770496	-21.085882	1.07E-98	3.46E-97
ENSG00000185015	CA13	107.2174773	138.30911	76.12584058	-0.85983555	0.231502689	-3.7141493	0.000204	0.000589
ENSG00000168661	ZNF30	68.16486342	87.913838	48.41588886	-0.860491996	0.289280395	-2.9745949	0.002934	0.007201
ENSG00000106772	PRUNE2	1050.983506	1355.7227	746.2443566	-0.860506654	0.084243396	-10.21453	1.71E-24	1.55E-23
ENSG00000100749	VRK1	355.3431568	458.16775	252.5185644	-0.860868332	0.128280878	-6.7108079	1.94E-11	1.02E-10
ENSG00000247315	ZCCHC3	688.1444883	887.55772	488.7312576	-0.861643012	0.099435418	-8.6653532	4.50E-18	3.24E-17
ENSG00000160352	ZNF714	173.1241481	223.3993	122.8489925	-0.861740213	0.180217727	-4.7816618	1.74E-06	6.29E-06
ENSG00000146410	MTFR2	58.15057096	74.988137	41.31300541	-0.86247172	0.298466069	-2.889681	0.003856	0.009295
ENSG00000167775	CD320	1380.41483	1780.9404	979.8892746	-0.862527138	0.0805546	-10.70736	9.40E-27	9.26E-26
ENSG00000181804	SLC9A9	61.06165527	78.81405	43.30926076	-0.862697218	0.31441932	-2.7437793	0.006074	0.014116
ENSG00000171492	LRRC8D	1002.068895	1293.213	710.9247791	-0.863051152	0.078894655	-10.939285	7.48E-28	7.60E-27
ENSG00000172465	TCEAL1	281.9220784	363.92198	199.9221806	-0.863084396	0.149975952	-5.7548186	8.67E-09	3.77E-08
ENSG00000166788	SAAL1	386.6177313	498.97697	274.2584973	-0.863502496	0.12413036	-6.9564166	3.49E-12	1.92E-11
ENSG00000110107	PRPF19	3537.463389	4566.7325	2508.194265	-0.864938483	0.058338526	-14.826197	9.92E-50	1.65E-48
ENSG00000234961	AL133415.1	40.8483628	52.776123	28.9206024	-0.865453279	0.353322914	-2.4494683	0.014307	0.031067
ENSG00000106588	PSMA2	48.0098906	62.04765	33.97213138	-0.865790784	0.3541615	-2.4446214	0.0145	0.031463
ENSG00000170836	PPM1D	705.7612702	911.38667	500.1358724	-0.865837317	0.093798289	-9.2308434	2.69E-20	2.12E-19
ENSG00000176692	FOXC2	875.704784	1130.5986	620.8110175	-0.866235342	0.123157721	-7.0335448	2.01E-12	1.12E-11
ENSG00000198542	ITGBL1	372.4161006	481.17927	263.6529267	-0.867182481	0.122477076	-7.0803657	1.44E-12	8.03E-12
ENSG00000139734	DIAPH3	783.4170458	1012.5378	554.2963096	-0.868427491	0.092414501	-9.3970912	5.61E-21	4.54E-20
ENSG00000151689	INPP1	900.9498121	1164.3187	637.5809112	-0.868536058	0.084990801	-10.219177	1.63E-24	1.48E-23
ENSG00000147604	RPL7	24789.17266	32035.891	17542.45416	-0.86884941	0.042870853	-20.266669	2.53E-91	7.62E-90
ENSG00000173068	BNC2	1317.327851	1702.2732	932.3825436	-0.868974041	0.080046234	-10.855902	1.87E-27	1.88E-26
ENSG00000184992	BRI3BP	293.577562	379.27721	207.8779133	-0.869500031	0.145221312	-5.9874134	2.13E-09	9.71E-09
ENSG00000182165	TP53TG1	351.5347358	454.47864	248.5908312	-0.870319559	0.122317072	-7.1152746	1.12E-12	6.26E-12
ENSG00000187840	EIF4EBP1	1671.302494	2160.4272	1182.177761	-0.870619017	0.073919324	-11.777962	5.07E-32	5.72E-31
ENSG00000003400	CASP10	902.5785469	1167.7844	637.3726696	-0.872779121	0.082736539	-10.548896	5.14E-26	4.94E-25

ENSG00000172728	FUT10	327.411804	423.76988	231.0537326	-0.874166896	0.131392891	-6.6530761	2.87E-11	1.49E-10
ENSG00000132383	RPA1	2668.937253	3453.5689	1884.305588	-0.874481122	0.061028998	-14.328945	1.44E-46	2.27E-45
ENSG00000119718	EIF2B2	2441.304835	3159.0924	1723.517266	-0.874721459	0.064504214	-13.560687	6.85E-42	9.85E-41
ENSG00000144040	SFXN5	431.7477495	558.7215	304.7739999	-0.875982948	0.117887298	-7.4306814	1.08E-13	6.34E-13
ENSG00000188483	IER5L	147.497953	190.88119	104.1147203	-0.876096999	0.19466978	-4.5004263	6.78E-06	2.31E-05
ENSG00000108561	C1QBP	2879.708191	3728.1731	2031.2433	-0.876643699	0.066459639	-13.190618	9.94E-40	1.35E-38
ENSG00000158352	SHROOM4	3845.819404	4980.0514	2711.587376	-0.876736164	0.049472934	-17.721532	2.86E-70	6.59E-69
ENSG00000173457	PPP1R14B	3730.157961	4829.8079	2630.50807	-0.877015035	0.074630061	-11.751498	6.94E-32	7.79E-31
ENSG00000161980	POLR3K	306.2308224	396.64089	215.8207502	-0.877106652	0.136085663	-6.4452539	1.15E-10	5.74E-10
ENSG00000170855	TRIAP1	1090.678767	1413.0088	768.3487221	-0.878009849	0.086025861	-10.206348	1.86E-24	1.69E-23
ENSG00000081181	ARG2	252.4819703	327.01262	177.9513191	-0.878486021	0.146746977	-5.9863994	2.15E-09	9.77E-09
ENSG00000164818	DNAAF5	1180.528554	1528.944	832.1130816	-0.878630006	0.077895411	-11.279612	1.65E-29	1.75E-28
ENSG00000150477	KIAA1328	109.5437366	141.79748	77.28999246	-0.878731098	0.227210569	-3.8674746	0.00011	0.000329
ENSG00000185900	POMK	144.1479617	186.75009	101.5458284	-0.879515285	0.233191917	-3.7716371	0.000162	0.000473
ENSG00000137700	SLC37A4	406.9680214	527.47073	286.4653174	-0.880224226	0.121077483	-7.269925	3.60E-13	2.06E-12
ENSG00000188352	FOCAD	3073.536366	3983.3536	2163.719097	-0.880282264	0.053797978	-16.362739	3.53E-60	6.94E-59
ENSG00000261373	VPS9D1-AS1	198.2449148	256.76449	139.7253431	-0.880493722	0.179776729	-4.8977069	9.70E-07	3.59E-06
ENSG00000183763	TRAIP	45.20195378	58.625769	31.77813827	-0.880515644	0.337243837	-2.6109169	0.00903	0.020381
ENSG00000080839	RBL1	252.9171435	327.73901	178.09528	-0.88054422	0.152907665	-5.7586663	8.48E-09	3.69E-08
ENSG00000227992	AC108463.1	46.45253668	60.252965	32.65210795	-0.881254256	0.350664045	-2.5131013	0.011967	0.026381
ENSG00000163002	NUP35	148.3697091	192.40312	104.3362981	-0.881657414	0.215023368	-4.1002865	4.13E-05	0.00013
ENSG00000157514	TSC22D3	642.8775592	833.27541	452.4797037	-0.882172481	0.111053386	-7.9436792	1.96E-15	1.26E-14
ENSG00000165501	LRR1	321.0394659	416.04053	226.0384051	-0.882558397	0.140420361	-6.285117	3.28E-10	1.58E-09
ENSG00000105711	SCN1B	551.6581995	715.354	387.962396	-0.882705854	0.102615175	-8.6020986	7.83E-18	5.58E-17
ENSG00000139880	CDH24	485.7177685	629.70257	341.7329696	-0.882782426	0.115339519	-7.6537724	1.95E-14	1.19E-13
ENSG00000175832	ETV4	312.6314759	405.28067	219.9822818	-0.883282268	0.140746264	-6.2757067	3.48E-10	1.67E-09
ENSG00000136159	NUDT15	388.9478606	504.52955	273.3661738	-0.883318212	0.124489951	-7.0954981	1.29E-12	7.21E-12
ENSG00000185909	KLHDC8B	1292.972205	1677.3959	908.5485457	-0.883961414	0.069885502	-12.648709	1.14E-36	1.44E-35
ENSG00000165507	DEPP1	10899.45657	14140.818	7658.09543	-0.884861458	0.04049067	-21.853466	7.21E-106	2.50E-104
ENSG00000239900	ADSL	81.54869916	105.86324	57.23415856	-0.885162112	0.249472305	-3.5481378	0.000388	0.001082
ENSG00000077348	EXOSC5	226.7528902	294.40244	159.1033411	-0.886700771	0.17335635	-5.1149022	3.14E-07	1.21E-06
ENSG00000186364	NUDT17	53.99595677	70.053906	37.93800724	-0.887238119	0.308243756	-2.8783653	0.003997	0.0096
ENSG00000129347	KRI1	566.9930799	735.95939	398.0267675	-0.887399415	0.098161112	-9.0402339	1.56E-19	1.20E-18
ENSG00000198890	PRMT6	486.7814095	632.11401	341.4488079	-0.887497345	0.11274192	-7.8719375	3.49E-15	2.21E-14
ENSG00000130520	LSM4	1147.61481	1490.4031	804.8265343	-0.889055041	0.074037561	-12.008162	3.22E-33	3.75E-32
ENSG00000132780	NASP	1739.492341	2259.9897	1218.995001	-0.889807489	0.065453176	-13.594566	4.31E-42	6.23E-41
ENSG00000137693	YAP1	971.7861402	1262.1629	681.4094167	-0.889974517	0.086050204	-10.342503	4.53E-25	4.22E-24
ENSG00000100784	RPS6KA5	244.3763042	317.28351	171.4690999	-0.890095486	0.175006495	-5.0860712	3.66E-07	1.40E-06
ENSG00000250903	GMDS-DT	48.99906464	63.768359	34.22976988	-0.891145092	0.364342567	-2.4458989	0.014449	0.031362
ENSG00000150995	ITPR1	627.1167849	814.85731	439.3762646	-0.891242042	0.100997294	-8.824415	1.10E-18	8.16E-18
ENSG00000072954	TMEM38A	82.48769151	107.1687	57.8066824	-0.891965212	0.245265259	-3.6367369	0.000276	0.000786
ENSG00000004660	CAMKK1	152.2742032	197.87476	106.6736427	-0.892329133	0.183489978	-4.8630947	1.16E-06	4.25E-06
ENSG00000142864	SERBP1	10763.87699	13990.547	7537.206725	-0.892417686	0.040615963	-21.972092	5.33E-107	1.86E-105
ENSG00000138796	HADH	425.9612064	553.89722	298.0251943	-0.893104051	0.119546526	-7.4707654	7.97E-14	4.71E-13
ENSG00000166037	CEP57	979.0635314	1272.8238	685.3032464	-0.893487107	0.084184541	-10.613434	2.58E-26	2.51E-25
ENSG00000088836	SLC4A11	284.8608687	370.43141	199.2903279	-0.89362332	0.142030993	-6.2917487	3.14E-10	1.52E-09
ENSG00000163827	LRRC2	42.6300708	55.41334	29.84680129	-0.894265956	0.337383871	-2.6505889	0.008035	0.018301
ENSG00000167972	ABCA3	5108.911887	6643.8395	3573.984226	-0.894899551	0.053329205	-16.780665	3.38E-63	6.99E-62
ENSG00000226084	AC113935.1	50.66218765	65.944681	35.37969434	-0.895413817	0.337361238	-2.6541692	0.00795	0.018132
ENSG00000122406	RPL5	18429.50719	23981.92	12877.09424	-0.897103294	0.037797633	-23.734378	1.59E-124	6.58E-123
ENSG00000083720	OXCT1	1134.013038	1475.4287	792.6333322	-0.897316054	0.077057151	-11.644812	2.44E-31	2.70E-30
ENSG00000155858	LSM11	445.1682897	579.27377	311.0628144	-0.897386146	0.118797312	-7.5539263	4.22E-14	2.53E-13
ENSG00000117791	2-Mar	198.0523574	257.95868	138.1460396	-0.897596382	0.179837177	-4.9911614	6.00E-07	2.26E-06
ENSG00000105538	RASIP1	2678.07945	3485.1437	1871.015207	-0.8980318	0.075543161	-11.887665	1.37E-32	1.57E-31
ENSG00000278619	MRM1	94.87846809	123.44891	66.30802233	-0.89843417	0.23753602	-3.7823071	0.000155	0.000455
ENSG00000188191	PRKAR1B	691.8734323	900.82983	482.9170352	-0.898833921	0.092772614	-9.6885695	3.37E-22	2.85E-21
ENSG00000224546	EIF4BP3	86.72383217	112.8773	60.57036702	-0.899297961	0.250224624	-3.5939627	0.000326	0.000917
ENSG00000196502	SULT1A1	100.2114287	130.50697	69.91589012	-0.899703874	0.248886967	-3.6149095	0.0003	0.000851
ENSG00000135972	MRPS9	577.9439251	752.62838	403.2594713	-0.899772728	0.108723376	-8.2757983	1.28E-16	8.68E-16
ENSG00000113719	ERGIC1	5721.707818	7452.2768	3991.138866	-0.901331323	0.055421465	-16.263217	1.80E-59	3.50E-58
ENSG00000136235	GPNMB	121.8048511	158.79977	84.80993108	-0.901951714	0.22497646	-4.0090937	6.10E-05	0.000188
ENSG00000171552	BCL2L1	6209.130158	8089.3412	4328.919154	-0.902308315	0.060090524	-15.015817	5.78E-51	9.84E-50
ENSG00000126970	ZC4H2	297.69549	388.01443	207.3765538	-0.902503519	0.141167641	-6.3931331	1.63E-10	8.01E-10
ENSG00000186298	PPP1CC	3529.457248	4599.8982	2459.016329	-0.903262855	0.057002053	-15.846146	1.49E-56	2.77E-55
ENSG00000245958	AC093752.1	99.28526935	129.39792	69.17262026	-0.903273005	0.223142807	-4.0479593	5.17E-05	0.000161
ENSG00000185112	FAM43A	8071.792501	10519.615	5623.970437	-0.903586597	0.055343119	-16.326991	6.34E-60	1.24E-58
ENSG00000123975	CKS2	700.6542241	912.99367	488.3147743	-0.904319428	0.097553411	-9.2699929	1.86E-20	1.48E-19
ENSG00000109458	GAB1	425.9212468	555.37027	296.4722261	-0.90443813	0.126667728	-7.1402412	9.32E-13	5.24E-12
ENSG00000254122	PCDHGB7	403.9355443	526.48469	281.3863938	-0.905024521	0.128780483	-7.0276528	2.10E-12	1.16E-11
ENSG00000185033	SEMA4B	664.8407709	866.63778	463.043765	-0.9059036	0.098564634	-9.1909599	3.89E-20	3.06E-19
ENSG00000189046	ALKBH2	118.5191937	154.72805	82.31033755	-0.907423613	0.214857483	-4.2233745	2.41E-05	7.76E-05
ENSG00000169252	ADRB2	94.6570832	123.43463	65.87953451	-0.907870761	0.237705581	-3.8193077	0.000134	0.000396
ENSG00000074071	MRPS34	1146.901317	1496.2914	797.5112547	-0.908467496	0.086309404	-10.525707	6.58E-26	6.29E-25
ENSG00000145220	LYAR	744.5973286	971.53369	517.6609664	-0.908763343	0.087921284	-10.336102	4.84E-25	4.50E-24
ENSG00000143819	EPHX1	870.5185501	1135.6682	605.3688646	-0.908797478	0.091783235	-9.9015629	4.10E-23	3.58E-22
ENSG00000204556	AL450124.1	49.74166271	64.89913	34.58419527	-0.90902463	0.313157003	-2.902776	0.003699	0.008937
ENSG00000082516	GEMIN5	1066.996769	1392.5525	741.441032	-0.909706399	0.078755603	-11.551005	7.30E-31	7.97E-30
ENSG00000005022	SLC25A5	5961.104813	7781.6848	4140.524832	-0.9102911	0.045572209	-19.974698	9.14E-89	2.66E-87
ENSG00000172878	METAP1D	80.21978975	104.72662	55.71295555	-0.911408223	0.262442404	-3.4727933	0.000515	0.001414
ENSG00000145284	SCD5	2263.768386	2956.6177	1570.919102	-0.912108484	0.05802165	-15.72014	1.10E-55	2.02E-54
ENSG00000172667	ZMAT3	5706.539258	7453.4906	3959.587951	-0.912883952	0.053123155	-17.184295	3.48E-66	7.62E-65
ENSG00000182054	IDH2	2146.604324	2804.1004	1489.108221	-0.913605786	0.063097738	-14.479216	1.64E-47	2.63E-46
ENSG00000163931	TKT	10891.36511	14229.288	7553.442295	-0.913796479	0.054413779	-16.793476	2.72E-63	5.64E-62
ENSG00000178035	IMPDH2	2936.42437	3836.6606	2036.188157	-0.914049368	0.052913936	-17.274265	7.35E-67	1.62E-65
ENSG00000239672	NME1	446.2272916	582.98144	309.4731479	-0.914106451	0.118265068	-7.7293022	1.08E-14	6.70E-14

ENSG00000197744	PTMAP2	35.5512042	46.470923	24.63148505	-0.914113321	0.378863532	-2.4127773	0.015831	0.034087
ENSG00000283041	AC008038.1	300.0489611	391.88469	208.2132344	-0.914465523	0.153687416	-5.9501652	2.68E-09	1.21E-08
ENSG00000182774	RPS17	81.38824402	106.31528	56.46120548	-0.91501672	0.286094225	-3.1983055	0.001382	0.003567
ENSG00000137269	LRRC1	252.623815	330.22028	175.0273548	-0.915385347	0.158151875	-5.7880145	7.12E-09	3.12E-08
ENSG00000119685	TTLL5	1995.072466	2607.5213	1382.62365	-0.915451368	0.061120012	-14.977932	1.02E-50	1.74E-49
ENSG00000171132	PRKCE	989.2649779	1293.2474	685.282557	-0.916216786	0.088182273	-10.390034	2.75E-25	2.58E-24
ENSG00000125457	MIF4GD	220.7287929	288.44547	153.0121141	-0.916364419	0.156267131	-5.8640893	4.52E-09	2.01E-08
ENSG00000125901	MRPS26	572.6257692	748.65079	396.6007474	-0.917192289	0.111834649	-8.2013249	2.38E-16	1.60E-15
ENSG00000156471	PTDSS1	2706.962107	3540.5682	1873.356048	-0.918003171	0.061324681	-14.969555	1.16E-50	1.97E-49
ENSG00000048991	R3HDM1	1324.21236	1731.9141	916.5106652	-0.918151144	0.07305927	-12.567209	3.20E-36	4.02E-35
ENSG00000166897	ELFN2	61.19286336	79.963734	42.42199244	-0.918635949	0.322591579	-2.8476749	0.004404	0.010482
ENSG00000122966	CIT	424.2375072	554.94926	293.5257534	-0.919151257	0.122922144	-7.4775075	7.57E-14	4.48E-13
ENSG00000178896	EXOSC4	190.0700207	248.66194	131.4780966	-0.919457031	0.175680464	-5.2336897	1.66E-07	6.54E-07
ENSG00000171700	RGS19	472.4860904	618.11809	326.8540932	-0.919529489	0.120197376	-7.6501628	2.01E-14	1.23E-13
ENSG00000256269	HMBS	348.5261108	456.14042	240.9118036	-0.920015725	0.123193502	-7.468054	8.14E-14	4.81E-13
ENSG00000078177	N4BP2	436.8838888	571.78306	301.9847209	-0.920533003	0.114415654	-8.0455162	8.59E-16	5.61E-15
ENSG00000173598	NUDT4	5019.206808	6570.0566	3468.356986	-0.921201935	0.05310264	-17.347573	2.06E-67	4.57E-66
ENSG00000189403	HMGB1	6017.932822	7880.7124	4155.15329	-0.923406028	0.047749191	-19.338674	2.54E-83	6.96E-82
ENSG00000094916	CBX5	2879.243805	3771.415	1987.072589	-0.924326599	0.055167009	-16.755061	5.20E-63	1.07E-61
ENSG00000103145	HCFC1R1	350.6792503	459.551	241.8075032	-0.92451034	0.142984449	-6.4658104	1.01E-10	5.03E-10
ENSG00000161682	FAM171A2	298.0876887	390.48772	205.6876531	-0.926409059	0.182963452	-5.0633558	4.12E-07	1.58E-06
ENSG00000099194	SCD	11072.03864	14510.944	7633.132943	-0.926782858	0.054736608	-16.931682	2.63E-64	5.56E-63
ENSG00000178031	ADAMTSL1	2594.998015	3400.6598	1789.336252	-0.927110038	0.061336109	-15.115241	1.29E-51	2.21E-50
ENSG00000227946	AC007383.2	44.75157553	58.740796	30.76235543	-0.92838195	0.35867632	-2.5883558	0.009644	0.021642
ENSG00000132423	COQ3	99.17928632	130.05003	68.30854498	-0.928927774	0.23256365	-3.9942948	6.49E-05	0.000199
ENSG00000165475	CRYL1	1576.942305	2069.0227	1084.861908	-0.930860608	0.070849693	-13.138527	1.98E-39	2.68E-38
ENSG00000171724	VAT1L	43.83162275	57.530557	30.13268804	-0.931184877	0.341007319	-2.7306888	0.00632	0.014654
ENSG00000112029	FBXO5	168.143535	220.58477	115.7023017	-0.931405267	0.192492137	-4.8386666	1.31E-06	4.79E-06
ENSG00000153574	RPIA	729.3958047	957.04061	501.7510018	-0.931613791	0.090232357	-10.324609	5.45E-25	5.05E-24
ENSG00000153885	KCTD15	1096.177193	1438.3156	754.038741	-0.931691628	0.075495841	-12.340966	5.45E-35	6.62E-34
ENSG00000175198	PCCA	398.1996575	522.72385	273.6754696	-0.932777505	0.116048618	-8.0378165	9.15E-16	5.96E-15
ENSG00000139354	GAS2L3	576.3619665	756.44834	396.275593	-0.93430903	0.119586223	-7.8128484	5.59E-15	3.50E-14
ENSG00000165512	ZNF22	1237.413219	1624.9763	849.8501774	-0.93483577	0.079013792	-11.831299	2.69E-32	3.06E-31
ENSG00000229638	RPL4P4	131.6857465	172.82438	90.54711779	-0.934858939	0.21194414	-4.4108742	1.03E-05	3.44E-05
ENSG00000130830	MPP1	926.4500954	1216.6427	636.2575117	-0.935719618	0.08056428	-11.614572	3.48E-31	3.82E-30
ENSG00000159733	ZFYVE28	371.2501369	487.56618	254.9340978	-0.935812146	0.129127331	-7.2472043	4.25E-13	2.43E-12
ENSG00000125510	OPRL1	107.2704257	140.89694	73.64391629	-0.935850245	0.224138815	-4.1753154	2.98E-05	9.51E-05
ENSG00000168393	DTYMK	649.840262	853.27655	446.40397	-0.936150568	0.104230584	-8.9815344	2.67E-19	2.03E-18
ENSG00000215883	CYB5RL	143.3003546	188.38399	98.21671953	-0.936299224	0.211101448	-4.4353046	9.19E-06	3.09E-05
ENSG00000280789	PAGR1	157.2916773	206.52817	108.0551802	-0.936455251	0.181222431	-5.1674356	2.37E-07	9.24E-07
ENSG00000127946	HIP1	2132.990783	2802.5243	1463.457229	-0.937254887	0.058227276	-16.096492	2.70E-58	5.15E-57
ENSG00000139675	HNRNPA1L2	102.1197032	134.15754	70.08186268	-0.937308836	0.233643536	-4.0117046	6.03E-05	0.000186
ENSG00000115380	EFEMP1	73798.45285	96978.254	50618.65142	-0.937981908	0.043712721	-21.45787	3.86E-102	1.29E-100
ENSG00000188856	RPSAP47	141.8497044	186.33393	97.3654743	-0.9387654	0.209322257	-4.4847854	7.30E-06	2.48E-05
ENSG00000198795	ZNF521	3109.218969	4088.3874	2130.05055	-0.940580201	0.050063088	-18.787898	9.49E-79	2.44E-77
ENSG00000279673	AC092919.2	51.56248396	67.819336	35.3056319	-0.941344865	0.307370374	-3.0625751	0.002194	0.005487
ENSG00000133574	GIMAP4	2042.32058	2686.6788	1397.962347	-0.942107492	0.067820791	-13.891131	7.17E-44	1.07E-42
ENSG00000095370	SH2D3C	2774.38647	3649.6817	1899.091268	-0.942611092	0.059546264	-15.829895	1.94E-56	3.58E-55
ENSG00000114346	ECT2	863.4681606	1136.2748	590.6615598	-0.943569521	0.085357785	-11.054288	2.09E-28	2.15E-27
ENSG00000131188	PRR7	107.1743938	140.92157	73.42721521	-0.943704024	0.231935666	-4.068818	4.73E-05	0.000148
ENSG00000213551	DNAJC9	1160.404485	1527.182	793.6269343	-0.94413192	0.078658103	-12.002984	3.43E-33	3.98E-32
ENSG00000050820	BCAR1	7463.55149	9822.4531	5104.649858	-0.944471183	0.062287433	-15.16311	6.21E-52	1.07E-50
ENSG00000149564	ESAM	11552.62857	15204.528	7900.728725	-0.944598981	0.041407078	-22.812501	3.45E-115	1.31E-113
ENSG00000126953	TIMM8A	209.2672259	275.26186	143.272588	-0.944681235	0.170790608	-5.5312247	3.18E-08	1.33E-07
ENSG00000126249	PDCD2L	53.01080292	69.8315	36.19010545	-0.944815265	0.312452015	-3.0238732	0.002496	0.006187
ENSG00000139679	LPAR6	378.7293901	498.63853	258.8202465	-0.946399152	0.120276892	-7.8685036	3.59E-15	2.27E-14
ENSG00000169570	DTWD2	62.10945751	81.842878	42.37603738	-0.946803649	0.301686626	-3.138368	0.001699	0.004317
ENSG00000177030	DEAF1	1578.643402	2079.1031	1078.183677	-0.947282744	0.066455508	-14.25439	4.21E-46	6.57E-45
ENSG00000197380	DACT3	76.28630763	100.41178	52.16083392	-0.948359967	0.268784985	-3.528322	0.000418	0.001161
ENSG00000187514	PTMA	19916.93945	26237.345	13596.53408	-0.948371931	0.036726395	-25.822625	4.94E-147	2.38E-145
ENSG00000137970	RPL7P9	166.8699334	219.78747	113.9523931	-0.948895879	0.179619621	-5.2828075	1.27E-07	5.06E-07
ENSG00000166669	ATF7IP2	134.9139686	177.69047	92.13746889	-0.948950905	0.194083941	-4.8893839	1.01E-06	3.74E-06
ENSG00000145425	RPS3A	18125.8828	23885.059	12366.70692	-0.949619008	0.044364312	-21.405021	1.20E-101	3.99E-100
ENSG00000174032	SLC25A30	962.1067311	1268.0692	656.1442966	-0.949856956	0.082510305	-11.51198	1.15E-30	1.25E-29
ENSG00000228716	DHFR	825.6479866	1088.2428	563.0531684	-0.949959987	0.086687156	-10.958486	6.05E-28	6.16E-27
ENSG00000259781	HMGB1P6	148.3147623	195.58495	101.0445722	-0.950427437	0.203998985	-4.6589812	3.18E-06	1.12E-05
ENSG00000161653	NAGS	208.4700044	274.64363	142.2963828	-0.951973331	0.192691636	-4.9403978	7.80E-07	2.91E-06
ENSG00000112559	MDFI	783.16031	1032.7655	533.5551279	-0.952176632	0.088495616	-10.759591	5.34E-27	5.27E-26
ENSG00000106333	PCOLCE	46.47068458	61.245753	31.69561638	-0.952272954	0.35492977	-2.6829898	0.007297	0.016737
ENSG00000153774	CFDP1	719.7911664	949.16974	490.41259	-0.952827086	0.089817903	-10.608432	2.72E-26	2.64E-25
ENSG00000108384	RAD51C	327.3661631	431.69847	223.03386	-0.952978671	0.129927693	-7.3346848	2.22E-13	1.29E-12
ENSG00000179041	RRS1	428.3001478	564.58711	292.0131882	-0.953060732	0.118082658	-8.0711321	6.96E-16	4.57E-15
ENSG00000138119	MYOF	21040.52176	27759.166	14321.87758	-0.954846128	0.037179023	-25.682389	1.84E-145	8.77E-144
ENSG00000212719	C17orf51	668.6974479	882.57256	454.8223373	-0.956006886	0.107194303	-8.9184486	4.73E-19	3.56E-18
ENSG00000100036	SLC35E4	773.3816	1020.3291	526.4341347	-0.956024104	0.097739203	-9.7813781	1.35E-22	1.16E-21
ENSG00000232956	SNHG15	154.594264	204.04107	105.1474596	-0.956291377	0.178998238	-5.3424625	9.17E-08	3.69E-07
ENSG00000072210	ALDH3A2	1137.121936	1501.0509	773.193004	-0.957256504	0.07307886	-13.098952	3.34E-39	4.49E-38
ENSG00000175611	LINC00476	58.95002916	77.807598	40.09246032	-0.957987597	0.301184424	-3.1807342	0.001469	0.003776
ENSG00000123219	CENPK	216.2873244	285.72599	146.8486624	-0.959406538	0.15614291	-6.1444131	8.03E-10	3.76E-09
ENSG00000132394	EEFSEC	306.0628756	404.36233	207.7634196	-0.960665516	0.142673008	-6.7333375	1.66E-11	8.73E-11
ENSG00000123473	STIL	339.6239246	448.88601	230.3618383	-0.961034413	0.137584742	-6.9850362	2.85E-12	1.57E-11
ENSG00000115758	ODC1	3688.703337	4874.1064	2503.300286	-0.961495822	0.067503953	-14.243548	4.92E-46	7.66E-45
ENSG00000025772	TOMM34	1321.088305	1746.087	896.0896203	-0.962009421	0.091126321	-10.556878	4.72E-26	4.54E-25
ENSG00000110921	MVK	428.2229599	565.84388	290.6020426	-0.963033767	0.137758068	-6.9907613	2.73E-12	1.51E-11
ENSG00000197971	MBP	76.15572312	100.70557	51.60587601	-0.963195412	0.268769031	-3.5837291	0.000339	0.000951

ENSG00000232527	AC245595.1	151.1527312	199.75851	102.5469528	-0.963222734	0.19896669	-4.8411256	1.29E-06	4.74E-06
ENSG00000136160	EDNRB	110.7243554	146.55894	74.88977398	-0.964672693	0.25045403	-3.8516956	0.000117	0.000349
ENSG00000114023	FAM162A	423.0159494	559.71503	286.3168732	-0.965914272	0.119068309	-8.11227	4.97E-16	3.28E-15
ENSG00000164125	GASK1B	11345.58358	15009.371	7681.796326	-0.96624636	0.056357331	-17.144999	6.85E-66	1.49E-64
ENSG00000123384	LRP1	811.1426176	1073.2005	549.0847826	-0.966859768	0.085332268	-11.330529	9.26E-30	9.89E-29
ENSG00000171208	NETO2	794.7004427	1051.7288	537.6721018	-0.96708835	0.093226593	-10.373525	3.27E-25	3.07E-24
ENSG00000007944	MYLIP	424.6908192	562.06584	287.3158029	-0.9674054	0.121625312	-7.9539808	1.81E-15	1.16E-14
ENSG00000118420	UBE3D	97.89721183	129.46944	66.32497879	-0.967433231	0.233381717	-4.1452829	3.39E-05	0.000108
ENSG00000168028	RPSA	17501.2087	23165.951	11836.46629	-0.968850097	0.052425894	-18.480373	2.97E-76	7.40E-75
ENSG00000131370	SH3BP5	3500.615261	4637.4827	2363.747818	-0.972054176	0.054947992	-17.69044	4.97E-70	1.14E-68
ENSG00000169684	CHRNA5	224.3139611	297.12163	151.5062917	-0.972200186	0.161708423	-6.0120565	1.83E-09	8.38E-09
ENSG00000156017	CARNMT1	495.7532719	656.7136	334.7929446	-0.972303993	0.104565379	-9.298527	1.42E-20	1.13E-19
ENSG00000215458	AATBC	44.63103181	59.180207	30.08185622	-0.974168695	0.335804499	-2.9009995	0.00372	0.008984
ENSG00000141337	ARSG	104.037498	137.85616	70.21883664	-0.974570236	0.232660392	-4.1888103	2.80E-05	8.99E-05
ENSG00000275832	ARHGAP23	5125.195609	6792.622	3457.769227	-0.974602041	0.058049514	-16.789151	2.93E-63	6.06E-62
ENSG00000137760	ALKBH8	393.944387	522.50866	265.3801109	-0.976213845	0.127890913	-7.633176	2.29E-14	1.40E-13
ENSG00000205403	CFI	87.44691757	116.07356	58.82027994	-0.976433544	0.275004263	-3.5506124	0.000384	0.001073
ENSG00000113328	CCNG1	6643.482997	8809.7675	4477.198507	-0.976613684	0.050237858	-19.439795	3.56E-84	9.87E-83
ENSG00000008394	MGST1	1695.045843	2247.9108	1142.180877	-0.976679971	0.06770397	-14.425742	3.56E-47	5.68E-46
ENSG00000115756	HPCAL1	2034.151228	2697.3786	1370.923854	-0.977012913	0.071044135	-13.752197	4.94E-43	7.29E-42
ENSG00000274349	ZNF658	118.5388472	157.29728	79.78041377	-0.977968924	0.214432474	-4.5607314	5.10E-06	1.76E-05
ENSG00000141480	ARRB2	683.4958302	907.08891	459.9027521	-0.979107028	0.095653507	-10.235976	1.37E-24	1.25E-23
ENSG00000163507	CIP2A	355.9494409	472.35005	239.5488359	-0.979869617	0.137492771	-7.1266992	1.03E-12	5.77E-12
ENSG00000063180	CA11	75.26239159	99.979895	50.54488845	-0.980119169	0.272044243	-3.6027933	0.000315	0.000889
ENSG00000183814	LIN9	152.7071133	202.54536	102.8688626	-0.980694499	0.193230159	-5.0752662	3.87E-07	1.48E-06
ENSG00000242125	SNHG3	230.7458945	306.23768	155.2541067	-0.980782616	0.156698226	-6.2590537	3.87E-10	1.86E-09
ENSG00000171843	MLLT3	266.970383	354.37708	179.5636819	-0.98084726	0.154571424	-6.3455925	2.22E-10	1.08E-09
ENSG00000174684	B4GAT1	1097.066788	1456.3592	737.7743791	-0.981118971	0.077557707	-12.65018	1.12E-36	1.42E-35
ENSG00000168785	TSPAN5	2483.957937	3297.4133	1670.502616	-0.981192248	0.068173924	-14.392486	5.77E-47	9.12E-46
ENSG00000008283	CYB561	1917.853826	2545.8913	1289.816383	-0.981365693	0.065352094	-15.016591	5.72E-51	9.73E-50
ENSG00000124641	MED20	691.2026915	917.59342	464.8119618	-0.981756368	0.092676678	-10.593349	3.20E-26	3.10E-25
ENSG00000149636	DSN1	373.6280961	496.21452	251.0416749	-0.981880047	0.120324034	-8.1602986	3.34E-16	2.22E-15
ENSG00000237945	LINC00649	143.928611	191.1992	96.65802091	-0.981973078	0.204395003	-4.804291	1.55E-06	5.65E-06
ENSG00000118922	KLF12	586.8812947	779.34885	394.4137412	-0.982490188	0.099731345	-9.8513681	6.76E-23	5.84E-22
ENSG00000089163	SIRT4	28.2995133	37.622637	18.9763892	-0.982639416	0.424952904	-2.312349	0.020758	0.043567
ENSG00000067177	PHKA1	380.7918241	505.49805	256.0855981	-0.982642595	0.123301684	-7.9694175	1.59E-15	1.03E-14
ENSG00000076248	UNG	775.3117863	1029.339	521.2845999	-0.983089384	0.092050319	-10.679913	1.26E-26	1.24E-25
ENSG00000077312	SNRPA	1022.963755	1358.3548	687.5726994	-0.983151852	0.087873424	-11.188273	4.65E-29	4.87E-28
ENSG00000167670	CHAF1A	501.3750394	666.07598	336.6740968	-0.983467844	0.112661032	-8.7294411	2.56E-18	1.86E-17
ENSG00000108773	KAT2A	516.2438346	685.71747	346.770196	-0.98365249	0.10249515	-9.5970638	8.23E-22	6.84E-21
ENSG00000128050	PAICS	3408.311835	4529.4848	2287.138849	-0.985728415	0.064712166	-15.232505	2.15E-52	3.75E-51
ENSG00000128944	KNSTRN	484.0258806	643.20655	324.8452144	-0.986527631	0.110950121	-8.8916318	6.02E-19	4.51E-18
ENSG00000172167	MTBP	76.9615855	102.2883	51.6348746	-0.987108926	0.261345043	-3.7770333	0.000159	0.000464
ENSG00000100034	PPM1F	6717.756877	8930.4812	4505.032508	-0.987273504	0.046742454	-21.121559	5.04E-99	1.64E-97
ENSG00000125089	SH3TC1	4287.127543	5701.2041	2873.051012	-0.988549883	0.050230282	-19.680357	3.18E-86	8.98E-85
ENSG00000234009	RPL5P34	51.43524079	68.426093	34.44438896	-0.989098833	0.318142193	-3.1089835	0.001877	0.004744
ENSG00000130299	GTPBP3	232.6569324	309.5495	155.7643661	-0.989553209	0.155923022	-6.3464214	2.20E-10	1.07E-09
ENSG00000130713	EXOSC2	494.4255927	657.81799	331.0331908	-0.990124226	0.11248767	-8.8020689	1.34E-18	9.92E-18
ENSG00000142657	PGD	11500.66697	15299.337	7701.997405	-0.9902023	0.038997918	-25.391158	3.16E-142	1.46E-140
ENSG00000246263	UBR5-AS1	28.15291333	37.455767	18.85005999	-0.992175601	0.428419794	-2.3158958	0.020564	0.043192
ENSG00000204366	ZBTB12	60.66832219	80.788236	40.54840848	-0.992308478	0.314944057	-3.1507452	0.001629	0.004152
ENSG00000197594	ENPP1	263.6954232	351.11537	176.2754729	-0.992837995	0.15716521	-6.3171614	2.66E-10	1.29E-09
ENSG00000089280	FUS	5207.488347	6933.6958	3481.280863	-0.994034959	0.051559172	-19.279498	7.98E-83	2.18E-81
ENSG00000258017	AC011603.2	42.82409185	57.069927	28.57825672	-0.995154299	0.3466298	-2.8709427	0.004092	0.009816
ENSG00000117543	DPH5	421.7279722	561.98031	281.4756303	-0.995624553	0.125390304	-7.9402036	2.02E-15	1.29E-14
ENSG00000105875	WDR91	217.8816604	290.27566	145.487664	-0.995663227	0.156691637	-6.3542844	2.09E-10	1.02E-09
ENSG00000149054	ZNF215	80.46834514	107.14453	53.79216002	-0.996272335	0.27704479	-3.5960695	0.000323	0.00091
ENSG00000073584	SMARCE1	112.8650476	150.32415	75.4059422	-0.996706098	0.248828568	-4.0055935	6.19E-05	0.00019
ENSG00000101868	POLA1	390.0444251	519.79759	260.2912647	-0.997051099	0.12367383	-8.0619409	7.51E-16	4.92E-15
ENSG00000280832	GSEC	84.46378748	112.6337	56.29387312	-0.998589158	0.273821376	-3.6468634	0.000265	0.000758
ENSG00000224167	LINC01357	52.05663245	69.345494	34.76777133	-0.998656325	0.319394257	-3.1267197	0.001768	0.004481
ENSG00000233426	EIF3FP3	28.77134236	38.312136	19.23054832	-0.998952688	0.427557407	-2.3364177	0.019469	0.041124
ENSG00000068489	PRR11	1160.710566	1547.1871	774.2340618	-0.999156272	0.089122633	-11.211027	3.60E-29	3.78E-28
ENSG00000149823	VPS51	1989.508291	2651.6665	1327.350061	-0.9991957	0.075208495	-13.285676	2.80E-40	3.86E-39
ENSG00000249115	HAUS5	162.1051455	216.10503	108.1052617	-0.999454962	0.179782865	-5.5592337	2.71E-08	1.14E-07
ENSG00000109685	NSD2	3119.053195	4159.0614	2079.044949	-1.000411823	0.053168867	-18.815745	5.61E-79	1.46E-77
ENSG00000172031	EPHX4	236.2666571	315.13687	157.3964425	-1.000516941	0.159410101	-6.276371	3.47E-10	1.67E-09
ENSG00000119699	TGFB3	60.62696476	80.844539	40.40939009	-1.00100451	0.288592558	-3.4685735	0.000523	0.001435
ENSG00000169291	SHE	4043.07583	5394.9778	2691.173854	-1.003173741	0.051375308	-19.526379	6.55E-85	1.83E-83
ENSG00000169083	AR	492.7181148	657.43064	328.0055935	-1.00320005	0.108339786	-9.2597566	2.05E-20	1.62E-19
ENSG00000165959	CLMN	316.6953465	422.66595	210.7247386	-1.003239481	0.146274388	-6.8586134	6.95E-12	3.74E-11
ENSG00000126785	RHOJ	11936.24765	15930.609	7941.886571	-1.004169787	0.03919727	-25.61836	9.53E-145	4.52E-143
ENSG00000064687	ABCA7	344.0290587	459.07901	228.9791099	-1.004660532	0.127424969	-7.8843302	3.16E-15	2.01E-14
ENSG00000130816	DNMT1	2394.952052	3196.999	1592.905054	-1.004936903	0.056271843	-17.85861	2.48E-71	5.85E-70
ENSG00000179456	ZBTB18	940.6958563	1255.9843	625.4074322	-1.005407735	0.084337744	-11.921207	9.18E-33	1.06E-31
ENSG00000168916	ZNF608	904.730081	1207.6765	601.783618	-1.005513325	0.085800287	-11.71923	1.02E-31	1.14E-30
ENSG00000143436	MRPL9	1144.555668	1528.2204	760.8909728	-1.006311538	0.073869887	-13.622757	2.93E-42	4.25E-41
ENSG00000154743	TSEN2	146.9902017	196.36227	97.61813273	-1.006512548	0.186651126	-5.3924805	6.95E-08	2.82E-07
ENSG00000095383	TBC1D2	561.5477571	749.7992	373.2963103	-1.006781856	0.118468136	-8.4983345	1.92E-17	1.35E-16
ENSG00000152284	TCF7L1	675.2377813	902.15329	448.3222703	-1.00772311	0.095182982	-10.587219	3.42E-26	3.30E-25
ENSG00000206560	ANKRD28	1894.863652	2531.1148	1258.612456	-1.007968472	0.074373718	-13.552751	7.63E-42	1.10E-40
ENSG00000183496	MEX3B	490.2534518	654.7143	325.7925995	-1.008332428	0.113423568	-8.8899728	6.11E-19	4.58E-18
ENSG00000185630	PBX1	335.6828197	448.73635	222.6292874	-1.009284997	0.144089576	-7.0045664	2.48E-12	1.37E-11
ENSG00000103495	MAZ	3359.298532	4489.867	2228.730064	-1.010810761	0.062655946	-16.132719	1.50E-58	2.88E-57
ENSG00000106025	TSPAN12	635.8695032	850.13055	421.6084535	-1.011277519	0.096281052	-10.503391	8.33E-26	7.95E-25

ENSG00000074527	NTN4	15343.12379	20514.071	10172.17704	-1.011976659	0.058084724	-17.422423	5.58E-68	1.25E-66
ENSG00000151503	NCAPD3	914.7081538	1222.8616	606.5547089	-1.012401037	0.089975219	-11.251999	2.26E-29	2.39E-28
ENSG00000182263	FIGN	189.3583286	253.27347	125.4431876	-1.013553727	0.178916781	-5.664945	1.47E-08	6.30E-08
ENSG00000197258	EIF4BP6	94.71915671	126.59631	62.84200526	-1.013613189	0.245334814	-4.1315506	3.60E-05	0.000114
ENSG00000216866	RPS2P55	31.02813625	41.564992	20.49128049	-1.016789544	0.410297252	-2.4781778	0.013206	0.02892
ENSG00000136367	ZFHX2	75.16980873	100.54929	49.7903268	-1.017234652	0.269083162	-3.7803727	0.000157	0.000458
ENSG00000130052	STARD8	619.9977277	829.84539	410.1500618	-1.01758351	0.103728503	-9.8100664	1.02E-22	8.77E-22
ENSG00000122035	RASL11A	25.73932583	34.440662	17.03799016	-1.018285509	0.446377663	-2.2812192	0.022535	0.046872
ENSG00000139428	MMAB	391.1729855	523.78063	258.5653371	-1.018416782	0.120064803	-8.4822259	2.21E-17	1.55E-16
ENSG00000257038	AP002761.3	84.70197097	113.42206	55.98188584	-1.018607162	0.251809986	-4.0451421	5.23E-05	0.000162
ENSG00000189343	RPS2P46	343.3553575	459.5782	227.1325177	-1.01886262	0.146823671	-6.9393621	3.94E-12	2.15E-11
ENSG00000188368	PRR19	32.86935228	44.035338	21.70336613	-1.019570457	0.392144195	-2.5999887	0.009323	0.020992
ENSG00000153048	CARHSP1	990.1484345	1326.3013	653.9956115	-1.020213505	0.082362318	-12.386996	3.08E-35	3.77E-34
ENSG00000250959	GLUD1P3	32.74274715	43.879562	21.6059322	-1.021557672	0.403206976	-2.5335813	0.01129	0.025015
ENSG00000197747	S100A10	7622.935093	10214.475	5031.394785	-1.021723775	0.04889365	-20.89686	5.72E-97	1.83E-95
ENSG00000225178	RPSAP58	643.4299229	862.32764	424.5322018	-1.022392257	0.120035301	-8.5174299	1.63E-17	1.15E-16
ENSG00000058668	ATP2B4	11290.91555	15143.965	7437.866304	-1.025762608	0.037184616	-27.585672	1.65E-167	9.03E-166
ENSG00000227051	C14orf132	39.03945334	52.40762	25.67128641	-1.026901857	0.365770066	-2.8075065	0.004993	0.011775
ENSG00000180035	ZNF48	474.7280593	636.9786	312.4775146	-1.027403379	0.106063278	-9.6867021	3.43E-22	2.90E-21
ENSG00000164048	ZNF589	376.1786203	504.80979	247.5474471	-1.027461231	0.121138451	-8.4817102	2.22E-17	1.56E-16
ENSG00000135119	RNFT2	28.47697886	38.219686	18.73427221	-1.027690814	0.418797107	-2.453911	0.014131	0.030715
ENSG00000154864	PIEZO2	4751.332758	6376.7311	3125.934411	-1.028764514	0.050886085	-20.21701	6.94E-91	2.08E-89
ENSG00000279348	AC012513.3	159.580593	214.26151	104.8996779	-1.029094898	0.203626573	-5.053834	4.33E-07	1.65E-06
ENSG00000120129	DUSP1	1134.140701	1522.1864	746.0950128	-1.029124171	0.091473249	-11.250548	2.30E-29	2.43E-28
ENSG00000134574	DDB2	2073.447731	2782.7627	1364.132733	-1.029353687	0.070504664	-14.599796	2.82E-48	4.58E-47
ENSG00000132563	REEP2	108.577813	145.70431	71.45132055	-1.029880735	0.218592789	-4.7114122	2.46E-06	8.78E-06
ENSG00000163590	PPM1L	72.68196656	97.56219	47.80174298	-1.030962543	0.27066205	-3.8090399	0.00014	0.000411
ENSG00000258429	PDF	57.21337604	76.84665	37.58010248	-1.033640421	0.293889272	-3.5171084	0.000436	0.001208
ENSG00000187678	SPRY4	806.6868787	1083.6824	529.6913383	-1.033930421	0.089016136	-11.61509	3.45E-31	3.80E-30
ENSG00000181450	ZNF678	129.1180319	173.4831	84.75296598	-1.036016797	0.215411022	-4.8094883	1.51E-06	5.51E-06
ENSG00000131477	RAMP2	450.8599457	606.07769	295.6422047	-1.036022011	0.118607276	-8.7348942	2.44E-18	1.78E-17
ENSG00000017427	IGF1	50.66628255	68.150306	33.18225942	-1.037861489	0.321897362	-3.2242	0.001263	0.003289
ENSG00000204103	MAFB	296.024351	398.04338	194.0053198	-1.039301704	0.156525812	-6.6398103	3.14E-11	1.62E-10
ENSG00000120278	PLEKHG1	2005.991607	2699.5834	1312.399852	-1.040598231	0.062820104	-16.564733	1.25E-61	2.52E-60
ENSG00000198794	SCAMP5	460.0008204	618.98305	301.0185885	-1.040960577	0.124702995	-8.3475186	6.97E-17	4.79E-16
ENSG00000160867	FGFR4	255.2580491	343.53894	166.9771614	-1.041914479	0.150025116	-6.9449337	3.79E-12	2.07E-11
ENSG00000166938	DIS3L	622.5383682	838.21413	406.8626032	-1.043059274	0.098129462	-10.62942	2.17E-26	2.12E-25
ENSG00000198585	NUDT16	1366.240921	1839.8692	892.6126214	-1.043518938	0.068113773	-15.320234	5.60E-53	9.83E-52
ENSG00000213853	EMP2	311.3266666	419.10967	203.5436663	-1.043563021	0.138295982	-7.5458665	4.49E-14	2.69E-13
ENSG00000132326	PER2	89.75570819	120.8294	58.68201189	-1.043643908	0.276565966	-3.7735804	0.000161	0.00047
ENSG00000183117	CSMD1	596.3181188	803.54371	389.0925316	-1.045129209	0.123438537	-8.4667983	2.52E-17	1.76E-16
ENSG00000176428	VPS37D	59.32514228	79.879283	38.77100193	-1.045531052	0.291420542	-3.5877054	0.000334	0.000938
ENSG00000174791	RIN1	315.8096859	425.44272	206.1766511	-1.046596379	0.135885115	-7.7020679	1.34E-14	8.26E-14
ENSG00000134461	ANKRD16	83.87760837	113.08138	54.6738388	-1.046603343	0.27325726	-3.8301026	0.000128	0.00038
ENSG00000075618	FSCN1	13456.95589	18135.907	8778.004817	-1.047043622	0.047801388	-21.904042	2.38E-106	8.29E-105
ENSG00000136379	ABHD17C	319.7437333	431.03222	208.4552481	-1.047139262	0.130973377	-7.9950543	1.30E-15	8.40E-15
ENSG00000099219	ERMP1	1473.928726	1986.3579	961.4995239	-1.047180768	0.070810208	-14.788556	1.74E-49	2.87E-48
ENSG00000140525	FANCI	645.3049738	869.55621	421.0537398	-1.047462807	0.098463081	-10.638127	1.98E-26	1.93E-25
ENSG00000152127	MGAT5	4636.627198	6249.9046	3023.349822	-1.047683187	0.05175404	-20.243505	4.05E-91	1.22E-89
ENSG00000235257	ITGA9-AS1	49.1183645	66.160624	32.07610472	-1.048050098	0.361379308	-2.9001386	0.00373	0.009006
ENSG00000167747	C19orf48	939.9917348	1267.0416	612.941858	-1.048084273	0.085683275	-12.232075	2.10E-34	2.50E-33
ENSG00000142875	PRKACB	2483.218934	3348.3734	1618.064421	-1.049094368	0.061715888	-16.998773	8.39E-65	1.79E-63
ENSG00000099377	HSD3B7	102.780832	138.64516	66.91650353	-1.050333291	0.220880499	-4.7552106	1.98E-06	7.12E-06
ENSG00000189319	FAM53B	537.69172	725.60819	349.7752472	-1.053151464	0.119495208	-8.8133364	1.21E-18	8.99E-18
ENSG00000070610	GBA2	1183.651025	1597.4237	769.8783292	-1.053605948	0.085369421	-12.341725	5.40E-35	6.57E-34
ENSG00000100060	MFNG	4829.757472	6518.9426	3140.572316	-1.054069915	0.05846818	-18.028095	1.17E-72	2.82E-71
ENSG00000172244	C5orf34	48.14507205	64.948203	31.34194134	-1.054481937	0.330168171	-3.1937722	0.001404	0.003618
ENSG00000198483	ANKRD35	61.2402461	82.74106	39.73943232	-1.055050963	0.309324467	-3.4108229	0.000648	0.001752
ENSG00000052723	SIKE1	666.1455375	899.72471	432.5663676	-1.056002313	0.100971261	-10.458444	1.34E-25	1.27E-24
ENSG00000161692	DBF4B	149.536129	201.9214	97.15085522	-1.056419297	0.184835034	-5.7154711	1.09E-08	4.72E-08
ENSG00000162909	CAPN2	19152.85286	25887.844	12417.86145	-1.059909827	0.046973971	-22.563769	9.84E-113	3.63E-111
ENSG00000120885	CLU	1987.252458	2686.461	1288.043965	-1.060639505	0.073459544	-14.438417	2.97E-47	4.74E-46
ENSG00000109576	AADAT	44.31569866	59.959974	28.67142335	-1.062984445	0.372416217	-2.8542915	0.004313	0.010296
ENSG00000196155	PLEKHG4	638.9568481	864.15982	413.7538783	-1.063108635	0.098215143	-10.824284	2.64E-27	2.64E-26
ENSG00000178105	DDX10	1050.288767	1420.7629	679.8144884	-1.063172654	0.082308908	-12.91686	3.62E-38	4.74E-37
ENSG00000154065	ANKRD29	29.93887815	40.512419	19.36533699	-1.063896427	0.451489515	-2.3564145	0.018452	0.039186
ENSG00000139714	MORN3	33.87702957	45.874967	21.87909221	-1.064038526	0.410683347	-2.5908977	0.009573	0.021505
ENSG00000139182	CLSTN3	1785.657201	2415.7632	1155.551239	-1.064219269	0.061838842	-17.20956	2.25E-66	4.94E-65
ENSG00000177054	ZDHHC13	1644.90827	2226.3872	1063.429301	-1.065944428	0.067871085	-15.705428	1.39E-55	2.54E-54
ENSG00000149489	ROM1	215.1293549	291.11216	139.1465451	-1.066923632	0.176351897	-6.0499697	1.45E-09	6.67E-09
ENSG00000204967	PCDHA4	44.12123382	59.670523	28.571945	-1.06724337	0.359517819	-2.968541	0.002992	0.007337
ENSG00000177595	PIDD1	407.8320467	552.22564	263.4384485	-1.068306202	0.123025169	-8.6836394	3.83E-18	2.77E-17
ENSG00000073464	CLCN4	537.7460482	728.14515	347.3469495	-1.068620766	0.102357073	-10.440126	1.63E-25	1.54E-24
ENSG00000100714	MTHFD1	1944.494832	2633.2253	1255.764317	-1.068753151	0.064042858	-16.688093	1.60E-62	3.27E-61
ENSG00000140534	TICRR	122.9858012	166.54626	79.42534135	-1.069512879	0.214277649	-4.991248	6.00E-07	2.26E-06
ENSG00000113532	ST8SIA4	506.7640017	686.45062	327.0773878	-1.070233984	0.106710551	-10.029317	1.13E-23	1.01E-22
ENSG00000242715	CCDC169	32.92347608	44.635632	21.21131974	-1.070793777	0.391719711	-2.7335713	0.006265	0.014532
ENSG00000005469	CROT	367.8506737	498.53714	237.1642047	-1.07111541	0.130668792	-8.1971785	2.46E-16	1.65E-15
ENSG00000129295	LRRC6	153.2546015	207.66913	98.8400752	-1.071739527	0.200728245	-5.3392562	9.33E-08	3.75E-07
ENSG00000139531	SUOX	649.9912128	880.90529	419.0771324	-1.07229066	0.096128782	-11.15473	6.79E-29	7.08E-28
ENSG00000179988	PSTK	30.8672445	41.858752	19.87573722	-1.072447769	0.42712727	-2.5108389	0.012044	0.026541
ENSG00000137124	ALDH1B1	737.5285333	999.74478	475.3122861	-1.07285374	0.097036241	-11.056217	2.05E-28	2.11E-27
ENSG00000277534	AC007996.1	60.98996033	82.727517	39.25240357	-1.073789668	0.293731211	-3.655688	0.000256	0.000733
ENSG00000163947	ARHGEF3	1041.574148	1412.6025	670.545785	-1.074982122	0.078607695	-13.675279	1.43E-42	2.08E-41
ENSG00000119326	CTNNAL1	1827.58119	2478.8179	1176.344472	-1.075042774	0.061496038	-17.481496	1.98E-68	4.46E-67

ENSG00000178401	DNAJC22	65.24593921	88.482852	42.00902613	-1.075236537	0.288224821	-3.730548	0.000191	0.000553
ENSG00000136108	CKAP2	1181.416816	1602.5022	760.3314199	-1.076499147	0.074429337	-14.463371	2.06E-47	3.30E-46
ENSG00000144827	ABHD10	498.0647716	675.60305	320.526489	-1.077025128	0.110618939	-9.7363538	2.11E-22	1.79E-21
ENSG00000143643	TTC13	638.9813152	867.19154	410.7710913	-1.07704311	0.098463564	-10.938494	7.54E-28	7.66E-27
ENSG00000277287	AL109976.1	62.33881346	84.603558	40.0740689	-1.077387738	0.304343814	-3.540035	0.0004	0.001114
ENSG00000012048	BRCA1	336.9424841	457.19656	216.6884081	-1.07748743	0.126834009	-8.4952565	1.97E-17	1.39E-16
ENSG00000101000	PROCR	9988.122988	13554.406	6421.840146	-1.07780857	0.061309236	-17.579873	3.51E-69	7.99E-68
ENSG00000198929	NOS1AP	48.77619445	66.20905	31.34333872	-1.079213269	0.334153887	-3.2296894	0.001239	0.003229
ENSG00000170100	ZNF778	250.8820259	340.69192	161.0721362	-1.079714477	0.149512732	-7.2215554	5.14E-13	2.93E-12
ENSG00000124766	SOX4	6477.101481	8797.9023	4156.300702	-1.081670526	0.044640218	-24.230852	1.05E-129	4.56E-128
ENSG00000162804	SNED1	3057.166664	4152.676	1961.657366	-1.081726231	0.051254324	-21.105073	7.14E-99	2.31E-97
ENSG00000133026	MYH10	8313.418669	11294.939	5331.898105	-1.08275608	0.060596273	-17.868361	2.08E-71	4.92E-70
ENSG00000165655	ZNF503	67.3943565	91.654531	43.13418172	-1.08287432	0.285590955	-3.7916968	0.00015	0.00044
ENSG00000178585	CTNNBIP1	1391.392713	1890.1246	892.660865	-1.083209041	0.074464497	-14.546651	6.13E-48	9.92E-47
ENSG00000171604	CXXC5	89.11461843	121.13485	57.09438983	-1.083245044	0.258761259	-4.1862721	2.84E-05	9.08E-05
ENSG00000067715	SYT1	42.83236958	58.278779	27.38595988	-1.083536285	0.400570393	-2.7049835	0.006831	0.015722
ENSG00000140988	RPS2	38118.12562	51801.81	24434.44151	-1.084148792	0.053574324	-20.23635	4.69E-91	1.40E-89
ENSG00000164107	HAND2	227.1010134	308.41633	145.7856962	-1.084651517	0.181711779	-5.9690765	2.39E-09	1.08E-08
ENSG00000109501	WFS1	3008.104238	4088.8021	1927.406335	-1.085181483	0.056808531	-19.102439	2.41E-81	6.47E-80
ENSG00000172716	SLFN11	2150.642085	2924.1208	1377.163346	-1.086320135	0.063660547	-17.06426	2.74E-65	5.89E-64
ENSG00000069812	HES2	1241.443914	1688.0673	794.8205628	-1.086429406	0.087848746	-12.367045	3.94E-35	4.81E-34
ENSG00000173588	CEP83	151.5023969	205.9774	97.02739591	-1.08673573	0.199368566	-5.450888	5.01E-08	2.06E-07
ENSG00000198130	HIBCH	533.6196025	725.93436	341.304847	-1.087429377	0.11156774	-9.7468083	1.90E-22	1.62E-21
ENSG00000187244	BCAM	824.1231901	1120.8043	527.442077	-1.087434016	0.092344319	-11.775863	5.20E-32	5.86E-31
ENSG00000178718	RPP25	794.9490849	1081.1883	508.7099149	-1.088239158	0.091152024	-11.938727	7.44E-33	8.58E-32
ENSG00000141540	TTYH2	24.10133533	32.83067	15.37200079	-1.089258885	0.476323738	-2.2868037	0.022207	0.046261
ENSG00000266865	AC138207.8	25.68882433	34.975741	16.40190775	-1.089272362	0.456278922	-2.3872949	0.016973	0.036332
ENSG00000196476	C20orf96	202.3221814	275.31997	129.3243939	-1.090588654	0.162672867	-6.7041829	2.03E-11	1.06E-10
ENSG00000144730	IL17RD	109.6107546	149.27031	69.95120044	-1.09086203	0.218872889	-4.9839979	6.23E-07	2.34E-06
ENSG00000101871	MID1	855.1466692	1163.6234	546.669962	-1.09102106	0.089076784	-12.248097	1.72E-34	2.06E-33
ENSG00000277476	AC005332.5	36.22307239	49.207348	23.23879656	-1.091586826	0.423967945	-2.5746919	0.010033	0.02243
ENSG00000263266	RPS7P1	87.97700917	119.76031	56.19371015	-1.092210431	0.268800154	-4.0632805	4.84E-05	0.000151
ENSG00000046889	PREX2	2704.150565	3683.5773	1724.723845	-1.094180101	0.065023684	-16.827409	1.54E-63	3.19E-62
ENSG00000147813	NAPRT	604.3126147	822.94408	385.6811451	-1.094881856	0.111631406	-9.8080092	1.04E-22	8.94E-22
ENSG00000166707	ZCCHC18	24.77688838	33.771642	15.78213476	-1.095854243	0.460509498	-2.3796561	0.017329	0.037038
ENSG00000178184	PARD6G	28.05313929	38.255833	17.8504457	-1.096101495	0.444989967	-2.463205	0.01377	0.030031
ENSG00000204138	PHACTR4	2179.829004	2970.981	1388.677006	-1.097884972	0.064589116	-16.997987	8.50E-65	1.81E-63
ENSG00000102760	RGCC	951.1045686	1296.9485	605.2606451	-1.098885778	0.084163233	-13.056601	5.83E-39	7.83E-38
ENSG00000171044	XKR6	60.7886191	82.878217	38.69902149	-1.098948218	0.363821406	-3.0205705	0.002523	0.00625
ENSG00000140451	PIF1	84.37845129	114.98374	53.77315913	-1.099068635	0.276557854	-3.9741002	7.06E-05	0.000216
ENSG00000165113	GKAP1	45.03291037	61.415546	28.65027473	-1.099496055	0.343066479	-3.2049067	0.001351	0.003495
ENSG00000111490	TBC1D30	355.1054032	484.25381	225.9569989	-1.099511383	0.125942106	-8.7302921	2.54E-18	1.85E-17
ENSG00000138658	ZGRF1	71.02176312	96.89391	45.14961641	-1.099836933	0.270729922	-4.0624875	4.86E-05	0.000151
ENSG00000153904	DDAH1	2372.307548	3235.3541	1509.260962	-1.100100234	0.065110449	-16.895909	4.82E-64	1.01E-62
ENSG00000161714	PLCD3	992.3486053	1353.2142	631.482979	-1.100969357	0.091794805	-11.993809	3.83E-33	4.45E-32
ENSG00000113448	PDE4D	594.4752887	811.40831	377.5422674	-1.102335475	0.101253186	-10.886921	1.33E-27	1.34E-26
ENSG00000139618	BRCA2	259.1445019	353.77204	164.516967	-1.102914798	0.153206427	-7.1988808	6.07E-13	3.44E-12
ENSG00000091972	CD200	444.3402553	606.55593	282.1245803	-1.103288153	0.114336279	-9.649502	4.94E-22	4.14E-21
ENSG00000112159	MDN1	2197.994252	3000.0134	1395.975113	-1.10353275	0.063417209	-17.401156	8.09E-68	1.81E-66
ENSG00000107833	NPM3	396.3865867	541.09675	251.6764256	-1.106226721	0.132335945	-8.3592309	6.31E-17	4.35E-16
ENSG00000079482	OPHN1	366.658928	500.80996	232.5078976	-1.106529868	0.123803888	-8.9377634	3.97E-19	3.00E-18
ENSG00000088766	CRLS1	120.8740515	164.94087	76.80723101	-1.106985878	0.254824768	-4.3441063	1.40E-05	4.62E-05
ENSG00000213347	MXD3	76.78296195	104.95735	48.60857141	-1.107196389	0.271270822	-4.0815167	4.47E-05	0.00014
ENSG00000114812	VIPR1	52.80352839	72.056248	33.55080893	-1.108544634	0.361999987	-3.0622781	0.002197	0.005492
ENSG00000082515	MRPL22	1042.156525	1424.3857	659.9273561	-1.109171399	0.082230056	-13.488637	1.82E-41	2.60E-40
ENSG00000122678	POLM	374.0227973	510.99571	237.04988	-1.109357365	0.138913214	-7.9859744	1.39E-15	9.02E-15
ENSG00000110104	CCDC86	2023.061365	2765.4502	1280.672517	-1.110844922	0.062960574	-17.643501	1.14E-69	2.61E-68
ENSG00000167325	RRM1	1811.945033	2477.0291	1146.860986	-1.110892275	0.078302893	-14.187117	1.10E-45	1.70E-44
ENSG00000240342	RPS2P5	3703.881193	5064.7382	2343.024184	-1.11243355	0.065137256	-17.078299	2.15E-65	4.64E-64
ENSG00000050438	SLC4A8	467.409579	639.33374	295.4854137	-1.112969932	0.116923158	-9.5188152	1.75E-21	1.44E-20
ENSG00000121057	AKAP1	724.9022623	991.40151	458.4030171	-1.114267296	0.093865297	-11.870919	1.68E-32	1.92E-31
ENSG00000133740	E2F5	45.67925409	62.43572	28.9227877	-1.114448738	0.347972585	-3.2026912	0.001361	0.003518
ENSG00000254726	MEX3A	810.4334457	1108.3065	512.5603881	-1.114565616	0.108641533	-10.259112	1.08E-24	9.88E-24
ENSG00000119943	PYROXD2	67.9036869	92.990122	42.81725195	-1.114685787	0.289880633	-3.8453269	0.00012	0.000358
ENSG00000198157	HMGN5	121.6744085	166.57073	76.77809154	-1.11509138	0.225784096	-4.9387508	7.86E-07	2.93E-06
ENSG00000132613	MTSS1L	1665.215452	2278.8576	1051.573286	-1.115669036	0.065875191	-16.936103	2.44E-64	5.16E-63
ENSG00000132970	WASF3	1126.836056	1542.2758	711.3963615	-1.115692906	0.0757135	-14.73572	3.80E-49	6.26E-48
ENSG00000111716	LDHB	232.5227702	318.49126	146.5542787	-1.11923237	0.153283565	-7.3017115	2.84E-13	1.64E-12
ENSG00000102967	DHODH	140.5275313	192.61246	88.44260532	-1.119734284	0.206929632	-5.4111838	6.26E-08	2.55E-07
ENSG00000230844	ZNF674-AS1	32.54842687	44.632387	20.46446719	-1.120227511	0.414088309	-2.7052865	0.006825	0.015709
ENSG00000082497	SERTAD4	74.27459566	101.76183	46.78735753	-1.120265499	0.272730047	-4.1075984	4.00E-05	0.000126
ENSG00000198336	MYL4	38.55981371	52.826258	24.29336909	-1.121078378	0.361061232	-3.1049536	0.001903	0.004804
ENSG00000185641	AC034236.1	82.61022517	113.23692	51.983532	-1.121779677	0.265320944	-4.2280103	2.36E-05	7.60E-05
ENSG00000143942	CHAC2	101.7957604	139.51983	64.07169441	-1.123037316	0.22349099	-5.024978	5.03E-07	1.91E-06
ENSG00000205476	CCDC85C	722.1452833	990.08421	454.2063536	-1.125466305	0.092142601	-12.214397	2.60E-34	3.10E-33
ENSG00000234797	RPS3AP6	116.0826649	159.14968	73.01564628	-1.126588543	0.212833884	-5.2932762	1.20E-07	4.79E-07
ENSG00000121988	ZRANB3	107.6673354	147.72075	67.61392164	-1.127007931	0.233741361	-4.8216025	1.42E-06	5.20E-06
ENSG00000100478	AP4S1	148.1340385	203.25316	93.01491795	-1.127424789	0.192505033	-5.856599	4.72E-09	2.10E-08
ENSG00000149646	CNBD2	41.67800844	57.286639	26.06937826	-1.128267004	0.398934894	-2.8281983	0.004681	0.011102
ENSG00000137491	SLCO2B1	65.33679377	89.639444	41.03414314	-1.129456221	0.325014604	-3.4750938	0.000511	0.001403
ENSG00000182118	FAM89A	194.3655633	266.7864	121.9447301	-1.129768879	0.174945147	-6.4578464	1.06E-10	5.29E-10
ENSG00000155463	OXA1L	4423.620732	6072.7445	2774.497011	-1.13053314	0.05676847	-19.914807	3.03E-88	8.75E-87
ENSG00000154930	ACSS1	372.0297225	510.99455	233.0649	-1.131623656	0.125723861	-9.0008662	2.24E-19	1.71E-18
ENSG00000156113	KCNMA1	22.31721402	30.68132	13.95310847	-1.133482332	0.487682312	-2.3242228	0.020114	0.042327
ENSG00000257337	AC068888.1	30.2393286	41.493021	18.98563657	-1.133788363	0.461318833	-2.457711	0.013983	0.03043

ENSG00000116120	FARSB	1204.043225	1654.2923	753.7941945	-1.133873492	0.094584044	-11.988	4.11E-33	4.77E-32
ENSG00000178531	CTXN1	503.9475857	692.35744	315.5377307	-1.134797026	0.114218865	-9.9352855	2.92E-23	2.56E-22
ENSG00000143416	SELENBP1	194.0253551	266.91083	121.1398804	-1.13775305	0.174603476	-6.5162108	7.21E-11	3.64E-10
ENSG00000107611	CUBN	339.8520651	467.62693	212.0772025	-1.138890791	0.139052256	-8.1903798	2.60E-16	1.75E-15
ENSG00000136449	MYCBPAP	34.44820742	47.447245	21.44916944	-1.139734593	0.435057971	-2.6197304	0.0088	0.019893
ENSG00000187994	RINL	130.9783527	180.24987	81.70683641	-1.143164957	0.21007675	-5.4416539	5.28E-08	2.17E-07
ENSG00000135002	RFK	1750.893458	2411.205	1090.58195	-1.144255802	0.07532956	-15.189997	4.12E-52	7.14E-51
ENSG00000162437	RAVER2	501.5172186	690.54205	312.492389	-1.144555087	0.105623616	-10.836166	2.32E-27	2.33E-26
ENSG00000143799	PARP1	3509.692892	4833.4033	2185.982532	-1.144953019	0.055372813	-20.677169	5.56E-95	1.74E-93
ENSG00000118707	TGIF2	400.139146	550.92853	249.3497634	-1.14496558	0.121400372	-9.4313185	4.05E-21	3.30E-20
ENSG00000187372	PCDHB13	115.7308761	159.46974	71.99201347	-1.145603427	0.231882324	-4.9404517	7.79E-07	2.91E-06
ENSG00000162062	TEDC2	74.99655844	103.16547	46.82764791	-1.145914522	0.317466285	-3.6095629	0.000307	0.000867
ENSG00000177602	HASPIN	130.2033266	179.38813	81.01852479	-1.146235151	0.208443634	-5.4990173	3.82E-08	1.59E-07
ENSG00000136153	LMO7	299.1682992	412.25109	186.0855069	-1.146479322	0.137108429	-8.3618442	6.17E-17	4.26E-16
ENSG00000121236	TRIM6	119.3202366	164.54484	74.09563472	-1.149693837	0.206669714	-5.5629527	2.65E-08	1.11E-07
ENSG00000136161	RCBTB2	333.7105703	460.22949	207.1916461	-1.150003782	0.139126302	-8.2658977	1.39E-16	9.42E-16
ENSG00000151276	MAGI1	871.7208941	1202.5699	540.8719176	-1.151247357	0.094073296	-12.23777	1.95E-34	2.34E-33
ENSG00000229119	AC026403.1	55.75398937	76.881411	34.62656764	-1.152048119	0.307154786	-3.7507087	0.000176	0.000512
ENSG00000110660	SLC35F2	2216.58445	3057.4262	1375.742654	-1.152829778	0.06413373	-17.975405	3.04E-72	7.27E-71
ENSG00000160957	RECQL4	221.7647543	306.16205	137.3674595	-1.154504899	0.159514271	-7.2376277	4.57E-13	2.60E-12
ENSG00000067445	TRO	35.9041643	49.620766	22.18756254	-1.154583656	0.447423053	-2.5805189	0.009865	0.022097
ENSG00000113594	LIFR	1845.545191	2547.8925	1143.197833	-1.156610097	0.063806334	-18.126885	1.96E-73	4.75E-72
ENSG00000125630	POLR1B	695.4296021	960.67607	430.1831337	-1.1596138	0.10516562	-11.026548	2.85E-28	2.92E-27
ENSG00000186615	KTN1-AS1	42.68789683	58.997195	26.37859892	-1.159619614	0.372039134	-3.1169291	0.001827	0.004623
ENSG00000146197	SCUBE3	136.2938165	188.16149	84.42614181	-1.160196855	0.214682364	-5.4042486	6.51E-08	2.65E-07
ENSG00000164509	IL31RA	52.86153282	73.03425	32.68881565	-1.160359239	0.324232806	-3.5787842	0.000345	0.000969
ENSG00000154229	PRKCA	1481.145469	2046.5742	915.7167326	-1.161162893	0.073654107	-15.76508	5.41E-56	9.94E-55
ENSG00000065057	NTHL1	297.9649381	412.11242	183.8174514	-1.164500859	0.13961074	-8.341055	7.36E-17	5.05E-16
ENSG00000182511	FES	857.6349778	1185.9994	529.2705595	-1.165172478	0.086749356	-13.431483	3.95E-41	5.58E-40
ENSG00000163788	SNRK	5774.48111	7990.1453	3558.816873	-1.166469964	0.049705082	-23.467821	8.70E-122	3.49E-120
ENSG00000228502	EEF1A1P11	24.17702049	33.430964	14.92307712	-1.171835788	0.475367606	-2.4651149	0.013697	0.029892
ENSG00000227260	LINC01985	32.1082874	44.53427	19.68230433	-1.172315167	0.415940889	-2.8184658	0.004825	0.011415
ENSG00000164035	EMCN	2424.049427	3358.6501	1489.448793	-1.172405391	0.070859906	-16.545399	1.73E-61	3.47E-60
ENSG00000097046	CDC7	140.968289	195.34906	86.58751607	-1.172502656	0.193751151	-6.0515907	1.43E-09	6.61E-09
ENSG00000263465	SRSF8	1044.830182	1447.8735	641.7868221	-1.173899369	0.079333101	-14.797094	1.53E-49	2.53E-48
ENSG00000185875	THNSL1	68.09816059	94.348935	41.8473866	-1.175390593	0.313381778	-3.7506667	0.000176	0.000512
ENSG00000185920	PTCH1	142.4725416	197.62344	87.32164188	-1.176404711	0.196649456	-5.9822424	2.20E-09	1.00E-08
ENSG00000100490	CDKL1	131.2191483	181.93505	80.50324779	-1.177075598	0.202129515	-5.8233732	5.77E-09	2.54E-08
ENSG00000132846	ZBED3	817.3057321	1133.3538	501.2576989	-1.177155264	0.088680637	-13.274096	3.27E-40	4.51E-39
ENSG00000175772	LINC01106	28.21616195	39.112677	17.31964719	-1.177809539	0.47491875	-2.4800232	0.013137	0.028779
ENSG00000119403	PHF19	959.3251013	1330.6378	588.012444	-1.178763047	0.086728597	-13.5914	4.50E-42	6.50E-41
ENSG00000198826	ARHGAP11A	782.9486397	1086.5445	479.3527306	-1.179437862	0.098156533	-12.015888	2.93E-33	3.42E-32
ENSG00000188486	H2AFX	869.448809	1206.1116	532.7860486	-1.179716216	0.096726012	-12.196473	3.25E-34	3.85E-33
ENSG00000161267	BDH1	39.63239932	55.003908	24.26089112	-1.180012298	0.366835103	-3.2167377	0.001297	0.003365
ENSG00000125864	BFSP1	111.8399233	155.19196	68.48789132	-1.18189824	0.217884439	-5.424427	5.81E-08	2.38E-07
ENSG00000101306	MYLK2	28.22561529	39.178185	17.27304509	-1.182518311	0.442390703	-2.673018	0.007517	0.017209
ENSG00000169299	PGM2	1627.441528	2260.4772	994.4058088	-1.183722416	0.073046472	-16.205059	4.64E-59	8.99E-58
ENSG00000131470	PSMC3IP	112.7966011	156.55537	69.03783159	-1.184537862	0.224893506	-5.2671057	1.39E-07	5.49E-07
ENSG00000085831	TTC39A	27.70988592	38.521588	16.89818386	-1.187946038	0.451993039	-2.6282397	0.008583	0.019452
ENSG00000156466	GDF6	1379.992844	1918.4138	841.5718503	-1.188708023	0.090264037	-13.169232	1.32E-39	1.79E-38
ENSG00000171914	TLN2	37.90367863	52.683701	23.12365583	-1.189102222	0.377657291	-3.1486277	0.00164	0.004178
ENSG00000057294	PKP2	310.2998421	431.502	189.097686	-1.191091901	0.139126099	-8.5612398	1.12E-17	7.93E-17
ENSG00000099260	PALMD	3914.089939	5450.3044	2377.875462	-1.196346458	0.058835931	-20.333603	6.49E-92	1.97E-90
ENSG00000159388	BTG2	2503.135953	3486.4448	1519.827143	-1.197310153	0.057322762	-20.887168	7.01E-97	2.24E-95
ENSG00000133627	ACTR3B	94.07301887	130.93794	57.2080956	-1.19835911	0.251790034	-4.7593588	1.94E-06	6.98E-06
ENSG00000114166	KAT2B	627.5769814	874.26405	380.8899085	-1.19895202	0.095092616	-12.608256	1.90E-36	2.40E-35
ENSG00000059804	SLC2A3	1210.928967	1687.1374	734.7205122	-1.199136479	0.079420856	-15.098509	1.66E-51	2.83E-50
ENSG00000169914	OTUD3	156.6177664	218.28082	94.95471438	-1.19914914	0.185532319	-6.4632898	1.02E-10	5.11E-10
ENSG00000151623	NR3C2	730.4216458	1017.7941	443.0491917	-1.200477315	0.098977502	-12.12879	7.43E-34	8.75E-33
ENSG00000143554	SLC27A3	530.7373239	739.52421	321.9504365	-1.201262848	0.120883698	-9.9373436	2.86E-23	2.51E-22
ENSG00000271270	TMCC1-AS1	42.70381681	59.480753	25.92688049	-1.203202304	0.351428733	-3.4237448	0.000618	0.001677
ENSG00000155189	AGPAT5	1588.254461	2215.0608	961.4481286	-1.203295001	0.074785597	-16.08993	3.00E-58	5.72E-57
ENSG00000133056	PIK3C2B	1766.568809	2463.9258	1069.211804	-1.203632478	0.065550192	-18.361998	2.65E-75	6.54E-74
ENSG00000091127	PUS7	764.7939597	1066.8295	462.7583844	-1.204730987	0.090169203	-13.360781	1.02E-40	1.43E-39
ENSG00000133315	MACROD1	218.0935479	304.39705	131.7900463	-1.205782713	0.174115412	-6.9251923	4.35E-12	2.37E-11
ENSG00000151131	C12orf45	198.5438312	277.28415	119.80351	-1.206974216	0.183221648	-6.5875088	4.47E-11	2.29E-10
ENSG00000067992	PDK3	406.6146358	567.31244	245.9168338	-1.207066566	0.117491491	-10.273651	9.26E-25	8.52E-24
ENSG00000105851	PIK3CG	415.6752688	580.14218	251.2083602	-1.208854769	0.125813918	-9.6082754	7.38E-22	6.15E-21
ENSG00000197299	BLM	115.7866369	161.7103	69.8629763	-1.210733227	0.219375329	-5.5190036	3.41E-08	1.42E-07
ENSG00000125454	SLC25A19	186.0629507	259.92643	112.1994736	-1.210906319	0.17552909	-6.8986076	5.25E-12	2.85E-11
ENSG00000101945	SUV39H1	211.510469	295.45847	127.5624712	-1.21500821	0.171756467	-7.0740173	1.51E-12	8.39E-12
ENSG00000272449	AL139246.5	22.20676065	31.022292	13.39122937	-1.215993076	0.505838166	-2.4039172	0.01622	0.034844
ENSG00000100504	PYGL	2022.092151	2826.9696	1217.214697	-1.216632016	0.072792607	-16.713676	1.04E-62	2.14E-61
ENSG00000028137	TNFRSF1B	955.100612	1335.8491	574.3521716	-1.217755623	0.082846207	-14.698991	6.54E-49	1.07E-47
ENSG00000150551	LYPD1	257.4033047	360.23041	154.5761956	-1.218216223	0.166373275	-7.322187	2.44E-13	1.41E-12
ENSG00000214389	RPS3AP26	101.5635554	142.04709	61.08001692	-1.218465853	0.225891701	-5.3940266	6.89E-08	2.80E-07
ENSG00000138185	ENTPD1	825.6009839	1155.3678	495.8341692	-1.221006511	0.107197546	-11.390247	4.68E-30	5.03E-29
ENSG00000115525	ST3GAL5	544.8345044	762.95529	326.713715	-1.222488169	0.110674496	-11.045798	2.30E-28	2.36E-27
ENSG00000274026	FAM27E3	27.26785238	38.106196	16.42950896	-1.222934296	0.458389784	-2.6678917	0.007633	0.017455
ENSG00000197147	LRRC8B	1235.004464	1728.8727	741.1361819	-1.223014038	0.073949294	-16.538549	1.94E-61	3.88E-60
ENSG00000260196	AC124798.1	72.80138492	101.92994	43.6728302	-1.223340086	0.291483919	-4.1969385	2.71E-05	8.68E-05
ENSG00000099937	SERPIND1	481.879765	674.65621	289.1033199	-1.223518585	0.121520781	-10.06839	7.62E-24	6.82E-23
ENSG00000102893	PHKB	1853.477836	2595.8877	1111.067923	-1.224307321	0.066723394	-18.348996	3.36E-75	8.30E-74
ENSG00000125648	SLC25A23	1362.156987	1907.9974	816.3166161	-1.224825534	0.07253258	-16.886557	5.65E-64	1.19E-62
ENSG00000221829	FANCG	259.8895147	364.10708	155.6719498	-1.22709692	0.156208994	-7.8554819	3.98E-15	2.52E-14

ENSG00000005249	PRKAR2B	2489.715333	3489.6226	1489.808114	-1.228045766	0.063156038	-19.44463	3.24E-84	8.99E-83
ENSG00000141448	GATA6	425.1318503	595.70637	254.557333	-1.228697635	0.128019744	-9.5977199	8.17E-22	6.80E-21
ENSG00000006625	GGCT	213.057507	298.89513	127.2198814	-1.229380798	0.169279217	-7.2624438	3.80E-13	2.18E-12
ENSG00000165185	KIAA1958	73.96861943	103.69049	44.24675143	-1.229702292	0.287155097	-4.2823628	1.85E-05	6.03E-05
ENSG00000143409	MINDY1	429.604768	602.39196	256.8175761	-1.230617858	0.113945092	-10.800095	3.44E-27	3.42E-26
ENSG00000173638	SLC19A1	663.0760631	929.69255	396.4595719	-1.230752301	0.104220247	-11.809148	3.50E-32	3.97E-31
ENSG00000136859	ANGPTL2	1954.766915	2741.0723	1168.46149	-1.230947183	0.069640701	-17.675686	6.46E-70	1.48E-68
ENSG00000167536	DHRS13	74.02204502	103.90398	44.14010772	-1.230970927	0.27661802	-4.450075	8.58E-06	2.89E-05
ENSG00000144118	RALB	4721.604797	6626.7973	2816.412269	-1.234324795	0.048706527	-25.342082	1.10E-141	5.07E-140
ENSG00000121691	CAT	1599.94055	2245.8157	954.06538	-1.235259263	0.0693986	-17.799484	7.13E-71	1.66E-69
ENSG00000196159	FAT4	2919.959102	4099.3111	1740.607138	-1.235434669	0.060806644	-20.317429	9.02E-92	2.73E-90
ENSG00000111077	TNS2	1882.304377	2644.4486	1120.16015	-1.239543028	0.062658405	-19.78255	4.21E-87	1.20E-85
ENSG00000276170	AC244153.1	40.25122805	56.55993	23.94252639	-1.240291737	0.363942708	-3.4079313	0.000655	0.00177
ENSG00000132967	HMGB1P5	87.71877053	123.46042	51.97711698	-1.241770111	0.288203648	-4.3086551	1.64E-05	5.39E-05
ENSG00000072163	LIMS2	1313.46324	1846.1605	780.7659973	-1.241867184	0.078491369	-15.821704	2.20E-56	4.06E-55
ENSG00000145390	USP53	1731.807477	2435.4086	1028.20636	-1.24454627	0.06422666	-19.377409	1.20E-83	3.30E-82
ENSG00000224877	NDUFAF8	495.2716903	696.67915	293.8642262	-1.246856079	0.147099392	-8.476283	2.33E-17	1.63E-16
ENSG00000142856	ITGB3BP	102.6065783	144.32442	60.88873175	-1.247207122	0.234913585	-5.3092167	1.10E-07	4.40E-07
ENSG00000158716	DUSP23	993.0689555	1398.5321	587.6058176	-1.251530044	0.099113639	-12.627223	1.49E-36	1.89E-35
ENSG00000179981	TSHZ1	685.8688279	966.36801	405.3696484	-1.253498535	0.099568078	-12.589362	2.42E-36	3.04E-35
ENSG00000233830	EIF4HP1	21.89424806	30.832817	12.95567949	-1.253811345	0.495837112	-2.5286759	0.011449	0.025331
ENSG00000204282	TNRC6C-AS1	63.65038707	89.816024	37.48475055	-1.256086407	0.326191571	-3.8507636	0.000118	0.000351
ENSG00000247092	SNHG10	30.56365777	43.063648	18.0636674	-1.256256501	0.412226791	-3.0474887	0.002308	0.005746
ENSG00000164904	ALDH7A1	1535.834915	2165.8193	905.8505298	-1.257118377	0.066140631	-19.006749	1.50E-80	3.96E-79
ENSG00000097096	SYDE2	48.3094119	68.099108	28.51971579	-1.259521901	0.340267819	-3.7015605	0.000214	0.000618
ENSG00000152782	PANK1	100.4418774	141.69349	59.1902644	-1.259816752	0.226731803	-5.5564183	2.75E-08	1.15E-07
ENSG00000109062	SLC9A3R1	587.1623974	828.57257	345.7522278	-1.260631436	0.100807076	-12.505386	6.98E-36	8.68E-35
ENSG00000235109	ZSCAN31	520.7350353	735.2937	306.1763668	-1.263238691	0.122113247	-10.344813	4.42E-25	4.12E-24
ENSG00000255468	AP001107.9	23.67404669	33.457109	13.89098487	-1.263886387	0.463209985	-2.7285387	0.006362	0.014741
ENSG00000126602	TRAP1	1759.259285	2485.2129	1033.30571	-1.26643618	0.071049084	-17.824806	4.54E-71	1.06E-69
ENSG00000173269	MMRN2	13014.64809	18389.364	7639.9322	-1.267381544	0.037925123	-33.417994	7.51E-245	6.59E-243
ENSG00000111348	ARHGDIB	9173.036656	12969.604	5376.465632	-1.270545662	0.048442085	-26.228137	1.27E-151	6.21E-150
ENSG00000249867	AC090833.1	59.72973442	84.434059	35.02540983	-1.270839251	0.309463787	-4.1065847	4.02E-05	0.000127
ENSG00000060140	STYK1	424.6028921	600.23361	248.9721732	-1.271417227	0.127422091	-9.9779969	1.90E-23	1.68E-22
ENSG00000171115	GIMAP8	4069.168422	5756.7182	2381.61869	-1.27284065	0.058890712	-21.613606	1.34E-103	4.55E-102
ENSG00000173890	GPR160	62.43583272	88.387214	36.48445165	-1.273732724	0.317589801	-4.0106223	6.06E-05	0.000187
ENSG00000205269	TMEM170B	169.3846887	239.56638	99.20299761	-1.274260579	0.183432797	-6.9467434	3.74E-12	2.05E-11
ENSG00000117676	RPS6KA1	299.6110763	424.06216	175.1599957	-1.27428458	0.144018028	-8.8480907	8.90E-19	6.62E-18
ENSG00000169750	RAC3	578.003378	818.38441	337.6223449	-1.277715394	0.123355375	-10.358003	3.85E-25	3.60E-24
ENSG00000184792	OSBP2	452.2239537	640.39618	264.0517313	-1.278215482	0.112017512	-11.410854	3.69E-30	3.98E-29
ENSG00000149503	INCENP	836.6856457	1185.5883	487.7829813	-1.280656407	0.088790285	-14.423384	3.69E-47	5.87E-46
ENSG00000186314	PRELID2	101.4426803	143.77701	59.10835199	-1.280857859	0.232583501	-5.5070882	3.65E-08	1.52E-07
ENSG00000189410	SH2D5	32.6491157	46.300656	18.99757539	-1.282889806	0.445462431	-2.8799057	0.003978	0.009557
ENSG00000137054	POLR1E	382.4937586	542.5707	222.4168161	-1.286421469	0.136143425	-9.4490165	3.42E-21	2.80E-20
ENSG00000198720	ANKRD13B	821.5081915	1165.4364	477.5799388	-1.288482727	0.089512931	-14.394375	5.61E-47	8.88E-46
ENSG00000242193	CRYZL2P	139.061839	197.41785	80.70582475	-1.289972095	0.20125555	-6.4096225	1.46E-10	7.21E-10
ENSG00000163006	CCDC138	91.35571221	129.81517	52.89625381	-1.289993178	0.254798133	-5.0628047	4.13E-07	1.58E-06
ENSG00000152518	ZFP36L2	1636.903711	2323.9133	949.8941699	-1.290853865	0.064517636	-20.007768	4.71E-89	1.38E-87
ENSG00000143815	LBR	1535.884911	2180.8149	890.9549412	-1.291052176	0.084925952	-15.202093	3.43E-52	5.95E-51
ENSG00000168496	FEN1	660.7476362	938.06619	383.4290796	-1.291713324	0.100990065	-12.790499	1.85E-37	2.40E-36
ENSG00000231298	MANCR	596.9402111	847.83812	346.0423067	-1.292489042	0.098336456	-13.143539	1.85E-39	2.51E-38
ENSG00000238164	TNFRSF14-AS1	24.06359444	34.192434	13.93475463	-1.294188943	0.458063856	-2.8253461	0.004723	0.011188
ENSG00000250479	CHCHD10	427.2926418	607.00999	247.5752925	-1.294812529	0.286292567	-4.52269	6.11E-06	2.09E-05
ENSG00000184220	CMSS1	450.0925093	639.65081	260.5342072	-1.295692543	0.125384725	-10.333735	4.96E-25	4.61E-24
ENSG00000145147	SLIT2	1555.471342	2211.0951	899.847602	-1.297422921	0.072711728	-17.843379	3.25E-71	7.65E-70
ENSG00000103319	EEF2K	1553.837214	2209.5063	898.1681637	-1.298729132	0.067369718	-19.277639	8.28E-83	2.25E-81
ENSG00000133466	C1QTNF6	1083.063112	1540.2127	625.91349	-1.298989583	0.086720094	-14.979107	1.01E-50	1.71E-49
ENSG00000138778	CENPE	936.9526348	1332.5445	541.3607664	-1.300055971	0.084520201	-15.381601	2.18E-53	3.83E-52
ENSG00000247626	MARS2	128.2538167	182.42533	74.08229851	-1.301597537	0.211306451	-6.1597624	7.29E-10	3.43E-09
ENSG00000196468	FGF16	20.57742661	29.216392	11.9384617	-1.30194725	0.541367369	-2.4049238	0.016176	0.034753
ENSG00000100116	GCAT	271.3978234	386.28941	156.5062385	-1.302414153	0.153011981	-8.5118443	1.71E-17	1.20E-16
ENSG00000185252	ZNF74	274.8219796	390.96402	158.6799367	-1.303288333	0.148819348	-8.7575195	2.00E-18	1.46E-17
ENSG00000182985	CADM1	16.60090569	23.628192	9.573619262	-1.303431386	0.550626199	-2.3671801	0.017924	0.038216
ENSG00000170899	GSTA4	398.1653691	566.79193	229.5388037	-1.303445901	0.118488401	-11.00062	3.80E-28	3.88E-27
ENSG00000104154	SLC30A4	396.5749793	564.65519	228.4947726	-1.304437951	0.117156285	-11.13417	8.55E-29	8.90E-28
ENSG00000118193	KIF14	259.5543045	369.6679	149.4407098	-1.304717782	0.149765046	-8.7117643	2.99E-18	2.17E-17
ENSG00000235505	CASP17P	601.5745019	856.7855	346.3635038	-1.305290596	0.11004508	-11.861417	1.88E-32	2.15E-31
ENSG00000154127	UBASH3B	2256.662156	3213.7481	1299.576175	-1.306389704	0.066872789	-19.535445	5.49E-85	1.54E-83
ENSG00000196368	NUDT11	78.24975159	111.53769	44.96181062	-1.306439469	0.265942357	-4.9124911	8.99E-07	3.34E-06
ENSG00000158106	RHPN1	170.7853424	243.31507	98.25561253	-1.307790013	0.185916056	-7.0343037	2.00E-12	1.11E-11
ENSG00000186106	ANKRD46	284.7056705	405.63628	163.775057	-1.307858912	0.140082722	-9.3363328	9.97E-21	7.99E-20
ENSG00000143314	MRPL24	701.8441675	999.70019	403.9881484	-1.307874261	0.103366518	-12.652784	1.08E-36	1.37E-35
ENSG00000080546	SESN1	933.4829662	1329.5766	537.3893291	-1.308262982	0.083960268	-15.581929	9.66E-55	1.74E-53
ENSG00000112312	GMNN	243.5066464	347.15183	139.8614635	-1.309404204	0.159893161	-8.1892466	2.63E-16	1.76E-15
ENSG00000144283	PKP4	1411.562396	2011.7594	811.3653534	-1.310108673	0.069473905	-18.857565	2.55E-79	6.66E-78
ENSG00000140263	SORD	278.6381804	397.28156	159.9948016	-1.310364124	0.151995273	-8.6210847	6.63E-18	4.74E-17
ENSG00000163762	TM4SF18	3712.021859	5292.5519	2131.491809	-1.311699716	0.05442356	-24.101689	2.40E-128	1.02E-126
ENSG00000107282	APBA1	421.5286972	601.06898	241.988416	-1.312875936	0.127048583	-10.333653	4.96E-25	4.61E-24
ENSG00000078018	MAP2	2173.776311	3100.2512	1247.301382	-1.312980007	0.073340053	-17.902632	1.12E-71	2.67E-70
ENSG00000189221	MAOA	900.7351876	1284.5738	516.8966137	-1.314981416	0.09129558	-14.403561	4.91E-47	7.79E-46
ENSG00000157734	SNX22	20.57649485	29.355732	11.79725801	-1.318514589	0.508800743	-2.5914164	0.009558	0.021478
ENSG00000105202	FBL	2022.294421	2886.9744	1157.614421	-1.319178526	0.077330769	-17.058909	3.00E-65	6.44E-64
ENSG00000185201	IFITM2	4074.426246	5817.1562	2331.696248	-1.319305655	0.069438928	-18.999511	1.72E-80	4.54E-79
ENSG00000126950	TMEM35A	422.0530756	603.05744	241.0487119	-1.321215411	0.129724493	-10.18478	2.32E-24	2.10E-23
ENSG00000108622	ICAM2	14507.99236	20731.405	8284.579341	-1.323348333	0.047944323	-27.601773	1.06E-167	5.82E-166

ENSG00000099204	ABLIM1	4800.694078	6862.104	2739.284189	-1.324727164	0.066177378	-20.017825	3.85E-89	1.13E-87
ENSG00000065923	SLC9A7	1044.809173	1493.5763	596.0420231	-1.325167105	0.088560492	-14.963412	1.27E-50	2.15E-49
ENSG00000279086	AC073130.3	15.55164719	22.232758	8.870536476	-1.326703255	0.574757393	-2.3082839	0.020983	0.04398
ENSG00000161681	SHANK1	182.2209875	260.51209	103.9298877	-1.328539813	0.193831151	-6.8541089	7.18E-12	3.86E-11
ENSG00000234614	C2CD4D-AS1	32.14847755	46.009525	18.28743054	-1.329128954	0.426051773	-3.1196419	0.001811	0.004582
ENSG00000185305	ARL15	459.6522585	658.08139	261.2231283	-1.33229647	0.110638863	-12.041849	2.14E-33	2.50E-32
ENSG00000163923	RPL39L	112.3022497	160.86744	63.73705783	-1.332962981	0.239023619	-5.5766999	2.45E-08	1.03E-07
ENSG00000127328	RAB3IP	550.7493793	788.34057	313.1581922	-1.333185249	0.115691631	-11.52361	1.00E-30	1.09E-29
ENSG00000146281	PM20D2	323.2591302	462.80459	183.7136681	-1.333365275	0.143075017	-9.3193438	1.17E-20	9.36E-20
ENSG00000169220	RGS14	67.51907368	96.789625	38.24852199	-1.333739337	0.299080906	-4.45946	8.22E-06	2.78E-05
ENSG00000205078	SYCE1L	75.91112625	108.6647	43.1575532	-1.334641053	0.268066147	-4.9787751	6.40E-07	2.40E-06
ENSG00000111261	MANSC1	2281.458201	3267.9825	1294.93388	-1.33509224	0.065867488	-20.269366	2.40E-91	7.23E-90
ENSG00000269190	FBXO17	372.5904064	534.04167	211.1391399	-1.337538886	0.143351847	-9.3304615	1.05E-20	8.44E-20
ENSG00000234618	RPSAP9	29.80117544	42.784644	16.8177064	-1.342534577	0.422292515	-3.1791579	0.001477	0.003794
ENSG00000166508	MCM7	1703.455591	2443.1463	963.7648598	-1.342659404	0.06567056	-20.445378	6.60E-93	2.02E-91
ENSG00000114378	HYAL1	45.9672913	66.032471	25.90211162	-1.343210603	0.371645728	-3.6142232	0.000301	0.000853
ENSG00000106688	SLC1A1	645.8174306	926.53419	365.1006741	-1.344297606	0.100667434	-13.353848	1.12E-40	1.57E-39
ENSG00000144645	OSBPL10	1085.219114	1557.4988	612.9393909	-1.34441716	0.083654571	-16.071054	4.07E-58	7.74E-57
ENSG00000135363	LMO2	1096.100786	1572.8118	619.3897238	-1.345349122	0.079627003	-16.895639	4.84E-64	1.02E-62
ENSG00000118292	C1orf54	345.1370519	495.60753	194.666574	-1.345649382	0.15153039	-8.8803928	6.66E-19	4.99E-18
ENSG00000260804	LINC01963	273.0209788	391.9196	154.1223576	-1.346078892	0.149491387	-9.0043909	2.17E-19	1.66E-18
ENSG00000116678	LEPR	331.9471068	476.72954	187.1646699	-1.346260866	0.152910901	-8.8042177	1.32E-18	9.74E-18
ENSG00000160588	MPZL3	544.5230357	781.67167	307.3744034	-1.346700301	0.117934708	-11.419033	3.36E-30	3.63E-29
ENSG00000183048	SLC25A10	46.17259964	66.335759	26.00943995	-1.347007252	0.364977158	-3.6906618	0.000224	0.000644
ENSG00000198298	ZNF485	25.48239458	36.556342	14.40844716	-1.347239604	0.45361913	-2.9699797	0.002978	0.007304
ENSG00000138376	BARD1	101.2505977	145.38207	57.11912112	-1.34780791	0.242661197	-5.5542787	2.79E-08	1.17E-07
ENSG00000176912	TYMSOS	21.80029606	31.263288	12.33730388	-1.348624384	0.541242766	-2.4917181	0.012713	0.027935
ENSG00000184178	SCFD2	505.7046525	726.65593	284.753373	-1.35041631	0.119863415	-11.266293	1.93E-29	2.04E-28
ENSG00000173530	TNFRSF10D	2602.048188	3737.8325	1466.263878	-1.35104605	0.064787877	-20.853377	1.42E-96	4.53E-95
ENSG00000204856	FAM216A	133.1108173	191.27182	74.9498156	-1.352582541	0.212682062	-6.3596456	2.02E-10	9.88E-10
ENSG00000185614	INKA1	228.3555014	328.12923	128.581771	-1.352936345	0.158575046	-8.5318364	1.44E-17	1.02E-16
ENSG00000260628	AC142381.3	14.96770502	21.535734	8.399676277	-1.352983675	0.589334256	-2.2957832	0.021688	0.045286
ENSG00000224383	PRR29	19.03650613	27.362158	10.71085455	-1.353603323	0.534577195	-2.5321008	0.011338	0.025114
ENSG00000064999	ANKS1A	1706.024347	2452.6771	959.3715742	-1.353721879	0.06354637	-21.302899	1.07E-100	3.50E-99
ENSG00000143450	OAZ3	33.98433689	48.855311	19.11336317	-1.353978166	0.40798167	-3.318723	0.000904	0.002398
ENSG00000156140	ADAMTS3	71.90269626	103.46519	40.34020442	-1.358061785	0.271093182	-5.0095756	5.46E-07	2.06E-06
ENSG00000112118	MCM3	1477.985491	2126.8308	829.1401608	-1.358617046	0.067722455	-20.061544	1.60E-89	4.73E-88
ENSG00000108846	ABCC3	110.3596818	158.84589	61.87347157	-1.360906894	0.221837073	-6.1347135	8.53E-10	3.99E-09
ENSG00000109072	VTN	29.32103456	42.288161	16.35390827	-1.362294896	0.449589526	-3.0300859	0.002445	0.006065
ENSG00000123892	RAB38	55.49441297	79.991416	30.99741036	-1.363744333	0.3362393	-4.0558743	4.99E-05	0.000156
ENSG00000177432	NAP1L5	256.4642178	369.37057	143.5578653	-1.366626652	0.160520963	-8.5136958	1.68E-17	1.19E-16
ENSG00000188488	SERPINA5	26.05090047	37.586813	14.51498758	-1.366982937	0.478544654	-2.8565421	0.004283	0.010232
ENSG00000197905	TEAD4	808.4796641	1165.0791	451.8802632	-1.367294217	0.09089856	-15.041979	3.90E-51	6.64E-50
ENSG00000112208	BAG2	504.9258807	728.42557	281.4261959	-1.370479356	0.127119511	-10.781031	4.23E-27	4.19E-26
ENSG00000180881	CAPS2	25.85249182	37.259829	14.44515486	-1.37196571	0.448237162	-3.0608031	0.002207	0.005516
ENSG00000216775	AL109918.1	27.33535274	39.378432	15.29227367	-1.373107872	0.457298024	-3.0026543	0.002676	0.006609
ENSG00000223749	MIR503HG	20.64556647	29.802345	11.48878769	-1.377423904	0.522547304	-2.6359794	0.008389	0.019043
ENSG00000104738	MCM4	1102.668669	1592.4465	612.8908853	-1.377543907	0.082228259	-16.752682	5.41E-63	1.12E-61
ENSG00000149218	ENDOD1	1657.92574	2394.4078	921.4436398	-1.377641393	0.064039972	-21.512211	1.20E-102	4.02E-101
ENSG00000181744	DIPK2A	1247.155375	1801.5585	692.7522075	-1.377845704	0.084342849	-16.336248	5.45E-60	1.07E-58
ENSG00000198846	TOX	460.8818436	665.93941	255.8242736	-1.379416151	0.124928012	-11.041688	2.40E-28	2.47E-27
ENSG00000112039	FANCE	150.052223	216.88913	83.21531268	-1.380972417	0.206991529	-6.6716374	2.53E-11	1.32E-10
ENSG00000120324	PCDHB10	44.5346277	64.461104	24.60815114	-1.381780534	0.421188512	-3.28067	0.001036	0.002724
ENSG00000189007	ADAT2	127.9167172	184.7816	71.05183133	-1.382807086	0.215336867	-6.4215994	1.35E-10	6.68E-10
ENSG00000132932	ATP8A2	17.86886612	25.881059	9.856673675	-1.388931177	0.551524322	-2.5183498	0.011791	0.026019
ENSG00000105767	CADM4	248.1394062	359.07285	137.2059608	-1.38910555	0.153612931	-9.0428946	1.53E-19	1.17E-18
ENSG00000136261	BZW2	2048.185203	2964.3078	1132.062579	-1.389120815	0.06226827	-22.308646	3.05E-110	1.11E-108
ENSG00000135324	MRAP2	58.11294195	84.035426	32.19045754	-1.389190373	0.318251866	-4.3650659	1.27E-05	4.22E-05
ENSG00000074181	NOTCH3	29.14876334	42.107302	16.19022431	-1.390873916	0.485466978	-2.8650227	0.00417	0.009988
ENSG00000091651	ORC6	168.5417768	244.17392	92.90963405	-1.391069282	0.189549128	-7.3388324	2.15E-13	1.25E-12
ENSG00000186638	KIF24	72.66429846	105.073	40.25560054	-1.391130438	0.303219205	-4.5878705	4.48E-06	1.55E-05
ENSG00000128591	FLNC	14527.62923	21035.218	8020.040509	-1.391291039	0.055328721	-25.14591	1.57E-139	7.11E-138
ENSG00000154639	CXADR	229.1453697	331.89669	126.3940521	-1.392382135	0.154895194	-8.9891887	2.49E-19	1.90E-18
ENSG00000250899	AC125807.2	350.6975209	508.12539	193.2696558	-1.393680127	0.128027971	-10.885747	1.35E-27	1.36E-26
ENSG00000240694	PNMA2	885.1245121	1281.985	488.2640176	-1.393773335	0.090574616	-15.388123	1.97E-53	3.47E-52
ENSG00000105784	RUNDC3B	57.20212597	82.791195	31.61305693	-1.396573434	0.330182455	-4.2297021	2.34E-05	7.55E-05
ENSG00000125966	MMP24	27.39572588	39.701108	15.09034376	-1.398103	0.442834509	-3.1571681	0.001593	0.004069
ENSG00000185551	NR2F2	5843.287463	8473.1367	3213.438222	-1.398605837	0.051432721	-27.192919	7.88E-163	4.22E-161
ENSG00000182568	SATB1	247.8316358	359.61505	136.0482239	-1.401631307	0.167692038	-8.3583653	6.36E-17	4.38E-16
ENSG00000147862	NFIB	5952.905707	8637.7585	3268.052883	-1.402276959	0.046384407	-30.231646	9.09E-201	6.04E-199
ENSG00000006468	ETV1	50.56018679	73.402609	27.71776412	-1.406805227	0.357917314	-3.9305314	8.48E-05	0.000257
ENSG00000138162	TACC2	1196.690426	1737.9222	655.4586962	-1.408414072	0.088172346	-15.973422	1.96E-57	3.67E-56
ENSG00000153707	PTPRD	28.16059847	40.940737	15.38046024	-1.411359132	0.43861391	-3.217771	0.001292	0.003355
ENSG00000188211	NCR3LG1	550.0592091	799.60063	300.5177915	-1.411637706	0.117123931	-12.052513	1.88E-33	2.20E-32
ENSG00000054598	FOXC1	551.0787617	801.22809	300.9294356	-1.41228455	0.102111743	-13.830775	1.66E-43	2.48E-42
ENSG00000185238	PRMT3	382.2367744	555.74507	208.7284739	-1.413782335	0.137373359	-10.291532	7.69E-25	7.10E-24
ENSG00000164087	POC1A	139.0538919	202.23395	75.87382918	-1.414499644	0.230562443	-6.1349959	8.52E-10	3.99E-09
ENSG00000179841	AKAP5	16.90898307	24.592357	9.2256089	-1.415199595	0.559493662	-2.5294292	0.011425	0.025286
ENSG00000079257	LXN	1375.22386	2000.8267	749.6210434	-1.415611747	0.077009292	-18.38235	1.82E-75	4.51E-74
ENSG00000145604	SKP2	550.8157611	801.38746	300.2440596	-1.417040259	0.102958861	-13.763169	4.25E-43	6.28E-42
ENSG00000101265	RASSF2	1460.718379	2125.7378	795.6989312	-1.417428471	0.06824939	-20.768368	8.37E-96	2.64E-94
ENSG00000164104	HMGB2	1146.527453	1668.4995	624.5554366	-1.417440982	0.078938448	-17.956281	4.29E-72	1.02E-70
ENSG00000198774	RASSF9	105.6778334	153.81508	57.54058446	-1.41954591	0.230008036	-6.1717231	6.75E-10	3.19E-09
ENSG00000198719	DLL1	157.8761176	229.82973	85.92250079	-1.419659845	0.22234488	-6.3849451	1.71E-10	8.44E-10
ENSG00000261534	AL596244.1	150.8300227	219.4745	82.18554659	-1.421748998	0.222247274	-6.3971493	1.58E-10	7.81E-10

ENSG00000157617	C2CD2	1031.760789	1503.1019	560.419715	-1.423597175	0.083419685	-17.065483	2.68E-65	5.78E-64
ENSG00000197008	ZNF138	93.12405728	135.70746	50.54065873	-1.423683794	0.248258284	-5.734688	9.77E-09	4.23E-08
ENSG00000225177	AL590617.2	66.22806386	96.517996	35.93813163	-1.423973064	0.307067962	-4.6373222	3.53E-06	1.24E-05
ENSG00000152642	GPD1L	522.9750604	761.88137	284.0687474	-1.424160833	0.11295586	-12.608118	1.90E-36	2.40E-35
ENSG00000077782	FGFR1	3991.842695	5816.938	2166.747369	-1.424493654	0.049119218	-29.00074	6.44E-185	3.93E-183
ENSG00000167992	VWCE	574.9051774	837.39332	312.4170325	-1.424679695	0.109946544	-12.957931	2.12E-38	2.80E-37
ENSG00000164850	GPER1	61.64724408	89.85459	33.43989792	-1.425395606	0.311652847	-4.5736646	4.79E-06	1.66E-05
ENSG00000172731	LRRC20	238.7935539	348.02343	129.5636785	-1.425943424	0.151427115	-9.4166981	4.65E-21	3.78E-20
ENSG00000109654	TRIM2	353.1713634	514.94805	191.3946744	-1.426278642	0.132522531	-10.762537	5.17E-27	5.11E-26
ENSG00000178445	GLDC	306.4383458	446.85036	166.0263345	-1.428491429	0.14700091	-9.7175686	2.54E-22	2.15E-21
ENSG00000182287	AP1S2	2104.96354	3069.6414	1140.285704	-1.428765438	0.072509138	-19.704626	1.97E-86	5.57E-85
ENSG00000226360	RPL10AP6	32.88936795	48.048308	17.73042821	-1.429245204	0.446996091	-3.1974445	0.001387	0.003576
ENSG00000112699	GMDS	403.9276349	589.46219	218.3930839	-1.430472911	0.134793262	-10.612347	2.61E-26	2.53E-25
ENSG00000135636	DYSF	13643.04442	19931.686	7354.402647	-1.438484049	0.037694733	-38.161407	0	0
ENSG00000137309	HMGA1	22968.02569	33555.469	12380.58272	-1.438499145	0.121374417	-11.851749	2.11E-32	2.41E-31
ENSG00000177076	ACER2	226.3177118	330.59892	122.0364994	-1.439861451	0.166320981	-8.6571246	4.84E-18	3.47E-17
ENSG00000162616	DNAJB4	4842.583661	7078.0508	2607.116534	-1.440894707	0.048281133	-29.843846	1.06E-195	6.74E-194
ENSG00000226702	MIR217HG	128.1993283	187.47736	68.92129348	-1.441598849	0.214489622	-6.7210657	1.80E-11	9.48E-11
ENSG00000117586	TNFSF4	2306.261043	3374.5565	1237.965575	-1.445975266	0.067432262	-21.443375	5.27E-102	1.76E-100
ENSG00000006634	DBF4	336.9852089	493.39461	180.5758069	-1.449297013	0.133313779	-10.871322	1.58E-27	1.59E-26
ENSG00000137225	CAPN11	168.7729121	247.14916	90.39666674	-1.450374537	0.181504942	-7.9908267	1.34E-15	8.68E-15
ENSG00000182545	RNASE10	35.64893897	52.219825	19.07805285	-1.452674158	0.395958138	-3.6687569	0.000244	0.000698
ENSG00000081853	PCDHGA2	73.41928993	107.72175	39.11682699	-1.45686425	0.275659379	-5.2850161	1.26E-07	5.00E-07
ENSG00000073670	ADAM11	14.4739405	21.200456	7.747425314	-1.457635331	0.633186103	-2.3020646	0.021332	0.04463
ENSG00000235750	KIAA0040	591.4340121	867.25283	315.6151974	-1.45820042	0.115130134	-12.665671	9.16E-37	1.17E-35
ENSG00000170421	KRT8	431.0656801	632.23024	229.9011166	-1.460545493	0.128171126	-11.395277	4.41E-30	4.75E-29
ENSG00000121005	CRISPLD1	60.65311254	89.016252	32.28997346	-1.461230288	0.311522601	-4.6906076	2.72E-06	9.68E-06
ENSG00000224945	AL353150.1	27.18394991	39.843665	14.52423494	-1.462656229	0.456493948	-3.2041087	0.001355	0.003503
ENSG00000214199	EEF1A1P12	15.78368361	23.200169	8.367198302	-1.464968725	0.580973516	-2.5215757	0.011683	0.025799
ENSG00000224189	HAGLR	94.81242821	139.21899	50.40587007	-1.46498719	0.239304842	-6.1218452	9.25E-10	4.31E-09
ENSG00000175322	ZNF519	18.00521708	26.425523	9.584911048	-1.465975936	0.563295344	-2.6024996	0.009255	0.020852
ENSG00000196154	S100A4	13.91185811	20.420072	7.403644675	-1.466158083	0.617608919	-2.3739263	0.0176	0.037563
ENSG00000168824	NSG1	535.9661308	787.39552	284.5367376	-1.469662591	0.11014871	-13.342531	1.31E-40	1.82E-39
ENSG00000102384	CENPI	198.0935398	291.13914	105.0479437	-1.472908713	0.181390516	-8.1200977	4.66E-16	3.08E-15
ENSG00000176974	SHMT1	302.3987269	444.37951	160.4179441	-1.473151747	0.150865796	-9.7646503	1.60E-22	1.37E-21
ENSG00000185955	C7orf61	13.13262257	19.321169	6.944076263	-1.473250267	0.630420384	-2.3369331	0.019443	0.041081
ENSG00000114315	HES1	569.5051002	837.89887	301.1113325	-1.473844809	0.115398209	-12.771817	2.36E-37	3.04E-36
ENSG00000113083	LOX	3085.392389	4537.2055	1633.579239	-1.473849686	0.054725183	-26.931837	9.31E-160	4.78E-158
ENSG00000102879	CORO1A	52.94680144	77.786853	28.10674948	-1.473873283	0.344719461	-4.2755732	1.91E-05	6.21E-05
ENSG00000198959	TGM2	48691.66772	71668.758	25714.57784	-1.478790031	0.044512293	-33.222059	5.17E-242	4.44E-240
ENSG00000120693	SMAD9	229.2419935	337.39627	121.0877169	-1.479353222	0.161609289	-9.1538873	5.49E-20	4.30E-19
ENSG00000130307	USHBP1	148.1971171	218.26852	78.12571619	-1.479571443	0.208122885	-7.1091242	1.17E-12	6.54E-12
ENSG00000182492	BGN	14908.45972	21952.049	7864.869977	-1.480866491	0.161255136	-9.1833756	4.18E-20	3.28E-19
ENSG00000167895	TMC8	24.26800059	35.77519	12.76081164	-1.481665955	0.469622903	-3.1550121	0.001605	0.004096
ENSG00000115641	FHL2	3833.684091	5645.8841	2021.484032	-1.482090724	0.051408117	-28.829897	9.05E-183	5.45E-181
ENSG00000272031	ANKRD34A	27.84865249	41.021325	14.67598007	-1.482333763	0.455071562	-3.2573641	0.001125	0.002944
ENSG00000179144	GIMAP7	1877.76879	2766.4139	989.1236988	-1.483155475	0.075669764	-19.600371	1.53E-85	4.32E-84
ENSG00000058804	NDC1	663.4645177	977.56744	349.3615963	-1.484301355	0.105829023	-14.025466	1.09E-44	1.66E-43
ENSG00000063660	GPC1	2508.031597	3696.0636	1319.99963	-1.485528671	0.059174136	-25.104358	4.46E-139	2.01E-137
ENSG00000213023	SYT3	40.54681057	59.766514	21.32710752	-1.48877607	0.374579279	-3.9745286	7.05E-05	0.000216
ENSG00000175130	MARCKSL1	6715.565954	9904.173	3526.958944	-1.489968062	0.045065112	-33.062562	1.03E-239	8.65E-238
ENSG00000144712	CAND2	71.57314049	105.68698	37.45929706	-1.49004494	0.303517685	-4.9092524	9.14E-07	3.39E-06
ENSG00000167799	NUDT8	74.08884005	109.2796	38.89808149	-1.495794024	0.304111648	-4.9185687	8.72E-07	3.24E-06
ENSG00000181019	NQO1	5359.222212	7914.9377	2803.506728	-1.4974985	0.062355792	-24.015387	1.92E-127	8.13E-126
ENSG00000132470	ITGB4	56.67159157	83.791478	29.55170475	-1.499523514	0.361453717	-4.1485907	3.35E-05	0.000106
ENSG00000141505	ASGR1	139.402598	205.98725	72.81794609	-1.500807824	0.204773935	-7.329096	2.32E-13	1.34E-12
ENSG00000164684	ZNF704	713.5805145	1055.2561	371.9049159	-1.503292987	0.120280085	-12.49827	7.63E-36	9.48E-35
ENSG00000076003	MCM6	836.646157	1237.4026	435.889746	-1.505468767	0.09369925	-16.067031	4.34E-58	8.25E-57
ENSG00000110237	ARHGEF17	3790.210937	5606.7271	1973.694762	-1.506167512	0.055412268	-27.18112	1.09E-162	5.80E-161
ENSG00000154529	CNTNAP3B	428.4618263	633.92367	222.9999847	-1.507220781	0.123178412	-12.236079	1.99E-34	2.38E-33
ENSG00000176894	PXMP2	14.94583744	22.093418	7.798257138	-1.508769108	0.592586058	-2.5460759	0.010894	0.024208
ENSG00000134986	NREP	4140.7809	6129.8718	2151.689972	-1.509813357	0.053422497	-28.261752	1.02E-175	5.90E-174
ENSG00000162817	C1orf115	1712.791755	2535.8332	889.750312	-1.511640327	0.074430751	-20.309352	1.06E-91	3.22E-90
ENSG00000145990	GFOD1	1017.577854	1506.9018	528.2538855	-1.511965967	0.080836933	-18.703901	4.60E-78	1.17E-76
ENSG00000253882	AC099548.2	51.42372256	76.105924	26.74152133	-1.513785207	0.344844584	-4.3897607	1.13E-05	3.78E-05
ENSG00000160712	IL6R	240.4283931	356.33345	124.523338	-1.5144991	0.161314604	-9.388481	6.09E-21	4.92E-20
ENSG00000134253	TRIM45	38.72764353	57.309832	20.14545543	-1.515123918	0.411194054	-3.6846932	0.000229	0.000658
ENSG00000237037	NDUFA6-DT	14.13429632	20.962382	7.30621075	-1.515663285	0.654284644	-2.3165197	0.02053	0.04313
ENSG00000105472	CLEC11A	396.0485018	586.89516	205.2018433	-1.517497223	0.154786394	-9.8038153	1.08E-22	9.32E-22
ENSG00000224078	SNHG14	981.0297393	1453.9614	508.0980295	-1.517577701	0.082075314	-18.490063	2.48E-76	6.20E-75
ENSG00000165140	FBP1	28.5516837	42.270662	14.83270526	-1.518309283	0.457677252	-3.3174235	0.000909	0.002408
ENSG00000178921	PFAS	956.3234061	1417.3554	495.2914318	-1.518691678	0.091267141	-16.640071	3.57E-62	7.23E-61
ENSG00000160796	NBEAL2	2511.064463	3723.5837	1298.545237	-1.520448484	0.074764177	-20.336591	6.10E-92	1.86E-90
ENSG00000164237	CMBL	875.9712657	1299.6509	452.291588	-1.52124271	0.089979005	-16.906641	4.02E-64	8.48E-63
ENSG00000232593	KANTR	92.0694982	136.71143	47.42756636	-1.522189429	0.268744881	-5.6640686	1.48E-08	6.33E-08
ENSG00000092470	WDR76	192.2895438	285.21026	99.36882929	-1.522303048	0.185559563	-8.2038512	2.33E-16	1.57E-15
ENSG00000156011	PSD3	942.7110514	1400.0237	485.3983876	-1.527664103	0.085571589	-17.852469	2.77E-71	6.52E-70
ENSG00000153044	CENPH	107.5171109	159.61418	55.42004431	-1.529962636	0.260531575	-5.8724653	4.29E-09	1.91E-08
ENSG00000111424	VDR	54.24065667	80.590618	27.89069544	-1.530322824	0.31234782	-4.8994189	9.61E-07	3.56E-06
ENSG00000254827	SLC22A18AS	28.0518695	41.662814	14.44092514	-1.531785339	0.442619159	-3.4607299	0.000539	0.001475
ENSG00000260398	AC068700.1	30.85304029	45.826512	15.87956869	-1.534120683	0.429494579	-3.5719209	0.000354	0.000992
ENSG00000111145	ELK3	5198.639258	7729.4449	2667.83359	-1.534575748	0.051273062	-29.929474	8.14E-197	5.27E-195
ENSG00000100297	MCM5	932.0848845	1386.0861	478.0836798	-1.535517261	0.095755674	-16.035784	7.19E-58	1.36E-56
ENSG00000078114	NEBL	28.53768659	42.513784	14.56158968	-1.538360376	0.450245351	-3.4167157	0.000634	0.001717
ENSG00000184185	KCNJ12	23.97595823	35.607551	12.34436595	-1.540021174	0.504787833	-3.0508286	0.002282	0.005692

ENSG00000283537	AC073264.3	20.09153635	29.896476	10.28659645	-1.540586812	0.53349183	-2.8877421	0.00388	0.009347
ENSG00000134291	TMEM106C	626.620142	933.02455	320.2157301	-1.542348628	0.101353417	-15.21753	2.71E-52	4.71E-51
ENSG00000182022	CHST15	1017.930216	1516.2033	519.6571561	-1.543731332	0.087029089	-17.738107	2.13E-70	4.92E-69
ENSG00000169126	ARMC4	15.92573761	23.684022	8.167453689	-1.544028014	0.598912691	-2.5780519	0.009936	0.022229
ENSG00000185532	PRKG1	73.80384389	110.0326	37.57508484	-1.545448592	0.291990086	-5.2928119	1.20E-07	4.80E-07
ENSG00000143816	WNT9A	164.5394599	245.2162	83.86272445	-1.547430798	0.187892262	-8.2357346	1.78E-16	1.20E-15
ENSG00000107719	PALD1	2198.029365	3278.4194	1117.639361	-1.552538605	0.073284273	-21.185154	1.31E-99	4.28E-98
ENSG00000260641	AC114811.2	22.37434712	33.394048	11.35464607	-1.553795358	0.509838506	-3.0476226	0.002307	0.005744
ENSG00000255198	SNHG9	14.61133973	21.765371	7.457308839	-1.553899579	0.642714934	-2.417712	0.015618	0.033659
ENSG00000115556	PLCD4	94.01221156	140.13012	47.89430014	-1.555121218	0.25948878	-5.9930191	2.06E-09	9.39E-09
ENSG00000180998	GPR137C	98.59661744	147.23708	49.95615848	-1.558197174	0.267734994	-5.8199235	5.89E-09	2.59E-08
ENSG00000205444	AL034376.1	19.82137558	29.55167	10.09108156	-1.560052258	0.55069067	-2.8329012	0.004613	0.010948
ENSG00000261308	FIGNL2	44.92989806	67.170293	22.68950333	-1.562244712	0.345116857	-4.5267123	5.99E-06	2.05E-05
ENSG00000185480	PARPBP	174.6246994	260.90552	88.34387731	-1.564021411	0.192963988	-8.1052502	5.26E-16	3.47E-15
ENSG00000161513	FDXR	998.5312395	1492.5203	504.5422125	-1.56519083	0.086815134	-18.029009	1.15E-72	2.77E-71
ENSG00000111863	ADTRP	141.8061455	211.90394	71.70834959	-1.565999971	0.215496558	-7.2669373	3.68E-13	2.11E-12
ENSG00000163629	PTPN13	181.9439688	272.11619	91.77175172	-1.566832632	0.172626988	-9.076406	1.12E-19	8.67E-19
ENSG00000142634	EFHD2	2624.387564	3927.1852	1321.589945	-1.571754254	0.082290463	-19.100078	2.52E-81	6.75E-80
ENSG00000267583	AC007998.3	683.4179997	1023.4127	343.4233052	-1.574047413	0.095957447	-16.403598	1.80E-60	3.55E-59
ENSG00000007129	CEACAM21	69.60194644	104.2053	34.99859654	-1.575428865	0.31324062	-5.0294526	4.92E-07	1.87E-06
ENSG00000107485	GATA3	329.4945878	493.44424	165.5449329	-1.577424736	0.134220268	-11.752508	6.86E-32	7.70E-31
ENSG00000280152	AC009078.3	23.04453918	34.491565	11.5975134	-1.578984937	0.54631623	-2.8902398	0.003849	0.009281
ENSG00000197061	HIST1H4C	23.37023717	35.047712	11.69276203	-1.580612645	0.484206024	-3.2643391	0.001097	0.002877
ENSG00000127586	CHTF18	193.1328334	289.52068	96.74498854	-1.581671897	0.17828477	-8.8716041	7.21E-19	5.39E-18
ENSG00000174514	MFSD4A	12.39267518	18.620605	6.16474574	-1.583687797	0.677329896	-2.3381336	0.01938	0.040972
ENSG00000128849	CGNL1	3459.003378	5188.5377	1729.469014	-1.584586491	0.054585775	-29.029294	2.81E-185	1.72E-183
ENSG00000236548	RNF217-AS1	11.78090802	17.687187	5.874629265	-1.587646382	0.692012131	-2.2942465	0.021776	0.045454
ENSG00000087586	AURKA	522.6468813	784.64334	260.650426	-1.58829373	0.130010447	-12.216662	2.53E-34	3.01E-33
ENSG00000148057	IDNK	49.03511015	73.694329	24.37589097	-1.589640652	0.387393087	-4.1034306	4.07E-05	0.000128
ENSG00000184500	PROS1	1007.543304	1513.1607	501.9259399	-1.590726681	0.091923313	-17.304932	4.32E-67	9.57E-66
ENSG00000003096	KLHL13	518.8025304	779.23391	258.3711538	-1.590911444	0.115548955	-13.768289	3.95E-43	5.86E-42
ENSG00000128918	ALDH1A2	26.59519611	39.95872	13.23167184	-1.592435081	0.478503076	-3.3279516	0.000875	0.002326
ENSG00000214595	EML6	28.98771268	43.551457	14.42396867	-1.593309073	0.507718065	-3.1381768	0.0017	0.004319
ENSG00000120437	ACAT2	693.220194	1041.089	345.351407	-1.593569459	0.111300232	-14.317755	1.70E-46	2.66E-45
ENSG00000017483	SLC38A5	413.8399515	621.54	206.1399059	-1.593609591	0.135414342	-11.768396	5.68E-32	6.39E-31
ENSG00000116133	DHCR24	6716.267461	10094.689	3337.846279	-1.596737053	0.069825845	-22.867422	9.81E-116	3.74E-114
ENSG00000111445	RFC5	256.9195292	385.9441	127.8949601	-1.596739678	0.162513498	-9.8252742	8.76E-23	7.56E-22
ENSG00000162595	DIRAS3	188.1609503	282.81071	93.51119406	-1.598075803	0.179465447	-8.9046433	5.36E-19	4.02E-18
ENSG00000160117	ANKLE1	16.95692218	25.502876	8.410968063	-1.598213271	0.568440101	-2.8115773	0.00493	0.01164
ENSG00000172572	PDE3A	722.3931556	1086.3192	358.4671126	-1.599338755	0.098326814	-16.26554	1.73E-59	3.37E-58
ENSG00000204936	CD177	22.90536592	34.484334	11.32639782	-1.600351091	0.489734308	-3.2677945	0.001084	0.002846
ENSG00000170545	SMAGP	708.565004	1065.9918	351.1381774	-1.601831287	0.120553665	-13.287288	2.74E-40	3.78E-39
ENSG00000119969	HELLS	268.4385695	404.29647	132.5806686	-1.606267115	0.15767621	-10.187124	2.26E-24	2.05E-23
ENSG00000166582	CENPV	179.1782774	269.7621	88.59445375	-1.606662695	0.178041175	-9.0241075	1.81E-19	1.39E-18
ENSG00000084674	APOB	95.11817149	143.28882	46.94752453	-1.607743375	0.31603179	-5.0872837	3.63E-07	1.40E-06
ENSG00000186862	PDZD7	14.96342795	22.526044	7.400812335	-1.607961866	0.692130778	-2.3232053	0.020168	0.042432
ENSG00000149582	TMEM25	523.781224	788.94488	258.6175663	-1.608461951	0.106216113	-15.143295	8.39E-52	1.45E-50
ENSG00000051180	RAD51	129.0138523	194.29413	63.73357845	-1.60952767	0.212115516	-7.5879771	3.25E-14	1.96E-13
ENSG00000260912	AL158206.1	118.4985156	178.59739	58.3996421	-1.61039501	0.227567225	-7.0765683	1.48E-12	8.24E-12
ENSG00000148082	SHC3	565.2875977	851.4922	279.0829998	-1.61069185	0.121629826	-13.242573	4.98E-40	6.84E-39
ENSG00000108798	ABI3	898.4859042	1353.7659	443.2058887	-1.611232062	0.097938266	-16.451507	8.18E-61	1.62E-59
ENSG00000250510	GPR162	161.5074627	243.43426	79.58066916	-1.61358154	0.191496191	-8.4261808	3.57E-17	2.49E-16
ENSG00000250073	AP000866.2	12.87678993	19.375612	6.377967438	-1.615692828	0.68840541	-2.3470077	0.018925	0.040104
ENSG00000162390	ACOT11	95.58450283	144.11695	47.05205809	-1.61570953	0.242309502	-6.6679578	2.59E-11	1.35E-10
ENSG00000198865	CCDC152	71.72866283	108.19407	35.26325952	-1.616911938	0.288412591	-5.606246	2.07E-08	8.77E-08
ENSG00000196872	KIAA1211L	334.744814	505.70751	163.782119	-1.625156017	0.133792442	-12.146845	5.96E-34	7.03E-33
ENSG00000164649	CDCA7L	1405.157156	2122.7539	687.5604036	-1.626124188	0.072561932	-22.410156	3.13E-111	1.15E-109
ENSG00000175745	NR2F1	1368.829513	2069.3833	668.275685	-1.631226029	0.087368621	-18.670617	8.59E-78	2.17E-76
ENSG00000109084	TMEM97	306.6575606	463.49167	149.8234492	-1.631355178	0.154625275	-10.550379	5.06E-26	4.86E-25
ENSG00000175874	CREG2	58.79074668	88.945343	28.63615061	-1.632845212	0.308308115	-5.2961474	1.18E-07	4.72E-07
ENSG00000125885	MCM8	66.36669615	100.22661	32.50677784	-1.633161953	0.341612186	-4.7807485	1.75E-06	6.32E-06
ENSG00000138650	PCDH10	2395.87479	3624.0964	1167.653151	-1.635039774	0.07095845	-23.042214	1.76E-117	6.81E-116
ENSG00000231527	FAM27C	38.48488	58.217134	18.75262606	-1.635499432	0.380898615	-4.293792	1.76E-05	5.74E-05
ENSG00000069702	TGFBR3	1170.293899	1771.1525	569.435328	-1.63632104	0.080372523	-20.35921	3.85E-92	1.17E-90
ENSG00000188610	FAM72B	42.43698212	64.210399	20.66356477	-1.636394077	0.367969951	-4.4470862	8.70E-06	2.93E-05
ENSG00000279692	AC110285.7	34.6728603	52.506828	16.83889259	-1.637521579	0.401319324	-4.0803457	4.50E-05	0.000141
ENSG00000251257	AC010457.1	23.52446433	35.577097	11.47183122	-1.638696995	0.495101784	-3.3098184	0.000934	0.00247
ENSG00000126878	AIF1L	1587.725862	2403.9924	771.4593672	-1.639826999	0.074282288	-22.075612	5.42E-108	1.91E-106
ENSG00000173207	CKS1B	299.5616253	453.41172	145.7115304	-1.640228701	0.14594275	-11.23885	2.63E-29	2.77E-28
ENSG00000199753	SNORD104	13.68094418	20.726282	6.635605939	-1.642857514	0.625226631	-2.6276192	0.008598	0.019478
ENSG00000196730	DAPK1	3082.117471	4669.1678	1495.067162	-1.642954195	0.05909238	-27.803148	3.97E-170	2.24E-168
ENSG00000234160	AL513165.1	19.66589115	29.823119	9.508663313	-1.645339987	0.522500868	-3.1489708	0.001638	0.004175
ENSG00000203727	SAMD5	104.7160609	158.7813	50.6508194	-1.645659758	0.261814197	-6.2856017	3.27E-10	1.57E-09
ENSG00000162599	NFIA	918.1263367	1391.8658	444.3868938	-1.646816254	0.086149958	-19.115694	1.87E-81	5.02E-80
ENSG00000198807	PAX9	104.6977833	158.65506	50.74050665	-1.649417159	0.251889501	-6.5481775	5.82E-11	2.96E-10
ENSG00000168497	CAVIN2	9260.726778	14056.65	4464.803552	-1.654400701	0.042879973	-38.58213	0	0
ENSG00000164604	GPR85	14.55089995	22.071581	7.030218401	-1.654886057	0.608252423	-2.7207225	0.006514	0.015066
ENSG00000169247	SH3TC2	73.68704948	111.86654	35.50756181	-1.659514959	0.2764029	-6.0039709	1.93E-09	8.79E-09
ENSG00000183323	CCDC125	182.8159708	277.61837	88.01357376	-1.660442003	0.182514638	-9.0975827	9.24E-20	7.15E-19
ENSG00000176105	YES1	5219.128626	7930.868	2507.389256	-1.661600616	0.048803599	-34.046682	4.54E-254	4.25E-252
ENSG00000116574	RHOU	40.2173322	61.218991	19.21567386	-1.661875543	0.406512832	-4.0881257	4.35E-05	0.000137
ENSG00000173221	GLRX	382.9250415	582.27554	183.5745465	-1.664590592	0.127076855	-13.099086	3.33E-39	4.49E-38
ENSG00000073111	MCM2	656.8006815	998.57199	315.0293749	-1.664648585	0.100833581	-16.508871	3.17E-61	6.31E-60
ENSG00000183018	AC118754.1	951.0818656	1446.1269	456.0368148	-1.665277342	0.09637986	-17.278271	6.86E-67	1.51E-65
ENSG00000197852	INKA2	895.423907	1362.5119	428.3359038	-1.669568206	0.091045449	-18.337745	4.14E-75	1.02E-73

ENSG00000147119	CHST7	347.7282351	529.16121	166.2952648	-1.671208306	0.150971728	-11.069677	1.76E-28	1.82E-27
ENSG00000175305	CCNE2	111.8422359	170.31313	53.37134373	-1.671244385	0.23893266	-6.9946251	2.66E-12	1.47E-11
ENSG00000273080	AC009309.1	10.72793758	16.340825	5.115049974	-1.672405273	0.724953927	-2.3069125	0.02106	0.04411
ENSG00000157193	LRP8	929.8397177	1415.8207	443.8587491	-1.67318521	0.095533079	-17.514197	1.12E-68	2.53E-67
ENSG00000146918	NCAPG2	568.4058615	865.56039	271.2513359	-1.673198411	0.106968877	-15.641918	3.77E-55	6.83E-54
ENSG00000198478	SH3BGRL2	859.5932024	1309.2234	409.9630439	-1.675818879	0.092583684	-18.100585	3.15E-73	7.62E-72
ENSG00000160949	TONSL	254.6046678	387.73755	121.4717879	-1.676625455	0.167089026	-10.034324	1.08E-23	9.59E-23
ENSG00000146376	ARHGAP18	3755.51121	5722.098	1788.92441	-1.676925421	0.058851178	-28.494339	1.38E-178	8.11E-177
ENSG00000135480	KRT7	5852.942868	8918.721	2787.164732	-1.678159505	0.131379834	-12.773342	2.31E-37	2.98E-36
ENSG00000235387	SPAAR	201.0410207	306.48641	95.59563598	-1.679219155	0.184097938	-9.121336	7.42E-20	5.77E-19
ENSG00000232445	AC006329.1	36.46339988	55.588799	17.33800104	-1.681053696	0.432784808	-3.8842715	0.000103	0.000307
ENSG00000081189	MEF2C	724.2585151	1104.4592	344.0578118	-1.681054909	0.103886121	-16.181708	6.79E-59	1.31E-57
ENSG00000227811	INKA2-AS1	16.01403397	24.450095	7.577973377	-1.681865791	0.671437076	-2.5048748	0.012249	0.026971
ENSG00000116396	KCNC4	486.3696122	742.06376	230.6754671	-1.686030677	0.119284812	-14.134496	2.33E-45	3.58E-44
ENSG00000114737	CISH	16.36147069	24.943038	7.779903289	-1.687167484	0.583560318	-2.8911621	0.003838	0.009256
ENSG00000079616	KIF22	451.4637041	689.65289	213.274517	-1.692088839	0.11411099	-14.828448	9.59E-50	1.60E-48
ENSG00000197121	PGAP1	894.8975234	1366.8001	422.9949712	-1.692816831	0.085024644	-19.90972	3.35E-88	9.67E-87
ENSG00000140350	ANP32A	2663.549578	4069.388	1257.711148	-1.69376635	0.056408641	-30.026718	4.40E-198	2.88E-196
ENSG00000275322	AC103746.1	15.48267907	23.695855	7.269503053	-1.695376039	0.614791482	-2.757644	0.005822	0.013581
ENSG00000131153	GINS2	165.4357708	252.76199	78.10954764	-1.696464571	0.204099424	-8.3119518	9.41E-17	6.44E-16
ENSG00000198721	ECI2	3087.121368	4719.7505	1454.49226	-1.698721662	0.071296994	-23.825993	1.80E-125	7.48E-124
ENSG00000109881	CCDC34	197.0797115	301.29625	92.86317282	-1.699633731	0.172055361	-9.878412	5.16E-23	4.48E-22
ENSG00000130005	GAMT	317.7416167	485.88501	149.5982229	-1.700085449	0.14274765	-11.909726	1.05E-32	1.21E-31
ENSG00000162174	ASRGL1	893.9368732	1367.2895	420.5842112	-1.700540924	0.090137107	-18.866158	2.16E-79	5.67E-78
ENSG00000187210	GCNT1	374.3727552	572.78565	175.9598654	-1.701556081	0.128624292	-13.228886	5.98E-40	8.19E-39
ENSG00000117632	STMN1	3428.031422	5244.0542	1612.008626	-1.701934459	0.052052309	-32.696618	1.74E-234	1.39E-232
ENSG00000156398	SFXN2	87.89054549	134.45963	41.32146485	-1.702830106	0.262069158	-6.4976364	8.16E-11	4.10E-10
ENSG00000095713	CRTAC1	207.1601673	316.99297	97.32736922	-1.704568592	0.170056691	-10.023531	1.20E-23	1.07E-22
ENSG00000276644	DACH1	179.4731517	274.80404	84.14226191	-1.709603586	0.207234854	-8.2495949	1.59E-16	1.08E-15
ENSG00000101134	DOK5	264.2415053	404.59268	123.8903321	-1.710720827	0.160055072	-10.688326	1.15E-26	1.13E-25
ENSG00000129990	SYT5	11.58845804	17.797166	5.379750537	-1.711815723	0.746824829	-2.2921248	0.021898	0.045684
ENSG00000108830	RND2	37.69802104	57.789111	17.60693133	-1.714634381	0.390923036	-4.3861175	1.15E-05	3.84E-05
ENSG00000085563	ABCB1	1010.652996	1549.5091	471.7969097	-1.715112098	0.082759275	-20.724107	2.10E-95	6.60E-94
ENSG00000167600	CYP2S1	22.67328193	34.798099	10.54846468	-1.715252822	0.501073131	-3.4231586	0.000619	0.00168
ENSG00000171388	APLN	36369.67459	55762.653	16976.69575	-1.715670379	0.037280809	-46.020202	0	0
ENSG00000188511	C22orf34	72.56725033	111.25766	33.87684518	-1.716898981	0.318203727	-5.3955967	6.83E-08	2.77E-07
ENSG00000173535	TNFRSF10C	1638.083033	2513.8301	762.3359281	-1.721122972	0.068878518	-24.987805	8.30E-138	3.71E-136
ENSG00000167693	NXN	1656.293015	2542.0616	770.5243989	-1.721144496	0.072166127	-23.849756	1.02E-125	4.27E-124
ENSG00000123179	EBPL	499.7455443	766.83976	232.6513242	-1.721398917	0.136788294	-12.584402	2.57E-36	3.23E-35
ENSG00000124019	FAM124B	2501.431106	3840.6354	1162.226788	-1.723771726	0.064097481	-26.892971	2.65E-159	1.35E-157
ENSG00000100629	CEP128	131.0717847	201.42002	60.72354711	-1.730649946	0.213670552	-8.0996185	5.51E-16	3.63E-15
ENSG00000166337	TAF10	66.560713	102.26368	30.85774493	-1.730655884	0.66758976	-2.5923943	0.009531	0.021422
ENSG00000004776	HSPB6	40.44806502	62.151963	18.74416662	-1.730969386	0.382500436	-4.525405	6.03E-06	2.07E-05
ENSG00000113209	PCDHB5	60.2044287	92.491958	27.91689927	-1.732219108	0.391976891	-4.4191868	9.91E-06	3.32E-05
ENSG00000078596	ITM2A	722.0020937	1110.463	333.541178	-1.734481087	0.109846478	-15.790047	3.64E-56	6.70E-55
ENSG00000117461	PIK3R3	826.8480247	1272.4138	381.2822981	-1.73872247	0.0932358	-18.648657	1.29E-77	3.27E-76
ENSG00000164949	GEM	18.99707865	29.278949	8.715208664	-1.740781231	0.621318472	-2.8017535	0.005083	0.011977
ENSG00000152932	RAB3C	27.09663346	41.764944	12.42832279	-1.742848764	0.46952345	-3.7119525	0.000206	0.000594
ENSG00000134323	MYCN	39.29531017	60.524121	18.06649974	-1.744790748	0.382377084	-4.5630108	5.04E-06	1.74E-05
ENSG00000125531	FNDC11	12.03911066	18.540311	5.537910689	-1.745121004	0.722896195	-2.4140686	0.015775	0.033974
ENSG00000125968	ID1	565.5558405	871.1775	259.9341855	-1.745443856	0.124054232	-14.070007	5.81E-45	8.85E-44
ENSG00000143127	ITGA10	4670.031606	7201.409	2138.654228	-1.751371665	0.057921909	-30.236774	7.79E-201	5.19E-199
ENSG00000138587	MNS1	31.86049828	49.194717	14.52627936	-1.752307773	0.458694399	-3.8202075	0.000133	0.000394
ENSG00000163535	SGO2	418.3112796	645.15014	191.4724228	-1.753420166	0.119020285	-14.732112	4.01E-49	6.60E-48
ENSG00000054654	SYNE2	1402.543158	2165.8516	639.2347458	-1.759583804	0.088935367	-19.784973	4.01E-87	1.15E-85
ENSG00000120802	TMPO	1750.863263	2703.412	798.3145004	-1.759882257	0.079520173	-22.131268	1.58E-108	5.61E-107
ENSG00000204634	TBC1D8	1393.706261	2151.6808	635.7316933	-1.759921661	0.077043046	-22.843355	1.70E-115	6.47E-114
ENSG00000164070	HSPA4L	1008.791096	1558.6486	458.9335714	-1.762789626	0.083324732	-21.155659	2.45E-99	7.98E-98
ENSG00000163584	RPL22L1	1096.576689	1695.7366	497.4167454	-1.769980352	0.083132635	-21.291041	1.37E-100	4.50E-99
ENSG00000178947	SMIM10L2A	46.15591216	71.411807	20.90001708	-1.770054798	0.343605887	-5.1514103	2.59E-07	1.00E-06
ENSG00000147536	GINS4	285.7657324	441.88025	129.651218	-1.770443139	0.141801657	-12.485349	8.97E-36	1.11E-34
ENSG00000233225	AC004987.2	14.6356184	22.679401	6.591836178	-1.779490331	0.623902407	-2.8521934	0.004342	0.010355
ENSG00000140876	NUDT7	45.88383851	71.161397	20.60628035	-1.779801243	0.47063881	-3.7816712	0.000156	0.000456
ENSG00000123689	G0S2	324.755254	502.97663	146.5338804	-1.780064516	0.133350723	-13.348743	1.20E-40	1.68E-39
ENSG00000100526	CDKN3	140.060215	217.24465	62.87577732	-1.784462054	0.212217955	-8.408629	4.15E-17	2.88E-16
ENSG00000122952	ZWINT	452.8621031	701.99919	203.7250195	-1.78628007	0.118598667	-15.061553	2.90E-51	4.95E-50
ENSG00000185527	AC139530.1	9.858681258	15.284209	4.433153376	-1.787530779	0.765076022	-2.3364094	0.01947	0.041124
ENSG00000276386	CNTNAP3P2	294.1385911	456.40591	131.8712741	-1.790391486	0.140016377	-12.787015	1.94E-37	2.51E-36
ENSG00000188153	COL4A5	5732.335766	8895.5169	2569.154601	-1.791287254	0.051245848	-34.954778	1.10E-267	1.09E-265
ENSG00000198056	PRIM1	61.28121128	95.086738	27.47568471	-1.797080921	0.354465357	-5.0698351	3.98E-07	1.53E-06
ENSG00000259291	ZNF710-AS1	45.23143348	70.222163	20.24070405	-1.797493605	0.387823304	-4.6348262	3.57E-06	1.25E-05
ENSG00000168306	ACOX2	18.97841055	29.466773	8.490048139	-1.798522695	0.547608967	-3.2843193	0.001022	0.00269
ENSG00000205336	ADGRG1	6264.505477	9735.0875	2793.923487	-1.801029982	0.057384655	-31.385219	3.22E-216	2.34E-214
ENSG00000275160	AL354718.1	44.61774917	69.284911	19.95058758	-1.801190357	0.366071753	-4.9203211	8.64E-07	3.21E-06
ENSG00000134508	CABLES1	2276.341903	3540.8998	1011.783989	-1.807186531	0.066556564	-27.152642	2.36E-162	1.25E-160
ENSG00000076554	TPD52	10.09912121	15.681779	4.516463175	-1.808211917	0.800173429	-2.259775	0.023835	0.049335
ENSG00000203760	CENPW	216.53589	337.06035	96.01143463	-1.809186592	0.168440608	-10.740798	6.55E-27	6.46E-26
ENSG00000088305	DNMT3B	119.2906244	185.81207	52.76917425	-1.810274138	0.245999564	-7.358851	1.85E-13	1.08E-12
ENSG00000231185	SPRY4-AS1	13.42146424	20.862406	5.980522636	-1.813665932	0.684073261	-2.6512744	0.008019	0.018266
ENSG00000258441	LINC00641	89.26290247	139.05595	39.46985499	-1.818368875	0.253391819	-7.1761152	7.17E-13	4.06E-12
ENSG00000181773	GPR3	25.04630769	39.026688	11.06592697	-1.82100353	0.468320927	-3.8883668	0.000101	0.000303
ENSG00000241399	CD302	460.3116914	717.50466	203.1187236	-1.822533549	0.127463978	-14.29842	2.24E-46	3.51E-45
ENSG00000160325	CACFD1	196.5083172	306.54068	86.47595802	-1.822786655	0.173314358	-10.517228	7.20E-26	6.87E-25
ENSG00000137841	PLCB2	168.7674103	263.12287	74.41195502	-1.82330817	0.186391254	-9.7821552	1.34E-22	1.15E-21
ENSG00000236901	MIR600HG	29.10173775	45.42431	12.77916549	-1.823906866	0.437529756	-4.1686465	3.06E-05	9.78E-05

ENSG00000176208	ATAD5	113.7243433	177.43176	50.01692228	-1.827307336	0.229999096	-7.9448457	1.94E-15	1.25E-14
ENSG00000117643	MAN1C1	54.13828564	84.564223	23.71234822	-1.830544568	0.350321008	-5.2253348	1.74E-07	6.83E-07
ENSG00000129596	CDO1	149.0441743	232.74746	65.34088602	-1.831687723	0.195019984	-9.3923078	5.87E-21	4.75E-20
ENSG00000076770	MBNL3	80.29387861	125.48262	35.10513695	-1.834723465	0.282058301	-6.5047668	7.78E-11	3.92E-10
ENSG00000171435	KSR2	340.8814271	532.77888	148.9839737	-1.836304874	0.145733236	-12.600454	2.10E-36	2.64E-35
ENSG00000259479	SORD2P	82.2044988	128.51464	35.89436187	-1.836867398	0.274070355	-6.7021747	2.05E-11	1.08E-10
ENSG00000189337	KAZN	28.63321544	44.747736	12.51869465	-1.836889946	0.4509114	-4.073727	4.63E-05	0.000145
ENSG00000055163	CYFIP2	806.0303492	1260.0795	351.9812073	-1.840420797	0.095051892	-19.362274	1.61E-83	4.41E-82
ENSG00000149809	TM7SF2	183.8518231	287.64018	80.06346819	-1.84508069	0.186647376	-9.8853824	4.82E-23	4.19E-22
ENSG00000139910	NOVA1	103.4897653	162.02622	44.9533136	-1.846018585	0.247845393	-7.4482667	9.46E-14	5.56E-13
ENSG00000122378	PRXL2A	7818.89361	12237.687	3400.099938	-1.847352208	0.047708744	-38.72146	0	0
ENSG00000129422	MTUS1	9486.270655	14855.865	4116.676727	-1.851223136	0.047595814	-38.894663	0	0
ENSG00000167642	SPINT2	67.83377773	106.2676	29.39995962	-1.85385965	0.296938748	-6.2432953	4.29E-10	2.05E-09
ENSG00000164142	FAM160A1	9.269466843	14.565349	3.973584964	-1.862308119	0.804171059	-2.3158109	0.020569	0.043197
ENSG00000275450	AL845472.1	28.00767051	43.960445	12.05489651	-1.862881904	0.450912757	-4.1313577	3.61E-05	0.000114
ENSG00000164045	CDC25A	288.8041942	452.98045	124.6279373	-1.864101937	0.150190265	-12.411603	2.26E-35	2.78E-34
ENSG00000171496	OR1L8	13.95077918	21.834894	6.066664774	-1.864473805	0.745193464	-2.5019997	0.012349	0.027181
ENSG00000176435	CLEC14A	10049.4999	15771.087	4327.913115	-1.865562088	0.039209964	-47.578776	0	0
ENSG00000102174	PHEX	108.8748356	170.9151	46.8345691	-1.866241237	0.284377142	-6.5625571	5.29E-11	2.70E-10
ENSG00000266903	AC243964.2	12.19493192	19.091238	5.298626038	-1.867292018	0.747945929	-2.4965602	0.01254	0.027573
ENSG00000176438	SYNE3	559.6385452	879.34515	239.9319406	-1.871992277	0.10909823	-17.158778	5.40E-66	1.18E-64
ENSG00000130193	THEM6	166.3105904	261.41352	71.2076653	-1.872300931	0.20560534	-9.1062855	8.53E-20	6.62E-19
ENSG00000137878	GCOM1	82.01547738	128.90607	35.12488818	-1.872632008	0.265899365	-7.0426344	1.89E-12	1.05E-11
ENSG00000102010	BMX	3037.100384	4774.7282	1299.472579	-1.876582645	0.064044524	-29.301219	1.00E-188	6.16E-187
ENSG00000113368	LMNB1	975.9661379	1535.0937	416.8385816	-1.880612597	0.081509553	-23.072297	8.79E-118	3.41E-116
ENSG00000010438	PRSS3	633.6436674	996.89574	270.3915937	-1.883313031	0.120163789	-15.672883	2.32E-55	4.21E-54
ENSG00000165194	PCDH19	28.47535805	44.85205	12.09866627	-1.889347787	0.473541006	-3.9898293	6.61E-05	0.000203
ENSG00000169439	SDC2	73.85832291	116.24479	31.47185324	-1.890888529	0.297175393	-6.3628705	1.98E-10	9.69E-10
ENSG00000285108	AC103718.1	26.04786618	41.04393	11.05180285	-1.893120735	0.47686875	-3.9698989	7.19E-05	0.00022
ENSG00000183785	TUBA8	19.18280844	30.202393	8.163223966	-1.894375532	0.609323815	-3.10898	0.001877	0.004744
ENSG00000183615	FAM167B	327.5011002	516.48536	138.5168402	-1.901404768	0.147218761	-12.915506	3.68E-38	4.83E-37
ENSG00000128815	WDFY4	232.7119994	367.16412	98.25987983	-1.902061717	0.169528949	-11.21987	3.26E-29	3.43E-28
ENSG00000253616	AC107959.3	14.72575695	23.278309	6.173205186	-1.907273699	0.704586692	-2.7069397	0.006791	0.015636
ENSG00000001617	SEMA3F	8744.877461	13811.99	3677.764796	-1.909066844	0.063792222	-29.926326	8.95E-197	5.78E-195
ENSG00000186603	HPDL	28.53222622	44.959371	12.1050813	-1.90934498	0.527716708	-3.6181249	0.000297	0.000841
ENSG00000121957	GPSM2	267.3063198	422.26411	112.3485273	-1.91143803	0.150740836	-12.680293	7.60E-37	9.71E-36
ENSG00000071539	TRIP13	398.9026861	630.15965	167.6457218	-1.911856198	0.13260995	-14.41714	4.04E-47	6.42E-46
ENSG00000175567	UCP2	30.7569782	48.582295	12.93166096	-1.913236904	0.43658806	-4.3822474	1.17E-05	3.91E-05
ENSG00000146054	TRIM7	69.4931755	109.75932	29.2270283	-1.914582247	0.297438611	-6.4368988	1.22E-10	6.05E-10
ENSG00000170379	TCAF2	287.2162995	454.18494	120.2476602	-1.917914042	0.15390303	-12.461834	1.21E-35	1.49E-34
ENSG00000183287	CCBE1	512.0687145	810.38355	213.7538742	-1.922249524	0.114108176	-16.845853	1.13E-63	2.34E-62
ENSG00000213160	KLHL23	155.3556372	245.87011	64.8411681	-1.922977302	0.216283289	-8.8910119	6.06E-19	4.54E-18
ENSG00000141655	TNFRSF11A	282.8521062	447.74296	117.9612508	-1.926601493	0.157894838	-12.201802	3.04E-34	3.61E-33
ENSG00000138606	SHF	33.59390259	53.190927	13.99687824	-1.926876763	0.423148351	-4.5536672	5.27E-06	1.82E-05
ENSG00000178878	APOLD1	271.2508492	429.64496	112.8567422	-1.929037308	0.15201516	-12.689769	6.74E-37	8.62E-36
ENSG00000158555	GDPD5	2725.074899	4316.2515	1133.898302	-1.929500764	0.06767852	-28.509795	8.86E-179	5.23E-177
ENSG00000176533	GNG7	15.86004589	25.156504	6.563587926	-1.930854399	0.594934019	-3.2454933	0.001172	0.003065
ENSG00000149573	MPZL2	3186.333149	5049.8822	1322.784144	-1.932020004	0.059208349	-32.630871	1.50E-233	1.18E-231
ENSG00000151150	ANK3	878.3186533	1392.2302	364.4070725	-1.932899384	0.212192693	-9.1091703	8.30E-20	6.44E-19
ENSG00000128833	MYO5C	545.6828165	865.91972	225.445914	-1.94117917	0.120673973	-16.086146	3.19E-58	6.07E-57
ENSG00000183856	IQGAP3	238.0199933	377.59513	98.44485327	-1.941774282	0.166491714	-11.662888	1.97E-31	2.19E-30
ENSG00000203722	RAET1G	30.6465128	48.582295	12.71073016	-1.944908093	0.484944208	-4.0105811	6.06E-05	0.000187
ENSG00000244694	PTCHD4	225.6517064	358.53423	92.76918071	-1.947577727	0.172285709	-11.304349	1.25E-29	1.33E-28
ENSG00000241360	PDXP	10.81701758	17.161342	4.472693414	-1.949499277	0.8325509	-2.3415977	0.019201	0.040635
ENSG00000146122	DAAM2	11.63614727	18.505255	4.767039612	-1.954472982	0.69328138	-2.8191627	0.004815	0.011392
ENSG00000178150	ZNF114	23.9951872	38.228302	9.76207209	-1.957018732	0.508633222	-3.847603	0.000119	0.000355
ENSG00000128645	HOXD1	19.94595762	31.759772	8.132143374	-1.968231628	0.54618941	-3.6035697	0.000314	0.000886
ENSG00000168672	FAM84B	1638.378159	2611.1462	665.6100907	-1.97120787	0.102221627	-19.283667	7.37E-83	2.01E-81
ENSG00000188312	CENPP	53.03621304	84.600636	21.47179058	-1.971454531	0.354750875	-5.5572929	2.74E-08	1.15E-07
ENSG00000171502	COL24A1	35.48081379	56.512243	14.44938459	-1.97240797	0.416308928	-4.7378469	2.16E-06	7.74E-06
ENSG00000151725	CENPU	148.8851544	237.45772	60.31258765	-1.973101986	0.219733688	-8.9795152	2.72E-19	2.06E-18
ENSG00000170522	ELOVL6	412.5659257	658.58949	166.5423619	-1.985096089	0.128657888	-15.429261	1.04E-53	1.84E-52
ENSG00000133119	RFC3	169.4082438	270.53759	68.27889934	-1.985894764	0.191570216	-10.366407	3.53E-25	3.30E-24
ENSG00000137310	TCF19	405.5607331	647.68879	163.4326737	-1.986150456	0.131088736	-15.151191	7.44E-52	1.28E-50
ENSG00000171346	KRT15	83.0621304	132.68297	33.4412953	-1.989407061	0.28888905	-6.8864052	5.72E-12	3.10E-11
ENSG00000074370	ATP2A3	25.73078456	41.080371	10.38119804	-1.991063233	0.482846396	-4.1235955	3.73E-05	0.000118
ENSG00000180592	SKIDA1	12.75315094	20.402544	5.103758188	-1.99131164	0.690837182	-2.8824616	0.003946	0.009489
ENSG00000231789	PIK3CD-AS2	8.279308647	13.237283	3.321334001	-1.9950929	0.828498433	-2.4080829	0.016037	0.034497
ENSG00000057704	TMCC3	1779.843388	2845.8161	713.8706705	-1.995397681	0.074184591	-26.897738	2.33E-159	1.19E-157
ENSG00000100479	POLE2	55.67318354	89.119265	22.22710258	-1.9972084	0.364616843	-5.4775539	4.31E-08	1.79E-07
ENSG00000090447	TFAP4	81.37508507	130.08742	32.66275269	-1.99984649	0.290551912	-6.8829232	5.86E-12	3.17E-11
ENSG00000166250	CLMP	179.6834099	287.58943	71.77739438	-1.999868787	0.184905588	-10.815621	2.90E-27	2.90E-26
ENSG00000105996	HOXA2	34.06763698	54.50899	13.6262843	-2.000115181	0.409940419	-4.8790387	1.07E-06	3.93E-06
ENSG00000204381	LAYN	43.85440775	70.248753	17.46006296	-2.000349836	0.384498739	-5.2024874	1.97E-07	7.70E-07
ENSG00000058404	CAMK2B	22.75366042	36.458226	9.0490949	-2.000893174	0.534257774	-3.7451831	0.00018	0.000523
ENSG00000225968	ELFN1	52.65962639	84.352882	20.96637041	-2.008802433	0.34622589	-5.8019995	6.55E-09	2.88E-08
ENSG00000196812	ZSCAN16	114.025638	182.63621	45.41506731	-2.009322451	0.243246127	-8.2604499	1.45E-16	9.84E-16
ENSG00000088756	ARHGAP28	17.02114189	27.193456	6.848827637	-2.009835668	0.705657944	-2.8481727	0.004397	0.010468
ENSG00000204131	NHSL2	2255.114774	3613.8017	896.4278677	-2.010513497	0.061735313	-32.566669	1.22E-232	9.48E-231
ENSG00000133101	CCNA1	1134.788837	1818.3243	451.2533625	-2.010546396	0.086893199	-23.138133	1.91E-118	7.51E-117
ENSG00000137804	NUSAP1	26.77063029	42.950424	10.59083705	-2.013035682	0.47989889	-4.1947079	2.73E-05	8.77E-05
ENSG00000203943	SAMD13	15.78995881	25.299384	6.280533514	-2.014258849	0.603815977	-3.335882	0.00085	0.002265
ENSG00000125378	BMP4	4143.716402	6643.9463	1643.486481	-2.014682815	0.062913477	-32.023072	5.21E-225	3.87E-223
ENSG00000272711	AC019069.1	51.48918787	82.653715	20.32466089	-2.016623133	0.368802413	-5.4680313	4.55E-08	1.88E-07
ENSG00000204767	INSYN2B	886.8338232	1422.124	351.543613	-2.017318134	0.090985574	-22.171846	6.42E-109	2.29E-107

ENSG00000171522	PTGER4	539.8687039	865.92762	213.8097894	-2.020788198	0.119945179	-16.847598	1.09E-63	2.28E-62
ENSG00000272674	PCDHB16	53.52641626	85.777204	21.27562865	-2.021388017	0.364604592	-5.5440553	2.96E-08	1.24E-07
ENSG00000253552	HOXA-AS2	60.38210662	96.945934	23.81827917	-2.023383479	0.34383929	-5.8846779	3.99E-09	1.78E-08
ENSG00000142910	TINAGL1	7467.390302	11989.207	2945.573562	-2.025340303	0.059496735	-34.041201	5.48E-254	5.09E-252
ENSG00000171130	ATP6V0E2	494.5265188	793.87173	195.1813072	-2.025445225	0.114059296	-17.757827	1.50E-70	3.47E-69
ENSG00000114654	EFCC1	8.331456406	13.395243	3.267669837	-2.026474826	0.848511994	-2.3882689	0.016928	0.03624
ENSG00000115602	IL1RL1	11194.452	17978.25	4410.653966	-2.027202227	0.148572111	-13.644568	2.17E-42	3.16E-41
ENSG00000144554	FANCD2	190.8940771	306.57145	75.21670146	-2.029146965	0.176695079	-11.483891	1.59E-30	1.73E-29
ENSG00000138346	DNA2	82.37600301	132.3152	32.43680425	-2.02975775	0.265090618	-7.6568449	1.91E-14	1.17E-13
ENSG00000254221	PCDHGB1	13.35277353	21.435169	5.270377786	-2.039433675	0.742036958	-2.748426	0.005988	0.013929
ENSG00000102271	KLHL4	1168.894741	1880.317	457.4724476	-2.039592267	0.085232362	-23.929787	1.50E-126	6.31E-125
ENSG00000164849	GPR146	251.3674718	404.53378	98.20116045	-2.039752881	0.171714065	-11.878776	1.53E-32	1.75E-31
ENSG00000189129	PLAC9	69.20662063	111.1938	27.21944359	-2.039841948	0.315219806	-6.4711732	9.72E-11	4.86E-10
ENSG00000177685	CRACR2B	151.2450052	243.36753	59.12247611	-2.041000926	0.202335435	-10.087214	6.29E-24	5.64E-23
ENSG00000137880	GCHFR	57.23428026	92.256979	22.21158107	-2.046772848	0.3273805	-6.2519693	4.05E-10	1.94E-09
ENSG00000135547	HEY2	164.7130064	265.43619	63.98981957	-2.053142519	0.210986381	-9.7311614	2.22E-22	1.89E-21
ENSG00000226332	AL354836.1	11.30972607	18.247025	4.372427149	-2.053457051	0.723355414	-2.8387941	0.004528	0.010761
ENSG00000198435	NRARP	1014.297853	1634.8967	393.6989638	-2.056154634	0.096120877	-21.391343	1.61E-101	5.33E-100
ENSG00000115425	PECR	85.27911916	137.43094	33.12730117	-2.057147147	0.589064989	-3.4922244	0.000479	0.00132
ENSG00000177721	ANXA2R	25.70953846	41.476261	9.942815813	-2.059717897	0.487040383	-4.2290495	2.35E-05	7.57E-05
ENSG00000138134	STAMBPL1	44.75627538	72.240941	17.27161014	-2.063793392	0.37364739	-5.5233716	3.33E-08	1.39E-07
ENSG00000164611	PTTG1	370.4510802	597.88298	143.0191792	-2.066889837	0.142395792	-14.515105	9.72E-48	1.57E-46
ENSG00000283632	EXOC3L2	26.06625965	42.020252	10.11226775	-2.067984265	0.504666038	-4.0977282	4.17E-05	0.000131
ENSG00000154146	NRGN	795.1905823	1284.0825	306.2986447	-2.068308215	0.111162472	-18.606173	2.86E-77	7.23E-76
ENSG00000188738	FSIP2	7.573986939	12.25373	2.894243563	-2.068728299	0.879606706	-2.3518787	0.018679	0.039636
ENSG00000134317	GRHL1	16.22118999	26.233864	6.208515501	-2.071187868	0.614159666	-3.3723932	0.000745	0.002001
ENSG00000071282	LMCD1	126.3273942	204.10336	48.55142787	-2.072181937	0.214654489	-9.6535691	4.75E-22	3.99E-21
ENSG00000166831	RBPMS2	12.66589863	20.441908	4.889889449	-2.078416476	0.705929437	-2.944227	0.003238	0.007876
ENSG00000171320	ESCO2	98.04650547	158.53832	37.55468656	-2.078997759	0.242930759	-8.5579849	1.15E-17	8.14E-17
ENSG00000184226	PCDH9	160.291917	259.28432	61.29951276	-2.079457504	0.195162696	-10.654995	1.65E-26	1.62E-25
ENSG00000047617	ANO2	72.19223873	116.81071	27.57376567	-2.080858836	0.326113124	-6.3807884	1.76E-10	8.66E-10
ENSG00000237529	AL137847.2	13.71997301	22.13709	5.302855761	-2.080966908	0.716022204	-2.9062882	0.003657	0.00884
ENSG00000110400	NECTIN1	1306.890033	2114.5276	499.2525061	-2.084663899	0.103416791	-20.157886	2.29E-90	6.82E-89
ENSG00000142731	PLK4	160.3505797	259.74474	60.95641674	-2.086368811	0.219719583	-9.4955979	2.19E-21	1.80E-20
ENSG00000171227	TMEM37	188.235554	304.98318	71.48792495	-2.087412427	0.1997731	-10.448916	1.48E-25	1.40E-24
ENSG00000065675	PRKCQ	7.774949508	12.583637	2.966261576	-2.090005268	0.871027226	-2.3994718	0.016419	0.035246
ENSG00000116667	C1orf21	853.171809	1382.1769	324.1667315	-2.090935095	0.091867686	-22.760289	1.13E-114	4.29E-113
ENSG00000196584	XRCC2	65.5943867	106.28976	24.89901795	-2.092492307	0.315970376	-6.6224319	3.53E-11	1.82E-10
ENSG00000101695	RNF125	7.789098945	12.611936	2.966261576	-2.092630171	0.875041731	-2.3914633	0.016781	0.035967
ENSG00000135476	ESPL1	239.592002	388.02278	91.1612261	-2.092935413	0.167009582	-12.531828	5.00E-36	6.25E-35
ENSG00000137807	KIF23	451.5688488	731.87002	171.2676761	-2.095354501	0.120062397	-17.452213	3.31E-68	7.44E-67
ENSG00000136305	CIDEB	37.54646838	60.951375	14.1415613	-2.097000218	0.412653433	-5.0817467	3.74E-07	1.44E-06
ENSG00000162337	LRP5	4395.383403	7126.6046	1664.162182	-2.098429066	0.049493846	-42.397778	0	0
ENSG00000164109	MAD2L1	291.4864967	472.76411	110.208883	-2.100570886	0.154752167	-13.573774	5.73E-42	8.25E-41
ENSG00000134222	PSRC1	130.9358307	212.22742	49.64424635	-2.10179626	0.222825707	-9.4324676	4.01E-21	3.26E-20
ENSG00000167513	CDT1	146.5777942	237.83015	55.32544273	-2.106755635	0.250808028	-8.3998732	4.47E-17	3.10E-16
ENSG00000138160	KIF11	503.2222457	817.06163	189.38286	-2.108178179	0.119008368	-17.714537	3.24E-70	7.45E-69
ENSG00000231607	DLEU2	54.11784154	87.82413	20.41155337	-2.111815714	0.365116886	-5.7839443	7.30E-09	3.19E-08
ENSG00000123338	NCKAP1L	8.746062441	14.228685	3.263440114	-2.114964741	0.82942013	-2.5499318	0.010774	0.023953
ENSG00000091409	ITGA6	6790.221015	11037.055	2543.386781	-2.117451642	0.06028495	-35.12405	2.90E-270	2.91E-268
ENSG00000215386	MIR99AHG	64.94247023	105.56193	24.32301473	-2.118738688	0.302533909	-7.0033098	2.50E-12	1.38E-11
ENSG00000178695	KCTD12	15344.06824	24955.882	5732.254263	-2.121972702	0.043601524	-48.667397	0	0
ENSG00000127423	AUNIP	27.93770924	45.350184	10.52523406	-2.122356873	0.499016646	-4.2530783	2.11E-05	6.84E-05
ENSG00000197245	FAM110D	109.0693082	177.55913	40.57948907	-2.124697428	0.244751909	-8.681025	3.92E-18	2.83E-17
ENSG00000138182	KIF20B	551.334747	897.36617	205.3033285	-2.127247344	0.110579349	-19.237293	1.80E-82	4.89E-81
ENSG00000125965	GDF5	14.97142855	24.347053	5.595804576	-2.130254787	0.645967454	-3.2977742	0.000975	0.002575
ENSG00000184785	SMIM10	62.81823863	102.41474	23.22173679	-2.132036341	0.336025855	-6.344858	2.23E-10	1.08E-09
ENSG00000263155	MYZAP	66.68359547	108.7647	24.60248646	-2.135479041	0.350355908	-6.0951706	1.09E-09	5.08E-09
ENSG00000100473	COCH	17.61713469	28.756824	6.477445787	-2.136837325	0.633310392	-3.3740759	0.000741	0.001989
ENSG00000242689	CNTF	7.859138933	12.831096	2.8871815	-2.13735303	0.882640664	-2.4215438	0.015455	0.033329
ENSG00000164512	ANKRD55	203.5937053	331.89804	75.28936651	-2.139073714	0.17406083	-12.289231	1.03E-34	1.25E-33
ENSG00000161800	RACGAP1	465.8397178	759.39598	172.283459	-2.139996498	0.128354212	-16.672585	2.07E-62	4.23E-61
ENSG00000267279	AC090409.1	22.92280112	37.3372	8.508401988	-2.140013243	0.513840718	-4.1647405	3.12E-05	9.94E-05
ENSG00000135914	HTR2B	338.7361969	552.15335	125.3190437	-2.142046046	0.163484694	-13.102426	3.19E-39	4.30E-38
ENSG00000246922	UBAP1L	24.96844381	40.747986	9.188901203	-2.145990316	0.54092338	-3.9672723	7.27E-05	0.000222
ENSG00000165091	TMC1	8.104274984	13.191457	3.0170934	-2.146471235	0.872660956	-2.4596852	0.013906	0.030297
ENSG00000100234	TIMP3	16.91770819	27.655149	6.180267249	-2.152242503	0.642031421	-3.3522386	0.000802	0.002141
ENSG00000120328	PCDHB12	21.19785398	34.593221	7.802486861	-2.154199272	0.55583257	-3.8756262	0.000106	0.000318
ENSG00000102098	SCML2	18.13225885	29.596434	6.668083913	-2.157454881	0.594370706	-3.6298136	0.000284	0.000806
ENSG00000106351	AGFG2	868.3415161	1418.8483	317.8347191	-2.157817443	0.089969854	-23.983783	4.11E-127	1.73E-125
ENSG00000187017	ESPN	20.14182641	32.898362	7.385290826	-2.160233649	0.545292729	-3.9616036	7.44E-05	0.000227
ENSG00000100399	CHADL	21.46065978	35.014337	7.906982849	-2.163926136	0.612790548	-3.5312655	0.000414	0.001149
ENSG00000007312	CD79B	8.056974192	13.191457	2.922491815	-2.168437044	0.939598013	-2.3078349	0.021008	0.044022
ENSG00000147036	LANCL3	41.59913653	68.050035	15.14823765	-2.169877016	0.387120826	-5.6051674	2.08E-08	8.81E-08
ENSG00000198643	FAM3D	8.149635094	13.314655	2.984615425	-2.171091258	0.854333683	-2.5412685	0.011045	0.024523
ENSG00000101412	E2F1	143.4265854	234.75729	52.09587797	-2.175806517	0.219703124	-9.9033936	4.02E-23	3.51E-22
ENSG00000170498	KISS1	22.55022639	36.870875	8.229577299	-2.177121674	0.537558878	-4.0500153	5.12E-05	0.000159
ENSG00000112562	SMOC2	605.8420261	992.45583	219.2282263	-2.177725858	0.104440669	-20.851321	1.48E-96	4.72E-95
ENSG00000166265	CYYR1	797.1832139	1306.3135	288.0529395	-2.182435183	0.095166984	-22.932693	2.19E-116	8.43E-115
ENSG00000011332	DPF1	28.47737318	46.71192	10.24282669	-2.184228662	0.476554203	-4.5833793	4.58E-06	1.59E-05
ENSG00000167861	HID1	641.2385909	1051.0612	231.4160079	-2.185011938	0.108280807	-20.179125	1.49E-90	4.45E-89
ENSG00000087842	PIR	2188.632866	3589.6641	787.6016474	-2.186864488	0.079275101	-27.585767	1.65E-167	9.03E-166
ENSG00000126460	PRRG2	50.14571998	82.370374	17.92106633	-2.195758554	0.380896639	-5.7647097	8.18E-09	3.56E-08
ENSG00000225511	LINC00475	10.29475141	16.906034	3.683468489	-2.196574596	0.772518259	-2.8433951	0.004464	0.010619
ENSG00000159860	TCAF2P1	26.94133632	44.172079	9.710593225	-2.198598892	0.523916215	-4.1964704	2.71E-05	8.70E-05

ENSG00000172197	MBOAT1	22.62069541	37.148787	8.092603336	-2.199868966	0.530859286	-4.1439776	3.41E-05	0.000108
ENSG00000139438	FAM222A	20.68992976	33.983277	7.396582612	-2.206647763	0.552268331	-3.995608	6.45E-05	0.000198
ENSG00000100065	CARD10	1366.574401	2246.5657	486.5830786	-2.207523182	0.073065618	-30.212886	1.60E-200	1.06E-198
ENSG00000013810	TACC3	906.6765847	1491.1595	322.1936316	-2.209194847	0.090244338	-24.480149	2.40E-132	1.06E-130
ENSG00000181544	FANCB	30.09646328	49.463718	10.7292084	-2.209819776	0.459154027	-4.8128071	1.49E-06	5.42E-06
ENSG00000071575	TRIB2	391.2082452	643.76134	138.6551458	-2.214994322	0.128096862	-17.291558	5.45E-67	1.20E-65
ENSG00000121621	KIF18A	145.5785523	239.58432	51.57278856	-2.217440109	0.241544798	-9.1802437	4.30E-20	3.38E-19
ENSG00000127564	PKMYT1	122.8290069	202.33036	43.32765218	-2.223129548	0.23029996	-9.6531912	4.76E-22	4.00E-21
ENSG00000184005	ST6GALNAC3	1100.925974	1814.8088	387.043184	-2.228488857	0.088888601	-25.070581	1.04E-138	4.68E-137
ENSG00000134057	CCNB1	994.1873979	1639.4749	348.8998802	-2.231708693	0.08731512	-25.559247	4.33E-144	2.05E-142
ENSG00000100292	HMOX1	5056.525502	8340.7395	1772.311541	-2.234442158	0.072540837	-30.802542	2.42E-208	1.69E-206
ENSG00000165092	ALDH1A1	5022.065079	8289.5014	1754.628719	-2.24005514	0.163471663	-13.703018	9.74E-43	1.43E-41
ENSG00000276043	UHRF1	548.6048054	905.59892	191.6106909	-2.240114271	0.119877905	-18.686632	6.36E-78	1.62E-76
ENSG00000137463	MGARP	477.5619388	788.22648	166.8973967	-2.24141951	0.13887451	-16.139891	1.34E-58	2.57E-57
ENSG00000146670	CDCA5	331.2744426	546.78159	115.7672952	-2.241966856	0.14040325	-15.968055	2.13E-57	3.99E-56
ENSG00000173597	SULT1B1	2211.36267	3651.0902	771.6351684	-2.24273526	0.066133436	-33.912275	4.39E-252	4.03E-250
ENSG00000015133	CCDC88C	733.5855156	1211.5147	255.6563223	-2.24444145	0.100875938	-22.249523	1.14E-109	4.12E-108
ENSG00000181754	AMIGO1	41.44993079	68.47729	14.42257129	-2.251064593	0.390721614	-5.7613004	8.35E-09	3.63E-08
ENSG00000249279	LINC02057	71.08244032	117.55315	24.61173382	-2.255578254	0.297136516	-7.5910504	3.17E-14	1.92E-13
ENSG00000072952	MRVI1	76.11326292	125.73702	26.48950993	-2.256606783	0.325181625	-6.9395274	3.93E-12	2.15E-11
ENSG00000115457	IGFBP2	328.9822592	544.13641	113.8281083	-2.258437851	0.142132429	-15.889673	7.47E-57	1.39E-55
ENSG00000138336	TET1	16.14485004	26.700958	5.588742513	-2.262254172	0.650768783	-3.4762795	0.000508	0.001397
ENSG00000137507	LRRC32	6494.54944	10755.435	2233.664015	-2.268165524	0.05484059	-41.359248	0	0
ENSG00000127325	BEST3	8.578480531	14.237302	2.919659475	-2.281073858	0.835323127	-2.7307682	0.006319	0.014653
ENSG00000226496	LINC00323	29.13601285	48.340502	9.931524027	-2.281781573	0.47828855	-4.7707217	1.84E-06	6.63E-06
ENSG00000130590	SAMD10	76.94874887	127.82593	26.07156356	-2.292141387	0.287073128	-7.9845209	1.41E-15	9.12E-15
ENSG00000272081	AC008972.2	6.465077321	10.76018	2.169974587	-2.29581277	0.973560141	-2.3581622	0.018366	0.039016
ENSG00000093009	CDC45	159.8814205	265.63963	54.12321391	-2.296004022	0.198751153	-11.552154	7.20E-31	7.87E-30
ENSG00000176890	TYMS	1384.069732	2301.7137	466.4257877	-2.304752437	0.08569133	-26.895982	2.45E-159	1.25E-157
ENSG00000213185	FAM24B	78.39840614	130.42101	26.37580416	-2.304838129	0.315725808	-7.3001258	2.87E-13	1.66E-12
ENSG00000147408	CSGALNACT1	1011.401012	1683.1973	339.6047109	-2.309976921	0.218202307	-10.5864	3.45E-26	3.33E-25
ENSG00000277013	AC008556.1	22.69788665	37.864402	7.531371276	-2.31093996	0.580456384	-3.9812465	6.86E-05	0.00021
ENSG00000117707	PROX1	91.85390858	152.82046	30.88735299	-2.313931059	0.306137259	-7.5584758	4.08E-14	2.45E-13
ENSG00000102452	NALCN	280.6965487	467.2041	94.18900181	-2.315118327	0.189114508	-12.241886	1.86E-34	2.22E-33
ENSG00000005108	THSD7A	1937.322628	3227.1159	647.5293166	-2.316428786	0.089579656	-25.858871	1.93E-147	9.34E-146
ENSG00000221866	PLXNA4	1835.785465	3058.8762	612.6946857	-2.319961746	0.070176292	-33.059053	1.15E-239	9.67E-238
ENSG00000105011	ASF1B	204.9470966	342.08124	67.81295348	-2.330165073	0.212778043	-10.951154	6.56E-28	6.68E-27
ENSG00000124097	HMGB1P1	6.599577806	11.029181	2.169974587	-2.332261005	0.978348255	-2.3838761	0.017131	0.036646
ENSG00000151632	AKR1C2	44.4698782	74.236963	14.70279336	-2.336825258	0.379858634	-6.1518287	7.66E-10	3.60E-09
ENSG00000142945	KIF2C	314.6297234	525.29927	103.9601804	-2.339414629	0.154870486	-15.10562	1.49E-51	2.55E-50
ENSG00000204314	PRRT1	13.3787681	22.342737	4.414799527	-2.33988045	0.713771309	-3.2781935	0.001045	0.002746
ENSG00000126803	HSPA2	74.90973092	125.16396	24.65550358	-2.344647713	0.287209765	-8.1635376	3.25E-16	2.17E-15
ENSG00000123080	CDKN2C	114.7209377	191.847	37.59487364	-2.346311714	0.247562596	-9.4776503	2.60E-21	2.13E-20
ENSG00000134690	CDCA8	342.1351682	572.12234	112.1479948	-2.35137422	0.149503563	-15.727881	9.74E-56	1.79E-54
ENSG00000085999	RAD54L	73.62365025	123.0252	24.22210142	-2.354118635	0.335082771	-7.025484	2.13E-12	1.18E-11
ENSG00000152128	TMEM163	79.57741061	133.24142	25.9134034	-2.357600386	0.293026217	-8.0456978	8.58E-16	5.60E-15
ENSG00000128011	LRFN1	13.42307641	22.49208	4.354073301	-2.360002485	0.689802057	-3.4212749	0.000623	0.001691
ENSG00000156103	MMP16	1209.204265	2024.5052	393.9033034	-2.361143206	0.084363417	-27.987761	2.29E-172	1.31E-170
ENSG00000186767	SPIN4	109.6215344	183.46091	35.78215678	-2.36137997	0.245713971	-9.6102796	7.24E-22	6.03E-21
ENSG00000113212	PCDHB7	30.46681989	51.05155	9.882089586	-2.363877562	0.470159914	-5.027816	4.96E-07	1.88E-06
ENSG00000212766	EWSAT1	8.881289683	14.914938	2.847641462	-2.364830864	0.906550415	-2.6086038	0.009091	0.02051
ENSG00000213203	GIMAP1	589.3571219	987.21296	191.5012805	-2.365622173	0.115423212	-20.495203	2.38E-93	7.32E-92
ENSG00000174004	NRROS	232.4595271	389.49558	75.42347056	-2.369651069	0.18514828	-12.798666	1.67E-37	2.16E-36
ENSG00000167371	PRRT2	7.823077305	13.139461	2.506693163	-2.369731246	0.957265759	-2.4755207	0.013304	0.029123
ENSG00000257167	TMPO-AS1	24.93142515	41.814017	8.048833575	-2.372674035	0.507569279	-4.6745816	2.95E-06	1.04E-05
ENSG00000088325	TPX2	1423.546441	2386.3583	460.7345654	-2.373097622	0.082771393	-28.670505	8.90E-181	5.31E-179
ENSG00000167874	TMEM88	40.95956975	68.636636	13.28250366	-2.373245883	0.390501236	-6.077435	1.22E-09	5.66E-09
ENSG00000167767	KRT80	419.216092	702.85346	135.5787235	-2.37413893	0.133304715	-17.809865	5.93E-71	1.38E-69
ENSG00000232453	AC105277.1	13.57154841	22.767837	4.375259489	-2.374666781	0.695187359	-3.4158659	0.000636	0.001722
ENSG00000163884	KLF15	30.70537131	51.482051	9.928691687	-2.375041043	0.448624066	-5.2940563	1.20E-07	4.77E-07
ENSG00000178726	THBD	1889.328254	3168.866	609.7904821	-2.376942838	0.069258007	-34.320116	3.93E-258	3.69E-256
ENSG00000182853	VMO1	9.065648886	15.2187	2.912597412	-2.377194622	0.836101218	-2.84319	0.004466	0.010625
ENSG00000147697	GSDMC	96.25836625	161.64077	30.87595791	-2.379101984	0.27523716	-8.6438255	5.44E-18	3.89E-17
ENSG00000125910	S1PR4	7.94997893	13.353725	2.546233201	-2.383151026	0.908459332	-2.6232886	0.008709	0.019699
ENSG00000101003	GINS1	208.8107323	350.64707	66.97439742	-2.389184926	0.180572707	-13.231152	5.80E-40	7.95E-39
ENSG00000131620	ANO1	11.47479718	19.273188	3.676406426	-2.39158221	0.730876753	-3.27221	0.001067	0.002804
ENSG00000109674	NEIL3	35.64230805	59.867846	11.41676968	-2.392167209	0.427972218	-5.5895385	2.28E-08	9.62E-08
ENSG00000007866	TEAD3	17.20327747	28.922308	5.484246525	-2.394033325	0.597393027	-4.0074678	6.14E-05	0.000189
ENSG00000212864	RNF208	55.72276241	93.537832	17.90769255	-2.395796841	0.352895102	-6.7889773	1.13E-11	6.00E-11
ENSG00000176124	DLEU1	16.38098488	27.502884	5.259086	-2.403493361	0.632566746	-3.7995885	0.000145	0.000427
ENSG00000141753	IGFBP4	22081.33182	37147.925	7014.738936	-2.404871469	0.036705588	-65.517858	0	0
ENSG00000169679	BUB1	441.1150805	742.20416	140.0260011	-2.405633209	0.121658235	-19.773698	5.02E-87	1.43E-85
ENSG00000110799	VWF	74434.48542	125277.84	23591.13503	-2.408811505	0.140494953	-17.145182	6.83E-66	1.48E-64
ENSG00000104368	PLAT	9739.136768	16392.304	3085.970006	-2.409401031	0.064430812	-37.395168	4.68E-306	5.64E-304
ENSG00000115290	GRB14	561.1636527	944.59348	177.7338301	-2.409534792	0.117358046	-20.531483	1.13E-93	3.48E-92
ENSG00000233170	AC138356.2	5.803604059	9.7852439	1.821964225	-2.418817839	1.028024373	-2.3528799	0.018629	0.039539
ENSG00000107821	KAZALD1	10.64048605	17.901744	3.379227888	-2.422632125	0.775462262	-3.1241135	0.001783	0.004519
ENSG00000106714	CNTNAP3	1206.566155	2034.0311	379.1012477	-2.424696079	0.085665515	-28.304226	3.07E-176	1.78E-174
ENSG00000136982	DSCC1	51.83507066	87.380439	16.28970266	-2.429429168	0.370386741	-6.5591688	5.41E-11	2.75E-10
ENSG00000094804	CDC6	264.0224574	445.42317	82.62174352	-2.433319706	0.159472068	-15.258595	1.44E-52	2.52E-51
ENSG00000236824	BCYRN1	191.5121425	323.40127	59.62301952	-2.440779284	0.192800109	-12.659636	9.90E-37	1.26E-35
ENSG00000185339	TCN2	6857.315418	11582.194	2132.43685	-2.44170551	0.051158023	-47.728692	0	0
ENSG00000143476	DTL	165.4479088	279.45864	51.43717441	-2.441809539	0.214182235	-11.400617	4.15E-30	4.47E-29
ENSG00000281344	HELLPAR	7.192724973	12.143457	2.2419926	-2.4461797	0.970626821	-2.5202062	0.011729	0.025888
ENSG00000115884	SDC1	170.81952	288.5633	53.07574102	-2.449105054	0.230654559	-10.618065	2.46E-26	2.39E-25

ENSG00000174059	CD34	7822.177467	13225.166	2419.188524	-2.450737633	0.118777273	-20.633052	1.39E-94	4.31E-93
ENSG00000154920	EME1	23.72261816	40.129131	7.316105153	-2.451943458	0.536395059	-4.5711522	4.85E-06	1.68E-05
ENSG00000075702	WDR62	121.1981709	205.00544	37.39089931	-2.452638348	0.241815719	-10.142593	3.57E-24	3.22E-23
ENSG00000143248	RGS5	3901.31498	6598.7053	1203.924681	-2.454710381	0.065657274	-37.386724	6.42E-306	7.68E-304
ENSG00000186205	1-Mar	27.53726495	46.559066	8.515464051	-2.45645764	0.485611595	-5.0584823	4.23E-07	1.62E-06
ENSG00000185352	HS6ST3	47.85347148	80.998485	14.70845804	-2.462195708	0.395404531	-6.2270296	4.75E-10	2.27E-09
ENSG00000166924	NYAP1	78.17038093	132.32199	24.01877412	-2.4666618	0.289852583	-8.5100563	1.74E-17	1.22E-16
ENSG00000181350	LRRC75A	49.56256929	83.904883	15.22025566	-2.470885381	0.376045733	-6.5707045	5.01E-11	2.56E-10
ENSG00000112182	BACH2	15.69284818	26.597471	4.788225801	-2.474567887	0.648228653	-3.8174306	0.000135	0.000398
ENSG00000260260	SNHG19	111.443838	189.08783	33.7998471	-2.474746962	0.259205549	-9.5474305	1.33E-21	1.10E-20
ENSG00000111247	RAD51AP1	104.4375202	177.17032	31.70472287	-2.476024964	0.263888309	-9.3828521	6.42E-21	5.18E-20
ENSG00000111057	KRT18	1902.708558	3225.8345	579.5825784	-2.477967833	0.070222654	-35.287299	9.20E-273	9.44E-271
ENSG00000165899	OTOGL	74.66935973	126.67323	22.6654848	-2.480290831	0.339715969	-7.3010722	2.85E-13	1.65E-12
ENSG00000145386	CCNA2	475.4351035	806.05936	144.8108508	-2.481417302	0.147215171	-16.855717	9.52E-64	1.99E-62
ENSG00000107807	TLX1	148.7122865	252.33852	45.08605784	-2.482263889	0.213350845	-11.634657	2.75E-31	3.03E-30
ENSG00000174669	SLC29A2	25.7457084	43.808947	7.682469364	-2.507271054	0.497719007	-5.0375232	4.72E-07	1.79E-06
ENSG00000168077	SCARA3	2046.770673	3482.6026	610.9387096	-2.513019389	0.072688686	-34.57236	6.58E-262	6.30E-260
ENSG00000173599	PC	317.8441723	541.00038	94.68796939	-2.515902375	0.150809474	-16.682655	1.75E-62	3.58E-61
ENSG00000021300	PLEKHB1	17.49649997	29.733914	5.259086	-2.517726079	0.621409646	-4.0516366	5.09E-05	0.000158
ENSG00000040608	RTN4R	10.06035859	17.121978	2.998739551	-2.53091255	0.844549321	-2.9967611	0.002729	0.006727
ENSG00000025434	NR1H3	67.36001965	114.84853	19.8715075	-2.530928242	0.317230251	-7.9782058	1.48E-15	9.60E-15
ENSG00000149922	TBX6	6.285621975	10.716802	1.8544422	-2.538520815	1.028918043	-2.4671749	0.013618	0.02974
ENSG00000283378	CNTNAP3C	113.8083916	194.28705	33.32973725	-2.539814729	0.256636551	-9.8965433	4.31E-23	3.76E-22
ENSG00000169607	CKAP2L	210.2088283	358.96496	61.45269285	-2.543234501	0.19756834	-12.872682	6.41E-38	8.36E-37
ENSG00000254810	AP001189.3	38.99675744	66.602161	11.39135376	-2.548685709	0.42762054	-5.9601574	2.52E-09	1.14E-08
ENSG00000111665	CDCA3	126.7401045	216.50859	36.97162127	-2.554860224	0.232049221	-11.009993	3.42E-28	3.50E-27
ENSG00000237649	KIFC1	305.6463948	522.98358	88.30921404	-2.563319183	0.153167135	-16.735439	7.23E-63	1.49E-61
ENSG00000163701	IL17RE	29.24771529	50.084463	8.410968063	-2.569275665	0.470186762	-5.4643726	4.65E-08	1.92E-07
ENSG00000255135	AP002360.1	23.15346773	39.573896	6.733039863	-2.569439485	0.560628122	-4.5831441	4.58E-06	1.59E-05
ENSG00000133863	TEX15	42.2643314	72.274669	12.25399409	-2.569545438	0.438986253	-5.853362	4.82E-09	2.14E-08
ENSG00000161888	SPC24	160.0530667	273.92303	46.18310605	-2.572678467	0.209384289	-12.286874	1.07E-34	1.28E-33
ENSG00000186377	CYP4X1	67.89489757	116.36796	19.42183349	-2.580427992	0.311167962	-8.2927175	1.11E-16	7.55E-16
ENSG00000167971	CASKIN1	11.89317107	20.330867	3.455475623	-2.588816568	0.820474535	-3.1552674	0.001604	0.004094
ENSG00000163072	NOSTRIN	279.1604132	478.83336	79.48746496	-2.591435477	0.156385997	-16.570764	1.13E-61	2.29E-60
ENSG00000021645	NRXN3	108.3639293	186.07706	30.65079738	-2.595839502	0.255742226	-10.150219	3.31E-24	2.98E-23
ENSG00000246228	CASC8	25.88484386	44.426769	7.342918448	-2.597320152	0.536601673	-4.8403132	1.30E-06	4.75E-06
ENSG00000160145	KALRN	718.4335138	1234.3838	202.4831849	-2.605749476	0.102158122	-25.507022	1.65E-143	7.76E-142
ENSG00000106484	MEST	19.63104626	33.71289	5.549202475	-2.608210489	0.581276146	-4.487042	7.22E-06	2.46E-05
ENSG00000163710	PCOLCE2	19.69016061	33.816995	5.563326601	-2.611977812	0.604525274	-4.320709	1.56E-05	5.12E-05
ENSG00000205221	VIT	9.288188861	15.921419	2.654958912	-2.612437523	0.878580638	-2.973475	0.002944	0.007225
ENSG00000102981	PARD6A	27.5454104	47.368811	7.722009402	-2.617090804	0.491228224	-5.3276475	9.95E-08	4.00E-07
ENSG00000132170	PPARG	201.4454415	346.44093	56.44995127	-2.620597166	0.201951494	-12.976369	1.67E-38	2.21E-37
ENSG00000023171	GRAMD1B	198.2029356	341.12563	55.28023801	-2.627617085	0.186323834	-14.102421	3.67E-45	5.62E-44
ENSG00000261087	AP003469.4	9.383290887	16.137039	2.629543	-2.635916667	0.840869671	-3.1347506	0.00172	0.004366
ENSG00000146021	KLHL3	456.7497491	786.97553	126.523964	-2.636652202	0.127807151	-20.629927	1.48E-94	4.59E-93
ENSG00000170312	CDK1	299.0014685	515.48923	82.51370242	-2.641551983	0.1688114	-15.647948	3.43E-55	6.22E-54
ENSG00000120322	PCDHB8	45.9805897	79.131829	12.82935027	-2.644185609	0.43212127	-6.1190823	9.41E-10	4.39E-09
ENSG00000117472	TSPAN1	8.036918008	13.857259	2.216576688	-2.65105887	0.918166978	-2.8873385	0.003885	0.009355
ENSG00000133083	DCLK1	24.21162925	41.752342	6.670916253	-2.652745634	0.595199776	-4.4568996	8.32E-06	2.81E-05
ENSG00000090889	KIF4A	419.0414125	723.23119	114.8516378	-2.653294847	0.134461918	-19.732686	1.13E-86	3.21E-85
ENSG00000065609	SNAP91	6.648712522	11.500877	1.796548313	-2.654895745	1.025738036	-2.5882785	0.009646	0.021644
ENSG00000244471	AL137058.1	11.98400779	20.737053	3.230962139	-2.660148744	0.778381101	-3.4175403	0.000632	0.001713
ENSG00000103647	CORO2B	431.3936249	745.14443	117.6428203	-2.66081836	0.136595245	-19.479583	1.64E-84	4.57E-83
ENSG00000172159	FRMD3	402.5477548	695.69499	109.4005163	-2.665151036	0.140035522	-19.031964	9.27E-81	2.46E-79
ENSG00000196196	HRCT1	55.85295858	96.396687	15.30923014	-2.666263434	0.40287246	-6.6181328	3.64E-11	1.87E-10
ENSG00000138678	GPAT3	138.0406369	238.84778	37.2334895	-2.68366954	0.227920744	-11.774573	5.28E-32	5.95E-31
ENSG00000176046	NUPR1	711.8837834	1232.5994	191.1681822	-2.68938083	0.126091579	-21.32879	6.14E-101	2.02E-99
ENSG00000117724	CENPF	1270.826917	2201.3783	340.2755495	-2.691049378	0.088377231	-30.449578	1.21E-203	8.29E-202
ENSG00000226950	DANCR	278.571339	482.96796	74.1747148	-2.701944497	0.161873753	-16.691678	1.51E-62	3.09E-61
ENSG00000271978	AL359643.2	38.38733819	66.634948	10.13972809	-2.70501799	0.46724715	-5.7892659	7.07E-09	3.10E-08
ENSG00000139970	RTN1	125.987531	218.64672	33.32833986	-2.711599297	0.251172816	-10.795751	3.61E-27	3.58E-26
ENSG00000230309	AL121718.1	7.054405764	12.221891	1.886920175	-2.71161403	1.046918524	-2.5900908	0.009595	0.021548
ENSG00000255690	TRIL	12.60476495	21.883966	3.325563724	-2.723616748	0.820341978	-3.3200992	0.0009	0.002387
ENSG00000175063	UBE2C	199.918169	347.36639	52.46995128	-2.725225085	0.188023879	-14.494037	1.32E-47	2.12E-46
ENSG00000064692	SNCAIP	99.29103113	172.64747	25.93458959	-2.728021047	0.280289472	-9.7328702	2.18E-22	1.86E-21
ENSG00000106852	LHX6	72.15489967	125.27193	19.03786577	-2.728590644	0.348712436	-7.8247586	5.09E-15	3.20E-14
ENSG00000116833	NR5A2	636.1585241	1108.0237	164.2933072	-2.755174084	0.114535565	-24.055184	7.37E-128	3.12E-126
ENSG00000154839	SKA1	130.1682161	226.78781	33.54862363	-2.758573363	0.233183389	-11.830059	2.73E-32	3.11E-31
ENSG00000158077	NLRP14	18.91520438	32.908041	4.922367424	-2.765283988	0.631895253	-4.3761747	1.21E-05	4.01E-05
ENSG00000184661	CDCA2	252.7668229	440.62329	64.91035377	-2.766026034	0.19311987	-14.322845	1.58E-46	2.48E-45
ENSG00000135312	HTR1B	859.3700874	1499.4054	219.3348043	-2.771850356	0.10883365	-25.468689	4.38E-143	2.05E-141
ENSG00000140009	ESR2	27.34434459	47.755905	6.932784477	-2.777538334	0.537583369	-5.1667118	2.38E-07	9.28E-07
ENSG00000184344	GDF3	111.6531215	194.81761	28.4886352	-2.77827688	0.271196343	-10.244522	1.25E-24	1.15E-23
ENSG00000140678	ITGAX	37.12254918	64.905887	9.339211375	-2.779355593	0.487699337	-5.698912	1.21E-08	5.19E-08
ENSG00000143850	PLEKHA6	388.2458244	677.74249	98.7491596	-2.780945805	0.323623053	-8.5931635	8.46E-18	6.03E-17
ENSG00000168758	SEMA4C	338.2196146	590.41001	86.02921966	-2.782166592	0.148998899	-18.672397	8.30E-78	2.11E-76
ENSG00000165272	AQP3	24.54185566	42.900612	6.183099589	-2.786684835	0.543470878	-5.1275697	2.94E-07	1.14E-06
ENSG00000091137	SLC26A4	56.56921566	98.883915	14.25451674	-2.787997216	0.380226483	-7.3324646	2.26E-13	1.31E-12
ENSG00000137727	ARHGAP20	157.1590216	274.73384	39.58420781	-2.800131528	0.229539038	-12.198934	3.15E-34	3.74E-33
ENSG00000015413	DPEP1	55.31056299	96.845929	13.77519709	-2.800868484	0.37257202	-7.5176565	5.58E-14	3.31E-13
ENSG00000073910	FRY	1703.151901	2980.5581	425.7456849	-2.806208912	0.075807368	-37.017627	5.96E-300	7.00E-298
ENSG00000080986	NDC80	229.3263912	401.27036	57.3824243	-2.806903729	0.181557006	-15.460179	6.44E-54	1.15E-52
ENSG00000123485	HJURP	223.5116297	390.98692	56.03633792	-2.810173038	0.193654455	-14.511275	1.03E-47	1.66E-46
ENSG00000138449	SLC40A1	880.71517	1542.3779	219.0524251	-2.815130728	0.106531682	-26.425291	7.02E-154	3.48E-152
ENSG00000187134	AKR1C1	23.58433406	41.401367	5.767300937	-2.826813358	0.571426811	-4.9469386	7.54E-07	2.82E-06

ENSG00000139174	PRICKLE1	1226.63855	2150.5151	302.762007	-2.828472506	0.086278822	-32.782929	1.03E-235	8.31E-234
ENSG00000100162	CENPM	68.57949626	120.22973	16.92926445	-2.829430926	0.323725442	-8.740218	2.33E-18	1.70E-17
ENSG00000101331	CCM2L	213.5668198	374.73858	52.39506336	-2.836334979	0.219464356	-12.923898	3.30E-38	4.33E-37
ENSG00000174371	EXO1	92.72978955	162.70229	22.75729162	-2.836371946	0.284206596	-9.9799652	1.87E-23	1.65E-22
ENSG00000270441	AC135506.1	4.525039252	7.9453212	1.104757313	-2.841954233	1.260462815	-2.2546911	0.024153	0.049921
ENSG00000249863	AC021106.1	22.4212969	39.409179	5.433414701	-2.844477691	0.563896852	-5.0443227	4.55E-07	1.73E-06
ENSG00000167900	TK1	412.9269045	725.66686	100.1869495	-2.85574445	0.146440374	-19.501073	1.08E-84	3.01E-83
ENSG00000128917	DLL4	1033.596226	1817.0422	250.1502801	-2.861942242	0.093139117	-30.727608	2.44E-207	1.68E-205
ENSG00000076382	SPAG5	335.1229904	589.20835	81.03762898	-2.863866806	0.159436982	-17.962375	3.84E-72	9.18E-71
ENSG00000112245	PTP4A1	4.645325647	8.153416	1.137235287	-2.866815082	1.245090335	-2.3024957	0.021307	0.044594
ENSG00000155011	DKK2	7.649226269	13.451072	1.847380137	-2.872607077	1.005683817	-2.856372	0.004285	0.010236
ENSG00000241717	VWFP1	93.54049911	164.92025	22.16074924	-2.88446942	0.291929283	-9.8807129	5.05E-23	4.38E-22
ENSG00000166387	PPFIBP2	33.93716232	59.790181	8.08414389	-2.885461067	0.46311355	-6.2305693	4.65E-10	2.22E-09
ENSG00000179314	WSCD1	1408.395497	2481.0167	335.7742802	-2.886291547	0.086287502	-33.449706	2.60E-245	2.29E-243
ENSG00000092853	CLSPN	382.5092209	674.15235	90.86609198	-2.89159502	0.145913615	-19.817171	2.12E-87	6.09E-86
ENSG00000179750	APOBEC3B	74.55365574	131.34365	17.76365652	-2.892968132	0.343528532	-8.4213329	3.72E-17	2.59E-16
ENSG00000144354	CDCA7	172.7871254	304.62621	40.94803858	-2.898672197	0.207624455	-13.961131	2.69E-44	4.05E-43
ENSG00000124749	COL21A1	15.53155724	27.405062	3.658052577	-2.900661413	0.689032688	-4.2097588	2.56E-05	8.22E-05
ENSG00000130768	SMPDL3B	6.223893331	10.987957	1.459829737	-2.905958071	1.057774631	-2.7472374	0.00601	0.013978
ENSG00000157150	TIMP4	4.652910235	8.2264791	1.079341401	-2.913373096	1.261416827	-2.3096038	0.02091	0.043841
ENSG00000008277	ADAM22	21.90810918	38.705398	5.110820251	-2.914390013	0.567612962	-5.134467	2.83E-07	1.10E-06
ENSG00000179546	HTR1D	84.65656937	149.61671	19.69642846	-2.919335597	0.319300173	-9.1429189	6.08E-20	4.75E-19
ENSG00000111206	FOXM1	910.4292238	1609.1307	211.7277572	-2.923895694	0.115803804	-25.248702	1.17E-140	5.36E-139
ENSG00000263513	FAM72C	6.315252564	11.170675	1.459829737	-2.926402071	1.101689341	-2.6562861	0.007901	0.018027
ENSG00000011426	ANLN	1337.111659	2366.1289	308.0944427	-2.940204712	0.082711237	-35.547827	8.98E-277	9.27E-275
ENSG00000007402	CACNA2D2	6.385915312	11.304939	1.4668918	-2.944063383	1.049920705	-2.8040817	0.005046	0.011895
ENSG00000109805	NCAPG	487.4768383	863.12261	111.831065	-2.949066857	0.134071621	-21.996205	3.13E-107	1.10E-105
ENSG00000271447	MMP28	22.8386225	40.385681	5.291563975	-2.953626794	0.63104345	-4.6805443	2.86E-06	1.01E-05
ENSG00000197149	AC107956.1	11.50152402	20.355151	2.647896849	-2.964898598	0.885548318	-3.3480935	0.000814	0.002172
ENSG00000169744	LDB2	2146.825272	3806.1717	487.478844	-2.965009061	0.071533241	-41.449388	0	0
ENSG00000157456	CCNB2	267.4585539	474.54802	60.36908415	-2.970909873	0.18060534	-16.449734	8.42E-61	1.66E-59
ENSG00000112742	TTK	187.5976813	332.83051	42.3648489	-2.982476076	0.222813281	-13.38554	7.35E-41	1.03E-39
ENSG00000137960	GIPC2	349.4794836	620.96603	77.99293437	-2.989424044	0.16811998	-17.781492	9.83E-71	2.28E-69
ENSG00000158321	AUTS2	5.064913473	8.9671757	1.162651199	-2.994707757	1.240674765	-2.4137734	0.015788	0.033998
ENSG00000135451	TROAP	106.956162	190.15477	23.75755294	-2.995976888	0.270827525	-11.062306	1.91E-28	1.97E-27
ENSG00000198885	ITPRIPL1	24.92285927	44.256976	5.588742513	-2.999224963	0.570606786	-5.2562028	1.47E-07	5.82E-07
ENSG00000168309	FAM107A	2112.809081	3756.1566	469.4615626	-2.999823045	0.087018542	-34.473378	2.01E-260	1.91E-258
ENSG00000137812	KNL1	496.9388175	883.57982	110.2978199	-3.001971155	0.126798928	-23.675051	6.52E-124	2.67E-122
ENSG00000186451	SPATA12	6.623617645	11.787406	1.459829737	-3.002927365	1.053352953	-2.8508273	0.004361	0.010393
ENSG00000036672	USP2	8.268712066	14.71546	1.821964225	-3.003134198	0.932662762	-3.2199572	0.001282	0.003332
ENSG00000047648	ARHGAP6	64.90602834	115.38949	14.42257129	-3.005426852	0.342799512	-8.767302	1.83E-18	1.35E-17
ENSG00000184260	HIST2H2AC	4.972606444	8.8475176	1.09769525	-3.008860692	1.232705066	-2.4408602	0.014652	0.031766
ENSG00000175899	A2M	211.8794785	376.84875	46.91020737	-3.009007677	0.218888172	-13.746781	5.32E-43	7.85E-42
ENSG00000138722	MMRN1	38488.28577	68473.431	8503.140112	-3.009350906	0.044420554	-67.746811	0	0
ENSG00000260788	AC009063.2	13.5450539	24.047598	3.042509312	-3.011337614	0.769279692	-3.9144899	9.06E-05	0.000274
ENSG00000101057	MYBL2	759.1065704	1353.2082	165.0049903	-3.040355157	0.125700425	-24.18731	3.03E-129	1.30E-127
ENSG00000165821	SALL2	61.89050215	110.31069	13.47030945	-3.048570098	0.370990617	-8.2173779	2.08E-16	1.40E-15
ENSG00000261253	AC137932.2	11.91428309	21.271041	2.557524987	-3.048907727	0.796535168	-3.8277126	0.000129	0.000383
ENSG00000151468	CCDC3	56.84507149	101.53358	12.15656016	-3.062940462	0.370947786	-8.2570663	1.49E-16	1.01E-15
ENSG00000124496	TRERF1	5.105469721	9.131598	1.079341401	-3.06295964	1.247404531	-2.4554662	0.01407	0.0306
ENSG00000075218	GTSE1	366.9375525	655.69579	78.17938035	-3.067397345	0.163492858	-18.76166	1.55E-78	3.98E-77
ENSG00000105088	OLFM2	12.13721123	21.709835	2.56458705	-3.075392346	0.789183006	-3.8969318	9.74E-05	0.000293
ENSG00000205038	PKHD1L1	416.4880413	744.83482	88.14126278	-3.081249395	0.160052818	-19.251454	1.37E-82	3.73E-81
ENSG00000115163	CENPA	94.04095441	168.19205	19.88986135	-3.081660958	0.284894379	-10.816854	2.86E-27	2.86E-26
ENSG00000118407	FILIP1	252.193026	451.21237	53.17368111	-3.082501253	0.407444642	-7.565448	3.87E-14	2.32E-13
ENSG00000051341	POLQ	106.4780412	190.46632	22.48975871	-3.084487988	0.267666956	-11.523604	1.00E-30	1.09E-29
ENSG00000251314	AC104123.1	65.27762196	116.88519	13.67005406	-3.100713819	0.339206049	-9.1410923	6.18E-20	4.83E-19
ENSG00000099998	GGT5	1071.849109	1920.5479	223.1503324	-3.105328643	0.092982594	-33.396881	1.52E-244	1.33E-242
ENSG00000110881	ASIC1	36.86404086	66.120463	7.607619011	-3.107565981	0.488497766	-6.3614743	2.00E-10	9.77E-10
ENSG00000147642	SYBU	164.063002	294.19485	33.93115639	-3.10952272	0.231934547	-13.406898	5.51E-41	7.74E-40
ENSG00000167779	IGFBP6	161.3635566	289.56116	33.16594999	-3.118717657	0.231242522	-13.486783	1.87E-41	2.66E-40
ENSG00000171848	RRM2	1026.410289	1841.5205	211.3000573	-3.121526787	0.094431836	-33.055873	1.28E-239	1.07E-237
ENSG00000131747	TOP2A	1466.73607	2631.8256	301.6465392	-3.122616071	0.084493174	-36.957022	5.62E-299	6.52E-297
ENSG00000104147	OIP5	44.77419456	80.373612	9.174777077	-3.129461421	0.412655081	-7.583722	3.36E-14	2.02E-13
ENSG00000137135	ARHGEF39	16.38126849	29.357786	3.404643799	-3.13563042	0.712632415	-4.4000671	1.08E-05	3.61E-05
ENSG00000233370	AC140479.3	5.486007432	9.8418416	1.130173225	-3.14611908	1.190204732	-2.6433428	0.008209	0.018657
ENSG00000172794	RAB37	9.040023495	16.258083	1.821964225	-3.150718492	0.919251097	-3.4274841	0.000609	0.001656
ENSG00000100336	APOL4	25.88064223	46.444305	5.316979887	-3.154857384	0.600555802	-5.2532294	1.49E-07	5.91E-07
ENSG00000237928	NFIA-AS2	9.286001578	16.659667	1.912336087	-3.158320351	0.981025761	-3.2194061	0.001285	0.003337
ENSG00000174175	SELP	121.1348359	217.77298	24.49669639	-3.161030503	0.258735655	-12.21722	2.52E-34	3.00E-33
ENSG00000185585	OLFML2A	20.11112576	36.244437	3.977814687	-3.1712916	0.648010805	-4.8938869	9.89E-07	3.66E-06
ENSG00000072571	HMMR	237.6596283	428.03358	47.28567806	-3.175585976	0.189301123	-16.775315	3.70E-63	7.64E-62
ENSG00000152154	TMEM178A	13.01614971	23.416881	2.615418874	-3.17912077	0.793422349	-4.0068455	6.15E-05	0.000189
ENSG00000140465	CYP1A1	330.8579513	596.0178	65.69810617	-3.184404449	0.17936895	-17.753376	1.62E-70	3.76E-69
ENSG00000156970	BUB1B	304.9466135	549.65252	60.24071051	-3.194781701	0.173653903	-18.397408	1.38E-75	3.43E-74
ENSG00000085840	ORC1	97.92979777	176.57678	19.2828151	-3.202733538	0.302964847	-10.571304	4.05E-26	3.91E-25
ENSG00000091879	ANGPT2	1762.562157	3180.1247	344.9995979	-3.204165747	0.170323816	-18.8122	6.00E-79	1.55E-77
ENSG00000197565	COL4A6	1503.510563	2713.0299	293.9912306	-3.209089643	0.094866538	-33.827414	7.80E-251	7.08E-249
ENSG00000086696	HSD17B2	22.72882942	41.071108	4.386551275	-3.218199749	0.68290714	-4.7124998	2.45E-06	8.73E-06
ENSG00000250770	AC005865.2	7.640106469	13.794967	1.485245649	-3.223876081	1.018202051	-3.1662439	0.001544	0.003955
ENSG00000225937	PCA3	5.669217603	10.259094	1.079341401	-3.224028943	1.183472462	-2.7242112	0.006446	0.014923
ENSG00000128408	RIBC2	7.582535617	13.716533	1.448537952	-3.22847926	1.061759429	-3.0406881	0.00236	0.005866
ENSG00000139055	ERP27	9.573584191	17.30685	1.840318074	-3.234267152	0.921350427	-3.5103551	0.000448	0.001238
ENSG00000171241	SHCBP1	400.2562933	723.6081	76.90448649	-3.235301387	0.161832291	-19.991692	6.51E-89	1.90E-87
ENSG00000181444	ZNF467	185.1131782	335.00403	35.2223221	-3.247363546	0.210137504	-15.453517	7.14E-54	1.27E-52

ENSG00000064042	LIMCH1	1767.498702	3203.3856	331.6118197	-3.269043935	0.097163994	-33.644602	3.74E-248	3.31E-246
ENSG00000154027	AK5	41.07634976	74.473063	7.679637024	-3.273409483	0.452142917	-7.2397672	4.49E-13	2.56E-12
ENSG00000121152	NCAPH	185.3910322	336.29247	34.48959368	-3.283762923	0.21277728	-15.432864	9.84E-54	1.75E-52
ENSG00000174640	SLCO2A1	560.1599573	1016.6952	103.6246807	-3.291083726	0.14141884	-23.27189	8.54E-120	3.39E-118
ENSG00000126882	FAM78A	624.7953644	1133.8594	115.7313379	-3.297547116	0.124474857	-26.491672	1.21E-154	6.02E-153
ENSG00000204403	CASP12	42.02538705	76.299119	7.751655037	-3.301328494	0.454444523	-7.2645358	3.74E-13	2.14E-12
ENSG00000146674	IGFBP3	623.2211902	1132.8734	113.5689691	-3.315753373	0.13265771	-24.994803	6.96E-138	3.12E-136
ENSG00000104611	SH2D4A	16.2118247	29.457388	2.966261576	-3.322487526	0.732523597	-4.535673	5.74E-06	1.97E-05
ENSG00000117152	RGS4	3246.80454	5905.307	588.3020543	-3.328425525	0.078446103	-42.429457	0	0
ENSG00000158458	NRG2	20.61605656	37.439919	3.792194199	-3.333666209	0.658492592	-5.0625721	4.14E-07	1.58E-06
ENSG00000198075	SULT1C4	102.6829748	186.88648	18.47946605	-3.343023682	0.285144654	-11.723957	9.61E-32	1.08E-30
ENSG00000146147	MLIP	33.70438741	61.33788	6.070894497	-3.346379282	1.040441215	-3.2163079	0.001299	0.003369
ENSG00000253736	AC022217.3	4.060354988	7.4105651	0.71014485	-3.349467551	1.435036542	-2.3340643	0.019592	0.041341
ENSG00000100024	UPB1	6.175324985	11.245893	1.104757313	-3.350779616	1.173439149	-2.8555206	0.004297	0.01026
ENSG00000019102	VSIG2	12.52874375	22.815495	2.2419926	-3.35864748	0.84773287	-3.9619172	7.44E-05	0.000227
ENSG00000002079	MYH16	10.73946949	19.599081	1.879858112	-3.405653785	0.89179499	-3.8188752	0.000134	0.000396
ENSG00000204161	TMEM273	110.5165665	202.14995	18.883185	-3.407856228	0.283834793	-12.006478	3.29E-33	3.82E-32
ENSG00000166851	PLK1	563.6362499	1030.0743	97.19824523	-3.409880424	0.153457367	-22.220376	2.18E-109	7.87E-108
ENSG00000270419	CAHM	4.332276501	7.9148681	0.749684888	-3.415081953	1.404079844	-2.4322562	0.015005	0.032453
ENSG00000249087	ZNF436-AS1	6.411537216	11.743733	1.079341401	-3.418977922	1.155697651	-2.9583671	0.003093	0.007552
ENSG00000163751	CPA3	133.7399862	244.65066	22.82930963	-3.422229891	0.264593757	-12.933903	2.90E-38	3.81E-37
ENSG00000106123	EPHB6	56.24247531	102.95793	9.527017162	-3.430913616	0.402048789	-8.5335753	1.42E-17	1.00E-16
ENSG00000164484	TMEM200A	6.503225013	11.901693	1.104757313	-3.431441137	1.137177038	-3.0175083	0.002549	0.006311
ENSG00000186479	RGS7BP	319.4033475	584.81339	53.99330201	-3.435822789	0.176818381	-19.431367	4.19E-84	1.16E-82
ENSG00000111907	TPD52L1	43.42444002	79.570918	7.277962498	-3.440614354	0.460826494	-7.4661817	8.26E-14	4.87E-13
ENSG00000184305	CCSER1	4.371886721	8.0011506	0.742622825	-3.440900377	1.413718556	-2.4339359	0.014936	0.032325
ENSG00000180730	SHISA2	24.02251935	43.995206	4.049832699	-3.443506386	0.59357225	-5.8013264	6.58E-09	2.89E-08
ENSG00000049283	EPN3	36.92384489	67.649069	6.198621098	-3.445636797	1.017499596	-3.3863766	0.000708	0.001907
ENSG00000148773	MKI67	2442.39668	4478.7848	406.0085632	-3.465121946	0.070149306	-49.396382	0	0
ENSG00000189057	FAM111B	234.6863641	430.63922	38.73350631	-3.46858513	0.201355327	-17.22619	1.69E-66	3.71E-65
ENSG00000024526	DEPDC1	304.5613487	558.5615	50.56119788	-3.469682066	0.173689961	-19.976296	8.86E-89	2.58E-87
ENSG00000095203	EPB41L4B	11.02341331	20.250278	1.796548313	-3.470750045	0.883364358	-3.929013	8.53E-05	0.000258
ENSG00000101447	FAM83D	303.9684055	557.78575	50.15106391	-3.478598345	0.176142852	-19.748734	8.23E-87	2.34E-85
ENSG00000138180	CEP55	562.6222994	1033.1397	92.10488762	-3.482666807	0.140297208	-24.823493	5.00E-136	2.23E-134
ENSG00000165244	ZNF367	60.96855733	111.96465	9.972461448	-3.493305413	0.383219485	-9.115678	7.82E-20	6.07E-19
ENSG00000271380	AL451085.2	4.496910557	8.2766142	0.717206913	-3.503989861	1.376762454	-2.545094	0.010925	0.024273
ENSG00000151715	TMEM45B	11.49953423	21.144626	1.8544422	-3.520711064	0.861307422	-4.0876358	4.36E-05	0.000137
ENSG00000151778	SERP2	4.661799466	8.573914	0.749684888	-3.537731571	1.373538549	-2.5756333	0.010006	0.022371
ENSG00000253177	AC104211.2	13.98231067	25.762169	2.202452562	-3.545212497	0.788970551	-4.4934662	7.01E-06	2.39E-05
ENSG00000084734	GCKR	54.20192085	99.874254	8.529588177	-3.557751727	0.409801629	-8.6816437	3.90E-18	2.82E-17
ENSG00000091622	PITPNM3	77.49898387	142.91482	12.08314477	-3.560444165	0.344343347	-10.339808	4.65E-25	4.33E-24
ENSG00000065328	MCM10	140.9598873	259.95877	21.96100463	-3.56131459	0.258894705	-13.755842	4.70E-43	6.94E-42
ENSG00000066279	ASPM	827.4806519	1526.0401	128.9212187	-3.56172939	0.117195337	-30.391392	7.14E-203	4.82E-201
ENSG00000204060	FOXO6	4.749774776	8.7569267	0.742622825	-3.567266098	1.361994588	-2.6191485	0.008815	0.019922
ENSG00000117650	NEK2	94.64780585	174.71567	14.57994353	-3.575720028	0.329577985	-10.84939	2.01E-27	2.02E-26
ENSG00000178999	AURKB	158.0541649	291.59611	24.5122179	-3.582091243	0.259120063	-13.82406	1.82E-43	2.72E-42
ENSG00000128040	SPINK2	4.742220788	8.7601726	0.724268976	-3.584968605	1.461082973	-2.4536379	0.014142	0.030734
ENSG00000213468	FIRRE	4.850955255	8.9847036	0.717206913	-3.618964772	1.362161239	-2.6567815	0.007889	0.018003
ENSG00000184611	KCNH7	4.869712825	9.0151567	0.724268976	-3.619636823	1.377315874	-2.6280368	0.008588	0.019458
ENSG00000178175	ZNF366	128.4645737	237.65906	19.27008836	-3.632402632	0.269380879	-13.484263	1.94E-41	2.75E-40
ENSG00000169282	KCNAB1	1129.732051	2092.3329	167.1311951	-3.651059592	0.103661958	-35.220824	9.60E-272	9.70E-270
ENSG00000136928	GABBR2	1625.543803	3012.7707	238.3168863	-3.66056181	0.095525658	-38.320195	0	0
ENSG00000092068	SLC7A8	45.18091355	83.837779	6.524047888	-3.667732728	0.460172045	-7.970351	1.58E-15	1.02E-14
ENSG00000120875	DUSP4	2194.202009	4069.4703	318.9337366	-3.673125196	0.083601647	-43.936039	0	0
ENSG00000186871	ERCC6L	89.11792938	165.43411	12.80174906	-3.686805208	0.331293105	-11.12853	9.11E-29	9.47E-28
ENSG00000112984	KIF20A	559.4940405	1038.8117	80.17642362	-3.696082155	0.136083164	-27.160466	1.90E-162	1.01E-160
ENSG00000038295	TLL1	177.1302131	329.10929	25.15113265	-3.715731163	0.535726117	-6.9358783	4.04E-12	2.20E-11
ENSG00000196421	C20orf204	28.6604331	53.311971	4.008895279	-3.729865198	1.18948629	-3.1356941	0.001714	0.004354
ENSG00000163053	SLC16A14	16.14861821	30.029828	2.267408512	-3.754708788	0.787615429	-4.7671854	1.87E-06	6.74E-06
ENSG00000108602	ALDH3A1	5.294750815	9.8722947	0.717206913	-3.754830463	1.345512796	-2.7906316	0.005261	0.012364
ENSG00000148123	PLPPR1	5.329659409	9.9421119	0.717206913	-3.762918532	1.330644353	-2.827892	0.004686	0.011111
ENSG00000159640	ACE	2112.083191	3935.3278	288.8385817	-3.768313211	0.090264767	-41.747332	0	0
ENSG00000267175	AC105094.2	5.422724033	10.095763	0.749684888	-3.773504522	1.317687084	-2.8637334	0.004187	0.010027
ENSG00000283429	MIR1244-3	2.90733678	5.4596011	0.355072425	-3.783221723	1.663166125	-2.2747107	0.022923	0.047595
ENSG00000179242	CDH4	1352.2819	2521.4184	183.1454492	-3.783540906	0.096225528	-39.319513	0	0
ENSG00000196169	KIF19	147.873354	275.83566	19.91104754	-3.794927025	0.257179496	-14.755947	2.82E-49	4.64E-48
ENSG00000126787	DLGAP5	951.7075004	1775.7736	127.641448	-3.798678526	0.105122268	-36.135812	6.22E-286	6.79E-284
ENSG00000158402	CDC25C	44.20072223	82.418089	5.983354976	-3.802277261	0.480351923	-7.9156075	2.46E-15	1.57E-14
ENSG00000171864	PRND	22.23842481	41.485172	2.991677488	-3.810061898	0.668623441	-5.6983672	1.21E-08	5.21E-08
ENSG00000237451	CDK2AP2P2	8.259530901	15.43972	1.079341401	-3.81573158	1.106806396	-3.4475149	0.000566	0.001544
ENSG00000186185	KIF18B	150.2298772	280.54448	19.91527726	-3.819270471	0.263974541	-14.468329	1.92E-47	3.08E-46
ENSG00000129810	SGO1	72.1509082	134.8214	9.480415061	-3.821288296	0.386778266	-9.8797906	5.09E-23	4.42E-22
ENSG00000123977	DAW1	106.8210121	199.60919	14.03283559	-3.8248291	0.755347402	-5.0636688	4.11E-07	1.57E-06
ENSG00000089685	BIRC5	491.4211089	918.21431	64.62790883	-3.827414147	0.150212678	-25.479968	3.29E-143	1.54E-141
ENSG00000211445	GPX3	382.4369018	714.54343	50.33037267	-3.834151152	0.168583234	-22.743372	1.67E-114	6.28E-113
ENSG00000163808	KIF15	123.8190842	231.35693	16.28124321	-3.835769731	0.28309764	-13.549282	8.00E-42	1.15E-40
ENSG00000240401	AC012358.3	3.015992321	5.6698502	0.362134488	-3.841400143	1.63193297	-2.3538958	0.018578	0.039435
ENSG00000035499	DEPDC1B	82.06032389	153.51569	10.60496118	-3.850486258	0.359932014	-10.697815	1.04E-26	1.02E-25
ENSG00000163520	FBLN2	11.49334403	21.461902	1.524785687	-3.852787465	0.968214073	-3.9792723	6.91E-05	0.000211
ENSG00000121211	MND1	11.74181739	21.965911	1.517723624	-3.887704427	0.936963992	-4.149257	3.34E-05	0.000106
ENSG00000167641	PPP1R14A	26.75394185	50.117364	3.390519674	-3.91137595	0.640711964	-6.1047337	1.03E-09	4.79E-09
ENSG00000165071	TMEM71	151.9096517	284.95652	18.86278673	-3.923587355	0.267883432	-14.646622	1.42E-48	2.31E-47
ENSG00000165480	SKA3	148.5047757	278.73972	18.26982704	-3.946252292	0.27062267	-14.58212	3.65E-48	5.92E-47
ENSG00000120756	PLS1	45.38884751	85.249679	5.528016286	-3.949491637	0.484073311	-8.1588709	3.38E-16	2.25E-15
ENSG00000136014	USP44	6.113978519	11.478272	0.749684888	-3.960441342	1.314937754	-3.011885	0.002596	0.006423

ENSG00000152253	SPC25	79.44413233	149.24263	9.645637275	-3.96212598	0.364582004	-10.867585	1.64E-27	1.66E-26
ENSG00000129195	PIMREG	115.9961518	218.08863	13.90367403	-3.969758578	0.630923793	-6.2919779	3.13E-10	1.51E-09
ENSG00000118777	ABCG2	242.3549936	455.80207	28.90791323	-3.973139113	0.211203035	-18.811941	6.03E-79	1.56E-77
ENSG00000079102	RUNX1T1	287.7944409	541.26233	34.32655677	-3.983116873	0.192196412	-20.7242	2.10E-95	6.60E-94
ENSG00000171345	KRT19	1893.833802	3562.9373	224.7302829	-3.987797466	0.186548154	-21.376773	2.20E-101	7.26E-100
ENSG00000112414	ADGRG6	457.131427	860.53569	53.72716649	-4.001852328	0.294151667	-13.604724	3.75E-42	5.43E-41
ENSG00000284128	AP000356.3	3.45296089	6.5183714	0.3875504	-4.044204217	1.602777163	-2.523248	0.011628	0.02569
ENSG00000265107	GJA5	300.101023	565.8543	34.34774295	-4.046868998	0.197903524	-20.448696	6.17E-93	1.89E-91
ENSG00000274736	CCL23	6.499553396	12.249422	0.749684888	-4.051508383	1.289743492	-3.1413288	0.001682	0.004278
ENSG00000139737	SLAIN1	19.58500933	36.992982	2.17703665	-4.071333301	0.737442398	-5.5208831	3.37E-08	1.41E-07
ENSG00000169851	PCDH7	194.2047815	366.63278	21.77678152	-4.0780853	0.255296451	-15.973921	1.94E-57	3.64E-56
ENSG00000148671	ADIRF	411.2193662	776.82184	45.61689392	-4.082082079	0.173219335	-23.565972	8.61E-123	3.50E-121
ENSG00000156509	FBXO43	6.632677055	12.522731	0.742622825	-4.087161178	1.269466981	-3.2195884	0.001284	0.003336
ENSG00000124440	HIF3A	99.48903332	188.05334	10.92472329	-4.093937707	0.335106511	-12.216825	2.53E-34	3.01E-33
ENSG00000118308	LRMP	3.572599215	6.7830639	0.362134488	-4.102556553	1.605857201	-2.5547456	0.010627	0.023658
ENSG00000143375	CGN	10.10924425	19.088315	1.130173225	-4.107760812	1.075163547	-3.8205916	0.000133	0.000394
ENSG00000268592	AL355312.3	3.645283331	6.9030163	0.3875504	-4.12403845	1.583003771	-2.6051981	0.009182	0.020697
ENSG00000205054	LINC01121	3.634429855	6.9137873	0.355072425	-4.125939011	1.57525054	-2.6192272	0.008813	0.01992
ENSG00000270547	LINC01235	1089.370921	2060.7588	117.983084	-4.126153319	0.126971582	-32.496668	1.19E-231	9.23E-230
ENSG00000203883	SOX18	1177.703948	2228.1038	127.30412	-4.133018552	0.110028554	-37.563145	0	0
ENSG00000139187	KLRG1	148.038847	280.03997	16.03772884	-4.141525872	0.283357221	-14.615918	2.22E-48	3.62E-47
ENSG00000227160	THEM7P	3.687564752	7.012995	0.362134488	-4.1524121	1.611696935	-2.5764224	0.009983	0.022328
ENSG00000260922	AC009139.1	6.979334654	13.208984	0.749684888	-4.15976772	1.262295065	-3.2954004	0.000983	0.002595
ENSG00000244476	ERVFRD-1	7.005479322	13.261274	0.749684888	-4.164480786	1.269168014	-3.2812683	0.001033	0.002719
ENSG00000186193	SAPCD2	284.3180922	538.42506	30.21112108	-4.170503724	0.221253304	-18.849453	2.97E-79	7.75E-78
ENSG00000117399	CDC20	773.5465286	1465.9086	81.18449735	-4.178035879	0.149498434	-27.947021	7.17E-172	4.08E-170
ENSG00000137033	IL33	652.1809167	1235.9466	68.41522626	-4.180012911	0.154170991	-27.112837	6.95E-162	3.66E-160
ENSG00000111341	MGP	2531.699387	4798.6686	264.7302237	-4.180794511	0.088246243	-47.376459	0	0
ENSG00000253490	LINC02099	10.58200279	20.052186	1.111819376	-4.183978509	1.046083787	-3.9996591	6.34E-05	0.000195
ENSG00000144821	MYH15	10.75164341	20.39853	1.104757313	-4.2070283	1.03350041	-4.0706595	4.69E-05	0.000147
ENSG00000128594	LRRC4	64.85210968	123.1844	6.519818165	-4.222453667	0.42697681	-9.8891873	4.64E-23	4.04E-22
ENSG00000110900	TSPAN11	391.8337075	743.92157	39.74584734	-4.227140796	0.177288369	-23.843306	1.19E-125	4.97E-124
ENSG00000158825	CDA	7.233021121	13.755897	0.71014485	-4.234711807	1.274202521	-3.3234213	0.000889	0.002362
ENSG00000243193	AC006387.1	7.319775749	13.889867	0.749684888	-4.235982094	1.256120732	-3.3722731	0.000746	0.002001
ENSG00000124721	DNAH8	11.03724774	20.962676	1.111819376	-4.244935383	1.024897316	-4.1418153	3.45E-05	0.000109
ENSG00000185313	SCN10A	1.923196384	3.8463928	0	-4.24515543	1.881490343	-2.2562728	0.024054	0.049738
ENSG00000147606	SLC26A7	1.949341053	3.8986821	0	-4.261862098	1.89071852	-2.2540966	0.02419	0.049993
ENSG00000143228	NUF2	180.5686567	343.49651	17.64080669	-4.283091494	0.261957572	-16.350325	4.33E-60	8.48E-59
ENSG00000187513	GJA4	442.3177005	842.01223	42.62317201	-4.302852609	0.177155996	-24.288495	2.59E-130	1.13E-128
ENSG00000131386	GALNT15	339.2423134	646.44602	32.03860911	-4.323425075	0.206492666	-20.937427	2.44E-97	7.83E-96
ENSG00000177098	SCN4B	7.831451564	14.938634	0.724268976	-4.348034378	1.266578769	-3.4328969	0.000597	0.001626
ENSG00000143355	LHX9	4.263307866	8.1644812	0.362134488	-4.365129381	1.61052508	-2.7103765	0.006721	0.015491
ENSG00000160180	TFF3	4.300223557	8.2383126	0.362134488	-4.376086743	1.562090805	-2.8014292	0.005088	0.011986
ENSG00000152217	SETBP1	80.69360684	154.00615	7.381061103	-4.386906405	0.405553115	-10.817095	2.86E-27	2.86E-26
ENSG00000169418	NPR1	397.8953818	759.54624	36.24451996	-4.394996191	0.192019412	-22.888291	6.08E-116	2.32E-114
ENSG00000154734	ADAMTS1	1870.060771	3573.4251	166.6963956	-4.423684866	0.236594408	-18.697335	5.20E-78	1.33E-76
ENSG00000223652	AC106786.1	2.181426332	4.3628527	0	-4.429610354	1.920088191	-2.3069828	0.021056	0.04411
ENSG00000149212	SESN3	130.5496773	249.63175	11.4676015	-4.449432985	0.331997811	-13.401995	5.89E-41	8.25E-40
ENSG00000207063	SNORD116-1	2.252173515	4.504347	0	-4.469954956	1.821272424	-2.4543033	0.014116	0.030692
ENSG00000214357	NEURL1B	2.256481924	4.5129638	0	-4.472309044	1.801696109	-2.4822771	0.013055	0.028626
ENSG00000120833	SOCS2	21.44020465	41.000551	1.879858112	-4.472794628	0.774672017	-5.7737914	7.75E-09	3.38E-08
ENSG00000127241	MASP1	12.89148677	24.645738	1.137235287	-4.475101367	1.000054421	-4.4748578	7.65E-06	2.59E-05
ENSG00000168078	PBK	186.7222294	357.66655	15.77790504	-4.500690345	0.270316999	-16.649676	3.04E-62	6.17E-61
ENSG00000166803	PCLAF	53.47926133	102.54656	4.411967187	-4.539274097	0.500699313	-9.0658684	1.24E-19	9.54E-19
ENSG00000122547	EEPD1	4.786083268	9.210032	0.362134488	-4.542269826	1.499261673	-3.0296711	0.002448	0.006072
ENSG00000235016	SEMA3F-AS1	2.414972874	4.8299457	0	-4.569582162	1.772107137	-2.5786151	0.00992	0.0222
ENSG00000224420	ADM5	9.133603868	17.550001	0.717206913	-4.582840586	1.213874817	-3.7753815	0.00016	0.000467
ENSG00000049540	ELN	13.87157337	26.645451	1.09769525	-4.594578625	0.994821796	-4.6184941	3.87E-06	1.35E-05
ENSG00000129173	E2F8	172.4492988	331.05203	13.84656806	-4.60031742	0.291995346	-15.754763	6.37E-56	1.17E-54
ENSG00000185432	METTL7A	282.6993744	543.16663	22.23212021	-4.619672565	0.233484479	-19.78578	3.95E-87	1.13E-85
ENSG00000167755	KLK6	2.496106919	4.9922138	0	-4.622463614	1.830584346	-2.5251301	0.011566	0.025561
ENSG00000138030	KHK	38.14297436	73.319687	2.966261576	-4.637588344	0.608298161	-7.6238737	2.46E-14	1.50E-13
ENSG00000159713	TPPP3	5.16806246	9.9485745	0.3875504	-4.650386074	1.475696532	-3.151316	0.001625	0.004146
ENSG00000110675	ELMOD1	274.0409036	527.26087	20.820937	-4.656153891	0.262818866	-17.716209	3.14E-70	7.24E-69
ENSG00000147113	DIPK2B	2466.539455	4745.5878	187.4910912	-4.662656518	0.201595628	-23.128758	2.38E-118	9.25E-117
ENSG00000259070	LINC00639	24.27395039	46.725937	1.821964225	-4.669929482	0.76016791	-6.1432868	8.08E-10	3.79E-09
ENSG00000255202	AL049629.1	14.5942736	28.08379	1.104757313	-4.671564738	0.989139049	-4.7228595	2.33E-06	8.31E-06
ENSG00000120949	TNFRSF8	5.272174521	10.189277	0.355072425	-4.686198455	1.47801671	-3.1705991	0.001521	0.003899
ENSG00000171476	HOPX	145.2242735	279.70238	10.74616486	-4.711702183	0.32417265	-14.534546	7.32E-48	1.18E-46
ENSG00000007350	TKTL1	2.678588332	5.3571767	0	-4.721920414	1.720793678	-2.7440364	0.006069	0.014106
ENSG00000164867	NOS3	2835.194471	5463.5813	206.8076783	-4.723864337	0.147217932	-32.087561	6.57E-226	4.92E-224
ENSG00000235888	AF064858.1	10.18574983	19.654293	0.717206913	-4.742569949	1.206782935	-3.9299279	8.50E-05	0.000257
ENSG00000113763	UNC5A	30.94126813	59.640544	2.2419926	-4.752044448	0.693242609	-6.8548072	7.14E-12	3.84E-11
ENSG00000152213	ARL11	10.21190355	19.713662	0.71014485	-4.755715001	1.238899326	-3.8386614	0.000124	0.000367
ENSG00000134242	PTPN22	78.98265183	152.30031	5.664990249	-4.781026625	0.443805885	-10.772788	4.63E-27	4.58E-26
ENSG00000053108	FSTL4	2.791399665	5.5827993	0	-4.783965102	1.712315926	-2.7938566	0.005208	0.012252
ENSG00000211767	TRBJ2-3	5.675045706	10.987957	0.362134488	-4.797009502	1.444462762	-3.3209645	0.000897	0.002381
ENSG00000144063	MALL	318.8665576	615.50383	22.22928787	-4.800567148	0.231977268	-20.694127	3.91E-95	1.22E-93
ENSG00000238121	LINC00426	2.9052881	5.8105762	0	-4.843090712	1.780406814	-2.7202158	0.006524	0.015086
ENSG00000074211	PPP2R2C	5.876697689	11.365845	0.3875504	-4.844207931	1.432833793	-3.3808582	0.000723	0.001944
ENSG00000130300	PLVAP	171.7964721	332.18747	11.40547789	-4.864146198	0.307104929	-15.838711	1.68E-56	3.11E-55
ENSG00000170011	MYRIP	353.5982994	683.68464	23.51195657	-4.868317314	0.435459914	-11.179714	5.13E-29	5.35E-28
ENSG00000269086	AC008555.2	3.03263303	6.0652661	0	-4.897251188	1.674376434	-2.9248209	0.003447	0.00836
ENSG00000240583	AQP1	142.2574724	275.28934	9.2256089	-4.903260834	0.340145392	-14.415191	4.15E-47	6.59E-46
ENSG00000137090	DMRT1	3.074697179	6.1493944	0	-4.923341387	1.780124961	-2.765728	0.00568	0.013277

ENSG00000067141	NEO1	3.174820338	6.3496407	0	-4.964114678	1.686918327	-2.9427119	0.003254	0.00791
ENSG00000130035	GALNT8	3.192201081	6.3844022	0	-4.970765866	1.655520142	-3.0025403	0.002677	0.006611
ENSG00000003137	CYP26B1	6.420444009	12.485816	0.355072425	-4.981113887	1.423264804	-3.4997801	0.000466	0.001285
ENSG00000004799	PDK4	122.8506957	238.41353	7.287856901	-5.019899596	0.380569926	-13.190479	9.96E-40	1.36E-38
ENSG00000249631	AC005699.1	18.61776778	36.123716	1.111819376	-5.033176914	0.959835579	-5.2437907	1.57E-07	6.21E-07
ENSG00000176244	ACBD7	6.659291626	12.956449	0.362134488	-5.03643015	1.425208296	-3.5338204	0.00041	0.001139
ENSG00000277494	GPIHBP1	7.189227798	13.990905	0.3875504	-5.14089135	1.424026281	-3.6101099	0.000306	0.000866
ENSG00000074317	SNCB	13.34511208	25.965955	0.724268976	-5.144099239	1.159691371	-4.4357485	9.18E-06	3.08E-05
ENSG00000167644	C19orf33	3.628456926	7.2569139	0	-5.156611624	1.600330168	-3.2222173	0.001272	0.003309
ENSG00000255856	AC069503.1	3.750416345	7.5008327	0	-5.200380007	1.732478975	-3.0016988	0.002685	0.006628
ENSG00000007968	E2F2	13.99953531	27.281864	0.717206913	-5.216022295	1.155182154	-4.5153245	6.32E-06	2.16E-05
ENSG00000139549	DHH	192.0299094	374.11984	9.939983473	-5.236398059	0.32603746	-16.060725	4.81E-58	9.12E-57
ENSG00000135218	CD36	8.011220195	15.63489	0.3875504	-5.30720509	1.383126641	-3.8371071	0.000124	0.000369
ENSG00000276953	TRBV12-4	4.128466073	8.2569321	0	-5.347862151	1.563336981	-3.4207994	0.000624	0.001694
ENSG00000215183	MSMP	83.21555074	162.73351	3.697592615	-5.466604414	0.511011018	-10.697625	1.04E-26	1.03E-25
ENSG00000226476	LINC01748	4.652996952	9.3059939	0	-5.516816615	1.508599467	-3.6569127	0.000255	0.00073
ENSG00000270885	RASL10B	17.6198566	34.490028	0.749684888	-5.549413333	1.128837351	-4.9160433	8.83E-07	3.28E-06
ENSG00000254975	AP001189.4	4.880626715	9.7612534	0	-5.588735525	1.506553422	-3.7096166	0.000208	0.000599
ENSG00000185739	SRL	5.087098593	10.174197	0	-5.646151512	1.481845729	-3.8102155	0.000139	0.00041
ENSG00000256616	AP002414.2	10.14418442	19.933296	0.355072425	-5.655867974	1.332939169	-4.2431554	2.20E-05	7.13E-05
ENSG00000267690	LDLRAD4-AS1	5.289114954	10.57823	0	-5.700358031	1.479682655	-3.8524193	0.000117	0.000349
ENSG00000161798	AQP5	5.39869498	10.79739	0	-5.731576812	1.507218241	-3.8027518	0.000143	0.000421
ENSG00000115155	OTOF	5.531971255	11.063943	0	-5.767336043	1.494155514	-3.8599302	0.000113	0.000338
ENSG00000163083	INHBB	145.1896403	285.62353	4.755747826	-5.901004685	0.434496264	-13.581255	5.17E-42	7.46E-41
ENSG00000183395	PMCH	6.211761777	12.423524	0	-5.931894575	1.445339359	-4.1041535	4.06E-05	0.000128
ENSG00000065618	COL17A1	161.8619295	318.57633	5.147527949	-5.95219954	0.427635713	-13.918855	4.87E-44	7.31E-43
ENSG00000211764	TRBJ2-1	6.708539617	13.417079	0	-6.046066476	1.411363172	-4.2838488	1.84E-05	5.99E-05
ENSG00000167037	SGSM1	7.356652851	14.713306	0	-6.17764956	1.395161826	-4.427909	9.52E-06	3.19E-05
ENSG00000140873	ADAMTS18	980.9288124	1936.1616	25.69605528	-6.244290694	0.309942189	-20.14663	2.88E-90	8.55E-89
ENSG00000231007	CDC20P1	7.718783099	15.437566	0	-6.249291543	1.400238903	-4.4630181	8.08E-06	2.73E-05
ENSG00000211772	TRBC2	695.44327	1373.6883	17.19819474	-6.314071062	0.244829337	-25.789683	1.16E-146	5.56E-145
ENSG00000275152	CCL16	8.322825086	16.64565	0	-6.354213833	1.383167367	-4.5939588	4.35E-06	1.51E-05
ENSG00000146678	IGFBP1	193.5830545	382.77956	4.386551275	-6.440319988	0.453856616	-14.190208	1.05E-45	1.63E-44
ENSG00000133800	LYVE1	5005.159263	9907.1194	103.1991661	-6.587792117	0.101121116	-65.147541	0	0
ENSG00000101188	NTSR1	19.8849729	39.414873	0.355072425	-6.640156041	1.266512995	-5.2428645	1.58E-07	6.24E-07
ENSG00000267316	AC090409.2	10.63190513	21.26381	0	-6.711954692	1.37002135	-4.8991607	9.62E-07	3.57E-06
ENSG00000102575	ACP5	23.07648104	45.79789	0.355072425	-6.854645704	1.249845368	-5.484395	4.15E-08	1.72E-07
ENSG00000211751	TRBC1	15.50484502	31.00969	0	-7.256554142	1.293969183	-5.6079807	2.05E-08	8.69E-08
ENSG00000124479	NDP	22.74614817	45.492296	0	-7.809300473	1.263123288	-6.1825323	6.31E-10	2.98E-09
ENSG00000136960	ENPP2	26.3295467	52.659093	0	-8.017999028	1.246493892	-6.4324415	1.26E-10	6.23E-10
ENSG00000276409	CCL14	33.20333409	66.406668	0	-8.352650649	1.229824567	-6.7917416	1.11E-11	5.89E-11

Table S3: RNA-Seq analysis of the differentially expressed genes in HPMVECs with or without cytokine treatment (adjusted *p* value < 0.05 and Log2|Fold Change| > 0.5).

EnsemblID	GeneSymbol	baseMean	baseMean_Control	baseMean_TGF-β1 + IL-1β	log2FoldChange	lfcSE	stat	pvalue	padj
ENSG00000163673	DCLK3	653.8876874	0	1307.775375	12.97492662	1.18077791	10.9884564	4.34E-28	6.44E-27
ENSG00000166923	GREM1	629.4743964	0	1258.948793	12.91999216	1.181032273	10.9395759	7.45E-28	1.10E-26
ENSG00000124102	PI3	293.6011142	0.305286	586.8969424	10.85670439	1.18657102	9.14964566	5.71E-20	6.40E-19
ENSG00000276085	CCL3L1	107.9140528	0	215.8281057	10.37635547	1.201404922	8.63685114	5.78E-18	5.94E-17
ENSG00000173432	SAA1	101.8910792	0	203.7821585	10.29334556	1.196127006	8.60556237	7.59E-18	7.76E-17
ENSG00000125730	C3	13801.99136	25.782772	27578.19995	10.07730935	0.438565864	22.9778699	7.76E-117	4.32E-115
ENSG00000116132	PRRX1	155.3874709	0.305286	310.4696559	9.93860633	1.188828068	8.3600031	6.27E-17	6.14E-16
ENSG00000178860	MSC	78.38909229	0	156.7781846	9.915377312	1.203938898	8.23578117	1.78E-16	1.71E-15
ENSG00000125538	IL1B	12430.96866	26.982587	24834.95472	9.850647703	0.155420291	63.3807054	0	0
ENSG00000108700	CCL8	491.1228765	1.2213911	981.0243619	9.711912268	0.727572009	13.3483863	1.21E-40	2.45E-39
ENSG00000277632	CCL3	61.43025466	0	122.8605093	9.562926123	1.205257192	7.93434479	2.12E-15	1.93E-14
ENSG00000115461	IGFBP5	1316.529805	3.3398779	2629.719732	9.551376198	0.426102166	22.415695	2.77E-111	1.48E-109
ENSG00000172137	CALB2	204.6800648	0.5566052	408.8035245	9.450098921	1.030086554	9.17408239	4.55E-20	5.13E-19
ENSG00000182752	PAPPA	1158.928283	3.5557455	2314.300821	9.365099101	0.423998367	22.0875829	4.16E-108	2.11E-106
ENSG00000175445	LPL	52.87572145	0	105.7514429	9.346308448	1.210705148	7.71972306	1.17E-14	1.02E-13
ENSG00000138316	ADAMTS14	98.79685847	0.2513191	197.3423978	9.28443282	1.196148995	7.76193673	8.36E-15	7.38E-14
ENSG00000178776	C5orf46	46.46991825	0	92.9398365	9.161140674	1.215271132	7.53835126	4.76E-14	4.04E-13
ENSG00000174348	PODN	406.8286216	1.4184963	812.2387468	9.118558432	0.653306947	13.9575409	2.83E-44	6.19E-43
ENSG00000041982	TNC	1219.83399	4.5792903	2435.08869	9.057730082	1.932801424	4.68632213	2.78E-06	1.30E-05
ENSG00000163735	CXCL5	7557.145384	30.863863	15083.42691	8.954093743	0.145399941	61.5825129	0	0
ENSG00000184838	PRR16	39.99218595	0	79.9843719	8.944235301	1.221157205	7.32439302	2.40E-13	1.97E-12
ENSG00000164400	CSF2	3800.558221	16.163867	7584.952575	8.907471506	0.20152205	44.200977	0	0
ENSG00000187037	GPR141	34.61923552	0	69.23847103	8.735764752	1.225480667	7.12843947	1.02E-12	8.01E-12
ENSG00000169436	COL22A1	306.5822396	1.5808911	611.5835881	8.707352809	0.656228194	13.268788	3.51E-40	7.05E-39
ENSG00000123610	TNFAIP6	234.0809796	1.1942842	466.9676751	8.641077117	0.733170652	11.7859015	4.61E-32	7.81E-31
ENSG00000236453	AC003092.1	48.82361778	0.305286	97.34194955	8.265366866	1.21236937	6.81753191	9.26E-12	6.89E-11
ENSG00000146938	NLGN4X	24.96173115	0	49.9234623	8.263167599	1.244309188	6.64076716	3.12E-11	2.24E-10
ENSG00000144191	CNGA3	173.4615683	1.2213911	345.7017456	8.205534656	0.740293644	11.084162	1.50E-28	2.25E-27
ENSG00000276070	CCL4L2	21.21835059	0	42.43670118	8.030735632	1.263330913	6.356795	2.06E-10	1.39E-09
ENSG00000170054	SERPINA9	20.14052146	0	40.28104291	7.955011631	1.273454782	6.24679552	4.19E-10	2.77E-09
ENSG00000124875	CXCL6	6653.409168	54.004771	13252.81357	7.94362437	0.430139471	18.4675551	3.77E-76	1.36E-74
ENSG00000108342	CSF3	11087.387	91.20864	22083.56536	7.927525046	0.212968976	37.2238492	2.81E-303	5.16E-301
ENSG00000163734	CXCL3	1177.110582	11.089048	2343.132115	7.720582509	0.242614091	31.8224819	3.16E-222	4.14E-220
ENSG00000136244	IL6	8138.033067	78.631921	16197.43421	7.688450044	0.236325854	32.5332583	3.61E-232	5.06E-230
ENSG00000117594	HSD11B1	16.60326391	0	33.20652783	7.6764249	1.277797258	6.00754529	1.88E-09	1.18E-08
ENSG00000235531	MSC-AS1	16.41401318	0	32.82802635	7.659848866	1.289194103	5.9415792	2.82E-09	1.74E-08
ENSG00000115919	KYNU	201.033369	1.9482415	400.1184964	7.613290765	0.565131125	13.4717244	2.29E-41	4.71E-40
ENSG00000135373	EHF	15.71566507	0	31.43133014	7.596450087	1.280950424	5.93032325	3.02E-09	1.86E-08
ENSG00000230067	HSPD1P6	56.39623843	0.5566052	112.2358717	7.587030042	1.055137963	7.19055736	6.45E-13	5.17E-12
ENSG00000182050	MGAT4C	28.33604646	0.2513191	56.42077378	7.479027979	1.238261107	6.03994419	1.54E-09	9.75E-09
ENSG00000145649	GZMA	13.77113435	0	27.54226869	7.403953888	1.305469569	5.67148715	1.42E-08	8.23E-08
ENSG00000232810	TNF	26.1077831	0.305286	51.91028017	7.358961402	1.241887143	5.92562814	3.11E-09	1.92E-08
ENSG00000227496	AC099066.2	12.44856381	0	24.89712761	7.261849179	1.316265448	5.51700965	3.45E-08	1.94E-07
ENSG00000179826	MRGPRX3	12.43397146	0	24.86794293	7.256913051	1.320511049	5.49553376	3.90E-08	2.18E-07
ENSG00000050730	TNIP3	711.7529303	9.203612	1414.302249	7.241208742	0.267261999	27.0940454	1.16E-161	9.57E-160
ENSG00000203685	STUM	379.3284668	5.1637436	753.4931901	7.241090515	0.369061626	19.6202748	1.04E-85	4.12E-84
ENSG00000168621	GDNF	11.71692267	0	23.43384534	7.170533431	1.33018365	5.39063416	7.02E-08	3.84E-07
ENSG00000113361	CDH6	11.27926176	0	22.55852352	7.116833601	1.342496567	5.30119315	1.15E-07	6.17E-07
ENSG00000081041	CXCL2	3243.653714	46.603845	6440.703583	7.11422192	0.123354354	57.6730507	0	0
ENSG00000155511	GRIA1	59.06595029	0.970072	117.1618286	7.055133655	0.875760967	8.05600377	7.88E-16	7.32E-15
ENSG00000259342	AC025580.1	21.0800588	0.305286	41.85483158	7.048171198	1.255486168	5.61389793	1.98E-08	1.14E-07
ENSG00000115009	CCL20	453.2498788	7.5799129	898.9198447	6.940057385	0.304641602	22.7810559	7.07E-115	3.90E-113
ENSG00000261040	WFDC21P	9.891696096	0	19.78339219	6.929058962	1.35640476	5.10840065	3.25E-07	1.67E-06
ENSG00000222000	AC092675.1	53.24369616	0.8889981	105.5983942	6.910983543	0.875743978	7.89155702	2.98E-15	2.69E-14
ENSG00000172986	GXYLT2	19.04246891	0.2513191	37.83361868	6.901839466	1.263403808	5.46289272	4.68E-08	2.60E-07
ENSG00000170209	ANKK1	33.48833203	0.5026383	66.4740258	6.8404566	1.081654084	6.32407042	2.55E-10	1.72E-09
ENSG00000172061	LRRC15	33.11220636	0.5837121	65.6407006	6.811019058	1.079547627	6.3091418	2.81E-10	1.89E-09
ENSG00000170743	SYT9	500.8954911	9.0599796	992.7310027	6.775041875	0.276657831	24.4888852	1.94E-132	1.25E-130
ENSG00000170419	VSTM2A	8.714397162	0	17.42879432	6.747822025	1.372622835	4.91600595	8.83E-07	4.37E-06
ENSG00000259354	AC025580.2	8.654745088	0	17.30949018	6.73442647	1.365185785	4.93297436	8.10E-07	4.03E-06
ENSG00000184937	WT1	8.449821409	0	16.89964282	6.700338938	1.372182765	4.8829785	1.04E-06	5.13E-06
ENSG00000166920	C15orf48	316.8366585	6.3580277	627.3152892	6.6715686	0.336216315	19.8430841	1.26E-87	5.14E-86
ENSG00000248371	LINC02056	44.32197198	0.942965	87.70097893	6.636568162	0.888493408	7.46946247	8.05E-14	6.80E-13
ENSG00000163739	CXCL1	30806.71167	625.11203	60988.3113	6.608877121	0.233067569	28.3560563	7.05E-177	6.41E-175
ENSG00000132185	FCRLA	7.805663582	0	15.61132716	6.588416766	1.389649868	4.74106242	2.13E-06	1.01E-05
ENSG00000178882	RFLNA	42.27314976	0.9161051	83.63019442	6.571566073	0.888630404	7.39516231	1.41E-13	1.18E-12
ENSG00000188064	WNT7B	14.92119358	0.305286	29.53710115	6.545186464	1.286513365	5.08753865	3.63E-07	1.86E-06
ENSG00000228741	AL445985.1	13.91028797	0.2513191	27.5692568	6.446446466	1.311668098	4.91469334	8.89E-07	4.40E-06
ENSG00000145824	CXCL14	6.922072025	0	13.84414405	6.413499038	1.423581855	4.50518459	6.63E-06	2.97E-05
ENSG00000128342	LIF	457.6915856	11.054832	904.3283397	6.382554394	0.260997107	24.4545025	4.51E-132	2.88E-130
ENSG00000145777	TSLP	6.748731708	0	13.49746342	6.376426866	1.409696074	4.52326355	6.09E-06	2.74E-05
ENSG00000142149	HUNK	48.46208851	1.1671772	95.75699983	6.355542936	0.777076211	8.17878973	2.87E-16	2.72E-15
ENSG00000182326	C1S	218.9398348	5.6043174	432.2753522	6.353588422	0.368105442	17.2602404	9.37E-67	3.00E-65
ENSG00000119946	CNNM1	12.93305763	0.305286	25.56082925	6.335231658	1.303202302	4.86128028	1.17E-06	5.69E-06
ENSG00000106236	NPTX2	1880.460193	45.95476	3714.965626	6.322139559	0.125839088	50.2398711	0	0
ENSG00000245648	AC022075.1	6.363788895	0	12.72757779	6.292094431	1.419930521	4.43126923	9.37E-06	4.12E-05
ENSG00000134339	SAA2	12.23115325	0.332393	24.12991351	6.255180702	1.317332992	4.74836715	2.05E-06	9.72E-06
ENSG00000164093	PITX2	22.46476597	0.5566052	44.37292678	6.249953018	1.10922258	5.63453461	1.76E-08	1.01E-07
ENSG00000250771	AC106865.1	6.142836058	0	12.28567212	6.242331893	1.462011664	4.26968679	1.96E-05	8.25E-05
ENSG00000140379	BCL2A1	5.838662365	0	11.67732473	6.16631743	1.449173759	4.25505733	2.09E-05	8.79E-05

ENSG00000169429	CXCL8	46345.93334	1283.3344	91408.53225	6.154591783	0.252848841	24.3409927	7.22E-131	4.53E-129
ENSG00000259721	AC090877.2	5.723638358	0	11.44727672	6.142244686	1.473042718	4.16976684	3.05E-05	0.000125
ENSG00000277586	NEFL	11.16793933	0.2513191	22.08455954	6.125404683	1.330679313	4.60321629	4.16E-06	1.91E-05
ENSG00000276980	AC008760.2	5.666776548	0	11.3335531	6.122872375	1.51557373	4.03996998	5.35E-05	0.000213
ENSG00000108688	CCL7	11.14437055	0.2513191	22.03742197	6.122774031	1.341110523	4.56545074	4.98E-06	2.27E-05
ENSG00000130558	OLFM1	681.145793	19.422424	1342.869162	6.119976359	0.19747066	30.9918261	6.95E-211	8.31E-209
ENSG00000087116	ADAMTS2	5.646740102	0	11.2934802	6.118401397	1.487339783	4.1136541	3.89E-05	0.000158
ENSG00000115232	ITGA4	2249.925713	67.0984	4432.753026	6.031905227	0.166105353	36.3137316	9.82E-289	1.75E-286
ENSG00000133048	CHI3L1	10.44992868	0.2513191	20.64853823	6.031574111	1.362721245	4.42612466	9.59E-06	4.21E-05
ENSG00000189292	ALKAL2	10.39096043	0.332393	20.44952788	6.011898456	1.359043375	4.42362515	9.71E-06	4.25E-05
ENSG00000275302	CCL4	5.059545136	0	10.11909027	5.96091976	1.476835414	4.03627899	5.43E-05	0.000216
ENSG00000184530	C6orf58	36.17760631	1.0052765	71.34993611	5.95558712	0.815549365	7.30254645	2.82E-13	2.30E-12
ENSG00000166922	SCG5	239.0215647	7.776277	470.2668524	5.947841597	0.312286625	19.0460978	7.08E-81	2.67E-79
ENSG00000113645	WWC1	4.858510008	0	9.717020015	5.903123865	1.490107405	3.96154253	7.45E-05	0.000289
ENSG00000138944	SHISAL1	43.5834608	1.3645294	85.80239217	5.885573674	0.719200977	8.18348955	2.76E-16	2.62E-15
ENSG00000166523	CLEC4E	4.664034957	0	9.328069913	5.844596841	1.504384476	3.88504198	0.000102	0.000389
ENSG00000224594	RPL29P19	4.658810642	0	9.317621285	5.843186564	1.520414235	3.84315434	0.000121	0.000457
ENSG00000170961	HAS2	4.619093964	0	9.238187928	5.832806544	1.530734277	3.81046314	0.000139	0.000517
ENSG00000128285	MCHR1	8.734246635	0.2513191	17.21717414	5.766214202	1.366542107	4.2195657	2.45E-05	0.000102
ENSG00000105246	EBI3	268.0806398	9.9403861	526.2208936	5.743345085	0.468423792	12.2610021	1.47E-34	2.63E-33
ENSG00000073756	PTGS2	4657.548612	171.42758	9143.669649	5.740925949	0.250650329	22.9041229	4.23E-116	2.34E-114
ENSG00000166394	CYB5R2	31.40301507	1.1942842	61.61174598	5.716624443	0.807212307	7.0819342	1.42E-12	1.11E-11
ENSG00000123572	NRK	8.246182944	0.2513191	16.24104676	5.68512846	1.396177635	4.07192346	4.66E-05	0.000187
ENSG00000163121	NEURL3	99.88824421	3.9154925	195.8609959	5.683712988	0.448078173	12.684646	7.19E-37	1.35E-35
ENSG00000227987	AC092675.2	4.121662245	0	8.24332449	5.665216148	1.55749413	3.63739165	0.000275	0.000977
ENSG00000178538	CA8	188.6753324	7.2014036	370.1492612	5.664996405	0.324994081	17.4310756	4.79E-68	1.57E-66
ENSG00000254143	AC022733.2	4.097855986	0	8.195711972	5.658145051	1.588173738	3.56267385	0.000367	0.001274
ENSG00000184374	COLEC10	125.3746074	5.0553157	245.6938992	5.624090516	0.404378229	13.9079953	5.66E-44	1.23E-42
ENSG00000126549	STATH	7.914582626	0.305286	15.52387923	5.61953701	1.417137337	3.96541455	7.33E-05	0.000285
ENSG00000003989	SLC7A2	10385.87717	415.01252	20356.74181	5.618888844	0.050632194	110.974627	0	0
ENSG00000197520	FAM177B	3.945769139	0	7.891538278	5.601555057	1.554252683	3.60401826	0.000313	0.0011
ENSG00000235885	LINC01828	3.937873299	0	7.875746597	5.59915759	1.5597641	3.58974642	0.000331	0.001157
ENSG00000087258	GNAO1	3.933866009	0	7.867732019	5.597949527	1.554526234	3.60106469	0.000317	0.001112
ENSG00000137203	TFAP2A	65.29828084	2.7209613	127.8756004	5.596580015	0.545645368	10.2568084	1.10E-24	1.47E-23
ENSG00000084636	COL16A1	187.442876	8.0007362	366.8850158	5.535598907	0.315927457	17.5217405	9.78E-69	3.25E-67
ENSG00000120708	TGFBI	8606.006436	365.55173	16846.46114	5.526786434	0.246968865	22.3784745	6.38E-111	3.36E-109
ENSG00000168685	IL7R	192.4529989	8.7118856	376.1941122	5.508727662	0.316536093	17.4031581	7.81E-68	2.55E-66
ENSG00000167613	LAIR1	3.687889888	0	7.375779777	5.508462338	1.606299316	3.42928761	0.000605	0.002026
ENSG00000100092	SH3BP1	3.683882599	0	7.367765199	5.506741601	1.655681069	3.32596761	0.000881	0.002854
ENSG00000134532	SOX5	7.206497252	0.2513191	14.16167537	5.485301119	1.427056981	3.84378563	0.000121	0.000456
ENSG00000042062	RIPOR3	32.62196795	1.4187434	63.82519255	5.454436312	0.745504763	7.31643389	2.55E-13	2.08E-12
ENSG00000243742	RPLP0P2	7.039005648	0.305286	13.77272527	5.445515827	1.41171646	3.8573722	0.000115	0.000433
ENSG00000183813	CCR4	319.164487	14.420828	623.9081457	5.443637236	0.236558767	23.0117755	3.55E-117	1.98E-115
ENSG00000180921	FAM83H	7.020542441	0.305286	13.73579886	5.441909153	1.463252324	3.71905041	0.0002	0.000725
ENSG00000137462	TLR2	1209.494975	54.82119	2364.16876	5.440785073	0.123060363	44.2123274	0	0
ENSG00000077943	ITGA8	115.0180683	5.3610958	224.6750408	5.412459998	0.38953963	13.8945041	6.84E-44	1.48E-42
ENSG00000152463	OLAH	31.07530901	1.3376695	60.81294852	5.398055719	0.756251616	7.13790967	9.48E-13	7.50E-12
ENSG00000179761	PIPOX	75.02027089	3.312771	146.7277708	5.396320591	0.480726822	11.225337	3.06E-29	4.74E-28
ENSG00000224173	AC007422.1	3.295051235	0	6.59010247	5.347240907	1.68803513	3.16773082	0.001536	0.00475
ENSG00000179362	HMGN2P46	12.03024096	0.610572	23.44990987	5.316991981	1.175486808	4.52322557	6.09E-06	2.74E-05
ENSG00000154451	GBP5	383.297298	18.334495	748.2601013	5.307723453	0.211150463	25.1371623	1.95E-139	1.35E-137
ENSG00000108821	COL1A1	41.31984434	1.9753485	80.66434016	5.307328572	0.628435921	8.44529792	3.03E-17	3.01E-16
ENSG00000273003	ARL2-SNX15	3.197136985	0	6.394273969	5.297837589	1.652371332	3.2062028	0.001345	0.004206
ENSG00000277147	LINC00869	3.193129696	0	6.386259391	5.296298809	1.62993649	3.24938968	0.001157	0.003668
ENSG00000237604	AP001056.1	3.17855504	0	6.35711008	5.29083746	1.648095133	3.21027431	0.001326	0.004155
ENSG00000027869	SH2D2A	28.38211742	1.3645294	55.39970541	5.260973667	0.769454568	6.83727654	8.07E-12	6.03E-11
ENSG00000171631	P2RY6	40.10802072	2.1101422	78.10589922	5.24620735	0.634420369	8.2692921	1.35E-16	1.30E-15
ENSG00000272419	LINC01145	11.15568993	0.5837121	21.72766775	5.216947192	1.178320596	4.42744293	9.54E-06	4.19E-05
ENSG00000124391	IL17C	2.974848385	0	5.949696771	5.196476447	1.722675183	3.01651553	0.002557	0.007537
ENSG00000163017	ACTG2	2.960392467	0	5.920784935	5.190475075	1.726473625	3.00640276	0.002644	0.007772
ENSG00000007908	SELE	14723.72254	792.84449	28654.6006	5.178136291	0.083901983	61.716495	0	0
ENSG00000131459	GFPT2	1245.049983	67.027991	2423.071974	5.161953344	0.11625279	44.4028341	0	0
ENSG00000182901	RGS7	42.65521936	2.3074945	83.00294425	5.152123796	0.59588138	8.64622384	5.32E-18	5.49E-17
ENSG00000256340	ABCC6P1	2.847903561	0	5.695807122	5.128848608	1.7200157	2.98186151	0.002865	0.008357
ENSG00000175093	SPSB4	2.831993142	0	5.663986284	5.12193479	1.693172264	3.02505238	0.002486	0.007351
ENSG00000176907	TCIM	1491.582559	83.834574	2899.330543	5.119460044	0.104814566	48.8430211	0	0
ENSG00000229292	RFPL4AL1	2.789723676	0	5.579447351	5.103872998	1.696223096	3.00896327	0.002621	0.007713
ENSG00000112303	VNN2	2.768588942	0	5.537177885	5.094393785	1.754638223	2.90338699	0.003692	0.010515
ENSG00000105963	ADAP1	49.70927926	2.648232	96.77032647	5.072429457	0.547836809	9.25901542	2.06E-20	2.36E-19
ENSG00000274265	AC245297.3	5.404306262	0.332393	10.47621954	5.04960548	1.451062802	3.47993586	0.000502	0.001704
ENSG00000103522	IL21R	5.40041771	0.332393	10.46844243	5.048679005	1.475619393	3.42139649	0.000623	0.002081
ENSG00000279668	AC024610.2	2.668003166	0	5.336006332	5.034077575	1.779560303	2.82883225	0.004672	0.012974
ENSG00000138378	STAT4	98.63634461	5.890841	191.3818482	5.031929721	0.382030641	13.1715344	1.28E-39	2.53E-38
ENSG00000251533	LINC00605	2.624397936	0	5.248795873	5.013822035	1.775844754	2.82334479	0.004753	0.013188
ENSG00000038427	VCAN	248.0318851	14.949833	481.1139378	5.008174568	0.483195575	10.3646946	3.59E-25	4.89E-24
ENSG00000144868	TMEM108	2.609823281	0	5.219646561	5.007141014	1.722185495	2.9074342	0.003644	0.010395
ENSG00000120658	ENOX1	38.09280598	2.1455938	74.04001818	5.004685216	0.610205287	8.20164184	2.37E-16	2.26E-15
ENSG00000188452	CERKL	2.599255914	0	5.198511828	5.002212849	1.741667378	2.87208276	0.004078	0.011493
ENSG00000280241	AC079298.3	2.595248625	0	5.19049725	5.000374645	1.742034942	2.87042155	0.004099	0.011546
ENSG00000198910	L1CAM	37.99795018	2.1995607	73.79633968	4.993867772	0.610017262	8.18643682	2.69E-16	2.56E-15
ENSG00000083454	P2RX5	5.100150255	0.332393	9.867907524	4.968106451	1.549010901	3.20727662	0.00134	0.004194
ENSG00000080224	EPHA6	9.146816024	0.5026383	17.79099379	4.96218175	1.249321149	3.97190246	7.13E-05	0.000278
ENSG00000062582	MRPS24	14.1230799	0.8889981	27.35716167	4.960054029	0.982421843	5.04880268	4.45E-07	2.26E-06
ENSG00000223804	AC244669.1	2.461624985	0	4.92324997	4.919060426	1.766168734	2.78515882	0.00535	0.014661
ENSG00000127561	SYNGR3	36.71389655	2.4965021	70.93129099	4.903273454	0.615691593	7.96384669	1.67E-15	1.53E-14

ENSG00000261618	LINC02605	48.6967364	3.0885588	94.30491399	4.883957561	0.521900124	9.35803104	8.12E-21	9.44E-20
ENSG00000286062	AC092353.1	17.73979259	1.1671772	34.31240798	4.875099427	0.867208611	5.62159943	1.89E-08	1.09E-07
ENSG00000104722	NEFM	4.669815436	0.305286	9.034344849	4.834393994	1.496742707	3.22994324	0.001238	0.003905
ENSG00000162366	PDZK1IP1	8.708124091	0.664786	16.75146221	4.808447332	1.223299568	3.93071939	8.47E-05	0.000326
ENSG00000258602	LINC01629	2.276500276	0	4.553000551	4.806128851	1.871760622	2.56770486	0.010237	0.026321
ENSG00000127863	TNFRSF19	337.0766818	22.886678	651.2666854	4.805151288	0.19849256	24.2082186	1.82E-129	1.12E-127
ENSG00000248362	AC011352.1	2.265814171	0	4.531628342	4.800382644	1.87321188	2.56264798	0.010388	0.026663
ENSG00000127928	GNGT1	2.263261383	0	4.526522766	4.798791992	1.815488188	2.64325156	0.008211	0.021666
ENSG00000104415	CCN4	2.259254094	0	4.518508188	4.79660778	1.789112967	2.68099772	0.00734	0.019558
ENSG00000160223	ICOSLG	507.9631122	35.116784	980.80944	4.785826625	0.160206618	29.8728397	4.44E-196	4.83E-194
ENSG00000272502	AC104958.2	2.228887757	0	4.457775514	4.780258732	1.840594864	2.59712706	0.009401	0.024384
ENSG00000138311	ZNF365	33.15901575	2.3074945	64.01053704	4.776296548	0.626389813	7.62511849	2.44E-14	2.10E-13
ENSG00000133107	TRPC4	2.215648864	0	4.431297728	4.773166483	1.821700202	2.62017124	0.008789	0.022983
ENSG00000168334	XIRP1	2.215648864	0	4.431297728	4.773166483	1.821700202	2.62017124	0.008789	0.022983
ENSG00000112299	VNN1	12.59850771	0.942965	24.25405039	4.770169017	1.003601011	4.75305322	2.00E-06	9.51E-06
ENSG00000204291	COL15A1	92.66160191	6.4284368	178.8947671	4.745957548	0.369383976	12.8483038	8.79E-38	1.68E-36
ENSG00000080573	COL5A3	24.51784188	1.8319632	47.20372059	4.738068983	0.713222324	6.64318659	3.07E-11	2.20E-10
ENSG00000205436	EXOC3L4	4.329444379	0.2513191	8.407569629	4.737234482	1.587536116	2.98401683	0.002845	0.008305
ENSG00000164761	TNFRSF11B	1007.302685	74.283459	1940.321911	4.717570477	0.232239973	20.3133441	9.80E-92	4.22E-90
ENSG00000096996	IL12RB1	27.53279957	1.9753485	53.09025063	4.707391023	0.664215301	7.08714631	1.37E-12	1.07E-11
ENSG00000165801	ARHGEF40	220.9050878	16.315103	425.4950725	4.701328391	0.240514329	19.5469783	4.38E-85	1.73E-83
ENSG00000285744	AC083837.1	189.4800452	14.143143	364.816947	4.698940364	0.255896224	18.3626796	2.61E-75	9.38E-74
ENSG00000205334	LINC01460	2.060890491	0	4.121780983	4.66521909	1.88126647	2.47982897	0.013145	0.033035
ENSG00000126550	HTN1	2.047651599	0	4.095303197	4.657495638	1.848547988	2.51954273	0.011751	0.029824
ENSG00000255491	AC100858.3	2.046315836	0	4.092631671	4.656745133	1.840135209	2.53065379	0.011385	0.028985
ENSG00000159403	C1R	295.9287764	23.006018	568.8515349	4.638616849	0.203857526	22.7542095	1.30E-114	7.17E-113
ENSG00000136688	IL36G	2.01194221	0	4.023884419	4.635909906	1.919402527	2.41528801	0.015723	0.038775
ENSG00000258788	CKS1BP1	18.23144379	1.3645294	35.09835814	4.611143156	0.838299731	5.50059005	3.79E-08	2.12E-07
ENSG00000276600	RAB7B	22.00736966	1.8319632	42.18277615	4.572650726	0.734296498	6.22725389	4.75E-10	3.13E-09
ENSG00000112096	SOD2	62752.87933	5120.4071	120385.3515	4.555470087	0.033395833	136.40834	0	0
ENSG00000227165	WDR11-AS1	1.903460593	0	3.806921185	4.547065	1.947413472	2.33492531	0.019547	0.04696
ENSG00000203364	AL390760.1	1.899453303	0	3.798906607	4.544382886	1.921928055	2.36449168	0.018055	0.043761
ENSG00000013293	SLC7A14	59.79759693	4.7583743	114.8368196	4.543058423	0.438524967	10.3598626	3.78E-25	5.13E-24
ENSG00000134775	FHOD3	24.57609981	1.9482415	47.20395806	4.541275461	0.676423214	6.71365998	1.90E-11	1.39E-10
ENSG00000263424	AC110597.3	1.891557463	0	3.783114926	4.539268821	1.91272194	2.37319849	0.017635	0.042834
ENSG00000163082	SGPP2	1.888885937	0	3.777771874	4.537507622	1.891675443	2.39867131	0.016455	0.040279
ENSG00000183570	PCBP3	1.865079677	0	3.730159355	4.522317999	1.89617941	2.38496314	0.017081	0.041667
ENSG00000138675	FGF5	715.9384789	59.386013	1372.490945	4.515151968	0.132157743	34.164869	8.04E-256	1.28E-253
ENSG00000155897	ADCY8	13.8189744	1.1403173	26.49763154	4.510446431	0.89734589	5.02643014	5.00E-07	2.53E-06
ENSG00000185269	NOTUM	3.714805101	0.305286	7.124324179	4.492733799	1.560308259	2.87938859	0.003984	0.011265
ENSG00000144802	NFKBIZ	4552.427009	396.63325	8708.220772	4.466239807	0.059216596	75.422096	0	0
ENSG00000143340	FAM163A	13.83218576	1.275358	26.38901349	4.465486512	0.906273337	4.92730651	8.34E-07	4.14E-06
ENSG00000278925	AC005753.1	3.596603906	0.2513191	6.941888682	4.460320662	1.724049131	2.58711923	0.009678	0.025029
ENSG00000234380	LINC01426	6.38759511	0.5026383	12.27255196	4.439387456	1.312382887	3.38269228	0.000718	0.00237
ENSG00000226067	LINC00623	3.53707057	0.2513191	6.822822012	4.427704236	1.610564294	2.74916329	0.005975	0.016202
ENSG00000006210	CX3CL1	3253.903598	293.11101	6214.696187	4.40800691	0.064516716	68.3234855	0	0
ENSG00000183760	ACP7	19.49967668	1.8588231	37.14053026	4.381890512	0.770573999	5.68652786	1.30E-08	7.56E-08
ENSG00000118503	TNFAIP3	1362.190809	127.39474	2596.986877	4.358969283	0.091953729	47.4039425	0	0
ENSG00000105855	ITGB8	2723.190474	253.85106	5192.529887	4.358377851	0.066761617	65.2826886	0	0
ENSG00000104043	ATP8B4	3.342080007	0.332393	6.351767027	4.328029555	1.660199607	2.60693325	0.009136	0.023778
ENSG00000204961	PCDHA9	3.338191456	0.332393	6.343989925	4.326652613	1.597184274	2.70892513	0.00675	0.018134
ENSG00000162551	ALPL	42.7085629	3.977557	81.43956884	4.323232048	0.492405368	8.77982314	1.64E-18	1.73E-17
ENSG00000110848	CD69	388.8640775	37.246924	740.4812307	4.308673606	0.167231586	25.7647117	2.21E-146	1.62E-144
ENSG00000069482	GAL	123.5415449	11.951433	235.1316566	4.295894821	0.295180791	14.5534363	5.55E-48	1.31E-46
ENSG00000277117	FP565260.3	1330.248048	130.95992	2529.536178	4.285298355	0.093843268	45.6644197	0	0
ENSG00000075223	SEMA3C	250.071028	24.981613	475.1604427	4.255539862	0.202519841	21.0129528	4.99E-98	2.29E-96
ENSG00000116147	TNR	48.9458542	4.6504405	93.24126792	4.253987791	0.469963428	9.0517422	1.41E-19	1.55E-18
ENSG00000205057	CLLU1OS	17.62722145	1.7779963	33.47644663	4.251632002	0.758862797	5.60263597	2.11E-08	1.21E-07
ENSG00000177234	LINC01561	3.135000313	0.2513191	6.018681498	4.248856175	1.767076478	2.40445517	0.016197	0.039755
ENSG00000267095	AC025048.1	3.143401816	0.305286	5.981517608	4.241298299	1.613928384	2.62793464	0.008591	0.022533
ENSG00000142661	MYOM3	5.960355293	0.637679	11.28303158	4.239267438	1.294221006	3.27553595	0.001055	0.003371
ENSG00000164251	F2RL1	5020.667034	510.61155	9530.722522	4.225056282	0.113214969	37.3188838	8.11E-305	1.51E-302
ENSG00000168398	BDKRB2	840.5838816	85.60778	1595.559983	4.21607106	0.113921776	37.0084739	8.37E-300	1.52E-297
ENSG00000143226	FCGR2A	5.499996264	0.5026383	10.49735427	4.215904331	1.343120319	3.13888806	0.001696	0.005199
ENSG00000170075	GPR37L1	5.85232469	0.664786	11.03986341	4.191329366	1.349102741	3.10675328	0.001892	0.005736
ENSG00000103044	HAS3	452.5644775	46.953669	858.1752865	4.183766656	0.155433261	26.9168042	1.40E-159	1.13E-157
ENSG00000101670	LIPG	11913.24926	1246.4834	22580.0151	4.181957075	0.040006232	104.53264	0	0
ENSG00000003987	MTMR7	28.13435888	3.1073212	53.16139656	4.157803926	0.608319866	6.8348975	8.21E-12	6.12E-11
ENSG00000155307	SAMSN1	24.91988041	2.6941014	47.14565944	4.1560846	0.620609936	6.69677418	2.13E-11	1.55E-10
ENSG00000258183	LINC02392	2.958591695	0.332393	5.584790404	4.143223751	1.69331513	2.44681198	0.014413	0.035899
ENSG00000163874	ZC3H12A	1049.481927	112.44599	1986.51786	4.142188958	0.192350608	21.5345769	7.39E-103	3.53E-101
ENSG00000163888	CAMK2N2	10.65036233	1.0863504	20.21437428	4.138194285	0.949188796	4.35971674	1.30E-05	5.61E-05
ENSG00000073282	TP63	2.890241218	0.2513191	5.529163307	4.131412268	1.680519077	2.45841438	0.013955	0.034875
ENSG00000267607	AC011511.5	8.164223865	0.9158581	15.41258966	4.113948802	1.114638548	3.69083665	0.000224	0.000805
ENSG00000231133	HAR1B	7.833914513	0.8079243	14.85990475	4.113416829	1.105408234	3.7211744	0.000198	0.00072
ENSG00000162692	VCAM1	2128.724917	237.02399	4020.425844	4.096804206	0.079397546	51.5986253	0	0
ENSG00000229656	ITGB1-DT	72.88897294	7.928254	137.8496919	4.087363031	0.36764167	11.1177904	1.03E-28	1.56E-27
ENSG00000232702	AL158050.1	7.531787947	0.7539574	14.30961851	4.08362357	1.137459896	3.59012532	0.000331	0.001156
ENSG00000230882	AC005077.4	2.806071522	0.305286	5.30685702	4.065001395	1.683213964	2.41502357	0.015734	0.038793
ENSG00000108309	RUNDC3A	2.784936788	0.305286	5.264587554	4.055492542	1.665082799	2.43561014	0.014867	0.036904
ENSG00000180914	OXTR	30.83962069	3.6370664	58.04217496	4.033686839	0.561913501	7.17848357	7.05E-13	5.62E-12
ENSG00000167034	NKX3-1	201.0121145	23.466342	378.5578868	4.022395515	0.439153157	9.15943663	5.22E-20	5.86E-19
ENSG00000182162	P2RY8	4.998160481	0.5837121	9.412608846	4.009638319	1.357090925	2.9545834	0.003131	0.009056
ENSG00000196358	NTNG2	10.30217678	1.302465	19.30188858	4.000908002	0.972595334	4.11364096	3.89E-05	0.000158
ENSG00000198535	C2CD4A	124.9212755	14.995702	234.8468492	3.977368146	0.518325756	7.67349123	1.67E-14	1.46E-13

ENSG00000185338	SOCS1	217.9212263	26.094824	409.7476289	3.976314979	0.204355817	19.4578018	2.50E-84	9.79E-83
ENSG00000183775	KCTD16	9.995212824	1.302465	18.68796066	3.955346875	0.957447343	4.13113776	3.61E-05	0.000147
ENSG00000103154	NECAB2	4.754541297	0.5566052	8.952477442	3.954664957	1.358991351	2.91000009	0.003614	0.010316
ENSG00000166793	YPEL4	4.824938901	0.5837121	9.066165687	3.954603277	1.362091858	2.90333082	0.003692	0.010515
ENSG00000159556	ISL2	2.569327004	0.305286	4.833367985	3.935332864	1.700558701	2.31414115	0.02066	0.049225
ENSG00000221869	CEBPD	1240.861083	153.18453	2328.537639	3.933204182	0.096234029	40.871241	0	0
ENSG00000149633	KIAA1755	7.053552721	0.942965	13.16414041	3.875108779	1.121086555	3.45656521	0.000547	0.001847
ENSG00000179148	ALOXE3	11.38433696	1.4724632	21.29621071	3.859656293	0.910689999	4.23816699	2.25E-05	9.43E-05
ENSG00000140092	FBLN5	65.32091812	8.3949466	122.2468896	3.854591279	0.388371534	9.92500981	3.24E-23	4.10E-22
ENSG00000187123	LYPD6	161.3117438	21.227775	301.3957131	3.836532948	0.229904969	16.6874729	1.62E-62	4.88E-61
ENSG00000164690	SHH	6.556463129	0.8350312	12.27789501	3.830123472	1.14118177	3.35627818	0.00079	0.002585
ENSG00000155966	AFF2	18.03491699	2.4425352	33.62729877	3.826481021	0.698154848	5.4808486	4.23E-08	2.36E-07
ENSG00000148680	HTR7	4.361702644	0.5566052	8.166800136	3.825102759	1.3968268	2.73842309	0.006173	0.016698
ENSG00000148848	ADAM12	174.2727001	23.128665	325.4167357	3.811409204	0.570222878	6.68406925	2.32E-11	1.68E-10
ENSG00000197859	ADAMTSL2	10.72266955	1.4187434	20.02659574	3.793588786	0.9122565	4.15846726	3.20E-05	0.000131
ENSG00000175841	FAM172BP	6.312725207	0.8079243	11.81752613	3.79259424	1.201495334	3.15656177	0.001596	0.004917
ENSG00000167207	NOD2	16.89895823	2.3267509	31.47116555	3.791531071	1.608624647	2.35700173	0.018423	0.04455
ENSG00000026559	KCNG1	23.8874802	3.2507065	44.52425387	3.784744008	0.628009474	6.02657151	1.67E-09	1.06E-08
ENSG00000064309	CDON	800.1068174	108.14222	1492.071417	3.769274126	0.112605338	33.4733165	1.18E-245	1.79E-243
ENSG00000121898	CPXM2	4.042329895	0.5026383	7.582021533	3.759592096	1.466914332	2.56292547	0.010379	0.026649
ENSG00000100767	PAPLN	3649.612953	505.66619	6793.559715	3.751355524	0.058679244	63.9298544	0	0
ENSG00000273812	BX640514.2	27.54437759	3.8339246	51.25483059	3.748039692	0.610791409	6.1363661	8.44E-10	5.46E-09
ENSG00000278709	NKILA	12.53736463	1.8048562	23.26987305	3.715074553	0.814737398	4.55984292	5.12E-06	2.32E-05
ENSG00000115008	IL1A	763.1985191	107.29149	1419.105553	3.712901249	0.110387768	33.6350785	5.15E-248	8.01E-246
ENSG00000177875	CCDC184	6.437837622	0.997179	11.87849628	3.698628265	1.178376507	3.13874916	0.001697	0.005199
ENSG00000113739	STC2	4125.869953	591.33287	7660.407037	3.696068036	0.159980353	23.1032621	4.29E-118	2.40E-116
ENSG00000151617	EDNRA	5.789764542	0.7539574	10.8255717	3.695886388	1.221968508	3.02453489	0.00249	0.007362
ENSG00000173114	LRRN3	7.779673683	1.0592434	14.50010396	3.677271375	1.030552646	3.56825184	0.000359	0.001249
ENSG00000010319	SEMA3G	3197.249597	470.73822	5923.760974	3.652324211	0.059562672	61.3190125	0	0
ENSG00000269800	PLEKHA3P1	7.975771548	1.2213911	14.73015197	3.633555298	1.006463474	3.61022073	0.000306	0.001075
ENSG00000106003	LFNG	313.3515248	47.258461	579.4445891	3.608882849	0.164597617	21.9254866	1.48E-106	7.44E-105
ENSG00000136167	LCP1	57.33209541	8.6738668	105.990324	3.602602981	0.389277631	9.25458515	2.15E-20	2.45E-19
ENSG00000173093	CCDC63	5.445755433	0.7539574	10.13755348	3.599807199	1.224602267	2.93957254	0.003287	0.009482
ENSG00000241280	AC106712.1	3.899583539	0.637679	7.161488069	3.57030248	1.438334453	2.48224776	0.013056	0.032829
ENSG00000143333	RGS16	24.8867826	3.9692124	45.80435284	3.568839162	0.569444592	6.26722813	3.68E-10	2.44E-09
ENSG00000101187	SLCO4A1	397.2585897	62.659532	731.8576477	3.551929189	0.141359682	25.1268901	2.53E-139	1.73E-137
ENSG00000198286	CARD11	228.3394148	36.447441	420.2313881	3.537865175	0.204465076	17.3030292	4.46E-67	1.43E-65
ENSG00000231574	LINC02015	7.519724547	1.2484981	13.790951	3.528303789	1.060445596	3.32718982	0.000877	0.002843
ENSG00000124143	ARHGAP40	17.85819068	2.8643467	32.85203472	3.499235625	0.676476148	5.17274059	2.31E-07	1.20E-06
ENSG00000163898	LIPH	7.088705901	1.1403173	13.03709454	3.492122753	1.027841187	3.39753144	0.00068	0.002257
ENSG00000028277	POU2F2	3523.257879	577.62354	6468.892215	3.484032431	0.052582028	66.2589965	0	0
ENSG00000283265	AL356234.3	7.306786288	1.2484981	13.36507449	3.482504161	1.078711572	3.22839233	0.001245	0.003924
ENSG00000006016	CRLF1	7.085323012	1.1942842	12.97636186	3.463950619	1.035995177	3.34359724	0.000827	0.002694
ENSG00000163814	CDCP1	105.4305154	17.698642	193.1623889	3.457019794	0.271134156	12.7502187	3.11E-37	5.89E-36
ENSG00000171533	MAP6	54.08533984	9.0328727	99.13780702	3.452711049	0.379610495	9.09540463	9.42E-20	1.05E-18
ENSG00000042980	ADAM28	33.88810749	5.9635703	61.81264469	3.436623504	0.494883259	6.9443115	3.80E-12	2.90E-11
ENSG00000174939	ASPHD1	65.55388724	11.305163	119.8026119	3.421249734	0.353339138	9.6826232	3.57E-22	4.36E-21
ENSG00000169245	CXCL10	88.17344821	15.472221	160.8746752	3.394559808	0.303709852	11.1769828	5.29E-29	8.09E-28
ENSG00000049249	TNFRSF9	61.47279227	10.757396	112.1881884	3.376800582	0.360931565	9.35579182	8.30E-21	9.63E-20
ENSG00000271503	CCL5	50.73225194	8.7630382	92.70146569	3.370989135	0.384676864	8.76317098	1.90E-18	2.00E-17
ENSG00000260549	MT1L	1561.744997	277.91942	2845.56937	3.355939565	0.376469172	8.914248	4.91E-19	5.26E-18
ENSG00000159261	CLDN14	756.4122382	135.9582	1376.866272	3.345169543	0.10760837	31.0865181	3.66E-212	4.41E-210
ENSG00000058866	DGKG	17.97717449	3.2504595	32.70388948	3.341159361	0.658793101	5.07163684	3.94E-07	2.02E-06
ENSG00000198848	CES1	6.599985495	1.2211441	11.9788269	3.328408353	1.101596291	3.02144114	0.002516	0.00743
ENSG00000151023	ENKUR	12.84965869	2.3077415	23.39157587	3.328221573	0.76269876	4.36374326	1.28E-05	5.52E-05
ENSG00000174938	SEZ6L2	346.7394836	62.503655	630.9753118	3.322763721	0.152159205	21.8374152	1.02E-105	5.06E-104
ENSG00000181072	CHRM2	8.007660576	1.4727103	14.5426109	3.312413673	0.973554038	3.40239323	0.000668	0.002219
ENSG00000124145	SDC4	6949.500607	1272.3827	12626.61854	3.310728009	0.040846924	81.0520761	0	0
ENSG00000087494	PTHLH	12.54220198	2.2535276	22.83087638	3.305258381	0.75356238	4.38617753	1.15E-05	5.00E-05
ENSG00000141668	CBLN2	67.58056844	12.716055	122.4450814	3.299156181	0.331968406	9.93816316	2.84E-23	3.60E-22
ENSG00000026508	CD44	12475.25325	2301.6287	22648.87779	3.298804157	0.165281085	19.9587519	1.26E-88	5.18E-87
ENSG00000188693	CYP51A1-AS1	4.805891947	0.9161051	8.695678792	3.285536623	1.246135224	2.63658113	0.008375	0.022044
ENSG00000188112	C6orf132	18.87837732	3.5288856	34.22786905	3.28471409	0.620910188	5.29015975	1.22E-07	6.54E-07
ENSG00000224982	TMEM233	13.86102449	2.5317066	25.19034235	3.278277556	0.748483893	4.37989059	1.19E-05	5.14E-05
ENSG00000104312	RIPK2	4729.060063	884.30173	8573.818392	3.274716058	0.048259489	67.8564178	0	0
ENSG00000124225	PMEPA1	1272.536549	243.83449	2301.238611	3.239412808	0.081670636	39.6643512	0	0
ENSG00000133657	ATP13A3	16075.5815	3076.4442	29074.71883	3.238921789	0.036658059	88.3549729	0	0
ENSG00000178752	ERFE	7.389041547	1.3916364	13.3864467	3.223195506	0.97963729	3.29019275	0.001001	0.003214
ENSG00000111817	DSE	3890.350735	754.70293	7025.998536	3.21984834	0.050893816	63.2660037	0	0
ENSG00000144476	ACKR3	784.4756527	152.33567	1416.615633	3.212080597	0.108715911	29.5456348	7.47E-192	7.81E-190
ENSG00000267577	AC010327.3	50.40505702	10.129888	90.6802262	3.201411162	0.404767789	7.90925376	2.59E-15	2.35E-14
ENSG00000121753	ADGRB2	64.90233777	12.597704	117.2069717	3.199632327	0.331597915	9.64913284	4.96E-22	6.01E-21
ENSG00000135378	PRRG4	45.32207907	8.9796469	81.66451128	3.177509351	0.421237845	7.54326656	4.58E-14	3.90E-13
ENSG00000173110	HSPA6	9.068576702	1.8859301	16.25122254	3.163526152	0.921712372	3.43222707	0.000599	0.002008
ENSG00000204482	LST1	45.04803252	9.1141936	80.98187149	3.156214495	0.387734162	8.14015067	3.95E-16	3.72E-15
ENSG00000090339	ICAM1	28723.10793	5898.5121	51547.7038	3.127148107	0.027248166	114.765451	0	0
ENSG00000185215	TNFAIP2	6106.066605	1269.039	10943.09425	3.108546536	0.103030758	30.1710538	5.68E-200	6.34E-198
ENSG00000197632	SERPINB2	16.67459503	3.4207048	29.9284853	3.106811273	0.662880061	4.68683772	2.77E-06	1.30E-05
ENSG00000117525	F3	128.4864751	26.910598	230.0623518	3.087101025	0.230279868	13.4058659	5.59E-41	1.14E-39
ENSG00000113946	CLDN16	18.75490231	3.8696232	33.64018145	3.073685231	0.622763494	4.93555782	7.99E-07	3.97E-06
ENSG00000087085	ACHE	41.75297227	8.870972	74.63497256	3.053281813	0.404343166	7.55121408	4.31E-14	3.68E-13
ENSG00000108691	CCL2	23552.24899	5088.0769	42016.42107	3.046112786	0.076947361	39.5869688	0	0
ENSG00000165617	DACT1	9.164417359	1.9213816	16.4074531	3.043105075	0.87976358	3.45900324	0.000542	0.001832
ENSG00000067798	NAV3	921.6951169	202.93894	1640.451293	3.015116271	0.263806042	11.4292919	2.99E-30	4.76E-29
ENSG00000156920	ADGRG4	138.6669393	30.438743	246.8951357	3.012012889	0.227663271	13.2301222	5.88E-40	1.17E-38

ENSG00000169583	CLIC3	10.69663816	2.4698892	18.92338711	2.985386456	0.822144913	3.63121684	0.000282	0.001
ENSG00000183762	KREMEN1	102.9743568	22.805357	183.1433564	2.984981091	0.269968392	11.0567799	2.03E-28	3.05E-27
ENSG00000232618	AL355304.1	7.921065708	1.8051032	14.03702818	2.980373666	0.946387246	3.14921157	0.001637	0.005032
ENSG00000138792	ENPEP	8.151167963	1.9132841	14.38905186	2.973892717	0.998828055	2.97738205	0.002907	0.008469
ENSG00000181634	TNFSF15	2989.449171	676.4342	5302.464142	2.970838775	0.332153478	8.94417483	3.75E-19	4.03E-18
ENSG00000258807	AL359237.1	23.74218419	5.5501034	41.93426493	2.961199458	0.542070788	5.46275417	4.69E-08	2.60E-07
ENSG00000151883	PARP8	30.54090331	6.9495904	54.13221627	2.951724019	0.474703367	6.21803894	5.03E-10	3.31E-09
ENSG00000138356	AOX1	239.523799	55.413591	423.634007	2.947443658	0.184460636	15.9787135	1.80E-57	4.99E-56
ENSG00000056558	TRAF1	1524.395718	353.02025	2695.771186	2.934779294	0.075001669	39.1295197	0	0
ENSG00000149591	TAGLN	3312.643376	776.78633	5848.500425	2.912134491	0.125836894	23.1421358	1.74E-118	9.83E-117
ENSG00000173918	C1QTNF1	1757.231939	412.05185	3102.412025	2.911609415	0.082253739	35.3978974	1.84E-274	3.13E-272
ENSG00000109321	AREG	24.19482022	5.8285295	42.5611109	2.904506916	0.5861282	4.95541234	7.22E-07	3.60E-06
ENSG00000134250	NOTCH2	485.0636401	114.65331	855.4739726	2.88975745	0.122565916	23.5771701	6.61E-123	3.85E-121
ENSG00000116711	PLA2G4A	948.8041158	227.36089	1670.247343	2.881461941	0.105838944	27.2249686	3.29E-163	2.77E-161
ENSG00000127585	FBXL16	12.13730909	2.8909595	21.38365864	2.875332192	0.762510567	3.77087521	0.000163	0.000598
ENSG00000197046	SIGLEC15	50.66418607	12.356555	88.97181666	2.872059481	0.372938104	7.70116931	1.35E-14	1.18E-13
ENSG00000078098	FAP	2948.499112	708.75958	5188.238648	2.870597005	0.066793378	42.9772695	0	0
ENSG00000211448	DIO2	9.769281304	2.3614614	17.17710125	2.86895023	0.874800241	3.27954897	0.00104	0.00333
ENSG00000123989	CHPF	28.9125135	6.9227304	50.90229658	2.866637581	0.46662259	6.14337506	8.08E-10	5.23E-09
ENSG00000213886	UBD	7.898773125	1.8132008	13.98434545	2.861891711	0.9926812	2.88299175	0.003939	0.011147
ENSG00000162840	MT2P1	9.889566179	2.4696422	17.30949018	2.851894943	0.813247748	3.50679722	0.000454	0.001554
ENSG00000091129	NRCAM	8651.765313	2119.6086	15183.92198	2.840726521	0.134552773	21.1123596	6.12E-99	2.83E-97
ENSG00000182667	NTM	14.32091369	3.5015316	25.14029578	2.839523881	0.719657327	3.94566104	7.96E-05	0.000308
ENSG00000148798	INA	21.97065702	5.2610125	38.68030151	2.836763535	0.562162582	5.04616213	4.51E-07	2.29E-06
ENSG00000259863	SH3RF3-AS1	90.25366677	22.233298	158.2740355	2.83405977	0.297159469	9.53716796	1.47E-21	1.75E-20
ENSG00000119714	GPR68	191.0275921	47.627637	334.4275471	2.816644013	0.180962023	15.5648349	1.26E-54	3.35E-53
ENSG00000107562	CXCL12	943.8775987	234.2973	1653.457897	2.815370706	0.094664235	29.7405952	2.29E-194	2.48E-192
ENSG00000081059	TCF7	48.47741249	12.248375	84.70645032	2.806533931	0.368938528	7.60705027	2.80E-14	2.41E-13
ENSG00000261488	TBILA	6.933427193	1.7237823	12.14307204	2.805534751	1.022737107	2.74316316	0.006085	0.016476
ENSG00000240184	PCDHGC3	2313.572541	582.86249	4044.282596	2.796790948	0.060864415	45.9511679	0	0
ENSG00000259436	AC010247.2	10.0892347	2.4508798	17.72758959	2.795270913	0.808405071	3.45776024	0.000545	0.001839
ENSG00000170153	RNF150	5.792011762	1.4727103	10.11131317	2.786226668	1.0660548	2.61358672	0.00896	0.023373
ENSG00000159674	SPON2	9.969492038	2.5048467	17.43413738	2.762145294	0.81168632	3.40297135	0.000667	0.002215
ENSG00000236393	AC091806.1	11.33348317	2.9180665	19.74889983	2.75699109	0.758201167	3.63622639	0.000277	0.000981
ENSG00000232124	AP001057.1	10.26000253	2.7209613	17.79904374	2.73806478	0.799558145	3.42447238	0.000616	0.002062
ENSG00000152377	SPOCK1	1538.6729	401.4204	2675.9254	2.736721529	0.272774318	10.0329149	1.09E-23	1.40E-22
ENSG00000099937	SERPIND1	41.71123277	10.665657	72.7568081	2.726017934	0.435633327	6.25759731	3.91E-10	2.59E-09
ENSG00000124212	PTGIS	384.490249	100.93701	668.0434849	2.721430412	0.136110533	19.9942676	6.18E-89	2.56E-87
ENSG00000126562	WNK4	875.3547108	230.77769	1519.931727	2.71948376	0.094402721	28.8072603	1.74E-182	1.62E-180
ENSG00000254887	AC010247.1	48.62441752	12.75911	84.48972456	2.719461177	0.361277335	7.52735064	5.18E-14	4.40E-13
ENSG00000215571	GRK6P1	9.669088158	2.5048467	16.83332962	2.708325131	0.799882382	3.38590422	0.000709	0.002345
ENSG00000232759	AC002480.1	310.1170239	82.269137	537.9649105	2.708084351	0.149501389	18.1141083	2.47E-73	8.61E-72
ENSG00000264672	SEPT4-AS1	17.80153189	4.7771366	30.82592712	2.706524571	0.626991739	4.31668298	1.58E-05	6.73E-05
ENSG00000273312	AL121749.1	15.1293037	4.0044169	26.25419052	2.694060678	0.640004027	4.20944332	2.56E-05	0.000106
ENSG00000164823	OSGIN2	3216.319949	862.50793	5570.131973	2.689822533	0.053573838	50.2077621	0	0
ENSG00000134569	LRP4	236.1152008	63.503402	408.7270001	2.687248315	0.162935161	16.4927465	4.14E-61	1.23E-59
ENSG00000268758	ADGRE4P	6.946679879	1.967251	11.9261088	2.683229764	1.011599563	2.65246236	0.007991	0.021124
ENSG00000224034	LINC02561	8.925447757	2.4698892	15.3810063	2.682556317	0.839162735	3.19670572	0.00139	0.004338
ENSG00000011028	AC080038.1	1984.131087	536.04608	3432.216093	2.678918023	0.060359683	44.3825727	0	0
ENSG00000163131	CTSS	7325.948497	2013.744	12638.15296	2.650095585	0.046066222	57.5279557	0	0
ENSG00000122641	INHBA	355.9337901	98.025165	613.8424152	2.64481238	0.151724761	17.4316464	4.75E-68	1.55E-66
ENSG00000138623	SEMA7A	64.66242849	17.850619	111.4742381	2.631324761	0.307720172	8.5510311	1.22E-17	1.23E-16
ENSG00000156966	B3GNT7	106.3594568	29.603959	183.1149549	2.623559698	0.24401141	10.7517911	5.81E-27	8.40E-26
ENSG00000107968	MAP3K8	303.6862345	85.634641	521.7378276	2.620198781	0.162932134	16.0815347	3.44E-58	9.68E-57
ENSG00000142173	COL6A2	22.12762517	6.2498469	38.00540345	2.61885655	0.53600665	4.88586578	1.03E-06	5.06E-06
ENSG00000168843	FSTL5	40.48757829	11.395075	69.58008147	2.612051984	0.399698865	6.5350498	6.36E-11	4.48E-10
ENSG00000023445	BIRC3	320.4216359	90.765993	550.0772783	2.607674871	0.144121722	18.093559	3.58E-73	1.25E-71
ENSG00000080031	PTPRH	37.55130608	10.694838	64.40777457	2.602940933	0.397780442	6.54366242	6.00E-11	4.24E-10
ENSG00000070404	FSTL3	1191.461607	337.48996	2045.433257	2.60118804	0.089564611	29.0425873	1.91E-185	1.82E-183
ENSG00000083857	FAT1	144.015129	40.926649	247.1036094	2.594728768	0.448836992	5.78100471	7.43E-09	4.44E-08
ENSG00000198947	DMD	111.5185293	31.93071	191.1063489	2.583156195	0.237044132	10.897364	1.19E-27	1.74E-26
ENSG00000253522	MIR3142HG	305.4974641	88.32899	522.6659383	2.577586717	0.16692204	15.4418597	8.56E-54	2.25E-52
ENSG00000248187	AC078850.1	114.9517856	33.225077	196.6784941	2.577132002	0.231219564	11.1458215	7.50E-29	1.14E-27
ENSG00000150630	VEGFC	1632.750951	469.68242	2795.819478	2.57375637	0.173894063	14.8007145	1.45E-49	3.53E-48
ENSG00000168811	IL12A	94.75730169	27.685885	161.8287179	2.568540789	0.26536948	9.67911153	3.70E-22	4.51E-21
ENSG00000164330	EBF1	47.67973859	13.575626	81.7838508	2.56706951	0.367106892	6.99270313	2.70E-12	2.07E-11
ENSG00000259727	AC103740.2	10.41926173	2.8995512	17.9389723	2.564031144	0.867635888	2.95519259	0.003125	0.009047
ENSG00000277496	AL357033.4	5.913349398	1.6969224	10.12977638	2.55077089	1.002990157	2.54316642	0.010985	0.028062
ENSG00000140968	IRF8	6.135751663	1.8322102	10.43929312	2.545784065	1.018562575	2.49938897	0.012441	0.031401
ENSG00000169760	NLGN1	301.3748887	88.353131	514.3966463	2.540778901	0.335091715	7.58233876	3.39E-14	2.91E-13
ENSG00000281903	LINC02246	8.899239085	2.6130275	15.18545065	2.537469078	0.833253752	3.04525371	0.002325	0.006921
ENSG00000100365	NCF4	7.982180482	2.3614614	13.60289961	2.528817278	0.943209355	2.68107739	0.007339	0.019556
ENSG00000135919	SERPINE2	4503.215323	1331.3659	7675.06476	2.526181426	0.045716894	55.2570654	0	0
ENSG00000233452	STXBP5-AS1	436.6860568	130.09251	743.2769019	2.519246619	0.126981791	19.8394321	1.36E-87	5.51E-86
ENSG00000159216	RUNX1	1011.043221	300.21968	1721.866758	2.51497371	0.085129932	29.5427666	8.13E-192	8.46E-190
ENSG00000125148	MT2A	34221.9718	10203.863	58240.08087	2.512918798	0.202573345	12.4049825	2.46E-35	4.47E-34
ENSG00000154319	FAM167A	5.668084641	1.6429555	9.693213756	2.510960451	1.013685622	2.47706034	0.013247	0.033265
ENSG00000182771	GRID1	26.75472654	7.7470969	45.76235623	2.509390042	0.553140427	4.53662383	5.72E-06	2.58E-05
ENSG00000072682	P4HA2	5203.18877	1562.2485	8844.12904	2.502583686	0.04555453	54.9360008	0	0
ENSG00000179862	CITED4	513.5909975	154.82037	872.361622	2.500547019	0.120512338	20.749303	1.24E-95	5.57E-94
ENSG00000114646	CSPG5	55.41872795	16.675344	94.1621118	2.500212002	0.349271092	7.15837084	8.16E-13	6.48E-12
ENSG00000131435	PDLIM4	4489.537003	1362.3501	7616.723886	2.483293595	0.050937822	48.7514676	0	0
ENSG00000146457	WTAP	7069.528599	2145.5743	11993.48292	2.482694128	0.037838248	65.6133478	0	0
ENSG00000172164	SNTB1	75.0755876	23.221638	126.9295368	2.461884812	0.298594456	8.24491131	1.65E-16	1.59E-15
ENSG00000087245	MMP2	23432.70892	7208.0436	39657.37421	2.4596698	0.036846115	66.755201	0	0

ENSG00000107242	PIP5K1B	9.810466233	3.1075682	16.51336424	2.454765896	0.788290154	3.11403851	0.001845	0.005614
ENSG00000005073	HOXA11	97.2589406	30.044533	164.4733487	2.450193487	0.259747069	9.43299764	3.99E-21	4.70E-20
ENSG00000106366	SERPINE1	437803.6463	135690.24	739917.0543	2.447050186	0.090122693	27.1524309	2.37E-162	1.99E-160
ENSG00000166444	ST5	1178.118499	365.39962	1990.837382	2.446261147	0.076467993	31.9906547	1.47E-224	1.95E-222
ENSG00000198732	SMOC1	81.35678276	25.13359	137.5799752	2.445427523	0.291530181	8.38824821	4.93E-17	4.86E-16
ENSG00000176463	SLCO3A1	7.267758419	2.1995607	12.33595616	2.441634447	0.900613047	2.71108048	0.006706	0.018028
ENSG00000053524	MCF2L2	90.41153993	28.375458	152.4476217	2.425682695	0.25752087	9.41936356	4.54E-21	5.32E-20
ENSG00000132965	ALOX5AP	11.41894642	3.6365723	19.20132049	2.421458359	0.832953089	2.90707651	0.003648	0.010405
ENSG00000105371	ICAM4	18.12008602	5.6207595	30.61941252	2.42000097	0.595748989	4.06211511	4.86E-05	0.000194
ENSG00000187957	DNER	1485.950524	469.74903	2502.152017	2.413137091	0.185249968	13.0263833	8.66E-39	1.69E-37
ENSG00000143387	CTSK	273.4561568	87.089824	459.822489	2.403884991	0.322019115	7.46503819	8.33E-14	7.03E-13
ENSG00000253159	PCDHGA12	312.5417294	99.746875	525.3365833	2.403780685	0.141384653	17.0017087	7.98E-65	2.48E-63
ENSG00000172602	RND1	709.9476342	225.93129	1193.963981	2.400664699	0.095881306	25.0378807	2.37E-138	1.61E-136
ENSG00000100979	PLTP	1739.307569	555.09483	2923.52031	2.397782192	0.078234786	30.6485429	2.76E-206	3.24E-204
ENSG00000242419	PCDHGC4	10.40269045	3.2048372	17.60054372	2.393221865	0.775080174	3.08770879	0.002017	0.006085
ENSG00000172183	ISG20	453.5574807	145.77412	761.3408434	2.388431795	0.117417618	20.3413409	5.54E-92	2.39E-90
ENSG00000185634	SHC4	6.23666598	2.0024555	10.47087648	2.373341548	0.987349793	2.40374948	0.016228	0.039821
ENSG00000197208	SLC22A4	837.0911178	270.1673	1404.014934	2.372793573	0.087440744	27.1360177	3.70E-162	3.08E-160
ENSG00000176928	GCNT4	47.34189306	15.083294	79.600492	2.368955211	0.376258006	6.29609251	3.05E-10	2.04E-09
ENSG00000064651	SLC12A2	22068.77182	7156.575	36980.96864	2.368742795	0.039703737	59.6604487	0	0
ENSG00000251136	AF117829.1	151.8282026	49.253065	254.4033398	2.36728347	0.207123131	11.4293535	2.98E-30	4.76E-29
ENSG00000167772	ANGPTL4	2006.026929	651.87668	3360.177175	2.366697999	0.064152345	36.8918393	6.25E-298	1.13E-295
ENSG00000205639	MFSD2B	16.33920625	5.3608488	27.31756373	2.362920891	0.587460347	4.02226448	5.76E-05	0.000228
ENSG00000105357	MYH14	31.99164869	10.451616	53.53168136	2.351439699	0.433262272	5.42728931	5.72E-08	3.14E-07
ENSG00000164114	MAP9	21.53802386	7.1390921	35.93695564	2.344449196	0.526500107	4.45289405	8.47E-06	3.74E-05
ENSG00000168389	MFSD2A	40.9204886	13.477863	68.36311384	2.342830503	0.396559394	5.90789309	3.47E-09	2.12E-08
ENSG00000263528	IKBKE	960.9852093	316.99719	1604.973232	2.342380645	0.086884073	26.9598392	4.37E-160	3.58E-158
ENSG00000260063	AL512408.1	16.91741118	5.6390278	28.19579455	2.330516168	0.590843807	3.94438621	8.00E-05	0.00031
ENSG00000163975	MELTF	51.62166963	17.404021	85.83931858	2.330095503	0.363413365	6.41169458	1.44E-10	9.86E-10
ENSG00000227517	LINC01483	40.23184582	13.524227	66.93946489	2.32554883	0.389309217	5.97352626	2.32E-09	1.44E-08
ENSG00000184261	KCNK12	6.758542308	2.1995607	11.31752394	2.316237416	0.924859876	2.50441983	0.012265	0.031013
ENSG00000152229	PSTPIP2	225.2523092	75.615006	374.8896121	2.311390987	0.177351472	13.0328266	7.96E-39	1.55E-37
ENSG00000088882	CPXM1	62.12131877	21.077527	103.1651108	2.309528547	0.357543347	6.4594365	1.05E-10	7.28E-10
ENSG00000223638	RFPL4A	18.82284569	6.4391016	31.2065898	2.308326766	0.570157108	4.04858018	5.15E-05	0.000205
ENSG00000145901	TNIP1	16485.6663	5547.8272	27423.50542	2.305639072	0.03966195	58.1322664	0	0
ENSG00000258875	AL135818.1	7.04217813	2.3885683	11.69578794	2.303998824	0.895857991	2.57183487	0.010116	0.026039
ENSG00000133805	AMPD3	648.5833989	219.45204	1077.714756	2.301033576	0.098665693	23.3215165	2.68E-120	1.54E-118
ENSG00000128596	CCDC136	14.09536173	4.8044906	23.38623282	2.300714208	0.644438164	3.57010856	0.000357	0.001241
ENSG00000105825	TFPI2	14166.36922	4782.2394	23550.49906	2.300237842	0.036332573	63.3106233	0	0
ENSG00000134516	DOCK2	38.52530776	13.346378	63.70423752	2.289560412	0.452507741	5.05971546	4.20E-07	2.14E-06
ENSG00000090376	IRAK3	1076.735779	366.59173	1786.879829	2.282792989	0.078366153	29.1298336	1.50E-186	1.46E-184
ENSG00000271605	MILR1	71.53671214	24.586662	118.4867624	2.276552575	0.293512926	7.75622595	8.75E-15	7.70E-14
ENSG00000171017	LRRC8E	55.63633237	19.298542	91.97412238	2.274635683	0.327516669	6.94509898	3.78E-12	2.88E-11
ENSG00000108679	LGALS3BP	392.6648675	135.18697	650.1427678	2.269599167	0.136680079	16.6051936	6.39E-62	1.91E-60
ENSG00000236452	AC123023.1	11.05302139	3.6535086	18.45253422	2.267003163	0.803949715	2.81983204	0.004805	0.013319
ENSG00000166592	RRAD	10.81630772	3.6722709	17.96034451	2.265367694	0.714620709	3.17002805	0.001524	0.004715
ENSG00000106034	CPED1	274.9908606	94.26965	455.7120712	2.261113857	0.158477531	14.2677252	3.48E-46	7.88E-45
ENSG00000148154	UGCG	7638.647634	2637.8923	12639.40295	2.259539856	0.037888585	59.6364271	0	0
ENSG00000170775	GPR37	52.74827491	18.390288	87.10626203	2.254321978	0.328874316	6.85466109	7.15E-12	5.36E-11
ENSG00000233930	KRTAP5-AS1	57.18060883	20.096887	94.26433077	2.245979809	0.412879002	5.43980149	5.33E-08	2.94E-07
ENSG00000138347	MYPN	30.16340846	10.55955	49.76726711	2.238560089	0.437312655	5.11890077	3.07E-07	1.58E-06
ENSG00000242173	ARHGDIG	18.32617973	6.3929852	30.25937425	2.238484823	0.554995253	4.03334049	5.50E-05	0.000218
ENSG00000221890	NPTXR	101.7083575	35.423059	167.9936563	2.237563288	0.255035207	8.77354665	1.73E-18	1.82E-17
ENSG00000144810	COL8A1	33337.89095	11685.782	54990.00001	2.234597066	0.030416785	73.4659193	0	0
ENSG00000150347	ARID5B	2415.138154	848.11978	3982.156529	2.2321907	0.052536307	42.4885345	0	0
ENSG00000128298	BAIAP2L2	14.15145857	4.9749829	23.3279342	2.230378564	0.672257788	3.31774299	0.000907	0.002935
ENSG00000179630	LACC1	774.8058109	273.89971	1275.711915	2.225953816	0.115800263	19.2223554	2.41E-82	9.22E-81
ENSG00000239264	TXNDC5	476.763049	167.9654	785.5606955	2.225584735	0.699306668	3.18255901	0.00146	0.004537
ENSG00000106624	AEBP1	3701.763963	1309.3716	6094.156364	2.221169553	0.04942568	44.9395847	0	0
ENSG00000100906	NFKBIA	4314.144017	1529.0036	7099.284457	2.216949768	0.045000356	49.2651611	0	0
ENSG00000249661	TNRC18P1	7.043988709	2.4237728	11.66420458	2.212349141	0.957903554	2.309574	0.020912	0.049783
ENSG00000115604	IL18R1	109.3080621	38.438147	180.1779771	2.211325104	0.235913142	9.37347145	7.02E-21	8.17E-20
ENSG00000157214	STEAP2	373.5119659	132.72226	614.3016675	2.20433144	0.132974536	16.5770944	1.02E-61	3.05E-60
ENSG00000154511	DIPK1A	24.35810083	8.6472539	40.06894778	2.20367884	0.491641025	4.48229242	7.38E-06	3.29E-05
ENSG00000102385	DRP2	27.85140123	9.7951746	45.90762783	2.200016102	0.464211592	4.73925284	2.15E-06	1.01E-05
ENSG00000126860	EVI2A	6.88796675	2.5509631	11.22497043	2.195644489	0.940738961	2.33395722	0.019598	0.047069
ENSG00000135124	P2RX4	6293.981202	2264.6825	10323.2799	2.187784468	0.04061229	53.8700102	0	0
ENSG00000243746	EEF1A1P10	8.449115102	2.9535181	13.94471214	2.187329355	0.907609296	2.40999003	0.015953	0.039258
ENSG00000237181	AC147651.4	40.70834627	14.853058	66.56363494	2.184585324	0.399205757	5.47232921	4.44E-08	2.47E-07
ENSG00000148288	GBGT1	190.3544907	68.74649	311.9624916	2.184521799	0.183634861	11.8960081	1.24E-32	2.11E-31
ENSG00000151320	AKAP6	21.05608687	7.6146234	34.49755038	2.183558821	0.542047284	4.02835488	5.62E-05	0.000223
ENSG00000227542	AC092614.1	11.86366582	4.282843	19.44448866	2.165971117	0.673604341	3.21549459	0.001302	0.004087
ENSG00000115738	ID2	160.1139864	58.790156	261.4378174	2.16053959	0.18762681	11.5150899	1.11E-30	1.81E-29
ENSG00000156466	GDF6	15.76164424	5.8829905	25.64029798	2.157565601	0.694151226	3.10820686	0.001882	0.005713
ENSG00000177679	SRRM3	81.71564894	29.70271	133.728588	2.156989429	0.283607666	7.60553994	2.84E-14	2.44E-13
ENSG00000091128	LAMB4	19.3879629	6.9847949	31.79113093	2.156045489	0.556342996	3.87538893	0.000106	0.000404
ENSG00000237499	AL357060.1	10.32542145	3.8070646	16.84377824	2.145471261	0.771687523	2.78023319	0.005432	0.01487
ENSG00000196878	LAMB3	363.0265963	133.87897	592.174222	2.143168882	0.147755548	14.5048285	1.13E-47	2.65E-46
ENSG00000161544	CYGB	45.06370741	16.928736	73.1986784	2.141988986	0.385763048	5.55260281	2.81E-08	1.60E-07
ENSG00000128165	ADM2	19.13172094	7.1124792	31.1509627	2.140343428	0.572009052	3.74179993	0.000183	0.000667
ENSG00000229563	LINC01204	11.2386363	4.2205315	18.2567411	2.134745073	0.704656292	3.02948416	0.00245	0.007256
ENSG00000256262	USP30-AS1	25.20798313	9.3655127	41.05045359	2.133809339	0.463724793	4.60145623	4.20E-06	1.92E-05
ENSG00000185518	SV2B	41.56389817	15.534533	67.59326359	2.124129849	0.365505191	5.81149024	6.19E-09	3.72E-08
ENSG00000205363	INSYN1	12.36865189	4.615483	20.12182077	2.117838419	0.676101722	3.13242572	0.001734	0.0053
ENSG00000225554	AL359764.1	16.89868847	6.4664556	27.33092136	2.113530212	0.597839155	3.53528235	0.000407	0.001404

ENSG00000102096	PIM2	427.6634781	160.90353	694.4234277	2.111189418	0.11437802	18.4579994	4.50E-76	1.62E-74
ENSG00000110042	DTX4	351.5425685	132.69244	570.3926956	2.10690196	0.134928445	15.6149577	5.76E-55	1.53E-53
ENSG00000157368	IL34	30.00511021	11.287388	48.72283206	2.105000979	0.475088725	4.43075339	9.39E-06	4.12E-05
ENSG00000171236	LRG1	38.20702313	14.474795	61.93925098	2.104124929	0.402572724	5.22669521	1.73E-07	9.13E-07
ENSG00000166741	NNMT	5019.583745	1904.1735	8134.993961	2.096848094	0.047081683	44.5363879	0	0
ENSG00000204959	ARHGEF34P	10.60958327	4.0046639	17.21450262	2.0896364	0.718665772	2.90766095	0.003641	0.010389
ENSG00000163291	PAQR3	1295.38476	492.9684	2097.801117	2.089035552	0.074401863	28.0777318	1.83E-173	1.64E-171
ENSG00000123342	MMP19	174.4448422	66.501554	282.3881306	2.08844994	0.18927433	11.0339841	2.62E-28	3.91E-27
ENSG00000119514	GALNT12	26.22548056	10.030052	42.4209095	2.087856184	0.468046188	4.46079091	8.17E-06	3.62E-05
ENSG00000197106	SLC6A17	80.20393866	30.447088	129.9607898	2.081750321	0.268414026	7.75574345	8.78E-15	7.73E-14
ENSG00000258808	LINC02310	13.75803139	5.3071289	22.20893389	2.070016887	0.631982994	3.27543131	0.001055	0.003371
ENSG00000110031	LPXN	440.4855427	170.1336	710.837482	2.066408018	0.125978561	16.4028546	1.82E-60	5.36E-59
ENSG00000279148	AC126474.1	14.51396756	5.4854717	23.54246338	2.060544248	0.629567172	3.27295377	0.001064	0.003398
ENSG00000144218	AFF3	108.5837013	42.150263	175.0171395	2.059834835	0.225236099	9.14522513	5.95E-20	6.66E-19
ENSG00000140479	PCSK6	93.66098782	36.376688	150.9452872	2.055673462	0.243158982	8.45403055	2.81E-17	2.80E-16
ENSG00000235001	EIF4A1P2	9.821986494	3.8344186	15.80955434	2.048866918	0.735091316	2.78722775	0.005316	0.01457
ENSG00000107249	GLIS3	1151.362149	449.81079	1852.913505	2.043812651	0.073029484	27.9861303	2.40E-172	2.14E-170
ENSG00000132031	MATN3	391.7703118	154.67402	628.8666005	2.029504697	0.126112421	16.0928217	2.86E-58	8.11E-57
ENSG00000137331	IER3	10966.2897	4342.6554	17589.92397	2.019093951	0.04178652	48.3192656	0	0
ENSG00000157168	NRG1	1872.86533	741.50399	3004.226671	2.017788354	0.063769732	31.6417881	9.84E-220	1.23E-217
ENSG00000152784	PRDM8	16.11473385	6.5204225	25.70904523	2.016628188	0.62474512	3.22792147	0.001247	0.003928
ENSG00000134853	PDGFRA	34.95616649	13.764387	56.14794598	2.016557337	0.423987893	4.75616727	1.97E-06	9.38E-06
ENSG00000152669	CCNO	23.03293465	9.1950204	36.87084893	2.013651185	0.502379893	4.00822409	6.12E-05	0.000241
ENSG00000116741	RGS2	229.3433186	92.07705	366.6095873	1.999277165	0.158231525	12.6351381	1.35E-36	2.53E-35
ENSG00000163545	NUAK2	447.5846934	179.61663	715.5527572	1.997241793	0.116368292	17.16311	5.02E-66	1.58E-64
ENSG00000280143	AP000892.3	29.06073265	11.745489	46.37597592	1.994637301	0.486498447	4.09998699	4.13E-05	0.000167
ENSG00000120262	CCDC170	18.66036783	7.6060317	29.71470392	1.989425762	0.589646899	3.37392729	0.000741	0.00244
ENSG00000115107	STEAP3	923.5083595	371.60897	1475.407746	1.988736173	0.086073654	23.1050511	4.12E-118	2.31E-116
ENSG00000240771	ARHGEF25	11.36866253	4.6608583	18.0764668	1.984925465	0.784625471	2.52977445	0.011414	0.029046
ENSG00000014914	MTMR11	76.9546782	31.186579	122.7227773	1.975868235	0.292696531	6.75056937	1.47E-11	1.08E-10
ENSG00000125657	TNFSF9	116.828335	47.438877	186.2177934	1.974027711	0.212073534	9.30822282	1.30E-20	1.50E-19
ENSG00000124243	BCAS4	76.70084304	31.074534	122.3262522	1.972375777	0.324456587	6.0790129	1.21E-09	7.70E-09
ENSG00000163293	NIPAL1	13.07356426	5.3610958	20.78603273	1.969991733	0.655206884	3.0066713	0.002641	0.007767
ENSG00000203805	PLPP4	195.6975456	79.518599	311.8764925	1.968712262	0.192626161	10.2203784	1.61E-24	2.13E-23
ENSG00000073670	ADAM11	10.01774198	4.0854907	15.94999322	1.966134341	0.744670951	2.64027265	0.008284	0.021826
ENSG00000227507	LTB	333.9507109	136.3814	531.5200203	1.962588289	0.144314418	13.5993916	4.04E-42	8.48E-41
ENSG00000226287	TMEM191A	12.01376014	4.8663081	19.16121222	1.962484182	0.701284098	2.79841534	0.005135	0.014149
ENSG00000139263	LRIG3	1276.127497	521.70569	2030.549308	1.962357608	0.159525574	12.3012101	8.92E-35	1.60E-33
ENSG00000105639	JAK3	326.6245441	133.77756	519.4715316	1.960126577	0.133786609	14.6511418	1.32E-48	3.17E-47
ENSG00000137193	PIM1	378.4855984	155.45622	601.5149725	1.946003328	0.125471012	15.5095851	2.99E-54	7.90E-53
ENSG00000168234	TTC39C	68.43455219	27.905457	108.9636472	1.944227547	0.300665517	6.46641347	1.00E-10	6.97E-10
ENSG00000184441	AP001062.1	163.0891821	67.055345	259.1230196	1.942821017	0.18546476	10.4754187	1.12E-25	1.56E-24
ENSG00000164161	HHIP	31166.07697	12900.565	49431.58859	1.937987035	0.080872145	23.9635914	6.67E-127	4.02E-125
ENSG00000236830	CBR3-AS1	22.0739089	9.1955144	34.95230336	1.93518236	0.49698026	3.89388174	9.87E-05	0.000376
ENSG00000062282	DGAT2	24.59134719	10.227404	38.95529052	1.928743036	0.458347883	4.20803304	2.58E-05	0.000107
ENSG00000175746	C15orf54	799.3617934	334.11024	1264.61335	1.9229004	0.094057454	20.4438916	6.81E-93	2.96E-91
ENSG00000128487	SPECC1	438.776172	184.06419	693.4881564	1.919602766	0.120366634	15.9479641	2.94E-57	8.14E-56
ENSG00000198832	SELENOM	1259.393069	527.93331	1990.852828	1.917965049	0.074809374	25.6380309	5.75E-145	4.17E-143
ENSG00000162772	ATF3	387.0070892	162.55838	611.4557944	1.915134984	0.121154224	15.8074141	2.77E-56	7.54E-55
ENSG00000154642	C21orf91	320.1480171	133.90746	506.3885714	1.913711046	0.140883155	13.5836754	5.01E-42	1.05E-40
ENSG00000176788	BASP1	1846.40638	777.70979	2915.102968	1.909169123	0.062984337	30.311808	8.01E-202	9.17E-200
ENSG00000184363	PKP3	8.239900346	3.4478117	13.03198896	1.90850338	0.823159376	2.31851016	0.020422	0.048757
ENSG00000136999	CCN3	51.81551653	22.000741	81.63029177	1.902458201	0.32370019	5.877223	4.17E-09	2.54E-08
ENSG00000169715	MT1E	2401.173975	1013.9888	3788.359109	1.901650689	0.249661661	7.61691116	2.60E-14	2.24E-13
ENSG00000138448	ITGAV	33153.86068	14076.145	52231.57616	1.891580422	0.033126046	57.1025112	0	0
ENSG00000011347	SYT7	14.80126834	6.3853817	23.21715496	1.887394657	0.617575413	3.05613633	0.002242	0.006703
ENSG00000145358	DDIT4L	1245.562436	531.89704	1959.227831	1.879570867	0.073538296	25.5590755	4.35E-144	3.13E-142
ENSG00000232517	AC112198.2	18.99996486	8.0899076	29.91002209	1.878963642	0.541595243	3.46931342	0.000522	0.001767
ENSG00000166670	MMP10	1608.50543	689.40144	2527.609416	1.8762257	0.062065475	30.2297805	9.62E-201	1.09E-198
ENSG00000155324	GRAMD2B	229.1081078	98.371187	359.8450285	1.873728225	0.153234791	12.2278251	2.21E-34	3.94E-33
ENSG00000171791	BCL2	95.07945657	40.975976	149.1829368	1.872537862	0.256792776	7.29201924	3.05E-13	2.48E-12
ENSG00000196209	SIRPB2	813.7697437	351.06267	1276.476813	1.864537522	0.08411384	22.1668339	7.18E-109	3.66E-107
ENSG00000204624	DISP3	31.87117642	13.837116	49.90523657	1.853597347	0.424654506	4.36495391	1.27E-05	5.49E-05
ENSG00000260101	AC008074.2	60.56135013	26.418625	94.70407524	1.851916466	0.293879188	6.30162509	2.95E-10	1.98E-09
ENSG00000119938	PPP1R3C	63.72631052	27.65547	99.79715088	1.848911373	0.301776699	6.12675326	8.97E-10	5.78E-09
ENSG00000128606	LRRC17	484.56199	211.02628	758.0976974	1.847848943	0.129443923	14.2752854	3.12E-46	7.10E-45
ENSG00000153714	LURAP1L	380.1845077	165.25653	595.1124819	1.847718757	0.12441222	14.8515857	6.79E-50	1.66E-48
ENSG00000143851	PTPN7	18.51934106	8.1628839	28.87579819	1.842822427	0.538551721	3.42181142	0.000622	0.002078
ENSG00000240476	LINC00973	12.49602453	5.4229132	19.56913586	1.840976733	0.709915083	2.59323513	0.009508	0.024628
ENSG00000171621	SPSB1	2761.655063	1207.7261	4315.584	1.838439869	0.058719001	31.3091133	3.51E-215	4.34E-213
ENSG00000133065	SLC41A1	3910.48627	1710.6214	6110.351107	1.835704505	0.047530405	38.6216885	0	0
ENSG00000105426	PTPRS	818.0863998	358.12972	1278.043075	1.835398376	0.113002903	16.2420463	2.54E-59	7.37E-58
ENSG00000267534	S1PR2	56.91078934	24.699385	89.12219384	1.835088803	0.339259475	5.40910111	6.33E-08	3.47E-07
ENSG00000179403	VWA1	624.8359877	273.76783	975.9041429	1.833433108	0.106607488	17.1979769	2.75E-66	8.70E-65
ENSG00000276116	FUT8-AS1	10.97465883	4.7586213	17.19069636	1.827223966	0.704542311	2.59349075	0.009501	0.024615
ENSG00000119900	OGFRL1	15976.41982	7028.5403	24924.29933	1.825953092	0.041810236	43.6723943	0	0
ENSG00000145365	TIFA	985.4966998	433.94105	1537.052347	1.824479135	0.080856727	22.5643457	9.71E-113	5.26E-111
ENSG00000164692	COL1A2	195.0636154	85.935287	304.1919438	1.823757063	0.185538473	9.82953582	8.40E-23	1.05E-21
ENSG00000058085	LAMC2	23279.6396	10258.54	36300.73958	1.823131867	0.041993611	43.414506	0	0
ENSG00000205364	MT1M	60.61885631	26.504488	94.73322455	1.822836329	0.317764268	5.73644211	9.67E-09	5.71E-08
ENSG00000234155	LINC02535	14.25673977	6.2039776	22.30950198	1.822015926	0.638945091	2.85160016	0.00435	0.012153
ENSG00000173083	HPSE	655.3880871	289.29288	1021.483292	1.821808137	0.097146826	18.7531412	1.82E-78	6.78E-77
ENSG00000111110	PPM1H	679.8423331	299.60877	1060.075897	1.821587859	0.105187286	17.3175668	3.47E-67	1.12E-65
ENSG00000105696	TMEM59L	17.02057242	7.6336328	26.40751208	1.817956161	0.586194737	3.10128366	0.001927	0.005833
ENSG00000105792	CFAP69	49.48802273	21.819831	77.15621425	1.817815291	0.333246883	5.45486059	4.90E-08	2.71E-07

ENSG00000198843	SELENOT	5331.506998	2355.6746	8307.339407	1.817671354	0.039170652	46.4039084	0	0
ENSG00000151014	NOCT	665.6400426	295.79647	1035.48362	1.812176209	0.097900472	18.5103931	1.70E-76	6.17E-75
ENSG00000180801	ARSJ	2625.6661	1167.1799	4084.152259	1.807567686	0.128049502	14.1161633	3.02E-45	6.77E-44
ENSG00000149269	PAK1	10.70247671	4.8308565	16.57409692	1.791787012	0.773963772	2.31507866	0.020609	0.04912
ENSG00000164674	SYTL3	117.0410386	52.203028	181.8790491	1.786642834	0.224755558	7.94927098	1.88E-15	1.71E-14
ENSG00000205362	MT1A	18.95198666	8.5568473	29.34712602	1.782199801	0.636215112	2.80125349	0.00509	0.014036
ENSG00000112715	VEGFA	324.2222063	146.79337	501.6510449	1.78100817	0.136562607	13.0416972	7.09E-39	1.38E-37
ENSG00000223776	LGALS8-AS1	14.90804201	6.5989291	23.21715496	1.780499348	0.628003269	2.83517529	0.00458	0.012741
ENSG00000189120	SP6	130.237109	58.879574	201.594644	1.779216434	0.223800839	7.94999894	1.87E-15	1.71E-14
ENSG00000101605	MYOM1	14.95057011	6.6524019	23.24873832	1.77411869	0.634351979	2.79674179	0.005162	0.014208
ENSG00000163347	CLDN1	1455.609013	660.43595	2250.782079	1.770390076	0.082100033	21.5638169	3.93E-103	1.88E-101
ENSG00000171451	DSEL	1434.34101	650.08788	2218.594138	1.768744299	0.068487864	25.8256603	4.57E-147	3.38E-145
ENSG00000116990	MYCL	46.46086489	21.247031	71.67469883	1.767926124	0.342595378	5.16039105	2.46E-07	1.28E-06
ENSG00000138135	CH25H	24.5164497	11.170369	37.86253052	1.763910889	0.454919235	3.87741549	0.000106	0.000401
ENSG00000253837	AC090197.1	51.31005022	23.318166	79.30193421	1.763637509	0.352225872	5.00712085	5.53E-07	2.78E-06
ENSG00000166016	ABTB2	14.04543723	6.3658782	21.72499622	1.763469236	0.617394287	2.85630961	0.004286	0.012004
ENSG00000150551	LYPD1	155.0998051	70.819945	239.3796649	1.760037059	0.225016459	7.82181476	5.21E-15	4.65E-14
ENSG00000101384	JAG1	10402.20631	4745.3475	16059.06508	1.758976169	0.037182184	47.3069617	0	0
ENSG00000010818	HIVEP2	1585.254057	724.68309	2445.825019	1.757417982	0.068921687	25.4987662	2.03E-143	1.45E-141
ENSG00000130309	COLGALT1	29152.00602	13314.04	44989.9718	1.756716907	0.028185	62.3280787	0	0
ENSG00000166173	LARP6	680.6776132	311.40427	1049.950953	1.752024104	0.090802897	19.294804	5.94E-83	2.29E-81
ENSG00000124134	KCNS1	14.98517613	6.7876897	23.18266259	1.751357048	0.586349107	2.98688448	0.002818	0.00824
ENSG00000170017	ALCAM	17543.45449	8036.1911	27050.71784	1.75115587	0.03225472	54.291461	0	0
ENSG00000152503	TRIM36	39.98089372	18.517231	61.44455647	1.747521634	0.402974738	4.33655381	1.45E-05	6.18E-05
ENSG00000140323	DISP2	26.72801181	12.138121	41.31790297	1.736755293	0.494488508	3.5122258	0.000444	0.001526
ENSG00000162407	PLPP3	1380.818504	638.52591	2123.111093	1.735834095	0.066714538	26.0188279	3.03E-149	2.29E-147
ENSG00000196876	SCN8A	16.90590076	7.8119756	25.99982592	1.727034567	0.568025109	3.04041941	0.002362	0.00702
ENSG00000158292	GPR153	309.699757	143.85861	475.5409033	1.726598832	0.144578528	11.9422908	7.12E-33	1.22E-31
ENSG00000260231	KDM7A-DT	56.81129703	26.326392	87.29620178	1.721844121	0.319797359	5.38417243	7.28E-08	3.97E-07
ENSG00000279118	AC093535.2	12.65090332	5.7910048	19.51080186	1.720625086	0.687271542	2.50355934	0.012295	0.031085
ENSG00000149948	HMGA2	1834.194423	855.62843	2812.760413	1.716833411	0.191172335	8.98055365	2.69E-19	2.91E-18
ENSG00000144366	GULP1	1532.436778	715.34459	2349.528966	1.71621981	0.070600902	24.3089752	1.58E-130	9.87E-129
ENSG00000163661	PTX3	55934.15962	26124.855	85743.46408	1.714680968	0.038982953	43.9854046	0	0
ENSG00000175040	CHST2	977.4372635	457.36883	1497.505699	1.713164357	0.081844294	20.931995	2.74E-97	1.25E-95
ENSG00000203635	AC144450.1	69.65536417	32.73103	106.5796979	1.709272486	0.296709724	5.76075655	8.37E-09	4.98E-08
ENSG00000008517	IL32	4033.574689	1892.6093	6174.540086	1.707489511	0.051114897	33.4049292	1.16E-244	1.75E-242
ENSG00000185972	CCIN	43.87221588	20.545461	67.19897044	1.7066345	0.398185805	4.28602547	1.82E-05	7.69E-05
ENSG00000272092	AC087623.2	13.20131093	6.2227399	20.17988192	1.705283938	0.634615125	2.68711518	0.007207	0.019243
ENSG00000170921	TANC2	1609.491365	756.79407	2462.188662	1.702209977	0.069271974	24.5728521	2.47E-133	1.61E-131
ENSG00000146197	SCUBE3	115.7575069	54.722439	176.7925747	1.701059709	0.230823155	7.3695367	1.71E-13	1.42E-12
ENSG00000145242	EPHA5	16.94304164	7.928254	25.9578293	1.696746405	0.588117342	2.88504739	0.003914	0.011082
ENSG00000270629	NBPF14	100.0168457	47.295491	152.7382003	1.693625374	0.231710984	7.30921488	2.69E-13	2.20E-12
ENSG00000187193	MT1X	29.32660354	13.89133	44.76187689	1.692813352	0.426171513	3.97214102	7.12E-05	0.000278
ENSG00000197594	ENPP1	20.1285293	9.6087343	30.64832435	1.690645949	0.502887743	3.36187543	0.000774	0.002538
ENSG00000153233	PTPRR	70.42793208	33.122674	107.7331906	1.690580255	0.298822271	5.65747743	1.54E-08	8.90E-08
ENSG00000132429	POPDC3	42.31655586	19.860431	64.77268094	1.687948162	0.361725793	4.6663749	3.07E-06	1.42E-05
ENSG00000188488	SERPINA5	28.14421208	13.507785	42.78063954	1.687674388	0.560454719	3.01125913	0.002602	0.007659
ENSG00000214530	STARD10	606.7756112	288.40191	925.1493145	1.683087621	0.09554936	17.6148498	1.89E-69	6.37E-68
ENSG00000179542	SLITRK4	199.8225751	94.685684	304.9594661	1.682040095	0.162636498	10.3423286	4.53E-25	6.13E-24
ENSG00000113070	HBEGF	1500.850533	713.78033	2287.920734	1.678917043	0.069608587	24.1193955	1.56E-128	9.49E-127
ENSG00000148143	ZNF462	1207.64577	576.72546	1838.566081	1.673988664	0.068521191	24.430233	8.16E-132	5.19E-130
ENSG00000128271	ADORA2A	19.24241664	9.2492343	29.23559898	1.672457718	0.510861473	3.27379888	0.001061	0.00339
ENSG00000123689	G0S2	47.10961058	22.610325	71.60889596	1.672292064	0.364679941	4.58564312	4.53E-06	2.07E-05
ENSG00000091972	CD200	193.4201523	92.278847	294.5614571	1.667173274	0.174814044	9.53683833	1.47E-21	1.76E-20
ENSG00000197467	COL13A1	1534.265814	736.30587	2332.225756	1.663732055	0.207695544	8.01043692	1.14E-15	1.06E-14
ENSG00000197880	MDS2	10.33384173	4.9744889	15.69319457	1.658404781	0.69073272	2.4009356	0.016353	0.040069
ENSG00000243649	CFB	21.77725957	10.578312	32.97620696	1.657561483	0.511529596	3.24040192	0.001194	0.003777
ENSG00000136048	DRAM1	6708.011623	3232.8488	10183.17442	1.654676568	0.039827533	41.5460476	0	0
ENSG00000217801	AL390719.1	21.35515926	10.389799	32.32051991	1.648878693	0.505056176	3.26474316	0.001096	0.003488
ENSG00000118515	SGK1	3934.905133	1904.9262	5964.88408	1.646981194	0.144913048	11.3653064	6.22E-30	9.84E-29
ENSG00000142920	AZIN2	25.25920536	12.35705	38.16136116	1.644762161	0.467632523	3.5172108	0.000436	0.001499
ENSG00000114529	C3orf52	208.8179907	101.65991	315.9760689	1.642626828	0.174322267	9.42293176	4.39E-21	5.15E-20
ENSG00000120820	GLT8D2	16.3895959	7.8742871	24.90490471	1.642413086	0.587097598	2.79751287	0.00515	0.01418
ENSG00000183196	CHST6	38.8414454	18.793831	58.8890599	1.641300105	0.368112652	4.45868974	8.25E-06	3.65E-05
ENSG00000088881	EBF4	49.3653438	23.783527	74.94716083	1.639172344	0.359418799	4.56061939	5.10E-06	2.32E-05
ENSG00000221843	C2orf16	31.17123646	15.220655	47.12181781	1.639168049	0.429639619	3.81521624	0.000136	0.000507
ENSG00000147416	ATP6V1B2	8701.453762	4232.485	13170.42252	1.636684993	0.036201544	45.2103647	0	0
ENSG00000020181	ADGRA2	4656.050484	2266.6505	7045.450419	1.635725556	0.043552931	37.5571863	0	0
ENSG00000121653	MAPK8IP1	262.5273602	127.81912	397.2356009	1.635241961	0.156362634	10.4580098	1.35E-25	1.86E-24
ENSG00000261888	AC144831.1	26.67013758	12.929356	40.41271941	1.633843096	0.449243307	3.63687799	0.000276	0.000979
ENSG00000116032	GRIN3B	77.01596519	37.702952	116.3289783	1.616818891	0.262468922	6.16003935	7.27E-10	4.72E-09
ENSG00000146555	SDK1	104.2670329	51.353134	157.1809319	1.616057365	0.22428902	7.2052451	5.79E-13	4.66E-12
ENSG00000168461	RAB31	2556.822234	1261.0085	3852.635937	1.610917125	0.054736909	29.4301805	2.26E-190	2.31E-188
ENSG00000112139	MDGA1	12.69614822	6.3314148	19.06088161	1.609075885	0.657590401	2.44692727	0.014408	0.035893
ENSG00000285106	AC016831.7	16.91691432	8.3414738	25.49235484	1.606979454	0.588288273	2.73161905	0.006302	0.017024
ENSG00000145779	TNFAIP8	349.783694	172.77132	526.7960673	1.605972814	0.132588026	12.1125026	9.07E-34	1.59E-32
ENSG00000277734	TRAC	97.34891064	47.991185	146.7066361	1.602830052	0.259062973	6.1870287	6.13E-10	4.01E-09
ENSG00000136010	ALDH1L2	50.18642535	24.827316	75.54553454	1.598429938	0.317210622	5.0390177	4.68E-07	2.38E-06
ENSG00000099822	HCN2	108.137538	53.604341	162.6707347	1.594079742	0.230288154	6.92210917	4.45E-12	3.37E-11
ENSG00000183691	NOG	548.4026999	273.1968	823.6085961	1.59320187	0.114747241	13.884446	7.87E-44	1.70E-42
ENSG00000188404	SELL	146.0081424	72.379904	219.6363809	1.5919812	0.199340794	7.98622885	1.39E-15	1.28E-14
ENSG00000233058	LINC00884	14.24030818	7.165705	21.31491139	1.584603122	0.602270909	2.63104709	0.008512	0.022356
ENSG00000164736	SOX17	1780.184363	889.79333	2670.575397	1.584510258	0.061166655	25.9048047	5.88E-148	4.38E-146
ENSG00000005471	ABCB4	21.50316831	10.6307	32.37563668	1.583277745	0.528189983	2.99755352	0.002722	0.007982
ENSG00000273132	AL355312.4	19.19986339	9.743528	28.6561988	1.578661586	0.552115755	2.85929458	0.004246	0.011908

ENSG00000215267	AKR1C7P	12.3694627	6.222987	18.51593842	1.578010389	0.651782264	2.42106985	0.015475	0.038244
ENSG00000243753	HLA-L	14.53830152	7.3093373	21.76726569	1.573936242	0.577736794	2.72431367	0.006444	0.017375
ENSG00000049130	KITLG	5999.216069	3028.452	8969.980156	1.565287048	0.046697078	33.520021	2.46E-246	3.76E-244
ENSG00000170689	HOXB9	54.17263054	27.405386	80.93987487	1.56247608	0.312287284	5.00332918	5.63E-07	2.84E-06
ENSG00000169248	CXCL11	164.4508511	82.884252	246.0174506	1.56170167	0.189114647	8.25796253	1.48E-16	1.42E-15
ENSG00000117228	GBP1	3920.359787	1989.3028	5851.416798	1.556938026	0.04826376	32.2589456	2.64E-228	3.61E-226
ENSG00000128641	MYO1B	15957.79105	8095.8475	23819.73456	1.55661721	0.040493581	38.441086	0	0
ENSG00000128602	SMO	67.48820492	34.263829	100.712581	1.556152368	0.330253583	4.71199239	2.45E-06	1.15E-05
ENSG00000151136	BTBD11	94.64925044	47.956475	141.3420261	1.554263705	0.254539453	6.10617995	1.02E-09	6.54E-09
ENSG00000205502	C2CD4B	1134.187726	578.11073	1690.264724	1.550812332	0.077982117	19.8867689	5.30E-88	2.17E-86
ENSG00000253250	C8orf88	33.17949485	16.998898	49.36009128	1.550039074	0.389609632	3.97844136	6.94E-05	0.000271
ENSG00000156804	FBXO32	1124.236029	573.33161	1675.140446	1.54616527	0.073123242	21.1446488	3.09E-99	1.45E-97
ENSG00000242265	PEG10	6445.775536	3288.8719	9602.679153	1.545275529	0.043478203	35.5413848	1.13E-276	1.94E-274
ENSG00000130635	COL5A1	16705.13865	8536.4711	24873.80615	1.542696194	0.032576567	47.3560085	0	0
ENSG00000140332	TLE3	2104.298453	1074.7993	3133.797607	1.542139446	0.05452542	28.282945	5.60E-176	5.07E-174
ENSG00000270607	AC009549.1	426.9892603	218.34372	635.6348046	1.541183926	0.279234659	5.51931459	3.40E-08	1.92E-07
ENSG00000213949	ITGA1	2045.03142	1046.089	3043.973861	1.540955934	0.055746062	27.6424177	3.44E-168	3.00E-166
ENSG00000105290	APLP1	111.6623635	57.344058	165.9806688	1.539861773	0.242975598	6.33751614	2.33E-10	1.58E-09
ENSG00000134363	FST	90.4293584	46.432612	134.4261049	1.536254544	0.244908366	6.27277283	3.55E-10	2.36E-09
ENSG00000109320	NFKB1	4552.349671	2338.4642	6766.23518	1.532659657	0.040235945	38.0918026	0	0
ENSG00000075420	FNDC3B	22333.78996	11480.123	33187.4573	1.531340524	0.029952932	51.1248956	0	0
ENSG00000074964	ARHGEF10L	319.0321453	164.10762	473.956666	1.530278728	0.127096746	12.0402668	2.18E-33	3.81E-32
ENSG00000197646	PDCD1LG2	2472.659515	1271.5788	3673.740277	1.528106568	0.05558673	27.4904924	2.28E-166	1.96E-164
ENSG00000235385	LINC02154	231.2141967	119.60839	342.8200021	1.523860154	0.162304129	9.38891796	6.06E-21	7.07E-20
ENSG00000152822	GRM1	18.2646765	9.4736935	27.0556595	1.520428117	0.518593003	2.93183307	0.00337	0.009689
ENSG00000108797	CNTNAP1	4783.556757	2474.2983	7092.815189	1.519692928	0.051858127	29.304817	9.00E-189	9.00E-187
ENSG00000135318	NT5E	20053.2578	10376.991	29729.52445	1.518436061	0.029083669	52.2092338	0	0
ENSG00000089041	P2RX7	606.8566816	314.29474	899.4186242	1.515290008	0.094018479	16.1169382	1.94E-58	5.50E-57
ENSG00000167984	NLRC3	26.32551045	13.748686	38.90233495	1.514217302	0.439643346	3.44419474	0.000573	0.001927
ENSG00000124920	MYRF	41.77960289	21.657189	61.90201635	1.506536343	0.380496231	3.95939886	7.51E-05	0.000292
ENSG00000149781	FERMT3	2827.520041	1474.196	4180.84407	1.503131297	0.058902841	25.5188252	1.22E-143	8.73E-142
ENSG00000196083	IL1RAP	313.1361763	163.84747	462.4248857	1.498213667	0.131169163	11.4219961	3.25E-30	5.16E-29
ENSG00000121895	TMEM156	24.69613722	12.775553	36.61672181	1.493439269	0.488769398	3.05550895	0.002247	0.006713
ENSG00000186469	GNG2	566.4437632	296.8131	836.0744249	1.488113519	0.106077561	14.028542	1.04E-44	2.31E-43
ENSG00000176510	OR10AC1	16.71590068	8.8813898	24.5504116	1.485022655	0.619155707	2.39846397	0.016464	0.040286
ENSG00000115828	QPCT	451.5600243	238.20025	664.9197983	1.48280243	0.113844064	13.0248551	8.84E-39	1.72E-37
ENSG00000258791	LINC00520	62.62629628	33.016566	92.2360266	1.482344663	0.290501388	5.10271112	3.35E-07	1.72E-06
ENSG00000223797	ENTPD3-AS1	25.28763047	13.31621	37.25905123	1.477700631	0.461099603	3.20473195	0.001352	0.004227
ENSG00000104951	IL4I1	520.2739259	275.60967	764.9381857	1.476595996	0.105858288	13.9487992	3.20E-44	6.99E-43
ENSG00000153823	PID1	114.5120163	60.837492	168.1865404	1.475144745	0.222373191	6.63364472	3.27E-11	2.35E-10
ENSG00000272688	AP005329.3	22.92148154	12.067959	33.77500442	1.474989951	0.480516317	3.06959389	0.002144	0.006433
ENSG00000180758	GPR157	262.1101041	139.10246	385.1177486	1.472392316	0.139921386	10.5229969	6.77E-26	9.48E-25
ENSG00000156453	PCDH1	22581.10163	11966.884	33195.3191	1.472040692	0.037130741	39.6447967	0	0
ENSG00000111981	ULBP1	167.6897741	88.659308	246.7202399	1.469468628	0.178989552	8.20980116	2.22E-16	2.12E-15
ENSG00000164136	IL15	83.87201326	44.574036	123.1699907	1.467125044	0.249292899	5.88514575	3.98E-09	2.43E-08
ENSG00000249464	LINC01091	116.5819294	61.811515	171.3523435	1.462509337	0.216524598	6.75447202	1.43E-11	1.05E-10
ENSG00000105499	PLA2G4C	6449.306979	3452.4581	9446.155888	1.45230554	0.040406352	35.942506	6.63E-283	1.16E-280
ENSG00000145247	OCIAD2	2463.66291	1318.9798	3608.346022	1.452138103	0.060692189	23.9262766	1.63E-126	9.77E-125
ENSG00000082458	DLG3	57.3307982	30.737167	83.92442981	1.451194329	0.324992308	4.46531901	7.99E-06	3.55E-05
ENSG00000138735	PDE5A	167.9572867	89.925334	245.9892398	1.447784241	0.199955894	7.24051797	4.47E-13	3.61E-12
ENSG00000167995	BEST1	249.1112681	133.68078	364.5417559	1.444476846	0.165232648	8.7420789	2.29E-18	2.40E-17
ENSG00000176697	BDNF	88.39406235	47.372516	129.4156091	1.443001159	0.258140362	5.58998658	2.27E-08	1.30E-07
ENSG00000025039	RRAGD	62.57998325	33.83639	91.32357627	1.441704563	0.32259332	4.46910854	7.85E-06	3.49E-05
ENSG00000108582	CPD	20664.1947	11120.756	30207.63373	1.441127583	0.03289111	43.8151094	0	0
ENSG00000228613	AC141930.1	41.74587219	22.45751	61.03423416	1.439456984	0.361777501	3.97884605	6.93E-05	0.000271
ENSG00000121281	ADCY7	286.2719195	154.32617	418.2176643	1.438321802	0.156631406	9.18284422	4.20E-20	4.74E-19
ENSG00000204850	AC011484.1	16.95076371	9.1684075	24.73311995	1.438149086	0.552445598	2.6032411	0.009235	0.024008
ENSG00000184545	DUSP8	401.7031185	217.29198	586.1142563	1.432040066	0.118929172	12.0411169	2.16E-33	3.78E-32
ENSG00000074410	CA12	38.31757524	20.706127	55.92902364	1.431181222	0.367256346	3.89695437	9.74E-05	0.000372
ENSG00000025708	TYMP	520.5463851	281.74536	759.3474105	1.429879603	0.108552078	13.1722914	1.27E-39	2.51E-38
ENSG00000164509	IL31RA	23.55776635	12.877215	34.23831768	1.428498655	0.574296253	2.48738983	0.012868	0.032412
ENSG00000171812	COL8A2	80.19382392	43.495536	116.8921119	1.425001493	0.249225226	5.71772575	1.08E-08	6.34E-08
ENSG00000135932	CAB39	6323.691698	3431.2632	9216.12024	1.424770808	0.043254817	32.9390089	6.08E-238	8.71E-236
ENSG00000131203	IDO1	54.38765092	29.743049	79.03225288	1.423878014	0.326631743	4.3592763	1.30E-05	5.62E-05
ENSG00000051523	CYBA	1508.327903	819.90098	2196.754824	1.422993694	0.077848543	18.2790022	1.22E-74	4.30E-73
ENSG00000285816	AP000944.2	20.69421643	11.20582	30.18261242	1.42180129	0.501257753	2.83646743	0.004562	0.012691
ENSG00000257093	KIAA1147	5091.342062	2774.9073	7407.776774	1.417344948	0.044792623	31.6423746	9.66E-220	1.22E-217
ENSG00000151062	CACNA2D4	55.63540652	30.222628	81.04818469	1.41419371	0.318030442	4.44672435	8.72E-06	3.84E-05
ENSG00000230291	AC078817.1	13.58498302	7.3445419	19.82542418	1.413871167	0.606857331	2.32982465	0.019815	0.047535
ENSG00000152413	HOMER1	81.81611516	44.763785	118.8684457	1.412834903	0.248850409	5.67744658	1.37E-08	7.96E-08
ENSG00000160255	ITGB2	20.58778047	11.30541	29.8701513	1.40702088	0.533329965	2.63818081	0.008335	0.021949
ENSG00000104081	BMF	2214.327091	1214.4016	3214.252595	1.405512016	0.065114016	21.5853991	2.46E-103	1.19E-101
ENSG00000213085	CFAP45	22.48907017	12.464736	32.51340403	1.404563822	0.483977664	2.90212529	0.003706	0.010553
ENSG00000232082	RPS6KA2-IT1	79.50768531	43.403797	115.6115733	1.404432082	0.258716258	5.42846514	5.68E-08	3.12E-07
ENSG00000283959	AP002851.1	16.52516523	9.0873336	23.96299685	1.400778764	0.538501483	2.60125331	0.009288	0.024123
ENSG00000169213	RAB3B	1744.028772	958.19141	2529.866131	1.40074686	0.180422028	7.76372418	8.25E-15	7.28E-14
ENSG00000186827	TNFRSF4	138.8342376	76.483417	201.185058	1.400531301	0.238708945	5.86710858	4.43E-09	2.69E-08
ENSG00000171105	INSR	1506.01467	829.68037	2182.348973	1.393022163	0.06770131	20.5760002	4.50E-94	1.98E-92
ENSG00000139278	GLIPR1	3858.699405	2128.9046	5588.494202	1.392041801	0.047415595	29.3583116	1.87E-189	1.90E-187
ENSG00000105559	PLEKHA4	79.79741999	44.443636	115.1512044	1.389536451	0.300894616	4.618017	3.87E-06	1.78E-05
ENSG00000143341	HMCN1	278.987886	153.87583	404.0999446	1.387651212	0.138199083	10.0409582	1.01E-23	1.29E-22
ENSG00000181458	TMEM45A	89.49103046	49.293553	129.6885077	1.384482702	0.283548596	4.8826999	1.05E-06	5.13E-06
ENSG00000164733	CTSB	132768.2226	73568.495	191967.9506	1.383720179	0.032884078	42.0787286	0	0
ENSG00000134668	SPOCD1	2608.383316	1445.535	3771.23162	1.383701371	0.118914426	11.6361103	2.70E-31	4.46E-30
ENSG00000260807	AC009041.2	19.18088168	10.594754	27.76700903	1.37849365	0.542545657	2.54078829	0.01106	0.028233

ENSG00000168268	NT5DC2	4663.035621	2597.432	6728.63928	1.373921502	0.040768618	33.7004677	5.69E-249	8.92E-247
ENSG00000104856	RELB	1050.53161	585.83017	1515.233051	1.371219354	0.08399323	16.3253556	6.52E-60	1.91E-58
ENSG00000241127	YAE1	367.6447564	205.40261	529.8869009	1.367078597	0.131895541	10.3648583	3.58E-25	4.88E-24
ENSG00000164932	CTHRC1	5622.735187	3145.8162	8099.65414	1.365011698	0.038800181	35.18055	3.97E-271	6.68E-269
ENSG00000172216	CEBPB	451.7486139	253.24592	650.2513037	1.362425159	0.135907836	10.0246255	1.19E-23	1.52E-22
ENSG00000108960	MMD	504.2329145	283.16223	725.3036039	1.360937552	0.108429223	12.5513908	3.91E-36	7.21E-35
ENSG00000136560	TANK	4305.295224	2413.2401	6197.350305	1.360844418	0.042165647	32.2737709	1.63E-228	2.25E-226
ENSG00000137563	GGH	4287.74818	2404.1841	6171.312247	1.359570148	0.04294823	31.6560225	6.27E-220	7.97E-218
ENSG00000141424	SLC39A6	1441.837114	810.99235	2072.681883	1.354493597	0.067337508	20.1149944	5.45E-90	2.30E-88
ENSG00000110911	SLC11A2	2951.877285	1662.0842	4241.670361	1.350465279	0.048814758	27.6651022	1.84E-168	1.61E-166
ENSG00000205562	AL049775.1	124.2433416	69.866466	178.6202176	1.347573262	0.203330765	6.62749318	3.41E-11	2.45E-10
ENSG00000164283	ESM1	20431.63507	11528.355	29334.91555	1.347362014	0.03951953	34.093574	9.18E-255	1.45E-252
ENSG00000141198	TOM1L1	80.65719337	45.398402	115.9159845	1.346780698	0.247348552	5.44486995	5.18E-08	2.86E-07
ENSG00000182263	FIGN	17.65525819	9.8764955	25.43402085	1.345909407	0.532459701	2.5277207	0.011481	0.029192
ENSG00000130707	ASS1	287.8401717	162.79193	412.8884144	1.344824412	0.139547542	9.63703401	5.58E-22	6.75E-21
ENSG00000182107	TMEM30B	34.04944205	19.233417	48.86546751	1.344499005	0.38317536	3.50883471	0.00045	0.001543
ENSG00000160326	SLC2A6	3221.2354	1822.2126	4620.258206	1.342698372	0.046010003	29.1827488	3.21E-187	3.16E-185
ENSG00000006118	TMEM132A	7915.127746	4479.8247	11350.43082	1.342111051	0.037498932	35.7906469	1.54E-280	2.68E-278
ENSG00000057019	DCBLD2	8645.90687	4903.5602	12388.25354	1.337038265	0.128794	10.3812155	3.02E-25	4.12E-24
ENSG00000163132	MSX1	426.6233857	242.14591	611.1008617	1.335765791	0.114027179	11.7144509	1.07E-31	1.80E-30
ENSG00000171608	PIK3CD	1142.109489	648.4034	1635.815581	1.335351063	0.072768748	18.350612	3.26E-75	1.16E-73
ENSG00000156273	BACH1	1901.565649	1078.6847	2724.446623	1.334475725	0.063183764	21.120548	5.15E-99	2.39E-97
ENSG00000011422	PLAUR	2559.94115	1454.2919	3665.590409	1.332325576	0.050526146	26.3690322	3.11E-153	2.40E-151
ENSG00000147650	LRP12	1386.844689	789.0388	1984.650573	1.330691551	0.064210472	20.7239024	2.11E-95	9.40E-94
ENSG00000080709	KCNN2	509.4621621	290.58404	728.3402844	1.330037303	0.113662227	11.701665	1.25E-31	2.09E-30
ENSG00000270062	AL606834.2	29.128789	16.63841	41.61916766	1.327884744	0.475848216	2.79056367	0.005262	0.01444
ENSG00000271425	NBPF10	40.6993547	23.329819	58.06889023	1.327360183	0.355677267	3.73192302	0.00019	0.000691
ENSG00000234518	PTGES3P1	58.77018873	33.439366	84.10101193	1.327197015	0.295706096	4.48823015	7.18E-06	3.20E-05
ENSG00000111266	DUSP16	1517.490779	864.50114	2170.480413	1.326515512	0.063575705	20.8651327	1.11E-96	5.03E-95
ENSG00000226835	AC097059.1	18.84682124	10.630206	27.06343661	1.324165808	0.533192218	2.48346799	0.013011	0.032735
ENSG00000135749	PCNX2	120.8836472	69.160451	172.6068438	1.322203835	0.208416893	6.34403389	2.24E-10	1.51E-09
ENSG00000104447	TRPS1	31.70781975	18.17442	45.2412193	1.31770679	0.396159145	3.32620566	0.00088	0.002852
ENSG00000125347	IRF1	2690.067291	1542.3124	3837.822146	1.317504538	0.060734171	21.6929698	2.39E-104	1.16E-102
ENSG00000247934	AC022364.1	24.67376114	14.069673	35.27784926	1.315235282	0.451864469	2.91068533	0.003606	0.010297
ENSG00000203688	LINC02487	31.45441846	18.08599	44.82284703	1.315182911	0.425792128	3.08879104	0.00201	0.006065
ENSG00000219507	FTH1P8	74.72759136	42.904467	106.5507153	1.31468006	0.25511927	5.1531978	2.56E-07	1.33E-06
ENSG00000152661	GJA1	23729.0054	13631.384	33826.6264	1.31133157	0.034353166	38.172073	0	0
ENSG00000134955	SLC37A2	168.0298468	97.083286	238.9764072	1.309286148	0.210884398	6.20854915	5.35E-10	3.51E-09
ENSG00000129116	PALLD	4869.595534	2803.3173	6935.873806	1.30822573	0.043036725	30.397892	5.86E-203	6.74E-201
ENSG00000118985	ELL2	1659.881708	954.80286	2364.960553	1.308052759	0.062348379	20.9797395	1.00E-97	4.59E-96
ENSG00000134352	IL6ST	32917.17833	18963.195	46871.16188	1.305374714	0.030580885	42.6859685	0	0
ENSG00000197122	SRC	1137.123858	655.66592	1618.581798	1.303453582	0.217689652	5.98766901	2.13E-09	1.33E-08
ENSG00000159128	IFNGR2	3545.634764	2044.2911	5046.978427	1.302004792	0.050221265	25.9253685	3.45E-148	2.58E-146
ENSG00000270164	LINC01480	46.83948301	27.091014	66.58795152	1.301245625	0.333141565	3.905984	9.38E-05	0.00036
ENSG00000132359	RAP1GAP2	371.8568565	215.85413	527.859582	1.288311524	0.115438968	11.1601095	6.39E-29	9.74E-28
ENSG00000112276	BVES	339.2137093	197.19964	481.2277813	1.28814225	0.140506627	9.16783984	4.83E-20	5.43E-19
ENSG00000135916	ITM2C	158.7622001	92.845228	224.6791725	1.284782514	0.207113117	6.20328897	5.53E-10	3.63E-09
ENSG00000147454	SLC25A37	1375.254798	800.66343	1949.846166	1.284570616	0.08362816	15.3605032	3.01E-53	7.78E-52
ENSG00000140416	TPM1	21858.84363	12740.545	30977.14234	1.282176408	0.029634118	43.2668991	0	0
ENSG00000168314	MOBP	78.82850476	45.910964	111.7460453	1.281707382	0.269021119	4.76433741	1.89E-06	9.02E-06
ENSG00000103811	CTSH	638.5232248	373.03102	904.0154252	1.277935686	0.094172045	13.5702234	6.01E-42	1.25E-40
ENSG00000178031	ADAMTSL1	2963.189141	1731.1501	4195.228148	1.277183377	0.147541564	8.65643103	4.87E-18	5.03E-17
ENSG00000172331	BPGM	973.008028	569.01934	1376.996712	1.275896822	0.07442233	17.1440053	6.97E-66	2.19E-64
ENSG00000123358	NR4A1	88.00572563	51.523379	124.4880721	1.275077864	0.271063287	4.70398584	2.55E-06	1.20E-05
ENSG00000048052	HDAC9	2476.199736	1448.1479	3504.251578	1.27444942	0.062955853	20.2435414	4.05E-91	1.73E-89
ENSG00000073150	PANX2	146.1888197	85.717787	206.6598524	1.27344009	0.197743495	6.43985832	1.20E-10	8.24E-10
ENSG00000105464	GRIN2D	55.20113437	32.28078	78.1214888	1.2705622	0.356191868	3.56707245	0.000361	0.001254
ENSG00000110697	PITPNM1	772.8294551	452.72505	1092.933855	1.269936268	0.089688138	14.1594675	1.63E-45	3.68E-44
ENSG00000196126	HLA-DRB1	168.029844	98.529038	237.5306497	1.267684099	0.179289549	7.07059675	1.54E-12	1.21E-11
ENSG00000122779	TRIM24	3061.993401	1798.4617	4325.525095	1.267348508	0.04741702	26.7277129	2.24E-157	1.80E-155
ENSG00000204262	COL5A2	24570.59069	14429.015	34712.16622	1.266378756	0.028137796	45.0063243	0	0
ENSG00000157111	TMEM171	100.2342228	59.140323	141.3281228	1.263849494	0.240572606	5.25350544	1.49E-07	7.92E-07
ENSG00000149294	NCAM1	21.72432042	12.686628	30.7620126	1.258063679	0.481661504	2.6119249	0.009003	0.023473
ENSG00000135905	DOCK10	720.821334	424.99374	1016.648927	1.257082317	0.110143915	11.4130891	3.60E-30	5.72E-29
ENSG00000100968	NFATC4	973.3218875	574.95423	1371.689546	1.254799493	0.073300129	17.1189552	1.08E-65	3.39E-64
ENSG00000124508	BTN2A2	726.2440133	429.40813	1023.079902	1.253049123	0.086445533	14.4952444	1.30E-47	3.04E-46
ENSG00000136842	TMOD1	354.372142	209.97188	498.7724011	1.250566358	0.129839834	9.63160777	5.88E-22	7.11E-21
ENSG00000116771	AGMAT	45.74673938	27.14424	64.34923847	1.248675248	0.368468795	3.38882224	0.000702	0.002324
ENSG00000108576	SLC6A4	206.1541372	121.85521	290.4530692	1.247446664	0.165453814	7.53954614	4.72E-14	4.01E-13
ENSG00000188015	S100A3	32.78270004	19.576721	45.98729866	1.246952625	0.408405783	3.05321981	0.002264	0.006759
ENSG00000124216	SNAI1	521.8090508	309.85182	733.7662789	1.246640861	0.109420178	11.3931533	4.52E-30	7.17E-29
ENSG00000183087	GAS6	6177.361205	3664.6614	8690.060987	1.246056471	0.03762113	33.121187	1.47E-240	2.16E-238
ENSG00000167996	FTH1	197569.7143	117193.92	277945.5058	1.245901274	0.038633817	32.2489821	3.64E-228	4.94E-226
ENSG00000152894	PTPRK	7381.766411	4380.3493	10383.18355	1.244594413	0.035425808	35.1324213	2.16E-270	3.60E-268
ENSG00000166073	GPR176	1868.077244	1108.6346	2627.519842	1.244158656	0.066598205	18.6815644	6.99E-78	2.57E-76
ENSG00000152952	PLOD2	13262.11543	7876.2531	18647.97775	1.243631202	0.030295387	41.0501833	0	0
ENSG00000172260	NEGR1	129.5542112	77.152101	181.9563213	1.242624663	0.211313705	5.88047359	4.09E-09	2.49E-08
ENSG00000064932	SBNO2	4160.954674	2473.8598	5848.049565	1.242218003	0.046132372	26.9272522	1.05E-159	8.56E-158
ENSG00000005187	ACSM3	340.7574285	202.88444	478.6304194	1.238358359	0.139874297	8.8533661	8.49E-19	9.03E-18
ENSG00000146592	CREB5	386.7001797	230.11	543.290362	1.235482024	0.117054113	10.5547937	4.83E-26	6.81E-25
ENSG00000127533	F2RL3	1193.72709	713.84616	1673.608023	1.228369254	0.076514062	16.0541633	5.35E-58	1.50E-56
ENSG00000164292	RHOBTB3	6065.425531	3633.743	8497.108023	1.225076827	0.042017971	29.1560202	7.01E-187	6.85E-185
ENSG00000060642	PIGV	382.1905411	228.96109	535.4199939	1.222531815	0.118017987	10.3588601	3.81E-25	5.18E-24
ENSG00000164220	F2RL2	66.21173496	39.688224	92.73524562	1.22249473	0.288997052	4.23012873	2.34E-05	9.76E-05
ENSG00000148483	TMEM236	31.31730232	18.785486	43.84911833	1.222347105	0.429932566	2.84311355	0.004468	0.012451

ENSG00000065621	GSTO2	40.55065652	24.270217	56.83109609	1.221531653	0.347556561	3.51462694	0.00044	0.001512
ENSG00000077150	NFKB2	2297.788157	1380.242	3215.33429	1.220533697	0.050828302	24.0128759	2.04E-127	1.23E-125
ENSG00000238000	AC116347.1	40.22445764	24.207905	56.24100981	1.216428047	0.365070089	3.33203975	0.000862	0.002798
ENSG00000159217	IGF2BP1	121.9349836	73.680888	170.1890792	1.21290257	0.233270874	5.19954571	2.00E-07	1.05E-06
ENSG00000160200	CBS	52.07878309	31.355342	72.80222405	1.212616551	0.308101014	3.93577592	8.29E-05	0.00032
ENSG00000197457	STMN3	23.25972276	13.934385	32.58506029	1.212366641	0.504062302	2.40519205	0.016164	0.039691
ENSG00000204991	SPIRE2	43.47338427	26.311185	60.63558323	1.210488754	0.355198903	3.40791805	0.000655	0.002178
ENSG00000101335	MYL9	5971.785069	3607.1786	8336.391519	1.208582582	0.040801876	29.6207601	8.08E-193	8.59E-191
ENSG00000163638	ADAMTS9	5124.599419	3100.644	7148.554823	1.204664939	0.040228975	29.9452062	5.08E-197	5.60E-195
ENSG00000075426	FOSL2	9344.708989	5662.6392	13026.77878	1.20278281	0.03648056	32.970514	2.15E-238	3.13E-236
ENSG00000184922	FMNL1	507.6980865	307.4277	707.9684683	1.201997947	0.102392111	11.7391656	8.03E-32	1.35E-30
ENSG00000162654	GBP4	1621.390288	983.62853	2259.152045	1.201021396	0.075195023	15.9720862	2.00E-57	5.54E-56
ENSG00000223361	FTH1P10	97.31454261	58.877501	135.7515845	1.200977001	0.229401741	5.23525669	1.65E-07	8.73E-07
ENSG00000143995	MEIS1	137.8347134	83.42565	192.243777	1.199483384	0.192068102	6.24509418	4.24E-10	2.80E-09
ENSG00000105810	CDK6	3047.575725	1849.4828	4245.668602	1.197335655	0.05000424	23.9446824	1.05E-126	6.30E-125
ENSG00000149289	ZC3H12C	2230.210617	1354.6745	3105.746705	1.196854622	0.052175919	22.9388317	1.90E-116	1.06E-114
ENSG00000131323	TRAF3	2528.452029	1535.6162	3521.287873	1.196610894	0.053851228	22.2206799	2.17E-109	1.13E-107
ENSG00000232926	AC000078.1	33.918506	20.572321	47.26469074	1.196511928	0.41560657	2.87895335	0.00399	0.011277
ENSG00000106006	HOXA6	38.8838078	23.62444	54.14317522	1.1961122	0.35209543	3.39712503	0.000681	0.00226
ENSG00000155380	SLC16A1	9180.543602	5583.0779	12778.00931	1.19393432	0.03552234	33.6108015	1.17E-247	1.80E-245
ENSG00000187583	PLEKHN1	74.08947482	45.403537	102.7754129	1.191656082	0.281716667	4.22998077	2.34E-05	9.76E-05
ENSG00000050327	ARHGEF5	56.11171115	34.056553	78.16866937	1.191229676	0.322703928	3.69140123	0.000223	0.000803
ENSG00000166825	ANPEP	15062.94526	9175.6167	20950.27381	1.191119179	0.109381889	10.8895466	1.29E-27	1.90E-26
ENSG00000140937	CDH11	42.50219204	26.077887	58.92649664	1.1893153	0.355555137	3.34495322	0.000823	0.002683
ENSG00000196639	HRH1	1553.727199	948.54319	2158.911207	1.18814315	0.067952058	17.4850209	1.86E-68	6.15E-67
ENSG00000130066	SAT1	8593.467477	5244.747	11942.18793	1.187654196	0.038156381	31.1259651	1.07E-212	1.31E-210
ENSG00000104635	SLC39A14	4400.478482	2688.4784	6112.478604	1.185596431	0.048564528	24.4128065	1.25E-131	7.93E-130
ENSG00000253873	PCDHGA11	1019.970762	622.7021	1417.239428	1.185055039	0.073901257	16.0356546	7.20E-58	2.00E-56
ENSG00000082126	MPP4	652.5162278	398.61585	906.4166035	1.183940814	0.109969131	10.7661196	4.98E-27	7.21E-26
ENSG00000196839	ADA	1202.367612	735.95878	1668.776448	1.182870578	0.070667285	16.7385881	6.86E-63	2.08E-61
ENSG00000153234	NR4A2	41.74268543	25.437888	58.04748264	1.182823528	0.360821652	3.278139	0.001045	0.003344
ENSG00000168874	ATOH8	748.2926043	458.50964	1038.075568	1.182577083	0.089075173	13.2761694	3.18E-40	6.41E-39
ENSG00000086062	B4GALT1	6212.129161	3801.1952	8623.063081	1.182195741	0.038010017	31.1022159	2.25E-212	2.73E-210
ENSG00000279822	AC016397.2	18.11059704	11.062435	25.15875899	1.181965534	0.509419812	2.32021902	0.020329	0.048562
ENSG00000280088	AC126474.2	34.92272759	21.271077	48.5743786	1.18121605	0.39158817	3.01647532	0.002557	0.007537
ENSG00000197461	PDGFA	1770.880119	1085.2769	2456.483352	1.179234316	0.058496633	20.1590119	2.24E-90	9.49E-89
ENSG00000273038	AL365203.2	411.4870236	252.34157	570.6324798	1.178052402	0.109192015	10.7888145	3.89E-27	5.66E-26
ENSG00000147394	ZNF185	1314.116396	805.92502	1822.30777	1.177584295	0.086002193	13.6924914	1.13E-42	2.40E-41
ENSG00000134871	COL4A2	218087.9431	133746.89	302428.9983	1.177090627	0.028817744	40.8460363	0	0
ENSG00000112837	TBX18	1768.052197	1087.2393	2448.865048	1.171349509	0.060842443	19.2521775	1.35E-82	5.20E-81
ENSG00000106799	TGFBR1	856.8644091	528.33928	1185.389542	1.162398948	0.084942685	13.6845091	1.26E-42	2.67E-41
ENSG00000103257	SLC7A5	601.9093409	371.66111	832.1575683	1.161134817	0.10506839	11.0512288	2.16E-28	3.24E-27
ENSG00000152760	TCTEX1D1	48.12850738	29.640151	66.61686336	1.160728956	0.321279888	3.61282794	0.000303	0.001066
ENSG00000259207	ITGB3	1602.398308	991.08643	2213.710189	1.159135202	0.067391095	17.2001242	2.65E-66	8.41E-65
ENSG00000144749	LRIG1	1655.062383	1024.9029	2285.22189	1.157811751	0.059234015	19.5464001	4.43E-85	1.75E-83
ENSG00000161681	SHANK1	59.19427689	36.481314	81.90723987	1.157448993	0.304544924	3.80058541	0.000144	0.000536
ENSG00000070961	ATP2B1	4038.730149	2500.9663	5576.494023	1.155841111	0.055954121	20.6569435	8.45E-95	3.74E-93
ENSG00000134294	SLC38A2	30981.5386	19212.401	42750.6764	1.153973141	0.02872883	40.1677739	0	0
ENSG00000129667	RHBDF2	2438.236363	1513.8404	3362.63228	1.153459641	0.055701081	20.7080297	2.93E-95	1.30E-93
ENSG00000275993	SIK1B	185.6511019	115.19505	256.1071541	1.152920172	0.173499599	6.64508841	3.03E-11	2.18E-10
ENSG00000158315	RHBDL2	24.77598851	15.345278	34.20669894	1.150312435	0.457923007	2.51202149	0.012004	0.0304
ENSG00000154175	ABI3BP	920.6455679	572.4154	1268.875735	1.14839175	0.252571092	4.54680597	5.45E-06	2.46E-05
ENSG00000234975	FTH1P2	110.1648213	68.585083	151.7445596	1.14675009	0.214786093	5.33903324	9.34E-08	5.05E-07
ENSG00000169554	ZEB2	1898.091579	1182.7375	2613.445613	1.146010446	0.059882996	19.1374936	1.23E-81	4.69E-80
ENSG00000136205	TNS3	3871.340019	2413.5247	5329.15532	1.142464299	0.063635958	17.953125	4.54E-72	1.57E-70
ENSG00000091986	CCDC80	1075.824466	671.28563	1480.363302	1.142381124	0.079129737	14.4368119	3.04E-47	7.05E-46
ENSG00000164647	STEAP1	784.915589	490.1005	1079.730676	1.138574878	0.088244476	12.902506	4.36E-38	8.40E-37
ENSG00000220323	HIST2H2BD	33.6388459	21.184719	46.09297233	1.137776389	0.399887066	2.84524429	0.004438	0.012379
ENSG00000131015	ULBP2	466.4913641	292.06707	640.9156588	1.135688632	0.104635861	10.8537228	1.91E-27	2.80E-26
ENSG00000183346	CABCOCO1	23.90184545	15.02355	32.78014099	1.135609381	0.491888135	2.30867407	0.020962	0.049883
ENSG00000100228	RAB36	142.1967885	89.001475	195.3921021	1.135455766	0.238591415	4.75899673	1.95E-06	9.25E-06
ENSG00000159200	RCAN1	1851.582662	1158.3652	2544.800102	1.135360332	0.056540011	20.080653	1.09E-89	4.55E-88
ENSG00000017427	IGF1	166.018616	104.17434	227.8628931	1.131069591	0.198135674	5.70856106	1.14E-08	6.69E-08
ENSG00000169902	TPST1	672.6235117	421.63417	923.6128564	1.129843898	0.087459784	12.9184392	3.54E-38	6.84E-37
ENSG00000160013	PTGIR	36.98000059	23.229489	50.73051181	1.127989359	0.379690281	2.97081441	0.00297	0.008633
ENSG00000010030	ETV7	57.47694413	36.03289	78.92099868	1.124184389	0.28990268	3.87779923	0.000105	0.0004
ENSG00000198814	GK	153.1472063	96.722207	209.5722053	1.123423602	0.189104964	5.94074094	2.84E-09	1.75E-08
ENSG00000272695	GAS6-DT	53.03188467	33.203994	72.85977487	1.122650448	0.314010915	3.57519562	0.00035	0.001219
ENSG00000260401	AP002761.4	79.6511893	50.367608	108.9347707	1.120696029	0.256160466	4.37497655	1.21E-05	5.25E-05
ENSG00000020577	SAMD4A	7489.072677	4721.3381	10256.80726	1.119626675	0.034359247	32.5858909	6.50E-233	9.16E-231
ENSG00000164176	EDIL3	662.497379	416.89841	908.0963501	1.118111912	0.100534793	11.1216414	9.84E-29	1.49E-27
ENSG00000128203	ASPHD2	147.9266196	93.458516	202.3947235	1.116256009	0.184811561	6.03996851	1.54E-09	9.75E-09
ENSG00000188227	ZNF793	314.8157907	199.24154	430.3900409	1.116250627	0.142967664	7.80771396	5.82E-15	5.18E-14
ENSG00000133424	LARGE1	133.2429817	84.354493	182.1300652	1.115014017	0.200198453	5.56954446	2.55E-08	1.45E-07
ENSG00000163297	ANTXR2	3936.885005	2488.6611	5385.108957	1.114200713	0.041600686	26.7832293	5.07E-158	4.08E-156
ENSG00000113916	BCL6	649.3192477	410.04297	888.5955219	1.112689142	0.098378405	11.3102987	1.17E-29	1.83E-28
ENSG00000075275	CELSR1	521.7373406	329.67976	713.7949175	1.111669475	0.106213621	10.4663551	1.23E-25	1.70E-24
ENSG00000249992	TMEM158	24.24230825	15.317924	33.16669241	1.110294527	0.478284041	2.32141245	0.020265	0.04844
ENSG00000117602	RCAN3	55.41718551	35.001	75.83337086	1.108769652	0.323977948	3.42236149	0.000621	0.002075
ENSG00000186866	POFUT2	3264.725191	2069.3499	4460.100451	1.108767149	0.04572365	24.2493141	6.72E-130	4.15E-128
ENSG00000166348	USP54	719.6543952	456.09278	983.2160081	1.108360096	0.083592915	13.2590195	4.00E-40	8.03E-39
ENSG00000150093	ITGB1	114406.3989	72670.32	156142.4773	1.103367837	0.029042453	37.991551	0	0
ENSG00000100321	SYNGR1	226.3725072	143.94803	308.7969854	1.101869571	0.15983315	6.89387382	5.43E-12	4.09E-11
ENSG00000231856	AL162377.1	42.75299188	27.307376	58.19860763	1.098320283	0.338752694	3.24224812	0.001186	0.003755
ENSG00000113248	PCDHB15	120.5944836	76.801096	164.3878713	1.096781189	0.218667104	5.01575761	5.28E-07	2.67E-06

ENSG00000196932	TMEM26	55.94017901	35.628852	76.25150564	1.095677317	0.323569852	3.38621571	0.000709	0.002343
ENSG00000165490	DDIAS	225.4779568	143.97074	306.9851695	1.095361551	0.157768715	6.94283114	3.84E-12	2.93E-11
ENSG00000261716	AC239868.1	72.15111417	46.000383	98.30184567	1.093985288	0.267440866	4.0905689	4.30E-05	0.000173
ENSG00000267325	LINC01415	55.50643643	35.142065	75.8708076	1.093841813	0.335057272	3.26464131	0.001096	0.003489
ENSG00000173926	3-Mar	2225.972338	1422.5995	3029.345168	1.090761137	0.061010047	17.8783854	1.74E-71	5.93E-70
ENSG00000109107	ALDOC	150.6978596	96.003356	205.3923632	1.090183214	0.186941319	5.83168674	5.49E-09	3.32E-08
ENSG00000078401	EDN1	18598.22889	11893.206	25303.2519	1.089495417	0.035472043	30.7141994	3.68E-207	4.35E-205
ENSG00000130821	SLC6A8	2339.076329	1496.1302	3182.022467	1.089331717	0.06491463	16.7809894	3.36E-63	1.02E-61
ENSG00000103005	USB1	3241.852669	2073.4648	4410.240545	1.089322359	0.05125385	21.253474	3.06E-100	1.44E-98
ENSG00000162645	GBP2	5932.256516	3800.8641	8063.648903	1.085759853	0.037557357	28.9093786	9.10E-184	8.54E-182
ENSG00000234814	SVIL2P	48.35851314	30.915262	65.80176389	1.082623004	0.32101465	3.37250342	0.000745	0.00245
ENSG00000188060	RAB42	23.99706713	15.372385	32.62174922	1.080800948	0.440678473	2.45258395	0.014183	0.035377
ENSG00000237989	LINC01679	88.39072605	56.975967	119.8054855	1.078235645	0.261881635	4.1172633	3.83E-05	0.000155
ENSG00000129757	CDKN1C	77.07427069	49.576867	104.5716745	1.077307101	0.267589291	4.0259724	5.67E-05	0.000225
ENSG00000204580	DDR1	1342.54519	864.05193	1821.038452	1.075961106	0.0661598	16.2630646	1.80E-59	5.25E-58
ENSG00000151789	ZNF385D	68.20413804	43.854789	92.55348718	1.07586497	0.28838523	3.73065212	0.000191	0.000694
ENSG00000161638	ITGA5	98270.8381	63247.357	133294.3193	1.075567333	0.027380277	39.2825588	0	0
ENSG00000128340	RAC2	5899.556198	3798.0955	8001.016895	1.075129659	0.0492011	21.8517401	7.48E-106	3.71E-104
ENSG00000256802	AC022613.1	92.07165434	59.201302	124.9420065	1.075005838	0.242869911	4.42626193	9.59E-06	4.21E-05
ENSG00000010278	CD9	17289.90917	11131.046	23448.77264	1.074960548	0.108651848	9.89362413	4.44E-23	5.58E-22
ENSG00000154640	BTG3	993.9199461	640.11663	1347.723262	1.074202045	0.074041884	14.5080324	1.08E-47	2.54E-46
ENSG00000227825	SLC9A7P1	69.31578694	44.490395	94.14117917	1.073644114	0.281007289	3.82069846	0.000133	0.000497
ENSG00000103489	XYLT1	275.7195002	177.58267	373.8563267	1.071702384	0.134861298	7.94670077	1.92E-15	1.75E-14
ENSG00000165661	QSOX2	1647.698532	1064.3582	2231.03882	1.069753372	0.065933926	16.2246271	3.38E-59	9.77E-58
ENSG00000141458	NPC1	4546.979599	2935.282	6158.677183	1.068690171	0.042749616	24.9988252	6.30E-138	4.24E-136
ENSG00000162636	FAM102B	113.3618416	73.299661	153.4240219	1.068244386	0.21147583	5.05137815	4.39E-07	2.24E-06
ENSG00000100368	CSF2RB	2731.718211	1764.489	3698.947384	1.067990671	0.049145727	21.7310992	1.04E-104	5.09E-103
ENSG00000006327	TNFRSF12A	2944.952986	1904.0667	3985.839291	1.067242112	0.055346261	19.2830029	7.46E-83	2.88E-81
ENSG00000226564	FTH1P20	63.56746744	40.999528	86.13540695	1.067072288	0.287671591	3.70934191	0.000208	0.000752
ENSG00000104320	NBN	2599.49373	1680.8936	3518.093869	1.064750746	0.048571078	21.9214973	1.62E-106	8.10E-105
ENSG00000221963	APOL6	1738.330508	1125.1286	2351.532428	1.063729551	0.059426558	17.8999019	1.18E-71	4.04E-70
ENSG00000269378	AC022149.1	371.3391118	240.91766	501.760566	1.062343679	0.126615441	8.39031693	4.85E-17	4.77E-16
ENSG00000273382	AL356488.3	44.9363417	29.248261	60.62442217	1.061926106	0.341258884	3.11179037	0.00186	0.005655
ENSG00000272463	AL357054.4	41.0502405	26.404652	55.69582915	1.061565051	0.401669124	2.64288437	0.00822	0.021684
ENSG00000151726	ACSL1	1473.533923	955.4594	1991.608445	1.05945986	0.073074337	14.4983849	1.24E-47	2.91E-46
ENSG00000232187	FTH1P7	51.88572044	33.654739	70.11670185	1.058912383	0.300346201	3.52563935	0.000422	0.001454
ENSG00000164099	PRSS12	33.11055042	21.532566	44.68853438	1.058207251	0.424529073	2.49266144	0.012679	0.031953
ENSG00000050344	NFE2L3	3620.434596	2348.7384	4892.13077	1.058159632	0.042572699	24.8553571	2.26E-136	1.51E-134
ENSG00000142178	SIK1	218.5748465	141.38398	295.7657088	1.057686649	0.158122776	6.6890215	2.25E-11	1.63E-10
ENSG00000188921	HACD4	120.5296094	78.354977	162.7042418	1.057139391	0.206986514	5.10728632	3.27E-07	1.68E-06
ENSG00000196923	PDLIM7	5436.686826	3531.5095	7341.864121	1.056601541	0.044388085	23.8037196	3.06E-125	1.79E-123
ENSG00000278384	AL354822.1	38.89273526	25.314253	52.47121714	1.055216329	0.362352481	2.91212668	0.00359	0.010256
ENSG00000225093	RPL3P7	50.42538422	32.937812	67.91295612	1.053240253	0.335147072	3.14262108	0.001674	0.005137
ENSG00000104723	TUSC3	741.9852953	481.79759	1002.172998	1.053089608	0.091738238	11.4792875	1.68E-30	2.71E-29
ENSG00000125744	RTN2	434.2312523	282.14708	586.3154279	1.051971866	0.111591896	9.42695579	4.22E-21	4.96E-20
ENSG00000120549	KIAA1217	1179.531073	767.71001	1591.352132	1.051614112	0.072376267	14.5298197	7.84E-48	1.85E-46
ENSG00000279227	AC009303.4	42.16514708	27.4596	56.87069403	1.050785732	0.338972525	3.09991417	0.001936	0.005859
ENSG00000117479	SLC19A2	143.4246621	93.866699	192.982625	1.047810167	0.199356078	5.25597302	1.47E-07	7.83E-07
ENSG00000227076	AL158166.1	46.93019026	30.574278	63.28610274	1.047344614	0.316649467	3.30758369	0.000941	0.003036
ENSG00000140859	KIFC3	3508.686577	2292.5302	4724.842965	1.044703236	0.045972339	22.7246047	2.56E-114	1.40E-112
ENSG00000008513	ST3GAL1	8695.549921	5681.7611	11709.33871	1.043106026	0.032793387	31.8084263	4.95E-222	6.43E-220
ENSG00000111424	VDR	44.09472478	28.731403	59.45804682	1.036721063	0.34657762	2.99130989	0.002778	0.008129
ENSG00000182489	XKRX	50.53589021	32.979535	68.09224513	1.036383809	0.339450969	3.05311784	0.002265	0.00676
ENSG00000143878	RHOB	25534.361	16737.751	34330.9708	1.036333233	0.037716416	27.4769804	3.31E-166	2.81E-164
ENSG00000162511	LAPTM5	7645.019023	5012.8661	10277.17191	1.03554138	0.038023323	27.2343734	2.55E-163	2.15E-161
ENSG00000170801	HTRA3	4189.87881	2748.5212	5631.236404	1.034124062	0.042493114	24.3362738	8.10E-131	5.07E-129
ENSG00000183853	KIRREL1	4274.972658	2805.4101	5744.535185	1.032455789	0.046707003	22.1049462	2.83E-108	1.44E-106
ENSG00000139725	RHOF	194.6708372	128.16647	261.1752007	1.03165882	0.169157118	6.09882003	1.07E-09	6.84E-09
ENSG00000175505	CLCF1	119.6892124	78.91489	160.4635343	1.030505446	0.220661237	4.67007917	3.01E-06	1.40E-05
ENSG00000134070	IRAK2	2893.42788	1903.1643	3883.691505	1.030092079	0.051047635	20.1790361	1.50E-90	6.35E-89
ENSG00000234745	HLA-B	41626.88081	27369.125	55884.63666	1.029996522	0.041391368	24.8843315	1.10E-136	7.35E-135
ENSG00000237264	FTH1P11	53.43958993	35.281005	71.59817448	1.027274632	0.304467459	3.37400468	0.000741	0.002439
ENSG00000117758	STX12	6959.949874	4580.5801	9339.319606	1.02700026	0.037973809	27.0449628	4.38E-161	3.60E-159
ENSG00000141682	PMAIP1	1876.56178	1236.2617	2516.861817	1.026539046	0.064981149	15.79749	3.24E-56	8.80E-55
ENSG00000153790	C7orf31	78.1705763	51.470647	104.8705052	1.025904123	0.255331376	4.01793207	5.87E-05	0.000232
ENSG00000177283	FZD8	1467.06782	966.61066	1967.524978	1.024366236	0.069678574	14.7013088	6.32E-49	1.52E-47
ENSG00000118596	SLC16A7	914.8204669	603.32297	1226.317959	1.023562279	0.083669716	12.2333663	2.06E-34	3.69E-33
ENSG00000197568	HHLA3	56.2111158	36.974866	75.44736513	1.022331705	0.313446424	3.26158357	0.001108	0.003525
ENSG00000197329	PELI1	2129.96953	1406.1344	2853.804644	1.021532971	0.059813189	17.0787244	2.14E-65	6.68E-64
ENSG00000054219	LY75	518.0796053	343.4125	692.7467077	1.013430105	0.099384317	10.1970827	2.04E-24	2.69E-23
ENSG00000103260	METRN	1016.575947	674.37765	1358.774247	1.012120133	0.090210717	11.219511	3.27E-29	5.06E-28
ENSG00000148488	ST8SIA6	93.98913608	62.208046	125.7702262	1.011815963	0.228570445	4.42671388	9.57E-06	4.20E-05
ENSG00000185022	MAFF	5852.801946	3888.2077	7817.3962	1.008615229	0.04012598	25.1362143	2.00E-139	1.38E-137
ENSG00000157224	CLDN12	516.603107	343.12267	690.083543	1.00826148	0.099306682	10.1530075	3.21E-24	4.20E-23
ENSG00000160606	TLCD1	69.91271951	46.377313	93.44812612	1.008159668	0.311896004	3.2323584	0.001228	0.003876
ENSG00000169604	ANTXR1	4404.231726	2925.0904	5883.373078	1.007591724	0.050145418	20.0933958	8.43E-90	3.53E-88
ENSG00000115594	IL1R1	528.8445812	351.93306	705.7561017	1.00404745	0.103903983	9.66322387	4.32E-22	5.25E-21
ENSG00000270157	AC004918.3	166.8278545	111.42467	222.2310359	1.002228618	0.199229793	5.03051577	4.89E-07	2.48E-06
ENSG00000173530	TNFRSF10D	2765.51809	1844.2043	3686.831922	0.998518344	0.05679625	17.5807091	3.46E-69	1.16E-67
ENSG00000113742	CPEB4	1216.910016	811.86382	1621.956217	0.997700573	0.066709368	14.955929	1.42E-50	3.52E-49
ENSG00000132669	RIN2	4492.119343	3000.0299	5984.208812	0.996884266	0.044513907	22.3948949	4.41E-111	2.33E-109
ENSG00000158966	CACHD1	1076.290133	719.71976	1432.860503	0.994751318	0.075627301	13.1533361	1.63E-39	3.22E-38
ENSG00000158246	TENT5B	40.35082248	27.055563	53.64608203	0.993578134	0.377947643	2.62887771	0.008567	0.02248
ENSG00000139926	FRMD6	12340.31542	8254.3581	16426.27277	0.993000582	0.029928533	33.1790599	2.16E-241	3.22E-239
ENSG00000273604	EPOP	116.5310712	77.923489	155.1386536	0.992671153	0.239746348	4.14050584	3.47E-05	0.000141

ENSG00000177989	ODF3B	53.78553835	36.144379	71.42669794	0.990724824	0.327826268	3.02210323	0.00251	0.007415
ENSG00000170006	TMEM154	867.512042	580.76044	1154.263644	0.99053193	0.079120866	12.5192251	5.86E-36	1.08E-34
ENSG00000144837	PLA1A	112.1619516	75.021864	149.3020388	0.990509431	0.20785264	4.76544069	1.88E-06	8.97E-06
ENSG00000110218	PANX1	1217.854826	815.33034	1620.379317	0.990181713	0.074597904	13.2735862	3.29E-40	6.63E-39
ENSG00000137501	SYTL2	96.65347495	64.880078	128.4268719	0.989867524	0.256705996	3.85603585	0.000115	0.000435
ENSG00000166452	AKIP1	920.474463	617.61177	1223.337157	0.986166746	0.076298905	12.9250445	3.25E-38	6.29E-37
ENSG00000175352	NRIP3	431.0075289	288.83246	573.182598	0.984485529	0.114126889	8.6262364	6.34E-18	6.50E-17
ENSG00000151208	DLG5	1067.230793	717.51724	1416.944349	0.981837803	0.07282664	13.4818496	2.00E-41	4.12E-40
ENSG00000183044	ABAT	69.12729672	46.368227	91.88636622	0.980918402	0.280684365	3.4947383	0.000475	0.001621
ENSG00000143641	GALNT2	6980.254814	4695.9331	9264.576509	0.980645468	0.041901811	23.4034151	3.94E-121	2.29E-119
ENSG00000229644	NAMPTP1	83.07892128	56.162168	109.9956745	0.979081291	0.270140545	3.6243404	0.00029	0.001024
ENSG00000253669	GASAL1	27.82023376	18.830862	36.80960593	0.976520653	0.419431908	2.3281983	0.019902	0.047691
ENSG00000169499	PLEKHA2	125.532143	84.312081	166.7522053	0.975836569	0.206945393	4.71543027	2.41E-06	1.13E-05
ENSG00000140545	MFGE8	3519.737271	2374.0777	4665.396839	0.975646298	0.047627742	20.484832	2.94E-93	1.29E-91
ENSG00000213190	MLLT11	3588.19486	2421.1055	4755.284194	0.974090421	0.0506903	19.2165053	2.69E-82	1.03E-80
ENSG00000246898	LINC00920	119.3766563	80.772134	157.9811781	0.970738363	0.220718977	4.39807385	1.09E-05	4.75E-05
ENSG00000107984	DKK1	816.473462	551.80728	1081.139639	0.97044206	0.211116666	4.59670986	4.29E-06	1.97E-05
ENSG00000187498	COL4A1	256373.4224	173300.36	339446.486	0.969903636	0.033433629	29.0098225	4.95E-185	4.69E-183
ENSG00000140548	ZNF710	777.3713353	526.05242	1028.690255	0.96901398	0.084395041	11.481883	1.63E-30	2.63E-29
ENSG00000082014	SMARCD3	71.8107963	48.724158	94.89743432	0.968465083	0.268218715	3.61072897	0.000305	0.001073
ENSG00000154654	NCAM2	45.42355285	30.709566	60.13754013	0.968422053	0.327759435	2.95467331	0.00313	0.009055
ENSG00000198742	SMURF1	1900.846116	1286.5316	2515.160591	0.967394203	0.062892851	15.3816244	2.17E-53	5.67E-52
ENSG00000243147	MRPL33	1418.703934	961.35454	1876.053328	0.966975341	0.066181613	14.6109365	2.39E-48	5.68E-47
ENSG00000176845	METRNL	1083.114377	733.507	1432.721751	0.966332831	0.086268659	11.2014356	4.01E-29	6.18E-28
ENSG00000110446	SLC15A3	550.0301578	373.04001	727.0203009	0.964937256	0.105788259	9.12140216	7.42E-20	8.25E-19
ENSG00000231925	TAPBP	12145.40826	8231.5797	16059.23681	0.964166048	0.032490868	29.6749857	1.61E-193	1.73E-191
ENSG00000180155	LYNX1	142.7955576	96.696087	188.8950281	0.963847757	0.200838777	4.79911187	1.59E-06	7.64E-06
ENSG00000118946	PCDH17	123.7969439	83.510428	164.0834601	0.963740589	0.236732553	4.07100998	4.68E-05	0.000187
ENSG00000145817	YIPF5	4013.510741	2722.194	5304.827454	0.963735149	0.052660723	18.300834	8.15E-75	2.88E-73
ENSG00000087303	NID2	4530.345151	3073.3561	5987.334248	0.961403023	0.047123096	20.4019493	1.61E-92	6.98E-91
ENSG00000145431	PDGFC	316.7873573	215.23778	418.3369331	0.95998205	0.135000714	7.11094056	1.15E-12	9.07E-12
ENSG00000150540	HNMT	297.275109	202.43695	392.1132699	0.95753979	0.135214024	7.08166032	1.42E-12	1.12E-11
ENSG00000160179	ABCG1	2555.454413	1735.7356	3375.173257	0.95749795	0.054629501	17.5271225	8.90E-69	2.96E-67
ENSG00000116661	FBXO2	39.88560158	27.244324	52.52687961	0.957308421	0.389461569	2.45803051	0.01397	0.034893
ENSG00000095739	BAMBI	33.8679549	23.013868	44.72204147	0.955501147	0.387878526	2.46340306	0.013763	0.034436
ENSG00000122694	GLIPR2	974.0343596	662.1272	1285.941523	0.955466667	0.082698824	11.5535702	7.08E-31	1.16E-29
ENSG00000095209	TMEM38B	121.4801405	82.461946	160.4983349	0.9550294	0.219538714	4.35016396	1.36E-05	5.84E-05
ENSG00000137496	IL18BP	248.8480351	169.57068	328.1253871	0.952433715	0.168331594	5.65808054	1.53E-08	8.87E-08
ENSG00000273888	FRMD6-AS1	39.84936413	27.208775	52.4899532	0.950809268	0.383581194	2.47876925	0.013184	0.033119
ENSG00000241316	SUCLG2-AS1	35.59941493	24.36245	46.83638017	0.950710998	0.37068222	2.56476019	0.010325	0.026519
ENSG00000082397	EPB41L3	2455.231564	1674.6067	3235.856424	0.95023446	0.05735332	16.5680811	1.19E-61	3.54E-60
ENSG00000196208	GREB1	56.88886899	38.634511	75.14322681	0.947904492	0.316414107	2.9957719	0.002738	0.008022
ENSG00000000971	CFH	1786.902883	1220.583	2353.222767	0.947439086	0.064792997	14.6225538	2.02E-48	4.80E-47
ENSG00000171729	TMEM51	831.2683003	568.17582	1094.360782	0.947112678	0.082367718	11.4985907	1.34E-30	2.18E-29
ENSG00000119669	IRF2BPL	1187.670697	811.45953	1563.881863	0.946437545	0.067602618	14.0000131	1.56E-44	3.43E-43
ENSG00000143479	DYRK3	721.9031755	493.43643	950.3699228	0.946204832	0.091168689	10.3786161	3.10E-25	4.23E-24
ENSG00000160209	PDXK	2447.323483	1675.2564	3219.390527	0.943938259	0.053880756	17.5190239	1.03E-68	3.40E-67
ENSG00000134954	ETS1	9550.309672	6532.8676	12567.75172	0.943552511	0.037199486	25.3646653	6.19E-142	4.33E-140
ENSG00000171016	PYGO1	228.5873779	156.32977	300.8449872	0.942002707	0.148812107	6.3301483	2.45E-10	1.65E-09
ENSG00000198053	SIRPA	9062.823826	6205.188	11920.45963	0.941427544	0.037638025	25.0126715	4.45E-138	3.01E-136
ENSG00000111647	UHRF1BP1L	740.2409299	506.69946	973.7823959	0.93980021	0.087203377	10.777108	4.42E-27	6.43E-26
ENSG00000105255	FSD1	228.338116	156.73193	299.9443043	0.939756728	0.166918121	5.63004618	1.80E-08	1.04E-07
ENSG00000167123	CERCAM	1777.465904	1218.8675	2336.064354	0.939239833	0.061585582	15.2509695	1.62E-52	4.13E-51
ENSG00000110811	P3H3	3310.497471	2270.9973	4349.99763	0.938964382	0.059189101	15.8638055	1.13E-56	3.09E-55
ENSG00000159231	CBR3	367.2350961	252.12422	482.3459745	0.935881619	0.114822358	8.15069152	3.62E-16	3.41E-15
ENSG00000240445	FOXO3B	95.44799619	65.466356	125.4296364	0.933816039	0.23644278	3.94943775	7.83E-05	0.000304
ENSG00000172985	SH3RF3	2284.075292	1573.4336	2994.716949	0.929225982	0.05183473	17.9267062	7.30E-72	2.51E-70
ENSG00000156642	NPTN	3693.264038	2544.2498	4842.2783	0.928930912	0.046342347	20.0449691	2.23E-89	9.30E-88
ENSG00000146648	EGFR	337.2243487	232.53925	441.9094488	0.927079316	0.129088211	7.18175045	6.88E-13	5.50E-12
ENSG00000189077	TMEM120A	1195.860874	825.88643	1565.835318	0.924001527	0.075950374	12.1658588	4.72E-34	8.39E-33
ENSG00000113580	NR3C1	1383.122152	953.91057	1812.333738	0.923989448	0.071670351	12.8922132	4.98E-38	9.59E-37
ENSG00000177674	AGTRAP	1238.15622	855.38962	1620.922823	0.922623899	0.068816675	13.4069817	5.50E-41	1.12E-39
ENSG00000164307	ERAP1	7415.445679	5127.7862	9703.105187	0.92008959	0.034456382	26.703024	4.34E-157	3.45E-155
ENSG00000172738	TMEM217	742.5435604	513.76933	971.3177905	0.919858931	0.087256189	10.542048	5.53E-26	7.78E-25
ENSG00000108175	ZMIZ1	4281.703752	2960.8198	5602.587649	0.919604044	0.04100845	22.4247452	2.26E-111	1.21E-109
ENSG00000117595	IRF6	80.96834398	55.930845	106.0058429	0.919398803	0.251262275	3.65911995	0.000253	0.000904
ENSG00000137094	DNAJB5	935.6160965	646.63192	1224.600277	0.918873295	0.091477574	10.044793	9.69E-24	1.25E-22
ENSG00000131979	GCH1	479.5510956	332.1541	626.9480903	0.916831444	0.121429834	7.55029809	4.34E-14	3.70E-13
ENSG00000170340	B3GNT2	1512.562663	1047.2265	1977.898792	0.915925568	0.076270602	12.008894	3.19E-33	5.53E-32
ENSG00000189056	RELN	21400.29457	14826.47	27974.11878	0.915870988	0.026452616	34.6230781	1.14E-262	1.86E-260
ENSG00000127329	PTPRB	21169.58871	14671.404	27667.77328	0.915071013	0.030603489	29.9008717	1.92E-196	2.10E-194
ENSG00000006451	RALA	20972.36964	14535.09	27409.64924	0.914875407	0.031024024	29.489257	3.95E-191	4.06E-189
ENSG00000188549	CCDC9B	1947.584726	1351.2365	2543.932913	0.914644689	0.068238843	13.4035784	5.76E-41	1.17E-39
ENSG00000179855	GIPC3	143.4098944	99.803163	187.0166262	0.914456557	0.197002971	4.64184144	3.45E-06	1.60E-05
ENSG00000146232	NFKBIE	850.4327438	590.34093	1110.524557	0.913521852	0.079138923	11.5432687	7.98E-31	1.30E-29
ENSG00000141068	KSR1	2155.34116	1495.6718	2815.010561	0.913355844	0.060685866	15.0505531	3.42E-51	8.52E-50
ENSG00000122591	FAM126A	3854.857879	2675.5811	5034.13461	0.910803229	0.043648198	20.8669146	1.07E-96	4.86E-95
ENSG00000213066	FGFR1OP	155.4938198	108.02396	202.963675	0.910776192	0.177024209	5.14492451	2.68E-07	1.39E-06
ENSG00000178015	GPR150	63.52126635	44.060486	82.98204699	0.90971541	0.275351505	3.30383308	0.000954	0.003073
ENSG00000163013	FBXO41	101.9606738	70.865224	133.056124	0.909207664	0.216319802	4.20307181	2.63E-05	0.000109
ENSG00000127527	EPS15L1	1645.391408	1146.6851	2144.097667	0.904708071	0.066364101	13.6324919	2.57E-42	5.43E-41
ENSG00000100600	LGMN	4533.395867	3157.5766	5909.215106	0.904660178	0.041086264	22.0185556	1.91E-107	9.64E-106
ENSG00000180346	TIGD2	117.3265888	81.466743	153.1864343	0.904311519	0.214825872	4.20950937	2.56E-05	0.000106
ENSG00000183723	CMTM4	464.8436217	324.04464	605.6426069	0.904157886	0.103333444	8.74990562	2.14E-18	2.24E-17
ENSG00000184371	CSF1	4245.53784	2958.5096	5532.566042	0.90287901	0.042741665	21.1240956	4.78E-99	2.23E-97

ENSG00000102934	PLLP	385.2128249	268.86064	501.5650104	0.902487434	0.120169472	7.51012231	5.91E-14	5.01E-13
ENSG00000250714	AC100861.2	71.06084072	49.364553	92.75712816	0.899899401	0.280348986	3.20992565	0.001328	0.004159
ENSG00000162512	SDC3	3561.292314	2490.6362	4631.948425	0.894675948	0.044305544	20.193318	1.12E-90	4.76E-89
ENSG00000140945	CDH13	18109.8366	12679.361	23540.31225	0.892471395	0.031087271	28.70858	2.98E-181	2.77E-179
ENSG00000166337	TAF10	72.14635465	50.390815	93.90189383	0.891685361	0.31199671	2.85799604	0.004263	0.011949
ENSG00000197442	MAP3K5	748.0281726	523.91571	972.1406317	0.891522788	0.086524721	10.3036771	6.78E-25	9.09E-24
ENSG00000153993	SEMA3D	381.8296813	267.64295	496.0164101	0.889936551	0.121557463	7.32111819	2.46E-13	2.02E-12
ENSG00000181104	F2R	31931.45997	22395.473	41467.44705	0.888618738	0.028003625	31.7322754	5.58E-221	7.20E-219
ENSG00000188517	COL25A1	302.6731404	211.81342	393.5328578	0.887914942	0.145973852	6.08269859	1.18E-09	7.54E-09
ENSG00000148908	RGS10	501.6255411	352.38805	650.863035	0.886818496	0.113202382	7.83392083	4.73E-15	4.23E-14
ENSG00000135622	SEMA4F	540.3111969	379.69938	700.923018	0.886750524	0.103563713	8.56236701	1.11E-17	1.12E-16
ENSG00000138411	HECW2	8163.913248	5730.6337	10597.19284	0.886095329	0.039896956	22.2095973	2.77E-109	1.43E-107
ENSG00000205220	PSMB10	576.3878733	404.30964	748.4661056	0.885974654	0.096913841	9.1418795	6.14E-20	6.86E-19
ENSG00000198467	TPM2	711.046469	500.76083	921.3321114	0.88417918	0.103965214	8.50456746	1.82E-17	1.82E-16
ENSG00000117122	MFAP2	3560.863821	2504.0178	4617.709871	0.883544735	0.057771432	15.2938003	8.41E-53	2.15E-51
ENSG00000133121	STARD13	2388.469583	1681.9732	3094.965943	0.880249983	0.051853637	16.9756653	1.24E-64	3.85E-63
ENSG00000115652	UXS1	2563.05742	1805.3212	3320.793607	0.880203156	0.057241959	15.3768873	2.34E-53	6.07E-52
ENSG00000111339	ART4	468.1680415	330.43624	605.8998451	0.879335662	0.114563595	7.67552433	1.65E-14	1.43E-13
ENSG00000107099	DOCK8	61.54232471	43.325291	79.75935871	0.87920103	0.289414651	3.03785944	0.002383	0.007074
ENSG00000175048	ZDHHC14	1852.51419	1305.7151	2399.313282	0.878403307	0.056239442	15.6189905	5.41E-55	1.44E-53
ENSG00000197321	SVIL	9104.092183	6420.505	11787.67933	0.877153616	0.036797553	23.8372808	1.37E-125	8.10E-124
ENSG00000151835	SACS	5135.30139	3622.7816	6647.821144	0.875542573	0.038506662	22.737431	1.91E-114	1.05E-112
ENSG00000100889	PCK2	322.6979682	227.96524	417.4306935	0.87542324	0.124443293	7.0347161	2.00E-12	1.55E-11
ENSG00000186174	BCL9L	4188.275947	2959.252	5417.299864	0.872730056	0.043822627	19.9150555	3.01E-88	1.24E-86
ENSG00000186575	NF2	4312.799861	3048.7894	5576.810357	0.870585514	0.054168013	16.0719483	4.01E-58	1.13E-56
ENSG00000178053	MLF1	223.7851834	157.94453	289.6258348	0.870245518	0.159601232	5.45262405	4.96E-08	2.74E-07
ENSG00000173334	TRIB1	866.5848457	612.62158	1120.548114	0.870092487	0.08364473	10.4022292	2.42E-25	3.32E-24
ENSG00000114861	FOXP1	1277.924314	904.14004	1651.708583	0.869416905	0.068863484	12.625224	1.53E-36	2.86E-35
ENSG00000101017	CD40	591.246931	418.35369	764.1401714	0.869191676	0.107646761	8.07448056	6.78E-16	6.31E-15
ENSG00000163430	FSTL1	26277.38285	18606.458	33948.30767	0.867384963	0.029289177	29.6145212	9.72E-193	1.03E-190
ENSG00000141526	SLC16A3	5288.349889	3758.9322	6817.767598	0.859323297	0.053241575	16.1400804	1.33E-58	3.80E-57
ENSG00000163932	PRKCD	940.9971148	669.40365	1212.590579	0.856980598	0.074598247	11.4879455	1.52E-30	2.46E-29
ENSG00000050165	DKK3	31410.71847	22363.347	40458.09024	0.855321667	0.028771616	29.7279677	3.34E-194	3.60E-192
ENSG00000145979	TBC1D7	669.4474793	477.45397	861.440992	0.854515356	0.090598226	9.43192151	4.03E-21	4.74E-20
ENSG00000135341	MAP3K7	2250.736166	1603.7281	2897.744275	0.853303482	0.051110752	16.6951853	1.42E-62	4.29E-61
ENSG00000116852	KIF21B	390.3098266	278.39161	502.2280464	0.853050894	0.113433154	7.52029599	5.47E-14	4.64E-13
ENSG00000128567	PODXL	19185.76248	13677.135	24694.38985	0.852289978	0.036816246	23.1498336	1.46E-118	8.25E-117
ENSG00000175591	P2RY2	181.9434765	129.5867	234.3002551	0.85174765	0.172282042	4.94391429	7.66E-07	3.81E-06
ENSG00000169621	APLF	158.3241759	112.70581	203.9425447	0.851677796	0.187702431	4.53738288	5.70E-06	2.57E-05
ENSG00000168575	SLC20A2	1280.470998	913.04582	1647.896175	0.850910026	0.078377612	10.8565445	1.86E-27	2.71E-26
ENSG00000229689	AC009237.3	68.45596444	48.665057	88.24687202	0.850137813	0.28248249	3.00952393	0.002617	0.0077
ENSG00000271303	SRXN1	59.97129943	42.733631	77.20896772	0.850102285	0.353136705	2.40728951	0.016071	0.039496
ENSG00000095303	PTGS1	581.7720856	414.96897	748.5751986	0.849484286	0.121256816	7.00566217	2.46E-12	1.89E-11
ENSG00000105835	NAMPT	15877.83009	11334.621	20421.03891	0.848890848	0.034851477	24.3573853	4.84E-131	3.06E-129
ENSG00000113583	C5orf15	1812.159051	1293.2426	2331.075541	0.848552136	0.05878636	14.4345073	3.14E-47	7.28E-46
ENSG00000115414	FN1	438438.3608	313158.59	563718.136	0.848078363	0.03335484	25.4259461	1.30E-142	9.19E-141
ENSG00000084112	SSH1	7315.24054	5227.8824	9402.598669	0.846284869	0.039385574	21.4871786	2.05E-102	9.72E-101
ENSG00000054967	RELT	463.0920747	331.73777	594.4463815	0.844684384	0.119308814	7.07981542	1.44E-12	1.13E-11
ENSG00000120217	CD274	1557.584961	1115.3968	1999.773155	0.841544072	0.062820875	13.3959305	6.39E-41	1.30E-39
ENSG00000237172	B3GNT9	319.0454963	228.80373	409.28726	0.841520436	0.140524133	5.98844068	2.12E-09	1.32E-08
ENSG00000272269	AL138724.1	168.7038529	120.75365	216.654058	0.840603652	0.174244756	4.82426965	1.41E-06	6.79E-06
ENSG00000214455	RCN1P2	306.0898885	219.69112	392.4886602	0.837504193	0.123770347	6.76659811	1.32E-11	9.72E-11
ENSG00000159363	ATP13A2	5465.428418	3921.4322	7009.424651	0.836974395	0.039717561	21.073157	1.40E-98	6.48E-97
ENSG00000065534	MYLK	2831.306622	2032.9465	3629.666716	0.835859248	0.049469109	16.8965898	4.77E-64	1.46E-62
ENSG00000257219	LNCOG	654.0415482	469.56462	838.5184767	0.835854682	0.09305419	8.9824508	2.65E-19	2.87E-18
ENSG00000176170	SPHK1	3457.190567	2484.397	4429.984126	0.835586837	0.054248614	15.4029159	1.56E-53	4.09E-52
ENSG00000170525	PFKFB3	3676.045598	2640.8834	4711.207843	0.834570265	0.047379723	17.6145029	1.91E-69	6.40E-68
ENSG00000143321	HDGF	11341.37653	8150.5053	14532.24781	0.834039378	0.032071035	26.0060011	4.24E-149	3.18E-147
ENSG00000100503	NIN	6141.379904	4417.7571	7865.002755	0.831764835	0.037114891	22.4105425	3.11E-111	1.65E-109
ENSG00000204592	HLA-E	35846.44506	25805.399	45887.49119	0.830422898	0.028290045	29.3538914	2.13E-189	2.15E-187
ENSG00000132205	EMILIN2	196.3487229	140.96035	251.7370994	0.830027386	0.177842678	4.66720022	3.05E-06	1.42E-05
ENSG00000136026	CKAP4	18583.65088	13409.72	23757.5819	0.825149811	0.027878309	29.5982734	1.57E-192	1.65E-190
ENSG00000059769	DNAJC25	345.3548063	249.28599	441.4236228	0.825030404	0.122346527	6.7433905	1.55E-11	1.14E-10
ENSG00000136867	SLC31A2	305.3396356	219.97937	390.6999028	0.824702302	0.133923194	6.15802442	7.37E-10	4.78E-09
ENSG00000095015	MAP3K1	1457.634897	1052.9967	1862.273098	0.823637854	0.061538987	13.3840009	7.50E-41	1.52E-39
ENSG00000182957	SPATA13	670.9374485	485.50561	856.3692886	0.821164145	0.088903406	9.23658809	2.54E-20	2.89E-19
ENSG00000130734	ATG4D	367.4552022	266.48027	468.4301388	0.817179653	0.122575907	6.66672328	2.62E-11	1.89E-10
ENSG00000184384	MAML2	2048.423531	1483.196	2613.651107	0.816227631	0.056470122	14.4541504	2.36E-47	5.51E-46
ENSG00000111199	TRPV4	452.7052204	328.70362	576.7068238	0.816142822	0.118154059	6.90744636	4.93E-12	3.73E-11
ENSG00000226419	SLC16A1-AS1	285.45456	206.95936	363.9497597	0.814104136	0.146069652	5.5734018	2.50E-08	1.42E-07
ENSG00000130479	MAP1S	3015.802027	2188.0827	3843.52135	0.813128865	0.046900574	17.3372903	2.46E-67	7.95E-66
ENSG00000101850	GPR143	116.4053438	84.7411	148.0695877	0.810342329	0.203922543	3.97377512	7.07E-05	0.000276
ENSG00000101460	MAP1LC3A	896.2075916	652.03533	1140.379855	0.805363303	0.077481145	10.3943133	2.63E-25	3.60E-24
ENSG00000120337	TNFSF18	1697.897218	1237.2506	2158.534858	0.803367139	0.073511736	10.9284201	8.43E-28	1.24E-26
ENSG00000152291	TGOLN2	22742.32345	16570.45	28914.19739	0.803064358	0.02743351	29.2731175	2.28E-188	2.27E-186
ENSG00000176692	FOXC2	350.2232904	255.93997	444.5066086	0.800835449	0.130470756	6.13804558	8.35E-10	5.41E-09
ENSG00000100902	PSMA6	118.5098708	86.618685	150.4010564	0.800547973	0.236285981	3.38804684	0.000704	0.00233
ENSG00000130508	PXDN	188842.6605	137831.84	239853.4811	0.799238487	0.027913298	28.632893	2.62E-180	2.42E-178
ENSG00000122729	ACO1	5812.291381	4241.3804	7383.202353	0.799095333	0.038669071	20.6649736	7.16E-95	3.18E-93
ENSG00000256043	CTSO	1092.374471	796.85888	1387.890057	0.797864307	0.088261924	9.03973387	1.57E-19	1.73E-18
ENSG00000112137	PHACTR1	485.3014214	354.47262	616.1302249	0.797101521	0.101912841	7.82140421	5.22E-15	4.67E-14
ENSG00000131016	AKAP12	58770.83629	42942.491	74599.1813	0.79665815	0.032358845	24.6194867	7.81E-134	5.13E-132
ENSG00000248890	HHIP-AS1	1410.404053	1031.6602	1789.147858	0.795729154	0.064361073	12.3635159	4.12E-35	7.45E-34
ENSG00000136052	SLC41A2	612.1665867	448.05373	776.2794451	0.795696361	0.095291774	8.35010543	6.82E-17	6.66E-16
ENSG00000065526	SPEN	5289.77812	3870.392	6709.164264	0.793235622	0.040202773	19.7308683	1.17E-86	4.67E-85

ENSG00000171940	ZNF217	2262.17646	1655.4592	2868.893757	0.792938711	0.053974153	14.6910822	7.35E-49	1.77E-47
ENSG00000197951	ZNF71	1098.899761	804.63293	1393.166591	0.791479155	0.073722337	10.7359477	6.90E-27	9.96E-26
ENSG00000163527	STT3B	8075.130047	5915.1351	10235.12499	0.790816994	0.033219613	23.8057255	2.91E-125	1.72E-123
ENSG00000105497	ZNF175	535.1131486	392.04635	678.1799469	0.790418954	0.108994437	7.25192016	4.11E-13	3.33E-12
ENSG00000101400	SNTA1	313.6826593	229.75943	397.6058857	0.790073847	0.141370691	5.58866792	2.29E-08	1.31E-07
ENSG00000149557	FEZ1	2328.793817	1706.9922	2950.595417	0.788896716	0.053623775	14.7116968	5.42E-49	1.31E-47
ENSG00000163704	PRRT3	162.6256991	119.54657	205.7048243	0.788672987	0.177497427	4.44329249	8.86E-06	3.90E-05
ENSG00000170085	SIMC1	354.6429169	260.30478	448.9810549	0.785939875	0.116876293	6.72454485	1.76E-11	1.29E-10
ENSG00000175832	ETV4	371.5524529	272.77712	470.3277871	0.785737659	0.11414974	6.88339419	5.84E-12	4.40E-11
ENSG00000108932	SLC16A6	2357.300912	1730.4612	2984.140646	0.785232238	0.055501232	14.1480146	1.92E-45	4.31E-44
ENSG00000198792	TMEM184B	7228.374326	5311.292	9145.456694	0.784712655	0.041042447	19.1195387	1.74E-81	6.58E-80
ENSG00000127666	TICAM1	1150.875022	845.70618	1456.043867	0.783591555	0.068970113	11.3613204	6.52E-30	1.03E-28
ENSG00000162734	PEA15	18804.36908	13831.482	23777.25568	0.781616554	0.031624526	24.7155185	7.28E-135	4.84E-133
ENSG00000049192	ADAMTS6	699.7534076	515.7271	883.7797141	0.781598355	0.102896026	7.59600138	3.05E-14	2.62E-13
ENSG00000102359	SRPX2	9209.51961	6782.1807	11636.85857	0.779283515	0.033305437	23.3980872	4.47E-121	2.58E-119
ENSG00000134470	IL15RA	1141.402016	840.93674	1441.867289	0.779261128	0.070118098	11.113552	1.08E-28	1.63E-27
ENSG00000180530	NRIP1	3031.254441	2231.1526	3831.356303	0.779253219	0.055172194	14.1240209	2.70E-45	6.06E-44
ENSG00000065989	AC011529.1	330.9031726	244.06532	417.7410288	0.779215526	0.131500387	5.92557593	3.11E-09	1.92E-08
ENSG00000136274	NACAD	117.0723726	85.94595	148.1987948	0.778278941	0.215677245	3.60853525	0.000308	0.001081
ENSG00000168615	ADAM9	14624.10831	10777.458	18470.75852	0.777071705	0.034418487	22.5771603	7.27E-113	3.95E-111
ENSG00000137522	RNF121	782.2417519	577.22365	987.2598513	0.77687315	0.085750845	9.05965595	1.31E-19	1.44E-18
ENSG00000100583	SAMD15	43.96004665	32.406735	55.51335828	0.77535328	0.323072254	2.39993769	0.016398	0.040157
ENSG00000102755	FLT1	6967.324876	5140.8017	8793.848029	0.774711785	0.03571777	21.6898139	2.56E-104	1.24E-102
ENSG00000167604	AD000864.1	73.5091217	54.215901	92.802342	0.771413371	0.266694769	2.89249532	0.003822	0.010844
ENSG00000152990	ADGRA3	1260.856515	930.77251	1590.940516	0.770975647	0.069841896	11.0388705	2.48E-28	3.71E-27
ENSG00000112763	BTN2A1	1326.183054	980.00336	1672.362749	0.769507597	0.064628174	11.9066894	1.09E-32	1.87E-31
ENSG00000182916	TCEAL7	149.0912959	110.13154	188.0510522	0.769138238	0.178883257	4.29966589	1.71E-05	7.25E-05
ENSG00000105643	ARRDC2	1090.668933	807.24354	1374.094321	0.768531472	0.081345244	9.4477739	3.46E-21	4.09E-20
ENSG00000139899	CBLN3	162.0115941	120.22375	203.7994343	0.768506472	0.194341566	3.95441124	7.67E-05	0.000298
ENSG00000083444	PLOD1	28312.55597	20948.085	35677.02671	0.768304441	0.027894722	27.5430045	5.37E-167	4.63E-165
ENSG00000176182	MYPOP	411.0995305	304.40619	517.7928662	0.76807252	0.117014575	6.56390468	5.24E-11	3.71E-10
ENSG00000158710	TAGLN2	19851.92862	14693.144	25010.82178	0.767208285	0.033022451	23.2329296	2.12E-119	1.21E-117
ENSG00000146072	TNFRSF21	7867.605504	5823.8942	9911.316825	0.766885966	0.036308663	21.1212943	5.07E-99	2.36E-97
ENSG00000285517	AC010198.2	79.6685928	59.130152	100.2070336	0.765358805	0.258257381	2.96355056	0.003041	0.008826
ENSG00000073849	ST6GAL1	7209.940347	5341.7225	9078.158181	0.765338871	0.038455922	19.901717	3.93E-88	1.61E-86
ENSG00000205213	LGR4	389.5151487	288.19873	490.8315641	0.765297733	0.126111016	6.06844474	1.29E-09	8.20E-09
ENSG00000196498	NCOR2	8613.870302	6383.8164	10843.92417	0.764377104	0.042675909	17.9112086	9.64E-72	3.32E-70
ENSG00000114554	PLXNA1	3481.232771	2580.1013	4382.364241	0.764238625	0.05346734	14.2935599	2.40E-46	5.49E-45
ENSG00000100311	PDGFB	18816.66956	13947.676	23685.66268	0.764097081	0.034391921	22.2173425	2.33E-109	1.21E-107
ENSG00000033170	FUT8	710.96987	527.95992	893.9798178	0.761773122	0.08889202	8.56964577	1.04E-17	1.05E-16
ENSG00000250565	ATP6V1E2	106.9421462	79.349095	134.5351979	0.76119109	0.222350854	3.4233783	0.000618	0.002069
ENSG00000152804	HHEX	2771.625171	2057.9396	3485.310737	0.76051104	0.050144257	15.1664635	5.90E-52	1.49E-50
ENSG00000088899	LZTS3	1018.342368	756.31207	1280.372666	0.760058559	0.073964753	10.2759562	9.04E-25	1.21E-23
ENSG00000151348	EXT2	7883.395251	5859.4764	9907.314087	0.757653783	0.037683072	20.1059452	6.55E-90	2.75E-88
ENSG00000119632	IFI27L2	448.117209	333.70462	562.5297963	0.757569853	0.114141799	6.63709404	3.20E-11	2.29E-10
ENSG00000176597	B3GNT5	2290.358136	1702.6942	2878.022103	0.757324559	0.057205164	13.2387445	5.24E-40	1.05E-38
ENSG00000078269	SYNJ2	5871.60716	4365.6563	7377.558051	0.757089829	0.03844512	19.6927422	2.49E-86	9.91E-85
ENSG00000157833	GAREM2	252.4983465	187.64057	317.3561195	0.756346719	0.137675069	5.49370868	3.94E-08	2.20E-07
ENSG00000090006	LTBP4	159.3655099	118.80096	199.9300588	0.756222055	0.180990986	4.17823049	2.94E-05	0.000121
ENSG00000185947	ZNF267	1191.82148	887.0176	1496.625356	0.753381494	0.077472259	9.7245324	2.37E-22	2.91E-21
ENSG00000090776	EFNB1	3738.317357	2784.4571	4692.177639	0.753091873	0.042614049	17.6723847	6.84E-70	2.31E-68
ENSG00000165030	NFIL3	406.4580718	302.83306	510.0830864	0.752491083	0.114595178	6.56651614	5.15E-11	3.65E-10
ENSG00000155090	KLF10	1906.489177	1419.679	2393.299374	0.752455997	0.056907998	13.2223241	6.52E-40	1.30E-38
ENSG00000160233	LRRC3	513.6957299	382.64396	644.7475028	0.751297338	0.104290464	7.20389296	5.85E-13	4.70E-12
ENSG00000138759	FRAS1	271.1058688	202.5153	339.6964571	0.748599526	0.156617429	4.77979707	1.75E-06	8.38E-06
ENSG00000130513	GDF15	8243.315814	6152.5317	10334.09991	0.748548041	0.036508643	20.5033102	2.01E-93	8.82E-92
ENSG00000148450	MSRB2	364.6088678	271.79969	457.4180467	0.748254267	0.121989336	6.13376782	8.58E-10	5.54E-09
ENSG00000148677	ANKRD1	26045.0544	19457.042	32633.06717	0.746080967	0.082885632	9.00133047	2.23E-19	2.42E-18
ENSG00000180611	MB21D2	188.1653455	140.90924	235.421452	0.745888081	0.198669503	3.75441661	0.000174	0.000636
ENSG00000104331	IMPAD1	4853.415721	3626.4286	6080.402863	0.744949039	0.040650757	18.3255882	5.17E-75	1.83E-73
ENSG00000171056	SOX7	2292.218428	1714.4821	2869.954731	0.743452581	0.055550448	13.3833768	7.56E-41	1.53E-39
ENSG00000110651	CD81	17686.74097	13228	22145.4817	0.743286621	0.031134929	23.8730791	5.83E-126	3.47E-124
ENSG00000130881	LRP3	547.928402	409.81185	686.0449583	0.743136215	0.122508309	6.06600661	1.31E-09	8.32E-09
ENSG00000077238	IL4R	7009.186653	5243.9173	8774.456024	0.742705649	0.038314045	19.384684	1.04E-83	4.04E-82
ENSG00000189420	ZFP92	155.3841285	116.26264	194.5056193	0.741308078	0.194196858	3.81730212	0.000135	0.000503
ENSG00000119729	RHOQ	3313.183336	2482.1467	4144.220021	0.738921428	0.045405314	16.2738977	1.51E-59	4.40E-58
ENSG00000099968	BCL2L13	3080.651716	2309.726	3851.577394	0.73820824	0.049958817	14.7763355	2.08E-49	5.05E-48
ENSG00000118058	KMT2A	4851.07773	3635.779	6066.376426	0.737712202	0.040448404	18.2383514	2.56E-74	9.03E-73
ENSG00000174516	PELI3	161.4192495	121.25258	201.585917	0.737261796	0.175518066	4.20048952	2.66E-05	0.00011
ENSG00000160213	CSTB	7346.691012	5512.1986	9181.183453	0.735844802	0.042130297	17.4659298	2.60E-68	8.55E-67
ENSG00000134851	TMEM165	4763.173476	3574.1199	5952.227054	0.735732489	0.045180187	16.2844054	1.27E-59	3.71E-58
ENSG00000204516	MICB	1720.677275	1290.6673	2150.687202	0.735595805	0.061771428	11.9083503	1.07E-32	1.83E-31
ENSG00000126561	STAT5A	756.7721326	567.46635	946.0779119	0.735267702	0.085618608	8.58770911	8.87E-18	9.03E-17
ENSG00000177464	GPR4	1034.463579	775.97421	1292.953144	0.734636532	0.072281489	10.16355	2.88E-24	3.77E-23
ENSG00000103187	COTL1	9688.116503	7274.206	12102.02698	0.734415007	0.101479247	7.23709555	4.58E-13	3.70E-12
ENSG00000176853	FAM91A1	3895.794254	2928.6967	4862.891834	0.731144756	0.042751155	17.1023394	1.43E-65	4.47E-64
ENSG00000089327	FXYD5	3075.96935	2314.6605	3837.278176	0.73006585	0.061205723	11.928065	8.45E-33	1.45E-31
ENSG00000111711	GOLT1B	1199.535826	902.17482	1496.89683	0.729953884	0.074133011	9.84654298	7.09E-23	8.86E-22
ENSG00000105738	SIPA1L3	2742.99461	2064.7875	3421.201698	0.728918862	0.051641869	14.1148816	3.08E-45	6.88E-44
ENSG00000166188	ZNF319	727.561492	547.7143	907.4086869	0.72843643	0.092318519	7.89046922	3.01E-15	2.72E-14
ENSG00000154589	LY96	359.6593583	270.96688	448.3518342	0.726520015	0.138335673	5.25186307	1.51E-07	7.99E-07
ENSG00000185164	NOMO2	7625.170704	5751.0824	9499.259022	0.723986432	0.036612956	19.7740505	4.98E-87	2.00E-85
ENSG00000103222	ABCC1	7183.33689	5420.0348	8946.638949	0.723504326	0.035629766	20.306177	1.13E-91	4.88E-90
ENSG00000100418	DESI1	3466.094383	2614.7443	4317.444473	0.722828376	0.044619939	16.1996719	5.07E-59	1.46E-57
ENSG00000100003	SEC14L2	626.2710059	472.52717	780.0148414	0.722817164	0.111502387	6.48252638	9.02E-11	6.28E-10

ENSG00000187676	B3GLCT	1325.121635	999.33395	1650.909322	0.722232083	0.066761012	10.8181715	2.82E-27	4.12E-26
ENSG00000103043	VAC14	3057.401147	2307.96	3806.842303	0.7216143	0.052377777	13.7771082	3.50E-43	7.53E-42
ENSG00000026950	BTN3A1	658.0356726	497.36091	818.7104344	0.721300376	0.094793004	7.60921529	2.76E-14	2.37E-13
ENSG00000239713	APOBEC3G	149.0486846	112.67574	185.4216321	0.720516105	0.184672762	3.90158298	9.56E-05	0.000366
ENSG00000273270	AC090114.2	327.5624482	247.48237	407.6425275	0.720474662	0.120162465	5.9958379	2.02E-09	1.27E-08
ENSG00000141696	P3H4	2130.891393	1609.2094	2652.573352	0.719845892	0.056157492	12.8183412	1.29E-37	2.47E-36
ENSG00000087903	RFX2	330.6762818	250.36301	410.9895548	0.719220947	0.129072729	5.57221459	2.52E-08	1.43E-07
ENSG00000261373	VPS9D1-AS1	115.5360984	87.516522	143.5556748	0.718526296	0.211277278	3.40086876	0.000672	0.002231
ENSG00000131669	NINJ1	2542.684012	1922.782	3162.586004	0.717568906	0.053018769	13.534243	9.82E-42	2.03E-40
ENSG00000166086	JAM3	5016.290939	3793.2511	6239.330827	0.717105196	0.045391613	15.7981871	3.20E-56	8.72E-55
ENSG00000117597	UTP25	1319.200001	999.1283	1639.271703	0.714762896	0.069487978	10.2861374	8.14E-25	1.09E-23
ENSG00000146426	TIAM2	274.9256201	208.12224	341.7289991	0.714231447	0.132763084	5.37974433	7.46E-08	4.07E-07
ENSG00000142156	COL6A1	3728.302646	2826.4085	4630.196796	0.712827628	0.048112443	14.8158686	1.16E-49	2.82E-48
ENSG00000268895	A1BG-AS1	137.7026018	104.50525	170.8999538	0.712558494	0.187148617	3.80744729	0.00014	0.000523
ENSG00000101842	VSIG1	76.73621617	58.078415	95.39401718	0.71119966	0.279973125	2.5402426	0.011078	0.02827
ENSG00000087074	PPP1R15A	4095.702762	3107.7242	5083.68128	0.71035879	0.044567986	15.938768	3.41E-57	9.41E-56
ENSG00000075651	PLD1	4914.938033	3727.5505	6102.325516	0.710205732	0.040650452	17.4710414	2.38E-68	7.83E-67
ENSG00000136603	SKIL	2484.338961	1887.1267	3081.55118	0.708710816	0.054972746	12.8920396	4.99E-38	9.60E-37
ENSG00000107130	NCS1	2324.533922	1766.3598	2882.708	0.706651228	0.141228883	5.00358861	5.63E-07	2.83E-06
ENSG00000111670	GNPTAB	4326.895911	3288.6034	5365.188461	0.70567407	0.041518432	16.9966456	8.70E-65	2.70E-63
ENSG00000121900	TMEM54	524.3592957	398.22865	650.4899372	0.705577217	0.105283133	6.70171182	2.06E-11	1.50E-10
ENSG00000164638	SLC29A4	107.2324209	81.347748	133.1170941	0.705362109	0.251816214	2.80109886	0.005093	0.014041
ENSG00000187479	C11orf96	358.8899246	272.93951	444.8403358	0.704788056	0.115239233	6.11586901	9.60E-10	6.16E-09
ENSG00000049323	LTBP1	6204.517309	4718.1644	7690.870218	0.70461914	0.040183522	17.5350271	7.74E-69	2.58E-67
ENSG00000168734	PKIG	4456.874376	3390.3266	5523.422141	0.704596034	0.040361949	17.456938	3.05E-68	9.99E-67
ENSG00000108179	PPIF	1150.221737	875.41397	1425.029506	0.704217885	0.082569182	8.52882238	1.48E-17	1.49E-16
ENSG00000126458	RRAS	2846.448234	2164.8064	3528.090116	0.703999361	0.058609786	12.011635	3.09E-33	5.36E-32
ENSG00000117691	NENF	503.1958562	383.38611	623.0055983	0.703998674	0.103782039	6.78343458	1.17E-11	8.68E-11
ENSG00000143845	ETNK2	514.1134162	391.28775	636.9390786	0.703489751	0.098683211	7.12876834	1.01E-12	7.99E-12
ENSG00000257594	GALNT4	83.81783496	63.635381	104.0002889	0.703001237	0.240573197	2.92219268	0.003476	0.009961
ENSG00000112893	MAN2A1	9213.909875	7013.8369	11413.98285	0.702187942	0.040845685	17.1912392	3.09E-66	9.76E-65
ENSG00000173705	SUSD5	5433.662159	4138.1829	6729.141406	0.701173106	0.036710693	19.0999692	2.53E-81	9.56E-80
ENSG00000122862	SRGN	17368.35033	13229.052	21507.64865	0.701144856	0.075971821	9.22901216	2.73E-20	3.10E-19
ENSG00000158201	ABHD3	101.3959403	77.378784	125.4130969	0.700291189	0.223568	3.1323409	0.001734	0.005301
ENSG00000069399	BCL3	748.2854723	570.73216	925.8387815	0.700264041	0.091136238	7.68370585	1.55E-14	1.35E-13
ENSG00000026297	RNASET2	222.671402	169.88426	275.4585435	0.700007451	0.149906292	4.66963354	3.02E-06	1.40E-05
ENSG00000083223	TUT7	1599.504658	1218.7221	1980.287215	0.6996047	0.060998241	11.4692603	1.88E-30	3.04E-29
ENSG00000196843	ARID5A	629.1686416	479.93931	778.3979749	0.698384164	0.094972987	7.3535032	1.93E-13	1.59E-12
ENSG00000120279	MYCT1	9013.468579	6874.8475	11152.08966	0.69780159	0.034454514	20.2528349	3.35E-91	1.44E-89
ENSG00000152484	USP12	2119.172066	1617.6365	2620.707658	0.695866356	0.054726941	12.7152431	4.87E-37	9.19E-36
ENSG00000166224	SGPL1	1198.712349	915.66591	1481.758789	0.695754185	0.075977759	9.1573402	5.32E-20	5.97E-19
ENSG00000133466	C1QTNF6	578.1830544	441.62806	714.7380481	0.695088949	0.104287378	6.66513016	2.64E-11	1.91E-10
ENSG00000105270	CLIP3	1268.878812	970.07487	1567.682754	0.694947588	0.077553069	8.96092957	3.22E-19	3.48E-18
ENSG00000166949	SMAD3	5574.508595	4258.4857	6890.531452	0.694752097	0.051220611	13.5639166	6.55E-42	1.36E-40
ENSG00000108395	TRIM37	3093.618399	2363.2689	3823.967874	0.693580057	0.050604147	13.7059925	9.35E-43	1.99E-41
ENSG00000251442	LINC01094	129.2105908	98.599941	159.8212402	0.693361894	0.196434153	3.52974208	0.000416	0.001432
ENSG00000100100	PIK3IP1	1318.783834	1008.0224	1629.545243	0.693292656	0.066061648	10.4946316	9.14E-26	1.28E-24
ENSG00000039139	DNAH5	248.431022	190.12444	306.7376081	0.693121669	0.144082395	4.81059235	1.50E-06	7.25E-06
ENSG00000103876	FAH	391.3253428	299.31834	483.3323484	0.692525892	0.141096775	4.90816244	9.19E-07	4.54E-06
ENSG00000116514	RNF19B	1268.14286	969.05715	1567.228571	0.692512593	0.067849464	10.2066037	1.85E-24	2.45E-23
ENSG00000173210	ABLIM3	6583.73859	5036.1259	8131.351291	0.691651882	0.036913772	18.7369606	2.47E-78	9.15E-77
ENSG00000049449	RCN1	11311.9167	8650.7896	13973.04382	0.691530164	0.030435004	22.7215402	2.74E-114	1.49E-112
ENSG00000112655	PTK7	2586.519494	1978.6454	3194.393579	0.691488243	0.050996772	13.5594512	6.97E-42	1.44E-40
ENSG00000197965	MPZL1	8113.041195	6206.6997	10019.38265	0.690547481	0.033446072	20.6465946	1.05E-94	4.63E-93
ENSG00000073331	ALPK1	166.3709819	127.11928	205.6226841	0.690484503	0.168298863	4.10272826	4.08E-05	0.000165
ENSG00000138821	SLC39A8	78.86431931	60.333769	97.39486974	0.690440383	0.26530912	2.60239973	0.009257	0.024051
ENSG00000070759	TESK2	146.5876578	112.24375	180.9315608	0.689053461	0.182945845	3.76643406	0.000166	0.000608
ENSG00000184349	EFNA5	916.1588336	701.68453	1130.63314	0.687786668	0.081681826	8.42031459	3.75E-17	3.71E-16
ENSG00000100647	SUSD6	3421.271856	2622.3589	4220.184793	0.68674681	0.044721363	15.3561244	3.22E-53	8.32E-52
ENSG00000124201	ZNFX1	8023.526796	6157.544	9889.509546	0.683703256	0.034617405	19.7502749	7.98E-87	3.19E-85
ENSG00000166450	PRTG	248.8977345	190.54309	307.2523814	0.683684271	0.153217901	4.46216968	8.11E-06	3.60E-05
ENSG00000188010	MORN2	154.8288572	119.10625	190.5514674	0.683269251	0.18236179	3.74677858	0.000179	0.000655
ENSG00000134874	DZIP1	649.5757433	498.85248	800.2990075	0.682521276	0.092786536	7.35582237	1.90E-13	1.57E-12
ENSG00000111799	COL12A1	3555.216003	2729.9209	4380.511102	0.682150749	0.143889954	4.74078092	2.13E-06	1.01E-05
ENSG00000122863	CHST3	497.4024367	382.05546	612.7494177	0.681397285	0.099473023	6.85007116	7.38E-12	5.53E-11
ENSG00000120156	TEK	4936.961295	3793.449	6080.473554	0.681080502	0.041501787	16.4108716	1.60E-60	4.71E-59
ENSG00000213694	S1PR3	864.6923916	664.19349	1065.191294	0.680919543	0.101243983	6.72553097	1.75E-11	1.28E-10
ENSG00000104825	NFKBIB	940.336856	723.02562	1157.648096	0.678950069	0.081742245	8.30598757	9.90E-17	9.60E-16
ENSG00000136770	DNAJC1	1190.473088	915.91891	1465.027262	0.678511899	0.070031714	9.6886376	3.37E-22	4.12E-21
ENSG00000033867	SLC4A7	2832.658116	2178.2354	3487.080878	0.67841804	0.058508053	11.5952934	4.35E-31	7.17E-30
ENSG00000135926	TMBIM1	6262.676827	4817.7422	7707.611446	0.678153085	0.042997449	15.7719377	4.85E-56	1.31E-54
ENSG00000177426	TGIF1	1495.129198	1149.4962	1840.762217	0.677365047	0.065583869	10.3282263	5.25E-25	7.08E-24
ENSG00000253953	PCDHGB4	640.2825529	492.84171	787.7233985	0.675654973	0.099074143	6.81969034	9.12E-12	6.79E-11
ENSG00000162695	SLC30A7	1977.483763	1522.5164	2432.451086	0.674317782	0.061817174	10.9082595	1.05E-27	1.55E-26
ENSG00000188167	TMPPE	90.86140233	70.012665	111.7101395	0.67418158	0.238883927	2.82221407	0.004769	0.013229
ENSG00000122786	CALD1	20879.08317	16087.567	25670.59941	0.674101847	0.030891752	21.8214187	1.45E-105	7.15E-104
ENSG00000204525	HLA-C	36319.51588	27988.829	44650.20272	0.673935307	0.034553103	19.5043351	1.01E-84	3.96E-83
ENSG00000257354	AC048341.1	90.8185938	70.104651	111.5325368	0.673678674	0.231238073	2.91335533	0.003576	0.010219
ENSG00000148498	PARD3	929.2727118	716.70097	1141.844456	0.671842304	0.081611667	8.23218455	1.84E-16	1.76E-15
ENSG00000148803	FUOM	287.6799482	222.15498	353.2049148	0.671710981	0.131106681	5.12339247	3.00E-07	1.55E-06
ENSG00000112210	RAB23	791.7415383	610.22249	973.2605819	0.671386841	0.083144574	8.07493269	6.75E-16	6.29E-15
ENSG00000171928	TVP23B	1221.177152	941.34322	1501.011082	0.670749232	0.075020317	8.94090111	3.86E-19	4.15E-18
ENSG00000038382	TRIO	16039.83115	12379.047	19700.6155	0.670240784	0.029874676	22.4350812	1.79E-111	9.61E-110
ENSG00000157227	MMP14	45623.36206	35213.114	56033.61054	0.670182239	0.029918584	22.4001989	3.92E-111	2.08E-109
ENSG00000123146	ADGRE5	162.6715247	125.53088	199.8121681	0.669794843	0.189337873	3.53756401	0.000404	0.001393

ENSG00000129038	LOXL1	368.765207	284.24507	453.2853421	0.669738209	0.118189047	5.666669	1.46E-08	8.45E-08
ENSG00000086619	ERO1B	251.7013839	194.52455	308.8782225	0.669737924	0.153569816	4.36112995	1.29E-05	5.58E-05
ENSG00000166147	FBN1	25740.78416	19876.026	31605.54195	0.669123102	0.025683913	26.0522256	1.27E-149	9.62E-148
ENSG00000144935	TRPC1	194.35472	150.37242	238.3370222	0.668424017	0.15989307	4.18044394	2.91E-05	0.00012
ENSG00000204152	TIMM23B	131.7354314	101.47841	161.9924527	0.666892696	0.213254489	3.12721527	0.001765	0.00539
ENSG00000155304	HSPA13	2226.850303	1721.2827	2732.417905	0.666508446	0.057301764	11.631552	2.85E-31	4.70E-30
ENSG00000079385	CEACAM1	539.7030536	417.86107	661.5450328	0.665496054	0.11800642	5.63949023	1.71E-08	9.86E-08
ENSG00000143369	ECM1	116.9422052	90.613425	143.270985	0.665067474	0.209137992	3.1800414	0.001473	0.004571
ENSG00000146242	TPBG	513.8621789	397.59478	630.1295789	0.665028003	0.098730504	6.73579063	1.63E-11	1.19E-10
ENSG00000197780	TAF13	1136.194521	879.10422	1393.284825	0.664704113	0.067097613	9.90652401	3.90E-23	4.91E-22
ENSG00000146425	DYNLT1	3128.326377	2420.6325	3836.020233	0.664657427	0.046510407	14.2905098	2.51E-46	5.73E-45
ENSG00000019991	HGF	302.9552254	234.6174	371.293053	0.664471181	0.125609289	5.28998442	1.22E-07	6.54E-07
ENSG00000164182	NDUFAF2	537.4193757	415.88325	658.9554972	0.664000687	0.103958075	6.38719682	1.69E-10	1.15E-09
ENSG00000127838	PNKD	2013.123682	1558.6373	2467.610059	0.663850326	0.061294774	10.8304556	2.47E-27	3.60E-26
ENSG00000104419	NDRG1	3712.886489	2876.3087	4549.464301	0.661615787	0.047883428	13.8172186	2.01E-43	4.33E-42
ENSG00000142552	RCN3	2802.594009	2171.6998	3433.488259	0.661130602	0.049007719	13.4903361	1.78E-41	3.67E-40
ENSG00000135414	GDF11	996.1980055	773.00257	1219.393442	0.658245048	0.078021173	8.43674891	3.26E-17	3.23E-16
ENSG00000198680	TUSC1	251.1685044	194.87017	307.4668398	0.657679736	0.141871821	4.63573195	3.56E-06	1.64E-05
ENSG00000008441	NFIX	1421.92926	1103.7861	1740.072408	0.656374139	0.065293137	10.0527279	8.94E-24	1.15E-22
ENSG00000270959	LPP-AS2	157.5315892	122.19239	192.8707897	0.656256051	0.187362701	3.5025971	0.000461	0.001577
ENSG00000011638	TMEM159	591.733699	459.76772	723.6996807	0.656243578	0.093200503	7.04120209	1.91E-12	1.48E-11
ENSG00000110330	BIRC2	1961.863323	1523.5793	2400.147357	0.655967826	0.061542536	10.6587675	1.59E-26	2.26E-25
ENSG00000083799	CYLD	2270.523676	1763.1787	2777.868618	0.655498959	0.057201001	11.4595715	2.11E-30	3.37E-29
ENSG00000167987	VPS37C	1457.975002	1132.2674	1783.682573	0.655195812	0.072256889	9.06758956	1.22E-19	1.34E-18
ENSG00000100911	PSME2	2721.426041	2114.4434	3328.408639	0.654933074	0.046888527	13.9678748	2.45E-44	5.36E-43
ENSG00000171992	SYNPO	12989.92357	10093.478	15886.36914	0.654407897	0.036687212	17.8374935	3.62E-71	1.23E-69
ENSG00000184232	OAF	2396.929082	1861.8905	2931.967619	0.654122555	0.062474095	10.4703006	1.18E-25	1.64E-24
ENSG00000163900	TMEM41A	738.047978	573.92945	902.166506	0.653872175	0.088274944	7.40722277	1.29E-13	1.08E-12
ENSG00000267470	ZNF571-AS1	64.30628757	49.998184	78.61439094	0.653417181	0.275220621	2.37415779	0.017589	0.042746
ENSG00000166435	XRRA1	168.9436229	131.24106	206.6461865	0.653202333	0.171265201	3.81398165	0.000137	0.00051
ENSG00000179954	SSC5D	218.426421	169.85459	266.9982558	0.652683694	0.149430654	4.36780324	1.26E-05	5.42E-05
ENSG00000153560	UBP1	4657.863557	3622.0632	5693.663939	0.652636416	0.041530333	15.7146924	1.20E-55	3.23E-54
ENSG00000072274	TFRC	8318.00885	6467.3583	10168.65937	0.652485449	0.03430156	19.0220344	1.12E-80	4.21E-79
ENSG00000017260	ATP2C1	5886.329376	4578.5134	7194.145393	0.651483076	0.041923171	15.5399284	1.86E-54	4.94E-53
ENSG00000078399	HOXA9	231.4806897	180.30902	282.6523628	0.651275255	0.145329635	4.4813658	7.42E-06	3.30E-05
ENSG00000198818	SFT2D1	301.77878	234.88714	368.6704247	0.650767562	0.131664447	4.94262178	7.71E-07	3.84E-06
ENSG00000108387	4-Sep	109.5819799	85.626297	133.5376629	0.650197921	0.23518419	2.76463278	0.005699	0.015537
ENSG00000127418	FGFRL1	944.1613619	735.31389	1153.008835	0.648989178	0.074575695	8.70242207	3.25E-18	3.38E-17
ENSG00000171223	JUNB	1281.045098	998.47136	1563.618832	0.648222337	0.067633825	9.58429211	9.31E-22	1.12E-20
ENSG00000167703	SLC43A2	3095.57895	2411.7573	3779.400555	0.64777774	0.055577942	11.6553027	2.16E-31	3.57E-30
ENSG00000196712	NF1	1949.143871	1520.0726	2378.215121	0.645301537	0.054820456	11.7711814	5.49E-32	9.27E-31
ENSG00000074695	LMAN1	6324.837325	4932.6804	7716.994207	0.645101105	0.041182124	15.6645905	2.64E-55	7.07E-54
ENSG00000106785	TRIM14	801.0459667	625.20461	976.8873211	0.644794313	0.114446022	5.6340474	1.76E-08	1.02E-07
ENSG00000198113	TOR4A	2316.564096	1807.2861	2825.842129	0.644227398	0.056194241	11.4642957	1.99E-30	3.20E-29
ENSG00000257315	ZBED6	75.57177131	59.021477	92.12206551	0.643953557	0.253746706	2.53778095	0.011156	0.028457
ENSG00000175471	MCTP1	6228.186473	4860.9861	7595.386844	0.643903364	0.036129267	17.8222097	4.75E-71	1.61E-69
ENSG00000062716	VMP1	4464.544736	3487.8836	5441.205848	0.642418678	0.061184753	10.499653	8.67E-26	1.21E-24
ENSG00000141179	PCTP	1029.659769	804.88119	1254.438346	0.641824597	0.073128291	8.7766935	1.68E-18	1.77E-17
ENSG00000132694	ARHGEF11	2054.468563	1606.7018	2502.235356	0.640713488	0.055794965	11.4833567	1.60E-30	2.59E-29
ENSG00000167553	TUBA1C	9280.414219	7253.4086	11307.4198	0.640582721	0.042636036	15.0244436	5.08E-51	1.26E-49
ENSG00000132475	H3F3B	6220.744703	4862.281	7579.208405	0.640179175	0.036910239	17.3442164	2.18E-67	7.07E-66
ENSG00000092445	TYRO3	853.4192706	666.86681	1039.971732	0.63987328	0.080764932	7.92266231	2.32E-15	2.11E-14
ENSG00000206341	HLA-H	229.9667789	179.74901	280.1845516	0.639407697	0.146841202	4.35441612	1.33E-05	5.74E-05
ENSG00000078081	LAMP3	2336.918872	1829.0869	2844.750805	0.638933196	0.060623195	10.539418	5.68E-26	7.99E-25
ENSG00000073803	MAP3K13	510.7860278	399.97658	621.5954737	0.638118929	0.099269484	6.42814793	1.29E-10	8.87E-10
ENSG00000136231	IGF2BP3	3280.557009	2565.7857	3995.328269	0.637882323	0.047815104	13.340603	1.34E-40	2.71E-39
ENSG00000154556	SORBS2	7042.367404	5510.8937	8573.841152	0.637857355	0.035032155	18.2077681	4.48E-74	1.58E-72
ENSG00000140564	FURIN	13051.18373	10213.292	15889.07523	0.637594302	0.033651121	18.9471937	4.66E-80	1.74E-78
ENSG00000164050	PLXNB1	1971.585026	1544.246	2398.924094	0.636587891	0.055345026	11.5021699	1.29E-30	2.10E-29
ENSG00000104213	PDGFRL	685.6858144	536.85178	834.5198473	0.636416899	0.084790671	7.50574202	6.11E-14	5.18E-13
ENSG00000148358	GPR107	6290.375774	4925.9262	7654.825325	0.635741257	0.037541867	16.9341939	2.52E-64	7.74E-63
ENSG00000142694	EVA1B	1398.3269	1096.7584	1699.895437	0.633450482	0.065614725	9.65409031	4.72E-22	5.73E-21
ENSG00000112559	MDFI	300.7008111	235.79731	365.6043106	0.633409971	0.142775664	4.4364001	9.15E-06	4.02E-05
ENSG00000112667	DNPH1	609.6455144	477.91908	741.3719514	0.632728945	0.107686769	5.87564242	4.21E-09	2.56E-08
ENSG00000090661	CERS4	327.8048065	256.94357	398.6660417	0.632336299	0.123116577	5.13607764	2.81E-07	1.45E-06
ENSG00000135111	TBX3	138.2751816	108.52611	168.0242544	0.631309059	0.1874416	3.36803068	0.000757	0.002485
ENSG00000105520	PLPPR2	1947.721664	1530.24	2365.203353	0.630163659	0.064309211	9.79896431	1.14E-22	1.41E-21
ENSG00000125170	DOK4	273.4840773	214.50629	332.461864	0.629358307	0.133143469	4.72691835	2.28E-06	1.08E-05
ENSG00000075131	TIPIN	174.5306948	137.04223	212.0191546	0.629085011	0.169386434	3.71390433	0.000204	0.000739
ENSG00000132963	POMP	3003.285421	2358.2512	3648.31966	0.628472184	0.054157687	11.6044872	3.91E-31	6.45E-30
ENSG00000134247	PTGFRN	6407.802467	5033.3723	7782.232599	0.628450107	0.043165465	14.5590952	5.11E-48	1.21E-46
ENSG00000138031	ADCY3	2322.880051	1825.5116	2820.248515	0.628258561	0.053974691	11.6398733	2.58E-31	4.28E-30
ENSG00000204070	SYS1	960.1743595	754.79176	1165.556957	0.627290788	0.074245186	8.44890853	2.94E-17	2.92E-16
ENSG00000116704	SLC35D1	342.4792886	269.13931	415.819266	0.627271487	0.117267255	5.34907621	8.84E-08	4.79E-07
ENSG00000117226	GBP3	2588.424073	2034.7951	3142.053006	0.626882317	0.051763211	12.1105763	9.28E-34	1.63E-32
ENSG00000081087	OSTM1	3078.474131	2418.8225	3738.125718	0.626809582	0.054683388	11.4625229	2.03E-30	3.26E-29
ENSG00000228742	LINC02577	135.389633	106.61465	164.1646151	0.62644796	0.190591485	3.28686226	0.001013	0.003249
ENSG00000116641	DOCK7	1151.868108	906.14501	1397.591203	0.62631931	0.07096804	8.82537142	1.09E-18	1.16E-17
ENSG00000163517	HDAC11	426.8482989	335.38688	518.3097161	0.626209246	0.10810678	5.7925067	6.93E-09	4.15E-08
ENSG00000152137	HSPB8	437.4166298	343.99838	530.8348757	0.625988325	0.123550631	5.06665423	4.05E-07	2.07E-06
ENSG00000176261	ZBTB8OS	530.3822827	417.06752	643.6970477	0.625571767	0.099181554	6.30733985	2.84E-10	1.91E-09
ENSG00000196776	CD47	2410.542314	1895.9823	2925.102365	0.624769528	0.053382642	11.7036082	1.22E-31	2.04E-30
ENSG00000182247	UBE2E2	1061.93188	835.60547	1288.258292	0.624768051	0.071002988	8.79917973	1.38E-18	1.46E-17
ENSG00000150687	PRSS23	35918.58157	28261.507	43575.65579	0.624624553	0.036963117	16.8985897	4.61E-64	1.41E-62
ENSG00000174125	TLR1	83.20299727	65.260068	101.1459264	0.624553323	0.257589541	2.42460668	0.015325	0.037943

ENSG00000147065	MSN	63271.76276	49807.64	76735.88504	0.623485773	0.033611805	18.5496072	8.22E-77	2.99E-75
ENSG00000213859	KCTD11	439.9397528	345.81593	534.0635726	0.623394353	0.110590854	5.6369431	1.73E-08	9.99E-08
ENSG00000082996	RNF13	1449.188865	1140.4289	1757.948843	0.623072726	0.062536553	9.96333659	2.21E-23	2.81E-22
ENSG00000067066	SP100	6556.387895	5163.6462	7949.129614	0.622750138	0.040239401	15.4761285	5.03E-54	1.33E-52
ENSG00000164430	CGAS	172.0120652	135.60754	208.4165868	0.62248726	0.17347811	3.58827554	0.000333	0.001163
ENSG00000154553	PDLIM3	391.8404544	308.15041	475.5305015	0.621943429	0.136979586	4.54040961	5.61E-06	2.54E-05
ENSG00000140511	HAPLN3	566.0512838	446.6968	685.4057639	0.621808836	0.108995713	5.7048926	1.16E-08	6.83E-08
ENSG00000054983	GALC	1265.185275	996.10349	1534.267064	0.621261581	0.072446822	8.57541527	9.87E-18	1.00E-16
ENSG00000198018	ENTPD7	1185.291543	934.11308	1436.470004	0.621056985	0.065677669	9.45613629	3.20E-21	3.78E-20
ENSG00000128578	STRIP2	344.9600132	272.08483	417.8351978	0.619619355	0.124800577	4.96487573	6.87E-07	3.44E-06
ENSG00000159885	ZNF222	78.3666527	61.576638	95.15666702	0.617822481	0.260803368	2.36892063	0.01784	0.043281
ENSG00000119771	KLHL29	631.2560577	498.17792	764.3341961	0.617533721	0.087795621	7.03376445	2.01E-12	1.56E-11
ENSG00000197930	ERO1A	4365.625767	3446.3189	5284.932593	0.617111143	0.043781256	14.0953275	4.06E-45	9.06E-44
ENSG00000268516	AC020915.3	195.4887178	154.49484	236.4825932	0.616888251	0.157410109	3.91898752	8.89E-05	0.000341
ENSG00000103253	HAGHL	254.0657843	200.45158	307.679991	0.616290683	0.152540374	4.04018074	5.34E-05	0.000212
ENSG00000121578	B4GALT4	197.8661191	156.11464	239.6175961	0.616003582	0.163423914	3.76936011	0.000164	0.000602
ENSG00000175482	POLD4	307.3365841	243.12457	371.5485935	0.614740984	0.131342252	4.68045107	2.86E-06	1.34E-05
ENSG00000106683	LIMK1	3636.204103	2875.5214	4396.886823	0.612882211	0.047499044	12.903043	4.33E-38	8.35E-37
ENSG00000003056	M6PR	4086.546092	3232.6197	4940.472501	0.612200112	0.043243141	14.157161	1.69E-45	3.79E-44
ENSG00000131389	SLC6A6	1266.729139	1002.2039	1531.254416	0.611819893	0.086983855	7.03371782	2.01E-12	1.56E-11
ENSG00000135241	PNPLA8	1696.870369	1343.6624	2050.078336	0.611334974	0.063704964	9.59634755	8.28E-22	1.00E-20
ENSG00000118276	B4GALT6	856.0203695	676.57805	1035.462687	0.611122715	0.087973951	6.94663259	3.74E-12	2.85E-11
ENSG00000213516	RBMXL1	366.1053016	289.41667	442.7939372	0.609978906	0.134157814	4.54672664	5.45E-06	2.47E-05
ENSG00000154102	C16orf74	612.3008952	484.41491	740.1868754	0.6087891	0.103097732	5.90497084	3.53E-09	2.16E-08
ENSG00000257605	AC073611.2	82.03210967	64.902641	99.1615779	0.608443751	0.241559892	2.51881116	0.011775	0.029882
ENSG00000092969	TGFB2	1819.803961	1442.2939	2197.314016	0.607675316	0.07131813	8.52062881	1.59E-17	1.59E-16
ENSG00000117151	CTBS	606.8060645	480.62844	732.9836888	0.607496252	0.096122622	6.32001335	2.62E-10	1.76E-09
ENSG00000151229	SLC2A13	118.6806954	93.903138	143.4582532	0.607459597	0.206312292	2.94436939	0.003236	0.009347
ENSG00000254087	LYN	3613.590395	2863.3798	4363.800966	0.6073069	0.044724071	13.5789719	5.34E-42	1.11E-40
ENSG00000198046	ZNF667	87.86202348	69.499115	106.2249319	0.607228794	0.237374105	2.55810883	0.010524	0.026994
ENSG00000147905	ZCCHC7	694.0995225	550.31641	837.8826308	0.606856645	0.085910072	7.06385915	1.62E-12	1.26E-11
ENSG00000154380	ENAH	3980.380765	3154.5285	4806.233284	0.606780569	0.042002315	14.4463601	2.64E-47	6.15E-46
ENSG00000184014	DENND5A	6642.228933	5266.6933	8017.764525	0.606267177	0.037314733	16.2473942	2.33E-59	6.76E-58
ENSG00000042286	AIFM2	314.1802496	248.78449	379.5760108	0.605851864	0.131599634	4.60375036	4.15E-06	1.90E-05
ENSG00000158186	MRAS	759.9878227	602.45589	917.5197512	0.605801143	0.088608262	6.83684715	8.10E-12	6.04E-11
ENSG00000169871	TRIM56	4198.067621	3330.0691	5066.066158	0.60572279	0.04206904	14.3983033	5.30E-47	1.23E-45
ENSG00000235162	C12orf75	524.5954963	416.04209	633.148899	0.605648065	0.098035671	6.17783362	6.50E-10	4.24E-09
ENSG00000276547	PCDHGB5	123.5798922	97.807224	149.3525603	0.605617958	0.213871403	2.83169207	0.00463	0.012867
ENSG00000116117	PARD3B	1125.452174	893.04522	1357.859129	0.605106692	0.070149647	8.62594065	6.36E-18	6.52E-17
ENSG00000143322	ABL2	9543.714544	7571.2659	11516.16323	0.604698797	0.034552588	17.5008251	1.41E-68	4.67E-67
ENSG00000115520	COQ10B	1149.932036	912.37408	1387.489995	0.604647839	0.068050639	8.88526325	6.38E-19	6.81E-18
ENSG00000169826	CSGALNACT2	1931.971459	1532.5865	2331.356384	0.604601384	0.054467783	11.1001651	1.25E-28	1.89E-27
ENSG00000099308	MAST3	370.1131571	293.65788	446.5684313	0.604131107	0.124108953	4.867748	1.13E-06	5.52E-06
ENSG00000172057	ORMDL3	1285.59218	1019.8418	1551.342528	0.603566357	0.066536483	9.07120923	1.18E-19	1.30E-18
ENSG00000144655	CSRNP1	871.2099815	692.65827	1049.76169	0.602186541	0.078950247	7.62741811	2.40E-14	2.07E-13
ENSG00000166833	NAV2	7375.96453	5858.3413	8893.587788	0.602052899	0.037428454	16.0854334	3.23E-58	9.12E-57
ENSG00000163659	TIPARP	1864.479222	1480.8899	2248.068501	0.601164061	0.059338197	10.1311482	4.02E-24	5.23E-23
ENSG00000273311	DGCR11	183.9170965	146.16626	221.6679377	0.600646973	0.16062526	3.73943035	0.000184	0.000673
ENSG00000206503	HLA-A	41699.42192	33157.241	50241.60278	0.599669714	0.032313999	18.5575828	7.08E-77	2.58E-75
ENSG00000100558	PLEK2	91.38338232	72.551728	110.2150364	0.599562527	0.230923884	2.59636429	0.009422	0.024427
ENSG00000105173	CCNE1	252.9972141	201.1096	304.8848301	0.598979688	0.141073362	4.24587376	2.18E-05	9.13E-05
ENSG00000162729	IGSF8	579.8423273	461.74721	697.9374424	0.59887893	0.098548923	6.07697085	1.22E-09	7.80E-09
ENSG00000065911	MTHFD2	1429.860206	1137.8225	1721.897891	0.598362344	0.065792506	9.09468844	9.49E-20	1.05E-18
ENSG00000165650	PDZD8	1269.184412	1009.775	1528.593781	0.597528363	0.07155922	8.35012409	6.82E-17	6.66E-16
ENSG00000145780	FEM1C	1631.807591	1297.7798	1965.835401	0.59703961	0.065543257	9.10909284	8.31E-20	9.23E-19
ENSG00000183337	BCOR	1310.191593	1043.7446	1576.63859	0.596947295	0.068284913	8.74200854	2.29E-18	2.40E-17
ENSG00000151176	PLBD2	10604.33716	8442.9586	12765.71575	0.596880162	0.039602726	15.0716938	2.49E-51	6.22E-50
ENSG00000128595	CALU	23443.29683	18664.376	28222.21753	0.596496424	0.030029041	19.863985	8.34E-88	3.40E-86
ENSG00000142949	PTPRF	37750.47697	30065.258	45435.69604	0.595638008	0.026628753	22.3682276	8.03E-111	4.22E-109
ENSG00000166889	PATL1	2401.665985	1914.8109	2888.521021	0.59407334	0.056195351	10.5715745	4.04E-26	5.71E-25
ENSG00000276256	AC011043.1	280.4644107	223.34305	337.5857752	0.593337859	0.134505686	4.41124742	1.03E-05	4.49E-05
ENSG00000100139	MICALL1	4923.38856	3925.2531	5921.524066	0.592994466	0.045060262	13.1600316	1.49E-39	2.95E-38
ENSG00000119636	BBOF1	147.2420405	117.56254	176.9215443	0.592845381	0.18404865	3.22113409	0.001277	0.004017
ENSG00000066629	EML1	1539.986273	1228.5159	1851.456611	0.592323048	0.062060288	9.54431677	1.37E-21	1.64E-20
ENSG00000013375	PGM3	3423.21416	2730.468	4115.960359	0.591657406	0.045015525	13.143408	1.86E-39	3.66E-38
ENSG00000135604	STX11	337.7296647	269.65815	405.8011834	0.591098105	0.128826221	4.58833691	4.47E-06	2.04E-05
ENSG00000119048	UBE2B	2056.8445	1640.9287	2472.76033	0.590836065	0.052836244	11.1824009	4.97E-29	7.63E-28
ENSG00000143367	TUFT1	248.1382983	197.84749	298.429111	0.590677107	0.151255812	3.90515313	9.42E-05	0.000361
ENSG00000111906	HDDC2	225.8764272	180.21644	271.5364146	0.59005605	0.157534081	3.74557713	0.00018	0.000658
ENSG00000106397	PLOD3	7943.930632	6342.2627	9545.598519	0.589565863	0.033723032	17.4825876	1.94E-68	6.41E-67
ENSG00000148426	PROSER2	398.5158882	317.72591	479.3058632	0.589092609	0.124787212	4.72077707	2.35E-06	1.11E-05
ENSG00000131724	IL13RA1	7261.299866	5800.2078	8722.391947	0.588361937	0.040002505	14.7081274	5.72E-49	1.38E-47
ENSG00000204520	MICA	1694.125234	1355.1186	2033.131864	0.585916332	0.057258047	10.2329081	1.41E-24	1.88E-23
ENSG00000143702	CEP170	7223.33224	5776.7812	8669.883319	0.585679083	0.037832558	15.4808217	4.67E-54	1.23E-52
ENSG00000005238	FAM214B	2921.398057	2337.2895	3505.506634	0.585270628	0.045572693	12.8425727	9.47E-38	1.81E-36
ENSG00000158158	CNNM4	423.5246167	338.89612	508.1531184	0.585113972	0.10985561	5.32620929	1.00E-07	5.40E-07
ENSG00000151151	IPMK	772.4772057	618.01008	926.9443295	0.584279284	0.08534263	6.84627698	7.58E-12	5.67E-11
ENSG00000170266	GLB1	5705.835266	4568.1575	6843.513022	0.583095051	0.040813938	14.2866647	2.65E-46	6.04E-45
ENSG00000123094	RASSF8	3501.598441	2804.0573	4199.139538	0.582420572	0.047965036	12.1426069	6.28E-34	1.11E-32
ENSG00000205084	TMEM231	423.4876887	338.76734	508.2080331	0.581861196	0.113982432	5.10483224	3.31E-07	1.70E-06
ENSG00000099139	PCSK5	109.2270606	87.321886	131.1322353	0.581102464	0.221372375	2.62499991	0.008665	0.022705
ENSG00000109861	CTSC	3246.179587	2601.9781	3890.381108	0.580714254	0.04717902	12.3087393	8.13E-35	1.46E-33
ENSG00000205683	DPF3	274.3663981	220.21484	328.5179586	0.579821534	0.133783783	4.33401957	1.46E-05	6.25E-05
ENSG00000100403	ZC3H7B	13015.7802	10440.556	15591.00489	0.578585959	0.039798708	14.5378075	6.98E-48	1.65E-46
ENSG00000096070	BRPF3	2098.828105	1684.6725	2512.98375	0.578196829	0.056847604	10.1709973	2.67E-24	3.51E-23

ENSG00000149925	ALDOA	247.0468893	198.58436	295.5094205	0.577575718	0.154068018	3.74883591	0.000178	0.00065
ENSG00000228649	SNHG26	92.78608074	74.630221	110.9419401	0.575587896	0.229219809	2.51107397	0.012036	0.030476
ENSG00000075568	TMEM131	3853.288387	3093.9823	4612.594486	0.575404127	0.045070103	12.7668696	2.51E-37	4.77E-36
ENSG00000131871	SELENOS	1836.66111	1475.7043	2197.617893	0.575144182	0.055102558	10.4377038	1.67E-25	2.30E-24
ENSG00000010626	LRRC23	132.3296435	106.28043	158.3788552	0.574173004	0.189428065	3.03108731	0.002437	0.007223
ENSG00000103653	CSK	2905.129303	2334.7704	3475.488196	0.573527936	0.047017574	12.198161	3.18E-34	5.67E-33
ENSG00000119681	LTBP2	38855.05965	31232.636	46477.48336	0.573438831	0.03831388	14.9668691	1.21E-50	2.99E-49
ENSG00000149577	SIDT2	1431.331815	1149.8865	1712.777095	0.573212929	0.066511145	8.6182989	6.80E-18	6.95E-17
ENSG00000118508	RAB32	2765.538025	2224.5659	3306.51016	0.573009144	0.051921963	11.0359684	2.56E-28	3.83E-27
ENSG00000145623	OSMR	1149.624784	924.26335	1374.986219	0.572894041	0.068875247	8.31785098	8.96E-17	8.71E-16
ENSG00000111885	MAN1A1	2683.349944	2156.751	3209.948914	0.572856816	0.050967539	11.2396405	2.60E-29	4.04E-28
ENSG00000155465	SLC7A7	1609.237682	1293.9333	1924.542087	0.572826027	0.078113557	7.33324727	2.25E-13	1.85E-12
ENSG00000270194	AC097359.2	79.83447332	64.176285	95.49266155	0.572639574	0.24279923	2.35849007	0.018349	0.044404
ENSG00000160058	BSDC1	5423.267285	4364.1235	6482.41104	0.571597683	0.040608903	14.0756741	5.36E-45	1.19E-43
ENSG00000107731	UNC5B	385.3384904	310.36206	460.3149162	0.57121299	0.114696113	4.98022972	6.35E-07	3.18E-06
ENSG00000178764	ZHX2	597.6954548	481.41681	713.9741004	0.569867619	0.092205797	6.18038821	6.39E-10	4.17E-09
ENSG00000151287	TEX30	331.8712796	267.58958	396.1529814	0.569779619	0.136412553	4.17688552	2.96E-05	0.000122
ENSG00000166130	IKBIP	2409.295879	1941.2445	2877.347227	0.567698982	0.051074746	11.1150623	1.06E-28	1.60E-27
ENSG00000197256	KANK2	2226.222985	1794.8352	2657.610814	0.567682706	0.055463245	10.2352956	1.38E-24	1.83E-23
ENSG00000159842	ABR	5308.901256	4281.9998	6335.802669	0.565181647	0.03863131	14.6301446	1.80E-48	4.30E-47
ENSG00000143162	CREG1	2621.313044	2114.2131	3128.413013	0.565136626	0.066355273	8.51683074	1.64E-17	1.64E-16
ENSG00000179104	TMTC2	484.1001151	390.46048	577.7397542	0.564441774	0.102465179	5.50862037	3.62E-08	2.03E-07
ENSG00000168778	TCTN2	493.9353997	398.91527	588.9555341	0.563626841	0.099199967	5.68172405	1.33E-08	7.77E-08
ENSG00000103335	PIEZO1	26538.05071	21423.956	31652.14499	0.56320728	0.027004661	20.8559284	1.35E-96	6.08E-95
ENSG00000143801	PSEN2	1076.88783	868.76068	1285.014979	0.562589549	0.082746625	6.79894252	1.05E-11	7.82E-11
ENSG00000102547	CAB39L	268.5701154	216.81586	320.3243725	0.561696376	0.13591916	4.13257687	3.59E-05	0.000146
ENSG00000159348	CYB5R1	1568.492446	1267.1722	1869.81272	0.561217588	0.063151653	8.88682337	6.29E-19	6.72E-18
ENSG00000137103	TMEM8B	114.6502207	92.675969	136.6244722	0.560121837	0.215430622	2.6000103	0.009322	0.0242
ENSG00000137177	KIF13A	6188.013078	5002.4828	7373.543404	0.559436903	0.03519656	15.8946471	6.90E-57	1.90E-55
ENSG00000127191	TRAF2	647.5460912	523.7345	771.3576793	0.559342776	0.089090318	6.27837891	3.42E-10	2.28E-09
ENSG00000146094	DOK3	210.1377056	169.71565	250.5597651	0.558699197	0.154107146	3.62539448	0.000289	0.00102
ENSG00000114405	C3orf14	95.29517938	76.978451	113.6119081	0.558054497	0.224374569	2.48715574	0.012877	0.032429
ENSG00000151773	CCDC122	106.1374382	86.089187	126.1856894	0.55801235	0.216680018	2.57528292	0.010016	0.025795
ENSG00000164713	BRI3	5381.505382	4356.1896	6406.82112	0.557663223	0.046471636	12.0000774	3.55E-33	6.15E-32
ENSG00000177628	GBA	4572.268384	3700.5747	5443.962098	0.556980688	0.040461256	13.7657785	4.09E-43	8.78E-42
ENSG00000164171	ITGA2	8817.105548	7137.6948	10496.51629	0.556101003	0.04162139	13.3609425	1.02E-40	2.07E-39
ENSG00000137834	SMAD6	229.7852741	186.03613	273.5344174	0.555722113	0.155047251	3.58421134	0.000338	0.00118
ENSG00000177191	B3GNT8	118.3096732	95.946673	140.6726732	0.5553699	0.198684332	2.79523752	0.005186	0.014263
ENSG00000154328	NEIL2	357.2776929	289.16999	425.3853985	0.555266103	0.116433459	4.76895653	1.85E-06	8.83E-06
ENSG00000171206	TRIM8	5161.152847	4180.7408	6141.564937	0.554719013	0.039546743	14.0269204	1.07E-44	2.36E-43
ENSG00000166979	EVA1C	1095.935203	888.08273	1303.787681	0.554306897	0.071854482	7.7142981	1.22E-14	1.06E-13
ENSG00000143164	DCAF6	2908.970633	2356.2862	3461.655025	0.553707582	0.050676776	10.9262591	8.63E-28	1.27E-26
ENSG00000137752	CASP1	830.0912505	673.58172	986.6007803	0.553686497	0.086778299	6.3804719	1.77E-10	1.20E-09
ENSG00000112365	ZBTB24	329.1003569	266.46057	391.7401468	0.553456708	0.121770085	4.54509585	5.49E-06	2.48E-05
ENSG00000157600	TMEM164	874.6883283	709.62033	1039.756324	0.552690422	0.085543294	6.46094392	1.04E-10	7.21E-10
ENSG00000132964	CDK8	607.7183643	492.16631	723.2704202	0.552263945	0.098722352	5.59411253	2.22E-08	1.27E-07
ENSG00000085998	POMGNT1	3400.796793	2758.2678	4043.325833	0.551916082	0.045443034	12.1452296	6.08E-34	1.07E-32
ENSG00000141428	C18orf21	338.4813813	274.03559	402.9271702	0.551627204	0.130766749	4.21840575	2.46E-05	0.000102
ENSG00000162851	TFB2M	443.3101323	360.11382	526.5064488	0.550143896	0.112692074	4.88183311	1.05E-06	5.15E-06
ENSG00000184840	TMED9	10296.43184	8357.758	12235.10565	0.549911166	0.034861812	15.7740271	4.70E-56	1.27E-54
ENSG00000143545	RAB13	864.1629167	701.12832	1027.197515	0.54929753	0.080880946	6.79143305	1.11E-11	8.22E-11
ENSG00000105185	PDCD5	1636.147699	1328.9222	1943.373182	0.548757948	0.058432435	9.39132427	5.93E-21	6.92E-20
ENSG00000110057	UNC93B1	563.1098311	457.90643	668.3132369	0.548734295	0.10685392	5.13536889	2.82E-07	1.46E-06
ENSG00000161040	FBXL13	212.2365893	172.22405	252.2491303	0.547916963	0.157107544	3.48752802	0.000488	0.00166
ENSG00000137821	LRRC49	158.5657028	129.17531	187.9561	0.54787577	0.184896336	2.96315104	0.003045	0.008833
ENSG00000170145	SIK2	2590.727265	2103.274	3078.18056	0.547801705	0.054100627	10.1256073	4.25E-24	5.53E-23
ENSG00000131067	GGT7	188.0186959	153.02139	223.0160008	0.545675781	0.168967897	3.22946424	0.00124	0.00391
ENSG00000055208	TAB2	2769.217655	2251.9223	3286.513012	0.545174096	0.050933506	10.7036436	9.79E-27	1.41E-25
ENSG00000148842	CNNM2	241.8754719	196.61676	287.134182	0.54493397	0.158733847	3.43300424	0.000597	0.002003
ENSG00000184916	JAG2	4759.151854	3872.2641	5646.039569	0.544614188	0.046723201	11.656183	2.13E-31	3.54E-30
ENSG00000135503	ACVR1B	1080.67664	878.20673	1283.146551	0.544136182	0.080178136	6.78659053	1.15E-11	8.49E-11
ENSG00000132003	ZSWIM4	1229.948214	1001.8154	1458.081039	0.543339804	0.068210358	7.96564951	1.64E-15	1.51E-14
ENSG00000188786	MTF1	1133.55673	921.89808	1345.215377	0.542995069	0.07434383	7.30383502	2.80E-13	2.28E-12
ENSG00000198346	ZNF813	222.7590731	181.30057	264.2175793	0.542308708	0.154052076	3.52029471	0.000431	0.001482
ENSG00000064763	FAR2	1750.298879	1425.2079	2075.389906	0.541971118	0.057363791	9.44796549	3.45E-21	4.08E-20
ENSG00000179476	C14orf28	119.2701666	96.984834	141.5554993	0.541913284	0.20909016	2.59176847	0.009548	0.024721
ENSG00000187720	THSD4	8004.021067	6518.6474	9489.394688	0.541680382	0.035944693	15.0698291	2.56E-51	6.38E-50
ENSG00000196428	TSC22D2	1509.302002	1229.6545	1788.949525	0.54059681	0.062657894	8.62775265	6.26E-18	6.42E-17
ENSG00000156671	SAMD8	1920.357087	1564.9313	2275.782874	0.540072497	0.053231952	10.1456451	3.46E-24	4.52E-23
ENSG00000116199	FAM20B	2655.782393	2165.107	3146.457781	0.539601624	0.047930426	11.2580185	2.11E-29	3.29E-28
ENSG00000132294	EFR3A	3425.056859	2791.5588	4058.554873	0.539454885	0.046953795	11.4890581	1.50E-30	2.43E-29
ENSG00000177663	IL17RA	1418.93649	1156.3463	1681.526658	0.539446257	0.065901798	8.18560763	2.71E-16	2.57E-15
ENSG00000144369	FAM171B	1414.830511	1152.8489	1676.812131	0.539047253	0.063673232	8.46583784	2.54E-17	2.53E-16
ENSG00000139112	GABARAPL1	2942.584539	2401.1267	3484.042415	0.53775417	0.050252969	10.7009433	1.01E-26	1.45E-25
ENSG00000127124	HIVEP3	185.5162256	151.37784	219.6546067	0.537624644	0.166544632	3.22811152	0.001246	0.003927
ENSG00000169891	REPS2	191.8667329	156.29323	227.4402338	0.537344857	0.165805172	3.24082084	0.001192	0.003772
ENSG00000077147	TM9SF3	9425.278017	7694.0949	11156.46112	0.535951948	0.034176418	15.6819229	2.01E-55	5.39E-54
ENSG00000142528	ZNF473	274.3040867	224.33346	324.2747123	0.535484861	0.148769801	3.5994191	0.000319	0.001118
ENSG00000155846	PPARGC1B	129.1216943	105.34581	152.8975773	0.535255355	0.189872192	2.81902974	0.004817	0.013347
ENSG00000254838	GVINP1	715.5959207	584.60345	846.5883894	0.535252759	0.087228591	6.1362078	8.45E-10	5.46E-09
ENSG00000134049	IER3IP1	1055.037606	861.43842	1248.636795	0.535224386	0.086507052	6.18706073	6.13E-10	4.01E-09
ENSG00000253537	PCDHGA7	196.935265	160.43244	233.4380888	0.53507753	0.187764282	2.84973012	0.004376	0.012223
ENSG00000239521	CASTOR3	393.8947921	322.34781	465.4417718	0.534926422	0.123327245	4.33745537	1.44E-05	6.16E-05
ENSG00000163453	IGFBP7	27621.09463	22559.406	32682.78305	0.534743411	0.034023423	15.71692	1.16E-55	3.12E-54
ENSG00000181396	OGFOD3	1192.746368	974.24	1411.252735	0.534574137	0.071838668	7.4413147	9.97E-14	8.36E-13

ENSG00000196743	GM2A	3140.781718	2565.8351	3715.728365	0.53420612	0.047392075	11.2720559	1.80E-29	2.81E-28
ENSG00000161091	MFSD12	2165.822529	1771.1082	2560.536905	0.533406692	0.067605822	7.88995204	3.02E-15	2.73E-14
ENSG00000254245	PCDHGA3	284.5394019	232.46865	336.6101581	0.533317628	0.130876414	4.07497126	4.60E-05	0.000185
ENSG00000166896	ATP23	161.547442	132.33254	190.7623398	0.531729341	0.185231239	2.87062454	0.004097	0.011541
ENSG00000090674	MCOLN1	1396.578694	1143.299	1649.858382	0.53012656	0.072578897	7.30414187	2.79E-13	2.28E-12
ENSG00000185483	ROR1	365.5117247	298.97558	432.0478696	0.530028984	0.121693055	4.35545795	1.33E-05	5.72E-05
ENSG00000185033	SEMA4B	551.4608545	451.29708	651.6246332	0.529635422	0.113180781	4.67955262	2.88E-06	1.34E-05
ENSG00000142556	ZNF614	327.8446568	268.33089	387.3584207	0.529403409	0.121823348	4.34566458	1.39E-05	5.95E-05
ENSG00000116260	QSOX1	16420.53065	13443.246	19397.81503	0.528980144	0.029184926	18.1251149	2.02E-73	7.06E-72
ENSG00000116044	NFE2L2	4054.51103	3319.0094	4790.012646	0.528969522	0.040422077	13.086154	3.95E-39	7.75E-38
ENSG00000106392	C1GALT1	1249.948713	1022.5916	1477.305799	0.528897553	0.076756792	6.89056352	5.56E-12	4.19E-11
ENSG00000105612	DNASE2	3150.788408	2580.1459	3721.430879	0.528853273	0.050952557	10.3793274	3.08E-25	4.20E-24
ENSG00000196511	TPK1	769.7861015	631.1037	908.4684993	0.528034417	0.088800409	5.94630616	2.74E-09	1.70E-08
ENSG00000203778	FAM229B	181.9819544	149.21433	214.7495824	0.527926308	0.16134702	3.27199292	0.001068	0.003409
ENSG00000137285	TUBB2B	135.5959364	111.18525	160.0066201	0.527411858	0.192497001	2.73984454	0.006147	0.016634
ENSG00000140931	CMTM3	3122.719331	2557.9265	3687.512126	0.527402163	0.054533372	9.67118188	4.00E-22	4.86E-21
ENSG00000145860	RNF145	4563.609977	3738.3349	5388.885092	0.527072322	0.044082438	11.9565148	6.00E-33	1.03E-31
ENSG00000137802	MAPKBP1	1835.436413	1504.5296	2166.343186	0.526994409	0.060980109	8.64207062	5.52E-18	5.68E-17
ENSG00000204209	DAXX	2490.379401	2041.4765	2939.282336	0.52617621	0.052790363	9.96727774	2.12E-23	2.70E-22
ENSG00000183060	LYSMD4	317.5971722	260.41893	374.7754136	0.526008545	0.140868517	3.73403906	0.000188	0.000686
ENSG00000115255	REEP6	98.84218087	80.802699	116.8816632	0.525934707	0.22699717	2.31692187	0.020508	0.048931
ENSG00000101160	CTSZ	12416.95982	10178.295	14655.62495	0.525662797	0.033248276	15.8102272	2.65E-56	7.23E-55
ENSG00000140450	ARRDC4	150.3482979	123.10617	177.5904222	0.525639855	0.203171153	2.58717759	0.009677	0.025028
ENSG00000174437	ATP2A2	14989.59053	12288.176	17691.00495	0.525607747	0.028670698	18.3325761	4.55E-75	1.62E-73
ENSG00000068366	ACSL4	5280.852102	4327.9049	6233.799313	0.525503543	0.042212577	12.4489803	1.42E-35	2.60E-34
ENSG00000151694	ADAM17	4547.284437	3728.6086	5365.96032	0.525192937	0.038708062	13.5680504	6.19E-42	1.29E-40
ENSG00000157800	SLC37A3	1725.6619	1415.526	2035.797806	0.524963748	0.057000605	9.20979248	3.27E-20	3.70E-19
ENSG00000105327	BBC3	1206.385672	990.47877	1422.292573	0.524625699	0.07337276	7.15014259	8.67E-13	6.87E-12
ENSG00000117385	P3H1	5255.675267	4313.6138	6197.73676	0.523597756	0.041351365	12.6621638	9.58E-37	1.80E-35
ENSG00000153071	DAB2	16779.31061	13768.423	19790.19845	0.52322314	0.029739591	17.5934883	2.76E-69	9.26E-68
ENSG00000127463	EMC1	6104.832649	5008.9646	7200.70074	0.523068841	0.042443857	12.323782	6.75E-35	1.21E-33
ENSG00000103855	CD276	4929.509247	4044.8569	5814.161585	0.523027035	0.044716388	11.6965402	1.33E-31	2.21E-30
ENSG00000175634	RPS6KB2	1270.085263	1042.4863	1497.684205	0.522332279	0.067131393	7.78074548	7.21E-15	6.39E-14
ENSG00000112378	PERP	3719.167343	3051.8936	4386.441095	0.522103827	0.046871574	11.139029	8.10E-29	1.23E-27
ENSG00000148110	MFSD14B	3648.801705	2995.8245	4301.778919	0.521945181	0.042806691	12.1930747	3.38E-34	6.02E-33
ENSG00000164291	ARSK	453.2368372	372.41616	534.0575172	0.521608262	0.106153134	4.91373397	8.94E-07	4.41E-06
ENSG00000109220	CHIC2	798.361499	655.46107	941.2619272	0.52148106	0.084105413	6.200327	5.63E-10	3.69E-09
ENSG00000180353	HCLS1	3685.059164	3026.4472	4343.671081	0.521314625	0.047574951	10.9577542	6.10E-28	9.01E-27
ENSG00000167657	DAPK3	6613.43637	5433.2224	7793.650372	0.520734311	0.036038717	14.4493022	2.53E-47	5.90E-46
ENSG00000168806	LCMT2	241.9062391	198.40152	285.4109546	0.520611685	0.154211959	3.37594885	0.000736	0.002425
ENSG00000196850	PPTC7	1054.833446	866.81728	1242.851604	0.520160734	0.075122536	6.92416366	4.39E-12	3.33E-11
ENSG00000169967	MAP3K2	930.284363	764.64352	1095.925202	0.519209332	0.078371391	6.62498558	3.47E-11	2.49E-10
ENSG00000116017	ARID3A	979.7914578	806.05272	1153.530198	0.517136109	0.07855862	6.5828054	4.62E-11	3.28E-10
ENSG00000149541	B3GAT3	1095.445175	901.66398	1289.226369	0.516739614	0.071065855	7.27127841	3.56E-13	2.89E-12
ENSG00000116786	PLEKHM2	4714.741261	3879.9668	5549.515733	0.516585879	0.042533508	12.1453863	6.07E-34	1.07E-32
ENSG00000130779	CLIP1	7604.686773	6258.2779	8951.09568	0.516485315	0.034399247	15.0144366	5.91E-51	1.46E-49
ENSG00000267317	AC027307.2	122.3763493	100.56635	144.1863443	0.514514885	0.211033987	2.43806646	0.014766	0.036664
ENSG00000013374	NUB1	2796.642044	2302.6203	3290.663811	0.51443376	0.048459368	10.6157752	2.52E-26	3.58E-25
ENSG00000144724	PTPRG	2342.309092	1928.5331	2756.085061	0.514273869	0.050780418	10.1274051	4.18E-24	5.43E-23
ENSG00000114446	IFT57	665.7570992	548.74569	782.7685046	0.513545931	0.085647493	5.99604157	2.02E-09	1.26E-08
ENSG00000213753	CENPBD1P1	858.4523976	707.56381	1009.340981	0.512890682	0.090496081	5.66754581	1.45E-08	8.41E-08
ENSG00000100097	LGALS1	47358.54876	39032.079	55685.0187	0.512650282	0.031964567	16.0380801	6.93E-58	1.93E-56
ENSG00000125266	EFNB2	3067.695286	2527.8809	3607.509709	0.512566669	0.060773295	8.43407738	3.34E-17	3.31E-16
ENSG00000082641	NFE2L1	28817.99484	23753.782	33882.20722	0.512423139	0.027272568	18.7889584	9.30E-79	3.47E-77
ENSG00000112290	WASF1	665.0090648	548.50776	781.5103726	0.511232888	0.098154649	5.20844027	1.90E-07	1.00E-06
ENSG00000142871	CCN1	34662.41392	28588.829	40735.99876	0.510931065	0.028541818	17.9011395	1.16E-71	3.96E-70
ENSG00000077585	GPR137B	758.7043458	625.79875	891.6099386	0.510826615	0.085328449	5.98659206	2.14E-09	1.34E-08
ENSG00000103415	HMOX2	3915.435265	3229.2537	4601.61685	0.510557466	0.046360212	11.0128371	3.31E-28	4.93E-27
ENSG00000084207	GSTP1	10135.77772	8360.6088	11910.94659	0.510526845	0.037546332	13.5972494	4.16E-42	8.72E-41
ENSG00000138764	CCNG2	1613.387681	1330.5901	1896.1853	0.510080587	0.061859395	8.24580629	1.64E-16	1.57E-15
ENSG00000150403	TMCO3	5064.012798	4178.0232	5950.002375	0.509860125	0.041251425	12.3598186	4.31E-35	7.79E-34
ENSG00000084764	MAPRE3	211.9191858	174.8194	249.0189732	0.509142598	0.15251148	3.33838868	0.000843	0.002741
ENSG00000040275	SPDL1	695.429656	573.98035	816.8789578	0.508352024	0.084245883	6.0341468	1.60E-09	1.01E-08
ENSG00000172638	EFEMP2	1869.748126	1544.9307	2194.565562	0.50816826	0.063849804	7.95880684	1.74E-15	1.59E-14
ENSG00000122884	P4HA1	1538.340037	1270.4614	1806.218681	0.50802838	0.064386833	7.89025259	3.02E-15	2.72E-14
ENSG00000144152	FBLN7	247.047786	204.08593	290.009644	0.507995894	0.139491048	3.64178133	0.000271	0.000962
ENSG00000214114	MYCBP	211.8345679	174.96204	248.7070931	0.506840851	0.155725288	3.25471128	0.001135	0.003605
ENSG00000185896	LAMP1	37296.51637	30815.897	43777.13558	0.506607361	0.029250889	17.3193834	3.36E-67	1.08E-65
ENSG00000149177	PTPRJ	166.1936889	137.42869	194.958686	0.506307552	0.200212562	2.52885007	0.011444	0.02911
ENSG00000009830	POMT2	1078.908086	891.27616	1266.540016	0.506009927	0.077256424	6.54974564	5.76E-11	4.08E-10
ENSG00000082146	STRADB	1079.102838	891.66884	1266.536835	0.505887919	0.072653905	6.9629832	3.33E-12	2.55E-11
ENSG00000138835	RGS3	13524.08023	11177.615	15870.5459	0.50582129	0.031896802	15.8580564	1.24E-56	3.38E-55
ENSG00000125912	NCLN	3648.277319	3016.0803	4280.474385	0.505537431	0.05511292	9.17275724	4.61E-20	5.19E-19
ENSG00000158669	GPAT4	4447.049946	3674.911	5219.188973	0.505283418	0.043114049	11.719693	1.01E-31	1.69E-30
ENSG00000091844	RGS17	232.3605934	192.28598	272.4352092	0.504268999	0.155431672	3.24431304	0.001177	0.003732
ENSG00000175166	PSMD2	18303.76924	15139.073	21468.46517	0.504013368	0.035923022	14.0303723	1.02E-44	2.25E-43
ENSG00000182963	GJC1	1002.741676	829.44129	1176.042059	0.503447095	0.07350296	6.84934446	7.42E-12	5.55E-11
ENSG00000182218	HHIPL1	406.0183497	335.47398	476.5627194	0.50322714	0.113935692	4.41676467	1.00E-05	4.39E-05
ENSG00000197312	DDI2	2014.82483	1666.2373	2363.412363	0.50321263	0.053941752	9.32881509	1.07E-20	1.24E-19
ENSG00000128039	SRD5A3	1063.087635	880.36743	1245.80784	0.502633909	0.081384088	6.17607102	6.57E-10	4.28E-09
ENSG00000165097	KDM1B	467.4650055	386.71325	548.2167585	0.502499508	0.102886821	4.88400265	1.04E-06	5.11E-06
ENSG00000267107	PCAT19	976.4146681	808.43817	1144.391165	0.501900674	0.07179759	6.99049477	2.74E-12	2.10E-11
ENSG00000174173	TRMT10C	739.4608932	612.0076	866.9141846	0.501505936	0.081627214	6.14385706	8.05E-10	5.22E-09
ENSG00000172115	CYCS	3029.392316	2507.9893	3550.795313	0.50147366	0.045127338	11.112414	1.09E-28	1.65E-27
ENSG00000101363	MANBAL	1054.301626	873.08579	1235.517464	0.50146622	0.072791704	6.88905733	5.62E-12	4.23E-11

ENSG00000163191	S100A11	8571.9952	7098.8452	10045.14521	0.501107541	0.039591381	12.6569855	1.02E-36	1.92E-35
ENSG00000273149	AL138963.3	735.7877707	610.07056	861.5049773	0.500484659	0.096214525	5.20175784	1.97E-07	1.04E-06
ENSG00000091483	FH	2056.165195	1703.0639	2409.266454	0.500225737	0.05343835	9.36080061	7.91E-21	9.20E-20
ENSG00000255717	SNHG1	258.0120857	302.6163	213.4078716	-0.500429624	0.157172046	-3.1839607	0.001453	0.004518
ENSG00000132780	NASP	1847.897598	2166.0984	1529.696833	-0.501357136	0.059145734	-8.4766407	2.32E-17	2.32E-16
ENSG00000221886	ZBED8	108.7441172	127.44283	90.04540102	-0.501554896	0.210496672	-2.3827213	0.017185	0.041882
ENSG00000197070	ARRDC1	389.104508	456.22625	321.9827708	-0.501666698	0.119485276	-4.198565	2.69E-05	0.000111
ENSG00000063176	SPHK2	665.4079833	780.03901	550.7769564	-0.501864084	0.093743316	-5.3535986	8.62E-08	4.68E-07
ENSG00000176155	CCDC57	205.0770296	240.70105	169.4530089	-0.501891408	0.185282061	-2.7087966	0.006753	0.018139
ENSG00000120458	MSANTD2	249.7358869	292.82315	206.6486206	-0.503360377	0.153520907	-3.2787741	0.001043	0.003337
ENSG00000198720	ANKRD13B	722.230822	846.99906	597.4625805	-0.503671834	0.090758382	-5.5495902	2.86E-08	1.62E-07
ENSG00000197182	MIRLET7BHG	116.0603467	136.31341	95.80728387	-0.503679508	0.21186054	-2.3774107	0.017435	0.042433
ENSG00000166900	STX3	3286.045663	3854.8643	2717.227014	-0.504198944	0.048065825	-10.48976	9.63E-26	1.34E-24
ENSG00000197943	PLCG2	681.8648645	799.5479	564.181831	-0.504441828	0.090607289	-5.5673427	2.59E-08	1.47E-07
ENSG00000137486	ARRB1	7876.836639	9239.3392	6514.334102	-0.504493408	0.042084785	-11.987549	4.13E-33	7.13E-32
ENSG00000131153	GINS2	170.3957858	200.08492	140.7066553	-0.505428813	0.181055063	-2.7915751	0.005245	0.0144
ENSG00000155849	ELMO1	1457.638978	1710.6129	1204.665059	-0.505726102	0.064972759	-7.7836636	7.05E-15	6.25E-14
ENSG00000157514	TSC22D3	415.5079143	487.66276	343.3530718	-0.506106377	0.108744073	-4.6541054	3.25E-06	1.51E-05
ENSG00000198001	IRAK4	796.7981636	935.26629	658.3300394	-0.506548117	0.082940069	-6.1073993	1.01E-09	6.49E-09
ENSG00000276141	WHAMMP3	286.8397269	336.88927	236.7901863	-0.507044515	0.145160533	-3.4929916	0.000478	0.001631
ENSG00000168502	MTCL1	919.134166	1079.445	758.8232939	-0.507438133	0.088958838	-5.7041902	1.17E-08	6.85E-08
ENSG00000078237	TIGAR	878.817061	1031.1747	726.4593777	-0.507598025	0.089499042	-5.671547	1.42E-08	8.23E-08
ENSG00000153391	INO80C	510.056017	598.74832	421.3637106	-0.507730017	0.101155838	-5.0192854	5.19E-07	2.62E-06
ENSG00000100478	AP4S1	164.2072484	192.57546	135.8390324	-0.507883874	0.177140657	-2.867122	0.004142	0.011648
ENSG00000054793	ATP9A	3793.683572	4454.7789	3132.588226	-0.508652945	0.048596872	-10.466784	1.23E-25	1.70E-24
ENSG00000111602	TIMELESS	664.3889239	780.75634	548.0215111	-0.508930394	0.091631986	-5.5540692	2.79E-08	1.58E-07
ENSG00000132680	KHDC4	985.5218106	1157.4622	813.5814668	-0.509044627	0.079555024	-6.3986484	1.57E-10	1.07E-09
ENSG00000088367	EPB41L1	1611.093722	1892.5907	1329.596758	-0.509225862	0.062446386	-8.154609	3.50E-16	3.31E-15
ENSG00000184304	PRKD1	679.7945418	798.72753	560.8615543	-0.509353907	0.090352397	-5.6374145	1.73E-08	9.97E-08
ENSG00000076248	UNG	960.0163568	1127.3019	792.7307832	-0.50970416	0.080934308	-6.2977515	3.02E-10	2.02E-09
ENSG00000123815	COQ8B	238.4949649	280.36977	196.6201601	-0.50988579	0.143221606	-3.5601178	0.000371	0.001285
ENSG00000203668	CHML	608.4169259	714.40616	502.427687	-0.510127811	0.096463096	-5.2883209	1.23E-07	6.60E-07
ENSG00000134324	LPIN1	2395.168003	2813.937	1976.399043	-0.510438947	0.053420904	-9.5550414	1.24E-21	1.48E-20
ENSG00000162769	FLVCR1	133.2383302	156.49266	109.9840031	-0.51113159	0.204224418	-2.5027937	0.012322	0.031139
ENSG00000177000	MTHFR	1501.432351	1764.8368	1238.027913	-0.511433257	0.061323473	-8.3399266	7.43E-17	7.24E-16
ENSG00000198837	DENND4B	1315.113311	1546.5035	1083.723084	-0.511469659	0.078396391	-6.524148	6.84E-11	4.80E-10
ENSG00000168096	ANKS3	241.3260279	283.94502	198.7070357	-0.511632348	0.161822534	-3.1616879	0.001569	0.004838
ENSG00000188191	PRKAR1B	1965.616618	2310.6275	1620.605755	-0.511657949	0.053894359	-9.4937199	2.23E-21	2.65E-20
ENSG00000143537	ADAM15	18938.50966	22262.838	15614.18149	-0.511670304	0.029584021	-17.295495	5.09E-67	1.63E-65
ENSG00000157107	FCHO2	1592.179624	1871.9361	1312.423137	-0.511953383	0.063272353	-8.0912651	5.90E-16	5.52E-15
ENSG00000163412	EIF4E3	389.9276934	458.38547	321.4699213	-0.513102243	0.11334493	-4.5269095	5.99E-06	2.69E-05
ENSG00000132436	FIGNL1	496.6355071	583.77484	409.4961733	-0.51310394	0.105151928	-4.8796437	1.06E-06	5.21E-06
ENSG00000160439	RDH13	197.7301959	232.63948	162.8209098	-0.513129851	0.16036279	-3.1998062	0.001375	0.004296
ENSG00000232859	LYRM9	124.4338993	146.20882	102.6589823	-0.513915453	0.205657996	-2.4988839	0.012459	0.031441
ENSG00000136859	ANGPTL2	2687.37669	3161.176	2213.577349	-0.514028503	0.049606214	-10.36218	3.68E-25	5.01E-24
ENSG00000121310	ECHDC2	501.7270898	590.64588	412.808304	-0.514329057	0.125661621	-4.0929685	4.26E-05	0.000171
ENSG00000184271	AC139768.1	255.187612	300.10168	210.2735458	-0.514525855	0.144508522	-3.5605226	0.00037	0.001283
ENSG00000114779	ABHD14B	581.2732705	684.23093	478.3156065	-0.514700752	0.099084301	-5.1945742	2.05E-07	1.07E-06
ENSG00000129197	RPAIN	790.7480499	930.4694	651.0266991	-0.514950106	0.080152688	-6.4246143	1.32E-10	9.08E-10
ENSG00000123473	STIL	374.0545605	440.32935	307.7797759	-0.515156323	0.117425874	-4.3870767	1.15E-05	4.98E-05
ENSG00000151689	INPP1	1250.441364	1471.2223	1029.660423	-0.51530032	0.066447425	-7.7550081	8.83E-15	7.77E-14
ENSG00000148090	AUH	177.0143108	208.17621	145.8524136	-0.515873741	0.17561904	-2.9374591	0.003309	0.009538
ENSG00000138778	CENPE	774.7950955	911.76094	637.8292537	-0.51615684	0.083281677	-6.1977239	5.73E-10	3.75E-09
ENSG00000169683	LRRC45	472.0354934	556.04821	388.0227742	-0.516762117	0.135649238	-3.8095468	0.000139	0.000518
ENSG00000176641	RNF152	887.4897202	1044.2182	730.7612309	-0.516993633	0.086460755	-5.9795179	2.24E-09	1.39E-08
ENSG00000165716	DIPK1B	4830.285712	5687.2723	3973.299096	-0.517458065	0.04345024	-11.909211	1.06E-32	1.82E-31
ENSG00000163743	RCHY1	570.096552	671.15188	469.0412285	-0.51765904	0.096367458	-5.3717204	7.80E-08	4.24E-07
ENSG00000103248	MTHFSD	270.2566097	318.06467	222.4485448	-0.51789691	0.153491166	-3.3741154	0.000741	0.002439
ENSG00000167191	GPRC5B	10922.3976	12865.09	8979.705125	-0.518676522	0.043567235	-11.905197	1.11E-32	1.90E-31
ENSG00000070214	SLC44A1	3438.217359	4049.3516	2827.08313	-0.519332408	0.05097345	-10.188292	2.24E-24	2.94E-23
ENSG00000099949	LZTR1	539.2412122	635.60578	442.8766471	-0.519337118	0.116868524	-4.4437724	8.84E-06	3.89E-05
ENSG00000138413	IDH1	2989.988518	3522.3451	2457.631906	-0.519546195	0.049193789	-10.561215	4.51E-26	6.36E-25
ENSG00000152818	UTRN	24241.82373	28559.857	19923.79026	-0.519630313	0.027164627	-19.128933	1.45E-81	5.51E-80
ENSG00000100490	CDKL1	113.8460886	134.08911	93.60306319	-0.519707175	0.224249709	-2.3175378	0.020474	0.048864
ENSG00000138434	ITPRID2	9242.6805	10888.728	7596.633227	-0.519755248	0.040340197	-12.884301	5.52E-38	1.06E-36
ENSG00000174652	ZNF266	752.358157	886.76007	617.9562409	-0.519860116	0.103173164	-5.0387145	4.69E-07	2.38E-06
ENSG00000143793	C1orf35	677.7946044	799.07606	556.5131447	-0.520371323	0.090206736	-5.7686526	7.99E-09	4.76E-08
ENSG00000126950	TMEM35A	115.5153417	136.03316	94.99752746	-0.520959885	0.224624409	-2.3192488	0.020382	0.048674
ENSG00000177042	TMEM80	383.2507455	451.71761	314.7838814	-0.521614891	0.120255442	-4.3375575	1.44E-05	6.16E-05
ENSG00000105750	ZNF85	135.5983142	159.88907	111.3075589	-0.522797571	0.190357656	-2.7463963	0.006025	0.016324
ENSG00000124006	OBSL1	994.4956541	1173.4778	815.5135115	-0.523290541	0.086807512	-6.0281712	1.66E-09	1.05E-08
ENSG00000130224	LRCH2	316.4899765	373.22769	259.7522642	-0.523643144	0.125590155	-4.1694601	3.05E-05	0.000125
ENSG00000196911	KPNA5	197.3829133	232.67237	162.0934604	-0.523663264	0.159205655	-3.2892253	0.001005	0.003223
ENSG00000198933	TBKBP1	729.9870112	860.62422	599.3498041	-0.523761236	0.099865928	-5.244644	1.57E-07	8.30E-07
ENSG00000106144	CASP2	1063.79461	1254.5375	873.0517307	-0.523888962	0.071056148	-7.3728872	1.67E-13	1.38E-12
ENSG00000197969	VPS13A	2348.317453	2770.0374	1926.59751	-0.523944985	0.058389123	-8.973332	2.88E-19	3.11E-18
ENSG00000164985	PSIP1	1766.185688	2083.6005	1448.770852	-0.524101019	0.06427143	-8.1544945	3.51E-16	3.31E-15
ENSG00000257621	PSMA3-AS1	391.5068112	461.93371	321.0799152	-0.524199038	0.132837947	-3.9461543	7.94E-05	0.000308
ENSG00000162944	RFTN2	225.2778647	265.78803	184.767702	-0.524640142	0.152504661	-3.4401581	0.000581	0.001954
ENSG00000114346	ECT2	842.5172665	994.18892	690.8456161	-0.524898942	0.081207173	-6.4637017	1.02E-10	7.09E-10
ENSG00000185252	ZNF74	351.7619919	415.12549	288.3984893	-0.524987674	0.119540529	-4.3917128	1.12E-05	4.89E-05
ENSG00000166965	RCCD1	236.7070179	279.64573	193.7683023	-0.525116907	0.154792786	-3.3923862	0.000693	0.002297
ENSG00000087586	AURKA	465.5148318	549.24487	381.7847914	-0.525494897	0.11169242	-4.7048394	2.54E-06	1.19E-05
ENSG00000050438	SLC4A8	275.3355726	324.85231	225.8188326	-0.52550591	0.138052612	-3.8065626	0.000141	0.000524
ENSG00000148926	ADM	2366.164166	2793.638	1938.690344	-0.526493904	0.059384006	-8.8659209	7.59E-19	8.08E-18

ENSG00000198612	COPS8	1558.084871	1839.5507	1276.619034	-0.52652528	0.065498941	-8.0386838	9.08E-16	8.42E-15
ENSG00000074582	BCS1L	472.9624467	558.81742	387.1074754	-0.526758765	0.111899172	-4.7074411	2.51E-06	1.18E-05
ENSG00000257122	RRN3P3	287.3599802	339.19503	235.5249291	-0.526976577	0.128172108	-4.1114762	3.93E-05	0.000159
ENSG00000135272	MDFIC	1080.475341	1275.6247	885.3260295	-0.527735521	0.087020389	-6.0645043	1.32E-09	8.40E-09
ENSG00000086200	IPO11	2184.386335	2579.6476	1789.125108	-0.527789333	0.053401577	-9.8834035	4.91E-23	6.16E-22
ENSG00000145335	SNCA	3654.42124	4315.7482	2993.094275	-0.528283614	0.04640192	-11.384952	4.97E-30	7.88E-29
ENSG00000185246	PRPF39	650.2601263	768.33332	532.1869277	-0.528487629	0.103375715	-5.1122997	3.18E-07	1.64E-06
ENSG00000204371	EHMT2	1837.404949	2171.0297	1503.780158	-0.529932047	0.056307459	-9.4114005	4.90E-21	5.74E-20
ENSG00000027075	PRKCH	5216.614463	6164.0542	4269.174684	-0.529974321	0.037620002	-14.087567	4.53E-45	1.01E-43
ENSG00000177374	HIC1	1672.741877	1976.8016	1368.682194	-0.530019136	0.073928476	-7.1693503	7.54E-13	6.00E-12
ENSG00000223509	AC135983.2	163.7253136	193.55902	133.8916104	-0.530101489	0.191356109	-2.7702355	0.005602	0.015302
ENSG00000213281	NRAS	3994.413479	4719.8926	3268.934349	-0.530252985	0.050942657	-10.408821	2.26E-25	3.10E-24
ENSG00000173166	RAPH1	2615.23197	3090.6649	2139.799069	-0.530274486	0.049946212	-10.616911	2.49E-26	3.54E-25
ENSG00000213015	ZNF580	534.2373495	631.6774	436.7973037	-0.53051681	0.112282956	-4.7248205	2.30E-06	1.09E-05
ENSG00000125648	SLC25A23	701.8047108	829.74993	573.8594905	-0.531047335	0.086595176	-6.1325279	8.65E-10	5.58E-09
ENSG00000187741	FANCA	270.8488898	320.33963	221.3581481	-0.531542933	0.137871014	-3.8553639	0.000116	0.000436
ENSG00000127328	RAB3IP	325.0454288	384.4222	265.668656	-0.531924052	0.126839263	-4.1936861	2.74E-05	0.000114
ENSG00000113448	PDE4D	1207.258211	1427.6827	986.8337374	-0.531993426	0.075478333	-7.0482933	1.81E-12	1.41E-11
ENSG00000203880	PCMTD2	984.7611231	1165.119	804.40325	-0.533674715	0.087478019	-6.1006721	1.06E-09	6.76E-09
ENSG00000018408	WWTR1	11540.27961	13651.767	9428.792469	-0.534096601	0.032420045	-16.474271	5.62E-61	1.66E-59
ENSG00000105767	CADM4	191.3018012	226.17169	156.4319081	-0.53410823	0.159831644	-3.3416926	0.000833	0.002711
ENSG00000160447	PKN3	970.6496817	1148.8101	792.4892521	-0.534654577	0.08092196	-6.6070394	3.92E-11	2.80E-10
ENSG00000177034	MTX3	300.8330546	355.94533	245.7207812	-0.535033629	0.127341402	-4.2015685	2.65E-05	0.00011
ENSG00000060140	STYK1	316.6012279	374.82058	258.381879	-0.535199107	0.138122852	-3.8748049	0.000107	0.000404
ENSG00000075711	DLG1	3728.818127	4414.5764	3043.059903	-0.536869208	0.048088369	-11.164222	6.10E-29	9.31E-28
ENSG00000071073	MGAT4A	2132.291121	2523.9792	1740.602998	-0.537037157	0.057090924	-9.4066993	5.12E-21	6.00E-20
ENSG00000136783	NIPSNAP3A	861.8333254	1020.2397	703.4269017	-0.537396513	0.081374619	-6.603982	4.00E-11	2.85E-10
ENSG00000051009	FAM160A2	535.1164628	634.01249	436.2204335	-0.53808835	0.112677989	-4.7754522	1.79E-06	8.56E-06
ENSG00000161281	COX7A1	543.3702858	643.48529	443.2552799	-0.538266349	0.111218513	-4.839719	1.30E-06	6.31E-06
ENSG00000104870	FCGRT	4821.590471	5711.8953	3931.28567	-0.538269261	0.044562661	-12.07893	1.36E-33	2.39E-32
ENSG00000131127	ZNF141	207.6285234	245.83833	169.4187186	-0.538292144	0.152152081	-3.5378559	0.000403	0.001392
ENSG00000198431	TXNRD1	21251.88894	25172.683	17331.09481	-0.538506361	0.081607644	-6.5987245	4.15E-11	2.95E-10
ENSG00000185379	RAD51D	557.5085639	660.12519	454.8919369	-0.539051799	0.105109557	-5.1284756	2.92E-07	1.51E-06
ENSG00000133943	DGLUCY	1412.372193	1673.5188	1151.225598	-0.53906397	0.071096434	-7.582152	3.40E-14	2.91E-13
ENSG00000149503	INCENP	797.2080721	944.52204	649.8941037	-0.539249032	0.0820622	-6.5712232	4.99E-11	3.54E-10
ENSG00000275832	ARHGAP23	5051.752146	5985.0348	4118.469535	-0.539287382	0.03975508	-13.565245	6.44E-42	1.34E-40
ENSG00000085871	MGST2	1623.475627	1923.5981	1323.353124	-0.539347665	0.059684778	-9.0366033	1.62E-19	1.77E-18
ENSG00000166140	ZFYVE19	723.4467968	857.40358	589.4900172	-0.539719952	0.098769432	-5.4644432	4.64E-08	2.57E-07
ENSG00000280789	PAGR1	186.6835344	221.23424	152.1328326	-0.539794359	0.163383934	-3.3038399	0.000954	0.003073
ENSG00000243679	AC018638.5	127.179398	150.82124	103.5375568	-0.539814893	0.22130077	-2.4392816	0.014716	0.036561
ENSG00000139117	CPNE8	873.8881603	1035.739	712.0372937	-0.540210313	0.083275737	-6.4870073	8.76E-11	6.11E-10
ENSG00000106355	LSM5	424.7305032	503.38768	346.0733239	-0.54040386	0.109205532	-4.9485026	7.48E-07	3.73E-06
ENSG00000088808	PPP1R13B	1264.622041	1499.3438	1029.900254	-0.540449503	0.073442886	-7.3587727	1.86E-13	1.53E-12
ENSG00000010361	FUZ	219.2162921	259.91561	178.516978	-0.540492506	0.157935393	-3.422238	0.000621	0.002076
ENSG00000172493	AFF1	5254.568284	6226.9786	4282.157934	-0.540512216	0.040900445	-13.215314	7.16E-40	1.42E-38
ENSG00000276107	AC037198.1	190.6974637	226.19831	155.1966188	-0.54052177	0.180504913	-2.9944989	0.002749	0.008053
ENSG00000096093	EFHC1	397.8184329	471.64929	323.9875769	-0.541023254	0.122870376	-4.4032034	1.07E-05	4.65E-05
ENSG00000107554	DNMBP	4500.566368	5334.8752	3666.257508	-0.541759836	0.045148043	-11.99963	3.57E-33	6.18E-32
ENSG00000168899	VAMP5	2837.347746	3364.4588	2310.236681	-0.541852759	0.053102308	-10.20394	1.90E-24	2.52E-23
ENSG00000101224	CDC25B	2742.133769	3251.5272	2232.740365	-0.541868317	0.047414175	-11.428403	3.02E-30	4.80E-29
ENSG00000145439	CBR4	604.9694201	717.39143	492.5474071	-0.542391436	0.094439265	-5.7432831	9.29E-09	5.49E-08
ENSG00000159733	ZFYVE28	406.633699	482.23017	331.0372298	-0.542705647	0.124804671	-4.3484402	1.37E-05	5.88E-05
ENSG00000121104	FAM117A	708.5279035	840.51316	576.5426494	-0.543039975	0.087991072	-6.1715349	6.76E-10	4.40E-09
ENSG00000102125	TAZ	523.3925825	621.157	425.6281624	-0.543974017	0.110769932	-4.9108455	9.07E-07	4.48E-06
ENSG00000065457	ADAT1	966.7523658	1146.6286	786.8761094	-0.544603381	0.077341291	-7.0415605	1.90E-12	1.48E-11
ENSG00000117569	PTBP2	187.2465912	222.27314	152.2200431	-0.544952129	0.182095015	-2.9926801	0.002765	0.008099
ENSG00000170264	FAM161A	97.82155221	116.07659	79.56650996	-0.544958715	0.225877306	-2.4126315	0.015838	0.039022
ENSG00000118707	TGIF2	573.874303	680.71489	467.0337154	-0.545234077	0.095893671	-5.6858192	1.30E-08	7.59E-08
ENSG00000136492	BRIP1	181.2337282	215.29456	147.1728937	-0.54560132	0.166373466	-3.279377	0.00104	0.003331
ENSG00000119929	CUTC	267.244569	317.24959	217.239549	-0.545864599	0.137323603	-3.9750239	7.04E-05	0.000275
ENSG00000198089	SFI1	447.460689	531.43682	363.4845581	-0.545883763	0.132226885	-4.1283871	3.65E-05	0.000149
ENSG00000225383	SFTA1P	96.04961382	113.99282	78.10640954	-0.545984203	0.224379113	-2.4333112	0.014961	0.037119
ENSG00000122592	HOXA7	419.4315362	497.99553	340.8675453	-0.546464846	0.112832865	-4.8431354	1.28E-06	6.21E-06
ENSG00000139133	ALG10	111.7062066	132.66642	90.7459937	-0.547389031	0.22733172	-2.4078867	0.016045	0.039453
ENSG00000153774	CFDP1	686.4087426	814.91776	557.8997257	-0.54795134	0.091514188	-5.9876108	2.13E-09	1.33E-08
ENSG00000107957	SH3PXD2A	1300.844122	1545.2047	1056.483565	-0.548291286	0.068370768	-8.0193817	1.06E-15	9.82E-15
ENSG00000024422	EHD2	16841.94684	20005.845	13678.04893	-0.548503757	0.030683026	-17.876456	1.80E-71	6.13E-70
ENSG00000004139	SARM1	422.6747175	502.07356	343.2758704	-0.54852905	0.122130535	-4.4913342	7.08E-06	3.16E-05
ENSG00000166971	AKTIP	1108.141125	1316.0065	900.2757556	-0.548704077	0.075508556	-7.2667802	3.68E-13	2.99E-12
ENSG00000161267	BDH1	134.392379	159.63404	109.1507133	-0.549309474	0.205777719	-2.6694313	0.007598	0.020177
ENSG00000012963	UBR7	774.3814529	920.33472	628.4281849	-0.550119722	0.095617433	-5.7533412	8.75E-09	5.19E-08
ENSG00000008283	CYB561	1976.152809	2349.0046	1603.301062	-0.550453043	0.055396329	-9.936634	2.88E-23	3.66E-22
ENSG00000162032	SPSB3	930.5530456	1106.701	754.4051163	-0.550761623	0.087192776	-6.316597	2.67E-10	1.80E-09
ENSG00000184445	KNTC1	591.3227237	703.04664	479.5988051	-0.551101701	0.103000154	-5.350494	8.77E-08	4.75E-07
ENSG00000079308	TNS1	3651.504603	4340.8688	2962.140435	-0.551276933	0.049839234	-11.061104	1.94E-28	2.91E-27
ENSG00000175556	LONRF3	245.5133793	291.76633	199.2604331	-0.551476662	0.147200374	-3.7464352	0.000179	0.000655
ENSG00000135164	DMTF1	811.5752925	965.22042	657.9301658	-0.551497022	0.109649794	-5.029622	4.91E-07	2.49E-06
ENSG00000163918	RFC4	185.9763431	221.33318	150.6195038	-0.55166272	0.170304865	-3.2392658	0.001198	0.003789
ENSG00000065057	NTHL1	304.440566	361.7741	247.1070288	-0.551794532	0.127228955	-4.3370201	1.44E-05	6.17E-05
ENSG00000078596	ITM2A	1052.740429	1251.8204	853.6604705	-0.551800363	0.084406009	-6.5374536	6.26E-11	4.41E-10
ENSG00000141858	SAMD1	701.8206803	834.57926	569.062099	-0.552398793	0.091490896	-6.037746	1.56E-09	9.88E-09
ENSG00000085276	MECOM	2646.171309	3147.4387	2144.903922	-0.552662233	0.050340627	-10.978454	4.85E-28	7.19E-27
ENSG00000111145	ELK3	4904.967466	5833.9119	3976.023043	-0.553103474	0.039438234	-14.02455	1.10E-44	2.44E-43
ENSG00000119139	TJP2	117.0312134	139.18393	94.87849616	-0.553131378	0.217745867	-2.5402612	0.011077	0.02827
ENSG00000171700	RGS19	609.4385353	725.50326	493.3738091	-0.554077986	0.100691334	-5.5027376	3.74E-08	2.10E-07

ENSG00000137414	FAM8A1	1080.322406	1284.8789	875.7658919	-0.55423155	0.074106861	-7.4788156	7.50E-14	6.35E-13
ENSG00000112992	NNT	3470.549321	4128.8229	2812.275765	-0.554370924	0.044837277	-12.364063	4.09E-35	7.41E-34
ENSG00000137145	DENND4C	3381.90318	4023.4395	2740.366909	-0.554667194	0.047942112	-11.569519	5.88E-31	9.66E-30
ENSG00000240602	AADACP1	262.2934355	312.22296	212.3639115	-0.554782752	0.140749894	-3.9416211	8.09E-05	0.000313
ENSG00000173894	CBX2	797.4094588	948.91494	645.9039753	-0.555428666	0.088667083	-6.2642037	3.75E-10	2.49E-09
ENSG00000182919	C11orf54	191.1942708	227.5148	154.8737444	-0.55652645	0.18655364	-2.983198	0.002853	0.008323
ENSG00000189362	NEMP2	140.8115114	167.61459	114.0084332	-0.556633006	0.213155021	-2.6113999	0.009017	0.023496
ENSG00000161714	PLCD3	862.3544585	1027.0292	697.6797053	-0.557234199	0.083648043	-6.6616525	2.71E-11	1.95E-10
ENSG00000129675	ARHGEF6	501.0086848	596.45767	405.5597014	-0.558072574	0.111021139	-5.0267235	4.99E-07	2.53E-06
ENSG00000003756	RBM5	2501.17286	2979.3238	2023.021963	-0.55819816	0.131888348	-4.2323539	2.31E-05	9.67E-05
ENSG00000135074	ADAM19	4697.842321	5595.5233	3800.161361	-0.558236588	0.050564331	-11.040126	2.45E-28	3.66E-27
ENSG00000101773	RBBP8	405.2673943	483.05424	327.4805529	-0.558259694	0.11839566	-4.715204	2.41E-06	1.13E-05
ENSG00000144827	ABHD10	501.6410161	597.23957	406.0424632	-0.558350726	0.106907657	-5.2227384	1.76E-07	9.31E-07
ENSG00000132881	CPLANE2	130.1677507	155.02864	105.3068657	-0.558722701	0.199316135	-2.8031986	0.00506	0.013964
ENSG00000130304	SLC27A1	477.2917832	568.89926	385.6843014	-0.559504078	0.102283676	-5.4701209	4.50E-08	2.50E-07
ENSG00000186153	WWOX	155.7689556	185.47903	126.058881	-0.559556839	0.196462614	-2.8481594	0.004397	0.012276
ENSG00000183496	MEX3B	823.854542	981.43894	666.2701401	-0.559900214	0.084521589	-6.6243456	3.49E-11	2.50E-10
ENSG00000002016	RAD52	162.6388806	193.77182	131.5059394	-0.560037662	0.176115907	-3.179938	0.001473	0.004571
ENSG00000149218	ENDOD1	1795.088107	2140.0965	1450.079707	-0.562089581	0.059508167	-9.4455873	3.53E-21	4.17E-20
ENSG00000173267	SNCG	913.9082658	1089.8207	737.9958102	-0.562163257	0.085664102	-6.5624135	5.29E-11	3.75E-10
ENSG00000160094	ZNF362	905.5163067	1079.946	731.0866569	-0.562249472	0.078149382	-7.1945479	6.27E-13	5.02E-12
ENSG00000130695	CEP85	398.9221968	475.73265	322.1117404	-0.562456623	0.115493354	-4.8700345	1.12E-06	5.46E-06
ENSG00000133119	RFC3	180.6569331	215.21314	146.1007227	-0.562522036	0.172389781	-3.2630823	0.001102	0.003508
ENSG00000105655	ISYNA1	1173.536683	1399.694	947.3793945	-0.562534509	0.073256395	-7.6789816	1.60E-14	1.40E-13
ENSG00000126106	TMEM53	145.4395393	173.47891	117.400164	-0.56285817	0.196464069	-2.864942	0.004171	0.011719
ENSG00000173207	CKS1B	340.5063292	406.37594	274.636723	-0.563041961	0.125917479	-4.4715155	7.77E-06	3.45E-05
ENSG00000143624	INTS3	2457.93444	2932.7356	1983.13323	-0.563425879	0.055526152	-10.147036	3.42E-24	4.45E-23
ENSG00000175745	NR2F1	372.4785942	444.52499	300.4321955	-0.563788837	0.116883125	-4.8235264	1.41E-06	6.81E-06
ENSG00000197147	LRRC8B	1498.638233	1788.1373	1209.139136	-0.564362828	0.061891966	-9.1185151	7.62E-20	8.46E-19
ENSG00000004534	RBM6	1644.861814	1962.8083	1326.91531	-0.564520865	0.150968464	-3.7393297	0.000185	0.000673
ENSG00000083750	RRAGB	526.5315164	628.18287	424.8801594	-0.564545234	0.101829022	-5.5440505	2.96E-08	1.67E-07
ENSG00000100151	PICK1	307.6203661	366.9652	248.2755299	-0.564845709	0.137158406	-4.1181997	3.82E-05	0.000155
ENSG00000184508	HDDC3	306.0161103	365.38011	246.6521091	-0.565160111	0.153717373	-3.6766183	0.000236	0.000848
ENSG00000172175	MALT1	452.4584799	539.86736	365.0495958	-0.565173303	0.105865808	-5.3385821	9.37E-08	5.06E-07
ENSG00000268575	AL031282.2	92.8546669	110.76971	74.93962121	-0.565631046	0.230361766	-2.4554033	0.014073	0.03513
ENSG00000105483	CARD8	2975.020796	3551.9005	2398.141078	-0.566337246	0.049547993	-11.430074	2.96E-30	4.72E-29
ENSG00000137831	UACA	12794.06732	15273.433	10314.70159	-0.566341934	0.040469922	-13.994145	1.69E-44	3.72E-43
ENSG00000187824	TMEM220	118.0511919	140.9326	95.16978717	-0.566593776	0.198208177	-2.8585792	0.004255	0.011929
ENSG00000160789	LMNA	19523.26114	23316.254	15730.26839	-0.567804809	0.036555003	-15.532889	2.08E-54	5.50E-53
ENSG00000121691	CAT	1290.94118	1542.3559	1039.526478	-0.568194664	0.065123431	-8.7248884	2.66E-18	2.78E-17
ENSG00000131080	EDA2R	177.1853525	211.62451	142.7461912	-0.56921578	0.170003473	-3.3482597	0.000813	0.002655
ENSG00000186638	KIF24	98.80980221	118.20155	79.4180565	-0.569270701	0.232862758	-2.4446619	0.014499	0.036089
ENSG00000115159	GPD2	941.7801682	1124.7476	758.8127745	-0.569516684	0.079882274	-7.1294501	1.01E-12	7.96E-12
ENSG00000147421	HMBOX1	719.227387	859.8036	578.6511701	-0.569638052	0.093765436	-6.0751389	1.24E-09	7.89E-09
ENSG00000187531	SIRT7	472.9231842	565.28788	380.5584904	-0.569831874	0.138062391	-4.1273505	3.67E-05	0.000149
ENSG00000168918	INPP5D	1763.796751	2107.9696	1419.623923	-0.570173069	0.059504949	-9.5819436	9.52E-22	1.15E-20
ENSG00000115457	IGFBP2	1509.744563	1804.6688	1214.820328	-0.570474866	0.060851024	-9.3749427	6.92E-21	8.06E-20
ENSG00000176700	SCAND2P	104.5294091	125.05303	84.00578689	-0.571156602	0.223990235	-2.5499174	0.010775	0.027582
ENSG00000134574	DDB2	1678.865922	2008.1018	1349.630004	-0.572784792	0.058798283	-9.7415224	2.01E-22	2.47E-21
ENSG00000101574	METTL4	287.0010525	343.15506	230.8470438	-0.573318334	0.133256502	-4.3023667	1.69E-05	7.17E-05
ENSG00000068137	PLEKHH3	255.2830694	305.52509	205.041052	-0.574108977	0.153221895	-3.7469121	0.000179	0.000655
ENSG00000130303	BST2	595.1919532	712.25786	478.1260457	-0.574201631	0.2447298	-2.3462677	0.018962	0.045711
ENSG00000233251	AC007743.1	592.549042	709.17715	475.9209357	-0.574718816	0.094177761	-6.1024897	1.04E-09	6.69E-09
ENSG00000119630	PGF	17141.7468	20519.858	13763.63592	-0.576041368	0.038285736	-15.045848	3.68E-51	9.14E-50
ENSG00000178896	EXOSC4	249.3253882	298.20523	200.4455445	-0.576875374	0.148324207	-3.8892868	0.000101	0.000383
ENSG00000165512	ZNF22	1440.017717	1724.6002	1155.43528	-0.578033265	0.064266106	-8.994372	2.38E-19	2.58E-18
ENSG00000125814	NAPB	207.8821503	248.99996	166.7643402	-0.578148624	0.171473667	-3.3716467	0.000747	0.002456
ENSG00000168887	C2orf68	540.7085276	647.84348	433.5735708	-0.578969204	0.096145978	-6.0217725	1.73E-09	1.09E-08
ENSG00000117155	SSX2IP	161.7221269	193.44901	129.9952468	-0.579084507	0.203940106	-2.8394832	0.004519	0.01258
ENSG00000180884	ZNF792	612.0664093	733.64225	490.4905701	-0.579125668	0.103496581	-5.5956019	2.20E-08	1.26E-07
ENSG00000115380	EFEMP1	127842.5207	153176.08	102508.961	-0.579422108	0.029562765	-19.599727	1.55E-85	6.16E-84
ENSG00000182287	AP1S2	1703.596066	2041.3473	1365.844811	-0.579663256	0.072696408	-7.9737537	1.54E-15	1.41E-14
ENSG00000228495	LINC01013	3484.627288	4175.982	2793.272624	-0.579837684	0.054699043	-10.600509	2.96E-26	4.21E-25
ENSG00000170390	DCLK2	105.7647124	126.86524	84.66418085	-0.580665699	0.216477895	-2.6823325	0.007311	0.019489
ENSG00000105971	CAV2	9926.07009	11900.222	7951.918366	-0.581903442	0.044485873	-13.080634	4.25E-39	8.32E-38
ENSG00000130005	GAMT	187.3501344	224.46924	150.2310286	-0.582020121	0.168410789	-3.4559551	0.000548	0.00185
ENSG00000124641	MED20	734.5077561	880.98975	588.0257611	-0.582500045	0.085736929	-6.7940391	1.09E-11	8.08E-11
ENSG00000011523	CEP68	1706.313494	2046.3322	1366.294766	-0.583524894	0.062680463	-9.3095179	1.28E-20	1.48E-19
ENSG00000076067	RBMS2	4838.29376	5803.8332	3872.754289	-0.583867154	0.038306523	-15.241977	1.86E-52	4.73E-51
ENSG00000165424	ZCCHC24	509.3643946	610.94288	407.7859108	-0.584712613	0.110386238	-5.2969702	1.18E-07	6.30E-07
ENSG00000183426	NPIPA1	147.4929656	176.90969	118.076238	-0.58527776	0.218718313	-2.6759431	0.007452	0.019812
ENSG00000130830	MPP1	1438.621922	1726.7979	1150.445905	-0.586106201	0.065724661	-8.9175995	4.77E-19	5.11E-18
ENSG00000099849	RASSF7	408.607945	490.45047	326.7654152	-0.586237707	0.121792059	-4.8134313	1.48E-06	7.15E-06
ENSG00000137343	ATAT1	287.0732862	344.82009	229.3264836	-0.586340332	0.142699289	-4.1089226	3.98E-05	0.000161
ENSG00000185324	CDK10	1065.313517	1279.4367	851.1902845	-0.586374358	0.093108415	-6.297759	3.02E-10	2.02E-09
ENSG00000101439	CST3	5162.399693	6198.2379	4126.561463	-0.586745114	0.040778633	-14.388543	6.11E-47	1.41E-45
ENSG00000152782	PANK1	122.1696337	146.53938	97.79988437	-0.586782081	0.215208288	-2.7265775	0.006399	0.017269
ENSG00000136169	SETDB2	345.5847929	414.81093	276.3586569	-0.586955095	0.119434381	-4.9144567	8.90E-07	4.40E-06
ENSG00000132256	TRIM5	1673.520175	2009.0498	1337.990556	-0.587278496	0.058831119	-9.9824465	1.82E-23	2.32E-22
ENSG00000166037	CEP57	957.8727745	1150.429	765.316581	-0.587302922	0.076605119	-7.6666276	1.77E-14	1.53E-13
ENSG00000113555	PCDH12	3495.167928	4196.913	2793.422895	-0.587367642	0.119275159	-4.9244759	8.46E-07	4.19E-06
ENSG00000176225	RTTN	279.2117468	335.46193	222.961561	-0.588145138	0.141473436	-4.1572832	3.22E-05	0.000132
ENSG00000248441	LINC01197	406.1723401	488.09523	324.2494458	-0.588485897	0.117950464	-4.9892631	6.06E-07	3.04E-06
ENSG00000185946	RNPC3	207.9059908	249.64455	166.1674267	-0.588502927	0.159248402	-3.6955029	0.000219	0.000791
ENSG00000136824	SMC2	1089.5611	1309.1713	869.9508514	-0.589307628	0.071088468	-8.2897781	1.13E-16	1.10E-15

ENSG00000107819	SFXN3	3436.379895	4129.1125	2743.647328	-0.589998085	0.046496858	-12.688988	6.81E-37	1.28E-35
ENSG00000179818	PCBP1-AS1	147.6216366	177.43035	117.8129204	-0.590531767	0.191902782	-3.0772444	0.002089	0.006277
ENSG00000143815	LBR	1782.392551	2141.9934	1422.791697	-0.59057268	0.060709157	-9.7279012	2.29E-22	2.82E-21
ENSG00000153904	DDAH1	2087.274048	2508.2762	1666.271901	-0.590589066	0.055220413	-10.695122	1.07E-26	1.54E-25
ENSG00000205356	TECPR1	2919.052632	3508.5748	2329.530427	-0.590608415	0.055473983	-10.646584	1.81E-26	2.57E-25
ENSG00000102760	RGCC	1262.064511	1517.1725	1006.956532	-0.59148396	0.090362336	-6.5456914	5.92E-11	4.18E-10
ENSG00000163462	TRIM46	251.1473768	302.11159	200.1831653	-0.591747725	0.143122728	-4.1345475	3.56E-05	0.000145
ENSG00000171843	MLLT3	163.8409118	196.95311	130.7287163	-0.592339747	0.182211408	-3.2508379	0.001151	0.003652
ENSG00000007392	LUC7L	1072.559004	1290.1678	854.9501743	-0.593175413	0.203719514	-2.911726	0.003594	0.010266
ENSG00000123700	KCNJ2	657.650566	790.66325	524.637879	-0.593416343	0.104719543	-5.6667201	1.46E-08	8.45E-08
ENSG00000148339	SLC25A25	825.2987944	993.08913	657.5084564	-0.593475822	0.08069074	-7.3549433	1.91E-13	1.58E-12
ENSG00000111731	C2CD5	830.4035803	999.40194	661.4052242	-0.59417712	0.082816235	-7.1746454	7.25E-13	5.78E-12
ENSG00000128159	TUBGCP6	1415.103448	1702.7753	1127.431596	-0.594371704	0.082315984	-7.2206111	5.18E-13	4.17E-12
ENSG00000115286	NDUFS7	1172.581182	1411.0346	934.127724	-0.594955938	0.068461783	-8.6903366	3.61E-18	3.75E-17
ENSG00000136237	RAPGEF5	2840.146322	3418.2043	2262.088359	-0.59549596	0.052604768	-11.32019	1.04E-29	1.64E-28
ENSG00000104205	SGK3	420.5871121	506.40578	334.7684451	-0.595752767	0.113520676	-5.247967	1.54E-07	8.16E-07
ENSG00000136108	CKAP2	1399.825431	1685.3831	1114.26774	-0.596557736	0.066692017	-8.9449647	3.72E-19	4.01E-18
ENSG00000101096	NFATC2	1186.944843	1428.9106	944.9790964	-0.597512004	0.072145461	-8.2820457	1.21E-16	1.17E-15
ENSG00000138772	ANXA3	3596.476682	4330.9059	2862.047507	-0.597653381	0.042126525	-14.187104	1.10E-45	2.49E-44
ENSG00000145819	ARHGAP26	1517.674823	1827.8988	1207.450865	-0.5976727	0.066909255	-8.9325864	4.16E-19	4.47E-18
ENSG00000187546	AGMO	91.61923433	110.25675	72.98171517	-0.598284161	0.238261366	-2.5110414	0.012038	0.030476
ENSG00000112367	FIG4	634.3799735	763.83989	504.9200522	-0.598464842	0.090359409	-6.6231602	3.52E-11	2.52E-10
ENSG00000237036	ZEB1-AS1	122.3531682	147.28539	97.42094332	-0.598504556	0.232736237	-2.5716002	0.010123	0.026053
ENSG00000173581	CCDC106	584.1499592	703.70027	464.5996489	-0.598776125	0.097738818	-6.1262877	9.00E-10	5.79E-09
ENSG00000197774	EME2	404.3363275	487.63007	321.0425846	-0.599914867	0.148034465	-4.0525351	5.07E-05	0.000202
ENSG00000072135	PTPN18	1180.515038	1422.9506	938.0794533	-0.600323924	0.070900122	-8.4671776	2.51E-17	2.51E-16
ENSG00000080839	RBL1	266.4741012	320.99177	211.9564275	-0.600402196	0.145726579	-4.1200596	3.79E-05	0.000154
ENSG00000122952	ZWINT	452.1706643	545.48037	358.8609573	-0.602109301	0.115560106	-5.2103561	1.88E-07	9.92E-07
ENSG00000047579	DTNBP1	698.2891312	842.29895	554.2793112	-0.602254719	0.095957088	-6.2762921	3.47E-10	2.31E-09
ENSG00000009413	REV3L	3083.218191	3718.5751	2447.861326	-0.603341414	0.054846952	-11.000455	3.80E-28	5.65E-27
ENSG00000106100	NOD1	1233.918527	1488.4934	979.3436414	-0.60381232	0.076088625	-7.9356451	2.09E-15	1.91E-14
ENSG00000109685	NSD2	2998.707908	3617.5545	2379.861277	-0.604062079	0.04781402	-12.633577	1.38E-36	2.58E-35
ENSG00000104177	MYEF2	1446.263377	1744.9155	1147.611217	-0.604704225	0.067917555	-8.903504	5.41E-19	5.79E-18
ENSG00000185219	ZNF445	806.9386926	974.00691	639.8704759	-0.605252762	0.089424987	-6.7682734	1.30E-11	9.62E-11
ENSG00000080561	MID2	1094.317237	1320.3569	868.2775405	-0.605669444	0.081221333	-7.4570242	8.85E-14	7.45E-13
ENSG00000152518	ZFP36L2	3128.870184	3776.8906	2480.849738	-0.606691129	0.056721946	-10.69588	1.06E-26	1.53E-25
ENSG00000022556	NLRP2	86.45858213	104.25813	68.65903548	-0.606864329	0.248006916	-2.4469653	0.014406	0.035893
ENSG00000132196	HSD17B7	217.0563625	261.82632	172.2864035	-0.607037559	0.154491982	-3.9292496	8.52E-05	0.000328
ENSG00000104365	IKBKB	1565.122233	1889.6682	1240.576238	-0.60709999	0.060174985	-10.08891	6.19E-24	8.00E-23
ENSG00000186994	KANK3	1694.101774	2045.8998	1342.3037	-0.607677421	0.075925081	-8.0036454	1.21E-15	1.11E-14
ENSG00000080546	SESN1	1158.676835	1399.388	917.9657178	-0.608871467	0.070853577	-8.5933765	8.45E-18	8.60E-17
ENSG00000280304	AC104260.2	174.5663087	210.77467	138.3579461	-0.609005681	0.185908647	-3.275833	0.001054	0.003368
ENSG00000251022	THAP9-AS1	118.9989579	143.83926	94.1586571	-0.609190028	0.225446474	-2.7021493	0.006889	0.018459
ENSG00000119969	HELLS	216.7729194	262.05038	171.4954539	-0.609330441	0.15458449	-3.9417308	8.09E-05	0.000313
ENSG00000163686	ABHD6	391.0588628	472.13208	309.9856474	-0.609510293	0.121737174	-5.0067722	5.54E-07	2.79E-06
ENSG00000135046	ANXA1	23704.37703	28639.028	18769.72599	-0.609672361	0.030860405	-19.755812	7.15E-87	2.87E-85
ENSG00000188153	COL4A5	2689.500235	3250.0884	2128.912028	-0.610163864	0.052828451	-11.54991	7.39E-31	1.21E-29
ENSG00000150593	PDCD4	2720.163251	3286.1258	2154.200732	-0.61025209	0.054334931	-11.231303	2.86E-29	4.43E-28
ENSG00000187079	TEAD1	1730.969726	2091.5853	1370.354188	-0.610396492	0.066872787	-9.1277263	7.00E-20	7.80E-19
ENSG00000197872	FAM49A	2179.841293	2634.41	1725.272584	-0.611259028	0.060941912	-10.030191	1.12E-23	1.44E-22
ENSG00000141295	SCRN2	621.2960967	750.94757	491.6446222	-0.611351676	0.095385324	-6.4092845	1.46E-10	1.00E-09
ENSG00000138685	FGF2	1668.886356	2016.9709	1320.801819	-0.611665149	0.065464881	-9.3434088	9.33E-21	1.08E-19
ENSG00000154783	FGD5	9990.295797	12078.783	7901.808999	-0.612012145	0.032638015	-18.751512	1.88E-78	6.97E-77
ENSG00000169750	RAC3	543.6448515	657.45572	429.8339835	-0.61202368	0.111230363	-5.5023077	3.75E-08	2.10E-07
ENSG00000136144	RCBTB1	1878.379567	2270.9327	1485.826439	-0.612053987	0.054747833	-11.179511	5.14E-29	7.87E-28
ENSG00000197971	MBP	518.2943627	626.42794	410.1607895	-0.612318469	0.104839726	-5.8405195	5.20E-09	3.15E-08
ENSG00000166439	RNF169	1522.400415	1840.6056	1204.19518	-0.61264688	0.062023046	-9.8777296	5.20E-23	6.51E-22
ENSG00000112029	FBXO5	178.4339082	215.69761	141.170206	-0.613339649	0.173165239	-3.5419329	0.000397	0.001371
ENSG00000116001	TIA1	1557.322075	1883.4697	1231.174456	-0.613433467	0.078213104	-7.8431035	4.40E-15	3.94E-14
ENSG00000178917	ZNF852	108.1259274	130.7675	85.48435052	-0.61432074	0.209403647	-2.9336678	0.00335	0.00964
ENSG00000135966	TGFBRAP1	3424.609208	4143.3947	2705.823762	-0.614419853	0.049673445	-12.369181	3.84E-35	6.97E-34
ENSG00000108848	LUC7L3	1836.449874	2222.3478	1450.551962	-0.614857102	0.078851311	-7.7976777	6.31E-15	5.60E-14
ENSG00000108389	MTMR4	1432.252784	1732.8307	1131.674879	-0.614896094	0.065189615	-9.4324241	4.01E-21	4.72E-20
ENSG00000138080	EMILIN1	138.4583618	167.44533	109.4713911	-0.616574156	0.197508953	-3.1217529	0.001798	0.005476
ENSG00000128656	CHN1	737.1273665	892.63911	581.6156247	-0.618523638	0.088161647	-7.0157904	2.29E-12	1.76E-11
ENSG00000106479	ZNF862	504.2514032	610.88462	397.6181873	-0.619440262	0.10748339	-5.7631255	8.26E-09	4.91E-08
ENSG00000140853	NLRC5	1613.96373	1955.7267	1272.20076	-0.619685204	0.074889453	-8.274666	1.29E-16	1.24E-15
ENSG00000113108	APBB3	90.6734972	109.78088	71.56611616	-0.620001009	0.243750702	-2.5435866	0.010972	0.028032
ENSG00000172613	RAD9A	228.0713906	276.34012	179.8026575	-0.620241867	0.148017766	-4.1903204	2.79E-05	0.000115
ENSG00000223496	EXOSC6	869.3374319	1053.9945	684.6803443	-0.621147871	0.086012241	-7.2216218	5.14E-13	4.14E-12
ENSG00000221829	FANCG	368.2566575	446.36273	290.1505817	-0.621200723	0.135435138	-4.5867028	4.50E-06	2.06E-05
ENSG00000158691	ZSCAN12	102.9144625	124.85212	80.97680129	-0.621205264	0.219442258	-2.830837	0.004643	0.012899
ENSG00000119711	ALDH6A1	416.5780553	504.84785	328.3082622	-0.621442151	0.108585046	-5.7230915	1.05E-08	6.16E-08
ENSG00000165359	INTS6L	111.1461822	134.80016	87.49210103	-0.621845908	0.267032147	-2.3287305	0.019873	0.047632
ENSG00000172037	LAMB2	24800.63599	30070.08	19531.19173	-0.622494695	0.036192903	-17.199358	2.68E-66	8.51E-65
ENSG00000159658	EFCAB14	6644.759671	8057.2623	5232.25707	-0.623076998	0.054915603	-11.346083	7.76E-30	1.22E-28
ENSG00000102384	CENPI	185.9222897	225.50014	146.3444366	-0.62333158	0.160152102	-3.8921224	9.94E-05	0.000379
ENSG00000128645	HOXD1	668.6581012	810.75031	526.5658879	-0.623604187	0.098544423	-6.328153	2.48E-10	1.67E-09
ENSG00000130517	PGPEP1	2058.559743	2497.1432	1619.976333	-0.624421276	0.06034372	-10.347742	4.28E-25	5.80E-24
ENSG00000165948	IFI27L1	314.3758026	381.30885	247.4427504	-0.624679833	0.125739453	-4.9680496	6.76E-07	3.39E-06
ENSG00000107960	STN1	666.0267413	807.95321	524.1002734	-0.6248648	0.087256137	-7.1612705	7.99E-13	6.35E-12
ENSG00000122483	CCDC18	193.1146808	234.52724	151.7021234	-0.625040088	0.173090577	-3.6110579	0.000305	0.001072
ENSG00000177084	POLE	1123.462948	1363.562	883.3639072	-0.625308395	0.070757373	-8.8373603	9.80E-19	1.04E-17
ENSG00000215440	NPEPL1	159.2118118	193.11133	125.3122913	-0.62542756	0.178296807	-3.5077889	0.000452	0.001549
ENSG00000079335	CDC14A	470.3389362	570.76989	369.9079813	-0.625572616	0.116440797	-5.3724522	7.77E-08	4.23E-07

ENSG00000121057	AKAP1	926.7456927	1124.1835	729.3079338	-0.625613336	0.077918735	-8.0290489	9.82E-16	9.09E-15
ENSG00000131149	GSE1	510.9785546	620.23828	401.7188336	-0.625728566	0.099378598	-6.2964117	3.05E-10	2.04E-09
ENSG00000162174	ASRGL1	747.3746814	907.4106	587.3387636	-0.626106601	0.084085173	-7.4460999	9.61E-14	8.07E-13
ENSG00000198130	HIBCH	429.1482085	520.92408	337.3723374	-0.626310779	0.118717525	-5.2756388	1.32E-07	7.05E-07
ENSG00000137411	VARS2	548.3817062	665.80871	430.9547054	-0.626492585	0.106456029	-5.8849892	3.98E-09	2.43E-08
ENSG00000170100	ZNF778	285.541582	346.79578	224.2873842	-0.626578841	0.138914316	-4.5105419	6.47E-06	2.90E-05
ENSG00000107798	LIPA	3260.864169	3958.3785	2563.349818	-0.626942135	0.05453274	-11.496619	1.37E-30	2.23E-29
ENSG00000185684	EP400P1	215.4252416	261.60379	169.2466964	-0.626960384	0.154894555	-4.0476593	5.17E-05	0.000206
ENSG00000125864	BFSP1	201.2326095	244.17118	158.2940434	-0.627879262	0.179633506	-3.4953349	0.000473	0.001618
ENSG00000132970	WASF3	1017.315791	1235.553	799.07856	-0.628167307	0.08121109	-7.7349942	1.03E-14	9.08E-14
ENSG00000145147	SLIT2	77.90172856	94.641394	61.16206321	-0.629045198	0.259856899	-2.420737	0.015489	0.038266
ENSG00000144791	LIMD1	1944.760853	2362.4887	1527.033017	-0.629428866	0.054343195	-11.582478	5.06E-31	8.32E-30
ENSG00000275023	MLLT6	2594.445422	3151.7742	2037.116614	-0.629549318	0.05219149	-12.062298	1.67E-33	2.92E-32
ENSG00000241717	VWFP1	611.1216087	742.29973	479.9434913	-0.629550506	0.098463496	-6.3937452	1.62E-10	1.10E-09
ENSG00000137504	CREBZF	462.6808918	562.08205	363.2797297	-0.629552283	0.25795743	-2.4405278	0.014666	0.036455
ENSG00000125871	MGME1	640.1968448	777.56987	502.8238194	-0.62955676	0.104399089	-6.0302898	1.64E-09	1.03E-08
ENSG00000153707	PTPRD	1148.836006	1395.8331	901.8388697	-0.63004903	0.069927439	-9.0100401	2.06E-19	2.25E-18
ENSG00000100605	ITPK1	2060.705246	2503.5568	1617.853717	-0.630176887	0.05351364	-11.776005	5.19E-32	8.77E-31
ENSG00000260804	LINC01963	286.9731968	348.94211	225.0042789	-0.631608922	0.132797479	-4.7561816	1.97E-06	9.38E-06
ENSG00000164087	POC1A	174.6818683	212.41081	136.9529271	-0.631811876	0.18097979	-3.4910632	0.000481	0.00164
ENSG00000149929	HIRIP3	205.3985217	249.70622	161.0908198	-0.632131792	0.16825479	-3.7569914	0.000172	0.00063
ENSG00000189319	FAM53B	824.9176094	1002.5147	647.3204773	-0.632311887	0.086682582	-7.2945668	3.00E-13	2.44E-12
ENSG00000181031	RPH3AL	178.6154491	217.19718	140.0337163	-0.633284745	0.202530856	-3.1268556	0.001767	0.005396
ENSG00000104522	TSTA3	2640.288931	3211.5339	2069.044003	-0.633288381	0.053359803	-11.868267	1.73E-32	2.94E-31
ENSG00000188211	NCR3LG1	404.8278696	492.25948	317.3962632	-0.633744934	0.118869409	-5.3314384	9.74E-08	5.25E-07
ENSG00000100445	SDR39U1	424.2325596	516.49558	331.9695429	-0.635367904	0.123890136	-5.1284786	2.92E-07	1.51E-06
ENSG00000146858	ZC3HAV1L	283.9378724	345.56017	222.3155748	-0.635747612	0.13230204	-4.8052745	1.55E-06	7.42E-06
ENSG00000092820	EZR	5476.2812	6663.3301	4289.232282	-0.635890875	0.038122526	-16.680188	1.83E-62	5.50E-61
ENSG00000186364	NUDT17	78.92253466	95.958572	61.88649751	-0.636158492	0.265028748	-2.4003377	0.01638	0.040124
ENSG00000235387	SPAAR	238.1670658	289.95189	186.3822406	-0.636258072	0.151967006	-4.1868172	2.83E-05	0.000117
ENSG00000011021	CLCN6	900.6126094	1096.4028	704.8224459	-0.636639331	0.092328087	-6.8954026	5.37E-12	4.05E-11
ENSG00000224078	SNHG14	792.2968934	964.48013	620.1136575	-0.637210152	0.091842345	-6.9380867	3.97E-12	3.02E-11
ENSG00000100234	TIMP3	5721.882874	6965.8089	4477.95681	-0.637528436	0.05455777	-11.685383	1.51E-31	2.52E-30
ENSG00000121966	CXCR4	6336.906216	7714.4085	4959.403895	-0.637692532	0.056553226	-11.275971	1.72E-29	2.69E-28
ENSG00000267321	LINC02001	83.02844093	101.1909	64.86598225	-0.639323292	0.273529485	-2.3373103	0.019423	0.046686
ENSG00000107872	FBXL15	271.8107503	331.02247	212.5990298	-0.640929005	0.13649439	-4.6956436	2.66E-06	1.24E-05
ENSG00000164684	ZNF704	1154.46425	1406.4799	902.4485597	-0.641483903	0.070328769	-9.1212161	7.43E-20	8.26E-19
ENSG00000177030	DEAF1	1546.431009	1885.3039	1207.558105	-0.642457641	0.063858118	-10.060704	8.24E-24	1.06E-22
ENSG00000141753	IGFBP4	17980.6693	21922.468	14038.87095	-0.642933374	0.031838412	-20.193638	1.11E-90	4.74E-89
ENSG00000160957	RECQL4	316.7034941	386.39004	247.0169447	-0.642985365	0.130678738	-4.9203518	8.64E-07	4.28E-06
ENSG00000135002	RFK	1476.070396	1799.998	1152.142775	-0.643089709	0.064096146	-10.033204	1.09E-23	1.40E-22
ENSG00000106462	EZH2	347.7454064	424.38406	271.1067499	-0.644465664	0.126065023	-5.1121687	3.18E-07	1.64E-06
ENSG00000115392	FANCL	185.5818707	226.3093	144.8544391	-0.644589512	0.170803404	-3.7738681	0.000161	0.000592
ENSG00000236824	BCYRN1	74.83445927	91.28226	58.38665902	-0.646348219	0.256432845	-2.520536	0.011718	0.029753
ENSG00000181722	ZBTB20	852.5758735	1040.6105	664.5412831	-0.6467378	0.090808825	-7.1219708	1.06E-12	8.38E-12
ENSG00000276023	DUSP14	1373.444365	1676.2925	1070.59625	-0.646868904	0.065897881	-9.8162322	9.59E-23	1.19E-21
ENSG00000130024	PHF10	1832.563795	2237.3131	1427.814469	-0.647404783	0.06751394	-9.5892016	8.88E-22	1.07E-20
ENSG00000145916	RMND5B	885.5608118	1081.4948	689.6268194	-0.647601823	0.083420497	-7.763102	8.29E-15	7.31E-14
ENSG00000256525	POLG2	130.1086608	158.88355	101.3337757	-0.647639403	0.193295414	-3.3505161	0.000807	0.002636
ENSG00000114857	NKTR	2084.408553	2545.5854	1623.231673	-0.648543702	0.073067089	-8.8760031	6.93E-19	7.40E-18
ENSG00000099260	PALMD	6458.196452	7887.1875	5029.205412	-0.649066036	0.042651528	-15.217885	2.69E-52	6.82E-51
ENSG00000180537	RNF182	394.1990685	481.20652	307.1916133	-0.649828758	0.115088584	-5.6463355	1.64E-08	9.48E-08
ENSG00000176105	YES1	3733.220362	4560.0581	2906.382593	-0.650102589	0.042667994	-15.236305	2.03E-52	5.15E-51
ENSG00000203706	SERTAD4-AS1	90.91570971	111.05609	70.77533328	-0.650216873	0.232579674	-2.7956737	0.005179	0.014248
ENSG00000078687	TNRC6C	808.491904	987.39274	629.5910702	-0.650723683	0.0902099	-7.2134393	5.46E-13	4.39E-12
ENSG00000143819	EPHX1	1400.1511	1711.2238	1089.078397	-0.651700052	0.065488298	-9.951397	2.49E-23	3.16E-22
ENSG00000050820	BCAR1	9067.102285	11081.902	7052.302815	-0.651785768	0.044379364	-14.686686	7.85E-49	1.88E-47
ENSG00000117519	CNN3	10140.04864	12394.709	7885.387927	-0.652617523	0.032643469	-19.992284	6.43E-89	2.66E-87
ENSG00000064933	PMS1	513.439151	627.6752	399.2031017	-0.652768147	0.099682683	-6.5484609	5.81E-11	4.11E-10
ENSG00000136935	GOLGA1	1101.291994	1346.1819	856.4021186	-0.65323944	0.077062469	-8.476752	2.32E-17	2.31E-16
ENSG00000243943	ZNF512	399.5176856	488.35248	310.6828915	-0.654578985	0.124353356	-5.2638627	1.41E-07	7.51E-07
ENSG00000122386	ZNF205	505.8576801	619.12092	392.59444	-0.65521873	0.109178595	-6.0013479	1.96E-09	1.22E-08
ENSG00000164241	C5orf63	68.61808853	83.839117	53.39706048	-0.655370887	0.270181708	-2.4256671	0.01528	0.037843
ENSG00000154217	PITPNC1	510.9751678	624.8589	397.0914345	-0.655558688	0.113451817	-5.7783005	7.55E-09	4.51E-08
ENSG00000214425	LRRC37A4P	274.7673332	336.50458	213.0300826	-0.656128835	0.141876901	-4.6246347	3.75E-06	1.73E-05
ENSG00000228203	RNF144A-AS1	116.0485192	142.08664	90.01039833	-0.656444862	0.223665408	-2.9349414	0.003336	0.009605
ENSG00000186665	C17orf58	605.904772	741.50113	470.3084094	-0.656716537	0.10197496	-6.4399784	1.19E-10	8.23E-10
ENSG00000189171	S100A13	446.5471988	546.65185	346.4425526	-0.656795087	0.112574012	-5.8343402	5.40E-09	3.27E-08
ENSG00000179715	PCED1B	64.72508286	79.161322	50.28884362	-0.657011724	0.283681862	-2.316016	0.020557	0.049023
ENSG00000155189	AGPAT5	2897.182795	3545.4662	2248.899368	-0.657369074	0.062238292	-10.562132	4.46E-26	6.31E-25
ENSG00000038945	MSR1	354.0909194	433.24758	274.9342601	-0.658662491	0.127087715	-5.1827393	2.19E-07	1.14E-06
ENSG00000169282	KCNAB1	169.5359934	207.68221	131.3897818	-0.659297281	0.231556404	-2.8472427	0.00441	0.012309
ENSG00000110799	VWF	443564.9968	543338.24	343791.7491	-0.660318624	0.033066295	-19.969538	1.01E-88	4.18E-87
ENSG00000037757	MRI1	723.2976829	886.96167	559.6336985	-0.662942863	0.093244431	-7.1097315	1.16E-12	9.15E-12
ENSG00000173588	CEP83	159.8073685	195.94255	123.6721895	-0.663174707	0.175319688	-3.7826596	0.000155	0.000573
ENSG00000271254	AC240274.1	428.5018949	525.03337	331.9704221	-0.663324547	0.114259303	-5.8054314	6.42E-09	3.85E-08
ENSG00000267080	ASB16-AS1	116.5805857	142.94108	90.22009479	-0.663948807	0.216510551	-3.0665887	0.002165	0.006493
ENSG00000101868	POLA1	464.9043151	570.03701	359.7716152	-0.664214532	0.10823851	-6.1365823	8.43E-10	5.45E-09
ENSG00000164125	GASK1B	11880.51182	14574.959	9186.064914	-0.666057667	0.081129706	-8.2097878	2.22E-16	2.12E-15
ENSG00000167513	CDT1	167.6719404	205.645	129.6988855	-0.666278525	0.168132331	-3.9628222	7.41E-05	0.000288
ENSG00000138182	KIF20B	431.4176698	528.99572	333.839622	-0.666329232	0.113418933	-5.8749383	4.23E-09	2.57E-08
ENSG00000235863	B3GALT4	179.642206	220.38721	138.8972027	-0.666515444	0.186437151	-3.5750141	0.00035	0.00122
ENSG00000167302	TEPSIN	522.2445376	641.38152	403.1075506	-0.666832882	0.139861796	-4.7677987	1.86E-06	8.87E-06
ENSG00000152926	ZNF117	780.4633488	958.19578	602.7309195	-0.667988091	0.107476827	-6.2151825	5.13E-10	3.37E-09
ENSG00000263956	NBPF11	223.5162631	274.53231	172.5002202	-0.668302271	0.173464022	-3.8526852	0.000117	0.00044

ENSG00000178971	CTC1	1285.966113	1578.976	992.9561826	-0.668642355	0.077245392	-8.6560808	4.88E-18	5.04E-17
ENSG00000150510	FAM124A	809.8711987	994.00381	625.7385915	-0.668798351	0.084309976	-7.9326122	2.15E-15	1.95E-14
ENSG00000168890	TMEM150A	737.295773	905.86733	568.7242162	-0.669778034	0.090994591	-7.3606357	1.83E-13	1.51E-12
ENSG00000185477	GPRIN3	224.0299868	274.97426	173.0857113	-0.66980763	0.155067162	-4.3194679	1.56E-05	6.66E-05
ENSG00000138162	TACC2	1383.211831	1699.0368	1067.386859	-0.670130851	0.065902645	-10.168497	2.74E-24	3.59E-23
ENSG00000145284	SCD5	1647.456371	2023.8468	1271.065897	-0.671159332	0.068711081	-9.7678471	1.55E-22	1.91E-21
ENSG00000278535	DHRS11	151.6559215	186.38497	116.9268771	-0.672064115	0.181071778	-3.7115895	0.000206	0.000746
ENSG00000076351	SLC46A1	335.2226247	411.78236	258.6628881	-0.672307265	0.124010419	-5.4213773	5.91E-08	3.25E-07
ENSG00000128694	OSGEPL1	109.8950397	135.06249	84.72758505	-0.672405383	0.220914499	-3.0437359	0.002337	0.006955
ENSG00000177432	NAP1L5	196.1209761	241.05028	151.1916725	-0.672870577	0.165183441	-4.0734748	4.63E-05	0.000186
ENSG00000145687	SSBP2	432.2125916	531.42862	332.9965606	-0.673766128	0.112562257	-5.9857198	2.15E-09	1.34E-08
ENSG00000178685	PARP10	1065.758625	1311.0069	820.5103883	-0.673830568	0.085287326	-7.9007116	2.77E-15	2.51E-14
ENSG00000198722	UNC13B	4666.690693	5737.6811	3595.700276	-0.674062679	0.04010444	-16.807682	2.14E-63	6.52E-62
ENSG00000164237	CMBL	147.1527199	181.03463	113.270808	-0.674349915	0.193044912	-3.4932281	0.000477	0.00163
ENSG00000044446	PHKA2	409.4005715	503.61184	315.1893002	-0.674616656	0.112701948	-5.9858473	2.15E-09	1.34E-08
ENSG00000187650	VMAC	97.54904286	120.12441	74.97367399	-0.674816894	0.25569113	-2.6391877	0.00831	0.02189
ENSG00000164649	CDCA7L	1587.419906	1952.0564	1222.783416	-0.676211682	0.062889376	-10.752399	5.77E-27	8.36E-26
ENSG00000175066	GK5	311.2253311	383.01625	239.4344128	-0.676826501	0.147865029	-4.5773264	4.71E-06	2.15E-05
ENSG00000166349	RAG1	140.907105	173.37992	108.4342936	-0.677465614	0.192939014	-3.5112941	0.000446	0.00153
ENSG00000263465	SRSF8	1348.08053	1659.2584	1036.902638	-0.677563116	0.064892619	-10.441297	1.61E-25	2.21E-24
ENSG00000150907	FOXO1	3913.855143	4816.667	3011.043294	-0.677709401	0.044202936	-15.331773	4.69E-53	1.20E-51
ENSG00000099326	MZF1	255.8577299	315.16986	196.5455948	-0.677827194	0.167118981	-4.0559557	4.99E-05	0.000199
ENSG00000239213	NCK1-DT	84.9747403	104.54979	65.39969364	-0.677876341	0.251043535	-2.7002342	0.006929	0.018552
ENSG00000279457	WASH9P	82.52099641	101.72454	63.31744862	-0.679328398	0.258146107	-2.6315655	0.008499	0.022326
ENSG00000160271	RALGDS	279.7230678	344.71813	214.7280081	-0.679445517	0.154081602	-4.4096473	1.04E-05	4.52E-05
ENSG00000099194	SCD	8589.541708	10575.924	6603.159877	-0.679724604	0.044187408	-15.382767	2.14E-53	5.58E-52
ENSG00000119397	CNTRL	479.976371	591.00651	368.9462322	-0.679933555	0.102670306	-6.6224946	3.53E-11	2.53E-10
ENSG00000132424	PNISR	1071.789934	1320.5011	823.0787561	-0.680987893	0.101420009	-6.714532	1.89E-11	1.38E-10
ENSG00000164659	KIAA1324L	4146.898565	5108.3491	3185.448038	-0.681330213	0.046453137	-14.667044	1.05E-48	2.51E-47
ENSG00000156876	SASS6	85.56356931	105.46821	65.65892634	-0.681374953	0.247477935	-2.7532756	0.0059	0.016019
ENSG00000183580	FBXL7	1926.43988	2374.5394	1478.34039	-0.683026216	0.056267201	-12.138976	6.56E-34	1.16E-32
ENSG00000068650	ATP11A	5676.478667	6994.8283	4358.129011	-0.683028269	0.037813099	-18.063272	6.20E-73	2.16E-71
ENSG00000144118	RALB	4685.974613	5774.8096	3597.139633	-0.683038092	0.045991699	-14.851334	6.82E-50	1.67E-48
ENSG00000139734	DIAPH3	621.3247733	765.9889	476.6606514	-0.683637565	0.091934265	-7.4361563	1.04E-13	8.69E-13
ENSG00000134243	SORT1	3356.620361	4137.5992	2575.641558	-0.684192038	0.050200915	-13.629075	2.69E-42	5.68E-41
ENSG00000185485	SDHAP1	204.989782	252.88148	157.0980792	-0.684306821	0.174082284	-3.9309389	8.46E-05	0.000326
ENSG00000261971	MMP25-AS1	99.19458686	122.44958	75.93959022	-0.686017753	0.241277389	-2.8432741	0.004465	0.012446
ENSG00000145390	USP53	817.7557231	1008.8229	626.6885494	-0.686126382	0.081243236	-8.4453355	3.03E-17	3.01E-16
ENSG00000175879	HOXD8	426.700851	526.47941	326.9222874	-0.686504099	0.108715184	-6.314703	2.71E-10	1.82E-09
ENSG00000126215	XRCC3	217.4792691	268.04798	166.9105617	-0.686896339	0.157467021	-4.36216	1.29E-05	5.55E-05
ENSG00000119403	PHF19	1079.34797	1331.6741	827.0218543	-0.686926265	0.070780538	-9.7050161	2.87E-22	3.52E-21
ENSG00000083720	OXCT1	1093.023358	1348.924	837.1227543	-0.687424584	0.071264392	-9.6461158	5.11E-22	6.18E-21
ENSG00000178718	RPP25	1271.559821	1568.7922	974.3274098	-0.688124182	0.067575862	-10.182988	2.36E-24	3.10E-23
ENSG00000174032	SLC25A30	976.964177	1205.1499	748.7784252	-0.688587159	0.078849659	-8.7329124	2.48E-18	2.60E-17
ENSG00000171488	LRRC8C	4517.847841	5575.9262	3459.769509	-0.688916318	0.045605909	-15.105857	1.48E-51	3.72E-50
ENSG00000167536	DHRS13	117.5863701	145.23568	89.93705583	-0.68900106	0.21174774	-3.2538768	0.001138	0.003615
ENSG00000063660	GPC1	3179.77171	3925.3671	2434.17632	-0.689096149	0.060824483	-11.329256	9.40E-30	1.48E-28
ENSG00000198039	ZNF273	102.6197759	126.68103	78.55852637	-0.689282378	0.213887878	-3.2226341	0.00127	0.003997
ENSG00000124831	LRRFIP1	6351.932849	7841.3985	4862.467184	-0.689669576	0.039844629	-17.308972	4.03E-67	1.29E-65
ENSG00000178026	LRRC75B	173.1179686	213.91764	132.3182966	-0.689768177	0.180280651	-3.8260799	0.00013	0.000487
ENSG00000165475	CRYL1	1570.621136	1939.6013	1201.640941	-0.69010687	0.061702128	-11.184491	4.86E-29	7.46E-28
ENSG00000214226	C17orf67	124.4041246	153.71279	95.09545939	-0.69039538	0.199388675	-3.4625607	0.000535	0.00181
ENSG00000126016	AMOT	78.14042445	96.341773	59.93907548	-0.6911006	0.280563002	-2.4632635	0.013768	0.034444
ENSG00000166938	DIS3L	712.506678	880.01144	545.0019181	-0.692557479	0.088385429	-7.8356521	4.66E-15	4.18E-14
ENSG00000146670	CDCA5	265.2087175	327.86073	202.5567013	-0.693557175	0.152761895	-4.540119	5.62E-06	2.54E-05
ENSG00000125434	SLC25A35	135.8415835	167.95715	103.7260124	-0.694130279	0.229886493	-3.0194479	0.002532	0.007472
ENSG00000143643	TTC13	784.3312191	969.8393	598.8231334	-0.694230891	0.090914751	-7.6360643	2.24E-14	1.94E-13
ENSG00000183688	RFLNB	2890.18913	3572.3516	2208.026684	-0.694536757	0.052434746	-13.245735	4.78E-40	9.56E-39
ENSG00000115641	FHL2	2884.922901	3567.7566	2202.08924	-0.696275933	0.053052749	-13.12422	2.39E-39	4.71E-38
ENSG00000111261	MANSC1	2958.042371	3658.5825	2257.502267	-0.696643951	0.055099337	-12.643418	1.22E-36	2.28E-35
ENSG00000137700	SLC37A4	404.3534941	500.19277	308.5142192	-0.696795595	0.112516059	-6.1928546	5.91E-10	3.87E-09
ENSG00000121060	TRIM25	6062.430499	7501.047	4623.813954	-0.697409486	0.043376988	-16.077868	3.65E-58	1.03E-56
ENSG00000145375	SPATA5	287.281497	355.35831	219.2046864	-0.698405693	0.130395202	-5.356069	8.51E-08	4.61E-07
ENSG00000197077	KIAA1671	6429.202319	7956.8721	4901.532526	-0.699185678	0.042040373	-16.631291	4.14E-62	1.24E-60
ENSG00000176593	AC008969.1	135.0862415	167.30743	102.8650574	-0.699654627	0.205584429	-3.4032472	0.000666	0.002213
ENSG00000160211	G6PD	3274.009034	4052.9936	2495.024498	-0.699955254	0.050446824	-13.87511	8.97E-44	1.93E-42
ENSG00000164070	HSPA4L	791.3127639	979.5933	603.032231	-0.700683559	0.085884629	-8.158428	3.39E-16	3.21E-15
ENSG00000229124	VIM-AS1	488.0244615	603.88833	372.1605977	-0.701364137	0.147366404	-4.7593218	1.94E-06	9.24E-06
ENSG00000109062	SLC9A3R1	399.9334943	495.58746	304.2795332	-0.702959393	0.120627562	-5.8275189	5.63E-09	3.39E-08
ENSG00000198205	ZXDA	347.7357275	430.83753	264.6339218	-0.702990929	0.124019143	-5.6684066	1.44E-08	8.37E-08
ENSG00000272674	PCDHB16	106.5307996	131.82344	81.23815981	-0.703133519	0.223053973	-3.1523022	0.00162	0.004984
ENSG00000117632	STMN1	4602.659309	5703.3613	3501.957274	-0.703596173	0.048577549	-14.483978	1.53E-47	3.57E-46
ENSG00000107902	LHPP	191.0804978	236.81967	145.3413211	-0.705835483	0.169072098	-4.1747603	2.98E-05	0.000123
ENSG00000058668	ATP2B4	11350.17665	14072.87	8627.483463	-0.706198246	0.036349409	-19.428053	4.47E-84	1.75E-82
ENSG00000179348	GATA2	1649.970927	2046.4083	1253.533555	-0.706489997	0.062112736	-11.374318	5.61E-30	8.89E-29
ENSG00000169169	CPT1C	1154.751144	1432.6909	876.8113476	-0.706787566	0.073662588	-9.5949326	8.40E-22	1.01E-20
ENSG00000185864	NPIPB4	190.5050994	236.55414	144.456061	-0.708524432	0.186541818	-3.798207	0.000146	0.000541
ENSG00000254221	PCDHGB1	70.34423738	87.299666	53.38880843	-0.708945542	0.272372739	-2.6028506	0.009245	0.024025
ENSG00000075391	RASAL2	2258.851656	2803.4035	1714.299827	-0.709068171	0.05921771	-11.973921	4.87E-33	8.39E-32
ENSG00000157978	LDLRAP1	1290.661993	1601.7641	979.5598683	-0.709092459	0.066071175	-10.732251	7.18E-27	1.04E-25
ENSG00000164124	TMEM144	151.5986257	188.36091	114.8363446	-0.709668746	0.190665461	-3.7220624	0.000198	0.000717
ENSG00000103145	HCFC1R1	447.809492	555.61544	340.0035398	-0.710104776	0.110353314	-6.4348296	1.24E-10	8.50E-10
ENSG00000065308	TRAM2	13067.27529	16220.63	9913.920135	-0.710383699	0.033238335	-21.372422	2.41E-101	1.14E-99
ENSG00000278175	GLIDR	77.76947307	96.536163	59.00278351	-0.710451663	0.255825865	-2.7770908	0.005485	0.015
ENSG00000108622	ICAM2	12990.46223	16127.343	9853.581462	-0.710684598	0.032794043	-21.671149	3.84E-104	1.85E-102

ENSG00000162909	CAPN2	19224.22315	23867.617	14580.82942	-0.711033819	0.028936634	-24.572099	2.51E-133	1.64E-131
ENSG00000125089	SH3TC1	4408.449523	5474.1092	3342.789806	-0.711127741	0.046315895	-15.353859	3.34E-53	8.60E-52
ENSG00000149582	TMEM25	573.4341538	711.78188	435.0864247	-0.711517106	0.097191127	-7.3208032	2.46E-13	2.02E-12
ENSG00000172465	TCEAL1	312.7771108	388.34959	237.2046289	-0.711965066	0.123917382	-5.7454818	9.17E-09	5.42E-08
ENSG00000157554	ERG	8376.952783	10407.877	6346.028124	-0.713889885	0.036002574	-19.828857	1.68E-87	6.78E-86
ENSG00000178075	GRAMD1C	506.2824426	628.68324	383.8816407	-0.713919871	0.110752502	-6.4460835	1.15E-10	7.93E-10
ENSG00000160062	ZBTB8A	464.9787463	578.0062	351.9512936	-0.714487961	0.106070085	-6.7359988	1.63E-11	1.19E-10
ENSG00000230715	AC018638.2	80.95723377	100.6815	61.23297166	-0.71511633	0.251074098	-2.8482282	0.004396	0.012275
ENSG00000118495	PLAGL1	1404.668402	1746.3636	1062.973241	-0.715465786	0.079866888	-8.9582279	3.30E-19	3.56E-18
ENSG00000107186	MPDZ	3859.671344	4798.0742	2921.268526	-0.716373804	0.044269808	-16.181995	6.76E-59	1.95E-57
ENSG00000116991	SIPA1L2	897.6393746	1116.1625	679.1162743	-0.716697453	0.079756104	-8.9861141	2.56E-19	2.77E-18
ENSG00000106546	AHR	1180.93554	1468.811	893.0601007	-0.717331233	0.070187297	-10.220243	1.61E-24	2.14E-23
ENSG00000124019	FAM124B	2667.069192	3316.6755	2017.462868	-0.717373259	0.057810916	-12.408959	2.34E-35	4.26E-34
ENSG00000177054	ZDHHC13	1690.933227	2103.3481	1278.51838	-0.719046806	0.06433373	-11.176824	5.30E-29	8.10E-28
ENSG00000274487	AC244154.1	85.6012705	106.5516	64.65094274	-0.720061592	0.24778475	-2.9059964	0.003661	0.010438
ENSG00000196700	ZNF512B	2156.410982	2684.839	1627.982995	-0.721502627	0.053554365	-13.47234	2.28E-41	4.67E-40
ENSG00000184216	IRAK1	4845.54197	6034.5096	3656.574336	-0.722428944	0.039368139	-18.350599	3.26E-75	1.16E-73
ENSG00000176208	ATAD5	111.0581154	138.33586	83.7803695	-0.722491937	0.234058152	-3.0868053	0.002023	0.006099
ENSG00000276043	UHRF1	502.9907508	626.71737	379.2641308	-0.723099566	0.112253009	-6.4416943	1.18E-10	8.15E-10
ENSG00000140525	FANCI	819.4837339	1020.7052	618.2623144	-0.723498443	0.078832676	-9.1776466	4.41E-20	4.97E-19
ENSG00000159423	ALDH4A1	683.9004229	852.41329	515.3875562	-0.724610238	0.097673277	-7.4187154	1.18E-13	9.90E-13
ENSG00000186787	SPIN2B	79.97230915	99.727372	60.21724634	-0.725034886	0.243013751	-2.9835138	0.00285	0.008316
ENSG00000077092	RARB	452.3247694	563.74149	340.9080463	-0.725257919	0.113230746	-6.4051324	1.50E-10	1.03E-09
ENSG00000106089	STX1A	137.5616954	171.39035	103.7330417	-0.726173699	0.192222627	-3.7777743	0.000158	0.000584
ENSG00000184381	PLA2G6	86.34021971	107.74909	64.93134555	-0.726417015	0.263766748	-2.7540129	0.005887	0.015985
ENSG00000165792	METTL17	571.5353297	712.54651	430.5241515	-0.727066519	0.117790936	-6.1725167	6.72E-10	4.37E-09
ENSG00000187609	EXD3	780.3431104	973.13223	587.5539937	-0.727367427	0.091988919	-7.9071201	2.63E-15	2.39E-14
ENSG00000169570	DTWD2	100.6568022	125.58584	75.72776794	-0.72755761	0.231481035	-3.1430549	0.001672	0.005131
ENSG00000141258	SGSM2	2069.911665	2582.0673	1557.756048	-0.727857681	0.058756605	-12.387674	3.05E-35	5.54E-34
ENSG00000139998	RAB15	1023.021311	1276.7233	769.3193307	-0.729952679	0.078082768	-9.3484478	8.89E-21	1.03E-19
ENSG00000198556	ZNF789	132.0252621	164.81191	99.23861258	-0.730160862	0.191692877	-3.809014	0.00014	0.000519
ENSG00000260027	HOXB7	1112.204264	1388.245	836.1635453	-0.730163475	0.077656878	-9.4024315	5.33E-21	6.24E-20
ENSG00000059588	TARBP1	995.160788	1241.7513	748.5702483	-0.730722088	0.08677457	-8.4209243	3.74E-17	3.69E-16
ENSG00000170421	KRT8	451.284588	563.5752	338.9939761	-0.731059294	0.109621898	-6.6689166	2.58E-11	1.86E-10
ENSG00000099204	ABLIM1	6142.104442	7665.7743	4618.434602	-0.731212462	0.047281979	-15.464929	5.98E-54	1.57E-52
ENSG00000186567	CEACAM19	125.330757	156.66656	93.99495766	-0.73160457	0.242232058	-3.0202632	0.002526	0.007453
ENSG00000113594	LIFR	1695.148569	2115.1176	1275.179522	-0.731860357	0.069673986	-10.504069	8.27E-26	1.16E-24
ENSG00000187994	RINL	101.2859972	126.61343	75.95856375	-0.7320005	0.239453762	-3.0569597	0.002236	0.006687
ENSG00000069702	TGFBR3	1312.55707	1639.2146	985.8995712	-0.732953986	0.066641239	-10.998505	3.89E-28	5.77E-27
ENSG00000119772	DNMT3A	1265.399015	1580.487	950.3110532	-0.734386179	0.079369481	-9.2527527	2.19E-20	2.49E-19
ENSG00000125454	SLC25A19	181.2153726	226.46923	135.9615184	-0.73465019	0.171419728	-4.2856805	1.82E-05	7.70E-05
ENSG00000116191	RALGPS2	1832.271664	2288.6592	1375.884136	-0.734675741	0.065036806	-11.296307	1.37E-29	2.14E-28
ENSG00000239900	ADSL	123.3817933	154.15129	92.61229613	-0.735009843	0.23585175	-3.1164061	0.001831	0.005573
ENSG00000139160	ETFBKMT	102.9880154	128.65554	77.32049476	-0.73566413	0.226016413	-3.2549146	0.001134	0.003603
ENSG00000112576	CCND3	2770.735176	3462.8918	2078.578528	-0.736339336	0.050001015	-14.726488	4.36E-49	1.05E-47
ENSG00000127220	ABHD8	211.694634	264.66904	158.7202282	-0.736751594	0.152166273	-4.8417535	1.29E-06	6.25E-06
ENSG00000168961	LGALS9	2955.466366	3697.2585	2213.67428	-0.738976642	0.069386724	-10.650116	1.74E-26	2.48E-25
ENSG00000214331	AC009053.1	135.0648025	169.09757	101.032036	-0.73975931	0.196736931	-3.7601446	0.00017	0.000623
ENSG00000132906	CASP9	601.3764832	752.66982	450.0831469	-0.740017707	0.104246578	-7.0987242	1.26E-12	9.89E-12
ENSG00000215375	MYL5	196.6585217	246.2156	147.1014396	-0.740701652	0.16954579	-4.3687411	1.25E-05	5.40E-05
ENSG00000132589	FLOT2	7622.652038	9537.451	5707.85304	-0.740704925	0.033818741	-21.902203	2.48E-106	1.23E-104
ENSG00000145555	MYO10	13297.71957	16644.28	9951.159409	-0.74222334	0.033229089	-22.336554	1.63E-110	8.55E-109
ENSG00000005469	CROT	355.748084	445.22306	266.2731091	-0.74272486	0.122316157	-6.072173	1.26E-09	8.02E-09
ENSG00000174749	FAM241A	3691.227595	4621.1156	2761.339546	-0.743344936	0.049076474	-15.146665	7.97E-52	2.01E-50
ENSG00000247287	AL359220.1	107.6712138	134.92903	80.41339489	-0.743637929	0.219314665	-3.3907351	0.000697	0.002309
ENSG00000205336	ADGRG1	3731.492813	4673.2186	2789.767013	-0.744201575	0.048596148	-15.314003	6.16E-53	1.58E-51
ENSG00000104154	SLC30A4	641.553412	803.50101	479.6058105	-0.744686828	0.092451834	-8.0548627	7.96E-16	7.38E-15
ENSG00000077782	FGFR1	3669.778315	4596.5883	2742.968295	-0.744786368	0.046307826	-16.08338	3.34E-58	9.41E-57
ENSG00000099860	GADD45B	2188.637519	2741.7391	1635.535938	-0.745137035	0.05354316	-13.916568	5.02E-44	1.09E-42
ENSG00000145604	SKP2	522.4293413	654.38968	390.4690009	-0.745673119	0.10698881	-6.9696365	3.18E-12	2.43E-11
ENSG00000164008	C1orf50	101.2150658	126.94533	75.48480187	-0.745965386	0.225381769	-3.3097858	0.000934	0.003014
ENSG00000130584	ZBTB46	549.3423384	688.21847	410.4662098	-0.746564952	0.103035827	-7.2456831	4.30E-13	3.48E-12
ENSG00000160949	TONSL	290.2609458	363.79374	216.7281483	-0.747435424	0.137666496	-5.4293198	5.66E-08	3.11E-07
ENSG00000143314	MRPL24	915.3295912	1147.3206	683.3386233	-0.74847701	0.077205325	-9.6946294	3.18E-22	3.89E-21
ENSG00000088448	ANKRD10	1179.499873	1478.9111	880.0885955	-0.74907338	0.090396197	-8.2865586	1.17E-16	1.13E-15
ENSG00000115183	TANC1	8247.099476	10343.197	6151.001508	-0.750096465	0.035597487	-21.071613	1.45E-98	6.67E-97
ENSG00000204397	CARD16	2459.746029	3086.7737	1832.718388	-0.750809585	0.058364327	-12.864186	7.16E-38	1.37E-36
ENSG00000110921	MVK	571.1570214	716.98369	425.3303525	-0.751778563	0.098105344	-7.6629726	1.82E-14	1.58E-13
ENSG00000164938	TP53INP1	3553.476441	4460.5265	2646.42639	-0.753219149	0.048827456	-15.426139	1.09E-53	2.86E-52
ENSG00000196155	PLEKHG4	411.5290174	516.90065	306.1573894	-0.753790744	0.122247465	-6.1661053	7.00E-10	4.55E-09
ENSG00000137198	GMPR	884.9955613	1111.6103	658.3808338	-0.756315143	0.077121353	-9.8068189	1.05E-22	1.31E-21
ENSG00000116133	DHCR24	3861.837639	4852.1388	2871.536518	-0.756686308	0.060772715	-12.451086	1.38E-35	2.53E-34
ENSG00000164904	ALDH7A1	1743.261293	2190.3727	1296.149914	-0.757093137	0.062267215	-12.158776	5.15E-34	9.13E-33
ENSG00000188811	NHLRC3	437.6970474	549.97681	325.4172814	-0.757464597	0.108322222	-6.9926981	2.70E-12	2.07E-11
ENSG00000215883	CYB5RL	146.5479918	184.265	108.8309854	-0.757777023	0.191637964	-3.9542114	7.68E-05	0.000298
ENSG00000138738	PRDM5	133.5045141	167.81559	99.19343412	-0.758834994	0.189117926	-4.0124964	6.01E-05	0.000237
ENSG00000219481	NBPF1	545.9793266	686.63965	405.3190027	-0.759505152	0.09791324	-7.7569198	8.70E-15	7.67E-14
ENSG00000143554	SLC27A3	540.0558665	679.2641	400.8476321	-0.761515002	0.09884606	-7.7040501	1.32E-14	1.15E-13
ENSG00000196535	MYO18A	1638.988233	2062.2182	1215.758271	-0.76197739	0.060668462	-12.559695	3.52E-36	6.51E-35
ENSG00000057704	TMCC3	1284.340992	1616.1601	952.5219242	-0.762897363	0.066704448	-11.436979	2.73E-30	4.37E-29
ENSG00000005249	PRKAR2B	2256.729197	2839.5908	1673.867623	-0.763221437	0.058606804	-13.022745	9.08E-39	1.76E-37
ENSG00000102174	PHEX	99.98711091	125.794	74.18021958	-0.764299117	0.249518775	-3.0630926	0.002191	0.00656
ENSG00000154065	ANKRD29	61.11331067	76.805638	45.42098327	-0.76464499	0.297115332	-2.5735629	0.010066	0.025916
ENSG00000143578	CREB3L4	162.234313	204.18211	120.2865142	-0.764759096	0.175501049	-4.3575756	1.32E-05	5.66E-05
ENSG00000242086	MUC20-OT1	217.3749673	273.77396	160.9759796	-0.764963288	0.193632491	-3.9505936	7.80E-05	0.000302

ENSG00000166002	SMCO4	110.8670574	139.62327	82.11084548	-0.765952552	0.235091337	-3.2581062	0.001122	0.003565
ENSG00000134321	RSAD2	141.3827453	178.24623	104.5192647	-0.766081831	0.191486334	-4.0007128	6.32E-05	0.000248
ENSG00000170899	GSTA4	342.6721992	431.79274	253.5516575	-0.766521528	0.127510665	-6.0114307	1.84E-09	1.16E-08
ENSG00000080503	SMARCA2	2700.28589	3401.3117	1999.260103	-0.766586267	0.049027244	-15.635924	4.14E-55	1.11E-53
ENSG00000259431	THTPA	110.9148379	139.74774	82.08193364	-0.766972516	0.219605049	-3.4925086	0.000479	0.001633
ENSG00000213918	DNASE1	300.2187996	378.38393	222.0536706	-0.767019936	0.150845468	-5.084806	3.68E-07	1.89E-06
ENSG00000122644	ARL4A	1001.263814	1261.6913	740.8363301	-0.768312261	0.076642744	-10.024592	1.19E-23	1.52E-22
ENSG00000006025	AC003665.1	384.1224343	484.35833	283.8865355	-0.768961217	0.136997852	-5.6129436	1.99E-08	1.14E-07
ENSG00000136098	NEK3	352.3070307	444.04166	260.5723985	-0.770246181	0.123102184	-6.256966	3.93E-10	2.60E-09
ENSG00000169607	CKAP2L	232.8233734	293.65961	171.9871333	-0.770465302	0.148719992	-5.1806438	2.21E-07	1.15E-06
ENSG00000186812	ZNF397	204.6159197	258.02489	151.2069539	-0.770713702	0.154370023	-4.9926384	5.96E-07	2.99E-06
ENSG00000140534	TICRR	107.9704783	136.2868	79.65416	-0.772637864	0.241975248	-3.193045	0.001408	0.004389
ENSG00000165644	COMTD1	200.9120686	253.42708	148.3970574	-0.772977772	0.163930766	-4.7152697	2.41E-06	1.13E-05
ENSG00000164611	PTTG1	319.2950037	402.85935	235.7306605	-0.773212171	0.13127544	-5.8899987	3.86E-09	2.36E-08
ENSG00000162946	DISC1	428.7205173	541.01272	316.4283171	-0.774570819	0.11587299	-6.6846538	2.31E-11	1.68E-10
ENSG00000172292	CERS6	1199.200199	1513.4697	884.9307019	-0.774597039	0.070323977	-11.014693	3.25E-28	4.83E-27
ENSG00000164104	HMGB2	1154.707319	1457.8396	851.5750082	-0.774597847	0.075105063	-10.313524	6.12E-25	8.23E-24
ENSG00000149743	TRPT1	355.0273518	448.31734	261.7373604	-0.775123326	0.120983629	-6.4068448	1.49E-10	1.02E-09
ENSG00000188312	CENPP	73.19976809	92.452154	53.94738209	-0.775297708	0.284031069	-2.7296229	0.006341	0.017118
ENSG00000055813	CCDC85A	301.7711441	380.94827	222.5940185	-0.775565494	0.128244663	-6.0475459	1.47E-09	9.31E-09
ENSG00000137693	YAP1	432.9336763	546.80471	319.0626407	-0.775888588	0.112533656	-6.8947248	5.40E-12	4.07E-11
ENSG00000172167	MTBP	72.44632087	91.582756	53.30988539	-0.77608895	0.264043292	-2.9392489	0.00329	0.00949
ENSG00000130299	GTPBP3	257.4759086	325.05949	189.8923256	-0.776794966	0.137999935	-5.6289517	1.81E-08	1.05E-07
ENSG00000227946	AC007383.2	80.87936634	102.21395	59.54478233	-0.778975569	0.266175156	-2.9265525	0.003427	0.009841
ENSG00000198885	ITPRIPL1	94.65999046	119.53102	69.78895937	-0.779510942	0.237652929	-3.2800393	0.001038	0.003324
ENSG00000168026	TTC21A	91.86599258	116.13436	67.59762137	-0.780967982	0.26337963	-2.9651799	0.003025	0.008782
ENSG00000197822	OCLN	618.4750076	782.2925	454.6575197	-0.782491098	0.098333516	-7.9575218	1.76E-15	1.61E-14
ENSG00000146859	TMEM140	2491.787491	3151.8792	1831.695825	-0.78283759	0.059075895	-13.251388	4.43E-40	8.87E-39
ENSG00000071539	TRIP13	374.5965736	473.95085	275.2422927	-0.784566744	0.119604863	-6.5596559	5.39E-11	3.82E-10
ENSG00000143127	ITGA10	973.9989182	1233.0209	714.9769178	-0.784654229	0.086613275	-9.0592837	1.31E-19	1.45E-18
ENSG00000138190	EXOC6	3880.751175	4911.0849	2850.417428	-0.784760389	0.051805599	-15.148177	7.79E-52	1.97E-50
ENSG00000148180	GSN	11867.05219	15031.952	8702.152244	-0.788716194	0.03223051	-24.471105	3.00E-132	1.92E-130
ENSG00000132846	ZBED3	948.7126707	1202.0156	695.4097411	-0.789219757	0.092407793	-8.540619	1.34E-17	1.34E-16
ENSG00000139354	GAS2L3	560.8898027	710.44412	411.3354876	-0.789245488	0.107262662	-7.3580636	1.87E-13	1.54E-12
ENSG00000143224	PPOX	449.9241464	570.28542	329.5628686	-0.789917854	0.11828214	-6.6782513	2.42E-11	1.75E-10
ENSG00000173269	MMRN2	12349.15785	15649.764	9048.552124	-0.790441803	0.031283995	-25.266651	7.43E-141	5.18E-139
ENSG00000100479	POLE2	58.96362733	74.792122	43.13513265	-0.791375795	0.286623624	-2.7610278	0.005762	0.015683
ENSG00000006062	MAP3K14	272.4745631	345.86886	199.0802649	-0.793403026	0.14551886	-5.452235	4.97E-08	2.74E-07
ENSG00000113368	LMNB1	771.8626391	979.46665	564.2586282	-0.793994316	0.08725135	-9.1000806	9.03E-20	1.00E-18
ENSG00000140350	ANP32A	2766.462866	3508.9044	2024.021328	-0.794530277	0.053158008	-14.946577	1.64E-50	4.04E-49
ENSG00000187678	SPRY4	1665.32837	2112.3767	1218.280034	-0.794801354	0.063431785	-12.530017	5.12E-36	9.43E-35
ENSG00000047634	SCML1	174.3741289	221.16324	127.5850218	-0.795537332	0.172408355	-4.6142621	3.94E-06	1.81E-05
ENSG00000272941	AC083862.2	205.1789096	260.49626	149.8615624	-0.795677605	0.185302477	-4.2939394	1.76E-05	7.44E-05
ENSG00000172731	LRRC20	328.4298207	417.01399	239.8456497	-0.797777197	0.13140775	-6.0710057	1.27E-09	8.08E-09
ENSG00000118193	KIF14	222.6371062	282.68146	162.5927501	-0.798360429	0.154633773	-5.1629111	2.43E-07	1.26E-06
ENSG00000135363	LMO2	1418.058561	1801.3732	1034.743904	-0.799977372	0.072947217	-10.966524	5.54E-28	8.20E-27
ENSG00000198625	MDM4	425.3981956	540.71859	310.0777967	-0.800138396	0.129561826	-6.1757265	6.59E-10	4.29E-09
ENSG00000249115	HAUS5	226.8175306	288.24455	165.3905118	-0.800605538	0.162521073	-4.9261645	8.39E-07	4.16E-06
ENSG00000055163	CYFIP2	769.7154463	978.04583	561.385066	-0.801639156	0.110571974	-7.2499308	4.17E-13	3.37E-12
ENSG00000196724	ZNF418	68.21763446	86.768095	49.66717397	-0.803514832	0.269609258	-2.9802939	0.00288	0.008396
ENSG00000162337	LRP5	2857.045609	3633.7051	2080.386092	-0.803834125	0.052179164	-15.40527	1.51E-53	3.95E-52
ENSG00000170498	KISS1	42.60953187	54.218716	31.00034804	-0.80414151	0.342145825	-2.3502888	0.018759	0.045239
ENSG00000225177	AL590617.2	92.25000135	117.46551	67.03448782	-0.805721687	0.242004246	-3.32937	0.00087	0.002824
ENSG00000074855	ANO8	291.7913503	371.64004	211.9426656	-0.807213947	0.157421914	-5.1277101	2.93E-07	1.51E-06
ENSG00000250571	GLI4	113.9806623	145.20206	82.75926576	-0.808338558	0.218159738	-3.70526	0.000211	0.000763
ENSG00000091651	ORC6	194.2049883	247.67123	140.7387489	-0.811475438	0.167409859	-4.8472381	1.25E-06	6.09E-06
ENSG00000276386	CNTNAP3P2	353.501373	450.35634	256.6464106	-0.811499447	0.122021788	-6.6504471	2.92E-11	2.10E-10
ENSG00000247809	NR2F2-AS1	56.85927383	72.363956	41.35459191	-0.81211853	0.298921241	-2.7168311	0.006591	0.017743
ENSG00000184979	USP18	886.5895622	1130.472	642.7071231	-0.812501363	0.092778897	-8.757394	2.00E-18	2.10E-17
ENSG00000256223	ZNF10	107.0629182	136.45092	77.67491712	-0.813591267	0.214134285	-3.7994442	0.000145	0.000538
ENSG00000237276	ANO7L1	41.73861391	53.2679	30.20932768	-0.813879455	0.351700134	-2.3141289	0.020661	0.049225
ENSG00000253552	HOXA-AS2	68.6676718	87.419896	49.91544772	-0.814466098	0.272976189	-2.9836525	0.002848	0.008314
ENSG00000102271	KLHL4	500.9125662	639.07393	362.7512061	-0.815561158	0.10804106	-7.5486224	4.40E-14	3.75E-13
ENSG00000134508	CABLES1	3610.38956	4606.7119	2614.067233	-0.817107414	0.043657992	-18.716102	3.66E-78	1.35E-76
ENSG00000152127	MGAT5	6969.340242	8891.2401	5047.440308	-0.817390384	0.039332398	-20.781606	6.35E-96	2.85E-94
ENSG00000279692	AC110285.7	58.13301134	74.260304	42.00571908	-0.81805597	0.302551488	-2.703857	0.006854	0.018381
ENSG00000211584	SLC48A1	525.9582035	671.26445	380.6519585	-0.819613809	0.10314794	-7.9460027	1.93E-15	1.76E-14
ENSG00000272335	AC093297.2	49.03039647	62.658296	35.40249646	-0.819650052	0.330342712	-2.4812112	0.013094	0.032916
ENSG00000100292	HMOX1	1869.42906	2386.7825	1352.075587	-0.819721649	0.140290071	-5.8430482	5.13E-09	3.10E-08
ENSG00000243716	NPIPB5	168.5441649	215.3077	121.780632	-0.819882269	0.182449298	-4.493754	7.00E-06	3.13E-05
ENSG00000170836	PPM1D	947.9460575	1210.3964	685.4957418	-0.819954035	0.074864535	-10.952503	6.46E-28	9.54E-27
ENSG00000052723	SIKE1	840.8383351	1073.6401	608.0366006	-0.820489364	0.079664788	-10.299273	7.10E-25	9.51E-24
ENSG00000206573	THUMPD3-AS1	126.3467168	161.44162	91.25181389	-0.821025826	0.225337735	-3.6435346	0.000269	0.000956
ENSG00000169247	SH3TC2	127.9264166	163.27571	92.57712671	-0.821104715	0.217409366	-3.7767679	0.000159	0.000586
ENSG00000184005	ST6GALNAC3	755.5054962	964.89632	546.1146722	-0.821275143	0.092542889	-8.8745354	7.02E-19	7.49E-18
ENSG00000186106	ANKRD46	293.2525329	374.61735	211.8877156	-0.821295708	0.134448063	-6.1086466	1.00E-09	6.44E-09
ENSG00000153885	KCTD15	1476.746738	1886.2923	1067.201171	-0.821892507	0.060969283	-13.480436	2.04E-41	4.19E-40
ENSG00000170915	PAQR8	490.0797561	625.99457	354.1649423	-0.822488456	0.11066361	-7.4323299	1.07E-13	8.94E-13
ENSG00000057657	PRDM1	242.5461562	309.93552	175.1567952	-0.822886302	0.173101483	-4.7537796	2.00E-06	9.48E-06
ENSG00000110237	ARHGEF17	4032.333928	5155.7633	2908.904593	-0.825304961	0.044346092	-18.610545	2.64E-77	9.68E-76
ENSG00000255310	AF131215.5	63.80514344	81.596748	46.01353897	-0.825593632	0.273103424	-3.0230073	0.002503	0.007395
ENSG00000248334	WHAMMP2	314.5088217	402.28166	226.7359843	-0.826443936	0.133455343	-6.1926628	5.92E-10	3.87E-09
ENSG00000197119	SLC25A29	2004.382193	2563.7785	1444.985908	-0.827002979	0.141927149	-5.8269541	5.64E-09	3.40E-08
ENSG00000259865	AL390728.6	82.1178569	105.05317	59.18254748	-0.827409125	0.268396084	-3.0827913	0.002051	0.006174
ENSG00000176533	GNG7	56.24191367	71.975373	40.50845478	-0.828741791	0.326645049	-2.5371326	0.011176	0.028494

ENSG00000167992	VWCE	1433.863	1834.7787	1032.947299	-0.828924501	0.061736098	-13.426902	4.21E-41	8.62E-40
ENSG00000025434	NR1H3	76.58213289	98.150874	55.01339146	-0.82895576	0.273328177	-3.0328222	0.002423	0.007185
ENSG00000154639	CXADR	781.8164599	1000.7151	562.9177965	-0.831640152	0.098168085	-8.4715939	2.42E-17	2.42E-16
ENSG00000165804	ZNF219	795.0128419	1018.0996	571.9261032	-0.831890199	0.082374819	-10.098841	5.59E-24	7.24E-23
ENSG00000128709	HOXD9	483.371303	619.07337	347.6692325	-0.832407005	0.107910384	-7.7138731	1.22E-14	1.07E-13
ENSG00000145911	N4BP3	3438.118986	4403.3563	2472.881678	-0.832515563	0.062899391	-13.23567	5.46E-40	1.09E-38
ENSG00000099337	KCNK6	668.4451261	856.15617	480.7340835	-0.833585334	0.101797235	-8.1886834	2.64E-16	2.51E-15
ENSG00000227671	AL390728.4	472.8306672	606.19868	339.4626563	-0.835950547	0.297114981	-2.8135591	0.0049	0.013561
ENSG00000138119	MYOF	29623.3404	37973.009	21273.67157	-0.835951365	0.034339548	-24.343691	6.76E-131	4.26E-129
ENSG00000100034	PPM1F	7494.596264	9608.0107	5381.181794	-0.836230559	0.034079871	-24.537374	5.90E-133	3.83E-131
ENSG00000171320	ESCO2	147.4411186	189.01197	105.8702721	-0.837061957	0.208173599	-4.0209804	5.80E-05	0.000229
ENSG00000239857	GET4	112.5190385	144.34239	80.69568605	-0.837420467	0.237177504	-3.5307753	0.000414	0.001427
ENSG00000136111	TBC1D4	4013.430061	5150.0632	2876.796942	-0.840358356	0.05291754	-15.880526	8.65E-57	2.38E-55
ENSG00000213366	GSTM2	38.10239903	48.892577	27.31222068	-0.84088569	0.353649825	-2.3777353	0.017419	0.042402
ENSG00000196476	C20orf96	482.716088	619.5141	345.9180786	-0.841062902	0.114786711	-7.3271801	2.35E-13	1.93E-12
ENSG00000181513	ACBD4	135.5842752	174.13659	97.03195785	-0.841692769	0.211397974	-3.9815555	6.85E-05	0.000268
ENSG00000171703	TCEA2	1071.605063	1375.5341	767.6760116	-0.841961131	0.074724727	-11.267504	1.90E-29	2.96E-28
ENSG00000144283	PKP4	1919.565639	2465.0193	1374.111942	-0.842191954	0.061825137	-13.622161	2.96E-42	6.23E-41
ENSG00000175274	TP53I11	8200.672265	10528.968	5872.376698	-0.842345075	0.033695688	-24.998601	6.33E-138	4.25E-136
ENSG00000091127	PUS7	878.7009569	1128.8863	628.515586	-0.843588658	0.082239027	-10.257766	1.09E-24	1.46E-23
ENSG00000106025	TSPAN12	1015.91389	1304.6938	727.1339538	-0.843676118	0.077487077	-10.887959	1.32E-27	1.93E-26
ENSG00000258634	AL160006.1	184.5735097	237.24628	131.9007429	-0.844913073	0.181684474	-4.6504418	3.31E-06	1.53E-05
ENSG00000232593	KANTR	90.97366722	116.8389	65.108438	-0.845227678	0.248771482	-3.3976068	0.00068	0.002257
ENSG00000142731	PLK4	170.4647554	219.20403	121.7254799	-0.845326604	0.175173403	-4.8256561	1.40E-06	6.75E-06
ENSG00000117461	PIK3R3	661.0537036	849.38116	472.7262447	-0.845948905	0.105275027	-8.0356086	9.31E-16	8.63E-15
ENSG00000167972	ABCA3	6440.336535	8276.8298	4603.843252	-0.846251095	0.043765264	-19.336136	2.67E-83	1.03E-81
ENSG00000137312	FLOT1	1347.832953	1732.6165	963.0494131	-0.848114682	0.067328598	-12.596648	2.20E-36	4.09E-35
ENSG00000144040	SFXN5	504.9594315	649.49271	360.4261503	-0.849409424	0.115176887	-7.3748254	1.65E-13	1.36E-12
ENSG00000146918	NCAPG2	639.330302	822.77719	455.8834164	-0.849621143	0.097417627	-8.7214314	2.75E-18	2.86E-17
ENSG00000138771	SHROOM3	127.6194757	164.14357	91.09538123	-0.850364692	0.195598965	-4.3474908	1.38E-05	5.90E-05
ENSG00000153208	MERTK	1848.777273	2378.5951	1318.959405	-0.850414112	0.060293369	-14.104604	3.56E-45	7.95E-44
ENSG00000092470	WDR76	266.2275675	342.77946	189.6756706	-0.850490655	0.14531281	-5.8528264	4.83E-09	2.93E-08
ENSG00000239779	WBP1	114.244614	147.04212	81.44710847	-0.851129335	0.213500144	-3.9865516	6.70E-05	0.000263
ENSG00000124343	XG	48.50094954	62.477633	34.52426564	-0.851965265	0.34301928	-2.4837241	0.013002	0.032722
ENSG00000181035	SLC25A42	198.2481167	255.20162	141.2946157	-0.852657628	0.158581255	-5.376787	7.58E-08	4.13E-07
ENSG00000161551	ZNF577	45.84385955	59.0237	32.66401869	-0.853370306	0.335396998	-2.5443588	0.010948	0.027978
ENSG00000132199	ENOSF1	436.4051025	562.18993	310.6202705	-0.853526135	0.12401595	-6.88239	5.89E-12	4.43E-11
ENSG00000128710	HOXD10	107.6761846	138.68006	76.67231196	-0.853858776	0.21480433	-3.9750538	7.04E-05	0.000275
ENSG00000149476	TKFC	611.1170525	787.15204	435.0820669	-0.854387775	0.094134106	-9.0762829	1.12E-19	1.24E-18
ENSG00000197535	MYO5A	12581.54426	16203.033	8960.055579	-0.854971359	0.036846585	-23.203544	4.19E-119	2.38E-117
ENSG00000170271	FAXDC2	563.8333319	726.4291	401.2375675	-0.855266885	0.100331592	-8.5244026	1.54E-17	1.54E-16
ENSG00000196159	FAT4	3069.587015	3954.0414	2185.132616	-0.855356314	0.053828311	-15.890454	7.38E-57	2.03E-55
ENSG00000105287	PRKD2	3594.678856	4630.6241	2558.733568	-0.855395251	0.046002573	-18.594509	3.56E-77	1.30E-75
ENSG00000115756	HPCAL1	2203.699365	2839.3027	1568.095984	-0.855612368	0.059873042	-14.290444	2.51E-46	5.73E-45
ENSG00000172716	SLFN11	1778.771032	2292.0041	1265.537957	-0.856068774	0.066998844	-12.777366	2.19E-37	4.17E-36
ENSG00000171115	GIMAP8	4704.567803	6060.7076	3348.427958	-0.856360952	0.047038671	-18.205466	4.67E-74	1.64E-72
ENSG00000268362	AC092279.1	45.20842551	58.276605	32.14024561	-0.856873945	0.330023227	-2.596405	0.00942	0.024427
ENSG00000155085	AK9	147.2226142	189.76494	104.6802926	-0.857792179	0.18261635	-4.6972365	2.64E-06	1.23E-05
ENSG00000107282	APBA1	986.1325616	1270.9075	701.3575747	-0.858066093	0.073009987	-11.752722	6.84E-32	1.15E-30
ENSG00000173221	GLRX	365.6025402	471.46857	259.7365079	-0.858144945	0.123408511	-6.9536934	3.56E-12	2.72E-11
ENSG00000183018	AC118754.1	4595.406221	5924.2277	3266.584702	-0.858555766	0.044865045	-19.136407	1.26E-81	4.78E-80
ENSG00000136720	HS6ST1	734.1530287	946.20839	522.0976638	-0.858582393	0.083604489	-10.269573	9.66E-25	1.29E-23
ENSG00000130787	HIP1R	4637.835226	5979.6113	3296.059135	-0.858748495	0.054514518	-15.752657	6.59E-56	1.78E-54
ENSG00000274602	PI4KAP1	67.23391691	86.631722	47.83611171	-0.858951951	0.281779782	-3.0483094	0.002301	0.006857
ENSG00000170962	PDGFD	984.8973963	1269.7535	700.0413325	-0.860041499	0.08202698	-10.484861	1.01E-25	1.41E-24
ENSG00000250510	GPR162	101.658567	131.16576	72.15136972	-0.860098321	0.227369503	-3.7828218	0.000155	0.000573
ENSG00000120802	TMPO	1325.023017	1708.6293	941.4167329	-0.860256711	0.066367694	-12.96198	2.01E-38	3.89E-37
ENSG00000100068	LRP5L	52.03042788	67.047644	37.01321154	-0.860491349	0.356769397	-2.4118979	0.01587	0.03909
ENSG00000106780	MEGF9	2281.442908	2942.5182	1620.367621	-0.861223426	0.05863991	-14.686643	7.85E-49	1.88E-47
ENSG00000101265	RASSF2	2416.456004	3116.6106	1716.301406	-0.861286806	0.065619848	-13.125401	2.36E-39	4.64E-38
ENSG00000139211	AMIGO2	522.8523863	675.02812	370.6766557	-0.864534911	0.1039774	-8.3146425	9.20E-17	8.93E-16
ENSG00000147813	NAPRT	560.2119142	723.44872	396.9751101	-0.864951851	0.101324587	-8.5364458	1.38E-17	1.39E-16
ENSG00000138435	CHRNA1	446.2938872	576.25201	316.3357636	-0.865488296	0.143982196	-6.0110786	1.84E-09	1.16E-08
ENSG00000263072	ZNF213-AS1	74.5668716	96.324097	52.80964572	-0.866496403	0.259955405	-3.3332502	0.000858	0.002788
ENSG00000075618	FSCN1	19292.02157	24923.298	13660.74535	-0.867443399	0.028081827	-30.889849	1.63E-209	1.94E-207
ENSG00000128815	WDFY4	199.2514289	257.59547	140.9073872	-0.869151087	0.183297682	-4.7417462	2.12E-06	1.00E-05
ENSG00000101412	E2F1	225.2126827	291.42717	158.9981969	-0.869972103	0.161442519	-5.3887421	7.10E-08	3.88E-07
ENSG00000230989	HSBP1	5137.099574	6642.4457	3631.753474	-0.871295359	0.048389296	-18.005953	1.75E-72	6.06E-71
ENSG00000171604	CXXC5	209.6074494	271.2731	147.9417941	-0.871453441	0.179803618	-4.8466958	1.26E-06	6.10E-06
ENSG00000144642	RBMS3	820.0031119	1060.166	579.8402603	-0.87195441	0.0888863	-9.8097729	1.02E-22	1.27E-21
ENSG00000204228	HSD17B8	38.6605156	50.043065	27.27796579	-0.872023796	0.350893489	-2.4851524	0.01295	0.032599
ENSG00000088305	DNMT3B	102.1042298	132.03575	72.17270655	-0.872577971	0.254219447	-3.4323809	0.000598	0.002007
ENSG00000101057	MYBL2	745.4536444	964.63463	526.2726618	-0.874954591	0.10056541	-8.7003532	3.31E-18	3.44E-17
ENSG00000132613	MTSS1L	2106.007663	2726.0105	1486.00485	-0.875000054	0.059799801	-14.632157	1.75E-48	4.18E-47
ENSG00000093009	CDC45	144.9995061	187.65702	102.3419967	-0.875182044	0.187112064	-4.6773149	2.91E-06	1.36E-05
ENSG00000185201	IFITM2	3590.259375	4648.0757	2532.443068	-0.87584492	0.056949642	-15.379288	2.25E-53	5.86E-52
ENSG00000198719	DLL1	82.444846	106.70975	58.17994232	-0.875959545	0.289596247	-3.0247614	0.002488	0.007357
ENSG00000206337	HCP5	148.9929486	193.07796	104.9079419	-0.876604614	0.202191923	-4.3355076	1.45E-05	6.21E-05
ENSG00000213983	AP1G2	564.090912	730.65575	397.5260734	-0.877014556	0.105730844	-8.2947844	1.09E-16	1.05E-15
ENSG00000130052	STARD8	556.2035258	720.34544	392.0616078	-0.877064934	0.107065724	-8.1918368	2.57E-16	2.45E-15
ENSG00000117791	2-Mar	199.4944751	258.41174	140.5772107	-0.877335811	0.162133404	-5.4111971	6.26E-08	3.43E-07
ENSG00000158555	GDPD5	1464.323412	1897.0291	1031.617758	-0.877821769	0.070071825	-12.527457	5.28E-36	9.73E-35
ENSG00000003096	KLHL13	322.9521778	418.36643	227.5379283	-0.879266301	0.12496443	-7.0361326	1.98E-12	1.53E-11
ENSG00000094804	CDC6	262.6906137	340.61007	184.7711567	-0.879282038	0.157129761	-5.5958975	2.19E-08	1.26E-07
ENSG00000140451	PIF1	88.79036336	115.14661	62.43411223	-0.879777817	0.237611105	-3.7025955	0.000213	0.000771

ENSG00000104368	PLAT	1135.933236	1472.0474	799.8190349	-0.879875784	0.202571773	-4.3435261	1.40E-05	6.00E-05
ENSG00000043462	LCP2	78.41980285	101.72321	55.1163936	-0.88215779	0.26476149	-3.3318962	0.000863	0.002799
ENSG00000176809	LRRC37A3	121.6496532	157.86257	85.436738	-0.884285078	0.205183372	-4.3097307	1.63E-05	6.94E-05
ENSG00000126460	PRRG2	54.56025332	70.869026	38.25148062	-0.88601843	0.301848743	-2.935306	0.003332	0.009595
ENSG00000136161	RCBTB2	417.2921681	541.67731	292.9070238	-0.886106282	0.134451239	-6.5905401	4.38E-11	3.12E-10
ENSG00000072210	ALDH3A2	1365.514904	1772.5051	958.5247181	-0.886781028	0.067948461	-13.050789	6.29E-39	1.23E-37
ENSG00000166803	PCLAF	79.91745859	103.79484	56.04007573	-0.886807228	0.247177604	-3.5877329	0.000334	0.001165
ENSG00000213398	LCAT	146.1494848	189.99972	102.2992523	-0.889376651	0.234770387	-3.7882829	0.000152	0.000561
ENSG00000006468	ETV1	239.0645256	310.42452	167.7045265	-0.889514034	0.143780742	-6.1866007	6.15E-10	4.02E-09
ENSG00000205476	CCDC85C	1024.938264	1331.6341	718.242435	-0.889915796	0.080650374	-11.034243	2.61E-28	3.90E-27
ENSG00000172031	EPHX4	499.1544151	648.21987	350.0889562	-0.890282623	0.109600868	-8.1229523	4.55E-16	4.27E-15
ENSG00000167549	CORO6	96.0373809	124.8899	67.18486501	-0.890531059	0.296367091	-3.0048244	0.002657	0.007809
ENSG00000118292	C1orf54	216.8381105	281.73124	151.9449833	-0.890971486	0.151572367	-5.8781921	4.15E-09	2.53E-08
ENSG00000168661	ZNF30	113.3307696	147.21947	79.44206486	-0.891390463	0.222347842	-4.0089908	6.10E-05	0.00024
ENSG00000163535	SGO2	333.8415111	434.03629	233.6467292	-0.89141305	0.129433576	-6.887031	5.70E-12	4.29E-11
ENSG00000119943	PYROXD2	122.6518952	159.58645	85.71734291	-0.893303126	0.203930253	-4.3804345	1.18E-05	5.13E-05
ENSG00000278864	AC055811.4	50.28045922	65.352054	35.20886453	-0.893880162	0.333948284	-2.6767024	0.007435	0.019773
ENSG00000182492	BGN	11725.77384	15248.456	8203.091178	-0.894127896	0.040512547	-22.070394	6.09E-108	3.08E-106
ENSG00000154928	EPHB1	610.2582308	793.79699	426.7194737	-0.894218277	0.106363976	-8.4071535	4.20E-17	4.14E-16
ENSG00000152284	TCF7L1	1492.129135	1940.4483	1043.809952	-0.894280062	0.071221603	-12.556303	3.67E-36	6.79E-35
ENSG00000113719	ERGIC1	7793.993679	10147.594	5440.393513	-0.899379451	0.036499035	-24.641184	4.57E-134	3.02E-132
ENSG00000134057	CCNB1	704.4927147	917.36965	491.6157812	-0.900092193	0.08614667	-10.448369	1.49E-25	2.05E-24
ENSG00000175104	TRAF6	816.7626152	1063.8427	569.6825574	-0.900783283	0.079342583	-11.353087	7.16E-30	1.13E-28
ENSG00000166707	ZCCHC18	35.6222515	46.387484	24.85701935	-0.903765378	0.380407704	-2.375781	0.017512	0.042593
ENSG00000162599	NFIA	751.1950912	979.1391	523.2510857	-0.905124718	0.086856379	-10.420935	1.99E-25	2.73E-24
ENSG00000072952	MRVI1	98.33710248	128.34531	68.32889433	-0.90622912	0.241459568	-3.7531299	0.000175	0.000639
ENSG00000163762	TM4SF18	7982.894848	10410.347	5555.442536	-0.906259683	0.038911216	-23.290448	5.54E-120	3.17E-118
ENSG00000047617	ANO2	283.7019617	370.0668	197.3371255	-0.907570152	0.14584494	-6.2228429	4.88E-10	3.21E-09
ENSG00000188234	AGAP4	88.85241441	116.04909	61.65573707	-0.908218155	0.309805545	-2.9315749	0.003372	0.009696
ENSG00000182575	NXPH3	181.417599	236.70784	126.1273554	-0.910146632	0.181862191	-5.0045951	5.60E-07	2.82E-06
ENSG00000146263	MMS22L	295.7193421	385.74215	205.6965369	-0.910711022	0.142539928	-6.3891643	1.67E-10	1.14E-09
ENSG00000080298	RFX3	519.3874033	677.85237	360.9224364	-0.910996497	0.104574955	-8.7114214	3.00E-18	3.12E-17
ENSG00000047346	FAM214A	2356.645393	3077.2963	1635.994456	-0.911588805	0.056833909	-16.039523	6.77E-58	1.89E-56
ENSG00000182796	TMEM198B	290.4906049	379.47324	201.5079678	-0.911995963	0.158830132	-5.7419581	9.36E-09	5.53E-08
ENSG00000153094	BCL2L11	2241.548906	2927.5013	1555.596495	-0.912335684	0.067124876	-13.591618	4.49E-42	9.40E-41
ENSG00000196295	GARS-DT	173.0089903	226.03344	119.9845371	-0.913082639	0.16844904	-5.4205274	5.94E-08	3.26E-07
ENSG00000242125	SNHG3	283.1284035	369.91847	196.3383324	-0.913709597	0.165003374	-5.5375207	3.07E-08	1.73E-07
ENSG00000088325	TPX2	1194.56794	1561.1436	827.9922459	-0.914213308	0.076501576	-11.950255	6.47E-33	1.11E-31
ENSG00000175305	CCNE2	227.6053852	297.4293	157.7814668	-0.914754542	0.157091262	-5.8230772	5.78E-09	3.48E-08
ENSG00000197989	SNHG12	116.8421538	152.80858	80.87572287	-0.914777703	0.21578729	-4.2392566	2.24E-05	9.39E-05
ENSG00000140400	MAN2C1	1539.862425	2012.6689	1067.055911	-0.915248843	0.171935319	-5.323216	1.02E-07	5.49E-07
ENSG00000206560	ANKRD28	2240.191176	2927.7849	1552.597491	-0.916089779	0.057019201	-16.066338	4.39E-58	1.23E-56
ENSG00000268573	AC011815.1	32.33263954	42.321249	22.34402972	-0.916566039	0.395631248	-2.316718	0.020519	0.048951
ENSG00000230551	AC021078.1	192.5026493	251.66661	133.338688	-0.916779079	0.168604282	-5.4374602	5.40E-08	2.98E-07
ENSG00000138658	ZGRF1	92.52158484	120.89777	64.1453954	-0.917172905	0.248276772	-3.6941551	0.000221	0.000795
ENSG00000259366	AC108449.2	60.37489928	78.940418	41.80938026	-0.917812049	0.301599611	-3.0431473	0.002341	0.006968
ENSG00000183323	CCDC125	281.0815676	367.47707	194.6860603	-0.919415288	0.144002839	-6.3847025	1.72E-10	1.17E-09
ENSG00000152642	GPD1L	606.4304446	793.62585	419.2350405	-0.921290944	0.091865924	-10.028647	1.14E-23	1.46E-22
ENSG00000097096	SYDE2	45.97755505	60.198234	31.75687604	-0.92132744	0.322560917	-2.8562897	0.004286	0.012004
ENSG00000110328	GALNT18	407.2262022	533.19403	281.2583735	-0.922012548	0.117785429	-7.8278999	4.96E-15	4.44E-14
ENSG00000137807	KIF23	496.38797	649.94114	342.836803	-0.922133385	0.108197818	-8.5226615	1.56E-17	1.57E-16
ENSG00000224660	SH3BP5-AS1	60.30045385	78.929259	41.67164828	-0.922654813	0.316311966	-2.916914	0.003535	0.010111
ENSG00000134222	PSRC1	93.8512481	123.08844	64.6140517	-0.92299281	0.257154525	-3.5892536	0.000332	0.001159
ENSG00000079482	OPHN1	507.4228041	664.30119	350.5444216	-0.923517496	0.105874862	-8.7227268	2.72E-18	2.83E-17
ENSG00000142875	PRKACB	3574.225829	4681.113	2467.338633	-0.92421949	0.047948895	-19.275095	8.69E-83	3.34E-81
ENSG00000165511	C10orf25	63.55518223	83.266316	43.84404813	-0.925886513	0.272985151	-3.3917102	0.000695	0.002302
ENSG00000111077	TNS2	1897.547907	2487.1484	1307.947397	-0.926807765	0.079043049	-11.725354	9.45E-32	1.58E-30
ENSG00000197978	GOLGA6L9	37.08835122	48.578947	25.59775566	-0.926834825	0.373188109	-2.4835594	0.013008	0.032731
ENSG00000167702	KIFC2	336.7979137	441.64362	231.9522116	-0.927169084	0.172441094	-5.3767293	7.59E-08	4.13E-07
ENSG00000180998	GPR137C	78.52039639	102.92461	54.11618711	-0.927607676	0.267563018	-3.4668755	0.000527	0.001781
ENSG00000120075	HOXB5	92.5005893	121.3339	63.66727574	-0.927632014	0.240576046	-3.8558785	0.000115	0.000435
ENSG00000154839	SKA1	79.17745125	103.64301	54.71188929	-0.927832803	0.261255979	-3.5514319	0.000383	0.001326
ENSG00000141519	CCDC40	100.9288713	132.3202	69.53753915	-0.929069815	0.232601254	-3.9942597	6.49E-05	0.000255
ENSG00000198691	ABCA4	264.3761645	346.88397	181.868363	-0.929104842	0.157829754	-5.8867534	3.94E-09	2.41E-08
ENSG00000166881	NEMP1	610.9521936	801.28851	420.6158743	-0.929779529	0.099157824	-9.3767641	6.80E-21	7.93E-20
ENSG00000154930	ACSS1	834.7656749	1095.037	574.4943864	-0.930156884	0.103232171	-9.0103392	2.05E-19	2.24E-18
ENSG00000205403	CFI	87.47481181	114.8672	60.08242336	-0.930744544	0.239660428	-3.8835971	0.000103	0.000391
ENSG00000162804	SNED1	2220.642785	2915.1117	1526.173866	-0.933513297	0.054214898	-17.21876	1.92E-66	6.13E-65
ENSG00000222009	BTBD19	328.213923	431.13374	225.2941097	-0.933803572	0.157050262	-5.9458899	2.75E-09	1.70E-08
ENSG00000135636	DYSF	30425.35521	39958.997	20891.71377	-0.935577394	0.028382384	-32.963312	2.73E-238	3.94E-236
ENSG00000179144	GIMAP7	2442.606103	3208.8865	1676.325734	-0.936124052	0.054420274	-17.201752	2.58E-66	8.19E-65
ENSG00000151967	SCHIP1	169.7122706	222.98769	116.4368486	-0.936542068	0.170003761	-5.5089491	3.61E-08	2.03E-07
ENSG00000278921	EPB41L4A-DT	38.29900687	50.256613	26.34140098	-0.936733503	0.361470479	-2.5914523	0.009557	0.02474
ENSG00000124785	NRN1	991.5891111	1303.0698	680.1083954	-0.939379507	0.07744495	-12.129642	7.36E-34	1.29E-32
ENSG00000138160	KIF11	463.0648746	609.03063	317.0991188	-0.939993257	0.106878951	-8.7949334	1.43E-18	1.52E-17
ENSG00000277476	AC005332.5	68.07295844	89.582127	46.56378984	-0.940948195	0.287941674	-3.267843	0.001084	0.003455
ENSG00000198185	ZNF334	36.69480571	48.331183	25.05842837	-0.941111403	0.383794196	-2.4521252	0.014202	0.035417
ENSG00000161010	MRNIP	221.6689277	291.73394	151.6039186	-0.941299275	0.156382815	-6.0191989	1.75E-09	1.10E-08
ENSG00000172159	FRMD3	53.5482747	70.366139	36.73041005	-0.941665226	0.353965479	-2.6603307	0.007806	0.02067
ENSG00000103168	TAF1C	808.9620464	1064.787	553.1370641	-0.944054799	0.112716098	-8.37551	5.50E-17	5.40E-16
ENSG00000154529	CNTNAP3B	526.9618922	693.76112	360.1626659	-0.944294348	0.116983236	-8.0720485	6.91E-16	6.43E-15
ENSG00000163092	XIRP2	472.5908575	621.93318	323.2485383	-0.944301557	0.111408812	-8.4760042	2.33E-17	2.33E-16
ENSG00000205090	TMEM240	57.05707169	75.093015	39.02112877	-0.94881959	0.301682878	-3.1450893	0.00166	0.005098
ENSG00000171163	ZNF692	223.5436092	294.64075	152.4464697	-0.950066276	0.364981648	-2.6031079	0.009238	0.024014
ENSG00000152953	STK32B	2315.46385	3051.5316	1579.396115	-0.950584307	0.063076561	-15.070325	2.54E-51	6.34E-50

ENSG00000129422	MTUS1	11013.77276	14519.295	7508.250982	-0.951575339	0.031083016	-30.613997	7.97E-206	9.30E-204
ENSG00000231298	MANCR	815.5918402	1075.6879	555.4958291	-0.95213671	0.092051461	-10.343526	4.48E-25	6.06E-24
ENSG00000182253	SYNM	1717.326878	2265.5696	1169.084176	-0.954951002	0.059568192	-16.031223	7.73E-58	2.15E-56
ENSG00000116774	OLFML3	396.7506988	523.52876	269.972635	-0.955867393	0.120363889	-7.9414798	2.00E-15	1.82E-14
ENSG00000134323	MYCN	186.7542798	246.32561	127.1829515	-0.956125183	0.174073425	-5.4926545	3.96E-08	2.21E-07
ENSG00000164929	BAALC	75.62954417	99.869672	51.3894161	-0.956499765	0.273613974	-3.4958001	0.000473	0.001616
ENSG00000121152	NCAPH	192.6906845	254.37468	131.0066851	-0.956528755	0.175482078	-5.4508629	5.01E-08	2.77E-07
ENSG00000175198	PCCA	394.0463484	519.9307	268.161995	-0.956547183	0.120663527	-7.9273929	2.24E-15	2.03E-14
ENSG00000118922	KLF12	654.3805253	863.65634	445.1047094	-0.956805263	0.093685897	-10.212906	1.74E-24	2.30E-23
ENSG00000182118	FAM89A	603.4085231	796.7832	410.0338497	-0.958142783	0.121085775	-7.912926	2.51E-15	2.28E-14
ENSG00000169291	SHE	4638.352538	6123.8102	3152.894834	-0.958208645	0.043911523	-21.821349	1.46E-105	7.15E-104
ENSG00000185522	LMNTD2	56.86064139	75.00409	38.71719255	-0.958474595	0.306578944	-3.1263549	0.00177	0.005403
ENSG00000151491	EPS8	725.8857932	958.54174	493.2298448	-0.959075582	0.085837131	-11.173202	5.52E-29	8.43E-28
ENSG00000182568	SATB1	313.4435164	414.02606	212.8609694	-0.95926797	0.12979177	-7.3908228	1.46E-13	1.21E-12
ENSG00000121621	KIF18A	89.03783307	117.53849	60.53717634	-0.960273717	0.243826376	-3.9383505	8.20E-05	0.000317
ENSG00000085840	ORC1	87.78914389	116.07817	59.50011418	-0.960505724	0.280049885	-3.4297665	0.000604	0.002024
ENSG00000078018	MAP2	1553.85449	2053.1227	1054.586272	-0.960839594	0.070277245	-13.67213	1.49E-42	3.16E-41
ENSG00000146281	PM20D2	176.7828807	233.66624	119.8995232	-0.962322974	0.179363111	-5.3652224	8.08E-08	4.39E-07
ENSG00000152056	AP1S3	38.37076852	50.815291	25.92624594	-0.962891359	0.390878283	-2.4634046	0.013762	0.034436
ENSG00000112039	FANCE	139.4604379	184.28267	94.63820162	-0.964086907	0.196628397	-4.9030909	9.43E-07	4.65E-06
ENSG00000196584	XRCC2	101.0358885	133.56119	68.51058202	-0.96531097	0.231336368	-4.1727592	3.01E-05	0.000124
ENSG00000084710	EFR3B	58.11886605	76.94374	39.29399195	-0.966106301	0.324582339	-2.9764599	0.002916	0.008492
ENSG00000162066	AMDHD2	897.4276349	1188.0697	606.7855549	-0.968504853	0.097970304	-9.8856982	4.80E-23	6.03E-22
ENSG00000235770	LINC00607	1606.647437	2128.2133	1085.081607	-0.970987067	0.064332146	-15.093342	1.79E-51	4.50E-50
ENSG00000162981	FAM84A	84.45440407	111.90549	57.00332044	-0.97255162	0.279343337	-3.4815637	0.000498	0.001694
ENSG00000151892	GFRA1	515.2827737	682.72288	347.8426682	-0.972698868	0.11274579	-8.6273631	6.28E-18	6.44E-17
ENSG00000173275	ZNF449	355.6676481	471.60401	239.7312844	-0.973849523	0.13017918	-7.4808393	7.38E-14	6.25E-13
ENSG00000182511	FES	1331.330572	1765.265	897.3961734	-0.975280842	0.064684923	-15.077406	2.28E-51	5.71E-50
ENSG00000083807	SLC27A5	41.81241178	55.413	28.2118237	-0.975483507	0.348910588	-2.795798	0.005177	0.014245
ENSG00000265972	TXNIP	3118.065036	4134.1893	2101.940799	-0.9759191	0.060401571	-16.157181	1.01E-58	2.90E-57
ENSG00000198336	MYL4	63.18549454	83.656627	42.71436171	-0.976052863	0.298482249	-3.2700533	0.001075	0.003431
ENSG00000175356	SCUBE2	59.13160207	78.289754	39.97344989	-0.976244985	0.311339626	-3.1356272	0.001715	0.005251
ENSG00000143409	MINDY1	626.1745838	830.61686	421.7323092	-0.976886252	0.092398314	-10.572555	3.99E-26	5.66E-25
ENSG00000176485	PLA2G16	962.5051403	1276.6836	648.3266433	-0.976992554	0.076738631	-12.731431	3.96E-37	7.48E-36
ENSG00000108773	KAT2A	841.6184331	1118.0566	565.1803158	-0.982399126	0.093287464	-10.53088	6.22E-26	8.73E-25
ENSG00000204131	NHSL2	2059.45208	2735.0701	1383.834056	-0.98333207	0.062656454	-15.694027	1.66E-55	4.46E-54
ENSG00000136235	GPNMB	967.5971534	1285.7286	649.4657086	-0.985100201	0.195468731	-5.0396818	4.66E-07	2.37E-06
ENSG00000107551	RASSF4	306.9130956	408.05607	205.7701169	-0.986172039	0.1588132	-6.2096352	5.31E-10	3.49E-09
ENSG00000153933	DGKE	384.062048	510.86879	257.2553037	-0.986905796	0.120515815	-8.1890148	2.63E-16	2.50E-15
ENSG00000112297	CRYBG1	3722.604345	4948.4835	2496.725155	-0.987145145	0.053148897	-18.573201	5.30E-77	1.93E-75
ENSG00000137841	PLCB2	47.68272413	63.302	32.06344841	-0.988252799	0.343503921	-2.8769768	0.004015	0.011337
ENSG00000255031	AP002807.1	50.17555807	66.702955	33.64816065	-0.988333961	0.353121266	-2.7988514	0.005128	0.014135
ENSG00000125945	ZNF436	1065.070588	1415.7563	714.3848977	-0.98838483	0.07846802	-12.596021	2.22E-36	4.12E-35
ENSG00000181450	ZNF678	177.3722025	236.07199	118.6724151	-0.992022707	0.175624887	-5.6485315	1.62E-08	9.37E-08
ENSG00000167693	NXN	2564.580946	3414.7774	1714.384497	-0.993602535	0.049432056	-20.100368	7.32E-90	3.07E-88
ENSG00000121957	GPSM2	249.3402256	332.14521	166.5352421	-0.993971664	0.141776233	-7.0108483	2.37E-12	1.82E-11
ENSG00000147883	CDKN2B	800.875807	1066.3147	535.4369376	-0.994056622	0.096266415	-10.3261	5.37E-25	7.24E-24
ENSG00000173598	NUDT4	5140.49387	6844.9831	3436.004646	-0.99470511	0.045481367	-21.870607	4.95E-106	2.46E-104
ENSG00000196550	FAM72A	31.51277347	41.99878	21.02676685	-0.994920122	0.430999148	-2.3084039	0.020977	0.049912
ENSG00000134690	CDCA8	310.9796806	414.32291	207.6364547	-0.997881622	0.134698789	-7.4082449	1.28E-13	1.07E-12
ENSG00000171241	SHCBP1	303.6909146	404.69294	202.6888881	-0.998639354	0.132157568	-7.5564296	4.14E-14	3.54E-13
ENSG00000105974	CAV1	80320.14384	107082.41	53557.88118	-0.999582323	0.033034932	-30.258343	4.05E-201	4.61E-199
ENSG00000137225	CAPN11	155.1450763	206.87389	103.4162581	-0.999809021	0.209196922	-4.7792721	1.76E-06	8.40E-06
ENSG00000177990	DPY19L2	75.68297574	101.05107	50.31488183	-1.001401234	0.300254288	-3.3351771	0.000852	0.00277
ENSG00000154874	CCDC144B	80.45448577	107.46183	53.44714242	-1.003177311	0.264070786	-3.7988955	0.000145	0.000539
ENSG00000138346	DNA2	90.3679978	120.62977	60.10622962	-1.004024068	0.238197495	-4.2150908	2.50E-05	0.000104
ENSG00000137507	LRRC32	6879.235969	9181.8888	4576.583176	-1.00418975	0.036988165	-27.148948	2.61E-162	2.17E-160
ENSG00000125510	OPRL1	100.6913051	134.50144	66.88116626	-1.005086021	0.237950588	-4.2239274	2.40E-05	0.0001
ENSG00000079616	KIF22	500.555327	668.23144	332.8792156	-1.005332706	0.107055649	-9.3907488	5.96E-21	6.96E-20
ENSG00000076555	ACACB	172.2713943	230.1848	114.3579875	-1.006091541	0.175743176	-5.724783	1.04E-08	6.10E-08
ENSG00000128923	MINDY2	1138.208442	1519.3655	757.0514094	-1.006266612	0.074278633	-13.547188	8.23E-42	1.70E-40
ENSG00000110455	ACCS	205.8743308	275.1295	136.6191645	-1.006621646	0.19351108	-5.2018812	1.97E-07	1.04E-06
ENSG00000170917	NUDT6	82.99935306	110.82378	55.1749297	-1.00680718	0.248726233	-4.0478528	5.17E-05	0.000206
ENSG00000159314	ARHGAP27	1264.664409	1689.1438	840.1850449	-1.007290953	0.070018305	-14.386109	6.33E-47	1.46E-45
ENSG00000130193	THEM6	387.5414251	517.54968	257.5331664	-1.008273861	0.117943728	-8.5487705	1.24E-17	1.26E-16
ENSG00000107611	CUBN	674.1716541	900.8253	447.5180101	-1.00937931	0.092826537	-10.873823	1.54E-27	2.25E-26
ENSG00000134884	ARGLU1	1687.383395	2255.0581	1119.708661	-1.009745976	0.177864172	-5.6770623	1.37E-08	7.98E-08
ENSG00000170214	ADRA1B	71.78493173	95.974521	47.59534222	-1.010054236	0.261589386	-3.8612203	0.000113	0.000427
ENSG00000221978	CCNL2	1808.015919	2416.7193	1199.312561	-1.01070465	0.231999091	-4.3565026	1.32E-05	5.69E-05
ENSG00000141505	ASGR1	130.7407829	174.71181	86.76975717	-1.010746256	0.210264007	-4.8070341	1.53E-06	7.37E-06
ENSG00000103319	EEF2K	2080.182817	2779.9584	1380.407263	-1.010839805	0.056253383	-17.969405	3.38E-72	1.17E-70
ENSG00000068024	HDAC4	391.9719516	524.14575	259.7981551	-1.010850755	0.120340848	-8.3998972	4.47E-17	4.40E-16
ENSG00000162631	NTNG1	80.75854647	107.85569	53.66139876	-1.012757655	0.257873645	-3.9273407	8.59E-05	0.000331
ENSG00000131089	ARHGEF9	455.0572785	608.69795	301.4166103	-1.013614613	0.121013355	-8.3760558	5.47E-17	5.38E-16
ENSG00000161996	WDR90	318.3968788	426.02278	210.7709826	-1.01380878	0.147743523	-6.8619508	6.79E-12	5.10E-11
ENSG00000162616	DNAJB4	2946.50562	3942.2955	1950.715728	-1.015149234	0.046295829	-21.927445	1.42E-106	7.15E-105
ENSG00000136153	LMO7	364.2799361	487.34523	241.2146453	-1.016348219	0.122868735	-8.2718213	1.32E-16	1.27E-15
ENSG00000158352	SHROOM4	4337.031508	5807.074	2866.988995	-1.018632873	0.042637026	-23.890805	3.82E-126	2.28E-124
ENSG00000178878	APOLD1	858.6400679	1149.5142	567.7659355	-1.019438171	0.086857515	-11.736902	8.25E-32	1.38E-30
ENSG00000176595	KBTBD11	219.763608	294.48107	145.0461473	-1.021284135	0.160253229	-6.3729395	1.85E-10	1.26E-09
ENSG00000187244	BCAM	1272.62012	1704.7995	840.4407636	-1.021564243	0.07441299	-13.728305	6.87E-43	1.47E-41
ENSG00000146856	AGBL3	37.46059496	50.278189	24.64300049	-1.021907715	0.366950824	-2.7848628	0.005355	0.014672
ENSG00000100526	CDKN3	250.4233959	335.42233	165.4244585	-1.022068134	0.151917072	-6.727803	1.72E-11	1.26E-10
ENSG00000198435	NRARP	2126.443998	2849.8991	1402.988892	-1.022785689	0.060423976	-16.926819	2.85E-64	8.76E-63
ENSG00000109654	TRIM2	727.3240572	974.45341	480.1947094	-1.023198825	0.091791183	-11.147027	7.40E-29	1.13E-27

ENSG00000011426	ANLN	1215.999796	1629.9156	802.0839414	-1.023334415	0.080233549	-12.754445	2.94E-37	5.59E-36
ENSG00000183090	FREM3	31.22659059	41.834065	20.61911607	-1.023430801	0.391812796	-2.6120403	0.009	0.023469
ENSG00000065923	SLC9A7	421.3629523	564.67957	278.0463359	-1.023684174	0.12302457	-8.3209734	8.72E-17	8.48E-16
ENSG00000132561	MATN2	129.1772558	173.1323	85.22220882	-1.024643792	0.204267022	-5.0161978	5.27E-07	2.66E-06
ENSG00000073584	SMARCE1	145.6225769	195.27386	95.97129153	-1.024772152	0.193961166	-5.2833883	1.27E-07	6.77E-07
ENSG00000075702	WDR62	121.0402925	162.37367	79.70691347	-1.026955685	0.201037547	-5.108278	3.25E-07	1.67E-06
ENSG00000159208	CIART	51.12736019	68.640779	33.61394114	-1.027230174	0.319319406	-3.2169363	0.001296	0.00407
ENSG00000214176	PLEKHM1P1	336.0064762	451.0631	220.9498557	-1.028792728	0.365289233	-2.8163785	0.004857	0.013451
ENSG00000225828	FAM229A	75.39661998	101.28521	49.50803441	-1.029113296	0.271327117	-3.7928877	0.000149	0.000552
ENSG00000142102	PGGHG	891.6684984	1197.1136	586.223444	-1.029617	0.23920143	-4.3043932	1.67E-05	7.11E-05
ENSG00000161888	SPC24	172.9739333	232.25362	113.6942505	-1.029657211	0.178775453	-5.7594999	8.44E-09	5.01E-08
ENSG00000133460	SLC2A11	85.3243129	114.58522	56.06340704	-1.03043998	0.26421837	-3.8999559	9.62E-05	0.000368
ENSG00000231999	LRRC8C-DT	34.7976038	46.655986	22.93922157	-1.030534674	0.388806321	-2.6505091	0.008037	0.021234
ENSG00000176890	TYMS	1476.985265	1982.9758	970.9947255	-1.030580659	0.062866621	-16.39313	2.14E-60	6.27E-59
ENSG00000163590	PPM1L	73.24372456	98.343982	48.14346725	-1.030714863	0.320349113	-3.2174738	0.001293	0.004063
ENSG00000175130	MARCKSL1	10222.68984	13727.732	6717.647657	-1.031209229	0.031864748	-32.36207	9.38E-230	1.30E-227
ENSG00000159388	BTG2	4677.728265	6284.4387	3071.017847	-1.032958198	0.046515559	-22.206724	2.96E-109	1.52E-107
ENSG00000197008	ZNF138	72.08582175	96.845745	47.32589836	-1.033433006	0.289421677	-3.5706828	0.000356	0.001239
ENSG00000138834	MAPK8IP3	1258.622228	1691.131	826.1134169	-1.033435108	0.26551997	-3.8921182	9.94E-05	0.000379
ENSG00000234409	CCDC188	314.5921909	423.01168	206.1726975	-1.034870715	0.143427862	-7.2152698	5.38E-13	4.33E-12
ENSG00000157404	KIT	2256.141661	3032.3693	1479.914037	-1.034908568	0.133072507	-7.7770277	7.42E-15	6.57E-14
ENSG00000123892	RAB38	56.2757715	75.675588	36.87595451	-1.035187315	0.339500692	-3.0491464	0.002295	0.006842
ENSG00000171130	ATP6V0E2	761.2707111	1023.8663	498.6750856	-1.036019975	0.100383662	-10.320603	5.69E-25	7.65E-24
ENSG00000116833	NR5A2	634.619191	853.30076	415.9376202	-1.036180355	0.106325052	-9.7454018	1.93E-22	2.38E-21
ENSG00000105784	RUNDC3B	73.36226399	98.533484	48.1910444	-1.036204391	0.288058367	-3.5972029	0.000322	0.001126
ENSG00000184226	PCDH9	253.5864304	340.96073	166.2121303	-1.036911263	0.152822693	-6.7850608	1.16E-11	8.58E-11
ENSG00000185614	INKA1	434.5959454	584.86973	284.3221597	-1.039077232	0.113878679	-9.124423	7.21E-20	8.03E-19
ENSG00000091879	ANGPT2	37807.00107	50868.229	24745.77331	-1.039530902	0.028800883	-36.093716	2.85E-285	5.03E-283
ENSG00000166436	TRIM66	254.9676595	343.23905	166.69627	-1.040180592	0.16595223	-6.2679519	3.66E-10	2.43E-09
ENSG00000100162	CENPM	92.64287729	124.67956	60.60619644	-1.040363743	0.228824757	-4.5465524	5.45E-06	2.47E-05
ENSG00000143919	CAMKMT	72.25266982	97.174336	47.33100394	-1.040917052	0.266347172	-3.9081213	9.30E-05	0.000357
ENSG00000166432	ZMAT1	54.31305323	73.21049	35.41561661	-1.042444064	0.309036023	-3.3732121	0.000743	0.002445
ENSG00000230373	GOLGA6L5P	46.84250216	63.071172	30.61383199	-1.04250882	0.331075447	-3.1488557	0.001639	0.005037
ENSG00000177595	PIDD1	614.0587368	827.11632	401.0011557	-1.043139691	0.10117913	-10.309831	6.36E-25	8.53E-24
ENSG00000184489	PTP4A3	129.3907691	174.37097	84.41056405	-1.045348976	0.195899219	-5.3361569	9.49E-08	5.12E-07
ENSG00000051180	RAD51	222.9219511	300.43846	145.4054377	-1.045764093	0.150152205	-6.9646935	3.29E-12	2.52E-11
ENSG00000178175	ZNF366	1419.419163	1912.52	926.318326	-1.046532426	0.064553418	-16.211882	4.16E-59	1.20E-57
ENSG00000147862	NFIB	7494.572734	10101.089	4888.056258	-1.047206869	0.037831465	-27.680843	1.19E-168	1.04E-166
ENSG00000225746	MEG8	60.03710605	81.010814	39.06339824	-1.047597121	0.322272184	-3.2506594	0.001151	0.003654
ENSG00000106404	CLDN15	92.03036649	124.06336	59.9973741	-1.048217032	0.261055075	-4.0153099	5.94E-05	0.000234
ENSG00000035664	DAPK2	326.060205	439.84528	212.2751347	-1.052730163	0.151445328	-6.9512224	3.62E-12	2.76E-11
ENSG00000167363	FN3K	359.4058557	485.35783	233.4538805	-1.052958409	0.127007575	-8.2905166	1.13E-16	1.09E-15
ENSG00000144677	CTDSPL	1919.062648	2589.7357	1248.389626	-1.053425611	0.059293618	-17.766256	1.29E-70	4.37E-69
ENSG00000165113	GKAP1	52.62523029	70.924968	34.32549276	-1.05449155	0.32956761	-3.1996213	0.001376	0.004298
ENSG00000075213	SEMA3A	558.1432516	753.4066	362.8799028	-1.054909102	0.104417862	-10.102765	5.37E-24	6.96E-23
ENSG00000226702	MIR217HG	92.47081515	124.99941	59.94222196	-1.055899889	0.24548192	-4.3013347	1.70E-05	7.20E-05
ENSG00000092853	CLSPN	388.639866	525.42367	251.8560599	-1.060416653	0.123958892	-8.5545832	1.18E-17	1.20E-16
ENSG00000277013	AC008556.1	27.23087465	36.816027	17.64572218	-1.060557559	0.440428861	-2.408011	0.01604	0.039444
ENSG00000152117	AC073869.1	458.4296145	620.22638	296.6328493	-1.061597678	0.132934767	-7.9858543	1.40E-15	1.28E-14
ENSG00000101871	MID1	1679.956573	2272.8703	1087.042826	-1.063301335	0.060671409	-17.525575	9.14E-69	3.04E-67
ENSG00000101347	SAMHD1	4166.193538	5636.1666	2696.220523	-1.063439949	0.104082219	-10.217307	1.66E-24	2.20E-23
ENSG00000269352	PTOV1-AS2	84.76103628	114.67049	54.85158037	-1.064889303	0.286934783	-3.711259	0.000206	0.000746
ENSG00000223745	CCDC18-AS1	37.23678172	50.441819	24.0317441	-1.065447907	0.390018866	-2.7317856	0.006299	0.017018
ENSG00000131370	SH3BP5	6812.262456	9223.6976	4400.827283	-1.06769523	0.042286339	-25.249176	1.16E-140	8.03E-139
ENSG00000188511	C22orf34	286.4380186	388.24052	184.6355152	-1.070628636	0.135341486	-7.9105725	2.56E-15	2.32E-14
ENSG00000146909	NOM1	1545.540526	2094.2889	996.7921571	-1.071302421	0.066321408	-16.153192	1.08E-58	3.09E-57
ENSG00000236144	TMEM147-AS1	61.98271366	84.034247	39.93118043	-1.072507715	0.304250978	-3.5250757	0.000423	0.001457
ENSG00000139618	BRCA2	309.7926635	419.92303	199.6623012	-1.073015427	0.126870086	-8.457592	2.73E-17	2.72E-16
ENSG00000111665	CDCA3	130.1766289	176.45095	83.9023098	-1.074314686	0.197545249	-5.4383221	5.38E-08	2.96E-07
ENSG00000196872	KIAA1211L	441.6040067	598.87304	284.3349716	-1.076015589	0.111104703	-9.6846989	3.50E-22	4.28E-21
ENSG00000166839	ANKDD1A	27.47684391	37.265934	17.68775417	-1.076509881	0.442032134	-2.4353657	0.014877	0.036924
ENSG00000179456	ZBTB18	1597.678725	2167.4917	1027.865777	-1.076931876	0.063335114	-17.003709	7.71E-65	2.40E-63
ENSG00000215769	ARHGAP27P1-	48.45197495	65.813957	31.08999255	-1.077948908	0.329892267	-3.2675786	0.001085	0.003457
ENSG00000178695	KCTD12	31720.36028	43063.96	20376.76068	-1.079690672	0.036584805	-29.511997	2.02E-191	2.09E-189
ENSG00000125246	CLYBL	22.79961212	30.961626	14.63759846	-1.082096597	0.466251592	-2.3208427	0.020295	0.048494
ENSG00000120278	PLEKHG1	3731.522335	5070.658	2392.386674	-1.083552181	0.052019412	-20.829766	2.33E-96	1.05E-94
ENSG00000125457	MIF4GD	411.5781575	559.67605	263.4802623	-1.085242571	0.116866887	-9.2861426	1.60E-20	1.83E-19
ENSG00000109805	NCAPG	507.0615919	689.33617	324.7870161	-1.085697846	0.104999789	-10.34	4.65E-25	6.27E-24
ENSG00000151692	RNF144A	1257.402697	1709.5361	805.2693105	-1.085731182	0.083910781	-12.939114	2.71E-38	5.24E-37
ENSG00000243243	AC073130.2	26.3046135	35.773226	16.83600114	-1.088424909	0.430266877	-2.5296507	0.011418	0.029052
ENSG00000271533	Z83843.1	34.8366278	47.395574	22.27768114	-1.090123457	0.398513783	-2.7354724	0.006229	0.016841
ENSG00000206530	CFAP44	32.90181068	44.808913	20.99470854	-1.090157082	0.391872343	-2.7819189	0.005404	0.014799
ENSG00000255284	AP006621.3	43.5000458	59.211473	27.78861871	-1.090874186	0.345280552	-3.159385	0.001581	0.004871
ENSG00000185909	KLHDC8B	2899.663773	3946.4553	1852.872221	-1.091027371	0.050548575	-21.583741	2.55E-103	1.23E-101
ENSG00000160145	KALRN	1403.681459	1910.8514	896.5115081	-1.092491905	0.068077593	-16.047746	5.93E-58	1.66E-56
ENSG00000198478	SH3BGRL2	412.0272269	560.92865	263.1258046	-1.093501995	0.122406071	-8.9333967	4.13E-19	4.44E-18
ENSG00000204682	CASC10	199.3987913	271.62772	127.1698668	-1.093808313	0.158459586	-6.9027589	5.10E-12	3.85E-11
ENSG00000164035	EMCN	5385.999306	7335.3507	3436.64796	-1.094229148	0.03981722	-27.481305	2.94E-166	2.51E-164
ENSG00000251257	AC010457.1	35.40137016	48.225718	22.57702211	-1.094529835	0.417221139	-2.6233806	0.008706	0.0228
ENSG00000156970	BUB1B	339.0839277	462.17263	215.9952244	-1.096198303	0.134867975	-8.1279363	4.37E-16	4.11E-15
ENSG00000110852	CLEC2B	222.3764807	303.05949	141.6934687	-1.098247114	0.161019643	-6.8205785	9.07E-12	6.75E-11
ENSG00000140876	NUDT7	85.79273729	116.9846	54.60087257	-1.098947067	0.242679274	-4.5283928	5.94E-06	2.68E-05
ENSG00000196912	ANKRD36B	91.7843521	125.21434	58.35436324	-1.099638076	0.279025739	-3.9409915	8.11E-05	0.000314
ENSG00000139679	LPAR6	585.6291753	798.98379	372.2745588	-1.100257427	0.123786285	-8.8883629	6.20E-19	6.63E-18
ENSG00000178999	AURKB	175.4912304	239.35494	111.6275243	-1.101061404	0.166621902	-6.6081433	3.89E-11	2.78E-10

ENSG00000172687	ZNF738	87.78246265	119.5725	55.99242784	-1.101137883	0.25888472	-4.2533908	2.11E-05	8.85E-05
ENSG00000167615	LENG8	2368.533419	3231.2492	1505.817676	-1.10144065	0.309709718	-3.5563645	0.000376	0.001303
ENSG00000187210	GCNT1	230.5590725	314.523	146.5951444	-1.101928577	0.15916206	-6.9233119	4.41E-12	3.35E-11
ENSG00000099889	ARVCF	3366.612933	4593.8565	2139.369404	-1.102015373	0.059908485	-18.39498	1.44E-75	5.18E-74
ENSG00000280106	AC008555.7	46.40900223	63.368114	29.44989069	-1.102949363	0.329966093	-3.3426142	0.00083	0.002703
ENSG00000105011	ASF1B	230.3392331	315.02154	145.6569287	-1.109411327	0.160446335	-6.9145321	4.69E-12	3.55E-11
ENSG00000024526	DEPDC1	163.3842043	223.24983	103.5185832	-1.109648804	0.194043675	-5.7185518	1.07E-08	6.31E-08
ENSG00000259972	AC009120.2	42.13621075	57.5581	26.71432192	-1.110433709	0.343305288	-3.2345372	0.001218	0.003848
ENSG00000184785	SMIM10	53.9266132	73.683454	34.16977253	-1.111030252	0.33701217	-3.2967066	0.000978	0.003146
ENSG00000158270	COLEC12	251.4949211	343.92828	159.0615658	-1.111282782	0.150746254	-7.3718766	1.68E-13	1.39E-12
ENSG00000260314	MRC1	366.7846398	501.46012	232.1091546	-1.111496298	0.123167985	-9.0242306	1.81E-19	1.98E-18
ENSG00000140043	PTGR2	34.95990038	47.779614	22.14018664	-1.111801089	0.37876272	-2.9353498	0.003332	0.009595
ENSG00000138650	PCDH10	295.8240987	404.63038	187.0178136	-1.112384232	0.160893865	-6.9137765	4.72E-12	3.57E-11
ENSG00000149636	DSN1	494.8408108	676.54833	313.1332955	-1.112697043	0.105443217	-10.552571	4.94E-26	6.97E-25
ENSG00000170312	CDK1	391.0575845	535.01034	247.1048322	-1.112818863	0.120300567	-9.250321	2.24E-20	2.55E-19
ENSG00000171388	APLN	88069.46511	120469.46	55669.46899	-1.113704322	0.038238766	-29.125007	1.73E-186	1.68E-184
ENSG00000175455	CCDC14	560.2487011	766.96129	353.536112	-1.114869309	0.119502493	-9.3292557	1.07E-20	1.23E-19
ENSG00000171435	KSR2	440.2708781	602.71718	277.8245753	-1.117190724	0.133663342	-8.3582433	6.37E-17	6.22E-16
ENSG00000148057	IDNK	41.69783266	57.12034	26.27532525	-1.11808653	0.349052988	-3.2032	0.001359	0.004248
ENSG00000105967	TFEC	529.8221142	725.63475	334.0094831	-1.118576555	0.100962362	-11.079144	1.58E-28	2.38E-27
ENSG00000218510	LINC00339	40.79664744	55.89737	25.69592507	-1.118891314	0.362983233	-3.0824876	0.002053	0.006179
ENSG00000198563	DDX39B	1133.742545	1552.6849	714.8001713	-1.119006804	0.309999098	-3.6097099	0.000307	0.001077
ENSG00000119718	EIF2B2	3940.843705	5399.2545	2482.432955	-1.121037215	0.042453211	-26.406418	1.16E-153	8.99E-152
ENSG00000186871	ERCC6L	77.67793781	106.41365	48.94222934	-1.121327595	0.258764058	-4.3333978	1.47E-05	6.26E-05
ENSG00000082497	SERTAD4	230.0099138	314.97108	145.0487481	-1.122199311	0.174798541	-6.4199581	1.36E-10	9.35E-10
ENSG00000111247	RAD51AP1	113.6774042	155.59828	71.75653087	-1.123235181	0.255923136	-4.3889552	1.14E-05	4.95E-05
ENSG00000168309	FAM107A	1872.13592	2567.9727	1176.29913	-1.125831716	0.06247574	-18.020302	1.35E-72	4.69E-71
ENSG00000130997	POLN	35.27148402	48.40011	22.14285816	-1.126771485	0.375430017	-3.0012824	0.002688	0.007891
ENSG00000196730	DAPK1	2769.572441	3799.8927	1739.252202	-1.127139196	0.048271381	-23.350051	1.38E-120	7.93E-119
ENSG00000269858	EGLN2	154.8312406	212.68855	96.97393208	-1.12907918	0.216617047	-5.2123284	1.86E-07	9.82E-07
ENSG00000152932	RAB3C	175.6444852	241.28352	110.0054461	-1.131351887	0.205475834	-5.5060095	3.67E-08	2.06E-07
ENSG00000183763	TRAIP	62.84816999	86.357442	39.33889757	-1.13216928	0.281338494	-4.0242246	5.72E-05	0.000226
ENSG00000166669	ATF7IP2	236.4942147	324.89774	148.0906871	-1.134060508	0.158331847	-7.1625546	7.92E-13	6.30E-12
ENSG00000235316	DUSP8P5	34.12578038	46.928635	21.32292597	-1.134405595	0.387047815	-2.9309185	0.00338	0.009715
ENSG00000260260	SNHG19	113.7132826	156.42649	71.00007362	-1.135287063	0.236643648	-4.7974542	1.61E-06	7.70E-06
ENSG00000171848	RRM2	1040.954835	1431.3289	650.580793	-1.138422592	0.079281864	-14.35918	9.33E-47	2.14E-45
ENSG00000022567	SLC45A4	181.7924773	249.90481	113.6801451	-1.138490732	0.168397813	-6.7607216	1.37E-11	1.01E-10
ENSG00000152253	SPC25	87.12201847	119.9473	54.29673425	-1.13942243	0.253875769	-4.4881102	7.19E-06	3.20E-05
ENSG00000111057	KRT18	2276.403411	3131.3227	1421.484159	-1.139486545	0.05654333	-20.152448	2.56E-90	1.08E-88
ENSG00000128849	CGNL1	823.83424	1133.3597	514.3088194	-1.139990573	0.085129568	-13.391241	6.80E-41	1.38E-39
ENSG00000158402	CDC25C	63.7485925	87.721725	39.77546019	-1.140302316	0.284689709	-4.0054216	6.19E-05	0.000244
ENSG00000079156	OSBPL6	205.5850531	282.75957	128.4105345	-1.140630385	0.161608845	-7.05797	1.69E-12	1.32E-11
ENSG00000160298	C21orf58	75.69950319	104.32987	47.06913509	-1.144523661	0.300268053	-3.8116731	0.000138	0.000514
ENSG00000178585	CTNNBIP1	1990.323941	2741.7122	1238.935637	-1.145410132	0.059271073	-19.324943	3.31E-83	1.28E-81
ENSG00000165480	SKA3	116.8180669	161.00593	72.63020181	-1.145427973	0.213055153	-5.376204	7.61E-08	4.14E-07
ENSG00000186918	ZNF395	367.8989873	506.72558	229.0723919	-1.145666174	0.12682992	-9.0330907	1.67E-19	1.83E-18
ENSG00000156398	SFXN2	74.57039693	102.67733	46.46345922	-1.145732613	0.271905647	-4.2137139	2.51E-05	0.000105
ENSG00000076382	SPAG5	303.2922602	418.01818	188.5663358	-1.147789746	0.138019242	-8.3161574	9.09E-17	8.83E-16
ENSG00000141401	IMPA2	32.0253792	44.08784	19.96291869	-1.148855822	0.399305507	-2.8771349	0.004013	0.011333
ENSG00000162817	C1orf115	911.9717012	1257.6331	566.3103458	-1.148943764	0.084419988	-13.609855	3.50E-42	7.36E-41
ENSG00000102010	BMX	3980.174193	5486.2553	2474.093122	-1.148962802	0.047522095	-24.177444	3.84E-129	2.34E-127
ENSG00000134291	TMEM106C	545.1262358	751.52338	338.7290921	-1.148969099	0.098486461	-11.666264	1.90E-31	3.15E-30
ENSG00000065328	MCM10	118.9349089	163.89324	73.97657861	-1.149343087	0.209275099	-5.4920203	3.97E-08	2.22E-07
ENSG00000179611	DGKZP1	21.14939356	29.155781	13.14300568	-1.152041578	0.489465249	-2.3536739	0.018589	0.044906
ENSG00000106688	SLC1A1	628.1558432	866.58991	389.721781	-1.152941886	0.093360654	-12.349334	4.91E-35	8.86E-34
ENSG00000107738	VSIR	432.8055673	597.0911	268.5200388	-1.153046613	0.113039338	-10.200401	1.97E-24	2.60E-23
ENSG00000135480	KRT7	2681.270353	3699.8031	1662.737581	-1.15381919	0.202398947	-5.7007174	1.19E-08	6.99E-08
ENSG00000259291	ZNF710-AS1	51.90114352	71.580174	32.22211302	-1.15406995	0.346852363	-3.3272656	0.000877	0.002843
ENSG00000144554	FANCD2	230.3354245	318.2599	142.4109445	-1.157073316	0.160642663	-7.2027772	5.90E-13	4.74E-12
ENSG00000093072	ADA2	27.74667356	38.268396	17.22495124	-1.157916473	0.434558018	-2.6645843	0.007708	0.020426
ENSG00000179981	TSHZ1	1477.10377	2039.8209	914.3866619	-1.158700298	0.075402423	-15.366884	2.73E-53	7.07E-52
ENSG00000165507	DEPP1	18735.74394	25882.832	11588.65601	-1.159358464	0.040006204	-28.979467	1.19E-184	1.13E-182
ENSG00000163644	PPM1K	439.9732003	607.80722	272.1391815	-1.159498121	0.120897441	-9.5907582	8.74E-22	1.05E-20
ENSG00000248124	RRN3P1	127.0019871	175.51203	78.49194032	-1.159875612	0.232297782	-4.993055	5.94E-07	2.99E-06
ENSG00000189337	KAZN	364.711498	503.73459	225.6884028	-1.160164034	0.131576239	-8.8174281	1.17E-18	1.24E-17
ENSG00000136630	HLX	477.7190545	660.01059	295.4275177	-1.160227644	0.110659673	-10.484647	1.02E-25	1.42E-24
ENSG00000125378	BMP4	3491.171946	4824.6319	2157.712004	-1.160922117	0.055986114	-20.735894	1.64E-95	7.35E-94
ENSG00000264964	AP001033.1	32.11604058	44.385522	19.84655892	-1.161362299	0.393018913	-2.9549782	0.003127	0.009048
ENSG00000235109	ZSCAN31	307.1453562	424.68115	189.6095595	-1.162943251	0.128873659	-9.0239019	1.82E-19	1.98E-18
ENSG00000161800	RACGAP1	467.0130953	645.59761	288.4285771	-1.163185494	0.122651672	-9.4836497	2.46E-21	2.92E-20
ENSG00000197852	INKA2	1801.700732	2491.1961	1112.205397	-1.163692428	0.058602173	-19.857496	9.49E-88	3.86E-86
ENSG00000163751	CPA3	175.6757777	243.07589	108.2756643	-1.164066833	0.174102866	-6.6860866	2.29E-11	1.66E-10
ENSG00000109452	INPP4B	208.052718	287.75026	128.3551803	-1.165112835	0.157868986	-7.3802516	1.58E-13	1.31E-12
ENSG00000133056	PIK3C2B	1631.952614	2257.3572	1006.548063	-1.165529541	0.062841752	-18.547057	8.61E-77	3.13E-75
ENSG00000160325	CACFD1	287.3826808	397.89626	176.869099	-1.16882072	0.135710445	-8.6126069	7.14E-18	7.30E-17
ENSG00000235944	ZNF815P	53.97908402	74.788567	33.16960141	-1.170431263	0.310417031	-3.7705124	0.000163	0.000599
ENSG00000237781	AL356356.1	43.27918655	59.997575	26.56079826	-1.170484471	0.400043233	-2.9258949	0.003435	0.009858
ENSG00000272449	AL139246.5	33.59560016	46.598562	20.59263828	-1.170484821	0.40350932	-2.9007628	0.003723	0.010587
ENSG00000251322	SHANK3	5456.825673	7557.7068	3355.944578	-1.171063139	0.105363199	-11.114537	1.07E-28	1.61E-27
ENSG00000137070	IL11RA	94.70378789	131.38603	58.02155056	-1.171691818	0.263578948	-4.4453164	8.78E-06	3.87E-05
ENSG00000205309	NT5M	20.75410519	28.799096	12.70911458	-1.171754169	0.498298937	-2.3515085	0.018697	0.04512
ENSG00000007944	MYLIP	680.41639	942.97029	417.8624941	-1.173643318	0.10500842	-11.176659	5.30E-29	8.11E-28
ENSG00000169679	BUB1	384.2227994	532.54944	235.8961638	-1.173925473	0.123203967	-9.528309	1.60E-21	1.91E-20
ENSG00000185305	ARL15	345.0845621	478.41599	211.7531301	-1.174415627	0.136569832	-8.5993782	8.01E-18	8.17E-17
ENSG00000134253	TRIM45	36.62415632	50.835536	22.41277697	-1.175112141	0.376371542	-3.1222131	0.001795	0.005468

ENSG00000176974	SHMT1	257.9531503	357.68871	158.2175898	-1.175448663	0.140611905	-8.3595245	6.30E-17	6.16E-16
ENSG00000189136	UBE2Q2P1	96.74909997	134.16747	59.33072809	-1.175756464	0.240197021	-4.8949669	9.83E-07	4.84E-06
ENSG00000117643	MAN1C1	398.1050278	552.2134	243.9966507	-1.177782285	0.119625724	-9.8455604	7.16E-23	8.94E-22
ENSG00000112742	TTK	187.7645857	260.378	115.1511691	-1.177782537	0.164039261	-7.179882	6.98E-13	5.57E-12
ENSG00000154760	SLFN13	69.09165511	96.037425	42.14588511	-1.179297463	0.321288322	-3.670527	0.000242	0.000867
ENSG00000151623	NR3C2	852.7776131	1183.1928	522.3624417	-1.179954774	0.079677838	-14.809071	1.28E-49	3.12E-48
ENSG00000234912	SNHG20	206.5771356	286.62214	126.5321326	-1.180819565	0.172306023	-6.8530371	7.23E-12	5.42E-11
ENSG00000085563	ABCB1	493.3267969	684.97166	301.6819339	-1.181461513	0.105585647	-11.189603	4.58E-29	7.05E-28
ENSG00000230606	AC092683.1	36.85340556	51.233301	22.47350964	-1.181565613	0.405765828	-2.9119397	0.003592	0.01026
ENSG00000173535	TNFRSF10C	1867.992765	2593.4747	1142.510829	-1.183405065	0.06307249	-18.762619	1.53E-78	5.68E-77
ENSG00000235750	KIAA0040	1268.535228	1762.4387	774.6317077	-1.185192941	0.075314692	-15.736544	8.50E-56	2.29E-54
ENSG00000186642	PDE2A	4554.14324	6326.9467	2781.339817	-1.185602926	0.044633396	-26.563135	1.81E-155	1.43E-153
ENSG00000266904	LINC00663	22.21199463	30.863122	13.56086761	-1.185974679	0.482043949	-2.460304	0.013882	0.034701
ENSG00000079435	LIPE	131.8990119	183.35037	80.44764978	-1.188330385	0.193978228	-6.1261019	9.01E-10	5.80E-09
ENSG00000151725	CENPU	177.9678764	247.44583	108.4899207	-1.189384977	0.167983554	-7.0803656	1.44E-12	1.13E-11
ENSG00000205269	TMEM170B	291.8561753	405.52921	178.1831447	-1.189553208	0.138410228	-8.5944025	8.37E-18	8.53E-17
ENSG00000171357	LURAP1	31.45461195	43.692147	19.2170768	-1.189670207	0.428565052	-2.7759385	0.005504	0.015047
ENSG00000104147	OIP5	36.77508222	51.211478	22.33868666	-1.191550489	0.386420917	-3.0835559	0.002045	0.006161
ENSG00000109046	WSB1	5810.923612	8083.6781	3538.169121	-1.191957731	0.215714793	-5.5256189	3.28E-08	1.85E-07
ENSG00000145386	CCNA2	508.9919767	708.47172	309.5122292	-1.193629128	0.101434862	-11.767445	5.74E-32	9.68E-31
ENSG00000184307	ZDHHC23	126.4717387	176.12542	76.81805851	-1.196255118	0.203637815	-5.8744252	4.24E-09	2.58E-08
ENSG00000184661	CDCA2	177.5424	247.40362	107.6811849	-1.196731551	0.190552899	-6.2803114	3.38E-10	2.26E-09
ENSG00000271270	TMCC1-AS1	56.11503081	78.224974	34.00508781	-1.197382818	0.357739758	-3.3470778	0.000817	0.002665
ENSG00000136367	ZFHX2	104.4123367	145.48024	63.34443673	-1.198169522	0.238768516	-5.0181219	5.22E-07	2.63E-06
ENSG00000215845	TSTD1	28.20712533	39.329465	17.08478521	-1.198850967	0.428860765	-2.7954317	0.005183	0.014257
ENSG00000053747	LAMA3	669.5338788	932.80874	406.2590222	-1.199053817	0.103770673	-11.554843	6.98E-31	1.14E-29
ENSG00000100429	HDAC10	35.92221464	50.127448	21.71698165	-1.19954431	0.389107564	-3.082809	0.002051	0.006174
ENSG00000174371	EXO1	97.966628	136.72663	59.20662659	-1.200943454	0.25460998	-4.7167965	2.40E-06	1.13E-05
ENSG00000156103	MMP16	1182.105624	1647.5646	716.6466692	-1.201362298	0.070845808	-16.957422	1.70E-64	5.24E-63
ENSG00000013573	DDX11	177.5932833	247.61766	107.5689101	-1.203011092	0.171140725	-7.0293677	2.07E-12	1.61E-11
ENSG00000146674	IGFBP3	161.5758154	225.40781	97.7438177	-1.204854001	0.189524905	-6.3572331	2.05E-10	1.39E-09
ENSG00000137269	LRRC1	594.3314255	828.96503	359.6978332	-1.205643498	0.097912655	-12.313459	7.67E-35	1.38E-33
ENSG00000135451	TROAP	108.1075355	150.96596	65.24911435	-1.205769839	0.224095164	-5.3806152	7.42E-08	4.05E-07
ENSG00000204839	MROH6	31.48422388	44.010568	18.95787947	-1.207799942	0.420647426	-2.8712881	0.004088	0.011518
ENSG00000137310	TCF19	493.964774	689.41714	298.5124033	-1.207829292	0.108256915	-11.157064	6.61E-29	1.01E-27
ENSG00000043591	ADRB1	52.3509322	73.071797	31.63006764	-1.2083739	0.333575457	-3.6224904	0.000292	0.00103
ENSG00000234465	PINLYP	34.68593339	48.538459	20.83340778	-1.209417916	0.417733839	-2.8951878	0.003789	0.010765
ENSG00000051341	POLQ	99.34813439	138.91024	59.78602677	-1.21026725	0.241311923	-5.0153645	5.29E-07	2.67E-06
ENSG00000185950	IRS2	143.443681	200.35786	86.529498	-1.211727369	0.185102417	-6.5462536	5.90E-11	4.17E-10
ENSG00000175063	UBE2C	228.9463624	319.90266	137.99006	-1.214455932	0.168066037	-7.2260639	4.97E-13	4.01E-12
ENSG00000102893	PHKB	2496.486872	3490.8152	1502.158579	-1.216631636	0.056915887	-21.375959	2.24E-101	1.06E-99
ENSG00000170379	TCAF2	293.1558173	409.93331	176.3783227	-1.218544909	0.14163615	-8.6033467	7.74E-18	7.90E-17
ENSG00000142945	KIF2C	300.2152793	420.17849	180.2520674	-1.220526755	0.130117344	-9.3802003	6.58E-21	7.68E-20
ENSG00000079257	LXN	1552.755705	2174.4648	931.0466266	-1.224455061	0.077173051	-15.866356	1.08E-56	2.97E-55
ENSG00000168405	CMAHP	102.6117821	143.73379	61.48977031	-1.226762519	0.229852807	-5.3371657	9.44E-08	5.10E-07
ENSG00000135473	PAN2	258.7496919	362.74467	154.7547131	-1.226769226	0.158777311	-7.7263509	1.11E-14	9.70E-14
ENSG00000159433	STARD9	2559.308624	3588.5265	1530.090783	-1.229272078	0.072435627	-16.970545	1.36E-64	4.20E-63
ENSG00000127564	PKMYT1	134.8339116	189.17229	80.49553515	-1.229492397	0.208591787	-5.8942512	3.76E-09	2.30E-08
ENSG00000168916	ZNF608	1059.891669	1486.2889	633.4944277	-1.229874808	0.073038799	-16.83865	1.27E-63	3.87E-62
ENSG00000173599	PC	262.2887251	367.92638	156.651068	-1.230889223	0.148745274	-8.2751484	1.28E-16	1.24E-15
ENSG00000176435	CLEC14A	11710.86543	16426.039	6995.691381	-1.231530314	0.032092905	-38.373912	0	0
ENSG00000197747	S100A10	9231.078039	12949.095	5513.061488	-1.232169242	0.040843354	-30.16817	6.20E-200	6.87E-198
ENSG00000126787	DLGAP5	483.481843	678.43721	288.5264736	-1.233471154	0.121291289	-10.169495	2.71E-24	3.56E-23
ENSG00000087842	PIR	2195.126133	3081.0228	1309.229467	-1.23437483	0.055410014	-22.277107	6.16E-110	3.21E-108
ENSG00000086300	SNX10	28.37448046	39.778137	16.97082412	-1.235075757	0.429655755	-2.8745705	0.004046	0.011414
ENSG00000109501	WFS1	7698.486129	10809.766	4587.20602	-1.236367776	0.038553039	-32.069269	1.18E-225	1.58E-223
ENSG00000261572	AC097639.1	55.73162751	78.254056	33.20919935	-1.236747797	0.302021	-4.0949066	4.22E-05	0.00017
ENSG00000101003	GINS1	220.9669869	310.46496	131.469013	-1.237389206	0.155553143	-7.9547683	1.79E-15	1.64E-14
ENSG00000213203	GIMAP1	748.3158656	1051.4123	445.2193829	-1.238175078	0.086763432	-14.270702	3.33E-46	7.56E-45
ENSG00000081189	MEF2C	1387.769207	1949.4525	826.085906	-1.238879115	0.071972417	-17.213249	2.11E-66	6.73E-65
ENSG00000196468	FGF16	846.7899503	1189.7961	503.7838468	-1.239028386	0.085109881	-14.557985	5.20E-48	1.23E-46
ENSG00000162415	ZSWIM5	38.42432477	53.975741	22.87290837	-1.239751895	0.376823226	-3.2900092	0.001002	0.003215
ENSG00000130653	PNPLA7	106.3738711	149.41721	63.3305334	-1.242314968	0.238343439	-5.2122894	1.87E-07	9.82E-07
ENSG00000213185	FAM24B	52.80019169	74.296099	31.30428426	-1.242463213	0.335433591	-3.7040513	0.000212	0.000767
ENSG00000166265	CYYR1	1060.429593	1490.7326	630.1273355	-1.243036341	0.090899328	-13.674868	1.43E-42	3.05E-41
ENSG00000184860	SDR42E1	135.8478977	191.03146	80.66434016	-1.243068521	0.195082818	-6.3720041	1.87E-10	1.27E-09
ENSG00000285589	AC010422.8	22.24260717	31.20228	13.28293423	-1.243336918	0.535049754	-2.3237781	0.020137	0.048174
ENSG00000231889	TRAF3IP2-AS1	41.91373156	58.980892	24.84657072	-1.246439025	0.375096843	-3.3229792	0.000891	0.002883
ENSG00000175265	GOLGA8A	26.58447507	37.446844	15.72210641	-1.247608773	0.473910937	-2.6325807	0.008474	0.022278
ENSG00000212864	RNF208	18.31213533	25.769787	10.85448353	-1.250059428	0.527541078	-2.3695964	0.017808	0.043214
ENSG00000182022	CHST15	3190.048216	4491.6617	1888.434713	-1.250351527	0.056070384	-22.299678	3.72E-110	1.95E-108
ENSG00000262185	AC005736.1	22.33504258	31.466584	13.20350087	-1.251024049	0.530452719	-2.3584082	0.018353	0.044408
ENSG00000157617	C2CD2	1351.331909	1903.1444	799.5194564	-1.251202615	0.064028617	-19.541303	4.89E-85	1.93E-83
ENSG00000156298	TSPAN7	28.4319544	40.057057	16.80685183	-1.253071439	0.43520098	-2.8792937	0.003986	0.011267
ENSG00000118898	PPL	24.7943107	34.995964	14.59265747	-1.254931836	0.464926946	-2.6992022	0.006951	0.018604
ENSG00000065413	ANKRD44	130.9666875	184.49632	77.43705663	-1.255353941	0.217414932	-5.7740006	7.74E-09	4.61E-08
ENSG00000273619	AL121832.2	25.58548511	36.062317	15.10865344	-1.257301851	0.482926767	-2.6035042	0.009228	0.023993
ENSG00000225138	SLC9A3-AS1	144.2397809	203.49254	84.98701985	-1.258016189	0.253766891	-4.9573693	7.15E-07	3.57E-06
ENSG00000168497	CAVIN2	14891.54487	21008.774	8774.315334	-1.259630713	0.031431798	-40.075045	0	0
ENSG00000174004	NRROS	499.2362146	704.23026	294.2421688	-1.260352517	0.108061469	-11.663293	1.96E-31	3.26E-30
ENSG00000267519	AC020916.1	314.6259631	444.05416	185.1977696	-1.261236785	0.426262427	-2.958827	0.003088	0.008948
ENSG00000196436	NPIPB15	37.16310462	52.469062	21.85714768	-1.265691054	0.406569189	-3.1131013	0.001851	0.005631
ENSG00000144354	CDCA7	141.6896254	200.25166	83.12759145	-1.266029649	0.202727472	-6.2449832	4.24E-10	2.80E-09
ENSG00000198208	RPS6KL1	523.793772	740.26814	307.319407	-1.266274692	0.111108028	-11.396788	4.34E-30	6.89E-29
ENSG00000262370	AC108134.3	24.91471988	35.228768	14.60067205	-1.26688333	0.474039023	-2.6725296	0.007528	0.020003

ENSG00000122378	PRXL2A	12774.07146	18051.755	7496.38768	-1.267743394	0.033006997	-38.408323	0	0
ENSG00000116678	LEPR	1282.278053	1812.5968	751.9593544	-1.268558789	0.067351769	-18.834825	3.91E-79	1.46E-77
ENSG00000164850	GPER1	72.97708474	103.30321	42.65095751	-1.271355292	0.280231413	-4.5368051	5.71E-06	2.58E-05
ENSG00000185745	IFIT1	579.2051874	819.26909	339.141289	-1.271403699	0.247403069	-5.1389973	2.76E-07	1.43E-06
ENSG00000198774	RASSF9	157.7302428	223.00537	92.45511566	-1.27234515	0.186969816	-6.8050832	1.01E-11	7.50E-11
ENSG00000227260	LINC01985	66.06804449	93.46918	38.66690851	-1.27250804	0.279537496	-4.5521909	5.31E-06	2.41E-05
ENSG00000221866	PLXNA4	150.3911201	212.85286	87.92937607	-1.27315724	0.214268449	-5.9418792	2.82E-09	1.74E-08
ENSG00000164849	GPR146	141.6267447	200.34577	82.90771921	-1.273879627	0.220853559	-5.7679833	8.02E-09	4.78E-08
ENSG00000160796	NBEAL2	2900.643116	4104.4698	1696.816403	-1.27418226	0.131733008	-9.6724601	3.95E-22	4.81E-21
ENSG00000197385	ZNF860	82.62069717	116.93957	48.30182364	-1.274800389	0.244273864	-5.2187343	1.80E-07	9.51E-07
ENSG00000072163	LIMS2	1156.972683	1638.1673	675.7780448	-1.276813445	0.071313	-17.904357	1.09E-71	3.74E-70
ENSG00000114315	HES1	862.3818256	1221.4334	503.3302698	-1.277345424	0.088844903	-14.37725	7.19E-47	1.65E-45
ENSG00000198889	DCAF12L1	30.9016649	43.739746	18.06358412	-1.277632667	0.407108543	-3.1383096	0.001699	0.005206
ENSG00000139190	VAMP1	64.62078012	91.52721	37.71434991	-1.278001725	0.287075342	-4.451799	8.52E-06	3.76E-05
ENSG00000178947	SMIM10L2A	51.96224703	73.633536	30.29095761	-1.278544715	0.325177139	-3.9318407	8.43E-05	0.000325
ENSG00000249898	MCPH1-AS1	24.37594872	34.563982	14.18791568	-1.279598987	0.473222718	-2.7040101	0.006851	0.018375
ENSG00000233237	LINC00472	156.7060886	222.13943	91.27274653	-1.281141821	0.192705708	-6.6481779	2.97E-11	2.14E-10
ENSG00000171121	KCNMB3	28.43872904	40.292675	16.58478302	-1.282456394	0.455799979	-2.8136386	0.004898	0.01356
ENSG00000176236	C10orf111	42.65181046	60.496411	24.80721025	-1.286813966	0.381676985	-3.3714738	0.000748	0.002458
ENSG00000167900	TK1	510.7024944	724.57763	296.8273604	-1.288079724	0.106297617	-12.117673	8.51E-34	1.50E-32
ENSG00000174501	ANKRD36C	237.0603925	336.39334	137.7274434	-1.288100378	0.158673833	-8.117913	4.74E-16	4.45E-15
ENSG00000244879	GABPB1-AS1	273.0884924	387.78761	158.3893746	-1.289086221	0.179990162	-7.1619816	7.95E-13	6.32E-12
ENSG00000166851	PLK1	398.9242208	566.41367	231.4347668	-1.28918379	0.120291627	-10.717153	8.46E-27	1.22E-25
ENSG00000273264	AL360219.1	23.88369289	33.822911	13.94447466	-1.289602641	0.4768759	-2.704273	0.006845	0.018368
ENSG00000178440	LINC00843	29.13816815	41.427215	16.8491213	-1.290122718	0.467791173	-2.7579031	0.005817	0.015815
ENSG00000129810	SGO1	60.40460326	85.817032	34.99217415	-1.290204179	0.305227007	-4.2270315	2.37E-05	9.88E-05
ENSG00000117724	CENPF	1298.112552	1842.7998	753.4252728	-1.290278838	0.067700522	-19.058625	5.57E-81	2.10E-79
ENSG00000272473	AC006273.1	17.32778617	24.584095	10.07147775	-1.291400476	0.54154729	-2.3846495	0.017095	0.041686
ENSG00000172824	CES4A	40.12849926	56.947528	23.30947099	-1.291447159	0.357357019	-3.613885	0.000302	0.001062
ENSG00000090889	KIF4A	441.1212844	626.48166	255.760913	-1.292851775	0.110476143	-11.702543	1.24E-31	2.07E-30
ENSG00000267002	AC060780.1	73.13079707	103.94129	42.32030603	-1.293466527	0.269505464	-4.7994074	1.59E-06	7.63E-06
ENSG00000076770	MBNL3	151.3364148	215.02893	87.64390306	-1.293583107	0.203154682	-6.3674787	1.92E-10	1.30E-09
ENSG00000013810	TACC3	925.5680393	1315.3396	535.7965009	-1.294955067	0.080178124	-16.150977	1.12E-58	3.20E-57
ENSG00000144712	CAND2	174.1445127	247.55535	100.7336803	-1.296622753	0.176752826	-7.3357965	2.20E-13	1.81E-12
ENSG00000115163	CENPA	79.14915184	112.46747	45.83083063	-1.296643921	0.253507285	-5.1148192	3.14E-07	1.62E-06
ENSG00000232442	MHENCR	33.70926728	47.952674	19.46586087	-1.297001626	0.40557399	-3.1979408	0.001384	0.004322
ENSG00000054654	SYNE2	3873.169005	5507.2471	2239.090896	-1.297885781	0.048873349	-26.556105	2.18E-155	1.70E-153
ENSG00000035499	DEPDC1B	55.251001	78.592077	31.90992475	-1.29809327	0.323176836	-4.0166656	5.90E-05	0.000233
ENSG00000213906	LTB4R2	38.40807585	54.662845	22.15330679	-1.299414125	0.410181054	-3.1679038	0.001535	0.004748
ENSG00000163808	KIF15	137.9036037	196.26403	79.54317865	-1.301171845	0.201277478	-6.4645675	1.02E-10	7.05E-10
ENSG00000102886	GDPD3	38.3997203	54.817883	21.9815574	-1.310343639	0.448187294	-2.9236519	0.00346	0.009916
ENSG00000099998	GGT5	436.5398967	622.51708	250.5627094	-1.311072829	0.11614326	-11.288411	1.50E-29	2.34E-28
ENSG00000143476	DTL	268.5835571	383.06552	154.1015915	-1.313936358	0.143705829	-9.1432363	6.06E-20	6.78E-19
ENSG00000119922	IFIT2	712.0282434	1016.7015	407.3549641	-1.317448434	0.101146581	-13.025141	8.80E-39	1.71E-37
ENSG00000213853	EMP2	985.5214656	1407.9978	563.0451506	-1.321839092	0.079299045	-16.669042	2.20E-62	6.62E-61
ENSG00000142910	TINAGL1	7935.187526	11337.108	4533.266641	-1.322166277	0.034792101	-38.00191	0	0
ENSG00000261526	AC012615.1	26.25832207	37.535274	14.98137009	-1.322299223	0.461611459	-2.8645286	0.004176	0.011731
ENSG00000254389	RHPN1-AS1	39.81002628	56.937357	22.68269577	-1.326315186	0.371496811	-3.5701927	0.000357	0.001241
ENSG00000183117	CSMD1	22.89752916	32.765741	13.02931743	-1.326648313	0.483055327	-2.7463693	0.006026	0.016324
ENSG00000235194	PPP1R3E	137.5472479	196.79229	78.30220267	-1.327079723	0.206193804	-6.4360795	1.23E-10	8.44E-10
ENSG00000124406	ATP8A1	85.57673087	122.31454	48.83891898	-1.32735272	0.325711293	-4.0752432	4.60E-05	0.000184
ENSG00000116667	C1orf21	1128.213815	1614.0736	642.354008	-1.329642921	0.075110912	-17.702393	4.02E-70	1.36E-68
ENSG00000178343	SHISA3	307.3087407	440.15915	174.4583284	-1.335823632	0.137527731	-9.713122	2.65E-22	3.25E-21
ENSG00000106133	NSUN5P2	68.22988034	97.801104	38.65865645	-1.337678719	0.316418874	-4.2275567	2.36E-05	9.86E-05
ENSG00000205885	C1RL-AS1	43.90014943	62.948623	24.85167629	-1.340204346	0.365299055	-3.6687868	0.000244	0.000872
ENSG00000133026	MYH10	8120.790173	11660.461	4581.119793	-1.347827477	0.04220604	-31.934469	8.88E-224	1.17E-221
ENSG00000215788	TNFRSF25	236.7263107	340.06636	133.3862651	-1.348771114	0.195056964	-6.914755	4.69E-12	3.55E-11
ENSG00000274471	AC242376.2	45.44148274	65.285447	25.59751818	-1.350217873	0.425427706	-3.1737892	0.001505	0.004659
ENSG00000144395	CCDC150	27.2684974	39.187809	15.34918546	-1.351480959	0.469273533	-2.8799429	0.003977	0.011249
ENSG00000159915	ZNF233	34.71955097	49.957449	19.48165255	-1.351521092	0.40724087	-3.3187266	0.000904	0.002925
ENSG00000146376	ARHGAP18	3825.773802	5499.1056	2152.442038	-1.353378329	0.05321776	-25.430953	1.15E-142	8.12E-141
ENSG00000137804	NUSAP1	35.9639161	51.721472	20.20635971	-1.355535963	0.37457959	-3.6188196	0.000296	0.001043
ENSG00000132563	REEP2	88.99640555	127.97919	50.01361713	-1.355562258	0.251148915	-5.3974442	6.76E-08	3.70E-07
ENSG00000120437	ACAT2	863.1821986	1241.9308	484.4335979	-1.358224319	0.079491686	-17.086369	1.87E-65	5.87E-64
ENSG00000204316	MRPL38	23.38196588	33.636718	13.127214	-1.360145651	0.501188875	-2.7138385	0.006651	0.017891
ENSG00000204588	LINC01123	37.33161961	53.747727	20.91551266	-1.36473391	0.376119231	-3.6284609	0.000285	0.001009
ENSG00000146411	SLC2A12	65.80048553	94.722618	36.87835319	-1.366300458	0.302667299	-4.5141991	6.36E-06	2.85E-05
ENSG00000227811	INKA2-AS1	28.8408721	41.491254	16.19048987	-1.366911424	0.430740896	-3.173396	0.001507	0.004665
ENSG00000183208	GDPGP1	47.5863888	68.614413	26.55836421	-1.367560966	0.348865606	-3.9200223	8.85E-05	0.00034
ENSG00000115525	ST3GAL5	920.8919194	1327.128	514.6558083	-1.367785994	0.080090285	-17.078051	2.16E-65	6.75E-64
ENSG00000176428	VPS37D	33.1007761	47.778132	18.42342029	-1.367999168	0.477099716	-2.8673234	0.00414	0.011644
ENSG00000139597	N4BP2L1	85.88405774	123.81006	47.95805201	-1.36812691	0.248814706	-5.4985774	3.83E-08	2.14E-07
ENSG00000154920	EME1	24.25287236	34.97646	13.52928425	-1.368840657	0.478421439	-2.8611608	0.004221	0.011849
ENSG00000276644	DACH1	598.0950629	862.23694	333.9593658	-1.368916068	0.110745258	-12.360946	4.25E-35	7.69E-34
ENSG00000176714	CCDC121	74.02466109	106.63934	41.40998153	-1.369222878	0.292375801	-4.6830924	2.83E-06	1.32E-05
ENSG00000228315	GUSBP11	94.62881297	136.6224	52.63522481	-1.370798309	0.263020292	-5.2117587	1.87E-07	9.85E-07
ENSG00000204282	TNRC6C-AS1	34.5024859	49.833073	19.17189833	-1.371635362	0.429926208	-3.1903972	0.001421	0.004425
ENSG00000184792	OSBP2	719.4722184	1038.6964	400.2480471	-1.374619933	0.089649776	-15.333222	4.59E-53	1.18E-51
ENSG00000196812	ZSCAN16	144.3359461	208.35396	80.31793238	-1.376017181	0.193193665	-7.1224757	1.06E-12	8.36E-12
ENSG00000100285	NEFH	36.99327376	53.436416	20.55013134	-1.376601965	0.410524532	-3.3532758	0.000799	0.002613
ENSG00000133624	ZNF767P	136.2462909	196.87658	75.61600342	-1.378690975	0.268977022	-5.1256831	2.96E-07	1.53E-06
ENSG00000074527	NTN4	31341.35379	45273.945	17408.76247	-1.378860367	0.044103552	-31.264157	1.43E-214	1.76E-212
ENSG00000138376	BARD1	81.56103215	117.80699	45.31507213	-1.380594456	0.250401487	-5.5135234	3.52E-08	1.98E-07
ENSG00000072571	HMMR	274.680501	396.93157	152.4294313	-1.381142862	0.140441408	-9.8342995	8.01E-23	9.98E-22
ENSG00000104938	CLEC4M	26.57845422	38.379144	14.77776449	-1.38289591	0.473767297	-2.9189349	0.003512	0.010053

ENSG00000173890	GPR160	348.3365899	503.51641	193.156773	-1.383521595	0.139344079	-9.9288151	3.12E-23	3.95E-22
ENSG00000107719	PALD1	5270.220196	7620.6321	2919.808245	-1.383890688	0.057233054	-24.17992	3.62E-129	2.21E-127
ENSG00000101104	PABPC1L	23.3808266	33.928129	12.83352431	-1.38645294	0.573762355	-2.4164237	0.015674	0.038675
ENSG00000226963	AC078883.2	18.07521417	26.211102	9.939326302	-1.389617653	0.571864008	-2.4299792	0.0151	0.037431
ENSG00000244041	LINC01011	18.86042097	27.323818	10.39702366	-1.390263165	0.538697735	-2.5807852	0.009858	0.025442
ENSG00000172197	MBOAT1	27.15098669	39.367978	14.93399505	-1.392780637	0.440830132	-3.1594497	0.001581	0.004871
ENSG00000134138	MEIS2	427.3159393	619.00953	235.6223507	-1.392909006	0.113670295	-12.25394	1.60E-34	2.86E-33
ENSG00000183615	FAM167B	646.4789089	937.03505	355.9227682	-1.394548696	0.095788596	-14.558609	5.15E-48	1.22E-46
ENSG00000183783	KCTD8	44.4519399	64.443552	24.46032752	-1.394659199	0.410999958	-3.3933317	0.00069	0.002289
ENSG00000110077	MS4A6A	89.68707202	130.00313	49.37101486	-1.397227203	0.259712812	-5.3798933	7.45E-08	4.06E-07
ENSG00000283537	AC073264.3	28.54037811	41.384653	15.69610357	-1.398575133	0.49000674	-2.8541957	0.004315	0.012073
ENSG00000284237	AL356275.1	65.19423145	94.636852	35.75161115	-1.398577955	0.288146585	-4.853703	1.21E-06	5.90E-06
ENSG00000168672	FAM84B	2840.846817	4120.4656	1561.228009	-1.400785727	0.058740282	-23.847106	1.09E-125	6.43E-124
ENSG00000143228	NUF2	125.041861	181.36372	68.72000563	-1.401732498	0.219839162	-6.3761728	1.82E-10	1.23E-09
ENSG00000251474	RPL32P3	170.2938376	247.10683	93.48085004	-1.40215828	0.241982196	-5.7944688	6.85E-09	4.11E-08
ENSG00000196549	MME	909.4084726	1319.6765	499.140454	-1.402956111	0.07903039	-17.752109	1.66E-70	5.61E-69
ENSG00000179750	APOBEC3B	74.92543166	108.86892	40.98194381	-1.404609345	0.271595043	-5.1717046	2.32E-07	1.21E-06
ENSG00000147174	GCNA	26.57158114	38.640387	14.50277548	-1.404712556	0.447130511	-3.1416164	0.00168	0.005152
ENSG00000004776	HSPB6	39.14334974	56.757532	21.52916772	-1.404946799	0.381799195	-3.6798056	0.000233	0.000838
ENSG00000046889	PREX2	3278.691274	4760.9185	1796.464032	-1.406081326	0.065323416	-21.52492	9.10E-103	4.33E-101
ENSG00000123485	HJURP	189.2740852	274.94992	103.5982541	-1.406756035	0.170189235	-8.2658344	1.39E-16	1.33E-15
ENSG00000146054	TRIM7	44.76965757	65.131741	24.40757405	-1.412189969	0.353382896	-3.9962025	6.44E-05	0.000253
ENSG00000152402	GUCY1A2	48.52406973	70.550508	26.49763154	-1.412362654	0.329362045	-4.2881767	1.80E-05	7.62E-05
ENSG00000175874	CREG2	528.6812058	768.39277	288.9696374	-1.4123725	0.111972959	-12.613514	1.78E-36	3.32E-35
ENSG00000232815	DUX4L50	23.22358316	33.854412	12.59275481	-1.41427614	0.499252558	-2.832787	0.004614	0.012827
ENSG00000102287	GABRE	48.33410879	70.303485	26.36473228	-1.416219355	0.405133147	-3.4956887	0.000473	0.001616
ENSG00000170835	CEL	17.72109808	25.778132	9.664064444	-1.416292819	0.55141741	-2.5684587	0.010215	0.026272
ENSG00000028137	TNFRSF1B	1458.163829	2121.7235	794.6041503	-1.416761043	0.069510756	-20.381896	2.42E-92	1.05E-90
ENSG00000285437	POLR2J3	28.95674605	42.170013	15.74347862	-1.417539713	0.42689243	-3.3206017	0.000898	0.002906
ENSG00000148773	MKI67	2303.019673	3354.1743	1251.865017	-1.421816214	0.055268598	-25.725571	6.05E-146	4.42E-144
ENSG00000073605	GSDMB	44.23509705	64.507197	23.96299685	-1.422064869	0.39321806	-3.616479	0.000299	0.001053
ENSG00000229807	XIST	4328.995324	6305.9585	2352.032146	-1.422757076	0.292731024	-4.860288	1.17E-06	5.72E-06
ENSG00000204248	COL11A2	15.2002548	22.125364	8.275145328	-1.422819862	0.576614734	-2.4675399	0.013605	0.034087
ENSG00000111206	FOXM1	877.6902533	1279.2163	476.1641746	-1.424239717	0.088126769	-16.161261	9.46E-59	2.72E-57
ENSG00000229152	ANKRD10-IT1	79.34228876	115.65049	43.03408961	-1.424546905	0.314617899	-4.5278635	5.96E-06	2.68E-05
ENSG00000066027	PPP2R5A	1950.42467	2841.6955	1059.153806	-1.424761019	0.064226306	-22.18345	4.96E-109	2.55E-107
ENSG00000173200	PARP15	93.68708982	136.64363	50.73054719	-1.426194799	0.250516903	-5.6930083	1.25E-08	7.30E-08
ENSG00000102452	NALCN	187.6577047	273.63762	101.6777848	-1.426609543	0.177807446	-8.0233397	1.03E-15	9.51E-15
ENSG00000280079	AC011447.7	16.5278382	24.119228	8.936448285	-1.427411861	0.593104297	-2.4066793	0.016098	0.039557
ENSG00000162755	KLHDC9	33.93376656	49.468192	18.39934118	-1.428086952	0.404462094	-3.5308301	0.000414	0.001427
ENSG00000240891	PLCXD2	155.2996898	226.50088	84.0925224	-1.429709892	0.19139695	-7.4698677	8.03E-14	6.79E-13
ENSG00000080986	NDC80	202.3747656	295.11173	109.6377974	-1.431613355	0.1660319	-8.6225199	6.55E-18	6.70E-17
ENSG00000104728	ARHGEF10	38.09045668	55.612081	20.56883202	-1.433451444	0.411668054	-3.4820566	0.000498	0.001692
ENSG00000117586	TNFSF4	2673.886412	3902.5227	1445.250081	-1.433591926	0.059088752	-24.261672	4.98E-130	3.09E-128
ENSG00000204634	TBC1D8	2704.552174	3950.5872	1458.517127	-1.437478322	0.048991379	-29.341455	3.07E-189	3.09E-187
ENSG00000064042	LIMCH1	2910.09766	4252.5928	1567.602513	-1.439711072	0.049312408	-29.195716	2.20E-187	2.17E-185
ENSG00000160180	TFF3	60.86329548	88.887914	32.83867708	-1.439800873	0.313975016	-4.585718	4.52E-06	2.07E-05
ENSG00000189057	FAM111B	221.1345608	323.33744	118.9316832	-1.441196769	0.171101913	-8.4230313	3.67E-17	3.63E-16
ENSG00000127586	CHTF18	257.4453139	376.57255	138.3180754	-1.441722957	0.150755012	-9.5633501	1.14E-21	1.37E-20
ENSG00000179532	DNHD1	43.97056042	64.425135	23.5159856	-1.445241886	0.374552984	-3.8585779	0.000114	0.000431
ENSG00000144645	OSBPL10	2229.84328	3262.3713	1197.315282	-1.445848028	0.056244402	-25.706523	9.88E-146	7.19E-144
ENSG00000249915	PDCD6	82.17856857	120.40738	43.94975717	-1.449943051	0.281034696	-5.1593027	2.48E-07	1.29E-06
ENSG00000280138	AC027290.2	48.19973592	70.690189	25.7092827	-1.451109283	0.344186815	-4.2160513	2.49E-05	0.000104
ENSG00000157456	CCNB2	248.7254919	364.23599	133.2149907	-1.451587904	0.148375286	-9.7832189	1.33E-22	1.64E-21
ENSG00000075218	GTSE1	335.0874503	490.81422	179.3606811	-1.452209139	0.13064837	-11.115402	1.06E-28	1.60E-27
ENSG00000180229	HERC2P3	759.9296731	1113.4493	406.4100296	-1.4534673	0.119662769	-12.146362	6.00E-34	1.06E-32
ENSG00000176438	SYNE3	626.4576017	917.61159	335.3036167	-1.45398079	0.107545347	-13.5197	1.20E-41	2.47E-40
ENSG00000138646	HERC5	106.6779416	156.41134	56.94454686	-1.459362352	0.223675068	-6.5244748	6.82E-11	4.79E-10
ENSG00000080947	CROCCP3	59.96092504	87.985685	31.93616506	-1.461089187	0.356330707	-4.1003741	4.12E-05	0.000166
ENSG00000254810	AP001189.3	30.70062885	45.123285	16.27797317	-1.461274235	0.436375765	-3.3486604	0.000812	0.002651
ENSG00000089685	BIRC5	473.0778264	694.17542	251.9802322	-1.462507354	0.106543909	-13.726804	7.02E-43	1.50E-41
ENSG00000123080	CDKN2C	125.8634967	184.80887	66.91812805	-1.463890814	0.214301843	-6.8309763	8.43E-12	6.29E-11
ENSG00000215158	AC138409.2	14.11492487	20.706127	7.52372291	-1.464261011	0.619889944	-2.3621306	0.01817	0.044017
ENSG00000255153	TOLLIP-AS1	13.35118869	19.638786	7.063591505	-1.465651753	0.626873274	-2.3380352	0.019385	0.046608
ENSG00000230844	ZNF674-AS1	34.15902434	50.161911	18.15613763	-1.467216736	0.422341244	-3.4740077	0.000513	0.001738
ENSG00000242193	CRYZL2P	235.7937693	346.18269	125.4048448	-1.467419149	0.167141379	-8.7795084	1.64E-18	1.73E-17
ENSG00000151690	MFSD6	204.8338527	300.80537	108.8623313	-1.467651925	0.181460112	-8.088014	6.06E-16	5.66E-15
ENSG00000105851	PIK3CG	364.2473391	535.02061	193.4740669	-1.468660615	0.12784647	-11.48769	1.52E-30	2.46E-29
ENSG00000225793	AL080250.1	16.06274593	23.67031	8.455182148	-1.47134265	0.608920717	-2.4163124	0.015679	0.038682
ENSG00000196696	AC009022.1	29.15601042	42.899431	15.41258966	-1.471535427	0.462195663	-3.1837932	0.001454	0.00452
ENSG00000064999	ANKS1A	2484.989835	3654.2736	1315.706118	-1.472879369	0.056027303	-26.2886	2.59E-152	1.99E-150
ENSG00000102904	TSNAXIP1	16.28170106	23.986755	8.576647495	-1.473091507	0.625992263	-2.3532104	0.018612	0.044956
ENSG00000151276	MAGI1	2222.060794	3267.1116	1177.010005	-1.473420716	0.057852165	-25.468722	4.38E-143	3.11E-141
ENSG00000278949	AC127070.4	15.66069513	23.033125	8.288265483	-1.475633487	0.590788182	-2.497737	0.012499	0.031525
ENSG00000091622	PITPNM3	14.96117862	22.046611	7.875746597	-1.477457697	0.603645443	-2.4475588	0.014383	0.03584
ENSG00000223403	MEG9	42.03444675	61.796555	22.27233809	-1.478188157	0.388709546	-3.802809	0.000143	0.000532
ENSG00000178150	ZNF114	28.18215026	41.520187	14.84411307	-1.478299671	0.443710111	-3.331679	0.000863	0.002801
ENSG00000085999	RAD54L	93.16163208	137.08248	49.24078713	-1.478357206	0.241053491	-6.132901	8.63E-10	5.57E-09
ENSG00000175899	A2M	6103.423419	8983.928	3222.918859	-1.478969161	0.117198367	-12.619367	1.65E-36	3.08E-35
ENSG00000070610	GBA2	1895.896247	2792.3492	999.443293	-1.481228358	0.07801979	-18.98529	2.26E-80	8.47E-79
ENSG00000168077	SCARA3	2900.400676	4271.7379	1529.063433	-1.48205957	0.054999198	-26.946931	6.20E-160	5.06E-158
ENSG00000004777	ARHGAP33	61.55062492	90.746985	32.35426447	-1.484537463	0.35205016	-4.2168351	2.48E-05	0.000103
ENSG00000237649	KIFC1	329.7692088	485.94253	173.5958892	-1.486482424	0.129629357	-11.467174	1.93E-30	3.10E-29
ENSG00000177076	ACER2	391.8964246	577.65194	206.140912	-1.486962342	0.124031518	-11.988585	4.08E-33	7.05E-32
ENSG00000129646	QRICH2	130.0531813	191.71772	68.38864173	-1.487045342	0.225291696	-6.6005333	4.10E-11	2.92E-10

ENSG00000008277	ADAM22	74.03652323	109.25014	38.82290159	-1.492881557	0.269451518	-5.5404459	3.02E-08	1.71E-07
ENSG00000204103	MAFB	29.2457156	43.163143	15.3282882	-1.493366069	0.440938609	-3.3867891	0.000707	0.002339
ENSG00000181350	LRRC75A	44.60119384	65.86106	23.3413272	-1.493385141	0.400767018	-3.7263175	0.000194	0.000706
ENSG00000138180	CEP55	546.5796908	807.11867	286.0407096	-1.496913328	0.100566478	-14.884814	4.14E-50	1.02E-48
ENSG00000134242	PTPN22	46.59591858	68.908637	24.2831997	-1.501298768	0.336941986	-4.4556595	8.36E-06	3.70E-05
ENSG00000147113	DIPK2B	11695.87892	17289.917	6101.841316	-1.502590833	0.031802309	-47.247854	0	0
ENSG00000101447	FAM83D	244.3496363	361.36009	127.3391821	-1.503896706	0.151658273	-9.9163513	3.53E-23	4.46E-22
ENSG00000135407	AVIL	20.80590203	30.773112	10.83869185	-1.505449702	0.527910912	-2.8517117	0.004348	0.01215
ENSG00000232931	LINC00342	116.8913479	172.99721	60.78548546	-1.50644946	0.244707494	-6.1561231	7.45E-10	4.84E-09
ENSG00000100399	CHADL	22.46708526	33.206562	11.72760877	-1.506605071	0.504586526	-2.9858211	0.002828	0.008264
ENSG00000121764	HCRTR1	39.01913247	57.636112	20.40215283	-1.50727484	0.38719365	-3.8928191	9.91E-05	0.000378
ENSG00000235505	CASP17P	889.519326	1316.7379	462.3007135	-1.509388864	0.093479241	-16.146781	1.20E-58	3.42E-57
ENSG00000135476	ESPL1	208.8859625	309.66871	108.0852143	-1.514647806	0.175057898	-8.6522677	5.05E-18	5.21E-17
ENSG00000137812	KNL1	409.6275917	607.08856	212.1666228	-1.518256955	0.129179246	-11.753103	6.81E-32	1.15E-30
ENSG00000285796	AL162458.1	30.14994212	44.783782	15.51610213	-1.518461081	0.473444731	-3.2072615	0.00134	0.004194
ENSG00000100027	YPEL1	282.9114959	419.58447	146.2385255	-1.521756337	0.170288309	-8.9363524	4.02E-19	4.32E-18
ENSG00000165194	PCDH19	358.2745934	531.55799	184.9911957	-1.522281219	0.151419332	-10.053414	8.87E-24	1.14E-22
ENSG00000215068	AC025171.2	75.4566623	111.93714	38.97618778	-1.523759939	0.27107246	-5.6212274	1.90E-08	1.09E-07
ENSG00000126803	HSPA2	81.0244958	120.2554	41.79358858	-1.526126485	0.265327251	-5.7518648	8.83E-09	5.23E-08
ENSG00000147852	VLDLR	2834.463202	4209.1054	1459.821008	-1.527936738	0.071057056	-21.502956	1.46E-102	6.94E-101
ENSG00000183287	CCBE1	218.7530667	324.81256	112.6935691	-1.528217349	0.169789764	-9.0006448	2.24E-19	2.44E-18
ENSG00000005108	THSD7A	3378.442223	5021.0857	1735.798779	-1.532906223	0.05385025	-28.466093	3.08E-178	2.82E-176
ENSG00000233429	HOTAIRM1	27.77797697	41.336217	14.21973652	-1.534997044	0.485665573	-3.160605	0.001574	0.004854
ENSG00000233836	AC139769.1	13.17780688	19.630688	6.724925448	-1.535389053	0.639285773	-2.4017257	0.016318	0.040003
ENSG00000213347	MXD3	143.3244788	213.21242	73.43653889	-1.537405035	0.210846681	-7.2915781	3.06E-13	2.49E-12
ENSG00000205444	AL034376.1	41.30277615	61.544495	21.06105711	-1.540755516	0.398741244	-3.8640485	0.000112	0.000422
ENSG00000261490	AC005674.2	28.70734898	42.771994	14.64270404	-1.542828057	0.464914538	-3.3185197	0.000905	0.002927
ENSG00000184292	TACSTD2	6988.106889	10410.995	3565.218323	-1.545784448	0.040309483	-38.347911	0	0
ENSG00000111341	MGP	227.9862005	339.98256	115.9898373	-1.548242187	0.162102198	-9.5510253	1.28E-21	1.54E-20
ENSG00000189058	APOD	2143.144817	3194.4134	1091.876218	-1.548775331	0.069866039	-22.167785	7.03E-109	3.59E-107
ENSG00000109674	NEIL3	35.04723434	52.28741	17.80705832	-1.549411614	0.411474558	-3.7655101	0.000166	0.00061
ENSG00000136014	USP44	44.38481382	66.123044	22.64573398	-1.55143918	0.377783247	-4.1066913	4.01E-05	0.000162
ENSG00000232653	GOLGA8N	32.54892927	48.600523	16.49733508	-1.552985607	0.417890772	-3.7162477	0.000202	0.000733
ENSG00000126878	AIF1L	510.9602642	762.24824	259.6722851	-1.554960659	0.11771162	-13.209916	7.69E-40	1.53E-38
ENSG00000136928	GABBR2	310.7760573	463.85908	157.6930336	-1.558251964	0.15717442	-9.9141576	3.61E-23	4.56E-22
ENSG00000003400	CASP10	1845.124473	2756.2002	934.0487164	-1.559979446	0.076010603	-20.523182	1.34E-93	5.87E-92
ENSG00000245213	AC105285.1	18.63958934	27.855884	9.423294951	-1.561772333	0.541526291	-2.8840194	0.003926	0.011114
ENSG00000198221	AFDN-DT	16.28949384	24.34344	8.235547388	-1.562423221	0.562807217	-2.7761251	0.005501	0.015041
ENSG00000251314	AC104123.1	21.46228746	32.112361	10.81221407	-1.562699519	0.498745417	-3.1332609	0.001729	0.005288
ENSG00000189152	GRAPL	31.30148754	46.700867	15.90210785	-1.563092379	0.431252996	-3.6245369	0.000289	0.001023
ENSG00000107485	GATA3	80.01492238	119.68053	40.34931521	-1.565347201	0.260583176	-6.0070923	1.89E-09	1.18E-08
ENSG00000184465	WDR27	291.9510144	436.60331	147.2987168	-1.567260739	0.183189731	-8.5553963	1.17E-17	1.19E-16
ENSG00000071575	TRIB2	747.2912198	1118.2808	376.3016251	-1.569162719	0.101401971	-15.474677	5.14E-54	1.35E-52
ENSG00000214548	MEG3	2958.978346	4428.8003	1489.156355	-1.572363624	0.454249581	-3.4614531	0.000537	0.001817
ENSG00000245532	NEAT1	3377.572284	5057.0969	1698.047689	-1.574370822	0.396250637	-3.9731692	7.09E-05	0.000277
ENSG00000139174	PRICKLE1	1904.00353	2851.7349	956.2721131	-1.575745049	0.059084622	-26.669292	1.07E-156	8.46E-155
ENSG00000198865	CCDC152	28.96347161	43.350077	14.57686579	-1.576019945	0.431905854	-3.6489895	0.000263	0.000938
ENSG00000147119	CHST7	604.1380451	904.92792	303.3481699	-1.576925756	0.108760748	-14.499034	1.23E-47	2.88E-46
ENSG00000185339	TCN2	2138.024418	3204.1632	1071.885681	-1.57981661	0.059128786	-26.718232	2.89E-157	2.31E-155
ENSG00000185885	IFITM1	43.89549992	65.875281	21.91571915	-1.580351513	0.372597288	-4.2414466	2.22E-05	9.31E-05
ENSG00000165821	SALL2	18.9133529	28.340007	9.486699151	-1.584841888	0.554791114	-2.8566461	0.004281	0.011996
ENSG00000197261	C6orf141	603.7259755	905.62066	301.8312904	-1.585168197	0.11346896	-13.97006	2.37E-44	5.20E-43
ENSG00000131747	TOP2A	1547.53091	2322.9583	772.1035566	-1.588414811	0.064858205	-24.490576	1.86E-132	1.20E-130
ENSG00000108001	EBF3	738.9358346	1109.6132	368.2584515	-1.590114159	0.091543621	-17.370016	1.39E-67	4.52E-66
ENSG00000114771	AADAC	62.22499108	93.531739	30.91824316	-1.591735227	0.295720313	-5.3825698	7.34E-08	4.01E-07
ENSG00000130307	USHBP1	554.7638055	833.38859	276.1390221	-1.593037269	0.128040811	-12.441637	1.55E-35	2.85E-34
ENSG00000135976	ANKRD36	268.4462699	403.42968	133.4628602	-1.593286707	0.185238806	-8.6012577	7.88E-18	8.05E-17
ENSG00000213160	KLHL23	172.1960391	258.79133	85.60074566	-1.59578692	0.185010384	-8.6253911	6.39E-18	6.54E-17
ENSG00000168010	ATG16L2	351.8500308	528.8911	174.8089626	-1.596870037	0.419965433	-3.8023845	0.000143	0.000533
ENSG00000141664	ZCCHC2	1078.884843	1625.1051	532.6645839	-1.608588534	0.081252521	-19.797398	3.13E-87	1.26E-85
ENSG00000266903	AC243964.2	20.47036785	30.882378	10.0583576	-1.608669516	0.526581629	-3.0549291	0.002251	0.006725
ENSG00000203943	SAMD13	34.9815782	52.693027	17.27012971	-1.610200851	0.441855978	-3.6441758	0.000268	0.000954
ENSG00000163884	KLF15	29.72230107	44.838587	14.6060151	-1.610606814	0.462917877	-3.4792495	0.000503	0.001707
ENSG00000143036	SLC44A3	33.36254885	50.275375	16.44972257	-1.610762772	0.401271445	-4.0141475	5.97E-05	0.000235
ENSG00000168078	PBK	154.6305896	233.05147	76.20971113	-1.611864944	0.208507478	-7.7304899	1.07E-14	9.40E-14
ENSG00000186862	PDZD7	29.59722536	44.617585	14.57686579	-1.615093953	0.420903432	-3.8372078	0.000124	0.000468
ENSG00000108465	CDK5RAP3	70.74531637	106.75019	34.7404457	-1.618939369	0.354575457	-4.5658529	4.97E-06	2.26E-05
ENSG00000145990	GFOD1	1831.201596	2765.6468	896.7563841	-1.624152219	0.067006625	-24.238681	8.70E-130	5.35E-128
ENSG00000149573	MPZL2	1603.466085	2423.4332	783.4989943	-1.629667741	0.064182002	-25.391351	3.14E-142	2.21E-140
ENSG00000138336	TET1	27.80588262	41.997942	13.61382319	-1.631182963	0.481807268	-3.3855508	0.00071	0.002348
ENSG00000269929	AL158152.1	13.65475676	20.698276	6.611237204	-1.631261493	0.657512932	-2.4809573	0.013103	0.032935
ENSG00000171132	PRKCE	1548.266919	2341.1784	755.3554783	-1.631455052	0.062367065	-26.15892	7.80E-151	5.94E-149
ENSG00000240731	AL139287.1	12.34775124	18.677058	6.018444022	-1.637323426	0.665266952	-2.4611525	0.013849	0.034638
ENSG00000188626	GOLGA8M	18.51245886	28.053977	8.970940649	-1.637820908	0.541914625	-3.0222859	0.002509	0.007412
ENSG00000177943	MAMDC4	48.05467706	72.802555	23.30679947	-1.640916635	0.370688003	-4.4266786	9.57E-06	4.20E-05
ENSG00000266173	STRADA	65.46632559	99.271	31.661651	-1.644833101	0.318808228	-5.1593182	2.48E-07	1.29E-06
ENSG00000186205	1-Mar	59.09644804	89.49666	28.69623632	-1.645326369	0.381423231	-4.3136501	1.61E-05	6.82E-05
ENSG00000079337	RAPGEF3	1070.88328	1623.3527	518.4138235	-1.647309244	0.099143733	-16.615364	5.39E-62	1.62E-60
ENSG00000272578	AP000347.1	11.51905811	17.464012	5.574104299	-1.647782039	0.68024734	-2.4223278	0.015421	0.038146
ENSG00000206149	HERC2P9	428.0347946	649.52708	206.5425073	-1.651451185	0.153347561	-10.769335	4.80E-27	6.97E-26
ENSG00000205085	FAM71F2	15.55988819	23.654115	7.465661762	-1.652489381	0.603256096	-2.7392834	0.006157	0.01666
ENSG00000215386	MIR99AHG	73.90935186	112.21798	35.60072364	-1.654305462	0.273227921	-6.0546721	1.41E-09	8.92E-09
ENSG00000269821	KCNQ1OT1	70.7937848	107.54241	34.0451607	-1.655154052	0.278963676	-5.9332243	2.97E-09	1.83E-08
ENSG00000121769	FABP3	17.04563713	25.887548	8.20372655	-1.658473336	0.594349717	-2.7903998	0.005264	0.014445
ENSG00000204172	AGAP9	25.77667837	39.209386	12.34397074	-1.65969572	0.503881913	-3.2938188	0.000988	0.003176

ENSG00000253882	AC099548.2	72.09498875	109.52338	34.66659287	-1.66146889	0.294047036	-5.6503508	1.60E-08	9.27E-08
ENSG00000226762	LINC02668	20.31649744	30.889735	9.743260325	-1.661998499	0.565558974	-2.9386829	0.003296	0.009503
ENSG00000002587	HS3ST1	69.71190453	105.93184	33.49196546	-1.663519175	0.285422203	-5.8282753	5.60E-09	3.38E-08
ENSG00000144668	ITGA9	216.5923578	329.31854	103.8661784	-1.66449036	0.182159539	-9.1375416	6.39E-20	7.13E-19
ENSG00000169126	ARMC4	107.5408373	163.62375	51.45792587	-1.66531115	0.229588322	-7.2534663	4.06E-13	3.29E-12
ENSG00000146006	LRRTM2	53.80293466	81.904601	25.70126813	-1.665432671	0.322390463	-5.1658869	2.39E-07	1.24E-06
ENSG00000253616	AC107959.3	23.24076401	35.425873	11.05565509	-1.668393065	0.540918014	-3.0843733	0.00204	0.006145
ENSG00000114166	KAT2B	741.9376715	1129.0012	354.874166	-1.66968784	0.089370115	-18.682843	6.83E-78	2.51E-76
ENSG00000106714	CNTNAP3	903.7287749	1375.2359	432.2216135	-1.67049695	0.079582972	-20.990633	7.99E-98	3.66E-96
ENSG00000138449	SLC40A1	965.6967178	1469.9411	461.452309	-1.67198479	0.083466977	-20.031692	2.92E-89	1.21E-87
ENSG00000143603	KCNN3	268.8196101	409.17837	128.4608539	-1.672161307	0.143247337	-11.673245	1.75E-31	2.90E-30
ENSG00000120129	DUSP1	1187.744823	1808.2938	567.1958788	-1.672236882	0.074276205	-22.513763	3.04E-112	1.64E-110
ENSG00000166912	MTMR10	3964.525615	6041.7285	1887.322703	-1.678205575	0.063739039	-26.329321	8.86E-153	6.83E-151
ENSG00000117399	CDC20	407.4237049	621.3306	193.5168113	-1.681126017	0.122628106	-13.709141	8.95E-43	1.91E-41
ENSG00000173597	SULT1B1	319.733039	487.68794	151.7781374	-1.683621619	0.139827965	-12.040665	2.17E-33	3.79E-32
ENSG00000260912	AL158206.1	247.43997	377.38482	117.4951162	-1.683975527	0.152549219	-11.0389	2.48E-28	3.71E-27
ENSG00000120068	HOXB8	68.8140659	104.95343	32.6747048	-1.684009299	0.278589323	-6.0447733	1.50E-09	9.47E-09
ENSG00000128652	HOXD3	34.0953737	51.97378	16.21696765	-1.685065282	0.410451697	-4.1053924	4.04E-05	0.000163
ENSG00000186166	CCDC84	140.3530148	214.16911	66.53691968	-1.686740028	0.226400955	-7.4502337	9.32E-14	7.83E-13
ENSG00000168758	SEMA4C	471.6245311	719.9301	223.3189632	-1.687419824	0.109706859	-15.38117	2.19E-53	5.70E-52
ENSG00000183856	IQGAP3	487.4812394	744.46235	230.5001257	-1.691798974	0.118269443	-14.304616	2.05E-46	4.69E-45
ENSG00000026036	RTEL1-TNFRSF	17.46335463	26.678042	8.248667542	-1.693523227	0.546829435	-3.0969862	0.001955	0.005913
ENSG00000109458	GAB1	1329.881917	2032.2427	627.5211267	-1.695537639	0.083610753	-20.278942	1.97E-91	8.47E-90
ENSG00000163463	KRTCAP2	29.5686759	45.121458	14.01589344	-1.695707057	0.468253356	-3.6213452	0.000293	0.001034
ENSG00000221995	TIAF1	13.49691865	20.626041	6.367796184	-1.696981089	0.641721964	-2.644418	0.008183	0.021595
ENSG00000112984	KIF20A	513.9238748	785.74415	242.1035976	-1.698586106	0.111136745	-15.283749	9.81E-53	2.51E-51
ENSG00000149809	TM7SF2	255.8324132	391.16402	120.5008059	-1.699446561	0.148334654	-11.456841	2.17E-30	3.48E-29
ENSG00000268471	MIR4453HG	23.76794117	36.395451	11.1404315	-1.699462339	0.502928933	-3.3791302	0.000727	0.002399
ENSG00000137135	ARHGEF39	19.820537	30.323453	9.317621285	-1.699706504	0.528127525	-3.2183638	0.001289	0.004052
ENSG00000239382	ALKBH6	18.34232562	28.063063	8.621588488	-1.700666208	0.626658876	-2.7138628	0.00665	0.017891
ENSG00000015133	CCDC88C	1691.417299	2586.8979	795.936706	-1.700908371	0.067848424	-25.069239	1.08E-138	7.34E-137
ENSG00000147408	CSGALNACT1	3117.356252	4768.1484	1466.564113	-1.701259505	0.053803211	-31.620037	1.96E-219	2.44E-217
ENSG00000066279	ASPM	685.9840602	1050.398	321.5700852	-1.706888304	0.105746803	-16.141276	1.31E-58	3.73E-57
ENSG00000088340	FER1L4	23.30039185	35.714717	10.8860669	-1.710168347	0.55356662	-3.0893632	0.002006	0.006056
ENSG00000071246	VASH1	8348.12238	12792.965	3903.279808	-1.712595161	0.11376829	-15.053361	3.28E-51	8.18E-50
ENSG00000129195	PIMREG	66.31074647	101.64495	30.97654178	-1.713040532	0.292969859	-5.8471562	5.00E-09	3.03E-08
ENSG00000213139	CRYGS	22.5688588	34.561167	10.57655015	-1.716360247	0.539509105	-3.1813369	0.001466	0.004553
ENSG00000251095	AC097478.1	24.09134592	37.023797	11.1588947	-1.720831223	0.492896307	-3.4912642	0.000481	0.001639
ENSG00000283378	CNTNAP3C	19.28396254	29.623462	8.944462864	-1.723245845	0.526481292	-3.2731379	0.001064	0.003397
ENSG00000228526	MIR34AHG	207.7710124	319.02897	96.51305287	-1.723709735	0.198816372	-8.6698581	4.33E-18	4.47E-17
ENSG00000237399	PITRM1-AS1	10.40283013	15.972292	4.833367985	-1.730388713	0.738817534	-2.3421056	0.019175	0.046145
ENSG00000258727	AL135999.1	90.57803844	139.34642	41.80965311	-1.730827738	0.289079511	-5.987376	2.13E-09	1.33E-08
ENSG00000146147	MLIP	46.3780159	71.42242	21.33361207	-1.740716739	0.389390551	-4.470362	7.81E-06	3.47E-05
ENSG00000203288	TDRKH-AS1	13.61601893	21.001736	6.23030168	-1.745822495	0.704204614	-2.4791409	0.01317	0.03309
ENSG00000169744	LDB2	2020.712522	3114.6173	926.807738	-1.748971954	0.062243332	-28.098945	1.01E-173	9.09E-172
ENSG00000267254	AC020928.1	13.97198577	21.570832	6.373139236	-1.751244338	0.667860822	-2.6221696	0.008737	0.022868
ENSG00000122176	FMOD	52.40105226	80.788824	24.01328089	-1.751270766	0.319577582	-5.479955	4.25E-08	2.37E-07
ENSG00000100065	CARD10	1753.004495	2703.0483	802.9606881	-1.751946077	0.06124742	-28.604406	5.92E-180	5.44E-178
ENSG00000015479	MATR3	19.72269493	30.450643	8.994746909	-1.75663495	0.520722822	-3.3734549	0.000742	0.002443
ENSG00000126882	FAM78A	348.8392509	538.56327	159.1152338	-1.758050004	0.139480215	-12.604297	2.00E-36	3.72E-35
ENSG00000173209	AHSA2P	276.6928829	427.16112	126.2246457	-1.758205915	0.521018384	-3.3745564	0.000739	0.002436
ENSG00000165028	NIPSNAP3B	14.18054918	21.884957	6.476141376	-1.76184254	0.673107274	-2.6174766	0.008858	0.023128
ENSG00000157306	ZFHX2-AS1	11.61859817	17.980623	5.256572975	-1.763328747	0.755219127	-2.3348571	0.019551	0.046962
ENSG00000224687	RASAL2-AS1	19.85377574	30.71791	8.989641332	-1.774671605	0.5836177	-3.0408118	0.002359	0.007012
ENSG00000075826	SEC31B	97.36990192	150.84084	43.8989628	-1.780133474	0.561178234	-3.1721356	0.001513	0.004684
ENSG00000261253	AC137932.2	16.5528756	25.634746	7.471004814	-1.781034586	0.579971331	-3.0709011	0.002134	0.006406
ENSG00000140465	CYP1A1	989.0579086	1534.4095	443.7063623	-1.788796511	0.082531247	-21.674173	3.60E-104	1.74E-102
ENSG00000258472	AC005726.1	22.7520951	35.279426	10.22476394	-1.796708713	0.51463041	-3.4912603	0.000481	0.001639
ENSG00000263681	MIR3197	10.06041529	15.623457	4.497373454	-1.800272935	0.726730944	-2.4772207	0.013241	0.033254
ENSG00000173531	MST1	52.43950069	81.55641	23.32259115	-1.806873887	0.396516394	-4.5568706	5.19E-06	2.36E-05
ENSG00000137877	SPTBN5	524.3344713	817.45476	231.2141822	-1.821455244	0.318305644	-5.7223467	1.05E-08	6.18E-08
ENSG00000278500	AC009336.2	14.49499527	22.593045	6.396945495	-1.822552747	0.648591328	-2.8100171	0.004954	0.013699
ENSG00000117650	NEK2	89.96537631	140.26144	39.66931157	-1.824645681	0.259972708	-7.0186047	2.24E-12	1.73E-11
ENSG00000166387	PPFIBP2	18.92052422	29.499827	8.341221054	-1.825986687	0.573425443	-3.1843489	0.001451	0.004513
ENSG00000204403	CASP12	82.70123473	129.05834	36.34413148	-1.831446584	0.267333407	-6.8507958	7.34E-12	5.50E-11
ENSG00000251562	MALAT1	15743.06948	24589.344	6896.795345	-1.834007958	0.227082916	-8.0763802	6.67E-16	6.22E-15
ENSG00000280063	AC012676.5	17.16482872	26.840189	7.489468021	-1.838979462	0.557256255	-3.3000607	0.000967	0.003114
ENSG00000154734	ADAMTS1	93.87597561	146.74404	41.00791127	-1.839992766	0.26616451	-6.9129906	4.75E-12	3.59E-11
ENSG00000113763	UNC5A	252.5103962	394.91139	110.1093981	-1.841654788	0.159325663	-11.559059	6.64E-31	1.09E-29
ENSG00000179399	GPC5	34.52857024	54.033263	15.02387704	-1.844725865	0.423288141	-4.3580854	1.31E-05	5.65E-05
ENSG00000162997	PRORSD1P	10.29885066	16.137254	4.46044704	-1.845258141	0.784476111	-2.3522171	0.018662	0.045047
ENSG00000264112	AC015813.1	92.3814315	144.60694	40.15592076	-1.846010545	0.286640212	-6.440166	1.19E-10	8.23E-10
ENSG00000259820	AC083843.3	10.42211196	16.307253	4.536971395	-1.849050495	0.738642528	-2.503309	0.012304	0.031102
ENSG00000132016	C19orf57	13.83628899	21.630329	6.042250282	-1.849373839	0.646691267	-2.8597477	0.00424	0.011896
ENSG00000262179	MYMX	13.84517004	21.695702	5.994637763	-1.851810083	0.655674967	-2.8242806	0.004739	0.013152
ENSG00000167578	RAB4B	12.14531196	19.028708	5.261916028	-1.852453582	0.671623971	-2.7581707	0.005813	0.015808
ENSG00000214021	TTLL3	201.7402185	316.03917	87.44127128	-1.853679577	0.418858007	-4.425556	9.62E-06	4.22E-05
ENSG00000216775	AL109918.1	217.6723275	341.13839	94.20626962	-1.859646609	0.171092291	-10.86926	1.62E-27	2.36E-26
ENSG00000105519	CAPS	70.89032598	111.31476	30.46588886	-1.860762412	0.300967452	-6.1826035	6.31E-10	4.12E-09
ENSG00000095713	CRTAC1	26.34961061	41.318293	11.38092814	-1.861963758	0.504725451	-3.6890625	0.000225	0.00081
ENSG00000169933	FRMPD4	10.39643805	16.335101	4.457775514	-1.864149518	0.779342951	-2.3919502	0.016759	0.040915
ENSG00000023171	GRAMD1B	176.9778243	277.95187	76.0037776	-1.864987085	0.227013464	-8.2153149	2.12E-16	2.02E-15
ENSG00000177721	ANXA2R	112.8526136	177.10166	48.60356328	-1.868377494	0.236554656	-7.8982909	2.83E-15	2.56E-14
ENSG00000198075	SULT1C4	158.6765301	249.20201	68.15105408	-1.869886661	0.197188731	-9.4827258	2.48E-21	2.94E-20
ENSG00000196689	TRPV1	20.988058	32.917965	9.058151108	-1.870051811	0.524946144	-3.5623689	0.000368	0.001275

ENSG00000254231	AC103760.1	19.21834868	30.161552	8.275145328	-1.871176228	0.532851854	-3.5116256	0.000445	0.001529
ENSG00000268350	FAM156A	8.779521479	13.802406	3.75663714	-1.87279694	0.788207277	-2.3760209	0.0175	0.042571
ENSG00000117707	PROX1	162.4877512	255.38525	69.59025724	-1.875068043	0.1857803	-10.092933	5.94E-24	7.68E-23
ENSG00000204161	TMEM273	107.9597251	169.71762	46.20182785	-1.875762471	0.268654438	-6.982064	2.91E-12	2.23E-11
ENSG00000204177	BMS1P1	22.72986697	35.681981	9.777752689	-1.87664507	0.501883688	-3.7392032	0.000185	0.000673
ENSG00000217930	PAM16	17.62930624	27.703069	7.555543747	-1.87826485	0.581119498	-3.2321491	0.001229	0.003878
ENSG00000244560	AC004890.2	21.18717729	33.32689	9.047465004	-1.879816577	0.605601195	-3.1040503	0.001909	0.005783
ENSG00000007968	E2F2	21.44151306	33.798124	9.084901743	-1.88017292	0.631708537	-2.9763298	0.002917	0.008494
ENSG00000073910	FRY	999.3130023	1571.8757	426.7503446	-1.880666859	0.081173968	-23.168349	9.50E-119	5.38E-117
ENSG00000181019	NQO1	2880.04011	4531.9339	1228.14635	-1.88337209	0.059464167	-31.672387	3.73E-220	4.78E-218
ENSG00000230109	LINC02643	8.860445753	13.945791	3.775100348	-1.887191997	0.789243747	-2.3911396	0.016796	0.041
ENSG00000236017	ASMTL-AS1	29.84610298	47.070539	12.62166665	-1.887818894	0.570053403	-3.3116527	0.000927	0.002995
ENSG00000223705	NSUN5P1	87.22358766	137.44198	37.00519696	-1.888778357	0.311952891	-6.054691	1.41E-09	8.92E-09
ENSG00000275719	AC008622.2	12.37655902	19.512337	5.240781294	-1.897105583	0.659928718	-2.8747129	0.004044	0.011412
ENSG00000138678	GPAT3	92.93385769	146.79312	39.07459467	-1.902411907	0.267723471	-7.1058839	1.20E-12	9.40E-12
ENSG00000245552	AP000787.1	16.9418019	26.751265	7.132338757	-1.904484925	0.573510122	-3.3207521	0.000898	0.002905
ENSG00000228672	PROB1	665.1446218	1050.0947	280.1945607	-1.905169388	0.103153154	-18.469328	3.65E-76	1.32E-74
ENSG00000171522	PTGER4	397.7816021	628.20316	167.3600424	-1.908056287	0.139467206	-13.681039	1.32E-42	2.80E-41
ENSG00000091409	ITGA6	18050.71248	28528.843	7572.582136	-1.913676792	0.035797912	-53.457777	0	0
ENSG00000197565	COL4A6	48.13643262	76.079626	20.19323955	-1.914508206	0.34974512	-5.4740098	4.40E-08	2.44E-07
ENSG00000111644	ACRBP	14.24879136	22.558335	5.939248142	-1.915341603	0.664395243	-2.8828346	0.003941	0.011151
ENSG00000260855	AL591848.4	14.36065057	22.681722	6.039578756	-1.915895932	0.644921221	-2.9707441	0.002971	0.008634
ENSG00000231160	KLF3-AS1	11.69100818	18.461438	4.920578444	-1.918284893	0.709072911	-2.7053422	0.006823	0.018318
ENSG00000186193	SAPCD2	150.8291492	238.71059	62.94770953	-1.91832877	0.212133071	-9.0430444	1.52E-19	1.68E-18
ENSG00000267583	AC007998.3	351.0752276	555.943	146.2074524	-1.925882233	0.142623558	-13.503255	1.50E-41	3.09E-40
ENSG00000168970	JMJD7-PLA2G4	8.132625451	12.894892	3.370358565	-1.934784367	0.825073214	-2.3449851	0.019028	0.045833
ENSG00000004468	CD38	84.75149077	134.4025	35.10048397	-1.943690916	0.276631036	-7.0262937	2.12E-12	1.64E-11
ENSG00000264175	MIR3189	9.002222073	14.313883	3.690561415	-1.944980207	0.837163588	-2.3232977	0.020163	0.048228
ENSG00000203727	SAMD5	70.95212346	112.55763	29.3466157	-1.946053099	0.315647146	-6.1652802	7.04E-10	4.57E-09
ENSG00000250271	AC068647.2	19.32838456	30.775679	7.881089649	-1.961908102	0.593124586	-3.3077504	0.00094	0.003034
ENSG00000215183	MSMP	88.9615323	141.61052	36.31254811	-1.965434154	0.259423237	-7.5761685	3.56E-14	3.05E-13
ENSG00000233225	AC004987.2	11.8591243	18.863746	4.854502719	-1.965557691	0.699496563	-2.8099605	0.004955	0.013699
ENSG00000139631	CSAD	81.82572906	130.38396	33.26749798	-1.965720511	0.307700807	-6.3884152	1.68E-10	1.14E-09
ENSG00000224975	INE1	16.70088908	26.618792	6.782986596	-1.967116799	0.632747951	-3.1088474	0.001878	0.005702
ENSG00000065618	COL17A1	30.27627892	48.22705	12.32550753	-1.967976185	0.430569119	-4.5706394	4.86E-06	2.22E-05
ENSG00000117616	RSRP1	558.7678281	890.11778	227.4178763	-1.968754022	0.141737297	-13.890162	7.27E-44	1.57E-42
ENSG00000169682	SPNS1	19.35493085	30.826338	7.8835237	-1.970146985	0.551518159	-3.5722251	0.000354	0.001232
ENSG00000245849	RAD51-AS1	33.40470353	53.251211	13.55819609	-1.973441607	0.4203387	-4.6948844	2.67E-06	1.25E-05
ENSG00000106351	AGFG2	1442.41505	2299.2559	585.5741812	-1.973608408	0.070922986	-27.827486	2.02E-170	1.78E-168
ENSG00000100918	REC8	320.2129235	510.51003	129.9158134	-1.974510553	0.141164201	-13.987332	1.86E-44	4.09E-43
ENSG00000146021	KLHL3	312.1381065	498.13817	126.1380415	-1.983817426	0.138897508	-14.282599	2.81E-46	6.40E-45
ENSG00000279342	AP000866.6	13.8702551	22.161063	5.579447351	-1.988170231	0.664679098	-2.9911731	0.002779	0.008131
ENSG00000204305	AGER	65.89677113	105.37267	26.42086971	-1.988891611	0.338560402	-5.8745547	4.24E-09	2.58E-08
ENSG00000110900	TSPAN11	1234.663761	1972.4661	496.8613952	-1.990455018	0.077174144	-25.791734	1.10E-146	8.09E-145
ENSG00000040608	RTN4R	12.11946854	19.349942	4.888995082	-1.990693674	0.710600726	-2.8014236	0.005088	0.014031
ENSG00000242689	CNTF	8.459442824	13.524721	3.394164824	-1.992438931	0.843045452	-2.3633826	0.018109	0.043887
ENSG00000254827	SLC22A18AS	22.57873385	36.070167	9.08730042	-1.996445076	0.514049022	-3.883764	0.000103	0.000391
ENSG00000167874	TMEM88	197.686504	316.17993	79.19307869	-1.997869163	0.177444848	-11.259099	2.09E-29	3.25E-28
ENSG00000130054	FAM155B	106.9680496	171.2477	42.68839425	-1.997991087	0.251551294	-7.9426786	1.98E-15	1.80E-14
ENSG00000143850	PLEKHA6	133.4458281	213.74334	53.14831178	-2.00751771	0.214735438	-9.3487955	8.87E-21	1.03E-19
ENSG00000156509	FBXO43	12.3900964	19.827794	4.952399282	-2.007541081	0.722336328	-2.7792332	0.005449	0.014909
ENSG00000162595	DIRAS3	259.2557082	415.26789	103.2435235	-2.008755693	0.153065583	-13.123497	2.42E-39	4.75E-38
ENSG00000198542	ITGBL1	291.8021823	468.01903	115.585333	-2.015945959	0.143242555	-14.073653	5.51E-45	1.22E-43
ENSG00000220785	MTMR9LP	562.5913623	902.16628	223.0164403	-2.01605816	0.260550123	-7.737698	1.01E-14	8.89E-14
ENSG00000157551	KCNJ15	70.78137707	113.54696	28.0157931	-2.016322838	0.302554888	-6.6643208	2.66E-11	1.92E-10
ENSG00000102387	TAF7L	14.01217734	22.510983	5.513371626	-2.016743391	0.678201548	-2.9736638	0.002943	0.008563
ENSG00000279500	AC108704.2	15.96334295	25.627637	6.299048932	-2.017215459	0.612057021	-3.2957966	0.000981	0.003155
ENSG00000167861	HID1	1660.221379	2662.6124	657.8303101	-2.01724578	0.069274677	-29.119526	2.03E-186	1.96E-184
ENSG00000137727	ARHGAP20	63.45234546	101.80132	25.10336937	-2.018049406	0.304876946	-6.619226	3.61E-11	2.58E-10
ENSG00000279759	AC118344.2	56.77793328	91.159119	22.39674781	-2.022904123	0.335524992	-6.0290714	1.65E-09	1.04E-08
ENSG00000275896	PRSS2	7.56898074	12.148538	2.989423041	-2.02426648	0.855936806	-2.3649719	0.018031	0.043716
ENSG00000159588	CCDC17	9.64919945	15.491478	3.806921185	-2.027877434	0.82245188	-2.4656487	0.013677	0.034239
ENSG00000262903	AC027796.4	22.85137166	36.747594	8.955148968	-2.029398101	0.539768421	-3.759757	0.00017	0.000624
ENSG00000230747	AC021188.1	11.4403174	18.391276	4.489358876	-2.030032255	0.744785694	-2.7256596	0.006417	0.017309
ENSG00000280434	AL031595.3	29.74784556	47.881771	11.61392053	-2.038562734	0.458287152	-4.4482214	8.66E-06	3.82E-05
ENSG00000270547	LINC01235	1803.673099	2901.796	705.550158	-2.040628328	0.070234485	-29.054507	1.35E-185	1.29E-183
ENSG00000184786	TCTE3	7.678497325	12.3649	2.992094567	-2.044114637	0.84578352	-2.4168296	0.015656	0.038638
ENSG00000237813	AC002066.1	10.59966438	17.080219	4.119109457	-2.045534672	0.738698338	-2.7691069	0.005621	0.015353
ENSG00000277511	AC116407.2	13.49656101	21.749669	5.24345282	-2.047494567	0.653593716	-3.1326717	0.001732	0.005297
ENSG00000102935	ZNF423	164.6137904	265.69851	63.5290688	-2.063753131	0.202204225	-10.206281	1.86E-24	2.46E-23
ENSG00000182600	SNORC	13.50768617	21.801069	5.214303509	-2.064351427	0.651065691	-3.1707268	0.001521	0.004706
ENSG00000071282	LMCD1	101.1474754	163.35836	38.93658984	-2.06812363	0.261847531	-7.898198	2.83E-15	2.56E-14
ENSG00000128833	MYO5C	1920.650053	3102.3728	738.9272593	-2.070461839	0.068504205	-30.223865	1.15E-200	1.29E-198
ENSG00000247157	LINC01252	10.64090869	17.231455	4.050362205	-2.076331157	0.751426766	-2.763185	0.005724	0.015599
ENSG00000177842	ZNF620	95.83207313	155.08483	36.57932044	-2.081755971	0.265699269	-7.8350083	4.69E-15	4.20E-14
ENSG00000154258	ABCA9	409.5235217	662.55484	156.4922012	-2.081775255	0.141972567	-14.663222	1.11E-48	2.65E-47
ENSG00000274422	AC245060.5	10.80765154	17.501537	4.113766405	-2.084039936	0.720378663	-2.8929784	0.003816	0.010829
ENSG00000227338	AL139280.1	20.56607984	33.285562	7.846597286	-2.087455243	0.534604819	-3.9046697	9.44E-05	0.000361
ENSG00000134215	VAV3	337.3188852	546.06083	128.5769409	-2.08809694	0.15187349	-13.748923	5.17E-43	1.11E-41
ENSG00000107165	TYRP1	7.884710613	12.758863	3.010557774	-2.089673869	0.872375463	-2.3953836	0.016603	0.040583
ENSG00000247982	LINC00926	18.59142639	30.172217	7.010635934	-2.091875038	0.582468794	-3.5913942	0.000329	0.001151
ENSG00000171724	VAT1L	811.908409	1315.9599	307.8568813	-2.094861063	0.105902087	-19.781112	4.33E-87	1.74E-85
ENSG00000196169	KIF19	333.3863817	540.47774	126.2950199	-2.096006319	0.1544055	-13.574687	5.66E-42	1.18E-40
ENSG00000124593	AL365205.1	39.45561842	64.00372	14.90751726	-2.10162143	0.391273458	-5.3712343	7.82E-08	4.25E-07
ENSG00000158258	CLSTN2	35.59923175	57.801568	13.39689533	-2.10326526	0.405748908	-5.1836622	2.18E-07	1.14E-06

ENSG00000196329	GIMAP5	87.37464276	141.82871	32.92057987	-2.103665438	0.282692877	-7.4415226	9.95E-14	8.35E-13
ENSG00000126217	MCF2L	43.82362127	71.136787	16.51045524	-2.107323993	0.380251467	-5.5419221	2.99E-08	1.69E-07
ENSG00000114656	KIAA1257	35.87915782	58.242389	13.51592662	-2.107672291	0.402324433	-5.238738	1.62E-07	8.57E-07
ENSG00000116396	KCNC4	521.0903182	846.65245	195.5281832	-2.111050466	0.121319229	-17.40079	8.14E-68	2.65E-66
ENSG00000135218	CD36	78.63286221	127.80515	29.46057679	-2.113099801	0.272366105	-7.7583068	8.61E-15	7.59E-14
ENSG00000186185	KIF18B	157.4810966	255.94896	59.01323214	-2.113740608	0.217698545	-9.7094843	2.75E-22	3.37E-21
ENSG00000138030	KHK	77.29884655	125.52634	29.07135384	-2.114006803	0.293563973	-7.2011793	5.97E-13	4.79E-12
ENSG00000114378	HYAL1	29.10532286	47.276729	10.93391689	-2.11802466	0.448589677	-4.721519	2.34E-06	1.10E-05
ENSG00000215252	GOLGA8B	90.54698157	147.21839	33.87557251	-2.120307381	0.294804415	-7.1922511	6.37E-13	5.11E-12
ENSG00000129173	E2F8	102.1309974	166.06347	38.19852505	-2.120687803	0.238930124	-8.8757657	6.95E-19	7.41E-18
ENSG00000185513	L3MBTL1	40.97304352	66.700141	15.24594585	-2.124434215	0.429095929	-4.950954	7.39E-07	3.68E-06
ENSG00000130768	SMPDL3B	15.25379315	24.803765	5.7038217	-2.125943951	0.727714207	-2.9213995	0.003485	0.009982
ENSG00000182310	SPACA6	66.79796613	108.83387	24.76206716	-2.1282491	0.368020546	-5.7829627	7.34E-09	4.39E-08
ENSG00000178404	CEP295NL	20.01634469	32.641118	7.391571458	-2.129921989	0.56809303	-3.7492486	0.000177	0.000649
ENSG00000172572	PDE3A	90.70244798	147.57937	33.82552594	-2.131328581	0.262597342	-8.1163372	4.80E-16	4.50E-15
ENSG00000279803	AC009090.5	12.04197243	19.639527	4.444417883	-2.131831251	0.748457991	-2.8482978	0.004395	0.012274
ENSG00000271978	AL359643.2	44.53382305	72.501564	16.56608234	-2.132797409	0.36834044	-5.7902885	7.03E-09	4.21E-08
ENSG00000241404	EGFL8	8.053051525	13.119352	2.986751515	-2.132835045	0.867626982	-2.4582396	0.013962	0.034879
ENSG00000228393	LINC01004	8.054016738	13.11861	2.989423041	-2.132883825	0.836711377	-2.5491273	0.010799	0.027637
ENSG00000102575	ACP5	11.20277885	18.228387	4.177170604	-2.134895782	0.743380978	-2.871873	0.00408	0.011499
ENSG00000132170	PPARG	163.8947685	267.1967	60.59283881	-2.138109294	0.190637808	-11.215557	3.42E-29	5.28E-28
ENSG00000213903	LTB4R	88.78669477	144.8671	32.70628816	-2.14507525	0.352631153	-6.0830566	1.18E-09	7.52E-09
ENSG00000172794	RAB37	10.10955377	16.531218	3.687889888	-2.150171039	0.769903826	-2.7927787	0.005226	0.014359
ENSG00000186479	RGS7BP	83.39526076	136.19224	30.59827779	-2.150492118	0.272693005	-7.8861287	3.12E-15	2.81E-14
ENSG00000088756	ARHGAP28	21.44940492	35.046869	7.851940338	-2.157062669	0.517935312	-4.1647337	3.12E-05	0.000128
ENSG00000038295	TLL1	216.7043681	354.13064	79.27809257	-2.163164963	0.174004052	-12.431693	1.76E-35	3.22E-34
ENSG00000111788	AC009533.1	17.65146059	28.834794	6.468126798	-2.16392438	0.629560645	-3.4371977	0.000588	0.001974
ENSG00000118407	FILIP1	2260.826679	3699.6861	821.9672715	-2.169502062	0.066370045	-32.68797	2.32E-234	3.29E-232
ENSG00000242028	HYPK	20.7120731	33.963827	7.46031871	-2.177290452	0.558873882	-3.8958529	9.79E-05	0.000373
ENSG00000185352	HS6ST3	10.42825449	17.078737	3.777771874	-2.178702398	0.819668324	-2.6580293	0.00786	0.020796
ENSG00000128408	RIBC2	10.51348905	17.196251	3.830727444	-2.179513772	0.81687054	-2.6681263	0.007628	0.020237
ENSG00000229372	SZT2-AS1	37.40399783	61.343254	13.46564258	-2.18331061	0.411561726	-5.3049408	1.13E-07	6.04E-07
ENSG00000124839	RAB17	17.84815742	29.2571	6.439214962	-2.189281457	0.625290332	-3.5012239	0.000463	0.001584
ENSG00000166292	TMEM100	11.53746584	18.940031	4.134901138	-2.191599949	0.749440114	-2.9243163	0.003452	0.009898
ENSG00000091137	SLC26A4	54.123374	88.757318	19.48942965	-2.191994194	0.337316636	-6.4983282	8.12E-11	5.68E-10
ENSG00000213928	IRF9	39.51436766	64.928268	14.10046775	-2.193729504	0.480744334	-4.5631937	5.04E-06	2.29E-05
ENSG00000273373	AL355488.1	8.304134263	13.610831	2.997437619	-2.194048173	0.905503953	-2.4230134	0.015392	0.038084
ENSG00000214826	DDX12P	20.94018446	34.401587	7.478781917	-2.1988653	0.551792462	-3.9849499	6.75E-05	0.000264
ENSG00000106511	MEOX2	1502.546515	2467.5227	537.5703697	-2.200253208	0.086024088	-25.577176	2.74E-144	1.98E-142
ENSG00000130590	SAMD10	120.3213338	197.82774	42.8149298	-2.207050757	0.239394672	-9.2192977	2.99E-20	3.39E-19
ENSG00000279861	AC073548.1	10.43548413	17.177735	3.693232941	-2.207844049	0.789132955	-2.79781	0.005145	0.014172
ENSG00000278899	AL358852.1	7.381308021	12.148785	2.61383057	-2.216875099	0.916444421	-2.4189957	0.015563	0.038429
ENSG00000113083	LOX	9184.425137	15129.436	3239.414773	-2.223552178	0.08461959	-26.277038	3.51E-152	2.68E-150
ENSG00000115252	PDE1A	7.535956671	12.392007	2.679906295	-2.223904681	0.922480708	-2.4107872	0.015918	0.039188
ENSG00000001561	ENPP4	62.41954865	102.82038	22.01872129	-2.223917022	0.320496029	-6.9389846	3.95E-12	3.01E-11
ENSG00000283445	AL136985.3	75.73805469	124.81741	26.65869482	-2.22471257	0.329277915	-6.756337	1.42E-11	1.04E-10
ENSG00000188732	FAM221A	70.14915437	115.58413	24.71418179	-2.225179958	0.302487968	-7.3562594	1.89E-13	1.56E-12
ENSG00000128573	FOXP2	53.32909053	87.90925	18.74893081	-2.228513292	0.356506383	-6.2509773	4.08E-10	2.70E-09
ENSG00000258315	C17orf49	9.685724423	15.974612	3.39683635	-2.237201413	0.781148166	-2.8639911	0.004183	0.011747
ENSG00000137960	GIPC2	365.8747528	603.95043	127.799076	-2.240816727	0.132248016	-16.944048	2.13E-64	6.57E-63
ENSG00000152270	PDE3B	11.85300081	19.547294	4.158707397	-2.244754087	0.7090794	-3.1657302	0.001547	0.004777
ENSG00000175567	UCP2	203.98327	336.94753	71.01901178	-2.246098929	0.173182281	-12.969565	1.82E-38	3.53E-37
ENSG00000165244	ZNF367	83.68600643	138.26857	29.10344753	-2.248743339	0.276071553	-8.1455091	3.78E-16	3.56E-15
ENSG00000135312	HTR1B	117.8857371	194.73222	41.03925716	-2.251124078	0.266982603	-8.4317257	3.41E-17	3.37E-16
ENSG00000279289	AL136164.3	8.620837801	14.297194	2.944482048	-2.256937206	0.863581785	-2.6134609	0.008963	0.023378
ENSG00000211445	GPX3	802.2371149	1327.1022	277.3719835	-2.258263413	0.095134782	-23.737516	1.48E-124	8.65E-123
ENSG00000176046	NUPR1	3876.538908	6413.9912	1339.086665	-2.259788734	0.049386645	-45.757082	0	0
ENSG00000174370	C11orf45	112.799013	186.68744	38.910587	-2.260866152	0.257331094	-8.7858258	1.55E-18	1.64E-17
ENSG00000141655	TNFRSF11A	214.0554773	354.19024	73.92071403	-2.26093227	0.171178239	-13.208059	7.88E-40	1.57E-38
ENSG00000254996	ANKHD1-EIF4E	8.743320177	14.494546	2.992094567	-2.267020279	0.835636726	-2.7129256	0.006669	0.017938
ENSG00000203709	MIR29B2CHG	54.6262141	90.538227	18.71420097	-2.269106315	0.365507834	-6.2080921	5.36E-10	3.52E-09
ENSG00000185495	AC138393.1	21.94903016	36.424384	7.47367634	-2.275869716	0.56211546	-4.0487584	5.15E-05	0.000205
ENSG00000064692	SNCAIP	799.0550589	1325.5514	272.5587296	-2.282868467	0.104941572	-21.753709	6.37E-105	3.12E-103
ENSG00000101331	CCM2L	400.6430685	665.12392	136.162215	-2.285752489	0.13181984	-17.339973	2.35E-67	7.60E-66
ENSG00000231784	DBIL5P	12.23372912	20.311422	4.156035871	-2.294793026	0.710914105	-3.227947	0.001247	0.003928
ENSG00000269086	AC008555.2	22.90767547	38.145846	7.669504841	-2.295076886	0.579517929	-3.9603208	7.48E-05	0.000291
ENSG00000242294	STAG3L5P	110.240057	183.40617	37.07394421	-2.303004199	0.300971882	-7.6518915	1.98E-14	1.72E-13
ENSG00000112414	ADGRG6	2771.305738	4613.9108	928.7006951	-2.312531404	0.053651557	-43.102783	0	0
ENSG00000182685	BRICD5	41.25682474	68.759625	13.75402459	-2.312946453	0.405896098	-5.6983708	1.21E-08	7.08E-08
ENSG00000213901	SLC23A3	6.744803712	11.243592	2.246015201	-2.313812692	0.955705932	-2.4210509	0.015476	0.038244
ENSG00000279425	AC092279.2	6.69905789	11.152101	2.246015201	-2.316544352	0.95753269	-2.4192849	0.015551	0.038404
ENSG00000140398	NEIL1	170.7417804	284.73433	56.74922869	-2.323130981	0.220668387	-10.527702	6.44E-26	9.02E-25
ENSG00000279722	AC007342.7	12.45895549	20.780338	4.137572664	-2.327875924	0.73017742	-3.1880963	0.001432	0.004458
ENSG00000167779	IGFBP6	105.4784218	175.97566	34.98117982	-2.332996302	0.653064563	-3.5723823	0.000354	0.001232
ENSG00000142405	NLRP12	102.9761118	171.84075	34.11147391	-2.33348932	0.252858261	-9.228448	2.75E-20	3.12E-19
ENSG00000163995	ABLIM2	14.76871111	24.629964	4.907458289	-2.336193183	0.644953382	-3.6222667	0.000292	0.001031
ENSG00000085465	OVGP1	14.76091734	24.712273	4.809561726	-2.352819797	0.700097816	-3.3607015	0.000777	0.002548
ENSG00000261326	LINC01355	19.29745883	32.346153	6.248764887	-2.357844765	0.647107391	-3.6436684	0.000269	0.000956
ENSG00000186767	SPIN4	122.9835487	205.83475	40.13235198	-2.357847981	0.236126026	-9.9855489	1.76E-23	2.25E-22
ENSG00000181444	ZNF467	26.50715445	44.313287	8.701021844	-2.358280268	0.500090392	-4.715708	2.41E-06	1.13E-05
ENSG00000262580	AC087741.1	48.65640737	81.413616	15.89919885	-2.359995991	0.422091218	-5.591199	2.26E-08	1.29E-07
ENSG00000196196	HRCT1	50.0703728	83.899699	16.24104676	-2.360981349	0.394802664	-5.9801556	2.23E-09	1.39E-08
ENSG00000261556	SMG1P7	37.11993417	62.173831	12.06603736	-2.364731812	0.511486223	-4.6232561	3.78E-06	1.74E-05
ENSG00000257178	AC103702.1	9.19193558	15.418254	2.965616782	-2.366149991	0.815365089	-2.9019516	0.003708	0.010555
ENSG00000140009	ESR2	27.67713912	46.322605	9.031673323	-2.366362211	0.480912756	-4.9205645	8.63E-07	4.27E-06

ENSG00000188603	CLN3	8.195724824	13.738021	2.65342851	-2.387219244	0.873381946	-2.733305	0.00627	0.01695
ENSG00000263155	MYZAP	143.5674914	241.35176	45.78321811	-2.399900727	0.214819403	-11.171713	5.61E-29	8.56E-28
ENSG00000151006	PRSS53	20.13526523	33.825972	6.444558014	-2.401335737	0.604552655	-3.972087	7.12E-05	0.000278
ENSG00000002079	MYH16	9.361933818	15.758251	2.965616782	-2.402106137	0.824213381	-2.9144226	0.003563	0.010185
ENSG00000232070	TMEM253	6.95479252	11.726974	2.182611001	-2.402162646	0.984042322	-2.4411172	0.014642	0.036406
ENSG00000235313	HM13-IT1	5.904562738	9.9413743	1.867751204	-2.402225536	1.022364072	-2.3496772	0.01879	0.045307
ENSG00000224934	AL391684.1	5.857357637	9.8496356	1.865079677	-2.406484905	1.032578691	-2.3305584	0.019777	0.047467
ENSG00000137491	SLCO2B1	35.56140979	59.910969	11.21185027	-2.415312808	0.434947391	-5.5531148	2.81E-08	1.59E-07
ENSG00000276710	CSPG4P10	15.31082826	25.835901	4.785755467	-2.415818078	0.681117725	-3.5468437	0.00039	0.001348
ENSG00000274737	AC004466.2	5.947565648	10.030052	1.865079677	-2.421555066	1.008246546	-2.4017489	0.016317	0.040003
ENSG00000175772	LINC01106	15.25189568	25.78144	4.722351267	-2.424296863	0.715916946	-3.3862823	0.000708	0.002343
ENSG00000226197	AL583785.1	16.76777772	28.297446	5.238109768	-2.425948763	0.629912014	-3.8512502	0.000118	0.000443
ENSG00000110169	HPX	7.173350874	12.124493	2.222208942	-2.427035815	0.999547364	-2.4281349	0.015177	0.037607
ENSG00000272909	AL122035.2	6.012620043	10.157489	1.867751204	-2.428825644	1.035423211	-2.3457323	0.01899	0.045759
ENSG00000255142	AP006621.2	8.46723954	14.259916	2.674563243	-2.430056975	0.926821499	-2.6219256	0.008743	0.022881
ENSG00000237232	ZNF295-AS1	13.17405236	22.252802	4.095303197	-2.433375583	0.707715269	-3.4383539	0.000585	0.001967
ENSG00000176244	ACBD7	39.96247557	67.501547	12.4234041	-2.442427747	0.409771222	-5.9604668	2.52E-09	1.56E-08
ENSG00000203886	LIME1	24.4305011	41.244726	7.616276421	-2.447103924	0.613333998	-3.989839	6.61E-05	0.000259
ENSG00000165092	ALDH1A1	2201.020115	3720.4755	681.5647192	-2.447977475	0.071163603	-34.399291	2.58E-259	4.20E-257
ENSG00000114654	EFCC1	128.8319768	217.84913	39.81482066	-2.455448895	0.234552968	-10.468633	1.20E-25	1.67E-24
ENSG00000261087	AP003469.4	8.438591111	14.313636	2.563546525	-2.462533375	0.887477631	-2.7747554	0.005524	0.015097
ENSG00000163075	CFAP221	20.69436705	35.065879	6.322855191	-2.463551921	0.554700503	-4.4412289	8.94E-06	3.94E-05
ENSG00000207721	MIR186	6.152746168	10.398143	1.907349144	-2.463805579	1.051739458	-2.3426007	0.01915	0.046102
ENSG00000240583	AQP1	18.17802376	30.845347	5.510700099	-2.468113387	0.616679098	-4.0022653	6.27E-05	0.000247
ENSG00000088992	TESC	12.29099588	20.851832	3.730159355	-2.472231967	0.731665525	-3.37891	0.000728	0.0024
ENSG00000100336	APOL4	34.54975594	58.530739	10.56877305	-2.472249483	0.4355403	-5.6762818	1.38E-08	8.01E-08
ENSG00000179546	HTR1D	382.1183888	647.34761	116.8891675	-2.472870066	0.134906187	-18.330294	4.74E-75	1.69E-73
ENSG00000089692	LAG3	7.333152295	12.46523	2.201074208	-2.476350293	0.971962928	-2.5477827	0.010841	0.027728
ENSG00000120885	CLU	15289.43233	25940.784	4638.080899	-2.483345868	0.036638449	-67.779777	0	0
ENSG00000212694	LINC01089	72.07660775	122.32545	21.82776089	-2.487575529	0.356211279	-6.9834272	2.88E-12	2.21E-11
ENSG00000138722	MMRN1	51965.24779	88219.906	15710.58916	-2.489396757	0.030878279	-80.619674	0	0
ENSG00000158106	RHPN1	467.4783318	793.94749	141.0091781	-2.490134578	0.152900135	-16.28602	1.24E-59	3.62E-58
ENSG00000168306	ACOX2	11.26555284	19.155404	3.375701617	-2.496819951	0.765983093	-3.259628	0.001116	0.003548
ENSG00000199161	MIR126	81.09541904	137.86537	24.32546916	-2.497814049	0.316441866	-7.8934374	2.94E-15	2.66E-14
ENSG00000240053	LY6G5B	13.89453777	23.609233	4.17984213	-2.504526386	0.713602253	-3.5096952	0.000449	0.001539
ENSG00000106484	MEST	39.01028901	66.285192	11.73538588	-2.507101667	0.417704755	-6.0020903	1.95E-09	1.22E-08
ENSG00000086696	HSD17B2	3247.952354	5527.7673	968.1373579	-2.512320331	0.056261029	-44.654717	0	0
ENSG00000121716	PILRB	43.7170173	74.534435	12.89960003	-2.51648744	0.436963895	-5.7590283	8.46E-09	5.02E-08
ENSG00000166924	NYAP1	95.79229713	163.10309	28.48150503	-2.517910026	0.256954459	-9.7990517	1.14E-22	1.41E-21
ENSG00000164116	GUCY1A1	12.62604497	21.487438	3.764651719	-2.518560322	0.748266476	-3.3658602	0.000763	0.002503
ENSG00000177685	CRACR2B	307.7892152	524.96458	90.61384225	-2.5362033	0.154652974	-16.399319	1.93E-60	5.67E-59
ENSG00000204136	GGTA1P	16.5653914	28.268266	4.862517297	-2.538630651	0.6625754	-3.8314593	0.000127	0.000477
ENSG00000167524	RSKR	41.24541878	70.364068	12.12677003	-2.544893643	0.43661285	-5.8287191	5.59E-09	3.37E-08
ENSG00000183668	PSG9	23.35300942	39.867405	6.838613693	-2.552584066	0.600942211	-4.2476365	2.16E-05	9.07E-05
ENSG00000266777	SH3GL1P1	18.36173204	31.42195	5.301513968	-2.572628514	0.662773443	-3.8816107	0.000104	0.000394
ENSG00000159640	ACE	1885.869421	3230.8419	540.8969848	-2.577665697	0.08474941	-30.415146	3.46E-203	4.01E-201
ENSG00000205038	PKHD1L1	155.3461182	266.28454	44.407692	-2.581285341	0.20520771	-12.578891	2.76E-36	5.12E-35
ENSG00000246922	UBAP1L	17.20319026	29.506937	4.899443711	-2.593862081	0.612687164	-4.2335832	2.30E-05	9.62E-05
ENSG00000242085	RPS20P33	6.616332844	11.341108	1.891557463	-2.593945245	0.988932528	-2.6229749	0.008717	0.022824
ENSG00000129048	ACKR4	10.56054368	18.11053	3.010557774	-2.599271409	0.818067844	-3.1773299	0.001486	0.00461
ENSG00000266967	AARSD1	10.62840855	18.267394	2.989423041	-2.601162368	0.841176145	-3.0922921	0.001986	0.005999
ENSG00000276550	HERC2P2	1323.636421	2274.5804	372.69248	-2.609411849	0.407880983	-6.3974835	1.58E-10	1.08E-09
ENSG00000250067	YJEFN3	10.76298513	18.491606	3.034364034	-2.610022212	0.83873499	-3.1118556	0.001859	0.005654
ENSG00000267767	LINC01801	6.544011787	11.267885	1.820138685	-2.61432304	1.036258589	-2.5228481	0.011641	0.02957
ENSG00000074317	SNCB	75.65646063	130.2147	21.098221	-2.618579119	0.32053037	-8.1695195	3.10E-16	2.93E-15
ENSG00000143816	WNT9A	262.9639482	452.62107	73.30682149	-2.625192127	0.17352704	-15.128433	1.05E-51	2.65E-50
ENSG00000108602	ALDH3A1	11.97291505	20.66001	3.285819632	-2.636012984	0.796894589	-3.3078565	0.00094	0.003034
ENSG00000166689	PLEKHA7	8.176812032	14.099594	2.254029779	-2.638252675	0.947598926	-2.7841449	0.005367	0.0147
ENSG00000280152	AC009078.3	57.15235096	98.551012	15.75368977	-2.645496075	0.735580621	-3.5964733	0.000323	0.001129
ENSG00000120875	DUSP4	570.0721824	983.44761	156.6967567	-2.648685372	0.119418317	-22.179892	5.37E-109	2.75E-107
ENSG00000120833	SOCS2	491.5561091	847.98183	135.1303898	-2.649454206	0.117464141	-22.55543	1.19E-112	6.41E-111
ENSG00000262877	AC110285.2	10.96857386	18.931933	3.005214722	-2.64977071	0.868610986	-3.0505839	0.002284	0.006815
ENSG00000072041	SLC6A15	35.74598811	61.600535	9.891440933	-2.649780482	0.460306764	-5.7565534	8.58E-09	5.09E-08
ENSG00000246985	SOCS2-AS1	43.87842774	75.680132	12.07672346	-2.65204936	0.397024774	-6.6798082	2.39E-11	1.73E-10
ENSG00000120049	KCNIP2	5.476205813	9.4655959	1.48681568	-2.654116902	1.119777257	-2.3702186	0.017778	0.043152
ENSG00000180730	SHISA2	13.67493134	23.598569	3.751294088	-2.655030981	0.713572153	-3.7207604	0.000199	0.000721
ENSG00000012171	SEMA3B	5.46953764	9.4549311	1.484144154	-2.667822872	1.084634286	-2.459652	0.013907	0.034759
ENSG00000169418	NPR1	518.0859959	895.81921	140.3527786	-2.669841727	0.137453147	-19.423649	4.87E-84	1.90E-82
ENSG00000171345	KRT19	313.7327644	542.87716	84.58836892	-2.681796387	0.34327716	-7.8123356	5.61E-15	5.00E-14
ENSG00000277152	AC110048.2	12.55303112	21.711897	3.394164824	-2.686016218	0.722933641	-3.7154395	0.000203	0.000735
ENSG00000278769	AC090510.3	14.05504589	24.298065	3.812026762	-2.687180856	0.772902807	-3.4767384	0.000508	0.001721
ENSG00000103067	ESRP2	11.21034296	19.431263	2.989423041	-2.700285411	0.761998605	-3.5436881	0.000395	0.001363
ENSG00000117152	RGS4	297.5581434	516.41469	78.7016014	-2.713939802	0.353757972	-7.6717417	1.70E-14	1.47E-13
ENSG00000154096	THY1	5.75018651	9.9950941	1.505278887	-2.727190634	1.078999531	-2.5275179	0.011487	0.029204
ENSG00000114115	RBP1	20.07025076	34.905063	5.235438242	-2.730082276	0.602696333	-4.5297808	5.90E-06	2.66E-05
ENSG00000128594	LRRC4	31.36311606	54.570118	8.156114031	-2.731239685	0.477289103	-5.7224011	1.05E-08	6.18E-08
ENSG00000236404	VLDLR-AS1	12.83730593	22.322716	3.351895357	-2.732233842	0.720819632	-3.7904543	0.00015	0.000557
ENSG00000272669	AL021707.6	7.111528792	12.402919	1.820138685	-2.733742225	1.04407052	-2.6183502	0.008836	0.023086
ENSG00000120645	IQSEC3	5.790723439	10.076168	1.505278887	-2.737136768	1.085153829	-2.5223491	0.011657	0.029608
ENSG00000167642	SPINT2	100.5342735	174.9018	26.16674258	-2.738323518	0.261274984	-10.480619	1.06E-25	1.48E-24
ENSG00000135842	FAM129A	77.87340587	135.60142	20.14538956	-2.746579096	0.320447519	-8.5710731	1.03E-17	1.04E-16
ENSG00000173991	TCAP	5.770774885	10.057406	1.484144154	-2.74878047	1.065848157	-2.5789607	0.00991	0.025555
ENSG00000178726	THBD	1724.103008	3002.6046	445.6014236	-2.753808532	0.0787689	-34.960607	8.93E-268	1.48E-265
ENSG00000102981	PARD6A	24.85916858	43.260659	6.457678169	-2.75935192	0.541996496	-5.0910881	3.56E-07	1.83E-06
ENSG00000147642	SYBU	118.0659783	205.7431	30.38885418	-2.76103467	0.246786574	-11.187945	4.67E-29	7.18E-28

ENSG00000106852	LHX6	2876.647535	5015.0286	738.2664906	-2.764780349	0.061579947	-44.897413	0	0
ENSG00000136160	EDNRB	1201.526455	2095.2945	307.758439	-2.766560365	0.086104443	-32.130286	1.67E-226	2.24E-224
ENSG00000224420	ADM5	37.98843264	66.288995	9.687870704	-2.767033729	0.426665046	-6.4852599	8.86E-11	6.17E-10
ENSG00000174059	CD34	14861.33015	25922.391	3800.269013	-2.769991755	0.114221544	-24.251045	6.45E-130	3.99E-128
ENSG00000179046	TRIML2	13.25302162	23.151476	3.354566884	-2.774568204	0.749670333	-3.7010511	0.000215	0.000775
ENSG00000259287	AC010809.1	5.847975613	10.209136	1.48681568	-2.779166048	1.110946082	-2.501621	0.012363	0.031212
ENSG00000188747	NOXA1	80.94630738	141.44309	20.44952788	-2.796257132	0.340908168	-8.2023765	2.36E-16	2.25E-15
ENSG00000174175	SELP	564.3566276	987.17642	141.536834	-2.802658837	0.128569647	-21.79876	2.38E-105	1.17E-103
ENSG00000258559	AC005519.1	17.88198608	31.335346	4.428626202	-2.803424604	0.705720429	-3.9724294	7.11E-05	0.000277
ENSG00000164604	GPR85	13.56924173	23.786588	3.351895357	-2.820939558	0.706094802	-3.9951286	6.47E-05	0.000254
ENSG00000057294	PKP2	54.80438971	96.288646	13.3201335	-2.842980456	0.388466072	-7.3184781	2.51E-13	2.05E-12
ENSG00000095059	DHPS	12.53584007	22.063794	3.007886248	-2.876611166	0.783380089	-3.6720504	0.000241	0.000862
ENSG00000110025	SNX15	6.234335494	10.981855	1.48681568	-2.880167205	1.075383192	-2.6782706	0.0074	0.019701
ENSG00000203434	AL353740.1	12.51502689	22.043302	2.986751515	-2.882987719	0.761930666	-3.7837927	0.000154	0.000571
ENSG00000224367	OACYLP	9.408766694	16.566175	2.251358253	-2.883001252	0.871581645	-3.3077811	0.00094	0.003034
ENSG00000163554	SPTA1	207.3061808	365.17284	49.43952463	-2.884017722	0.187707748	-15.364404	2.84E-53	7.34E-52
ENSG00000132470	ITGB4	14.05639225	24.829636	3.283148106	-2.891212777	0.742964765	-3.8914534	9.96E-05	0.000379
ENSG00000267216	AC020915.1	4.754630999	8.3875902	1.121671837	-2.89515028	1.225057227	-2.3632776	0.018114	0.043893
ENSG00000253438	PCAT1	14.41456724	25.437641	3.391493298	-2.915582546	0.703593789	-4.1438435	3.42E-05	0.000139
ENSG00000231177	LINC00852	4.761665892	8.4227947	1.100537104	-2.917707639	1.223439071	-2.384841	0.017087	0.041672
ENSG00000226149	AL356124.1	6.318215989	11.197229	1.439203161	-2.923028091	1.114468179	-2.6228009	0.008721	0.022832
ENSG00000236871	LINC00106	14.6212522	25.890609	3.351895357	-2.941041658	0.76209702	-3.8591434	0.000114	0.00043
ENSG00000238121	LINC00426	8.112685144	14.40256	1.822810211	-2.960223998	0.961745897	-3.0779689	0.002084	0.006263
ENSG00000148541	FAM13C	27.78648658	49.287282	6.285691302	-2.96141483	0.542383828	-5.4599984	4.76E-08	2.63E-07
ENSG00000140873	ADAMTS18	3809.141912	6751.77	866.5138714	-2.961643425	0.051141474	-57.910795	0	0
ENSG00000274213	AC015912.3	9.850119264	17.520299	2.179939475	-2.973334802	0.901711972	-3.297433	0.000976	0.003139
ENSG00000154330	PGM5	11.51611839	20.444884	2.587352784	-2.976145246	0.806872057	-3.6884971	0.000226	0.000812
ENSG00000163072	NOSTRIN	815.4670203	1447.8537	183.0803564	-2.986171012	0.112420235	-26.562576	1.84E-155	1.44E-153
ENSG00000185482	STAC3	4.995382762	8.8902284	1.100537104	-2.990695542	1.208268751	-2.4751907	0.013317	0.033426
ENSG00000171864	PRND	13.24447048	23.541787	2.947153575	-2.99124996	0.778901927	-3.8403422	0.000123	0.000462
ENSG00000204149	AGAP6	5.07468275	9.0250221	1.124343364	-2.998739854	1.183145286	-2.5345491	0.011259	0.028693
ENSG00000267474	AC008569.2	5.083150856	9.0419583	1.124343364	-3.014601382	1.244569865	-2.4222034	0.015427	0.038154
ENSG00000137033	IL33	53.05372048	94.536027	11.57141359	-3.026294955	0.375291001	-8.0638623	7.39E-16	6.87E-15
ENSG00000167968	DNASE1L2	10.31876908	18.391523	2.246015201	-3.02965766	0.871459278	-3.4765338	0.000508	0.001722
ENSG00000186301	MST1P2	13.90439221	24.822033	2.986751515	-3.044645768	0.749949186	-4.0598027	4.91E-05	0.000196
ENSG00000128917	DLL4	2040.224362	3646.3645	434.0842591	-3.070342433	0.071427723	-42.985304	0	0
ENSG00000004799	PDK4	577.2849996	1032.4584	122.1115563	-3.076454768	0.122398992	-25.134641	2.08E-139	1.43E-137
ENSG00000137878	GCOM1	226.5490405	405.39145	47.70663178	-3.083336861	0.186999462	-16.48848	4.44E-61	1.31E-59
ENSG00000185432	METTL7A	330.5464338	592.40249	68.69038137	-3.109880416	0.159715939	-19.471322	1.92E-84	7.54E-83
ENSG00000197599	CCDC154	5.51239396	9.8793098	1.145478097	-3.115216795	1.212789422	-2.5686378	0.01021	0.026262
ENSG00000179314	WSCD1	1145.970299	2056.4847	235.4558736	-3.128570641	0.094296254	-33.178101	2.23E-241	3.30E-239
ENSG00000229108	LINC02587	112.6135533	202.3542	22.87290837	-3.146162213	0.273179068	-11.51685	1.09E-30	1.77E-29
ENSG00000163053	SLC16A14	16.64141053	29.928254	3.354566884	-3.152075522	0.680693244	-4.6306843	3.64E-06	1.68E-05
ENSG00000185527	AC139530.1	18.61613915	33.523254	3.709024622	-3.161521862	0.655803017	-4.8208407	1.43E-06	6.89E-06
ENSG00000243970	PPIEL	7.478635222	13.451992	1.505278887	-3.163621647	1.048018831	-3.0186687	0.002539	0.007489
ENSG00000160781	PAQR6	11.1519182	20.123897	2.179939475	-3.179824138	0.911179616	-3.4897885	0.000483	0.001647
ENSG00000164112	TMEM155	9.523604206	17.142531	1.904677618	-3.183399197	0.952542489	-3.3420023	0.000832	0.002708
ENSG00000148671	ADIRF	193.4213625	348.63909	38.20363063	-3.189842811	0.229793387	-13.881352	8.22E-44	1.78E-42
ENSG00000179242	CDH4	136.5021892	246.13917	26.86520943	-3.190774529	0.240041747	-13.292582	2.56E-40	5.16E-39
ENSG00000239218	RPS20P22	5.682732854	10.264929	1.100537104	-3.191284892	1.236118849	-2.5816975	0.009832	0.025382
ENSG00000152213	ARL11	36.10107143	65.120088	7.082054712	-3.193737179	0.468666377	-6.8145215	9.46E-12	7.03E-11
ENSG00000185739	SRL	13.37156259	24.092368	2.650756984	-3.193951287	0.761085522	-4.1965734	2.71E-05	0.000112
ENSG00000140022	STON2	7.644337648	13.783396	1.505278887	-3.199166346	0.994910449	-3.215532	0.001302	0.004087
ENSG00000281406	BLACAT1	20.98027287	37.867914	4.092631671	-3.200473245	0.606604853	-5.2760429	1.32E-07	7.04E-07
ENSG00000173947	PIFO	7.760743372	13.992402	1.529085146	-3.204947179	1.063934852	-3.0123528	0.002592	0.007633
ENSG00000068615	REEP1	21.84019528	39.561281	4.119109457	-3.26656552	0.607800925	-5.3744004	7.68E-08	4.18E-07
ENSG00000076554	TPD52	75.98534039	137.63726	14.33342477	-3.270862167	0.332187932	-9.8464208	7.10E-23	8.87E-22
ENSG00000271009	AC116667.1	4.022323814	7.3012398	0.74340784	-3.279120754	1.40771167	-2.329398	0.019838	0.04757
ENSG00000204936	CD177	22.27798851	40.407718	4.148258768	-3.290461087	0.626280665	-5.253972	1.49E-07	7.91E-07
ENSG00000154027	AK5	422.696324	766.97432	78.41832496	-3.292342303	0.139702808	-23.566758	8.45E-123	4.91E-121
ENSG00000126838	PZP	12.19936673	22.152718	2.246015201	-3.301930556	0.817425089	-4.0394289	5.36E-05	0.000213
ENSG00000062524	LTK	4.041329392	7.3630572	0.719601581	-3.313311379	1.426461662	-2.3227483	0.020193	0.04828
ENSG00000079102	RUNX1T1	57.17504608	103.96375	10.38633755	-3.315206697	0.389715621	-8.5067329	1.79E-17	1.79E-16
ENSG00000111907	TPD52L1	6.123235277	11.143262	1.10320863	-3.317452641	1.148950569	-2.8873763	0.003885	0.011005
ENSG00000249631	AC005699.1	6.293016375	11.437883	1.148149623	-3.347325149	1.177833116	-2.841935	0.004484	0.012489
ENSG00000141854	MISP3	8.370248925	15.256354	1.484144154	-3.351059361	0.975576261	-3.4349538	0.000593	0.001989
ENSG00000170166	HOXD4	6.321968593	11.522265	1.121671837	-3.351065962	1.161369034	-2.8854446	0.003909	0.011069
ENSG00000144063	MALL	218.8525002	398.50387	39.20113022	-3.351982937	0.202382775	-16.56259	1.30E-61	3.87E-60
ENSG00000176194	CIDEA	25.14195098	45.791872	4.492030402	-3.352522041	0.589058819	-5.6913197	1.26E-08	7.36E-08
ENSG00000153291	SLC25A27	6.359668971	11.594995	1.124343364	-3.353422562	1.177067577	-2.8489635	0.004386	0.01225
ENSG00000221826	PSG3	12.96261483	23.650065	2.275164513	-3.390259778	0.865248177	-3.9182513	8.92E-05	0.000342
ENSG00000036672	USP2	4.252111592	7.7846216	0.719601581	-3.39771849	1.412026118	-2.4062717	0.016116	0.039595
ENSG00000094755	GABRP	4.332844794	7.901147	0.764542573	-3.398798611	1.378128584	-2.466242	0.013654	0.034195
ENSG00000186377	CYP4X1	149.462843	273.37263	25.55305214	-3.422774729	0.245610138	-13.935804	3.84E-44	8.37E-43
ENSG00000207744	MIR10B	4.458452227	8.1550334	0.761871047	-3.423672757	1.442261414	-2.3738226	0.017605	0.042779
ENSG00000170381	SEMA3E	10.97473779	20.084396	1.865079677	-3.431851258	0.89870778	-3.8186509	0.000134	0.000501
ENSG00000249087	ZNF436-AS1	13.4392732	24.632531	2.246015201	-3.448772425	0.798940452	-4.3166827	1.58E-05	6.73E-05
ENSG00000259498	TPM1-AS	4.488447854	8.2150247	0.761871047	-3.458242065	1.459350134	-2.3697137	0.017802	0.043206
ENSG00000237928	NFIA-AS2	11.25259529	20.661246	1.843944944	-3.473863045	0.885223037	-3.92428	8.70E-05	0.000335
ENSG00000262528	AL022341.2	6.845318743	12.5901	1.100537104	-3.494971546	1.189373068	-2.938499	0.003298	0.009507
ENSG00000236740	AL033384.1	6.937678146	12.751013	1.124343364	-3.501284419	1.104446343	-3.1701716	0.001523	0.004714
ENSG00000101695	RNF125	53.10386446	97.578126	8.629603066	-3.503016873	0.409190818	-8.5608394	1.12E-17	1.14E-16
ENSG00000019102	VSIG2	35.16821852	64.675122	5.661314758	-3.518632404	0.537673569	-6.5441796	5.98E-11	4.22E-10
ENSG00000127241	MASP1	44.40069859	81.700879	7.10051792	-3.524259624	0.44505481	-7.9187092	2.40E-15	2.18E-14
ENSG00000137880	GCHFR	244.0615344	450.27606	37.84701169	-3.563738319	0.215880859	-16.507894	3.22E-61	9.55E-60

ENSG00000164867	NOS3	698.5074304	1289.6831	107.3317974	-3.584853615	0.128105853	-27.983527	2.58E-172	2.29E-170
ENSG00000211772	TRBC2	4.882261642	9.023787	0.740736314	-3.593539227	1.508728928	-2.3818323	0.017227	0.041967
ENSG00000174669	SLC29A2	4.799335557	8.8790695	0.719601581	-3.594593351	1.407668643	-2.5535792	0.010662	0.027316
ENSG00000139737	SLAIN1	7.374502342	13.62199	1.12701489	-3.600097519	1.11629414	-3.2250438	0.00126	0.003966
ENSG00000255176	AP000941.1	7.49325053	13.838351	1.148149623	-3.611625404	1.131712005	-3.1912937	0.001416	0.004413
ENSG00000118777	ABCG2	430.4810651	796.18654	64.77558994	-3.620427335	0.147584171	-24.531271	6.85E-133	4.44E-131
ENSG00000132639	SNAP25	9.956374958	18.407471	1.505278887	-3.62067109	0.953257877	-3.7982074	0.000146	0.000541
ENSG00000211695	TRGV9	9.932007076	18.37987	1.484144154	-3.624524183	0.944949417	-3.8356806	0.000125	0.00047
ENSG00000198237	AC131392.1	4.94792979	9.176258	0.719601581	-3.634324493	1.363948529	-2.6645613	0.007709	0.020426
ENSG00000232334	AL683842.1	4.976222933	9.2117096	0.740736314	-3.634711419	1.350140544	-2.6920986	0.0071	0.018974
ENSG00000106066	CPVL	7.577063469	14.027112	1.12701489	-3.636316535	1.10317876	-3.296217	0.00098	0.003151
ENSG00000248019	FAM13A-AS1	5.05337153	9.3660067	0.740736314	-3.647734427	1.348969413	-2.7040898	0.006849	0.018373
ENSG00000167641	PPP1R14A	61.93996496	114.84024	9.039687901	-3.676967735	0.394359194	-9.3239052	1.12E-20	1.29E-19
ENSG00000261286	ATP2C2-AS1	5.219253435	9.6712928	0.767214099	-3.680598702	1.351199352	-2.7239494	0.006451	0.017391
ENSG00000102878	HSF4	13.22967634	24.570467	1.888885937	-3.703031529	0.851742506	-4.3475951	1.38E-05	5.90E-05
ENSG00000115339	GALNT3	5.274870681	9.8063335	0.74340784	-3.709369607	1.32859539	-2.7919483	0.005239	0.014391
ENSG00000118308	LRMP	5.315010992	9.8681509	0.761871047	-3.715192061	1.330744426	-2.7918149	0.005241	0.014393
ENSG00000177570	SAMD12	63.93817378	118.83666	9.039687901	-3.720974692	0.383299641	-9.7077437	2.79E-22	3.43E-21
ENSG00000270130	AC068790.7	5.291978873	9.8405499	0.74340784	-3.721326332	1.352767155	-2.7508994	0.005943	0.016131
ENSG00000157856	DRC1	62.67404897	116.81105	8.537049555	-3.766437359	0.395978445	-9.5117232	1.88E-21	2.24E-20
ENSG00000271447	MMP28	69.49535034	129.56206	9.428638003	-3.785829016	0.39500908	-9.5841569	9.32E-22	1.12E-20
ENSG00000079841	RIMS1	2.959000893	5.558201	0.35980079	-3.786997639	1.626119507	-2.3288557	0.019867	0.047626
ENSG00000260788	AC009063.2	158.5364917	296.04645	21.02652938	-3.815914137	0.25605695	-14.902599	3.17E-50	7.79E-49
ENSG00000235703	LINC00894	8.5393525	15.978168	1.100537104	-3.826633067	1.134294292	-3.3735805	0.000742	0.002442
ENSG00000186326	RGS9BP	3.069456624	5.7553062	0.38360705	-3.841151422	1.629916263	-2.3566557	0.01844	0.044583
ENSG00000261408	TEN1-CDK3	3.083257139	5.7829072	0.38360705	-3.845779306	1.64979892	-2.3310594	0.01975	0.047409
ENSG00000095596	CYP26A1	3.108904823	5.8368741	0.380935524	-3.857396589	1.628254121	-2.3690384	0.017834	0.043273
ENSG00000279744	AC132938.5	8.722930283	16.324189	1.121671837	-3.865373996	1.080361062	-3.5778539	0.000346	0.001207
ENSG00000092421	SEMA6A	133.5787667	250.12547	17.03206712	-3.865738567	0.284304802	-13.597162	4.16E-42	8.72E-41
ENSG00000250722	SELENOP	43.99798386	82.337324	5.658643232	-3.867597227	0.490222847	-7.8894675	3.03E-15	2.73E-14
ENSG00000267838	AC245884.8	3.13588827	5.890841	0.380935524	-3.868797206	1.614866031	-2.3957388	0.016587	0.040549
ENSG00000266714	MYO15B	73.76516161	138.18379	9.346533121	-3.880893215	0.431159722	-9.001057	2.24E-19	2.43E-18
ENSG00000175287	PHYHD1	3.177637445	5.9716678	0.38360705	-3.885859641	1.602773147	-2.4244602	0.015331	0.037953
ENSG00000260400	AL513534.2	5.968982566	11.197229	0.740736314	-3.904221511	1.295534106	-3.0135999	0.002582	0.007605
ENSG00000274290	HIST1H2BE	3.210320022	6.0608393	0.35980079	-3.91484551	1.597172425	-2.4511101	0.014242	0.035507
ENSG00000267281	ATF7	6.027368788	11.314001	0.740736314	-3.921995556	1.28642423	-3.0487575	0.002298	0.00685
ENSG00000163687	DNASE1L3	30.11052638	56.578104	3.642948896	-3.929635748	0.603233499	-6.5142863	7.30E-11	5.12E-10
ENSG00000130300	PLVAP	183.0535965	343.47773	22.62946735	-3.930515089	0.243590501	-16.135749	1.43E-58	4.07E-57
ENSG00000215481	BCRP3	3.288531282	6.196127	0.380935524	-3.941410163	1.592693679	-2.4746819	0.013335	0.033455
ENSG00000172348	RCAN2	58.42666962	109.8189	7.034442194	-3.952526934	0.427327916	-9.2494003	2.26E-20	2.57E-19
ENSG00000260589	STAM-AS1	3.318500844	6.2772009	0.35980079	-3.957357926	1.593728083	-2.4830823	0.013025	0.032757
ENSG00000074211	PPP2R2C	12.67605647	23.823028	1.529085146	-3.984752305	0.933406324	-4.2690436	1.96E-05	8.27E-05
ENSG00000203883	SOX18	2841.305206	5347.1221	335.4882842	-3.994084516	0.144748296	-27.593309	1.34E-167	1.16E-165
ENSG00000167612	ANKRD33	3.426434632	6.4930685	0.35980079	-4.000266649	1.609519705	-2.4853791	0.012941	0.032583
ENSG00000280069	AC127024.8	3.491215926	6.6014963	0.380935524	-4.021066652	1.623812505	-2.4763122	0.013275	0.03333
ENSG00000273001	AL731533.2	6.57827481	12.392007	0.764542573	-4.040219147	1.263507964	-3.1976206	0.001386	0.004326
ENSG00000169129	AFAP1L2	483.5307769	913.19875	53.86280779	-4.0822523	0.157793886	-25.870789	1.42E-147	1.05E-145
ENSG00000273723	AL139089.1	3.610356902	6.860913	0.35980079	-4.086083949	1.567799986	-2.6062533	0.009154	0.023818
ENSG00000244476	ERVFRD-1	98.99887624	187.07696	10.92079674	-4.104248921	0.342534888	-11.981988	4.42E-33	7.62E-32
ENSG00000263586	HID1-AS1	13.95914149	26.389198	1.529085146	-4.136855791	0.893990518	-4.6274045	3.70E-06	1.71E-05
ENSG00000205221	VIT	3.790922506	7.2009095	0.380935524	-4.164136246	1.556044299	-2.6761039	0.007448	0.019805
ENSG00000128886	ELL3	3.816966807	7.2741328	0.35980079	-4.167447474	1.549797856	-2.6890265	0.007166	0.019139
ENSG00000214776	AC092821.1	3.806058784	7.2285105	0.38360705	-4.16792364	1.557099756	-2.6767223	0.007435	0.019773
ENSG00000170807	LMOD2	3.86130548	7.3628102	0.35980079	-4.191840644	1.565053046	-2.6784016	0.007397	0.019696
ENSG00000124440	HIF3A	185.2170678	351.44943	19.09270245	-4.200599642	0.260541966	-16.122545	1.77E-58	5.03E-57
ENSG00000259070	LINC00639	18.25304674	34.617208	1.888885937	-4.203404162	0.788924124	-5.3280208	9.93E-08	5.35E-07
ENSG00000149212	SESN3	2574.761258	4887.8389	261.6836217	-4.224160912	0.078481076	-53.823943	0	0
ENSG00000122679	RAMP3	7.466086769	14.188766	0.74340784	-4.238989755	1.249988085	-3.3912241	0.000696	0.002305
ENSG00000158825	CDA	4.054485421	7.7491701	0.35980079	-4.263281095	1.552131639	-2.7467265	0.006019	0.016311
ENSG00000196421	C20orf204	103.5224786	196.83866	10.20630073	-4.277709398	0.350263067	-12.212847	2.65E-34	4.73E-33
ENSG00000133800	LYVE1	9485.21693	18040.847	929.5869759	-4.277747731	0.047011351	-90.993934	0	0
ENSG00000279900	AP001767.4	2.002331959	4.0046639	0	-4.279930993	1.839711962	-2.3264136	0.019996	0.047893
ENSG00000122477	LRRC39	2.015885441	4.0317709	0	-4.288012042	1.851296097	-2.3162216	0.020546	0.04901
ENSG00000161798	AQP5	4.127487787	7.8740401	0.380935524	-4.297785898	1.527880986	-2.8129062	0.00491	0.013587
ENSG00000240038	AMY2B	11.61665861	22.08784	1.145478097	-4.297792802	1.025845694	-4.189512	2.80E-05	0.000115
ENSG00000214357	NEURL1B	290.8154127	553.62548	28.00534447	-4.300859487	0.20519489	-20.959876	1.52E-97	6.95E-96
ENSG00000250131	AC078881.1	2.05642237	4.1128447	0	-4.312835783	1.842913806	-2.3402265	0.019272	0.046353
ENSG00000271926	AC008972.1	4.204636384	8.0283372	0.380935524	-4.313484292	1.526534382	-2.8256712	0.004718	0.013099
ENSG00000253704	AC023632.2	2.069852335	4.1397047	0	-4.321282517	1.815752334	-2.3798854	0.017318	0.042172
ENSG00000167037	SGSM1	4.340642348	8.2976776	0.38360705	-4.35660486	1.5576865	-2.7968432	0.00516	0.014206
ENSG00000281641	SAMD12-AS1	2.137372711	4.2747454	0	-4.361631209	1.814913401	-2.4032173	0.016252	0.039863
ENSG00000229981	LINC01435	8.12388998	15.480566	0.767214099	-4.363366665	1.230842792	-3.5450235	0.000393	0.001356
ENSG00000277494	GPIHBP1	270.4825209	516.03876	24.92627692	-4.364371723	0.240216411	-18.168499	9.17E-74	3.21E-72
ENSG00000275180	AC048341.2	8.047426572	15.354117	0.740736314	-4.365795927	1.245975361	-3.5039183	0.000458	0.00157
ENSG00000233718	MYCNOS	2.141421489	4.282843	0	-4.377565644	1.80275994	-2.4282577	0.015172	0.037599
ENSG00000101134	DOK5	2.181834901	4.3636698	0	-4.401271805	1.818296485	-2.4205468	0.015497	0.038281
ENSG00000105369	CD79A	4.462496339	8.5651919	0.35980079	-4.415864392	1.552529049	-2.8443039	0.004451	0.012412
ENSG00000248858	AC104211.1	2.212990642	4.4259813	0	-4.43061189	1.840141892	-2.4077556	0.016051	0.039456
ENSG00000136960	ENPP2	311.3614893	595.20986	27.51311938	-4.442102196	0.208472527	-21.307854	9.60E-101	4.51E-99
ENSG00000272139	AC113349.2	2.263032275	4.5260646	0	-4.446176209	1.777203881	-2.5017817	0.012357	0.031202
ENSG00000233351	AL356124.2	2.316875653	4.6337513	0	-4.477881355	1.849226092	-2.4214894	0.015457	0.038212
ENSG00000135914	HTR2B	183.0847389	350.5239	15.64558205	-4.48072902	0.268253663	-16.703328	1.24E-62	3.75E-61
ENSG00000243137	PSG4	44.35288013	84.925317	3.7804434	-4.500595373	0.558431357	-8.0593529	7.67E-16	7.13E-15
ENSG00000146233	CYP39A1	2.375261875	4.7505237	0	-4.518818845	1.761433562	-2.5654211	0.010305	0.02648
ENSG00000223749	MIR503HG	77.17536381	148.09929	6.251436413	-4.543715162	0.447440808	-10.154897	3.15E-24	4.12E-23

ENSG00000100604	CHGA	5.025867113	9.6707987	0.380935524	-4.585612109	1.461828222	-3.1369022	0.001707	0.00523
ENSG00000264954	PRR29-AS1	5.057269888	9.7336043	0.380935524	-4.59889282	1.492848308	-3.0806163	0.002066	0.006211
ENSG00000187513	GJA4	33.95286458	65.299913	2.605815991	-4.638581018	0.640136954	-7.2462322	4.29E-13	3.47E-12
ENSG00000267690	LDLRAD4-AS1	14.70566653	28.260512	1.150821149	-4.640088267	1.016644681	-4.5641199	5.02E-06	2.28E-05
ENSG00000177359	AC024940.2	2.608978744	5.2179575	0	-4.648181788	1.730346809	-2.6862718	0.007225	0.019289
ENSG00000260228	AC009119.1	2.653440934	5.3068819	0	-4.680523159	1.696511548	-2.7589103	0.005799	0.015778
ENSG00000104490	NCALD	5.430076287	10.479217	0.380935524	-4.702348225	1.453125749	-3.2360229	0.001212	0.00383
ENSG00000272720	AL022322.1	2.711703639	5.4234073	0	-4.717589422	1.705005053	-2.7669064	0.005659	0.015442
ENSG00000204410	MSH5	15.53881948	29.929489	1.148149623	-4.729821589	0.975087844	-4.850662	1.23E-06	5.98E-06
ENSG00000033122	LRRC7	2.81951391	5.6390278	0	-4.767280565	1.68965716	-2.8214484	0.004781	0.013256
ENSG00000168356	SCN11A	5.885288477	11.410776	0.35980079	-4.833892019	1.47524289	-3.2766754	0.00105	0.00336
ENSG00000188277	C15orf62	34.41023116	66.566433	2.254029779	-4.88585821	0.681876879	-7.1653085	7.76E-13	6.18E-12
ENSG00000260701	AC025284.1	3.097940003	6.19588	0	-4.903794801	1.631676696	-3.0053716	0.002653	0.007796
ENSG00000278467	AC138393.3	3.202195564	6.4043911	0	-4.938692399	1.725668957	-2.8619002	0.004211	0.011823
ENSG00000152207	CYSLTR2	3.187111416	6.3742228	0	-4.956476627	1.664015212	-2.9786246	0.002895	0.00844
ENSG00000231880	KF459542.1	3.331903906	6.6638078	0	-5.003548699	1.644430384	-3.0427245	0.002344	0.006975
ENSG00000235726	AC010148.1	3.322522719	6.6450454	0	-5.007711496	1.68997055	-2.9631945	0.003045	0.008833
ENSG00000267682	AC016590.2	3.497976882	6.9959538	0	-5.072886713	1.603108837	-3.1644057	0.001554	0.004797
ENSG00000131386	GALNT15	911.3624692	1770.8914	51.83351834	-5.0897063	0.148325802	-34.314369	4.79E-258	7.71E-256
ENSG00000233370	AC140479.3	3.604627039	7.2092541	0	-5.134072163	1.606928668	-3.1949596	0.001399	0.004363
ENSG00000169432	SCN9A	80.87106636	157.33998	4.402148417	-5.137311499	0.490775675	-10.467739	1.22E-25	1.68E-24
ENSG00000171509	RXFP1	3.722312564	7.4446251	0	-5.166422744	1.57823783	-3.2735388	0.001062	0.003392
ENSG00000255690	TRIL	176.3855354	343.47215	9.298920602	-5.199157569	0.332570428	-15.633253	4.32E-55	1.15E-53
ENSG00000227885	AL590652.1	4.090946669	8.1818933	0	-5.294917771	1.608022819	-3.2928126	0.000992	0.003187
ENSG00000253177	AC104211.2	22.63828388	44.152224	1.124343364	-5.295046031	0.928205113	-5.7046077	1.17E-08	6.83E-08
ENSG00000116031	CD207	4.207225046	8.4144501	0	-5.341559318	1.530016436	-3.4911777	0.000481	0.00164
ENSG00000171873	ADRA1D	31.68809742	61.849781	1.52641362	-5.366159526	0.803498748	-6.6784915	2.41E-11	1.75E-10
ENSG00000276409	CCL14	16.52547173	32.310207	0.740736314	-5.429549317	1.137029779	-4.7752042	1.80E-06	8.56E-06
ENSG00000274403	AC090510.2	4.574699035	9.1493981	0	-5.472339062	1.502456748	-3.6422606	0.00027	0.00096
ENSG00000130600	H19	4.70998682	9.4199736	0	-5.508156464	1.504366723	-3.6614453	0.000251	0.000897
ENSG00000170011	MYRIP	473.9807796	928.5144	19.44716019	-5.577073859	0.226200679	-24.655425	3.22E-134	2.13E-132
ENSG00000236107	SCN1A-AS1	10.12766974	19.874404	0.380935524	-5.616843169	1.349504507	-4.1621522	3.15E-05	0.000129
ENSG00000152583	SPARCL1	116.2831496	228.09273	4.473567195	-5.667610767	0.460541774	-12.306399	8.37E-35	1.50E-33
ENSG00000166828	SCNN1G	5.571754478	11.143509	0	-5.751730029	1.44493725	-3.9806089	6.87E-05	0.000269
ENSG00000167434	CA4	11.26968949	22.179578	0.35980079	-5.783637695	1.317847411	-4.3887006	1.14E-05	4.95E-05
ENSG00000049540	ELN	12.07902092	23.798241	0.35980079	-5.87926066	1.320102486	-4.4536396	8.44E-06	3.73E-05
ENSG00000213561	AC103591.2	12.43815032	24.5165	0.35980079	-5.921382291	1.313726736	-4.5073166	6.57E-06	2.94E-05
ENSG00000100053	CRYBB3	6.352571795	12.705144	0	-5.944191579	1.421189256	-4.1825475	2.88E-05	0.000119
ENSG00000275152	CCL16	7.147066111	14.294132	0	-6.116068877	1.397008848	-4.3779743	1.20E-05	5.18E-05
ENSG00000168447	SCNN1B	28.07166302	55.378783	0.764542573	-6.204664716	1.091679335	-5.6835964	1.32E-08	7.69E-08
ENSG00000162630	B3GALT2	8.11308933	16.226179	0	-6.292624673	1.366789917	-4.6039443	4.15E-06	1.90E-05
ENSG00000166106	ADAMTS15	8.215937742	16.431875	0	-6.315282629	1.366666783	-4.6209381	3.82E-06	1.76E-05
ENSG00000237371	AL355803.1	9.694103922	19.388208	0	-6.547165538	1.34666255	-4.8617714	1.16E-06	5.68E-06
ENSG00000134817	APLNR	54.14274037	107.14	1.145478097	-6.575969595	0.87543314	-7.5116754	5.84E-14	4.95E-13
ENSG00000115155	OTOF	174.5245073	345.60189	3.447120395	-6.677229522	0.51072246	-13.074086	4.63E-39	9.06E-38
ENSG00000162894	FCMR	26.493432	52.605928	0.380935524	-7.025286534	1.246102829	-5.6378064	1.72E-08	9.95E-08
ENSG00000268894	PLCE1-AS1	19.61088934	39.221779	0	-7.56891492	1.259552646	-6.0092089	1.86E-09	1.17E-08
ENSG00000163083	INHBB	588.7760562	1176.0891	1.463009421	-9.616978032	0.728887601	-13.194048	9.49E-40	1.88E-38

Table S4: RNA-Seq analysis of the differentially expressed genes in cytokine-treated HUVECs with or without acetate treatment (adjusted p value < 0.05 and Log2|Fold Change| > 1).

EnsemblID	GeneSymbol	baseMean	baseMean_TGF-β1 + IL-1β	baseMean_TGF-β1 + IL-1β + acetate	log2FoldChange	lfcSE	stat	pvalue	padj
ENSG00000198829	SUCNR1	110.8544851	0	221.7089703	10.29468923	1.194125008	8.62111517	6.63E-18	5.52E-17
ENSG00000060718	COL11A1	48.43406537	0.295380681	96.57275006	8.134636426	1.215653784	6.69157332	2.21E-11	1.25E-10
ENSG00000105048	TNNT1	325.5851016	3.631211455	647.5389917	7.52515893	0.450158392	16.7166914	9.91E-63	3.20E-61
ENSG00000103485	QPRT	105.3328367	1.284808994	209.3808644	7.35630067	0.751472723	9.78917857	1.25E-22	1.29E-21
ENSG00000149451	ADAM33	48.02221679	0.637217645	95.40721594	7.224256808	1.072876878	6.73353761	1.66E-11	9.43E-11
ENSG00000226320	LINC01811	10.82064506	0	21.64129013	6.938717093	1.33177941	5.2101099	1.89E-07	7.87E-07
ENSG00000104783	KCNN4	38.90121489	0.637217645	77.16521214	6.916449168	1.074876138	6.43464761	1.24E-10	6.64E-10
ENSG00000157542	KCNJ6	38.02956336	0.637217645	75.42190908	6.885486437	1.073381562	6.41476123	1.41E-10	7.55E-10
ENSG00000139835	GRTP1	10.18712756	0	20.37425512	6.85048696	1.339653138	5.11362738	3.16E-07	1.29E-06
ENSG00000142235	AC008403.1	114.331043	2.245822313	226.4162637	6.661898545	0.579323081	11.499453	1.33E-30	1.84E-29
ENSG00000227906	SNAP25-AS1	16.87393961	0.323795675	33.42408355	6.60333605	1.285902627	5.1351758	2.82E-07	1.16E-06
ENSG00000248187	AC078850.1	908.0271275	19.79934622	1796.254909	6.516696743	0.302298821	21.5571359	4.54E-103	3.02E-101
ENSG00000167641	PPP1R14A	43.93684104	0.979054608	86.89462748	6.502287212	0.890370067	7.3029041	2.82E-13	1.81E-12
ENSG00000162670	BRINP3	14.26142292	0.341836963	28.18100887	6.35776241	1.291549162	4.92258645	8.54E-07	3.34E-06
ENSG00000101210	EEF1A2	2078.679684	52.68479182	4104.674577	6.284057267	0.303735916	20.6892137	4.33E-95	2.50E-93
ENSG00000130643	CALY	6.475784217	0	12.95156843	6.199772173	1.429634211	4.33661431	1.45E-05	4.93E-05
ENSG00000229563	LINC01204	94.34902019	2.662530551	186.0355098	6.182574725	0.554137584	11.1571113	6.61E-29	8.67E-28
ENSG00000003137	CYP26B1	5.939479192	0	11.87895838	6.069874954	1.471980373	4.12361134	3.73E-05	0.000122
ENSG00000274516	FAM74A6	5.646745069	0	11.29349014	6.001245468	1.459311203	4.11238224	3.92E-05	0.000127
ENSG00000146250	PRSS35	50.00070801	1.5053184	98.49609762	5.955200037	0.708684679	8.40317311	4.35E-17	3.48E-16
ENSG00000134873	CLDN10	152.3365674	5.073430582	299.5997042	5.899883164	1.109060105	5.31971454	1.04E-07	4.43E-07
ENSG00000167414	GNG8	5.215300051	0	10.4306001	5.883378073	1.528797379	3.84837007	0.000119	0.000364
ENSG00000184905	TCEAL2	9.936053799	0.323795675	19.54831192	5.827151486	1.380861903	4.21993791	2.44E-05	8.13E-05
ENSG00000250033	SLC7A11-AS1	273.5560387	9.926261283	537.1858161	5.762251337	0.291886982	19.7413783	9.51E-87	4.74E-85
ENSG00000186310	NAP1L3	9.288950918	0.295380681	18.28252115	5.736568658	1.373840497	4.17557109	2.97E-05	9.80E-05
ENSG00000129990	SYT5	4.589561668	0	9.179123336	5.70339613	1.551575908	3.67587309	0.000237	0.000699
ENSG00000146938	NLGN4X	16.34109842	0.683673926	31.99852291	5.637211971	1.136113833	4.96183728	6.98E-07	2.75E-06
ENSG00000008118	CAMK1G	4.299791906	0	8.599583812	5.606947489	1.537203731	3.64749797	0.000265	0.000777
ENSG00000006283	CACNA1G	8.448071598	0.295380681	16.60076251	5.592883113	1.375622369	4.06571108	4.79E-05	0.000154
ENSG00000172346	CSDC2	8.295299022	0.341836963	16.24876108	5.56203315	1.37709782	4.03895284	5.37E-05	0.000171
ENSG00000184697	CLDN6	22.82266538	0.914557038	44.73077372	5.554673995	0.941365178	5.90065803	3.62E-09	1.74E-08
ENSG00000143320	CRABP2	29.8317483	1.349306564	58.31419003	5.504109865	0.819036647	6.72022417	1.81E-11	1.03E-10
ENSG00000105376	ICAM5	285.627856	12.51121444	558.7444976	5.487830841	0.263626796	20.8166656	3.06E-96	1.82E-94
ENSG00000104833	TUBB4A	3.941894976	0	7.883789953	5.482365637	1.567938308	3.49654423	0.000471	0.001335
ENSG00000149294	NCAM1	7.781980669	0.295380681	15.26858066	5.476404556	1.394857406	3.92613935	8.63E-05	0.00027
ENSG00000150275	PCDH15	3.903376874	0	7.806753748	5.470276918	1.584257154	3.45289709	0.000555	0.001556
ENSG00000010310	GIPR	28.52702533	1.227979008	55.82607166	5.460343795	0.857523068	6.36757657	1.92E-10	1.02E-09
ENSG00000132464	ENAM	121.319986	5.478366818	237.1609692	5.447857019	0.397812888	13.6945212	1.09E-42	2.21E-41
ENSG00000228933	AC107419.1	7.531908109	0.295380681	14.76843554	5.425713791	1.383893892	3.92061402	8.83E-05	0.000275
ENSG00000166165	CKB	632.2737084	30.46881754	1234.078599	5.359127312	0.172502844	31.0668926	6.75E-212	1.34E-209
ENSG00000248781	PSMC1P4	3.587535344	0	7.175070689	5.347124448	1.618659867	3.30342684	0.000955	0.002597
ENSG00000147231	RADX	3.336856621	0	6.673713242	5.246893656	1.704494433	3.07826975	0.002082	0.005354
ENSG00000168824	NSG1	186.3799312	10.50394282	362.2559197	5.104940853	0.293664418	17.3835866	1.10E-67	3.93E-66
ENSG00000227502	LINC01268	27.86570212	1.59056338	54.14084086	5.086308239	0.804660236	6.32106324	2.60E-10	1.36E-09
ENSG00000178826	TMEM139	2.959389582	0	5.918779164	5.065490537	1.704924853	2.97109314	0.002967	0.007439
ENSG00000230309	AL121718.1	5.872650713	0.295380681	11.44992074	5.056152538	1.443804963	3.50196368	0.000462	0.001312
ENSG00000131409	LRRC4B	326.3902177	19.23064009	633.5497953	5.04702522	0.222169124	22.7170416	3.04E-114	2.36E-112
ENSG00000170748	RBMXL2	5.753509928	0.295380681	11.21163917	5.024163333	1.493603129	3.36378736	0.000769	0.002114
ENSG00000132470	ITGB4	137.4166032	8.322618078	266.5105883	5.004814091	0.336151084	14.8885853	3.91E-50	9.43E-49
ENSG00000187902	SHISA7	5.41469768	0.341836963	10.4875584	4.934914251	1.503764651	3.28170651	0.001032	0.002793
ENSG00000069188	SDK2	14.7924929	0.942972031	28.64201377	4.904971381	0.983903797	4.9852144	6.19E-07	2.45E-06
ENSG00000186862	PDZD7	48.653427	3.085508076	94.22134592	4.894047397	0.557311098	8.78153587	1.61E-18	1.38E-17
ENSG00000182580	EPHB3	2.610925445	0	5.22185089	4.88464325	1.740843093	2.80590667	0.005018	0.012077
ENSG00000127951	FGL2	2.608567247	0	5.217134494	4.883547055	1.74105458	2.80493622	0.005033	0.012112
ENSG00000268262	AC011445.1	2.594941813	0	5.189883627	4.877115978	1.737353122	2.80721053	0.004997	0.012034
ENSG00000074706	IPCEF1	2.587211875	0	5.174423751	4.873466279	1.763199705	2.76398996	0.00571	0.013613
ENSG00000181392	SYNE4	2.587211875	0	5.174423751	4.873466279	1.763199705	2.76398996	0.00571	0.013613
ENSG00000130294	KIF1A	42.62153528	2.836583676	82.40648688	4.847019266	0.592307421	8.18328303	2.76E-16	2.12E-15
ENSG00000105642	KCNN1	59.78314274	4.0568951	115.5093904	4.804550186	0.482549097	9.95660382	2.36E-23	2.49E-22
ENSG00000284930	AC005280.2	2.431387431	0	4.862774862	4.782365535	1.775421426	2.69365091	0.007067	0.016561
ENSG00000141750	STAC2	13.56555908	0.979054608	26.15206356	4.766338244	0.997110134	4.78015224	1.75E-06	6.62E-06
ENSG00000235213	OR6E1P	4.721389111	0.341836963	9.100941258	4.723195247	1.531836343	3.083355	0.002047	0.005269
ENSG00000254585	MAGEL2	60.9974876	4.398732063	117.5962431	4.721787757	0.464752616	10.1597874	3.00E-24	3.28E-23
ENSG00000228262	LINC01320	4.608678431	0.295380681	8.92197618	4.698849197	1.497938985	3.13687623	0.001708	0.004451
ENSG00000250722	SELENOP	12.68705011	0.886142044	24.48795818	4.696308232	1.023468432	4.5886205	4.46E-06	1.60E-05
ENSG00000106128	GHRHR	4.582606663	0.295380681	8.869832645	4.691783059	1.504959665	3.11754738	0.001824	0.004729
ENSG00000237596	AL138828.1	4.584367825	0.323795675	8.844939976	4.688362554	1.510264408	3.10433228	0.001907	0.004934
ENSG00000154269	ENPP3	2.26547485	0	4.5309497	4.679886525	1.85781087	2.5190328	0.011768	0.026427
ENSG00000144834	TAGLN3	2.251849417	0	4.503698833	4.672427896	1.877101179	2.48917211	0.012804	0.028576
ENSG00000225213	AC073367.1	2.246477677	0	4.492955353	4.669322994	1.827234028	2.55540501	0.010606	0.024046
ENSG00000241280	AC106712.1	2.232852243	0	4.465704487	4.661851139	1.823615431	2.55637842	0.010577	0.02399
ENSG00000161798	AQP5	2.228135847	0	4.456271694	4.659288573	1.824242227	2.55409534	0.010646	0.024119
ENSG00000248538	AC022784.1	4.44806431	0.323795675	8.572332945	4.641345094	1.51195127	3.06977162	0.002142	0.005499
ENSG00000259869	AL022344.1	12.18221367	0.914557038	23.44987031	4.629395693	1.031580015	4.48767485	7.20E-06	2.53E-05
ENSG00000092758	COL9A3	106.2800567	8.353739192	204.2063742	4.619079891	0.342914903	13.4700471	2.35E-41	4.59E-40
ENSG00000140873	ADAMTS18	1366.669156	110.2303074	2623.108004	4.577619565	0.197229699	23.2095856	3.64E-119	3.03E-117
ENSG00000104826	LHB	2.09301143	0	4.186022861	4.56523938	1.856684613	2.45881252	0.01394	0.030839
ENSG00000168779	SHOX2	7.90654863	0.647591349	15.16550591	4.565126369	1.254675835	3.63849071	0.000274	0.000803

ENSG00000243762	AC006547.2	2.066939663	0	4.133879325	4.549851204	1.841925886	2.47015976	0.013505	0.029962
ENSG00000159167	STC1	470.3069704	38.60466297	902.0092779	4.545473106	0.167126962	27.1977248	6.91E-163	9.20E-161
ENSG00000262160	AC020978.5	2.056851526	0	4.113703053	4.543805603	1.897457229	2.39468144	0.016635	0.036147
ENSG00000135218	CD36	19.4452774	1.608604668	37.28195013	4.542817065	0.788710226	5.75980495	8.42E-09	3.93E-08
ENSG00000116032	GRIN3B	236.3425406	19.44844338	453.2366378	4.541452431	0.230097515	19.7370772	1.04E-86	5.15E-85
ENSG00000129951	PLPPR3	135.4708166	11.136199	259.8054342	4.540335885	0.303881283	14.9411502	1.78E-50	4.33E-49
ENSG00000204338	CYP21A1P	2.02842156	0	4.056843121	4.526777167	1.880455435	2.40727703	0.016072	0.03506
ENSG00000286107	AC007655.2	2.027242461	0	4.054484922	4.52607335	1.880650833	2.40665267	0.016099	0.035108
ENSG00000130751	NPAS1	112.8421414	9.424308066	216.2599747	4.503361986	0.339220152	13.2756323	3.21E-40	6.05E-39
ENSG00000124440	HIF3A	267.1347691	22.95476411	511.3147742	4.491332875	0.216820626	20.7145093	2.56E-95	1.48E-93
ENSG00000168280	KIF5C	45.01885286	3.898177143	86.13952858	4.486872106	0.518182672	8.65886173	4.76E-18	3.99E-17
ENSG00000204257	HLA-DMA	115.1138572	10.03450901	220.1932053	4.472361059	0.321636621	13.9050119	5.91E-44	1.23E-42
ENSG00000242021	AC112493.1	18.71800327	1.691143527	35.74486302	4.468197369	0.818752991	5.45732036	4.83E-08	2.11E-07
ENSG00000258667	HIF1A-AS2	3.928389133	0.295380681	7.561397584	4.459754678	1.544838745	2.88687392	0.003891	0.009563
ENSG00000258451	AL163636.1	1.92709885	0	3.854197699	4.445404099	1.946057348	2.284313	0.022353	0.047309
ENSG00000152578	GRIA4	1.925919751	0	3.851839501	4.444756234	1.966321551	2.26044221	0.023794	0.049959
ENSG00000221887	HMSD	29.26559913	2.716654417	55.81454384	4.429585255	0.654061135	6.77243306	1.27E-11	7.28E-11
ENSG00000226953	NCKAP5-AS2	1.87967171	0	3.75934342	4.414504434	1.942188662	2.27295346	0.023029	0.048578
ENSG00000186897	C1QL4	1.876134413	0	3.752268826	4.412303241	1.910089426	2.30999826	0.020888	0.044496
ENSG00000172828	CES3	3.807472213	0.323795675	7.291148751	4.404827068	1.567421346	2.81023802	0.00495	0.011936
ENSG00000175229	GAL3ST3	1.851241744	0	3.702483488	4.395814355	1.942048037	2.26349414	0.023605	0.049629
ENSG00000151715	TMEM45B	13.97694742	1.274435289	26.67945954	4.387518751	0.904492163	4.85080903	1.23E-06	4.72E-06
ENSG00000166816	LDHD	31.30153039	2.919122535	59.68393824	4.37254178	0.613644844	7.12552516	1.04E-12	6.43E-12
ENSG00000163975	MELTF	622.1617865	57.73070897	1186.592864	4.366692355	0.137036758	31.8651173	8.13E-223	1.78E-220
ENSG00000215861	AC245297.1	27.31540351	2.455958015	52.17484901	4.364822004	0.65438894	6.670073	2.56E-11	1.44E-10
ENSG00000115009	CCL20	2812.234483	264.7254096	5359.743556	4.339009998	0.069891946	62.0816878	0	0
ENSG00000151090	THRB	16.63748129	1.562148387	31.7128142	4.318275682	0.813494698	5.30830218	1.11E-07	4.70E-07
ENSG00000087085	ACHE	387.4136987	37.39837889	737.4290185	4.312117413	0.173328891	24.8839807	1.11E-136	1.15E-134
ENSG00000124882	EREG	16.54886234	1.65506095	31.44266373	4.289514282	0.820225563	5.22967641	1.70E-07	7.11E-07
ENSG00000105605	CACNG7	379.8895253	36.99560752	722.7834431	4.287433176	0.171046884	25.0658362	1.17E-138	1.28E-136
ENSG00000152207	CYSLTR2	6.405052602	0.637217645	12.17288756	4.258021368	1.318532761	3.22936334	0.001241	0.003314
ENSG00000229848	AC139149.1	3.399207883	0.295380681	6.503035084	4.243239623	1.596148371	2.6584243	0.007851	0.018273
ENSG00000110881	ASIC1	91.21383501	9.325126216	173.1025438	4.222932027	0.342632179	12.324972	6.65E-35	1.07E-33
ENSG00000261645	DISC1FP1	6.09571273	0.637217645	11.55420782	4.175151037	1.333952466	3.12990991	0.001749	0.004545
ENSG00000258881	AC007040.2	6.066685728	0.665632638	11.46773882	4.150349088	1.305523184	3.17906962	0.001477	0.003893
ENSG00000277297	ATP5F1AP10	5.984166177	0.647591349	11.320741	4.143351134	1.317089818	3.14583795	0.001656	0.004328
ENSG00000197632	SERPINB2	537.640537	58.80894542	1016.472129	4.112876352	0.138655789	29.6624929	2.34E-193	3.94E-191
ENSG00000074181	NOTCH3	1788.741013	196.6204292	3380.861596	4.109708395	0.086690772	47.4065265	0	0
ENSG00000159307	SCUBE1	43.3327807	4.712154033	81.95340736	4.102428724	0.480173241	8.54364295	1.30E-17	1.07E-16
ENSG00000214491	SEC14L6	3.084060023	0.341836963	5.826283083	4.084351522	1.643553119	2.48507424	0.012952	0.02886
ENSG00000270547	LINC01235	1407.020459	157.690212	2656.350706	4.077963443	0.173686246	23.4789083	6.70E-122	5.72E-120
ENSG00000135144	DTX1	3.043669152	0.295380681	5.791957622	4.077269622	1.648509056	2.47330738	0.013387	0.029738
ENSG00000116661	FBXO2	390.8876616	43.49258422	738.2827389	4.076612352	0.291638138	13.9783239	2.11E-44	4.44E-43
ENSG00000285018	AC245140.3	5.442553565	0.590761363	10.29434577	4.041266074	1.376966542	2.93490506	0.003336	0.008279
ENSG00000196154	S100A4	5.554085146	0.637217645	10.47095265	4.033615463	1.342523818	3.00450197	0.00266	0.006722
ENSG00000154096	THY1	5.532021131	0.647591349	10.41645091	4.020014621	1.344203383	2.99062975	0.002784	0.007016
ENSG00000147145	LPAR4	5.458707112	0.637217645	10.28019658	4.011407709	1.322174074	3.03394824	0.002414	0.006145
ENSG00000168032	ENTPD3	2.868362105	0.341836963	5.394887246	3.976856016	1.724978291	2.30545279	0.021141	0.044969
ENSG00000174807	CD248	13.22954227	1.59056338	24.86852116	3.961659315	0.868244565	4.56283803	5.05E-06	1.80E-05
ENSG00000220378	KRT8P42	5.36386196	0.665632638	10.06209128	3.961012323	1.329428059	2.97948603	0.002887	0.007257
ENSG00000135916	ITM2C	2017.67257	246.156839	3789.188301	3.944542689	0.073868937	53.3992077	0	0
ENSG00000167157	PRRX2	10.36836637	1.266767705	19.46996503	3.930871137	0.974173832	4.03508184	5.46E-05	0.000174
ENSG00000157613	CREB3L1	315.4790972	39.50979382	591.4484005	3.910959953	0.175268386	22.3141209	2.69E-110	1.96E-108
ENSG00000132840	BHMT2	2.732544018	0.295380681	5.169707354	3.910494793	1.679558569	2.32828724	0.019897	0.042574
ENSG00000128482	RNF112	2.73430518	0.323795675	5.144814686	3.904723923	1.686146079	2.31576847	0.020571	0.043864
ENSG00000183929	DUSP5P1	12.75388277	1.65506095	23.85270459	3.887329563	0.890036905	4.36760492	1.26E-05	4.30E-05
ENSG00000256379	TRAV8-5	4.924154122	0.637217645	9.211090599	3.853922001	1.436192737	2.68342953	0.007287	0.017023
ENSG00000166963	MAP1A	2030.371723	263.1361541	3797.607292	3.849490856	0.082710788	46.5415812	0	0
ENSG00000171873	ADRA1D	14.51752347	1.847155364	27.18789159	3.84138716	0.793809565	4.83917973	1.30E-06	4.99E-06
ENSG00000106976	DNM1	207.8539979	27.36247158	388.3455242	3.823728227	0.210506046	18.164458	9.87E-74	3.93E-72
ENSG00000134548	SPX	44.81565237	6.015004315	83.61630042	3.78953459	0.446576458	8.48574644	2.14E-17	1.74E-16
ENSG00000173610	UGT2A1	7.084267418	0.989428312	13.17910652	3.771689747	1.155634745	3.26373862	0.0011	0.002965
ENSG00000163710	PCOLCE2	4.710872709	0.665632638	8.756112781	3.761961599	1.447002678	2.59983043	0.009327	0.02139
ENSG00000250548	LINC01303	13.80402366	1.90398535	25.70406197	3.746567313	0.81529508	4.59535131	4.32E-06	1.55E-05
ENSG00000154118	JPH3	32.15465296	4.527727203	59.78157871	3.734339092	0.567751854	6.57741418	4.79E-11	2.65E-10
ENSG00000100626	GALNT16	116.7295816	16.373309	217.0858541	3.733574091	0.290751305	12.8411258	9.65E-38	1.70E-36
ENSG00000281903	LINC02246	27.06237876	3.797596995	50.32716052	3.720495897	0.56623614	6.57057301	5.01E-11	2.77E-10
ENSG00000181444	ZNF467	348.6584678	49.86444487	647.4524908	3.699502028	0.165111263	22.4061156	3.43E-111	2.54E-109
ENSG00000197635	DPP4	1291.148065	184.8238749	2397.472256	3.699246219	0.086704388	42.6650405	0	0
ENSG00000237813	AC002066.1	11.12608386	1.598230964	20.65393675	3.693433482	0.876843821	4.21219081	2.53E-05	8.40E-05
ENSG00000235385	LINC02154	521.6313109	74.56068775	968.7019341	3.69088035	0.145005233	25.4534286	6.47E-143	7.20E-141
ENSG00000225783	MIAT	8.693713251	1.209937719	16.17748878	3.688535075	1.043694032	3.53411533	0.000409	0.001171
ENSG00000163995	ABLIM2	4.264526525	0.590761363	7.938291687	3.667793117	1.422327895	2.57872543	0.009917	0.022627
ENSG00000239823	RF00019	8.4620558	1.181522726	15.74258888	3.660554513	1.082232871	3.3824091	0.000719	0.001987
ENSG00000165215	CLDN3	4.362956428	0.637217645	8.08869521	3.660542229	1.489425244	2.45768779	0.013983	0.030922
ENSG00000134242	PTPN22	265.978667	39.17833056	492.7790034	3.659170019	0.180758656	20.2434014	4.06E-91	2.19E-89
ENSG00000224081	SLC44A3-AS1	6.485692394	0.942972031	12.02841276	3.658065665	1.20708336	3.03049962	0.002441	0.006212
ENSG00000141576	RNF157	30.19726972	4.545768492	55.84877094	3.635971208	0.533182248	6.81937785	9.14E-12	5.31E-11
ENSG00000162409	PRKAA2	163.6697975	24.22099056	303.1186045	3.632340875	0.240088769	15.1291578	1.04E-51	2.65E-50
ENSG00000234695	AC002076.1	12.90637192	1.950441632	23.8623022	3.629904651	0.813626838	4.46138756	8.14E-06	2.85E-05
ENSG00000249279	LINC02057	4.258145601	0.637217645	7.879073557	3.627911122	1.419563832	2.55565198	0.010599	0.024035
ENSG00000144857	BOC	8.580672084	1.313223987	15.84812018	3.622998891	1.049608647	3.45176167	0.000557	0.001562
ENSG00000102057	KCND1	262.9534816	40.29179255	485.6151706	3.600097814	0.188716834	19.076718	3.94E-81	1.78E-79
ENSG00000147255	IGSF1	14.42520529	2.170951038	26.67945954	3.591702703	0.763178097	4.70624447	2.52E-06	9.33E-06
ENSG00000234380	LINC01426	4.032844975	0.590761363	7.474928587	3.589046688	1.488561793	2.41108344	0.015905	0.03474

ENSG00000268297	CLEC4GP1	46.69432356	7.075199484	86.31344764	3.580214447	0.494791381	7.23580601	4.63E-13	2.93E-12
ENSG00000133800	LYVE1	1593.331451	247.0618794	2939.601023	3.571582117	0.076494919	46.6904491	0	0
ENSG00000145506	NKD2	4.0665734	0.619176356	7.513970445	3.571556083	1.419954868	2.51526028	0.011894	0.026677
ENSG00000172508	CARNS1	8.202511375	1.266767705	15.13825504	3.570565652	1.020140168	3.50007358	0.000465	0.00132
ENSG00000139200	PIANP	263.8889147	41.22935234	486.548477	3.569785346	0.181569178	19.6607452	4.68E-86	2.31E-84
ENSG00000271225	BNIP3P4	6.245746296	0.989428312	11.50206428	3.567849046	1.171144977	3.04646232	0.002316	0.005916
ENSG00000228560	AL513323.1	47.1916367	7.421140866	86.96213253	3.560571773	0.42508544	8.37613203	5.47E-17	4.35E-16
ENSG00000228623	ZNF883	4.064326809	0.637217645	7.491435974	3.556534354	1.450069229	2.45266521	0.01418	0.031331
ENSG00000242574	HLA-DMB	37.05922816	5.913116345	68.20533998	3.555267181	0.490947802	7.24163988	4.43E-13	2.81E-12
ENSG00000163075	CFAP221	9.997745519	1.523359689	18.47213135	3.554032833	0.957644626	3.71122307	0.000206	0.000614
ENSG00000230109	LINC02643	13.97289033	2.124494756	25.82128591	3.553575688	0.783785395	4.53386311	5.79E-06	2.05E-05
ENSG00000107742	SPOCK2	80.75186132	12.60002258	148.9037	3.550991123	0.329960876	10.7618551	5.21E-27	6.36E-26
ENSG00000125430	HS3ST3B1	4.033538645	0.637217645	7.429859646	3.546761375	1.43504001	2.4715418	0.013453	0.029866
ENSG00000188747	NOXA1	70.90010773	11.21107028	130.5891452	3.546212249	0.369389185	9.60020595	7.98E-22	7.92E-21
ENSG00000129194	SOX15	4.081659455	0.683673926	7.479644983	3.521040486	1.442566079	2.44081747	0.014654	0.032277
ENSG00000237786	GFOD1-AS1	4.000813558	0.647591349	7.354035766	3.513935737	1.508979067	2.32868422	0.019876	0.042534
ENSG00000226963	AC078883.2	3.92319529	0.619176356	7.227214224	3.513838305	1.432824765	2.45238524	0.014191	0.031348
ENSG00000183090	FREM3	9.99558059	1.673102239	18.31805894	3.49725829	0.915248028	3.82110443	0.000133	0.000405
ENSG00000236393	AC091806.1	57.0731798	9.353541209	104.7928184	3.496315676	0.379873214	9.20390159	3.45E-20	3.16E-19
ENSG00000138759	FRAS1	556.1074396	90.32293876	1021.89194	3.493774934	0.127183684	27.4703077	3.97E-166	5.69E-164
ENSG00000161544	CYGB	23.52327389	3.836385693	43.21016209	3.493612972	0.594272271	5.87880866	4.13E-09	1.97E-08
ENSG00000105696	TMEM59L	310.0578802	50.46147552	569.6542849	3.490964985	0.1684473	20.7243748	2.09E-95	1.22E-93
ENSG00000130203	APOE	1047.837058	171.8951856	1923.778929	3.489048319	0.112477232	31.0200406	2.89E-211	5.68E-209
ENSG00000166924	NYAP1	284.8155918	46.70406552	522.9271181	3.487226016	0.172578904	20.2065602	8.57E-91	4.59E-89
ENSG00000171724	VAT1L	87.98149189	14.41605683	161.5469269	3.481849521	0.692302327	5.02937717	4.92E-07	1.97E-06
ENSG00000260877	AP005233.2	3.809999182	0.619176356	7.000822007	3.477009509	1.512418329	2.2989734	0.021506	0.045695
ENSG00000007312	CD79B	9.466779008	1.523359689	17.41019833	3.471841991	0.939679167	3.69470998	0.00022	0.000652
ENSG00000111087	GLI1	32.45297179	5.3132891	59.59265449	3.466972152	0.511282906	6.78092718	1.19E-11	6.88E-11
ENSG00000074370	ATP2A3	71.20746015	11.82754051	130.5873798	3.466460674	0.351874906	9.85140065	6.76E-23	7.02E-22
ENSG00000274355	FAM74A3	132.8288347	22.07489135	243.5827781	3.465879505	0.250745426	13.822304	1.87E-43	3.85E-42
ENSG00000109472	CPE	426.1640056	71.18173854	781.1462726	3.460314426	0.145412004	23.7966215	3.62E-125	3.18E-123
ENSG00000121904	CSMD2	59.47205903	9.941596451	109.0025216	3.460290769	0.373764178	9.25795187	2.08E-20	1.93E-19
ENSG00000204789	ZNF204P	9.370036987	1.533733394	17.20634058	3.455052563	0.970162342	3.56131383	0.000369	0.001063
ENSG00000115844	DLX2	5.665067728	0.961013319	10.36912214	3.440819164	1.249393815	2.75399087	0.005887	0.014002
ENSG00000168772	CXXC4	5.685121539	0.989428312	10.38081476	3.418531019	1.26071853	2.71157355	0.006696	0.015754
ENSG00000128268	MGAT3	3.789943643	0.665632638	6.914254648	3.416223532	1.440756892	2.37113114	0.017734	0.038338
ENSG00000260788	AC009063.2	9.318707855	1.626645957	17.01076975	3.407685363	0.922460321	3.69412677	0.000221	0.000653
ENSG00000168993	CPLX1	228.8442736	39.84666932	417.8418779	3.397225518	0.186758102	18.1905121	6.14E-74	2.46E-72
ENSG00000102109	PCSK1N	9.299608201	1.637019662	16.96219674	3.39488742	0.9428276	3.600751	0.000317	0.000923
ENSG00000197332	AC008543.1	5.461655356	0.942972031	9.980338681	3.388047681	1.187510807	2.85306682	0.00433	0.010544
ENSG00000198133	TMEM229B	3.575910253	0.619176356	6.532644149	3.368875435	1.457318761	2.31169427	0.020795	0.044316
ENSG00000185519	FAM131C	34.08113743	6.004630611	62.15764425	3.362893987	0.480166057	7.00360623	2.49E-12	1.50E-11
ENSG00000204219	TCEA3	152.9676277	27.12112429	278.8141311	3.356640582	0.554030581	6.05858358	1.37E-09	6.81E-09
ENSG00000179111	HES7	37.50727271	6.644554376	68.36999105	3.355256848	0.472992622	7.09367693	1.31E-12	8.03E-12
ENSG00000091879	ANGPT2	31940.21172	5690.472785	58189.95066	3.354398398	0.030640104	109.477382	0	0
ENSG00000166960	CCDC178	25.08070512	4.594930894	45.56647934	3.332919119	0.595839013	5.59365709	2.22E-08	1.00E-07
ENSG00000166145	SPINT1	1129.344042	205.9526818	2052.735402	3.320250947	0.087571281	37.9148383	0	0
ENSG00000006534	ALDH3B1	24.4946206	4.509685915	44.47955529	3.310326241	0.554380999	5.97121158	2.35E-09	1.15E-08
ENSG00000141574	SECTM1	148.6222147	27.30022935	269.9442001	3.300249129	0.230922143	14.2916097	2.47E-46	5.40E-45
ENSG00000049540	ELN	6.965852386	1.284808994	12.64689578	3.30006716	1.104280253	2.98843265	0.002804	0.007061
ENSG00000251435	C1GALT1P2	5.000665427	0.886142044	9.115188809	3.297394519	1.294642965	2.5469528	0.010867	0.024571
ENSG00000129451	KLK10	12.13587539	2.299946179	21.9718046	3.282190495	0.839855927	3.90803993	9.30E-05	0.000289
ENSG00000184060	ADAP2	21.04442128	4.037545988	38.05129657	3.278520219	0.683553284	4.79629064	1.62E-06	6.13E-06
ENSG00000080031	PTPRH	100.7461056	18.86309425	182.629117	3.276754773	0.276324158	11.8583724	1.95E-32	2.90E-31
ENSG00000132170	PPARG	52.80252345	9.998426438	95.60662046	3.267496058	0.374868479	8.71637984	2.87E-18	2.43E-17
ENSG00000141338	ABCA8	95.33219844	17.89256428	172.7718326	3.256479982	0.296201244	10.9941469	4.08E-28	5.17E-27
ENSG00000163520	FBLN2	123.9046862	23.54412717	224.2652451	3.249187039	0.246617398	13.1750114	1.22E-39	2.27E-38
ENSG00000085741	WNT11	13.43070213	2.605700565	24.25570369	3.235972261	0.747393806	4.32967498	1.49E-05	5.08E-05
ENSG00000183760	ACP7	17.99193106	3.40930375	32.57455836	3.228450176	0.673464499	4.79379415	1.64E-06	6.21E-06
ENSG00000231789	PIK3CD-AS2	13.19767156	2.559244283	23.83609884	3.22058214	0.749213711	4.29861612	1.72E-05	5.81E-05
ENSG00000056998	GYG2	20.17174365	4.001463411	36.34202389	3.219767163	0.641013328	5.02293326	5.09E-07	2.03E-06
ENSG00000104804	TULP2	11.74966923	2.346402461	21.152936	3.212925163	0.847701895	3.79015923	0.000151	0.000456
ENSG00000204161	TMEM273	210.4300136	40.8278888	380.0321383	3.210553373	0.195688092	16.4064831	1.72E-60	5.29E-59
ENSG00000132563	REEP2	180.0931772	35.23910938	324.947245	3.208426326	0.211228147	15.1893882	4.16E-52	1.07E-50
ENSG00000088320	REM1	6.501907223	1.256394001	11.74742044	3.208424971	1.047867463	3.06186143	0.0022	0.005642
ENSG00000258789	AL162171.2	9.884886369	1.940067927	17.82970481	3.208303098	0.901832788	3.55753654	0.000374	0.001078
ENSG00000111907	TPD52L1	22.65890693	4.409105767	40.9087081	3.201796692	0.596658656	5.36621176	8.04E-08	3.45E-07
ENSG00000240476	LINC00973	13.2798756	2.670198135	23.88955307	3.199362965	0.780721128	4.09795873	4.17E-05	0.000135
ENSG00000072195	SPEG	21.13537724	4.178222656	38.09253182	3.196345498	0.635627036	5.02864937	4.94E-07	1.98E-06
ENSG00000168542	COL3A1	112.8835311	22.07845452	203.6886078	3.195710863	0.266747512	11.9802837	4.51E-33	6.84E-32
ENSG00000272914	AL359532.1	6.485141667	1.295182698	11.67510064	3.183987837	1.116257675	2.85237711	0.004339	0.010566
ENSG00000163072	NOSTRIN	30.29411506	5.996963027	54.59126709	3.176547814	0.499323093	6.3617082	2.00E-10	1.06E-09
ENSG00000170011	MYRIP	266.9296003	53.26012754	480.5990731	3.163760611	0.179769015	17.5990318	2.51E-69	9.36E-68
ENSG00000144681	STAC	26.83204713	5.349371677	48.31472259	3.162780062	0.519926275	6.08313181	1.18E-09	5.87E-09
ENSG00000114378	HYAL1	71.93532544	14.69750065	129.1731502	3.135983935	0.333632062	9.39952808	5.48E-21	5.24E-20
ENSG00000165272	AQP3	17.41656853	3.631211455	31.2019256	3.130740378	0.673717778	4.64696121	3.37E-06	1.23E-05
ENSG00000253304	TMEM200B	94.22158622	19.68753532	168.7556371	3.120941063	0.314451032	9.92504633	3.24E-23	3.41E-22
ENSG00000274583	FAM74A4	41.23290341	8.396091056	74.06971577	3.119139492	0.4330156	7.20329589	5.88E-13	3.70E-12
ENSG00000141293	SKAP1	12.51634898	2.634115558	22.3985824	3.118328495	0.784407377	3.97539415	7.03E-05	0.000221
ENSG00000285793	AC125232.2	7.642064514	1.59056338	13.69356565	3.103318643	1.032168874	3.00659972	0.002642	0.006678
ENSG00000171388	APLN	37033.63317	7760.970955	66306.29538	3.094916659	0.0404229	76.5634496	0	0
ENSG00000091622	PITPNM3	127.5732949	26.86462278	228.281967	3.088160412	0.24012246	12.8607728	7.48E-38	1.32E-36
ENSG00000078596	ITM2A	52.34789892	10.92962647	93.76617138	3.081877545	0.378473554	8.14291385	3.86E-16	2.93E-15
ENSG00000152128	TMEM163	201.4493302	42.69448375	360.2041767	3.075504255	0.196614208	15.6423296	3.75E-55	1.03E-53
ENSG00000171798	KNDC1	4.386727161	0.914557038	7.858897284	3.062589667	1.266401181	2.41834082	0.015591	0.034113

ENSG00000251314	AC104123.1	48.09319287	10.3894258	85.79695994	3.061113571	0.393930866	7.77068728	7.81E-15	5.50E-14
ENSG00000158458	NRG2	8.738012187	1.847155364	15.62886901	3.046373358	0.910766295	3.34484639	0.000823	0.002254
ENSG00000211445	GPX3	647.910563	141.5575281	1154.263598	3.028177587	0.108438413	27.9253219	1.31E-171	1.94E-169
ENSG00000150722	PPP1R1C	17.33372798	3.751140714	30.91631525	3.024694179	0.642621653	4.70680402	2.52E-06	9.30E-06
ENSG00000160469	BRSK1	43.72466238	9.607427072	77.84189769	3.022029494	0.42866898	7.04979747	1.79E-12	1.09E-11
ENSG00000198003	CCDC151	11.65401766	2.559244283	20.74879103	3.020077071	0.76603076	3.94250104	8.06E-05	0.000252
ENSG00000109321	AREG	19.07258611	4.250387811	33.89478442	3.015291098	0.618840248	4.87248706	1.10E-06	4.25E-06
ENSG00000159904	ZNF890P	8.632060588	1.893611645	15.37050953	3.012852765	0.892846023	3.37443712	0.00074	0.002041
ENSG00000224389	C4B	4.439078938	1.007469601	7.870688275	3.011119157	1.282270076	2.34827219	0.018861	0.040553
ENSG00000279108	AC008537.3	7.159839948	1.580189675	12.73949022	3.002076973	0.998857219	3.00551161	0.002651	0.006701
ENSG00000277511	AC116407.2	7.078029039	1.551774682	12.6042834	2.999551017	0.997368609	3.00746483	0.002634	0.006662
ENSG00000124143	ARHGAP40	15.59054987	3.448092448	27.73300729	2.990563824	0.706806212	4.23109443	2.33E-05	7.76E-05
ENSG00000259347	AC087482.1	4.249826609	0.942972031	7.556681188	2.98847699	1.274854111	2.34417175	0.019069	0.040956
ENSG00000205221	VIT	23.97626008	5.331330389	42.62118977	2.981926694	0.550378557	5.4179558	6.03E-08	2.62E-07
ENSG00000174175	SELP	232.5427163	52.59458538	412.4908473	2.970871587	0.186527535	15.9272548	4.10E-57	1.16E-55
ENSG00000105613	MAST1	26.55733021	5.968548034	47.14611239	2.96600766	0.520489094	5.69850108	1.21E-08	5.56E-08
ENSG00000121753	ADGRB2	446.8588559	102.0748414	791.6428703	2.95710152	0.127936903	23.1137494	3.37E-118	2.75E-116
ENSG00000105290	APLP1	1352.878342	309.3268103	2396.429874	2.956512974	0.081559349	36.2498352	1.00E-287	2.93E-285
ENSG00000169252	ADRB2	127.8416289	29.56052979	226.122728	2.941485478	0.233885147	12.5766237	2.84E-36	4.75E-35
ENSG00000261286	ATP2C2-AS1	12.39704626	2.864998669	21.92909386	2.934769354	0.805422999	3.64376155	0.000269	0.000788
ENSG00000127824	TUBA4A	1167.697194	274.8403372	2060.554051	2.908944574	0.087375078	33.2926122	4.94E-243	1.13E-240
ENSG00000123384	LRP1	5820.178959	1373.489644	10266.86827	2.903608358	0.052843679	54.9471268	0	0
ENSG00000125848	FLRT3	24.03492921	5.608669782	42.46118864	2.900852097	0.542142874	5.35071516	8.76E-08	3.75E-07
ENSG00000161958	FGF11	9.683200023	2.356776165	17.00962388	2.892120616	0.887375461	3.2591848	0.001117	0.003008
ENSG00000103145	HCFC1R1	1084.09514	256.9216131	1911.268666	2.890121135	0.095175851	30.3661181	1.54E-202	2.85E-200
ENSG00000167600	CYP2S1	62.15132138	14.79172103	109.5109217	2.875459412	0.350895901	8.19462241	2.51E-16	1.94E-15
ENSG00000125731	SH2D3A	17.07769058	4.100645261	30.05473591	2.863124466	0.641611146	4.46239827	8.10E-06	2.83E-05
ENSG00000111058	ACSS3	13.63644382	3.382287055	23.89060058	2.861889904	0.780722786	3.66569281	0.000247	0.000726
ENSG00000255408	PCDHA3	10.53260323	2.551576699	18.51362977	2.858875864	0.811865251	3.52136745	0.000429	0.001224
ENSG00000158825	CDA	19.83391123	4.843855294	34.82396718	2.854890099	0.589793376	4.84049197	1.30E-06	4.97E-06
ENSG00000225968	ELFN1	184.0720603	44.91328936	323.2308313	2.852229982	0.199319294	14.309854	1.90E-46	4.17E-45
ENSG00000101307	SIRPB1	41.80312108	10.17518568	73.43105648	2.849759652	0.420340795	6.77959092	1.20E-11	6.93E-11
ENSG00000130649	CYP2E1	9.285596765	2.328361172	16.24283236	2.838668139	0.903062328	3.14338009	0.00167	0.004358
ENSG00000173868	PHOSPHO1	50.70755677	12.44175541	88.97335813	2.837752833	0.383658095	7.39656708	1.40E-13	9.14E-13
ENSG00000120068	HOXB8	148.8512604	36.80220529	260.9003155	2.835094679	0.222227714	12.7576108	2.83E-37	4.87E-36
ENSG00000272031	ANKRD34A	90.80178222	22.50964088	159.0939236	2.828086016	0.270287279	10.4632598	1.27E-25	1.48E-24
ENSG00000163154	TNFAIP8L2	5.111719561	1.256394001	8.967045122	2.820631588	1.124987172	2.50725667	0.012167	0.027241
ENSG00000265778	AC018413.1	7.746855178	1.940067927	13.55364243	2.819569311	0.940101131	2.99921915	0.002707	0.006835
ENSG00000181790	ADGRB1	31.13301449	7.799060406	54.46696856	2.818923206	0.464174814	6.0729775	1.26E-09	6.24E-09
ENSG00000099864	PALM	901.347295	224.5775411	1578.117049	2.812086915	0.093523232	30.0683248	1.26E-198	2.24E-196
ENSG00000011465	DCN	52.94582036	13.34733704	92.54430368	2.809532111	0.377715951	7.4382141	1.02E-13	6.71E-13
ENSG00000181856	SLC2A4	15.16333063	3.787223291	26.53943796	2.799453871	0.679212823	4.12161516	3.76E-05	0.000123
ENSG00000130768	SMPDL3B	11.43247055	2.883039958	19.98190114	2.795713606	0.769894191	3.63129588	0.000282	0.000825
ENSG00000006747	SCIN	11.58852734	2.947537528	20.22951715	2.795339215	0.83063622	3.36529897	0.000765	0.002104
ENSG00000104888	SLC17A7	5.117129628	1.302850282	8.931408973	2.795049483	1.138098398	2.4558944	0.014053	0.031062
ENSG00000005471	ABCB4	42.72869912	10.67303448	74.78436376	2.794345908	0.402002419	6.9510674	3.63E-12	2.16E-11
ENSG00000173114	LRRN3	16.19656113	4.0568951	28.33622715	2.78761886	0.666463301	4.18270422	2.88E-05	9.51E-05
ENSG00000086696	HSD17B2	165.3419061	42.06493369	288.6188785	2.776587197	0.210518608	13.1892721	1.01E-39	1.89E-38
ENSG00000148671	ADIRF	97.11385847	24.89483204	169.3328849	2.776174133	0.271203958	10.2364809	1.36E-24	1.52E-23
ENSG00000171345	KRT19	165.1516203	42.32973451	287.9735062	2.77510391	0.219057082	12.6684054	8.85E-37	1.50E-35
ENSG00000130035	GALNT8	7.507156322	1.960815336	13.05349731	2.753154016	0.972748541	2.83028337	0.004651	0.011269
ENSG00000131386	GALNT15	107.7341476	27.949219	187.5190763	2.752301778	0.256890391	10.7139149	8.76E-27	1.06E-25
ENSG00000122691	TWIST1	6.15491169	1.598230964	10.71159242	2.744910142	1.01034665	2.71680036	0.006592	0.015542
ENSG00000225265	TAF1A-AS1	6.152368604	1.608604668	10.69613254	2.738670154	1.029297735	2.66071717	0.007797	0.018153
ENSG00000091262	ABCC6	4.775008089	1.209937719	8.340078458	2.738192862	1.19157542	2.29796018	0.021564	0.045802
ENSG00000257732	AC089983.1	25.61966854	6.677930833	44.56140625	2.736701084	0.5337355	5.12744812	2.94E-07	1.20E-06
ENSG00000257261	AC008014.1	153.209837	39.9931645	266.4265094	2.733014689	0.229772986	11.8944125	1.27E-32	1.90E-31
ENSG00000281103	TRG-AS1	12.28208708	3.24291821	21.32125596	2.730846548	0.722035116	3.78215198	0.000155	0.000469
ENSG00000186765	FSCN2	6.211868256	1.673102239	10.75063427	2.71932571	1.057229167	2.57212513	0.010108	0.023017
ENSG00000167642	SPINT2	211.4175913	55.98142763	366.8537549	2.718088599	0.184261056	14.7512918	3.02E-49	7.11E-48
ENSG00000155980	KIF5A	28.8873177	7.644356393	50.130279	2.71166543	0.498557889	5.4390182	5.36E-08	2.34E-07
ENSG00000123977	DAW1	64.19169827	17.13952179	111.2438748	2.70876163	0.326400652	8.29888548	1.05E-16	8.24E-16
ENSG00000167971	CASKIN1	34.86875678	9.289043638	60.44846993	2.705204927	0.434982343	6.21911433	5.00E-10	2.57E-09
ENSG00000174326	SLC16A11	35.27590982	9.334101623	61.21771801	2.694850325	0.474672613	5.67728209	1.37E-08	6.27E-08
ENSG00000021645	NRXN3	237.4590737	63.52109946	411.397048	2.694501126	0.173906554	15.4939596	3.81E-54	1.03E-52
ENSG00000204314	PRRT1	45.19922515	11.93299165	78.46545865	2.690217531	0.442435156	6.08047868	1.20E-09	5.96E-09
ENSG00000184451	CCR10	79.01774364	21.05564975	136.9798375	2.689728444	0.292646093	9.19106221	3.89E-20	3.56E-19
ENSG00000163661	PTX3	44254.77878	11922.92759	76586.62997	2.683615632	0.03570274	75.1655366	0	0
ENSG00000125538	IL1B	1281.228497	348.6835237	2213.773469	2.66692305	0.076604811	34.8140411	1.49E-265	3.74E-263
ENSG00000138028	CGREF1	227.3105528	61.76631541	392.8547902	2.662639688	0.172523582	15.4334826	9.75E-54	2.62E-52
ENSG00000175779	C15orf53	35.58315771	9.606028775	61.56028665	2.657670693	0.44842828	5.9266349	3.09E-09	1.49E-08
ENSG00000185585	OLFML2A	63.6556186	17.53818874	109.7730485	2.657290418	0.345392886	7.69352967	1.43E-14	9.92E-14
ENSG00000099365	STX1B	10.33302351	2.790127394	17.87591962	2.655502818	0.786102983	3.37805971	0.00073	0.002016
ENSG00000177106	EPS8L2	214.8748412	59.0461883	370.7034942	2.654172005	0.181468087	14.6261089	1.91E-48	4.41E-47
ENSG00000172426	RSPH9	12.71520461	3.473801321	21.9566079	2.652367829	0.725786075	3.65447605	0.000258	0.000757
ENSG00000145362	ANK2	9.272415562	2.559244283	15.98558684	2.642306942	0.827561162	3.19288418	0.001409	0.003723
ENSG00000128011	LRFN1	85.74774276	23.75069971	147.7447858	2.641257998	0.273272952	9.66527415	4.23E-22	4.26E-21
ENSG00000235169	SMIM1	14.68271614	3.989691409	25.37574088	2.639098774	0.679163694	3.88580661	0.000102	0.000315
ENSG00000125878	TCF15	45.42733875	12.65599553	78.19868197	2.637238296	0.4185986	6.30016034	2.97E-10	1.56E-09
ENSG00000185664	PMEL	12.37242773	3.380888757	21.3639667	2.629295985	0.735136254	3.57661042	0.000348	0.001007
ENSG00000074410	CA12	536.6819063	150.6731503	922.6906622	2.621442404	0.12515726	20.9451884	2.08E-97	1.28E-95
ENSG00000250742	LINC02381	12.4350416	3.476507441	21.39357577	2.612824403	0.765416139	3.41359983	0.000641	0.001785
ENSG00000160867	FGFR4	327.7817206	92.22877318	563.3346681	2.60733039	0.146794043	17.7618269	1.40E-70	5.31E-69
ENSG00000062524	LTK	12.3009132	3.445386328	21.15644007	2.60598442	0.744587993	3.49990121	0.000465	0.001321
ENSG00000212766	EWSAT1	15.79629041	4.473603338	27.11897749	2.600857241	0.664579845	3.91353614	9.10E-05	0.000283

ENSG00000164142	FAM160A1	47.19723584	13.54213757	80.85233412	2.585089429	0.384623761	6.72108614	1.80E-11	1.03E-10
ENSG00000182985	CADM1	118.7599812	34.02646554	203.4934968	2.584663305	0.265276822	9.74326849	1.97E-22	2.00E-21
ENSG00000204060	FOXO6	13.31645548	3.789929411	22.84298156	2.581272352	0.705218374	3.66024546	0.000252	0.000741
ENSG00000266865	AC138207.8	26.89871003	7.734562836	46.06285722	2.580206077	0.487658267	5.29101269	1.22E-07	5.16E-07
ENSG00000186462	NAP1L2	67.21672729	19.43721263	114.9962419	2.576057941	0.32348388	7.96348165	1.67E-15	1.23E-14
ENSG00000186868	MAPT	14.49057534	4.175516536	24.80563415	2.575707627	0.656727085	3.92203655	8.78E-05	0.000274
ENSG00000139537	CCDC65	9.88754091	2.808168683	16.96691314	2.574806457	0.793575802	3.24456271	0.001176	0.003154
ENSG00000089041	P2RX7	420.754757	120.8728969	720.6366172	2.572802265	0.12720872	20.2250465	5.89E-91	3.16E-89
ENSG00000167612	ANKRD33	30.84904229	8.833546703	52.86453787	2.572318221	0.451553524	5.6965965	1.22E-08	5.62E-08
ENSG00000138356	AOX1	56.08191852	16.06886244	96.0949746	2.570472987	0.339739045	7.56602169	3.85E-14	2.60E-13
ENSG00000273143	AL355512.1	21.25804847	6.17512057	36.34097638	2.569020539	0.566084007	4.53823197	5.67E-06	2.01E-05
ENSG00000120885	CLU	8236.751336	2379.5489	14093.95377	2.566873401	0.03744411	68.5521275	0	0
ENSG00000267278	MAP3K14-AS1	9.942188459	2.883039958	17.00133696	2.566057186	0.796538966	3.22150867	0.001275	0.003398
ENSG00000036448	MYOM2	15.57226147	4.545768492	26.59875445	2.564041724	0.662139008	3.87236168	0.000108	0.000332
ENSG00000183807	FAM162B	16.4805914	4.805066596	28.1561162	2.554394607	0.604029394	4.22892434	2.35E-05	7.83E-05
ENSG00000272369	AC008035.1	43.39249495	12.78995213	73.99503776	2.552995543	0.406454094	6.28114117	3.36E-10	1.75E-09
ENSG00000152527	PLEKHH2	115.035821	33.66658729	196.4050548	2.547857461	0.257857609	9.88086978	5.04E-23	5.26E-22
ENSG00000130707	ASS1	313.1350448	91.14092959	535.12916	2.545545706	0.15830734	16.0797705	3.54E-58	1.03E-56
ENSG00000105737	GRIK5	66.53791222	19.47956449	113.59626	2.544078691	0.303615243	8.37928514	5.33E-17	4.24E-16
ENSG00000068831	RASGRP2	41.95172297	12.26089174	71.64255421	2.534478181	0.390326689	6.49322286	8.40E-11	4.55E-10
ENSG00000139549	DHH	94.4239853	27.9121889	160.9357817	2.523498072	0.28486549	8.85856014	8.11E-19	7.01E-18
ENSG00000178878	APOLD1	709.2255487	209.776213	1208.674884	2.523277366	0.097669939	25.8347388	3.61E-147	4.09E-145
ENSG00000197380	DACT3	151.7059111	44.99533148	258.4164907	2.521819276	0.249075049	10.1247366	4.29E-24	4.67E-23
ENSG00000112964	GHR	44.94765054	13.29407022	76.60123086	2.51932943	0.373841541	6.73903018	1.59E-11	9.10E-11
ENSG00000115468	EFHD1	21.82319992	6.43798184	37.208418	2.504926537	0.586642624	4.26993613	1.96E-05	6.57E-05
ENSG00000158270	COLEC12	7003.450184	2107.781758	11899.11861	2.498033564	0.040279168	62.0180035	0	0
ENSG00000147852	VLDLR	299.7315903	90.26168856	509.201492	2.49547847	0.151879096	16.4306908	1.15E-60	3.55E-59
ENSG00000240240	BX664727.3	5.30451717	1.598230964	9.010803376	2.494572999	1.079891926	2.31002097	0.020887	0.044496
ENSG00000021300	PLEKHB1	20.34918279	6.211203147	34.48716244	2.489642635	0.584150614	4.26198753	2.03E-05	6.80E-05
ENSG00000267060	PTGES3L	7.192692607	2.142536045	12.24284917	2.485482112	0.946339273	2.62641759	0.008629	0.019916
ENSG00000120217	CD274	1507.85249	458.0973734	2557.607606	2.481443845	0.070806049	35.0456478	4.54E-269	1.17E-266
ENSG00000258791	LINC00520	179.1410585	54.45256523	303.8295517	2.477745255	0.187800255	13.1935138	9.56E-40	1.79E-38
ENSG00000130475	FCHO1	98.63460076	29.96902919	167.3001723	2.47189897	0.263367644	9.38573522	6.25E-21	5.94E-20
ENSG00000143882	ATP6V1C2	8.539482327	2.662530551	14.4164341	2.468432957	0.874433728	2.82289312	0.004759	0.011506
ENSG00000124496	TRERF1	337.4496366	103.5377175	571.3615557	2.462514868	0.137408442	17.9211323	8.07E-72	3.12E-70
ENSG00000204381	LAYN	187.6877948	57.63639811	317.7391915	2.462118908	0.202537433	12.1563647	5.31E-34	8.31E-33
ENSG00000272636	DOC2B	71.43545052	21.88139864	120.9895024	2.460974058	0.301817415	8.15385042	3.53E-16	2.69E-15
ENSG00000233922	LINC01694	15.8034214	4.908352864	26.69848994	2.458857085	0.671157761	3.66360523	0.000249	0.000732
ENSG00000226800	CACTIN-AS1	11.12278952	3.370515053	18.87506399	2.455742254	0.767330455	3.20037115	0.001373	0.003637
ENSG00000030419	IKZF2	11.4312884	3.538298891	19.3242779	2.454843087	0.732699892	3.35040733	0.000807	0.002212
ENSG00000172350	ABCG4	68.65885442	21.33578573	115.9819231	2.45198585	0.309766738	7.91558793	2.46E-15	1.78E-14
ENSG00000229618	AC011287.1	6.039962401	1.847155364	10.23276944	2.443977883	1.007128609	2.42667904	0.015238	0.033412
ENSG00000163704	PRRT3	296.4968913	92.25457253	500.73921	2.442961457	0.149725962	16.3162182	7.57E-60	2.30E-58
ENSG00000198483	ANKRD35	47.71751324	14.79938862	80.63563787	2.436423497	0.365068987	6.67387148	2.49E-11	1.41E-10
ENSG00000233559	LINC00513	36.17813789	11.38381558	60.97246021	2.43623471	0.427013617	5.70528576	1.16E-08	5.35E-08
ENSG00000163083	INHBB	14.25446182	4.445188344	24.0637353	2.436190971	0.680975423	3.57750205	0.000347	0.001004
ENSG00000185669	SNAI3	18.44098736	5.812536197	31.06943851	2.428397412	0.607382848	3.99813301	6.38E-05	0.000202
ENSG00000159164	SV2A	831.8979638	260.3563099	1403.439618	2.427635583	0.090696456	26.7665979	7.91E-158	9.92E-156
ENSG00000162687	KCNT2	36.39675136	11.34362858	61.44987414	2.427275244	0.421357728	5.76060455	8.38E-09	3.92E-08
ENSG00000274736	CCL23	10.24128154	3.227583041	17.25498004	2.424639695	0.83507941	2.90348399	0.00369	0.009095
ENSG00000196468	FGF16	287.2588016	91.9985218	482.5190814	2.398526293	0.160220036	14.9702019	1.15E-50	2.81E-49
ENSG00000132692	BCAN	18.05135454	5.794494909	30.30821418	2.392474254	0.622569182	3.84290505	0.000122	0.000372
ENSG00000140379	BCL2A1	54.07028127	17.15476648	90.98579606	2.391482696	0.357199123	6.69509677	2.16E-11	1.22E-10
ENSG00000121236	TRIM6	140.9814224	45.31466243	236.6481823	2.390022635	0.209277555	11.4203486	3.31E-30	4.54E-29
ENSG00000151632	AKR1C2	5.976075328	1.922026639	10.03012402	2.385	0.981283765	2.43048962	0.015078	0.033082
ENSG00000171812	COL8A2	287.9328258	92.8318478	483.0338037	2.383816646	0.156626347	15.2197679	2.61E-52	6.74E-51
ENSG00000155657	TTN	71.7845812	23.14049876	120.4286636	2.373918899	0.305426819	7.77246381	7.70E-15	5.43E-14
ENSG00000108001	EBF3	5.947756969	1.940067927	9.955446012	2.370588506	0.987010714	2.40178599	0.016315	0.035513
ENSG00000118407	FILIP1	586.2834084	190.31036	982.2564567	2.368723076	0.111761348	21.1944748	1.07E-99	6.89E-98
ENSG00000251095	AC097478.1	36.40393595	11.69444095	61.11343094	2.362301941	0.4507277	5.24108445	1.60E-07	6.69E-07
ENSG00000137691	CFAP300	7.629579774	2.437916727	12.82124282	2.360061196	0.928972708	2.54050649	0.011069	0.024976
ENSG00000181885	CLDN7	585.5125913	191.2006065	979.8245761	2.359734291	0.113160966	20.8528998	1.43E-96	8.61E-95
ENSG00000142621	FHAD1	19.38654765	6.292343708	32.4807516	2.355630829	0.557628831	4.22437058	2.40E-05	7.98E-05
ENSG00000025039	RRAGD	79.54577711	25.92792003	133.1636342	2.354153859	0.278348903	8.45756472	2.73E-17	2.20E-16
ENSG00000006453	BAIAP2L1	120.6873292	39.86340279	201.5112556	2.348401868	0.238149413	9.86104413	6.14E-23	6.40E-22
ENSG00000135439	AGAP2	9.871273101	3.281706907	16.46083929	2.347947439	0.78686149	2.98393995	0.002846	0.007161
ENSG00000171435	KSR2	585.705	191.7355298	979.6744703	2.347155153	0.121553399	19.3096629	4.45E-83	2.08E-81
ENSG00000108176	DNAJC12	168.3062151	55.3707759	281.2416542	2.347095578	0.201426115	11.6523897	2.23E-31	3.17E-30
ENSG00000115290	GRB14	337.2221285	111.3134148	563.1308422	2.34238311	0.139087205	16.8411114	1.22E-63	4.02E-62
ENSG00000253490	LINC02099	6.830820405	2.263863602	11.39777721	2.341232114	0.91618512	2.55541382	0.010606	0.024046
ENSG00000276649	AL117335.1	10.76420146	3.569420005	17.95898291	2.340585907	0.801535419	2.92012786	0.003499	0.008658
ENSG00000251141	MRPS30-DT	11.72870783	3.900883263	19.55653239	2.340242917	0.712834094	3.28301205	0.001027	0.002781
ENSG00000198932	GPRASP1	132.2849995	43.45023235	221.1197667	2.338567162	0.227633496	10.2733877	9.29E-25	1.04E-23
ENSG00000127399	LRRC61	6.804748638	2.263863602	11.34563367	2.335967662	0.916009033	2.55015789	0.010767	0.024376
ENSG00000251432	LINC02615	25.38015802	8.438984171	42.32133187	2.335795431	0.511138215	4.5697922	4.88E-06	1.75E-05
ENSG00000174899	PQLC2L	97.75104779	32.19951633	163.3025793	2.335105609	0.253865864	9.19818668	3.64E-20	3.33E-19
ENSG00000151778	SERP2	9.761638389	3.253291914	16.26998486	2.334939588	0.783021781	2.98195995	0.002864	0.007204
ENSG00000249867	AC090833.1	41.38885315	13.88126841	68.89643789	2.321190902	0.399670595	5.80776001	6.33E-09	2.98E-08
ENSG00000275888	AC132872.3	12.18008552	4.000065113	20.36010593	2.320097912	0.719815521	3.22318406	0.001268	0.003379
ENSG00000106789	CORO2A	207.7550206	69.29299788	346.2170434	2.318187689	0.175112727	13.2382593	5.28E-40	9.89E-39
ENSG00000222019	URAHP	12.1301153	4.010438818	20.24979178	2.313080657	0.750469251	3.08217912	0.002055	0.005288
ENSG00000072954	TMEM38A	86.56152764	28.98370529	144.13935	2.312653574	0.261206107	8.85375001	8.46E-19	7.31E-18
ENSG00000141854	MISP3	9.6320465	3.271333203	15.9927598	2.310516185	0.800014231	2.88809385	0.003876	0.009528
ENSG00000145703	IQGAP2	777.7605094	261.4139799	1294.107039	2.307278897	0.093652733	24.6365357	5.13E-134	5.04E-132
ENSG00000129538	RNASE1	3155.317304	1065.768236	5244.866372	2.297567348	0.055160076	41.6527224	0	0

ENSG00000147113	DIPK2B	10504.96512	3553.972619	17455.95761	2.295723746	0.037408422	61.3691682	0	0
ENSG00000165092	ALDH1A1	12276.34003	4163.149794	20389.53026	2.292212969	0.038869779	58.9715982	0	0
ENSG00000164692	COL1A2	7788.880544	2657.547724	12920.21336	2.281529305	0.040988361	55.6628578	0	0
ENSG00000239264	TXNDC5	1440.941322	491.9438726	2389.938771	2.2804701	0.855235832	2.666481	0.007665	0.017858
ENSG00000240875	LINC00886	31.57867055	10.76324093	52.39410018	2.27850507	0.432379068	5.26969328	1.37E-07	5.77E-07
ENSG00000205710	C17orf107	244.4283493	83.85352001	405.0031786	2.277026971	0.164120615	13.874107	9.09E-44	1.88E-42
ENSG00000178568	ERBB4	15.79854193	5.388160375	26.20892349	2.27599924	0.610949071	3.72535019	0.000195	0.000582
ENSG00000013588	GPRC5A	1048.014796	359.1424531	1736.887139	2.275100086	0.086933837	26.1704781	5.76E-151	6.78E-149
ENSG00000108852	MPP2	71.41835299	24.4379368	118.3987692	2.272139077	0.286416069	7.9330014	2.14E-15	1.56E-14
ENSG00000237803	LINC00211	37.03256084	12.66722628	61.39789541	2.269673576	0.419088916	5.41573277	6.10E-08	2.65E-07
ENSG00000198185	ZNF334	9.11270181	3.09317566	15.13222796	2.269668305	0.855683598	2.65246209	0.007991	0.018578
ENSG00000155367	PPM1J	48.04980498	16.49053214	79.60907782	2.268748347	0.37651586	6.02563819	1.68E-09	8.28E-09
ENSG00000143416	SELENBP1	669.1903986	230.4123135	1107.968484	2.268614782	0.099738629	22.7455981	1.59E-114	1.25E-112
ENSG00000196549	MME	1209.535671	415.8632576	2003.208085	2.267701196	0.074743383	30.3398256	3.42E-202	6.27E-200
ENSG00000262728	AC123768.3	7.503372775	2.598032981	12.40871257	2.267536586	0.897245786	2.52721899	0.011497	0.025867
ENSG00000212864	RNF208	119.4757724	41.17739334	197.7741515	2.257875875	0.240100774	9.40386755	5.26E-21	5.03E-20
ENSG00000163629	PTPN13	386.1824681	134.0462712	638.318665	2.254750189	0.130891716	17.2260725	1.69E-66	5.89E-65
ENSG00000072071	ADGRL1	915.9927256	318.0918455	1513.893606	2.253015782	0.094400159	23.8666525	6.80E-126	6.11E-124
ENSG00000185532	PRKG1	44.34181354	15.43304309	73.25058399	2.252159105	0.370246106	6.08287048	1.18E-09	5.88E-09
ENSG00000077616	NAALAD2	39.23261345	13.7383364	64.72689051	2.24844537	0.457784438	4.91158104	9.03E-07	3.52E-06
ENSG00000236098	AC097059.2	19.17125085	6.65222196	31.69027973	2.246033202	0.543789128	4.13033856	3.62E-05	0.000118
ENSG00000281358	RASSF1-AS1	7.978736437	2.733297408	13.22417547	2.244470481	0.912531031	2.45961003	0.013909	0.030785
ENSG00000258704	SRP54-AS1	24.85245122	8.695576155	41.00932629	2.243975001	0.478322549	4.69134271	2.71E-06	9.99E-06
ENSG00000188488	SERPINA5	27.63873609	9.656589474	45.62088272	2.242878064	0.501972243	4.46813165	7.89E-06	2.76E-05
ENSG00000137285	TUBB2B	237.1966198	82.77970376	391.6135358	2.242759764	0.174944295	12.8198508	1.27E-37	2.22E-36
ENSG00000205517	RGL3	25.69003761	8.993662957	42.38641227	2.240439484	0.487093195	4.59961154	4.23E-06	1.52E-05
ENSG00000088340	FER1L4	100.2391882	35.03664126	165.4417352	2.240231473	0.642696482	3.48567564	0.000491	0.001387
ENSG00000243592	RPL17P22	8.102117316	2.808168683	13.39606595	2.238117488	0.86129666	2.59854425	0.009362	0.021462
ENSG00000275763	C18orf65	11.80964996	4.129060254	19.49023967	2.237308419	0.725154657	3.08528449	0.002034	0.005238
ENSG00000169306	IL1RAPL1	26.30690077	9.167716082	43.44608546	2.236784091	0.479098794	4.66873246	3.03E-06	1.11E-05
ENSG00000104332	SFRP1	699.1310034	244.6839051	1153.578102	2.236247699	0.103992707	21.5038897	1.43E-102	9.44E-101
ENSG00000156113	KCNMA1	110.846268	38.95782116	182.7347148	2.235995111	0.234396136	9.53938556	1.44E-21	1.41E-20
ENSG00000171611	PTCRA	10.13969143	3.602796461	16.67658639	2.231383459	0.797746774	2.79710747	0.005156	0.012393
ENSG00000166126	AMN	7.314814117	2.579991692	12.04963654	2.229960691	0.919764489	2.42449096	0.01533	0.033599
ENSG00000237352	LINC01358	37.18065854	13.12001709	61.24129999	2.224750382	0.395474209	5.62552584	1.85E-08	8.37E-08
ENSG00000166510	CCDC68	405.6868516	143.2387487	668.1349545	2.222949501	0.121047081	18.3643379	2.54E-75	1.03E-73
ENSG00000225518	LINC01703	6.423831809	2.310319884	10.53734373	2.216330925	0.975707819	2.27151088	0.023116	0.048724
ENSG00000213468	FIRRE	24.98901985	8.818211535	41.15982817	2.214299901	0.526986535	4.20181495	2.65E-05	8.77E-05
ENSG00000235448	LURAP1L-AS1	18.12939283	6.447047722	29.81173794	2.214262262	0.624834077	3.5437604	0.000394	0.001132
ENSG00000187634	SAMD11	8.923531143	3.160379351	14.68668294	2.210865354	0.799916895	2.76386881	0.005712	0.013616
ENSG00000107779	BMPR1A	12.25622301	4.313487083	20.19895893	2.204242148	0.717845253	3.07063693	0.002136	0.005484
ENSG00000102349	KLF8	11.01572772	4.001463411	18.02999203	2.199128373	0.7712654	2.85132507	0.004354	0.010596
ENSG00000196460	RFX8	10.72250384	3.844053277	17.60095439	2.196776968	0.721384337	3.0452241	0.002325	0.005935
ENSG00000267577	AC010327.3	26.54917405	9.517220629	43.58112747	2.191237931	0.460746681	4.75584095	1.98E-06	7.40E-06
ENSG00000280407	AC132872.4	16.8401267	6.053793013	27.62646038	2.190892155	0.610102422	3.59102353	0.000329	0.000956
ENSG00000008516	MMP25	16.71304368	6.004630611	27.42145676	2.185523618	0.615214436	3.55245828	0.000382	0.001097
ENSG00000162595	DIRAS3	56.97022982	20.4184321	93.52202754	2.183759903	0.333328207	6.55138046	5.70E-11	3.13E-10
ENSG00000167103	PIP5KL1	40.828732	14.78545175	66.87201225	2.181759834	0.378380723	5.76604384	8.12E-09	3.80E-08
ENSG00000090539	CHRD	50.23810807	18.16363438	82.31258175	2.179897463	0.341293714	6.38715972	1.69E-10	8.99E-10
ENSG00000174370	C11orf45	51.70052574	18.6150269	84.78602458	2.179860231	0.340482549	6.40226713	1.53E-10	8.17E-10
ENSG00000265962	GACAT2	9.708038168	3.509883898	15.90619244	2.178919127	0.780723674	2.7908967	0.005256	0.012624
ENSG00000161681	SHANK1	191.3629252	69.26101972	313.4648307	2.178788953	0.177879714	12.2486646	1.71E-34	2.72E-33
ENSG00000128918	ALDH1A2	615.4018242	222.7781054	1008.025543	2.176258873	0.118855445	18.3101318	6.87E-75	2.78E-73
ENSG00000182472	CAPN12	14.21929135	5.185692257	23.25289043	2.172109558	0.702827749	3.09052902	0.001998	0.005151
ENSG00000249631	AC005699.1	18.41497786	6.691010658	30.13894507	2.171159668	0.556019406	3.90482714	9.43E-05	0.000292
ENSG00000147642	SYBU	70.11563166	25.42240365	114.8088597	2.17070363	0.302003144	7.18768553	6.59E-13	4.14E-12
ENSG00000163814	CDCP1	166.659804	60.60197692	272.7176311	2.170171788	0.191592846	11.326998	9.65E-30	1.30E-28
ENSG00000145358	DDIT4L	575.5815479	209.9526119	941.2104839	2.163114289	0.105086198	20.5841902	3.80E-94	2.14E-92
ENSG00000120756	PLS1	37.01676668	13.60392902	60.42960434	2.162239613	0.398831519	5.42143614	5.91E-08	2.57E-07
ENSG00000167964	RAB26	31.59556328	11.41439544	51.77673112	2.161897392	0.500093904	4.32298289	1.54E-05	5.23E-05
ENSG00000180448	ARHGAP45	645.3249573	235.5770774	1055.072837	2.161873855	0.108403433	19.9428542	1.73E-88	8.88E-87
ENSG00000205639	MFSD2B	9.605871108	3.530631307	15.68111091	2.15684754	0.81541011	2.64510767	0.008166	0.018952
ENSG00000160255	ITGB2	25.89724218	9.413934361	42.38055	2.156808463	0.496826604	4.34116943	1.42E-05	4.83E-05
ENSG00000180881	CAPS2	17.67309848	6.532292701	28.81390426	2.154463256	0.602361915	3.57669236	0.000348	0.001007
ENSG00000115194	SLC30A3	56.6863533	20.83924476	92.53346184	2.153913176	0.315880644	6.81875644	9.18E-12	5.34E-11
ENSG00000101104	PABPC1L	109.2920821	40.11719818	178.466966	2.153284476	0.612800098	3.51384486	0.000442	0.001257
ENSG00000196502	SULT1A1	22.70057665	8.400195474	37.00095782	2.146266461	0.511185916	4.19860249	2.69E-05	8.89E-05
ENSG00000272084	AL137127.1	24.48880174	9.094243105	39.88336038	2.145313586	0.488637137	4.39040225	1.13E-05	3.89E-05
ENSG00000243137	PSG4	9.524777969	3.520257602	15.52929834	2.14241572	0.758896832	2.82306584	0.004757	0.011503
ENSG00000059915	PSD	14.96119107	5.607361959	24.31502018	2.137970113	0.685961323	3.11675023	0.001829	0.00474
ENSG00000267260	AC020928.2	18.36805073	6.704947528	30.03115392	2.134716977	0.627130585	3.40394334	0.000664	0.001845
ENSG00000139874	SSTR1	81.73293351	30.51797994	132.9478871	2.134283056	0.28482071	7.49342651	6.71E-14	4.46E-13
ENSG00000198121	LPAR1	117.5289117	43.74831916	191.3095043	2.120005892	0.237191599	8.93794681	3.96E-19	3.48E-18
ENSG00000153132	CLGN	94.13476142	35.44392332	152.8255995	2.1196543	0.286350335	7.40231122	1.34E-13	8.75E-13
ENSG00000164542	KIAA0895	58.37724947	21.79344754	94.96105139	2.11712225	0.345209454	6.13286289	8.63E-10	4.35E-09
ENSG00000169783	LINGO1	13.78578051	5.203733546	22.36782747	2.11669279	0.656256598	3.2254042	0.001258	0.003357
ENSG00000143110	C1orf162	15.37627143	5.791788788	24.96075407	2.111246657	0.666450848	3.16789552	0.001535	0.004031
ENSG00000251493	FOXD1	19.34859244	7.281772021	31.41541286	2.105329245	0.5486003	3.83763779	0.000124	0.000379
ENSG00000150782	IL18	26.06001746	9.945700869	42.17433406	2.104902595	0.533617198	3.94459287	7.99E-05	0.00025
ENSG00000138347	MYPN	21.70919793	8.167914065	35.2504818	2.100170188	0.532737772	3.9422213	8.07E-05	0.000253
ENSG00000105649	RAB3A	90.86829554	34.30240663	147.4341844	2.099767345	0.253507527	8.28285997	1.20E-16	9.42E-16
ENSG00000157551	KCNJ15	61.5517405	23.34165906	99.76182193	2.097848336	0.304440945	6.89082191	5.55E-12	3.27E-11
ENSG00000157214	STEAP2	229.370116	86.82613468	371.9140972	2.093455352	0.164218716	12.7479705	3.20E-37	5.49E-36
ENSG00000248712	CCDC153	35.11905115	13.32248521	56.91561709	2.091639482	0.405659888	5.15614076	2.52E-07	1.04E-06
ENSG00000184292	TACSTD2	3370.443641	1281.227764	5459.659517	2.091635704	0.048390089	43.2244645	0	0

ENSG00000257913	DDN-AS1	32.75251925	12.46746428	53.03757423	2.091171842	0.428340095	4.88203618	1.05E-06	4.06E-06
ENSG00000143375	CGN	10.69310666	4.010438818	17.3757745	2.091099387	0.790563815	2.64507349	0.008167	0.018952
ENSG00000133256	PDE6B	38.80107327	14.81386674	62.7882798	2.089918707	0.407645015	5.12681041	2.95E-07	1.21E-06
ENSG00000277142	LINC00235	10.93253746	4.157475247	17.70759966	2.089580939	0.725173213	2.88149218	0.003958	0.009712
ENSG00000171130	ATP6V0E2	534.2454387	203.3008407	865.1900368	2.088392424	0.104841048	19.9196064	2.75E-88	1.41E-86
ENSG00000126803	HSPA2	613.0499122	233.9776291	992.1221954	2.086220954	0.100687383	20.7197853	2.30E-95	1.33E-93
ENSG00000115318	LOXL3	57.28991627	21.97300338	92.60682916	2.083648512	0.325356736	6.40419663	1.51E-10	8.07E-10
ENSG00000253161	LINC01605	33.06659922	12.55627242	53.57692602	2.081927724	0.418623227	4.97327332	6.58E-07	2.60E-06
ENSG00000169760	NLGN1	1533.432216	585.8842251	2480.980207	2.080042435	0.069598495	29.8863135	2.96E-196	5.18E-194
ENSG00000177875	CCDC184	32.42584269	12.46475816	52.38692722	2.071850188	0.411421964	5.03582786	4.76E-07	1.91E-06
ENSG00000104213	PDGFRL	1516.621947	584.8961507	2448.347744	2.066310154	0.066388544	31.1244985	1.12E-212	2.32E-210
ENSG00000187037	GPR141	18.16312398	7.012100212	29.31414774	2.065356199	0.579580688	3.56353523	0.000366	0.001055
ENSG00000197558	SSPO	10.6202482	4.100645261	17.13985113	2.053107519	0.808163361	2.54046102	0.011071	0.024976
ENSG00000110900	TSPAN11	338.1370853	131.2415752	545.0325954	2.052371844	0.142397192	14.4130078	4.29E-47	9.55E-46
ENSG00000259803	SLC22A31	23.9143855	9.33549992	38.49327108	2.051604264	0.511369542	4.01197978	6.02E-05	0.000191
ENSG00000157168	NRG1	4040.855974	1574.062328	6507.64962	2.048325533	0.052055331	39.3490059	0	0
ENSG00000163827	LRRC2	45.58908585	17.93685569	73.24131601	2.04225336	0.378077413	5.40168042	6.60E-08	2.86E-07
ENSG00000167733	HSD11B1L	135.4015596	52.77039711	218.0327221	2.040708081	0.215914218	9.45147616	3.34E-21	3.23E-20
ENSG00000137033	IL33	362.4703771	142.5437995	582.3969547	2.031282178	0.154860401	13.1168599	2.64E-39	4.85E-38
ENSG00000002587	HS3ST1	45.01945939	17.7848578	72.25406099	2.030290551	0.378436508	5.36494367	8.10E-08	3.48E-07
ENSG00000013297	CLDN11	9369.660862	3689.295039	15050.02668	2.028474304	0.038290491	52.9759284	0	0
ENSG00000092096	SLC22A17	146.7932129	57.8731902	235.7132357	2.027267275	0.21202026	9.56166772	1.16E-21	1.14E-20
ENSG00000144278	GALNT13	14.39259271	5.673167352	23.11201807	2.021198645	0.645704726	3.13022124	0.001747	0.004541
ENSG00000253716	MINCR	9.680309855	3.833679573	15.52694014	2.018069241	0.754428231	2.67496517	0.007474	0.017433
ENSG00000108830	RND2	39.58284537	15.62870067	63.53699007	2.009769758	0.40590946	4.95127597	7.37E-07	2.90E-06
ENSG00000022267	FHL1	3312.966903	1320.966637	5304.967169	2.00500168	0.050810677	39.4602431	0	0
ENSG00000167874	TMEM88	352.1724044	141.0636932	563.2811155	1.996654829	0.134969654	14.7933611	1.62E-49	3.84E-48
ENSG00000106688	SLC1A1	677.4511796	271.6933534	1083.209006	1.995274643	0.100454174	19.862536	8.59E-88	4.37E-86
ENSG00000184986	TMEM121	220.2673009	88.47050614	352.0640956	1.994613647	0.179307546	11.1239805	9.59E-29	1.25E-27
ENSG00000276107	AC037198.1	726.0159186	291.5605794	1160.471258	1.991059869	0.101534747	19.6096404	1.28E-85	6.26E-84
ENSG00000100321	SYNGR1	367.4982416	148.0189634	586.9775198	1.987131483	0.123971269	16.0289678	8.02E-58	2.30E-56
ENSG00000186854	TRABD2A	17.92972832	7.175779632	28.68367701	1.985593926	0.633402561	3.13480565	0.00172	0.004479
ENSG00000254810	AP001189.3	102.9146383	41.59053842	164.2387382	1.983524546	0.230637516	8.60018172	7.96E-18	6.59E-17
ENSG00000174059	CD34	2496.664541	1009.028353	3984.300729	1.981492116	0.056359906	35.1578324	8.82E-271	2.37E-268
ENSG00000178175	ZNF366	134.6041358	54.31094104	214.8973305	1.980515668	0.231037455	8.57227097	1.01E-17	8.37E-17
ENSG00000225383	SFTA1P	21.85818292	8.802425589	34.91394024	1.979713112	0.531347981	3.72583163	0.000195	0.000581
ENSG00000154917	RAB6B	340.699659	138.0593257	543.3399923	1.976147589	0.129501126	15.2596943	1.42E-52	3.69E-51
ENSG00000197565	COL4A6	411.1897539	166.6132429	655.7662649	1.97571186	0.11877	16.6347719	3.90E-62	1.24E-60
ENSG00000259577	CERNA1	10.02435699	4.046521395	16.00219259	1.96941865	0.757470869	2.5999926	0.009323	0.021382
ENSG00000275874	PICSAR	17.08768019	6.929561353	27.24579903	1.965723615	0.586271235	3.35292523	0.0008	0.002194
ENSG00000142173	COL6A2	314.428127	128.2255262	500.6307277	1.964693327	0.137867178	14.2506241	4.44E-46	9.65E-45
ENSG00000225969	ABHD11-AS1	14.71600732	5.976215618	23.45579903	1.962443428	0.629359022	3.11816207	0.00182	0.00472
ENSG00000143036	SLC44A3	14.81642153	6.053793013	23.57905005	1.962058396	0.619859177	3.16532927	0.001549	0.004066
ENSG00000164078	MST1R	11.75658986	4.823107885	18.69007183	1.961328141	0.766688808	2.55818022	0.010522	0.023878
ENSG00000198542	ITGBL1	125.2364872	51.40895824	199.0640161	1.950267044	0.238056573	8.192452	2.56E-16	1.97E-15
ENSG00000169231	THBS3	1510.09481	620.5913426	2399.598278	1.950119126	0.065940404	29.5724473	3.38E-192	5.65E-190
ENSG00000169432	SCN9A	52.27384398	21.45161058	83.09607738	1.947969024	0.343382176	5.67288917	1.40E-08	6.43E-08
ENSG00000089820	ARHGAP4	1096.337127	452.404676	1740.269578	1.942837822	0.08379888	23.1845322	6.52E-119	5.38E-117
ENSG00000166823	MESP1	11.59530617	4.776651603	18.41396073	1.942450247	0.695889294	2.79132078	0.005249	0.012609
ENSG00000114200	BCHE	68.72902454	28.36994118	109.0881079	1.942012264	0.28638162	6.78120427	1.19E-11	6.87E-11
ENSG00000164674	SYTL3	167.5547294	69.54796625	265.5614925	1.940945232	0.198357528	9.78508481	1.30E-22	1.34E-21
ENSG00000156463	SH3RF2	32.46852341	13.47222776	51.46481906	1.935899314	0.412886918	4.68869133	2.75E-06	1.01E-05
ENSG00000272275	AC092687.3	18.70312051	7.801766527	29.60447448	1.935340433	0.563548969	3.434201	0.000594	0.001662
ENSG00000249464	LINC01091	390.2992371	161.9945427	618.6039314	1.934765841	0.121596298	15.9113877	5.28E-57	1.49E-55
ENSG00000143590	EFNA3	45.68147315	19.01779827	72.34514802	1.932279246	0.360284003	5.36321133	8.18E-08	3.51E-07
ENSG00000170396	ZNF804A	237.4055674	98.73544748	376.0756873	1.932086926	0.161054075	11.9965106	3.71E-33	5.64E-32
ENSG00000157927	RADIL	36.94186343	15.36358406	58.5201428	1.9305275	0.423974426	4.55340554	5.28E-06	1.88E-05
ENSG00000160180	TFF3	10.86833372	4.574183485	17.16248396	1.924480701	0.711366188	2.70533058	0.006824	0.016034
ENSG00000166897	ELFN2	212.4150628	88.48530005	336.3448255	1.924332179	0.16216791	11.8662945	1.77E-32	2.65E-31
ENSG00000261578	AP003119.3	53.44426527	22.32156041	84.56697013	1.921908233	0.360638608	5.32918049	9.87E-08	4.21E-07
ENSG00000230630	DNM3OS	139.2871176	58.24389293	220.3303423	1.921790935	0.20771167	9.25220491	2.20E-20	2.04E-19
ENSG00000111981	ULBP1	130.5484642	54.78326191	206.3136664	1.921639815	0.227957328	8.4298225	3.46E-17	2.78E-16
ENSG00000169093	ASMTL	2828.086966	1181.951529	4474.222403	1.920361559	0.05379844	35.6954877	4.64E-279	1.30E-276
ENSG00000198915	RASGEF1A	77.72670993	32.45750661	122.9959132	1.918921832	0.272903754	7.03149666	2.04E-12	1.24E-11
ENSG00000089723	OTUB2	354.550501	148.574093	560.526909	1.914782909	0.12943105	14.7938451	1.61E-49	3.82E-48
ENSG00000243243	AC073130.2	47.79203481	20.00582828	75.57824133	1.913254758	0.34750195	5.50573819	3.68E-08	1.62E-07
ENSG00000092421	SEMA6A	94.07877809	39.51110164	148.6464545	1.910356548	0.243084985	7.85880111	3.88E-15	2.78E-14
ENSG00000164949	GEM	28.39458117	11.86989238	44.91926996	1.910063656	0.481602405	3.96605922	7.31E-05	0.00023
ENSG00000139266	9-Mar	341.0175239	143.4240325	538.6110152	1.908079663	0.12729544	14.9893795	8.62E-51	2.12E-49
ENSG00000276900	AC023157.3	101.1205025	42.67418712	159.5668178	1.901880523	0.236032262	8.05771424	7.77E-16	5.80E-15
ENSG00000140057	AK7	13.6078497	5.740371043	21.47532837	1.900676821	0.655204901	2.9008892	0.003721	0.009166
ENSG00000152192	POU4F1	124.7485941	52.957711	196.5394773	1.899545319	0.220890764	8.59947823	8.01E-18	6.63E-17
ENSG00000133110	POSTN	770.9281483	326.1215636	1215.734733	1.898071475	0.092506596	20.5182286	1.48E-93	8.22E-92
ENSG00000250303	AP002884.1	85.77859862	36.46307444	135.0941228	1.896120132	0.260212075	7.28682607	3.17E-13	2.04E-12
ENSG00000250748	AC025419.1	36.08347518	15.28330054	56.88364982	1.895567029	0.395279357	4.79551233	1.62E-06	6.15E-06
ENSG00000095752	IL11	101.6592882	43.20045091	160.1181254	1.8866229	0.240481786	7.84517999	4.32E-15	3.09E-14
ENSG00000010438	PRSS3	528.1697948	225.1010988	831.2384908	1.884330578	0.104814899	17.9776977	2.91E-72	1.13E-70
ENSG00000173727	AP000769.1	68.24228016	29.15193991	107.3326204	1.880797691	0.318792269	5.89975941	3.64E-09	1.75E-08
ENSG00000262902	MTCO1P40	12.00873719	5.164944848	18.85252952	1.876994703	0.66801093	2.80982634	0.004957	0.011947
ENSG00000130005	GAMT	620.1115554	265.4961776	974.7269332	1.876344369	0.101246879	18.5323676	1.13E-76	4.72E-75
ENSG00000198719	DLL1	247.3145983	106.2814791	388.3477176	1.871772925	0.149775344	12.4972033	7.73E-36	1.28E-34
ENSG00000236452	AC123023.1	47.44779921	20.38600317	74.50959525	1.870445612	0.404220513	4.62729018	3.70E-06	1.34E-05
ENSG00000105427	CNFN	11.97121329	5.175318553	18.76710804	1.868368413	0.726990166	2.57000507	0.01017	0.023141
ENSG00000272734	ADIRF-AS1	12.56547783	5.406201664	19.72475399	1.862973183	0.659885269	2.82317741	0.004755	0.0115
ENSG00000112981	NME5	27.60173808	11.99803047	43.20544569	1.856747622	0.4666007	3.97930741	6.91E-05	0.000218

ENSG00000176771	NCKAP5	78.33014037	33.7589586	122.9013221	1.85640166	0.282462138	6.57221416	4.96E-11	2.74E-10
ENSG00000087237	CETP	260.7363214	113.1536692	408.3189736	1.849398681	0.152561745	12.1222963	8.05E-34	1.25E-32
ENSG00000247134	AC090204.1	27.15682408	11.81220534	42.50144282	1.845891664	0.460002304	4.01278787	6.00E-05	0.000191
ENSG00000135454	B4GALNT1	55.84394605	24.34912866	87.33876345	1.844246972	0.313650016	5.87995179	4.10E-09	1.96E-08
ENSG00000136160	EDNRB	107.9802922	47.08284211	168.8777423	1.841351177	0.232484228	7.92032729	2.37E-15	1.72E-14
ENSG00000145287	PLAC8	56.6693686	24.79425189	88.54448531	1.841162676	0.33578911	5.48309228	4.18E-08	1.84E-07
ENSG00000125746	EML2	891.2838764	389.7713424	1392.79641	1.838344183	0.088552786	20.7598685	9.98E-96	5.85E-94
ENSG00000286009	AC244213.1	10.21713558	4.488938506	15.94533266	1.835427207	0.778729078	2.35695219	0.018426	0.03973
ENSG00000001561	ENPP4	138.1200999	60.60152615	215.6386736	1.831375663	0.203324531	9.00715549	2.11E-19	1.87E-18
ENSG00000260001	TGFBR3L	41.86728731	18.4942406	65.24033403	1.829494194	0.397288559	4.60495062	4.13E-06	1.49E-05
ENSG00000235944	ZNF815P	68.54967703	30.02856529	107.0707888	1.828531917	0.285554118	6.40345141	1.52E-10	8.11E-10
ENSG00000253250	C8orf88	127.7821699	56.26905025	199.2952895	1.821590027	0.226714293	8.03473837	9.38E-16	6.97E-15
ENSG00000250479	CHCHD10	955.4970679	422.2620875	1488.732048	1.81792052	0.323090444	5.62666138	1.84E-08	8.32E-08
ENSG00000163328	GPR155	344.8000931	152.8232635	536.7769228	1.815477402	0.140840986	12.8902633	5.11E-38	9.04E-37
ENSG00000253177	AC104211.2	16.13954904	7.046784491	25.23231359	1.815429285	0.62953914	2.88374331	0.00393	0.00965
ENSG00000087494	PTHLH	253.1140254	112.2829973	393.9450535	1.813808769	0.155425897	11.6699263	1.82E-31	2.59E-30
ENSG00000268049	AC012313.2	16.67841899	7.42880845	25.92802954	1.813589695	0.577608098	3.13982733	0.00169	0.004409
ENSG00000069424	KCNAB2	222.6982103	98.93660778	346.4598127	1.810780719	0.17253942	10.4948812	9.12E-26	1.06E-24
ENSG00000264235	AP005329.1	8.702394277	3.87246827	13.53232028	1.809707388	0.791187128	2.28733169	0.022176	0.046977
ENSG00000184347	SLIT3	117.8985213	52.22564125	183.5714014	1.808759223	0.225958598	8.00482583	1.20E-15	8.83E-15
ENSG00000184524	CEND1	131.1264721	58.41979513	203.833149	1.805742267	0.221206645	8.16314658	3.26E-16	2.49E-15
ENSG00000262691	AC040160.1	17.38613025	7.755310245	27.01695025	1.805226905	0.648520078	2.78360989	0.005376	0.012889
ENSG00000226686	LINC01535	13.63290029	6.082208006	21.18359257	1.799395065	0.69928792	2.57318197	0.010077	0.022952
ENSG00000233695	GAS6-AS1	48.28465989	21.47235798	75.09696179	1.798637497	0.363001791	4.95489979	7.24E-07	2.85E-06
ENSG00000090661	CERS4	164.4215995	73.55335313	255.289846	1.79593603	0.194605353	9.22860552	2.74E-20	2.53E-19
ENSG00000088367	EPB41L1	871.8444599	389.4836736	1354.205246	1.795102079	0.091751184	19.5648929	3.08E-85	1.50E-83
ENSG00000277778	PGM5P2	33.88034633	15.21523981	52.54545285	1.793559396	0.483581121	3.70891112	0.000208	0.000619
ENSG00000188921	HACD4	356.1018138	159.269589	552.9340386	1.791814308	0.136057019	13.1695838	1.31E-39	2.44E-38
ENSG00000187098	MITF	180.8211219	80.89412	280.7481238	1.791143828	0.185245656	9.66901934	4.08E-22	4.11E-21
ENSG00000161281	COX7A1	456.3977102	204.6366612	708.1587593	1.791034661	0.114870634	15.5917539	8.28E-55	2.27E-53
ENSG00000141668	CBLN2	305.6441162	136.9809988	474.3072336	1.789679129	0.140775462	12.7130048	5.01E-37	8.55E-36
ENSG00000196972	SMIM10L2B	14.23975292	6.431712553	22.0477933	1.782000536	0.607130438	2.93511974	0.003334	0.008274
ENSG00000230266	XXYLT1-AS2	155.2760681	70.12600809	240.4261282	1.77938891	0.19932518	8.92706536	4.37E-19	3.83E-18
ENSG00000004799	PDK4	50.53633217	22.90830783	78.16435651	1.77590032	0.338208736	5.25090021	1.51E-07	6.36E-07
ENSG00000145242	EPHA5	203.0491946	92.13780017	313.960589	1.772171088	0.173516071	10.2132965	1.73E-24	1.91E-23
ENSG00000150054	MPP7	11.54422747	5.296646109	17.79180883	1.770831633	0.725526789	2.44075292	0.014657	0.032279
ENSG00000102387	TAF7L	26.26985203	11.87530462	40.66439945	1.763370093	0.499559061	3.52985309	0.000416	0.001188
ENSG00000227051	C14orf132	203.0382606	92.2165504	313.8599708	1.763115349	0.174758093	10.088891	6.19E-24	6.69E-23
ENSG00000249602	AL589765.4	14.75690392	6.734760819	22.77904703	1.760415367	0.60939352	2.88879896	0.003867	0.009509
ENSG00000139182	CLSTN3	2554.508981	1168.434018	3940.583945	1.753080533	0.052708642	33.259831	1.47E-242	3.34E-240
ENSG00000100092	SH3BP1	67.75890245	31.07351577	104.4442891	1.749858547	0.293322926	5.96563853	2.44E-09	1.19E-08
ENSG00000105472	CLEC11A	465.2606757	213.6164333	716.9049181	1.748791935	0.11632656	15.0334707	4.43E-51	1.10E-49
ENSG00000260329	AC007541.1	16.20592191	7.467597148	24.94424668	1.748656722	0.588035381	2.97372706	0.002942	0.007388
ENSG00000064687	ABCA7	371.4528245	170.6188108	572.2868383	1.746780935	0.132538932	13.1793799	1.15E-39	2.15E-38
ENSG00000172927	MYEOV	434.2165328	199.4450154	668.9880502	1.743775156	0.113413329	15.3753987	2.39E-53	6.37E-52
ENSG00000158887	MPZ	103.3934593	47.45936335	159.3275552	1.741566268	0.242038758	7.19540242	6.23E-13	3.91E-12
ENSG00000103528	SYT17	63.0766123	29.00715882	97.14606578	1.741468333	0.298753356	5.82911722	5.57E-09	2.64E-08
ENSG00000115919	KYNU	258.2238351	118.8539437	397.5937264	1.741163138	0.160285458	10.862889	1.73E-27	2.15E-26
ENSG00000146678	IGFBP1	60.38817806	28.02819469	92.74816143	1.738173436	0.330600129	5.25763085	1.46E-07	6.14E-07
ENSG00000164591	MYOZ3	52.71344387	24.35409012	81.07279761	1.738111584	0.327556237	5.30629977	1.12E-07	4.75E-07
ENSG00000136244	IL6	4322.600562	1995.651479	6649.549645	1.736299847	0.046701902	37.1783538	1.53E-302	4.93E-300
ENSG00000136014	USP44	74.20290041	34.33988751	114.0659133	1.734743011	0.279815737	6.19959059	5.66E-10	2.90E-09
ENSG00000154065	ANKRD29	56.12493491	25.72761678	86.52225305	1.734721151	0.371623406	4.6679545	3.04E-06	1.11E-05
ENSG00000154188	ANGPT1	38.44540029	17.87506424	59.01573634	1.73266517	0.399443734	4.3376952	1.44E-05	4.91E-05
ENSG00000159733	ZFYVE28	311.3174329	144.0473133	478.5875525	1.73244466	0.132826486	13.0429157	6.97E-39	1.26E-37
ENSG00000204963	PCDHA7	18.25849343	8.529190614	27.98779624	1.729713515	0.566299628	3.05441401	0.002255	0.005776
ENSG00000176399	DMRTA1	30.25815012	14.01382672	46.50247352	1.729096315	0.441480868	3.91658267	8.98E-05	0.00028
ENSG00000169429	CXCL8	76413.6552	35440.72121	117386.5892	1.727798831	0.029734335	58.1078682	0	0
ENSG00000203930	LINC00632	263.9527074	122.2901737	405.615241	1.724197909	0.161007385	10.7088126	9.25E-27	1.12E-25
ENSG00000101115	SALL4	26.65985108	12.36147189	40.95823026	1.723954192	0.459646939	3.75060518	0.000176	0.000529
ENSG00000115756	HPCAL1	2524.467924	1172.692298	3876.243549	1.723676545	0.054776781	31.4672846	2.44E-217	5.27E-215
ENSG00000174669	SLC29A2	82.96603329	38.54512686	127.3869397	1.722309	0.261704198	6.58112867	4.67E-11	2.59E-10
ENSG00000143850	PLEKHA6	186.82004	86.78012918	286.8599508	1.720704935	0.189141322	9.09745644	9.25E-20	8.38E-19
ENSG00000006016	CRLF1	261.1058979	121.7361266	400.4756691	1.718168382	0.147313719	11.6633291	1.96E-31	2.79E-30
ENSG00000139187	KLRG1	138.1935985	64.29985082	212.0873461	1.716490389	0.213963426	8.02235419	1.04E-15	7.69E-15
ENSG00000186205	1-Mar	38.3754091	17.94853722	58.80228099	1.715999509	0.390711391	4.39198741	1.12E-05	3.87E-05
ENSG00000084764	MAPRE3	362.1487258	169.0471902	555.2502613	1.715853263	0.132669455	12.9332955	2.92E-38	5.19E-37
ENSG00000152137	HSPB8	770.2178219	359.431204	1181.00444	1.714065488	0.093780396	18.2774392	1.25E-74	5.06E-73
ENSG00000132622	HSPA12B	597.9587192	279.6574916	916.2599469	1.712771453	0.096967467	17.6633618	8.03E-70	3.01E-68
ENSG00000089163	SIRT4	37.05201392	17.21926405	56.88476379	1.711699507	0.420174118	4.07378616	4.63E-05	0.000149
ENSG00000164509	IL31RA	189.3781085	88.79340026	289.9628168	1.710309046	0.178346338	9.58981869	8.82E-22	8.74E-21
ENSG00000283537	AC073264.3	13.74613454	6.460127547	21.03214154	1.709716072	0.63369133	2.69802661	0.006975	0.016371
ENSG00000123685	BATF3	31.57296963	14.82424044	48.32169882	1.709563301	0.424041794	4.03159152	5.54E-05	0.000177
ENSG00000136010	ALDH1L2	325.2629618	152.4086296	498.1172939	1.706792515	0.130524775	13.0763873	4.49E-39	8.17E-38
ENSG00000162817	C1orf115	1519.683787	713.3007344	2326.06684	1.703814987	0.074126179	22.9853339	6.53E-117	5.25E-115
ENSG00000124126	PREX1	13220.33046	6218.169001	20222.49193	1.701165503	0.034888898	48.7595082	0	0
ENSG00000154027	AK5	139.8790203	66.10979673	213.6482438	1.69920375	0.246568328	6.89141124	5.52E-12	3.26E-11
ENSG00000146411	SLC2A12	31.05043325	14.62037403	47.48049247	1.698593897	0.438607916	3.87269321	0.000108	0.000331
ENSG00000175344	CHRNA7	18.96575884	8.95758038	28.9739373	1.696838725	0.56897856	2.98225424	0.002861	0.007198
ENSG00000175906	ARL4D	220.865806	104.2335697	337.4980423	1.694241547	0.171345945	9.8878415	4.70E-23	4.92E-22
ENSG00000113739	STC2	11601.24056	5490.553644	17711.92748	1.689687799	0.032323456	52.274355	0	0
ENSG00000164035	EMCN	1964.892835	930.2700493	2999.51562	1.689531731	0.064243502	26.2988736	1.98E-152	2.37E-150
ENSG00000164694	FNDC1	23.36426662	11.01216533	35.71636791	1.686737281	0.495090642	3.4069262	0.000657	0.001826
ENSG00000142408	CACNG8	471.531342	223.9477658	719.1149183	1.685823415	0.13411948	12.5695642	3.10E-36	5.19E-35
ENSG00000110675	ELMOD1	97.10661068	46.15930966	148.0539117	1.684476521	0.247554856	6.8044576	1.01E-11	5.87E-11

ENSG00000259345	AC013652.1	158.4739957	74.91822018	242.0297712	1.682955844	0.220151898	7.64452116	2.10E-14	1.44E-13
ENSG00000196391	ZNF774	91.60247533	43.32394333	139.8810073	1.682140975	0.264975385	6.34829147	2.18E-10	1.15E-09
ENSG00000131398	KCNC3	147.0167237	69.61399696	224.4194504	1.681316534	0.222753444	7.54788122	4.42E-14	2.97E-13
ENSG00000169744	LDB2	2430.011897	1155.105128	3704.918666	1.680600657	0.054010877	31.1159668	1.46E-212	3.00E-210
ENSG00000075213	SEMA3A	249.3573115	118.7994136	379.9152095	1.677368991	0.147120799	11.4013043	4.12E-30	5.63E-29
ENSG00000187243	MAGED4B	11.24926537	5.331330389	17.16720036	1.67619179	0.682640612	2.45545278	0.014071	0.031097
ENSG00000154734	ADAMTS1	1014.676031	484.5675226	1544.784539	1.671102145	0.090214855	18.5235806	1.33E-76	5.53E-75
ENSG00000154277	UCHL1	10500.18083	5027.792606	15972.56905	1.66777209	0.033565087	49.6877029	0	0
ENSG00000167608	TMC4	43.76239928	20.99525659	66.52954197	1.662693875	0.354235325	4.69375514	2.68E-06	9.88E-06
ENSG00000272944	AC079834.2	20.34190046	9.709315043	30.97448587	1.661669044	0.513908769	3.23339306	0.001223	0.003274
ENSG00000149201	CCDC81	53.98138394	25.99652202	81.96624586	1.659486426	0.333242255	4.97981994	6.36E-07	2.52E-06
ENSG00000277702	AC239859.6	13.8945244	6.71671953	21.07232927	1.654292242	0.642488239	2.57482105	0.010029	0.022864
ENSG00000262587	AC133552.2	18.18814083	8.809236128	27.56704552	1.653721055	0.607227945	2.72339419	0.006461	0.015252
ENSG00000186088	GSAP	109.472579	52.92649942	166.0186585	1.648912049	0.225102573	7.3251586	2.39E-13	1.54E-12
ENSG00000142910	TINAGL1	12083.87606	5845.535287	18322.21683	1.648409497	0.036243581	45.4814196	0	0
ENSG00000105971	CAV2	5062.208691	2449.410647	7675.006735	1.64733066	0.045143324	36.4911248	1.53E-291	4.56E-289
ENSG00000173890	GPR160	48.35929948	23.36100817	73.3575908	1.645517079	0.357776098	4.59929293	4.24E-06	1.53E-05
ENSG00000181019	NQO1	3338.885973	1618.107203	5059.664744	1.645055467	0.058591032	28.0769156	1.88E-173	2.83E-171
ENSG00000227517	LINC01483	38.33271536	18.61913132	58.0462994	1.64453278	0.376089399	4.37271773	1.23E-05	4.21E-05
ENSG00000231652	AL590428.1	79.19588944	38.48202759	119.9097513	1.6420341	0.262114639	6.26456465	3.74E-10	1.94E-09
ENSG00000140678	ITGAX	22.3437913	10.83315074	33.85443187	1.641388211	0.508216042	3.22970563	0.001239	0.00331
ENSG00000103742	IGDCC4	16.5460469	8.122856081	24.96923771	1.63290579	0.570919517	2.86013307	0.004235	0.010335
ENSG00000163347	CLDN1	72.73087659	35.51599799	109.9457552	1.632045066	0.292722813	5.57539418	2.47E-08	1.10E-07
ENSG00000164530	PI16	16.17671941	7.859453559	24.49398526	1.631180067	0.615233421	2.65131901	0.008018	0.018636
ENSG00000132965	ALOX5AP	17.60136437	8.602663592	26.60006514	1.630104238	0.553530971	2.94491966	0.00323	0.008042
ENSG00000183621	ZNF438	731.4710575	357.1792474	1105.762868	1.629620374	0.088100322	18.497326	2.17E-76	8.96E-75
ENSG00000274528	AC090970.2	15.2515842	7.51675955	22.98640885	1.625650187	0.640891117	2.53654661	0.011195	0.025231
ENSG00000143126	CELSR2	76.26220756	37.30948027	115.2149349	1.625458638	0.298424769	5.44679533	5.13E-08	2.24E-07
ENSG00000259065	AC005520.2	27.84991429	13.60253072	42.09729785	1.623220625	0.437312206	3.71181184	0.000206	0.000613
ENSG00000105784	RUNDC3B	28.04658104	13.75994086	42.33322122	1.622774273	0.42981541	3.77551441	0.00016	0.000481
ENSG00000272767	JMJD1C-AS1	25.99476338	12.7651003	39.22442645	1.619825767	0.493910741	3.27959211	0.00104	0.002812
ENSG00000110844	PRPF40B	228.4556215	112.1461996	344.7710433	1.61790646	0.160858263	10.057963	8.47E-24	9.12E-23
ENSG00000141540	TTYH2	69.75583072	34.09669114	105.4149703	1.617767856	0.308578435	5.24264716	1.58E-07	6.64E-07
ENSG00000213366	GSTM2	63.89431349	31.35983057	96.42879642	1.616269337	0.31738713	5.09242241	3.54E-07	1.44E-06
ENSG00000058404	CAMK2B	11.02212041	5.413869248	16.63037158	1.615739085	0.694988645	2.32484242	0.02008	0.042943
ENSG00000107551	RASSF4	394.5361412	194.2148065	594.857476	1.615493986	0.121070148	13.3434543	1.29E-40	2.47E-39
ENSG00000095303	PTGS1	6266.74389	3086.204359	9447.283421	1.613958276	0.038336158	42.1001567	0	0
ENSG00000253368	TRNP1	658.5674094	324.6614381	992.4733806	1.613128196	0.099317068	16.2422053	2.54E-59	7.61E-58
ENSG00000245105	A2M-AS1	18.76509665	9.27100235	28.25919095	1.61111799	0.574115406	2.80626155	0.005012	0.012067
ENSG00000248015	AC005329.1	14.12905756	6.947602641	21.31051248	1.610679884	0.613132112	2.62697036	0.008615	0.019893
ENSG00000106034	CPED1	45.1736237	22.56020158	67.78704582	1.599775115	0.376681861	4.24701925	2.17E-05	7.26E-05
ENSG00000101695	RNF125	14.85974155	7.400393457	22.31908964	1.598228774	0.6072428	2.63194356	0.00849	0.019628
ENSG00000187688	TRPV2	1288.93603	640.1833038	1937.688756	1.597402545	0.07167684	22.2861742	5.03E-110	3.64E-108
ENSG00000143153	ATP1B1	3379.862228	1679.571055	5080.1534	1.596518708	0.051269244	31.1398915	6.95E-213	1.45E-210
ENSG00000095539	SEMA4G	421.3426922	209.4399692	633.2454151	1.596449435	0.121473108	13.1424103	1.88E-39	3.47E-38
ENSG00000124243	BCAS4	134.5362278	66.63804444	202.4344113	1.595498079	0.222032472	7.18587722	6.68E-13	4.19E-12
ENSG00000231999	LRRC8C-DT	32.15884969	15.99809558	48.3196038	1.595068339	0.425885052	3.74530247	0.00018	0.00054
ENSG00000177675	CD163L1	290.4947631	144.8366638	436.1528623	1.59458518	0.15529741	10.2679445	9.83E-25	1.10E-23
ENSG00000100312	ACR	13.90099063	6.875437487	20.92654378	1.594126838	0.644534381	2.47329993	0.013387	0.029738
ENSG00000120875	DUSP4	1924.110617	956.9878593	2891.233375	1.593290833	0.065557281	24.3037968	1.79E-130	1.68E-128
ENSG00000167617	CDC42EP5	2126.460911	1062.706767	3190.215056	1.585002013	0.058428131	27.1273785	4.68E-162	6.19E-160
ENSG00000041353	RAB27B	11.90007318	5.904050463	17.8960959	1.583438791	0.677980318	2.33552324	0.019516	0.041835
ENSG00000113083	LOX	6373.356057	3191.458477	9555.253637	1.581704786	0.045691963	34.6166958	1.42E-262	3.46E-260
ENSG00000163219	ARHGAP25	63.31896357	31.59653219	95.04139494	1.580118226	0.367365731	4.30121291	1.70E-05	5.75E-05
ENSG00000181634	TNFSF15	17597.6109	8821.673955	26373.54784	1.580025886	0.07841126	20.1504973	2.66E-90	1.41E-88
ENSG00000104381	GDAP1	132.5008824	66.41676846	198.5849964	1.579790816	0.199375904	7.92367975	2.31E-15	1.68E-14
ENSG00000151651	ADAM8	28.41754315	14.20095967	42.63412663	1.579171833	0.430953786	3.66436468	0.000248	0.00073
ENSG00000120820	GLT8D2	90.94895981	45.52470766	136.373212	1.57711248	0.253014797	6.23328464	4.57E-10	2.35E-09
ENSG00000127561	SYNGR3	109.8099969	55.16920934	164.4507845	1.576963295	0.252043171	6.256719	3.93E-10	2.04E-09
ENSG00000175356	SCUBE2	67.20649768	33.68052416	100.7324712	1.57685955	0.27899235	5.65198131	1.59E-08	7.22E-08
ENSG00000185100	ADSSL1	58.74950459	29.59390624	87.90510294	1.573372331	0.332658303	4.72969506	2.25E-06	8.37E-06
ENSG00000213889	PPM1N	14.27393415	7.142403175	21.40546512	1.571936923	0.667040122	2.35658527	0.018444	0.03976
ENSG00000115138	POMC	15.59222022	7.808035813	23.37640463	1.569549755	0.606230749	2.58903026	0.009625	0.022009
ENSG00000183023	SLC8A1	28.10543878	14.13375598	42.07712158	1.563733361	0.442369759	3.53490113	0.000408	0.001168
ENSG00000123095	BHLHE41	61.99162068	31.20327748	92.77996387	1.562600398	0.339566063	4.60175668	4.19E-06	1.51E-05
ENSG00000198865	CCDC152	114.8372167	58.20379641	171.470637	1.561644734	0.230095096	6.78695357	1.15E-11	6.61E-11
ENSG00000073737	DHRS9	202.622668	102.9740633	302.2712727	1.55548531	0.166014171	9.36959356	7.28E-21	6.89E-20
ENSG00000140450	ARRDC4	331.7673911	168.2425045	495.2922776	1.554946631	0.138660487	11.2140572	3.48E-29	4.61E-28
ENSG00000065923	SLC9A7	840.5107156	428.2396584	1252.781773	1.548639984	0.081833604	18.9242549	7.20E-80	3.20E-78
ENSG00000123358	NR4A1	301.2598649	153.3285989	449.191131	1.548465896	0.140557317	11.0166153	3.18E-28	4.05E-27
ENSG00000250130	AC090519.1	15.08162439	7.708853963	22.45439483	1.548169305	0.649934674	2.38203833	0.017217	0.037323
ENSG00000159915	ZNF233	12.03579312	6.164746865	17.90683938	1.547679525	0.675617028	2.29076453	0.021977	0.046596
ENSG00000102554	KLF5	210.093291	107.0637936	313.1227884	1.546366328	0.170550178	9.06692885	1.22E-19	1.10E-18
ENSG00000105974	CAV1	22852.28679	11657.09155	34047.48203	1.546177369	0.032082606	48.1936341	0	0
ENSG00000275450	AL845472.1	14.46264197	7.382352168	21.54293178	1.545643691	0.624847291	2.4736343	0.013375	0.02972
ENSG00000108556	CHRNE	174.1951824	88.90060999	259.4897548	1.541786	0.17764525	8.67901621	3.99E-18	3.35E-17
ENSG00000138400	MDH1B	17.22362559	8.774010596	25.67324058	1.540565047	0.590279128	2.60989246	0.009057	0.020816
ENSG00000173442	EHBP1L1	3066.56553	1569.170482	4563.960577	1.53977812	0.049526248	31.0901429	3.27E-212	6.63E-210
ENSG00000132639	SNAP25	22.56229079	11.56057482	33.56400677	1.539618984	0.482024244	3.1940696	0.001403	0.003709
ENSG00000228672	PROB1	213.121027	109.4356796	316.8063744	1.538615403	0.183768044	8.37259496	5.64E-17	4.47E-16
ENSG00000280734	LINC01232	20.21213965	10.29240882	30.13187047	1.537715307	0.52956598	2.90372751	0.003687	0.009089
ENSG00000143603	KCNN3	276.3182812	142.053212	410.5833505	1.533216021	0.148589034	10.3185005	5.81E-25	6.57E-24
ENSG00000285106	AC016831.7	20.93834543	10.72445223	31.15223863	1.533204763	0.514616159	2.97931718	0.002889	0.007259
ENSG00000260641	AC114811.2	17.46733473	9.008998125	25.92567134	1.532475437	0.551074716	2.78088505	0.005421	0.012981
ENSG00000154102	C16orf74	276.7721557	142.119784	411.4245275	1.531567553	0.142721643	10.7311514	7.27E-27	8.83E-26

ENSG00000172819	RARG	909.6871963	468.3527522	1351.02164	1.527623958	0.078938489	19.3520801	1.96E-83	9.25E-82
ENSG00000215483	LINC00598	35.66492468	18.33272604	52.99712332	1.526099858	0.412958158	3.69553144	0.000219	0.00065
ENSG00000170049	KCNAB3	48.22577278	24.83660375	71.6149418	1.525017589	0.350355085	4.3527771	1.34E-05	4.59E-05
ENSG00000238164	TNFRSF14-AS1	38.53390729	19.8831929	57.18462169	1.524860342	0.390691711	3.90297592	9.50E-05	0.000294
ENSG00000250241	AC105383.1	39.73016585	20.40309693	59.05723476	1.524753997	0.392019946	3.88948065	0.0001	0.00031
ENSG00000215580	BCORP1	12.17349347	6.245887426	18.10109952	1.524384458	0.673726915	2.26261475	0.023659	0.04971
ENSG00000262831	AC145207.3	43.02303105	22.23996907	63.80609303	1.523884734	0.374129301	4.07314993	4.64E-05	0.000149
ENSG00000171860	C3AR1	12.90277515	6.662595664	19.14295463	1.520880424	0.636714354	2.38863851	0.016911	0.036692
ENSG00000197822	OCLN	321.5698652	166.3260711	476.8136593	1.520506627	0.12790488	11.8877922	1.37E-32	2.06E-31
ENSG00000184185	KCNJ12	46.60945487	24.01085486	69.20805487	1.519961266	0.349836908	4.34477104	1.39E-05	4.76E-05
ENSG00000238005	AL391832.2	27.65809179	14.26545724	41.05072634	1.519900588	0.432911374	3.51088162	0.000447	0.00127
ENSG00000164093	PITX2	14.90869965	7.726895252	22.09050404	1.519354543	0.608815533	2.49559096	0.012575	0.02811
ENSG00000167371	PRRT2	37.30237504	19.37000894	55.23474114	1.518326611	0.409193357	3.71053583	0.000207	0.000616
ENSG00000128591	FLNC	13082.05491	6769.944535	19394.16528	1.51826954	0.032898105	46.1506682	0	0
ENSG00000258940	AL132639.2	24.10367894	12.53962943	35.66772845	1.513681922	0.460341806	3.28816957	0.001008	0.002734
ENSG00000267279	AC090409.1	29.0731734	15.12458259	43.02176422	1.513150678	0.426974329	3.54389146	0.000394	0.001132
ENSG00000114270	COL7A1	62.57472512	32.45028981	92.69916042	1.513014072	0.337921474	4.47741321	7.56E-06	2.65E-05
ENSG00000144063	MALL	258.877865	134.3334431	383.422287	1.512667042	0.149407729	10.124423	4.31E-24	4.69E-23
ENSG00000251003	ZFPM2-AS1	14.85725383	7.729601372	21.98490628	1.512591511	0.645872336	2.34193574	0.019184	0.041174
ENSG00000165424	ZCCHC24	563.9457312	292.6367415	835.2547208	1.512461861	0.102408517	14.7689069	2.32E-49	5.49E-48
ENSG00000007516	BAIAP3	15.45598347	8.029943517	22.88202342	1.51221497	0.589753111	2.5641492	0.010343	0.023507
ENSG00000160886	LY6K	65.61463243	34.16583438	97.06343048	1.509260186	0.325146753	4.64178151	3.45E-06	1.26E-05
ENSG00000178773	CPNE7	141.5281928	73.52308906	209.5332965	1.508842663	0.200487434	7.5258715	5.24E-14	3.50E-13
ENSG00000131188	PRR7	211.5193123	110.3460863	312.6925384	1.505129612	0.199126197	7.55867201	4.07E-14	2.74E-13
ENSG00000171714	ANO5	15.36921797	8.022275933	22.71616002	1.504617573	0.577008732	2.60761664	0.009118	0.02094
ENSG00000261377	PDCD6IPP2	219.6931729	114.8377769	324.5485689	1.502697329	0.165260681	9.09289082	9.64E-20	8.74E-19
ENSG00000111674	ENO2	669.3839345	349.7267601	989.0411088	1.501208305	0.096053501	15.6288766	4.63E-55	1.27E-53
ENSG00000179909	ZNF154	54.6049952	28.64326663	80.56672377	1.497770953	0.321540676	4.65810725	3.19E-06	1.17E-05
ENSG00000240694	PNMA2	1234.517811	646.1896944	1822.845927	1.495685942	0.071562087	20.9005354	5.29E-97	3.23E-95
ENSG00000107738	VSIR	222.2044269	116.7417622	327.6670915	1.493417479	0.164897455	9.05664359	1.35E-19	1.21E-18
ENSG00000171757	LRRC34	77.88942011	40.7115227	115.0673175	1.492640808	0.293442408	5.08665676	3.64E-07	1.48E-06
ENSG00000183971	NPW	44.17252063	23.25285091	65.09219034	1.492460808	0.366128721	4.07632814	4.58E-05	0.000147
ENSG00000159761	C16orf86	21.82488594	11.42616744	32.22360444	1.491152083	0.520534566	2.86465526	0.004175	0.010196
ENSG00000248905	FMN1	13.47434875	7.079303903	19.86939361	1.489633086	0.645873491	2.30638524	0.021089	0.044874
ENSG00000115457	IGFBP2	4875.798506	2567.598237	7183.998775	1.484416554	0.042719072	34.7483336	1.47E-264	3.63E-262
ENSG00000228049	POLR2J2	12.76503032	6.745134523	18.78492611	1.483607829	0.649170823	2.28538895	0.02229	0.047192
ENSG00000144959	NCEH1	2586.707427	1364.980614	3808.43424	1.4806206	0.062429003	23.7168707	2.42E-124	2.09E-122
ENSG00000083807	SLC27A5	79.80876991	42.12446979	117.49307	1.478481811	0.260858833	5.66774678	1.45E-08	6.61E-08
ENSG00000177989	ODF3B	71.03952322	37.394184	104.6848624	1.478033963	0.305799009	4.83335105	1.34E-06	5.13E-06
ENSG00000215475	SIAH3	17.70404665	9.2822331	26.1258602	1.476907695	0.585913311	2.52069319	0.011712	0.026318
ENSG00000272941	AC083862.2	149.6576598	78.95630742	220.3589703	1.476629288	0.189650543	7.78605355	6.91E-15	4.89E-14
ENSG00000049089	COL9A2	19.14887296	10.09760829	28.20013763	1.475819411	0.558821842	2.64094798	0.008267	0.019154
ENSG00000185008	ROBO2	18.61796089	9.838310183	27.3976116	1.475809774	0.534018871	2.76359105	0.005717	0.013626
ENSG00000078081	LAMP3	1817.37135	962.5546298	2672.188069	1.474319776	0.064589332	22.8260571	2.53E-115	2.01E-113
ENSG00000175746	C15orf54	2221.748418	1175.288752	3268.208083	1.474043276	0.066878542	22.0406014	1.18E-107	8.26E-106
ENSG00000275294	LINC02340	14.0230158	7.485638436	20.56039316	1.470523872	0.610953914	2.40693093	0.016087	0.035089
ENSG00000163053	SLC16A14	19.18252811	10.15443827	28.21061794	1.470519569	0.510154346	2.88249935	0.003945	0.009685
ENSG00000158321	AUTS2	48.96523429	25.94551055	71.98495803	1.469400065	0.38524877	3.814159	0.000137	0.000415
ENSG00000137880	GCHFR	28.51701188	15.20215998	41.83186378	1.469223558	0.429443194	3.42122911	0.000623	0.001738
ENSG00000204282	TNRC6C-AS1	121.6146901	64.45229949	178.7770808	1.467954727	0.215652413	6.80704058	9.96E-12	5.77E-11
ENSG00000121064	SCPEP1	3683.216194	1957.058499	5409.373889	1.467239615	0.049371717	29.7182212	4.47E-194	7.59E-192
ENSG00000196793	ZNF239	21.90622017	11.62096798	32.19147236	1.463503302	0.557132598	2.62684917	0.008618	0.019895
ENSG00000139725	RHOF	795.8437087	424.0551665	1167.632251	1.461814816	0.087037867	16.795159	2.65E-63	8.67E-62
ENSG00000182272	B4GALNT4	625.5417747	334.3790065	916.7045428	1.456602973	0.114222227	12.7523601	3.02E-37	5.19E-36
ENSG00000223658	C1GALT1C1L	25.15877577	13.3391282	36.97842335	1.455413576	0.499094831	2.91610629	0.003544	0.008762
ENSG00000183186	C2CD4C	27.56634578	14.74666305	40.38602851	1.455113093	0.443171016	3.28341214	0.001026	0.002778
ENSG00000159713	TPPP3	53.87320779	28.84929791	78.89711766	1.449076678	0.348895387	4.15332714	3.28E-05	0.000108
ENSG00000060656	PTPRU	458.6631471	245.9227085	671.4035856	1.447857201	0.110567661	13.094762	3.53E-39	6.47E-38
ENSG00000168062	BATF2	87.24808383	47.02299021	127.4731774	1.445768996	0.304762688	4.74391733	2.10E-06	7.82E-06
ENSG00000076554	TPD52	66.3498196	35.63863337	97.06100583	1.44352927	0.281788688	5.12273676	3.01E-07	1.23E-06
ENSG00000255471	AP001528.2	158.9760472	85.64515321	232.3069412	1.440131455	0.184079741	7.8234109	5.14E-15	3.66E-14
ENSG00000168405	CMAHP	39.63307134	21.35242873	57.91371395	1.439802767	0.404457692	3.55983529	0.000371	0.001069
ENSG00000119900	OGFRL1	7055.776204	3802.725912	10308.8265	1.438514875	0.038896493	36.9831514	2.14E-299	6.78E-297
ENSG00000282851	BISPR	127.4870171	68.68419523	186.2898389	1.437245354	0.209657699	6.85519949	7.12E-12	4.17E-11
ENSG00000180096	1-Sep	31.89043003	17.18457977	46.59628029	1.43646854	0.405028023	3.5465905	0.00039	0.001121
ENSG00000156140	ADAMTS3	107.180724	57.92009726	156.4413508	1.436128777	0.236692927	6.06747651	1.30E-09	6.45E-09
ENSG00000131149	GSE1	616.6580009	333.6956944	899.6203074	1.431919797	0.094849211	15.0968025	1.70E-51	4.29E-50
ENSG00000223802	CERS1	135.1738426	73.1826504	197.1650349	1.430532048	0.19554839	7.31548875	2.56E-13	1.65E-12
ENSG00000135124	P2RX4	5879.299874	3182.975608	8575.62414	1.429926916	0.037735803	37.8931096	0	0
ENSG00000197959	DNM3	52.6585505	28.47408449	76.8430165	1.428108448	0.317435302	4.49889612	6.83E-06	2.41E-05
ENSG00000115556	PLCD4	48.70803239	26.37574939	71.0403154	1.427521615	0.340753647	4.18930693	2.80E-05	9.25E-05
ENSG00000123892	RAB38	118.5868666	64.21320754	172.9605256	1.423116044	0.228988489	6.21479293	5.14E-10	2.64E-09
ENSG00000128536	CDHR3	20.81446882	11.36081283	30.26812481	1.422716435	0.521108556	2.73017286	0.00633	0.014966
ENSG00000128298	BAIAP2L2	18.33141111	9.972717565	26.69010466	1.422305278	0.545217785	2.60869201	0.009089	0.020881
ENSG00000204767	INSYN2B	1675.99063	911.4823216	2440.498938	1.421380878	0.065914614	21.5639717	3.92E-103	2.63E-101
ENSG00000162433	AK4	829.4716695	451.467748	1207.475591	1.41942298	0.092834061	15.2898942	8.93E-53	2.33E-51
ENSG00000184254	ALDH1A3	1397.861206	761.3111245	2034.411287	1.417711947	0.068125051	20.8104349	3.48E-96	2.07E-94
ENSG00000262227	AC004771.3	30.95254397	16.95509496	44.94999298	1.416419435	0.442171787	3.20332386	0.001359	0.003606
ENSG00000105464	GRIN2D	382.1169778	208.4843681	555.7495875	1.415988918	0.11964535	11.8348847	2.58E-32	3.82E-31
ENSG00000137070	IL11RA	174.8300316	95.33913308	254.3209302	1.414820288	0.184249295	7.67883692	1.61E-14	1.11E-13
ENSG00000205771	CATSPER2P1	25.88436803	14.14552798	37.62320808	1.414607198	0.451536888	3.13287183	0.001731	0.004504
ENSG00000268912	AC012313.5	31.02844883	16.95640278	45.10049487	1.412514594	0.399758984	3.53341551	0.00041	0.001174
ENSG00000023171	GRAMD1B	176.1885374	96.16659607	256.2104786	1.410584208	0.173028528	8.15232161	3.57E-16	2.72E-15
ENSG00000180190	TDRP	89.22587542	48.90072543	129.5510254	1.406746806	0.240378356	5.85221909	4.85E-09	2.31E-08
ENSG00000132429	POPDC3	183.7943451	100.7990078	266.7896823	1.404835406	0.176675765	7.95148901	1.84E-15	1.35E-14

ENSG00000183569	SERHL2	29.0502175	15.97464206	42.12579295	1.40296164	0.450990732	3.11084362	0.001866	0.004831
ENSG00000198835	GJC2	36.87282269	20.29223356	53.45341183	1.401520873	0.398555806	3.51649845	0.000437	0.001245
ENSG00000186654	PRR5	87.7779285	48.18994433	127.3659127	1.399085977	0.256106556	5.46290574	4.68E-08	2.05E-07
ENSG00000155465	SLC7A7	1177.815208	648.1593503	1707.471065	1.398068074	0.075667415	18.476488	3.19E-76	1.32E-74
ENSG00000184831	APOO	676.0325692	372.0291967	980.0359416	1.397248395	0.092441173	15.1150008	1.29E-51	3.27E-50
ENSG00000105426	PTPRS	1003.783297	552.2814592	1455.285135	1.396710031	0.088217927	15.8324967	1.86E-56	5.19E-55
ENSG00000261468	AC096921.2	841.2561565	463.266964	1219.245349	1.396624851	0.095341666	14.6486307	1.37E-48	3.18E-47
ENSG00000119042	SATB2	155.3130666	85.6749665	224.9511667	1.395619304	0.202707964	6.88487654	5.78E-12	3.41E-11
ENSG00000105967	TFEC	706.5062008	389.1076937	1023.904708	1.394710437	0.089490479	15.5850148	9.20E-55	2.52E-53
ENSG00000142459	EVI5L	1749.737313	964.2163647	2535.258262	1.393996865	0.068813257	20.2576788	3.04E-91	1.64E-89
ENSG00000258808	LINC02310	16.89659987	9.399997491	24.39320226	1.387316581	0.60129684	2.3072075	0.021043	0.044791
ENSG00000272273	IER3-AS1	22.38442404	12.40522205	32.36362602	1.381905735	0.532744273	2.59393823	0.009488	0.021726
ENSG00000130176	CNN1	19.00798102	10.55310522	27.46285681	1.381637649	0.512562186	2.69555126	0.007027	0.016479
ENSG00000151320	AKAP6	17.21168318	9.527594334	24.89577203	1.38112606	0.550271287	2.50990029	0.012077	0.027057
ENSG00000149809	TM7SF2	180.5706794	100.1477628	260.9935959	1.379818465	0.174674541	7.89936792	2.80E-15	2.02E-14
ENSG00000111424	VDR	65.65597262	36.47204985	94.8398954	1.379322497	0.278748778	4.94826384	7.49E-07	2.94E-06
ENSG00000158315	RHBDL2	48.28425305	26.79741909	69.77108701	1.378954533	0.325417987	4.23748714	2.26E-05	7.56E-05
ENSG00000205795	CYS1	17.0036474	9.510951343	24.49634346	1.378483984	0.578395073	2.38329137	0.017159	0.037209
ENSG00000230043	TMSB4XP6	30.01340948	16.75393467	43.27288429	1.374703975	0.424944543	3.23501971	0.001216	0.003256
ENSG00000179820	MYADM	9300.713109	5179.273682	13422.15254	1.37378553	0.044210673	31.0736173	5.47E-212	1.10E-209
ENSG00000226702	MIR217HG	462.1904713	257.6934636	666.6874789	1.371056181	0.109812361	12.4854449	8.96E-36	1.48E-34
ENSG00000163638	ADAMTS9	5070.156566	2829.886858	7310.426274	1.368790131	0.04763667	28.7339592	1.44E-181	2.21E-179
ENSG00000138606	SHF	96.51111514	53.97686213	139.0453682	1.36865542	0.237184476	5.77042581	7.91E-09	3.70E-08
ENSG00000117791	2-Mar	392.1051357	219.5975644	564.6127071	1.363759151	0.122368467	11.1446943	7.60E-29	9.95E-28
ENSG00000179403	VWA1	426.5828831	239.0505629	614.1152032	1.362488387	0.123519445	11.0305579	2.72E-28	3.47E-27
ENSG00000127220	ABHD8	315.9609737	176.8821982	455.0397492	1.362475532	0.139480968	9.76818234	1.54E-22	1.57E-21
ENSG00000091409	ITGA6	8403.759593	4705.983715	12101.53547	1.362467637	0.044769328	30.4330597	2.01E-203	3.75E-201
ENSG00000113719	ERGIC1	7969.545941	4468.408927	11470.68295	1.360165625	0.034023048	39.9777711	0	0
ENSG00000227199	ST7-AS1	17.62198905	9.89784629	25.34613181	1.358050696	0.537094154	2.52851513	0.011455	0.025782
ENSG00000198624	CCDC69	798.3716704	448.5242244	1148.219116	1.3557768	0.092578964	14.644545	1.46E-48	3.37E-47
ENSG00000182916	TCEAL7	473.2921565	266.1518428	680.4324702	1.354061766	0.109837547	12.3278588	6.41E-35	1.03E-33
ENSG00000204634	TBC1D8	1229.679936	692.4049325	1766.95494	1.35128567	0.074159055	18.2214522	3.49E-74	1.40E-72
ENSG00000152465	NMT2	3173.32644	1787.312569	4559.340311	1.350356708	0.05405649	24.9804736	9.97E-138	1.07E-135
ENSG00000174749	FAM241A	2147.32304	1209.954016	3084.692063	1.349331821	0.06191655	21.7927487	2.72E-105	1.88E-103
ENSG00000171357	LURAP1	55.78574763	31.39591315	80.17558212	1.348851732	0.304893823	4.42400478	9.69E-06	3.36E-05
ENSG00000232926	AC000078.1	22.35703026	12.53552501	32.1785355	1.348563786	0.497141106	2.71263786	0.006675	0.015707
ENSG00000257453	AC011611.3	16.60707743	9.407665075	23.80648978	1.347831029	0.559764505	2.40785369	0.016047	0.035008
ENSG00000128645	HOXD1	828.5006351	467.5161773	1189.485093	1.346822759	0.09539657	14.1181465	2.94E-45	6.30E-44
ENSG00000242028	HYPK	20.96253595	11.83024663	30.09482527	1.346376284	0.580536003	2.31919515	0.020384	0.043501
ENSG00000149582	TMEM25	595.9822505	337.074869	854.889632	1.344420655	0.099441801	13.5196733	1.20E-41	2.37E-40
ENSG00000263432	RN7SL689P	18.68098386	10.57114651	26.79082121	1.344361189	0.511020796	2.63073675	0.00852	0.019691
ENSG00000143369	ECM1	294.1102366	166.4564645	421.7640087	1.343586231	0.141580349	9.48992031	2.31E-21	2.24E-20
ENSG00000091490	SEL1L3	6605.560754	3736.370802	9474.750707	1.342660747	0.041621503	32.2588243	2.65E-228	5.93E-226
ENSG00000247828	TMEM161B-AS	78.44342061	44.41900373	112.4678375	1.340326795	0.284158114	4.7168345	2.40E-06	8.88E-06
ENSG00000257219	LNCOG	534.5095073	302.8620371	766.1569775	1.340004234	0.101982524	13.1395477	1.95E-39	3.60E-38
ENSG00000153233	PTPRR	55.14903667	31.33466295	78.96341038	1.339377526	0.341508274	3.92194752	8.78E-05	0.000274
ENSG00000172345	STARD5	38.23458266	21.76286768	54.70629765	1.339319317	0.379729429	3.52703587	0.00042	0.0012
ENSG00000204516	MICB	3795.870671	2151.560476	5440.180866	1.338061614	0.043878596	30.4946315	3.07E-204	5.79E-202
ENSG00000236453	AC003092.1	96.56961713	54.80346807	138.3357662	1.337334013	0.235776439	5.67204263	1.41E-08	6.45E-08
ENSG00000142347	MYO1F	18.22088313	10.2897027	26.15206356	1.33339272	0.555432903	2.40063689	0.016367	0.035621
ENSG00000136895	GARNL3	59.17621894	33.87455812	84.47787976	1.328371645	0.343682167	3.86511659	0.000111	0.000341
ENSG00000164659	KIAA1324L	3002.951082	1710.618546	4295.283617	1.328265074	0.051793909	25.6451984	4.78E-145	5.36E-143
ENSG00000106772	PRUNE2	516.7389556	294.2727691	739.2051422	1.327702695	0.102895777	12.903374	4.31E-38	7.64E-37
ENSG00000160013	PTGIR	88.93640417	50.66038047	127.2124279	1.326829528	0.265899263	4.98997069	6.04E-07	2.40E-06
ENSG00000235162	C12orf75	1563.660274	892.2243029	2235.096246	1.324385231	0.069841105	18.9628333	3.46E-80	1.55E-78
ENSG00000172403	SYNPO2	37.96016558	21.66088923	54.25944193	1.32411896	0.371932449	3.56010605	0.000371	0.001068
ENSG00000011332	DPF1	77.26823089	44.3284815	110.2079803	1.323121083	0.316258705	4.18366693	2.87E-05	9.47E-05
ENSG00000181773	GPR3	75.77838344	43.25231943	108.3044475	1.322426759	0.283443571	4.6655733	3.08E-06	1.13E-05
ENSG00000135299	ANKRD6	249.3307412	142.4364533	356.225029	1.321856854	0.156562513	8.44299717	3.09E-17	2.49E-16
ENSG00000101298	SNPH	832.4939074	475.8867864	1189.101028	1.321524562	0.08329049	15.866452	1.08E-56	3.03E-55
ENSG00000187266	EPOR	887.9682708	507.6412255	1268.295316	1.321398636	0.08561345	15.4344748	9.60E-54	2.58E-52
ENSG00000141198	TOM1L1	118.0579537	67.58931578	168.5265916	1.319659731	0.211913665	6.22734609	4.74E-10	2.44E-09
ENSG00000169851	PCDH7	1544.462109	883.3928765	2205.531342	1.319133946	0.079087772	16.6793667	1.85E-62	5.93E-61
ENSG00000126709	IFI6	6303.060996	3611.006895	8995.115098	1.316738598	0.146821542	8.96829294	3.01E-19	2.66E-18
ENSG00000164867	NOS3	1694.587712	970.2928905	2418.882533	1.316571602	0.063702772	20.6674147	6.81E-95	3.90E-93
ENSG00000215845	TSTD1	87.88615554	50.26257057	125.5097405	1.315992379	0.249448556	5.27560633	1.32E-07	5.60E-07
ENSG00000043591	ADRB1	40.62555056	23.29520277	57.95589834	1.315657443	0.381123731	3.45204808	0.000556	0.001561
ENSG00000184785	SMIM10	46.45954075	26.70590482	66.21317668	1.315486339	0.331546539	3.96772756	7.26E-05	0.000228
ENSG00000105711	SCN1B	490.3498604	280.9683252	699.7313956	1.314945859	0.105656139	12.4455225	1.48E-35	2.42E-34
ENSG00000139832	RAB20	99.41188918	57.08266684	141.7411115	1.314325273	0.262284524	5.01106681	5.41E-07	2.16E-06
ENSG00000128596	CCDC136	66.27381694	38.19841891	94.34921498	1.312575182	0.310665035	4.2250496	2.39E-05	7.96E-05
ENSG00000166664	CHRFAM7A	42.90106955	24.55245382	61.24968527	1.31200311	0.371720391	3.52954302	0.000416	0.00119
ENSG00000232527	AC245595.1	100.5449807	57.89132196	143.1986394	1.31173199	0.281660584	4.65713721	3.21E-06	1.17E-05
ENSG00000122707	RECK	1537.394385	883.3203956	2191.468374	1.310626891	0.064927551	20.1859898	1.30E-90	6.92E-89
ENSG00000170430	MGMT	61.80358004	35.64142997	87.96573012	1.310410222	0.305735971	4.28608455	1.82E-05	6.13E-05
ENSG00000128340	RAC2	9431.639815	5423.897198	13439.38243	1.309099477	0.034851325	37.5624022	0	0
ENSG00000118402	ELOVL4	627.2232101	361.3463758	893.1000444	1.307107495	0.103062667	12.6826477	7.38E-37	1.25E-35
ENSG00000235034	C19orf81	20.883884	11.99392605	29.77384195	1.307053589	0.504302979	2.59180224	0.009547	0.021851
ENSG00000114812	VIPR1	31.76753037	18.38599286	45.14906788	1.302266703	0.444524824	2.92957026	0.003394	0.008416
ENSG00000146530	VWDE	100.0209218	57.80828636	142.2335573	1.301699878	0.238396515	5.4602303	4.76E-08	2.08E-07
ENSG00000116774	OLFML3	152.3917484	87.85800533	216.9254915	1.30150588	0.193539356	6.72476083	1.76E-11	1.00E-10
ENSG00000140876	NUDT7	38.19813969	22.06451765	54.33176174	1.301375715	0.363409196	3.58102032	0.000342	0.000991
ENSG00000137872	SEMA6D	1863.886954	1076.527282	2651.246627	1.299810921	0.058106307	22.3695324	7.79E-111	5.73E-109
ENSG00000146856	AGBL3	35.21060451	20.33233008	50.08887895	1.298823064	0.395844038	3.28114848	0.001034	0.002798
ENSG00000038295	TLL1	763.2637624	441.9786725	1084.548852	1.296744018	0.107050713	12.1133619	8.97E-34	1.40E-32

ENSG00000260456	C16orf95	20.0941274	11.68956997	28.49868484	1.296010367	0.508509316	2.54864626	0.010814	0.024473
ENSG00000130303	BST2	5437.011608	3150.315729	7723.707488	1.293941774	0.110502162	11.7096512	1.14E-31	1.64E-30
ENSG00000139289	PHLDA1	6265.362028	3633.115517	8897.608538	1.292125666	0.038484061	33.5756058	3.81E-247	8.85E-245
ENSG00000105996	HOXA2	48.21591888	28.05922533	68.37261243	1.284584686	0.337823103	3.80253652	0.000143	0.000434
ENSG00000161921	CXCL16	504.9235947	294.2466095	715.6005799	1.281042159	0.114956893	11.1436741	7.69E-29	1.01E-27
ENSG00000103260	METRN	968.9341944	565.0493547	1372.819034	1.280870696	0.091094301	14.0609311	6.60E-45	1.40E-43
ENSG00000227220	AL133346.1	59.85036673	34.95951464	84.74121882	1.279636129	0.352536244	3.6298002	0.000284	0.00083
ENSG00000145022	TCTA	1278.826022	745.9099246	1811.742118	1.279450804	0.069395023	18.4372127	6.61E-76	2.71E-74
ENSG00000142156	COL6A1	1856.848335	1083.053078	2630.643592	1.279325335	0.067863234	18.8515232	2.86E-79	1.26E-77
ENSG00000181804	SLC9A9	77.04728036	44.8951576	109.1994031	1.278812778	0.269508159	4.74498725	2.09E-06	7.79E-06
ENSG00000181513	ACBD4	193.2880372	112.6769281	273.8991463	1.278019793	0.180342959	7.08660766	1.37E-12	8.43E-12
ENSG00000120913	PDLIM2	388.4396223	226.9867271	549.8925176	1.277820124	0.12068464	10.5880924	3.38E-26	3.99E-25
ENSG00000128573	FOXP2	46.27788506	27.0892366	65.46652953	1.276187557	0.356969797	3.57505752	0.00035	0.001012
ENSG00000269821	KCNQ1OT1	141.963797	83.03954312	200.888051	1.275559912	0.44258556	2.882064	0.003951	0.009697
ENSG00000105877	DNAH11	165.9631127	97.10113394	234.8250914	1.275520735	0.178477209	7.14668693	8.89E-13	5.53E-12
ENSG00000141401	IMPA2	126.841181	74.28398008	179.3983819	1.275480072	0.223036709	5.71870019	1.07E-08	4.96E-08
ENSG00000227260	LINC01985	16.98238712	9.951970156	24.01280409	1.275152429	0.537502636	2.37236498	0.017675	0.038223
ENSG00000265972	TXNIP	1086.968536	635.6862484	1538.250823	1.274700618	0.089081386	14.3093936	1.91E-46	4.19E-45
ENSG00000079691	CARMIL1	215.6378791	126.3243375	304.9514208	1.273220223	0.17286213	7.36552432	1.76E-13	1.15E-12
ENSG00000142606	MMEL1	29.3849087	17.12635149	41.64346591	1.272144044	0.457727072	2.77926328	0.005448	0.013041
ENSG00000012061	ERCC1	2354.306107	1381.052679	3327.559534	1.268642281	0.05635415	22.5119584	3.17E-112	2.40E-110
ENSG00000141480	ARRB2	572.8475084	335.7775213	809.9174954	1.268078802	0.107276017	11.8207111	3.05E-32	4.51E-31
ENSG00000231770	TMEM44-AS1	20.55442617	12.02234105	29.08651129	1.267637382	0.505513879	2.50762132	0.012155	0.027216
ENSG00000260549	MT1L	2250.944484	1320.535225	3181.353744	1.267536737	0.065584205	19.3268597	3.19E-83	1.50E-81
ENSG00000197081	IGF2R	20471.52977	12017.87504	28925.18451	1.267012588	0.033228942	38.1297905	0	0
ENSG00000154258	ABCA9	480.9008552	283.392621	678.4090895	1.262720191	0.120691624	10.4623681	1.29E-25	1.49E-24
ENSG00000115008	IL1A	373.3244061	219.547364	527.1014483	1.26238424	0.134782317	9.36609687	7.53E-21	7.11E-20
ENSG00000175182	FAM131A	1074.066672	632.7328463	1515.400499	1.259618025	0.072807334	17.3007025	4.65E-67	1.64E-65
ENSG00000156345	CDK20	30.48049675	17.96657851	42.99441499	1.259106481	0.425164496	2.96145725	0.003062	0.007656
ENSG00000177842	ZNF620	93.40379501	55.07940917	131.7281809	1.255825637	0.233326574	5.38226578	7.36E-08	3.17E-07
ENSG00000162426	SLC45A1	76.79369742	45.35972042	108.2276744	1.255503712	0.256611681	4.89262106	9.95E-07	3.86E-06
ENSG00000153823	PID1	21.05637067	12.46205204	29.6506893	1.254052784	0.518659487	2.41787303	0.015612	0.034149
ENSG00000178537	SLC25A20	693.4366619	409.7269258	977.146398	1.253599637	0.090486812	13.8539485	1.20E-43	2.49E-42
ENSG00000090530	P3H2	340.3595561	201.3573001	479.361812	1.253352881	0.124376889	10.0770561	6.98E-24	7.52E-23
ENSG00000114251	WNT5A	56.54971618	33.5232045	79.57622786	1.252947782	0.32294907	3.87970704	0.000105	0.000323
ENSG00000116678	LEPR	1263.540952	747.388812	1779.693092	1.251465459	0.070518692	17.7465779	1.83E-70	6.92E-69
ENSG00000235374	SSR4P1	89.87282628	53.12445685	126.6211957	1.251380968	0.276076613	4.53273081	5.82E-06	2.07E-05
ENSG00000005884	ITGA3	6001.197258	3551.216272	8451.178244	1.250306701	0.042038451	29.7419786	2.20E-194	3.81E-192
ENSG00000136883	KIF12	56.37874774	33.39010494	79.36739054	1.248950755	0.31121203	4.01318276	5.99E-05	0.00019
ENSG00000167601	AXL	5381.141138	3189.621021	7572.661255	1.247410122	0.041184876	30.2880634	1.65E-201	2.99E-199
ENSG00000186019	AC021092.1	82.71057992	49.10364433	116.3175155	1.244182924	0.271318348	4.58569401	4.52E-06	1.63E-05
ENSG00000187244	BCAM	1193.7819	708.5409527	1679.022846	1.243721549	0.072415853	17.1747137	4.11E-66	1.41E-64
ENSG00000130702	LAMA5	5416.445414	3217.044965	7615.845863	1.243163057	0.050421904	24.6552187	3.24E-134	3.19E-132
ENSG00000167107	ACSF2	826.519729	491.7235484	1161.315909	1.241307867	0.083633424	14.8422462	7.81E-50	1.87E-48
ENSG00000157064	NMNAT2	53.39263493	31.70933512	75.07593473	1.240979874	0.30967788	4.00732488	6.14E-05	0.000195
ENSG00000170891	CYTL1	1629.267	970.0766205	2288.45738	1.237571852	0.061873974	20.0014929	5.34E-89	2.79E-87
ENSG00000050165	DKK3	26382.50818	15712.75119	37052.26517	1.237553692	0.031929041	38.7595009	0	0
ENSG00000138449	SLC40A1	1160.617708	691.4961021	1629.739314	1.236052537	0.074817712	16.5208546	2.60E-61	8.15E-60
ENSG00000216775	AL109918.1	69.04544868	41.3015175	96.78937986	1.235305524	0.301664512	4.09496468	4.22E-05	0.000137
ENSG00000286190	AC055839.2	150.5675691	89.96459379	211.1705445	1.234602819	0.220738668	5.59305186	2.23E-08	1.00E-07
ENSG00000182685	BRICD5	77.00919112	46.01412231	108.0042599	1.234501976	0.350009035	3.52705746	0.00042	0.0012
ENSG00000154511	DIPK1A	44.56896177	26.52634898	62.61157456	1.233283069	0.345335023	3.57126555	0.000355	0.001027
ENSG00000063180	CA11	132.4889084	79.0771842	185.9006326	1.230938765	0.224373826	5.48610676	4.11E-08	1.81E-07
ENSG00000106686	SPATA6L	40.58970562	24.17994652	56.99946471	1.230078572	0.394855456	3.11526294	0.001838	0.004762
ENSG00000124920	MYRF	87.47767001	52.27124048	122.6840995	1.229528817	0.241075768	5.10017587	3.39E-07	1.38E-06
ENSG00000198682	PAPSS2	16524.89993	9881.307853	23168.492	1.229385794	0.031503547	39.0237259	0	0
ENSG00000198753	PLXNB3	806.252626	482.4982801	1130.006972	1.227324947	0.090411877	13.5748199	5.65E-42	1.13E-40
ENSG00000255248	MIR100HG	451.9159408	270.9345384	632.8973431	1.226666922	0.113433935	10.8139326	2.96E-27	3.64E-26
ENSG00000283378	CNTNAP3C	42.74406294	25.62627007	59.86185581	1.225983538	0.340350386	3.60212178	0.000316	0.000918
ENSG00000184515	BEX5	84.80757995	50.92518129	118.6899786	1.225012028	0.275111826	4.45277851	8.48E-06	2.96E-05
ENSG00000166402	TUB	222.8912312	133.6755225	312.1069399	1.224126058	0.160366159	7.63331904	2.29E-14	1.57E-13
ENSG00000065833	ME1	1758.734704	1055.682625	2461.786783	1.222370912	0.062489783	19.5611324	3.32E-85	1.61E-83
ENSG00000116574	RHOU	51.15232284	30.71904976	71.58559591	1.220198729	0.325407325	3.74975803	0.000177	0.000531
ENSG00000286215	AL356534.1	44.72745294	26.7676058	62.68730007	1.220017227	0.343701153	3.54964544	0.000386	0.001108
ENSG00000267058	AC006213.3	25.68000782	15.51558195	35.84443369	1.216957146	0.461212682	2.63860295	0.008325	0.019275
ENSG00000171604	CXXC5	250.7588448	151.1424936	350.3751959	1.215469769	0.153612572	7.91256699	2.52E-15	1.83E-14
ENSG00000182993	C12orf60	54.67323875	32.97750112	76.36897637	1.211864107	0.308893539	3.923242	8.74E-05	0.000272
ENSG00000152076	CCDC74B	56.38944708	33.95786355	78.8210306	1.211395557	0.296426576	4.08666313	4.38E-05	0.000141
ENSG00000228536	LYPLAL1-AS1	33.55824918	20.32831613	46.78818223	1.20876104	0.390131488	3.09834268	0.001946	0.005027
ENSG00000025708	TYMP	374.9421771	226.5162108	523.3681434	1.20777949	0.122335021	9.8727207	5.47E-23	5.71E-22
ENSG00000232818	RPS2P32	42.17783117	25.4336344	58.92202793	1.20495665	0.362145088	3.32727598	0.000877	0.002393
ENSG00000198416	ZNF658B	27.38142082	16.55773583	38.20510581	1.204815279	0.43023399	2.80037214	0.005104	0.012279
ENSG00000231312	MAP4K3-DT	102.4352435	61.91605796	142.9544291	1.204708923	0.225032248	5.35349459	8.63E-08	3.69E-07
ENSG00000148225	WDR31	60.26663736	36.36270045	84.15057427	1.204567282	0.303991062	3.96250888	7.42E-05	0.000233
ENSG00000175352	NRIP3	1415.003449	855.8070085	1974.199889	1.204522357	0.069233368	17.3980031	8.54E-68	3.08E-66
ENSG00000187229	AC100800.1	38.1025668	23.09769612	53.10743748	1.203261597	0.377428599	3.18805094	0.001432	0.003782
ENSG00000134013	LOXL2	40975.37626	24819.00516	57131.74737	1.202906694	0.027581439	43.6129059	0	0
ENSG00000273415	AP000904.1	68.93026452	41.79526188	96.06526717	1.200407665	0.284200871	4.22380009	2.40E-05	8.00E-05
ENSG00000101842	VSIG1	236.7639128	143.6869843	329.8408413	1.199187965	0.153709895	7.80163155	6.11E-15	4.33E-14
ENSG00000163751	CPA3	98.69630325	59.92100912	137.4715974	1.198179105	0.240133318	4.98964123	6.05E-07	2.40E-06
ENSG00000134864	GGACT	202.1478958	122.7737698	281.5220218	1.196971319	0.163305245	7.3296563	2.31E-13	1.49E-12
ENSG00000141934	PLPP2	847.5779237	514.9996274	1180.15622	1.196591159	0.081006227	14.7715947	2.23E-49	5.28E-48
ENSG00000135749	PCNX2	463.6450423	281.5708126	645.7192719	1.196571152	0.119099275	10.0468383	9.49E-24	1.02E-22
ENSG00000064115	TM7SF3	2451.556618	1490.207816	3412.905419	1.196071316	0.057608671	20.7620014	9.55E-96	5.61E-94
ENSG00000164638	SLC29A4	401.619451	243.7380905	559.5008115	1.195671855	0.131533188	9.09026745	9.88E-20	8.94E-19

ENSG00000163513	TGFBR2	62454.3538	37973.5909	86935.11671	1.194936203	0.035097132	34.0465487	4.57E-254	1.10E-251
ENSG00000167779	IGFBP6	74.42334401	45.37645388	103.4702341	1.193879362	0.268929272	4.43938049	9.02E-06	3.14E-05
ENSG00000176438	SYNE3	559.4767451	340.5573298	778.3961604	1.190357302	0.102279612	11.6382657	2.63E-31	3.73E-30
ENSG00000100504	PYGL	1421.513366	866.23139	1976.795341	1.189551958	0.07209829	16.4990315	3.73E-61	1.16E-59
ENSG00000134594	RAB33A	34.72794468	21.30110146	48.15478791	1.18888678	0.423679075	2.80610219	0.005014	0.012071
ENSG00000162694	EXTL2	2405.600277	1467.551184	3343.649371	1.188742252	0.057050609	20.8366267	2.02E-96	1.20E-94
ENSG00000169213	RAB3B	4603.90532	2807.562005	6400.248636	1.188488481	0.043430625	27.3652173	7.12E-165	9.90E-163
ENSG00000168843	FSTL5	164.2076794	99.95197028	228.4633886	1.188343669	0.198077569	5.99938536	1.98E-09	9.70E-09
ENSG00000256073	URB1-AS1	75.28118651	45.92477291	104.6376001	1.187414229	0.254334097	4.66871821	3.03E-06	1.11E-05
ENSG00000144647	POMGNT2	385.5303044	235.6128902	535.4477187	1.186644732	0.121495704	9.76696862	1.56E-22	1.59E-21
ENSG00000129355	CDKN2D	156.4363524	95.69098345	217.1817214	1.186359255	0.188753447	6.28523228	3.27E-10	1.71E-09
ENSG00000099282	TSPAN15	4342.010388	2650.524392	6033.496384	1.186322405	0.044615782	26.589748	8.92E-156	1.10E-153
ENSG00000116117	PARD3B	979.2568748	598.6945644	1359.819185	1.185094016	0.077569528	15.2778295	1.07E-52	2.80E-51
ENSG00000158528	PPP1R9A	84.84287676	51.92584035	117.7599132	1.182337502	0.250385759	4.72206369	2.33E-06	8.67E-06
ENSG00000074527	NTN4	9895.415586	6058.691621	13732.13955	1.180758557	0.042876851	27.5383691	6.10E-167	8.87E-165
ENSG00000132879	FBXO44	595.3987709	364.4894344	826.3081073	1.18070783	0.107589023	10.9742406	5.08E-28	6.39E-27
ENSG00000158186	MRAS	692.6630324	424.4858116	960.8402533	1.177988003	0.092025231	12.8007069	1.62E-37	2.83E-36
ENSG00000053747	LAMA3	565.7895385	347.1836636	784.3954134	1.176589383	0.103032474	11.4195975	3.34E-30	4.57E-29
ENSG00000214063	TSPAN4	1968.653543	1208.176861	2729.130225	1.174998651	0.060518155	19.4156392	5.69E-84	2.72E-82
ENSG00000213073	AL353625.1	73.68288399	45.20501641	102.1607516	1.174851163	0.261601158	4.49100139	7.09E-06	2.49E-05
ENSG00000069122	ADGRF5	9133.754614	5611.307194	12656.20203	1.172887574	0.040477264	28.976454	1.30E-184	2.07E-182
ENSG00000133740	E2F5	117.4703063	72.20715895	162.7334536	1.172414652	0.232440613	5.04393204	4.56E-07	1.83E-06
ENSG00000168679	SLC16A4	112.7643168	69.34757253	156.1810611	1.170912506	0.224815692	5.20832196	1.91E-07	7.94E-07
ENSG00000124370	MCEE	156.6641068	96.59020532	216.7380082	1.169283308	0.199461532	5.86219956	4.57E-09	2.18E-08
ENSG00000004776	HSPB6	103.1155607	63.487723	142.7433984	1.168596053	0.22687565	5.15082184	2.59E-07	1.07E-06
ENSG00000215105	TTC3P1	186.9229268	115.2664824	258.5793712	1.164760995	0.179449782	6.49073506	8.54E-11	4.63E-10
ENSG00000101955	SRPX	12374.73194	7643.933268	17105.5306	1.162183545	0.033115376	35.0949825	8.04E-270	2.10E-267
ENSG00000184194	GPR173	366.4078123	226.2447344	506.5708903	1.162090824	0.153694531	7.56104213	4.00E-14	2.69E-13
ENSG00000153029	MR1	106.2892802	65.86488627	146.7136742	1.160767561	0.233276165	4.97593727	6.49E-07	2.57E-06
ENSG00000137960	GIPC2	332.3845344	205.6816117	459.0874572	1.159318053	0.130320206	8.89591943	5.79E-19	5.05E-18
ENSG00000178015	GPR150	54.22989429	33.48928679	74.97050179	1.157571605	0.314776256	3.67744257	0.000236	0.000695
ENSG00000140945	CDH13	12675.47668	7847.370498	17503.58286	1.157280728	0.03217741	35.9656273	2.88E-283	8.20E-281
ENSG00000189410	SH2D5	39.73126983	24.73115261	54.73138704	1.156719191	0.416467748	2.77745203	0.005479	0.013106
ENSG00000103202	NME4	5900.605624	3653.932615	8147.278632	1.156677249	0.046623142	24.8090798	7.15E-136	7.25E-134
ENSG00000001461	NIPAL3	1074.070678	665.1920671	1482.94929	1.15589116	0.073211552	15.7883713	3.74E-56	1.04E-54
ENSG00000206535	LNP1	24.49114218	15.15159928	33.83068508	1.155236942	0.467880771	2.46908403	0.013546	0.030045
ENSG00000163879	DNALI1	177.4289541	109.7289398	245.1289683	1.154855582	0.179801809	6.42293639	1.34E-10	7.16E-10
ENSG00000134243	SORT1	1555.250222	963.8093539	2146.691091	1.15411593	0.065828334	17.5322062	8.14E-69	2.99E-67
ENSG00000174684	B4GAT1	1122.947694	696.1304532	1549.764934	1.152508956	0.082193536	14.0218929	1.15E-44	2.42E-43
ENSG00000103034	NDRG4	3533.824415	2193.588875	4874.059955	1.151341311	0.052008888	22.1373951	1.38E-108	9.73E-107
ENSG00000225746	MEG8	119.6782297	74.4508164	164.9056295	1.150396581	0.2366846	4.86046232	1.17E-06	4.50E-06
ENSG00000255836	AC131206.1	44.07460369	27.35931468	60.7898927	1.149968404	0.40700039	2.82547248	0.004721	0.011425
ENSG00000247708	STX18-AS1	23.04274392	14.28620465	31.7992832	1.149811428	0.467876155	2.45751235	0.01399	0.030933
ENSG00000119632	IFI27L2	588.9513565	365.6392503	812.2634627	1.149778347	0.104053968	11.049827	2.20E-28	2.82E-27
ENSG00000186951	PPARA	1120.594152	696.3073031	1544.881002	1.149440979	0.078433988	14.6548838	1.25E-48	2.90E-47
ENSG00000203722	RAET1G	23.38444379	14.48596665	32.28292093	1.149387924	0.463775006	2.47833089	0.0132	0.029359
ENSG00000184867	ARMCX2	5130.027763	3191.204502	7068.851023	1.14746427	0.045424857	25.2607126	8.64E-141	9.45E-139
ENSG00000128595	CALU	46553.63732	28971.87478	64135.39987	1.146510686	0.029029029	39.4953169	0	0
ENSG00000176697	BDNF	462.5345688	288.0896652	636.9794724	1.145905416	0.117073815	9.78788821	1.27E-22	1.30E-21
ENSG00000114023	FAM162A	610.3714214	379.590338	841.1525048	1.145803733	0.101076886	11.335962	8.71E-30	1.18E-28
ENSG00000136367	ZFHX2	131.60855	81.93677063	181.2803294	1.143458893	0.212090085	5.39138308	6.99E-08	3.02E-07
ENSG00000158106	RHPN1	250.2053304	155.9898216	344.4208392	1.14343291	0.200945779	5.69025594	1.27E-08	5.82E-08
ENSG00000213903	LTB4R	140.3825781	87.53109727	193.2340589	1.142562257	0.492612524	2.31939344	0.020374	0.043484
ENSG00000099937	SERPIND1	177.2937471	110.7231043	243.8643899	1.142059841	0.172368997	6.62566853	3.46E-11	1.93E-10
ENSG00000133107	TRPC4	147.8098596	92.22746536	203.3922539	1.14188214	0.187061114	6.10432662	1.03E-09	5.17E-09
ENSG00000119782	FKBP1B	137.1392617	85.5350564	188.743467	1.139990894	0.19072904	5.97701792	2.27E-09	1.11E-08
ENSG00000181035	SLC25A42	251.6927357	157.1420278	346.2434435	1.139874481	0.154363093	7.38437186	1.53E-13	9.99E-13
ENSG00000107338	SHB	531.5423924	331.8217017	731.2630832	1.138762802	0.104586072	10.8882835	1.31E-27	1.63E-26
ENSG00000185495	AC138393.1	29.87453778	18.7043763	41.04469926	1.138085587	0.435636226	2.61246774	0.008989	0.020677
ENSG00000180801	ARSJ	7411.282681	4631.848033	10190.71733	1.137555332	0.037038014	30.7131841	3.80E-207	7.30E-205
ENSG00000121297	TSHZ3	139.9964898	87.59203168	192.400948	1.13701939	0.203810172	5.57881572	2.42E-08	1.08E-07
ENSG00000072133	RPS6KA6	98.45962565	61.76554883	135.1537025	1.133745239	0.237893799	4.76576205	1.88E-06	7.08E-06
ENSG00000198885	ITPRIPL1	240.6126203	150.6607466	330.5644941	1.133729594	0.143663409	7.89156824	2.98E-15	2.15E-14
ENSG00000170689	HOXB9	86.69362471	54.30963321	119.0776162	1.132913654	0.245140699	4.62148333	3.81E-06	1.38E-05
ENSG00000285894	AL136372.2	49.41148341	30.97293562	67.8500312	1.132873639	0.321041658	3.52874342	0.000418	0.001193
ENSG00000144730	IL17RD	101.6786796	63.60850931	139.7488499	1.131958017	0.262237816	4.31653235	1.58E-05	5.38E-05
ENSG00000186104	CYP2R1	326.9931033	204.9884211	448.9977854	1.131833674	0.125020257	9.05320226	1.39E-19	1.24E-18
ENSG00000120306	CYSTM1	1427.53663	894.9317121	1960.141547	1.131101902	0.06855825	16.498407	3.77E-61	1.17E-59
ENSG00000203778	FAM229B	223.386516	140.1630731	306.609959	1.128519755	0.149159356	7.56586635	3.85E-14	2.60E-13
ENSG00000102032	RENBP	220.4803334	138.2973351	302.6633316	1.126542605	0.156574839	7.19491468	6.25E-13	3.93E-12
ENSG00000211584	SLC48A1	524.2416765	329.4055703	719.0777826	1.125645265	0.121509316	9.26385977	1.97E-20	1.83E-19
ENSG00000198929	NOS1AP	50.84584124	32.10313108	69.58855141	1.124986871	0.332227044	3.38619896	0.000709	0.001962
ENSG00000179965	ZNF771	237.7944782	149.6010022	325.9879543	1.124501509	0.156792988	7.17188645	7.40E-13	4.63E-12
ENSG00000169047	IRS1	46.28691207	29.23587707	63.33794708	1.124082807	0.370529867	3.03371714	0.002416	0.006149
ENSG00000116151	MORN1	130.215686	81.99102948	178.4403425	1.123139627	0.233275324	4.81465251	1.47E-06	5.61E-06
ENSG00000069702	TGFBR3	684.6846612	430.8222212	938.5471012	1.122428077	0.091284137	12.2959817	9.52E-35	1.53E-33
ENSG00000145687	SSBP2	279.2630847	175.9492841	382.5768854	1.121793321	0.135209148	8.29672651	1.07E-16	8.39E-16
ENSG00000076351	SLC46A1	260.1307521	163.9991082	356.262396	1.121703688	0.15482538	7.24496002	4.33E-13	2.75E-12
ENSG00000011638	TMEM159	951.9020487	599.4840053	1304.320092	1.121591802	0.077477562	14.4763435	1.71E-47	3.83E-46
ENSG00000172037	LAMB2	24384.84757	15372.14474	33397.5504	1.119266381	0.030312421	36.9243485	1.88E-298	5.87E-296
ENSG00000102452	NALCN	213.9602144	135.0510347	292.8693942	1.119066184	0.167079922	6.69778973	2.12E-11	1.20E-10
ENSG00000166387	PPFIBP2	32.86412617	20.73099703	44.99725531	1.117884995	0.434270934	2.5741649	0.010048	0.0229
ENSG00000214595	EML6	39.36943667	24.84427134	53.894602	1.11693858	0.358366639	3.11674821	0.001829	0.00474
ENSG00000198794	SCAMP5	684.1148969	432.4793122	935.7504817	1.114465808	0.090842133	12.2681598	1.34E-34	2.14E-33
ENSG00000145730	PAM	11629.11297	7350.26542	15907.96053	1.11397288	0.038727396	28.7644667	5.97E-182	9.25E-180

ENSG00000185164	NOMO2	6011.928536	3800.818545	8223.038527	1.113200826	0.038658457	28.7957907	2.42E-182	3.78E-180
ENSG00000164929	BAALC	61.93552464	39.23290521	84.63814407	1.112412125	0.318249543	3.4954084	0.000473	0.00134
ENSG00000013016	EHD3	666.8895328	421.8480424	911.9310231	1.111507579	0.091120824	12.198173	3.18E-34	5.02E-33
ENSG00000272902	TBC1D8-AS1	30.42800359	19.18774697	41.66826021	1.110855529	0.412321197	2.69415091	0.007057	0.016542
ENSG00000285533	AP001362.2	32.60482247	20.62229852	44.58734643	1.110203692	0.388239054	2.85958788	0.004242	0.010349
ENSG00000133315	MACROD1	260.8061926	165.2838267	356.3285585	1.107887797	0.147168842	7.52800512	5.15E-14	3.45E-13
ENSG00000169169	CPT1C	1287.161412	815.6848489	1758.637975	1.107601803	0.086642936	12.7835211	2.03E-37	3.51E-36
ENSG00000130997	POLN	31.9540285	20.24167286	43.66638414	1.107382714	0.432546048	2.56014988	0.010463	0.02376
ENSG00000247809	NR2F2-AS1	36.28488056	22.91814028	49.65162084	1.106814564	0.394230192	2.80753374	0.004992	0.012025
ENSG00000165821	SALL2	210.8280988	133.5594722	288.0967253	1.105648361	0.179712167	6.15232892	7.64E-10	3.87E-09
ENSG00000119514	GALNT12	84.65290341	53.75720977	115.5485971	1.104519863	0.260256901	4.24395994	2.20E-05	7.35E-05
ENSG00000106333	PCOLCE	64.11905822	40.88038905	87.3577274	1.10445355	0.343600811	3.21435082	0.001307	0.00348
ENSG00000164176	EDIL3	2939.977785	1867.322531	4012.63304	1.103112212	0.049188815	22.4260781	2.19E-111	1.63E-109
ENSG00000013583	HEBP1	1829.437798	1162.329801	2496.545795	1.102617559	0.057471637	19.1854211	4.90E-82	2.25E-80
ENSG00000166825	ANPEP	37367.42693	23744.44121	50990.41264	1.102545258	0.028853422	38.2119413	0	0
ENSG00000065485	PDIA5	5709.400932	3627.945433	7790.856432	1.102530723	0.040181295	27.438905	9.42E-166	1.34E-163
ENSG00000110628	SLC22A18	47.37095049	30.16662632	64.57527466	1.101562159	0.329140279	3.34678625	0.000818	0.00224
ENSG00000188060	RAB42	57.53190638	36.59468523	78.46912753	1.100082222	0.301725025	3.64597608	0.000266	0.000782
ENSG00000226200	SGMS1-AS1	88.97409723	56.7926984	121.1554961	1.097322388	0.246661072	4.44870517	8.64E-06	3.01E-05
ENSG00000187091	PLCD1	564.1124483	359.6979444	768.5269521	1.095186631	0.09575827	11.4369927	2.73E-30	3.76E-29
ENSG00000137720	C11orf1	102.1143682	65.23393791	138.9947985	1.094739979	0.23332342	4.6919421	2.71E-06	9.97E-06
ENSG00000271605	MILR1	101.1591682	64.6098001	137.7085364	1.094413342	0.230380291	4.75046427	2.03E-06	7.59E-06
ENSG00000223478	AL441992.1	43.78772153	28.02409027	59.55135279	1.093798291	0.350861692	3.11746285	0.001824	0.00473
ENSG00000101844	ATG4A	1495.762114	954.5107033	2037.013524	1.093224278	0.071240713	15.3454989	3.80E-53	1.00E-51
ENSG00000132793	LPIN3	101.0635432	64.56194552	137.565141	1.092966515	0.228017307	4.79334893	1.64E-06	6.22E-06
ENSG00000138604	GLCE	7732.950273	4937.943923	10527.95662	1.091901735	0.038981096	28.0110575	1.19E-172	1.77E-170
ENSG00000168803	ADAL	173.5497341	110.7441675	236.3553007	1.091785396	0.173411591	6.29591938	3.06E-10	1.60E-09
ENSG00000253837	AC090197.1	43.26358651	27.66691814	58.86025488	1.091159122	0.352985276	3.09123127	0.001993	0.005141
ENSG00000072062	PRKACA	5698.515943	3640.829017	7756.202869	1.091045563	0.040255243	27.1031917	9.03E-162	1.18E-159
ENSG00000130224	LRCH2	471.2893882	301.1626847	641.4160916	1.089683598	0.104485918	10.4289996	1.83E-25	2.11E-24
ENSG00000095397	WHRN	81.53265359	52.08951977	110.9757874	1.089538829	0.271250191	4.01673018	5.90E-05	0.000188
ENSG00000188266	HYKK	37.45595652	23.95316783	50.95874522	1.089077494	0.366480982	2.97171626	0.002961	0.007427
ENSG00000125457	MIF4GD	238.5853025	152.5106081	324.659997	1.088711918	0.154033451	7.06802264	1.57E-12	9.59E-12
ENSG00000125740	FOSB	45.4577092	29.08969768	61.82572073	1.08718684	0.331713732	3.27748518	0.001047	0.002831
ENSG00000213693	SEC14L1P1	58.87953333	37.54879754	80.21026912	1.086755829	0.391429145	2.77637943	0.005497	0.013144
ENSG00000132321	IQCA1	147.565934	94.61179947	200.5200685	1.086205744	0.206004498	5.27272828	1.34E-07	5.68E-07
ENSG00000154678	PDE1C	1111.747996	711.9963969	1511.499594	1.085623207	0.072096581	15.0579013	3.06E-51	7.68E-50
ENSG00000125648	SLC25A23	1721.48743	1103.61539	2339.359469	1.084137971	0.064069461	16.9212907	3.13E-64	1.04E-62
ENSG00000058668	ATP2B4	7509.466045	4813.640886	10205.2912	1.08396189	0.034651668	31.2816653	8.29E-215	1.77E-212
ENSG00000236397	DDX11L2	117.8354985	75.4942781	160.1767188	1.083394504	0.22798566	4.75202915	2.01E-06	7.54E-06
ENSG00000133138	TBC1D8B	1000.329156	641.7362069	1358.922105	1.083352916	0.085816748	12.6240267	1.56E-36	2.62E-35
ENSG00000198934	MAGEE1	103.1009929	65.97110398	140.2308818	1.082282394	0.263793424	4.10276487	4.08E-05	0.000133
ENSG00000111144	LTA4H	3753.105105	2409.42425	5096.78596	1.080593028	0.046110785	23.4347134	1.89E-121	1.61E-119
ENSG00000167771	RCOR2	63.63300926	40.89423544	86.37178307	1.080178718	0.289643314	3.72934111	0.000192	0.000573
ENSG00000119986	AVPI1	143.965932	92.50751087	195.4243532	1.079716995	0.19161885	5.63471179	1.75E-08	7.96E-08
ENSG00000205181	LINC00654	71.23227023	45.91448968	96.55005078	1.079675205	0.288980648	3.73615054	0.000187	0.000559
ENSG00000272077	AC124045.1	23.82174045	15.30134183	32.34213906	1.079049528	0.446829777	2.41490067	0.01574	0.034419
ENSG00000124785	NRN1	966.8385665	621.4008993	1312.276234	1.077915503	0.077312148	13.942382	3.50E-44	7.32E-43
ENSG00000167549	CORO6	207.4510382	133.3940787	281.5079977	1.077654014	0.457133941	2.35741413	0.018403	0.039694
ENSG00000196878	LAMB3	2781.547551	1789.067171	3774.027931	1.0775704	0.053883243	19.9982471	5.70E-89	2.97E-87
ENSG00000169330	MINAR1	129.9558495	83.53459532	176.3771036	1.077183079	0.197990344	5.44058389	5.31E-08	2.32E-07
ENSG00000120725	SIL1	2419.870113	1558.006908	3281.733318	1.075129118	0.055426021	19.397552	8.09E-84	3.85E-82
ENSG00000113070	HBEGF	2043.41317	1316.406977	2770.419363	1.07416069	0.05493516	19.5532458	3.87E-85	1.87E-83
ENSG00000148926	ADM	1130.745885	728.5175089	1532.974261	1.073174872	0.075698788	14.1769096	1.27E-45	2.74E-44
ENSG00000259207	ITGB3	4122.947434	2656.374498	5589.52037	1.073043848	0.050844306	21.1045037	7.23E-99	4.58E-97
ENSG00000197283	SYNGAP1	799.9296396	516.1353701	1083.723909	1.071055333	0.110837041	9.6633339	4.32E-22	4.34E-21
ENSG00000102879	CORO1A	321.3250543	207.4322913	435.2178172	1.070491267	0.129868124	8.24291006	1.68E-16	1.31E-15
ENSG00000170175	CHRNB1	282.0139904	182.0742948	381.953686	1.069423242	0.13749448	7.77793582	7.37E-15	5.20E-14
ENSG00000154556	SORBS2	2315.327122	1493.875605	3136.778638	1.069173164	0.055399189	19.299437	5.43E-83	2.53E-81
ENSG00000170425	ADORA2B	429.1032702	277.2520497	580.9544906	1.068455188	0.129064568	8.27845477	1.25E-16	9.77E-16
ENSG00000001617	SEMA3F	5232.844585	3379.890335	7085.798834	1.068117161	0.04481165	23.8357024	1.42E-125	1.27E-123
ENSG00000145945	FAM50B	366.6585448	237.0676924	496.2493972	1.067444397	0.122593054	8.70721759	3.11E-18	2.63E-17
ENSG00000147697	GSDMC	59.86541959	38.5847726	81.14606657	1.067307368	0.315943362	3.37816044	0.00073	0.002016
ENSG00000114779	ABHD14B	543.7187362	351.5952945	735.8421779	1.064584357	0.100405732	10.6028246	2.89E-26	3.42E-25
ENSG00000104154	SLC30A4	455.155762	294.1151335	616.1963904	1.064424308	0.131298372	8.10691169	5.19E-16	3.91E-15
ENSG00000101335	MYL9	6994.132107	4525.630782	9462.633432	1.063781596	0.040777579	26.0874142	5.07E-150	5.88E-148
ENSG00000157379	DHRS1	478.0007319	309.1709333	646.8305304	1.063593484	0.113511355	9.36993026	7.26E-21	6.87E-20
ENSG00000144802	NFKBIZ	6634.520318	4294.639208	8974.401429	1.063490313	0.042761199	24.8704514	1.55E-136	1.60E-134
ENSG00000246898	LINC00920	310.693551	201.1858626	420.2012393	1.062940992	0.130523381	8.14368265	3.83E-16	2.91E-15
ENSG00000117152	RGS4	1000.640309	648.5189128	1352.761705	1.062008207	0.089192962	11.9068611	1.09E-32	1.64E-31
ENSG00000162745	OLFML2B	23.72434816	15.38388069	32.06481564	1.061197393	0.452388038	2.34576802	0.018988	0.040804
ENSG00000154639	CXADR	708.0033167	458.8928123	957.113821	1.060918566	0.099864193	10.6236132	2.31E-26	2.76E-25
ENSG00000213906	LTB4R2	60.77549165	39.39162316	82.15936013	1.060885728	0.334144111	3.17493468	0.001499	0.003944
ENSG00000164690	SHH	119.7635982	77.75823218	161.7689643	1.060865127	0.20856726	5.0864413	3.65E-07	1.48E-06
ENSG00000152558	TMEM123	17241.97194	11176.01768	23307.9262	1.060402366	0.03636218	29.1622328	5.85E-187	9.36E-185
ENSG00000258655	ARHGAP5-AS1	43.94117171	28.48630727	59.39603615	1.060102485	0.360887763	2.93748526	0.003309	0.008215
ENSG00000081377	CDC14B	143.6757731	92.93351031	194.4180359	1.059637742	0.208228705	5.08881684	3.60E-07	1.47E-06
ENSG00000107957	SH3PXD2A	1502.855154	974.2792901	2031.431018	1.059544967	0.066308251	15.9790818	1.79E-57	5.10E-56
ENSG00000187994	RINL	153.4683177	99.29445601	207.6421794	1.059287253	0.19432484	5.45111604	5.01E-08	2.19E-07
ENSG00000165475	CRYL1	704.8571489	457.4543373	952.2599604	1.058300941	0.086811837	12.1907447	3.48E-34	5.49E-33
ENSG00000131018	SYNE1	3531.490043	2291.956011	4771.024076	1.057223705	0.048522628	21.7882613	3.00E-105	2.07E-103
ENSG00000224167	LINC01357	58.15048107	37.73114997	78.56981217	1.057202001	0.304089791	3.4766113	0.000508	0.001432
ENSG00000256235	SMIM3	41.54099093	26.95343092	56.12855093	1.056514497	0.350698096	3.01260403	0.00259	0.006554
ENSG00000174130	TLR6	164.964138	107.3764935	222.5517824	1.054258347	0.202763064	5.19945953	2.00E-07	8.31E-07
ENSG00000226711	FAM66C	42.15105344	27.35209787	56.950009	1.053965411	0.381612173	2.76187576	0.005747	0.013693

ENSG00000204947	ZNF425	56.04286621	36.49550338	75.59022904	1.05352556	0.31510235	3.34343924	0.000827	0.002264
ENSG00000142892	PIGK	1377.020257	895.8119006	1858.228614	1.052990659	0.077661207	13.5587727	7.03E-42	1.40E-40
ENSG00000164512	ANKRD55	35.72592863	23.31960382	48.13225344	1.052695752	0.401497133	2.62192595	0.008743	0.020161
ENSG00000285331	AC090517.5	46.63698074	30.3875865	62.88637497	1.052052832	0.348352256	3.0200833	0.002527	0.006409
ENSG00000168067	MAP4K2	2756.349649	1794.019141	3718.680156	1.051856833	0.051082844	20.5911956	3.29E-94	1.86E-92
ENSG00000144810	COL8A1	50944.11656	33156.37915	68731.85396	1.051612917	0.029857144	35.2214845	9.38E-272	2.55E-269
ENSG00000105514	RAB3D	1938.439461	1262.214791	2614.66413	1.051050025	0.068972667	15.2386455	1.96E-52	5.06E-51
ENSG00000159784	FAM131B	112.2952198	73.10281766	151.4876218	1.051000859	0.210796615	4.98585263	6.17E-07	2.45E-06
ENSG00000178695	KCTD12	24466.54043	15939.47213	32993.60873	1.049580619	0.042728059	24.5642009	3.05E-133	2.93E-131
ENSG00000181031	RPH3AL	282.0124996	183.5695552	380.455444	1.048947928	0.137546641	7.62612538	2.42E-14	1.65E-13
ENSG00000197576	HOXA4	61.93052026	40.49340362	83.3676369	1.048518658	0.348162378	3.01157944	0.002599	0.006575
ENSG00000277283	AC004812.2	51.70693094	33.68548562	69.72837627	1.046340499	0.31356177	3.33695175	0.000847	0.002316
ENSG00000112561	TFEB	247.4381753	161.5421582	333.3341924	1.044700689	0.152758281	6.83891363	7.98E-12	4.65E-11
ENSG00000087076	HSD17B14	814.1278913	531.5708938	1096.684889	1.044696751	0.081450037	12.8262281	1.17E-37	2.05E-36
ENSG00000214274	ANG	106.6556982	69.66013745	143.651259	1.044245436	0.221611358	4.71205739	2.45E-06	9.09E-06
ENSG00000148218	ALAD	1988.717124	1298.860264	2678.573984	1.043750123	0.055545788	18.7908059	8.98E-79	3.91E-77
ENSG00000261308	FIGNL2	169.2147342	110.7285165	227.7009519	1.042345812	0.180690018	5.76869614	7.99E-09	3.74E-08
ENSG00000197043	ANXA6	17530.90875	11462.5272	23599.2903	1.04175888	0.034504556	30.1919227	3.02E-200	5.43E-198
ENSG00000235314	LINC00957	25.9652222	16.9085482	35.0218962	1.041631837	0.45846845	2.2719815	0.023088	0.048669
ENSG00000199161	MIR126	102.8459139	67.29524292	138.3965849	1.040687599	0.316122269	3.29204141	0.000995	0.002699
ENSG00000180938	ZNF572	27.53059422	18.03874366	37.02244478	1.040165101	0.4326638	2.40409552	0.016213	0.035326
ENSG00000119950	MXI1	282.9014433	185.0984176	380.704469	1.03951644	0.132286526	7.85806744	3.90E-15	2.80E-14
ENSG00000286169	AHRR	281.3728458	184.3724823	378.3732093	1.038459036	0.133973623	7.75122005	9.10E-15	6.37E-14
ENSG00000153714	LURAP1L	381.2082474	249.9559693	512.4605255	1.038015476	0.12955182	8.01235734	1.13E-15	8.32E-15
ENSG00000189067	LITAF	610.235002	399.7297167	820.7402873	1.036618934	0.097946982	10.5834699	3.56E-26	4.19E-25
ENSG00000204934	ATP6V0E2-AS1	30.91189658	20.28952744	41.53426572	1.036280052	0.419872242	2.46808421	0.013584	0.030125
ENSG00000164953	TMEM67	383.3628514	251.112821	515.6128818	1.035998267	0.129147789	8.02180411	1.04E-15	7.72E-15
ENSG00000090006	LTBP4	3551.103994	2327.974186	4774.233803	1.03589033	0.046066473	22.4868599	5.58E-112	4.17E-110
ENSG00000135740	SLC9A5	254.9159032	167.4101706	342.4216359	1.033087053	0.165569913	6.23958202	4.39E-10	2.26E-09
ENSG00000162241	SLC25A45	168.7902127	110.9129433	226.6674821	1.032796316	0.179630942	5.74954573	8.95E-09	4.17E-08
ENSG00000133069	TMCC2	217.7348992	143.0416928	292.4281056	1.03213893	0.157952403	6.53449338	6.38E-11	3.49E-10
ENSG00000234912	SNHG20	238.4623544	156.5273162	320.3973926	1.031854295	0.170844385	6.03973197	1.54E-09	7.61E-09
ENSG00000255775	AC005845.1	45.50390964	29.87525958	61.1325597	1.03140463	0.350204582	2.94514887	0.003228	0.008037
ENSG00000106366	SERPINE1	743829.1101	488716.9166	998941.3035	1.031400153	0.022710265	45.4155921	0	0
ENSG00000178531	CTXN1	1075.060695	706.8829145	1443.238476	1.031294567	0.09592742	10.7507797	5.88E-27	7.16E-26
ENSG00000107282	APBA1	391.740507	257.3276778	526.1533361	1.030514381	0.136301495	7.56055081	4.01E-14	2.70E-13
ENSG00000088298	EDEM2	2191.461855	1441.115854	2941.807856	1.030096658	0.054973536	18.7380461	2.42E-78	1.04E-76
ENSG00000146416	AIG1	439.1448078	288.5554008	589.7342147	1.030015885	0.111684317	9.22256508	2.90E-20	2.67E-19
ENSG00000108679	LGALS3BP	232.2550644	152.6751445	311.8349843	1.029922324	0.170182568	6.05186735	1.43E-09	7.08E-09
ENSG00000267458	AC092069.1	31.36153893	20.67556534	42.04751251	1.029462765	0.408828469	2.51807994	0.0118	0.026485
ENSG00000104635	SLC39A14	5518.025257	3629.938272	7406.112241	1.028686497	0.039562011	26.0018761	4.72E-149	5.44E-147
ENSG00000135318	NT5E	10981.11987	7225.574499	14736.66524	1.02812549	0.038508043	26.6989802	4.84E-157	6.02E-155
ENSG00000076344	RGS11	79.86401219	52.59959135	107.128433	1.027986628	0.324913153	3.16388124	0.001557	0.004085
ENSG00000258920	FOXN3-AS1	33.42021923	21.97927267	44.86116579	1.02741389	0.388751755	2.64285338	0.008221	0.019067
ENSG00000169241	SLC50A1	898.5992346	592.1276779	1205.070791	1.0271562	0.084265256	12.1895577	3.53E-34	5.56E-33
ENSG00000280145	CU638689.4	37.729132	24.93091461	50.52734938	1.026504937	0.377856134	2.71665548	0.006595	0.015546
ENSG00000123689	G0S2	118.5122199	78.02510739	158.9993325	1.026446436	0.208442465	4.92436335	8.46E-07	3.31E-06
ENSG00000114166	KAT2B	283.5082648	186.7043162	380.3122134	1.025974828	0.133534967	7.68319228	1.55E-14	1.07E-13
ENSG00000135046	ANXA1	13910.81679	9175.547736	18646.08585	1.023082442	0.038100215	26.8524059	7.91E-159	1.01E-156
ENSG00000135643	KCNMB4	232.5238142	153.3732966	311.6743317	1.022902213	0.147427746	6.93832907	3.97E-12	2.36E-11
ENSG00000050344	NFE2L3	3998.220761	2638.167769	5358.273732	1.022389899	0.044640113	22.9029416	4.34E-116	3.46E-114
ENSG00000175106	TVP23C	145.2491604	95.76165983	194.736661	1.022296029	0.185379097	5.51462405	3.50E-08	1.54E-07
ENSG00000185513	L3MBTL1	57.03607854	37.75329568	76.3188614	1.020414247	0.361918069	2.81946201	0.00481	0.011627
ENSG00000261051	AC107021.2	29.44285697	19.4123608	39.47335314	1.020302027	0.414694789	2.46036858	0.013879	0.030735
ENSG00000262454	MIR193BHG	32.5646653	21.53284161	43.59648898	1.019379544	0.447290151	2.27901182	0.022666	0.047887
ENSG00000205683	DPF3	499.1537436	330.1783682	668.129119	1.016185992	0.117875239	8.62086049	6.65E-18	5.53E-17
ENSG00000219626	FAM228B	125.7614687	83.14219766	168.3807397	1.015571133	0.20698539	4.90648705	9.27E-07	3.60E-06
ENSG00000120337	TNFSF18	797.6013026	528.37141	1066.831195	1.014827699	0.08922652	11.3736107	5.66E-30	7.69E-29
ENSG00000100307	CBX7	230.7772726	152.5709107	308.9836344	1.014658872	0.154360475	6.57330751	4.92E-11	2.72E-10
ENSG00000240891	PLCXD2	190.2225657	125.9391106	254.5060207	1.014482854	0.163359312	6.21013176	5.29E-10	2.71E-09
ENSG00000183091	NEB	52.74884832	34.9825174	70.51517924	1.012275417	0.335261917	3.01935701	0.002533	0.006421
ENSG00000169715	MT1E	2904.208649	1925.814628	3882.60267	1.011953024	0.052814902	19.1603691	7.93E-82	3.61E-80
ENSG00000165948	IFI27L1	252.9144985	167.8688704	337.9601266	1.011945134	0.143669459	7.04356475	1.87E-12	1.14E-11
ENSG00000128165	ADM2	50.53645989	33.36159947	67.7113203	1.011646232	0.356743143	2.83578326	0.004571	0.011093
ENSG00000183208	GDPGP1	43.39504575	28.81506441	57.9750271	1.011065342	0.340594489	2.96853112	0.002992	0.007498
ENSG00000232949	AC002480.2	31.34693716	20.81353589	41.88033843	1.010835607	0.412660068	2.44956003	0.014303	0.031559
ENSG00000114735	HEMK1	481.2561637	319.122813	643.3895144	1.010431376	0.13416459	7.5312821	5.02E-14	3.36E-13
ENSG00000268858	AL118506.1	307.4344602	204.1893731	410.6795474	1.009388046	0.135668516	7.44010529	1.01E-13	6.62E-13
ENSG00000125510	OPRL1	110.8704014	73.79966188	147.941141	1.007483578	0.234782081	4.29114341	1.78E-05	6.00E-05
ENSG00000152642	GPD1L	435.0299985	289.2425476	580.8174494	1.007456481	0.124465285	8.0942769	5.76E-16	4.33E-15
ENSG00000216937	CCDC7	43.12037269	28.61886558	57.62187979	1.006606699	0.339128807	2.96821349	0.002995	0.007504
ENSG00000273018	FAM106A	90.0664406	59.92736888	120.2055123	1.005842156	0.301422401	3.33698542	0.000847	0.002316
ENSG00000148180	GSN	8420.41783	5600.323942	11240.51172	1.005078755	0.036937009	27.2106157	4.86E-163	6.57E-161
ENSG00000265688	MAFG-DT	102.1601272	67.93787281	136.3823816	1.004591227	0.220160415	4.56299661	5.04E-06	1.80E-05
ENSG00000173200	PARP15	41.14068669	27.44370261	54.83767077	1.004193677	0.405253899	2.47793711	0.013214	0.029388
ENSG00000233461	AL445524.1	104.5145066	69.44900972	139.5800035	1.003436151	0.226476293	4.43064543	9.40E-06	3.26E-05
ENSG00000167861	HID1	972.7736505	647.5544266	1297.992874	1.002914086	0.077476144	12.9448116	2.51E-38	4.48E-37
ENSG00000140465	CYP1A1	567.0024206	377.5608304	756.4440109	1.002405609	0.110185582	9.09742987	9.25E-20	8.38E-19
ENSG00000011478	QPCTL	782.0469614	520.7656613	1043.328262	1.002176467	0.082838066	12.0980186	1.08E-33	1.68E-32
ENSG00000245937	LINC01184	551.5901774	367.4695819	735.7107729	1.002122052	0.098480042	10.1758898	2.54E-24	2.79E-23
ENSG00000151575	TEX9	40.40467504	26.94445552	53.86489457	1.000796768	0.349357555	2.86467762	0.004174	0.010196
ENSG00000173221	GLRX	361.9671294	241.2146589	482.7195999	1.000501736	0.121640863	8.22504633	1.95E-16	1.51E-15
ENSG00000168496	FEN1	1623.953411	2165.911405	1081.995417	-1.000227041	0.067091319	-14.908442	2.90E-50	7.05E-49
ENSG00000137573	SULF1	2629.354445	3506.525301	1752.18359	-1.000459337	0.053100412	-18.840896	3.49E-79	1.53E-77
ENSG00000198408	OGA	3313.799097	4419.830466	2207.767729	-1.001496238	0.050359946	-19.886762	5.30E-88	2.71E-86

ENSG00000000460	C1orf112	306.9742655	409.4470152	204.5015157	-1.001504808	0.128953107	-7.7664264	8.07E-15	5.68E-14
ENSG00000144749	LRIG1	5100.034441	6802.048838	3398.020044	-1.001593264	0.040377939	-24.805458	7.83E-136	7.89E-134
ENSG00000198464	ZNF480	586.9220288	783.3507903	390.4932673	-1.004022757	0.09887557	-10.154407	3.17E-24	3.46E-23
ENSG00000185972	CCIN	35.28002508	47.1244274	23.43562276	-1.005263556	0.388986743	-2.5843131	0.009757	0.022293
ENSG00000205502	C2CD4B	1481.298331	1978.006348	984.5903137	-1.006332239	0.158741455	-6.3394419	2.31E-10	1.21E-09
ENSG00000254726	MEX3A	1806.142955	2412.866297	1199.419613	-1.008558906	0.060181822	-16.758531	4.91E-63	1.60E-61
ENSG00000162551	ALPL	171.5002405	229.045145	113.955336	-1.008708474	0.19186943	-5.2572652	1.46E-07	6.15E-07
ENSG00000139508	SLC46A3	381.6032882	509.7206607	253.4859157	-1.009028183	0.123400856	-8.1768329	2.91E-16	2.23E-15
ENSG00000282936	AC004706.3	49.28371409	65.85442209	32.71300608	-1.009407621	0.328288846	-3.0747545	0.002107	0.005415
ENSG00000237440	AC008554.1	153.7326293	205.4741376	101.9911209	-1.010918115	0.183717295	-5.5025746	3.74E-08	1.65E-07
ENSG00000237181	AC147651.4	56.07927648	74.95128084	37.20727212	-1.012733139	0.302190867	-3.3513029	0.000804	0.002205
ENSG00000104356	POP1	733.1430395	980.3725925	485.9134866	-1.013016762	0.086131527	-11.761277	6.18E-32	9.01E-31
ENSG00000122644	ARL4A	1284.366255	1717.90519	850.8273201	-1.013249801	0.070147177	-14.444627	2.71E-47	6.05E-46
ENSG00000128487	SPECC1	926.3964804	1238.993107	613.7998535	-1.013872763	0.081630843	-12.420217	2.03E-35	3.31E-34
ENSG00000101331	CCM2L	70.84044512	94.71269541	46.96819482	-1.014820965	0.286809198	-3.5383139	0.000403	0.001154
ENSG00000162772	ATF3	194.8261338	260.768463	128.8838046	-1.016239841	0.174319912	-5.8297404	5.55E-09	2.63E-08
ENSG00000108576	SLC6A4	39.61952855	53.0324918	26.20656529	-1.017937316	0.353637867	-2.8784737	0.003996	0.009795
ENSG00000130038	CRACR2A	380.2376144	509.2944359	251.1807929	-1.019031417	0.118748727	-8.5814092	9.37E-18	7.74E-17
ENSG00000010292	NCAPD2	2409.232974	3226.782158	1591.68379	-1.019308375	0.052832067	-19.293365	6.11E-83	2.84E-81
ENSG00000071073	MGAT4A	2483.212979	3325.882396	1640.543562	-1.019613498	0.050824995	-20.061261	1.61E-89	8.47E-88
ENSG00000213096	ZNF254	442.3907743	592.7915518	291.9899967	-1.022029575	0.120983639	-8.4476677	2.97E-17	2.39E-16
ENSG00000259583	AC015712.2	79.7381824	106.8807651	52.59559973	-1.022612127	0.255152319	-4.0078496	6.13E-05	0.000194
ENSG00000163840	DTX3L	1972.013899	2644.212788	1299.815011	-1.023572659	0.074436312	-13.750986	5.02E-43	1.02E-41
ENSG00000141562	NARF	1798.230364	2411.680898	1184.779831	-1.025150959	0.059580604	-17.206119	2.39E-66	8.26E-65
ENSG00000134326	CMPK2	89.61230082	120.2931409	58.93146073	-1.025151917	0.248405242	-4.1269335	3.68E-05	0.00012
ENSG00000145246	ATP10D	2224.034367	2985.207892	1462.860842	-1.028302145	0.058875662	-17.465657	2.62E-68	9.55E-67
ENSG00000187764	SEMA4D	201.6746632	270.7370317	132.6122947	-1.028852929	0.168152948	-6.1185542	9.44E-10	4.75E-09
ENSG00000185252	ZNF74	277.2135399	371.8869868	182.5400931	-1.029008573	0.143319498	-7.1798226	6.98E-13	4.37E-12
ENSG00000138696	BMPR1B	254.5048848	341.8033057	167.2064639	-1.030179471	0.145089416	-7.1003075	1.24E-12	7.67E-12
ENSG00000272667	AC012306.2	91.23146205	122.4978287	59.96509537	-1.030863302	0.245198224	-4.2042038	2.62E-05	8.69E-05
ENSG00000156802	ATAD2	1604.034169	2155.16156	1052.906779	-1.032627708	0.06295655	-16.402228	1.84E-60	5.66E-59
ENSG00000164087	POC1A	244.1579301	328.1896791	160.1261811	-1.033785302	0.148375807	-6.9673441	3.23E-12	1.93E-11
ENSG00000251247	ZNF345	42.98311523	57.76268713	28.20354334	-1.034179681	0.334509236	-3.0916327	0.001991	0.005134
ENSG00000078967	UBE2D4	254.4651952	342.1281378	166.8022525	-1.034832412	0.153126337	-6.7580302	1.40E-11	8.02E-11
ENSG00000126822	PLEKHG3	206.5118754	277.4366559	135.587095	-1.035800959	0.167924207	-6.1682647	6.90E-10	3.50E-09
ENSG00000102226	USP11	1356.726467	1823.70298	889.7499532	-1.035830116	0.065659985	-15.775668	4.58E-56	1.27E-54
ENSG00000127586	CHTF18	411.603748	553.3510807	269.8564152	-1.036022791	0.356920679	-2.9026696	0.0037	0.009116
ENSG00000107731	UNC5B	326.9168943	439.3864564	214.4473322	-1.037640754	0.13539659	-7.6637141	1.81E-14	1.24E-13
ENSG00000166432	ZMAT1	47.99353534	64.50782165	31.47924903	-1.037826146	0.332080405	-3.1252255	0.001777	0.004613
ENSG00000119865	CNRIP1	1612.524642	2168.896828	1056.152455	-1.038318775	0.067419616	-15.400841	1.62E-53	4.32E-52
ENSG00000143458	GABPB2	165.480981	222.7143745	108.2475875	-1.038402487	0.185248126	-5.6054682	2.08E-08	9.37E-08
ENSG00000187605	TET3	1142.48338	1537.066494	747.9002662	-1.039754459	0.072875183	-14.267607	3.48E-46	7.60E-45
ENSG00000260136	AC008915.2	47.650397	64.14383898	31.15695502	-1.040748674	0.328398775	-3.1691613	0.001529	0.004016
ENSG00000179051	RCC2	7032.934434	9465.695906	4600.172962	-1.041052161	0.0389977	-26.695219	5.35E-157	6.61E-155
ENSG00000165490	DDIAS	522.7948993	703.75068	341.8391186	-1.042215847	0.10122158	-10.29638	7.32E-25	8.26E-24
ENSG00000165304	MELK	936.5623923	1261.094339	612.0304455	-1.042452581	0.079988939	-13.032459	8.00E-39	1.45E-37
ENSG00000262903	AC027796.4	60.9325318	82.05769192	39.80737168	-1.042469536	0.32834144	-3.1749557	0.001499	0.003944
ENSG00000114405	C3orf14	157.7092209	212.4411798	102.977262	-1.042804722	0.184948879	-5.6383403	1.72E-08	7.80E-08
ENSG00000132475	H3F3B	9406.756405	12668.30849	6145.204316	-1.043922371	0.039419503	-26.482383	1.55E-154	1.86E-152
ENSG00000128606	LRRC17	798.9223406	1076.259864	521.5848174	-1.044349161	0.088554443	-11.7933	4.23E-32	6.22E-31
ENSG00000109458	GAB1	689.9062903	929.8691715	449.9434091	-1.045502093	0.096115629	-10.877545	1.47E-27	1.83E-26
ENSG00000258976	AC013451.2	25.14258279	33.91551169	16.3696539	-1.045629709	0.460552006	-2.2703836	0.023184	0.048841
ENSG00000164109	MAD2L1	531.0160535	715.608486	346.4236209	-1.046539871	0.11543335	-9.066183	1.23E-19	1.11E-18
ENSG00000111247	RAD51AP1	257.1523491	346.587444	167.7172542	-1.047500382	0.139134888	-7.528668	5.13E-14	3.43E-13
ENSG00000255031	AP002807.1	77.82634541	104.9583322	50.69435865	-1.047504747	0.293663296	-3.5670265	0.000361	0.001042
ENSG00000117632	STMN1	4565.584279	6155.245024	2975.923535	-1.048660748	0.048166618	-21.771525	4.32E-105	2.97E-103
ENSG00000146592	CREB5	388.8404381	524.2958419	253.3850344	-1.048750103	0.113750361	-9.2197518	2.98E-20	2.73E-19
ENSG00000177169	ULK1	1429.00817	1926.89805	931.1182891	-1.048836974	0.064033044	-16.379621	2.67E-60	8.20E-59
ENSG00000119397	CNTRL	613.5343878	827.3569204	399.7118553	-1.049068169	0.101059344	-10.380714	3.03E-25	3.47E-24
ENSG00000138074	SLC5A6	1184.570707	1597.494826	771.6465887	-1.04954587	0.068040623	-15.425283	1.11E-53	2.96E-52
ENSG00000156876	SASS6	176.9650506	238.7468384	115.1832627	-1.049578066	0.189581167	-5.5362992	3.09E-08	1.37E-07
ENSG00000077044	DGKD	574.3506919	774.5214369	374.179947	-1.050208045	0.103314543	-10.165152	2.84E-24	3.11E-23
ENSG00000186281	GPAT2	198.9985221	268.4175555	129.5794886	-1.051207883	0.15991872	-6.5733885	4.92E-11	2.72E-10
ENSG00000129646	QRICH2	86.73777779	117.1349264	56.34062915	-1.051685828	0.264882253	-3.9703899	7.18E-05	0.000226
ENSG00000269834	ZNF528-AS1	79.75628012	107.5593871	51.95317319	-1.051761864	0.251677947	-4.1789989	2.93E-05	9.66E-05
ENSG00000117877	CD3EAP	799.4330488	1078.890554	519.9755434	-1.051965339	0.089697183	-11.727964	9.16E-32	1.33E-30
ENSG00000120800	UTP20	2280.787359	3078.730042	1482.844676	-1.054118386	0.052791951	-19.967407	1.06E-88	5.48E-87
ENSG00000107796	ACTA2	197.5612576	266.6097745	128.5127408	-1.055277863	0.174970495	-6.0311761	1.63E-09	8.01E-09
ENSG00000146918	NCAPG2	1146.351153	1549.19438	743.5079264	-1.057491508	0.086916691	-12.166725	4.67E-34	7.34E-33
ENSG00000165259	HDX	335.3412566	453.3892334	217.2932798	-1.058842077	0.140563957	-7.5328136	4.97E-14	3.33E-13
ENSG00000137727	ARHGAP20	74.41019635	100.5968555	48.2235372	-1.060209602	0.290081466	-3.6548685	0.000257	0.000756
ENSG00000100629	CEP128	251.487202	340.0275933	162.9468106	-1.061016592	0.142452548	-7.4482107	9.46E-14	6.24E-13
ENSG00000088448	ANKRD10	563.5087524	761.8962481	365.1212566	-1.062196916	0.109656849	-9.6865534	3.44E-22	3.47E-21
ENSG00000065534	MYLK	997.5175774	1348.833226	646.2019286	-1.062263165	0.075024729	-14.15884	1.65E-45	3.54E-44
ENSG00000123473	STIL	669.9643336	906.2192772	433.70939	-1.062300071	0.094346429	-11.259568	2.08E-29	2.77E-28
ENSG00000203727	SAMD5	670.4744017	906.9008752	434.0479282	-1.063205837	0.092788505	-11.458379	2.13E-30	2.95E-29
ENSG00000189057	FAM111B	797.1947113	1078.644155	515.7452679	-1.063434227	0.090404223	-11.763103	6.05E-32	8.83E-31
ENSG00000280287	AC131212.3	80.19262567	108.466502	51.91874937	-1.063694212	0.284477676	-3.7391131	0.000185	0.000553
ENSG00000161692	DBF4B	263.0642305	355.7552949	170.3731661	-1.064019213	0.146132947	-7.2811726	3.31E-13	2.12E-12
ENSG00000196584	XRCC2	235.7004761	318.8185474	152.5824048	-1.064108581	0.149644915	-7.1108904	1.15E-12	7.12E-12
ENSG00000168237	GLYCTK	200.1104239	270.5644213	129.6564264	-1.064564263	0.17494105	-6.0852742	1.16E-09	5.80E-09
ENSG00000111877	MCM9	315.9079634	427.451976	204.3639507	-1.066059999	0.133206613	-8.0030561	1.21E-15	8.95E-15
ENSG00000197261	C6orf141	218.766284	296.0648531	141.467715	-1.06657661	0.17052298	-6.2547383	3.98E-10	2.06E-09
ENSG00000107443	CCNJ	1225.919088	1660.215253	791.6229227	-1.068069397	0.068252324	-15.648836	3.38E-55	9.33E-54
ENSG00000123080	CDKN2C	181.2708748	245.4456517	117.096098	-1.068321565	0.170712765	-6.2580063	3.90E-10	2.02E-09

ENSG00000272221	AL645933.2	56.53566786	76.69478964	36.37654608	-1.070289388	0.333236403	-3.2118021	0.001319	0.003509
ENSG00000187824	TMEM220	59.19558015	80.17531102	38.21584929	-1.070454665	0.287058814	-3.729043	0.000192	0.000574
ENSG00000176209	SMIM19	169.7570979	230.1002898	109.413906	-1.070874786	0.176280482	-6.0748347	1.24E-09	6.17E-09
ENSG00000154237	LRRK1	1859.957175	2520.195477	1199.718873	-1.070896619	0.066739626	-16.045889	6.11E-58	1.77E-56
ENSG00000090447	TFAP4	147.631675	199.994417	95.26893303	-1.071331534	0.19531525	-5.4851402	4.13E-08	1.82E-07
ENSG00000110446	SLC15A3	272.1929412	369.001601	175.3842814	-1.072368574	0.15119975	-7.0923965	1.32E-12	8.10E-12
ENSG00000058091	CDK14	1260.119815	1708.161417	812.0782125	-1.072695744	0.077067551	-13.918903	4.86E-44	1.01E-42
ENSG00000171914	TLN2	75.43250672	102.2646805	48.60033294	-1.073948521	0.254451508	-4.2206412	2.44E-05	8.11E-05
ENSG00000119392	GLE1	721.7808879	979.1273848	464.434391	-1.075833754	0.085752853	-12.545749	4.19E-36	6.99E-35
ENSG00000065328	MCM10	545.9347253	740.9678794	350.9015711	-1.077411825	0.123424862	-8.7292934	2.56E-18	2.17E-17
ENSG00000138182	KIF20B	777.3675453	1055.208547	499.5265438	-1.077619814	0.093205372	-11.561778	6.44E-31	9.03E-30
ENSG00000185278	ZBTB37	187.671628	254.7495797	120.5936763	-1.078112455	0.163007962	-6.6138638	3.74E-11	2.09E-10
ENSG00000221843	C2orf16	34.21633831	46.45956133	21.97311529	-1.081953448	0.395913761	-2.7328008	0.00628	0.01486
ENSG00000185010	F8	31.07767001	42.17579706	19.97954295	-1.082328926	0.430796446	-2.5123906	0.011992	0.026873
ENSG00000145476	CYP4V2	43.97810977	59.67573836	28.28048118	-1.082597634	0.386162649	-2.8034758	0.005056	0.012164
ENSG00000181450	ZNF678	156.1182864	212.0525247	100.1840482	-1.082764582	0.215461427	-5.0253291	5.03E-07	2.01E-06
ENSG00000101224	CDC25B	1265.158436	1719.595476	810.721396	-1.084798713	0.067182297	-16.147092	1.19E-58	3.53E-57
ENSG00000123104	ITPR2	6562.282039	8921.225017	4203.339061	-1.085731647	0.039729316	-27.328224	1.96E-164	2.71E-162
ENSG00000189403	HMGB1	8657.837996	11773.73044	5541.945549	-1.08711659	0.047829068	-22.729203	2.30E-114	1.80E-112
ENSG00000196632	WNK3	82.55449692	112.1876944	52.92129944	-1.087722803	0.267685339	-4.0634381	4.84E-05	0.000155
ENSG00000026103	FAS	179.4240893	243.9969378	114.8512408	-1.088395007	0.183672973	-5.9257222	3.11E-09	1.50E-08
ENSG00000120802	TMPO	2441.176136	3321.157302	1561.194971	-1.088719996	0.054340223	-20.035251	2.71E-89	1.42E-87
ENSG00000138600	SPPL2A	2544.176628	3461.997508	1626.355748	-1.090244141	0.052400603	-20.805946	3.82E-96	2.26E-94
ENSG00000163006	CCDC138	163.2803498	222.3046117	104.256088	-1.091353291	0.179031794	-6.095863	1.09E-09	5.44E-09
ENSG00000188368	PRR19	32.85588401	44.76268977	20.94907825	-1.091621904	0.392625303	-2.7803147	0.005431	0.013002
ENSG00000005339	CREBBP	2935.311485	3996.510118	1874.112851	-1.092773371	0.049334019	-22.150504	1.03E-108	7.30E-107
ENSG00000169087	HSPBAP1	126.1602385	171.9397928	80.3806841	-1.09288108	0.21873069	-4.9964689	5.84E-07	2.32E-06
ENSG00000131037	EPS8L1	236.9300256	322.5632838	151.2967674	-1.094011042	0.153245399	-7.1389487	9.40E-13	5.85E-12
ENSG00000108932	SLC16A6	54.99223752	74.84528846	35.13918659	-1.097581575	0.337887356	-3.2483653	0.001161	0.003117
ENSG00000170323	FABP4	5288.542382	7208.755295	3368.32947	-1.097868905	0.045610424	-24.07057	5.08E-128	4.68E-126
ENSG00000171016	PYGO1	462.1043154	629.7311404	294.4774904	-1.098089314	0.112492385	-9.7614547	1.65E-22	1.68E-21
ENSG00000213347	MXD3	123.8947354	168.7707079	79.0187629	-1.098320264	0.225302293	-4.8748739	1.09E-06	4.21E-06
ENSG00000133101	CCNA1	37.05630343	50.52412401	23.58848284	-1.098557413	0.388796784	-2.8255311	0.00472	0.011425
ENSG00000149554	CHEK1	990.9268947	1350.941665	630.9121242	-1.098961867	0.075624399	-14.531843	7.61E-48	1.72E-46
ENSG00000196118	CCDC189	69.50704697	94.83587204	44.17822189	-1.100450721	0.348725921	-3.1556321	0.001602	0.004196
ENSG00000253731	PCDHGA6	111.5931082	152.143378	71.04283841	-1.101803224	0.22446491	-4.9085767	9.17E-07	3.57E-06
ENSG00000168685	IL7R	680.3154199	928.5229313	432.1079085	-1.102532994	0.090677814	-12.158796	5.15E-34	8.07E-33
ENSG00000145779	TNFAIP8	363.1238206	495.6951081	230.5525331	-1.1032836	0.12161827	-9.0716929	1.17E-19	1.05E-18
ENSG00000077943	ITGA8	87.32274134	119.1592918	55.48619084	-1.106446895	0.25729011	-4.3003864	1.71E-05	5.77E-05
ENSG00000140525	FANCI	1179.194414	1610.961327	747.4275022	-1.107035364	0.073811045	-14.998234	7.54E-51	1.86E-49
ENSG00000102935	ZNF423	58.30288153	79.6733578	36.93240526	-1.107575183	0.303424131	-3.6502541	0.000262	0.000769
ENSG00000086991	NOX4	2213.386414	3024.002888	1402.76994	-1.107788532	0.05369525	-20.631034	1.45E-94	8.24E-93
ENSG00000123572	NRK	330.3726758	451.6053582	209.1399934	-1.109915865	0.130924741	-8.4775105	2.30E-17	1.86E-16
ENSG00000170873	MTSS1	1718.063637	2348.097326	1088.029947	-1.110261128	0.065113491	-17.051169	3.43E-65	1.16E-63
ENSG00000185291	IL3RA	720.129884	984.4688175	455.7909505	-1.111745087	0.086590917	-12.83905	9.91E-38	1.75E-36
ENSG00000136492	BRIP1	399.9680307	546.9053867	253.0306747	-1.112317258	0.118360586	-9.3976998	5.58E-21	5.32E-20
ENSG00000278053	DDX52	2074.345532	2836.883668	1311.807397	-1.112404824	0.054972146	-20.235791	4.74E-91	2.55E-89
ENSG00000134253	TRIM45	34.61539696	47.34074191	21.890052	-1.112893073	0.414041869	-2.6878757	0.007191	0.016817
ENSG00000129534	MIS18BP1	927.9529349	1269.050224	586.8556459	-1.113321547	0.080060913	-13.905931	5.83E-44	1.21E-42
ENSG00000206417	H1FX-AS1	34.91460165	47.70332628	22.12587701	-1.113695648	0.402715697	-2.7654637	0.005684	0.01356
ENSG00000116001	TIA1	1709.979649	2340.694047	1079.26525	-1.11678577	0.074200919	-15.050835	3.41E-51	8.51E-50
ENSG00000130305	NSUN5	132.7675558	181.8276257	83.70748581	-1.117215509	0.196612838	-5.6823121	1.33E-08	6.09E-08
ENSG00000084636	COL16A1	348.1503899	476.7303512	219.5704287	-1.118312166	0.142433632	-7.8514614	4.11E-15	2.95E-14
ENSG00000235505	CASP17P	340.8971423	466.6812735	215.1130111	-1.118410151	0.141772883	-7.8887452	3.05E-15	2.20E-14
ENSG00000150048	CLEC1A	466.9874139	639.4477168	294.5271109	-1.119509846	0.105971473	-10.564257	4.36E-26	5.13E-25
ENSG00000163545	NUAK2	184.4598515	252.5717276	116.3479754	-1.120599438	0.189853007	-5.9024582	3.58E-09	1.72E-08
ENSG00000196739	COL27A1	3597.038293	4929.484871	2264.591715	-1.122133047	0.188937468	-5.9391769	2.86E-09	1.39E-08
ENSG00000177337	DLGAP1-AS1	133.9582248	183.5917914	84.32465814	-1.122582802	0.226907366	-4.9473176	7.52E-07	2.95E-06
ENSG00000081803	CADPS2	1238.959663	1699.086431	778.8328947	-1.124094314	0.077211171	-14.5587	5.14E-48	1.17E-46
ENSG00000196693	ZNF33B	567.7817491	778.2862001	357.277298	-1.124594716	0.100206453	-11.222777	3.15E-29	4.18E-28
ENSG00000100592	DAAM1	2379.469113	3263.613272	1495.324953	-1.125226901	0.055730651	-20.19045	1.19E-90	6.34E-89
ENSG00000171208	NETO2	1376.569372	1888.341061	864.7976828	-1.126026965	0.067729635	-16.625322	4.57E-62	1.45E-60
ENSG00000254837	AP001372.2	46.91476783	64.40638446	29.42315121	-1.126387892	0.346834972	-3.2476191	0.001164	0.003124
ENSG00000139192	TAPBPL	349.4072327	479.42955	219.3849154	-1.126551084	0.124148229	-9.0742421	1.14E-19	1.03E-18
ENSG00000169857	AVEN	475.9032425	652.8156663	298.9908187	-1.127173185	0.112963373	-9.9782182	1.90E-23	2.01E-22
ENSG00000176714	CCDC121	54.14745514	74.24726578	34.0476445	-1.127245884	0.327274786	-3.4443408	0.000572	0.001604
ENSG00000135821	GLUL	3261.636138	4475.72868	2047.543596	-1.128621001	0.058356544	-19.340093	2.47E-83	1.16E-81
ENSG00000188312	CENPP	111.2744977	152.6557049	69.89329052	-1.128991978	0.225132255	-5.0147944	5.31E-07	2.12E-06
ENSG00000228794	LINC01128	234.4874208	321.7624313	147.2124103	-1.129770335	0.153063247	-7.3810687	1.57E-13	1.02E-12
ENSG00000115526	CHST10	537.3047141	737.7739888	336.8354395	-1.131028838	0.101120341	-11.184978	4.83E-29	6.35E-28
ENSG00000035499	DEPDC1B	158.2466281	217.2316778	99.26157845	-1.131444823	0.185460921	-6.1007182	1.06E-09	5.28E-09
ENSG00000163507	CIP2A	578.4764581	794.6101659	362.3427502	-1.131926589	0.096842363	-11.688341	1.46E-31	2.09E-30
ENSG00000254245	PCDHGA3	40.89076238	56.16995888	25.61156589	-1.132969151	0.367571334	-3.082311	0.002054	0.005287
ENSG00000197256	KANK2	2600.176613	3571.877814	1628.475411	-1.132975219	0.065522447	-17.291406	5.46E-67	1.92E-65
ENSG00000152503	TRIM36	24.90913864	34.20958455	15.60869274	-1.133204183	0.460898911	-2.4586827	0.013945	0.030847
ENSG00000163535	SGO2	560.2768629	769.7641359	350.7895899	-1.133546901	0.105533259	-10.741134	6.52E-27	7.94E-26
ENSG00000105122	RASAL3	56.43671306	77.54931382	35.3241123	-1.134061486	0.30838501	-3.6774209	0.000236	0.000695
ENSG00000171621	SPSB1	3344.26775	4595.948704	2092.586795	-1.134694263	0.050919915	-22.2839	5.29E-110	3.82E-108
ENSG00000159314	ARHGAP27	590.7979095	812.1366286	369.4591904	-1.136097784	0.100928475	-11.256464	2.15E-29	2.87E-28
ENSG00000123810	B9D2	144.2309785	198.3816634	90.08029364	-1.137305399	0.198290611	-5.7355484	9.72E-09	4.51E-08
ENSG00000137968	SLC44A5	78.69872794	108.094266	49.30318993	-1.137374329	0.292430968	-3.8893772	0.000101	0.00031
ENSG00000168763	CNNM3	440.4364074	605.5017602	275.3710546	-1.137395604	0.111887652	-10.165515	2.83E-24	3.10E-23
ENSG00000183117	CSMD1	181.0912385	249.1621541	113.0203229	-1.137669604	0.180506459	-6.3026531	2.93E-10	1.53E-09
ENSG00000110066	KMT5B	1225.748988	1685.94923	765.5487461	-1.139135862	0.066987172	-17.005284	7.50E-65	2.52E-63
ENSG00000272086	AC025181.2	23.99298219	33.05864168	14.9273227	-1.141252678	0.502455578	-2.2713504	0.023126	0.048739

ENSG00000106852	LHX6	116.4649637	160.3084052	72.6215223	-1.141531006	0.23078166	-4.946368	7.56E-07	2.97E-06
ENSG00000179922	ZNF784	168.1979132	231.4346215	104.9612048	-1.141826404	0.184387461	-6.1925383	5.92E-10	3.02E-09
ENSG00000166224	SGPL1	1546.59807	2128.806917	964.3892226	-1.142507971	0.065924266	-17.330613	2.76E-67	9.80E-66
ENSG00000197275	RAD54B	26.34291781	36.28893084	16.39690477	-1.143029283	0.446258342	-2.5613623	0.010426	0.023683
ENSG00000265982	AC103810.3	29.34540233	40.42619993	18.26460472	-1.143770318	0.451756925	-2.5318269	0.011347	0.025552
ENSG00000134107	BHLHE40	2250.71815	3099.221631	1402.214669	-1.14421928	0.052103127	-21.960664	6.85E-107	4.80E-105
ENSG00000188211	NCR3LG1	440.0103181	605.9786821	274.041954	-1.144357095	0.129046255	-8.8678056	7.46E-19	6.46E-18
ENSG00000102174	PHEX	84.9788378	116.9923547	52.96532087	-1.146857157	0.259941791	-4.4119768	1.02E-05	3.54E-05
ENSG00000263528	IKBKE	1916.56145	2640.857924	1192.264976	-1.147314321	0.055716041	-20.592172	3.23E-94	1.83E-92
ENSG00000152760	TCTEX1D1	107.5968037	148.2290991	66.96450833	-1.14862153	0.218038452	-5.2679769	1.38E-07	5.82E-07
ENSG00000101447	FAM83D	465.3372869	641.4807418	289.1938319	-1.148686799	0.10982883	-10.458882	1.33E-25	1.55E-24
ENSG00000203326	ZNF525	71.43139494	98.55512507	44.30766481	-1.149830928	0.273732914	-4.2005578	2.66E-05	8.82E-05
ENSG00000160957	RECQL4	490.2064783	676.0068163	304.4061402	-1.15017835	0.121770301	-9.4454751	3.54E-21	3.41E-20
ENSG00000271936	AC012073.1	28.701203	39.62841526	17.77399075	-1.150717761	0.44367891	-2.5935823	0.009498	0.021746
ENSG00000099956	SMARCB1	2172.435894	2995.841099	1349.030689	-1.151185077	0.054217814	-21.232599	4.77E-100	3.08E-98
ENSG00000171962	DRC3	23.78727985	32.8629841	14.7115756	-1.15361266	0.468340731	-2.463191	0.013771	0.030515
ENSG00000253958	CLDN23	22.81394777	31.51367754	14.11421801	-1.156875916	0.468073875	-2.4715669	0.013452	0.029866
ENSG00000273038	AL365203.2	732.0694041	1010.811506	453.327302	-1.157450179	0.088477145	-13.081911	4.18E-39	7.61E-38
ENSG00000175354	PTPN2	464.6805491	641.7683199	287.5927784	-1.158613243	0.1073564	-10.792214	3.75E-27	4.59E-26
ENSG00000168961	LGALS9	669.1508337	924.6852822	413.6163852	-1.160134597	0.101041823	-11.481727	1.63E-30	2.26E-29
ENSG00000120051	CFAP58	42.67678758	58.94908084	26.40449431	-1.16048594	0.36026707	-3.2211824	0.001277	0.003401
ENSG00000115129	TP53I3	1780.60553	2461.387932	1099.823128	-1.162131573	0.059199106	-19.630897	8.42E-86	4.13E-84
ENSG00000167900	TK1	968.5668407	1339.245691	597.8879903	-1.163527052	0.079293096	-14.67375	9.49E-49	2.21E-47
ENSG00000259781	HMGB1P6	253.0864932	349.9531785	156.2198079	-1.163695969	0.147678459	-7.8799304	3.28E-15	2.36E-14
ENSG00000166508	MCM7	2724.410549	3768.207913	1680.613186	-1.164334563	0.05674964	-20.517039	1.52E-93	8.40E-92
ENSG00000142733	MAP3K6	763.8759867	1056.661589	471.0903845	-1.164348216	0.086506087	-13.459726	2.70E-41	5.27E-40
ENSG00000132003	ZSWIM4	1446.594122	2003.736808	889.4514354	-1.172279421	0.078176756	-14.995243	7.89E-51	1.95E-49
ENSG00000078687	TNRC6C	645.4695619	894.3258409	396.6132828	-1.173184258	0.091406426	-12.834812	1.05E-37	1.84E-36
ENSG00000184162	NR2C2AP	251.8638991	349.3036017	154.4241965	-1.175423835	0.163845183	-7.1739908	7.28E-13	4.56E-12
ENSG00000128284	APOL3	2142.92613	2970.589275	1315.262985	-1.175706957	0.055450058	-21.202989	8.96E-100	5.77E-98
ENSG00000005302	MSL3	2264.114704	3139.514143	1388.715265	-1.176961799	0.0560083	-21.01406	4.88E-98	3.06E-96
ENSG00000071246	VASH1	9503.717117	13180.81279	5826.621449	-1.177921887	0.033516184	-35.144869	1.39E-270	3.68E-268
ENSG00000158195	WASF2	12418.60656	17224.43768	7612.775448	-1.178026261	0.030763518	-38.292963	0	0
ENSG00000010319	SEMA3G	3707.318091	5142.735696	2271.900487	-1.178626113	0.054067967	-21.798972	2.37E-105	1.65E-103
ENSG00000110848	CD69	525.544756	729.3510604	321.7384516	-1.179241752	0.111052049	-10.61882	2.44E-26	2.90E-25
ENSG00000167562	ZNF701	199.0678868	276.234973	121.9008006	-1.179633475	0.167030132	-7.0623992	1.64E-12	9.98E-12
ENSG00000186638	KIF24	190.66199	264.5269555	116.7970245	-1.180239787	0.206473574	-5.7161784	1.09E-08	5.03E-08
ENSG00000139618	BRCA2	528.1553752	732.949527	323.3612234	-1.18051618	0.101275802	-11.656449	2.13E-31	3.03E-30
ENSG00000074219	TEAD2	2966.434312	4116.157515	1816.711108	-1.180596639	0.05501538	-21.459393	3.73E-102	2.44E-100
ENSG00000164989	CCDC171	55.06954074	76.43269494	33.70638655	-1.182677002	0.321993604	-3.6729829	0.00024	0.000707
ENSG00000101311	FERMT1	82.55071959	114.6166357	50.48480345	-1.186331127	0.256372137	-4.6273793	3.70E-06	1.34E-05
ENSG00000077063	CTTNBP2	51.06690264	70.86899266	31.26481262	-1.188909617	0.363690858	-3.2690116	0.001079	0.002912
ENSG00000172936	MYD88	1999.100427	2780.223528	1217.977325	-1.191090938	0.055247844	-21.559048	4.35E-103	2.91E-101
ENSG00000156650	KAT6B	574.5688507	799.4694914	349.6682101	-1.193368705	0.10108502	-11.805594	3.65E-32	5.38E-31
ENSG00000277117	FP565260.3	928.8004151	1292.953731	564.647099	-1.194699596	0.082303039	-14.515862	9.61E-48	2.16E-46
ENSG00000093009	CDC45	492.8856373	686.4525309	299.3187438	-1.195100711	0.113367957	-10.541786	5.54E-26	6.50E-25
ENSG00000171617	ENC1	2691.495303	3746.842492	1636.148114	-1.195158749	0.051792992	-23.075685	8.13E-118	6.59E-116
ENSG00000134057	CCNB1	1324.737628	1844.258436	805.2168193	-1.195334339	0.069048341	-17.311558	3.85E-67	1.36E-65
ENSG00000185614	INKA1	178.8026537	248.8291116	108.7761958	-1.195395534	0.171829602	-6.9568661	3.48E-12	2.08E-11
ENSG00000269743	SLC25A53	123.2157888	171.4837087	74.94786896	-1.196630479	0.212612307	-5.6282277	1.82E-08	8.25E-08
ENSG00000144589	STK11IP	451.7264029	628.9160796	274.5367261	-1.197852491	0.119457462	-10.02744	1.15E-23	1.23E-22
ENSG00000131153	GINS2	406.4681662	566.5451119	246.3912205	-1.200372437	0.118581583	-10.122756	4.38E-24	4.76E-23
ENSG00000244694	PTCHD4	198.0279504	275.9183482	120.1375526	-1.200634252	0.170249876	-7.0521887	1.76E-12	1.07E-11
ENSG00000172197	MBOAT1	36.68771697	51.08840993	22.28702401	-1.200860373	0.419860704	-2.8601399	0.004235	0.010335
ENSG00000183814	LIN9	195.7544925	273.3186916	118.1902934	-1.206880464	0.172439717	-6.9988544	2.58E-12	1.55E-11
ENSG00000160602	AC010761.1	67.23451769	93.88036144	40.58867393	-1.207589497	0.285685968	-4.2269822	2.37E-05	7.90E-05
ENSG00000166801	FAM111A	1353.968368	1890.344094	817.5926422	-1.208460374	0.080673096	-14.97972	9.96E-51	2.45E-49
ENSG00000111057	KRT18	2275.577942	3176.88997	1374.265914	-1.208798452	0.057945202	-20.861062	1.21E-96	7.29E-95
ENSG00000163590	PPM1L	40.02070583	55.84020971	24.20120196	-1.208923738	0.366855671	-3.2953661	0.000983	0.002669
ENSG00000228716	DHFR	845.4266471	1180.348022	510.5052719	-1.209628003	0.084336635	-14.342854	1.18E-46	2.60E-45
ENSG00000110002	VWA5A	845.7051488	1180.813577	510.5967205	-1.209797501	0.083319404	-14.519997	9.05E-48	2.04E-46
ENSG00000224152	AC009506.1	37.20271897	51.98546694	22.419971	-1.210273163	0.412007155	-2.9375052	0.003309	0.008215
ENSG00000231365	WARS2-AS1	128.4564854	179.3707661	77.54220461	-1.21029409	0.203752159	-5.9400307	2.85E-09	1.38E-08
ENSG00000127528	KLF2	169.8666122	237.3983891	102.3348354	-1.211150748	0.188876305	-6.4124017	1.43E-10	7.66E-10
ENSG00000258947	TUBB3	19.36402117	27.0670909	11.66095145	-1.213832442	0.517726815	-2.3445423	0.01905	0.040924
ENSG00000120647	CCDC77	309.1991122	432.204452	186.1937723	-1.214761534	0.147601774	-8.2299928	1.87E-16	1.45E-15
ENSG00000163491	NEK10	72.23056497	101.0791438	43.38198612	-1.215185686	0.28398605	-4.279033	1.88E-05	6.32E-05
ENSG00000279821	AC145098.2	34.80921117	48.70366955	20.91475279	-1.216470992	0.386951078	-3.1437333	0.001668	0.004354
ENSG00000168675	LDLRAD4	94.16003245	131.6663114	56.65375354	-1.217203908	0.253151476	-4.8082039	1.52E-06	5.79E-06
ENSG00000112029	FBXO5	403.3865767	564.2858495	242.4873039	-1.218540573	0.114084017	-10.681081	1.25E-26	1.50E-25
ENSG00000173473	SMARCC1	6681.413709	9349.659156	4013.168263	-1.220279275	0.038199595	-31.944822	6.38E-224	1.41E-221
ENSG00000205885	C1RL-AS1	52.81212272	73.84278032	31.78146512	-1.220464923	0.324737522	-3.758312	0.000171	0.000514
ENSG00000138764	CCNG2	1111.219129	1555.076643	667.3616148	-1.220556564	0.079739073	-15.306882	6.88E-53	1.80E-51
ENSG00000229619	MBNL1-AS1	70.80947745	99.1362793	42.4826756	-1.220775128	0.295653328	-4.1290762	3.64E-05	0.000119
ENSG00000120616	EPC1	690.2228507	965.9523409	414.4933606	-1.221624972	0.095905738	-12.737767	3.65E-37	6.25E-36
ENSG00000113368	LMNB1	1638.332675	2294.330513	982.3348381	-1.222566264	0.065931042	-18.543106	9.27E-77	3.88E-75
ENSG00000214425	LRRC37A4P	121.6610152	170.3319992	72.99003112	-1.222684974	0.209608192	-5.8331927	5.44E-09	2.58E-08
ENSG00000137807	KIF23	861.2004402	1206.324835	516.0760455	-1.22333457	0.087418885	-13.993939	1.70E-44	3.57E-43
ENSG00000079616	KIF22	758.1941357	1062.084886	454.3033856	-1.224617125	0.08915201	-13.736282	6.16E-43	1.25E-41
ENSG00000146410	MTFR2	103.7411403	145.4075348	62.07474578	-1.227808895	0.244296495	-5.0258965	5.01E-07	2.00E-06
ENSG00000269226	TMSB15B	21.55470014	30.14718673	12.96221355	-1.227941798	0.536468942	-2.2889336	0.022083	0.046801
ENSG00000204427	ABHD16A	19.12326181	26.77171021	11.47481341	-1.228794695	0.525015913	-2.3404904	0.019258	0.041311
ENSG00000151012	SLC7A11	5733.952411	8039.964477	3427.940344	-1.229924556	0.051811909	-23.73826	1.45E-124	1.26E-122
ENSG00000099889	ARVCF	975.924854	1368.663829	583.1858794	-1.230448437	0.082729368	-14.873176	4.92E-50	1.18E-48
ENSG00000141994	DUS3L	319.2743575	447.5370054	191.0117095	-1.230487229	0.139180957	-8.8409166	9.49E-19	8.18E-18

ENSG00000082512	TRAF5	605.0023097	848.4362935	361.5683258	-1.230598668	0.098337522	-12.51403	6.26E-36	1.04E-34
ENSG00000171984	SHLD1	64.02862811	89.74891086	38.30834537	-1.230742357	0.290329313	-4.2391254	2.24E-05	7.50E-05
ENSG00000088325	TPX2	2176.58167	3054.763436	1298.399904	-1.234571168	0.058315103	-21.170693	1.78E-99	1.14E-97
ENSG00000104938	CLEC4M	22.42792687	31.50957312	13.34628061	-1.237162413	0.485644144	-2.547467	0.010851	0.024544
ENSG00000140534	TICRR	316.6695711	444.8686119	188.4705303	-1.237455974	0.146986948	-8.4188154	3.80E-17	3.05E-16
ENSG00000273174	AC108673.2	38.50764083	54.11298361	22.90229805	-1.237562917	0.373126964	-3.3167341	0.000911	0.002481
ENSG00000173918	C1QTNF1	753.0764907	1058.16623	447.9867517	-1.239707184	0.089145282	-13.906593	5.78E-44	1.20E-42
ENSG00000127423	AUNIP	52.05172942	73.16460911	30.93884972	-1.240961574	0.313563224	-3.9357612	7.57E-05	0.000238
ENSG00000189180	ZNF33A	772.3389588	1085.461905	459.2160122	-1.241078535	0.087910046	-14.117596	2.96E-45	6.34E-44
ENSG00000130477	UNC13A	19.60014182	27.57129946	11.62898418	-1.241104499	0.544570392	-2.2790525	0.022664	0.047887
ENSG00000160229	ZNF66	20.384919	28.61119799	12.15864001	-1.241238887	0.516187886	-2.4046261	0.016189	0.035279
ENSG00000121988	ZRANB3	136.4576071	191.6279138	81.28730048	-1.241262849	0.204546519	-6.0683646	1.29E-09	6.42E-09
ENSG00000164104	HMGB2	1559.379144	2191.950776	926.807513	-1.241431968	0.070859005	-17.519749	1.01E-68	3.71E-67
ENSG00000135929	CYP27A1	544.3772225	765.0259572	323.7284879	-1.241894114	0.100238541	-12.389387	2.98E-35	4.85E-34
ENSG00000260196	AC124798.1	63.89398801	89.87434283	37.91363319	-1.24557608	0.283916017	-4.3871286	1.15E-05	3.95E-05
ENSG00000171241	SHCBP1	948.8026415	1335.015103	562.5901798	-1.246670495	0.077295264	-16.128679	1.60E-58	4.74E-57
ENSG00000138778	CENPE	1571.999998	2212.636443	931.3635521	-1.24858289	0.07340302	-17.009966	6.93E-65	2.33E-63
ENSG00000246695	RASSF8-AS1	95.50888321	134.5563428	56.4614236	-1.249149073	0.26908843	-4.6421508	3.45E-06	1.25E-05
ENSG00000122641	INHBA	3786.89624	5332.582214	2241.210266	-1.250567959	0.048553213	-25.756647	2.72E-146	3.06E-144
ENSG00000259863	SH3RF3-AS1	81.80771564	115.2936341	48.32179718	-1.251515122	0.258665812	-4.8383476	1.31E-06	5.01E-06
ENSG00000187583	PLEKHN1	99.48876142	140.088608	58.8889148	-1.251921309	0.245205442	-5.1056017	3.30E-07	1.35E-06
ENSG00000147536	GINS4	475.1556023	669.4966232	280.8145813	-1.255213289	0.110914934	-11.316901	1.08E-29	1.46E-28
ENSG00000168939	SPRY3	112.3141736	158.4115016	66.21684557	-1.257218028	0.220925669	-5.6906834	1.27E-08	5.81E-08
ENSG00000003989	SLC7A2	50600.36457	71353.53013	29847.19902	-1.257332552	0.026233461	-47.92858	0	0
ENSG00000072952	MRVI1	44.68233372	63.10114385	26.26352358	-1.263128535	0.378946194	-3.3332662	0.000858	0.002345
ENSG00000197927	C2orf27A	432.9998091	611.5335885	254.4660297	-1.263260646	0.114039041	-11.07744	1.61E-28	2.08E-27
ENSG00000100577	GSTZ1	276.0089971	389.7634938	162.2545003	-1.265238314	0.145617802	-8.6887613	3.66E-18	3.08E-17
ENSG00000123870	ZNF137P	37.09376175	52.4633349	21.7241886	-1.265982983	0.399560443	-3.1684392	0.001533	0.004024
ENSG00000115232	ITGA4	2885.543587	4077.309839	1693.777335	-1.267960439	0.056064712	-22.616016	3.02E-113	2.31E-111
ENSG00000261061	AC092718.4	135.6402436	191.8111677	79.46931941	-1.268587745	0.208597587	-6.0815073	1.19E-09	5.93E-09
ENSG00000138642	HERC6	568.9458728	804.4087875	333.4829581	-1.269349227	0.243244664	-5.2184052	1.80E-07	7.53E-07
ENSG00000006756	ARSD	783.3468439	1107.982164	458.7115442	-1.272050845	0.086949791	-14.629717	1.82E-48	4.19E-47
ENSG00000160190	SLC37A1	467.4428292	661.0659384	273.8197199	-1.272065327	0.107012567	-11.887065	1.38E-32	2.07E-31
ENSG00000121621	KIF18A	278.0192022	393.6257694	162.4126351	-1.273690715	0.155444043	-8.1938857	2.53E-16	1.95E-15
ENSG00000174371	EXO1	329.6880925	466.8048566	192.5713284	-1.274168996	0.163623934	-7.7871798	6.85E-15	4.85E-14
ENSG00000182791	CCDC87	18.65607191	26.42987325	10.88227057	-1.274288701	0.533850822	-2.3869753	0.016988	0.036846
ENSG00000106236	NPTX2	43.20034586	61.06644927	25.33424246	-1.274881951	0.367865279	-3.4656219	0.000529	0.001488
ENSG00000259891	AC107375.1	18.29882536	25.8954911	10.70215962	-1.276044676	0.562430839	-2.2688028	0.02328	0.049016
ENSG00000134986	NREP	4367.072946	6183.726727	2550.419166	-1.277363866	0.046959143	-27.201601	6.22E-163	8.34E-161
ENSG00000158716	DUSP23	1147.175293	1624.051934	670.298652	-1.27760423	0.08895979	-14.361592	9.01E-47	1.99E-45
ENSG00000013810	TACC3	1495.590796	2118.720945	872.4606477	-1.279861834	0.062364032	-20.522436	1.36E-93	7.56E-92
ENSG00000099998	GGT5	726.7034717	1031.319694	422.0872492	-1.286943885	0.098743349	-13.033221	7.92E-39	1.43E-37
ENSG00000131944	FAAP24	89.21725793	126.6010896	51.83342624	-1.288115558	0.253625903	-5.0788013	3.80E-07	1.54E-06
ENSG00000119699	TGFB3	123.7231278	175.6922413	71.75401423	-1.289979801	0.211257101	-6.106208	1.02E-09	5.12E-09
ENSG00000274897	PANO1	29.08642746	41.31730344	16.85555147	-1.290139472	0.427267941	-3.0195092	0.002532	0.006418
ENSG00000085840	ORC1	290.9549964	413.2470485	168.6629443	-1.291904635	0.139018043	-9.2930716	1.50E-20	1.40E-19
ENSG00000135999	EPC2	747.779572	1062.037301	433.5218429	-1.292294798	0.085220646	-15.164105	6.11E-52	1.56E-50
ENSG00000187957	DNER	70.07753916	99.53679533	40.618283	-1.292656693	0.276402311	-4.6767217	2.91E-06	1.07E-05
ENSG00000100526	CDKN3	196.5196058	279.1582904	113.8809212	-1.293031253	0.17184375	-7.524459	5.29E-14	3.54E-13
ENSG00000197461	PDGFA	1305.393779	1854.129538	756.6580193	-1.293677202	0.071011023	-18.217977	3.72E-74	1.49E-72
ENSG00000142279	WTIP	452.7756322	643.2476596	262.3036048	-1.293785983	0.108735407	-11.898479	1.21E-32	1.82E-31
ENSG00000114796	KLHL24	1308.587282	1859.111226	758.0633372	-1.2946328	0.069839427	-18.537277	1.03E-76	4.32E-75
ENSG00000186480	INSIG1	2048.025063	2910.208166	1185.84196	-1.295563898	0.057033964	-22.715656	3.14E-114	2.43E-112
ENSG00000152270	PDE3B	18.53208164	26.30042733	10.76373595	-1.295885362	0.548059974	-2.3644955	0.018055	0.038996
ENSG00000173207	CKS1B	524.039168	745.0157087	303.0626273	-1.296193322	0.109383647	-11.849973	2.15E-32	3.20E-31
ENSG00000259877	AC009113.1	45.83545505	65.19776486	26.47314524	-1.301007985	0.332742935	-3.9099493	9.23E-05	0.000287
ENSG00000164308	ERAP2	994.3205817	1416.18584	572.4553231	-1.306782731	0.081365116	-16.060725	4.81E-58	1.40E-56
ENSG00000221963	APOL6	669.7148258	953.6435499	385.7861016	-1.307366999	0.100878217	-12.959854	2.07E-38	3.70E-37
ENSG00000164307	ERAP1	5049.286325	7195.887227	2902.685423	-1.30953434	0.045221327	-28.958335	2.20E-184	3.47E-182
ENSG00000196550	FAM72A	46.6514098	66.53453285	26.76828674	-1.309985138	0.332831795	-3.9358774	8.29E-05	0.000259
ENSG00000180573	HIST1H2AC	42.89955462	61.20676563	24.59234361	-1.310030423	0.350698648	-3.7354875	0.000187	0.00056
ENSG00000100100	PIK3IP1	687.6551487	980.0803243	395.2299731	-1.310844663	0.090055414	-14.555978	5.35E-48	1.21E-46
ENSG00000075643	MOCOS	107.2802105	152.9705252	61.58989572	-1.313588936	0.221671894	-5.9258254	3.11E-09	1.50E-08
ENSG00000148488	ST8SIA6	18.02187615	25.71377039	10.32998192	-1.313992274	0.535917575	-2.4518552	0.014212	0.031377
ENSG00000254389	RHPN1-AS1	16.85066029	24.04337427	9.657946312	-1.316047309	0.54362825	-2.4208589	0.015484	0.033905
ENSG00000144554	FANCD2	440.5961286	629.3363526	251.8559045	-1.319128973	0.12683989	-10.399954	2.48E-25	2.85E-24
ENSG00000132141	CCT6B	15.60485468	22.28773317	8.92197618	-1.320013388	0.561324033	-2.3516068	0.018693	0.040228
ENSG00000071539	TRIP13	651.0920495	929.9937922	372.1903067	-1.321267809	0.095750985	-13.799	2.58E-43	5.30E-42
ENSG00000250508	AP000808.1	60.20876626	86.03439398	34.38313855	-1.321287831	0.309217062	-4.2730108	1.93E-05	6.48E-05
ENSG00000135063	FAM189A2	155.5118687	222.2888257	88.73491175	-1.323187889	0.192208089	-6.8841426	5.81E-12	3.42E-11
ENSG00000115350	POLE4	171.7517332	245.2716889	98.23177751	-1.323320407	0.193109136	-6.8527074	7.25E-12	4.24E-11
ENSG00000129173	E2F8	223.3713595	319.5135871	127.229132	-1.326812777	0.177884934	-7.458826	8.73E-14	5.77E-13
ENSG00000256043	CTSO	520.087832	743.8207458	296.3549182	-1.327294758	0.10537575	-12.595827	2.23E-36	3.74E-35
ENSG00000166503	HDGFL3	6484.423785	9280.157515	3688.690054	-1.331002533	0.044447419	-29.945553	5.03E-197	8.87E-195
ENSG00000169136	ATF5	1857.925408	2659.693684	1056.157132	-1.332385804	0.061607321	-21.627069	9.99E-104	6.77E-102
ENSG00000231503	PTMAP4	17.51893964	25.10496774	9.932911541	-1.332425286	0.543369311	-2.4521541	0.0142	0.031358
ENSG00000151725	CENPU	565.2638998	809.5216812	321.0061184	-1.334919937	0.097639297	-13.671954	1.49E-42	3.01E-41
ENSG00000132434	LANCL2	579.3782621	830.3586706	328.3978535	-1.34042613	0.105909811	-12.656298	1.03E-36	1.74E-35
ENSG00000112877	CEP72	68.958625	98.85406892	39.06318108	-1.340659024	0.28242107	-4.747022	2.06E-06	7.71E-06
ENSG00000213420	GPC2	111.848144	160.3822844	63.31400356	-1.340830237	0.245926076	-5.4521678	4.98E-08	2.17E-07
ENSG00000138180	CEP55	912.7129128	1308.726448	516.6993778	-1.341489767	0.080162342	-16.734663	7.33E-63	2.37E-61
ENSG00000174348	PODN	286.5747205	411.0685647	162.0808764	-1.34187062	0.145454987	-9.2253325	2.83E-20	2.60E-19
ENSG00000012048	BRCA1	855.6248709	1227.911167	483.3385748	-1.343885169	0.089243661	-15.058606	3.03E-51	7.61E-50
ENSG00000166582	CENPV	229.1354649	328.7530175	129.5179123	-1.344321604	0.151125123	-8.8954211	5.82E-19	5.07E-18
ENSG00000125347	IRF1	1188.209968	1705.179103	671.2408328	-1.345780597	0.071369244	-18.85659	2.59E-79	1.14E-77

ENSG00000182057	OGFRP1	49.77595728	71.56637814	27.98553641	-1.349680804	0.331512074	-4.071287	4.68E-05	0.00015
ENSG00000176890	TYMS	1971.437501	2836.671503	1106.203499	-1.357215325	0.073173012	-18.548031	8.46E-77	3.55E-75
ENSG00000260077	AC104794.2	46.25191083	66.47265093	26.03117074	-1.357971089	0.36721289	-3.6980485	0.000217	0.000644
ENSG00000126561	STAT5A	703.6425801	1013.456448	393.8287123	-1.363337359	0.093338698	-14.606346	2.56E-48	5.87E-47
ENSG00000128917	DLL4	1236.42546	1780.469107	692.381813	-1.36384479	0.077513116	-17.59502	2.69E-69	1.00E-67
ENSG00000166803	PCLAF	116.7648361	168.2313643	65.29830793	-1.36772679	0.217007149	-6.3026808	2.93E-10	1.53E-09
ENSG00000101945	SUV39H1	329.2038459	474.832951	183.5747407	-1.368221325	0.140262347	-9.75473	1.76E-22	1.79E-21
ENSG00000138346	DNA2	253.6049689	365.7477235	141.4622142	-1.370437692	0.144812014	-9.4635635	2.98E-21	2.88E-20
ENSG00000138160	KIF11	1001.680583	1445.504483	557.856683	-1.373378224	0.077044412	-17.825799	4.46E-71	1.70E-69
ENSG00000109906	ZBTB16	362.9713582	524.1599014	201.7828151	-1.375870452	0.124914679	-11.014482	3.25E-28	4.14E-27
ENSG00000161011	SQSTM1	20766.34278	29996.3516	11536.33396	-1.378641104	0.032558927	-42.342952	0	0
ENSG00000256663	AC112777.1	29.97661518	43.26364066	16.68958971	-1.379208437	0.426940742	-3.2304447	0.001236	0.003303
ENSG00000130783	CCDC62	15.5880552	22.51730846	8.658801942	-1.380414299	0.607396187	-2.2726753	0.023046	0.048597
ENSG00000182551	ADI1	1487.550157	2150.750334	824.3499796	-1.382924517	0.064045097	-21.59298	2.09E-103	1.41E-101
ENSG00000105662	CRTC1	336.3473554	486.2460832	186.4486277	-1.383126963	0.133659906	-10.348107	4.27E-25	4.86E-24
ENSG00000113248	PCDHB15	36.68805467	53.0207198	20.35538954	-1.384328031	0.38434765	-3.6017601	0.000316	0.000919
ENSG00000188610	FAM72B	53.1726439	76.94362356	29.40166425	-1.386543036	0.341460338	-4.0606269	4.89E-05	0.000157
ENSG00000110721	CHKA	545.0938812	788.8954578	301.2923046	-1.387432834	0.108795739	-12.752639	3.01E-37	5.18E-36
ENSG00000251442	LINC01094	110.5182945	159.8925999	61.14398915	-1.38790245	0.246615821	-5.6277916	1.83E-08	8.27E-08
ENSG00000160179	ABCG1	2971.25948	4304.1668	1638.352159	-1.393592721	0.051107878	-27.267669	1.03E-163	1.41E-161
ENSG00000185347	TEDC1	183.9937027	266.5676035	101.4198019	-1.394028081	0.169459818	-8.2263046	1.93E-16	1.50E-15
ENSG00000109805	NCAPG	1303.3869	1889.801773	716.9720271	-1.397875479	0.072478614	-19.28673	6.94E-83	3.22E-81
ENSG00000158234	FAIM	160.9234844	233.3273761	88.51959264	-1.398012089	0.214996773	-6.5024794	7.90E-11	4.29E-10
ENSG00000109674	NEIL3	81.8042026	118.7733444	44.83506079	-1.400895949	0.261262341	-5.3620278	8.23E-08	3.53E-07
ENSG00000261008	LINC01572	28.61371327	41.5594173	15.66800923	-1.400920473	0.436662544	-3.2082451	0.001335	0.003549
ENSG00000164050	PLXNB1	1427.769738	2071.360854	784.1786214	-1.402575007	0.083699857	-16.757197	5.02E-63	1.63E-61
ENSG00000248429	FAM198B-AS1	302.8398257	439.6374109	166.0422405	-1.404480198	0.14668179	-9.5750141	1.02E-21	1.00E-20
ENSG00000137266	SLC22A23	386.098616	560.3992635	211.7979685	-1.404951331	0.146143729	-9.6134904	7.01E-22	6.98E-21
ENSG00000169258	GPRIN1	507.9682129	737.8521074	278.0843183	-1.405045378	0.119770163	-11.73118	8.82E-32	1.28E-30
ENSG00000019991	HGF	24.77569707	35.9006376	13.65075654	-1.405110049	0.504586922	-2.7846739	0.005358	0.012853
ENSG00000196814	MVB12B	361.8969825	525.3606827	198.4332822	-1.405126432	0.121777786	-11.538446	8.44E-31	1.18E-29
ENSG00000230330	HMGN2P3	38.39541358	55.73877252	21.05205464	-1.405473205	0.42175861	-3.3324114	0.000861	0.00235
ENSG00000135362	PRR5L	581.4544716	844.4375807	318.4713624	-1.406688052	0.100234979	-14.033904	9.67E-45	2.04E-43
ENSG00000243649	CFB	31.73060968	46.1529499	17.30826945	-1.407879899	0.422386089	-3.3331588	0.000859	0.002345
ENSG00000234028	EIF2AK3-DT	25.90773544	37.61478388	14.200687	-1.409798177	0.449989892	-3.1329552	0.001731	0.004503
ENSG00000163734	CXCL3	1996.947895	2903.574797	1090.320993	-1.413653247	0.062810121	-22.506775	3.56E-112	2.68E-110
ENSG00000169242	EFNA1	3239.860224	4710.860404	1768.860044	-1.413840561	0.050452458	-28.023225	8.47E-173	1.27E-170
ENSG00000104361	NIPAL2	321.3036963	467.3273318	175.2800608	-1.414135487	0.132567946	-10.66725	1.45E-26	1.74E-25
ENSG00000279393	AL139005.1	20.04553116	29.19569007	10.89537225	-1.415795737	0.5781651	-2.4487741	0.014334	0.031617
ENSG00000154930	ACSS1	745.3938471	1084.292694	406.495	-1.416318794	0.088215663	-16.055185	5.26E-58	1.53E-56
ENSG00000105443	CYTH2	1719.30772	2502.985236	935.6302032	-1.419199651	0.081551043	-17.402593	7.89E-68	2.84E-66
ENSG00000237649	KIFC1	692.4979235	1009.104532	375.8913147	-1.423909724	0.091476276	-15.565891	1.24E-54	3.38E-53
ENSG00000238083	LRRC37A2	43.92320981	64.01267897	23.83374065	-1.427016268	0.349995538	-4.0772413	4.56E-05	0.000147
ENSG00000125730	C3	1610.085617	2347.099013	873.0722209	-1.42710356	0.064329145	-22.184401	4.86E-109	3.47E-107
ENSG00000170312	CDK1	803.42002	1171.661331	435.1787089	-1.427939359	0.084296667	-16.939452	2.30E-64	7.68E-63
ENSG00000187801	ZFP69B	128.9180713	187.9787515	69.85739119	-1.428683911	0.215347107	-6.6343306	3.26E-11	1.83E-10
ENSG00000182901	RGS7	78.5401748	114.5421248	42.53822484	-1.429757897	0.271843085	-5.259497	1.44E-07	6.08E-07
ENSG00000162636	FAM102B	474.0900304	691.4767084	256.7033524	-1.431003671	0.110069814	-13.000873	1.21E-38	2.17E-37
ENSG00000215014	AL645728.1	27.99355178	40.83469934	15.15240423	-1.431063616	0.425039145	-3.3668984	0.00076	0.002092
ENSG00000166741	NNMT	7525.87379	10981.76422	4069.983362	-1.431955542	0.038782215	-36.922995	1.98E-298	6.07E-296
ENSG00000160223	ICOSLG	612.9188635	894.2112334	331.6264935	-1.432983314	0.100180857	-14.303963	2.07E-46	4.52E-45
ENSG00000276672	AL161891.1	22.76358702	33.27017568	12.25699836	-1.434445353	0.522092229	-2.7474942	0.006005	0.014256
ENSG00000186280	KDM4D	20.35125859	29.7408522	10.96166498	-1.439881675	0.503560959	-2.8593989	0.004244	0.010353
ENSG00000118193	KIF14	424.6266682	620.9645261	228.2888103	-1.442344825	0.12167479	-11.854097	2.05E-32	3.05E-31
ENSG00000196843	ARID5A	993.0562229	1452.368824	533.7436223	-1.443446223	0.080126486	-18.014355	1.50E-72	5.85E-71
ENSG00000184661	CDCA2	412.6959237	604.223311	221.1685364	-1.446782493	0.136109181	-10.629573	2.17E-26	2.59E-25
ENSG00000198826	ARHGAP11A	1292.112167	1892.181192	692.0431419	-1.450342266	0.070881946	-20.461378	4.76E-93	2.61E-91
ENSG00000139734	DIAPH3	839.4647156	1229.423477	449.5059543	-1.451085297	0.082822202	-17.520487	1.00E-68	3.67E-67
ENSG00000134321	RSAD2	62.27016848	91.23573719	33.30459977	-1.453640084	0.328641482	-4.4231789	9.73E-06	3.37E-05
ENSG00000177943	MAMDC4	69.3471039	101.6648532	37.02935456	-1.455743999	0.310648419	-4.6861465	2.78E-06	1.02E-05
ENSG00000227076	AL158166.1	38.47729909	56.38713044	20.56746775	-1.45648184	0.381419175	-3.8185858	0.000134	0.000408
ENSG00000260920	AL031985.3	76.32745992	111.7984536	40.8564662	-1.457686128	0.283179626	-5.1475671	2.64E-07	1.09E-06
ENSG00000141441	GAREM1	53.46916232	78.48470875	28.4536159	-1.460548995	0.313763491	-4.6549361	3.24E-06	1.18E-05
ENSG00000162614	NEXN	918.4022909	1348.438711	488.3658704	-1.464741428	0.088695651	-16.514242	2.90E-61	9.05E-60
ENSG00000232807	AL137186.2	110.9674091	162.7888546	59.14596359	-1.46561462	0.241965588	-6.0571201	1.39E-09	6.87E-09
ENSG00000104856	RELB	1118.199679	1642.607742	593.7916161	-1.467231119	0.073975717	-19.833956	1.52E-87	7.66E-86
ENSG00000179988	PSTK	28.59086803	41.9724719	15.20926417	-1.468521789	0.435491787	-3.3720999	0.000746	0.002056
ENSG00000112182	BACH2	61.54476077	90.47949184	32.6100297	-1.471393745	0.287997707	-5.1090467	3.24E-07	1.32E-06
ENSG00000262001	DLGAP1-AS2	212.0738384	312.2334387	111.9142381	-1.482717871	0.185082213	-8.0111311	1.14E-15	8.40E-15
ENSG00000120328	PCDHB12	15.5264664	22.89653582	8.156396984	-1.482766901	0.598021344	-2.4794548	0.013158	0.029274
ENSG00000147251	DOCK11	26.67337812	39.31900723	14.02774901	-1.483090767	0.448613596	-3.3059425	0.000947	0.002576
ENSG00000178295	GEN1	427.4752253	630.1008511	224.8495994	-1.485220134	0.122821342	-12.092525	1.16E-33	1.79E-32
ENSG00000221949	LINC01465	18.85866498	27.86852921	9.848800742	-1.489956248	0.572513585	-2.6024819	0.009255	0.021233
ENSG00000179144	GIMAP7	1215.371287	1793.037742	637.7048319	-1.490646855	0.077771337	-19.167047	6.98E-82	3.19E-80
ENSG00000120549	KIAA1217	595.5087457	878.3859736	312.6315178	-1.491565544	0.098958806	-15.07259	2.45E-51	6.16E-50
ENSG00000099953	MMP11	422.4055303	623.0095943	221.8014664	-1.492476689	0.125628007	-11.880127	1.50E-32	2.25E-31
ENSG00000113657	DPYSL3	23295.58085	34404.23639	12186.92531	-1.497190504	0.028577011	-52.391431	0	0
ENSG00000112149	CD83	35.58154737	52.54046151	18.62263324	-1.498350321	0.395654517	-3.7870169	0.000152	0.000461
ENSG00000132967	HMGB1P5	112.238183	165.8529836	58.62338236	-1.501490684	0.218124614	-6.8836371	5.83E-12	3.43E-11
ENSG00000233251	AC007743.1	1133.430332	1676.362866	590.4977973	-1.505203108	0.071489889	-21.054769	2.07E-98	1.30E-96
ENSG00000270157	AC004918.3	221.2281143	327.6332862	114.8229424	-1.511802307	0.155630634	-9.7140407	2.63E-22	2.66E-21
ENSG00000099377	HSD3B7	53.9615853	80.02069748	27.90247312	-1.513124836	0.336372781	-4.4983569	6.85E-06	2.41E-05
ENSG00000146670	CDCA5	691.4673692	1024.661835	358.2729038	-1.514168029	0.094954559	-15.946238	3.03E-57	8.59E-56
ENSG00000184441	AP001062.1	216.830286	321.1250327	112.5355393	-1.514323152	0.172404511	-8.7835471	1.58E-18	1.35E-17
ENSG00000159708	LRRC36	26.39562765	39.10607493	13.68518037	-1.514789474	0.465589284	-3.2534887	0.00114	0.003064

ENSG00000171848	RRM2	2710.144969	4016.25205	1404.037887	-1.515206269	0.065426399	-23.158943	1.18E-118	9.70E-117
ENSG00000137804	NUSAP1	50.54821006	74.91474749	26.18167262	-1.51910804	0.323902154	-4.6900214	2.73E-06	1.01E-05
ENSG00000134690	CDCA8	570.6029729	846.6186356	294.5873101	-1.522185799	0.101349289	-15.019206	5.50E-51	1.36E-49
ENSG00000184635	ZNF93	157.2328838	233.3906119	81.07515581	-1.522851366	0.198745869	-7.6623045	1.83E-14	1.26E-13
ENSG00000271978	AL359643.2	18.6105345	27.64251709	9.578551909	-1.526963311	0.547275738	-2.7901169	0.005269	0.012651
ENSG00000007968	E2F2	65.89600464	97.86175354	33.93025575	-1.528028316	0.293751392	-5.2017739	1.97E-07	8.21E-07
ENSG00000161800	RACGAP1	570.3883014	847.1488688	293.6277339	-1.528122485	0.098983773	-15.438111	9.07E-54	2.44E-52
ENSG00000106100	NOD1	872.6021562	1295.713685	449.4906273	-1.528371888	0.084338794	-18.121813	2.14E-73	8.53E-72
ENSG00000095713	CRTAC1	129.8778962	193.0351328	66.72065958	-1.5292093	0.228184237	-6.701643	2.06E-11	1.17E-10
ENSG00000159261	CLDN14	924.9370394	1374.526926	475.3471533	-1.531073823	0.087874953	-17.423325	5.49E-68	1.98E-66
ENSG00000131477	RAMP2	405.0364037	601.7269866	208.3458207	-1.531312551	0.133392164	-11.479779	1.67E-30	2.31E-29
ENSG00000178301	AQP11	12.14975873	18.01930407	6.28021339	-1.531989333	0.674579004	-2.2710303	0.023145	0.048774
ENSG00000203706	SERTAD4-AS1	46.74619173	69.54115571	23.95122776	-1.532420832	0.359214858	-4.2660285	1.99E-05	6.68E-05
ENSG00000184678	HIST2H2BE	29.10981573	43.33030309	14.88932836	-1.534060877	0.461531433	-3.3238492	0.000888	0.002421
ENSG00000232600	TONSL-AS1	12.04206642	17.93545739	6.148675452	-1.538690648	0.65056227	-2.3651704	0.018022	0.03893
ENSG00000151640	DPYSL4	913.7629818	1359.572606	467.9533578	-1.539187404	0.086173953	-17.8614	2.36E-71	9.05E-70
ENSG00000010030	ETV7	18.18251013	27.05671719	9.308303076	-1.541577996	0.539940081	-2.8550909	0.004302	0.010482
ENSG00000165480	SKA3	275.9151092	410.8482363	140.9819822	-1.543545085	0.146230924	-10.555531	4.79E-26	5.63E-25
ENSG00000266835	GAPLINC	43.07154934	64.17365227	21.9694464	-1.545093828	0.364858085	-4.2347803	2.29E-05	7.64E-05
ENSG00000250318	AC003072.1	21.71755395	32.35972306	11.07538484	-1.54672735	0.514402041	-3.0068453	0.00264	0.006673
ENSG00000204428	LY6G5C	56.22197633	83.74743715	28.69651551	-1.548974009	0.310590062	-4.9871976	6.13E-07	2.43E-06
ENSG00000122966	CIT	752.3852902	1122.321301	382.4492795	-1.55236967	0.089336957	-17.376568	1.24E-67	4.43E-66
ENSG00000175063	UBE2C	422.9186229	630.657514	215.1797318	-1.552884682	0.115876033	-13.401259	5.94E-41	1.15E-39
ENSG00000065675	PRKCQ	17.54531028	26.21842973	8.872190843	-1.554294573	0.561287924	-2.7691573	0.00562	0.013415
ENSG00000128815	WDFY4	87.10188142	130.0252778	44.17848507	-1.555636588	0.249499267	-6.2350347	4.52E-10	2.33E-09
ENSG00000277013	AC008556.1	42.11406481	62.87490641	21.35322322	-1.557246963	0.352074869	-4.4230563	9.73E-06	3.37E-05
ENSG00000237840	FAM21FP	16.13780121	24.11684725	8.158755182	-1.557777467	0.581648891	-2.6782093	0.007402	0.017276
ENSG00000166707	ZCCHC18	29.94676519	44.71623349	15.1772969	-1.558250757	0.419216304	-3.7170567	0.000202	0.000601
ENSG00000063127	SLC6A16	32.90223416	49.1504164	16.65405192	-1.55923977	0.44694567	-3.4886562	0.000485	0.001372
ENSG00000156804	FBXO32	948.6308647	1416.631867	480.6298626	-1.559638279	0.077954138	-20.007126	4.77E-89	2.50E-87
ENSG00000183496	MEX3B	741.2958141	1107.241595	375.3500327	-1.559898877	0.0881956	-17.686811	5.30E-70	1.99E-68
ENSG00000204482	LST1	41.42002513	61.84118668	20.99886359	-1.560551052	0.355565471	-4.3889274	1.14E-05	3.92E-05
ENSG00000275202	AL161421.1	22.21435421	33.23950535	11.18920307	-1.56163887	0.505908212	-3.0868028	0.002023	0.005212
ENSG00000153707	PTPRD	1129.516868	1688.223781	570.8099556	-1.563205532	0.080939521	-19.313254	4.16E-83	1.94E-81
ENSG00000094755	GABRP	11.54304103	17.26215717	5.823924885	-1.564330899	0.666509735	-2.3470488	0.018923	0.040673
ENSG00000175536	LIPT2	22.00598879	32.87606393	11.13591366	-1.565926319	0.5339534	-2.9327022	0.00336	0.008333
ENSG00000165617	DACT1	24.6720408	36.86304922	12.48103238	-1.566162978	0.464908333	-3.3687565	0.000755	0.002079
ENSG00000198336	MYL4	18.6562537	27.89153197	9.420975429	-1.569833054	0.589147407	-2.6645845	0.007708	0.017952
ENSG00000273132	AL355312.4	121.2253298	181.3928762	61.05778333	-1.572379171	0.214741437	-7.3221973	2.44E-13	1.58E-12
ENSG00000177548	RABEP2	1029.8776	1541.371639	518.3835611	-1.572853039	0.07542639	-20.852821	1.44E-96	8.61E-95
ENSG00000124721	DNAH8	34.74524881	52.0755829	17.41491472	-1.573719405	0.400018164	-3.9341199	8.35E-05	0.000261
ENSG00000114737	CISH	56.47910291	84.63272215	28.32548367	-1.575320189	0.369684658	-4.2612539	2.03E-05	6.82E-05
ENSG00000237976	AL391069.2	15.67491313	23.50403065	7.845795606	-1.57599982	0.633818359	-2.4865165	0.0129	0.028766
ENSG00000170801	HTRA3	991.1895812	1484.214144	498.1650187	-1.576139979	0.096524579	-16.328898	6.15E-60	1.88E-58
ENSG00000050730	TNIP3	745.5697665	1117.229604	373.9099294	-1.580465183	0.091327041	-17.305556	4.27E-67	1.51E-65
ENSG00000234664	HMGN2P5	139.669298	209.4357743	69.90282167	-1.581469393	0.20997777	-7.5316039	5.01E-14	3.36E-13
ENSG00000169607	CKAP2L	510.8176047	765.9902194	255.6449899	-1.583285038	0.112324741	-14.095604	4.04E-45	8.61E-44
ENSG00000245648	AC022075.1	409.9027374	614.8917903	204.9136844	-1.586748804	0.119017213	-13.332095	1.51E-40	2.86E-39
ENSG00000259953	AL138756.1	149.9073154	224.9320071	74.88262374	-1.588814618	0.190582343	-8.3366307	7.64E-17	6.03E-16
ENSG00000091137	SLC26A4	19.97745023	29.99473806	9.960162408	-1.592203526	0.53715245	-2.9641558	0.003035	0.007597
ENSG00000203865	ATP1A1-AS1	13.18527793	19.82009363	6.550462223	-1.593605425	0.651039307	-2.4477868	0.014374	0.031696
ENSG00000198346	ZNF813	189.0143989	284.0304702	93.99832751	-1.595852828	0.170356128	-9.3677454	7.41E-21	7.01E-20
ENSG00000149633	KIAA1755	66.10302886	99.45024252	32.75581519	-1.595880031	0.295491591	-5.4007629	6.64E-08	2.87E-07
ENSG00000085999	RAD54L	234.4116146	352.558022	116.2652072	-1.596635815	0.181150146	-8.8138809	1.21E-18	1.04E-17
ENSG00000171105	INSR	1197.196805	1799.187603	595.2060079	-1.597489218	0.078500113	-20.350152	4.63E-92	2.51E-90
ENSG00000185499	MUC1	20.20370817	30.42236126	9.985055077	-1.603906297	0.520559741	-3.0811186	0.002062	0.005306
ENSG00000104290	FZD3	71.44136131	107.3784329	35.5042897	-1.604139966	0.339857685	-4.7200344	2.36E-06	8.75E-06
ENSG00000135476	ESPL1	407.3306204	613.640764	201.0204768	-1.608267953	0.122017579	-13.180625	1.13E-39	2.12E-38
ENSG00000092853	CLSPN	999.3098569	1507.58873	491.0309839	-1.61727967	0.078327411	-20.647685	1.02E-94	5.86E-93
ENSG00000153885	KCTD15	1540.46013	2323.566525	757.3537353	-1.617427437	0.065508124	-24.690486	1.35E-134	1.35E-132
ENSG00000127564	PKMYT1	341.232313	514.8246713	167.6399548	-1.618921401	0.133966578	-12.084517	1.28E-33	1.97E-32
ENSG00000113319	RASGRF2	224.0056648	337.9751733	110.0361562	-1.619180833	0.154495551	-10.480437	1.06E-25	1.24E-24
ENSG00000279191	AC068491.4	26.01079171	39.26086942	12.760714	-1.620590477	0.458206816	-3.5368101	0.000405	0.00116
ENSG00000203666	EFCAB2	90.95382704	137.3986545	44.50899954	-1.623789746	0.266308919	-6.0973915	1.08E-09	5.39E-09
ENSG00000213976	AC010615.1	13.93779164	21.09994116	6.775462115	-1.625126468	0.648062749	-2.5076684	0.012153	0.027215
ENSG00000197061	HIST1H4C	47.0642672	71.16099113	22.96754326	-1.628310856	0.365504982	-4.4549621	8.39E-06	2.93E-05
ENSG00000172031	EPHX4	170.0903005	257.3334503	82.8471507	-1.631413943	0.204738085	-7.9682974	1.61E-15	1.18E-14
ENSG00000260231	KDM7A-DT	54.86389795	82.96196572	26.76583018	-1.636167969	0.339350241	-4.8214728	1.43E-06	5.43E-06
ENSG00000136161	RCBTB2	234.5991964	355.244006	113.9543868	-1.639834763	0.158863464	-10.32229	5.59E-25	6.32E-24
ENSG00000187951	AC091057.1	64.51616924	97.69406017	31.3382783	-1.641882941	0.286160743	-5.7376247	9.60E-09	4.46E-08
ENSG00000237945	LINC00649	23.41801426	35.48672596	11.34930256	-1.642160446	0.539596353	-3.0433127	0.00234	0.005972
ENSG00000127533	F2RL3	464.2923633	703.389448	225.1952787	-1.644110069	0.12326807	-13.33768	1.40E-40	2.66E-39
ENSG00000177191	B3GNT8	127.8113343	193.5895861	62.03308254	-1.644535204	0.209202882	-7.8609586	3.81E-15	2.74E-14
ENSG00000236901	MIR600HG	44.5515723	67.55539807	21.54774654	-1.646720777	0.396512232	-4.1530138	3.28E-05	0.000108
ENSG00000106799	TGFBR1	1321.835109	2004.014102	639.6561163	-1.646916426	0.073750038	-22.331059	1.85E-110	1.35E-108
ENSG00000255468	AP001107.9	14.13967769	21.4764624	6.802892982	-1.649798964	0.614710636	-2.6838627	0.007278	0.017005
ENSG00000140691	ARMC5	517.1562993	785.0198786	249.2927199	-1.655360942	0.103700182	-15.962951	2.32E-57	6.58E-56
ENSG00000251429	AC098679.2	15.00545719	22.76483456	7.24607981	-1.655607563	0.637979356	-2.5950801	0.009457	0.021664
ENSG00000170458	CD14	20.616456	31.36393499	9.868977015	-1.660616868	0.527930707	-3.1455205	0.001658	0.004331
ENSG00000163092	XIRP2	30.32998823	46.06824617	14.59173029	-1.662019792	0.465921225	-3.5671691	0.000361	0.001042
ENSG00000169679	BUB1	736.6208183	1119.919287	353.3223492	-1.66245854	0.096584169	-17.212537	2.14E-66	7.43E-65
ENSG00000144218	AFF3	28.7251734	43.71773929	13.73260751	-1.669280255	0.426929547	-3.9099666	9.23E-05	0.000287
ENSG00000235217	TSPY26P	150.4580818	229.2297528	71.68641082	-1.675115812	0.197110142	-8.4983746	1.92E-17	1.56E-16
ENSG00000280202	AC005831.1	76.63004468	116.7827603	36.47732908	-1.677826552	0.295433279	-5.6792063	1.35E-08	6.20E-08
ENSG00000072571	HMMR	479.5844157	731.0576755	228.1111559	-1.678065381	0.114687793	-14.631595	1.77E-48	4.08E-47

ENSG00000143127	ITGA10	840.7842531	1281.606903	399.9616034	-1.678880262	0.092049783	-18.238829	2.54E-74	1.02E-72
ENSG00000123485	HJURP	445.4162698	679.6040212	211.2285184	-1.683696337	0.1286036	-13.09214	3.65E-39	6.69E-38
ENSG00000198246	SLC29A3	148.2601423	226.2679181	70.25236654	-1.686945179	0.197673134	-8.5340134	1.41E-17	1.16E-16
ENSG00000104147	OIP5	70.37995022	107.553659	33.20624142	-1.698076882	0.293550797	-5.78461	7.27E-09	3.41E-08
ENSG00000113645	WWC1	28.77609844	43.95358387	13.59861301	-1.701336497	0.453185228	-3.7541747	0.000174	0.000522
ENSG00000197142	ACSL5	189.0077654	289.1844542	88.83107671	-1.701782421	0.174568193	-9.7485251	1.87E-22	1.90E-21
ENSG00000278531	AL512324.3	17.54265111	26.8673289	8.217973313	-1.705476265	0.563742069	-3.0252776	0.002484	0.006309
ENSG00000134516	DOCK2	68.89028634	105.4515353	32.32903739	-1.707512149	0.292900376	-5.8296687	5.55E-09	2.63E-08
ENSG00000198830	HMGN2	3901.134483	5978.26179	1824.007175	-1.713289814	0.047339059	-36.191886	8.17E-287	2.36E-284
ENSG00000214826	DDX12P	43.67068486	67.04546148	20.29590823	-1.72308553	0.432209503	-3.9866905	6.70E-05	0.000211
ENSG00000125531	FNDC11	17.81706521	27.3367627	8.297367715	-1.724579593	0.560209648	-3.0784539	0.002081	0.005352
ENSG00000140451	PIF1	90.18467324	138.4926769	41.87666954	-1.724935465	0.285230097	-6.0475226	1.47E-09	7.27E-09
ENSG00000163071	SPATA18	220.5460453	339.0049695	102.0871211	-1.730094154	0.159648481	-10.836897	2.30E-27	2.85E-26
ENSG00000010327	STAB1	20509.93758	31520.62871	9499.246454	-1.730413111	0.100023854	-17.300004	4.70E-67	1.66E-65
ENSG00000112742	TTK	373.1697867	574.0724721	172.2671014	-1.733778866	0.136686217	-12.684372	7.22E-37	1.23E-35
ENSG00000111962	UST	23.99717249	36.89642567	11.09791931	-1.737074902	0.478464465	-3.6305202	0.000283	0.000827
ENSG00000167528	ZNF641	103.7807894	159.6890938	47.87248491	-1.739001245	0.24366259	-7.1369234	9.54E-13	5.93E-12
ENSG00000078177	N4BP2	455.603184	701.4756316	209.7307363	-1.740103813	0.128283003	-13.56457	6.50E-42	1.29E-40
ENSG00000270574	AC010680.2	8.987000421	13.84247971	4.131521127	-1.742299341	0.765432183	-2.2762295	0.022832	0.0482
ENSG00000131747	TOP2A	2579.416781	3973.02855	1185.805011	-1.743463641	0.05910051	-29.499976	2.88E-191	4.73E-189
ENSG00000111341	MGP	8159.15277	12567.2887	3751.016834	-1.74421206	0.039865256	-43.752687	0	0
ENSG00000276136	AC016957.2	15.8367044	24.37754365	7.295865147	-1.745420659	0.605216624	-2.8839602	0.003927	0.009646
ENSG00000214401	KANSL1-AS1	13.34373213	20.58621594	6.101248312	-1.747010229	0.659117303	-2.6505301	0.008037	0.018673
ENSG00000011201	ANOS1	23.29308661	35.87222261	10.71395061	-1.748089875	0.48206803	-3.6262307	0.000288	0.00084
ENSG00000267374	AC016205.1	12.02531683	18.52892487	5.521708788	-1.748563899	0.662290468	-2.6401768	0.008286	0.019193
ENSG00000171320	ESCO2	356.9144389	550.2658439	163.5630338	-1.74922704	0.130259523	-13.428784	4.10E-41	7.98E-40
ENSG00000226363	HAGLROS	155.5935125	239.7864212	71.40060375	-1.749722264	0.191922314	-9.1168256	7.74E-20	7.02E-19
ENSG00000142731	PLK4	346.2723456	534.0848089	158.4598823	-1.751691356	0.134748265	-12.999732	1.23E-38	2.20E-37
ENSG00000090889	KIF4A	792.0436349	1221.473322	362.6139482	-1.751713543	0.08830153	-19.837862	1.40E-87	7.11E-86
ENSG00000241764	AC002467.1	45.20155969	69.72062108	20.6824983	-1.752650886	0.356044352	-4.9225634	8.54E-07	3.34E-06
ENSG00000117724	CENPF	2315.263661	3572.51634	1058.010983	-1.754970623	0.059629107	-29.431442	2.18E-190	3.54E-188
ENSG00000130558	OLFM1	12.24113979	18.85628372	5.62599586	-1.761643661	0.716067578	-2.460164	0.013887	0.030747
ENSG00000121152	NCAPH	461.4609745	712.9391435	209.9828055	-1.762427014	0.115502323	-15.258801	1.44E-52	3.73E-51
ENSG00000145386	CCNA2	913.2884646	1412.983698	413.5932312	-1.771229036	0.087774607	-20.179287	1.49E-90	7.90E-89
ENSG00000138336	TET1	53.06395949	82.12218949	24.00572949	-1.771965086	0.32562215	-5.441783	5.27E-08	2.30E-07
ENSG00000186193	SAPCD2	288.3622229	446.3838977	130.3405481	-1.774245812	0.145907464	-12.160076	5.07E-34	7.96E-33
ENSG00000011426	ANLN	1995.268146	3089.835348	900.7009437	-1.777375016	0.073192231	-24.283657	2.92E-130	2.72E-128
ENSG00000129195	PIMREG	223.0032902	345.2431894	100.763391	-1.777923039	0.162517602	-10.93988	7.43E-28	9.32E-27
ENSG00000153094	BCL2L11	810.6199043	1257.337469	363.9023399	-1.790806783	0.094383997	-18.973627	2.82E-80	1.26E-78
ENSG00000133466	C1QTNF6	762.9960581	1183.828542	342.1635741	-1.792569062	0.096629715	-18.550909	8.02E-77	3.37E-75
ENSG00000084710	EFR3B	69.66650213	108.043299	31.28970529	-1.795276681	0.300748526	-5.9693615	2.38E-09	1.16E-08
ENSG00000120833	SOCS2	22.81426661	35.50476724	10.12376597	-1.79589064	0.527680331	-3.4033685	0.000666	0.001849
ENSG00000203635	AC144450.1	21.63145385	33.56460884	9.698298857	-1.79738678	0.532722413	-3.373965	0.000741	0.002044
ENSG00000257093	KIAA1147	7298.424159	11336.06002	3260.7883	-1.797661073	0.035614949	-50.474902	0	0
ENSG00000154642	C21orf91	292.0332228	453.5685178	130.4979279	-1.798692176	0.14416003	-12.477052	9.96E-36	1.64E-34
ENSG00000101057	MYBL2	1704.154157	2647.801241	760.5070726	-1.800279215	0.060989902	-29.51766	1.71E-191	2.83E-189
ENSG00000255326	AP001922.5	12.36455928	19.22522785	5.503890714	-1.80340814	0.721007852	-2.5012323	0.012376	0.027692
ENSG00000181754	AMIGO1	40.25465223	62.54691584	17.96238862	-1.804987479	0.394671961	-4.5733867	4.80E-06	1.72E-05
ENSG00000156966	B3GNT7	17.73933165	27.61504962	7.86361368	-1.806303438	0.568406523	-3.1778373	0.001484	0.003908
ENSG00000120708	TGFBI	18364.64731	28563.57202	8165.722593	-1.806508613	0.030221813	-59.774991	0	0
ENSG00000260852	FBXL19-AS1	123.2936117	191.9519349	54.63528852	-1.811099926	0.220613023	-8.2093972	2.22E-16	1.72E-15
ENSG00000182050	MGAT4C	11.60794221	18.07342794	5.142456488	-1.812821747	0.676110584	-2.6812504	0.007335	0.017128
ENSG00000152253	SPC25	134.2203844	209.0269146	59.41385422	-1.816665913	0.209003741	-8.6920258	3.56E-18	3.00E-17
ENSG00000178999	AURKB	408.1744518	636.2004689	180.1484348	-1.818458071	0.120907097	-15.040127	4.01E-51	9.97E-50
ENSG00000259673	IQCH-AS1	9.385739219	14.61270644	4.158771994	-1.819318514	0.781951522	-2.3266385	0.019985	0.042752
ENSG00000196503	ARL9	31.17790559	48.58049292	13.77531825	-1.820236783	0.434218005	-4.1919883	2.77E-05	9.15E-05
ENSG00000221866	PLXNA4	1779.260406	2774.077183	784.44363	-1.822396108	0.067885862	-26.845002	9.65E-159	1.22E-156
ENSG00000166851	PLK1	728.8038221	1136.683278	320.9243658	-1.824526931	0.088774487	-20.552379	7.33E-94	4.12E-92
ENSG00000156466	GDF6	107.5651369	167.8134832	47.31679054	-1.82719322	0.248780728	-7.3445931	2.06E-13	1.34E-12
ENSG00000138623	SEMA7A	287.9584561	449.4649856	126.4519266	-1.828712062	0.148110846	-12.346915	5.06E-35	8.18E-34
ENSG00000089685	BIRC5	847.4358819	1323.405998	371.465766	-1.83306215	0.086639265	-21.157407	2.36E-99	1.50E-97
ENSG00000186871	ERCC6L	206.7665322	322.8367443	90.6963201	-1.833145347	0.167442457	-10.947912	6.80E-28	8.54E-27
ENSG00000169403	PTAFR	17.2347791	26.92230981	7.547248395	-1.834697522	0.62733816	-2.924575	0.003449	0.008544
ENSG00000185155	MIXL1	25.64365479	40.10046471	11.18684487	-1.835089642	0.487696035	-3.7627733	0.000168	0.000506
ENSG00000111206	FOXM1	1287.704096	2012.311644	563.0965487	-1.836588036	0.069915079	-26.26884	4.36E-152	5.18E-150
ENSG00000136999	CCN3	17.8716773	27.96856811	7.774786485	-1.837311862	0.608152	-3.0211392	0.002518	0.00639
ENSG00000137135	ARHGEF39	38.60324543	60.35801399	16.84847688	-1.840387796	0.386674285	-4.7595298	1.94E-06	7.28E-06
ENSG00000156970	BUB1B	571.6129066	893.8588433	249.3669699	-1.841422164	0.107411036	-17.143696	7.01E-66	2.40E-64
ENSG00000151364	KCTD14	10.2602636	16.05718091	4.463346288	-1.843768701	0.73578688	-2.5058461	0.012216	0.027343
ENSG00000164010	ERMAP	279.1074066	436.7155362	121.499277	-1.844613945	0.15219164	-12.120337	8.24E-34	1.28E-32
ENSG00000169860	P2RY1	74.92206569	117.2435345	32.60059691	-1.845318706	0.274922856	-6.7121328	1.92E-11	1.09E-10
ENSG00000117650	NEK2	162.480942	254.6480075	70.31387642	-1.852518265	0.203065503	-9.122762	7.32E-20	6.66E-19
ENSG00000142178	SIK1	42.99632229	67.37236954	18.62027504	-1.856615885	0.36710669	-5.0574286	4.25E-07	1.71E-06
ENSG00000213793	ZNF888	146.5025964	229.7608876	63.24430513	-1.861327971	0.201363719	-9.2436114	2.38E-20	2.20E-19
ENSG00000197046	SIGLEC15	19.17520348	30.10754099	8.242865981	-1.866719047	0.529901362	-3.522767	0.000427	0.001218
ENSG00000267648	AC060766.5	17.45506823	27.45538414	7.454752314	-1.870534312	0.575054586	-3.2527944	0.001143	0.00307
ENSG00000267658	AC099811.3	19.30895148	30.42010591	8.19779704	-1.884578859	0.592123312	-3.1827473	0.001459	0.003848
ENSG00000178776	C5orf46	25.07375677	39.50352454	10.643989	-1.887070423	0.493888469	-3.8208432	0.000133	0.000405
ENSG00000213025	COX20P1	8.848144294	13.94166156	3.754627024	-1.889028021	0.780980736	-2.4187895	0.015572	0.034079
ENSG00000129810	SGO1	148.0979224	233.4101863	62.78565842	-1.892723739	0.19967663	-9.4789447	2.57E-21	2.49E-20
ENSG00000182168	UNC5C	40.50011032	63.83537847	17.16484216	-1.894750934	0.368937987	-5.1356895	2.81E-07	1.15E-06
ENSG00000215458	AATBC	46.74966494	73.80182675	19.69750312	-1.908501339	0.346882536	-5.5018663	3.76E-08	1.66E-07
ENSG00000135451	TROAP	232.2403568	366.8498642	97.63084938	-1.909150745	0.155214895	-12.300049	9.05E-35	1.45E-33
ENSG00000228624	HDAC2-AS2	8.903504135	14.07963211	3.727376157	-1.912231611	0.806523009	-2.3709573	0.017742	0.038352
ENSG00000163131	CTSS	2974.840637	4704.336863	1245.344411	-1.91721986	0.0492699	-38.912599	0	0

ENSG00000109576	AADAT	44.38391275	70.2020522	18.5657733	-1.92158345	0.381572687	-5.0359565	4.75E-07	1.91E-06
ENSG00000152495	CAMK4	47.14230436	74.60597119	19.67863754	-1.923118951	0.355718452	-5.4062952	6.43E-08	2.79E-07
ENSG00000196872	KIAA1211L	125.3391439	198.3943369	52.28395084	-1.923995727	0.214284991	-8.978677	2.74E-19	2.42E-18
ENSG00000143340	FAM163A	15.63379919	24.74910343	6.51849496	-1.924458091	0.637194645	-3.0202044	0.002526	0.006407
ENSG00000165029	ABCA1	439.0500184	694.8467713	183.2532656	-1.924995907	0.13264004	-14.512932	1.00E-47	2.26E-46
ENSG00000136982	DSCC1	74.87955163	118.6149867	31.14411652	-1.925876135	0.308972089	-6.2331719	4.57E-10	2.36E-09
ENSG00000185860	CCDC190	14.20932371	22.46318459	5.955462823	-1.926043339	0.648285812	-2.9709787	0.002969	0.007441
ENSG00000154721	JAM2	42.02239561	66.52794763	17.5168436	-1.928657058	0.41184029	-4.6830218	2.83E-06	1.04E-05
ENSG00000144445	KANSL1L	305.8451625	484.5271562	127.1631689	-1.931323512	0.140577152	-13.738531	5.97E-43	1.21E-41
ENSG00000227683	AL358394.1	37.34090799	59.19994479	15.4818712	-1.93439308	0.38986663	-4.961679	6.99E-07	2.75E-06
ENSG00000107099	DOCK8	94.98346279	150.7377827	39.22914284	-1.943197802	0.244280989	-7.9547648	1.79E-15	1.31E-14
ENSG00000140511	HAPLN3	2714.333306	4310.797375	1117.869236	-1.946914609	0.054917516	-35.451615	2.74E-275	7.56E-273
ENSG00000279443	AL513497.1	9.296606076	14.72722346	3.86598869	-1.94726386	0.837933343	-2.3238887	0.020131	0.043033
ENSG00000143469	SYT14	264.1029822	419.8517317	108.3542328	-1.953720569	0.151804671	-12.869963	6.64E-38	1.17E-36
ENSG00000170153	RNF150	14.97390589	23.85127986	6.096531916	-1.954511538	0.623957125	-3.1324453	0.001734	0.00451
ENSG00000171004	HS6ST2	56.21832758	89.43950283	22.99715233	-1.95947053	0.326439247	-6.002558	1.94E-09	9.52E-09
ENSG00000226864	ATE1-AS1	14.48944033	23.03049243	5.948388229	-1.962993729	0.672200413	-2.9202507	0.003497	0.008656
ENSG00000205220	PSMB10	255.8551666	407.3631581	104.347175	-1.966831471	0.156955666	-12.531128	5.04E-36	8.39E-35
ENSG00000234993	CUBNP2	22.01611087	35.08535289	8.946868849	-1.969156944	0.503870065	-3.908065	9.30E-05	0.000289
ENSG00000214814	FER1L6	7.632646894	12.18741876	3.077875023	-1.97970058	0.859481032	-2.3033674	0.021258	0.045203
ENSG00000142945	KIF2C	515.7980805	823.2357975	208.3603634	-1.980644865	0.107798263	-18.373625	2.14E-75	8.73E-74
ENSG00000029534	ANK1	44.98076651	71.83789903	18.12363399	-1.981756788	0.371994613	-5.3273803	9.96E-08	4.25E-07
ENSG00000224982	TMEM233	43.5567833	69.61323039	17.50033621	-1.990056435	0.376371235	-5.2874828	1.24E-07	5.26E-07
ENSG00000158856	DMTN	553.567248	884.5319126	222.6025833	-1.993025552	0.114600136	-17.391127	9.63E-68	3.45E-66
ENSG00000163735	CXCL5	2046.85104	3271.38563	822.3164508	-1.993247779	0.064931735	-30.69759	6.13E-207	1.17E-204
ENSG00000183763	TRAIP	93.65394748	149.6631999	37.64469504	-1.993783251	0.257060135	-7.7560966	8.76E-15	6.14E-14
ENSG00000163808	KIF15	299.930045	479.6904718	120.1696182	-1.996227547	0.151949108	-13.137475	2.01E-39	3.70E-38
ENSG00000168078	PBK	295.4303785	472.399364	118.461393	-1.996245857	0.140123317	-14.24635	4.72E-46	1.02E-44
ENSG00000178078	STAP2	294.4850573	471.2148194	117.7552951	-2.000566111	0.144538239	-13.841085	1.44E-43	2.97E-42
ENSG00000158164	TMSB15A	13.40888894	21.53189409	5.285883779	-2.005607899	0.775357267	-2.5866887	0.00969	0.022154
ENSG00000171791	BCL2	220.4182852	353.0790529	87.75751751	-2.006470797	0.184164148	-10.895013	1.22E-27	1.52E-26
ENSG00000174428	GTF2IRD2B	89.19865598	143.0185996	35.37871239	-2.009576553	0.302105864	-6.6518952	2.89E-11	1.62E-10
ENSG00000134775	FHOD3	54.7044169	87.59329499	21.81553881	-2.00981679	0.355118098	-5.659573	1.52E-08	6.92E-08
ENSG00000121966	CXCR4	2254.068715	3613.024985	895.1124452	-2.012792003	0.059336086	-33.921887	3.17E-252	7.55E-250
ENSG00000188483	IER5L	136.0529013	218.2667499	53.83905275	-2.017407461	0.243695394	-8.278398	1.25E-16	9.77E-16
ENSG00000214960	ISPD	77.76109379	124.6271945	30.89499311	-2.018992468	0.288461619	-6.9991719	2.57E-12	1.55E-11
ENSG00000162849	KIF26B	73.04465551	117.2456993	28.84361168	-2.023681215	0.291298639	-6.9471015	3.73E-12	2.22E-11
ENSG00000182010	RTKN2	44.68437517	71.69311794	17.6756324	-2.024621675	0.363163604	-5.5749575	2.48E-08	1.11E-07
ENSG00000235947	EGOT	18.21339054	29.29442114	7.132359946	-2.029860726	0.584202946	-3.4745815	0.000512	0.001442
ENSG00000076382	SPAG5	594.582947	956.6766485	232.4892455	-2.039086546	0.103862832	-19.632495	8.16E-86	4.02E-84
ENSG00000108187	PBLD	127.6325903	205.3552004	49.90998032	-2.044315646	0.228592095	-8.9430724	3.78E-19	3.32E-18
ENSG00000117399	CDC20	800.5766199	1289.458732	311.694508	-2.047779799	0.089984852	-22.756939	1.23E-114	9.68E-113
ENSG00000235863	B3GALT4	51.31406047	82.63311811	19.99500282	-2.048737811	0.336017498	-6.0971164	1.08E-09	5.40E-09
ENSG00000244701	AC004918.1	115.5471138	186.2308684	44.86335917	-2.050476206	0.246853256	-8.306458	9.86E-17	7.74E-16
ENSG00000188763	FZD9	27.52916481	44.31476994	10.74355968	-2.051834036	0.461056971	-4.4502831	8.58E-06	2.99E-05
ENSG00000080709	KCNN2	239.5690179	386.5132238	92.62481205	-2.064821703	0.165614048	-12.467672	1.12E-35	1.84E-34
ENSG00000281501	SEPSECS-AS1	7.152133244	11.56057482	2.743691664	-2.070989843	0.891452305	-2.3231639	0.02017	0.043102
ENSG00000073350	LLGL2	33.70901154	54.50078011	12.91724297	-2.071414486	0.474284681	-4.3674497	1.26E-05	4.31E-05
ENSG00000257167	TMPO-AS1	51.11481045	82.64583763	19.58378326	-2.074274343	0.338369417	-6.1302063	8.78E-10	4.42E-09
ENSG00000137812	KNL1	825.1386811	1333.866912	316.4104499	-2.075461792	0.09222836	-22.50351	3.83E-112	2.88E-110
ENSG00000158402	CDC25C	76.69389927	123.9765367	29.41126186	-2.079270727	0.284847572	-7.2995908	2.89E-13	1.85E-12
ENSG00000146147	MLIP	117.1007062	189.4327426	44.76866971	-2.081612058	0.219615865	-9.4784229	2.58E-21	2.50E-20
ENSG00000151023	ENKUR	158.2629744	256.0466574	60.47929132	-2.084293568	0.199913618	-10.425971	1.89E-25	2.18E-24
ENSG00000024526	DEPDC1	340.8950409	551.7528956	130.0371862	-2.08546037	0.138231631	-15.086709	1.98E-51	4.98E-50
ENSG00000064692	SNCAIP	582.9770767	943.716838	222.2373154	-2.088311126	0.105971759	-19.706299	1.90E-86	9.44E-85
ENSG00000204103	MAFB	27.38189603	44.27737954	10.48641252	-2.089254607	0.484109736	-4.3156633	1.59E-05	5.40E-05
ENSG00000224738	AC099850.1	14.42369538	23.38725829	5.46013246	-2.090316936	0.710377147	-2.9425453	0.003255	0.008097
ENSG00000124225	PMEPA1	3957.645935	6412.149315	1503.142554	-2.093421612	0.053084738	-39.435471	0	0
ENSG00000197093	GAL3ST4	115.0687098	186.4436198	43.69379982	-2.094091303	0.225646753	-9.2803964	1.69E-20	1.57E-19
ENSG00000157456	CCNB2	382.6656463	620.1951565	145.1361362	-2.094939784	0.124160274	-16.872867	7.13E-64	2.36E-62
ENSG00000271856	LINC01215	23.74895639	38.50728568	8.990627103	-2.100573093	0.567167713	-3.703619	0.000213	0.000632
ENSG00000105246	EBI3	480.3374658	779.4053458	181.2695859	-2.10397706	0.112990176	-18.620885	2.18E-77	9.29E-76
ENSG00000169245	CXCL10	164.5530199	267.1323402	61.9736996	-2.104303883	0.205733473	-10.228301	1.48E-24	1.65E-23
ENSG00000119714	GPR68	86.8948079	140.9450718	32.84454402	-2.105032135	0.271370024	-7.7570548	8.69E-15	6.10E-14
ENSG00000121211	MND1	42.78633681	69.46619396	16.10647966	-2.106151715	0.364111409	-5.7843607	7.28E-09	3.42E-08
ENSG00000197299	BLM	431.3934811	700.3389861	162.4479761	-2.108085142	0.116621844	-18.076246	4.90E-73	1.93E-71
ENSG00000186185	KIF18B	340.7600125	553.4647851	128.05524	-2.11080216	0.146548502	-14.403437	4.92E-47	1.09E-45
ENSG00000065320	NTN1	20.9243695	33.98984171	7.858897284	-2.111354221	0.537088084	-3.9311135	8.46E-05	0.000264
ENSG00000154839	SKA1	300.0607539	487.3560723	112.7654355	-2.111493824	0.142269899	-14.841466	7.90E-50	1.89E-48
ENSG00000167895	TMC8	11.91031166	19.35963523	4.46098809	-2.112611154	0.689674801	-3.063199	0.00219	0.005618
ENSG00000023445	BIRC3	349.284756	567.4422593	131.1272527	-2.114826409	0.141625667	-14.932508	2.03E-50	4.92E-49
ENSG00000230805	AL132709.1	9.151873792	14.8964056	3.407341987	-2.118284311	0.841661331	-2.5167894	0.011843	0.026568
ENSG00000166670	MMP10	1733.895812	2820.847368	646.9442554	-2.12470834	0.074784728	-28.410992	1.48E-177	2.25E-175
ENSG00000164684	ZNF704	973.2149195	1583.656674	362.773165	-2.126416018	0.086319588	-24.634223	5.43E-134	5.31E-132
ENSG00000069482	GAL	181.2146373	295.2157413	67.21353338	-2.13441018	0.200648703	-10.637548	1.99E-26	2.38E-25
ENSG00000277763	AL138995.1	7.452118715	12.1332949	2.77094253	-2.135615202	0.870901024	-2.4521905	0.014199	0.031358
ENSG00000112294	ALDH5A1	10.03712525	16.39901788	3.675232621	-2.135882491	0.81015053	-2.636402	0.008379	0.019391
ENSG00000272644	AC097468.3	9.369826253	15.23273984	3.506912662	-2.138239047	0.830857091	-2.5735341	0.010067	0.022939
ENSG00000126787	DLGAP5	1180.243222	1924.997494	435.4889488	-2.143208261	0.076829769	-27.895545	3.02E-171	4.43E-169
ENSG00000029993	HMGB3	1419.327795	2315.75028	522.9053093	-2.148623289	0.080199556	-26.790962	4.12E-158	5.19E-156
ENSG00000259985	AC017100.1	40.26370113	65.89050467	14.6368976	-2.162046239	0.388051186	-5.5715491	2.52E-08	1.13E-07
ENSG00000170745	KCNS3	17.99945744	29.39630911	6.602605759	-2.164133633	0.585171918	-3.6982869	0.000217	0.000644
ENSG00000143228	NUF2	267.6045333	438.0676854	97.14138129	-2.168901375	0.160472784	-13.515696	1.26E-41	2.49E-40
ENSG00000140092	FBLN5	1740.362159	2848.000472	632.7238469	-2.170595339	0.064441927	-33.682967	1.03E-248	2.41E-246
ENSG00000266714	MYO15B	46.5803179	76.27442776	16.88620805	-2.177308383	0.39658427	-5.4901532	4.02E-08	1.77E-07

ENSG00000042980	ADAM28	70.72664601	116.1071602	25.34613181	-2.191683552	0.289010089	-7.5834154	3.37E-14	2.28E-13
ENSG00000280143	AP000892.3	48.85985224	80.24661913	17.47308534	-2.196016669	0.367106265	-5.9819646	2.20E-09	1.08E-08
ENSG00000197580	BCO2	6.71075404	11.05235232	2.369155759	-2.204694219	0.944981777	-2.3330547	0.019645	0.042083
ENSG00000180921	FAM83H	9.458623791	15.59401639	3.323231188	-2.207230736	0.851196247	-2.5930927	0.009512	0.021774
ENSG00000123610	TNFAIP6	146.3228451	240.6913711	51.95431906	-2.208715678	0.221450363	-9.9738635	1.98E-23	2.10E-22
ENSG00000148773	MKI67	3736.811759	6146.989745	1326.633774	-2.21242204	0.047411532	-46.664218	0	0
ENSG00000162650	ATXN7L2	95.25803208	156.7895013	33.72656282	-2.216026316	0.265084245	-8.3597059	6.29E-17	4.98E-16
ENSG00000080986	NDC80	422.2026205	695.3321734	149.0730675	-2.219493681	0.139226887	-15.941559	3.26E-57	9.24E-56
ENSG00000265107	GJA5	196.2554034	323.2955791	69.21522783	-2.223167109	0.178309323	-12.468036	1.12E-35	1.84E-34
ENSG00000167984	NLRC3	202.9913528	334.3866295	71.59607622	-2.228190802	0.190583427	-11.69142	1.41E-31	2.02E-30
ENSG00000075218	GTSE1	606.0961128	999.0235226	213.168703	-2.229274407	0.103865345	-21.46312	3.44E-102	2.26E-100
ENSG00000204261	PSMB8-AS1	28.28337678	46.61602394	9.950729615	-2.229290757	0.474924169	-4.6939931	2.68E-06	9.87E-06
ENSG00000215784	FAM72D	9.604007361	15.88714173	3.320872989	-2.235798711	0.865598652	-2.5829508	0.009796	0.022379
ENSG00000104081	BMF	825.614786	1362.271762	288.9578102	-2.23728196	0.088451651	-25.293841	3.73E-141	4.11E-139
ENSG00000115163	CENPA	133.0205365	219.5587757	46.48229725	-2.240158427	0.223398009	-10.027656	1.15E-23	1.23E-22
ENSG00000178665	ZNF713	59.07763574	97.59487832	20.56039316	-2.244031786	0.315039341	-7.1230208	1.06E-12	6.54E-12
ENSG00000232517	AC112198.2	117.7646809	194.4558378	41.07352399	-2.246198338	0.272200125	-8.2520107	1.56E-16	1.21E-15
ENSG00000275993	SIK1B	193.0672393	318.9350944	67.19938419	-2.247162254	0.183990664	-12.213458	2.63E-34	4.17E-33
ENSG00000066279	ASPM	1207.994486	1998.026433	417.9625395	-2.254958663	0.090390969	-24.946725	2.32E-137	2.45E-135
ENSG00000258602	LINC01629	9.13538248	15.08624467	3.184520293	-2.261166138	0.907007635	-2.4929957	0.012667	0.02829
ENSG00000148680	HTR7	10.90930016	18.08650776	3.732092554	-2.268237086	0.768810301	-2.9503209	0.003174	0.007914
ENSG00000138134	STAMBPL1	18.88459323	31.34318758	6.425998879	-2.272754865	0.574398893	-3.9567536	7.60E-05	0.000239
ENSG00000213088	ACKR1	100.1383778	166.0062894	34.27046619	-2.27314428	0.244625521	-9.2923431	1.51E-20	1.41E-19
ENSG00000132185	FCRLA	26.34326933	43.75512969	8.931408973	-2.293049354	0.497771966	-4.6066261	4.09E-06	1.48E-05
ENSG00000135378	PRRG4	17.4486692	29.10773897	5.789599423	-2.321469217	0.596523729	-3.8916628	9.96E-05	0.000308
ENSG00000056558	TRAF1	907.9907224	1512.9008	303.0806447	-2.321896773	0.095265586	-24.372881	3.32E-131	3.14E-129
ENSG00000228613	AC141930.1	11.31948891	18.88199259	3.756985222	-2.327274806	0.742164889	-3.1357921	0.001714	0.004466
ENSG00000143851	PTPN7	25.84014936	43.15539292	8.524905805	-2.332839035	0.501416698	-4.6524957	3.28E-06	1.20E-05
ENSG00000211772	TRBC2	371.3502836	620.0091964	122.6913709	-2.33469752	0.133125651	-17.537548	7.41E-69	2.73E-67
ENSG00000233427	AL009181.1	18.00321492	30.01061448	5.995815368	-2.336670925	0.676293736	-3.4551125	0.00055	0.001544
ENSG00000165124	SVEP1	11.50480382	19.19812068	3.811486956	-2.341596483	0.737485851	-3.175107	0.001498	0.003943
ENSG00000187068	C3orf70	12.51969888	20.9280529	4.111334854	-2.346219059	0.693434989	-3.3834737	0.000716	0.001981
ENSG00000165685	TMEM52B	13.4459652	22.51505312	4.376877291	-2.346435707	0.733636959	-3.1983608	0.001382	0.00366
ENSG00000260643	AC092718.3	9.438595182	15.78385546	3.093334899	-2.35297682	0.859387876	-2.7379684	0.006182	0.014641
ENSG00000080224	EPHA6	12.34520223	20.71295574	3.977448718	-2.356531006	0.792986856	-2.9717151	0.002961	0.007427
ENSG00000162882	HAAO	45.71336928	76.48641253	14.94032602	-2.363540288	0.385705603	-6.127835	8.91E-10	4.49E-09
ENSG00000125637	PSD4	11.76008467	19.71811518	3.802054164	-2.379287975	0.721237973	-3.2988945	0.000971	0.002638
ENSG00000110077	MS4A6A	53.71963679	90.22700428	17.2122693	-2.389976567	0.335257747	-7.1287736	1.01E-12	6.28E-12
ENSG00000171631	P2RY6	72.14812431	121.2102691	23.08597952	-2.393537978	0.316489696	-7.5627675	3.95E-14	2.66E-13
ENSG00000111665	CDCA3	154.9183779	260.4434945	49.39326136	-2.396211568	0.199683352	-12.000057	3.55E-33	5.41E-32
ENSG00000082497	SERTAD4	191.2740687	321.3399505	61.20818686	-2.396285588	0.184074085	-13.01805	9.66E-39	1.74E-37
ENSG00000185101	ANO9	19.58672966	32.911064	6.262395316	-2.406206428	0.637073539	-3.7769681	0.000159	0.000479
ENSG00000100336	APOL4	11.86367145	19.96564129	3.761701619	-2.408619161	0.791495583	-3.0431239	0.002341	0.005974
ENSG00000164061	BSN	14.25343761	23.97120912	4.535666097	-2.41266112	0.665028089	-3.6279086	0.000286	0.000835
ENSG00000214578	HMGN2P15	11.80015455	19.90254201	3.697767092	-2.413416379	0.76572837	-3.1517918	0.001623	0.004247
ENSG00000137462	TLR2	234.6015709	395.2804239	73.92271796	-2.415944075	0.173201196	-13.948773	3.20E-44	6.70E-43
ENSG00000149591	TAGLN	3296.589295	5553.528545	1039.650046	-2.41779691	0.051515651	-46.933249	0	0
ENSG00000259456	ADNP-AS1	9.801291161	16.5270655	3.075516825	-2.422009124	0.82339144	-2.9415039	0.003266	0.00812
ENSG00000168140	VASN	30.59402048	51.59167097	9.596369983	-2.422468987	0.449736069	-5.3864236	7.19E-08	3.10E-07
ENSG00000230337	EXOSC10-AS1	7.7288067	13.00906324	2.448550162	-2.424454888	0.892763226	-2.7156751	0.006614	0.015587
ENSG00000184371	CSF1	4942.756041	8339.725576	1545.786507	-2.432013017	0.050788891	-47.884743	0	0
ENSG00000172602	RND1	2189.112404	3697.852867	680.3719414	-2.442361875	0.066892855	-36.511551	7.27E-292	2.20E-289
ENSG00000255690	TRIL	13.42530641	22.66930635	4.181306464	-2.444302238	0.765128342	-3.1946304	0.0014	0.003703
ENSG00000162366	PDZK1IP1	7.793361146	13.1677812	2.418941096	-2.452677594	0.905358417	-2.7090681	0.006747	0.015863
ENSG00000226496	LINC00323	27.98900544	47.33099993	8.647010951	-2.45292316	0.488750145	-5.0187671	5.20E-07	2.08E-06
ENSG00000268883	PNMA6B	11.43717009	19.35196765	3.522372538	-2.471539986	0.83995389	-2.942471	0.003256	0.008098
ENSG00000154451	GBP5	111.3342277	188.8482505	33.82020477	-2.476212571	0.255141512	-9.7052516	2.86E-22	2.90E-21
ENSG00000049249	TNFRSF9	25.98613972	44.05416402	7.918115414	-2.476509659	0.48771255	-5.0778059	3.82E-07	1.55E-06
ENSG00000162692	VCAM1	2036.490534	3455.306222	617.674847	-2.484664866	0.064084229	-38.771862	0	0
ENSG00000237499	AL357060.1	12.47639507	21.20052131	3.752268826	-2.486471721	0.732188819	-3.3959433	0.000684	0.001897
ENSG00000233452	STXBP5-AS1	89.77439789	152.4308658	27.11792998	-2.488108632	0.263238399	-9.4519213	3.33E-21	3.21E-20
ENSG00000099958	DERL3	11.33066638	19.25634896	3.404983788	-2.489776148	0.748559561	-3.3260896	0.000881	0.002403
ENSG00000054690	PLEKHH1	5.685937813	9.659295595	1.71258003	-2.489949569	1.046933585	-2.3783262	0.017391	0.037666
ENSG00000271874	AC025754.2	6.837587868	11.61059427	2.064581464	-2.495688951	0.974855623	-2.5600601	0.010465	0.02376
ENSG00000211751	TRBC1	8.890774615	15.16467911	2.616870121	-2.503171961	0.969338478	-2.5823508	0.009813	0.022413
ENSG00000250240	AC008840.1	5.760829508	9.784186317	1.737472699	-2.504660058	1.038543888	-2.4117036	0.015878	0.034689
ENSG00000145113	MUC4	6.830736488	11.64667685	2.014796127	-2.515601776	1.012001615	-2.4857685	0.012927	0.028821
ENSG00000183856	IQGAP3	551.198983	938.3182133	164.0797527	-2.517812942	0.119241396	-21.115259	5.76E-99	3.66E-97
ENSG00000139055	ERP27	13.94583774	23.73761988	4.154055598	-2.51825485	0.682116687	-3.6918241	0.000223	0.000659
ENSG00000100399	CHADL	5.867949568	9.998426438	1.737472699	-2.530253023	1.032520823	-2.4505588	0.014263	0.031482
ENSG00000166927	MS4A7	7.021412214	11.97588476	2.066939663	-2.539117343	0.943299277	-2.691741	0.007108	0.016648
ENSG00000180596	HIST1H2BC	7.074162598	12.1062782	2.042046994	-2.552778168	0.964829135	-2.6458345	0.008149	0.018918
ENSG00000157404	KIT	2982.171032	5103.05487	861.2871944	-2.566425759	0.06436759	-39.871398	0	0
ENSG00000135547	HEY2	85.91255689	147.0786975	24.74641602	-2.570578641	0.270154309	-9.5152236	1.81E-21	1.77E-20
ENSG00000159753	CARMIL2	9.649243634	16.50501027	2.793477001	-2.571170553	0.85105297	-3.0211639	0.002518	0.00639
ENSG00000135914	HTR2B	51.72890283	88.47136318	14.98644247	-2.571477749	0.369017397	-6.9684459	3.20E-12	1.92E-11
ENSG00000096996	IL12RB1	6.070534772	10.37634598	1.764723566	-2.572382421	1.053973447	-2.440652	0.014661	0.032285
ENSG00000237989	LINC01679	15.39145412	26.42856543	4.354342821	-2.578531064	0.67862844	-3.7996213	0.000145	0.000439
ENSG00000187689	AMTN	553.4442423	949.6932791	157.1952055	-2.594395247	0.110192263	-23.54426	1.44E-122	1.23E-120
ENSG00000189120	SP6	138.8512936	238.4709878	39.2315994	-2.597056403	0.226130276	-11.48478	1.57E-30	2.18E-29
ENSG00000160224	AIRE	6.069524156	10.42411008	1.714938229	-2.603948786	1.024217188	-2.5423795	0.01101	0.024863
ENSG00000103888	CEMIP	31.58020133	54.27039374	8.890008917	-2.604742849	0.462219155	-5.6352984	1.75E-08	7.93E-08
ENSG00000143494	VASH2	38.25021691	65.77233401	10.7280998	-2.62057544	0.442155536	-5.9268181	3.09E-09	1.49E-08
ENSG00000138135	CH25H	23.23333648	40.07128315	6.396389814	-2.627762265	0.561051676	-4.6836368	2.82E-06	1.04E-05
ENSG00000245614	DDX11-AS1	4.978384338	8.566581014	1.390187662	-2.633457251	1.159049586	-2.2720833	0.023081	0.048662

ENSG00000197291	RAMP2-AS1	56.75385813	97.96652858	15.54118769	-2.641791058	0.365345654	-7.230936	4.80E-13	3.04E-12
ENSG00000140479	PCSK6	14.98291638	25.82959523	4.136237523	-2.647403296	0.66705831	-3.9687734	7.22E-05	0.000227
ENSG00000109846	CRYAB	39.38593652	68.0780987	10.69377434	-2.67355576	0.407592653	-6.5593816	5.40E-11	2.97E-10
ENSG00000272512	AL645608.7	17.72722547	30.68653035	4.767920583	-2.680007989	0.62940971	-4.2579705	2.06E-05	6.92E-05
ENSG00000054392	HHAT	39.52163053	68.48574106	10.55752001	-2.690600966	0.413649527	-6.5045426	7.79E-11	4.24E-10
ENSG00000164619	BMPER	9.031059947	15.6431788	2.418941096	-2.696898182	0.839261567	-3.2134179	0.001312	0.00349
ENSG00000104951	IL4I1	800.0427542	1388.161569	211.9239392	-2.713295903	0.098547569	-27.532855	7.10E-167	1.02E-164
ENSG00000253520	AC136628.3	5.205556191	9.025641116	1.385471265	-2.715839265	1.129163946	-2.4051771	0.016165	0.03523
ENSG00000278828	HIST1H3H	6.486834878	11.26108972	1.71258003	-2.718357299	1.008089615	-2.6965433	0.007006	0.016434
ENSG00000142661	MYOM3	79.3774439	137.8721023	20.88278553	-2.719914545	0.294880437	-9.2237877	2.87E-20	2.64E-19
ENSG00000248636	AC002070.1	7.633548256	13.30444392	1.962652591	-2.723610363	1.012643582	-2.6896041	0.007154	0.016744
ENSG00000196208	GREB1	5.351485369	9.292606804	1.410363934	-2.74662254	1.164436411	-2.358757	0.018336	0.039573
ENSG00000275055	AC011468.5	9.369782687	16.29573161	2.443833765	-2.749096072	0.867370787	-3.1694589	0.001527	0.004013
ENSG00000164932	CTHRC1	6096.182218	10614.55845	1577.805988	-2.74933985	0.045585668	-60.311496	0	0
ENSG00000273312	AL121749.1	5.386266314	9.359810495	1.412722132	-2.75521424	1.146141721	-2.4039036	0.016221	0.03534
ENSG00000164742	ADCY1	13.37634446	23.32988705	3.422801863	-2.767495345	0.720371545	-3.8417611	0.000122	0.000373
ENSG00000100027	YPEL1	43.94486146	76.75973798	11.12998494	-2.769544052	0.415692873	-6.6624766	2.69E-11	1.51E-10
ENSG00000116147	TNR	67.32568473	117.3963894	17.25498004	-2.769563146	0.3258882	-8.498507	1.92E-17	1.56E-16
ENSG00000260442	ATP2A1-AS1	11.98560685	20.95019861	3.021015091	-2.7705665	0.764746584	-3.6228557	0.000291	0.00085
ENSG00000231871	IPO9-AS1	5.413715928	9.464495061	1.362936795	-2.782451497	1.140805568	-2.4390234	0.014727	0.032419
ENSG00000151617	EDNRA	14.88785234	26.02579406	3.749910628	-2.788388382	0.684223143	-4.0752617	4.60E-05	0.000148
ENSG00000143869	GDF7	238.9058993	417.3728598	60.43893877	-2.791883301	0.179543154	-15.549929	1.59E-54	4.33E-53
ENSG00000240288	GHRLOS	9.673285034	16.89330351	2.453266558	-2.79471421	0.903016472	-3.0948652	0.001969	0.005082
ENSG00000230838	LINC01614	11.25491753	19.68203261	2.827802462	-2.823126615	0.804570554	-3.5088615	0.00045	0.001279
ENSG00000165478	HEPACAM	5.62586408	9.843722424	1.408005736	-2.824676869	1.187231257	-2.3792137	0.01735	0.037593
ENSG00000237548	TTLL11-IT1	6.956365349	12.19779247	1.714938229	-2.826117661	0.973015993	-2.9044925	0.003678	0.009068
ENSG00000119681	LTBP2	27898.58845	48917.08733	6880.089566	-2.830013204	0.030605291	-92.468103	0	0
ENSG00000198535	C2CD4A	834.7468107	1464.063806	205.4298158	-2.83471384	0.106738005	-26.55768	2.09E-155	2.56E-153
ENSG00000165449	SLC16A9	12.39478498	21.79344754	2.996122422	-2.837623878	0.780550906	-3.6354117	0.000278	0.000812
ENSG00000235618	FAM21EP	16.9232808	29.71739867	4.129162929	-2.849504914	0.662164277	-4.3033202	1.68E-05	5.70E-05
ENSG00000258875	AL135818.1	5.620191799	9.879805001	1.360578596	-2.850446313	1.093020571	-2.6078615	0.009111	0.020927
ENSG00000008735	MAPK8IP2	9.989841648	17.52641674	2.453266558	-2.852137289	0.880972578	-3.237487	0.001206	0.00323
ENSG00000155966	AFF2	33.17498423	58.41167677	7.938291687	-2.880864906	0.454848494	-6.3336802	2.39E-10	1.26E-09
ENSG00000078098	FAP	241.4817908	425.7245246	57.23905697	-2.891975554	0.173292779	-16.688379	1.59E-62	5.11E-61
ENSG00000182545	RNASE10	29.27822713	51.5628052	6.993649051	-2.894630946	0.5227951	-5.5368364	3.08E-08	1.37E-07
ENSG00000010671	BTK	14.58904303	25.7434932	3.434592853	-2.905578149	0.716620853	-4.0545543	5.02E-05	0.000161
ENSG00000233588	CYP51A1P2	4.3703909	7.734562836	1.006218964	-2.916636085	1.258656997	-2.3172605	0.02049	0.043715
ENSG00000079385	CEACAM1	118.0458688	208.5175191	27.57421848	-2.921559428	0.25334208	-11.532073	9.09E-31	1.26E-29
ENSG00000211767	TRBJ2-3	7.294659536	12.92652438	1.662794693	-2.93173298	1.006646561	-2.9123757	0.003587	0.008862
ENSG00000203685	STUM	133.0987871	235.8476967	30.34987741	-2.960386818	0.228988866	-12.928082	3.13E-38	5.55E-37
ENSG00000179750	APOBEC3B	69.16316928	122.7484672	15.57787135	-2.966100877	0.348969448	-8.4996004	1.90E-17	1.55E-16
ENSG00000114631	PODXL2	194.4585465	345.0384659	43.87862717	-2.971251882	0.193203973	-15.378834	2.27E-53	6.05E-52
ENSG00000138944	SHISAL1	33.31365749	59.14767	7.479644983	-2.972532015	0.504575095	-5.8911588	3.83E-09	1.84E-08
ENSG00000183032	SLC25A21	26.11840741	46.3513136	5.885501213	-2.984855723	0.566019418	-5.2734158	1.34E-07	5.66E-07
ENSG00000186827	TNFRSF4	66.87017912	118.7088468	15.03151141	-2.993474838	0.351530778	-8.5155412	1.66E-17	1.35E-16
ENSG00000152932	RAB3C	27.80104967	49.40128035	6.200818988	-2.995653956	0.502236901	-5.9646234	2.45E-09	1.19E-08
ENSG00000108700	CCL8	7.687751952	13.66292387	1.71258003	-2.998470416	0.981237958	-3.0558035	0.002245	0.005751
ENSG00000115461	IGFBP5	40.17849758	71.65076608	8.706229081	-3.050138857	0.444115235	-6.8678996	6.52E-12	3.83E-11
ENSG00000254231	AC103760.1	4.844429496	8.65538916	1.033469831	-3.075022523	1.236491239	-2.4868939	0.012886	0.028739
ENSG00000146267	FAXC	26.21136989	46.82399478	5.598744993	-3.079959899	0.540028954	-5.7033236	1.17E-08	5.41E-08
ENSG00000112984	KIF20A	634.7986624	1138.479603	131.1177216	-3.120664161	0.114538415	-27.245568	1.88E-163	2.55E-161
ENSG00000189058	APOD	207.1813494	372.5036825	41.85901628	-3.158552832	0.193843207	-16.294369	1.08E-59	3.27E-58
ENSG00000167034	NKX3-1	164.3886277	295.6836863	33.09356907	-3.160252589	0.223386884	-14.146993	1.95E-45	4.19E-44
ENSG00000148848	ADAM12	597.322554	1076.508202	118.1369056	-3.189159214	0.117577577	-27.123873	5.15E-162	6.76E-160
ENSG00000169248	CXCL11	134.3225067	242.2855881	26.35942537	-3.196525408	0.240720053	-13.279016	3.06E-40	5.79E-39
ENSG00000248458	AL139147.1	36.78865795	66.38573782	7.191578076	-3.200642871	0.489311538	-6.5411147	6.11E-11	3.34E-10
ENSG00000128594	LRRC4	22.82298367	41.23476458	4.411202753	-3.209492044	0.586510739	-5.4721795	4.45E-08	1.95E-07
ENSG00000261239	ANKRD26P1	51.39746537	92.80280108	9.992129671	-3.218302449	0.405483572	-7.936949	2.07E-15	1.51E-14
ENSG00000162654	GBP4	1395.601492	2521.747793	269.4551919	-3.224477985	0.084991522	-37.938819	0	0
ENSG00000110042	DTX4	42.71895792	77.16544079	8.272475046	-3.226177303	0.438073967	-7.3644579	1.78E-13	1.16E-12
ENSG00000187513	GJA4	133.7427245	241.6547302	25.8307187	-3.227332673	0.247698166	-13.029296	8.34E-39	1.51E-37
ENSG00000166920	C15orf48	279.1795842	504.7040602	53.6551081	-3.227590634	0.17553406	-18.387261	1.66E-75	6.81E-74
ENSG00000133401	PDZD2	253.9743992	459.0213567	48.9274417	-3.231451783	0.17358762	-18.615681	2.40E-77	1.02E-75
ENSG00000111110	PPM1H	229.3344322	414.8506012	43.81826316	-3.245006954	0.188945762	-17.174277	4.14E-66	1.42E-64
ENSG00000124102	PI3	112.308092	203.501144	21.11504001	-3.258896748	0.267773953	-12.170328	4.47E-34	7.03E-33
ENSG00000130208	APOC1	7.360110047	13.30985616	1.410363934	-3.262306176	1.072315866	-3.0422996	0.002348	0.005988
ENSG00000125144	MT1G	3.663011387	6.644554376	0.681468397	-3.278255285	1.446174199	-2.2668468	0.0234	0.049256
ENSG00000166257	SCN3B	42.58717237	77.23133666	7.943008083	-3.287049574	0.431488695	-7.6179275	2.58E-14	1.76E-13
ENSG00000173825	TIGD3	3.709467668	6.737466939	0.681468397	-3.294709766	1.436210918	-2.2940292	0.021789	0.046238
ENSG00000232810	TNF	3.79719338	6.912918362	0.681468397	-3.326094583	1.459904003	-2.2782968	0.022709	0.047966
ENSG00000175445	LPL	581.986231	1058.701605	105.270857	-3.330744986	0.142567778	-23.362537	1.03E-120	8.70E-119
ENSG00000171236	LRG1	28.5893343	52.06110478	5.117563819	-3.338206621	0.52404862	-6.3700323	1.89E-10	1.00E-09
ENSG00000080573	COL5A3	3.88381184	7.061262614	0.706361066	-3.346286897	1.421560594	-2.3539531	0.018575	0.040002
ENSG00000143816	WNT9A	84.54993179	154.1015318	14.99833182	-3.353508293	0.324951229	-10.320036	5.72E-25	6.47E-24
ENSG00000172164	SNTB1	120.1387739	219.212293	21.06525468	-3.368355841	0.269590297	-12.494351	8.01E-36	1.33E-34
ENSG00000099617	EFNA2	35.03889209	63.91967593	6.158108244	-3.372404313	0.515225356	-6.5454937	5.93E-11	3.25E-10
ENSG00000173862	AC008080.1	3.952797114	7.199233162	0.706361066	-3.37919211	1.453783059	-2.3244129	0.020103	0.042978
ENSG00000170054	SERPINA9	13.81442542	25.21226795	2.416582898	-3.386554278	0.774129277	-4.3746624	1.22E-05	4.17E-05
ENSG00000275713	HIST1H2BH	13.90783836	25.37184295	2.443833765	-3.394020107	0.786336907	-4.3162416	1.59E-05	5.38E-05
ENSG00000168398	BDKRB2	39.91687052	72.85659938	6.977141664	-3.397253852	0.467799885	-7.2621947	3.81E-13	2.43E-12
ENSG00000270885	RASL10B	6.023108625	10.98785475	1.0583625	-3.397955488	1.162179776	-2.9237779	0.003458	0.008565
ENSG00000139044	B4GALNT3	67.60252254	123.5346608	11.67038424	-3.400428279	0.346973851	-9.8002437	1.12E-22	1.16E-21
ENSG00000279168	AC105052.4	3.996526854	7.311585311	0.681468397	-3.40644277	1.497117649	-2.2753341	0.022886	0.048297
ENSG00000187554	TLR5	6.018381302	11.00093457	1.035828029	-3.40952737	1.193948848	-2.8556729	0.004295	0.010465
ENSG00000255921	AC026310.2	4.07141855	7.436476034	0.706361066	-3.418710513	1.434795423	-2.3827164	0.017185	0.037262

ENSG00000128342	LIF	97.50020464	178.494501	16.50590824	-3.434269873	0.289975662	-11.843304	2.33E-32	3.46E-31
ENSG00000162881	OXER1	4.066865188	7.452261979	0.681468397	-3.438644152	1.458032167	-2.3584145	0.018353	0.039605
ENSG00000213940	AC026477.1	8.273992722	15.23955038	1.308435061	-3.494448422	1.048604459	-3.3324753	0.000861	0.00235
ENSG00000148798	INA	10.58304386	19.4511495	1.714938229	-3.500729729	0.878017867	-3.9870826	6.69E-05	0.000211
ENSG00000176532	PRR15	8.720723606	16.05361775	1.387829463	-3.534754363	1.010362026	-3.4985028	0.000468	0.001327
ENSG00000184344	GDF3	71.88128264	132.3375372	11.42502808	-3.539468119	0.345844701	-10.23427	1.39E-24	1.55E-23
ENSG00000125910	S1PR4	15.31047737	28.22926451	2.39169023	-3.552673486	0.742215522	-4.7865793	1.70E-06	6.42E-06
ENSG00000175175	PPM1E	6.596410762	12.16170989	1.031111633	-3.558429337	1.135710829	-3.1332178	0.001729	0.0045
ENSG00000112299	VNN1	24.35458855	44.90476474	3.804412362	-3.563746031	0.601271635	-5.9270151	3.08E-09	1.49E-08
ENSG00000188322	SBK1	6.682084265	12.3330569	1.031111633	-3.58129406	1.138648598	-3.1452145	0.00166	0.004334
ENSG00000269155	AL009178.2	13.49044332	24.94119784	2.039688796	-3.602457126	0.811546352	-4.4390035	9.04E-06	3.14E-05
ENSG00000227507	LTB	167.098646	309.055018	25.14227407	-3.608204165	0.241284721	-14.954134	1.46E-50	3.57E-49
ENSG00000229056	HECW2-AS1	4.583454425	8.485440453	0.681468397	-3.623412757	1.394211696	-2.5988971	0.009352	0.021445
ENSG00000197444	OGDHL	11.58414751	21.45571499	1.71258003	-3.64156462	0.861807682	-4.2254957	2.38E-05	7.95E-05
ENSG00000196275	GTF2IRD2	16.54688951	30.64287066	2.45090836	-3.659741666	0.747241976	-4.8976661	9.70E-07	3.77E-06
ENSG00000183307	TMEM121B	21.25146322	39.35508981	3.147836633	-3.660466515	0.654104567	-5.5961488	2.19E-08	9.87E-08
ENSG00000230067	HSPD1P6	11.85559546	21.96664362	1.744547294	-3.669355062	0.885084473	-4.1457682	3.39E-05	0.000111
ENSG00000185090	MANEAL	33.11867711	61.39475563	4.842598589	-3.67065339	0.51882546	-7.0749292	1.50E-12	9.15E-12
ENSG00000228741	AL445985.1	11.88401045	22.0234736	1.744547294	-3.672476453	0.888203675	-4.1347233	3.55E-05	0.000116
ENSG00000283511	AC137936.2	14.2541185	26.43893913	2.069297861	-3.675675428	0.790793953	-4.6480824	3.35E-06	1.22E-05
ENSG00000146122	DAAM2	4.853074806	9.027039414	0.679110199	-3.716003832	1.357466314	-2.7374556	0.006192	0.014662
ENSG00000113396	SLC27A6	9.726023912	18.09146923	1.360578596	-3.722167448	0.954299558	-3.9004183	9.60E-05	0.000297
ENSG00000273802	HIST1H2BG	7.247299047	13.5132718	0.981326296	-3.733970223	1.219359883	-3.062238	0.002197	0.005635
ENSG00000166016	ABTB2	59.3977208	110.482614	8.312827591	-3.739956565	0.394632333	-9.4770657	2.62E-21	2.53E-20
ENSG00000113361	CDH6	9.885267056	18.40759732	1.362936795	-3.746697371	0.963234374	-3.8897048	0.0001	0.00031
ENSG00000178573	MAF	87.78031352	163.4790232	12.0816038	-3.762158501	0.324053472	-11.609684	3.68E-31	5.20E-30
ENSG00000255202	AL049629.1	7.547530327	14.06159082	1.033469831	-3.77253463	1.108016345	-3.4047644	0.000662	0.00184
ENSG00000075223	SEMA3C	32.92786641	61.36277747	4.492955353	-3.776164569	0.532467056	-7.0918276	1.32E-12	8.13E-12
ENSG00000165323	FAT3	199.4544266	371.875034	27.03381918	-3.776213345	0.219210987	-17.226387	1.68E-66	5.87E-65
ENSG00000174640	SLCO2A1	70.24083367	131.1330117	9.348655621	-3.815894396	0.371450093	-10.272967	9.33E-25	1.05E-23
ENSG00000058866	DGKG	5.30338489	9.927659581	0.679110199	-3.849404146	1.400154325	-2.7492713	0.005973	0.014184
ENSG00000151136	BTBD11	53.23573563	99.61879293	6.85267832	-3.857911159	0.421224794	-9.1587941	5.25E-20	4.78E-19
ENSG00000124635	HIST1H2BJ	2.865302154	5.403495543	0.327108765	-3.868948224	1.6868918	-2.2935367	0.021817	0.046288
ENSG00000166923	GREM1	183.8104678	344.4046305	23.21630514	-3.883210349	0.233463273	-16.633067	4.02E-62	1.28E-60
ENSG00000149527	PLCH2	2.916363225	5.478366818	0.354359632	-3.885719303	1.665972193	-2.3324035	0.019679	0.042133
ENSG00000274818	AC004825.2	2.921550077	5.488740523	0.354359632	-3.888046012	1.65139665	-2.3543986	0.018553	0.039963
ENSG00000154856	APCDD1	19.49063515	36.61683094	2.364439363	-3.932037035	0.723926285	-5.4315434	5.59E-08	2.43E-07
ENSG00000133985	TTC9	3.011231333	5.670461232	0.352001434	-3.940250616	1.704280602	-2.3119729	0.020779	0.044288
ENSG00000137571	SLCO5A1	3.044833178	5.737664922	0.352001434	-3.955054405	1.631737968	-2.4238294	0.015358	0.033652
ENSG00000104415	CCN4	3.036220636	5.745332507	0.327108765	-3.95650861	1.667951282	-2.3720768	0.017688	0.038249
ENSG00000231550	PTCHD3P2	5.691458936	10.70144948	0.681468397	-3.965808659	1.330392565	-2.9809312	0.002874	0.007225
ENSG00000072858	SIDT1	3.059448777	5.791788788	0.327108765	-3.966378651	1.668023608	-2.3778912	0.017412	0.037694
ENSG00000172575	RASGRP1	3.078261062	5.802162493	0.354359632	-3.968517499	1.637004081	-2.4242563	0.01534	0.033617
ENSG00000142149	HUNK	8.675025852	16.31422368	1.035828029	-3.980873115	1.091891736	-3.6458497	0.000267	0.000782
ENSG00000198771	RCSD1	29.55844168	55.60761251	3.50927086	-4.004448297	0.605646286	-6.6118597	3.80E-11	2.11E-10
ENSG00000170961	HAS2	145.82993	274.8375865	16.82227352	-4.027320319	0.268976764	-14.972744	1.11E-50	2.71E-49
ENSG00000043462	LCP2	27.16083256	51.1667539	3.154911228	-4.042157462	0.62390255	-6.4788282	9.24E-11	4.99E-10
ENSG00000105639	JAK3	493.5843062	931.0377033	56.13090913	-4.046772874	0.14972727	-27.027627	7.00E-161	9.07E-159
ENSG00000116132	PRRX1	66.4457616	125.2874149	7.604108327	-4.048172538	0.399420902	-10.135104	3.86E-24	4.21E-23
ENSG00000013293	SLC7A14	64.09178632	120.8497132	7.333859494	-4.059677348	0.436609012	-9.2981987	1.43E-20	1.34E-19
ENSG00000278993	AC002350.1	3.373815705	6.395629976	0.352001434	-4.111094152	1.600175902	-2.5691514	0.010195	0.023193
ENSG00000129214	SHBG	3.369036955	6.410965144	0.327108765	-4.113744521	1.617116658	-2.5438762	0.010963	0.024772
ENSG00000163121	NEURL3	37.76817739	71.46169358	4.074661195	-4.119032634	0.539527243	-7.6345221	2.27E-14	1.55E-13
ENSG00000100206	DMC1	6.493346197	12.28033133	0.706361066	-4.146472819	1.281550812	-3.2355118	0.001214	0.003251
ENSG00000184261	KCNK12	3.438022229	6.548935692	0.327108765	-4.150681285	1.608310893	-2.5807705	0.009858	0.022505
ENSG00000131242	RAB11FIP4	3.499457004	6.644554376	0.354359632	-4.168613281	1.591133211	-2.6199021	0.008796	0.020268
ENSG00000170075	GPR37L1	6.694461255	12.68256144	0.706361066	-4.196828519	1.293032789	-3.2457247	0.001172	0.003143
ENSG00000264672	SEPT4-AS1	3.583348923	6.812338214	0.354359632	-4.198981245	1.59105898	-2.639111	0.008312	0.019248
ENSG00000136514	RTP4	23.48747774	44.55837258	2.416582898	-4.207438816	0.701042187	-6.0016913	1.95E-09	9.57E-09
ENSG00000107518	ATRNL1	3.693603626	7.032847621	0.354359632	-4.248153121	1.565331766	-2.7138995	0.00665	0.015655
ENSG00000204850	AC011484.1	6.941778033	13.1795532	0.704002868	-4.250875967	1.281686337	-3.3166274	0.000911	0.002481
ENSG00000163362	INAVA	3.724673313	7.097345191	0.352001434	-4.259156144	1.563426709	-2.7242442	0.006445	0.015215
ENSG00000134539	KLRD1	3.740662392	7.12932335	0.352001434	-4.273849309	1.617228753	-2.6426993	0.008225	0.019073
ENSG00000188158	NHS	42.98536574	81.77999222	4.190739257	-4.301228271	0.534231114	-8.05125	8.20E-16	6.10E-15
ENSG00000145936	KCNMB1	18.00937158	34.30380493	1.714938229	-4.317616725	0.79641636	-5.4213059	5.92E-08	2.57E-07
ENSG00000183778	B3GALT5	1.945254779	3.890509559	0	-4.352787594	1.878858669	-2.316719	0.020519	0.043768
ENSG00000007306	CEACAM7	1.98582506	3.97165012	0	-4.394265724	1.9300929	-2.276712	0.022803	0.04815
ENSG00000159450	TCHH	4.07345829	7.819807815	0.327108765	-4.397104724	1.554922474	-2.8278611	0.004686	0.011346
ENSG00000232554	RSU1P2	2.023260698	4.046521395	0	-4.41800053	1.871804225	-2.3602899	0.018261	0.039419
ENSG00000198732	SMOC1	19.37236045	37.03214088	1.71258003	-4.429412972	0.79387768	-5.5794653	2.41E-08	1.08E-07
ENSG00000076641	PAG1	4.200044547	8.045729463	0.354359632	-4.441481963	1.634336297	-2.7176059	0.006576	0.015506
ENSG00000196593	ANKRD20A19I	2.078737624	4.157475247	0	-4.451321781	1.840586933	-2.4184252	0.015588	0.034109
ENSG00000227210	AC079145.1	2.09294512	4.18589024	0	-4.459767441	1.833419819	-2.4324857	0.014996	0.032923
ENSG00000135373	EHF	4.224283274	8.121457783	0.327108765	-4.46086243	1.540023693	-2.8966194	0.003772	0.009287
ENSG00000144837	PLA1A	39.81232801	76.14263602	3.482019993	-4.467139541	0.57194172	-7.8104803	5.70E-15	4.05E-14
ENSG00000171246	NPTX1	56.0711665	107.306809	4.835523995	-4.478156152	0.478502078	-9.3586974	8.07E-21	7.62E-20
ENSG00000271503	CCL5	53.52361398	102.4974127	4.549815286	-4.512247109	0.50595324	-8.9183085	4.73E-19	4.13E-18
ENSG00000164082	GRM2	2.165764186	4.331528372	0	-4.51710276	1.854106601	-2.4362692	0.01484	0.032629
ENSG00000237988	OR2I1P	2.185158535	4.37031707	0	-4.52848739	1.831099043	-2.473098	0.013395	0.029751
ENSG00000142748	FCN3	4.464886178	8.602663592	0.327108765	-4.539526413	1.503977387	-3.0183475	0.002542	0.00644
ENSG00000130300	PLVAP	183.7932505	352.5158494	15.07065163	-4.544434176	0.275480721	-16.496378	3.90E-61	1.21E-59
ENSG00000107105	ELAVL2	17.11621218	32.84223669	1.390187662	-4.57231751	0.87397324	-5.2316447	1.68E-07	7.04E-07
ENSG00000171496	OR1L8	4.594535231	8.861961696	0.327108765	-4.58394079	1.502319281	-3.0512427	0.002279	0.005829
ENSG00000233705	SLC26A4-AS1	2.347056372	4.694112744	0	-4.631098133	1.797132075	-2.5769381	0.009968	0.022737
ENSG00000275793	RIMBP3	4.91002983	9.468058227	0.352001434	-4.679703406	1.497535147	-3.1249373	0.001778	0.004616

ENSG00000277449	CEBPB-AS1	4.982246412	9.610133193	0.354359632	-4.698050293	1.474409854	-3.1863937	0.001441	0.003802
ENSG00000249014	HMGN2P4	2.555410491	5.110820982	0	-4.749610731	1.736860325	-2.7345957	0.006246	0.014784
ENSG00000279266	AC068860.1	5.151286646	9.950571858	0.352001434	-4.754608496	1.496168182	-3.177857	0.001484	0.003908
ENSG00000179826	MRGPRX3	2.592846129	5.185692257	0	-4.767675406	1.745767298	-2.7309914	0.006314	0.01493
ENSG00000106302	HYAL4	2.69791398	5.395827959	0	-4.829130207	1.707038068	-2.8289529	0.00467	0.011312
ENSG00000206337	HCP5	11.32725631	21.94815155	0.706361066	-4.990013107	1.198551324	-4.1633704	3.14E-05	0.000103
ENSG00000224396	METTL15P3	3.07853964	6.157079281	0	-5.015599559	1.687201407	-2.9727332	0.002952	0.007406
ENSG00000162975	KCNF1	3.153839438	6.307678876	0	-5.05516483	1.720882847	-2.9375415	0.003308	0.008215
ENSG00000127472	PLA2G5	6.344407413	12.33445519	0.354359632	-5.055303661	1.436559858	-3.5190345	0.000433	0.001234
ENSG00000261618	LINC02605	41.91647638	81.41636985	2.416582898	-5.079386443	0.627861098	-8.0899843	5.97E-16	4.48E-15
ENSG00000224429	LINC00539	6.475991588	12.62487441	0.327108765	-5.089519409	1.422337787	-3.5782776	0.000346	0.001001
ENSG00000162873	KLHDC8A	3.336484685	6.672969369	0	-5.135507008	1.632703341	-3.1454012	0.001659	0.004332
ENSG00000188064	WNT7B	3.439770953	6.879541905	0	-5.174302773	1.709396381	-3.0269766	0.00247	0.006278
ENSG00000148408	CACNA1B	3.512590018	7.025180037	0	-5.208753387	1.623929552	-3.2074996	0.001339	0.003558
ENSG00000140323	DISP2	33.10725513	64.4474285	1.767081764	-5.221645686	0.735205673	-7.1022924	1.23E-12	7.57E-12
ENSG00000148346	LCN2	3.571900736	7.143801473	0	-5.229267625	1.603366409	-3.2614302	0.001109	0.002988
ENSG00000156427	FGF18	3.57503538	7.150070759	0	-5.239402086	1.615512906	-3.2431818	0.001182	0.003169
ENSG00000002079	MYH16	3.60031573	7.200631459	0	-5.239443509	1.624675015	-3.2249179	0.00126	0.003361
ENSG00000108309	RUNDC3A	3.701549789	7.403099577	0	-5.282578429	1.586804692	-3.3290666	0.000871	0.002378
ENSG00000006210	CX3CL1	1024.527185	1999.294957	49.75941198	-5.325527079	0.138190737	-38.537511	0	0
ENSG00000151025	GPR158	4.176869596	8.353739192	0	-5.457143667	1.539802947	-3.5440533	0.000394	0.001131
ENSG00000158714	SLAMF8	15.82561481	30.94722675	0.704002868	-5.487714607	1.137926569	-4.822556	1.42E-06	5.41E-06
ENSG00000042832	TG	4.564463692	9.128927384	0	-5.589502287	1.519305637	-3.6789848	0.000234	0.000691
ENSG00000182326	C1S	266.6940897	522.763056	10.62512342	-5.615597735	0.288306796	-19.477854	1.69E-84	8.12E-83
ENSG00000235531	MSC-AS1	4.681957457	9.363914913	0	-5.621074924	1.525254944	-3.6853347	0.000228	0.000675
ENSG00000163082	SGPP2	4.892792308	9.785584615	0	-5.682363602	1.510743109	-3.7613037	0.000169	0.000509
ENSG00000256616	AP002414.2	4.870218078	9.740436156	0	-5.683315931	1.521242633	-3.7359694	0.000187	0.000559
ENSG00000169436	COL22A1	9.879046473	19.40609151	0.352001434	-5.709901558	1.343425205	-4.2502564	2.14E-05	7.15E-05
ENSG00000173391	OLR1	53.87309619	105.7835398	1.962652591	-5.721572313	1.161482206	-4.9260955	8.39E-07	3.28E-06
ENSG00000285744	AC083837.1	99.13152153	194.8082739	3.454769126	-5.822729584	0.498413026	-11.682539	1.57E-31	2.24E-30
ENSG00000276085	CCL3L1	5.935645337	11.87129067	0	-5.964346911	1.456144574	-4.0959854	4.20E-05	0.000136
ENSG00000166592	RRAD	6.005983671	12.01196734	0	-5.984603131	1.441167826	-4.1526067	3.29E-05	0.000108
ENSG00000226979	LTA	6.235513722	12.47102744	0	-6.041294497	1.448139209	-4.1717636	3.02E-05	9.96E-05
ENSG00000166523	CLEC4E	6.285803796	12.57160759	0	-6.051173216	1.447669332	-4.1799416	2.92E-05	9.62E-05
ENSG00000038427	VCAN	1798.148172	3543.033523	53.26282057	-6.061994742	0.131963754	-45.936817	0	0
ENSG00000234678	ELF3-AS1	6.508365411	13.01673082	0	-6.099436426	1.427026179	-4.2742288	1.92E-05	6.45E-05
ENSG00000180914	OXTR	13.18953124	26.05195371	0.327108765	-6.13871427	1.307229544	-4.6959727	2.65E-06	9.78E-06
ENSG00000172379	ARNT2	7.302947952	14.6058959	0	-6.269736787	1.422273506	-4.4082497	1.04E-05	3.60E-05
ENSG00000173432	SAA1	7.762977829	15.52595566	0	-6.350001495	1.389771053	-4.569099	4.90E-06	1.75E-05
ENSG00000163673	DCLK3	116.6882839	230.6056253	2.77094253	-6.38822332	0.543226532	-11.759778	6.29E-32	9.17E-31
ENSG00000100365	NCF4	7.980781115	15.96156223	0	-6.392455245	1.414666083	-4.5187026	6.22E-06	2.20E-05
ENSG00000259342	AC025580.1	8.018870664	16.03774133	0	-6.402554706	1.385246137	-4.6219618	3.80E-06	1.38E-05
ENSG00000185269	NOTUM	8.47096233	16.94192466	0	-6.482040181	1.380505991	-4.6954089	2.66E-06	9.81E-06
ENSG00000213886	UBD	9.45860906	18.91721812	0	-6.636836221	1.349874678	-4.9166314	8.80E-07	3.43E-06
ENSG00000145649	GZMA	19.57738533	38.80041102	0.354359632	-6.711297017	1.26192009	-5.3183217	1.05E-07	4.46E-07
ENSG00000172986	GXYLT2	76.2047844	151.0240975	1.385471265	-6.778765925	0.758810412	-8.9334119	4.13E-19	3.62E-18
ENSG00000126549	STATH	21.26870282	42.1854042	0.352001434	-6.831121357	1.257916927	-5.4305028	5.62E-08	2.45E-07
ENSG00000178860	MSC	22.41903877	44.51096877	0.327108765	-6.911296432	1.251978135	-5.5203012	3.38E-08	1.50E-07
ENSG00000197859	ADAMTSL2	22.82084036	45.31457196	0.327108765	-6.937801115	1.252907118	-5.5373627	3.07E-08	1.36E-07
ENSG00000183813	CCR4	31.85263811	63.37816745	0.327108765	-7.419707041	1.232987221	-6.0176674	1.77E-09	8.69E-09
ENSG00000114646	CSPG5	115.4677734	230.2564365	0.679110199	-8.387774958	1.03834565	-8.0780181	6.58E-16	4.93E-15
ENSG00000041982	TNC	704.5921576	1406.738123	2.446191963	-9.190115317	0.55120868	-16.672661	2.07E-62	6.62E-61

Table S5: RNA-Seq analysis of the differentially expressed genes in cytokine-treated HAECs with or without acetate treatment (adjusted p value < 0.05 and Log2|Fold Change| > 1).

EnsemblID	GeneSymbol	baseMean	baseMean_TGF-β1 + IL-1β	baseMean_TGF-β1 + IL-1β + acetate	log2FoldChange	lfcSE	stat	pvalue	padj
ENSG00000006283	CACNA1G	38.61006761	0	77.22013522	8.548292373	1.227457852	6.96422476	3.30E-12	2.93E-11
ENSG00000134873	CLDN10	19.55017072	0	39.10034143	7.565592106	1.273587919	5.94037678	2.84E-09	1.93E-08
ENSG00000149451	ADAM33	19.44989598	0	38.89979196	7.562352818	1.288020665	5.87129774	4.32E-09	2.88E-08
ENSG00000101210	EEF1A2	1127.428416	13.89765295	2240.959179	7.325582153	0.652529803	11.2264331	3.02E-29	6.52E-28
ENSG00000259869	AL022344.1	30.73098226	0.393976105	61.06798842	7.248500945	1.23943475	5.84823118	4.97E-09	3.30E-08
ENSG00000132464	ENAM	85.50335451	1.148670348	169.8580387	7.243827348	0.869576875	8.33028977	8.06E-17	9.70E-16
ENSG00000103485	QPRT	19.71789851	0.360718139	39.07507887	6.600563267	1.274642394	5.17836477	2.24E-07	1.25E-06
ENSG00000154096	THY1	18.81252537	0.368458623	37.25659212	6.533199253	1.274858709	5.12464574	2.98E-07	1.63E-06
ENSG00000074181	NOTCH3	755.696121	16.46073241	1494.93151	6.514472858	0.542144155	12.0161267	2.92E-33	7.32E-32
ENSG00000159713	TPPP3	17.27113433	0.393976105	34.14829255	6.411350553	1.288396756	4.97622376	6.48E-07	3.41E-06
ENSG00000157542	KCNJ6	47.83816017	1.181928314	94.49439203	6.394872642	0.902180598	7.08824005	1.36E-12	1.24E-11
ENSG00000114200	BCHE	16.53792023	0.360718139	32.71512232	6.344894634	1.287599981	4.92769084	8.32E-07	4.32E-06
ENSG00000267316	AC090409.2	8.051684523	0	16.10336905	6.288261553	1.400834507	4.48893964	7.16E-06	3.32E-05
ENSG00000198734	F5	15.79732718	0.393976105	31.20067826	6.276724656	1.292003108	4.85813433	1.18E-06	6.04E-06
ENSG00000109255	NMU	7.497049639	0	14.99409928	6.181477972	1.445058118	4.27766738	1.89E-05	8.27E-05
ENSG00000070526	ST6GALNAC1	6.917202678	0	13.83440536	6.068726689	1.435430329	4.22780999	2.36E-05	0.000102
ENSG00000124496	TRERF1	37.33492645	1.097635385	73.57221751	6.036780072	0.906338639	6.6606231	2.73E-11	2.22E-10
ENSG00000111087	GLI1	71.01965212	2.238565249	139.800739	5.964252848	0.646533354	9.22497318	2.84E-20	4.07E-19
ENSG00000012124	CD22	6.099411638	0	12.19882328	5.893230092	1.489233358	3.95722407	7.58E-05	0.000303
ENSG00000154269	ENPP3	6.023400964	0	12.04680193	5.875596382	1.540141358	3.81497215	0.000136	0.000522
ENSG00000164287	CDC20B	21.53069236	0.729176762	42.33220796	5.830111803	1.138381467	5.12140436	3.03E-07	1.66E-06
ENSG00000105048	TNNT1	790.4064269	27.68452059	1553.128333	5.819712542	0.302103686	19.2639574	1.08E-82	8.62E-81
ENSG00000250548	LINC01303	63.5911404	2.279563699	124.9027171	5.800530564	0.673439455	8.61329185	7.10E-18	8.98E-17
ENSG00000130035	GALNT8	5.768210355	0	11.53642071	5.798271926	1.496085991	3.87562745	0.000106	0.000415
ENSG00000151090	THRB	5.467909226	0	10.93581845	5.721618314	1.527021896	3.74691308	0.000179	0.000671
ENSG00000152578	GRIA4	10.73989937	0.360718139	21.11908061	5.718783988	1.366695115	4.18438899	2.86E-05	0.000122
ENSG00000255856	AC069503.1	5.315081507	0	10.63016301	5.695917306	1.584062528	3.59576545	0.000323	0.001151
ENSG00000008118	CAMK1G	5.267916491	0	10.53583298	5.668694736	1.527875615	3.71018078	0.000207	0.000767
ENSG00000006016	CRLF1	10.37545701	0.368458623	20.38245541	5.661796248	1.347758603	4.20089787	2.66E-05	0.000114
ENSG00000214140	PRCD	5.219121906	0	10.43824381	5.656882472	1.516448051	3.73035032	0.000191	0.000712
ENSG00000127951	FGL2	48.09290955	1.911105076	94.27471402	5.64974476	0.72586667	7.78344701	7.06E-15	7.59E-14
ENSG00000164142	FAM160A1	102.520217	4.039859913	201.0005741	5.613479948	0.484542721	11.5851084	4.90E-31	1.14E-29
[truncated: 555,541 more chars]
