# Supplementary material for: Transcriptome analysis reveals molecular signature and cell-type difference of Homo sapiens endothelial-to-mesenchymal transition
Source: G3 (Bethesda). 2023 Oct 20;13(12):jkad243. doi: 10.1093/g3journal/jkad243 (PMC10700110; doi:10.1093/g3journal/jkad243)
Supplement: jkad243_Supplementary_Data [file jkad243_supplementary_data.zip › Supplemental_Material_Legends_G3-2023-404591.docx]

**Supporting information**

**Supporting Figure Legends:**

**Figure S1. TGF-β1 and IL-1β induce EndoMT markers.** (A) hierarchically clustered heatmap of the expression of 186 pan-endothelial genes showing vascular heterogeneity of normal endothelial cells without induction of EndoMT. (B) Real-time qPCR analysis of endothelial markers and mesenchymal markers in cytokine-treated endothelial cells (n = 2-3 biological replicates). **p*< 0.05; ***p*< 0.01; ****p*< 0.001.

**Figure S2. EndoMT regulates cell chemotaxis and cell cycle processes.** (A) Hierarchically clustered circular heatmap of all 49 genes of the GO category “cell chemotaxis” in Fig 2C. (B) Venn diagram showing overlapping and unique sets of downregulated genes in cytokine-treated endothelial cells (log_2_[Fold Change] < -0.5 and adjusted *p* value < 0.05). (C) Bubble plot depicting top 12 enriched GO biological processes for the shared 716 downregulated genes in cytokine-treated endothelial cells.

**Figure S3. Vascular-bed-specific EndoMT has unique gene signatures.** (A-C) From the STRING database-based networks (with a medium confidence cutoff of score 0.4) showing the protein-protein interactions of ten most strongly upregulated or downregulated, highly expressed signature genes in HUVECs (A), HAECs (B) and HPMVECs (C). Edges represent protein-protein associations (blue edge: known interactions from curated databases; pink edge: experimentally determined known interactions; others based on text mining [light green edge], co-expression [black], and protein homology [light purple]). (D-F) Rcircos plots showing the chromosome positions of the ten most strongly upregulated or downregulated, highly expressed signature genes in HUVECs (D), HAECs (E), and HPMVECs (F).

**Figure S4.** **Vascular-bed-specific EndoMT has unique experimentally validated protein-protein interactions**. STRING database-based analyses (with the highest confidence cutoff of score 0.9) showing the experimentally validated protein-protein associations of the differentially expressed genes in HUVECs only (of 502 upregulated genes in panel A; of 434 downregulated genes in panel B), HPMVECs only (no experimentally validated protein-protein interactions of 351 upregulated genes; of 774 downregulated genes in panel C) or HAECs only (of 1098 upregulated genes in panel D; of 1225 downregulated genes in panel E). Network type: physical subnetwork (the edges indicate that the proteins are part of a physical complex). Meaning of network edges: confidence (line thickness indicates the strength of data support). Active interaction sources: experiments.

**Figure S5. Acetate regulates the expression of genes involving EndoMT-associated leukocyte chemotaxis and related biological processes. (**A and B) Venn diagrams showing common differentially expressed genes of cytokine-upregulated genes and acetate-suppressed genes (A), and of cytokine-downregulated genes and acetate-restored genes (B) (adjusted *p* value < 0.05 and Log2|Fold Change| > 1). (C) Tabular forms showing the common enriched GO biological process categories “leukocyte chemotaxis,” “leukocyte cell-cell adhesion,” and “leukocyte migration” in acetate-treated endothelial cells. (D) Venn diagrams showing the overlapping gene sets between differentially expressed genes of the common categories “leukocyte chemotaxis,” “leukocyte cell-cell adhesion,” and “leukocyte migration” in Figure 5A-5C. (E) Hierarchically clustered heatmap analysis of 24 differentially expressed genes shared by the common categories “leukocyte chemotaxis,” “leukocyte cell-cell adhesion,” and “leukocyte migration” in Figure S5D.

**Figure S6. A potential role for SLC transporter genes in EndoMT.** (A) Violin plot showing the frequency distribution of 85 highly expressed SLC genes in the presence or absence of acetate following control or cytokine treatment (n = 3 biological replicates). The horizon lines indicate the median (red line) and quartiles (black line). (B) Hierarchically clustered heatmap of the expression of 85 highly expressed SLC genes in the presence or absence of acetate following control or cytokine treatment (n = 3 biological replicates).

**Supporting Table Legends:**

**Table S1. RNA-Seq analysis of the differentially expressed genes in HUVECs with or without cytokine treatment.**

**Table S2. RNA-Seq analysis of the differentially expressed genes in HAECs with or without cytokine treatment.**

**Table S3. RNA-Seq analysis of the differentially expressed genes in HPMVECs with or without cytokine treatment.**

**Table S4. RNA-Seq analysis of the differentially expressed genes in cytokine-treated HUVECs with or without acetate treatment.**

**Table S5. RNA-Seq analysis of the differentially expressed genes in cytokine-treated HAECs with or without acetate treatment.**

**Table S6. RNA-Seq analysis of the differentially expressed genes in cytokine-treated HPMVECs with or without acetate treatment.**
